# Yixin-Shu Capsules Ameliorated Ischemia-Induced Heart Failure by Restoring Trx2 and Inhibiting JNK/p38 Activation

**DOI:** 10.1155/2021/8049079

**Published:** 2021-02-16

**Authors:** Changpei Xiang, Fangbo Zhang, Jinhuan Gao, Feifei Guo, Mao Zhang, Rui Zhou, Junying Wei, Ping Wang, Yi Zhang, Jingjing Zhang, Hongjun Yang

**Affiliations:** ^1^Institute of Chinese Materia Medica, China Academy of Chinese Medical Sciences, Beijing 100700, China; ^2^Institute of Molecular Medicine, Peking University, Beijing 100871, China

## Abstract

Traditional Chinese medicine has shown great safety and efficacy in the treatment of heart failure (HF), whereas the mechanism remains unclear. In this study, the protective effect of Yixin-shu (YXS) capsules, a conventional medicine for various cardiovascular diseases, against myocardial ischemia-induced HF in rats was systematically investigated by RNA-seq technology. HF rats treated with YXS (0.8 or 1.6 g/kg/d, ig) for 6 weeks had significantly decreased brain natriuretic peptide (BNP) and atrial natriuretic peptide (ANP) and collagen III and attenuated cardiac structure rupture and collagen deposition. Additionally, YXS treatment decreased the levels of interleukin-1*β* (IL-1*β*), interleukin 6 (IL-6), tumor necrosis factor-*α* (TNF-*α*), and lactate dehydrogenase (LDH) and TUNEL-positive rate and the nitrotyrosine staining, but increased levels of glutathione (GSH), total antioxidant capacity (T-AOC) activity, and mitochondrial membrane potential. Further experiments demonstrated that YXS restored Trx2 and inhibited the phosphorylation of JNK and p38, thereby improving cardiac function in the rats with HF. Silencing Trx2 decreased the protection of YXS in the response to H_2_O_2_ as evidenced by the increase of caspase-3 activity and decrease of GSH level. Thus, YXS enhanced heart function and decreased myocardial damage through restoring Trx2 and inhibiting JNK and p38 activation in ischemia-induced HF.

## 1. Introduction

Heart failure (HF), characterized by reduced heart contractility and cardiac function, is an end stage of various cardiovascular diseases, which results in serious myocardial injury at the beginning and leads to damage of many other organs in the end [1]. Multiple insults including myocardial ischemia, atherosclerosis, hypertension, cardiomyopathy, and genetic problems cause myocardial damage and result in HF. Among all these insults, myocardial ischemia has emerged as one of the major causes of HF in recent decades. Several symptoms such as cardiomyocyte hypertrophy, elevated neurohumoral level, and enhanced filling pressure happened which attempted to compensate the dysfunction of the heart and finally resulted in the acceleration of cardiac remodeling and contraction declining [2, 3]. Due to the complexity of HF, multiple pharmacologic remedies were applied to halt HF progression, with unexpected side effects. For example, *β*-adrenergic agonists improve cardiac contractility, but also increase mortality in HF, while neurohormonal inhibition, a meaningful intervention for HF, is restricted by its side effect, such as deterioration of renal function, potassium retention, and angioedema caused by angiotensin-converting enzyme inhibitor (ACEI) [4, 5]. Some other therapies such as diuretics only focus on the alleviation of the symptoms of HF, but do not prevent deterioration of HF. Thus, it is of importance to explore effective therapies for halting HF progression and revealing the underlying mechanism.

Great attention has been paid to traditional Chinese medicine (TCM) due to their effectiveness and few side effects in halting HF [6–8]. For example, in a randomized, multicenter placebo-controlled design with double-blind study, 640 patients with ischemia HF who were treated with Qishenyiqi dripping pills for 6 months in addition to standard treatment demonstrated improved exercise tolerance when compared to placebo [9]. Additionally, Qiliqiangxin capsule has been demonstrated to reduce important cardiac damage markers such as NT-pro-brain natriuretic peptide and improve the life quality of patients [10]. Even though considerable researches have been conducted to investigate the efficacy and the mechanism of TCM, an ample amount of researches is still needed to draw concrete conclusion whether TCM has an effect on the HF treatment. In particular, the ambiguous mechanism and poor clinical locations of TCM hinder their further application.

Yixin-shu (YXS) capsules, which originated from an ancient TCM formula named ShenMaiSan, is a standardized Chinese Materia Medicine product recorded in the Chinese Pharmacopeia and is widely used for various cardiovascular diseases in clinic [11, 12]. YXS is composed of seven herbs, including *Radix Ginseng*, *Fructus Schisandrae Chinensis*, *Radix Ophiopogonis*, *Astragalus Membranaceus*, *Salvia Miltiorrhiza*, *Ligusticum Wallichii*, and *Fructus Crataegi*. Recent researches revealed that YXS remarkably decreased myocardial ischemia/reperfusion injury, reduced hydrogen peroxide-caused damage, and improved ET-1-induced cardiomyocyte dysfunction [6, 13, 14]. As early as 24 h after reperfusion, YXS significantly decreased myocardial injury and ameliorated heart function through decreasing oxidative damage and cell apoptosis via upregulating liver-X-receptor *α* [13]. And potential HF biomarkers such as TNNI3, GAL-3, HSP 70, FABP3, and CKAP5 were identified in the protection of YXS against HF which facilitated the clinical location of YXS [6]. *In vitro*, YXS protected against H_2_O_2_-induced damage through APEX1, TFCP2, and 5 other transcription factors by using an integrated strategy of transcriptome, proteomics, and pharmacological network analysis [15] and then identified schisandrin A and schisandrin B as the effective components of YXS against ET-1-induced cardiomyocyte dysfunction [14]. Additionally, YXS was proved to promote mesenchymal stem cells to differentiate into cardiac-like cells *in vitro* [11]. It is worth noting that although it was previously determined that YXS has a significant protective effect on HF induced by myocardial ischemia, the mechanism remains unclear. Here, taking advantage of RNA-seq technology, the protective effect of YXS on MI-induced HF was systematically investigated and the underlying mechanism was revealed, to provide reference for promoting the clinical application of YXS.

## 2. Materials and Methods

### 2.1. Animal Model

Male Sprague-Dawley (SD) rats with weight at 250-270 g were provided by Beijing Huafukang Biotechnology Co., Ltd., China ((certificate no. SCXK (Jing) 2014-0004)). The rats were kept in the Laboratory Animal Center of China Academy of Chinese Medical Sciences (Beijing, China) with a 12-hour alternating of day and night. Free access to food and water was allowed for the rats. All the animal experiments were approved by the Committee on the Animal Care and Use of Institute of Experimental Research Center, China Academy of Chinese Medical Sciences (Beijing, China).

The SD rats were anesthetized with 10 mg/ml pentobarbital sodium by intraperitoneal injection before the following surgery. Briefly, the left anterior descending coronary artery (LAD) of these rats was tightened for four weeks to prepare the HF rats. Briefly, the LAD was tightened with a 6-0 silk suture. The sham normal group rats received the same procedures but without tightening the silk suture at LAD. After the surgery for 4 weeks, the rats with HF were randomly divided into four groups according to the echocardiography results. The rats received different treatments such as normal (saline, ig), HF (heart failure, saline, ig), HF+YXS-H (1.6 g/kg/d, ig), HF+YXS-L (0.8 g/kg/d, ig), and HF+VST (Valsartan 8 mg/kg/d, ig). YXS was purchased from Guizhou Xinbang Pharmaceutical Co., Ltd. (Guiyang, China) (drug approval number: Z52020038, batch number: 20140810). Collectively, 1000 YXS capsules were prepared by using 200 g *Radix Ginseng*, 133 g *Fructus Schisandrae Chinensis*, 200 g *Radix Ophiopogonis*, 200 g *Astragalus Membranaceus*, 267 g *Salvia Miltiorrhiza*, 133 g *Ligusticum Wallichii*, and 200 g *Fructus Crataegi* after extraction from 85% alcohol. According to Chinese Pharmacopeia, every capsule of YXS contained total content of ginsenoside Rg1 and ginsenoside Re no less than 0.4 mg and salvianolic acid B no less than 1 mg. Additionally, 276 components were identified in YXS capsule, including 149 ginsenosides, 30 tanshinones, 50 lignans, 18 flavones, 14 phenolic acids, 7 astragalus saponins, 4 lactones, 3 ophiopogonins, and 1 triterpenoid acid [11]. Valsartan was purchased from Hainan Aumei Pharmaceutical Co., Ltd. (Hainan, China). Visual Sonics Vevo 770 Ultrasound System (Toronto, Canada) with a 17.5 MHz probe was applied to detect the heart function at 0 and 6 weeks after drug administration, and the left ventricular fractional shortening (LVFS) and left ventricular ejection fraction (LVEF) were calculated.

### 2.2. RNA-Seq Analysis

TRIzol reagent (Cat# 15596-018, Life Technologies, USA) was applied to obtain RNA from the harvested samples. After the RNA integrity was assessed, the RNA was used for the following sequencing libraries based on Illumina® NEBNext® Ultra™ RNA Library Prep Kit (NEB, USA). The next step was the same to the previous researches [16]. All the sequencing and mapping were finished by Novogene Bioinformatics Technology Co., Ltd. (Beijing, China). After sequencing, the read mapping referred to rat reference genome (ensemble release 83) and the read number was obtained by using HTSeq v0.6.1. Fragments per Kilobase Million (FPKM) were used to represent the gene expression level, and edgeR software was applied to calculate differentially expressed genes (DEs) [17]. The detected genes with fold change no less than 2 and FDR < 0.05 were defined as DEs. The raw data of YXS-mediated protection against HF has been uploaded into https://www.ncbi.nlm.nih.gov/sra/PRJNA532999. The DEs after YXS treatment were enriched using DAVID [18]. The DEs were associated with disease targets of HF using String, and then, the network was generated by using Cytoscape 3.4.0.

### 2.3. Hematoxylin and Eosin (H&E) Staining, Masson's Trichrome Staining, and Immunofluorescence Staining (IF)

The heart samples were harvested and fixed in 4% (*v*/*v*) polyformaldehyde and then dehydrated before being embedded by paraffin for further analysis. The embedded samples were sectioned, rehydrated, and stained with H&E staining and Masson's trichrome staining (D026-1-3) to evaluate heart injury and collagen deposition. As for the IF for heart tissue, the samples were rehydrated and then incubated with sodium citrate before permeabilizing with Triton X-100, which was followed by 5% BSA blocking. For IF staining, the cell samples were incubated with 10% (*v*/*v*) formalin for 20 min and then followed with 0.5% Triton X-100 for 20 min. After that, the samples were treated with 5% BSA for 30 min, before the primary antibodies such as thioredoxin 2 (Trx2, ab185544) and p-JNK (ab131499) and nitrotyrosine (sc101358) were added, respectively, and incubated at 4°C overnight. Then, samples were treated with second antibodies such as goat anti-mouse (ab6785) or goat anti-rabbit (ab150080). The nucleus was counterstained with DAPI for 10 min and observed with LSM-880 confocal microscope (Carl Zeiss, Oberkochen, Germany).

### 2.4. JC-1 Staining and TUNEL Staining

TUNEL detection kit (G002) purchased from Nanjing Jiancheng Bioengineering Institute and mitochondrial membrane potential assay kit with JC-1 (C2006) from Beyotime Biotechnology were used for cell apoptosis detection. All procedures were performed based on the instruction from the kits. Briefly, the cells with different treatments were incubated with JC-1 staining solution at 37°C for 20 minutes and then washed twice with JC-1 staining buffer before being observed under an LSM-880 confocal microscope (Carl Zeiss, Oberkochen, Germany). For terminal deoxynucleotidyl transferase dUTP nick-end labeling (TUNEL) staining, the cells after various treatments were fixed with 4% paraformaldehyde for 30 min and then incubated with 1% BSA for 10 min. After that, the samples were incubated with 0.5% Triton X-100 for 20 min, which was followed by TdT enzyme reaction solution for 60 min treatment. Finally, DAPI was used for nucleus staining for 5 min before observing with an LSM-880 confocal microscope (Carl Zeiss, Oberkochen, Germany). And the quantitative result of TUNEL-positive cells and the fluorescence ratio of red and green were carried out through ImageJ.

### 2.5. Enzyme-Linked Immunosorbent Assay (ELISA)

The ELISA kits of BNP (DY-0127), ANP (DY-0111), IL-1*β* (DY-0078), IL-6 (DY-0045), CRP (CRP4020), and TNF-*α* (DY-0026) were obtained from Beijing Deyi Diagnostics and IL-6 (SEA079Ra), TNF-*α* (HEA133Ra), and Trx2 (SED378Ra) from Cloud-Clone Corp., Wuhan. Additionally, the JNK (Thr183/Tyr185) In-Cell ELISA Kit (ab126424) and p38MAPK Alpha (Thr180/Tyr182) In-Cell ELISA Kits (ab126425) were used to detect the p-JNK/JNK and p-p38/p38, respectively. And the ELISA experiments were performed according to the instructions. Briefly, the serum or protein extract was incubated into the plates immobilized with specific antibodies and followed by incubation with a secondary antibody conjugated with a horseradish peroxidase for detection through a microplate reader (DNM-9602G).

### 2.6. Western Blotting

Heart tissue was lysed using RIPA lysis buffer (R0020, Solarbio), with protease inhibitor (0.1% phenylmethanesulfonyl fluoride (PMSF)). The method of protein extraction is as follows: the lysate was prepared by using 1 ml RIPA with 10 *μ*l phenylmethanesulfonyl fluoride (PMSF, protease inhibitor), and the final concentration of PMSF is 1 mM. The myocardial tissue was cut into small fragments, and the lysate was added according to the proportion of 150-250 *μ*l lysate per 20 mg tissue and homogenized with a glass homogenizer until it was fully cracked. The pyrolyzed sample was centrifuged at 10000-14000 g for 3-5 minutes, and the supernatant was collected. The solution was boiled after the loading buffer of 4′ sodium dodecyl sulfonate (SDS, P1015, Solarbio) was added. Then, the separation of proteins was carried out on 10% polyacrylamide gels by electrophoresis and then was transferred to polyvinylidene difluoride membranes. Bovine serum albumin was used to block, and primary antibodies such as anti-Trx2 antibody (ab185544, Abcam), anti-JNK1/2 antibody (sc-137019), anti-JNK1+JNK2 (phospho T183+Y185) antibody (ab131499), anti-collagen III antibody (Sigma C7805), and anti-*β*-actin antibody (cst4970) were loaded for 24 h at 4°C and then incubated with secondary antibody conjugated with HRP (Jackson 111-035-003). The signals on PVDF membranes were captured using scanning densitometry with image analysis software (Science Lab 2005 Image Gauge; Tokyo, Japan) after applying an enhanced chemiluminescence plus detection system (Pierce Biotechnology).

### 2.7. Cell Culture, Cell Viability, LDH and GSH Levels, and T-AOC Activity

Peking Union Medical Collage provided H9C2 cells for our *in vitro* experiments. The cells were cultured at 37°C in an incubator with culture medium containing high-glucose DMEM (Gibco, USA), 10% (*v*/*v*) fetal bovine serum, 100 *μ*g/ml penicillin, and 100 *μ*g/ml streptomycin. The culture medium was changed every three days, and cells were passaged when 80% of cell confluence was reached. As for cell viability detection, H9C2 cells were incubated with various concentrations of YXS extract (0, 3.91, 15.625, and 62.5 *μ*g/ml) and then incubated with 500 *μ*m H_2_O_2_ for 1 hour. After that, cell viability was measured by using Cell Counting Kit-8 (CCK-8) which was obtained from Dongren Chemical Technology Co., Ltd. The YXS extract was prepared by dissolving the powder from YXS capsule in ethanol (95%, *v*/*v*) and extracting for 2 hours, followed by rotary evaporation and then resuspending with Tyrode buffer solution (glucose 1.00 g, NaCl 8.00 g, NaHCO_3_ 1.00 g, MgCl_2_ 0.10 g, CaCl_2_ 0.20 g, KCl 0.28 g, NaH_2_PO_4_ 0.05 g, pH 7.4) at a concentration of 16% (*w*/*v*). The kits for LDH (A020-2), GSH (A006-2-1), and T-AOC (A015-2-1) were obtained from Nanjing Jiancheng Bioengineering Institute, and the detection was performed according to their instructions.

### 2.8. *In Vitro* siRNA Transfection

To inhibit Trx2 expression, specific siRNA included three independent sequences: (1) Trx2 siRNA-1: sense 5′-GUCAACAGUGAGACACCAGUUTT-3′, antisense 5′-AACUGGUGUCUCACUGUUGACTT-3′; (2) Trx2 siRNA-2: sense 5′-GCCAUUGAGUACGAGGUGUCUTT-3′, antisense 5′-AGACACCUCGUACUCAAUGGCTT-3′; and (3) siRNA-3: sense 5′-GAAGCUAAUUGGCUGACAATT-3′, antisense 5′-UUGUCAGCCAAUUAGCUUCTT-3′. The sequences used for control siRNA are listed as follows: sense 5′-GUGAGCGUCUAUAUACCAUdTdT-3′, antisense 5′-AUGGUAUAUAGACGCUCACdTdT-3′. The transfection of Trx2 siRNA in H9C2 was performed by using Lipofectamine 2000 reagent (Invitrogen, California, USA). Lipofectamine 2000 reagent was mixed with siRNA and then incubated with H9C2 cells at a density of 7∗10^4^ cells per well in 6-well plates for 7 hours. After 48 h transfection, RT-PCR was performed to detect Trx2 expression.

### 2.9. Real-Time RT-PCR

After the transfection, H9C2 cells were extracted with TRIzol (Invitrogen, 15596-026) and evaluated using the NanoDrop Lite. Superscript III (ABI-Invitrogen, 11752050) was applied for cDNA synthesis according to the instruction. Real-time PCR was carried out by following SYBR qPCR Mix (ABI-Invitrogen, 4472920) on Applied Biosystems (USA) for predenaturation at 95°C for 5 min, 40 cycles of denaturation at 95°C for 10 sec, followed by annealing at 58°C for 20 sec, and elongation at 72°C for 20 sec. The primer sequences used in real-time PCR analysis are listed: Trx2 (F: 5′-TTCAACAACCTTTAACGACC-3′; R: 5′-CTGAGCATGAAAGTCCACG-3′) and *β*-actin (F: 5′-CTGAACGTGAAATTGTCCGAGA-3′; R: 5′-TTGCCAATGGTGATGACCTG-3′).

### 2.10. Measurement of Caspase-3 Activity

Caspase-3 activity (G015-1-2) was obtained from Nanjing Jiancheng Bioengineering Institute, and the detection was performed according to their instructions. Briefly, the cell proteins were added with 25 *μ*g Ac-DEVD-pNA at 37°C for 90 min, which was used as a colorimetric-specific substrate. The OD value was measured on a microplate reader (Molecular Devices, USA). One unit of caspase-3 activity is the enzyme amount that cleaves 1 nmol Ac-DEVD-pNA at 37°C per hour under saturated substrate concentrations.

### 2.11. Statistical Analysis

The comparison of the results was performed using SPSS V17.0 software. One-way ANOVA with LSD's post hoc multiple-comparison analysis was used for the comparison for multiple groups while the Student *t*-test was used to analyze data between two groups. Data was expressed as mean ± standard deviation (SD), and *P* < 0.05 was deemed statistically significant, *P* < 0.01 for highly significant, while *P* < 0.001 for remarkably significant.

## 3. Results

### 3.1. YXS Treatment Improved MI-Induced HF

**Figure 1:**
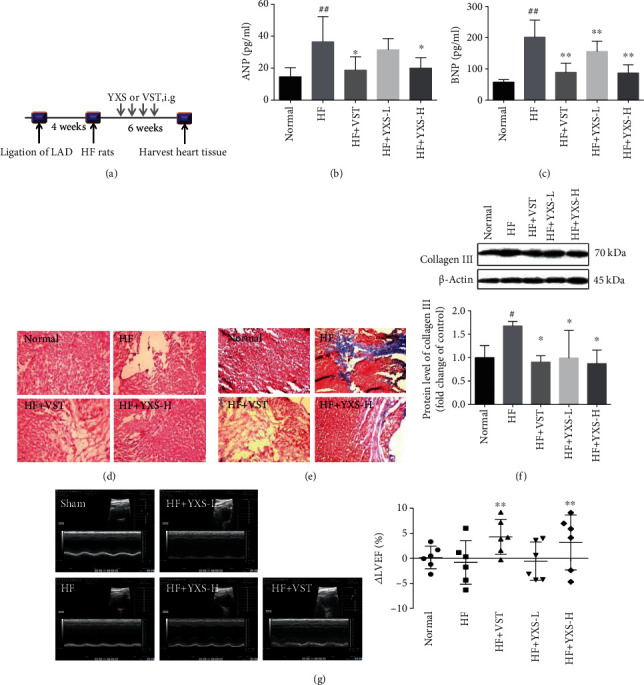
YXS demonstrated an obvious protective effect in the rats with ischemia-induced HF. (a) Schematic showing induction of HF and treatment of YXS and VST for 6 weeks; (b) serum ANP content (*n* = 5–7); (c) serum BNP content (*n* = 5–7); (d) HE staining, magnification: 10x; (e) Masson trichrome staining, magnification: 10x; (f) western blotting of collagen III and its semiquantitative data by ImageJ (*n* = 3); (g) the representative echocardiography images of rats receiving different treatments in short-axis M-mode and the increased LVEF (?%LVEF) after various treatments (*n* = 6). Data were expressed as mean ± SD (*n* = 5–7). ^#^*P* < 0.05 and ^##^*P* < 0.01*vs*. normal; ^∗^*P* < 0.05 and ^∗∗^*P* < 0.01*vs*. HF.

To investigate the protective effect of YXS against HF, histology staining, cardiac contraction function, and ANP and BNP were measured. As shown in Figure 1, an increase of ANP and BNP in HF was remarkably reduced after YXS treatment, which exhibited the same protection effect as the positive drug VST. In addition, an obvious rupture and breakage in myocardial fiber and collagen deposition were observed in the failing hearts, while treatment of YXS or VST apparently ameliorated rupture and collagen deposition in myocardial tissue. Additionally, the increase of collagen III in HF was reduced by YXS or VST treatment, and this was consistent with the Masson staining result, further confirming the attenuation of myocardial fibrosis by YXS and VST. Moreover, an obvious reduction in left ventricular ejection fraction (*Δ*%LVEF) was found in the HF group, indicating an obvious myocardial dysfunction occurred in the HF group. However, *Δ*%LVEF was significantly higher with the treatment of YXS or VST. These data indicated that YXS showed an obvious protection in rats with HF.

### 3.2. RNA-Seq Analysis of the Potential Mechanism of YXS

**Figure 2:**
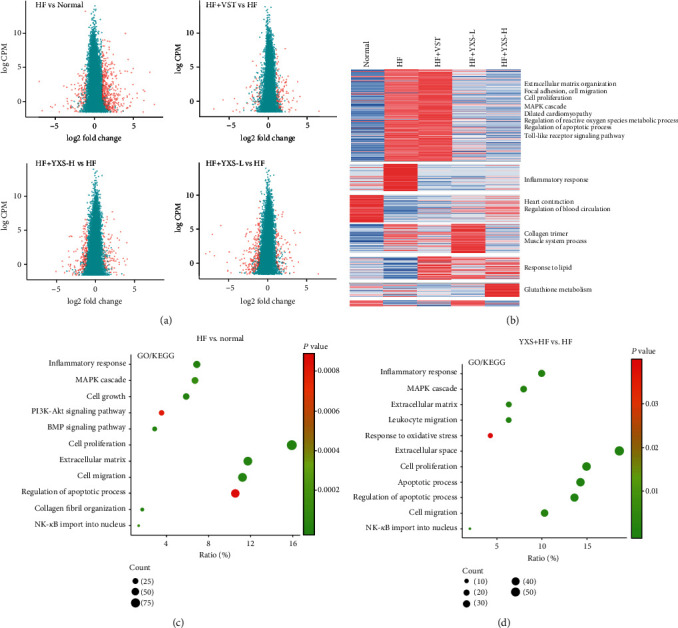
RNA-seq analysis of the transcriptional gene expression in failing myocardium. (a) Volcano plot, the dots in red indicated DEs and dots in green indicated genes with no significance; (b) hierarchical cluster analysis; (c) GO enrichment plot of DEs in HF vs. normal; (d) GO enrichment plot of DEs in HF+HF vs. HF.

The high-throughput RNA-seq technology is a widely used tool for the transcriptome gene profile analysis and is suitable for the investigation of the mechanism of TCM in multiple pathological processes. To reveal the underlying mechanism of YXS-mediated protection effect, the transcriptional gene expression was analyzed by RNA-seq technology (Table S1). As indicated by Figure 2(a), there were 637 DEs with 519 upregulated and 120 downregulated in HF compared to the normal group. In the YXS-treated group, 333 DEs including 113 upregulated and 220 downregulated were found when compared to the HF group. Different from YXS treatment, 125 upregulated genes with only 74 downregulated genes were observed after VST treatment, indicating these two drugs work in a different manner. Further hierarchical cluster analysis of the RNA-seq data indicated that YXS treatment reversed the upregulated genes involved in biological process including “inflammatory response,” “Toll-like receptor signaling pathway,” “extracellular matrix organization,” “focal adhesion,” “cell migration,” “cell proliferation,” “dilated cardiomyopathy,” and “regulation of apoptotic process” while it increased the downregulated genes involved in “heart contraction” and “regulation of blood circulation” in the HF group (Figure 2(b)).

To further reveal the key biological process in YXS-mediated protection against HF, the DEs between various treatments were enriched into Kyoto Encyclopedia of Genes and Genomes (KEGG) pathways and Gene Ontology (GO) as shown in Figures 2(c) and 2(d). The enriched KEGG and GO terms in HF included “inflammatory response,” “MAPK cascade,” “cell growth,” “PI3K-AkT signaling pathway,” “cell proliferation,” “extracellular matrix,” “cell migration,” “regulation of apoptotic process,” “collagen fibril organization,” and “NF-kappaB (NF-KB) import into nucleus.” It is noteworthy that DEs between HF with or without YXS treatment were also enriched into terms such as “inflammatory response,” “MAPK cascade,” “extracellular matrix,” “cell migration,” “cell proliferation,” and “NF-kappaB import into nucleus.” Moreover, YXS also showed an obvious effect on genes involved in “leukocyte migration,” “cellular response to interleukin-1,” and “negative regulation of cell death.” The *in vitro* RNA-seq analysis indicated that upregulated genes were enriched into GO terms such as “cell cycle,” “DNA repair,” and “DNA replication” while downregulated genes were enriched into GO terms such as “MAPK cascade,” “inflammatory response,” “apoptotic process,” and “response to oxidative stress”(Figure S1).

**Figure 3:**
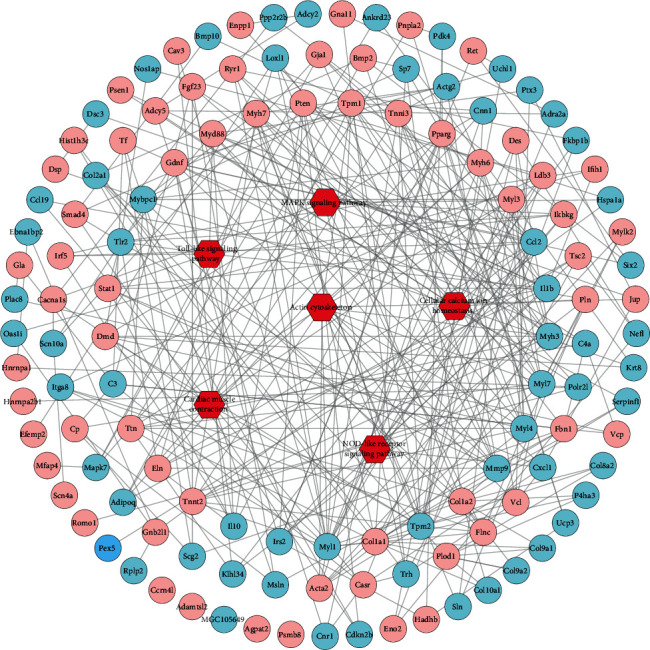
A network of DEs after YXS treatment in HF associated with HF disease target and their enriched KEGG signaling pathway; the pink nodes indicated disease targets of HF and green nodes indicated DEs and red nodes represented the enriched KEGG signaling pathways.

Further associating the DEs with disease targets of HF demonstrated that 60 DE genes were associated with 63 disease targets, which were enriched into GO terms such as “actin cytoskeleton,” “Toll-like signaling pathway,” “MAPK signaling pathway,” “NOD-like receptor signaling pathway,” “cellular calcium ion homeostasis,” and “cardiac muscle contraction” (Figure 3). To verify the network analysis results, the levels of Tlr4 and Myd88 which were critical targets in the Toll-like signaling pathway and changes in F-actin of heart tissue were also confirmed in Figure S2. The increased expression of Tlr4 and Myd88 in H_2_O_2_-induced cell model was significantly decreased by YXS treatment (Figure S2A). Additionally, YXS at high dose obviously improved the F-actin arrangement and size in HF rats and the F-actin became larger and regular than that in HF (Figure S2B).

### 3.3. YXS Inhibited Inflammation, Oxidative Damage, and JNK Activation, While Enhancing Trx2 Level in HF Rats

In this work, both the *in vitro* and *in vivo* RNA-seq analysis indicated MAPK cascade was involved in YXS-mediated protection, and it has been well known that MAPK cascade played a vital role in mediating reactive oxygen species (ROS) and inflammation signals in HF development. Thus, in our study, we focused on the MAPK cascade and we speculated that YXS may protect against HF through modulating MAPK cascades. Recently, Trx2 has been identified as an important upstream molecule that regulated MAPK cascade; thus, the effect of YXS on Trx2 and its downstream MAPK cascade was investigated.

**Figure 4:**
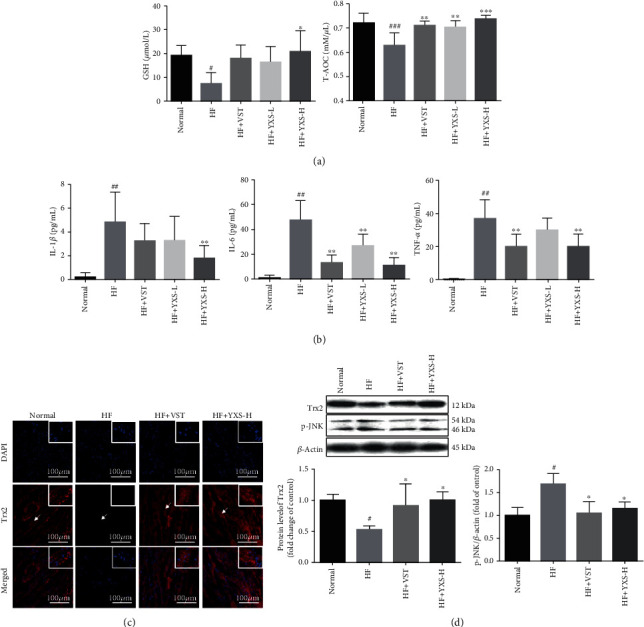
YXS enhanced antioxidant capacity and Trx2 level while inhibiting inflammation and JNK activation. (a) Serum GSH level and T-AOC activity (*n* = 6); (b) serum levels of IL-1*β*, IL-6, and TNF-*α* (*n* = 5–7); (c) IF for Trx2 (red), nucleus (blue), scale bar: 100 *μ*m; (d) representative western blotting results of Trx2 and p-JNK and their semiquantitative data by ImageJ. Data were expressed as mean ± SD. ^#^*P* < 0.05 and ^##^*P* < 0.01*vs*. normal; ^∗^*P* < 0.05 and ^∗∗^*P* < 0.01*vs*. HF.

As indicated in Figure 4, to confirm the vital role of antioxidation and anti-inflammation of YXS in resisting HF, antioxidant molecules such as GSH level and T-AOC and inflammatory factors such as IL-1*β*, IL-6, and TNF-*α* were detected. As shown in Figure 4(a), only YXS at high dose significantly increased GSH levels in contrast to the effect of VST, when compared with the untreated HF group. And higher T-AOC was observed after the treatment of YXS at both high and low dose as well as VST than that in the untreated HF group. As indicated in Figure 4(b), the levels of IL-1*β*, IL-6, and TNF-*α* were significantly higher in HF than in the normal group, whereas these were remarkably reduced after YXS treatment. In contrast, VST treatment only significantly decreased IL-6 and TNF-*α*, but had no effect on IL-1*β*. Taken together, YXS demonstrated an obvious protective effect against HF by inhibiting inflammation and oxidative damage.

As indicated by Figures 4(c) and 4(d), a significant reduction in expression of Trx2 was observed in HF and YXS and VST remarkably improved expression of Trx2 as indicated by the IF and western blotting result. And an increase in JNK activation was observed in HF which was remarkably reversed by YXS and VST treatment as evidenced by the decreased JNK phosphorylation. These results demonstrated YXS treatment inhibited oxidative damage and inflammation through restoring Trx2/JNK signaling.

### 3.4. YXS Restored Trx2 and Inhibited Activation of JNK and p38 in H9C2 Cells

**Figure 5:**
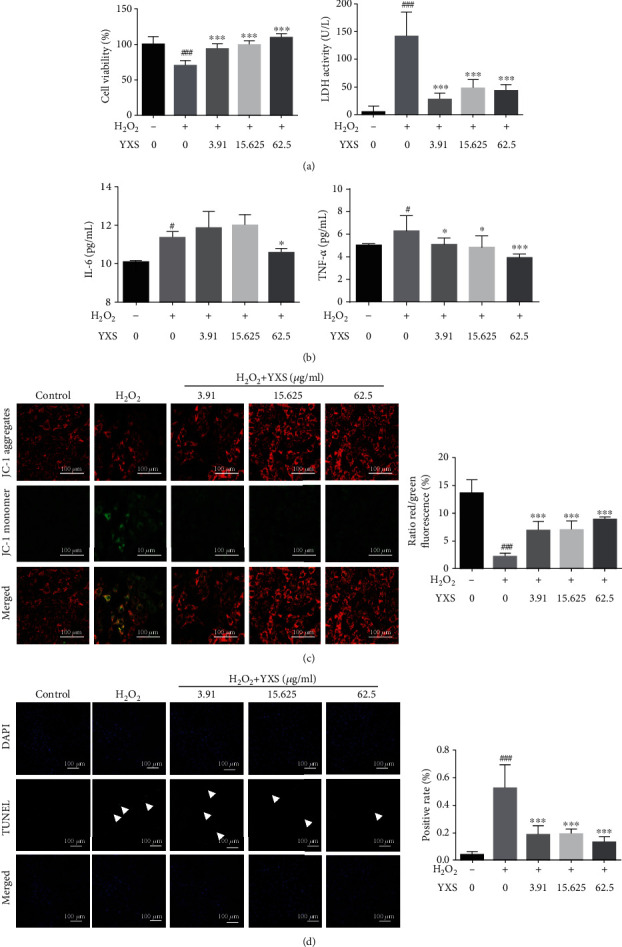
YXS inhibited inflammation and cell apoptosis in H2O2-induced H9C2 cell injury. (a) Cell viability and LDH level (*n* = 3–5); (b) levels of IL-6 and TNF-*α* (*n* = 3–5); (c) JC-1 staining and its semiquantitative data calculated by ImageJ, scale bar: 100 *μ*m (*n* = 3–5); (d) TUNEL staining and the TUNEL-positive cell rate calculated by ImageJ, scale bar: 100 *μ*m (*n* = 3–5). The data were expressed as mean ± SD. ^#^*P* < 0.05 and ^##^*P* < 0.01*vs*. normal cells; ^∗^*P* < 0.05, ^∗∗^*P* < 0.01, and ^∗∗∗^*P* < 0.001*vs*. H_2_O_2_-treated cells.

To further confirm the protection of YXS, an *in vitro* experiment by using H9C2 cells with H_2_O_2_ induction was performed. As indicated by Figure 5, H_2_O_2_ caused a significant decrease in cell viability and a rise in LDH level, whereas YXS treatment obviously enhanced cell viability and decreased LDH level. Additionally, the increase of inflammation factors such as IL-6, and TNF-*α* after H_2_O_2_ induction was obviously decreased by YXS. As indicated by Figure 5(c), YXS treatment also prevented H9C2 cells from the depolarization of mitochondrial membrane, as evidenced by the obvious increase in red/green ratio by JC-1 staining. Consistent with this result, H_2_O_2_ caused obvious increase of TUNEL-positive rate, whereas this was significantly decreased by YXS treatment (Figure 5(d)).

**Figure 6:**
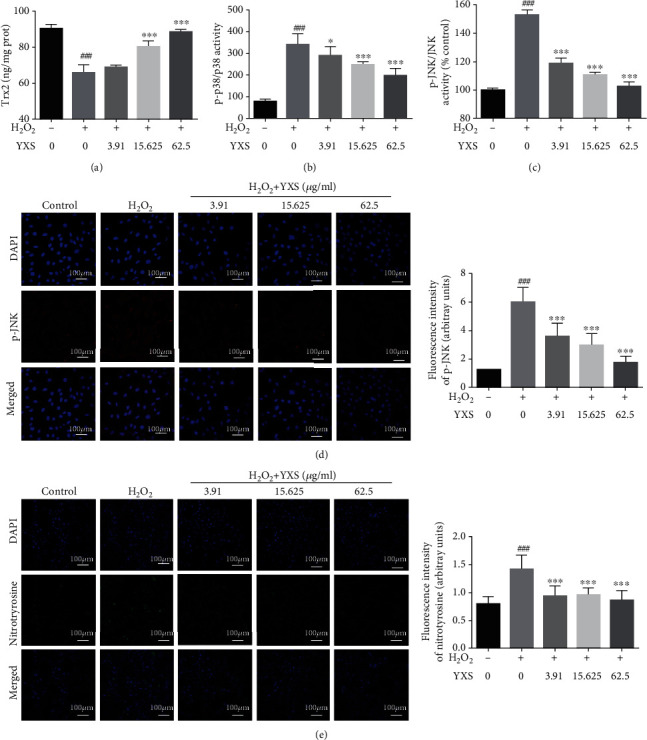
YXS increased Trx2, inhibited activation of JNK and p38, and inhibited oxidative damage in vitro. (a) ELISA result of Trx2 (*n* = 4); (b) ELISA result of p-JNK/JNK (*n* = 3); (c) ELISA result of p-p38/p38 (*n* = 6); (d) IF for p-JNK (red) and nucleus (blue), scale bar: 100 *μ*m (*n* = 3–5); (e) IF for nitrotyrosine (green) and nucleus (blue), scale bar: 100 *μ*m (*n* = 3–5). The data were expressed as mean ± SD. ^###^*P* < 0.001*vs*. normal cells; ^∗^*P* < 0.05 and ^∗∗∗^*P* < 0.001*vs.* H_2_O_2_-treated cells.

To further confirm the mechanism, we evaluated the effect of YXS on Trx2 and phosphorylation of JNK and p38 in H_2_O_2_-induced model. As indicated by Figure 6, exposure of cells to H_2_O_2_ led to an obvious reduction in Trx2, which was significantly ameliorated by YXS treatment. Additionally, the increased phosphorylation of JNK and p38 was also reduced by YXS treatment (Figures 6(b)–6(d)). Apart from that, the oxidative damage was also reduced as indicated by the decrease of nitrotyrosine (protein oxidation marker, Figure 6(e)). Thus, YXS treatment restored Trx2 and inhibited JNK and p38 phosphorylation, causing inhibition of cell apoptosis, inflammation, and oxidative damage which were consistent with our *in vivo* results. Apart from Trx2, Nrf2 is an important regulator in modulating oxidative stress and the effect of YXS on Nrf2 was evaluated accordingly. As shown in Figure S2, HF and H_2_O_2_ led to obvious reduction of Nrf2 expression whereas YXS enhanced the Nrf2 expression and enhanced its nucleus staining (Figure S2B–C).

### 3.5. Silencing Trx2 Attenuated the Protection of YXS against H_2_O_2_-Induced Cell Injury

**Figure 7:**
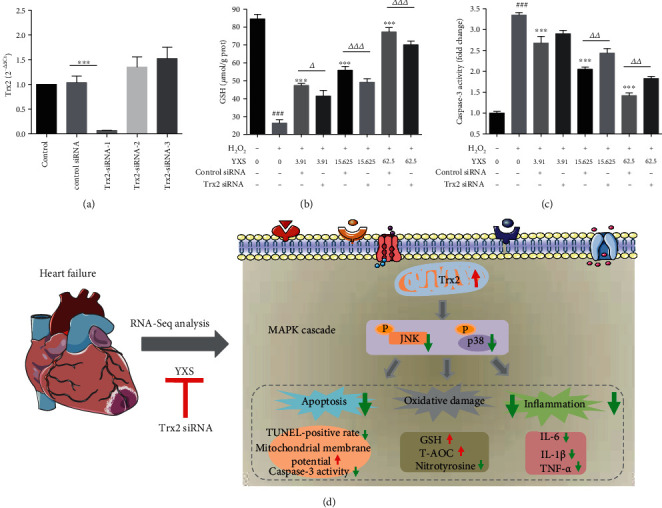
The silencing of Trx2 reduced the protective effect of YXS against H2O2-induced H9C2 cell damage. (a) RT-PCR of Trx2 expression with three sequences to silence Trx2 (*n* = 3); (b) GSH level (*n* = 5); (c) caspase-3 activity normalized to control group (*n* = 5). The data were expressed as mean ± SD. (d) Schematic diagram indicated the potential mechanism of YXS in the protection against HF. ^###^*P* < 0.01 vs. normal cells; ^∗^*P* < 0.05, ^∗∗^*P* < 0.01, and ^∗∗∗^*P* < 0.001 vs. H_2_O_2_-treated cells; *^Δ^P* < 0.05, *^ΔΔ^P* < 0.01, and *^ΔΔΔ^P* < 0.001 compared to the related control siRNA group.

To confirm the hypothesis that Trx2 played a vital role in the protection of YXS, silencing Trx2 was used to investigate the effect of YXS on H9C2 cells in the response to H_2_O_2_ (Figure 7). Three sequences to silence Trx2 were tested and Trx2 siRNA-1 successfully downregulated Trx2 expression, which was used in the following experiments. Importantly, YXS increased cellular GSH level in a concentration-dependent manner while knockdown of Trx2 significantly decreased cellular GSH level (Figure 7(b)). Additionally, the increase of caspase-3 activity in response to H_2_O_2_ was obviously decreased by different concentrations of YXS treatment. Notably, silencing Trx2 significantly increased the caspase-3 activity treated with 15.625 and 62.5 *μ*g/ml YXS (Figure 7(c)).

## 4. Discussion

In this study, YXS treatment obviously preserved cardiac function, decreased BNP and ANP levels, and attenuated structure rupture and collagen deposition. RNA-seq analysis indicated YXS protected against HF through affecting multiple processes such as oxidative damage, inflammation, apoptosis, and MAPK cascade. Further experiments confirmed that YXS decreased inflammation factors such as IL-1*β*, IL-6, and TNF-*α*, prevented cell apoptosis as evidenced by decreased TUNEL-positive rate and caspase-3 activity, and increased mitochondrial membrane potential while reducing oxidative damage as indicated by the increased levels of GSH and T-AOC. Additionally, YXS obviously elevated Trx2 while it inhibited JNK and p38 activation, thereby attenuating cardiac damage in HF. Silencing Trx2 decreased the protection of YXS in H_2_O_2_-induced cell injury as evidenced by the increase of caspase-3 activity and decrease of GSH level. Collectively, these data indicated that YXS is an effective treatment which enhanced heart function and decreased myocardial damage by restoring Trx2 and inhibiting JNK and p38 activation in ischemia-induced HF.

The ambiguous mechanism and poor clinical location hinder the further precise application of TCM in clinic, whereas systematical investigation could guide the use of TCM and benefit the treatment of patients with HF. Evidence has been accumulated that YXS is an effective treatment in the intervention of various cardiovascular diseases including myocardial ischemia/reperfusion injury, H_2_O_2_-caused damage, and ET-1-induced cardiomyocyte dysfunction [6, 13, 14]. Recent research showed YXS decreased oxidative damage and cell apoptosis and ameliorated heart function via upregulating liver-X-receptor *α* [13] while it decreased H9C2 cell apoptosis and oxidative damage by regulating APEX1, TFCP2, and 5 other transcription factors *in vitro* [15]. Our research was consistent with these results that YXS can decrease oxidative damage and apoptosis. However, the inhibition of inflammation was also proved in our study as indicated by the decreased inflammation factors such as IL-1*β*, IL-6, and TNF-*α*. Moreover, previous research indicated that YXS may improve heart function in HF with the elevation of the biomarkers such as TNNI3, GAL-3, HSP 70, FABP3, and CKAP5 [6], which benefited the clinical location of YXS. In this present study, the mechanism of YXS against HF was systematically investigated by using RNA-seq analysis and multiple processes such as oxidative damage, inflammation, apoptosis, and MAPK cascade were proved to be affected by YXS, indicating a multicomponent and multitarget way of YXS in treating HF. We also proved that YXS enhanced heart function and decreased myocardial damage by restoring Trx2 and inhibiting JNK and p38 activation against HF. All these results facilitated the understanding of the mechanism and the usage of YXS in clinic.

A large amount of studies demonstrated that increased oxidative stress and persistent inflammation are associated with the development of HF [19–22]. Oxidative stress can also lead to myocardial tissue injury and inflammation, which leads to the progress of HF [23]. Oxidative stress during HF or ischemia/reperfusion is caused by excessive production or accumulation of free radicals or their oxidative products [24]. High ROS impair myocardial contractile function, cause DNA damage and cell apoptosis, and activate matrix metalloproteinases (MMPs) which induced cardiac remodeling, augmenting the progress of HF. Moreover, ROS also induced inflammation response through regulating proinflammatory pathways such as nuclear enzyme PARP-1 and NF-KB [25]. Inhibiting oxidative damage benefits and halts HF [26], consistent with these studies that HF or H_2_O_2_ led to an increase of oxidative stress as evidenced by the lower GSH and T-AOC and higher ROS level. Importantly, an obvious inhibition of oxidative stress was observed as indicated by the increased GSH and T-AOC as well as lower ROS after YXS treatment. Additionally, it is worth mentioning that persistent inflammation after MI deteriorates ischemic damage and exaggerates cardiac remodeling in the following HF [27, 28]. Targeting inflammation has been proved to be a promising strategy in resisting HF [29–32]. Notably, a remarkable inhibition of inflammation by YXS was observed as indicated by the decrease of IL-1*β*, IL-6, and TNF-*α* level *in vivo* and *in vitro*. In addition, cell apoptosis plays an important role in cardiomyocyte loss after acute myocardial infarction and participates in the subsequent development of left ventricular remodeling and symptomatic HF [33]. Apart from that, cell apoptosis induced by inflammatory cytokines also participates in the occurrence and development of chronic HF [34]. Related studies have found clusters of biomarkers related to the mechanism of HF in the aspects of inflammation, apoptosis, vascular function, and so on [35]. Our results showed that YXS decreased the TUNEL-positive rate and caspase-3 activity while it increased the mitochondrial membrane potential, which demonstrated an obvious inhibition of cell apoptosis by YXS. Thus, YXS treatment showed a beneficial protection on HF through inhibiting oxidative damage, inflammation, and apoptosis.

Thioredoxin system, including Trx, peroxidase Prx, and thioredoxin reductase (TrxR), plays a key role in modulating redox hemostasis and gene knockout of Trxs and TrxRs led to embryonic lethality due to elevation of oxidative stress [36–38]. Specifically, Trx2 is located in mitochondria and is characterized by a dithiol motif Cys-Gly-Pro-Cys, which serves a critical function in cell survival and apoptosis in cardiomyocytes [26, 39]. Mitochondrial Trx2 deficiency led to extensive apoptosis and embryonic lethality [40]. In contrast, the overexpression of Trx2 preserved cardiac function and prevented cell apoptosis through inhibiting ROS generation in dilated cardiomyopathy and HF [26]. Recent studies indicated decreased Trx2 attenuated inflammation after liver ischemia/reperfusion injury through mitochondrial quality [41], and PGC-1 can upregulate Trx2 expression in the protection against transverse aortic constriction-induced oxidative damage [42], whereas TXNIP shuttled to bind to Trx2 and caused inflammation and apoptosis [43]. In the present study, the expression of Trx2 was decreased in both H_2_O_2_-treated H9C2 and MI-induced HF which was associated with increased oxidative damage, inflammation, and apoptosis, whereas increased Trx2 by YXS obviously attenuated these damages and halted progress of HF. Additionally, Trx2 silencing attenuated the protection of YXS in H_2_O_2_-induced cell injury as evidenced by decreased GSH and increased caspase-3 activity. These data indicated that Trx2 played an important role in the protection of YXS against MI-induced HF. Additionally, Nrf2 played a critical role in regulating oxidative stress and a close relationship between Nrf2 and Trx2 in modulating cellular oxidative stress was observed. For example, Nrf2 activation prevented Trx2 oxidation while Trx2 overexpression affected Nrf2 activity [44–46]. In our research, YXS enhanced Nrf2 activation both *in vitro* and *in vivo*, which protected myocardiocytes against damage from myocardial ischemia or H_2_O_2_.

MAPK is a common pathway of extracellular signal-induced cell response, which participates in many biological processes such as cell proliferation, differentiation, apoptosis, and cytoskeleton reorganization. In several subfamilies of MAPK cascade signaling pathway, p38 and JNK are associated with oxidative stress and inflammation. The p38 MAPK signal pathway plays a vital role in maintaining normal heart function. However, p38 is also involved in the pathogenesis of HF by affecting the heart contraction and survival of cardiomyocytes [47]. Recent research suggested that inhibition of p38 MAPK activation showed a beneficial effect on HF [48]. Our study indicated that YXS has an inhibitory effect on p38 activation, which helped to halt HF progression. Apart from that, JNK activation resulted in cell apoptosis [49]. Growing evidence shows that increased Trx levels prevent ASK1 activation and subsequently inhibit the activation of the downstream p38 and JNK pathways [50, 51]. However, it indicated that the inhibition of Trx2 on cell apoptosis was not in a JNK-dependent manner in dilated cardiomyopathy [26, 52]. In contrast to these studies, we found that increased Trx2 was associated with decreased p-JNK/JNK and p-p38/p38 in YXS-mediated protection against HF. This may be caused by the multicomponent and multitarget characteristics of YXS, which maintained cardiac function through affecting multiple pathways. Recent studies indicated activated JNK caused cell apoptosis through phosphorylating c-Jun and upregulating Fas ligand (FasL) [53, 54]. Therefore, YXS inhibited the activation of JNK and p38, resulting in an important protective effect in resisting HF.

## 5. Conclusions

In summary, YXS enhanced myocardial function, decreased BNP and ANP levels, and attenuated structure rupture and collagen deposition, through restoring Trx2 and inhibiting JNK and p38 activation in ischemia-induced HF. This study clarified the important role of YXS in the management of HF and its potential mechanism, facilitating the clinical usage of YXS. Additionally, this also provided a better understanding of the pathogenesis of HF, highlighting the importance of restoring Trx2 in HF.

## Data Availability

All data in this study can be obtained from the authors.

## Abbreviations

YXS: Yixin-shu
HF: Heart failure
IL-1*β*: Interleukin-1*β*
IL-6: Interleukin 6
TNF-*α*: Tumor necrosis factor-*α*
LDH: Lactate dehydrogenase
GSH: Glutathione
T-AOC: Total antioxidant capacity
BNP: Brain natriuretic peptide
ANP: Atrial natriuretic peptide
TCM: Traditional Chinese medicine
MI: Myocardial ischemia
H2O2: Hydrogen peroxide
Trx2: Thioredoxin 2
IF: Immunofluorescence staining
DEs: Differentially expressed genes
ROS: Reactive oxygen species
H&E: Hematoxylin and eosin
VST: Valsartan
SD: Sprague-Dawley
KEGG: Kyoto Encyclopedia of Genes and Genomes
GO: Gene Ontology
ELISA: Enzyme-linked immunosorbent assay.

## Supplementary Materials

Supplementary Materials Antibodies such as Nrf2 (ab89443), Tlr4 (19811-1-AP), and Myd88 (sc-74532) were used for IF staining. As for F-actin staining, the samples were incubated with 0.1% Triton X-100 for 15 min. Rhodamine phalloidin (PHDR1, cytoskeleton) was used for the staining of F-actin after the treatment of 0.1% Triton X-100 for 15 min and then followed by 4,6-DAPI for 10 min before observation. Table S1: the RNA-seq data of failing heart treated with or without YXS or VST. Figure S1: the enrichment of DEs in YXS-mediated protection against H_2_O_2_-induced damage; (A) enriched GO terms of upregulated DEs; (B) enriched GO terms of downregulated DEs. Figure S2: YXS decreased the levels of Tlr4 and Myd88, enhanced Nrf2 expression, and improved cytoskeleton arrangement; (A) the IF staining of Tlr4 (red) and Myd88 (green) in H_2_O_2_-induced H9C2 cell and the related quantification, nucleus (blue), scale bar: 100 *μ*m (*n* = 3–5); (B) the IF staining of Nrf2 (green) and F-actin (red) in heart tissue, nucleus (blue), scale bar: 100 *μ*m; (C) the IF staining of Nrf2 (green) in H_2_O_2_-induced H9C2 cell and the related quantification, nucleus (blue), scale bar: 100 *μ*m (*n* = 3–5).

## References

[1] Mathers C. D., Loncar D. (2006). Projections of global mortality and burden of disease from 2002 to 2030. *PLoS Medicine*.

[2] Kurokawa J., Abriel H. (2009). Neurohormonal regulation of cardiac ion channels in chronic heart failure. *Journal of Cardiovascular Pharmacology.*.

[3] Pagliaro B. R., Cannata F., Stefanini G. G., Bolognese L. (2020). Myocardial ischemia and coronary disease in heart failure. *Heart Failure Reviews*.

[4] Dube P., Weber K. T., Weber K. T. (2011). Congestive heart failure: pathophysiologic consequences of neurohormonal activation and the potential for recovery: part II. *The American Journal of the Medical Sciences*.

[5] Murphy S. P., Ibrahim N. E., Januzzi J. L. (2020). Heart failure with reduced ejection fraction: a review. *Journal of the American Medical Association*.

[6] Wei J., Guo F., Zhang M. (2018). Signature-oriented investigation of the efficacy of multicomponent drugs against heart failure. *The FASEB Journal*.

[7] Shen N., Li X., Zhou T. (2014). Shensong Yangxin capsule prevents diabetic myocardial fibrosis by inhibiting TGF-*β*1/Smad signaling. *Journal of Ethnopharmacology*.

[8] Zhang L., Wang Y., Yu L. (2010). QI-SHEN-YI-QI accelerates angiogenesis after myocardial infarction in rats. *International Journal of Cardiology*.

[9] Mao J., Zhang J., Lam C. S. P. (2020). Qishen Yiqi dripping pills for chronic ischaemic heart failure: results of the CACT-IHF randomized clinical trial. *ESC Heart Fail*.

[10] Li X., Zhang J., Huang J. (2013). A multicenter, randomized, double-blind, parallel-group, placebo-controlled study of the effects of qili qiangxin capsules in patients with chronic heart failure. *Journal of the American College of Cardiology*.

[11] Zhang J., Guo F., Wu H. (2018). Yixin-Shu facilitated cardiac-like differentiation of mesenchymal stem cellsin vitro. *RSC Advances*.

[12] Committee C P (2015). *China Pharmacopoeia*.

[13] Zhao Y., Xu L., Qiao Z. (2016). YiXin-Shu, a ShengMai-San-based traditional Chinese medicine formula, attenuates myocardial ischemia/reperfusion injury by suppressing mitochondrial mediated apoptosis and upregulating liver-X-receptor *α*. *Scientific Reports*.

[14] Zhang M., Wu H., Guo F. (2017). Identification of active components in Yixinshu capsule with protective effects against myocardial dysfunction on human induced pluripotent stem cell-derived cardiomyocytes by an integrative approach. *Molecular BioSystems*.

[15] Zhang J., Geng Y., Guo F. (2017). Screening and identification of critical transcription factors involved in the protection of cardiomyocytes against hydrogen peroxide-induced damage by Yixin-shu. *Scientific Reports*.

[16] Zhang J., Guo F., Wei J. (2017). An integrated approach to identify critical transcription factors in the protection against hydrogen peroxide-induced oxidative stress by Danhong injection. *Free Radical Biology & Medicine*.

[17] Robinson M. D., McCarthy D. J., Smyth G. K. (2009). edgeR: a Bioconductor package for differential expression analysis of digital gene expression data. *Biogeosciences*.

[18] Huang D. W., Sherman B. T., Lempicki R. A. (2009). Systematic and integrative analysis of large gene lists using DAVID bioinformatics resources. *Nature Protocols*.

[19] Mann D. L., Bristow M. R. (2005). Mechanisms and models in heart failure. *Circulation*.

[20] Santos C. X. C., Anilkumar N., Zhang M., Brewer A. C., Shah A. M. (2011). Redox signaling in cardiac myocytes. *Free Radical Biology & Medicine*.

[21] van der Pol A., van Gilst W. H., Voors A. A., van der Meer P. (2019). Treating oxidative stress in heart failure: past, present and future. *European Journal of Heart Failure*.

[22] Ayoub K. F., Pothineni N. V. K., Rutland J., Ding Z., Mehta J. L. (2017). Immunity, inflammation, and oxidative stress in heart failure: emerging molecular targets. *Cardiovascular Drugs and Therapy*.

[23] Aimo A., Castiglione V., Borrelli C. (2020). Oxidative stress and inflammation in the evolution of heart failure: from pathophysiology to therapeutic strategies. *European Journal of Preventive Cardiology*.

[24] Neri M., Fineschi V., Di Paolo M. (2015). Cardiac oxidative stress and inflammatory cytokines response after myocardial infarction. *Current Vascular Pharmacology*.

[25] Rajesh M., Mukhopadhyay P., Bátkai S. (2010). Cannabidiol attenuates cardiac dysfunction, oxidative stress, fibrosis, and inflammatory and cell death signaling pathways in diabetic cardiomyopathy. *Journal of the American College of Cardiology*.

[26] Huang Q., Zhou H. J., Zhang H. (2015). Thioredoxin-2 inhibits mitochondrial reactive oxygen species generation and apoptosis stress kinase-1 activity to maintain cardiac function. *Circulation*.

[27] Ruparelia N., Chai J. T., Fisher E. A., Choudhury R. P. (2017). Erratum: inflammatory processes in cardiovascular disease: a route to targeted therapies. *Nature Reviews. Cardiology*.

[28] Frangogiannis N. G. (2012). Regulation of the inflammatory response in cardiac repair. *Circulation Research*.

[29] Hamid T., Gu Y., Ortines R. V. (2009). Divergent tumor necrosis factor receptor-related remodeling responses in heart failure: role of nuclear factor-kappaB and inflammatory activation. *Circulation*.

[30] Leuschner F., Dutta P., Gorbatov R. (2011). Therapeutic siRNA silencing in inflammatory monocytes in mice. *Nature Biotechnology*.

[31] Wang J., Seo M. J., Deci M. B., Weil B. R., Canty J. M., Nguyen J. (2018). Effect of CCR2 inhibitor-loaded lipid micelles on inflammatory cell migration and cardiac function after myocardial infarction. *International Journal of Nanomedicine*.

[32] Wang L., Zhang Y. L., Lin Q.-Y. (2019). CXCL1–CXCR2 axis mediates angiotensin II-induced cardiac hypertrophy and remodelling through regulation of monocyte infiltration. *European Heart Journal*.

[33] Teringova E., Tousek P. (2017). Apoptosis in ischemic heart disease. *Journal of Translational Medicine*.

[34] Chen C., Zong M., Lu Y. (2020). Differentially expressed lnc-NOS2P3-miR-939-5p axis in chronic heart failure inhibits myocardial and endothelial cells apoptosis via iNOS/TNF*α* pathway. *Journal of Cellular and Molecular Medicine*.

[35] Ferreira J. P., Verdonschot J., Collier T. (2019). Proteomic bioprofiles and mechanistic pathways of progression to heart failure. *Circulation. Heart Failure*.

[36] Lee S., Kim S. M., Lee R. T. (2013). Thioredoxin and thioredoxin target proteins: from molecular mechanisms to functional significance. *Antioxidants & Redox Signaling*.

[37] Zhang J., Zhou R., Xiang C. (2020). Enhanced thioredoxin, glutathione and Nrf2 antioxidant systems by safflower extract and aceglutamide attenuate cerebral ischaemia/reperfusion injury. *Journal of Cellular and Molecular Medicine*.

[38] Jakupoglu C., Przemeck G. K., Schneider M. (2005). Cytoplasmic thioredoxin reductase is essential for embryogenesis but dispensable for cardiac development. *Molecular and Cellular Biology*.

[39] Ago T., Sadoshima J. (2006). Thioredoxin and ventricular remodeling. *Journal of Molecular and Cellular Cardiology*.

[40] Nonn L., Williams R. R., Erickson R. P., Powis G. (2003). The absence of mitochondrial thioredoxin 2 causes massive apoptosis, exencephaly, and early embryonic lethality in homozygous mice. *Molecular and Cellular Biology*.

[41] Kang J. W., Choi H. S., Lee S. M. (2018). Resolvin D1 attenuates liver ischaemia/reperfusion injury through modulating thioredoxin 2-mediated mitochondrial quality control. *British Journal of Pharmacology*.

[42] Lu Z., Xu X., Hu X. (2010). PGC-1*α* regulates expression of myocardial mitochondrial antioxidants and myocardial oxidative stress after chronic systolic overload. *Antioxidants&Redox Signalling.*.

[43] Xu L., Lin X., Guan M., Zeng Y., Liu Y. (2019). Verapamil attenuated prediabetic neuropathy in high-fat diet-fed mice through inhibiting TXNIP-mediated apoptosis and inflammation. *Oxidative Medicine and Cellular Longevity*.

[44] Harris C., Hansen J. M. (2012). Nrf2-mediated resistance to oxidant-induced redox disruption in embryos. *Birth Defects Research. Part B, Developmental and Reproductive Toxicology*.

[45] Imhoff B. R., Hansen J. M. (2010). *tert*-Butylhydroquinone induces mitochondrial oxidative stress causing Nrf2 activation. *Cell Biology and Toxicology*.

[46] Imhoff B. R., Hansen J. M. (2009). Extracellular redox status regulates Nrf2 activation through mitochondrial reactive oxygen species. *The Biochemical Journal*.

[47] Dumont A. A., Dumont L., Berthiaume J., Auger-Messier M. (2019). p38*α* MAPK proximity assay reveals a regulatory mechanism of alternative splicing in cardiomyocytes. *Biochimica et Biophysica Acta (BBA) - Molecular Cell Research*.

[48] Arabacilar P., Marber M. (2015). The case for inhibiting p38 mitogen-activated protein kinase in heart failure. *Frontiers in Pharmacology*.

[49] Kwon S. H., Pimentel D. R., Remondino A., Sawyer D. B., Colucci W. S. (2003). H_2_O_2_ regulates cardiac myocyte phenotype via concentration-dependent activation of distinct kinase pathways. *Journal of Molecular and Cellular Cardiology*.

[50] Lu J., Holmgren A. (2014). The thioredoxin antioxidant system. *Free Radical Biology & Medicine*.

[51] Commission G O o N H (2020). Novel coronavirus pneumonia diagnosis and treatment plan(version 7).

[52] Zhang R., al-Lamki R., Bai L. (2004). Thioredoxin-2 inhibits mitochondria-located ASK1-mediated apoptosis in a JNK-independent manner. *Circulation Research*.

[53] Pei D. S., Wang X. T., Liu Y. (2006). Neuroprotection against ischaemic brain injury by a GluR6-9c peptide containing the TAT protein transduction sequence. *Brain*.

[54] Yu H. M., Xu J., Li C. (2008). Coupling between neuronal nitric oxide synthase and glutamate receptor 6-mediated c-Jun N-terminal kinase signaling pathway via S-nitrosylation contributes to ischemia neuronal death. *Neuroscience*.

