# Supplementary material for: Yixin-Shu Capsules Ameliorated Ischemia-Induced Heart Failure by Restoring Trx2 and Inhibiting JNK/p38 Activation
Source: Oxid Med Cell Longev. 2021 Feb 16;2021:8049079. doi: 10.1155/2021/8049079 (PMC7902134; doi:10.1155/2021/8049079)
Supplement: Supplementary Materials — Antibodies such as Nrf2 (ab89443), Tlr4 (19811-1-AP), and Myd88 (sc-74532) were used for IF staining. As for F-actin staining, the samples were incubated with 0.1% Triton X-100 for 15 min. Rhodamine phalloidin (PHDR1, cytoskeleton) was used for the staining of F-actin after the treatment of 0.1% Triton X-100 for 15 min and then followed by 4,6-DAPI for 10 min before observation. Table S1: the RNA-seq data of failing heart treated with or without YXS or VST. Figure S1: the enrichment of DEs in YXS-mediated protection against H2O2-induced damage; (A) enriched GO terms of upregulated DEs; (B) enriched GO terms of downregulated DEs. Figure S2: YXS decreased the levels of Tlr4 and Myd88, enhanced Nrf2 expression, and improved cytoskeleton arrangement; (A) the IF staining of Tlr4 (red) and Myd88 (green) in H2O2-induced H9C2 cell and the related quantification, nucleus (blue), scale bar: 100 μm (n = 3–5); (B) the IF staining of Nrf2 (green) and F-actin (red) in heart tissue, nucleus (blue), scale bar: 100 μm; (C) the IF staining of Nrf2 (green) in H2O2-induced H9C2 cell and the related quantification, nucleus (blue), scale bar: 100 μm (n = 3–5). [file 8049079.f1.zip › Xiang.Table S1 HF vs Normal.pdf]

HF VS Normal

Gene	FDR	LR	PValue	logCPM	logFC
Cntn2	6.09E-06	2.70E+01	2.03E-07	1.913046	1.30E+00
Ankrd2	1.95E-05	2.46E+01	7.05E-07	0.248852	1.83E+00
Itga10	1.64E-07	3.45E+01	4.21E-09	0.229267	2.28E+00
LOC100912	1.87E-02	9.98E+00	1.58E-03	-1.58949	2.20E+00
Crym	2.66E-43	2.02E+02	7.80E-46	5.287436	1.06E+00
Rab15	4.37E-02	8.12E+00	4.37E-03	-1.09953	1.85E+00
Ptgs2	6.23E-15	6.96E+01	7.26E-17	1.822978	2.15E+00
Prg4	4.17E-06	2.78E+01	1.36E-07	2.374763	1.07E+00
LOC680663	2.42E-02	9.42E+00	2.15E-03	-2.4407	4.59E+00
RGD156166	9.04E-04	1.65E+01	4.84E-05	-0.29769	1.74E+00
Slamf9	4.55E-04	1.80E+01	2.24E-05	1.456927	1.11E+00
Gpr183	1.26E-03	1.58E+01	6.99E-05	1.161678	1.08E+00
Islr2	7.31E-11	5.05E+01	1.22E-12	0.531237	2.67E+00
Nmnat2	4.78E-03	1.30E+01	3.19E-04	-0.14546	1.50E+00
Necab3	1.74E-03	1.51E+01	1.01E-04	1.383293	1.00E+00
B4galnt3	2.50E-07	3.36E+01	6.63E-09	-0.5988	3.64E+00
Postn	########	5.51E+02	########	4.86533	2.10E+00
Svep1	2.79E-10	4.77E+01	4.97E-12	2.916498	1.08E+00
Gfap	2.59E-04	1.92E+01	1.18E-05	-0.11879	1.80E+00
H3f3c	6.49E-14	6.48E+01	8.19E-16	3.723686	1.01E+00
AABR07035	1.13E-03	1.61E+01	6.15E-05	-1.42667	3.46E+00
Nod2	3.30E-02	8.74E+00	3.11E-03	-0.1499	1.40E+00
Cpz	2.12E-04	1.96E+01	9.46E-06	1.442207	1.31E+00
Hapln1	4.78E-03	1.30E+01	3.19E-04	0.646863	1.11E+00
Cd33	5.61E-03	1.26E+01	3.86E-04	0.973578	1.04E+00
Csrp2	1.53E-22	1.05E+02	1.03E-24	3.650553	1.27E+00
Cilp	5.69E-68	3.16E+02	1.00E-70	4.371508	2.15E+00
Kcnt1	4.05E-02	8.29E+00	3.99E-03	0.110081	1.03E+00
Cp	1.69E-41	1.94E+02	5.21E-44	4.692023	1.33E+00
Mis18a	3.67E-06	2.80E+01	1.19E-07	-0.04654	2.71E+00
Cacna1g	2.94E-40	1.88E+02	9.26E-43	4.463507	1.43E+00
Olfm2	2.56E-02	9.30E+00	2.29E-03	0.248813	1.15E+00
Adamts8	8.05E-10	4.55E+01	1.52E-11	1.141437	2.14E+00
Cdkl2	9.70E-05	2.12E+01	4.04E-06	1.88144	1.09E+00
NEWGENE_1	3.04E-02	8.92E+00	2.83E-03	1.253773	1.91E+00
Plekha4	9.80E-17	7.81E+01	9.77E-19	4.289194	1.10E+00
Tmem45b	4.30E-04	1.81E+01	2.09E-05	-1.67502	3.94E+00
Gdf6	3.37E-06	2.82E+01	1.08E-07	0.164192	2.18E+00
Cdhr1	1.75E-09	4.39E+01	3.44E-11	1.412175	1.78E+00
Mybpc2	3.99E-17	8.00E+01	3.80E-19	2.25724	1.89E+00
Tnfsf18	3.01E-03	1.40E+01	1.87E-04	-0.34547	1.78E+00
Kctd15	1.07E-07	3.54E+01	2.65E-09	2.414659	1.14E+00
Tmem119	2.55E-19	9.03E+01	2.06E-21	1.031421	3.27E+00
Pcdhb21	4.61E-02	8.00E+00	4.68E-03	0.198017	1.00E+00
Trappc2b	4.07E-02	8.28E+00	4.01E-03	0.552562	2.02E+00
Testin	4.76E-05	2.27E+01	1.85E-06	-0.61837	2.57E+00
Fxyd7	4.32E-04	1.81E+01	2.11E-05	1.482888	1.06E+00
Egr2	3.03E-13	6.17E+01	4.07E-15	2.52569	1.59E+00
Ccl20	6.86E-03	1.22E+01	4.87E-04	-0.74968	2.01E+00
Slc44a3	2.24E-02	9.59E+00	1.96E-03	-0.53629	1.50E+00
Tceal7	6.24E-73	3.39E+02	9.83E-76	4.12297	2.21E+00

LOC10091C	4.92E-02	7.85E+00	5.07E-03	1.484368	1.29E+00
Lrrc15	1.66E-02	1.02E+01	1.37E-03	-2.26016	4.92E+00
Glyatl1	6.81E-03	1.22E+01	4.82E-04	-1.03941	1.95E+00
Mfap5	4.43E-58	2.70E+02	9.70E-61	5.184714	1.65E+00
Klkb1	6.14E-05	2.22E+01	2.44E-06	-0.03291	1.93E+00
Adra2a	2.08E-03	1.48E+01	1.23E-04	-0.61688	1.90E+00
AABR07057	3.01E-02	8.94E+00	2.79E-03	-1.26999	2.69E+00
Cdkn2b	1.07E-05	2.58E+01	3.72E-07	-0.3826	2.52E+00
RGD156272	1.38E-02	1.06E+01	1.10E-03	-0.61319	1.64E+00
Sfrp2	########	6.85E+02	########	4.496921	3.26E+00
Mxra8	7.13E-67	3.11E+02	1.30E-69	6.019204	1.11E+00
Tnfaip8l3	5.39E-03	1.27E+01	3.69E-04	0.940736	1.02E+00
Runx2	2.70E-03	1.42E+01	1.65E-04	0.778823	1.20E+00
Mybphl	1.15E-10	4.95E+01	1.95E-12	-0.23925	3.77E+00
Bmp8a	1.52E-03	1.54E+01	8.61E-05	-2.13823	5.19E+00
Pqlc3	3.01E-10	4.75E+01	5.39E-12	3.168731	1.17E+00
Olfml2b	8.99E-16	7.36E+01	9.73E-18	3.617023	1.08E+00
Pnma2	3.77E-02	8.45E+00	3.66E-03	-0.41488	1.36E+00
RGD156613	2.65E-02	9.23E+00	2.39E-03	-1.98013	2.55E+00
Ctsk	1.78E-59	2.77E+02	3.78E-62	4.721871	1.53E+00
Egr3	2.44E-09	4.32E+01	4.90E-11	2.363404	1.29E+00
Col17a1	6.21E-10	4.60E+01	1.15E-11	1.705884	1.75E+00
AABR07034	2.96E-06	2.85E+01	9.38E-08	-0.90587	4.17E+00
Ncam1	1.04E-98	4.59E+02	########	5.117889	1.68E+00
Prrt2	4.46E-03	1.31E+01	2.95E-04	1.025319	1.05E+00
Chad	1.94E-24	1.14E+02	1.13E-26	2.559435	2.08E+00
Abl1	4.29E-03	1.32E+01	2.83E-04	2.814852	1.10E+00
Dmp1	3.11E-08	3.80E+01	7.19E-10	1.536953	1.69E+00
C1qtnf5	3.05E-63	2.94E+02	5.94E-66	4.062056	2.10E+00
Vgll2	3.73E-05	2.33E+01	1.42E-06	-1.84158	5.69E+00
Kcne1	8.71E-16	7.36E+01	9.41E-18	0.485132	3.62E+00
LOC10369C	1.33E-02	1.07E+01	1.06E-03	-0.53314	2.30E+00
Mustn1	1.94E-13	6.26E+01	2.55E-15	3.574119	1.01E+00
Capn6	3.75E-03	1.35E+01	2.41E-04	0.105981	1.48E+00
LOC103693	2.96E-02	8.98E+00	2.74E-03	0.017483	2.52E+00
Ptgfr	3.15E-92	4.29E+02	3.30E-95	6.681162	1.14E+00
Piezo2	3.72E-06	2.80E+01	1.21E-07	1.462306	1.56E+00
LOC688459	4.61E-02	8.00E+00	4.67E-03	-2.59062	4.21E+00
Hamp	3.10E-22	1.04E+02	2.14E-24	3.551544	1.41E+00
Mup5	3.39E-02	8.68E+00	3.21E-03	-0.86577	1.52E+00
Dusp15	1.17E-10	4.95E+01	1.99E-12	3.021942	1.12E+00
Aldh1a3	6.00E-12	5.56E+01	8.96E-14	3.004609	1.14E+00
Fam111a	########	5.25E+02	########	6.011028	1.45E+00
Mrgpre	1.87E-02	9.99E+00	1.57E-03	-1.84169	2.80E+00
Cercam	2.87E-12	5.71E+01	4.16E-14	3.339396	1.01E+00
Thbs4	########	8.70E+02	########	4.475331	3.93E+00
Fzd2	4.03E-17	7.99E+01	3.85E-19	3.298657	1.25E+00
Prrx2	2.19E-14	6.70E+01	2.69E-16	1.177821	2.82E+00
LOC10255C	5.15E-23	1.08E+02	3.37E-25	4.910582	1.35E+00
Cgref1	2.86E-16	7.59E+01	2.97E-18	1.036145	2.97E+00
Mmp16	6.19E-04	1.73E+01	3.17E-05	-0.71776	2.06E+00
Pi16	0.00E+00	1.76E+03	0.00E+00	6.924527	2.43E+00
Slc25a27	1.95E-02	9.89E+00	1.66E-03	0.737275	1.09E+00
Syt15	4.83E-02	7.89E+00	4.96E-03	-0.50083	1.22E+00

Mfsd7	1.36E-02	1.07E+01	1.08E-03	0.78787	1.09E+00
Vtcn1	3.22E-02	8.79E+00	3.03E-03	-0.98767	1.66E+00
Tnc	4.09E-26	1.22E+02	2.24E-28	2.598352	2.17E+00
Ighv8-4	7.63E-04	1.69E+01	4.00E-05	-1.79448	3.75E+00
Actg2	4.00E-36	1.69E+02	1.38E-38	3.421713	1.74E+00
AC103179.	4.81E-04	1.79E+01	2.39E-05	0.604593	1.36E+00
Aspa	3.97E-02	8.33E+00	3.89E-03	0.271564	1.11E+00
Wasf1	2.26E-02	9.58E+00	1.97E-03	-2.03358	3.35E+00
Grin3a	9.97E-04	1.63E+01	5.38E-05	-2.02658	5.40E+00
Grem1	2.31E-08	3.86E+01	5.26E-10	-0.49319	3.46E+00
Pla2g2a	4.94E-72	3.35E+02	8.08E-75	5.89477	1.25E+00
Sphk1	1.28E-05	2.55E+01	4.51E-07	1.978259	1.20E+00
Ighm	5.71E-29	1.35E+02	2.77E-31	3.705479	1.45E+00
Kif1a	4.91E-03	1.29E+01	3.30E-04	-0.51239	1.77E+00
Ighv1-47	3.59E-05	2.33E+01	1.36E-06	-1.8436	5.68E+00
Surf2	8.85E-03	1.16E+01	6.55E-04	2.395601	1.99E+00
Brms1l	2.78E-03	1.41E+01	1.71E-04	1.656172	1.03E+00
Trh	1.04E-34	1.62E+02	3.91E-37	1.879195	3.93E+00
Fam167a	3.92E-04	1.83E+01	1.89E-05	1.872941	1.01E+00
Nrip3	2.98E-03	1.40E+01	1.85E-04	0.41327	1.38E+00
Ltbp2	0.00E+00	3.13E+03	0.00E+00	6.169502	4.33E+00
Serpinf1	########	8.34E+02	########	6.398549	1.72E+00
LOC100911	6.32E-03	1.23E+01	4.41E-04	-2.31759	4.84E+00
Adamtsl2	3.23E-42	1.97E+02	9.86E-45	4.190442	1.54E+00
Col11a1	1.10E-73	3.43E+02	1.70E-76	2.529939	4.92E+00
Ttc29	5.73E-04	1.75E+01	2.91E-05	-1.11315	2.61E+00
Crispld1	3.59E-05	2.33E+01	1.36E-06	0.316487	2.12E+00
Scrg1	3.95E-02	8.35E+00	3.86E-03	-2.37115	4.78E+00
AABR07011	3.88E-10	4.70E+01	7.03E-12	4.162463	3.39E+00
Gxylt2	3.78E-05	2.32E+01	1.44E-06	2.276866	1.15E+00
Cnn1	3.14E-17	8.05E+01	2.93E-19	2.935993	1.40E+00
Acp5	1.31E-12	5.87E+01	1.87E-14	1.939266	1.82E+00
Myl7	3.05E-27	1.27E+02	1.58E-29	4.514814	1.17E+00
Ptprv	9.80E-17	7.81E+01	9.76E-19	0.597361	3.75E+00
Grm4	1.71E-03	1.52E+01	9.89E-05	0.43853	1.31E+00
Gdf10	8.64E-07	3.11E+01	2.48E-08	-0.08998	2.37E+00
Chodl	2.17E-14	6.70E+01	2.65E-16	0.289599	3.71E+00
Kcnma1	7.40E-04	1.69E+01	3.85E-05	0.51407	1.79E+00
Wisp1	5.55E-08	3.68E+01	1.34E-09	-0.15251	2.83E+00
Ypel4	8.83E-05	2.15E+01	3.63E-06	1.816178	1.08E+00
Olfml3	6.47E-17	7.90E+01	6.31E-19	3.718201	1.09E+00
Jakmip3	4.93E-02	7.85E+00	5.08E-03	-2.58728	4.23E+00
Mir199a2	1.71E-02	1.02E+01	1.42E-03	-0.88664	1.63E+00
Ntrk3	1.14E-04	2.09E+01	4.78E-06	0.888534	1.36E+00
Lnpep	2.84E-02	9.07E+00	2.60E-03	0.650927	1.44E+00
Zfp383	4.56E-02	8.02E+00	4.61E-03	-1.29939	2.28E+00
AABR07065	6.95E-05	2.20E+01	2.79E-06	-1.56152	4.12E+00
Myl4	3.23E-78	3.64E+02	4.49E-81	3.766406	2.65E+00
Nlrp3	2.23E-05	2.43E+01	8.16E-07	1.321428	1.34E+00
Mepe	1.83E-06	2.95E+01	5.58E-08	-0.51527	2.71E+00
Mmp12	2.63E-05	2.40E+01	9.70E-07	-1.29351	3.67E+00
LOC10090	2.15E-02	9.68E+00	1.86E-03	-2.44125	4.59E+00
Sectm1b	1.26E-08	3.98E+01	2.76E-10	-0.28101	3.45E+00
Olr1	4.52E-02	8.05E+00	4.56E-03	0.353593	1.07E+00

Ccl12	4.27E-02	8.17E+00	4.26E-03	-0.19889	1.23E+00
Comp	########	9.42E+02	########	4.269184	4.74E+00
Crlf1	2.56E-17	8.09E+01	2.35E-19	3.929631	1.10E+00
Grem2	1.78E-03	1.51E+01	1.03E-04	-0.75321	2.01E+00
Ccdc80	########	4.88E+02	########	8.14283	1.14E+00
Cldn4	1.75E-02	1.01E+01	1.45E-03	-2.44509	4.57E+00
Clec11a	1.19E-81	3.80E+02	1.47E-84	3.863041	2.82E+00
Zfp697	3.61E-05	2.33E+01	1.38E-06	-1.48158	6.24E+00
Ina	1.27E-02	1.08E+01	9.94E-04	-2.38149	4.70E+00
Mmp9	4.60E-12	5.61E+01	6.78E-14	0.881583	2.65E+00
Slit2	5.78E-06	2.71E+01	1.92E-07	2.139192	1.09E+00
Grhl3	1.06E-03	1.62E+01	5.76E-05	-0.84409	2.25E+00
Xirp2	6.10E-79	3.67E+02	7.91E-82	8.966698	1.09E+00
Clec2d	2.60E-16	7.61E+01	2.68E-18	-0.35283	7.65E+00
AABR07051	2.13E-03	1.47E+01	1.26E-04	-2.09082	5.26E+00
Dnah7	1.25E-08	3.99E+01	2.72E-10	2.420168	1.23E+00
Ngef	1.32E-02	1.07E+01	1.05E-03	0.655218	1.08E+00
Tnmd	3.11E-03	1.39E+01	1.95E-04	-1.63103	3.16E+00
Fstl3	1.56E-07	3.46E+01	3.99E-09	2.824591	1.15E+00
B4gat1	2.44E-02	9.40E+00	2.17E-03	-2.19303	3.03E+00
Prkg2	3.90E-04	1.83E+01	1.87E-05	-2.08402	5.28E+00
Slc1a7	2.28E-17	8.12E+01	2.08E-19	3.091449	1.31E+00
Bmpr1b	3.25E-03	1.38E+01	2.05E-04	-1.41666	2.49E+00
Nppb	########	5.70E+02	########	8.117551	1.29E+00
Ibsp	1.93E-30	1.42E+02	8.56E-33	1.919439	3.39E+00
Gpr27	2.23E-02	9.61E+00	1.94E-03	1.015904	1.13E+00
Tcf23	4.78E-03	1.30E+01	3.19E-04	-0.65651	1.70E+00
Nbl1	2.20E-81	3.78E+02	2.78E-84	5.392774	1.43E+00
Il1rl2	4.07E-02	8.28E+00	4.02E-03	-1.62861	2.13E+00
Cfap69	2.21E-03	1.46E+01	1.31E-04	0.938127	1.15E+00
Dlx5	2.17E-02	9.66E+00	1.88E-03	0.371872	1.06E+00
Arntl2	3.55E-05	2.34E+01	1.35E-06	-0.87136	2.95E+00
Mmp13	2.35E-20	9.51E+01	1.81E-22	0.211689	6.34E+00
Kcna6	4.28E-08	3.73E+01	1.01E-09	-0.87204	4.24E+00
Pmfbp1	1.86E-04	1.99E+01	8.15E-06	-0.84814	2.47E+00
Gba3	4.21E-03	1.32E+01	2.77E-04	-0.47244	1.60E+00
Ankrd23	########	1.14E+03	########	5.84394	2.25E+00
RGD156265	4.99E-02	7.83E+00	5.15E-03	0.652564	1.03E+00
Prrx1	6.56E-07	3.17E+01	1.84E-08	2.539744	1.09E+00
Cacna1a	3.12E-25	1.18E+02	1.74E-27	4.242774	1.23E+00
Vcan	1.20E-28	1.34E+02	5.95E-31	5.034493	1.01E+00
Fbln2	1.89E-90	4.20E+02	2.04E-93	6.816823	1.12E+00
Uts2r	4.98E-03	1.29E+01	3.36E-04	-1.11152	2.07E+00
Abca4	1.13E-03	1.61E+01	6.16E-05	0.079555	1.66E+00
Ildr2	2.42E-09	4.32E+01	4.85E-11	0.270103	2.73E+00
Tpm2	6.95E-84	3.90E+02	7.93E-87	5.566953	1.41E+00
Lrrc17	1.58E-06	2.98E+01	4.72E-08	2.47483	1.01E+00
Igfbp6	########	4.92E+02	########	5.420876	1.87E+00
Rbm44	1.20E-08	3.99E+01	2.62E-10	1.004047	1.88E+00
Robo2	2.50E-08	3.84E+01	5.72E-10	0.713001	2.08E+00
Pdgfrl	2.59E-11	5.26E+01	4.09E-13	3.123297	1.25E+00
Phyhipl	1.64E-03	1.53E+01	9.42E-05	-0.10573	1.74E+00
Itga11	7.15E-11	5.05E+01	1.19E-12	2.043043	1.67E+00
Scn3b	4.13E-17	7.99E+01	3.96E-19	2.321545	1.81E+00

Plekhg4	3.62E-02	8.54E+00	3.47E-03	-1.84607	2.79E+00
LOC10091C	1.85E-02	1.00E+01	1.56E-03	1.031496	2.09E+00
Fgf23	4.25E-02	8.18E+00	4.23E-03	-1.24841	1.85E+00
Aebp1	########	5.19E+02	########	6.292016	1.28E+00
LOC103689	2.99E-02	8.95E+00	2.77E-03	2.261827	1.54E+00
Loxl1	1.31E-63	2.96E+02	2.51E-66	5.659651	1.56E+00
Nlrp10	9.37E-03	1.15E+01	7.02E-04	-0.70018	1.63E+00
Adh7	2.95E-09	4.28E+01	5.97E-11	1.058637	2.09E+00
Osr1	6.08E-19	8.85E+01	5.01E-21	2.409355	1.88E+00
Lilrb4	2.14E-08	3.88E+01	4.82E-10	2.03327	1.48E+00
RGD130564	2.93E-13	6.17E+01	3.93E-15	2.512564	1.53E+00
Scara5	4.73E-36	1.68E+02	1.68E-38	5.035703	1.02E+00
Igsf10	1.11E-78	3.66E+02	1.50E-81	4.65024	1.83E+00
Tceal5	6.86E-13	6.00E+01	9.55E-15	0.923789	2.65E+00
Bcat1	8.11E-17	7.85E+01	7.99E-19	2.623784	1.70E+00
Bgn	########	1.31E+03	########	7.963918	1.90E+00
Phex	1.83E-04	1.99E+01	8.04E-06	-1.63322	4.01E+00
Cxcl1	2.95E-05	2.37E+01	1.10E-06	1.175433	1.40E+00
Prss35	4.88E-02	7.87E+00	5.01E-03	-0.28543	1.18E+00
Chi3l1	4.95E-49	2.29E+02	1.25E-51	3.870989	2.22E+00
Col9a3	8.85E-12	5.48E+01	1.34E-13	0.991182	2.43E+00
Fhad1	2.02E-04	1.97E+01	8.96E-06	0.047965	1.79E+00
Prph	2.43E-14	6.68E+01	2.98E-16	2.993717	1.23E+00
Nkd1	1.66E-06	2.97E+01	4.99E-08	1.672533	1.37E+00
AABR07066	3.68E-04	1.84E+01	1.75E-05	-0.03849	4.67E+00
Pcdhac1	1.07E-07	3.54E+01	2.66E-09	1.865486	1.38E+00
Fez1	6.88E-05	2.20E+01	2.76E-06	0.65595	1.54E+00
Klra5	1.40E-02	1.06E+01	1.12E-03	-1.14682	2.02E+00
Fbln7	2.25E-13	6.23E+01	2.98E-15	0.903724	2.62E+00
S100b	3.35E-09	4.26E+01	6.84E-11	1.427774	1.83E+00
Lgi4	1.00E-23	1.11E+02	6.11E-26	3.850244	1.28E+00
Scube3	1.39E-06	3.01E+01	4.13E-08	1.193813	1.66E+00
Marf1	5.34E-03	1.27E+01	3.64E-04	2.095671	1.38E+00
Cpxm1	1.70E-34	1.61E+02	6.49E-37	3.777297	1.57E+00
Pcdhb7	9.34E-03	1.15E+01	6.99E-04	-0.28371	1.46E+00
Stx1b	4.40E-02	8.11E+00	4.41E-03	-1.0344	1.77E+00
Igsf11	2.63E-02	9.24E+00	2.36E-03	0.449085	1.07E+00
LOC102553	4.75E-02	7.94E+00	4.84E-03	-2.58951	4.22E+00
Sfrp4	2.91E-09	4.29E+01	5.89E-11	0.495153	2.82E+00
Pnoc	1.44E-02	1.06E+01	1.16E-03	-1.44998	2.08E+00
Lingo1	1.44E-03	1.55E+01	8.05E-05	0.810376	1.17E+00
Rprm	7.79E-03	1.19E+01	5.64E-04	-1.26497	2.71E+00
Aox1	1.41E-35	1.66E+02	5.18E-38	4.2943	1.52E+00
Fam46c	1.76E-12	5.81E+01	2.51E-14	2.530671	1.53E+00
Pappa2	2.72E-21	9.95E+01	1.97E-23	0.723726	4.22E+00
Tcp11x2	1.49E-02	1.05E+01	1.21E-03	-2.08371	3.25E+00
P2rx2	3.97E-03	1.34E+01	2.58E-04	-1.75534	2.95E+00
Brinp2	1.64E-02	1.03E+01	1.34E-03	-0.73794	1.57E+00
LOC100911	4.01E-03	1.33E+01	2.61E-04	0.512316	4.88E+00
Bnc2	1.18E-07	3.52E+01	2.97E-09	0.288253	2.27E+00
Ppp2r2b	7.43E-03	1.20E+01	5.31E-04	0.112301	1.50E+00
Nalcn	2.85E-04	1.90E+01	1.31E-05	0.775373	1.31E+00
Itga8	4.10E-16	7.52E+01	4.32E-18	3.118603	1.22E+00
Podnl1	1.91E-02	9.93E+00	1.63E-03	-1.04104	1.75E+00

Slc27a2	1.60E-02	1.03E+01	1.31E-03	-0.03294	1.27E+00
AABR07013	1.86E-03	1.50E+01	1.08E-04	-1.17709	2.51E+00
Tnfrsf12a	2.05E-15	7.19E+01	2.30E-17	3.363863	1.09E+00
Mgp	########	9.97E+02	########	8.84249	1.71E+00
Espn	1.49E-02	1.05E+01	1.21E-03	-1.5585	2.26E+00
Mrgprx3	2.12E-02	9.72E+00	1.82E-03	-1.42063	2.13E+00
Slc27a6	7.71E-04	1.68E+01	4.05E-05	-0.77685	2.36E+00
Cilp2	6.66E-23	1.07E+02	4.38E-25	1.8907	2.97E+00
Thbs3	1.16E-13	6.36E+01	1.49E-15	3.243842	1.09E+00
Frzb	2.52E-08	3.84E+01	5.76E-10	2.634178	1.13E+00
Tgfb2	4.09E-29	1.36E+02	1.96E-31	4.411976	1.42E+00
C3ar1	4.20E-03	1.32E+01	2.76E-04	1.202107	1.11E+00
Adcy7	1.20E-23	1.11E+02	7.38E-26	3.930436	1.30E+00
RGD131108	3.59E-02	8.56E+00	3.44E-03	-0.23233	1.19E+00
Asic4	3.23E-03	1.38E+01	2.03E-04	-1.4165	2.49E+00
Thy1	1.93E-17	8.15E+01	1.74E-19	2.229123	2.01E+00
Fst	1.19E-06	3.04E+01	3.48E-08	0.83104	1.68E+00
Hoxb6	1.66E-02	1.02E+01	1.37E-03	-0.74408	1.85E+00
Maoa	########	1.14E+03	########	8.774505	1.97E+00
Reep2	2.32E-02	9.51E+00	2.04E-03	-1.14074	1.81E+00
Pon3	2.81E-05	2.38E+01	1.05E-06	1.002068	1.60E+00
Ndst2	4.83E-02	7.89E+00	4.96E-03	-0.40415	1.38E+00
Slco1a4	6.84E-03	1.22E+01	4.85E-04	-2.31598	4.84E+00
Col2a1	########	1.25E+03	########	4.305869	7.09E+00
Krt8	8.00E-04	1.68E+01	4.24E-05	-0.34254	1.91E+00
Esyt3	2.90E-04	1.90E+01	1.34E-05	0.483753	1.49E+00
LOC10091C	4.83E-02	7.89E+00	4.97E-03	-0.24	3.03E+00
Msln	8.74E-04	1.66E+01	4.67E-05	0.373415	1.44E+00
Ephb2	2.84E-02	9.07E+00	2.60E-03	-0.25733	1.24E+00
LOC100361	4.32E-02	8.15E+00	4.31E-03	-2.58673	4.23E+00
Fstl1	1.29E-93	4.35E+02	1.32E-96	6.721257	1.11E+00
Lum	1.15E-87	4.08E+02	1.28E-90	7.281672	1.04E+00
Pi15	3.79E-03	1.35E+01	2.44E-04	-0.08016	1.43E+00
AABR07051	7.10E-03	1.21E+01	5.07E-04	-1.71135	3.04E+00
Tlcd2	1.94E-03	1.49E+01	1.13E-04	-1.48188	2.82E+00
Uchl1	9.63E-28	1.30E+02	4.81E-30	1.129174	4.50E+00
Ctgf	########	1.35E+03	########	7.611421	1.91E+00
Atp10a	1.62E-06	2.98E+01	4.84E-08	2.452412	1.03E+00
Sfrp1	2.52E-27	1.28E+02	1.29E-29	4.053609	1.36E+00
Fgl2	1.13E-31	1.48E+02	4.73E-34	5.335263	1.02E+00
Rn50_5_08	1.78E-02	1.01E+01	1.49E-03	-2.51522	4.41E+00
Nudt11	8.96E-03	1.16E+01	6.66E-04	-1.47957	2.39E+00
Fam180a	4.96E-21	9.83E+01	3.63E-23	1.507516	3.33E+00
C4b	########	9.74E+02	########	5.645272	2.19E+00
Cd163l1	1.51E-05	2.51E+01	5.36E-07	-0.25117	2.56E+00
Rnd1	4.79E-32	1.50E+02	1.96E-34	3.693575	1.50E+00
Apopt1	1.48E-03	1.55E+01	8.32E-05	2.002886	2.35E+00
LOC259244	1.34E-02	1.07E+01	1.06E-03	-0.8648	1.86E+00
Camk1g	9.85E-03	1.14E+01	7.45E-04	-2.44399	4.57E+00
LOC299282	1.53E-13	6.31E+01	1.99E-15	2.07069	1.94E+00
LOC10091C	4.59E-02	8.01E+00	4.65E-03	-0.71069	1.61E+00
Cdh22	1.72E-06	2.96E+01	5.21E-08	2.061518	1.21E+00
Ifit1	5.43E-36	1.68E+02	1.98E-38	4.444898	1.34E+00
Klhl29	1.27E-08	3.98E+01	2.78E-10	1.426973	1.72E+00

Mpzl2	2.72E-03	1.42E+01	1.67E-04	-0.98656	2.27E+00
Ppic	1.64E-16	7.70E+01	1.67E-18	4.041066	1.04E+00
Exoc3l2	3.06E-03	1.39E+01	1.91E-04	0.519083	1.23E+00
Sypl2	1.55E-11	5.37E+01	2.37E-13	1.163502	2.22E+00
Ch25h	2.35E-04	1.94E+01	1.06E-05	1.192253	1.24E+00
Tex15	1.07E-02	1.12E+01	8.26E-04	-1.75832	2.94E+00
Epn3	3.66E-09	4.24E+01	7.55E-11	1.76808	1.71E+00
LOC100361	2.41E-02	9.43E+00	2.13E-03	0.91373	1.04E+00
AABR07027	9.34E-03	1.15E+01	6.99E-04	-2.19882	5.06E+00
Obp3	5.96E-04	1.74E+01	3.03E-05	-1.88307	5.64E+00
Aff2	3.43E-04	1.86E+01	1.62E-05	-0.61085	2.39E+00
Mpz	1.27E-08	3.98E+01	2.79E-10	2.218205	1.47E+00
C1qtnf7	1.29E-23	1.10E+02	8.08E-26	3.965099	1.21E+00
Gdf15	6.44E-12	5.54E+01	9.65E-14	1.213935	2.50E+00
Bcan	2.72E-02	9.17E+00	2.46E-03	-0.64809	1.57E+00
Tubb3	1.26E-02	1.09E+01	9.84E-04	0.771054	1.07E+00
LOC100912	########	7.80E+02	########	4.911282	2.45E+00
Col1a1	1.11E-78	3.66E+02	1.48E-81	9.007469	1.36E+00
Prelp	########	5.52E+02	########	6.739396	1.24E+00
Vil1	2.74E-03	1.42E+01	1.68E-04	-0.37778	1.73E+00
Gap43	6.23E-05	2.22E+01	2.48E-06	0.891224	1.57E+00
Pcdhb3	2.58E-02	9.29E+00	2.31E-03	0.292299	1.04E+00
LOC100911	1.23E-03	1.59E+01	6.78E-05	-0.4265	3.08E+00
Upk1b	4.66E-03	1.30E+01	3.10E-04	-0.87072	2.20E+00
Myh3	1.08E-06	3.06E+01	3.15E-08	-0.83135	4.28E+00
Fbxo17	7.97E-05	2.17E+01	3.25E-06	-0.27947	2.11E+00
Hk3	8.81E-07	3.10E+01	2.54E-08	0.374696	2.00E+00
Lrrc16a	1.68E-10	4.88E+01	2.89E-12	3.655052	1.10E+00
Tsku	8.75E-17	7.83E+01	8.64E-19	3.014294	1.35E+00
Il6	2.98E-02	8.96E+00	2.75E-03	-1.88736	2.71E+00
Bmp3	3.17E-03	1.38E+01	1.99E-04	-1.48299	2.83E+00
Prima1	5.00E-08	3.70E+01	1.19E-09	1.756642	1.54E+00
Fibin	1.44E-82	3.84E+02	1.69E-85	4.325151	2.47E+00
Tmem178a	1.35E-03	1.57E+01	7.52E-05	1.324926	1.06E+00
Mt2A	1.90E-20	9.55E+01	1.45E-22	3.893302	1.27E+00
C4a	1.06E-02	1.12E+01	8.11E-04	0.613451	1.15E+00
Prdm6	1.61E-03	1.53E+01	9.21E-05	0.178863	1.45E+00
Col10a1	3.65E-21	9.89E+01	2.66E-23	0.857237	4.20E+00
C4a	########	7.73E+02	########	5.440913	2.56E+00
Kcnc3	1.92E-04	1.98E+01	8.45E-06	0.488943	1.65E+00
Ephx4	1.77E-02	1.01E+01	1.48E-03	-1.1733	1.96E+00
Srpx2	1.55E-09	4.42E+01	3.01E-11	2.00195	1.51E+00
Spp1	########	7.75E+02	########	4.42082	4.23E+00
Col9a1	6.42E-07	3.17E+01	1.80E-08	-0.65504	3.24E+00
Zfp365	4.55E-04	1.80E+01	2.24E-05	1.820837	1.01E+00
Gng8	4.15E-08	3.74E+01	9.73E-10	1.232196	1.71E+00
C3	3.05E-16	7.58E+01	3.17E-18	2.236125	1.91E+00
Shc4	1.87E-02	9.99E+00	1.58E-03	-1.63789	2.56E+00
Hoxb3	3.11E-05	2.36E+01	1.17E-06	1.625089	1.23E+00
Tnn	2.38E-22	1.04E+02	1.62E-24	0.619364	5.46E+00
Nacad	1.61E-02	1.03E+01	1.33E-03	0.494396	1.01E+00
Bglap	5.14E-31	1.45E+02	2.24E-33	1.243096	5.45E+00
Ccl7	1.64E-03	1.53E+01	9.39E-05	1.274988	1.06E+00
Pex5	4.21E-02	8.21E+00	4.18E-03	-0.90838	4.16E+00

Fam198a	6.08E-04	1.74E+01	3.09E-05	1.592889	1.11E+00
Gcnt4	1.38E-02	1.06E+01	1.11E-03	-1.84387	2.80E+00
Clec3b	1.04E-72	3.38E+02	1.66E-75	5.28123	1.43E+00
LOC69197C	2.71E-04	1.91E+01	1.24E-05	-0.00751	1.96E+00
Col8a1	########	1.03E+03	########	5.573256	2.33E+00
Tspan17	6.61E-04	1.72E+01	3.41E-05	1.619747	1.04E+00
Panx3	3.68E-04	1.84E+01	1.75E-05	-1.93834	5.52E+00
AABR07065	4.40E-02	8.10E+00	4.42E-03	-1.83114	2.83E+00
Nppa	0.00E+00	3.16E+03	0.00E+00	9.948681	3.99E+00
Rn50_15_C	3.09E-06	2.84E+01	9.83E-08	0.286416	2.04E+00
Slit3	5.07E-17	7.95E+01	4.88E-19	4.066421	1.02E+00
Nhs	6.49E-03	1.23E+01	4.57E-04	0.8784	1.06E+00
Pak1	7.09E-05	2.19E+01	2.86E-06	1.262847	1.39E+00
Serping1	########	5.12E+02	########	6.960551	1.18E+00
Ly6g6e	2.33E-03	1.45E+01	1.39E-04	-1.07129	2.38E+00
Adamts16	2.91E-02	9.02E+00	2.68E-03	-2.44673	4.55E+00
Slc41a2	5.54E-08	3.68E+01	1.34E-09	1.035865	1.80E+00
AABR0706C	2.60E-71	3.32E+02	4.42E-74	4.041321	2.26E+00
Chrdl1	1.90E-04	1.99E+01	8.34E-06	2.117002	1.04E+00
RT1-CE6	3.59E-03	1.36E+01	2.30E-04	-1.93178	3.52E+00
Il1rn	1.22E-03	1.59E+01	6.71E-05	0.749669	1.27E+00
Has1	3.35E-05	2.35E+01	1.27E-06	0.995515	1.50E+00
Myh4	1.45E-04	2.04E+01	6.22E-06	0.88506	1.42E+00
Pamr1	3.56E-02	8.59E+00	3.39E-03	0.160281	1.11E+00
Cacna1b	1.95E-02	9.89E+00	1.66E-03	-2.44838	4.55E+00
Slc7a3	1.22E-02	1.09E+01	9.50E-04	-2.44454	4.57E+00
Pcdha3	3.30E-02	8.74E+00	3.11E-03	-0.29596	1.26E+00
Catsperg1	3.05E-06	2.84E+01	9.69E-08	0.944997	1.60E+00
Mdk	4.31E-36	1.69E+02	1.52E-38	3.591851	1.76E+00
RGD131174	5.24E-36	1.68E+02	1.89E-38	3.465709	1.68E+00
S100a4	8.14E-21	9.73E+01	6.08E-23	4.471835	1.02E+00
Myrf	1.28E-05	2.55E+01	4.48E-07	1.742115	1.24E+00
Sez6l	3.60E-02	8.55E+00	3.45E-03	0.092986	1.07E+00
1500009L1	2.25E-02	9.59E+00	1.96E-03	-1.00747	1.62E+00
Agmo	1.10E-02	1.11E+01	8.47E-04	-0.34793	1.58E+00
Bend6	1.44E-04	2.04E+01	6.15E-06	1.176027	1.45E+00
Sln	3.21E-29	1.37E+02	1.51E-31	2.136228	2.78E+00
Ccdc3	1.50E-02	1.05E+01	1.22E-03	0.928913	1.12E+00
Dnah2	1.58E-02	1.03E+01	1.30E-03	-1.63564	2.57E+00
Efna3	1.13E-02	1.11E+01	8.71E-04	-2.44564	4.57E+00
Col12a1	2.17E-55	2.58E+02	5.03E-58	3.397298	2.33E+00
Pcgf1	5.30E-04	1.76E+01	2.67E-05	0.227269	2.05E+00
Krt18	1.45E-10	4.91E+01	2.48E-12	-0.60538	4.57E+00
Adamts17	6.81E-04	1.71E+01	3.52E-05	0.764303	1.39E+00
Wisp2	0.00E+00	1.70E+03	0.00E+00	4.868408	4.96E+00
Dhrs9	3.61E-02	8.55E+00	3.45E-03	-0.52523	1.41E+00
AABR07051	9.64E-03	1.14E+01	7.26E-04	-2.31813	4.83E+00
Igh-6	2.61E-09	4.31E+01	5.25E-11	2.360949	1.37E+00
C2	8.34E-22	1.02E+02	5.87E-24	3.816448	1.29E+00
AABR07051	4.24E-02	8.19E+00	4.21E-03	-2.2501	2.91E+00
Dupd1	2.24E-02	9.59E+00	1.96E-03	0.170185	1.35E+00
Pcdhb2	2.67E-02	9.21E+00	2.40E-03	-0.43816	1.33E+00
Cpeb1	1.18E-24	1.15E+02	6.87E-27	2.410295	2.20E+00
Rplp2	4.77E-02	7.92E+00	4.89E-03	4.127533	1.61E+00

Crtac1	3.64E-04	1.85E+01	1.73E-05	-0.25443	1.93E+00
Fbln1	########	9.67E+02	########	7.143437	1.60E+00
MGC105649	8.30E-09	4.07E+01	1.77E-10	2.412496	1.23E+00
Adig	4.79E-02	7.91E+00	4.90E-03	-1.67606	3.93E+00
Tgfa	1.75E-14	6.75E+01	2.11E-16	1.212598	2.56E+00
AABR07051	1.40E-02	1.06E+01	1.12E-03	-1.84667	3.65E+00
Nol8	9.71E-04	1.64E+01	5.23E-05	0.069394	2.06E+00
Plod2	3.10E-45	2.11E+02	8.89E-48	4.807517	1.31E+00
Fmod	4.28E-23	1.08E+02	2.77E-25	3.493343	1.30E+00
Ahnak2	8.00E-94	4.36E+02	7.90E-97	4.804008	1.98E+00
Hecw1	3.12E-02	8.86E+00	2.91E-03	-2.38421	4.67E+00
Vash2	3.84E-17	8.01E+01	3.65E-19	3.651158	1.13E+00
LOC102552	1.02E-38	1.81E+02	3.38E-41	2.939837	2.30E+00
Atp1a3	1.77E-15	7.22E+01	1.97E-17	2.680827	1.46E+00
Tnfrsf11b	2.06E-04	1.97E+01	9.15E-06	1.790428	1.09E+00
LOC689757	4.93E-03	1.29E+01	3.32E-04	-0.66267	1.96E+00
Pdcd5	5.71E-03	1.26E+01	3.94E-04	2.484206	3.09E+00
Dact2	1.46E-05	2.52E+01	5.20E-07	0.99249	1.47E+00
Cpxm2	2.68E-77	3.59E+02	3.80E-80	3.413935	2.82E+00
Figf	4.40E-18	8.45E+01	3.81E-20	3.557405	1.14E+00
Camkk1	7.62E-06	2.65E+01	2.59E-07	2.322285	1.03E+00
RGD130492	5.15E-04	1.77E+01	2.58E-05	0.849994	1.23E+00
Rn50_10_C	2.28E-02	9.55E+00	2.00E-03	-2.51466	4.41E+00
Myl1	3.96E-30	1.41E+02	1.77E-32	2.298344	2.76E+00
Wscd2	1.02E-02	1.13E+01	7.76E-04	-0.67966	1.67E+00
Clec3a	2.31E-39	1.84E+02	7.43E-42	1.520865	6.44E+00
Pgbd5	5.52E-05	2.24E+01	2.17E-06	-1.8892	5.61E+00
Fn1	1.65E-76	3.56E+02	2.45E-79	7.158078	1.05E+00
Lrrn4cl	2.41E-02	9.43E+00	2.13E-03	0.591039	1.07E+00
Kcnj9	3.31E-02	8.73E+00	3.13E-03	-0.86154	1.51E+00
Akip1	9.93E-63	2.92E+02	1.96E-65	4.588187	1.70E+00
Timp1	9.92E-39	1.81E+02	3.24E-41	3.938892	1.59E+00
Serpine2	########	6.63E+02	########	6.297844	1.46E+00
Rxfp1	1.69E-34	1.61E+02	6.42E-37	2.871092	2.42E+00
Dpysl4	3.49E-04	1.86E+01	1.65E-05	0.746961	1.35E+00
Lbp	9.68E-22	1.02E+02	6.87E-24	3.844461	1.44E+00
Fuom	2.69E-02	9.20E+00	2.42E-03	1.146911	3.10E+00
Col14a1	3.70E-82	3.82E+02	4.45E-85	6.516815	1.04E+00
Aldh3a1	2.77E-07	3.34E+01	7.39E-09	2.226972	1.33E+00
AABR07051	3.77E-02	8.45E+00	3.65E-03	-2.51411	4.41E+00
LOC257642	2.20E-02	9.64E+00	1.90E-03	-2.51577	4.40E+00
Ptgis	6.31E-43	2.00E+02	1.89E-45	5.187508	1.09E+00
Atp6v0d2	1.21E-02	1.09E+01	9.48E-04	-2.44454	4.57E+00
Gpnmb	4.95E-49	2.29E+02	1.24E-51	4.62392	1.45E+00
RGD156184	5.31E-11	5.11E+01	8.71E-13	0.692922	2.48E+00
Rell2	2.27E-02	9.56E+00	1.99E-03	-2.4429	4.58E+00
Islr	########	7.86E+02	########	5.45005	2.12E+00
LOC100912	1.98E-06	2.93E+01	6.10E-08	-0.03029	2.58E+00
Scd	2.10E-06	2.92E+01	6.49E-08	-0.20558	2.47E+00
Tceal9	1.39E-59	2.77E+02	2.93E-62	5.657897	1.03E+00
Tnnt1	4.46E-03	1.31E+01	2.95E-04	0.773424	1.07E+00
Il17re	1.17E-10	4.95E+01	1.98E-12	0.32034	2.89E+00
Tgfb3	3.46E-64	2.99E+02	6.51E-67	5.591945	1.11E+00
Phf24	7.55E-15	6.92E+01	8.88E-17	3.942137	1.06E+00

AABR07025	2.27E-02	9.57E+00	1.98E-03	-1.92992	2.65E+00
Cdh3	2.99E-02	8.95E+00	2.77E-03	-0.90589	1.97E+00
Gpm6a	5.07E-15	7.00E+01	5.89E-17	3.069257	1.17E+00
Cthrc1	1.81E-07	3.43E+01	4.66E-09	0.44326	2.57E+00
NEWGENE_6	5.20E-04	1.77E+01	2.61E-05	0.559902	2.97E+00
Tgm1	3.04E-09	4.28E+01	6.17E-11	-0.07906	3.09E+00
Olr1085	4.03E-03	1.33E+01	2.62E-04	-2.31436	4.85E+00
Sbsn	1.00E-29	1.39E+02	4.57E-32	2.607318	2.42E+00
Hs3st2	1.36E-08	3.97E+01	3.00E-10	-0.60112	4.56E+00
Cacna2d3	3.89E-03	1.34E+01	2.51E-04	-0.78598	2.34E+00
Tbx15	1.19E-03	1.59E+01	6.51E-05	-2.07982	5.30E+00
LOC365238	3.28E-02	8.76E+00	3.08E-03	-2.19041	3.04E+00
Itgbl1	4.45E-56	2.61E+02	1.00E-58	5.620825	1.05E+00
RGD131144	1.07E-03	1.62E+01	5.79E-05	-0.91886	2.36E+00
Antxr1	2.19E-25	1.19E+02	1.21E-27	3.882242	1.45E+00
Nov	1.96E-44	2.07E+02	5.68E-47	3.79598	1.82E+00
Acan	7.37E-43	2.00E+02	2.23E-45	1.720096	6.27E+00
Matn4	1.48E-18	8.67E+01	1.25E-20	1.6647	2.72E+00
Arhgef19	8.81E-03	1.16E+01	6.51E-04	-0.35253	1.46E+00
Fam114a1l	8.50E-03	1.17E+01	6.24E-04	0.152937	1.29E+00
Olr63	4.26E-03	1.32E+01	2.80E-04	0.978545	1.10E+00
Il1b	1.77E-03	1.51E+01	1.03E-04	1.489058	1.02E+00
N4bp3	6.45E-03	1.23E+01	4.53E-04	0.058639	1.63E+00
LOC10090S	1.89E-03	1.50E+01	1.10E-04	0.231626	1.87E+00
Fcnb	1.55E-03	1.54E+01	8.77E-05	0.810834	1.17E+00
Fcrl2	2.28E-17	8.12E+01	2.07E-19	0.925512	3.06E+00
Slc9b2	3.89E-03	1.34E+01	2.51E-04	-0.3346	1.82E+00
LOC10369C	1.48E-02	1.05E+01	1.19E-03	1.743527	2.48E+00
Scg2	2.23E-04	1.95E+01	9.95E-06	-1.98552	5.45E+00
Ighv12-3	6.75E-05	2.20E+01	2.69E-06	-1.51908	4.19E+00
Rarres1	6.14E-17	7.91E+01	5.97E-19	2.810025	1.57E+00
C1qtnf3	1.90E-10	4.85E+01	3.30E-12	0.33631	2.82E+00
Ccl19	3.74E-06	2.80E+01	1.21E-07	1.942468	1.66E+00
Sh2d4a	1.49E-05	2.52E+01	5.29E-07	2.255216	1.11E+00
Col8a2	2.36E-75	3.50E+02	3.57E-78	3.696095	2.75E+00
Capns1	3.41E-02	8.67E+00	3.23E-03	3.457336	1.68E+00
Nr4a3	9.13E-16	7.35E+01	9.92E-18	2.420558	########
Ccnj	2.76E-03	1.41E+01	1.69E-04	-1.46754	########
LOC100362	7.78E-03	1.19E+01	5.62E-04	1.747328	########
Hhip	4.40E-02	8.10E+00	4.42E-03	-2.50314	########
Fam131c	1.10E-22	1.06E+02	7.24E-25	4.516776	########
Myh6	1.42E-32	1.52E+02	5.70E-35	13.15967	########
Zdbf2	2.98E-02	8.96E+00	2.76E-03	-1.46158	########
Psme1-ps1	8.06E-03	1.18E+01	5.87E-04	0.601397	########
Grid1	1.60E-05	2.50E+01	5.71E-07	0.644109	########
Epha3	1.69E-06	2.97E+01	5.09E-08	2.646792	########
LOC103693	3.86E-02	8.40E+00	3.76E-03	-2.65872	########
Crybb1	3.51E-10	4.72E+01	6.34E-12	2.764344	########
Smco1	4.38E-04	1.81E+01	2.14E-05	-0.85342	########
Ankrd24	3.65E-02	8.52E+00	3.51E-03	1.108259	########
Nkrf	7.87E-03	1.19E+01	5.70E-04	-0.09527	########
Reg3g	2.12E-05	2.44E+01	7.69E-07	-0.87964	########
RGD156535	1.34E-31	1.48E+02	5.68E-34	4.079798	########
Mc2r	3.59E-02	8.56E+00	3.43E-03	-2.30147	########

Gata3	4.88E-02	7.87E+00	5.02E-03	0.088042	########
Myo3b	2.45E-06	2.89E+01	7.70E-08	1.828626	########
Zfp420	1.21E-04	2.08E+01	5.11E-06	-0.79753	########
Rack1	3.75E-02	8.46E+00	3.63E-03	0.718219	########
Dcun1d2	8.57E-04	1.66E+01	4.56E-05	2.519624	########
Mrpl53	8.34E-03	1.17E+01	6.09E-04	2.528726	########
Myo16	3.38E-05	2.35E+01	1.28E-06	0.11408	########
Ica1l	2.85E-02	9.06E+00	2.61E-03	-2.57753	########
Retnlg	8.62E-04	1.66E+01	4.59E-05	-0.54585	########
Rpl38-ps3	2.61E-03	1.43E+01	1.59E-04	0.099783	########
Tsc22d4	1.82E-04	1.99E+01	7.95E-06	1.30905	########
AABR07009	4.82E-02	7.90E+00	4.95E-03	-0.02748	########
Acot1	4.36E-02	8.13E+00	4.36E-03	0.247563	########
RGD156307	3.35E-02	8.71E+00	3.17E-03	1.180387	########
Zfp36l2	4.04E-02	8.30E+00	3.97E-03	3.834556	########
Fbxw10	1.74E-10	4.87E+01	3.01E-12	2.982626	########
Ano5	9.12E-30	1.39E+02	4.14E-32	4.169842	########
Phka2	9.37E-06	2.61E+01	3.23E-07	1.070199	########
Fam81a	3.39E-07	3.30E+01	9.18E-09	2.548962	########
Il27ra	1.02E-02	1.13E+01	7.81E-04	0.310238	########
Gabrb2	2.05E-10	4.83E+01	3.60E-12	2.019837	########
AABR0700C	5.45E-04	1.76E+01	2.75E-05	-1.9159	########
AC115371.	7.69E-04	1.69E+01	4.04E-05	0.712275	########
Trpc5	4.16E-02	8.24E+00	4.11E-03	-2.65675	########
Rassf6	1.91E-02	9.93E+00	1.62E-03	-0.69078	########
AC130940.	2.93E-04	1.89E+01	1.35E-05	0.253612	########
Cyp2w1	4.76E-02	7.93E+00	4.86E-03	-1.46345	########
Rcor2	8.09E-03	1.18E+01	5.90E-04	2.698547	########
AABR07002	2.80E-03	1.41E+01	1.72E-04	-0.23693	########
Clec4g	4.81E-02	7.90E+00	4.93E-03	-2.07091	########
AABR07016	3.18E-02	8.82E+00	2.98E-03	-0.8261	########
Gorasp2	3.72E-03	1.35E+01	2.39E-04	1.722847	########
Hspa1b	7.42E-05	2.18E+01	3.01E-06	3.365697	########
Gpr63	2.09E-06	2.92E+01	6.46E-08	2.115163	########
Rn50_11_C	4.47E-02	8.07E+00	4.49E-03	-0.11039	########
Ankra2	2.64E-02	9.23E+00	2.38E-03	-0.53059	########
RGD156457	1.05E-02	1.12E+01	8.00E-04	-2.50602	########
Nxpe2	5.15E-03	1.28E+01	3.48E-04	-0.20586	########
Klhl33	1.49E-22	1.05E+02	9.98E-25	3.556024	########
Adra1d	2.74E-17	8.08E+01	2.52E-19	2.353609	########
Prkar1b	1.01E-02	1.13E+01	7.65E-04	0.615806	########
Stc2	5.00E-18	8.42E+01	4.37E-20	4.012999	########
Cfap52	2.19E-04	1.96E+01	9.78E-06	1.044312	########
Fcer2	3.45E-14	6.61E+01	4.26E-16	3.276752	########
Gad2	2.25E-02	9.58E+00	1.97E-03	-2.57949	########
Slc17a7	6.91E-03	1.22E+01	4.91E-04	0.685307	########
Tnks2	5.38E-03	1.27E+01	3.68E-04	2.101412	########
Pcdh15	2.59E-02	9.28E+00	2.32E-03	-2.58124	########
Ap1s3	3.64E-11	5.19E+01	5.82E-13	1.966125	########
Coch	5.40E-10	4.63E+01	9.93E-12	-0.60024	########
RGD156482	7.33E-07	3.14E+01	2.09E-08	-0.52549	########
Ebna1bp2	4.39E-12	5.62E+01	6.44E-14	1.167289	########
Mns1	3.71E-03	1.35E+01	2.38E-04	0.677293	########
Frem2	2.05E-06	2.93E+01	6.34E-08	2.370553	########

Serpinb7	4.39E-02	8.11E+00	4.40E-03	-0.48349	########
Fbxl18	2.69E-04	1.91E+01	1.23E-05	1.934301	########
Kcnk2	2.19E-18	8.59E+01	1.87E-20	3.816358	########
Pik3c2g	1.34E-06	3.02E+01	3.95E-08	1.59813	########
Dlgap1	9.57E-05	2.13E+01	3.97E-06	1.878892	########
LOC10091C	2.70E-03	1.42E+01	1.64E-04	3.770561	########
RGD156535	3.97E-08	3.75E+01	9.30E-10	1.857993	########
Sema3a	1.43E-02	1.06E+01	1.15E-03	0.994903	########
Cyp2e1	7.70E-32	1.49E+02	3.21E-34	3.809311	########
Zmat4	1.43E-02	1.06E+01	1.15E-03	-2.50551	########
Ccdc158	9.64E-06	2.60E+01	3.33E-07	1.069628	########
AABR07032	2.38E-02	9.45E+00	2.11E-03	-2.57805	########
RGD156314	3.04E-02	8.91E+00	2.83E-03	-2.50703	########
Sbk2	4.59E-02	8.01E+00	4.65E-03	-0.01361	########
Pak6	3.11E-14	6.63E+01	3.83E-16	3.9202	########
Pitx2	2.89E-06	2.85E+01	9.16E-08	-0.66091	########
Sall1	7.27E-06	2.66E+01	2.46E-07	0.546798	########
Slc26a3	1.53E-06	2.99E+01	4.57E-08	1.265162	########
AABR07072	7.46E-03	1.20E+01	5.35E-04	-0.67326	########
Igf2bp2	2.18E-02	9.66E+00	1.88E-03	-0.17146	########
Slc4a1	1.47E-11	5.38E+01	2.24E-13	3.177939	########
Unc79	1.51E-02	1.04E+01	1.23E-03	-0.72164	########
Cacng6	1.06E-06	3.06E+01	3.10E-08	2.429319	########
Cetn3	2.69E-02	9.19E+00	2.43E-03	2.716501	########
Ptp4a1	2.98E-02	8.96E+00	2.75E-03	0.791112	########
Cxcl14	7.19E-06	2.67E+01	2.42E-07	1.36321	########
Lrrc7	7.08E-03	1.21E+01	5.05E-04	0.374816	########
Igf2bp3	6.95E-05	2.20E+01	2.79E-06	0.408101	########
Bmx	1.80E-02	1.01E+01	1.51E-03	0.971923	########
Il1a	5.69E-03	1.26E+01	3.93E-04	-1.38856	########
Wwc1	3.78E-05	2.32E+01	1.44E-06	1.852062	########
Brms1	4.83E-02	7.90E+00	4.95E-03	1.910923	########
LOC100911	2.99E-02	8.95E+00	2.77E-03	0.332265	########
Vwa2	2.81E-03	1.41E+01	1.73E-04	1.484241	########
Igf2	1.58E-47	2.22E+02	4.15E-50	5.195454	########
MGC94199	8.93E-03	1.16E+01	6.64E-04	-0.22642	########
Scn10a	2.15E-02	9.68E+00	1.86E-03	-2.58051	########
LOC10035S	3.06E-03	1.39E+01	1.91E-04	-1.29273	########
Siglech	2.72E-02	9.16E+00	2.47E-03	-2.12877	########
LOC100912	1.31E-03	1.57E+01	7.27E-05	0.201348	########
A930018M2	3.05E-08	3.80E+01	7.01E-10	2.35917	########
Apln	1.77E-14	6.75E+01	2.15E-16	3.760816	########
Abhd10	2.95E-17	8.06E+01	2.74E-19	2.117577	########
Rpusd2	3.95E-02	8.35E+00	3.86E-03	0.933033	########
Agr2	5.48E-06	2.72E+01	1.81E-07	-0.83605	########
Plin1	5.42E-03	1.27E+01	3.71E-04	0.785107	########
Slc23a3	1.38E-02	1.07E+01	1.10E-03	-1.53678	########
Foxo6	2.72E-04	1.91E+01	1.24E-05	1.588097	########
