# Supplementary material for: Yixin-Shu Capsules Ameliorated Ischemia-Induced Heart Failure by Restoring Trx2 and Inhibiting JNK/p38 Activation
Source: Oxid Med Cell Longev. 2021 Feb 16;2021:8049079. doi: 10.1155/2021/8049079 (PMC7902134; doi:10.1155/2021/8049079)
Supplement: Supplementary Materials — Antibodies such as Nrf2 (ab89443), Tlr4 (19811-1-AP), and Myd88 (sc-74532) were used for IF staining. As for F-actin staining, the samples were incubated with 0.1% Triton X-100 for 15 min. Rhodamine phalloidin (PHDR1, cytoskeleton) was used for the staining of F-actin after the treatment of 0.1% Triton X-100 for 15 min and then followed by 4,6-DAPI for 10 min before observation. Table S1: the RNA-seq data of failing heart treated with or without YXS or VST. Figure S1: the enrichment of DEs in YXS-mediated protection against H2O2-induced damage; (A) enriched GO terms of upregulated DEs; (B) enriched GO terms of downregulated DEs. Figure S2: YXS decreased the levels of Tlr4 and Myd88, enhanced Nrf2 expression, and improved cytoskeleton arrangement; (A) the IF staining of Tlr4 (red) and Myd88 (green) in H2O2-induced H9C2 cell and the related quantification, nucleus (blue), scale bar: 100 μm (n = 3–5); (B) the IF staining of Nrf2 (green) and F-actin (red) in heart tissue, nucleus (blue), scale bar: 100 μm; (C) the IF staining of Nrf2 (green) in H2O2-induced H9C2 cell and the related quantification, nucleus (blue), scale bar: 100 μm (n = 3–5). [file 8049079.f1.zip › Xiang.Table S1 HF+VST vs HF.pdf]

HF+VST VS HF

Gene	FDR	LR	PValue	logCPM	logFC
Mpv17l	2.66E-02	8.98E+00	2.73E-03	0.03638	1.24E+00
Dkc1	3.30E-02	8.50E+00	3.54E-03	-2.65564	4.31E+00
Vps8	0.00E+00	1.80E+03	0.00E+00	7.719116	2.17E+00
LOC68939€	4.30E-02	7.94E+00	4.84E-03	-0.86106	1.36E+00
Vps8	9.57E-06	2.58E+01	3.85E-07	2.058501	1.11E+00
LOC68939€	1.61E-03	1.50E+01	1.09E-04	1.45662	1.42E+00
Neurog3	2.78E-02	8.88E+00	2.88E-03	0.134741	1.18E+00
Vps8	1.17E-19	9.23E+01	7.34E-22	3.784618	1.24E+00
Dkc1	2.25E-02	9.34E+00	2.24E-03	-0.67448	1.42E+00
Dkc1	4.24E-03	1.29E+01	3.26E-04	-1.3939	6.45E+00
Gnaz	########	6.31E+02	########	6.313021	1.08E+00
LOC68939€	2.47E-04	1.89E+01	1.37E-05	1.494523	2.35E+00
Gnaz	4.67E-02	7.75E+00	5.37E-03	-0.32048	1.80E+00
Vps8	3.17E-02	8.59E+00	3.38E-03	0.358455	1.04E+00
Gnaz	########	5.45E+02	########	5.832141	1.27E+00
Vps8	3.65E-02	8.28E+00	4.00E-03	-0.54171	1.22E+00
Gnaz	1.18E-02	1.07E+01	1.06E-03	0.060064	1.28E+00
Dkc1	1.70E-02	9.93E+00	1.63E-03	0.016333	1.22E+00
Vps8	7.34E-03	1.17E+01	6.10E-04	0.062656	2.54E+00
LOC68939€	6.50E-05	2.18E+01	3.10E-06	1.612015	1.10E+00
Gnaz	4.72E-03	1.27E+01	3.68E-04	-0.82458	1.84E+00
Vps8	1.61E-31	1.48E+02	3.87E-34	2.822703	2.15E+00
Gnaz	1.34E-04	2.02E+01	6.91E-06	1.437771	1.13E+00
Vps8	3.10E-02	8.64E+00	3.29E-03	-1.26171	1.65E+00
Dkc1	3.10E-02	8.64E+00	3.29E-03	0.71964	1.80E+00
Vps8	4.18E-02	8.00E+00	4.68E-03	1.003912	1.04E+00
LOC68939€	4.45E-02	7.86E+00	5.06E-03	-2.14551	2.51E+00
Gnaz	4.96E-25	1.18E+02	2.01E-27	2.868859	1.68E+00
Vps8	1.75E-03	1.48E+01	1.20E-04	-1.65698	2.81E+00
Pdxdc1	2.90E-02	8.79E+00	3.03E-03	-0.83831	1.54E+00
Gnaz	2.61E-04	1.88E+01	1.45E-05	0.694362	1.29E+00
Gnaz	7.56E-03	1.17E+01	6.31E-04	-0.86647	2.93E+00
Dkc1	4.95E-02	7.62E+00	5.78E-03	-2.37844	2.94E+00
Vps8	1.23E-05	2.52E+01	5.10E-07	1.044049	1.39E+00
Neurog3	4.69E-02	7.74E+00	5.40E-03	-2.73374	4.10E+00
Vps8	1.22E-05	2.52E+01	5.05E-07	2.125236	1.06E+00
Gnaz	9.47E-05	2.09E+01	4.72E-06	-1.095	2.61E+00
Vps8	2.97E-02	8.73E+00	3.12E-03	3.157256	1.93E+00
Gnaz	8.21E-05	2.12E+01	4.04E-06	1.027727	1.30E+00
Vps8	2.06E-51	2.42E+02	1.65E-54	3.238617	2.33E+00
Vps8	1.98E-02	9.61E+00	1.93E-03	0.287607	1.13E+00
Vps8	2.27E-02	9.32E+00	2.27E-03	-0.14628	1.27E+00
Gnaz	1.05E-02	1.10E+01	9.28E-04	0.75457	1.37E+00
Gnaz	4.52E-02	7.82E+00	5.17E-03	1.739226	1.18E+00
LOC68939€	7.93E-22	1.03E+02	4.11E-24	4.022365	1.09E+00
Vps8	1.62E-03	1.50E+01	1.10E-04	0.883924	1.18E+00
Vps8	2.43E-02	9.17E+00	2.46E-03	-2.31977	3.06E+00
LOC68939€	2.38E-02	9.22E+00	2.40E-03	0.678271	1.03E+00
Dkc1	3.30E-02	8.51E+00	3.54E-03	1.442525	1.53E+00
Dkc1	3.41E-02	8.43E+00	3.69E-03	-1.15457	1.63E+00
LOC68939€	1.58E-02	1.01E+01	1.49E-03	-1.51538	2.23E+00

Neurog3	2.34E-02	9.25E+00	2.35E-03	-0.28626	1.22E+00
Mpv17l	3.05E-19	9.03E+01	2.02E-21	4.108809	1.04E+00
LOC68939€	2.83E-03	1.38E+01	2.06E-04	0.530186	1.83E+00
Vps8	3.23E-02	8.55E+00	3.46E-03	3.261363	1.38E+00
Mpv17l	7.74E-03	1.16E+01	6.50E-04	1.606864	1.59E+00
Vps8	1.25E-02	1.06E+01	1.14E-03	0.803094	1.02E+00
Dkc1	2.83E-02	8.84E+00	2.95E-03	-1.01901	1.52E+00
Vps8	1.24E-03	1.55E+01	8.19E-05	1.55043	1.09E+00
Gnaz	4.00E-02	8.09E+00	4.46E-03	-1.51227	1.68E+00
Neurog3	5.43E-03	1.24E+01	4.32E-04	0.388003	2.38E+00
LOC68939€	4.00E-03	1.30E+01	3.05E-04	1.368108	1.23E+00
Vps8	3.10E-03	1.36E+01	2.28E-04	-1.21651	2.76E+00
Gnaz	1.01E-23	1.12E+02	4.57E-26	3.456373	1.37E+00
Dkc1	1.01E-02	1.11E+01	8.83E-04	0.042895	1.25E+00
Mpv17l	1.87E-02	9.72E+00	1.82E-03	0.406901	1.11E+00
LOC68939€	4.67E-03	1.27E+01	3.64E-04	0.567607	1.17E+00
Gnaz	4.43E-02	7.87E+00	5.03E-03	-2.73555	4.09E+00
Vps8	4.17E-02	8.01E+00	4.66E-03	-1.94594	2.30E+00
Neurog3	6.25E-03	1.21E+01	5.07E-04	0.109487	1.41E+00
Neurog3	2.60E-03	1.39E+01	1.88E-04	-2.15494	5.33E+00
LOC68939€	9.75E-12	5.45E+01	1.57E-13	0.044249	3.65E+00
Dkc1	1.90E-84	3.96E+02	4.11E-88	6.926796	1.05E+00
Gnaz	2.48E-02	9.12E+00	2.52E-03	-2.65827	4.30E+00
49314000C	6.03E-04	1.71E+01	3.63E-05	0.538743	3.21E+00
Gnaz	7.84E-03	1.16E+01	6.59E-04	-2.04082	3.62E+00
Gnaz	2.14E-02	9.45E+00	2.11E-03	-0.04778	1.11E+00
Vps8	1.56E-02	1.01E+01	1.47E-03	-2.58499	4.48E+00
Neurog3	9.57E-03	1.12E+01	8.32E-04	2.997184	1.29E+00
Reep3	2.28E-02	9.31E+00	2.28E-03	-0.75672	1.54E+00
Gnaz	2.49E-05	2.38E+01	1.09E-06	0.788109	4.03E+00
Mpv17l	1.53E-28	1.34E+02	4.51E-31	4.602388	1.13E+00
LOC68939€	4.36E-02	7.90E+00	4.93E-03	-2.73583	4.09E+00
Gnaz	3.58E-02	8.32E+00	3.92E-03	-0.55792	1.39E+00
Dkc1	7.19E-06	2.64E+01	2.82E-07	1.30255	1.38E+00
Vps8	2.36E-05	2.39E+01	1.03E-06	4.249449	1.02E+00
Vps8	2.72E-02	8.93E+00	2.81E-03	-0.69385	1.40E+00
Vps8	1.65E-05	2.46E+01	6.98E-07	0.380023	1.79E+00
Vps8	7.53E-03	1.17E+01	6.27E-04	-1.94416	2.89E+00
Vps8	1.17E-10	4.93E+01	2.24E-12	1.805854	1.61E+00
Reep3	4.34E-02	7.91E+00	4.90E-03	-2.14705	2.52E+00
Gnaz	2.47E-04	1.89E+01	1.37E-05	1.403355	1.11E+00
Gnaz	1.60E-06	2.95E+01	5.50E-08	2.861257	2.26E+00
LOC68939€	1.96E-13	6.27E+01	2.41E-15	3.576489	1.78E+00
Reep3	3.15E-02	8.61E+00	3.35E-03	-2.31574	3.08E+00
Gnaz	2.75E-02	8.90E+00	2.85E-03	1.246648	2.30E+00
LOC68939€	5.49E-04	1.73E+01	3.27E-05	-0.07096	2.64E+00
Vps8	1.82E-03	1.47E+01	1.26E-04	0.990936	1.10E+00
Gnaz	3.54E-02	8.35E+00	3.87E-03	0.038485	1.40E+00
Neurog3	3.35E-02	8.48E+00	3.60E-03	-0.86149	1.37E+00
Mpv17l	2.46E-03	1.41E+01	1.77E-04	2.761616	2.14E+00
Gnaz	6.44E-28	1.31E+02	2.01E-30	3.468487	1.45E+00
Gnaz	4.56E-03	1.28E+01	3.54E-04	-1.89931	2.97E+00
Gnaz	9.95E-08	3.53E+01	2.84E-09	2.929569	3.72E+00
Vps8	2.06E-02	9.52E+00	2.03E-03	-2.6562	4.30E+00

Gnaz	2.55E-02	9.06E+00	2.61E-03	-0.87766	1.47E+00
Dkc1	4.14E-03	1.30E+01	3.17E-04	-2.10637	5.39E+00
LOC68939€	7.54E-04	1.66E+01	4.68E-05	-0.00634	1.49E+00
Vps8	8.20E-72	3.37E+02	2.53E-75	3.744595	2.52E+00
Gnaz	1.08E-05	2.55E+01	4.44E-07	1.355599	1.30E+00
Vps8	1.25E-03	1.55E+01	8.31E-05	7.506601	1.03E+00
Dkc1	2.28E-02	9.31E+00	2.28E-03	1.489702	1.90E+00
Vps8	1.14E-02	1.08E+01	1.02E-03	-1.69856	2.30E+00
Gnaz	6.65E-04	1.68E+01	4.06E-05	2.273048	2.06E+00
Mpv17l	4.06E-02	8.06E+00	4.53E-03	-0.86044	1.36E+00
LOC68939€	1.94E-02	9.65E+00	1.89E-03	-2.58004	4.50E+00
Gnaz	6.44E-03	1.20E+01	5.24E-04	-1.852	3.04E+00
Vps8	1.25E-17	8.26E+01	9.80E-20	3.968951	1.02E+00
Vps8	1.70E-02	9.93E+00	1.62E-03	4.739325	1.32E+00
Vps8	7.34E-71	3.32E+02	2.94E-74	5.63143	1.10E+00
Gnaz	2.90E-02	8.79E+00	3.04E-03	-2.0958	2.62E+00
Dkc1	4.36E-02	7.90E+00	4.94E-03	-2.73583	4.09E+00
Vps8	2.38E-02	9.21E+00	2.40E-03	0.164108	1.10E+00
Gnaz	1.13E-02	1.08E+01	1.00E-03	0.210753	3.09E+00
LOC68939€	1.51E-02	1.02E+01	1.41E-03	-1.17029	3.45E+00
Vps8	1.82E-02	9.78E+00	1.76E-03	-0.40707	########
Vps8	1.16E-02	1.08E+01	1.04E-03	-1.6552	########
LOC68939€	9.15E-04	1.62E+01	5.83E-05	-1.47129	########
LOC68939€	3.54E-02	8.34E+00	3.87E-03	1.958956	########
Vps8	1.12E-03	1.57E+01	7.32E-05	2.96861	########
Gnaz	1.42E-02	1.03E+01	1.31E-03	-2.32708	########
Vps8	4.43E-02	7.87E+00	5.03E-03	-1.53757	########
Vps8	1.59E-02	1.01E+01	1.50E-03	-1.69115	########
LOC68939€	4.67E-02	7.75E+00	5.37E-03	-2.73568	########
Vps8	3.32E-02	8.49E+00	3.56E-03	-2.65798	########
Vps8	2.17E-02	9.41E+00	2.15E-03	0.106671	########
Gnaz	1.81E-03	1.47E+01	1.25E-04	-0.79656	########
LOC68939€	3.23E-02	8.55E+00	3.45E-03	2.442645	########
Neurog3	4.95E-02	7.62E+00	5.78E-03	-1.28446	########
LOC68939€	1.36E-02	1.04E+01	1.26E-03	-2.10328	########
Gnaz	1.08E-02	1.09E+01	9.57E-04	-0.0341	########
Vps8	8.91E-04	1.62E+01	5.65E-05	-0.14525	########
LOC68939€	4.17E-02	8.00E+00	4.67E-03	-2.58748	########
Neurog3	2.37E-13	6.23E+01	2.94E-15	3.471283	########
Vps8	3.05E-11	5.21E+01	5.35E-13	-0.27906	########
Vps8	8.51E-07	3.09E+01	2.78E-08	0.271918	########
Vps8	1.21E-05	2.53E+01	5.01E-07	2.023934	########
LOC68939€	2.35E-03	1.42E+01	1.68E-04	1.706794	########
Mpv17l	4.74E-03	1.27E+01	3.71E-04	0.501974	########
Reep3	2.85E-04	1.86E+01	1.59E-05	-1.85678	########
Vps8	########	1.40E+03	########	5.886512	########
Gnaz	3.32E-02	8.50E+00	3.56E-03	1.057121	########
LOC68939€	6.15E-03	1.21E+01	4.97E-04	-2.38458	########
Vps8	3.27E-12	5.68E+01	4.84E-14	2.38596	########
LOC68939€	2.94E-02	8.76E+00	3.08E-03	-2.32282	########
LOC68939€	2.25E-02	9.34E+00	2.25E-03	0.281198	########
Gnaz	4.51E-04	1.77E+01	2.63E-05	1.425038	########
Vps8	4.61E-03	1.27E+01	3.59E-04	0.690798	########
LOC68939€	3.63E-05	2.30E+01	1.64E-06	-0.89462	########

Neurog3	9.36E-03	1.12E+01	8.10E-04	-1.3896	########
LOC68939€	1.90E-03	1.46E+01	1.32E-04	-2.26174	########
Vps8	2.11E-02	9.48E+00	2.08E-03	6.928976	########
LOC68939€	5.43E-03	1.24E+01	4.32E-04	3.336857	########
Vps8	1.05E-02	1.10E+01	9.28E-04	-1.11817	########
LOC68939€	5.23E-03	1.25E+01	4.14E-04	-1.7764	########
LOC68939€	7.65E-04	1.65E+01	4.77E-05	0.802215	########
LOC68939€	3.92E-02	8.13E+00	4.35E-03	-2.38158	########
Gnaz	1.32E-02	1.05E+01	1.22E-03	2.05721	########
Vps8	2.95E-04	1.85E+01	1.66E-05	1.228282	########
LOC68939€	1.99E-04	1.94E+01	1.07E-05	0.466734	########
LOC68939€	3.01E-06	2.82E+01	1.10E-07	-1.18288	########
Mpv17l	1.70E-02	9.93E+00	1.62E-03	-2.58535	########
Vps8	4.54E-04	1.77E+01	2.65E-05	0.758206	########
Pdxdc1	2.88E-05	2.35E+01	1.27E-06	2.315851	########
Dkc1	1.71E-02	9.91E+00	1.64E-03	-2.57948	########
Dkc1	2.28E-03	1.42E+01	1.62E-04	-2.31982	########
Gnaz	1.08E-02	1.09E+01	9.60E-04	0.632492	########
Vps8	4.90E-02	7.65E+00	5.69E-03	-2.7346	########
LOC68939€	6.57E-03	1.20E+01	5.38E-04	0.784851	########
Dkc1	1.70E-27	1.29E+02	5.50E-30	5.26263	########
Gnaz	8.19E-03	1.15E+01	6.93E-04	-1.57703	########
LOC68939€	2.77E-02	8.89E+00	2.87E-03	-2.07726	########
Vps8	4.45E-02	7.85E+00	5.07E-03	-1.94662	########
Reep3	1.16E-03	1.56E+01	7.64E-05	0.040095	########
LOC68939€	2.92E-03	1.37E+01	2.13E-04	1.011426	########
Vps8	5.34E-04	1.73E+01	3.17E-05	-2.2034	########
Vps8	4.44E-02	7.86E+00	5.05E-03	-1.3273	########
Gnaz	9.36E-08	3.54E+01	2.67E-09	-0.23837	########
Vps8	1.88E-02	9.72E+00	1.82E-03	0.470782	########
LOC68939€	8.28E-03	1.15E+01	7.01E-04	-2.10203	########
Gnaz	3.22E-02	8.56E+00	3.44E-03	-0.33022	########
Vps8	1.08E-08	3.99E+01	2.70E-10	2.014265	########
LOC68939€	1.93E-02	9.67E+00	1.88E-03	2.146526	########
Dkc1	7.56E-03	1.17E+01	6.31E-04	-1.91064	########
LOC68939€	1.33E-02	1.05E+01	1.22E-03	0.018569	########
Vps8	4.90E-02	7.65E+00	5.69E-03	-2.7346	########
Vps8	4.00E-02	8.09E+00	4.45E-03	-2.32334	########
LOC68939€	4.51E-02	7.83E+00	5.14E-03	-2.04351	########
Gnaz	3.46E-02	8.40E+00	3.76E-03	0.751594	########
