# Supplementary material for: Yixin-Shu Capsules Ameliorated Ischemia-Induced Heart Failure by Restoring Trx2 and Inhibiting JNK/p38 Activation
Source: Oxid Med Cell Longev. 2021 Feb 16;2021:8049079. doi: 10.1155/2021/8049079 (PMC7902134; doi:10.1155/2021/8049079)
Supplement: Supplementary Materials — Antibodies such as Nrf2 (ab89443), Tlr4 (19811-1-AP), and Myd88 (sc-74532) were used for IF staining. As for F-actin staining, the samples were incubated with 0.1% Triton X-100 for 15 min. Rhodamine phalloidin (PHDR1, cytoskeleton) was used for the staining of F-actin after the treatment of 0.1% Triton X-100 for 15 min and then followed by 4,6-DAPI for 10 min before observation. Table S1: the RNA-seq data of failing heart treated with or without YXS or VST. Figure S1: the enrichment of DEs in YXS-mediated protection against H2O2-induced damage; (A) enriched GO terms of upregulated DEs; (B) enriched GO terms of downregulated DEs. Figure S2: YXS decreased the levels of Tlr4 and Myd88, enhanced Nrf2 expression, and improved cytoskeleton arrangement; (A) the IF staining of Tlr4 (red) and Myd88 (green) in H2O2-induced H9C2 cell and the related quantification, nucleus (blue), scale bar: 100 μm (n = 3–5); (B) the IF staining of Nrf2 (green) and F-actin (red) in heart tissue, nucleus (blue), scale bar: 100 μm; (C) the IF staining of Nrf2 (green) in H2O2-induced H9C2 cell and the related quantification, nucleus (blue), scale bar: 100 μm (n = 3–5). [file 8049079.f1.zip › Xiang.Table S1 HF+YXS-H vs HF.pdf]

HF+YXS-H vs HF

Gene	FDR	LR	PValue	logCPM	logFC
Mmp9	5.38E-05	2.23E+01	2.36E-06	2.192278	1.02E+00
Ovch2	2.13E-02	9.65E+00	1.90E-03	-1.87457	2.42E+00
Gpr63	1.23E-05	2.53E+01	4.81E-07	2.076417	1.14E+00
Eya2	3.51E-22	1.03E+02	3.04E-24	4.22937	1.02E+00
AABR07042	3.47E-05	2.32E+01	1.46E-06	0.457461	1.67E+00
Angptl4	1.96E-26	1.23E+02	1.39E-28	2.806553	1.79E+00
Grid1	1.03E-04	2.09E+01	4.82E-06	0.550321	1.56E+00
Rn50_2_22	1.27E-02	1.08E+01	1.04E-03	-1.47713	3.65E+00
Zfp36l2	3.81E-02	8.38E+00	3.78E-03	4.045232	2.04E+00
Amer2	5.36E-03	1.26E+01	3.87E-04	-2.43923	4.72E+00
RGD156457	2.09E-02	9.69E+00	1.86E-03	-2.58662	4.37E+00
Tmco3	7.32E-03	1.19E+01	5.47E-04	1.082635	1.81E+00
Myo16	2.68E-03	1.41E+01	1.77E-04	-0.13726	1.52E+00
Ghrhr	1.90E-02	9.89E+00	1.66E-03	-0.56471	1.51E+00
LOC102554	2.23E-03	1.45E+01	1.44E-04	0.532287	3.75E+00
AABR07072	1.46E-02	1.05E+01	1.22E-03	-0.68439	1.67E+00
Cetn3	3.44E-02	8.61E+00	3.34E-03	2.430605	1.67E+00
Rn50_11_C	2.51E-14	6.64E+01	3.61E-16	0.828851	2.83E+00
RGD156414	1.40E-02	1.06E+01	1.16E-03	-1.09463	1.95E+00
Ebna1bp2	4.87E-02	7.84E+00	5.11E-03	0.534847	1.58E+00
Ano5	1.47E-71	3.33E+02	2.18E-74	4.456854	1.72E+00
LOC691153	2.46E-09	4.29E+01	5.74E-11	2.553212	1.18E+00
LOC10369C	2.11E-02	9.66E+00	1.88E-03	0.554769	2.64E+00
Gal	5.83E-10	4.59E+01	1.27E-11	0.817906	2.39E+00
Irs2	########	1.20E+03	########	6.62145	1.52E+00
Hspa1b	9.87E-47	2.18E+02	2.77E-49	4.801752	3.24E+00
Garem2	8.02E-03	1.18E+01	6.08E-04	0.518515	1.10E+00
Kcnq3	8.41E-03	1.17E+01	6.41E-04	-2.18655	3.30E+00
Dusp15	4.86E-21	9.79E+01	4.49E-23	4.109262	1.09E+00
Sp7	5.17E-03	1.27E+01	3.72E-04	-1.06888	1.97E+00
RGD156488	2.21E-02	9.56E+00	1.99E-03	1.608357	1.05E+00
Hspa1a	1.27E-02	1.08E+01	1.03E-03	0.539227	1.85E+00
Atp1b4	2.55E-02	9.26E+00	2.34E-03	-0.35099	1.43E+00
Smco1	1.43E-04	2.02E+01	6.88E-06	-1.06817	2.66E+00
Esrp2	9.28E-03	1.14E+01	7.18E-04	-1.50694	2.27E+00
Ogn	8.06E-04	1.66E+01	4.60E-05	1.956805	1.96E+00
Notch4	3.80E-96	4.47E+02	3.17E-99	6.47428	1.06E+00
Fam222a	3.53E-11	5.16E+01	6.74E-13	0.956992	2.22E+00
Sbk3	4.47E-11	5.11E+01	8.57E-13	0.549231	2.41E+00
Nos1ap	6.32E-06	2.67E+01	2.33E-07	2.303522	1.01E+00
Tshb	9.54E-03	1.14E+01	7.40E-04	-1.83085	3.07E+00
Mybphl	1.39E-13	6.30E+01	2.10E-15	1.722709	1.91E+00
AABR07064	########	4.75E+02	########	5.637497	1.24E+00
Unc80	4.95E-03	1.28E+01	3.53E-04	-2.13052	3.41E+00
Myl4	8.81E-43	1.99E+02	2.96E-45	5.134262	1.01E+00
Rpusd2	2.84E-02	9.02E+00	2.66E-03	1.224101	2.47E+00
Nr4a3	4.41E-94	4.37E+02	3.95E-97	3.598317	3.07E+00
Slc4a1	1.04E-38	1.80E+02	4.16E-41	3.700769	1.76E+00
Ccdc142	1.93E-06	2.92E+01	6.56E-08	0.642422	2.25E+00
Col9a2	2.21E-05	2.41E+01	9.00E-07	1.370666	1.29E+00
RGD156307	1.46E-04	2.02E+01	7.03E-06	1.719356	2.63E+00
Fermt1	1.55E-02	1.03E+01	1.30E-03	-2.58687	4.37E+00

Siglech	7.99E-03	1.18E+01	6.05E-04	-2.18868	3.30E+00
Natd1	1.13E-02	1.10E+01	9.04E-04	0.048577	3.06E+00
Slc8a3	4.96E-02	7.80E+00	5.21E-03	-0.48714	1.30E+00
Ptp4a1	6.28E-03	1.23E+01	4.62E-04	0.902683	2.00E+00
Pcdh9	4.18E-02	8.17E+00	4.25E-03	-1.70122	1.91E+00
Myl7	0.00E+00	2.10E+03	0.00E+00	6.621567	2.39E+00
Ccnj	3.77E-02	8.41E+00	3.74E-03	-1.86583	3.89E+00
Hs3st3b1	8.80E-05	2.12E+01	4.05E-06	0.045185	1.80E+00
Noct	1.31E-24	1.14E+02	1.02E-26	4.075286	1.18E+00
Ajap1	4.01E-03	1.32E+01	2.78E-04	0.523289	1.21E+00
LOC100912	2.50E-02	9.30E+00	2.29E-03	-1.51884	4.32E+00
Col9a1	3.04E-05	2.35E+01	1.27E-06	0.913114	1.49E+00
Mrpl53	1.05E-02	1.12E+01	8.24E-04	2.242397	2.14E+00
Col10a1	5.67E-13	6.01E+01	9.08E-15	2.555668	1.40E+00
Sbk2	3.09E-08	3.77E+01	8.20E-10	0.627803	1.99E+00
LOC103694	1.14E-05	2.55E+01	4.42E-07	6.985856	1.52E+00
Mybpc1	9.29E-04	1.63E+01	5.38E-05	-1.09854	2.37E+00
Kcnh1	1.08E-02	1.11E+01	8.54E-04	-1.50929	2.27E+00
Rmt1	3.66E-02	8.48E+00	3.60E-03	-2.02639	2.74E+00
LOC100912	6.32E-03	1.23E+01	4.65E-04	0.351918	3.05E+00
Scn10a	2.04E-02	9.74E+00	1.81E-03	-2.58712	4.37E+00
Ucp3	2.01E-41	1.93E+02	7.12E-44	3.242398	1.91E+00
Dyrk3	3.78E-08	3.73E+01	1.01E-09	1.980922	1.38E+00
Hif3a	1.51E-04	2.01E+01	7.33E-06	1.076322	1.24E+00
Pik3c2g	1.45E-06	2.98E+01	4.85E-08	1.562687	1.38E+00
Fam134b	1.88E-02	9.91E+00	1.64E-03	1.880821	2.14E+00
Sytl2	1.53E-02	1.04E+01	1.28E-03	-0.01203	3.87E+00
Abhd10	3.75E-02	8.42E+00	3.72E-03	0.833521	4.07E+00
LOC10091C	4.86E-03	1.28E+01	3.45E-04	3.885267	2.82E+00
Reg3b	5.91E-03	1.24E+01	4.31E-04	0.56211	1.21E+00
Ntsr1	6.70E-06	2.66E+01	2.49E-07	1.144269	1.48E+00
Csdc2	1.42E-40	1.89E+02	5.23E-43	4.60143	1.13E+00
Adra2c	3.95E-03	1.32E+01	2.74E-04	0.262476	1.36E+00
Cadm2	4.32E-02	8.10E+00	4.43E-03	0.098403	1.04E+00
LOC100302	4.43E-02	8.05E+00	4.56E-03	-0.32075	1.16E+00
Lvrn	4.28E-02	8.12E+00	4.37E-03	0.382388	1.07E+00
Sh2d4b	1.67E-02	1.02E+01	1.43E-03	-1.87678	2.42E+00
LOC10369C	1.22E-02	1.08E+01	9.90E-04	0.884655	1.21E+00
Vwa5a	1.10E-02	1.11E+01	8.73E-04	-0.67594	2.08E+00
Srrm3	3.09E-02	8.84E+00	2.94E-03	0.059983	1.16E+00
Gria2	4.24E-02	8.15E+00	4.32E-03	-0.45519	1.40E+00
Abat	4.65E-03	1.29E+01	3.28E-04	-0.31728	1.42E+00
Sln	2.40E-75	3.51E+02	3.26E-78	4.336414	2.14E+00
Dr1	6.75E-05	2.18E+01	3.02E-06	2.526851	2.97E+00
Magea11	1.18E-03	1.58E+01	7.03E-05	-1.43202	2.73E+00
Ncbp2	7.53E-03	1.19E+01	5.66E-04	1.9671	1.79E+00
Brms1	6.71E-04	1.70E+01	3.74E-05	1.753127	1.67E+00
Slc17a7	6.20E-03	1.23E+01	4.54E-04	0.659163	1.06E+00
Klhl34	4.96E-05	2.25E+01	2.15E-06	1.798023	1.06E+00
Adcy1	9.47E-15	6.85E+01	1.30E-16	3.173317	1.18E+00
RGD156366	2.98E-02	8.92E+00	2.82E-03	-2.66467	4.16E+00
Col2a1	########	8.71E+02	########	6.157645	1.41E+00
LOC100911	4.34E-02	8.09E+00	4.45E-03	-0.35544	2.12E+00
Mgarp	4.85E-02	7.85E+00	5.08E-03	-0.8449	1.51E+00

Bmp10	7.94E-20	9.21E+01	8.23E-22	0.937077	3.57E+00
RGD15607§	2.03E-02	9.75E+00	1.79E-03	3.731454	1.65E+00
LOC69012€	3.46E-02	8.60E+00	3.36E-03	-1.87802	2.42E+00
RGD13091C	2.24E-03	1.44E+01	1.45E-04	-2.3061	5.01E+00
Pdk4	0.00E+00	2.98E+03	0.00E+00	7.712467	2.16E+00
Mapk7	5.22E-03	1.27E+01	3.76E-04	-1.98098	5.54E+00
LOC10091C	2.02E-03	1.47E+01	1.28E-04	3.79529	1.94E+00
LOC10091C	2.86E-02	9.01E+00	2.69E-03	-0.25846	########
LOC100911	7.83E-03	1.18E+01	5.91E-04	0.533742	########
Ntrk3	1.95E-04	1.96E+01	9.74E-06	0.905337	########
RT1-CE3	9.36E-04	1.63E+01	5.42E-05	1.42356	########
C4a	1.41E-70	3.28E+02	2.18E-73	5.658235	########
Ccl12	5.77E-03	1.24E+01	4.19E-04	-0.25235	########
LOC100361	4.03E-02	8.27E+00	4.04E-03	-2.59278	########
RGD156184	8.07E-05	2.14E+01	3.68E-06	0.893095	########
Fcar	3.70E-03	1.34E+01	2.54E-04	-2.32293	########
LOC10091C	3.88E-03	1.33E+01	2.68E-04	-0.80311	########
Rn50_5_08	1.61E-02	1.02E+01	1.37E-03	-2.52122	########
LOC103694	2.57E-02	9.24E+00	2.36E-03	-1.43176	########
Clec2d	9.32E-08	3.55E+01	2.61E-09	-0.18857	########
Pabpc4l	1.44E-02	1.05E+01	1.20E-03	-1.93748	########
Prima1	1.43E-05	2.50E+01	5.63E-07	1.867655	########
Esm1	5.63E-08	3.65E+01	1.54E-09	1.556524	########
AABR0700C	5.93E-03	1.24E+01	4.32E-04	1.425097	########
Sncg	3.26E-17	8.00E+01	3.80E-19	3.180462	########
Mcoln2	1.37E-02	1.06E+01	1.13E-03	0.055804	########
AABR07034	3.44E-05	2.32E+01	1.45E-06	-0.86394	########
Il1rn	6.95E-04	1.69E+01	3.90E-05	0.73665	########
Arr3	1.85E-02	9.95E+00	1.60E-03	0.316814	########
Dpep3	2.58E-02	9.23E+00	2.38E-03	-0.17055	########
Msln	2.51E-03	1.42E+01	1.65E-04	0.385244	########
Polr2i	6.89E-04	1.69E+01	3.86E-05	2.004538	########
LOC257642	1.94E-02	9.85E+00	1.70E-03	-2.52177	########
Zfp136	4.02E-02	8.27E+00	4.03E-03	0.095308	########
Adh7	8.78E-05	2.12E+01	4.04E-06	1.233108	########
C1qtnf3	7.97E-04	1.66E+01	4.54E-05	0.595605	########
AABR07051	3.56E-02	8.54E+00	3.48E-03	-2.52012	########
Tas1r3	3.51E-02	8.57E+00	3.42E-03	-0.54792	########
Dsc3	2.29E-02	9.49E+00	2.07E-03	-1.93774	########
AABR07051	9.01E-03	1.15E+01	6.92E-04	-2.32401	########
Ptges	4.15E-02	8.19E+00	4.21E-03	-1.6751	########
C1qtnf5	1.35E-22	1.05E+02	1.13E-24	4.321953	########
Ccl26	3.81E-02	8.38E+00	3.79E-03	-2.51516	########
Ighg	9.77E-03	1.13E+01	7.61E-04	-2.0928	########
Fuom	2.35E-02	9.43E+00	2.13E-03	1.123098	########
Sfrp4	2.34E-04	1.92E+01	1.19E-05	0.732358	########
Cgref1	3.24E-06	2.81E+01	1.14E-07	1.29652	########
Lingo1	8.52E-03	1.16E+01	6.49E-04	0.849748	########
Ppial4d	3.39E-02	8.64E+00	3.28E-03	3.911851	########
Cnr1	3.54E-02	8.55E+00	3.46E-03	-1.09465	########
Ighv8-4	2.56E-05	2.38E+01	1.05E-06	-1.8456	########
Chi3l1	6.85E-27	1.25E+02	4.78E-29	4.061427	########
Ppp2r2b	3.03E-03	1.38E+01	2.04E-04	0.098638	########
AABR07051	1.12E-03	1.59E+01	6.64E-05	-1.89881	########

RGD130492	2.60E-03	1.41E+01	1.71E-04	0.874647	########
AABR07065	2.91E-03	1.39E+01	1.95E-04	-2.20195	########
AABR07051	3.03E-03	1.38E+01	2.04E-04	-2.31863	########
Gsta2	3.16E-04	1.86E+01	1.65E-05	-0.14867	########
Slc7a3	1.05E-02	1.12E+01	8.21E-04	-2.45051	########
Ighv1-47	2.40E-05	2.40E+01	9.86E-07	-1.84914	########
Fam3b	2.89E-03	1.39E+01	1.93E-04	-0.45901	########
Polr2l	7.52E-04	1.68E+01	4.26E-05	1.428876	########
Myh3	9.24E-03	1.15E+01	7.14E-04	-0.62297	########
Csf3r	1.27E-03	1.56E+01	7.64E-05	1.193464	########
AABR07037	1.31E-02	1.07E+01	1.07E-03	0.555764	########
Rack1	9.73E-06	2.58E+01	3.74E-07	-1.12962	########
Ms4a7	2.98E-04	1.87E+01	1.54E-05	0.64172	########
Syt15	1.16E-03	1.58E+01	6.87E-05	-0.65951	########
Fam169b	4.49E-02	8.02E+00	4.63E-03	-0.05915	########
Krt8	4.83E-02	7.86E+00	5.06E-03	-0.18922	########
LOC291276	1.77E-02	1.00E+01	1.53E-03	-2.51902	########
Adipoq	9.49E-04	1.63E+01	5.51E-05	0.30691	########
Cyp4f37	2.03E-03	1.47E+01	1.29E-04	0.464635	########
P4ha3	3.51E-03	1.35E+01	2.39E-04	-1.42297	########
LOC103690	1.39E-02	1.06E+01	1.15E-03	-1.65887	########
Ly6g6e	4.03E-02	8.26E+00	4.05E-03	-0.94838	########
Il10	3.55E-02	8.54E+00	3.47E-03	-2.38849	########
Klkb1	3.79E-04	1.82E+01	2.00E-05	0.012802	########
Pcdhb21	1.80E-02	1.00E+01	1.56E-03	0.170557	########
C3	2.35E-07	3.36E+01	6.94E-09	2.396528	########
Sbsn	1.03E-17	8.23E+01	1.16E-19	2.788517	########
Fbln7	1.75E-05	2.46E+01	7.03E-07	1.126618	########
Foxl1	4.96E-02	7.80E+00	5.22E-03	-2.52507	########
Six2	7.47E-03	1.19E+01	5.61E-04	-0.25029	########
Fam180a	2.19E-05	2.41E+01	8.94E-07	1.859561	########
Cdkn2b	8.78E-03	1.16E+01	6.72E-04	-0.22931	########
RT1-N2	5.88E-25	1.16E+02	4.49E-27	4.124251	########
Serpinf1	########	4.78E+02	########	6.574195	########
Ccl7	9.81E-05	2.10E+01	4.56E-06	1.216084	########
LOC100911	4.43E-03	1.30E+01	3.11E-04	-0.38008	########
Pcgf1	6.07E-09	4.11E+01	1.48E-10	0.089303	########
LOC688459	4.28E-02	8.13E+00	4.37E-03	-2.59666	########
Cd7	1.14E-02	1.10E+01	9.15E-04	0.905952	########
Igsf10	6.21E-46	2.14E+02	1.82E-48	4.862746	########
Tceal5	9.08E-05	2.12E+01	4.20E-06	1.175828	########
Brms1l	5.02E-03	1.27E+01	3.59E-04	1.66636	########
Car3	5.14E-05	2.24E+01	2.24E-06	2.016857	########
LOC691828	3.26E-02	8.72E+00	3.14E-03	-2.59223	########
Adcy2	3.77E-02	8.41E+00	3.73E-03	0.524802	########
Zfp383	3.57E-02	8.53E+00	3.49E-03	-1.30704	########
AABR07046	3.32E-45	2.11E+02	9.95E-48	5.310033	########
Cd163l1	2.71E-02	9.13E+00	2.52E-03	-0.02587	########
Clec11a	3.56E-20	9.37E+01	3.62E-22	4.235137	########
Il17re	1.35E-06	2.99E+01	4.46E-08	0.461992	########
Ccdc3	2.64E-04	1.89E+01	1.35E-05	0.840898	########
RT1-CE6	4.37E-02	8.07E+00	4.49E-03	-1.84468	########
Col8a2	2.29E-17	8.07E+01	2.64E-19	4.039962	########
LOC100910	3.11E-02	8.82E+00	2.97E-03	0.414606	########

Apopt1	1.73E-04	1.98E+01	8.50E-06	1.950734	########
LOC10369C	1.06E-03	1.60E+01	6.24E-05	3.547534	########
AC103179.	1.18E-03	1.58E+01	7.00E-05	0.623753	########
Slc27a6	2.40E-02	9.38E+00	2.20E-03	-0.65125	########
Grem1	2.07E-03	1.46E+01	1.32E-04	-0.28078	########
Rhbg	1.77E-02	1.00E+01	1.53E-03	-2.51902	########
Scube3	7.26E-07	3.12E+01	2.32E-08	1.173505	########
Zfp39	1.95E-03	1.47E+01	1.23E-04	1.148055	########
Pex5	1.64E-02	1.02E+01	1.40E-03	-0.9378	########
Ptx3	1.45E-03	1.54E+01	8.84E-05	-0.16952	########
Cd180	2.68E-03	1.41E+01	1.77E-04	0.974778	########
Cxcl13	1.03E-07	3.52E+01	2.91E-09	2.026777	########
Oasli	5.24E-04	1.75E+01	2.85E-05	1.125105	########
Cxcl1	1.36E-03	1.55E+01	8.26E-05	1.267469	########
LOC102553	8.12E-07	3.10E+01	2.61E-08	1.705363	########
Trh	7.21E-11	5.02E+01	1.41E-12	2.177009	########
LOC10036C	7.97E-04	1.66E+01	4.54E-05	1.647026	########
Dsg3	4.49E-02	8.02E+00	4.63E-03	-1.71575	########
LOC10091C	8.06E-04	1.66E+01	4.60E-05	2.283385	########
Uchl1	1.34E-07	3.47E+01	3.84E-09	1.479039	########
Testin	1.93E-02	9.86E+00	1.69E-03	-0.4602	########
Ccna1	3.01E-02	8.89E+00	2.86E-03	-2.44449	########
Actg2	2.56E-32	1.50E+02	1.37E-34	3.432206	########
C4b	8.01E-90	4.18E+02	7.66E-93	5.883861	########
Espn	4.25E-02	8.14E+00	4.33E-03	-1.52937	########
Fkbp1b	3.20E-02	8.76E+00	3.07E-03	-1.49106	########
Adra2a	8.64E-03	1.16E+01	6.60E-04	-0.60271	########
Ngp	3.27E-02	8.72E+00	3.15E-03	-2.37927	########
Cacna2d4	3.80E-02	8.39E+00	3.77E-03	-2.51571	########
Pcdhb3	1.42E-02	1.05E+01	1.18E-03	0.262937	########
Cnn1	3.54E-18	8.45E+01	3.90E-20	2.930552	########
Ighm	5.68E-21	9.75E+01	5.33E-23	3.758549	########
Tceal7	1.03E-30	1.43E+02	5.96E-33	4.367601	########
AABR07066	1.47E-02	1.04E+01	1.23E-03	0.018298	########
Plac8	7.33E-03	1.19E+01	5.48E-04	1.980862	########
Ankrd23	########	4.86E+02	########	6.138714	########
Islr	########	4.94E+02	########	5.636712	########
Figf	1.93E-14	6.70E+01	2.74E-16	3.583276	########
Scd	3.06E-02	8.86E+00	2.91E-03	0.047712	########
AABR07011	4.41E-09	4.17E+01	1.06E-10	4.190444	########
Egr2	9.53E-13	5.90E+01	1.56E-14	2.55459	########
Grin3a	1.07E-02	1.11E+01	8.41E-04	-1.98081	########
Tpcr12	1.15E-02	1.10E+01	9.22E-04	-2.44941	########
Fam50a	4.39E-02	8.06E+00	4.51E-03	0.474508	########
Cilp	7.56E-53	2.46E+02	1.75E-55	4.526486	########
Pmfbp1	2.59E-03	1.41E+01	1.70E-04	-0.76221	########
AABR07065	1.49E-06	2.97E+01	4.99E-08	-1.60506	########
Tcf23	2.63E-02	9.19E+00	2.44E-03	-0.59983	########
Tlr2	3.05E-06	2.83E+01	1.06E-07	2.0871	########
Polq	1.58E-02	1.03E+01	1.34E-03	0.061733	########
Tcte3	3.76E-02	8.41E+00	3.72E-03	-1.84771	########
Loxl1	7.96E-84	3.90E+02	8.59E-87	5.781265	########
Chodl	1.91E-04	1.96E+01	9.46E-06	0.568838	########
LOC100361	5.18E-08	3.67E+01	1.41E-09	0.609629	########

LOC103693	4.45E-03	1.30E+01	3.12E-04	-0.05007	########
AABR07053	2.41E-02	9.37E+00	2.20E-03	-2.09023	########
LOC299282	5.42E-22	1.02E+02	4.76E-24	1.978569	########
Prrx2	2.56E-04	1.90E+01	1.31E-05	1.526319	########
Dram1	5.96E-07	3.16E+01	1.87E-08	2.316425	########
Osr1	3.73E-13	6.09E+01	5.87E-15	2.496635	########
Bcas1	1.56E-02	1.03E+01	1.32E-03	-1.89143	########
Grhl3	3.14E-04	1.86E+01	1.63E-05	-0.87214	########
Il21r	2.17E-02	9.60E+00	1.94E-03	0.8693	########
MGC105649	1.13E-06	3.03E+01	3.72E-08	2.478988	########
Fcnb	2.29E-09	4.31E+01	5.31E-11	0.571687	########
S100a9	1.66E-05	2.47E+01	6.65E-07	1.364483	########
AABR07065	3.26E-02	8.72E+00	3.14E-03	-1.83721	########
AABR07051	5.65E-05	2.22E+01	2.48E-06	-1.80188	########
Lrrc17	1.41E-09	4.41E+01	3.18E-11	2.402857	########
Slc27a2	3.98E-02	8.29E+00	3.98E-03	0.012141	########
Cpxm2	9.14E-15	6.85E+01	1.25E-16	3.776989	########
Tpm2	2.31E-64	3.00E+02	3.78E-67	5.604167	########
LOC100125	6.78E-03	1.21E+01	5.02E-04	-1.76916	########
AABR07051	1.81E-03	1.49E+01	1.13E-04	-2.09653	########
Mpz	2.58E-05	2.38E+01	1.07E-06	2.325172	########
Ccl19	6.83E-05	2.18E+01	3.06E-06	2.106963	########
RGD131174	8.97E-17	7.79E+01	1.07E-18	3.620736	########
Ifit1	########	6.49E+02	########	4.110884	########
Krt18	9.67E-08	3.54E+01	2.71E-09	-0.53333	########
Il1b	1.76E-04	1.98E+01	8.63E-06	1.452884	########
Sectm1b	7.87E-03	1.18E+01	5.94E-04	0.000666	########
Igfbp2	4.22E-05	2.28E+01	1.81E-06	-1.79938	########
Egr3	4.47E-08	3.70E+01	1.21E-09	2.40365	########
Nlrp12	4.31E-04	1.79E+01	2.31E-05	-1.21285	########
Mff	1.57E-02	1.03E+01	1.33E-03	2.438716	########
Rassf10	1.16E-03	1.58E+01	6.89E-05	0.87665	########
Pappa2	1.47E-07	3.45E+01	4.23E-09	0.980609	########
Wisp2	########	5.73E+02	########	5.261935	########
P2ry12	2.69E-04	1.89E+01	1.38E-05	0.825723	########
Nefl	1.26E-02	1.08E+01	1.03E-03	-2.08842	########
LOC102556	1.97E-06	2.91E+01	6.70E-08	3.162039	########
Thbs4	5.28E-75	3.49E+02	7.33E-78	4.824104	########
Igh-6	3.28E-11	5.18E+01	6.21E-13	2.312991	########
Scg2	4.52E-02	8.00E+00	4.67E-03	-1.84747	########
Adamts17	3.58E-05	2.31E+01	1.51E-06	0.714912	########
Map3k7	4.12E-02	8.21E+00	4.17E-03	1.58362	########
RGD156166	3.86E-02	8.36E+00	3.84E-03	-0.15278	########
Islr2	8.28E-04	1.66E+01	4.73E-05	0.796819	########
Endou	9.87E-05	2.10E+01	4.59E-06	1.370723	########
Itga8	2.85E-13	6.15E+01	4.44E-15	3.18451	########
Matn4	2.65E-05	2.38E+01	1.10E-06	2.002978	########
Scn2a	1.08E-02	1.11E+01	8.60E-04	0.142418	########
NEWGENE_6	9.53E-03	1.14E+01	7.39E-04	0.655765	########
Rplp2	1.20E-02	1.09E+01	9.66E-04	4.055655	########
Rasal1	1.59E-02	1.03E+01	1.35E-03	-1.18876	########
AABR0703C	1.70E-02	1.01E+01	1.46E-03	-1.49775	########
Kcna6	3.68E-02	8.47E+00	3.62E-03	-0.55853	########
Ch25h	7.42E-06	2.64E+01	2.78E-07	1.128653	########

Selp	4.24E-04	1.80E+01	2.26E-05	0.342353	########
Fibin	9.05E-32	1.48E+02	4.94E-34	4.580475	########
LOC10090	1.82E-08	3.88E+01	4.67E-10	3.274685	########
Myl1	2.73E-10	4.74E+01	5.72E-12	2.613781	########
LOC100912	9.87E-83	3.85E+02	1.13E-85	5.165579	########
Ltbp2	########	8.32E+02	########	6.597682	########
AABR07051	4.96E-02	7.80E+00	5.22E-03	-2.59	########
Arntl2	4.49E-02	8.02E+00	4.64E-03	-0.64311	########
Ccl2	9.95E-08	3.53E+01	2.80E-09	2.192658	########
Cst6	2.85E-02	9.01E+00	2.68E-03	-1.76213	########
AABR0706C	5.07E-51	2.38E+02	1.25E-53	4.148926	########
