# Supplementary material for: Yixin-Shu Capsules Ameliorated Ischemia-Induced Heart Failure by Restoring Trx2 and Inhibiting JNK/p38 Activation
Source: Oxid Med Cell Longev. 2021 Feb 16;2021:8049079. doi: 10.1155/2021/8049079 (PMC7902134; doi:10.1155/2021/8049079)
Supplement: Supplementary Materials — Antibodies such as Nrf2 (ab89443), Tlr4 (19811-1-AP), and Myd88 (sc-74532) were used for IF staining. As for F-actin staining, the samples were incubated with 0.1% Triton X-100 for 15 min. Rhodamine phalloidin (PHDR1, cytoskeleton) was used for the staining of F-actin after the treatment of 0.1% Triton X-100 for 15 min and then followed by 4,6-DAPI for 10 min before observation. Table S1: the RNA-seq data of failing heart treated with or without YXS or VST. Figure S1: the enrichment of DEs in YXS-mediated protection against H2O2-induced damage; (A) enriched GO terms of upregulated DEs; (B) enriched GO terms of downregulated DEs. Figure S2: YXS decreased the levels of Tlr4 and Myd88, enhanced Nrf2 expression, and improved cytoskeleton arrangement; (A) the IF staining of Tlr4 (red) and Myd88 (green) in H2O2-induced H9C2 cell and the related quantification, nucleus (blue), scale bar: 100 μm (n = 3–5); (B) the IF staining of Nrf2 (green) and F-actin (red) in heart tissue, nucleus (blue), scale bar: 100 μm; (C) the IF staining of Nrf2 (green) in H2O2-induced H9C2 cell and the related quantification, nucleus (blue), scale bar: 100 μm (n = 3–5). [file 8049079.f1.zip › Xiang.Table S1 HF+YXS-L vs HF.pdf]

	HF+YXS-L vs HF				
gene	FDR	LR	PValue	logCPM	logFC
Gch1	1.88E-06	2.97E+01	5.02E-08	2.21E+00	1.15E+00
AABR07037	1.03E-02	1.13E+01	7.75E-04	########	1.82E+00
Atrx	1.27E-03	1.58E+01	6.94E-05	########	2.61E+00
Slc40a1	1.56E-03	1.54E+01	8.79E-05	2.99E+00	1.33E+00
LOC100362	6.97E-03	1.21E+01	4.91E-04	1.63E+00	2.75E+00
Cxcl14	2.99E-03	1.40E+01	1.86E-04	1.11E+00	1.03E+00
LOC100361	6.42E-04	1.73E+01	3.17E-05	########	2.77E+00
Foxd2	7.16E-03	1.21E+01	5.07E-04	7.97E-01	1.06E+00
Avp	4.28E-02	8.18E+00	4.24E-03	########	1.30E+00
Snip1	2.16E-02	9.70E+00	1.85E-03	9.14E-01	2.95E+00
AABR07002	7.20E-15	6.96E+01	7.40E-17	2.48E-01	3.89E+00
Hmgb3	3.83E-02	8.43E+00	3.69E-03	2.80E+00	1.92E+00
Dyrk3	1.45E-14	6.81E+01	1.56E-16	2.21E+00	1.70E+00
Irs2	########	7.73E+02	########	6.85E+00	1.81E+00
M6pr	1.31E-03	1.58E+01	7.20E-05	1.79E+00	1.53E+00
LOC10036C	2.84E-03	1.41E+01	1.75E-04	1.26E+00	1.37E+00
Ucp3	6.83E-16	7.43E+01	6.60E-18	2.86E+00	1.40E+00
NEWGENE_1	1.47E-02	1.05E+01	1.18E-03	1.90E+00	1.47E+00
Hs3st3b1	3.76E-02	8.47E+00	3.61E-03	########	1.25E+00
Mfsd14a	1.04E-02	1.13E+01	7.86E-04	1.87E+00	1.14E+00
Noct	2.29E-41	1.93E+02	5.52E-44	4.19E+00	1.34E+00
Fhl5	2.89E-02	9.04E+00	2.64E-03	########	1.40E+00
Prrg3	4.35E-02	8.14E+00	4.33E-03	########	2.64E+00
LOC100912	3.10E-03	1.39E+01	1.94E-04	4.41E-01	3.08E+00
Ano5	6.18E-36	1.68E+02	1.93E-38	4.11E+00	1.24E+00
Pdk4	########	1.22E+03	########	7.52E+00	1.92E+00
LOC690326	2.20E-02	9.65E+00	1.89E-03	########	1.51E+00
Hspa1a	1.04E-03	1.63E+01	5.46E-05	8.63E-01	2.26E+00
Klf2	2.18E-22	1.05E+02	1.33E-24	7.78E+00	1.01E+00
LOC100911	1.36E-03	1.57E+01	7.45E-05	########	3.85E+00
Eya2	4.24E-43	2.01E+02	9.93E-46	4.46E+00	1.36E+00
Cetn3	7.24E-03	1.21E+01	5.13E-04	2.99E+00	2.36E+00
Smco1	2.14E-03	1.47E+01	1.26E-04	########	2.31E+00
Spert	4.58E-02	8.02E+00	4.62E-03	########	4.51E+00
Tmprss11g	4.28E-03	1.32E+01	2.82E-04	########	2.96E+00
Gpr63	4.20E-13	6.12E+01	5.12E-15	2.48E+00	1.71E+00
AABR07041	4.35E-02	8.14E+00	4.34E-03	########	3.31E+00
Rpl39	2.86E-02	9.07E+00	2.60E-03	7.32E-01	2.11E+00
Ell2	7.14E-24	1.12E+02	3.97E-26	3.92E+00	1.12E+00
AABR07049	2.75E-02	9.16E+00	2.48E-03	########	4.80E+00
Gal	1.21E-07	3.54E+01	2.69E-09	6.10E-01	2.10E+00
Tlk1	2.81E-02	9.11E+00	2.55E-03	9.98E-01	1.03E+00
Angptl4	1.06E-04	2.12E+01	4.21E-06	2.27E+00	1.02E+00
Grid1	8.43E-03	1.17E+01	6.12E-04	3.03E-01	1.18E+00
Kif4a	1.57E-02	1.04E+01	1.27E-03	3.42E-01	1.08E+00
NEWGENE_6	1.23E-02	1.09E+01	9.55E-04	########	6.11E+00
Tmprss2	1.25E-04	2.08E+01	5.08E-06	1.35E+00	1.34E+00
LOC10255C	1.38E-02	1.06E+01	1.10E-03	4.31E+00	1.07E+00
Cdh26	5.30E-06	2.75E+01	1.56E-07	2.46E+00	1.10E+00
LOC10090S	4.95E-02	7.86E+00	5.06E-03	########	1.27E+00
Nr4a3	########	1.26E+03	########	4.54E+00	4.09E+00
LOC103694	4.16E-04	1.82E+01	1.94E-05	7.20E+00	1.81E+00

Gstm5	1.86E-02	1.00E+01	1.56E-03	8.96E-01	2.82E+00
Slc4a1	2.07E-46	2.17E+02	4.34E-49	3.69E+00	1.75E+00
Atf3	1.72E-27	1.29E+02	7.68E-30	4.14E+00	1.09E+00
Sall1	2.22E-02	9.63E+00	1.92E-03	1.50E-01	1.12E+00
Hspa1b	1.23E-49	2.32E+02	2.23E-52	4.84E+00	3.28E+00
RGD156414	2.20E-02	9.65E+00	1.89E-03	########	1.70E+00
LOC10091C	4.36E-07	3.27E+01	1.05E-08	4.45E+00	3.45E+00
Coq10b	9.09E-36	1.67E+02	2.89E-38	5.01E+00	1.03E+00
Oasl2	4.90E-32	1.50E+02	1.75E-34	4.72E+00	1.03E+00
LOC103692	2.31E-05	2.44E+01	7.95E-07	3.51E+00	2.19E+00
Kcna5	1.52E-19	9.15E+01	1.11E-21	3.89E+00	1.01E+00
Nhp2	5.57E-03	1.26E+01	3.80E-04	1.73E+00	2.79E+00
Notch4	6.68E-52	2.43E+02	1.09E-54	6.54E+00	1.15E+00
LOC10091C	2.97E-03	1.40E+01	1.85E-04	3.14E+00	2.53E+00
Fbxo41	2.66E-02	9.23E+00	2.39E-03	1.09E-01	1.05E+00
Elovl6	1.31E-02	1.08E+01	1.03E-03	########	5.30E+00
Ap1s3	2.25E-04	1.96E+01	9.65E-06	1.60E+00	1.07E+00
Fam134b	6.69E-05	2.21E+01	2.53E-06	1.83E+00	2.08E+00
LOC100359	3.16E-02	8.85E+00	2.93E-03	########	1.88E+00
LOC100365	3.93E-04	1.84E+01	1.82E-05	3.09E+00	1.28E+00
Tprkb	2.13E-02	9.72E+00	1.82E-03	########	4.30E+00
Myo16	4.02E-02	8.31E+00	3.93E-03	########	1.31E+00
Mrpl53	2.68E-03	1.42E+01	1.63E-04	2.80E+00	2.78E+00
Myom3	7.97E-20	9.28E+01	5.71E-22	3.80E+00	1.03E+00
Inhbb	6.80E-14	6.49E+01	7.87E-16	2.84E+00	1.21E+00
Pcdh15	3.98E-02	8.34E+00	3.88E-03	########	4.09E+00
Crem	2.55E-38	1.79E+02	7.33E-41	4.74E+00	1.12E+00
LOC100911	2.68E-04	1.92E+01	1.17E-05	4.34E+00	1.20E+00
Rn50_1_13	8.01E-03	1.19E+01	5.76E-04	########	3.30E+00
Natd1	1.93E-02	9.94E+00	1.62E-03	########	1.88E+00
LOC10369C	1.12E-04	2.11E+01	4.47E-06	########	2.33E+00
Myo7b	2.70E-04	1.92E+01	1.18E-05	1.57E+00	1.06E+00
LOC100912	5.60E-03	1.26E+01	3.83E-04	3.21E+00	1.11E+00
Chrnb2	3.73E-02	8.49E+00	3.57E-03	########	2.29E+00
Cebpb	7.81E-40	1.86E+02	2.05E-42	7.12E+00	1.33E+00
RGD130518	7.92E-05	2.18E+01	3.04E-06	2.02E+00	1.01E+00
Cldn15	1.16E-03	1.60E+01	6.26E-05	1.16E+00	1.08E+00
AABR07027	4.58E-02	8.03E+00	4.61E-03	1.02E-01	1.96E+00
RGD156535	1.29E-16	7.77E+01	1.19E-18	3.71E+00	1.55E+00
Fam222a	6.25E-07	3.20E+01	1.54E-08	7.07E-01	1.88E+00
Vwa5a	1.80E-03	1.51E+01	1.03E-04	########	2.40E+00
Hist1h1t	1.36E-02	1.07E+01	1.08E-03	########	2.79E+00
AABR07036	9.65E-03	1.14E+01	7.17E-04	########	2.21E+00
Adra1d	4.95E-05	2.28E+01	1.82E-06	1.81E+00	1.07E+00
Mepce	7.74E-03	1.19E+01	5.54E-04	9.84E-02	2.07E+00
Fosl2	3.56E-37	1.74E+02	1.08E-39	4.21E+00	1.35E+00
Habp2	2.09E-03	1.47E+01	1.23E-04	9.63E-01	1.08E+00
Ogn	2.61E-04	1.93E+01	1.14E-05	4.02E+00	1.74E+00
Retnlg	1.82E-02	1.01E+01	1.51E-03	########	1.52E+00
Col9a3	5.08E-07	3.24E+01	1.23E-08	1.12E+00	########
Ltbp2	########	5.83E+02	########	6.57E+00	########
Clec2d	1.78E-04	2.01E+01	7.46E-06	########	########
Tnc	1.34E-17	8.23E+01	1.16E-19	2.72E+00	########
Glyatl1	3.47E-07	3.32E+01	8.27E-09	########	########

Acp5	2.22E-10	4.84E+01	3.50E-12	1.99E+00	########
LOC10091C	4.94E-03	1.29E+01	3.30E-04	3.52E-01	########
Cdhr1	2.08E-05	2.46E+01	7.06E-07	1.55E+00	########
LOC100911	2.33E-02	9.52E+00	2.03E-03	5.44E-01	########
Gdf10	1.46E-06	3.02E+01	3.84E-08	########	########
Aass	4.68E-02	7.98E+00	4.74E-03	########	########
AABR07065	2.05E-03	1.48E+01	1.20E-04	########	########
LOC103693	3.99E-02	8.33E+00	3.89E-03	1.58E-02	########
Cd180	1.82E-03	1.50E+01	1.05E-04	9.46E-01	########
Mmp12	1.63E-02	1.03E+01	1.33E-03	########	########
Adamts17	6.52E-03	1.23E+01	4.54E-04	8.34E-01	########
Mup5	5.72E-03	1.26E+01	3.92E-04	########	########
Kcne1	3.95E-04	1.84E+01	1.83E-05	8.07E-01	########
Selp	6.96E-03	1.22E+01	4.91E-04	4.07E-01	########
Col8a1	2.53E-93	4.34E+02	1.95E-96	5.83E+00	########
Pi16	1.14E-81	3.81E+02	9.12E-85	7.24E+00	########
Pmfbp1	1.07E-02	1.12E+01	8.15E-04	########	########
Tpm2	5.41E-64	2.99E+02	6.01E-67	5.64E+00	########
Grem1	6.13E-06	2.72E+01	1.83E-07	########	########
Cd163l1	3.17E-06	2.86E+01	8.81E-08	########	########
Pex5	1.45E-02	1.06E+01	1.16E-03	########	########
Igsf10	3.71E-40	1.88E+02	9.62E-43	4.84E+00	########
Asic5	3.56E-02	8.59E+00	3.38E-03	########	########
Asns	9.97E-03	1.14E+01	7.45E-04	2.40E-01	########
Panx3	2.10E-04	1.97E+01	8.91E-06	########	########
Itga8	1.94E-14	6.75E+01	2.13E-16	3.16E+00	########
Tas1r3	1.27E-05	2.57E+01	4.05E-07	########	########
Snai2	5.32E-03	1.27E+01	3.60E-04	6.51E-01	########
Grhl3	1.14E-03	1.61E+01	6.12E-05	########	########
AABR07051	2.14E-03	1.47E+01	1.27E-04	########	########
AABR0706C	########	5.18E+02	########	3.95E+00	########
Greb1l	3.34E-02	8.74E+00	3.12E-03	1.95E-01	########
Tlr10	1.58E-02	1.04E+01	1.28E-03	########	########
Podnl1	3.14E-02	8.86E+00	2.91E-03	########	########
LOC102553	3.49E-02	8.63E+00	3.30E-03	########	########
Clec2d	4.59E-02	8.02E+00	4.63E-03	########	########
LOC684208	2.32E-02	9.53E+00	2.02E-03	########	########
Lrrc17	3.43E-06	2.85E+01	9.57E-08	2.45E+00	########
Col8a2	1.07E-47	2.23E+02	2.15E-50	3.88E+00	########
NEWGENE_6	9.51E-03	1.15E+01	7.05E-04	6.40E-01	########
Actg2	8.21E-28	1.30E+02	3.65E-30	3.50E+00	########
Fst	4.16E-04	1.82E+01	1.94E-05	9.15E-01	########
Rpl30	2.26E-50	2.35E+02	3.97E-53	5.62E+00	########
Igh-6	1.18E-16	7.79E+01	1.07E-18	2.15E+00	########
Il1rn	3.64E-03	1.35E+01	2.33E-04	7.56E-01	########
AABR07051	6.75E-03	1.22E+01	4.72E-04	########	########
Uchl1	1.16E-07	3.55E+01	2.58E-09	1.45E+00	########
LOC100912	1.21E-02	1.09E+01	9.38E-04	2.23E-01	########
RGD131174	8.06E-36	1.68E+02	2.54E-38	3.48E+00	########
LOC100911	1.39E-02	1.06E+01	1.11E-03	########	########
Col11a1	1.94E-29	1.38E+02	8.04E-32	2.81E+00	########
Chi3l1	1.06E-26	1.25E+02	5.02E-29	4.11E+00	########
Col10a1	6.46E-21	9.79E+01	4.35E-23	8.36E-01	########
AABR07051	1.31E-03	1.58E+01	7.15E-05	########	########

Zfp7	3.10E-02	8.89E+00	2.87E-03	########	########
Fbln7	1.06E-07	3.57E+01	2.34E-09	1.04E+00	########
AABR07051	1.31E-02	1.08E+01	1.03E-03	########	########
Rab17	2.24E-02	9.61E+00	1.93E-03	########	########
Fam180a	4.05E-11	5.19E+01	5.99E-13	1.63E+00	########
Wisp1	5.43E-07	3.23E+01	1.32E-08	########	########
Igfbp2	1.29E-02	1.08E+01	1.01E-03	########	########
Slc27a2	5.93E-05	2.24E+01	2.22E-06	########	########
Evi2a	8.37E-04	1.67E+01	4.31E-05	1.30E+00	########
C1ql3	2.34E-02	9.51E+00	2.04E-03	1.59E-01	########
Col1a1	6.96E-96	4.46E+02	4.72E-99	9.11E+00	########
Tnfsf18	5.32E-04	1.77E+01	2.55E-05	########	########
Sncg	1.50E-17	8.21E+01	1.31E-19	3.19E+00	########
Knop1	5.33E-03	1.27E+01	3.61E-04	########	########
AABR07066	1.19E-02	1.10E+01	9.19E-04	1.43E-02	########
LOC100912	6.80E-04	1.72E+01	3.40E-05	########	########
Esyt3	5.71E-06	2.74E+01	1.69E-07	3.90E-01	########
LOC682793	1.04E-07	3.57E+01	2.30E-09	1.70E+00	########
Nov	2.42E-29	1.37E+02	1.01E-31	3.94E+00	########
Adamts18	1.10E-02	1.12E+01	8.39E-04	########	########
Pak1	1.70E-03	1.52E+01	9.72E-05	1.35E+00	########
Myh3	1.05E-02	1.13E+01	7.89E-04	########	########
Fam221a	4.12E-02	8.26E+00	4.05E-03	########	########
Sectm1b	7.47E-04	1.70E+01	3.79E-05	########	########
LOC100361	1.24E-06	3.06E+01	3.21E-08	6.42E-01	########
Bmp3	3.80E-02	8.45E+00	3.65E-03	########	########
Grem2	1.21E-04	2.09E+01	4.90E-06	########	########
RGD130492	4.99E-06	2.76E+01	1.46E-07	6.95E-01	########
Rn50_11_C	5.44E-06	2.75E+01	1.60E-07	########	########
Anxa8	8.03E-05	2.18E+01	3.10E-06	########	########
Nmnat2	2.39E-02	9.47E+00	2.09E-03	########	########
Scd	1.10E-06	3.08E+01	2.82E-08	########	########
Kcnab1	2.17E-05	2.45E+01	7.41E-07	1.19E+00	########
Bnc2	1.78E-02	1.01E+01	1.47E-03	5.29E-01	########
Timp1	4.58E-28	1.31E+02	2.01E-30	4.08E+00	########
Serpinf1	2.45E-73	3.42E+02	2.42E-76	6.59E+00	########
AABR07051	2.76E-02	9.15E+00	2.49E-03	########	########
Hapln1	9.60E-08	3.59E+01	2.11E-09	3.96E-01	########
Zcchc12	4.85E-02	7.90E+00	4.94E-03	########	########
Surf2	1.20E-03	1.60E+01	6.47E-05	2.34E+00	########
Figf	3.64E-20	9.44E+01	2.54E-22	3.54E+00	########
Cnn1	9.88E-18	8.29E+01	8.44E-20	2.94E+00	########
Ighv1-47	1.80E-03	1.51E+01	1.03E-04	########	########
Olr1637	2.24E-02	9.61E+00	1.93E-03	########	########
Fmod	2.14E-31	1.47E+02	8.08E-34	3.44E+00	########
LOC100911	7.02E-05	2.20E+01	2.67E-06	3.63E+00	########
Pcgf1	2.89E-09	4.31E+01	5.15E-11	5.92E-02	########
AABR07065	7.65E-07	3.16E+01	1.92E-08	########	########
Cp	1.44E-35	1.66E+02	4.66E-38	4.79E+00	########
Srpx2	2.08E-05	2.46E+01	7.04E-07	2.13E+00	########
Slc27a6	1.34E-02	1.07E+01	1.05E-03	########	########
Atp6ap1l	1.31E-04	2.07E+01	5.36E-06	1.39E+00	########
LOC103689	4.76E-02	7.94E+00	4.84E-03	2.18E+00	########
Msln	8.54E-05	2.16E+01	3.30E-06	3.16E-01	########

Cpz	2.10E-03	1.47E+01	1.24E-04	1.52E+00	########
Col12a1	3.87E-23	1.08E+02	2.23E-25	3.65E+00	########
Six2	6.35E-03	1.23E+01	4.42E-04	########	########
NEWGENE_1	1.82E-02	1.01E+01	1.51E-03	4.05E-01	########
RGD156184	1.41E-03	1.56E+01	7.82E-05	9.52E-01	########
Tmem45b	1.23E-02	1.09E+01	9.50E-04	########	########
Trh	5.31E-13	6.07E+01	6.58E-15	2.15E+00	########
Btnl10	1.75E-02	1.01E+01	1.45E-03	########	########
AABR07034	3.19E-05	2.37E+01	1.13E-06	########	########
Clec11a	1.67E-46	2.17E+02	3.44E-49	4.09E+00	########
C3	4.41E-16	7.52E+01	4.18E-18	2.23E+00	########
Bcan	5.92E-04	1.75E+01	2.88E-05	########	########
Cilp2	3.09E-23	1.09E+02	1.77E-25	1.91E+00	########
Gpm6a	1.50E-12	5.86E+01	1.93E-14	3.10E+00	########
Rn50_14_C	2.99E-02	8.97E+00	2.74E-03	########	########
Selenbp1	6.06E-20	9.34E+01	4.29E-22	3.27E+00	########
Testin	7.97E-03	1.19E+01	5.73E-04	########	########
AABR07066	2.32E-02	9.53E+00	2.03E-03	########	########
Chodl	5.42E-08	3.70E+01	1.15E-09	4.37E-01	########
Comp	########	7.28E+02	########	4.42E+00	########
Triap1	9.60E-06	2.63E+01	2.99E-07	1.39E+00	########
Ptx3	1.13E-03	1.61E+01	6.04E-05	########	########
Hk3	3.26E-03	1.38E+01	2.06E-04	5.45E-01	########
Col2a1	0.00E+00	1.68E+03	0.00E+00	4.30E+00	########
Sfrp2	########	5.49E+02	########	4.62E+00	########
Islr	1.85E-79	3.70E+02	1.60E-82	5.66E+00	########
AABR07051	3.66E-05	2.34E+01	1.32E-06	########	########
Pcsk1	4.35E-02	8.14E+00	4.34E-03	########	########
Pnoc	1.49E-04	2.04E+01	6.14E-06	########	########
Sez6l	5.94E-04	1.75E+01	2.89E-05	########	########
Krt18	3.12E-03	1.39E+01	1.95E-04	########	########
Tnfrsf19	1.78E-02	1.01E+01	1.47E-03	7.25E-01	########
Dpep3	3.03E-03	1.39E+01	1.89E-04	########	########
Ccl26	3.05E-02	8.92E+00	2.81E-03	########	########
Bgn	########	6.06E+02	########	8.16E+00	########
Ptgs2	2.14E-07	3.42E+01	4.97E-09	2.02E+00	########
Ntrk3	1.40E-05	2.55E+01	4.49E-07	8.27E-01	########
Bmp8a	4.26E-02	8.19E+00	4.21E-03	########	########
Fcnb	3.12E-04	1.89E+01	1.40E-05	7.44E-01	########
Ighg	6.90E-03	1.22E+01	4.85E-04	########	########
Pi15	2.94E-02	9.00E+00	2.69E-03	########	########
Ahnak2	1.03E-45	2.14E+02	2.22E-48	5.02E+00	########
Ibsp	4.52E-61	2.85E+02	5.44E-64	1.79E+00	########
Adh7	2.44E-04	1.94E+01	1.06E-05	1.24E+00	########
Matn4	7.05E-09	4.13E+01	1.33E-10	1.91E+00	########
Scube3	2.96E-04	1.90E+01	1.31E-05	1.29E+00	########
Osr1	2.08E-09	4.38E+01	3.65E-11	2.59E+00	########
Acan	1.32E-38	1.81E+02	3.72E-41	1.73E+00	########
Gpr39	1.50E-03	1.55E+01	8.37E-05	########	########
AABR07051	8.39E-04	1.67E+01	4.33E-05	########	########
Prrx2	2.57E-05	2.41E+01	8.95E-07	1.44E+00	########
Mmp13	1.11E-19	9.22E+01	8.02E-22	1.93E-01	########
Cemip	1.12E-02	1.11E+01	8.58E-04	2.22E-01	########
Ighv12-3	6.58E-04	1.73E+01	3.27E-05	########	########

AABR0703C	1.31E-03	1.58E+01	7.21E-05	########	########
Mfap5	5.35E-48	2.24E+02	1.01E-50	5.34E+00	########
Krt8	2.68E-02	9.21E+00	2.41E-03	########	########
Cdkn2b	2.30E-02	9.55E+00	2.00E-03	########	########
Nlrp10	9.94E-03	1.14E+01	7.42E-04	########	########
Dnah7	1.27E-08	4.00E+01	2.48E-10	2.42E+00	########
LOC257642	1.44E-02	1.06E+01	1.15E-03	########	########
N4bp3	9.63E-03	1.15E+01	7.14E-04	7.36E-02	########
C4b	1.98E-59	2.78E+02	2.57E-62	5.92E+00	########
Apopt1	3.43E-05	2.35E+01	1.23E-06	1.91E+00	########
Col17a1	7.16E-06	2.69E+01	2.18E-07	1.83E+00	########
AABR07021	4.35E-02	8.14E+00	4.33E-03	########	########
Chad	2.27E-20	9.54E+01	1.55E-22	2.64E+00	########
Il1b	7.76E-04	1.69E+01	3.94E-05	1.49E+00	########
C1qtnf3	9.88E-07	3.10E+01	2.52E-08	4.63E-01	########
Aox1	5.87E-25	1.17E+02	3.10E-27	4.44E+00	########
Cilp	6.38E-33	1.54E+02	2.21E-35	4.65E+00	########
R3hdml	4.68E-02	7.97E+00	4.75E-03	########	########
Wisp2	########	1.03E+03	########	5.04E+00	########
Has1	2.89E-03	1.41E+01	1.78E-04	1.12E+00	########
Cnr1	1.20E-02	1.10E+01	9.29E-04	########	########
Postn	6.36E-63	2.94E+02	7.26E-66	5.07E+00	########
Cdhr3	1.13E-02	1.11E+01	8.66E-04	########	########
Plekhg4	1.89E-02	9.98E+00	1.58E-03	########	########
Ctsk	1.52E-39	1.85E+02	4.13E-42	4.83E+00	########
Rbpjl	6.55E-03	1.23E+01	4.56E-04	########	########
Esm1	5.81E-07	3.22E+01	1.42E-08	1.58E+00	########
Arhgef16	6.01E-03	1.25E+01	4.16E-04	########	########
Igfbp6	8.50E-60	2.79E+02	1.05E-62	5.61E+00	########
Bglap	5.41E-37	1.73E+02	1.65E-39	1.20E+00	########
Clec3a	2.80E-45	2.12E+02	6.13E-48	1.49E+00	########
Fcrl2	1.07E-05	2.60E+01	3.36E-07	1.19E+00	########
Retn	3.93E-02	8.37E+00	3.81E-03	########	########
AABR07011	2.21E-06	2.94E+01	5.98E-08	4.22E+00	########
LOC100911	4.07E-03	1.33E+01	2.65E-04	8.13E-01	########
Cx3cr1	1.59E-02	1.04E+01	1.29E-03	########	########
Necab1	3.36E-02	8.72E+00	3.15E-03	########	########
Fibin	1.44E-37	1.76E+02	4.31E-40	4.56E+00	########
Nr5a2	4.16E-02	8.24E+00	4.09E-03	########	########
Fam3b	2.24E-02	9.61E+00	1.93E-03	########	########
LOC299282	1.65E-09	4.43E+01	2.86E-11	2.21E+00	########
AABR07065	2.48E-03	1.44E+01	1.49E-04	########	########
Robo2	1.73E-03	1.52E+01	9.90E-05	9.20E-01	########
AABR07025	1.41E-02	1.06E+01	1.13E-03	########	########
Ephb2	4.26E-02	8.19E+00	4.22E-03	########	########
C1qtnf5	2.21E-33	1.56E+02	7.56E-36	4.27E+00	########
Klkb1	1.36E-10	4.94E+01	2.12E-12	########	########
Ildr2	1.13E-02	1.11E+01	8.62E-04	5.81E-01	########
Egr2	6.03E-09	4.16E+01	1.12E-10	2.63E+00	########
Trappc2b	4.88E-02	7.89E+00	4.98E-03	5.44E-01	########
Rims2	2.68E-02	9.21E+00	2.40E-03	########	########
Nkd1	4.66E-06	2.78E+01	1.35E-07	1.72E+00	########
Samsn1	4.09E-03	1.33E+01	2.67E-04	6.27E-01	########
Frzb	7.31E-12	5.54E+01	9.99E-14	2.59E+00	########

RGD130564	6.60E-13	6.03E+01	8.28E-15	2.53E+00	########
Sfrp4	7.33E-05	2.20E+01	2.79E-06	6.95E-01	########
C4a	4.97E-81	3.78E+02	4.14E-84	5.63E+00	########
Rfxapl1	3.25E-02	8.79E+00	3.02E-03	5.51E-01	########
Spp1	########	5.01E+02	########	4.64E+00	########
Fam111a	0.00E+00	3.66E+03	0.00E+00	5.58E+00	########
Il17re	5.67E-07	3.22E+01	1.38E-08	4.36E-01	########
Rprm	6.58E-03	1.23E+01	4.59E-04	########	########
Cpxm2	5.24E-22	1.03E+02	3.30E-24	3.69E+00	########
Crispld1	3.08E-05	2.38E+01	1.09E-06	3.16E-01	########
Cgref1	1.06E-07	3.57E+01	2.33E-09	1.24E+00	########
Gng8	3.69E-05	2.34E+01	1.33E-06	1.33E+00	########
Ccl20	8.11E-03	1.18E+01	5.86E-04	########	########
Pla2g2a	3.58E-69	3.23E+02	3.64E-72	5.90E+00	########
Wscd2	3.08E-03	1.39E+01	1.93E-04	########	########
Tcf23	3.82E-04	1.84E+01	1.76E-05	########	########
Tnn	1.28E-14	6.84E+01	1.36E-16	6.88E-01	########
Espn	2.40E-03	1.44E+01	1.45E-04	########	########
Capn6	6.21E-04	1.74E+01	3.06E-05	3.58E-02	########
Mmp16	4.96E-03	1.29E+01	3.31E-04	########	########
Slc7a11	2.42E-02	9.44E+00	2.12E-03	########	########
Myl1	1.41E-10	4.93E+01	2.19E-12	2.60E+00	########
Mroh2a	3.33E-02	8.74E+00	3.11E-03	9.31E-02	########
Itga10	4.83E-11	5.15E+01	7.17E-13	1.24E-01	########
Wbp11l1	3.52E-05	2.35E+01	1.26E-06	2.16E+00	########
Pappa2	9.45E-08	3.59E+01	2.07E-09	9.75E-01	########
Hcar1	1.24E-02	1.09E+01	9.68E-04	########	########
Kcna6	9.80E-04	1.64E+01	5.14E-05	########	########
Lrrc15	1.25E-02	1.09E+01	9.76E-04	########	########
Uts2r	2.53E-05	2.42E+01	8.75E-07	########	########
P4ha3	1.78E-03	1.51E+01	1.02E-04	########	########
Loxl1	1.91E-58	2.73E+02	2.54E-61	5.79E+00	########
LOC102552	1.06E-24	1.16E+02	5.65E-27	3.08E+00	########
Pamr1	3.23E-03	1.38E+01	2.04E-04	6.49E-02	########
Mmp9	5.71E-14	6.53E+01	6.50E-16	8.20E-01	########
Emx2	3.99E-02	8.33E+00	3.90E-03	########	########
Syt15	8.65E-05	2.16E+01	3.37E-06	########	########
Ms4a6bl	2.59E-02	9.28E+00	2.31E-03	5.34E-01	########
Mepe	8.57E-06	2.65E+01	2.64E-07	########	########
Cxcl1	8.13E-04	1.68E+01	4.17E-05	1.22E+00	########
P2rx2	1.28E-02	1.08E+01	1.00E-03	########	########
Kcne4	2.97E-02	8.99E+00	2.72E-03	5.42E-01	########
Arntl2	4.85E-02	7.90E+00	4.94E-03	########	########
Cacna2d3	1.05E-02	1.13E+01	7.89E-04	########	########
Tnmd	2.52E-02	9.35E+00	2.23E-03	########	########
S100b	4.29E-04	1.82E+01	2.02E-05	1.62E+00	########
Ptprv	6.02E-09	4.16E+01	1.11E-10	7.38E-01	########
Col9a1	7.93E-04	1.68E+01	4.05E-05	########	########
B4galnt3	3.18E-03	1.38E+01	2.00E-04	########	########
LOC10091C	1.55E-02	1.04E+01	1.25E-03	2.37E+00	########
Gdf6	3.61E-03	1.36E+01	2.31E-04	3.49E-01	########
Lst1	2.22E-02	9.64E+00	1.91E-03	9.16E-03	########
Ighm	7.81E-48	2.24E+02	1.49E-50	3.62E+00	########
LOC10369C	2.54E-02	9.33E+00	2.26E-03	1.76E+00	########

Ighv8-4	3.64E-04	1.85E+01	1.67E-05	########	########
