# Supplementary figures and images for: Yixin-Shu Capsules Ameliorated Ischemia-Induced Heart Failure by Restoring Trx2 and Inhibiting JNK/p38 Activation
Source: Oxid Med Cell Longev. 2021 Feb 16;2021:8049079. doi: 10.1155/2021/8049079 (PMC7902134; doi:10.1155/2021/8049079)

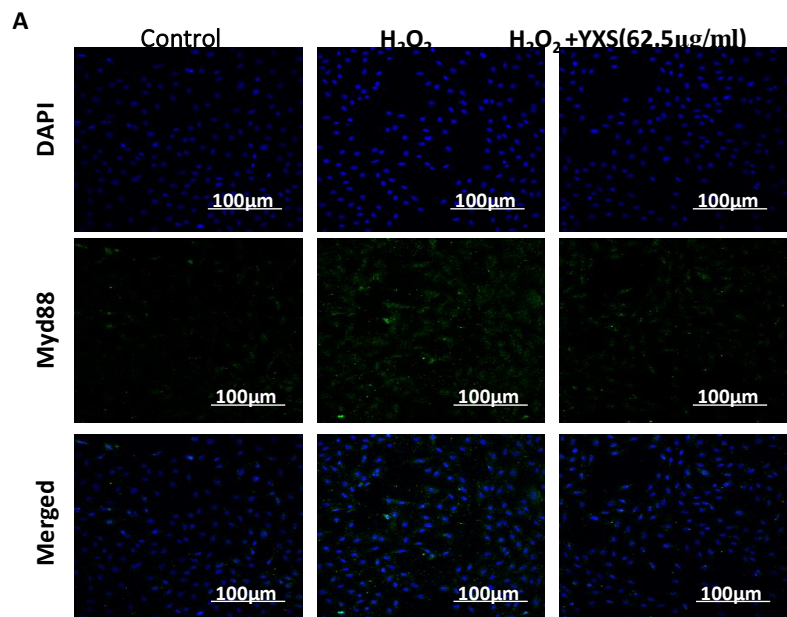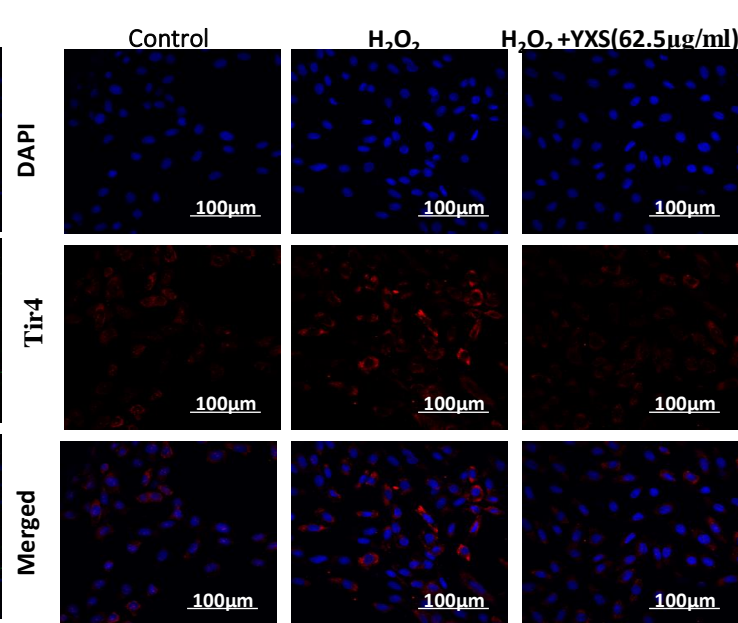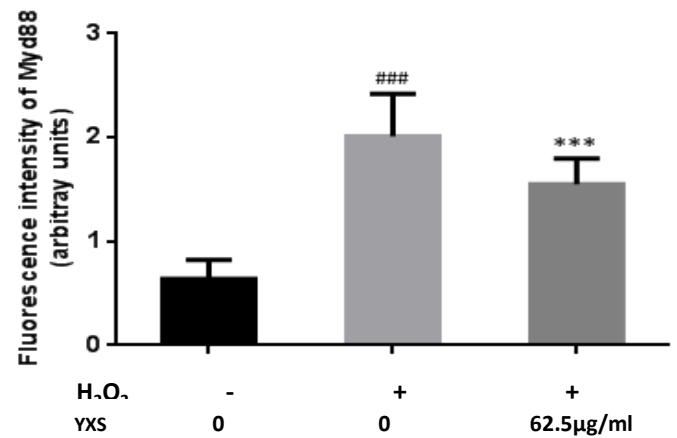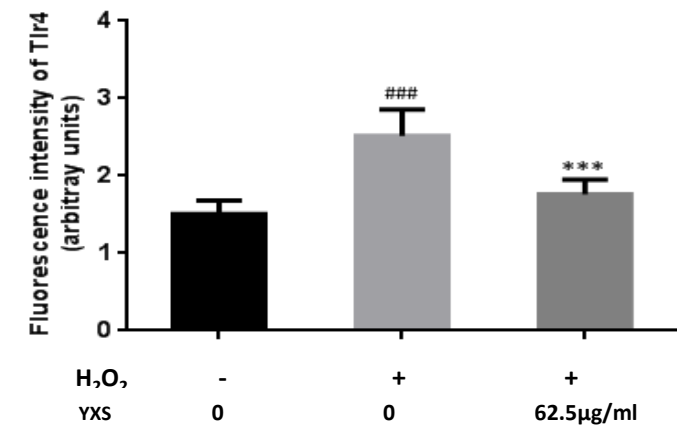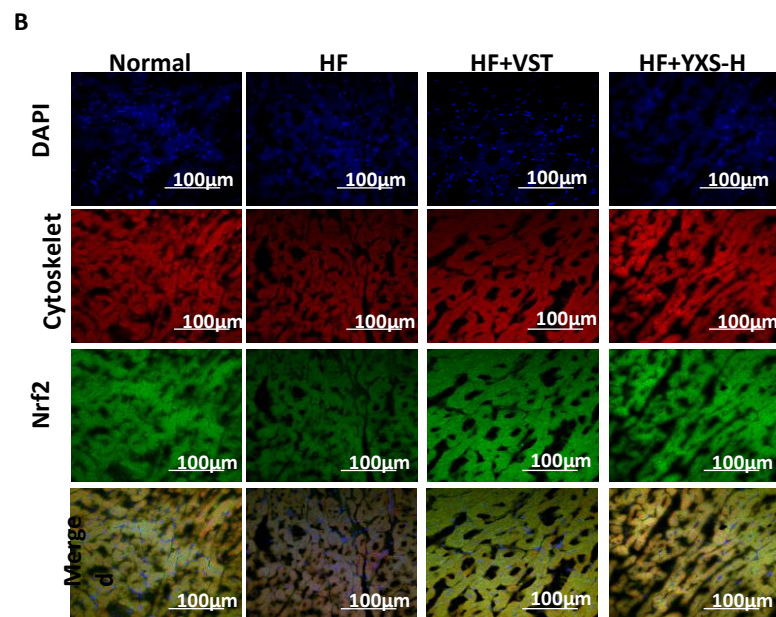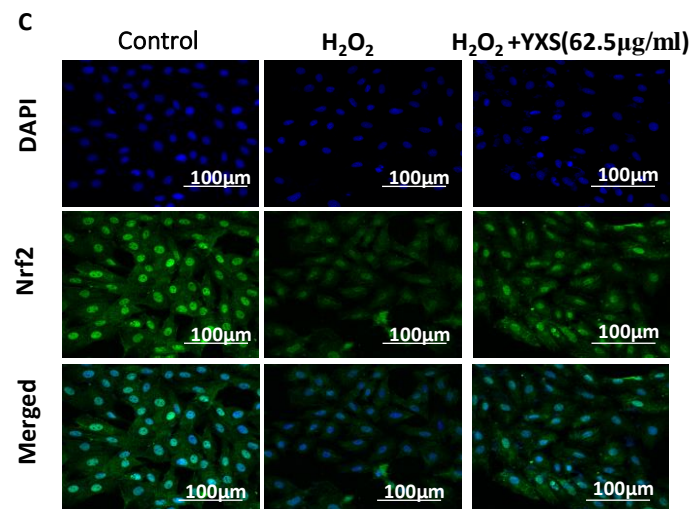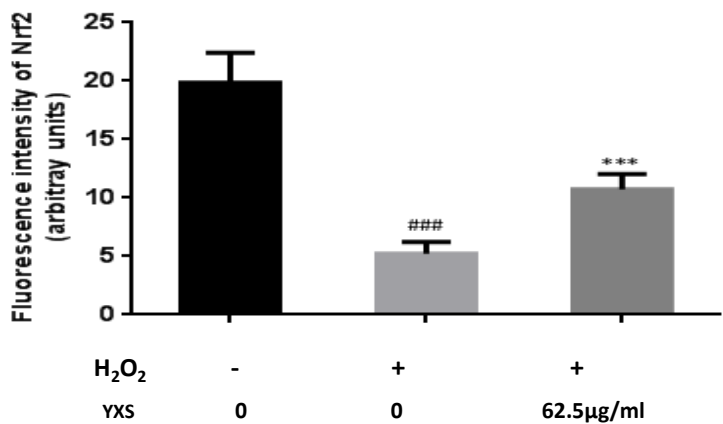

Supplement: Supplementary Materials — Antibodies such as Nrf2 (ab89443), Tlr4 (19811-1-AP), and Myd88 (sc-74532) were used for IF staining. As for F-actin staining, the samples were incubated with 0.1% Triton X-100 for 15 min. Rhodamine phalloidin (PHDR1, cytoskeleton) was used for the staining of F-actin after the treatment of 0.1% Triton X-100 for 15 min and then followed by 4,6-DAPI for 10 min before observation. Table S1: the RNA-seq data of failing heart treated with or without YXS or VST. Figure S1: the enrichment of DEs in YXS-mediated protection against H2O2-induced damage; (A) enriched GO terms of upregulated DEs; (B) enriched GO terms of downregulated DEs. Figure S2: YXS decreased the levels of Tlr4 and Myd88, enhanced Nrf2 expression, and improved cytoskeleton arrangement; (A) the IF staining of Tlr4 (red) and Myd88 (green) in H2O2-induced H9C2 cell and the related quantification, nucleus (blue), scale bar: 100 μm (n = 3–5); (B) the IF staining of Nrf2 (green) and F-actin (red) in heart tissue, nucleus (blue), scale bar: 100 μm; (C) the IF staining of Nrf2 (green) in H2O2-induced H9C2 cell and the related quantification, nucleus (blue), scale bar: 100 μm (n = 3–5). [file 8049079.f1.zip › Xiang.Figure. S2 (1).pdf]
