# Supplementary material for: Yixin-Shu Capsules Ameliorated Ischemia-Induced Heart Failure by Restoring Trx2 and Inhibiting JNK/p38 Activation
Source: Oxid Med Cell Longev. 2021 Feb 16;2021:8049079. doi: 10.1155/2021/8049079 (PMC7902134; doi:10.1155/2021/8049079)
Supplement: Supplementary Materials — Antibodies such as Nrf2 (ab89443), Tlr4 (19811-1-AP), and Myd88 (sc-74532) were used for IF staining. As for F-actin staining, the samples were incubated with 0.1% Triton X-100 for 15 min. Rhodamine phalloidin (PHDR1, cytoskeleton) was used for the staining of F-actin after the treatment of 0.1% Triton X-100 for 15 min and then followed by 4,6-DAPI for 10 min before observation. Table S1: the RNA-seq data of failing heart treated with or without YXS or VST. Figure S1: the enrichment of DEs in YXS-mediated protection against H2O2-induced damage; (A) enriched GO terms of upregulated DEs; (B) enriched GO terms of downregulated DEs. Figure S2: YXS decreased the levels of Tlr4 and Myd88, enhanced Nrf2 expression, and improved cytoskeleton arrangement; (A) the IF staining of Tlr4 (red) and Myd88 (green) in H2O2-induced H9C2 cell and the related quantification, nucleus (blue), scale bar: 100 μm (n = 3–5); (B) the IF staining of Nrf2 (green) and F-actin (red) in heart tissue, nucleus (blue), scale bar: 100 μm; (C) the IF staining of Nrf2 (green) in H2O2-induced H9C2 cell and the related quantification, nucleus (blue), scale bar: 100 μm (n = 3–5). [file Xiang.Figure. S1 (1).pdf › Xiang.Figure. S1 (1).pdf]

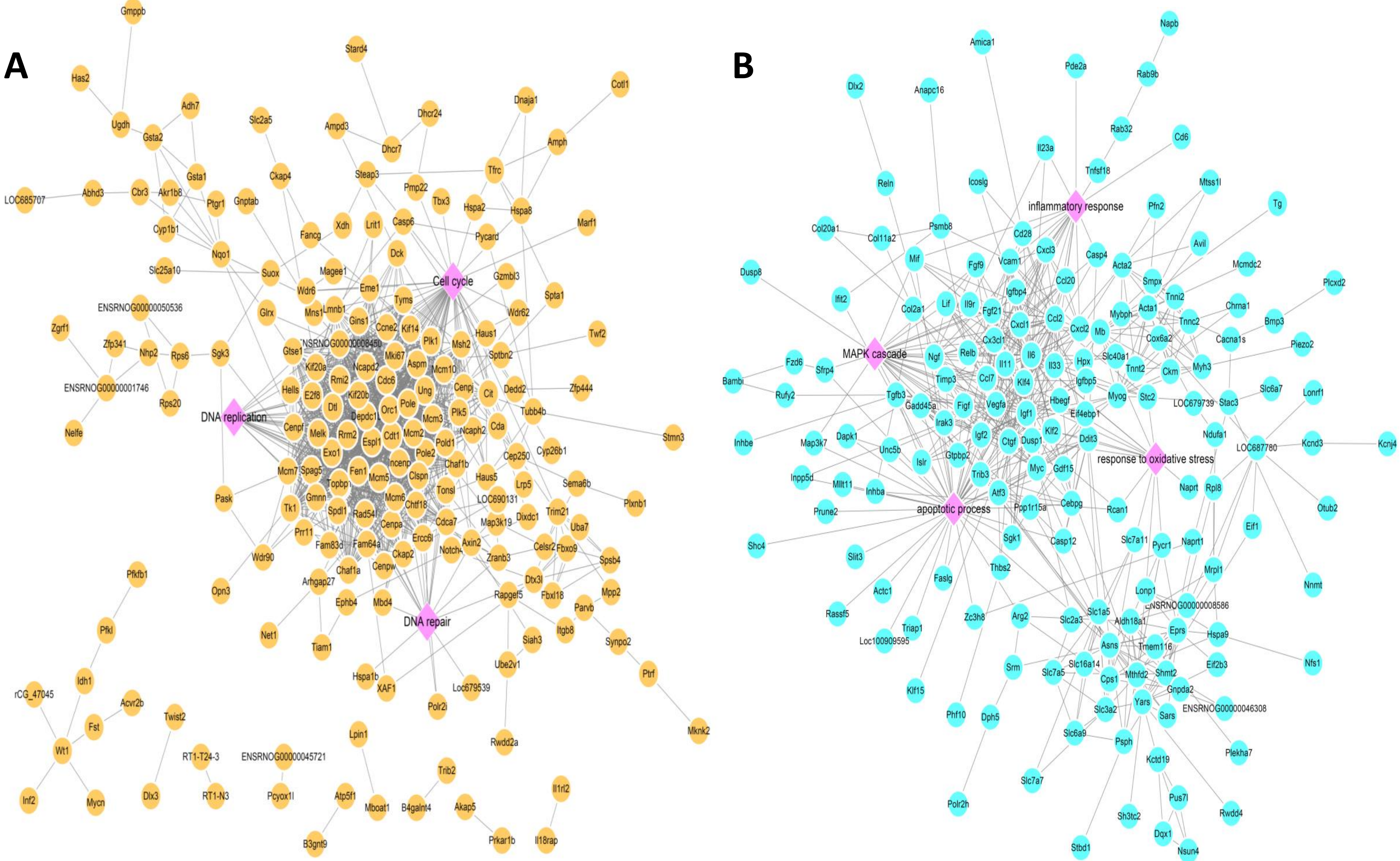
A
B
DNA replication
Cell cycle
DNA repair
Gmppb
Has2
Ugdh
Adh7
Gsta2
Gsta1
Stard4
Slc2a5
Ampd3
Dhcr24
Dhcr7
Dnaja1
Cotl1
Amph
Steap3
Tfrc
Ckap4
Pmp22
Tbx3
Hspa2
Hspa8
LOC685707
Abhd3
Cbr3
Akr1b8
Ptgr1
Gnptab
Cyp1b1
Nqo1
Xdh
Lrit1
Casp6
Fancg
Pycard
Marf1
Slc25a10
Suox
Magee1
Dck
Gzmbl3
ENSRNOG00000050536
Wdr6
Eme1
Spta1
Zgrf1
Zfp341
Nhp2
Rps6
Glrx
Mns1
Lmnb1
Tyms
Wdr62
Twf2
Sgk3
Gins1
Ccne2
Kif14
Plk1
Msh2
Haus1
ENSRNOG00000001746
Rps20
Gtse1
Mki67
Sptbn2
Nelfe
Kif20a
Ncapd2
Aspm
Mcm10
Zfp444
Hells
Rmi2
Cdc6
Cenpj
Dedd2
E2f8
Kif20b
Ung
Cit
Tubb4b
Dtl
Orc1
Pole
Plk5
Stmn3
Depdc1
Mcm3
Cenpf
Melk
Rrm2
Espl1
Cdt1
Mcm2
Pold1
Ncaph2
Cda
Cep250
Cyp26b1
Exo1
Pole2
Incenp
Chaf1b
Mcm7
Spag5
Fen1
Mcm5
Clspn
Haus5
Sema6b
Plxnb1
Pask
Topbp1
Tonsl
Lrp5
Gmnn
Mcm6
LOC690131
Tk1
Spdl1
Rad54l
Chtf18
Trim21
Dixdc1
Prr11
Cenpa
Cdca7
Map3k19
Uba7
Wdr90
Fam83d
Fam64a
Ercc6l
Celsr2
Fbxo9
Axin2
Notch4
Spsb4
Chaf1a
Ckap2
Cenpw
Zranb3
Dtx3l
Opn3
Arhgap27
Rapgef5
Fbxl18
Mpp2
Ephb4
Mbd4
Parvb
Net1
Tiam1
Siah3
Itgb8
Synpo2
Ptrf
Hspa1b
XAF1
Ube2v1
Polr2i
Loc679539
Rwdd2a
Mknk2
Pfkfb1
Pfkl
Idh1
rCG_47045
Acvr2b
Fst
Wt1
Twist2
RT1-T24-3
ENSRNOG00000045721
Lpin1
Trib2
Il1rl2
Inf2
Mycn
Dlx3
RT1-N3
Pcyox1l
Atp5f1
Mboat1
B4galnt4
Akap5
Prkar1b
Il18rap
B3gnt9
MAPK cascade
inflammatory response
apoptotic process
response to oxidative stress
Napb
Amica1
Pde2a
Rab9b
Dlx2
Anapc16
Rab32
Cd6
Il23a
Tnfsf18
Mtss1l
Reln
Icoslg
Tg
Col20a1
Col11a2
Psmb8
Pfn2
Cd28
Avil
Cxcl3
Casp4
Mif
Fgf9
Vcam1
Acta2
Mcmdc2
Dusp8
Ccl20
Smpx
Plcxd2
Igfbp4
Tnni2
Chrna1
Ifit2
Col2a1
Lif
Il9r
Fgf21
Cxcl1
Ccl2
Mybph
Acta1
Bmp3
Tnnc2
Cxcl2
Mb
Cox6a2
Cacna1s
Piezo2
Fzd6
Ngf
Relb
Cx3cl1
Il11
Il6
Hpx
Slc40a1
Bambi
Sfrp4
Il33
Ckm
Myh3
Timp3
Ccl7
Klf4
Igfbp5
Tnnt2
Slc6a7
Rufy2
Tgfb3
Gadd45a
Figf
Vegfa
Hbegf
Myog
Stc2
LOC679739
Stac3
Lonrf1
Eif4ebp1
Igf1
Irak3
Inhbe
Map3k7
Dapk1
Igf2
Ctgf
Dusp1
Klf2
Ddit3
Ndufa1
LOC687780
Kcnd3
Kcnj4
Unc5b
Gtpbp2
Islr
Myc
Gdf15
Inpp5d
Mllt11
Trib3
Naprt
Rpl8
Otub2
Inhba
Atf3
Prune2
Ppp1r15a
Cebpg
Rcan1
Shc4
Sgk1
Casp12
Eif1
Slc7a11
Pycr1
Naprt1
Slit3
Thbs2
Faslg
Mrpl1
Nnmt
Actc1
Lonp1
ENSRNOG00000008586
Rassf5
Zc3h8
Arg2
Slc2a3
Slc1a5
Aldh18a1
Eprs
Hspa9
Triap1
Loc100909595
Asns
Tmem116
Slc16a14
Srm
Nfs1
Slc7a5
Mthfd2
Shmt2
Eif2b3
Cps1
Klf15
Gnpda2
Yars
Phf10
Dph5
Slc3a2
ENSRNOG00000046308
Sars
Slc6a9
Psph
Plekha7
Kctd19
Slc7a7
Polr2h
Pus7l
Sh3tc2
Rwdd4
Dqx1
Stbd1
Nsun4
