# Supplementary material for: Yixin-Shu Capsules Ameliorated Ischemia-Induced Heart Failure by Restoring Trx2 and Inhibiting JNK/p38 Activation
Source: Oxid Med Cell Longev. 2021 Feb 16;2021:8049079. doi: 10.1155/2021/8049079 (PMC7902134; doi:10.1155/2021/8049079)

| Symbol | FPKM | | | | | | | |
|---|---|---|---|---|---|---|---|---|
| | Sham 1 | Sham 2 | Sham 3 | Vehicle 1 | Vehicle 2 | Vehicle 3 | VST 1 | VST 2 |
| Gprc5c | 1.746719 | 1.310831 | 1.473383 | 1.625957 | 1.40689 | 1.797197 | 1.455644 | 1.406022 |
| Mrpl52 | 28.09003 | 29.85326 | 27.65144 | 31.38314 | 29.06331 | 30.84572 | 27.45999 | 26.80667 |
| Pkp2 | 27.60272 | 27.072 | 25.78143 | 27.24 | 27.65673 | 28.69897 | 24.82276 | 27.19707 |
| Ggta1 | 14.5018 | 14.08392 | 13.85705 | 14.23863 | 13.5589 | 11.34421 | 16.70418 | 15.16353 |
| Fgfbp3 | 2.828125 | 3.440742 | 4.3747 | 3.054351 | 2.104437 | 3.119625 | 3.436151 | 2.776246 |
| AABR0703 | 0 | 0 | 0 | 0 | 0 | 0 | 0.157486 | 0 |
| Rps4x | 370.0608 | 351.6795 | 350.3908 | 347.7911 | 330.7484 | 324.7436 | 348.6417 | 349.272 |
| Plekhg3 | 1.928225 | 2.062694 | 2.283353 | 2.918232 | 2.729405 | 2.930494 | 3.987575 | 3.563177 |
| Acr | 0.072279 | 0.69513 | 0.698362 | 0.499515 | 0.312616 | 0.443618 | 0.202156 | 0.640642 |
| Tm7sf2 | 1.912174 | 1.694335 | 1.607647 | 1.761993 | 1.295368 | 1.292797 | 2.010384 | 2.041994 |
| Dok1 | 1.87887 | 2.400156 | 1.724284 | 2.150169 | 2.752483 | 2.311667 | 2.949575 | 2.444283 |
| Sccpdh | 0.056368 | 0.095667 | 0 | 0.103882 | 0.06095 | 0.049424 | 0.031531 | 0.449656 |
| NEWGENE_1 | 0 | 0 | 0 | 0 | 0 | 0 | 0 | 0.067267 |
| Dtl | 0.081618 | 0.147755 | 0.087622 | 0.225623 | 0.158854 | 0.271937 | 0.127834 | 0.245962 |
| Pde6c | 0.080999 | 0.114558 | 0.065218 | 0.093297 | 0.021896 | 0.088775 | 0.135927 | 0.053845 |
| Fah | 1.48098 | 1.331934 | 1.549159 | 2.554372 | 2.504694 | 2.297389 | 2.421556 | 2.558015 |
| Acaa2 | 574.6824 | 589.4995 | 605.9869 | 570.6861 | 546.7863 | 561.177 | 580.5056 | 564.0488 |
| Adamts10 | 13.14413 | 12.94595 | 15.71245 | 22.97845 | 22.12528 | 21.41855 | 25.95473 | 25.83596 |
| Prodh1 | 22.11113 | 22.03745 | 22.10331 | 20.08506 | 17.34995 | 20.82784 | 22.19567 | 21.29028 |
| Cwc25 | 2.848287 | 2.981926 | 2.759066 | 3.00168 | 3.062111 | 3.114549 | 3.09497 | 3.43865 |
| Dsg1 | 0 | 0 | 0.018646 | 0.032009 | 0.01878 | 0.030457 | 0.019431 | 0.015395 |
| Pus1 | 7.543745 | 8.103642 | 8.413817 | 7.275837 | 7.871291 | 8.518887 | 8.078379 | 9.210056 |
| LOC69108 | 3.595272 | 3.802585 | 3.356308 | 2.232608 | 2.324058 | 2.809692 | 1.573916 | 2.182175 |
| Prl4a1 | 0 | 0 | 0 | 0 | 0 | 0 | 0 | 0 |
| Iffo1 | 13.15771 | 13.46354 | 14.50485 | 18.04407 | 14.10789 | 14.71122 | 18.74611 | 17.4348 |
| Apip | 7.35112 | 6.800736 | 6.592055 | 6.773768 | 7.247308 | 6.597127 | 6.289023 | 7.167229 |
| Dnal1 | 2.980174 | 3.413814 | 2.94107 | 3.053132 | 3.42257 | 3.083688 | 3.479047 | 4.036045 |
| Wee1 | 12.00819 | 12.46503 | 13.73268 | 12.92153 | 12.1371 | 12.68782 | 18.19255 | 19.16105 |
| Cdh19 | 0.477465 | 0.621263 | 0.717624 | 1.055915 | 1.071272 | 1.025671 | 1.014916 | 0.973361 |
| Ly6d | 0 | 0 | 0 | 0 | 0 | 0 | 0 | 0 |
| Cct7 | 41.20183 | 42.20877 | 44.11972 | 42.95422 | 42.59216 | 46.2654 | 46.49539 | 43.91269 |
| Rasl2-9 | 0.222078 | 0 | 0 | 0 | 0 | 0 | 0.04969 | 0.078735 |
| Gclm | 16.28066 | 15.70951 | 15.17058 | 16.07355 | 15.54642 | 13.22436 | 17.07077 | 15.77344 |
| RGD13118 | 24.92381 | 25.09936 | 24.68987 | 28.2216 | 25.30794 | 27.43829 | 31.32249 | 29.34553 |
| Eya3 | 12.28143 | 13.48194 | 12.10018 | 10.2574 | 10.19055 | 9.928282 | 11.34274 | 11.76576 |
| Dok2 | 3.970787 | 3.480306 | 3.622459 | 3.418431 | 3.769459 | 3.9883 | 4.296348 | 4.444822 |
| Rps6ka5 | 3.917236 | 4.215613 | 3.64377 | 3.609579 | 3.918322 | 4.340843 | 4.453805 | 4.376808 |
| Sin3b | 42.96569 | 42.43623 | 43.22122 | 47.2032 | 46.25284 | 45.24845 | 46.10289 | 49.78094 |
| Traf3ip3 | 0.636126 | 1.007646 | 1.256577 | 0.890971 | 0.907941 | 1.003961 | 1.053271 | 0.902129 |
| Fam227b | 1.122127 | 1.334743 | 1.112008 | 1.219585 | 1.337785 | 1.513664 | 1.384143 | 1.453641 |
| Eda | 0.966755 | 1.184992 | 0.951384 | 1.262005 | 1.161487 | 1.012473 | 1.412041 | 1.285325 |
| Eef1akmt1 | 2.041464 | 2.739554 | 3.261979 | 3.71851 | 3.952796 | 3.288526 | 2.868152 | 2.735216 |
| Mto1 | 7.869412 | 7.320296 | 7.030814 | 6.253684 | 8.023664 | 7.849224 | 7.740385 | 7.255926 |
| LOC100361 | 1.840989 | 1.08007 | 1.903219 | 1.193762 | 1.253363 | 1.853319 | 1.06795 | 2.115245 |
| Stx2 | 10.7971 | 10.63546 | 10.05722 | 11.6463 | 12.46451 | 12.47404 | 14.77939 | 14.77721 |
| LOC103692 | 0.086842 | 0.196516 | 0 | 0 | 0.093901 | 0 | 0.097155 | 0.076973 |
| Fbxo10 | 8.281793 | 7.340495 | 7.29168 | 7.69039 | 8.036967 | 7.261483 | 7.329804 | 7.11339 |
| Afap1l1 | 19.94965 | 20.36489 | 19.37203 | 14.99836 | 13.77661 | 14.29817 | 15.03832 | 13.33269 |
| Psph | 3.107896 | 2.842053 | 3.747877 | 2.942264 | 3.314489 | 2.836993 | 2.810159 | 3.320713 |
| Cpt2 | 81.06794 | 82.52003 | 78.22777 | 70.98295 | 70.77224 | 73.41346 | 62.92789 | 63.07812 |
| Wdr82 | 36.24865 | 34.88912 | 37.34819 | 34.57861 | 28.80448 | 35.03579 | 42.45857 | 40.91593 |

| | | | | | | | | |
|---|---|---|---|---|---|---|---|---|
| Akap8l | 15.85894 | 15.66368 | 15.8175 | 16.83193 | 18.87417 | 17.98948 | 19.58678 | 19.11322 |
| Trnau1ap | 19.7963 | 19.09212 | 18.49481 | 17.37287 | 18.11818 | 17.62604 | 16.34676 | 16.41846 |
| AABR07007 | 2.834072 | 3.352375 | 4.632985 | 3.026949 | 2.855502 | 2.371966 | 2.377975 | 4.281782 |
| Sfn | 0.981423 | 0.872484 | 1.279396 | 1.42111 | 1.364398 | 1.167838 | 1.568532 | 1.304826 |
| Rn50_13_( | 0 | 0 | 0.038825 | 0 | 0.039104 | 0 | 0.080918 | 0 |
| Lims2 | 90.22565 | 95.42334 | 99.42257 | 93.85936 | 86.71972 | 86.26282 | 94.5453 | 94.35667 |
| Cask | 1.60566 | 1.526052 | 1.42499 | 1.49928 | 1.736177 | 1.370301 | 1.987951 | 2.087325 |
| Lrrc2 | 51.79115 | 51.56176 | 50.42225 | 59.41321 | 60.83979 | 61.21331 | 66.57662 | 67.57488 |
| Fam193a | 2.337372 | 2.483218 | 2.568223 | 2.002121 | 2.78841 | 2.357303 | 2.455351 | 2.820664 |
| Dph7 | 4.94567 | 3.661953 | 4.411564 | 5.663042 | 6.134106 | 5.101609 | 4.678637 | 5.253866 |
| Atg5 | 10.78122 | 9.4281 | 8.564154 | 10.51671 | 11.27326 | 10.49177 | 10.38265 | 11.16609 |
| Xpo1 | 10.25564 | 9.764482 | 9.637032 | 11.0252 | 11.2056 | 11.14063 | 13.21197 | 12.76638 |
| Sema4a | 2.56163 | 2.494349 | 2.616736 | 2.861141 | 2.853788 | 3.008331 | 3.439005 | 3.123657 |
| Agr2 | 0.738198 | 0.887439 | 0.594377 | 0.042514 | 0.049888 | 0.161813 | 0 | 0.163575 |
| RGD130462 | 1.948 | 1.752843 | 2.486509 | 2.31796 | 2.305369 | 2.313208 | 2.230816 | 2.637498 |
| Tns4 | 0 | 0 | 0 | 0.020853 | 0.02447 | 0.019842 | 0.050635 | 0.020058 |
| Fasn | 1.850771 | 1.789351 | 1.962315 | 2.587158 | 2.366758 | 2.863689 | 2.782115 | 2.950742 |
| Ncl | 59.3832 | 57.68917 | 56.52299 | 61.2228 | 59.67074 | 59.19803 | 65.36887 | 64.98392 |
| Rimbp2 | 10.44004 | 11.07414 | 11.44155 | 9.725068 | 10.16876 | 10.35255 | 10.27781 | 10.2817 |
| Ly6g6d | 0.165453 | 0.280804 | 0.444061 | 0.762293 | 0.715608 | 0.942952 | 0.462754 | 0.293299 |
| Gucy2g | 0.015209 | 0.017209 | 0.065313 | 0 | 0 | 0 | 0 | 0 |
| RGD156141 | 0.162375 | 0.09186 | 0.2179 | 0.074811 | 0.043893 | 0.14237 | 0 | 0.107941 |
| Klhl2 | 4.881716 | 4.2503 | 4.664714 | 4.466406 | 4.342619 | 4.583859 | 5.07411 | 5.124777 |
| AABR07051 | 0.300954 | 0 | 0 | 0 | 0 | 0.131938 | 0 | 0.26675 |
| Rn50_X_07 | 0.831205 | 0.769475 | 0.56786 | 0.696293 | 0.612798 | 0.695674 | 0.76084 | 1.004643 |
| Hps3 | 1.950439 | 1.537171 | 1.530728 | 2.082334 | 2.152615 | 2.571117 | 2.513136 | 2.527582 |
| RGD130860 | 9.409584 | 8.828223 | 8.399241 | 7.784021 | 8.15099 | 8.519163 | 9.935619 | 8.921183 |
| AABR07064 | 11.96622 | 12.54551 | 18.50367 | 9.205525 | 8.190678 | 11.06953 | 13.75573 | 10.99547 |
| AABR07049 | 0 | 0.192682 | 0 | 0 | 0 | 0 | 0.09526 | 0 |
| Ramp1 | 4.860698 | 3.666434 | 5.21827 | 4.538661 | 4.975493 | 3.523141 | 3.045243 | 5.629484 |
| Cdip1 | 27.81749 | 28.99301 | 30.6268 | 28.66375 | 29.28826 | 27.96885 | 30.48493 | 30.03716 |
| Ampd1 | 0.929317 | 1.100384 | 0.510441 | 1.174963 | 0.677694 | 0.985371 | 0.797893 | 1.091881 |
| Rn50_8_05 | 1.014843 | 1.607547 | 1.525299 | 1.496221 | 0.219467 | 0.711852 | 0.908289 | 0.539704 |
| Camk2n1 | 24.71057 | 23.61291 | 24.74149 | 25.36534 | 25.05788 | 24.9218 | 40.67982 | 34.38533 |
| Pnmal2 | 0.16269 | 0.345143 | 0.109161 | 0.074956 | 0.131936 | 0.035662 | 0.113757 | 0.180251 |
| RGD156039 | 0.078746 | 0 | 0 | 0.072561 | 0.085147 | 0.138089 | 0 | 0 |
| Trappc5 | 27.96915 | 28.98399 | 30.91531 | 24.52801 | 24.16585 | 25.82212 | 26.0268 | 27.22316 |
| Mypn | 19.55404 | 20.52595 | 19.27794 | 16.49946 | 18.055 | 17.04626 | 21.55549 | 23.74717 |
| Mrps23 | 50.54485 | 52.93084 | 50.04894 | 46.35135 | 51.37158 | 49.49823 | 46.36066 | 48.88948 |
| Myo15a | 0.029923 | 0.045143 | 0.021416 | 0.073529 | 0.032356 | 0.087456 | 0.050215 | 0.048625 |
| LOC100359 | 30.13712 | 34.58241 | 29.60058 | 25.2098 | 30.50682 | 24.55019 | 29.65101 | 32.20599 |
| Hao1 | 0 | 0 | 0 | 0 | 0 | 0 | 0 | 0 |
| Thap6 | 4.999761 | 4.987087 | 5.367563 | 5.940713 | 6.046374 | 5.479744 | 8.16947 | 6.763925 |
| Dsp | 96.17661 | 98.14237 | 96.17672 | 77.32506 | 76.93488 | 76.7785 | 94.4752 | 99.49018 |
| Rab12 | 62.51271 | 70.24848 | 73.96726 | 57.01454 | 57.8501 | 58.85515 | 65.49099 | 68.86707 |
| Abcd4 | 1.805721 | 2.244875 | 2.584743 | 2.218535 | 2.121235 | 2.052372 | 2.743428 | 2.469908 |
| Ddx18 | 6.31414 | 5.351219 | 5.679395 | 6.694336 | 5.98385 | 6.30576 | 6.382128 | 5.985485 |
| Mn1 | 7.998677 | 8.747447 | 9.362514 | 8.405782 | 7.433965 | 8.995252 | 13.33306 | 12.2705 |
| Tnpo2 | 7.54932 | 7.346109 | 7.110598 | 7.458307 | 7.868491 | 7.927818 | 8.738341 | 9.385253 |
| Def8 | 7.026737 | 6.379267 | 6.932975 | 7.276511 | 7.199923 | 7.085207 | 6.644587 | 6.717501 |
| Aqr | 3.732912 | 3.688227 | 3.793033 | 3.846686 | 3.820314 | 3.512735 | 4.587956 | 4.380487 |
| AABR07054 | 0.200636 | 0.45402 | 0.646186 | 1.109267 | 0 | 1.055505 | 0 | 0.711334 |
| Nup88 | 10.52963 | 10.85751 | 10.23208 | 12.92352 | 12.13675 | 11.45959 | 11.82866 | 11.2765 |
| RGD131012 | 3.322867 | 3.574005 | 3.611351 | 4.158124 | 4.213975 | 4.280316 | 5.530319 | 4.76326 |

| | | | | | | | | |
|---|---|---|---|---|---|---|---|---|
| AABR07051 | 0 | 0 | 0.153602 | 0.131839 | 0 | 0 | 0 | 0.253631 |
| Uhrf2 | 9.735347 | 9.789348 | 9.225625 | 9.982477 | 8.9279 | 9.5115 | 10.38375 | 10.92564 |
| Tmem192 | 3.997928 | 4.49732 | 4.837838 | 4.216293 | 4.172974 | 3.525649 | 3.050746 | 3.789358 |
| Sh2d3c | 24.77667 | 24.97694 | 31.42655 | 21.68833 | 20.6282 | 21.1888 | 31.15545 | 31.4241 |
| AABR07050 | 0.054378 | 0.215341 | 0.175134 | 0.075161 | 0.117596 | 0.119196 | 0.060836 | 0.289187 |
| Cct3 | 106.4324 | 110.3502 | 100.9457 | 127.0159 | 124.0612 | 119.8513 | 121.9814 | 115.5708 |
| Adgre5 | 10.07231 | 9.986098 | 9.710245 | 10.78673 | 10.12612 | 10.61837 | 12.64404 | 13.3466 |
| Erg | 16.01156 | 16.06125 | 17.84942 | 13.28635 | 12.56935 | 14.43097 | 15.66218 | 15.99988 |
| Zbtb7a | 15.56516 | 15.97499 | 18.87557 | 15.41225 | 14.13505 | 13.2805 | 19.64885 | 18.48485 |
| LOC100909 | 0.418091 | 0.354788 | 0.149616 | 0.064209 | 0.113019 | 0.274937 | 0.155914 | 0.617625 |
| Rn50_X_07 | 0.053654 | 0 | 0 | 0 | 0 | 0 | 0 | 0.047556 |
| Disp2 | 0.071986 | 0.081449 | 0.068695 | 0.125295 | 0.129729 | 0.1613 | 0.178966 | 0.141789 |
| Hoxb7 | 0.278542 | 0.495247 | 0.256314 | 0.696664 | 0.172105 | 0.453563 | 0.712276 | 0.493771 |
| RGD155987 | 0 | 0.133445 | 0 | 0.326035 | 0 | 0.103411 | 0.395842 | 0.104537 |
| AABR07065 | 0 | 0 | 0 | 0 | 0 | 0 | 0 | 0 |
| Unc119 | 36.61523 | 39.34542 | 39.61624 | 39.09258 | 36.40651 | 37.77184 | 40.46003 | 40.86653 |
| Plvap | 3.535252 | 4.035685 | 4.269734 | 4.130166 | 5.426751 | 4.372808 | 5.967938 | 4.64425 |
| Ankrd10 | 26.85432 | 24.97218 | 29.41543 | 25.13184 | 24.68161 | 25.53257 | 27.8371 | 26.74042 |
| Zfhx3 | 1.41936 | 1.26029 | 1.36736 | 1.264577 | 1.255229 | 1.578285 | 1.866591 | 2.15368 |
| AABR07018 | 0.698212 | 0.315998 | 0 | 0.25735 | 0.301987 | 0.122439 | 0.624903 | 0.123772 |
| Cd79a | 0.310163 | 0.467914 | 0.49947 | 0.428705 | 0.558959 | 0.181301 | 0.693994 | 0.458189 |
| Npepps | 15.43403 | 15.00739 | 15.4319 | 15.42171 | 15.23921 | 13.70047 | 16.80989 | 17.37999 |
| AABR07072 | 0 | 0 | 0 | 0 | 0.133859 | 0 | 0.138498 | 0 |
| Ifi44 | 11.76661 | 10.69643 | 9.160764 | 15.2912 | 16.75366 | 14.84542 | 19.94692 | 21.45577 |
| Ovol1 | 0.349365 | 0.40993 | 0.305609 | 0.166925 | 0.293816 | 0.521884 | 0.434282 | 0.286722 |
| AC130970. | 0.241955 | 0.508412 | 0.296862 | 0.509604 | 0.523244 | 0.363679 | 0.657386 | 0.45955 |
| Rpl391 | 0.62965 | 0.593683 | 0 | 0.0967 | 0 | 0.368052 | 0.587021 | 0.279045 |
| Cercam | 2.417209 | 2.404877 | 3.087189 | 5.414797 | 5.362578 | 5.188903 | 6.550852 | 6.316659 |
| Clec2d2 | 0.204076 | 0.138541 | 0.233695 | 0.162975 | 0.191243 | 0.167005 | 0.273973 | 0.120589 |
| Slc6a19 | 0 | 0.045635 | 0.028867 | 0.024777 | 0.014537 | 0 | 0.045123 | 0 |
| Dynll2 | 152.1835 | 159.4357 | 163.0157 | 130.5722 | 130.283 | 130.1932 | 153.7879 | 154.8483 |
| AABR07005 | 1.484704 | 1.225853 | 1.335451 | 1.183219 | 1.735556 | 1.231423 | 1.167204 | 1.1737 |
| Slc9a3r1 | 7.124854 | 7.227495 | 7.799705 | 6.532931 | 6.793183 | 6.339399 | 6.557399 | 7.590578 |
| Pld5 | 0.267583 | 0.090827 | 0.287267 | 0.197253 | 0.3472 | 0.375387 | 0.568783 | 0.901253 |
| Napa | 42.38335 | 43.71439 | 46.6351 | 53.08787 | 47.52312 | 48.4238 | 47.49284 | 46.6863 |
| RGD130813 | 12.41563 | 12.04628 | 12.28988 | 11.07147 | 9.996454 | 11.17867 | 13.89005 | 12.51328 |
| LOC679739 | 16.25801 | 9.197588 | 14.30823 | 18.2038 | 7.972123 | 10.85704 | 9.728861 | 18.43176 |
| Tmem14c | 98.79344 | 90.34033 | 96.2084 | 97.79965 | 90.77114 | 93.29634 | 81.47496 | 84.98163 |
| Nkx2-2 | 0 | 0.042534 | 0.040358 | 0.03464 | 0.040648 | 0 | 0 | 0.03332 |
| LOC689986 | 0.025544 | 0.057804 | 0 | 0.070614 | 0.110483 | 0.111987 | 0.114312 | 0 |
| Mmp16 | 0.059439 | 0.050439 | 0.047859 | 0.191698 | 0.241015 | 0.247553 | 0.365739 | 0.26342 |
| LOC498460 | 0 | 0 | 0 | 0 | 0 | 0 | 0 | 0 |
| Nags | 0.074419 | 0 | 0.053262 | 0.091432 | 0 | 0.02175 | 0 | 0.065961 |
| Plet1 | 0 | 0 | 0.037108 | 0.063701 | 0.037375 | 0 | 0.077339 | 0.09191 |
| Ppip5k2 | 59.09504 | 60.80118 | 53.75305 | 64.97423 | 62.38132 | 63.69866 | 72.67781 | 72.36368 |
| Tmem184c | 7.497374 | 7.802198 | 7.477537 | 7.761441 | 7.881614 | 7.527297 | 8.879602 | 8.142538 |
| Krt72 | 0.12986 | 0.367325 | 0.034853 | 0.508558 | 0.772285 | 0.341583 | 0.726406 | 0.748157 |
| Ighg | 0 | 0.120794 | 0 | 0.245939 | 0.173158 | 0.280823 | 0.059719 | 0.047313 |
| Zfp94 | 4.20661 | 4.038433 | 4.516065 | 6.107992 | 6.23013 | 6.102558 | 6.759769 | 7.118098 |
| Gabrb3 | 0.045417 | 0 | 0 | 0 | 0.049109 | 0 | 0 | 0 |
| Lpgat1 | 10.31724 | 10.19263 | 8.759331 | 8.531863 | 7.663084 | 7.434704 | 9.424016 | 10.05792 |
| Commd1 | 38.11639 | 38.68443 | 40.02051 | 41.38306 | 38.11406 | 40.65385 | 40.66872 | 37.18636 |
| Arhgap20 | 0.756117 | 0.767766 | 0.947029 | 0.937908 | 0.597461 | 0.603467 | 0.791685 | 0.936539 |
| AABR07014 | 8.497978 | 6.366731 | 10.35598 | 5.290914 | 4.470198 | 6.444138 | 8.99326 | 6.819677 |

| | | | | | | | | |
|---|---|---|---|---|---|---|---|---|
| Foxred2 | 2.071544 | 2.010848 | 1.810745 | 1.637644 | 1.481047 | 1.647602 | 2.000946 | 2.096981 |
| RGD130555 | 2.810773 | 2.661737 | 2.427155 | 1.91436 | 1.783906 | 2.035882 | 2.221706 | 2.626726 |
| Zdhhc6 | 6.666709 | 6.448106 | 5.617968 | 7.893566 | 9.146415 | 8.013822 | 8.300497 | 8.259942 |
| Mansc1 | 2.295744 | 1.636442 | 2.144226 | 2.073129 | 2.507178 | 2.636913 | 2.722479 | 2.502843 |
| Nav1 | 6.871823 | 6.467732 | 7.527708 | 7.542073 | 6.143098 | 6.762063 | 10.02854 | 10.27296 |
| Stx3 | 1.168809 | 1.024435 | 1.042713 | 1.486579 | 1.441818 | 1.602172 | 1.694369 | 1.692576 |
| Iqub | 3.729324 | 3.37955 | 3.54028 | 3.627338 | 3.211036 | 2.921693 | 4.500569 | 3.963521 |
| Thoc2 | 4.535547 | 3.857832 | 4.196406 | 4.981916 | 4.433325 | 4.582135 | 5.097935 | 5.168676 |
| Esx1 | 0 | 0.071818 | 0 | 0 | 0 | 0 | 0 | 0.05626 |
| Lpcat2 | 0.524772 | 0.55777 | 0.734097 | 0.923154 | 0.653403 | 1.059672 | 1.120812 | 0.859789 |
| Tle6 | 1.362195 | 1.363422 | 1.040556 | 1.134516 | 1.274644 | 1.033593 | 1.201586 | 0.975192 |
| Ric3 | 0.132673 | 0.060045 | 0.04273 | 0.220055 | 0.172149 | 0.151226 | 0.178114 | 0.21167 |
| Jkamp | 14.72004 | 14.45608 | 14.10284 | 16.2502 | 13.89295 | 15.21016 | 13.89121 | 14.86541 |
| RGD156114 | 1.894087 | 2.177451 | 1.750701 | 1.717326 | 1.379968 | 1.616332 | 2.685603 | 2.406014 |
| AABR07017 | 0 | 0 | 0 | 0 | 0 | 0 | 0 | 0 |
| Vps39 | 11.77843 | 11.47731 | 11.3675 | 13.14585 | 12.74884 | 13.00401 | 11.21405 | 11.83533 |
| Tmem229a | 0.232221 | 0.167203 | 0.22664 | 0.175077 | 0.262511 | 0.277653 | 0.188945 | 0.168406 |
| Snx2 | 19.92685 | 17.47775 | 17.35743 | 20.09361 | 19.8485 | 19.48092 | 19.90266 | 21.15352 |
| Casp7 | 9.714945 | 8.798477 | 9.180835 | 6.74869 | 7.104017 | 6.534929 | 6.603126 | 7.045231 |
| Apoa4 | 0 | 0 | 0 | 0 | 0 | 0.032633 | 0.041638 | 0 |
| Rnase4 | 10.28317 | 9.990857 | 8.519721 | 10.86597 | 13.4758 | 11.07436 | 15.81849 | 16.04942 |
| LOC100361 | 93.25438 | 93.2031 | 91.77166 | 109.3958 | 116.6062 | 105.5614 | 138.7039 | 125.8914 |
| Ppp2r2d | 14.12562 | 15.3931 | 13.35551 | 12.59268 | 12.92146 | 13.62121 | 16.8658 | 16.07805 |
| Ogfod3 | 2.829513 | 2.840733 | 3.422719 | 3.415176 | 3.490429 | 3.249655 | 4.012648 | 3.320369 |
| AABR07041 | 2.493615 | 3.103549 | 3.747877 | 5.514644 | 3.235572 | 3.279606 | 1.952822 | 2.87328 |
| Dcp2 | 0.410177 | 0.464096 | 0.264211 | 0.352764 | 0.473086 | 0.263739 | 0.24474 | 0.193899 |
| Tet2 | 4.01517 | 4.428448 | 4.107693 | 3.867713 | 3.677545 | 4.035268 | 4.265507 | 4.641452 |
| Rnf145 | 6.688432 | 6.385201 | 6.645377 | 6.148312 | 6.275937 | 6.343727 | 7.932403 | 7.852134 |
| Gjc3 | 0 | 0.152509 | 0 | 0.186306 | 0.145746 | 0 | 0.301594 | 0.119471 |
| Idh2 | 1201.384 | 1234.133 | 1258.363 | 1066.557 | 1037.468 | 1048.143 | 1091.367 | 1081.233 |
| Deptor | 12.6968 | 13.68175 | 11.86387 | 8.294958 | 8.970982 | 8.157994 | 9.883109 | 9.556806 |
| Slc4a10 | 0.013748 | 0.015555 | 0.044278 | 0 | 0.029731 | 0.036163 | 0 | 0.024371 |
| Krit1 | 9.647712 | 9.262003 | 9.175838 | 11.77408 | 11.82657 | 11.89705 | 12.90979 | 13.56615 |
| Rn50_X_07 | 0 | 0 | 0 | 0 | 0 | 0.113932 | 0.145371 | 0.230345 |
| Magi2 | 0.182999 | 0.207055 | 0.166236 | 0.20754 | 0.41097 | 0.209824 | 0.299223 | 0.299449 |
| Snx18 | 5.517847 | 5.366712 | 5.514344 | 6.863498 | 5.92771 | 6.19647 | 7.639739 | 7.943495 |
| Sdccag3 | 14.68308 | 14.35585 | 13.11597 | 16.81423 | 16.9916 | 17.41448 | 22.1448 | 20.44544 |
| Birc6 | 4.695691 | 4.599938 | 4.411571 | 4.254309 | 4.381786 | 4.236135 | 5.106452 | 5.356715 |
| Trpm4 | 4.777166 | 5.052101 | 5.244102 | 4.223513 | 4.502345 | 4.113153 | 4.586143 | 4.777825 |
| Bcam | 74.00705 | 75.38636 | 83.59759 | 69.36761 | 67.26111 | 72.39997 | 86.92352 | 86.44154 |
| Atg4a | 6.44438 | 4.877104 | 5.681379 | 5.780938 | 6.368302 | 5.949799 | 6.875462 | 7.414218 |
| Sergef | 6.817724 | 6.975366 | 6.307024 | 7.886246 | 8.156151 | 7.758403 | 8.276504 | 8.935759 |
| Wdr48 | 10.18985 | 10.5251 | 9.418558 | 11.40271 | 11.90402 | 10.77524 | 11.5909 | 12.3298 |
| Qars | 34.5513 | 32.39576 | 34.18974 | 32.7264 | 34.3743 | 33.19639 | 32.8785 | 33.69201 |
| Rab36 | 0.251961 | 0.237568 | 0.360662 | 0.309563 | 0.272442 | 0.22092 | 0.187922 | 0.223326 |
| AC124896. | 48.90188 | 53.45387 | 52.97405 | 50.96971 | 50.20715 | 49.27487 | 55.12153 | 48.92959 |
| Mycbp2 | 3.311086 | 3.33733 | 3.265665 | 3.433744 | 3.522014 | 3.520212 | 4.164644 | 4.587937 |
| Myo3b | 1.61652 | 1.283829 | 1.45176 | 0.58723 | 0.65547 | 0.572398 | 0.52168 | 0.633739 |
| Msi2 | 45.71375 | 45.21995 | 46.9056 | 40.30902 | 39.99253 | 38.35539 | 45.60554 | 44.99944 |
| Plekhh2 | 0.833171 | 1.210215 | 1.051598 | 1.130852 | 1.16873 | 1.125403 | 0.730576 | 1.237454 |
| AABR07045 | 0 | 0 | 0 | 0.106932 | 0.03137 | 0 | 0.129828 | 0 |
| Hsf4 | 4.911975 | 5.026065 | 4.906479 | 5.903728 | 6.789159 | 5.467794 | 7.98013 | 7.230973 |
| Olr1086 | 0 | 0 | 0 | 0 | 0 | 0 | 0 | 0.048198 |
| Nefm | 0.034248 | 0.038751 | 0.036768 | 0.142014 | 0.129613 | 0.105102 | 0.019158 | 0.106246 |

| | | | | | | | | |
|---|---|---|---|---|---|---|---|---|
| Gucy2c | 0.138902 | 0.125729 | 0.089472 | 0.102394 | 0.060077 | 0.048716 | 0.062159 | 0.049246 |
| Alkbh1 | 5.490078 | 5.332606 | 4.923021 | 5.399281 | 4.862016 | 4.914224 | 5.814289 | 5.995162 |
| Gins2 | 2.348472 | 2.190764 | 2.092088 | 1.876255 | 2.336759 | 2.092001 | 2.669292 | 2.424804 |
| Car9 | 0.050693 | 0 | 0.081633 | 0.303626 | 0.219255 | 0.088895 | 0.113426 | 0.157261 |
| Capn2 | 30.10444 | 29.37173 | 29.74184 | 30.87266 | 31.29393 | 29.87318 | 38.53376 | 38.47171 |
| Slc2a4 | 115.5561 | 118.8538 | 119.9067 | 106.7114 | 108.4097 | 107.5491 | 98.95806 | 99.07073 |
| Emg1 | 7.41946 | 11.24705 | 10.79463 | 9.080459 | 8.982784 | 9.368765 | 7.627544 | 8.175861 |
| #N/A | 0 | 0.265693 | 0 | 0 | 0 | 0 | 0 | 0 |
| Adh6 | 10.11227 | 9.939136 | 11.33136 | 19.29498 | 19.40186 | 19.37484 | 17.59825 | 20.00834 |
| Ly49si3 | 0 | 0.147387 | 0.069923 | 0.120033 | 0.070426 | 0.057108 | 0.145733 | 0.115459 |
| Fam50b | 0 | 0.047286 | 0 | 0.07702 | 0 | 0 | 0 | 0 |
| Zfp13 | 0.763926 | 0.585525 | 0.846579 | 1.044538 | 0.666147 | 1.037127 | 1.213047 | 0.764474 |
| Rn60_12_( | 0 | 0.19331 | 0 | 0 | 0 | 0 | 0 | 0 |
| Diras1 | 0 | 0.031233 | 0.088906 | 0.101746 | 0.029848 | 0 | 0.154414 | 0.073402 |
| Mettl24 | 0.738588 | 0.877462 | 0.753276 | 0.408348 | 0.31945 | 0.518076 | 0.371835 | 0.458253 |
| Pgpep1l | 3.659984 | 2.707642 | 2.720234 | 3.502244 | 2.739799 | 2.962225 | 3.464685 | 3.618336 |
| AABR07068 | 0 | 0 | 0 | 0 | 0 | 0 | 0 | 0 |
| Cyth2 | 19.61233 | 18.66099 | 21.66074 | 19.3563 | 20.81182 | 19.75661 | 24.24321 | 24.23673 |
| Ctsb | 144.1007 | 145.1645 | 143.3245 | 193.1616 | 188.2363 | 187.3226 | 210.3558 | 218.7362 |
| Fancd2 | 0.636266 | 0.719905 | 0.502638 | 0.707978 | 0.649043 | 0.621035 | 0.819272 | 0.691642 |
| Zbed5 | 27.70951 | 29.99406 | 29.38625 | 30.22854 | 30.34894 | 29.96349 | 29.79879 | 32.36139 |
| LOC10035( | 11.05503 | 8.887436 | 8.432724 | 9.114481 | 11.3245 | 8.290115 | 8.136759 | 9.669691 |
| AABR07025 | 1.082189 | 1.42852 | 1.290888 | 1.274193 | 1.007634 | 1.528726 | 2.017842 | 1.94504 |
| Hace1 | 3.621938 | 3.719241 | 3.381912 | 3.60391 | 3.965715 | 3.269122 | 4.562828 | 4.141022 |
| Rfxap | 0 | 0.036172 | 0.034321 | 0.029459 | 0 | 0.028031 | 0.035766 | 0 |
| AABR07043 | 0 | 0.188094 | 0.17847 | 0.306369 | 0.359508 | 0.14576 | 0.371966 | 0 |
| Cyc1 | 570.5913 | 575.5886 | 596.6182 | 555.7388 | 542.1073 | 564.9211 | 502.1123 | 503.3353 |
| Arhgef10 | 7.512788 | 7.526366 | 8.449527 | 7.674762 | 7.917964 | 7.802718 | 10.40616 | 9.522705 |
| Golt1b | 7.418505 | 6.068967 | 6.793028 | 7.673589 | 8.119826 | 8.050977 | 8.340178 | 8.203121 |
| AABR0704( | 0 | 0 | 0 | 0 | 0 | 0.478276 | 0 | 0 |
| Sall1 | 0.342327 | 0.455678 | 0.421555 | 0.129887 | 0.141529 | 0.097108 | 0.157698 | 0.142786 |
| Arl11 | 2.447342 | 1.913904 | 1.545516 | 3.283203 | 2.451696 | 2.556064 | 4.066702 | 2.998601 |
| Trim55 | 34.83824 | 33.07524 | 32.82395 | 42.61481 | 41.31971 | 43.30118 | 40.8304 | 42.51311 |
| #N/A | 0 | 0 | 0 | 0 | 0 | 0 | 0 | 0.154715 |
| Fmn1 | 1.710196 | 2.126908 | 1.987741 | 2.109854 | 2.261844 | 2.193486 | 2.3086 | 2.117153 |
| Nup155 | 2.992682 | 3.304486 | 2.864455 | 3.067734 | 2.963033 | 2.897976 | 2.86402 | 2.876273 |
| Rnf32 | 0.509757 | 0.262166 | 0.298504 | 0.469721 | 0.751626 | 0.609484 | 0.570293 | 0.821496 |
| AABR07066 | 0.837855 | 0 | 0 | 0 | 0 | 0 | 1.406032 | 0.371316 |
| Ttc7a | 7.596029 | 7.394193 | 7.790361 | 7.403506 | 6.852211 | 7.553193 | 8.355674 | 9.190125 |
| Fam134c | 10.25549 | 9.082754 | 9.293616 | 9.82615 | 9.415631 | 9.901662 | 10.90257 | 9.979347 |
| Timm10b | 11.5396 | 11.97598 | 9.056421 | 10.11996 | 9.293665 | 10.04815 | 9.704755 | 9.24058 |
| AC128059. | 0.393729 | 0 | 0 | 0 | 0 | 0 | 0 | 0 |
| LOC100912 | 7.845436 | 20.4272 | 6.900424 | 12.62943 | 6.950056 | 6.464494 | 5.816167 | 5.78087 |
| Btbd9 | 3.778708 | 5.488318 | 4.459475 | 3.802959 | 4.839283 | 3.947612 | 3.59783 | 4.465675 |
| Nek6 | 3.970749 | 4.191191 | 4.262853 | 5.942625 | 6.310601 | 6.402325 | 5.128022 | 5.999637 |
| Gucy1a2 | 1.480477 | 1.215792 | 1.461211 | 1.584234 | 1.058606 | 1.025905 | 1.789864 | 1.820177 |
| Rabepk | 2.829691 | 2.824996 | 2.859156 | 3.128938 | 3.311679 | 3.152425 | 3.202976 | 3.658869 |
| Fam58b | 8.825211 | 9.88342 | 9.281072 | 9.335301 | 10.61368 | 9.475044 | 7.807931 | 7.263493 |
| AC118094. | 0 | 0 | 0 | 0 | 0 | 0 | 0 | 0.057801 |
| AABR07047 | 2.909218 | 2.025627 | 2.52261 | 2.165205 | 2.298777 | 1.962157 | 3.004342 | 2.181879 |
| Gm19345 | 0 | 0 | 0 | 0 | 0 | 0 | 0.182507 | 0 |
| AABR07021 | 0.038448 | 0.130506 | 0.041276 | 0.106284 | 0.083146 | 0.033711 | 0.086027 | 0 |
| Ndufa9 | 301.4703 | 309.1303 | 290.5279 | 296.346 | 313.7095 | 308.8859 | 265.2935 | 263.3866 |
| AABR07007 | 0.127722 | 0 | 0 | 0 | 0.069052 | 0.055993 | 0.071445 | 0 |

| | | | | | | | | |
|---|---|---|---|---|---|---|---|---|
| Olah | 0.463415 | 0.749046 | 0.817331 | 0.427019 | 0.608459 | 0.406323 | 0.444384 | 0.410748 |
| AABR07027 | 0 | 0 | 0 | 0 | 0 | 0 | 0 | 0 |
| Cit | 0.304884 | 0.326316 | 0.362698 | 0.303718 | 0.240568 | 0.252873 | 0.304216 | 0.284841 |
| Zfp414 | 9.117281 | 11.2151 | 11.595 | 10.16762 | 10.11116 | 8.731951 | 10.20001 | 9.945968 |
| AABR07001 | 0 | 0.110643 | 0 | 0 | 0 | 0 | 0 | 0 |
| AABR07027 | 0 | 0.203607 | 0 | 0 | 0 | 0.157782 | 0 | 0.1595 |
| Galr3 | 0.045027 | 0.152836 | 0 | 0 | 0 | 0 | 0.100747 | 0 |
| AC136867. | 0.206165 | 0 | 0 | 0 | 0 | 0 | 0 | 0 |
| Actg1 | 179.7525 | 151.1179 | 194.6703 | 167.6026 | 199.5843 | 196.0321 | 185.9336 | 243.4705 |
| Adam33 | 3.762687 | 4.307686 | 4.708749 | 8.390958 | 9.00375 | 7.925715 | 8.49378 | 8.229105 |
| Pcsk4 | 2.229249 | 2.25551 | 2.692397 | 2.192427 | 1.483357 | 1.860636 | 2.110295 | 2.108887 |
| AABR07058 | 0.671358 | 0 | 0 | 0.61863 | 1.451859 | 0 | 0 | 0.595058 |
| Mcpt114 | 0.072832 | 0.412028 | 0.312758 | 0.335557 | 0.157504 | 0.447012 | 0.325923 | 0.193663 |
| Baz2a | 9.509776 | 9.28302 | 8.752462 | 7.70332 | 8.423369 | 8.574337 | 10.39574 | 10.78874 |
| Lrrc3b | 8.526246 | 8.659553 | 9.15347 | 8.042189 | 8.275598 | 8.829709 | 5.332707 | 5.355521 |
| Psca | 0.121499 | 0 | 0 | 0.111956 | 0.262749 | 0.10653 | 0 | 0.215381 |
| Pla2g7 | 5.689358 | 7.111297 | 6.843394 | 11.5555 | 13.26989 | 12.2752 | 9.764111 | 9.029445 |
| Mcmdc2 | 2.101727 | 2.134475 | 1.889343 | 2.55499 | 2.683272 | 2.475563 | 1.841394 | 2.569856 |
| RGD156541 | 0 | 0 | 0 | 0.133296 | 0 | 0 | 0 | 0.128217 |
| AC133827. | 0 | 0.510772 | 0 | 0 | 0 | 0 | 0 | 0 |
| Cyp2d1 | 0.615688 | 0.879944 | 0.45225 | 1.403402 | 1.366509 | 0.93761 | 1.305104 | 1.23504 |
| Gtf2f1 | 17.7393 | 15.93989 | 18.35828 | 21.89756 | 22.83325 | 22.65043 | 23.59046 | 23.54438 |
| Zmynd11 | 19.00237 | 19.44478 | 17.41069 | 18.93957 | 19.09881 | 20.18424 | 20.81395 | 21.97902 |
| Slc5a7 | 0 | 0 | 0 | 0 | 0.017086 | 0 | 0.017678 | 0 |
| Cyp4f6 | 16.06609 | 15.94627 | 16.42827 | 19.20158 | 16.23683 | 16.84833 | 16.65712 | 16.29866 |
| Cyp4f4 | 0 | 0 | 0 | 0 | 0 | 0.04142 | 0 | 0.020936 |
| Pdxdc1 | 2.891741 | 3.573045 | 2.403041 | 2.764967 | 3.5062 | 3.309917 | 4.169149 | 3.142203 |
| AABR07030 | 1.611259 | 3.190362 | 3.459579 | 2.227068 | 1.306673 | 1.059565 | 6.759769 | 2.856278 |
| Sccpdh | 0.896581 | 0.79253 | 3.459116 | 1.807234 | 7.058879 | 0.835255 | 1.222473 | 1.490033 |
| Ikbkg | 6.929191 | 5.349204 | 5.811664 | 5.287559 | 6.516857 | 5.727444 | 7.186816 | 7.261265 |
| Polh | 0.955402 | 0.857339 | 0.937264 | 1.199118 | 1.2468 | 1.22766 | 1.290005 | 1.387033 |
| Tuba3a | 0.034248 | 0.155002 | 0.147072 | 0.063117 | 0.037032 | 0.150145 | 0.229894 | 0.121425 |
| Spint1 | 0.480631 | 0.284854 | 0.466847 | 0.316345 | 0.272224 | 0.24081 | 0.230447 | 0.507152 |
| Asap2 | 9.675534 | 10.66411 | 10.73291 | 8.948583 | 9.340394 | 7.636733 | 11.80497 | 12.01578 |
| Sycp1 | 0 | 0 | 0 | 0 | 0 | 0 | 0 | 0.01316 |
| Ddc | 4.635706 | 4.111958 | 4.289517 | 5.45131 | 5.548381 | 5.66689 | 5.7291 | 5.68285 |
| Crebl2 | 4.844796 | 5.004992 | 4.568008 | 4.347829 | 5.375267 | 4.801998 | 5.561537 | 4.854294 |
| U2af2 | 39.12078 | 40.16036 | 44.671 | 40.83393 | 40.83865 | 39.74405 | 45.38249 | 44.86192 |
| Znf750 | 0.073651 | 0.055555 | 0.052713 | 0.022622 | 0 | 0.021526 | 0.027466 | 0.04352 |
| Dnah8 | 0.151214 | 0.133897 | 0.127047 | 0.09693 | 0.092416 | 0.092232 | 0.13975 | 0.192301 |
| Esm1 | 1.879679 | 1.635975 | 1.629887 | 1.843079 | 1.719782 | 2.260861 | 0.916649 | 1.345658 |
| Mllt6 | 15.36889 | 16.79681 | 17.88589 | 13.13266 | 13.1964 | 13.36325 | 17.27213 | 17.91202 |
| Sephs1 | 5.191664 | 5.320601 | 4.448148 | 4.783914 | 5.138668 | 4.271929 | 4.981651 | 5.229929 |
| Srrm1 | 25.05486 | 25.95628 | 24.98397 | 24.16291 | 22.31463 | 21.45627 | 28.63041 | 29.33946 |
| AC099450. | 6.940062 | 1.427701 | 1.354654 | 1.162726 | 3.410995 | 9.404172 | 2.823357 | 0.559211 |
| Ttc9 | 5.026355 | 4.429364 | 4.280571 | 3.963571 | 3.78877 | 4.00454 | 4.109307 | 4.43369 |
| Sstr1 | 0.026813 | 0.045507 | 0.043178 | 0.024707 | 0.072481 | 0.058774 | 0.10499 | 0.047532 |
| AABR07030 | 0 | 0 | 0 | 0 | 0 | 0 | 0 | 0.143255 |
| Tas2r108 | 0 | 0 | 0 | 0 | 0 | 0.05018 | 0 | 0 |
| Thg1l | 2.182851 | 2.087866 | 2.125998 | 2.384663 | 2.189955 | 2.288818 | 2.316197 | 2.23396 |
| Cdk1 | 1.249783 | 1.036985 | 1.341722 | 1.343564 | 1.216235 | 1.314973 | 1.071955 | 1.255444 |
| Mcam | 27.34297 | 30.56228 | 30.00674 | 30.47209 | 31.99451 | 32.78914 | 39.73622 | 39.10309 |
| LOC102550 | 26.24875 | 21.43703 | 15.67893 | 53.22394 | 56.19571 | 52.95163 | 61.01343 | 59.18434 |
| Hk3 | 0.117337 | 0.151727 | 0.179956 | 0.64873 | 0.61625 | 0.543802 | 0.48758 | 0.520009 |

| | | | | | | | | |
|---|---|---|---|---|---|---|---|---|
| Gys2 | 0.080968 | 0 | 0.021731 | 0 | 0.021887 | 0.053244 | 0.045292 | 0.017941 |
| Cwh43 | 0 | 0 | 0 | 0 | 0 | 0.017626 | 0 | 0 |
| Setd4 | 3.667772 | 4.289404 | 3.772143 | 4.458942 | 4.165871 | 4.134736 | 5.827433 | 4.72614 |
| Topors | 5.146171 | 4.951148 | 5.09331 | 4.711135 | 5.093733 | 4.756862 | 5.207803 | 5.758527 |
| Hoxd8 | 0.252062 | 0.142598 | 0 | 0.406464 | 0.408827 | 0.221008 | 0.422994 | 0.502684 |
| AABR0701§ | 1.921685 | 1.087148 | 0 | 0.885379 | 1.038945 | 0.421234 | 1.074948 | 0.851643 |
| AABR07021 | 2.084216 | 3.684675 | 1.678154 | 3.000817 | 4.507265 | 3.654884 | 6.849451 | 7.273917 |
| AABR07054 | 0 | 0 | 0 | 0 | 0.080774 | 0.065499 | 0 | 0 |
| AABR07048 | 0.306233 | 0.692977 | 0.328761 | 0.282182 | 0.993377 | 0 | 0 | 0 |
| Dstn | 65.72141 | 63.39634 | 60.35283 | 83.22664 | 83.52324 | 85.97392 | 76.47974 | 78.62388 |
| AC135285. | 0 | 0 | 0 | 0.446788 | 0 | 0.425134 | 0 | 1.719056 |
| Svil | 28.7329 | 28.704 | 27.63924 | 30.03573 | 28.98785 | 29.12548 | 30.51841 | 31.07125 |
| AABR07012 | 0 | 0 | 0 | 0 | 0 | 0 | 0 | 0.627223 |
| Mir3565 | 0 | 0 | 0 | 0 | 0 | 0.402759 | 0 | 0 |
| LOC10255( | 0.250664 | 0.438313 | 0.415887 | 0.356964 | 0.221759 | 0.259743 | 0.305925 | 0.343362 |
| Aspg | 0 | 0 | 0.021359 | 0.036667 | 0.043026 | 0.017445 | 0 | 0 |
| AABR07034 | 0.023378 | 0.017634 | 0 | 0 | 0.008426 | 0.013665 | 0.026154 | 0.006907 |
| AABR07058 | 3.035706 | 4.293448 | 1.629512 | 2.097962 | 2.461848 | 0.665427 | 3.820739 | 3.699708 |
| AABR07002 | 0.429229 | 0 | 0 | 0 | 0.464119 | 0 | 0 | 0 |
| AABR07062 | 0.781581 | 1.768644 | 0 | 0 | 0 | 1.370582 | 1.748796 | 1.385508 |
| AABR07021 | 0 | 0.093824 | 0.044512 | 0.07641 | 0.044832 | 0.072707 | 0.046385 | 0.183747 |
| Vps13d | 7.547122 | 7.324049 | 7.282674 | 6.984671 | 7.090158 | 7.072144 | 7.887576 | 7.683408 |
| Hk1 | 42.44725 | 43.91803 | 48.27384 | 48.3251 | 45.15867 | 47.64355 | 54.32515 | 56.29621 |
| C3 | 0.316688 | 0.439228 | 0.372887 | 1.29905 | 1.391814 | 1.540634 | 1.817199 | 1.901492 |
| Lig1 | 1.389335 | 1.688409 | 1.307399 | 1.485684 | 1.094244 | 1.21817 | 1.362434 | 1.596307 |
| Efemp1 | 10.71698 | 10.68906 | 11.66895 | 9.407255 | 9.830679 | 9.663864 | 11.02369 | 12.02005 |
| Mybl1 | 0.410589 | 0.428827 | 0.259955 | 0.601467 | 0.512266 | 0.581547 | 0.706691 | 0.485234 |
| AC129839. | 0.770087 | 0.871317 | 0 | 0 | 1.665368 | 1.350426 | 2.584618 | 0 |
| Sap130 | 4.067133 | 4.931491 | 5.060043 | 4.483233 | 4.274431 | 4.432586 | 5.627416 | 5.289436 |
| Cstf2t | 6.797146 | 7.109285 | 7.565102 | 7.737817 | 7.571892 | 6.899401 | 8.754031 | 7.833344 |
| Ppara | 11.17362 | 10.91274 | 12.47303 | 9.809422 | 9.497194 | 9.504591 | 13.62 | 13.40207 |
| Pole3 | 5.619174 | 7.845834 | 5.775839 | 6.499852 | 5.300281 | 5.031724 | 5.751463 | 6.040245 |
| Lrrk2 | 3.440144 | 3.623672 | 3.031743 | 4.323206 | 4.372127 | 4.136187 | 4.408749 | 4.733041 |
| Gatb | 36.50922 | 32.74566 | 33.03449 | 37.19246 | 36.41946 | 31.86548 | 35.72765 | 36.48786 |
| Hook1 | 2.056379 | 1.828891 | 1.807196 | 1.621658 | 1.923614 | 1.492745 | 1.872569 | 1.61921 |
| Ager | 5.077028 | 5.470875 | 5.398605 | 5.435718 | 6.273963 | 5.426643 | 6.76185 | 4.842881 |
| Abca7 | 2.602894 | 2.36127 | 2.604224 | 2.512001 | 2.289881 | 2.417263 | 3.17908 | 3.38553 |
| Dip2b | 5.365658 | 5.201361 | 4.670576 | 4.002164 | 3.731974 | 4.26594 | 4.956415 | 5.225007 |
| Pmf1 | 4.620522 | 4.773303 | 3.738292 | 4.25763 | 5.502955 | 5.108133 | 4.345154 | 4.748288 |
| Cd200r11 | 0.604222 | 0.227883 | 0.216224 | 0.494904 | 0.217779 | 0.470918 | 0.150217 | 0.357035 |
| Fam65c | 0.282772 | 0.279951 | 0.246654 | 0.37456 | 0.229318 | 0.418391 | 0.415214 | 0.454276 |
| AABR07049 | 0.065294 | 0 | 0 | 0 | 0 | 0 | 0 | 0 |
| Otud4 | 10.48138 | 9.972854 | 10.18147 | 9.695959 | 9.752312 | 9.453687 | 11.69553 | 12.02756 |
| Ccdc61 | 2.83059 | 2.457871 | 2.773806 | 2.593113 | 2.259918 | 2.683876 | 3.534964 | 2.742278 |
| Cyp27a1 | 14.9091 | 15.38511 | 16.27758 | 18.31753 | 19.42978 | 18.96292 | 19.12496 | 18.63922 |
| Socs4 | 3.710559 | 3.340919 | 3.618834 | 3.876624 | 4.181703 | 4.101144 | 3.624999 | 3.937359 |
| Grn | 39.30513 | 41.36081 | 41.85393 | 50.53798 | 50.99477 | 51.79102 | 58.00788 | 59.6012 |
| Dgcr2 | 0.255943 | 0.202711 | 0.357202 | 0.259425 | 0.387446 | 0.359058 | 0.200436 | 0.70325 |
| AABR07057 | 0.238569 | 0.26993 | 0.12806 | 0.109916 | 0.257961 | 0.104589 | 0.400351 | 0.317183 |
| Sntb2 | 5.24276 | 6.560024 | 6.456149 | 4.973085 | 5.135375 | 4.164211 | 5.865367 | 5.986325 |
| Adamtsl5 | 20.87661 | 20.52031 | 24.87293 | 24.56273 | 23.22008 | 24.72654 | 35.95348 | 31.9293 |
| LOC300249 | 9.668193 | 12.30063 | 14.255 | 12.92358 | 12.96665 | 13.13402 | 12.81249 | 11.87947 |
| AABR07044 | 0 | 0 | 0.53036 | 0 | 0.534175 | 0 | 0 | 0 |
| Akap3 | 0.125449 | 0.060831 | 0.01924 | 0.13211 | 0.19378 | 0.109994 | 0.140347 | 0.079423 |

| | | | | | | | | |
|---|---|---|---|---|---|---|---|---|
| AABR07008 | 13.02221 | 11.91261 | 9.518419 | 6.893305 | 7.19016 | 11.17495 | 12.70884 | 10.31434 |
| AC141172. | 0 | 0 | 0 | 0 | 0.544447 | 0 | 0 | 0.446293 |
| Aplar | 11.1198 | 13.30206 | 12.04299 | 11.32978 | 9.746064 | 10.56592 | 14.13924 | 13.11243 |
| Rnf17 | 0.010049 | 0.01137 | 0 | 0 | 0.010866 | 0.008811 | 0 | 0 |
| Olr1462 | 0 | 0 | 0 | 0 | 0 | 0 | 0 | 0 |
| Clcn7 | 5.505184 | 6.25041 | 6.298725 | 6.617473 | 5.252361 | 5.996108 | 8.076969 | 6.736774 |
| AABR07008 | 0 | 0 | 0 | 0 | 0.592906 | 0.120195 | 0.153363 | 0.243008 |
| RGD156127 | 1.118224 | 0.848271 | 1.009499 | 1.159199 | 1.222859 | 1.336991 | 0.710806 | 1.013663 |
| LOC100911 | 0 | 0 | 0 | 0.093514 | 0.054867 | 0.044491 | 0 | 0 |
| Epb4112 | 17.36562 | 18.10274 | 17.04321 | 20.25487 | 21.05999 | 20.01726 | 27.92613 | 29.775 |
| Cacng3 | 0 | 0 | 0 | 0 | 0.055268 | 0 | 0.028592 | 0 |
| LOC100364 | 2.131946 | 1.356861 | 2.002683 | 2.701193 | 2.737475 | 2.687107 | 2.832338 | 2.952577 |
| RGD156146 | 1.051525 | 0.4759 | 0.225776 | 0.581363 | 0.454799 | 0.553187 | 0.47056 | 0.745615 |
| Btnl2 | 0 | 0 | 0 | 0 | 0.034974 | 0 | 0 | 0 |
| Bcap31 | 43.86354 | 41.83484 | 41.94128 | 45.76213 | 46.91041 | 44.84404 | 49.9994 | 46.83963 |
| Gdf11 | 3.497151 | 3.764924 | 3.978559 | 4.677416 | 5.135957 | 4.690989 | 4.438011 | 4.55702 |
| Mkrn2 | 9.221523 | 8.985336 | 9.416351 | 11.46804 | 11.63722 | 11.68128 | 11.27138 | 11.68242 |
| Ptprf | 1.792532 | 1.54446 | 1.89219 | 2.425788 | 2.522143 | 2.288491 | 2.827704 | 2.918358 |
| Olr1418 | 0 | 0 | 0 | 0 | 0 | 0 | 0.061994 | 0 |
| LOC681282 | 22.778 | 20.9012 | 23.61331 | 23.36923 | 19.88982 | 19.69725 | 19.35308 | 17.83039 |
| Ybey | 1.196868 | 0.951962 | 1.19586 | 1.081027 | 0.755992 | 0.924732 | 1.179913 | 1.05034 |
| Fam107a | 7.699834 | 7.874691 | 8.039273 | 6.055996 | 5.791805 | 5.654341 | 11.09797 | 11.60363 |
| Ubl3 | 24.77423 | 26.05223 | 25.92919 | 22.79267 | 23.97265 | 22.76132 | 27.56414 | 26.84981 |
| Gtf2a2 | 62.1434 | 60.20693 | 60.45723 | 57.78248 | 58.24901 | 60.17518 | 55.95519 | 57.664 |
| Hist1h2ai | 2.293398 | 1.874074 | 2.462109 | 2.817701 | 1.377677 | 1.899139 | 2.280668 | 1.806891 |
| Idi1 | 1.453057 | 1.454365 | 1.399954 | 1.476261 | 1.349594 | 1.617052 | 1.271314 | 1.386987 |
| LOC103692 | 0 | 0 | 0.077596 | 0 | 0 | 0 | 0 | 0 |
| Zfp329 | 0.675223 | 0.388813 | 0.453059 | 0.61108 | 0.560619 | 0.539175 | 0.580046 | 0.641232 |
| Slc16a3 | 0.923517 | 0.677784 | 1.232619 | 2.000964 | 1.646317 | 1.685136 | 1.870912 | 1.570749 |
| Wdr88 | 0.040688 | 0 | 0 | 0 | 0 | 0 | 0.091041 | 0 |
| AC141541. | 0 | 0 | 0 | 0 | 0 | 0 | 0 | 0.080025 |
| Ptafr | 1.755547 | 2.165267 | 1.833755 | 2.390068 | 2.377085 | 2.080088 | 3.326462 | 3.168131 |
| Cul4a | 76.82935 | 74.61355 | 74.18361 | 76.83912 | 76.90234 | 76.00604 | 77.82201 | 78.90782 |
| AABR07070 | 8.10425 | 10.58028 | 6.692638 | 5.744421 | 8.763008 | 4.372808 | 7.671801 | 9.945968 |
| AABR07064 | 0.54125 | 0.612399 | 0.581066 | 0.748111 | 1.170491 | 1.186421 | 0.151382 | 0.359802 |
| AABR07071 | 0.146274 | 0 | 0 | 0 | 0 | 0 | 0 | 0 |
| LOC102547 | 0.134272 | 0.050641 | 0.240249 | 0.082484 | 0.241977 | 0.078486 | 0.300434 | 0.317364 |
| Mt-co2 | 30726.09 | 30996.54 | 29828.83 | 30771.67 | 31744.2 | 30608.45 | 31492 | 31086.26 |
| Slc25a22 | 0.144817 | 0.098312 | 0.031094 | 0.026689 | 0.031318 | 0.025395 | 0.064806 | 0.025672 |
| Txn1 | 283.2747 | 246.7792 | 228.799 | 264.3965 | 285.6291 | 286.4189 | 269.8614 | 265.3732 |
| Zdhhc11 | 0.035098 | 0.039712 | 0 | 0 | 0 | 0.061548 | 0.078532 | 0 |
| AABR07044 | 0 | 0 | 0 | 0 | 0 | 0 | 0 | 0 |
| Eya2 | 7.913073 | 8.368088 | 9.161478 | 6.386094 | 6.067696 | 5.804498 | 10.6465 | 10.70399 |
| Cmtm6 | 9.848784 | 9.395446 | 9.778574 | 9.831539 | 10.61455 | 9.877117 | 12.53071 | 11.22564 |
| Lrrc43 | 0 | 0 | 0.137722 | 0.094568 | 0.11097 | 0.089984 | 0.114816 | 0.20467 |
| AABR07013 | 0.065621 | 0 | 0 | 0 | 0 | 0.057537 | 0 | 0 |
| Psma8 | 0 | 0 | 0 | 0 | 0 | 0 | 0.028774 | 0.022797 |
| Rpl10 | 472.7308 | 457.3448 | 437.6198 | 490.4789 | 471.0401 | 466.1071 | 490.2732 | 482.4683 |
| AABR07057 | 0.278542 | 0 | 0.299033 | 0 | 0 | 0 | 0.623241 | 0 |
| Ppia | 248.1287 | 233.2893 | 211.6077 | 260.2416 | 244.213 | 211.9584 | 227.474 | 234.4566 |
| Rhox5 | 0.672316 | 0.422608 | 0.962365 | 0.826017 | 1.050061 | 0.851481 | 2.172898 | 1.78772 |
| Cbx5 | 16.72632 | 12.46395 | 13.5654 | 13.58402 | 15.72118 | 15.26932 | 16.9456 | 14.32281 |
| AABR07034 | 0 | 0 | 0 | 0.099903 | 0 | 0 | 0 | 0.192193 |
| Arhgef3 | 8.674759 | 9.455993 | 8.517903 | 10.26801 | 9.551474 | 9.863112 | 8.797563 | 8.923465 |

| | | | | | | | | |
|---|---|---|---|---|---|---|---|---|
| Nrg1 | 0.725834 | 0.51328 | 0.649358 | 0.459819 | 0.703081 | 0.517085 | 0.642858 | 0.670149 |
| Penk | 120.7897 | 123.2002 | 126.3402 | 154.141 | 149.0237 | 154.0219 | 165.7875 | 165.6856 |
| Parp11 | 0.912091 | 0.94225 | 0.808894 | 0.822185 | 0.793273 | 0.712796 | 1.153503 | 0.949028 |
| Ythdc1 | 14.07952 | 12.93589 | 12.67182 | 13.21759 | 12.01893 | 12.79356 | 15.35676 | 15.63831 |
| Setd6 | 5.569519 | 5.280711 | 6.513691 | 6.164245 | 5.954952 | 5.756362 | 5.15183 | 6.37062 |
| Zfp831 | 0 | 0.010106 | 0.019177 | 0 | 0.019315 | 0 | 0.009992 | 0.007917 |
| Asic1 | 2.071244 | 1.681817 | 1.80505 | 1.324772 | 1.080281 | 1.47422 | 1.662943 | 1.706257 |
| Tpcn2 | 2.773161 | 1.976344 | 2.06855 | 2.787664 | 3.46589 | 3.142016 | 3.646432 | 3.543337 |
| Itga9 | 10.08267 | 11.12239 | 11.13159 | 12.87371 | 12.01249 | 13.03198 | 17.98404 | 18.30621 |
| Bglap | 0 | 0.243325 | 0.346313 | 10.30457 | 9.417707 | 7.1653 | 8.420794 | 8.482325 |
| Ccrl11 | 0.586733 | 0.33193 | 0.314948 | 0.180217 | 0.211475 | 0.214353 | 0.164103 | 0.17335 |
| Wdr91 | 3.243727 | 3.761878 | 3.699995 | 4.707623 | 4.49385 | 4.763872 | 5.670215 | 5.049353 |
| Ppm1a | 35.35571 | 34.02581 | 32.65889 | 35.22693 | 34.71425 | 35.37757 | 38.83994 | 36.81772 |
| Abhd14b | 18.08463 | 17.03285 | 14.3479 | 17.62567 | 18.96371 | 18.03472 | 18.95386 | 15.72105 |
| Dag1b | 5.949264 | 5.784722 | 5.355695 | 5.424909 | 5.72926 | 6.19438 | 7.938396 | 7.635051 |
| Hipk2 | 34.99697 | 36.51953 | 35.36958 | 28.58782 | 26.91641 | 29.83353 | 40.98874 | 42.08977 |
| Serpina1 | 0.103015 | 0.038852 | 0.221186 | 0 | 0 | 0.030108 | 0.038416 | 0.091307 |
| Mrpl43 | 34.91061 | 39.49972 | 37.47877 | 31.44856 | 34.6496 | 31.98024 | 34.02866 | 35.6191 |
| Trmo | 0.820172 | 0.927986 | 0.65665 | 0.794185 | 0.916903 | 0.731316 | 0.948677 | 0.702317 |
| Brms1 | 11.34595 | 1.08154 | 1.204675 | 1.18718 | 0.89877 | 1.639803 | 1.255386 | 0.994597 |
| RGD15637( | 0.606087 | 0.822911 | 0 | 0.446788 | 0.131071 | 0.318851 | 0.678063 | 0.752087 |
| Cd164l2 | 0.122637 | 0.138758 | 0 | 0.169508 | 0.265211 | 0.107528 | 0.0686 | 0.05435 |
| Bmi1 | 14.05734 | 13.7459 | 13.69225 | 12.05256 | 14.34437 | 13.50906 | 14.99767 | 15.67779 |
| Cep19 | 17.4892 | 16.14503 | 14.04544 | 18.11445 | 17.37157 | 18.78185 | 20.13492 | 21.20948 |
| Grasp | 17.16915 | 14.8763 | 19.20828 | 17.11134 | 15.68234 | 17.78741 | 19.71356 | 18.94225 |
| Stx8 | 12.14192 | 13.38575 | 12.50035 | 13.77022 | 11.51302 | 14.24932 | 13.86248 | 13.13514 |
| Aqp1 | 312.2645 | 319.1573 | 321.1203 | 246.5254 | 244.6682 | 245.5827 | 367.6156 | 371.4923 |
| Mgat4c | 0 | 0 | 0 | 0 | 0 | 0 | 0 | 0 |
| Isca2 | 45.76298 | 51.26087 | 54.04243 | 55.72303 | 51.39147 | 53.30895 | 50.31991 | 42.06859 |
| AABR07012 | 0.382233 | 0 | 0.410352 | 0.704425 | 0.413303 | 0 | 0 | 0.338792 |
| Tshz1 | 6.339976 | 6.454202 | 6.648896 | 5.616767 | 4.687703 | 5.13019 | 6.47294 | 6.37062 |
| AABR07013 | 0 | 0 | 0 | 0.067676 | 0.238243 | 0.128792 | 0 | 0.26039 |
| Vps8 | 4.450469 | 5.178958 | 4.246989 | 5.058985 | 4.784808 | 4.491393 | 4.879691 | 4.686405 |
| Tmbim1 | 12.10996 | 12.8885 | 13.47574 | 21.34959 | 18.11681 | 22.10877 | 24.06487 | 24.94719 |
| RGD156445 | 6.02387 | 6.680761 | 5.698652 | 6.979668 | 7.029446 | 5.856973 | 6.205437 | 6.502262 |
| Smo | 4.665504 | 5.41022 | 5.736123 | 5.529934 | 5.358729 | 5.058232 | 7.103797 | 7.258167 |
| Pfdn5 | 98.9496 | 102.558 | 99.49653 | 88.77676 | 89.46885 | 94.82805 | 99.67593 | 101.6355 |
| Cd22 | 0.478016 | 0.488512 | 0.397301 | 0.440473 | 0.516872 | 0.621928 | 0.707294 | 0.68337 |
| Zfp146 | 8.87659 | 9.16717 | 6.907351 | 10.86931 | 10.6288 | 8.879935 | 10.26398 | 11.03599 |
| Clstn2 | 1.807061 | 2.396365 | 2.39892 | 1.503994 | 1.722837 | 1.37999 | 2.15209 | 2.480034 |
| Phlda2 | 0 | 0 | 0.06124 | 0.052563 | 0.06168 | 0 | 0.127635 | 0.05056 |
| Nectin2 | 12.04817 | 12.19138 | 13.13698 | 10.47186 | 10.22316 | 10.56385 | 13.50533 | 12.55971 |
| LOC102547 | 0.332683 | 0.11651 | 0.127556 | 0.255462 | 0.154168 | 0.069452 | 0.141787 | 0.491456 |
| Fam19a3 | 2.214 | 1.63372 | 2.170186 | 1.774012 | 2.498052 | 1.519229 | 3.015387 | 2.13302 |
| Olr833 | 0 | 0 | 0 | 0 | 0 | 0 | 0 | 0 |
| LOC100911 | 0 | 0 | 0.05254 | 0 | 0 | 0 | 0 | 0 |
| LOC681355 | 5.480847 | 3.774717 | 3.965331 | 3.568207 | 3.156431 | 3.656444 | 2.466021 | 2.957011 |
| Arhgef40 | 5.905931 | 6.294904 | 6.680386 | 8.809865 | 8.755295 | 8.855687 | 10.53337 | 11.12188 |
| RGD156381 | 2.922749 | 2.342425 | 1.895729 | 2.693198 | 1.975204 | 1.655057 | 2.179895 | 1.673081 |
| Nrm | 1.95588 | 2.212986 | 2.13938 | 2.108312 | 2.43409 | 2.103201 | 2.766154 | 2.158815 |
| Zg16 | 0.083518 | 0 | 0 | 0 | 0 | 0 | 0 | 0 |
| AABR07005 | 2.819703 | 2.734596 | 2.162237 | 1.113534 | 3.048905 | 2.825507 | 1.802605 | 2.142209 |
| Ppil2 | 13.53376 | 12.77736 | 12.53513 | 15.24212 | 15.49467 | 15.92529 | 14.25032 | 14.68748 |
| Rpia | 11.38641 | 11.34636 | 10.92097 | 10.1992 | 10.46829 | 10.26234 | 10.86338 | 10.98887 |

| | | | | | | | | |
|---|---|---|---|---|---|---|---|---|
| Rn50_19_( | 0.032265 | 0.109519 | 0 | 0.029731 | 0 | 0.141449 | 0.072193 | 0.14299 |
| Top1mt | 1.959269 | 1.928922 | 2.294619 | 2.133642 | 1.430695 | 1.628648 | 2.647412 | 2.616173 |
| Mrpl40 | 23.64209 | 26.67588 | 24.63203 | 22.99108 | 20.96519 | 23.06242 | 21.6917 | 22.90762 |
| Oxtr | 0.042993 | 0.048645 | 0.046156 | 0.079233 | 0.046488 | 0 | 0 | 0.076214 |
| AABR07058 | 0.110477 | 0 | 0.118604 | 0 | 0 | 0 | 0 | 0 |
| AABR07041 | 0.425739 | 1.445112 | 1.371175 | 2.157661 | 2.301728 | 0.373289 | 1.667043 | 2.264123 |
| AABR07072 | 0 | 0 | 0 | 0 | 0 | 0.096257 | 0 | 0 |
| BC068281 | 0.90615 | 0.647536 | 0.665607 | 1.03274 | 0.928238 | 0.731788 | 1.01376 | 1.310428 |
| AABR07014 | 0.255444 | 0 | 0.54847 | 0.353072 | 0.138104 | 0.111987 | 0.428668 | 0.226412 |
| Ndfip1 | 57.60251 | 54.22549 | 54.64011 | 57.45227 | 57.61589 | 56.19826 | 65.26537 | 66.39176 |
| Tmem14a | 0.226692 | 0 | 0 | 0 | 0 | 0 | 0 | 0 |
| Sec11a | 20.01329 | 19.98008 | 20.83021 | 24.30737 | 24.37455 | 21.02662 | 20.82902 | 23.30388 |
| AABR0707( | 1.10244 | 0 | 0 | 1.015855 | 0 | 0 | 0 | 0 |
| Ptrh2 | 3.779934 | 4.109918 | 3.919435 | 4.213654 | 4.107126 | 3.831596 | 3.568714 | 4.102128 |
| AABR07062 | 0 | 0.221081 | 0 | 0 | 0 | 0 | 0 | 0 |
| Lix1l | 29.20861 | 28.99021 | 31.15133 | 27.39059 | 25.86953 | 26.83953 | 33.42034 | 34.54925 |
| Dock2 | 0.990372 | 1.120559 | 0.908174 | 0.988638 | 0.803156 | 1.013086 | 1.131067 | 0.950967 |
| LOC10369( | 0.831205 | 1.097214 | 0.743626 | 0.510615 | 0.29959 | 0.728801 | 0.929915 | 0.982318 |
| Mtss1l | 7.140117 | 7.425534 | 8.056795 | 7.769217 | 6.587069 | 7.872196 | 7.461174 | 7.755951 |
| Neurl1 | 0.063887 | 0.09638 | 0 | 0.098115 | 0.06908 | 0.074688 | 0.166772 | 0.188754 |
| Eef1a2 | 452.753 | 474.8216 | 508.5913 | 360.5668 | 356.9668 | 380.2843 | 437.0981 | 445.0578 |
| Wipf2 | 4.15429 | 4.112836 | 4.54792 | 4.180595 | 3.989582 | 3.019429 | 5.014554 | 4.626917 |
| Foxp2 | 1.000178 | 0.771981 | 0.682542 | 0.63585 | 0.829972 | 0.849765 | 0.650555 | 0.749065 |
| Slc36a4 | 2.613881 | 2.957483 | 2.749286 | 1.985458 | 1.776018 | 2.338309 | 2.627913 | 2.942978 |
| Rab8b | 4.275978 | 4.114368 | 3.992876 | 4.431299 | 4.060017 | 4.039976 | 5.327083 | 5.616776 |
| AABR07008 | 0 | 0 | 0 | 0.107229 | 0 | 0 | 0 | 0 |
| Osgin2 | 3.148414 | 2.587982 | 1.675567 | 2.950731 | 4.073562 | 2.878503 | 3.55241 | 3.291471 |
| 43162 | 1.549817 | 1.315158 | 2.367754 | 2.636483 | 2.545918 | 2.14285 | 2.100645 | 2.113353 |
| Klk1c2 | 0 | 0 | 0 | 0 | 0 | 0 | 0 | 0 |
| Trim25 | 20.05961 | 19.11669 | 19.61743 | 15.01341 | 15.68581 | 14.07818 | 24.97012 | 25.2962 |
| Prpf40a | 10.54959 | 9.176702 | 8.186651 | 9.545023 | 9.055789 | 9.005114 | 10.48738 | 10.21015 |
| Taar8a | 0.046547 | 0 | 0 | 0 | 0.100662 | 0 | 0 | 0.041257 |
| Ccdc34 | 1.13839 | 1.264615 | 0.999928 | 1.525791 | 1.723294 | 1.397397 | 1.667232 | 1.81622 |
| AABR07004 | 0.089058 | 0.050382 | 0.095609 | 0.205158 | 0.192594 | 0.273301 | 0.099634 | 0.118404 |
| Rab15 | 0.050791 | 0.019156 | 0.072704 | 0.187209 | 0.073227 | 0.267204 | 0.113646 | 0.090038 |
| AABR07048 | 2.218895 | 2.00846 | 0.95285 | 0.408925 | 0.959704 | 1.167317 | 0.49648 | 0.393343 |
| Rag2 | 0 | 0 | 0 | 0 | 0.015968 | 0 | 0 | 0 |
| Rspo1 | 1.105673 | 1.251017 | 1.681599 | 2.914999 | 2.258259 | 2.77372 | 2.611399 | 3.67505 |
| AABR07071 | 0 | 0.140402 | 0 | 0 | 0 | 0 | 0 | 0 |
| RGD131117 | 22.36555 | 24.74661 | 26.08407 | 25.57499 | 27.43715 | 24.61111 | 28.58892 | 27.18792 |
| Ephb6 | 0.321974 | 0.291439 | 0.34566 | 0.415361 | 0.33422 | 0.225846 | 0.216126 | 0.376704 |
| Srrd | 7.490941 | 7.676058 | 7.8144 | 6.902608 | 7.794192 | 6.815916 | 9.092087 | 7.26597 |
| Anln | 0.49103 | 0.734795 | 0.374108 | 0.613016 | 0.565197 | 0.486088 | 0.531621 | 0.505421 |
| AABR07041 | 0 | 0 | 0 | 0 | 0 | 0 | 0 | 0.359802 |
| Gga3 | 5.539972 | 4.858697 | 5.034932 | 5.523519 | 4.880978 | 5.512822 | 5.951927 | 6.183406 |
| Ildr2 | 0.250255 | 0.106182 | 0.134333 | 1.210652 | 0.913266 | 1.23426 | 2.484774 | 1.497243 |
| Srpk1 | 12.14889 | 11.34206 | 11.27576 | 10.61569 | 10.67739 | 9.628924 | 13.3573 | 13.07563 |
| Zfp420 | 0.765006 | 0 | 0.556353 | 0 | 0.026684 | 0 | 0 | 0.043746 |
| Txlna | 10.42811 | 8.854984 | 8.907807 | 8.155459 | 8.794655 | 8.352987 | 11.36854 | 11.3494 |
| Kri1 | 5.177501 | 6.112799 | 6.16255 | 6.404366 | 6.450281 | 6.118642 | 6.768245 | 7.10808 |
| Wfdc11 | 0.259451 | 0 | 0.139269 | 0.079691 | 0.14027 | 0.41706 | 0.048377 | 0.076655 |
| Wars2 | 6.115535 | 6.08072 | 5.570657 | 5.342486 | 5.038201 | 4.758579 | 7.197206 | 5.702087 |
| AABR07003 | 0 | 0 | 0 | 0 | 0 | 0 | 0 | 0 |
| RGD156011 | 0 | 0 | 0 | 0.059134 | 0 | 0 | 0 | 0 |

| | | | | | | | | |
|---|---|---|---|---|---|---|---|---|
| LOC680322 | 0 | 0 | 0 | 0.24937 | 0 | 0 | 0 | 0 |
| Ppil1 | 13.0389 | 15.70471 | 12.079 | 14.92165 | 18.87417 | 14.93604 | 17.52834 | 13.98028 |
| Insr | 10.5528 | 11.12547 | 10.52897 | 8.826508 | 8.168734 | 9.416071 | 12.36503 | 12.19104 |
| Wdr1 | 37.18975 | 35.01762 | 36.25537 | 36.45237 | 36.00761 | 38.52376 | 38.02438 | 38.07754 |
| Chdh | 0.015379 | 0.017401 | 0 | 0.028343 | 0 | 0.013484 | 0 | 0.013631 |
| Txnip | 254.346 | 255.5232 | 247.6087 | 270.039 | 259.0218 | 265.1283 | 385.8471 | 397.377 |
| Znrf2 | 4.979025 | 5.182029 | 5.296619 | 4.621403 | 4.236392 | 4.437181 | 5.560166 | 6.004788 |
| Fat4 | 1.453208 | 1.383869 | 1.553343 | 1.083459 | 1.032785 | 1.127685 | 1.456505 | 1.385842 |
| LOC499584 | 0 | 0 | 0 | 0.069329 | 0 | 0.065969 | 0 | 0 |
| Trim3 | 8.60064 | 10.14182 | 10.10992 | 9.530245 | 9.672522 | 9.450175 | 11.32718 | 10.58238 |
| Sp7 | 0 | 0.082006 | 0 | 0.133573 | 0.156741 | 0.031775 | 0.202715 | 0.096362 |
| Rn60_14_( | 0 | 0 | 0.278308 | 0 | 0 | 0.227299 | 0 | 0 |
| Hist1h4m | 0 | 0 | 0 | 0 | 0.181482 | 0 | 0 | 0.446293 |
| Mon2 | 12.39808 | 12.66271 | 11.65753 | 14.04401 | 14.60549 | 13.71598 | 13.7463 | 13.82848 |
| Ankrd49 | 4.727139 | 4.378594 | 4.075685 | 4.468719 | 5.217322 | 4.982302 | 4.329456 | 4.689212 |
| Slc25a39 | 53.01823 | 56.13334 | 59.01934 | 51.3587 | 49.76511 | 55.8282 | 55.70611 | 56.5968 |
| Synrg | 13.76903 | 13.92242 | 13.4442 | 13.721 | 12.6988 | 13.13793 | 15.78754 | 16.18019 |
| Dguok | 31.52223 | 30.0314 | 31.85696 | 27.67459 | 27.47857 | 28.44898 | 29.2349 | 29.30485 |
| Mb21d2 | 2.721617 | 2.090938 | 2.867721 | 1.656428 | 1.780239 | 2.253743 | 2.499763 | 2.754792 |
| AC113785. | 0 | 0.128524 | 0 | 0 | 0 | 0 | 0.127082 | 0 |
| AABR07014 | 0.166241 | 0 | 0.892352 | 0.459554 | 0.179754 | 0.14576 | 0 | 0.147348 |
| Mogat3 | 0 | 0 | 0 | 0 | 0 | 0 | 0.059719 | 0 |
| Pigc | 9.594639 | 8.828579 | 9.617897 | 9.639975 | 10.81203 | 11.45328 | 9.182144 | 10.50218 |
| Zfx | 9.654394 | 10.11691 | 7.871855 | 9.083826 | 9.580238 | 8.500698 | 10.27681 | 10.23611 |
| Anxa11 | 78.08953 | 77.51448 | 80.82828 | 61.93897 | 57.35572 | 62.12073 | 77.20744 | 74.72078 |
| AABR07037 | 0 | 0 | 0 | 0 | 0 | 0 | 0 | 0 |
| Rpusd2 | 0.545242 | 0.591211 | 2.731642 | 0.71176 | 0.04913 | 0.119517 | 2.490801 | 2.517056 |
| Rpusd1 | 3.928847 | 3.77851 | 3.404422 | 3.542701 | 4.521305 | 3.789298 | 4.426816 | 4.502158 |
| RGD130694 | 2.081232 | 1.636398 | 1.742025 | 2.129054 | 2.212264 | 2.041331 | 1.716694 | 2.407489 |
| Ankrd52 | 7.174714 | 7.657482 | 8.117489 | 6.186299 | 6.328053 | 6.742687 | 7.928124 | 7.783838 |
| Tubgcp5 | 4.640914 | 4.242404 | 4.511428 | 5.00654 | 5.018153 | 5.26013 | 5.160389 | 5.116759 |
| Naalad2 | 1.145141 | 1.032146 | 0.854316 | 1.216165 | 1.070329 | 1.123186 | 0.759994 | 0.705336 |
| LOC102547 | 4.500304 | 4.360021 | 4.29947 | 4.489254 | 4.300632 | 4.68195 | 5.050137 | 4.769534 |
| Usp53 | 2.246213 | 2.415381 | 2.236577 | 2.409497 | 2.734715 | 2.510714 | 1.956659 | 2.14291 |
| #N/A | 0.108418 | 0.12267 | 0 | 0 | 0 | 0 | 0 | 0 |
| Idi2 | 0 | 0.109115 | 0.103533 | 0 | 0 | 0 | 0 | 0.085478 |
| AABR07008 | 0 | 0.057974 | 0 | 0 | 0 | 0 | 0 | 0 |
| Ptpn9 | 6.588585 | 6.46359 | 5.887575 | 8.229353 | 7.618302 | 6.912217 | 8.478799 | 8.540271 |
| Mex3a | 0.290231 | 0.375294 | 0.356093 | 0.458462 | 0.448318 | 0.436242 | 0.742165 | 0.404244 |
| Tspan7 | 40.35123 | 41.91074 | 40.78859 | 40.73317 | 38.53272 | 36.33406 | 42.70848 | 42.47632 |
| Ank1 | 24.04704 | 24.4445 | 24.86151 | 21.69696 | 21.11741 | 22.43752 | 22.23793 | 21.60395 |
| Fuz | 5.283209 | 6.065609 | 6.088911 | 6.264317 | 6.972802 | 5.892586 | 6.99713 | 6.714266 |
| #N/A | 0 | 0 | 0.211346 | 0 | 0 | 0 | 0 | 0.523472 |
| Dab1 | 0.067761 | 0.092002 | 0.043648 | 0.037464 | 0.058615 | 0.106944 | 0.030323 | 0.048048 |
| Klhdc1 | 4.35753 | 4.30336 | 4.110226 | 4.061712 | 3.921906 | 4.748251 | 3.747841 | 4.219502 |
| Kctd11 | 1.476119 | 1.943956 | 1.558729 | 2.14062 | 2.093254 | 2.036872 | 3.356977 | 2.745406 |
| AABR07052 | 0.170019 | 0 | 0 | 0 | 0 | 0 | 0 | 0.150696 |
| AABR07013 | 0 | 0 | 0 | 0 | 0 | 0 | 0.280309 | 0 |
| Usp43 | 1.695179 | 1.747103 | 1.819883 | 1.206328 | 1.125191 | 1.192011 | 1.596057 | 1.353757 |
| Col5a1 | 14.96819 | 15.88568 | 18.19886 | 21.43093 | 20.32704 | 21.46133 | 26.80338 | 26.5341 |
| Ttll1 | 1.668924 | 1.857353 | 1.468604 | 1.941218 | 2.100417 | 1.727191 | 1.775293 | 2.134001 |
| Rara | 6.85672 | 7.185928 | 6.362272 | 7.976957 | 7.698387 | 8.317456 | 10.49953 | 10.29043 |
| Tcap | 120.7777 | 122.7926 | 127.6839 | 94.40831 | 89.91608 | 73.00688 | 98.24758 | 104.6489 |
| AABR07054 | 2.380269 | 0.897721 | 0 | 1.82777 | 1.715834 | 2.434859 | 0.887646 | 2.461376 |

| | | | | | | | | |
|---|---|---|---|---|---|---|---|---|
| Rnf126 | 10.92824 | 11.7797 | 13.02751 | 12.48419 | 10.02729 | 10.97232 | 13.42164 | 12.19183 |
| Ctcf | 9.844526 | 9.322539 | 9.362514 | 8.676937 | 8.663317 | 8.326765 | 9.247848 | 9.994237 |
| Zmat3 | 3.877445 | 4.063884 | 3.549237 | 3.986619 | 3.927828 | 3.363961 | 4.246589 | 4.087951 |
| Rpl41 | 951.4521 | 1008.848 | 991.7493 | 991.8579 | 1019.589 | 1015.094 | 998.7165 | 995.3452 |
| Bcl2l11 | 5.082523 | 5.069375 | 4.372735 | 4.764936 | 4.653118 | 4.316614 | 5.131359 | 4.489196 |
| AABR07064 | 5.975691 | 4.448166 | 6.415286 | 4.781842 | 4.93109 | 4.825847 | 3.518599 | 4.878403 |
| Fdps | 24.08177 | 23.94263 | 25.14939 | 25.07405 | 25.00801 | 24.93023 | 26.44146 | 24.30356 |
| Usb1 | 2.935035 | 3.735961 | 3.387269 | 3.358112 | 3.543866 | 3.13102 | 4.706475 | 3.620376 |
| Cdh9 | 0 | 0 | 0 | 0 | 0 | 0 | 0 | 0 |
| Tor1aip2 | 10.27447 | 8.763521 | 8.581815 | 9.73802 | 9.864379 | 9.89963 | 8.084128 | 9.607145 |
| Ttc28 | 3.717702 | 3.576569 | 4.028541 | 3.297481 | 3.584588 | 3.573451 | 4.059112 | 4.083737 |
| AABR07035 | 0 | 0 | 0 | 0 | 0 | 0 | 0.468677 | 0 |
| Ppat | 10.25002 | 10.26151 | 9.875787 | 9.3613 | 10.01697 | 9.408147 | 9.464123 | 9.625607 |
| Exosc3 | 10.20589 | 10.58519 | 10.63058 | 9.124433 | 9.721731 | 8.735471 | 10.33047 | 10.23058 |
| Itpkc | 3.325578 | 3.402665 | 3.057749 | 2.947092 | 2.959305 | 2.985627 | 3.684906 | 3.963075 |
| AABR07052 | 0 | 0 | 0 | 0 | 0 | 0.433156 | 0 | 0 |
| Ubac1 | 42.81052 | 46.17063 | 46.48989 | 42.49916 | 42.39622 | 42.55303 | 41.1035 | 44.22637 |
| Cngb1 | 0.176079 | 0.139458 | 0.207935 | 0.22715 | 0.171353 | 0.077193 | 0.137893 | 0.265315 |
| AABR07007 | 5.855974 | 7.262851 | 3.868777 | 5.60359 | 4.627216 | 4.542078 | 6.803381 | 6.587867 |
| Arid3c | 0 | 0 | 0 | 0.039135 | 0 | 0 | 0 | 0 |
| Qrsl1 | 9.094864 | 9.826617 | 8.416222 | 8.54581 | 9.197003 | 8.064237 | 8.799165 | 8.969537 |
| Bmt2 | 4.757133 | 3.81435 | 3.619195 | 4.210932 | 3.199698 | 4.006844 | 3.645827 | 4.448888 |
| Ttk | 0.100639 | 0.265693 | 0.144057 | 0.247293 | 0.199503 | 0.147068 | 0.281477 | 0.148669 |
| Spry2 | 12.35373 | 13.29024 | 11.95801 | 10.5837 | 9.541363 | 10.42589 | 11.52273 | 11.97546 |
| Blcap | 17.97879 | 16.75777 | 17.78982 | 14.12165 | 14.58015 | 14.50556 | 17.23613 | 16.19948 |
| Rab42 | 0.163134 | 0.830602 | 0.262702 | 0.977088 | 0.529182 | 0.357589 | 1.186294 | 0.939858 |
| Ppwd1 | 2.625768 | 3.502319 | 2.268894 | 3.088358 | 3.162366 | 3.200724 | 3.24807 | 3.632934 |
| Olfml1 | 4.52545 | 3.982482 | 3.87512 | 6.652181 | 6.524405 | 6.188063 | 6.549589 | 5.873442 |
| Tnfrsf26 | 3.935783 | 3.396472 | 4.105955 | 3.729117 | 4.135487 | 3.72385 | 4.92559 | 4.473926 |
| Rnf220 | 16.02186 | 15.70906 | 16.0661 | 15.37488 | 13.84342 | 14.65127 | 18.69431 | 16.59684 |
| Snx15 | 7.299492 | 5.924958 | 8.551975 | 7.486547 | 7.549668 | 7.457626 | 9.338041 | 8.579653 |
| LOC100910 | 0.711923 | 0.358003 | 0.084922 | 0.43734 | 0.384896 | 0.450822 | 0.619475 | 0.595957 |
| Rmi2 | 0.93095 | 0.658329 | 0.374788 | 0.321688 | 0.377483 | 0.204064 | 0.781129 | 1.23772 |
| AC095278. | 0 | 0 | 0 | 0.252634 | 0 | 0 | 0 | 0 |
| Ccdc12 | 20.54702 | 20.11425 | 20.53726 | 19.70485 | 20.82427 | 21.40417 | 21.69002 | 20.38128 |
| Cilp | 1.598378 | 2.052493 | 1.865767 | 7.644755 | 8.518067 | 8.208549 | 6.116761 | 7.286 |
| LOC691153 | 2.23786 | 3.739618 | 4.324474 | 2.696592 | 2.23363 | 2.052719 | 3.5821 | 3.234674 |
| Olr219 | 0 | 0 | 0 | 0 | 0 | 0 | 0 | 0 |
| Zbtb44 | 5.494552 | 4.582356 | 4.320213 | 4.421223 | 4.546537 | 4.591449 | 5.425575 | 5.258788 |
| Ceacam1 | 1.488437 | 1.803174 | 1.404243 | 1.413098 | 1.219262 | 1.278698 | 1.093311 | 1.37258 |
| Kars | 50.75241 | 50.31512 | 52.36005 | 50.48332 | 48.05776 | 48.42242 | 50.89919 | 52.98016 |
| AABR07043 | 1.068692 | 0 | 0 | 0.984758 | 0.577781 | 0.468515 | 1.195605 | 0.947235 |
| Lgals2 | 0.086128 | 0.29235 | 0.184928 | 0 | 0.093129 | 0 | 0.096356 | 0.229019 |
| LOC686066 | 0 | 0 | 0.267706 | 0 | 0 | 0 | 0 | 0 |
| Rimbp3 | 0.10789 | 0.077683 | 0.115827 | 0.063265 | 0.106055 | 0.085998 | 0.065838 | 0.147789 |
| Cib4 | 0 | 0 | 0 | 0 | 0 | 0.05812 | 0 | 0 |
| Rab11fip5 | 21.75971 | 24.94719 | 26.25721 | 21.15182 | 20.04816 | 22.37423 | 31.56715 | 29.58776 |
| AABR07051 | 0 | 0 | 0 | 0 | 0 | 0 | 0 | 0 |
| Nudt8 | 200.8971 | 216.9569 | 215.5375 | 188.027 | 179.827 | 184.2792 | 171.3583 | 163.0503 |
| AABR07069 | 0.030623 | 0.057748 | 0.032876 | 0.028218 | 0.011038 | 0.0179 | 0.04568 | 0.018095 |
| Mut | 41.8779 | 40.76382 | 38.97031 | 39.15866 | 38.9029 | 39.36457 | 34.56412 | 36.40591 |
| E2f4 | 11.04351 | 10.88198 | 11.9394 | 10.43892 | 10.90404 | 11.615 | 14.06612 | 13.55672 |
| Rnf213 | 5.70517 | 5.455528 | 5.610508 | 6.869598 | 6.657582 | 6.748187 | 9.788821 | 10.19186 |
| LOC100911 | 0.05435 | 0.215229 | 0.175044 | 0.125203 | 0.176303 | 0.095308 | 0.060804 | 0.120432 |

| | | | | | | | | |
|---|---|---|---|---|---|---|---|---|
| Cacng4 | 0.81384 | 0.920821 | 0.429693 | 0.540927 | 0.692454 | 0.5732 | 0.820932 | 0.473014 |
| Urgcp | 18.38477 | 19.74986 | 20.87476 | 20.88057 | 20.03999 | 20.03156 | 23.52121 | 22.82006 |
| Olr36 | 0.657313 | 0.892462 | 0.4234 | 0.444171 | 0.379063 | 0.614755 | 0.539273 | 0.62145 |
| Olr1521 | 0 | 0 | 0 | 0 | 0 | 0.049054 | 0 | 0 |
| Pcsk6 | 28.25336 | 29.02486 | 29.35596 | 28.74482 | 26.64589 | 28.02687 | 35.16775 | 37.14952 |
| Procr | 6.091546 | 6.166793 | 6.807369 | 4.037507 | 3.813353 | 2.748622 | 6.257002 | 5.367665 |
| Ube2q1 | 19.05006 | 19.71705 | 20.26469 | 18.54246 | 17.40061 | 18.91494 | 23.35601 | 21.97365 |
| Thsd1 | 4.504433 | 4.755648 | 5.466553 | 4.469939 | 4.43076 | 5.125092 | 6.202289 | 6.075548 |
| Hectd3 | 7.127921 | 6.575649 | 7.326187 | 7.015924 | 7.510256 | 7.315069 | 8.359529 | 7.681908 |
| Dhx34 | 2.511081 | 2.569823 | 2.620081 | 2.612845 | 2.593165 | 2.461472 | 3.156501 | 3.075962 |
| Dusp6 | 8.537509 | 8.455005 | 8.185724 | 9.811821 | 10.03333 | 9.60303 | 9.870474 | 9.977267 |
| Pkd2 | 8.614307 | 8.281017 | 8.33248 | 11.43911 | 11.10039 | 11.21597 | 15.18057 | 15.51935 |
| Nabp1 | 4.758448 | 5.331942 | 4.417494 | 8.091614 | 8.997959 | 8.243646 | 8.975438 | 8.6798 |
| Adar | 16.32807 | 17.60409 | 17.7162 | 15.99522 | 16.4312 | 16.47984 | 18.38078 | 17.50836 |
| LOC103692 | 2.867055 | 12.68952 | 5.839084 | 5.478013 | 2.279489 | 2.107187 | 3.018856 | 3.363371 |
| Letmd1 | 9.005604 | 9.088709 | 10.59312 | 10.50095 | 9.827793 | 9.992006 | 12.00302 | 12.68762 |
| Foxp1 | 4.089735 | 4.798726 | 4.204747 | 4.466406 | 3.884024 | 3.984315 | 5.567964 | 5.312736 |
| AC115401. | 2.111529 | 1.433457 | 0.906745 | 0.778276 | 4.109698 | 1.481112 | 1.417371 | 0.748621 |
| Rhpn1 | 0.207801 | 0.047023 | 0.089235 | 0.421258 | 0.31457 | 0.1822 | 0.418462 | 0.184185 |
| Arl15 | 6.118355 | 4.377546 | 5.505902 | 4.97455 | 3.502423 | 3.865653 | 3.925777 | 5.582502 |
| Cxcr1 | 0 | 0 | 0 | 0 | 0 | 0 | 0 | 0.036404 |
| Polrmt | 9.060912 | 9.619157 | 12.29444 | 10.15313 | 8.814666 | 9.734607 | 10.9504 | 11.50138 |
| Rn60_17_( | 0.375652 | 0.510039 | 0.725916 | 0.692297 | 0.812375 | 0.922242 | 1.344842 | 1.198653 |
| Mcm9 | 1.020895 | 1.223042 | 1.068366 | 1.059292 | 1.020393 | 1.128305 | 1.286098 | 1.444751 |
| Nid1 | 33.53845 | 33.61184 | 33.85519 | 34.26574 | 35.25121 | 34.86891 | 45.71434 | 43.564 |
| Elmod3 | 9.491493 | 10.30961 | 9.636567 | 6.522045 | 6.128485 | 6.75283 | 8.494928 | 8.268562 |
| Ccs | 16.05888 | 17.01526 | 16.7213 | 19.10329 | 20.03566 | 21.09712 | 20.42952 | 18.66102 |
| AABR07012 | 0 | 0 | 0 | 0 | 0 | 0 | 0 | 0 |
| Cbx7 | 11.691 | 11.55465 | 12.58839 | 11.94305 | 11.71954 | 10.89134 | 13.81897 | 14.66452 |
| Tfb1m | 5.880842 | 4.485772 | 4.185327 | 5.906062 | 5.572941 | 5.648785 | 5.987833 | 5.271049 |
| LOC103689 | 13.11971 | 4.650031 | 6.303028 | 12.9216 | 9.864379 | 6.533756 | 14.19775 | 12.22909 |
| Rn50_10_( | 0.1639 | 0.278167 | 0.087978 | 0.226541 | 0.177222 | 0.215561 | 0.275045 | 0.217909 |
| Pex16 | 22.57636 | 23.15927 | 24.84058 | 19.4221 | 19.65075 | 23.409 | 21.58934 | 22.04482 |
| Rps6kc1 | 3.762578 | 3.601056 | 3.691896 | 3.591336 | 3.601793 | 3.913905 | 4.677117 | 4.661772 |
| LOC685963 | 4.671139 | 5.841507 | 4.22296 | 2.718486 | 5.848335 | 3.448975 | 4.950817 | 3.922354 |
| Hmgb2l1 | 1.571805 | 0.842411 | 0.355249 | 0.609834 | 0.089451 | 0.797882 | 0.740407 | 3.152961 |
| AABR07063 | 0 | 0 | 0.432447 | 0 | 0 | 0 | 0 | 0 |
| Wnt8b | 0 | 0 | 0.077223 | 0.033141 | 0 | 0.031535 | 0.040237 | 0.031878 |
| RGD130959 | 6.010646 | 5.540134 | 7.334172 | 5.835766 | 5.865154 | 5.372972 | 5.805983 | 5.821306 |
| Ntpcr | 24.10168 | 23.02535 | 26.04021 | 22.01934 | 21.61546 | 22.34895 | 20.58225 | 21.04368 |
| #N/A | 0 | 0 | 0 | 0 | 0 | 0.49907 | 0 | 0 |
| AABR07059 | 4.344432 | 2.545537 | 3.581305 | 4.003223 | 4.02649 | 2.992944 | 2.777347 | 3.438112 |
| AABR07065 | 1.786764 | 3.265725 | 2.951085 | 1.646432 | 1.932002 | 2.169188 | 2.767779 | 2.436458 |
| AABR07043 | 0 | 0 | 0 | 0 | 0 | 0.173919 | 0 | 0 |
| Magee1 | 0.630915 | 0.581041 | 0.756086 | 0.567843 | 0.666334 | 0.64324 | 0.64018 | 0.611231 |
| Tppp3 | 29.42568 | 28.6848 | 27.56244 | 34.27602 | 32.45507 | 33.22573 | 38.31689 | 33.92013 |
| Erich6b | 0 | 0.085869 | 0 | 0 | 0.082062 | 0.066543 | 0.254716 | 0.067267 |
| Mir195 | 0 | 0 | 0 | 0 | 0.650833 | 0 | 0 | 0 |
| Rn60_1_21 | 0.432776 | 0 | 0.116153 | 0.099697 | 0.350966 | 0.094865 | 0.242085 | 0.191796 |
| Cysltr1 | 1.711761 | 1.225308 | 0.862587 | 1.351989 | 1.737582 | 1.837805 | 1.836877 | 2.446129 |
| Tmem150b | 0.571859 | 0.492976 | 0.7601 | 0.627316 | 0.853902 | 0.692418 | 0.670235 | 0.651686 |
| AABR07040 | 0 | 0 | 0 | 0.106519 | 0 | 0 | 0 | 0 |
| Pclo | 0.027946 | 0.00527 | 0.015001 | 0.021459 | 0.005036 | 0.012251 | 0.010422 | 0.008257 |
| Cbl | 4.095659 | 3.368444 | 3.565595 | 2.854277 | 3.274914 | 2.821561 | 3.657932 | 4.255554 |

| | | | | | | | | |
|---|---|---|---|---|---|---|---|---|
| Pask | 0.147286 | 0.179466 | 0.26759 | 0.167038 | 0.122507 | 0.327819 | 0.291529 | 0.180758 |
| Man2c1 | 16.8006 | 17.00716 | 17.28717 | 17.8743 | 17.56948 | 19.31369 | 18.142 | 19.73984 |
| Rcsd1 | 29.73496 | 27.4386 | 32.1708 | 26.1842 | 24.27037 | 26.11222 | 28.08854 | 29.20648 |
| AABR07037 | 3.112958 | 2.991428 | 3.43352 | 2.78988 | 2.674353 | 2.505106 | 2.91015 | 2.87256 |
| Unc5c1 | 0.304256 | 0.497253 | 0.344785 | 0.342663 | 0.25588 | 0.400159 | 0.586225 | 0.569319 |
| Ranbp1 | 23.79364 | 18.52957 | 22.76515 | 24.53938 | 23.30565 | 21.77883 | 23.16656 | 21.53069 |
| Hlcs | 3.116357 | 3.316129 | 3.425265 | 2.324629 | 2.507196 | 2.325813 | 3.341173 | 3.041688 |
| Megf9 | 1.668011 | 1.959863 | 1.498003 | 1.596122 | 1.474093 | 1.701578 | 2.38645 | 2.217662 |
| AC113756. | 1.762315 | 1.566696 | 1.891957 | 0.927945 | 1.633342 | 1.324456 | 1.267457 | 0.892587 |
| Tex40 | 2.523659 | 1.998781 | 2.370645 | 2.092907 | 1.364398 | 1.991472 | 1.341095 | 2.125002 |
| LOC102552 | 0.469096 | 0.307282 | 0.185538 | 0.455004 | 0.293658 | 1.255559 | 0.165727 | 0.350133 |
| Slc22a13 | 0.078549 | 0.177749 | 0.084327 | 0.193013 | 0.22649 | 0.114786 | 0.439385 | 0.185658 |
| Chd1 | 4.849072 | 4.396084 | 3.931568 | 4.393447 | 3.872094 | 4.198302 | 4.948259 | 4.828477 |
| Ddx20 | 3.589524 | 3.384481 | 3.171178 | 4.806364 | 4.265387 | 4.753731 | 3.848475 | 4.109532 |
| Rundc3b | 0.135513 | 0.240941 | 0.103915 | 0.196223 | 0.08373 | 0.135791 | 0.064974 | 0.120112 |
| Oosp2 | 0 | 0 | 0 | 0 | 0 | 0 | 0 | 0 |
| Gpr62 | 0 | 0.055014 | 0 | 0 | 0 | 0 | 0 | 0 |
| Adck5 | 3.700772 | 3.977887 | 4.9911 | 4.43315 | 4.37674 | 5.374266 | 6.055129 | 4.530746 |
| Rnf6 | 14.44238 | 13.962 | 14.09146 | 14.06855 | 13.2792 | 13.69682 | 15.41008 | 15.74436 |
| Ndufa2 | 307.6498 | 307.0398 | 301.9718 | 266.0816 | 266.2269 | 245.3145 | 252.751 | 246.8258 |
| Trim17 | 3.282028 | 3.71346 | 3.366169 | 3.96934 | 3.770609 | 3.083233 | 4.786436 | 3.947961 |
| Nkx1-2 | 0 | 0 | 0.06124 | 0 | 0.06168 | 0 | 0 | 0 |
| Polr3e | 5.886159 | 6.127908 | 6.319168 | 6.162023 | 6.364619 | 6.48941 | 6.468277 | 6.930535 |
| Prdx1 | 152.9745 | 154.0222 | 161.1292 | 185.5222 | 188.1472 | 179.4229 | 167.4507 | 172.71 |
| Hrh4 | 0 | 0.03717 | 0 | 0 | 0 | 0 | 0.036753 | 0.029118 |
| AABR07056 | 0 | 0.103947 | 0 | 0.084655 | 0 | 0 | 0 | 0 |
| Ccdc86 | 7.464185 | 8.093483 | 9.140147 | 8.275037 | 9.9205 | 9.816906 | 7.828686 | 8.269847 |
| LOC100909 | 0.795432 | 0.149999 | 0.498136 | 0.732959 | 0.215022 | 0.464957 | 0.815736 | 0.411268 |
| Abcg1 | 5.604108 | 5.743464 | 5.01364 | 6.511086 | 5.269253 | 6.355747 | 7.519009 | 7.396808 |
| Rce1 | 3.483489 | 4.669706 | 4.30884 | 6.001113 | 5.690911 | 5.311871 | 6.820051 | 5.973814 |
| Fam173b | 18.58268 | 16.72575 | 15.50283 | 17.82355 | 17.13468 | 17.39286 | 16.66559 | 16.63917 |
| Fam3b | 0.44005 | 0.373422 | 0.236211 | 0.608233 | 0.713729 | 0.64306 | 0.328205 | 0.455044 |
| AABR07006 | 0 | 0 | 0 | 0 | 0.152211 | 0.123426 | 0 | 0 |
| LOC100363 | 1.402659 | 1.692845 | 0.602337 | 2.498823 | 2.022233 | 2.213734 | 1.569232 | 1.823427 |
| Ssbp4 | 20.64734 | 22.2337 | 29.12185 | 28.34175 | 24.05783 | 26.03173 | 33.45419 | 33.7302 |
| Krt8 | 0.222644 | 0.151147 | 0.334632 | 0.656505 | 0.962968 | 1.093202 | 1.145789 | 0.434149 |
| Tirap | 3.546696 | 4.091604 | 4.330217 | 3.075897 | 2.782248 | 3.780476 | 4.279092 | 3.636728 |
| AC133821. | 0 | 0 | 0 | 0 | 0 | 0 | 0 | 0 |
| Plekhf2 | 5.510269 | 5.652713 | 5.107157 | 5.077496 | 5.560962 | 5.234026 | 5.774216 | 5.681749 |
| LOC100909 | 9.495755 | 9.712576 | 10.22148 | 8.469965 | 7.173645 | 9.369397 | 10.36847 | 11.71585 |
| Trim35 | 39.97997 | 42.23588 | 42.56911 | 41.27915 | 39.73131 | 40.1783 | 41.82569 | 42.52348 |
| Ddx58 | 4.903832 | 5.911747 | 4.575168 | 5.836639 | 5.744313 | 5.912066 | 7.641478 | 9.624415 |
| Asnsd1 | 15.66313 | 14.02768 | 12.44021 | 13.14349 | 14.441 | 13.17378 | 12.16737 | 12.77324 |
| Ptch2 | 0.064226 | 0.121115 | 0.080443 | 0.118364 | 0.12732 | 0.065699 | 0.155683 | 0.142318 |
| Tab2 | 18.88194 | 17.66093 | 17.93981 | 16.19554 | 16.38446 | 15.05189 | 18.80061 | 18.60486 |
| Atp6v0d1 | 30.85612 | 28.91229 | 28.6428 | 34.72393 | 28.52628 | 31.8786 | 32.85191 | 35.5453 |
| AC127784. | 0 | 0 | 0 | 0 | 0 | 0 | 0 | 0 |
| AABR07048 | 12.87964 | 12.4635 | 15.10067 | 12.9612 | 12.55224 | 13.67033 | 12.98722 | 14.64536 |
| AABR07065 | 0 | 1.173259 | 0 | 0 | 0 | 0 | 0 | 0 |
| Rsl24d1 | 15.588 | 14.33013 | 16.47323 | 16.89661 | 17.19368 | 16.01668 | 16.85526 | 15.66683 |
| Lsm12 | 24.37805 | 22.06609 | 21.71722 | 20.95145 | 20.78355 | 21.97072 | 22.55274 | 22.50086 |
| Mafb | 5.318842 | 5.335564 | 6.740293 | 7.402183 | 6.492319 | 6.754958 | 10.21398 | 9.331505 |
| Zfp46 | 6.608274 | 6.555496 | 6.357486 | 6.367999 | 5.623253 | 6.028763 | 5.622878 | 5.238519 |
| AABR07053 | 0 | 0 | 0 | 0 | 0 | 0 | 0 | 0.083932 |

| | | | | | | | | |
|---|---|---|---|---|---|---|---|---|
| C4a | 2.425216 | 2.473149 | 2.581275 | 16.47762 | 13.76453 | 13.9085 | 20.85572 | 19.20792 |
| Cds1 | 0.770087 | 0.630954 | 0.342098 | 0.415975 | 0.287132 | 0.325965 | 0.445624 | 0.329515 |
| Ddx54 | 7.632671 | 8.308059 | 8.643629 | 8.071865 | 7.800395 | 7.751246 | 9.962276 | 9.876644 |
| Rundc1 | 8.075913 | 7.662689 | 8.578745 | 7.441636 | 8.15021 | 8.969226 | 9.351989 | 8.865977 |
| Hck | 1.626113 | 1.954862 | 1.418411 | 2.083711 | 2.417652 | 2.004999 | 2.984663 | 3.378058 |
| Zcchc17 | 9.502121 | 9.706415 | 9.689478 | 12.70831 | 13.07664 | 10.96933 | 10.33063 | 10.87758 |
| Nsg2 | 0.370077 | 0.444895 | 0.347639 | 0.319698 | 0.400159 | 0.385325 | 0.284643 | 0.205011 |
| Agtr1a | 1.175415 | 0.895665 | 0.927097 | 1.702012 | 2.075035 | 1.240932 | 1.127144 | 1.275708 |
| AABR0700( | 0 | 0 | 0.110394 | 0 | 0 | 0 | 0 | 0 |
| AC108312. | 0.297534 | 0.112215 | 0 | 0 | 0 | 0 | 0.332867 | 0 |
| Chrdl2 | 0.809115 | 0.476047 | 0.729655 | 0.447341 | 0.349954 | 0.340528 | 0.543121 | 0.286864 |
| Pitx1 | 0 | 0 | 0 | 0 | 0 | 0 | 0 | 0 |
| Slc6a17 | 14.2148 | 15.11951 | 15.69087 | 10.22012 | 10.36725 | 10.3985 | 14.93115 | 13.97617 |
| Arsb | 9.533711 | 9.104433 | 9.697931 | 8.708103 | 7.964419 | 7.6646 | 10.5726 | 9.977643 |
| Smpdl3a | 14.83995 | 15.494 | 13.81793 | 15.73236 | 14.71169 | 13.2951 | 13.3147 | 13.7264 |
| LOC36198 | 54.63111 | 53.06832 | 50.53197 | 60.56198 | 57.19882 | 56.19559 | 57.05669 | 57.13237 |
| Adk | 36.60475 | 36.40909 | 33.96386 | 39.70228 | 40.62989 | 41.40816 | 36.47966 | 37.68332 |
| Tnnc2 | 0.208907 | 0 | 0 | 0.128333 | 0.150592 | 0 | 0 | 0.061721 |
| Nle1 | 5.565749 | 6.297383 | 5.782439 | 6.176401 | 6.05052 | 6.375554 | 6.193236 | 5.861491 |
| AABR0703 | 0.82104 | 0.315849 | 0.740409 | 0.696031 | 0.514912 | 0.503922 | 0.330675 | 0.785947 |
| Impad1 | 0.992557 | 0.580878 | 0.698134 | 0.78845 | 0.666147 | 2.79088 | 0.574359 | 0.879752 |
| AABR0703 | 0.344891 | 0.195114 | 0.06171 | 0 | 0 | 0.1512 | 0.128616 | 0 |
| Prph | 2.449518 | 2.737716 | 2.822132 | 5.973149 | 6.49237 | 6.364643 | 7.486004 | 6.116231 |
| Myo16 | 0.248134 | 0.313147 | 0.297125 | 0.04397 | 0.103194 | 0.083679 | 0.074739 | 0.126885 |
| Mfsd9 | 0.5672 | 0.755012 | 0.644745 | 0.891584 | 0.90192 | 0.804491 | 0.597231 | 0.547097 |
| Clca4l | 0 | 0 | 0 | 0 | 0 | 0 | 0.020307 | 0 |
| Apobr | 3.058986 | 2.749914 | 2.789165 | 2.702896 | 2.476952 | 2.486167 | 3.484764 | 3.800815 |
| Olfml | 0.791768 | 1.173871 | 0.937946 | 1.358535 | 1.387517 | 1.340569 | 2.015948 | 1.645562 |
| Klhl41 | 32.3887 | 31.86184 | 30.99238 | 41.85027 | 45.22881 | 43.9304 | 33.95916 | 36.89255 |
| AC093965. | 0 | 0.470235 | 0.223088 | 0 | 0 | 0 | 0.232479 | 0 |
| Kcnh2 | 19.75897 | 20.46708 | 23.03833 | 14.40328 | 12.95614 | 13.97633 | 14.30455 | 14.85484 |
| Mdk | 6.899809 | 6.98504 | 8.732918 | 24.56158 | 26.07306 | 25.98212 | 17.71353 | 20.14944 |
| Rgs10 | 8.094613 | 10.35635 | 9.960174 | 11.1883 | 14.07147 | 16.54232 | 13.44452 | 15.06678 |
| AABR0705 | 0.530736 | 0.667225 | 0.379852 | 0.869426 | 0.255056 | 0.258528 | 0.065974 | 0.574955 |
| Gpihbp1 | 61.43417 | 69.07633 | 66.43341 | 36.48408 | 40.67154 | 38.46738 | 42.79538 | 44.3202 |
| Ppp4r4 | 0.713473 | 0.48772 | 0.510639 | 0.465685 | 0.562529 | 0.625573 | 0.764943 | 0.711435 |
| Mybpc2 | 0.501041 | 0.518312 | 0.599374 | 1.87314 | 2.012282 | 2.121254 | 1.921859 | 2.44888 |
| Deaf1 | 9.855662 | 10.87174 | 11.08452 | 10.21965 | 9.508309 | 9.269528 | 11.35769 | 11.7131 |
| LOC10255 | 0 | 0 | 0 | 0 | 0 | 0 | 0 | 0.071297 |
| Zfp277 | 4.603258 | 3.880061 | 4.477553 | 5.181163 | 4.910625 | 4.659169 | 4.942541 | 4.381311 |
| LOC683961 | 2.909218 | 3.544846 | 3.003107 | 2.68073 | 1.693836 | 3.433776 | 2.253256 | 3.471171 |
| AABR07021 | 0 | 0 | 0 | 0 | 0 | 0 | 0 | 0 |
| Rec8 | 0.31001 | 0.204611 | 0.055469 | 0.142831 | 0.055868 | 0.407726 | 0.144511 | 0.114491 |
| Map3k4 | 5.542713 | 5.76501 | 5.371788 | 4.563853 | 5.080521 | 4.895524 | 5.017642 | 5.147153 |
| Camk1g | 0 | 0 | 0 | 0.066925 | 0.078533 | 0.063682 | 0 | 0.042917 |
| AABR0705 | 0 | 0 | 0 | 0 | 0 | 0 | 0 | 0.338792 |
| Cgref1 | 0.314746 | 0.489666 | 0.16895 | 2.610237 | 2.509939 | 2.552721 | 1.892668 | 2.162059 |
| Bsg | 371.0982 | 382.5557 | 406.213 | 414.4131 | 394.5323 | 375.8574 | 463.6412 | 456.4146 |
| Atxn1 | 6.249381 | 5.557657 | 6.147842 | 4.380538 | 4.036014 | 4.488042 | 6.216204 | 6.541354 |
| Cwc27 | 4.288963 | 4.164193 | 3.857804 | 3.498152 | 2.976834 | 3.96384 | 3.306937 | 3.441918 |
| AC098214. | 0.489401 | 1.661203 | 0.525403 | 0 | 0 | 0 | 0 | 0 |
| Fam104b | 14.33829 | 10.93296 | 9.202378 | 11.34523 | 13.31303 | 10.52207 | 13.60001 | 12.5706 |
| AABR0705 | 0.368774 | 0 | 0 | 0 | 0 | 0 | 0 | 0 |
| 3100002H( | 0 | 0 | 0 | 0 | 0 | 0 | 0 | 0 |

| | | | | | | | | |
|---|---|---|---|---|---|---|---|---|
| Lrrn4cl | 0.299738 | 0.379038 | 0.227144 | 0.438665 | 0.743528 | 0.726593 | 0.907372 | 0.812645 |
| Arglu1 | 56.42485 | 53.75153 | 51.00141 | 57.52448 | 57.85881 | 55.68886 | 59.09632 | 57.10607 |
| LOC103694 | 5.333566 | 4.754596 | 7.981591 | 5.21253 | 5.767108 | 5.951878 | 26.39926 | 4.58415 |
| Ptma | 23.85862 | 23.12745 | 21.21025 | 30.23695 | 28.0895 | 23.25695 | 29.59826 | 28.84244 |
| Gal3st3 | 3.695853 | 3.828685 | 3.967735 | 3.383469 | 2.543083 | 2.840722 | 3.893115 | 3.679978 |
| Dact2 | 0.317709 | 0.338327 | 0.401272 | 1.0677 | 0.868939 | 0.999566 | 1.651745 | 1.408006 |
| Lrrc27 | 13.45473 | 13.77668 | 12.16708 | 11.13946 | 9.992208 | 9.172705 | 10.98869 | 9.865017 |
| Palmd | 16.195 | 16.684 | 16.03334 | 16.37471 | 16.52342 | 19.25535 | 19.38722 | 19.74432 |
| Pcif1 | 8.848353 | 9.74333 | 9.541679 | 8.602332 | 8.699109 | 8.785749 | 10.60623 | 9.698382 |
| Zfp862 | 0.364312 | 0.698951 | 0.595171 | 0.773568 | 0.753597 | 0.763853 | 1.027801 | 0.912566 |
| Heph | 3.855459 | 3.023219 | 3.536557 | 2.552065 | 2.71767 | 2.695818 | 3.767327 | 3.601126 |
| AABR0703( | 1.829992 | 1.592731 | 2.115737 | 0.907989 | 1.978744 | 1.481112 | 1.574857 | 1.372472 |
| Stc2 | 14.63017 | 13.58516 | 13.4317 | 6.632094 | 6.545955 | 7.047888 | 8.202606 | 7.750658 |
| Slirp | 11.69264 | 10.74779 | 10.31764 | 9.421095 | 9.427035 | 9.160078 | 9.171401 | 8.897352 |
| AABR0707 | 0 | 0.161884 | 0 | 0 | 0 | 0 | 0 | 0 |
| Mcc | 3.015312 | 3.025026 | 3.625586 | 3.574992 | 2.608331 | 2.643828 | 3.305929 | 2.494446 |
| Fbxw12 | 0 | 0 | 0 | 0 | 0.049976 | 0.081049 | 0 | 0 |
| Atad2b | 4.839435 | 5.009585 | 4.247945 | 4.418686 | 4.692034 | 4.772011 | 5.907836 | 5.345492 |
| Tm6sf2 | 0.090027 | 0.101862 | 0.1933 | 0.359479 | 0.324484 | 0.210496 | 0.436447 | 0.265986 |
| Mrgpre | 0 | 0 | 0.120899 | 0.20754 | 0.304422 | 0.444334 | 0.251977 | 0.399265 |
| AABR0703 | 13.96425 | 14.26827 | 13.57649 | 15.29657 | 16.79416 | 15.58594 | 17.89423 | 18.31321 |
| Tacr3 | 0.048509 | 0 | 0.026039 | 0.111749 | 0.052453 | 0.042533 | 0.135675 | 0.021498 |
| AABR0700 | 0 | 0 | 1.050807 | 0.901928 | 0 | 0 | 0 | 0 |
| Tgfbr3 | 15.62748 | 16.75062 | 16.3028 | 12.90741 | 12.61713 | 13.36799 | 17.63839 | 17.01482 |
| Dusp2 | 1.226066 | 1.460248 | 1.454814 | 1.010848 | 0.976852 | 0.763827 | 1.624344 | 1.115321 |
| Idnk | 0.92373 | 0.937037 | 0.991683 | 1.203393 | 1.239909 | 1.424354 | 1.24724 | 1.637495 |
| NEWGENE_1 | 0.029092 | 0 | 0 | 0 | 0 | 0 | 0 | 0 |
| AABR0700 | 0 | 0 | 0 | 0 | 0.086579 | 0.210617 | 0.089579 | 0.354851 |
| Ubd | 2.154059 | 2.017007 | 2.631488 | 2.532434 | 3.051994 | 3.060966 | 2.160569 | 1.975086 |
| Gltpd2 | 0.171411 | 0.145457 | 0.299033 | 0.078974 | 0.208512 | 0.244226 | 0.215737 | 0.284868 |
| Gabra1 | 0.045794 | 0.051814 | 0.036872 | 0 | 0.012379 | 0.030114 | 0 | 0.020295 |
| Rnf103 | 7.533411 | 7.011784 | 6.715409 | 8.048138 | 8.606454 | 7.369411 | 8.319716 | 8.805713 |
| Acvr2b | 1.431881 | 1.289472 | 1.537215 | 1.373276 | 1.421882 | 0.999255 | 1.634617 | 1.036038 |
| Cep72 | 0.338312 | 0.519493 | 0.311314 | 0.423078 | 0.627107 | 0.254256 | 0.216279 | 0.492632 |
| Rn50_2_22 | 0.137443 | 0.155511 | 2.655976 | 0 | 0 | 0.241021 | 0.153766 | 0.121823 |
| Mboat2 | 0.239872 | 0.250527 | 0.316945 | 0.527078 | 0.359128 | 0.42064 | 0.433502 | 0.425221 |
| Etv3l | 0 | 0 | 0.032198 | 0.055273 | 0.03243 | 0.078891 | 0 | 0.026583 |
| #N/A | 0.17113 | 0.193626 | 0.183719 | 0 | 0 | 0 | 0 | 0 |
| Insrr | 0 | 0 | 0.033751 | 0 | 0 | 0.018377 | 0 | 0.009288 |
| Fabp3 | 3392.049 | 3460.16 | 3445.002 | 2466.918 | 2447.013 | 2543.049 | 2372.333 | 2381.436 |
| Smg8 | 1.848209 | 1.568371 | 1.69857 | 1.69015 | 1.57453 | 1.780107 | 2.083358 | 2.295905 |
| Trh | 0.263524 | 0.511139 | 0.161663 | 4.474949 | 4.721935 | 5.11628 | 6.738711 | 6.973856 |
| Xiap | 9.860963 | 8.328254 | 8.50422 | 8.182147 | 8.262187 | 6.802146 | 10.97972 | 12.11624 |
| Slc35a3 | 5.331803 | 4.493734 | 4.468249 | 4.236249 | 4.529801 | 4.937298 | 5.41718 | 5.256293 |
| Dgkh | 2.163989 | 2.5222 | 2.043279 | 1.921957 | 1.761965 | 1.577346 | 1.998033 | 2.65754 |
| Nsf | 5.068797 | 5.833987 | 6.454956 | 5.975271 | 5.329622 | 5.547745 | 5.064562 | 6.630645 |
| Egr3 | 1.553466 | 1.502012 | 1.667745 | 4.216293 | 3.512184 | 3.789059 | 4.297473 | 4.38109 |
| LOC317588 | 0 | 0 | 0 | 0 | 0 | 0 | 0 | 0 |
| Tshb | 0.280532 | 0.529014 | 0.301169 | 0.086166 | 0.101112 | 0 | 0.732308 | 0.414415 |
| Dlg4 | 3.824691 | 3.509332 | 3.125501 | 4.541265 | 4.238458 | 4.504692 | 6.27997 | 6.358384 |
| AABR0706( | 0.425739 | 1.445112 | 0.457058 | 1.176906 | 0.460346 | 2.613019 | 0.952596 | 0.754708 |
| Zfp839 | 1.471176 | 1.327155 | 1.643431 | 1.868572 | 1.418787 | 1.499106 | 2.090721 | 1.88548 |
| Lmcd1 | 50.31545 | 53.14616 | 51.5446 | 62.38026 | 57.71403 | 62.39945 | 62.21974 | 58.99677 |
| Sec22b | 13.85283 | 13.78288 | 12.63912 | 15.16038 | 17.02691 | 17.16095 | 20.06836 | 20.67257 |

| | | | | | | | | |
|---|---|---|---|---|---|---|---|---|
| #N/A | 2.073898 | 0 | 0.556615 | 0.477754 | 0 | 0 | 0 | 0 |
| Slc7a5 | 1.33262 | 0.908105 | 1.219307 | 1.33959 | 1.490066 | 1.672997 | 2.066897 | 2.201267 |
| AABR07032 | 0.185695 | 0.630315 | 0.598066 | 0 | 0 | 0 | 0 | 0 |
| Timd2 | 0 | 0 | 0 | 0.080691 | 0 | 0.051187 | 0.032656 | 0.077616 |
| Rln3 | 0.445667 | 0.756378 | 0.478452 | 0.102666 | 0.36142 | 0 | 0.124648 | 0.197509 |
| AABR07071 | 0.534346 | 0 | 1.147309 | 0 | 0 | 0 | 0 | 0 |
| Csnk2a2 | 7.865237 | 7.767237 | 7.740182 | 8.709723 | 7.758552 | 8.529568 | 10.26582 | 9.723199 |
| Mad2l1 | 1.25031 | 1.289843 | 1.855515 | 1.389311 | 1.709809 | 1.773383 | 1.974764 | 1.82529 |
| AABR07028 | 0 | 0 | 0.12116 | 0 | 0 | 0.296861 | 0.25252 | 0 |
| Tsga13 | 0 | 0 | 0 | 0.040111 | 0 | 0 | 0.048699 | 0 |
| Hgd | 0.030986 | 0.070118 | 0 | 0.057104 | 0 | 0.027168 | 0.103996 | 0 |
| AABR07071 | 0 | 0.116176 | 0.055116 | 0 | 0 | 0 | 0 | 0 |
| RGD156255 | 0.570803 | 0.717597 | 0.453921 | 0.603895 | 0.731498 | 0.778526 | 0.80415 | 0.374764 |
| LOC690326 | 0.525411 | 0.792636 | 0.564062 | 0.403454 | 0.284059 | 0.46068 | 0 | 0.388081 |
| RGD156187 | 0 | 0 | 0 | 0 | 0 | 0 | 0 | 0 |
| AABR07032 | 0 | 0 | 0 | 0 | 0 | 0 | 0 | 0 |
| LOC100909 | 0 | 0 | 0 | 0 | 0 | 0 | 0 | 0 |
| Fv1 | 4.887091 | 5.060343 | 4.419867 | 7.505437 | 7.334025 | 6.310645 | 7.853261 | 6.641897 |
| Zbtb25 | 2.769232 | 2.342885 | 1.955181 | 2.597715 | 2.212028 | 2.231195 | 2.372414 | 2.498733 |
| Raf1 | 23.6623 | 24.94508 | 22.37292 | 25.27156 | 23.81984 | 26.22573 | 24.00978 | 25.40998 |
| AABR07071 | 0.447572 | 0 | 0 | 0 | 0 | 0 | 0.500724 | 0 |
| AC121443. | 0 | 0.617183 | 0 | 0.502637 | 0 | 0.478276 | 0 | 0 |
| AC142138. | 0.471765 | 0 | 0 | 0 | 0 | 0 | 0 | 0.139383 |
| Inhba | 0.861147 | 0.904751 | 0.858461 | 1.265842 | 1.551909 | 1.294379 | 1.353365 | 1.635594 |
| Uqcrq | 516.0532 | 572.7062 | 589.3123 | 498.2282 | 494.2152 | 440.7975 | 527.9286 | 507.8568 |
| Copa | 22.8392 | 23.10092 | 23.1494 | 25.52016 | 24.14633 | 25.39179 | 26.72793 | 27.52197 |
| Hmgcr | 3.345094 | 3.000327 | 3.107996 | 3.642803 | 3.827445 | 3.3276 | 4.096164 | 3.85979 |
| Got1l1 | 0 | 0 | 0 | 0 | 0 | 0 | 0 | 0 |
| Papd7 | 4.9363 | 4.654326 | 4.931418 | 4.750767 | 5.070673 | 4.941308 | 5.783299 | 6.125404 |
| Fap | 5.211477 | 5.07955 | 4.819663 | 8.128975 | 7.434251 | 8.285528 | 7.551381 | 7.930538 |
| Csrp3 | 471.84 | 590.0213 | 685.5746 | 715.1764 | 683.6085 | 716.3086 | 869.2551 | 666.4414 |
| AC108261. | 0.811875 | 0.459299 | 0 | 0.748111 | 0 | 1.067779 | 0.454145 | 0.719605 |
| Npb | 0.290922 | 0.329164 | 0.312323 | 0.268073 | 0.31457 | 0.12754 | 0.488206 | 0.257858 |
| Shroom3 | 12.3329 | 13.51922 | 15.30327 | 11.53204 | 10.81267 | 11.09728 | 14.04644 | 13.78211 |
| Ndfip2 | 9.753463 | 10.69862 | 11.85644 | 11.96037 | 10.49261 | 11.42422 | 12.10565 | 12.53852 |
| Adipor2 | 17.50363 | 17.30334 | 16.65147 | 12.92316 | 15.39976 | 13.75846 | 15.24418 | 16.83129 |
| Cpa1 | 0.032067 | 0 | 0 | 0 | 0 | 0 | 0 | 0.028423 |
| AABR07049 | 0 | 0 | 0 | 0 | 0 | 0.072079 | 0 | 0 |
| Zmym4 | 2.901219 | 2.777578 | 2.820955 | 2.713161 | 2.623287 | 2.587976 | 2.681976 | 2.769302 |
| Siva1 | 5.837654 | 7.045367 | 5.849288 | 6.694077 | 7.107037 | 7.924135 | 5.950383 | 5.480823 |
| Sucla2 | 211.8417 | 207.0337 | 192.9745 | 206.1368 | 204.7018 | 196.7607 | 187.8798 | 194.6437 |
| Fam216b | 0 | 0 | 0.051958 | 0 | 0 | 0 | 0 | 0 |
| AC105645. | 1.13839 | 0.644017 | 0.611067 | 0.524491 | 0 | 0 | 0 | 0 |
| Cfap97 | 1.929948 | 2.445683 | 1.850917 | 1.896929 | 2.309419 | 2.842862 | 2.072774 | 2.212387 |
| Syk | 1.546589 | 1.435809 | 1.383635 | 1.635235 | 1.372147 | 1.790682 | 2.351372 | 2.460444 |
| Hopx | 44.2941 | 43.03871 | 40.72838 | 62.57102 | 61.15013 | 63.87333 | 69.42114 | 66.4917 |
| Dok7 | 9.317166 | 9.712245 | 10.34988 | 10.59264 | 9.46818 | 10.1738 | 13.97056 | 14.35639 |
| AABR07030 | 0.062564 | 0.035394 | 0.033583 | 0.259425 | 0.372071 | 0.438848 | 0.349968 | 0.24954 |
| AABR07016 | 0 | 0.29699 | 0 | 0 | 0 | 0 | 0.146829 | 0 |
| Rassf7 | 0.07412 | 0.125795 | 0.039786 | 0.068299 | 0.120218 | 0.129977 | 0.041461 | 0 |
| AABR07066 | 0 | 0 | 0 | 0 | 0 | 0 | 0 | 0.096697 |
| Dhx16 | 9.896645 | 9.327861 | 11.33431 | 9.694617 | 8.755445 | 9.723825 | 12.03738 | 11.26187 |
| AABR07028 | 0.232737 | 0 | 0.249858 | 3.002417 | 0 | 0 | 0.260376 | 0 |
| Tmem106c | 13.62487 | 12.66306 | 12.25628 | 12.55478 | 11.98017 | 13.25908 | 14.53101 | 12.0764 |

| | | | | | | | | |
|---|---|---|---|---|---|---|---|---|
| Kcnj4 | 0 | 0 | 0 | 0.018147 | 0 | 0 | 0 | 0.017456 |
| AABR07031 | 0 | 0.411455 | 0.390404 | 0.167546 | 0 | 0 | 0.813676 | 0.322323 |
| AABR07009 | 2.55116 | 2.430752 | 2.594684 | 2.845698 | 1.451859 | 1.530483 | 2.103039 | 2.380232 |
| Gdpd2 | 0 | 0 | 0 | 0.093969 | 0 | 0.089415 | 0 | 0 |
| Ccdc62 | 2.862622 | 2.228048 | 1.918308 | 2.268166 | 2.050397 | 2.270145 | 2.876197 | 2.876666 |
| Tmem59l | 0.035551 | 0.080447 | 0.076332 | 0.131034 | 0.115321 | 0.155854 | 0.159089 | 0.06302 |
| Gpr156 | 0.044023 | 0.024905 | 0.023631 | 0.060849 | 0.047602 | 0 | 0.024626 | 0 |
| LOC100910 | 0 | 0 | 0 | 0.037683 | 0.044219 | 0 | 0.022876 | 0 |
| Josd2 | 38.99721 | 42.65969 | 44.70997 | 32.8689 | 31.97507 | 32.78834 | 39.56188 | 38.76977 |
| Bcl11b | 0.808432 | 0.90182 | 0.85568 | 0.681986 | 0.443229 | 0.638949 | 0.738837 | 0.555077 |
| Col20a1 | 0.410998 | 0.34193 | 0.35039 | 0.378718 | 0.548972 | 0.593539 | 0.540948 | 0.32143 |
| AABR07052 | 0 | 0 | 0.387711 | 0.33278 | 0.781 | 0.316652 | 0 | 0 |
| Esco2-ps2 | 0 | 0 | 0 | 0 | 0 | 0.026388 | 0 | 0.026675 |
| LOC103692 | 0 | 0 | 0 | 0 | 0 | 0 | 0 | 0 |
| AABR07028 | 1.106322 | 0.469407 | 0.643342 | 0.722098 | 0.996875 | 1.131695 | 0.876707 | 1.062304 |
| AABR07054 | 0 | 0 | 0 | 0.251318 | 0 | 0 | 0 | 0 |
| AABR07014 | 0 | 0 | 0 | 0 | 0 | 0.454599 | 0 | 0 |
| Sertm1 | 0.017068 | 0 | 0 | 0 | 0.018456 | 0 | 0 | 0 |
| RT1-CE16 | 1.683605 | 2.167667 | 2.181414 | 2.674786 | 2.573751 | 1.934313 | 3.052638 | 1.749549 |
| Rack1 | 1.021774 | 0.52024 | 5.704087 | 0.423686 | 0.552415 | 0.895892 | 0 | 0.045282 |
| Fam73a | 2.178079 | 2.98504 | 2.634712 | 2.883316 | 2.255613 | 2.017332 | 2.436738 | 1.95773 |
| Hormad1 | 0.03193 | 0.108383 | 0.034279 | 0 | 0 | 0.027997 | 0.071445 | 0.084905 |
| Zfp365 | 0.497243 | 0.48056 | 0.367003 | 0.849561 | 0.8737 | 0.980962 | 1.448681 | 1.404831 |
| Rps28 | 202.6669 | 242.2416 | 234.9886 | 247.2439 | 233.6723 | 242.3039 | 194.4181 | 189.7656 |
| Kif14 | 0.073035 | 0.153466 | 0.10081 | 0.057685 | 0.078971 | 0.073185 | 0.04669 | 0.092478 |
| LOC681182 | 0.0368 | 0 | 0 | 0 | 0 | 0.016133 | 0 | 0.048926 |
| Mex3d | 5.369726 | 6.627919 | 8.647116 | 6.440567 | 5.950162 | 6.264604 | 7.372731 | 7.611194 |
| Rapgef5 | 14.38953 | 14.58854 | 14.29802 | 12.76138 | 11.89636 | 11.89749 | 15.41815 | 15.8764 |
| AABR07004 | 1.84487 | 2.247951 | 2.285291 | 3.007648 | 3.222419 | 2.613019 | 2.540257 | 1.760984 |
| Mtmr3 | 14.57242 | 15.4278 | 14.1297 | 13.90429 | 13.32294 | 14.29752 | 17.50792 | 16.87801 |
| Parp9 | 7.110601 | 7.08664 | 6.77757 | 11.25192 | 10.83229 | 11.44949 | 14.08857 | 14.35728 |
| AC123251. | 0 | 0 | 0 | 0 | 0 | 0.052775 | 0 | 0.05335 |
| 4933428G2 | 0 | 0.774504 | 0.183719 | 0.78845 | 0.925204 | 0.450142 | 0.191453 | 0.455044 |
| Nme1 | 13.44723 | 13.28285 | 13.13794 | 15.86584 | 18.77159 | 16.03264 | 15.04416 | 15.95499 |
| Tatdn2 | 14.44389 | 15.80676 | 15.5651 | 15.25638 | 16.83904 | 15.76264 | 16.3222 | 17.0644 |
| LOC102552 | 1.117692 | 0.936752 | 0.977707 | 1.068054 | 1.074261 | 1.179621 | 0.81046 | 1.155777 |
| AABR07031 | 0 | 0 | 0 | 0 | 0 | 0 | 0 | 0.058678 |
| Tfr2 | 0.017531 | 0.059507 | 0.037642 | 0.080772 | 0.113738 | 0.1076 | 0.039226 | 0.13985 |
| LOC103693 | 3083.968 | 3137.884 | 3115.185 | 2266.807 | 2273.858 | 2141.898 | 2304.33 | 2258.053 |
| Synj1 | 4.543778 | 4.133218 | 4.047321 | 4.211786 | 4.09589 | 4.267996 | 5.556484 | 5.542627 |
| Dgcr14 | 7.160114 | 7.929694 | 7.035413 | 6.45798 | 6.167458 | 6.916435 | 7.161857 | 7.099324 |
| Ivns1abp | 308.5246 | 302.7098 | 285.4518 | 421.6405 | 422.7118 | 423.2808 | 459.5432 | 477.3352 |
| Mapkap1 | 8.680423 | 7.526434 | 8.470352 | 9.001938 | 8.531276 | 8.513338 | 9.84476 | 9.994126 |
| Arl3 | 11.7312 | 12.70569 | 11.51706 | 13.0856 | 14.27038 | 13.87247 | 13.5129 | 12.99743 |
| Tbc1d2b | 9.737161 | 9.604952 | 9.241612 | 9.099259 | 9.685169 | 9.656043 | 12.04343 | 12.4862 |
| Gxylt1 | 8.645733 | 7.842904 | 8.023434 | 7.235067 | 7.576923 | 7.193822 | 9.446866 | 9.506319 |
| Rn50_10_0 | 1.282028 | 1.338973 | 1.058722 | 1.272211 | 1.279605 | 1.124083 | 0.99296 | 1.136325 |
| Bbs7 | 2.302795 | 2.48241 | 2.452731 | 2.606471 | 2.568403 | 2.305263 | 2.616836 | 2.330369 |
| Nt5c2 | 13.25531 | 13.00929 | 13.68679 | 16.05693 | 15.46007 | 15.64436 | 16.69563 | 19.00936 |
| Trak2 | 64.50971 | 65.20915 | 62.68675 | 57.43312 | 59.614 | 57.41352 | 65.45947 | 65.5132 |
| Mboat4 | 0.040035 | 0.045298 | 0.128941 | 0.036891 | 0.043289 | 0.140411 | 0.223948 | 0.035485 |
| Dnaaf3 | 2.695905 | 3.66651 | 3.0404 | 2.208152 | 2.826709 | 3.629226 | 2.772337 | 2.896382 |
| Ppp2r4 | 33.65007 | 31.83668 | 33.32721 | 29.86536 | 29.77822 | 28.3055 | 31.71842 | 29.63636 |
| Atf6 | 6.081781 | 6.792703 | 6.481171 | 5.604122 | 5.476088 | 6.008877 | 6.804026 | 7.441781 |

| | | | | | | | | |
|---|---|---|---|---|---|---|---|---|
| Kcnma1 | 0.054905 | 0.264022 | 0.147361 | 0.354151 | 0.682735 | 0.565657 | 0.859958 | 0.729979 |
| Pxmp2 | 68.14743 | 63.08641 | 63.98929 | 62.73508 | 55.56481 | 58.77969 | 49.8298 | 50.28721 |
| Ptrh1 | 4.560903 | 4.873755 | 5.440467 | 7.704936 | 6.940824 | 6.887173 | 6.614398 | 8.384558 |
| AC123280. | 0 | 0.099246 | 0.094168 | 0 | 0.094845 | 0.076909 | 0 | 0 |
| LOC298795 | 0 | 0 | 0 | 0 | 0 | 0 | 0 | 0 |
| Nr4a1 | 94.88441 | 103.0719 | 115.371 | 95.68688 | 88.74421 | 93.70598 | 126.7861 | 123.918 |
| AC099450. | 3.927444 | 2.221859 | 0.702727 | 3.015821 | 4.246688 | 5.16538 | 6.590775 | 2.320726 |
| AABR07017 | 0 | 0 | 0 | 0 | 0 | 0.017372 | 0 | 0 |
| Rn60_20_( | 2.432297 | 0.971305 | 1.382414 | 0.856955 | 1.315004 | 2.069915 | 2.801179 | 1.331564 |
| RGD156181 | 0 | 0 | 0 | 0 | 0 | 0.043645 | 0 | 0 |
| Tmem167a | 47.03236 | 43.61427 | 42.68416 | 40.88113 | 46.92329 | 47.18989 | 52.07526 | 43.62105 |
| Kmt2d | 30.06702 | 31.01944 | 32.37482 | 28.80364 | 27.70323 | 28.85083 | 35.1708 | 34.57159 |
| Socs1 | 1.56729 | 1.564689 | 1.385659 | 3.185726 | 2.492188 | 2.465479 | 3.197403 | 3.22777 |
| Lrrc58 | 19.0145 | 19.62077 | 22.08716 | 17.17069 | 21.42362 | 19.63953 | 21.86821 | 21.33652 |
| LOC100911 | 0 | 0 | 0 | 0.028318 | 0 | 0.026945 | 0 | 0.027239 |
| Gabrg3 | 0 | 0 | 0 | 0 | 0 | 0 | 0 | 0 |
| AABR07072 | 0 | 0.049211 | 0.046693 | 0.040077 | 0 | 0.038135 | 0.048658 | 0 |
| Pih1d1 | 4.244782 | 5.218396 | 4.513227 | 4.964469 | 5.957942 | 4.795433 | 6.073079 | 5.788249 |
| AABR07065 | 0.140769 | 0 | 0 | 0 | 0.152211 | 0 | 0 | 0 |
| Emc9 | 5.12737 | 5.40661 | 6.139701 | 5.283802 | 5.101275 | 4.588788 | 5.193195 | 4.732882 |
| RGD156177 | 0.252975 | 0.057246 | 0.108634 | 0.046621 | 0.164123 | 0.310533 | 0.056604 | 0.044845 |
| Kcne1 | 0.447572 | 0.303844 | 0.192199 | 3.629296 | 4.549159 | 3.924315 | 1.802605 | 2.618255 |
| Cldn20 | 0.555396 | 0.538633 | 0.596253 | 0.438665 | 0.085792 | 0.347837 | 0.177529 | 0.773575 |
| Rpl35a | 0.94353 | 1.06756 | 1.350586 | 0.724522 | 2.210488 | 0.275763 | 1.05558 | 0.836298 |
| Atp5j2 | 1181.17 | 1175.941 | 1151.064 | 1002.491 | 1020.421 | 911.934 | 948.6801 | 923.3055 |
| Sepn1 | 30.43584 | 34.64594 | 39.42418 | 33.70223 | 31.03042 | 31.32302 | 41.58022 | 41.66162 |
| Larp1b | 24.86851 | 25.47492 | 25.28679 | 24.4196 | 24.52878 | 23.06878 | 25.11788 | 24.67298 |
| Adrm1 | 52.802 | 61.75771 | 65.89346 | 53.94573 | 51.63219 | 57.1621 | 61.7558 | 62.8738 |
| Hykk | 0.355519 | 0.331268 | 0.157159 | 0.308327 | 0.31658 | 0.293383 | 0.350946 | 0.203898 |
| Grcc10 | 4.810518 | 4.976342 | 6.63994 | 5.319243 | 4.904312 | 5.543481 | 5.381793 | 3.411041 |
| AC113785. | 0 | 0 | 0 | 0.04944 | 0 | 0 | 0 | 0 |
| Gtf3a | 9.245674 | 9.659773 | 7.982894 | 8.265751 | 7.99777 | 7.830645 | 9.199245 | 9.624648 |
| AABR07067 | 0 | 0 | 0 | 0 | 0 | 0 | 0.283018 | 0.224225 |
| Tle4 | 2.651584 | 3.013131 | 3.068462 | 2.792378 | 2.395472 | 2.777816 | 3.043526 | 2.90981 |
| Nudt10 | 0.9538 | 0.605393 | 0.574419 | 0.964634 | 0.981021 | 1.305432 | 0.806808 | 0.989737 |
| Cep85 | 10.11945 | 11.1352 | 10.32136 | 9.243182 | 9.152493 | 10.35707 | 10.88304 | 9.88758 |
| Mir349 | 0 | 0 | 0 | 0.497455 | 0.583737 | 0 | 0 | 0 |
| Arhgap17 | 7.401934 | 7.517803 | 6.590978 | 7.198705 | 6.791971 | 6.64224 | 8.669401 | 8.63103 |
| AABR07058 | 0 | 0 | 0.535411 | 0.919107 | 0 | 0 | 0 | 0.442043 |
| Ctc1 | 6.777202 | 7.682052 | 6.79866 | 7.371059 | 7.314742 | 7.100401 | 8.921663 | 7.549745 |
| Nub1 | 22.12524 | 19.42767 | 20.0539 | 23.56243 | 22.38421 | 23.26933 | 21.02549 | 22.89177 |
| Slc9b2 | 0.291954 | 0.090091 | 0.056987 | 0.611418 | 0.401782 | 0.581785 | 0.534477 | 0.446972 |
| Wfdc10a | 0 | 0 | 0 | 0 | 0 | 0 | 0.120297 | 0 |
| Zfand3 | 40.01242 | 41.01718 | 42.89077 | 38.43483 | 37.71191 | 37.52933 | 44.6284 | 44.15651 |
| Cyb5r4 | 9.068302 | 8.293346 | 8.600446 | 9.654957 | 9.09415 | 8.424865 | 8.59452 | 9.16213 |
| Sorcs1 | 0.225944 | 0.360911 | 0.442326 | 0.171458 | 0.229939 | 0.361256 | 0.148692 | 0.270948 |
| Ccdc54 | 0 | 0 | 0 | 0 | 0 | 0 | 0 | 0 |
| C1rl | 0.832634 | 1.324013 | 1.159635 | 2.757914 | 1.411305 | 1.99285 | 1.938556 | 2.313745 |
| Ebi3 | 0.565413 | 0.83658 | 0.513621 | 0.400774 | 0.752459 | 0.572024 | 0.340609 | 0.809556 |
| LOC100911 | 2.109338 | 2.657824 | 3.087967 | 1.693351 | 2.021615 | 1.541225 | 2.538609 | 2.138722 |
| Iqcc | 4.270538 | 3.893678 | 4.629207 | 3.514876 | 3.721036 | 3.708058 | 4.174679 | 3.932188 |
| Slc29a1 | 14.55847 | 15.32365 | 16.42687 | 20.4648 | 20.66789 | 20.94916 | 19.38972 | 20.23366 |
| Cyp2c22 | 0 | 0 | 0 | 0 | 0.066264 | 0 | 0 | 0 |
| Slc25a25 | 6.247163 | 5.726514 | 6.634998 | 5.263978 | 4.569536 | 4.642709 | 6.932986 | 6.943671 |

| | | | | | | | | |
|---|---|---|---|---|---|---|---|---|
| Zfp617 | 2.680007 | 2.673213 | 2.687872 | 2.209571 | 3.12663 | 2.875453 | 2.801018 | 3.969457 |
| Cog4 | 20.56491 | 19.7641 | 19.82739 | 22.48217 | 22.88995 | 23.11454 | 21.97189 | 23.04825 |
| Chrdl1 | 0.714197 | 0.46176 | 0.835194 | 1.280953 | 1.379019 | 1.487245 | 0.88462 | 1.107799 |
| Nfkbib | 15.09547 | 15.08787 | 17.17107 | 13.25408 | 14.29739 | 14.91071 | 16.88814 | 15.83671 |
| Ly49si2 | 0.651317 | 0.221081 | 0.069923 | 0.060016 | 0.70426 | 0.399753 | 0.145733 | 0.173189 |
| Hemk1 | 2.206764 | 2.76678 | 2.593207 | 2.418153 | 2.450633 | 2.61472 | 2.101842 | 2.008829 |
| Bcr | 3.123486 | 3.718464 | 3.761485 | 4.692667 | 4.243752 | 4.667655 | 5.742999 | 5.392558 |
| Btbd16 | 0.055238 | 0.093749 | 0.059302 | 0.07635 | 0.059728 | 0.07265 | 0.030899 | 0.122401 |
| Pxn | 43.6987 | 46.00486 | 50.99115 | 42.62122 | 39.54055 | 41.96339 | 51.66963 | 50.53107 |
| AC126877. | 0 | 0 | 0 | 0.54217 | 0 | 0 | 0 | 0 |
| Fas | 1.169157 | 1.485301 | 1.277185 | 1.644349 | 1.929557 | 1.654576 | 1.606317 | 1.581693 |
| Erich6 | 0.02181 | 0.049354 | 0.070243 | 0.060291 | 0 | 0.038246 | 0.0244 | 0.193313 |
| Med26 | 2.694916 | 2.55094 | 2.325878 | 2.045037 | 2.018832 | 2.023141 | 3.881998 | 3.575151 |
| Lztfl1 | 5.795919 | 5.227904 | 4.090176 | 5.378058 | 5.741137 | 5.650467 | 4.035631 | 5.927551 |
| Rpl5 | 366.3165 | 356.218 | 340.3066 | 336.6435 | 327.8703 | 328.8319 | 367.1817 | 358.8984 |
| LOC10369( | 2.32919 | 1.941851 | 6.821634 | 2.01447 | 2.010397 | 1.898921 | 4.25156 | 3.29592 |
| Ndufaf7 | 7.092599 | 6.449954 | 6.357159 | 7.32959 | 6.665688 | 6.35441 | 6.006782 | 6.306108 |
| Mir628 | 1.246808 | 1.410704 | 1.338528 | 0.574442 | 0.674078 | 3.279606 | 2.789746 | 1.657661 |
| Rrad | 124.2666 | 124.5491 | 130.4231 | 145.6134 | 149.8627 | 149.7144 | 102.8522 | 102.5606 |
| Spink1 | 0.081567 | 0 | 0 | 0 | 0.088197 | 0 | 0 | 0 |
| AABR0701 | 0.057043 | 0.129084 | 0.06124 | 0.026282 | 0.06168 | 0.025008 | 0.031909 | 0.075841 |
| Osbpl2 | 13.51068 | 14.23996 | 12.95273 | 13.31978 | 13.73362 | 13.46847 | 16.55982 | 15.23804 |
| Trpt1 | 2.271249 | 2.1792 | 2.886984 | 2.645384 | 2.337987 | 2.517172 | 2.927201 | 2.963314 |
| LOC10090 | 1.720233 | 1.602887 | 1.792463 | 1.818234 | 1.914771 | 1.818835 | 1.698106 | 2.421627 |
| Zfp771 | 28.92751 | 31.55009 | 35.47519 | 35.05181 | 33.653 | 30.22463 | 37.88966 | 33.76486 |
| Pitrm1 | 17.31314 | 18.37362 | 17.84057 | 19.08291 | 19.69404 | 18.43505 | 18.69761 | 17.4177 |
| RGD15622( | 6.25404 | 6.284046 | 6.613901 | 5.375795 | 5.954952 | 6.506598 | 6.474598 | 7.570282 |
| Edc3 | 4.421397 | 4.127147 | 4.479653 | 3.310241 | 3.884394 | 4.02206 | 4.235409 | 4.114849 |
| Pde6g | 0 | 0 | 0.314068 | 0.269571 | 0 | 0 | 0.327289 | 0.518598 |
| AABR0703 | 0.195395 | 0 | 0 | 0.360098 | 0.211278 | 0.256984 | 0.1093 | 0.086594 |
| Dusp7 | 32.29092 | 34.52591 | 39.50563 | 23.45488 | 21.07263 | 23.02336 | 29.90161 | 28.39826 |
| Fam110c | 0 | 0.076181 | 0 | 0.031021 | 0.036401 | 0.073794 | 0.056494 | 0.014919 |
| Ptrhd1 | 4.687779 | 2.716684 | 3.375545 | 5.26781 | 3.523453 | 4.410999 | 3.645552 | 4.155011 |
| Rassf6 | 0.184474 | 0.166979 | 0.184841 | 0.079326 | 0.06649 | 0.053916 | 0.013759 | 0.076304 |
| Rbp7 | 57.93851 | 57.58057 | 55.69029 | 35.79341 | 31.90001 | 34.20234 | 33.00545 | 35.37382 |
| Sos2 | 5.602468 | 5.640353 | 4.983531 | 4.783171 | 4.895746 | 4.421024 | 6.127086 | 5.766345 |
| Scai | 1.824684 | 1.58376 | 1.502729 | 1.704407 | 1.756784 | 1.512219 | 2.013411 | 2.038251 |
| Acap3 | 3.98001 | 4.474872 | 4.299668 | 5.224348 | 5.115514 | 4.323687 | 5.908874 | 5.779628 |
| Bbof1 | 1.596522 | 2.331885 | 2.24374 | 1.792106 | 2.35404 | 2.188828 | 1.916017 | 1.981107 |
| Plac8l1 | 0.062789 | 0 | 0.067408 | 0.115715 | 0 | 0.055053 | 0.210736 | 0.278264 |
| Lypd1 | 3.281007 | 2.298094 | 2.907354 | 3.263265 | 2.365137 | 2.968117 | 2.359701 | 3.554369 |
| LOC10036 | 14.16455 | 15.54087 | 17.35697 | 11.60185 | 10.67474 | 12.67039 | 11.84499 | 14.5838 |
| Mal2 | 0 | 0 | 0 | 0 | 0 | 0 | 0 | 0 |
| AABR0705 | 0.508407 | 0 | 0 | 0 | 0 | 0 | 0 | 0 |
| Rbm4b | 4.715553 | 4.215645 | 4.81245 | 5.820417 | 4.815589 | 5.027879 | 6.122244 | 4.902034 |
| AABR0701 | 0 | 0 | 0 | 0 | 0 | 0 | 0 | 0 |
| Prr16 | 0.734894 | 0.298487 | 0.525971 | 0.711903 | 0.509378 | 0.346961 | 0.611355 | 0.400845 |
| RGD156323 | 0 | 0.170257 | 0.161546 | 0.138658 | 0 | 0 | 0 | 0.400125 |
| Capzb | 97.62727 | 96.65798 | 99.30263 | 91.74802 | 92.33479 | 91.03891 | 93.82852 | 96.48445 |
| Hlx | 7.560466 | 9.1575 | 8.532882 | 7.614215 | 6.576701 | 6.777751 | 7.265475 | 7.044869 |
| Smarcd1 | 5.050602 | 4.37206 | 5.181159 | 5.008516 | 4.90636 | 4.751713 | 5.650389 | 5.78405 |
| Tspan32 | 1.369732 | 1.370966 | 1.244265 | 0.679622 | 1.367143 | 1.108599 | 0.70726 | 1.027283 |
| AABR0701 | 0 | 0 | 0 | 0 | 0.036227 | 0 | 0.074964 | 0 |
| AC134746. | 0 | 0 | 0 | 0.507928 | 0 | 0 | 0 | 0 |

| | | | | | | | | |
|---|---|---|---|---|---|---|---|---|
| AABR0705 | 0 | 0.884322 | 0.419539 | 0 | 0.845112 | 0 | 0.437199 | 0 |
| AABR0707 | 0 | 0 | 0 | 0.371178 | 0.435558 | 0 | 0 | 0.71407 |
| Snx25 | 2.447006 | 3.152026 | 1.879327 | 2.410922 | 2.401674 | 2.07952 | 2.716543 | 2.752838 |
| Mael | 0 | 0.029625 | 0 | 0 | 0 | 0 | 0 | 0 |
| Sel1l3 | 0.240734 | 0.2007 | 0.12242 | 0.116751 | 0.109601 | 0.077765 | 0.042525 | 0.101072 |
| RGD15612 | 0.05559 | 0 | 0 | 0.153672 | 0.060109 | 0 | 0.062192 | 0.049272 |
| Hspd1 | 131.5614 | 126.3477 | 116.3996 | 117.6102 | 116.652 | 104.7147 | 124.4299 | 123.3205 |
| Smad6 | 10.35096 | 12.01414 | 12.79363 | 10.55867 | 8.590432 | 9.00875 | 14.91324 | 12.797 |
| AABR0706 | 0 | 0.383907 | 0.364265 | 0.521092 | 0.122295 | 0.297502 | 0.379598 | 0.200495 |
| AABR0707 | 0 | 0 | 0 | 0 | 0 | 0 | 0.130478 | 0 |
| LOC10368 | 0 | 0 | 0 | 0.012063 | 0.014156 | 0.011479 | 0 | 0 |
| AABR0705 | 0.047692 | 0.107923 | 0 | 0.043946 | 0 | 0.125449 | 0.106712 | 0 |
| Lypla1 | 39.49124 | 39.12233 | 34.54254 | 38.0288 | 42.12426 | 36.71209 | 36.71803 | 38.13677 |
| AC131483. | 0 | 0.133746 | 0.084602 | 0.036308 | 0.042605 | 0.034548 | 0.088164 | 0 |
| LOC10091 | 0.066879 | 0.07567 | 0.143597 | 0 | 0.072315 | 0 | 0 | 0 |
| Clcc1 | 8.202923 | 8.481534 | 8.208541 | 8.683591 | 8.916019 | 9.389465 | 9.392715 | 9.453755 |
| Umps | 10.52909 | 9.066661 | 9.836511 | 10.67522 | 9.638589 | 11.35607 | 11.53625 | 11.45222 |
| LOC10091 | 0.191817 | 0.434063 | 0.617782 | 0.353503 | 0 | 0 | 0.214596 | 0.340033 |
| Crygs | 0 | 0.085129 | 0.161546 | 0.069329 | 0.406771 | 0.263876 | 0 | 0.26675 |
| Ghdc | 1.777179 | 2.03834 | 1.960187 | 3.006007 | 2.632381 | 2.924354 | 2.641893 | 3.77617 |
| AABR0705 | 0 | 0.117559 | 0 | 0.09574 | 0 | 0 | 0.232479 | 0 |
| Cyp2f4 | 0 | 0 | 0.029495 | 0.050633 | 0 | 0.024089 | 0 | 0 |
| Tmem9b | 13.14844 | 12.59376 | 12.29881 | 13.88515 | 15.97677 | 15.06703 | 16.42118 | 13.78878 |
| Wrn | 2.063793 | 2.052822 | 2.081703 | 2.016643 | 2.452253 | 2.515454 | 2.156646 | 2.371985 |
| AC134760. | 0 | 0 | 0 | 0 | 0 | 0.376348 | 0 | 0 |
| Capn1 | 11.88942 | 11.47404 | 13.1207 | 12.24454 | 11.49465 | 12.37162 | 14.45545 | 15.45084 |
| Grm6 | 0 | 0 | 0 | 0.010907 | 0 | 0 | 0.013242 | 0 |
| Pate3 | 0 | 0 | 0 | 0 | 0 | 0.309188 | 0 | 0 |
| Sez6 | 0.456036 | 0.383302 | 0.545536 | 0.540281 | 0.563548 | 0.651188 | 0.553923 | 0.415756 |
| AABR0701 | 0.127411 | 17.58747 | 10.53236 | 12.79706 | 9.505969 | 7.931699 | 0 | 0.338792 |
| AC097312. | 0.10288 | 0.116404 | 0.441793 | 0.0948 | 0 | 0 | 0.115098 | 0.091188 |
| Mios | 2.359958 | 1.895986 | 1.903922 | 1.917258 | 2.234702 | 2.020237 | 2.06218 | 2.203142 |
| Fgd2 | 1.190994 | 1.6684 | 1.420676 | 2.160068 | 1.737514 | 1.773592 | 2.537964 | 2.865301 |
| AABR0703 | 0.172825 | 0.097772 | 0.278308 | 0.398128 | 0 | 0.075766 | 0.483372 | 0.306366 |
| Crmp1 | 0.717593 | 0.791105 | 0.730876 | 0.542551 | 0.537178 | 0.661453 | 0.741057 | 1.288386 |
| AABR0701 | 0 | 0 | 0 | 0 | 0 | 0 | 0.582932 | 0 |
| Bms1 | 9.711346 | 9.590759 | 9.077593 | 9.450054 | 9.55024 | 9.854548 | 11.16902 | 11.4645 |
| Papd5 | 12.498 | 12.77816 | 12.83648 | 12.38497 | 12.13609 | 13.33051 | 14.91956 | 14.00171 |
| Klhl9 | 18.99438 | 18.82961 | 16.65723 | 16.64935 | 17.49412 | 18.50842 | 17.42841 | 19.15361 |
| Ubald2 | 84.50923 | 99.35142 | 118.0353 | 82.08918 | 84.35834 | 68.49844 | 95.97407 | 89.0555 |
| Wisp3 | 0.020871 | 0.070844 | 0 | 0.019232 | 0.022568 | 0.0549 | 0.070049 | 0.036998 |
| Ccdc71l | 15.60521 | 20.84202 | 28.15226 | 18.82688 | 14.78622 | 18.126 | 27.08753 | 28.30501 |
| Higd2al1 | 0.815669 | 0.738312 | 0.875672 | 1.20257 | 0.352788 | 1.00125 | 1.09504 | 0.433781 |
| Fam105a | 1.525894 | 1.177144 | 1.489223 | 1.78952 | 2.774878 | 2.554183 | 3.33661 | 2.643476 |
| LOC69000 | 18.92979 | 18.03248 | 20.22923 | 16.48399 | 15.70894 | 17.39617 | 18.80046 | 19.58443 |
| Rp9 | 4.913889 | 3.319304 | 5.275374 | 3.987304 | 4.440981 | 3.922666 | 6.400006 | 4.485437 |
| Kcnip1 | 0 | 0.046037 | 0 | 0.074985 | 0 | 0 | 0.182081 | 0.036064 |
| AABR0704 | 0 | 0 | 0.061914 | 0.212569 | 0 | 0 | 0 | 0.051117 |
| Dvl1 | 14.69081 | 15.23847 | 17.61693 | 17.8806 | 16.7664 | 20.94509 | 22.36328 | 22.16274 |
| Pter | 6.246632 | 5.756813 | 5.137783 | 5.454299 | 5.011324 | 5.123694 | 4.90319 | 5.358097 |
| Ccdc114 | 0.040421 | 0.022867 | 0.021697 | 0.11174 | 0.087414 | 0.088604 | 0.226108 | 0.125396 |
| AABR0701 | 0.056036 | 0 | 0.030079 | 0 | 0 | 0 | 0.031345 | 0 |
| Reep2 | 0.197906 | 0.044784 | 0.084986 | 0.43767 | 0.470784 | 0.312343 | 0.664225 | 0.666573 |
| Rtn1 | 14.10346 | 14.21263 | 14.83056 | 12.1965 | 12.29716 | 12.73211 | 13.37024 | 15.20574 |

| | | | | | | | | |
|---|---|---|---|---|---|---|---|---|
| 5S_rRNA | 0 | 0 | 1.703581 | 0.974811 | 0 | 0 | 0.591764 | 1.406501 |
| Prrt2 | 0.499136 | 0.392869 | 0.652345 | 0.999858 | 1.079418 | 1.141678 | 1.699514 | 1.327228 |
| AABR07004 | 0 | 0 | 0.224873 | 0 | 0 | 0.122439 | 0 | 0.061886 |
| C7 | 130.9354 | 123.8685 | 120.9421 | 237.6153 | 238.3812 | 224.2718 | 371.798 | 366.1407 |
| Plppr4 | 0.028301 | 0.021347 | 0.030383 | 0.008693 | 0.0102 | 0 | 0 | 0.008361 |
| Odf2l | 1.644519 | 1.777069 | 1.785333 | 2.179394 | 1.898073 | 1.927955 | 2.19124 | 1.801552 |
| Ttll9 | 0.301146 | 0.45431 | 0.251455 | 0.46249 | 0.470347 | 0.616105 | 1.085594 | 0.830419 |
| Cnga1 | 0 | 0 | 0 | 0 | 0.027594 | 0.022375 | 0 | 0 |
| Zfp316 | 2.008133 | 1.767195 | 2.505955 | 1.850415 | 1.633163 | 1.670439 | 1.939381 | 2.388423 |
| Sac3d1 | 4.578441 | 5.319049 | 5.266338 | 5.499577 | 3.536144 | 3.297527 | 4.847755 | 4.89146 |
| Ccdc142 | 0.290922 | 0.246873 | 1.249292 | 0.245734 | 0.157285 | 0.297594 | 0.949289 | 0.709111 |
| AABR07024 | 0 | 0.039765 | 0 | 0 | 0 | 0 | 0 | 0 |
| Neurog2 | 0 | 0 | 0 | 0.060926 | 0 | 0 | 0 | 0 |
| Mllt10 | 9.628055 | 10.51039 | 10.33633 | 9.118315 | 9.909422 | 10.25533 | 11.44964 | 11.34682 |
| AABR07026 | 0.969739 | 3.291643 | 2.082154 | 1.340365 | 3.669978 | 1.275402 | 1.084901 | 1.289292 |
| Zfp622 | 3.603511 | 6.274845 | 3.402173 | 3.956333 | 4.228035 | 4.32479 | 5.060754 | 5.210024 |
| Dnlz | 47.47288 | 49.29612 | 47.05337 | 49.11651 | 49.19292 | 44.40834 | 46.82114 | 44.93811 |
| AABR07030 | 0.671358 | 0 | 0 | 0 | 0 | 0 | 0 | 0 |
| Med31 | 11.5287 | 10.55129 | 11.22163 | 11.18982 | 11.13613 | 9.164927 | 9.458385 | 10.94511 |
| Rps17 | 521.3318 | 525.0536 | 521.5102 | 452.6263 | 462.0677 | 459.8251 | 498.9099 | 494.9736 |
| Slc38a9 | 1.192792 | 1.428976 | 1.16755 | 1.217643 | 1.504707 | 1.332935 | 1.635347 | 1.668767 |
| Pdhx | 43.98847 | 45.60272 | 44.06051 | 53.05582 | 54.52757 | 53.42733 | 43.50346 | 45.01168 |
| Rab4b | 11.69927 | 10.48384 | 12.00727 | 13.45396 | 13.20686 | 14.19697 | 14.18806 | 14.64193 |
| AC112350. | 0 | 0 | 0 | 0 | 0 | 0 | 0 | 0 |
| Zfp266 | 14.48271 | 13.49014 | 12.59084 | 14.58875 | 14.35119 | 13.44255 | 13.6656 | 14.51379 |
| Cul5 | 13.03838 | 11.84992 | 12.06805 | 10.5088 | 10.74149 | 12.16268 | 12.13735 | 13.06268 |
| Rab24 | 13.78755 | 15.50909 | 14.25573 | 18.00858 | 16.67411 | 17.01841 | 19.049 | 17.46477 |
| Cyp4v3 | 4.217772 | 4.949556 | 4.344478 | 4.831879 | 4.468171 | 5.259864 | 7.205515 | 6.693794 |
| Kansl3 | 20.60342 | 20.61382 | 21.54382 | 19.71355 | 18.81623 | 18.45271 | 21.71634 | 23.58128 |
| #N/A | 0.185695 | 0 | 0.39871 | 0.342221 | 0 | 0 | 0 | 0.329181 |
| Capn11 | 0 | 0 | 0 | 0 | 0 | 0.019163 | 0 | 0 |
| LOC103692 | 0 | 0 | 0 | 0 | 0 | 0 | 0 | 0 |
| LOC100363 | 23.08605 | 24.75558 | 24.0935 | 25.64606 | 28.18079 | 26.16632 | 25.19771 | 25.16793 |
| RGD130753 | 0.067222 | 0.228176 | 0.108251 | 0.030971 | 0.036343 | 0.147351 | 0.112807 | 0.119164 |
| Dlc1 | 31.33483 | 32.16929 | 32.98453 | 21.93951 | 22.34993 | 23.11564 | 26.58124 | 28.13804 |
| Hip1 | 12.91399 | 13.47896 | 12.07288 | 10.81378 | 10.86237 | 11.2732 | 14.22521 | 14.43532 |
| Ccz1b | 29.73033 | 30.30739 | 30.82931 | 33.44365 | 31.57292 | 30.51092 | 32.55904 | 30.8432 |
| Prkcsh | 26.51439 | 32.12977 | 32.19379 | 33.10532 | 33.2281 | 32.59347 | 35.3881 | 35.95656 |
| AABR07033 | 0 | 0.255386 | 0 | 0 | 0.244063 | 0 | 0.25252 | 0.200063 |
| Mir10a | 0 | 0 | 0.511074 | 0.438665 | 0.51475 | 0 | 0 | 0.8439 |
| Fabp7 | 0 | 0 | 0 | 0 | 0 | 0.055119 | 0 | 0 |
| AABR07030 | 295.012 | 226.439 | 212.0705 | 354.9711 | 329.0833 | 314.1278 | 371.2296 | 402.5655 |
| Zbtb17 | 8.862548 | 8.127357 | 8.884555 | 8.278359 | 8.379607 | 7.91262 | 10.3904 | 8.769977 |
| AABR07004 | 0 | 0 | 0.12248 | 0 | 0 | 0 | 0.127635 | 0 |
| Celf5 | 0.04779 | 0.013518 | 0.064132 | 0.033027 | 0.025837 | 0.052378 | 0.026733 | 0.031769 |
| Zmynd8 | 11.6282 | 10.84834 | 11.45429 | 10.24236 | 9.861021 | 9.29629 | 11.2754 | 11.81856 |
| Cfap73 | 0.436383 | 0.423211 | 0.535411 | 0.516998 | 0.539262 | 0.491941 | 0.418462 | 0.442043 |
| Lgals3 | 21.10609 | 26.97856 | 25.65949 | 43.73269 | 43.23789 | 45.86447 | 51.69235 | 51.26832 |
| Slc35b4 | 3.412026 | 3.124425 | 3.771677 | 3.996671 | 3.673741 | 3.473376 | 4.140716 | 3.716237 |
| Rint1 | 4.624708 | 4.561788 | 4.150163 | 4.938953 | 4.128725 | 4.533224 | 5.094319 | 5.150162 |
| Wdr47 | 2.144679 | 2.368828 | 2.083169 | 2.435007 | 2.388029 | 2.719946 | 2.613602 | 2.692993 |
| Lama1 | 0 | 0.005605 | 0 | 0.004565 | 0.005357 | 0 | 0.016628 | 0.004391 |
| LOC100912 | 0 | 0 | 0 | 0 | 0 | 0 | 0.203419 | 0 |
| Mvd | 3.733811 | 4.818174 | 4.505404 | 4.009247 | 3.570188 | 3.327921 | 3.693906 | 3.911182 |

| | | | | | | | | |
|---|---|---|---|---|---|---|---|---|
| AABR07005 | 0 | 0 | 0.010959 | 0 | 0.011038 | 0.0179 | 0 | 0.018095 |
| AABR07071 | 0.060173 | 0.051063 | 0.04845 | 0 | 0.016266 | 0.01319 | 0.01683 | 0.026667 |
| AABR07065 | 0 | 0.152313 | 0 | 0.124044 | 0.291118 | 0 | 0 | 0 |
| AABR07060 | 0 | 0 | 0 | 0 | 0 | 0.91829 | 0 | 0.30943 |
| Olr1106 | 0.056307 | 0 | 0 | 0 | 0 | 0 | 0.062994 | 0 |
| LOC100151 | 4.443058 | 4.447856 | 3.474377 | 5.273475 | 4.883296 | 5.402647 | 5.13434 | 4.926681 |
| Exosc7 | 9.548488 | 9.438992 | 9.765343 | 8.19655 | 10.92238 | 8.900889 | 10.12019 | 9.354173 |
| Spink13 | 0 | 0 | 0 | 0.073895 | 0 | 0 | 0.089716 | 0 |
| Tasp1 | 9.992864 | 9.494924 | 10.46126 | 8.317752 | 10.35741 | 10.18977 | 7.535402 | 8.636966 |
| LOC691931 | 5.199973 | 6.297857 | 6.447398 | 4.994031 | 5.226693 | 5.71523 | 5.243942 | 5.907302 |
| Ptpn12 | 14.87693 | 15.39047 | 14.57945 | 13.97174 | 13.0448 | 14.10382 | 17.65161 | 16.84791 |
| Htr7 | 0 | 0 | 0 | 0.033626 | 0 | 0.031996 | 0 | 0 |
| Stra6 | 0.044416 | 0 | 0 | 0.081854 | 0.120065 | 0.019472 | 0.124225 | 0.059052 |
| Zfp54 | 0.885414 | 0.744198 | 0.434537 | 1.025671 | 0.930032 | 0.687608 | 0.594337 | 0.807209 |
| Faxc | 0.374265 | 0.211732 | 0.133932 | 0.114957 | 0.134896 | 0.218771 | 0.104678 | 0.221153 |
| Spata31d1 | 0.393219 | 0.46025 | 0.567713 | 0.524762 | 0.425182 | 0.427996 | 0.455085 | 0.624949 |
| AABR07012 | 0.077579 | 0.263331 | 0.291502 | 0.178715 | 0.083885 | 0.136043 | 0.347168 | 0.378192 |
| Zfp458 | 0.384926 | 0.362938 | 0.258276 | 0.206905 | 0.416214 | 0.26719 | 0.394751 | 0.326963 |
| Klrd1 | 2.101843 | 1.630722 | 1.450583 | 1.217396 | 1.461017 | 1.474318 | 0.806211 | 0.984712 |
| Hephl1 | 0 | 0 | 0 | 0.028393 | 0.033317 | 0 | 0.017236 | 0.013655 |
| Eda2r | 1.332823 | 1.111178 | 1.807416 | 1.195825 | 1.554938 | 0.984101 | 1.294906 | 1.834197 |
| AABR07030 | 0.148767 | 0.336645 | 0 | 0.137083 | 0 | 0 | 0.166434 | 0 |
| #N/A | 0 | 0 | 0.646186 | 0.554634 | 0 | 1.055505 | 0.673387 | 0 |
| AABR07002 | 0 | 0 | 0 | 0 | 0 | 0 | 0.960404 | 0.380447 |
| MAST1 | 0 | 0.050286 | 0.023857 | 0.020477 | 0.060071 | 0.038968 | 0.037291 | 0.009848 |
| RGD156366 | 0 | 0 | 0 | 0 | 0 | 0 | 0.037291 | 0 |
| Ydjc | 2.167137 | 2.452014 | 2.072754 | 2.759397 | 2.854559 | 3.005689 | 3.129805 | 3.457515 |
| AC136847. | 0 | 0 | 0 | 0 | 0 | 0 | 0 | 0 |
| Psma1 | 85.3734 | 86.85564 | 88.73275 | 81.38093 | 81.0271 | 86.31454 | 80.24203 | 82.54984 |
| Dcaf11 | 82.31147 | 87.03104 | 86.76336 | 79.40321 | 76.85982 | 82.81613 | 85.98927 | 88.72049 |
| Eml6 | 0.178194 | 0.13149 | 0.182986 | 0.121365 | 0.234566 | 0.095103 | 0.346706 | 0.185411 |
| Dtx3 | 11.72997 | 11.34206 | 13.04261 | 18.14318 | 17.47209 | 16.73911 | 18.94795 | 18.93712 |
| #N/A | 0.752252 | 0.816397 | 0.46148 | 0.735609 | 0.680599 | 0.780721 | 1.082039 | 1.006941 |
| AABR07063 | 0 | 0 | 0 | 0 | 0 | 0 | 0 | 0.059967 |
| Ankib1 | 4.995236 | 5.067666 | 5.259576 | 4.912725 | 5.128616 | 5.053647 | 5.628749 | 6.162349 |
| #N/A | 0 | 0 | 0.747085 | 0.160309 | 0 | 0 | 0 | 0 |
| Ncs1 | 7.174393 | 7.5107 | 7.97085 | 5.688467 | 5.605551 | 5.590407 | 6.359783 | 6.242827 |
| AABR07063 | 0 | 0 | 0 | 0 | 0 | 0.533889 | 0 | 0 |
| Patl1 | 7.501346 | 6.110943 | 7.604998 | 5.830287 | 6.108009 | 5.879433 | 7.385112 | 7.758879 |
| Fabp4 | 1022.03 | 1008.495 | 1001.597 | 729.6031 | 734.8037 | 671.2474 | 804.9438 | 807.2353 |
| AABR07062 | 4.122099 | 4.270499 | 4.197696 | 3.180924 | 3.154866 | 3.264733 | 4.080241 | 4.533196 |
| #N/A | 10.13058 | 9.72455 | 8.814805 | 14.61474 | 14.69056 | 15.74507 | 14.24139 | 14.39819 |
| Fcho2 | 5.48156 | 5.704962 | 5.896598 | 6.74147 | 7.376249 | 6.857796 | 7.287751 | 7.711411 |
| Spert | 0 | 0.07567 | 0 | 0 | 0 | 0 | 0 | 0 |
| Cdh3 | 0.0286 | 0.032359 | 0.076759 | 0.263534 | 0.231933 | 0.06269 | 0.175978 | 0.228143 |
| AABR07033 | 0.148345 | 0 | 0.159258 | 0 | 0.320807 | 0 | 0.331924 | 0 |
| Zfp398 | 1.303087 | 1.045782 | 1.073611 | 1.103009 | 1.032181 | 0.969837 | 1.135756 | 1.168421 |
| Vom2r47 | 0 | 0 | 0 | 0 | 0 | 0 | 0 | 0 |
| LOC100912 | 0.567652 | 0.642272 | 0.152353 | 0.784604 | 0.613794 | 0.497718 | 0.317532 | 0.125785 |
| Gpr33 | 0.056307 | 0.127418 | 0 | 0.10377 | 0 | 0 | 0 | 0.049908 |
| Capg | 21.14505 | 19.83014 | 23.30997 | 33.1822 | 30.34445 | 31.32513 | 34.17439 | 37.6096 |
| Gyltl1b | 0.087422 | 0.074186 | 0.093853 | 0.080556 | 0.047264 | 0.038326 | 0.024451 | 0.019372 |
| Tufm | 126.1063 | 130.0409 | 123.6561 | 147.8329 | 144.5317 | 149.3439 | 121.8337 | 119.1897 |
| Ccdc188 | 0.033654 | 0.076156 | 0.03613 | 0.124044 | 0.181949 | 0.14754 | 0.376508 | 0.208806 |

| | | | | | | | | |
|---|---|---|---|---|---|---|---|---|
| Desi2 | 9.202234 | 8.958159 | 8.499828 | 9.279449 | 8.898896 | 8.220763 | 10.45045 | 10.31092 |
| Hn1l | 5.288245 | 4.564082 | 5.069933 | 5.892821 | 5.558528 | 5.887578 | 6.631707 | 6.823741 |
| Epcam | 0 | 0.162106 | 0 | 0.099015 | 0.077459 | 0.125621 | 0.080143 | 0.063495 |
| Arse | 10.32487 | 9.778116 | 12.09487 | 15.24337 | 13.60051 | 14.42955 | 15.44389 | 15.69903 |
| #N/A | 0.858458 | 0 | 0.184322 | 0.158207 | 0.371295 | 0.150539 | 0 | 0.152179 |
| AABR07007 | 0 | 0 | 0 | 0 | 0 | 0.04174 | 0 | 0 |
| Gabarapl2 | 39.0113 | 40.93878 | 35.84261 | 41.16065 | 45.1344 | 40.34822 | 36.10022 | 41.48735 |
| Fam129a | 4.987919 | 4.972908 | 4.966817 | 7.313907 | 7.410014 | 6.896046 | 7.44035 | 7.419659 |
| Il17ra | 3.908718 | 4.407121 | 4.152395 | 5.032382 | 4.830217 | 4.884012 | 6.186053 | 5.782199 |
| Cpeb1 | 0.675145 | 0.787766 | 0.770112 | 3.227244 | 3.399176 | 3.662799 | 3.0921 | 3.496984 |
| Ift81 | 12.0328 | 12.32905 | 11.7537 | 14.06193 | 12.08952 | 11.70502 | 12.04626 | 13.0455 |
| Pja1 | 33.23721 | 30.01652 | 30.10692 | 27.03771 | 25.08237 | 25.19605 | 25.46738 | 29.15292 |
| Hbb | 730.5046 | 738.6447 | 742.7612 | 599.8742 | 596.9385 | 620.5412 | 554.1577 | 565.0616 |
| Col6a5 | 0.033151 | 0.022506 | 0.014236 | 0.030548 | 0.064523 | 0.023254 | 0.029671 | 0.058767 |
| LOC100362 | 22.94559 | 17.22222 | 15.73133 | 15.07256 | 17.07273 | 11.45372 | 16.6477 | 20.4385 |
| Cables1 | 3.081533 | 3.258727 | 3.329845 | 2.486892 | 2.700458 | 2.913804 | 4.348785 | 4.052344 |
| Aldh18a1 | 0.324486 | 0.157346 | 0.215649 | 0.38443 | 0.200493 | 0.799337 | 0.276587 | 0.301304 |
| Pkn1 | 17.98117 | 17.31232 | 19.7228 | 18.2102 | 17.95706 | 17.38712 | 21.50192 | 21.96683 |
| Rnf224 | 0.333541 | 0.125795 | 0 | 0 | 0 | 0.194966 | 0.248767 | 0 |
| Inmt | 0.236308 | 0.106949 | 0 | 0.740346 | 0.051103 | 0.497269 | 0.264371 | 0.586465 |
| Tuba1b | 63.73141 | 61.93939 | 64.08451 | 71.29941 | 74.15859 | 73.38323 | 77.16562 | 77.8771 |
| Dctn3 | 27.49759 | 26.58794 | 26.2273 | 30.99103 | 26.59363 | 30.44956 | 27.82159 | 28.49929 |
| Rel | 1.923042 | 1.874563 | 1.9057 | 1.635699 | 1.599506 | 1.893648 | 2.01902 | 2.045386 |
| Hsd3b7 | 4.9011 | 3.729918 | 4.948451 | 6.774256 | 6.119648 | 7.827205 | 7.408757 | 6.87814 |
| Snora58 | 1.378051 | 1.559199 | 0 | 0 | 0 | 0 | 0.770851 | 2.442869 |
| Tmem37 | 1.096031 | 0.505229 | 0.958759 | 1.196977 | 1.316803 | 1.138964 | 1.589506 | 1.223328 |
| Lrrtm4 | 0.167074 | 0.135026 | 0.128118 | 0.109966 | 0.051616 | 0.209273 | 0.186916 | 0.084621 |
| AABR07024 | 1.163687 | 0.658329 | 0.832862 | 1.251007 | 0.629139 | 0.510161 | 0.650941 | 0.171906 |
| Nt5c1a | 6.550529 | 7.194901 | 8.060541 | 4.200529 | 4.224942 | 4.299235 | 5.485616 | 5.873966 |
| Coq6 | 24.455 | 25.68144 | 24.74479 | 28.95728 | 27.32955 | 28.17013 | 26.50727 | 25.10226 |
| Stag3 | 0 | 0 | 0 | 0 | 0 | 0.021854 | 0.027884 | 0.011046 |
| Bmf | 2.110719 | 1.990149 | 1.591589 | 2.130177 | 2.010588 | 1.894744 | 2.248932 | 2.516718 |
| AABR07008 | 0 | 0 | 0 | 0 | 0 | 0 | 0 | 0.24954 |
| Ccnyl1 | 3.85516 | 3.692337 | 3.684949 | 3.536797 | 3.309227 | 3.617415 | 3.9914 | 4.376183 |
| Ltbr | 16.15142 | 17.24662 | 18.8297 | 19.44078 | 17.92819 | 18.49856 | 21.51398 | 21.56318 |
| Bean1 | 5.925936 | 5.883907 | 7.400543 | 4.847601 | 5.949941 | 5.726056 | 5.209029 | 6.645959 |
| Gmpr2 | 8.619139 | 9.75215 | 8.687354 | 10.22772 | 9.31975 | 10.52037 | 10.68329 | 10.82731 |
| Whsc1l1 | 5.67168 | 5.839562 | 5.743356 | 5.952379 | 5.544638 | 6.062892 | 7.549698 | 7.525881 |
| AC094647. | 0 | 0.106182 | 0 | 0 | 0 | 0.082284 | 0.10499 | 0.08318 |
| AC117091. | 0.387896 | 0 | 0 | 0 | 1.258278 | 1.020322 | 0 | 0.343811 |
| Pde12 | 2.860517 | 2.76672 | 3.070948 | 2.975964 | 2.968317 | 3.276717 | 2.942137 | 3.353296 |
| Coro2a | 0.049425 | 0.027961 | 0.053061 | 0.045543 | 0.053443 | 0.195012 | 0 | 0.065712 |
| Ppm1g | 15.49159 | 18.02175 | 16.55313 | 16.8886 | 17.22268 | 14.92221 | 17.92799 | 17.44842 |
| Pdgfra | 9.78344 | 9.559611 | 9.182702 | 14.46705 | 14.32513 | 14.83727 | 20.38861 | 19.82984 |
| Jade3 | 0.466363 | 0.276398 | 0.500671 | 0.368345 | 0.768414 | 0.428379 | 0.322986 | 0.570832 |
| Bmp2k | 3.7111 | 3.325705 | 3.613899 | 3.934091 | 4.012759 | 4.276137 | 4.611085 | 5.356081 |
| Acvr1b | 5.612291 | 5.575968 | 5.464963 | 5.524108 | 5.178277 | 4.78869 | 6.758771 | 6.403066 |
| Baiap2l2 | 0.133757 | 0.45402 | 0.107698 | 0.277317 | 0.433889 | 0.175918 | 0.635977 | 0.26675 |
| Cmklr1 | 5.195196 | 5.175556 | 5.844022 | 7.495447 | 5.796534 | 6.859951 | 8.799278 | 8.860953 |
| LOC361914 | 0.030078 | 0 | 0 | 0 | 0 | 0 | 0 | 0 |
| AC105648. | 9.7251 | 3.38569 | 8.834283 | 6.893305 | 4.044465 | 5.24737 | 1.673848 | 4.641452 |
| AC105645. | 0.8801 | 1.493687 | 0.157474 | 0.135163 | 0.793032 | 0.514448 | 0.656411 | 0.520051 |
| Cyp2r1 | 0.247739 | 0.175191 | 0.166228 | 0.085606 | 0.133939 | 0.271523 | 0.242515 | 0.192136 |
| Kctd7 | 0.92181 | 1.323789 | 1.065747 | 1.437466 | 0.805059 | 1.119107 | 1.11061 | 0.942746 |

| | | | | | | | | |
|---|---|---|---|---|---|---|---|---|
| RGD130481 | 0.420441 | 0.570851 | 0.428802 | 0.464904 | 0.591002 | 0.442372 | 0.635 | 0.83848 |
| AABR07053 | 5.691948 | 5.251217 | 6.016659 | 5.406288 | 4.82901 | 5.374605 | 4.506513 | 5.821219 |
| Rab29 | 19.69162 | 18.95658 | 20.24477 | 21.98792 | 21.29226 | 22.22592 | 21.90839 | 21.50368 |
| Kcnb2 | 0 | 0 | 0 | 0 | 0.026178 | 0.021227 | 0 | 0.021458 |
| Kng2 | 0 | 0 | 0 | 0.029262 | 0.034337 | 0 | 0 | 0 |
| Spata7 | 2.695497 | 3.020218 | 1.966652 | 2.532024 | 2.34866 | 2.524035 | 3.366935 | 3.038632 |
| Rn50_1_04 | 14.18346 | 14.38204 | 11.59137 | 12.93383 | 14.11583 | 14.37248 | 13.45196 | 13.65901 |
| AABR07046 | 0 | 0.452287 | 0 | 0 | 0 | 0 | 0 | 0 |
| Tcf3 | 10.68413 | 12.35379 | 13.1686 | 11.73482 | 11.08796 | 11.62847 | 13.17663 | 12.58611 |
| LOC100365 | 0.066286 | 0.074999 | 0.142324 | 0.06108 | 0.215022 | 0.05812 | 0.148316 | 0.176258 |
| Ept1 | 13.13278 | 12.8966 | 11.85251 | 13.62352 | 16.07579 | 14.60477 | 15.43161 | 15.78875 |
| Atp6v1b2 | 21.9879 | 22.01946 | 21.82466 | 23.69113 | 22.33617 | 22.526 | 21.4271 | 22.53198 |
| Nckipsd | 2.222275 | 2.208159 | 2.69162 | 2.192131 | 2.325898 | 2.448106 | 2.964295 | 2.790427 |
| Ncf4 | 1.435162 | 2.138688 | 2.292318 | 2.290089 | 2.006011 | 2.547395 | 3.641961 | 3.350781 |
| AABR07035 | 0.020384 | 0.06919 | 0 | 0.018783 | 0 | 0.017873 | 0 | 0 |
| AABR07010 | 0.126488 | 0 | 0.045264 | 0.038851 | 0 | 0 | 0 | 0 |
| Zfp691 | 7.644922 | 8.690479 | 8.785292 | 7.540586 | 6.830406 | 7.42688 | 6.786024 | 7.666826 |
| RGD156266 | 0.166241 | 0 | 0.356941 | 0.459554 | 0.179754 | 0.291521 | 0 | 0 |
| Zfp580 | 3.632318 | 3.424831 | 4.224486 | 6.043264 | 3.272978 | 4.423361 | 4.628076 | 4.292672 |
| Sesn3 | 6.543499 | 6.470597 | 6.101047 | 9.366494 | 8.606777 | 7.985128 | 11.23157 | 9.883543 |
| Cacna2d4 | 0 | 0.017114 | 0 | 0.013938 | 0.081777 | 0.026525 | 0 | 0.026814 |
| Gpr157 | 8.089045 | 8.518553 | 9.477944 | 5.05863 | 5.596834 | 5.383213 | 7.144471 | 7.427912 |
| Amz1 | 2.917059 | 2.395535 | 2.550779 | 3.858517 | 3.917281 | 3.815894 | 4.158301 | 4.107664 |
| Snx1 | 10.92248 | 10.83905 | 11.47649 | 14.10952 | 13.06673 | 12.90496 | 13.02844 | 13.98386 |
| Rab28 | 76.27427 | 72.17402 | 73.32959 | 67.22393 | 67.78903 | 67.83814 | 65.82881 | 65.57485 |
| Pcdh7 | 12.00052 | 12.04741 | 12.6647 | 10.54867 | 10.11341 | 10.07568 | 10.57779 | 10.30144 |
| Rbm28 | 3.479918 | 3.748232 | 3.279121 | 3.402644 | 3.302706 | 3.757366 | 3.706169 | 4.175421 |
| Epha6 | 0 | 0.025998 | 0 | 0.042346 | 0 | 0 | 0 | 0 |
| AC142178. | 0 | 0 | 0.0465 | 0 | 0 | 0 | 0 | 0 |
| Slc28a1 | 0.208177 | 0.085652 | 0.243808 | 0.43597 | 0.327416 | 0.315278 | 0.550488 | 0.503229 |
| AABR07049 | 4.84244 | 4.204809 | 6.165863 | 4.773428 | 5.114291 | 7.10934 | 7.6853 | 7.486213 |
| Chek1 | 12.31163 | 12.44692 | 11.72102 | 13.83685 | 13.65327 | 14.15551 | 15.8342 | 17.14808 |
| AC115401. | 0.396712 | 0.897721 | 0.425895 | 0.731108 | 0.857917 | 1.043511 | 0.443823 | 1.406501 |
| Pdk2 | 223.6247 | 222.6216 | 232.7726 | 184.2213 | 172.5044 | 176.043 | 189.1079 | 193.6593 |
| Mthfd1l | 1.584721 | 1.983449 | 2.062637 | 2.481147 | 2.320098 | 2.201043 | 3.075146 | 2.858955 |
| Sftpb | 0 | 0 | 0 | 0.0316 | 0 | 0 | 0 | 0 |
| Ccdc109b | 0.514653 | 0.485255 | 0.50647 | 0.908945 | 0.881104 | 0.488852 | 0.52779 | 0.760271 |
| Ybx2 | 5.236592 | 7.109949 | 6.20844 | 4.951191 | 4.923697 | 5.230259 | 6.724501 | 6.619114 |
| Srcin1 | 0.646087 | 0.693847 | 0.881715 | 0.52471 | 0.497312 | 0.633701 | 0.722814 | 0.650308 |
| AABR07006 | 0 | 0 | 0 | 0 | 0 | 0 | 0.580046 | 0 |
| Rad54l2 | 3.046052 | 2.881029 | 3.040201 | 1.973543 | 2.341581 | 2.107413 | 2.209737 | 2.72096 |
| Olr1392 | 0 | 0 | 0 | 0 | 0 | 0 | 0 | 0.049588 |
| Rcc2 | 6.362215 | 6.183367 | 7.924834 | 5.599468 | 6.923469 | 6.7942 | 8.167177 | 7.735753 |
| AABR07011 | 9.282591 | 9.703688 | 11.59026 | 6.22921 | 6.655054 | 6.281172 | 8.127353 | 7.065023 |
| AC121615. | 1.32572 | 0.749995 | 0 | 0 | 0 | 0.581196 | 0.741578 | 1.175051 |
| Rn60_16_0 | 0 | 0 | 0 | 0 | 0 | 0.074658 | 0 | 0 |
| Klhl10 | 0.025937 | 0.088038 | 0.027845 | 0.0239 | 0 | 0.022741 | 0 | 0.045978 |
| Golga5 | 8.18472 | 7.348606 | 6.775906 | 9.249143 | 8.872034 | 9.354273 | 8.63281 | 8.589934 |
| Slc2a6 | 1.379347 | 1.086892 | 1.692362 | 1.656857 | 1.598001 | 1.619749 | 1.460483 | 2.336008 |
| Lmf1 | 7.425381 | 7.275782 | 8.414488 | 7.982562 | 6.874466 | 7.233978 | 7.574199 | 8.603247 |
| Hsd11b2 | 0.140542 | 0.09541 | 0.271585 | 0.025901 | 0.09118 | 0.123227 | 0.031446 | 0.074742 |
| Ppp2r2b | 0.449345 | 0.234652 | 0.296862 | 1.114759 | 0.523244 | 1.15165 | 0.618716 | 0.888463 |
| AABR07043 | 0 | 0 | 0 | 0 | 0 | 0 | 0 | 0 |
| Prlhr | 0 | 0 | 0 | 0 | 0.093591 | 0 | 0 | 0 |

| | | | | | | | | |
|---|---|---|---|---|---|---|---|---|
| Haus4 | 2.873533 | 2.142012 | 1.379141 | 2.336336 | 2.887785 | 2.756648 | 2.042332 | 1.677994 |
| Trmt1 | 11.00188 | 11.16627 | 11.18958 | 11.06574 | 9.500604 | 10.24246 | 12.30841 | 11.26891 |
| Txndc15 | 17.21431 | 17.60737 | 18.70243 | 18.27338 | 18.46467 | 18.83671 | 16.52388 | 18.79773 |
| Rnf165 | 0.032546 | 0.036824 | 0.03494 | 0 | 0.070382 | 0.057072 | 0.072821 | 0.028847 |
| Gbx1 | 0 | 0.064718 | 0 | 0 | 0 | 0.025076 | 0 | 0 |
| Nek2 | 0.31451 | 0.355853 | 0.084412 | 0.434713 | 0.170038 | 0.585996 | 0.087965 | 0.278766 |
| Adad1 | 0.026676 | 0.030183 | 0.028639 | 0 | 0.05769 | 0 | 0 | 0.118223 |
| AABR07037 | 0 | 0 | 0 | 0 | 0.943709 | 0 | 0.976411 | 0 |
| AABR07028 | 0 | 0.058202 | 0 | 0 | 0 | 0 | 0 | 0.045594 |
| Rs1 | 0.077579 | 0 | 0.166572 | 0 | 0.083885 | 0 | 0 | 0 |
| Tspyl5 | 0.806936 | 0.624691 | 0.683919 | 0.860964 | 0.688838 | 0.670284 | 1.235362 | 0.828158 |
| Nox1 | 0 | 0 | 0.043699 | 0.018754 | 0 | 0.053534 | 0.091076 | 0 |
| AABR07063 | 1.454609 | 3.84025 | 1.561616 | 2.233941 | 2.09713 | 2.125671 | 2.169802 | 1.719056 |
| Atp5g2 | 611.3848 | 628.217 | 617.7671 | 575.7794 | 573.2347 | 587.1444 | 514.3583 | 513.9923 |
| Taldo1 | 33.40074 | 36.11494 | 35.22156 | 41.77778 | 42.02059 | 42.94508 | 38.36183 | 37.33423 |
| LOC100911 | 0.153117 | 0 | 0 | 0.141091 | 0 | 0 | 0 | 0 |
| Bahcc1 | 2.180236 | 2.360571 | 2.463056 | 2.144996 | 2.125339 | 2.276314 | 2.559233 | 2.342726 |
| Ms4a1 | 0.170019 | 0.240461 | 0.091263 | 0.234999 | 0.229799 | 0.372683 | 0 | 0.188371 |
| Bin2a | 0 | 0 | 0 | 0 | 0 | 0.035251 | 0.067468 | 0.017817 |
| Phc1 | 4.585527 | 4.749647 | 5.238608 | 4.656545 | 4.380041 | 4.219866 | 4.830954 | 5.059739 |
| Bcdin3d | 3.371563 | 3.476748 | 3.02396 | 2.83148 | 3.184151 | 3.554911 | 3.533224 | 2.874901 |
| AABR07027 | 0 | 0.452287 | 0 | 0 | 1.296699 | 0.350492 | 0 | 0 |
| AC121204. | 1.732696 | 1.089147 | 1.240107 | 0.887006 | 0 | 0.168803 | 0.215385 | 0.682566 |
| Kansl1 | 7.242095 | 7.067164 | 6.633091 | 6.144419 | 7.410941 | 6.823526 | 7.894388 | 8.169673 |
| AABR07072 | 0.18374 | 0 | 0 | 0.112873 | 0.066225 | 0.053701 | 0.06852 | 0 |
| Fendrr | 0 | 0 | 0 | 0.064423 | 0 | 0.061301 | 0.078217 | 0.371812 |
| Aurkc | 2.930223 | 2.780666 | 3.348735 | 3.919478 | 3.832751 | 3.688077 | 3.701197 | 3.853913 |
| Rab1a | 130.6297 | 117.2088 | 113.9668 | 124.7926 | 127.8888 | 121.1609 | 124.9842 | 126.7344 |
| Sesn1 | 28.87607 | 29.68122 | 29.39407 | 26.10265 | 26.69347 | 27.50496 | 28.88317 | 29.35166 |
| Zfp148 | 7.416633 | 7.480365 | 7.47967 | 7.403646 | 7.958744 | 7.701679 | 7.144983 | 7.287544 |
| LOC100910 | 0 | 0.064332 | 0 | 0 | 0 | 0 | 0 | 0 |
| #N/A | 7.190469 | 5.950831 | 5.973691 | 5.127335 | 5.412244 | 5.970443 | 6.253579 | 5.607576 |
| Smpx | 281.7897 | 280.3063 | 275.7337 | 263.4989 | 262.1013 | 252.4267 | 265.1416 | 258.0897 |
| Olr1193 | 0 | 0.061526 | 0 | 0 | 0 | 0 | 0 | 0 |
| Slc5a6 | 9.052717 | 9.305889 | 8.355686 | 8.612998 | 7.421011 | 7.679303 | 8.769173 | 8.757764 |
| Cyb561 | 1.816498 | 1.635386 | 1.929157 | 1.943804 | 2.048632 | 1.986602 | 2.31629 | 2.75267 |
| AABR07032 | 0 | 0 | 0 | 0.221853 | 0 | 0 | 0 | 0 |
| Mzt2b | 14.10515 | 13.32383 | 14.35554 | 13.47431 | 11.24055 | 11.72446 | 11.14749 | 11.69921 |
| Cdrt4 | 0 | 0 | 0 | 0 | 0 | 0 | 0.476298 | 0 |
| Nos1ap | 2.649369 | 3.293699 | 3.757241 | 2.019338 | 2.298853 | 1.720718 | 3.256737 | 3.623869 |
| AABR07012 | 0.272739 | 1.234366 | 0.585606 | 0 | 0 | 0 | 0.305128 | 0 |
| Asb10 | 14.45312 | 15.59562 | 17.18231 | 10.68242 | 10.33089 | 10.48483 | 12.49307 | 13.37687 |
| Nxph4 | 0.100446 | 0.2273 | 0.14378 | 0.246819 | 0.072407 | 0.117428 | 0.112375 | 0.118707 |
| AABR07020 | 0.719649 | 0.485084 | 0.50958 | 0.691346 | 0.910596 | 0.362483 | 0.479641 | 0.665003 |
| Vars2 | 11.6441 | 11.53097 | 13.80429 | 9.450821 | 9.777055 | 10.05746 | 12.3234 | 11.9736 |
| Kif21b | 0.613093 | 0.638631 | 0.794012 | 0.609778 | 0.620838 | 0.563159 | 0.773 | 0.681425 |
| Syt1 | 0.051933 | 0.039173 | 0.055754 | 0.047854 | 0.037436 | 0.015178 | 0.096834 | 0.092062 |
| Ophn1 | 12.78263 | 11.2965 | 11.1516 | 18.39985 | 17.33843 | 18.37022 | 19.03931 | 20.08987 |
| AABR07024 | 0.793423 | 0 | 0 | 0 | 0 | 0 | 0 | 0 |
| Rn60_1_04 | 0 | 0.109317 | 0.311171 | 0.178056 | 0.522348 | 0.338852 | 0 | 0.513814 |
| Nkx2-3 | 0 | 0 | 0 | 0 | 0 | 0 | 0 | 0.031532 |
| Gtpbp2 | 9.002231 | 9.653021 | 9.875025 | 10.04822 | 10.60347 | 10.33506 | 12.11189 | 11.50802 |
| Slc30a6 | 2.952864 | 2.819949 | 2.733837 | 4.143828 | 3.310059 | 3.254156 | 3.515686 | 3.193549 |
| C5ar1 | 2.55116 | 3.585359 | 2.882983 | 4.181938 | 4.64595 | 3.673159 | 5.618119 | 5.593544 |

| | | | | | | | | |
|---|---|---|---|---|---|---|---|---|
| Hepacam2 | 0.027035 | 0.091765 | 0.029023 | 0.049823 | 0.029232 | 0 | 0.030245 | 0.023962 |
| Fuca1 | 18.82784 | 16.6795 | 18.087 | 24.15553 | 24.53869 | 24.50059 | 24.43958 | 25.24103 |
| AABR07034 | 0 | 0.289022 | 0 | 0 | 0 | 0 | 0 | 0 |
| Mir155 | 2.416889 | 0 | 0 | 0 | 0.871116 | 0 | 0 | 0 |
| Kctd9 | 8.750133 | 8.12631 | 7.457142 | 7.876478 | 7.638388 | 7.775602 | 8.600968 | 9.42935 |
| Mllt4 | 9.815534 | 9.991891 | 9.459533 | 7.647564 | 8.771752 | 7.492719 | 9.373106 | 10.15727 |
| Mtmr9 | 6.85291 | 6.490824 | 6.873999 | 6.378471 | 6.352724 | 6.266584 | 7.1924 | 7.239806 |
| Kbtbd7 | 0 | 0 | 0 | 0 | 0 | 0 | 0 | 0.026402 |
| Tnik | 9.024592 | 8.853839 | 8.975904 | 5.847889 | 6.194858 | 5.901395 | 5.601825 | 5.903737 |
| Hist3h2ba | 0.831205 | 0.282141 | 0.17847 | 0.765923 | 0.89877 | 0.437281 | 0.836924 | 0.221022 |
| AABR07024 | 0 | 0 | 0.344897 | 0 | 0 | 0 | 0.359415 | 0 |
| Jph3 | 0.072609 | 0.065723 | 0.140312 | 0.133813 | 0.141321 | 0.140061 | 0.097479 | 0.0901 |
| Rps29 | 11.0244 | 18.3639 | 20.71195 | 12.98038 | 12.91391 | 12.61977 | 10.96321 | 11.67149 |
| Ttll3 | 0.756932 | 0.908338 | 1.20661 | 0.908841 | 0.843261 | 1.005573 | 1.231741 | 1.606109 |
| Mir21 | 2.276779 | 1.932051 | 0.611067 | 3.146944 | 4.923697 | 3.493493 | 2.547159 | 1.009011 |
| Mettl14 | 7.598192 | 6.854363 | 7.010735 | 8.174649 | 8.482274 | 8.246602 | 8.937033 | 8.154394 |
| Zmat1 | 4.215213 | 4.433018 | 4.641334 | 4.083341 | 4.616283 | 4.382962 | 4.836711 | 4.694141 |
| Gpx7 | 7.146937 | 8.340713 | 8.879092 | 12.79847 | 13.07421 | 12.13877 | 15.28733 | 12.94826 |
| Myoz3 | 0.276483 | 0.187697 | 0 | 0.152861 | 0.059791 | 0 | 0.309317 | 0.196049 |
| Ccdc127 | 19.36079 | 16.90147 | 16.97744 | 15.14879 | 15.74602 | 16.82921 | 14.9846 | 15.45918 |
| Lrrc30 | 0 | 0 | 0.311286 | 0.106873 | 0.12541 | 0.101693 | 0.194633 | 0.102801 |
| Fpgs | 4.920818 | 4.942427 | 4.350551 | 5.74673 | 6.003694 | 6.252676 | 7.595399 | 6.297447 |
| Dhx38 | 4.756236 | 5.622418 | 5.144229 | 5.124034 | 4.592743 | 4.937934 | 6.234383 | 6.13477 |
| Pabpc1 | 49.45925 | 49.77172 | 47.6675 | 52.11254 | 50.11389 | 49.24569 | 61.65729 | 60.55391 |
| AABR07064 | 0 | 0 | 0 | 0.196151 | 0.230173 | 0.186644 | 1.190745 | 0.188677 |
| Sgo1 | 0.02361 | 0.173635 | 0.101385 | 0.130531 | 0.063822 | 0.144906 | 0.066033 | 0.104631 |
| AC130555. | 0 | 1.545641 | 0.488854 | 0 | 0.984739 | 0.798513 | 0 | 2.018023 |
| Lsmem1 | 14.12115 | 14.64596 | 13.45212 | 10.92372 | 11.07473 | 11.36876 | 9.87382 | 9.155423 |
| Snx32 | 2.981228 | 3.275348 | 2.829462 | 4.459036 | 4.484951 | 4.280806 | 5.558776 | 5.284821 |
| Habp2 | 0.969739 | 0.647075 | 0.480497 | 0.526981 | 0.645271 | 0.523242 | 1.001447 | 0.418744 |
| Polr3h | 9.607632 | 9.548485 | 8.688261 | 8.813176 | 8.095615 | 9.97976 | 9.296079 | 8.784235 |
| AC134759. | 0.215794 | 0.162774 | 0.463336 | 0.331409 | 0.233335 | 0.126139 | 0 | 0.255025 |
| AABR07019 | 0 | 0 | 0 | 0 | 0 | 0 | 0.305128 | 0 |
| AC121204. | 0.106435 | 0.240852 | 0.228529 | 0.196151 | 0.115086 | 0.373289 | 0 | 0.377354 |
| Pgm2l1 | 1.630068 | 1.743482 | 1.804669 | 1.384696 | 1.500937 | 1.630232 | 2.137087 | 2.031764 |
| Snapc4 | 2.11976 | 2.590873 | 2.669028 | 2.254707 | 2.433551 | 2.501089 | 2.883853 | 2.412349 |
| Il6 | 0 | 0.113396 | 0 | 0.184701 | 0.216737 | 0.395436 | 0.280309 | 0.310911 |
| Timp1 | 10.26089 | 10.08844 | 10.33199 | 31.0385 | 31.60148 | 29.96851 | 30.40069 | 34.87361 |
| Prrx2 | 0.256068 | 0.289729 | 0.733081 | 3.224741 | 2.630386 | 3.25555 | 4.201671 | 4.236696 |
| Gabrr2 | 0.947574 | 0.733566 | 0.749575 | 1.102929 | 1.051561 | 0.874562 | 0.864821 | 1.016699 |
| AABR07016 | 0.200636 | 0 | 0 | 0.184878 | 0 | 0 | 0 | 0.177833 |
| Zc3h12c | 2.301799 | 1.953283 | 2.251915 | 2.035492 | 2.007179 | 1.660148 | 2.222105 | 2.665421 |
| Rala | 27.23243 | 27.58485 | 25.65351 | 30.54875 | 30.14436 | 28.73784 | 30.46643 | 28.57379 |
| Scgb3a1 | 0.126896 | 0.239296 | 0.04541 | 0.11693 | 0.091474 | 0.222526 | 0.141966 | 0.187458 |
| Ythdc2 | 1.682604 | 3.234891 | 1.923871 | 1.928613 | 2.248334 | 2.362893 | 2.678244 | 1.879376 |
| Kdm2a | 10.26011 | 9.879297 | 9.788242 | 9.802863 | 9.249262 | 9.253295 | 12.61 | 11.83115 |
| LOC103690 | 0.123797 | 0 | 0 | 0 | 0 | 2.713622 | 0 | 0 |
| Kcna10 | 0 | 0 | 0 | 0 | 0 | 0 | 0 | 0.026447 |
| Mob3a | 4.980167 | 4.939463 | 5.301025 | 6.170777 | 5.889108 | 5.927447 | 7.4683 | 6.442808 |
| Agbl2 | 0.162186 | 0.183506 | 0.108823 | 0.168129 | 0.109606 | 0.12443 | 0.204128 | 0.251569 |
| #N/A | 5.473505 | 5.499766 | 5.306082 | 6.4739 | 5.300079 | 4.405212 | 5.712233 | 6.37204 |
| Sik2 | 5.184226 | 5.450961 | 5.543111 | 5.182387 | 4.937483 | 5.252617 | 7.03016 | 6.674408 |
| AABR07034 | 0 | 0 | 0.160166 | 0 | 0.161318 | 0 | 0 | 0 |
| Tmem196 | 20.16589 | 19.11799 | 19.1269 | 19.88112 | 20.78884 | 19.65204 | 14.4233 | 13.88995 |

| | | | | | | | | |
|---|---|---|---|---|---|---|---|---|
| Ggta1l1 | 0 | 0 | 0 | 0 | 0 | 0 | 0 | 0.048048 |
| Car2 | 12.93183 | 14.41499 | 11.68926 | 8.591412 | 8.010014 | 8.482981 | 9.073479 | 8.943285 |
| #N/A | 31.52291 | 30.82667 | 28.81797 | 29.65557 | 32.90563 | 31.39 | 34.17439 | 32.39347 |
| Ube2r2 | 64.73766 | 66.55673 | 69.37471 | 57.3947 | 52.66819 | 55.67046 | 64.54759 | 65.27688 |
| S1pr1 | 43.89287 | 45.21496 | 45.78795 | 35.64088 | 35.32575 | 35.73508 | 55.91862 | 53.74408 |
| AABR07035 | 0 | 0 | 0 | 0 | 0 | 0 | 0 | 0.041777 |
| Thap12 | 13.13051 | 13.51399 | 10.74414 | 12.21434 | 13.33685 | 12.89548 | 12.97806 | 12.84218 |
| Mdm4 | 14.96169 | 14.71089 | 13.21734 | 12.3876 | 13.5512 | 11.56939 | 13.98991 | 13.79957 |
| AABR07026 | 0.189474 | 0.267976 | 0.152559 | 0.065472 | 0.204876 | 0.394561 | 0.132485 | 0.230918 |
| AC096601. | 24.07143 | 22.61677 | 24.63323 | 15.69524 | 13.85121 | 11.3552 | 20.47314 | 18.21645 |
| AABR07073 | 0 | 0.811638 | 0 | 0 | 0 | 0 | 0 | 0 |
| Rab23 | 4.091864 | 3.917482 | 3.841545 | 4.793257 | 4.764818 | 4.633572 | 4.985535 | 5.697195 |
| Slc39a5 | 1.740593 | 1.613231 | 1.828879 | 1.945141 | 1.341481 | 1.57486 | 1.88515 | 2.346986 |
| #N/A | 8.559501 | 8.727253 | 8.036157 | 8.277107 | 7.319753 | 7.818874 | 12.23396 | 11.68296 |
| Cd36 | 227.3157 | 238.173 | 165.3108 | 204.9592 | 242.3433 | 201.109 | 237.2849 | 204.6009 |
| LOC100909 | 1.781154 | 4.164936 | 2.16714 | 6.346217 | 2.182727 | 12.07728 | 3.055436 | 0.841987 |
| Chpf2 | 6.808036 | 6.700531 | 7.233771 | 8.894389 | 8.924474 | 10.09873 | 10.01626 | 8.948124 |
| Ldah | 2.516484 | 3.281983 | 2.30977 | 3.221596 | 3.24032 | 3.217046 | 2.965768 | 3.60964 |
| 43348 | 1.826206 | 1.867108 | 2.007791 | 3.34528 | 3.735183 | 3.5304 | 4.406158 | 5.050992 |
| Slco1a1 | 0 | 0 | 0 | 0 | 0 | 0.020406 | 0 | 0 |
| Timm10 | 56.4818 | 59.13736 | 59.14619 | 53.69075 | 51.79673 | 57.48007 | 57.91893 | 50.72193 |
| Glrx | 7.393316 | 8.596272 | 6.753196 | 6.511539 | 6.050924 | 7.664352 | 6.626191 | 6.661678 |
| AABR07056 | 0.163644 | 0.555465 | 0 | 0.150791 | 0.176945 | 0 | 0.549231 | 0.290091 |
| Lrp2bp | 0 | 0 | 0.037305 | 0 | 0 | 0 | 0 | 0 |
| Hoxc6 | 0.042333 | 0 | 0.090894 | 0 | 0.091548 | 0.037118 | 0.04736 | 0.112566 |
| Rn50_1_17 | 0.124385 | 0 | 0 | 0 | 0 | 0 | 0 | 0 |
| Cklf | 1.415295 | 0.978597 | 0.928528 | 1.231687 | 0.935207 | 1.654576 | 1.407439 | 1.463521 |
| #N/A | 0.053617 | 0.020222 | 0.038374 | 0.082343 | 0.096625 | 0 | 0.079979 | 0.047523 |
| Tmem50b | 40.22276 | 38.3395 | 37.44567 | 38.32117 | 39.59074 | 38.9178 | 35.37334 | 35.15941 |
| Klk1c3 | 0.092356 | 0.104497 | 0 | 0.085103 | 0.499317 | 0.080978 | 0.103324 | 0.245579 |
| Vwa5b1 | 0.031654 | 0.011938 | 0.011327 | 0 | 0.022818 | 0.018503 | 0.047217 | 0.028056 |
| Fat2 | 0 | 0 | 0 | 0 | 0.004337 | 0.01055 | 0.004487 | 0.003555 |
| Capn3 | 0.217614 | 0.201453 | 0.148669 | 0.109376 | 0.149738 | 0.225496 | 0.26559 | 0.192882 |
| Lrp3 | 0.376733 | 0.319692 | 0.404447 | 0.371941 | 0.363711 | 0.259537 | 0.632209 | 0.560504 |
| Mcm6 | 2.208093 | 2.173365 | 2.062168 | 2.580558 | 2.096411 | 2.502709 | 2.932246 | 2.625436 |
| Cd40 | 2.014074 | 2.149839 | 1.59108 | 2.346125 | 3.163958 | 2.132458 | 2.168228 | 2.492507 |
| Gna14 | 0.153341 | 0.216873 | 0.246932 | 0.194284 | 0.165805 | 0.201674 | 0.257326 | 0.254838 |
| Adarb2 | 0 | 0.02531 | 0 | 0.020612 | 0.048375 | 0 | 0 | 0 |
| Cdk20 | 3.883538 | 4.701903 | 5.151782 | 4.079977 | 4.466679 | 3.903924 | 4.815178 | 4.253385 |
| Cd226 | 0.61739 | 0.428654 | 0.451914 | 0.362028 | 0.197238 | 0.369088 | 0.392448 | 0.335796 |
| Pagr1 | 10.4457 | 12.20759 | 10.40257 | 12.15827 | 11.74063 | 12.83436 | 10.07164 | 8.04031 |
| Catsperd | 0.207651 | 0.277666 | 0.162129 | 0.313106 | 0.142883 | 0.148966 | 0.211192 | 0.301176 |
| AABR07069 | 0 | 0.580878 | 0 | 0 | 0 | 0.450142 | 0.574359 | 0.455044 |
| Gpr107 | 11.12279 | 11.56492 | 11.94102 | 12.24042 | 11.49652 | 11.92614 | 13.57088 | 12.91381 |
| Pmaip1 | 1.4783 | 1.69407 | 1.180113 | 1.571763 | 1.680436 | 1.32941 | 2.183938 | 2.183817 |
| Ccdc153 | 0.038905 | 0 | 0.083534 | 0.071699 | 0.042067 | 0.034112 | 0.043525 | 0.034483 |
| RGD131076 | 2.333597 | 2.886789 | 2.371651 | 4.271965 | 3.297092 | 3.928512 | 2.958822 | 3.116364 |
| AC120262. | 0 | 0 | 0 | 0 | 0.422556 | 0 | 0 | 0.346377 |
| Trabd | 31.89581 | 29.81017 | 32.95224 | 23.33141 | 24.16555 | 24.15443 | 24.34307 | 23.60421 |
| AABR07052 | 0 | 0 | 0 | 0 | 0.067168 | 0 | 0 | 0 |
| AABR07068 | 0 | 0 | 0 | 0.463972 | 0 | 1.324456 | 0 | 0 |
| RGD156505 | 5.952565 | 5.3221 | 4.290098 | 5.369983 | 6.481432 | 5.10972 | 4.47069 | 4.759517 |
| Mrpl1 | 6.911494 | 7.135065 | 8.882253 | 6.36867 | 11.61907 | 7.47548 | 5.192475 | 5.94714 |
| LOC100366 | 0 | 0 | 0 | 0.434713 | 0 | 0 | 0 | 0 |

| | | | | | | | | |
|---|---|---|---|---|---|---|---|---|
| Creb3 | 21.43117 | 20.80918 | 21.10763 | 26.09427 | 24.58773 | 26.3477 | 29.57213 | 25.64564 |
| Npap60 | 7.570977 | 7.495428 | 7.329648 | 7.059409 | 7.406731 | 7.230939 | 8.495281 | 7.66918 |
| AABR07027 | 0.247009 | 0 | 0 | 0.227609 | 0 | 0 | 0 | 0 |
| LOC100912 | 0.24511 | 1.485696 | 0.187958 | 0.080664 | 0.132517 | 0.046053 | 0.176283 | 0.248289 |
| Smim17 | 0 | 0.028748 | 0.027277 | 0.023412 | 0.054947 | 0.044556 | 0.028425 | 0.02252 |
| Acadvl | 233.0259 | 233.1898 | 230.861 | 194.3197 | 189.5736 | 186.6304 | 194.7443 | 189.9202 |
| AABR07065 | 0.149191 | 0.337604 | 0 | 0.137473 | 0 | 0 | 0 | 0 |
| Wnt7a | 0 | 0 | 0 | 0 | 0 | 0 | 0 | 0 |
| Mtmr4 | 5.127187 | 5.601854 | 5.136078 | 5.237106 | 5.343449 | 5.170257 | 6.503645 | 6.738652 |
| Htra2 | 14.69605 | 15.30444 | 15.55176 | 14.95129 | 14.00969 | 16.33041 | 17.11236 | 14.96642 |
| Zbtb6 | 2.891103 | 2.385724 | 2.286999 | 2.363582 | 2.632512 | 2.439624 | 3.307395 | 2.678131 |
| Dnph1 | 3.658163 | 5.058825 | 4.872726 | 3.495699 | 3.662517 | 2.732298 | 2.879971 | 3.062277 |
| Lrrc14 | 5.776212 | 6.019551 | 5.629976 | 5.836131 | 5.889618 | 6.352948 | 6.972341 | 7.050875 |
| AABR07033 | 0.187691 | 0.212364 | 0 | 0 | 0.608844 | 0 | 0 | 0.16636 |
| Gnl1 | 15.77426 | 18.16049 | 17.70106 | 18.43974 | 17.42997 | 18.95941 | 20.48145 | 19.71699 |
| Ltc4s | 4.450068 | 3.981197 | 3.888608 | 6.770697 | 6.154621 | 4.264784 | 3.820739 | 3.577404 |
| Hnrnpu | 65.41531 | 65.21264 | 63.39456 | 62.155 | 62.1209 | 60.61893 | 73.95608 | 74.96467 |
| Rln1 | 0 | 0 | 0.070983 | 0 | 0 | 0 | 0 | 0.117208 |
| Klhl14 | 0 | 0 | 0.027639 | 0 | 0 | 0.022573 | 0 | 0.034229 |
| Map6 | 1.769524 | 1.90033 | 2.318275 | 2.83273 | 2.334949 | 2.537656 | 3.388918 | 3.243168 |
| Acta2 | 46.079 | 49.96388 | 51.00454 | 84.78121 | 81.04631 | 81.62942 | 88.23499 | 90.49329 |
| LOC688684 | 47.04751 | 66.34718 | 69.10149 | 62.32697 | 64.87996 | 55.48 | 59.80518 | 54.99637 |
| Tcf7l2 | 2.733964 | 2.838405 | 2.999592 | 3.142129 | 2.842496 | 3.253264 | 3.461974 | 3.901163 |
| AABR07071 | 0 | 0.143115 | 0.271585 | 0 | 0.136769 | 0 | 0 | 0 |
| Rn50_X_11 | 0 | 0 | 0.262089 | 0.224956 | 0 | 0.107027 | 0 | 0.108192 |
| AC132627. | 0 | 0 | 0 | 0 | 0 | 0 | 0.623241 | 0 |
| Elf1 | 9.508488 | 9.296099 | 8.944362 | 9.82501 | 10.43112 | 9.713069 | 10.74095 | 10.39162 |
| AC113837. | 8.404407 | 8.777715 | 7.634565 | 10.72292 | 14.67991 | 13.60429 | 5.78614 | 8.595281 |
| Ptpn7 | 0.795041 | 0.966682 | 0.98092 | 0.896614 | 0.949482 | 0.957202 | 1.101864 | 1.104356 |
| Tmem161a | 9.268743 | 7.598975 | 8.620295 | 8.6778 | 8.092759 | 7.670522 | 9.371328 | 8.413043 |
| AABR07009 | 0 | 0 | 0 | 0.368345 | 0 | 0 | 0 | 0 |
| Tomm7 | 140.1067 | 141.5056 | 147.7222 | 128.8461 | 145.3211 | 125.7764 | 128.8551 | 132.0839 |
| Hk2 | 13.29393 | 13.6562 | 12.8954 | 13.40464 | 12.53993 | 12.24781 | 14.21476 | 14.16702 |
| AABR07017 | 0.043894 | 0.082774 | 0 | 0.013482 | 0.015821 | 0.012829 | 0.016369 | 0 |
| Cadm3 | 14.57946 | 14.88962 | 14.27437 | 25.309 | 23.02688 | 23.84298 | 31.67482 | 31.58026 |
| AABR07065 | 0 | 0 | 0.082552 | 0 | 0 | 0 | 0 | 0 |
| Babam1 | 19.68585 | 20.77697 | 21.25932 | 20.1473 | 17.83651 | 19.10261 | 20.32618 | 21.20723 |
| AABR07056 | 0 | 0.01155 | 0 | 0 | 0 | 0 | 0 | 0.009048 |
| Ctf1 | 6.217985 | 7.120121 | 6.474338 | 6.592524 | 5.791859 | 6.305852 | 6.998311 | 6.374526 |
| Atp6v1g2 | 0.254698 | 0.749265 | 0.109374 | 0.563266 | 0.660963 | 1.250589 | 0.797846 | 0.632104 |
| Clptm1l | 21.97013 | 21.24477 | 23.02708 | 19.07585 | 19.10275 | 20.83231 | 24.80726 | 23.52847 |
| Med16 | 14.85111 | 15.17392 | 17.72157 | 15.32688 | 13.81986 | 14.12632 | 15.74878 | 16.01931 |
| AABR07027 | 0.709565 | 0.695794 | 0.558627 | 0.784604 | 0.716093 | 0.871006 | 0.635064 | 0.754708 |
| AC105645. | 0 | 0.569707 | 0 | 0 | 1.088894 | 0 | 0 | 0 |
| Atad1 | 32.74203 | 29.49205 | 27.54804 | 31.566 | 32.28982 | 32.09338 | 30.47357 | 30.45775 |
| Ccnd1 | 18.13664 | 18.10452 | 18.60292 | 17.58365 | 15.53697 | 17.5874 | 16.502 | 17.34629 |
| Cldnd1 | 17.02023 | 15.68371 | 15.21758 | 16.50132 | 14.42378 | 15.58712 | 16.99715 | 17.76987 |
| Rn60_14_( | 0 | 0 | 0 | 0 | 0.081589 | 0 | 0 | 0 |
| AABR07049 | 0.079103 | 0 | 0 | 0.14578 | 0 | 0.069357 | 0.176993 | 0 |
| AABR07024 | 4.848696 | 5.266629 | 4.164308 | 4.467883 | 4.823399 | 4.591449 | 5.207526 | 4.297641 |
| Trps1 | 1.018258 | 1.087386 | 1.191427 | 1.518124 | 1.546387 | 1.715398 | 1.804771 | 1.977459 |
| Vps53 | 7.136459 | 7.057129 | 7.168483 | 6.999085 | 8.006179 | 6.760518 | 7.084956 | 6.749353 |
| Senp1 | 1.745531 | 1.522385 | 1.952019 | 1.474401 | 1.494205 | 0.956552 | 1.220514 | 1.805009 |
| Vipas39 | 12.14771 | 11.59698 | 11.81872 | 12.30134 | 12.90711 | 12.53726 | 13.6847 | 10.99144 |

| | | | | | | | | |
|---|---|---|---|---|---|---|---|---|
| Morn4 | 3.707322 | 3.670328 | 3.38304 | 3.757766 | 3.5744 | 3.9007 | 3.836518 | 3.121684 |
| Mir6332 | 0 | 0 | 0 | 0.797572 | 0 | 0.758917 | 0 | 0 |
| Wdfy4 | 0.86229 | 1.110819 | 1.070716 | 1.55084 | 1.387339 | 1.275276 | 1.388923 | 1.468726 |
| Kazald1 | 3.099339 | 2.703125 | 3.604617 | 5.801086 | 6.143996 | 6.14269 | 5.201105 | 5.8662 |
| Riok2 | 6.551784 | 6.373569 | 6.514662 | 7.618916 | 7.058208 | 6.931688 | 6.491376 | 7.028607 |
| Rn60_12_( | 0.10579 | 0.119696 | 0 | 0.682368 | 0.114389 | 0.185513 | 0.118353 | 0.468834 |
| Phf10 | 13.53742 | 13.29872 | 11.48984 | 13.76565 | 11.84802 | 11.50655 | 14.89561 | 14.70922 |
| Dok5 | 0.694684 | 1.010574 | 0.852328 | 1.950853 | 1.538072 | 1.566255 | 0.962224 | 1.143504 |
| Ndufv3-ps | 17.61545 | 23.19249 | 24.75662 | 19.47833 | 18.87417 | 17.69182 | 16.12422 | 18.87808 |
| Champ1 | 3.054788 | 3.486146 | 3.647042 | 3.457919 | 3.260386 | 3.151786 | 3.859488 | 3.547904 |
| Rab11b | 115.3387 | 117.0368 | 116.6467 | 96.25987 | 93.05368 | 99.95681 | 116.8203 | 118.3866 |
| LOC68976( | 0 | 0.079851 | 0 | 0 | 0 | 0 | 0 | 0 |
| AABR07066 | 0 | 0.586629 | 0 | 0 | 0 | 0 | 0 | 0 |
| Abca3 | 7.011337 | 6.640344 | 6.569427 | 5.480033 | 6.328996 | 6.696434 | 7.598848 | 7.85135 |
| Rabgap1l | 11.59226 | 11.18559 | 10.58636 | 11.65289 | 12.04619 | 11.41812 | 13.02505 | 12.54326 |
| Syde2 | 3.380849 | 3.69046 | 3.277794 | 3.087871 | 2.673304 | 3.042683 | 4.198901 | 4.250704 |
| Nf2 | 18.04675 | 19.01903 | 16.99327 | 17.16719 | 16.62324 | 16.02811 | 20.66004 | 21.17934 |
| LOC69135 | 6.06923 | 5.959009 | 5.707974 | 4.205973 | 3.525348 | 5.321506 | 4.657593 | 4.31246 |
| Dab2 | 13.11655 | 12.48076 | 12.31589 | 15.26507 | 15.61402 | 14.38455 | 19.04955 | 19.28753 |
| Mat1a | 0.296504 | 0.354118 | 0.371369 | 0.197323 | 0.178114 | 0.173317 | 0.202715 | 0.204405 |
| Rnf31 | 8.705706 | 8.774036 | 8.969145 | 7.10517 | 7.435759 | 7.158503 | 7.578849 | 8.520352 |
| Auh | 55.85966 | 56.88686 | 55.2697 | 52.76762 | 50.54341 | 54.6819 | 54.85573 | 56.06048 |
| Olr202 | 0 | 0 | 0 | 0 | 0 | 0.047383 | 0 | 0 |
| Klhl17 | 3.979096 | 3.72294 | 4.052746 | 4.207166 | 4.743825 | 4.405823 | 6.021123 | 5.945942 |
| AABR07068 | 3.969897 | 3.453196 | 3.424331 | 2.918025 | 2.630143 | 3.319847 | 3.619824 | 3.132268 |
| LOC69033 | 0 | 0 | 0 | 0 | 0 | 0 | 0 | 0.097305 |
| Dock10 | 1.749241 | 1.43041 | 1.980354 | 1.963535 | 1.728079 | 2.251804 | 2.125985 | 2.417276 |
| Iqck | 0.242435 | 0.068576 | 0.195202 | 0 | 0 | 0.053142 | 0.339032 | 0.161162 |
| LOC10090 | 0.017543 | 0 | 0 | 0 | 0 | 0 | 0 | 0 |
| FAM187A | 0 | 0 | 0.034426 | 0 | 0 | 0 | 0 | 0 |
| Brinp2 | 0.063443 | 0.04307 | 0.06811 | 0.222149 | 0.15092 | 0.155755 | 0.042586 | 0.146205 |
| Fsip1 | 0.063016 | 0 | 0.033826 | 0.116133 | 0.034069 | 0.027626 | 0.070499 | 0 |
| Ube3a | 8.203349 | 7.830582 | 7.494889 | 8.78546 | 9.092571 | 8.115661 | 9.096325 | 9.244296 |
| Erich3 | 0.066879 | 0.037835 | 0.071798 | 0 | 0 | 0.02932 | 0.074821 | 0.029639 |
| RGD156111 | 6.201228 | 4.394107 | 5.043495 | 5.367873 | 7.111679 | 4.833104 | 6.09673 | 6.662371 |
| Nipal4 | 0.016039 | 0 | 0 | 0 | 0 | 0 | 0.017943 | 0.028432 |
| Avp | 0.455356 | 1.030427 | 0.879936 | 0.419592 | 0.787791 | 0.479108 | 0.509432 | 0.726488 |
| Josd1 | 6.349731 | 5.848295 | 6.297265 | 5.797512 | 5.672718 | 5.991798 | 6.778928 | 6.400227 |
| AABR07034 | 0 | 0 | 0.063957 | 0 | 0 | 0 | 0.066649 | 0 |
| Tmprss6 | 0.3143 | 0.296347 | 0.243693 | 0.353974 | 0.39649 | 0.229649 | 0.683716 | 0.27858 |
| Kitlg | 23.53586 | 22.19143 | 21.02659 | 18.02226 | 18.56768 | 18.63998 | 22.35669 | 23.07353 |
| AABR07004 | 1.434683 | 0 | 0.770112 | 0.991503 | 0.775651 | 1.886897 | 0 | 0 |
| Pdcd10 | 18.84667 | 16.41051 | 15.29931 | 17.28876 | 18.91976 | 16.48781 | 20.66029 | 20.14285 |
| AC109942. | 0.54125 | 0.765498 | 2.033732 | 1.122166 | 0.877868 | 0.237284 | 0.908289 | 0.719605 |
| Nmbr | 0 | 0.056698 | 0 | 0 | 0.054184 | 0 | 0.056062 | 0.044416 |
| Ankfy1 | 5.724446 | 5.363073 | 5.347027 | 6.397018 | 6.03206 | 6.010252 | 7.424042 | 7.032307 |
| Naca | 120.9368 | 123.4437 | 118.034 | 104.4044 | 103.693 | 99.03189 | 118.8899 | 125.1962 |
| Ptpn4 | 2.625727 | 2.550478 | 2.111505 | 2.652325 | 2.603457 | 2.502057 | 2.710303 | 2.779602 |
| Lrrc16a | 1.450383 | 1.985314 | 1.186864 | 3.271082 | 3.322995 | 3.281512 | 3.290636 | 3.26331 |
| AC129054. | 0.056126 | 0.063504 | 0.060255 | 0.155155 | 0.060689 | 0.049212 | 0 | 0.149243 |
| Rnmt | 5.751537 | 4.643159 | 4.711779 | 6.292618 | 5.568023 | 5.334694 | 5.831877 | 5.533228 |
| Fam25a | 0 | 0 | 0 | 0.120633 | 0 | 0.114786 | 0.146462 | 0 |
| Atp1a2 | 49.7415 | 52.02824 | 52.98408 | 34.87249 | 33.10341 | 35.23274 | 39.22019 | 40.17825 |
| Gfi1 | 0.152227 | 0.196842 | 0.140078 | 0.180348 | 0.117572 | 0.133472 | 0.072988 | 0.154201 |

| | | | | | | | | |
|---|---|---|---|---|---|---|---|---|
| LOC690422 | 0.499516 | 0.586111 | 0.685224 | 0.937616 | 1.160256 | 0.875952 | 1.003835 | 0.852696 |
| Usf1 | 12.79572 | 15.14704 | 13.97098 | 15.43412 | 13.46552 | 14.57689 | 15.53434 | 16.80526 |
| Tomm34 | 9.016454 | 10.36784 | 10.6887 | 11.58292 | 11.59126 | 12.43786 | 12.19008 | 12.2349 |
| AC108583. | 0 | 0 | 0 | 0 | 0 | 0 | 0 | 0.348981 |
| LOC100360 | 0 | 0 | 0.191218 | 0 | 0 | 0 | 0 | 0 |
| Fbxo27 | 7.576346 | 8.656321 | 8.532402 | 5.543977 | 5.541778 | 7.359343 | 10.63665 | 8.097852 |
| Rdh12 | 0.15641 | 0.212364 | 0.167916 | 0.17295 | 0.304422 | 0.219424 | 0.069994 | 0.08318 |
| AABR07030 | 0 | 0 | 0 | 0 | 0 | 0 | 0 | 0 |
| AC136013. | 0 | 0.580878 | 1.653475 | 2.36535 | 1.665368 | 3.150994 | 2.297438 | 1.820177 |
| Pdpk1 | 8.723009 | 9.270556 | 8.676566 | 6.420055 | 6.87064 | 6.695353 | 9.727736 | 9.633668 |
| AABR07064 | 0 | 0 | 0 | 0.643375 | 0 | 0 | 0 | 0 |
| AABR07067 | 0 | 0 | 0.066452 | 0 | 0 | 0 | 0 | 0 |
| Ephx3 | 0.716895 | 0.774263 | 0.629699 | 0.780698 | 0.810403 | 0.685717 | 0.692663 | 0.750951 |
| LOC501467 | 0.07273 | 0 | 0 | 0.067018 | 0 | 0 | 0.081368 | 0 |
| Prim1 | 3.773234 | 3.007027 | 3.451992 | 5.230446 | 4.150898 | 3.76867 | 4.147912 | 4.565839 |
| Rpl11 | 0.056277 | 0.116737 | 0.060417 | 0.086429 | 0.091278 | 0.090464 | 0.06296 | 0.124703 |
| Gipc1 | 18.10934 | 18.81574 | 18.88903 | 18.49506 | 16.72938 | 18.55757 | 17.95685 | 18.84521 |
| AABR07063 | 0 | 0 | 0 | 0 | 0 | 0.143933 | 0 | 0 |
| Col28a1 | 0.167986 | 0.224627 | 0.229529 | 0.323658 | 0.264205 | 0.254411 | 0.461297 | 0.243646 |
| Trip13 | 0.353824 | 0.37174 | 0.379852 | 0.442476 | 0.273275 | 0.310233 | 0.282745 | 0.515219 |
| Llgl1 | 5.138074 | 5.933532 | 5.629952 | 5.87976 | 5.244343 | 5.262558 | 6.545204 | 6.421459 |
| Slc5a1 | 0 | 0 | 0 | 0.016363 | 0 | 0 | 0 | 0.015739 |
| AABR07047 | 0.988036 | 0 | 1.06072 | 0 | 0 | 0 | 1.658057 | 0 |
| Slc35g3 | 0.321263 | 0.454368 | 0.344897 | 0.259028 | 0.130267 | 0.457736 | 0.044927 | 0.427127 |
| LOC685157 | 0.339763 | 0.048053 | 0.319162 | 0.508752 | 0.091845 | 0.223428 | 0.190056 | 0.075287 |
| LOC502684 | 0.291529 | 0.247389 | 0.117366 | 0.235054 | 0.433436 | 0.19171 | 0.244612 | 0.387595 |
| LOC103690 | 0.34565 | 0.391086 | 0.463846 | 0.637005 | 0.654055 | 0.151533 | 0.580046 | 0.382958 |
| LOC689479 | 0.086412 | 0 | 0.092769 | 0 | 0 | 0 | 0 | 0 |
| Pum3 | 6.922861 | 6.613764 | 5.494573 | 7.570579 | 8.446774 | 7.959456 | 7.986078 | 6.709094 |
| AABR07044 | 0 | 0 | 0 | 0 | 0.152621 | 0 | 0.15791 | 0 |
| RGD131200 | 0.16589 | 0.125131 | 0.118729 | 0.305722 | 0.269062 | 0.436357 | 0.216522 | 0.269567 |
| Tfap2b | 0.046188 | 0.039195 | 0 | 0.02128 | 0.024971 | 0.050622 | 0.012918 | 0.010235 |
| AABR07013 | 0 | 3.703098 | 0.702727 | 5.428478 | 4.246688 | 5.16538 | 0 | 3.481089 |
| Dbt | 38.95453 | 40.1453 | 36.21798 | 35.55817 | 36.37066 | 36.67622 | 32.33219 | 31.49294 |
| Ergic2 | 30.95007 | 32.73028 | 29.46607 | 28.98516 | 27.49121 | 26.37708 | 26.82774 | 28.71298 |
| AABR07039 | 0.581844 | 0.658329 | 1.749009 | 0.643375 | 0.754967 | 0.510161 | 0.781129 | 0.412573 |
| Foxp4 | 2.292995 | 2.906996 | 3.79632 | 3.411195 | 3.151504 | 4.045222 | 4.806075 | 4.603497 |
| Dcstamp | 0 | 0 | 0 | 0 | 0 | 0 | 0 | 0 |
| H1fx | 9.858179 | 12.2797 | 13.39915 | 13.41754 | 13.49552 | 11.26055 | 11.43362 | 12.26498 |
| RGD156296 | 0.828051 | 0.487188 | 0.711172 | 0.671454 | 0.895359 | 0.551787 | 0.741109 | 0.616512 |
| Perp | 33.21989 | 31.31178 | 32.97095 | 25.3207 | 24.7102 | 23.45817 | 26.25242 | 25.68978 |
| Whsc1 | 2.168894 | 2.15245 | 1.854864 | 1.99855 | 2.553876 | 2.4174 | 3.043359 | 2.785849 |
| Adh7 | 0.44005 | 0.263592 | 0.389053 | 1.288022 | 1.79132 | 1.634129 | 1.24525 | 1.00951 |
| 5S_rRNA | 1.892744 | 1.427701 | 4.063963 | 3.488178 | 5.457592 | 2.765933 | 1.411679 | 2.796055 |
| Ccdc113 | 0 | 0.052387 | 0.049707 | 0 | 0.050064 | 0.040596 | 0 | 0.041038 |
| RGD156461 | 0 | 0 | 0 | 0 | 0 | 0.017949 | 0 | 0 |
| Prkcq | 1.072181 | 0.843911 | 0.884143 | 1.131157 | 0.856899 | 0.899215 | 0.990898 | 1.06051 |
| Ckap5 | 7.359034 | 7.592335 | 7.67154 | 8.002334 | 8.237824 | 8.093952 | 8.243329 | 7.93565 |
| AABR07047 | 0 | 0 | 0.374788 | 0.160844 | 0 | 0.153048 | 0 | 0 |
| Tgm4 | 1.117828 | 1.021544 | 1.269293 | 1.109267 | 0.906518 | 1.168595 | 1.707517 | 1.962519 |
| Efhd2 | 13.81844 | 12.96348 | 15.18184 | 17.77144 | 16.68846 | 17.82537 | 22.60528 | 21.41192 |
| Zfp579 | 2.199222 | 2.57126 | 2.93814 | 2.679479 | 2.219455 | 2.635316 | 2.925133 | 2.967237 |
| LOC685067 | 4.628724 | 5.047887 | 4.370528 | 7.117209 | 7.296405 | 7.090096 | 7.892396 | 7.711038 |
| Tonsl | 0.209661 | 0.083725 | 0.198604 | 0.136372 | 0.186697 | 0.129763 | 0.331143 | 0.284215 |

| | | | | | | | | |
|---|---|---|---|---|---|---|---|---|
| Cox6b2 | 0.668501 | 0.630315 | 0.956905 | 1.129329 | 1.566155 | 1.953808 | 1.869723 | 2.863875 |
| Dcp1a | 3.440826 | 3.020003 | 3.03118 | 3.145489 | 2.797749 | 2.889556 | 3.47364 | 3.460167 |
| AABR07042 | 0.160878 | 0.091013 | 0 | 0.148243 | 0 | 0.141058 | 0 | 0.142595 |
| Mageb4 | 0 | 0 | 0.035158 | 0.060354 | 0 | 0.028715 | 0 | 0.029027 |
| Plbd2 | 23.98311 | 24.59263 | 26.10679 | 27.93834 | 31.12945 | 31.74179 | 37.23738 | 35.96595 |
| #N/A | 0 | 0.339539 | 0 | 0 | 0.162242 | 0 | 0.839322 | 0 |
| Foxo3 | 22.39496 | 24.52248 | 27.11104 | 19.33194 | 19.23425 | 18.249 | 29.24576 | 28.6309 |
| Pfkp | 6.516648 | 5.424628 | 5.771731 | 8.428214 | 8.229138 | 7.44835 | 8.436192 | 8.932216 |
| Ndufb8 | 622.5231 | 617.3722 | 627.8093 | 582.9389 | 583.0914 | 575.7872 | 599.8904 | 582.387 |
| Lipe | 30.22633 | 28.96895 | 31.14462 | 21.7556 | 21.14172 | 23.32912 | 33.78425 | 31.95384 |
| Timm9 | 17.78234 | 19.28733 | 14.48243 | 14.80366 | 18.16694 | 17.0432 | 15.91527 | 16.35922 |
| Ifnar1 | 11.85842 | 12.22987 | 11.61823 | 12.78853 | 11.84364 | 11.78917 | 12.72367 | 13.71972 |
| AABR07065 | 0 | 0 | 0 | 0 | 0 | 0 | 0 | 0.131114 |
| Sectm1b | 0.090286 | 0 | 0.193856 | 1.039938 | 0.878625 | 1.385351 | 0.959576 | 1.080338 |
| LOC688672 | 1.163687 | 1.170362 | 1.203022 | 1.628295 | 1.491293 | 1.24706 | 1.012574 | 1.337044 |
| Fam151b | 2.836807 | 4.077204 | 3.045493 | 3.85036 | 2.901593 | 4.403219 | 2.744816 | 2.854187 |
| Il10rb | 11.63365 | 12.57207 | 12.5829 | 11.8962 | 11.44998 | 12.89676 | 13.04767 | 12.60006 |
| Lrpprc | 42.84374 | 40.60403 | 39.77357 | 48.15279 | 48.28875 | 46.94353 | 37.71185 | 37.17022 |
| C2cd4a | 0 | 0 | 0 | 0.141505 | 0.055349 | 0.044882 | 0.171803 | 0.090742 |
| Blzf1 | 9.616697 | 9.520735 | 9.880523 | 9.622935 | 9.870352 | 8.563677 | 9.918207 | 8.956604 |
| Lrrc32 | 8.906929 | 9.85514 | 10.53386 | 8.340347 | 7.574254 | 7.476056 | 10.97729 | 10.91764 |
| Rergl | 1.304799 | 0.78737 | 0.6537 | 0.440851 | 0.70543 | 0.800834 | 0.535242 | 0.693905 |
| Nxph3 | 1.438159 | 1.722928 | 1.180672 | 1.169301 | 0.975725 | 1.483505 | 1.388113 | 1.499661 |
| Kif27 | 0.069806 | 0.026327 | 0.02498 | 0.021441 | 0 | 0.051005 | 0.06508 | 0.10312 |
| Ankle1 | 0.455922 | 0.663242 | 1.048846 | 0.390106 | 0.563408 | 0.456861 | 0.364333 | 0.54843 |
| #N/A | 0 | 0 | 0 | 0 | 0 | 0 | 0.046349 | 0.03672 |
| RGD156636 | 0 | 0.084562 | 0.05349 | 0.114779 | 0.053875 | 0.087373 | 0.055742 | 0.044162 |
| AC105662. | 5.454783 | 7.159324 | 7.261513 | 5.629532 | 6.841887 | 5.834966 | 5.24821 | 5.801815 |
| Rasd2 | 7.686185 | 8.148743 | 10.05462 | 6.134464 | 5.235251 | 6.64639 | 6.381514 | 6.571255 |
| Stx6 | 10.15865 | 10.53219 | 11.49917 | 10.35954 | 10.82354 | 11.44135 | 11.24616 | 11.84851 |
| Snhg11 | 0 | 0 | 0 | 0.022967 | 0 | 0 | 0 | 0 |
| Golga3 | 4.733901 | 4.79674 | 4.639792 | 5.214275 | 5.029592 | 4.632393 | 5.449736 | 5.153562 |
| Clock | 4.387673 | 4.142572 | 3.670683 | 4.036307 | 4.014431 | 4.438979 | 4.925151 | 5.345766 |
| Srrm2 | 31.33186 | 31.50347 | 32.84758 | 30.68331 | 32.30293 | 31.1185 | 38.63082 | 38.73878 |
| Ncam1 | 6.77021 | 6.072089 | 6.603473 | 20.92174 | 20.53319 | 20.90313 | 23.34612 | 24.86296 |
| P2rx4 | 7.309964 | 6.887387 | 6.563542 | 7.568639 | 8.277809 | 7.574725 | 7.166957 | 7.04464 |
| Gpr161 | 0.812066 | 1.113714 | 0.818968 | 0.793637 | 0.771641 | 0.71202 | 1.156276 | 0.98151 |
| Wdr35 | 2.372324 | 2.557027 | 2.479819 | 2.542666 | 2.133132 | 2.255218 | 2.318802 | 2.545384 |
| Plekhm1 | 4.224344 | 5.000091 | 4.744269 | 4.039456 | 4.108077 | 3.820383 | 4.617023 | 4.905982 |
| H2afy | 23.52266 | 23.52557 | 23.52444 | 21.48168 | 21.72548 | 21.48405 | 26.43359 | 23.33136 |
| AABR07027 | 0.312866 | 0.132747 | 0.062978 | 0.126128 | 0.169149 | 0.240031 | 0.043753 | 0.19065 |
| Olr1746 | 0 | 0 | 0 | 0 | 0 | 0 | 0 | 0.049908 |
| Gabra2 | 0.03057 | 0 | 0.032819 | 0.028169 | 0 | 0.053607 | 0.2736 | 0.216764 |
| LOC680579 | 0.167036 | 0.566982 | 0.268986 | 0.230876 | 0.090307 | 0.073229 | 0.093436 | 0.296105 |
| Tdrd3 | 5.073897 | 5.460071 | 5.003091 | 5.107362 | 5.784501 | 4.932362 | 5.583899 | 6.257039 |
| LOC102548 | 1.237358 | 0.871706 | 1.228127 | 1.032613 | 1.060252 | 1.207737 | 1.279826 | 1.034653 |
| Hnrnpdl | 44.83051 | 45.49802 | 44.4377 | 48.70111 | 51.25314 | 48.98966 | 48.36731 | 47.98424 |
| Cyp21a1 | 0.363092 | 0.365175 | 0.606359 | 0.2974 | 0.523475 | 0.318359 | 0.586749 | 0.572136 |
| Rpl35a | 4.919223 | 3.052251 | 3.236803 | 1.315995 | 3.774834 | 4.313179 | 1.775293 | 5.063402 |
| LOC102555 | 4.124885 | 2.902717 | 4.104304 | 0.741643 | 2.828406 | 2.911005 | 1.744596 | 2.586015 |
| Mettl6 | 5.287557 | 4.072507 | 4.479671 | 4.930977 | 4.752985 | 5.166776 | 5.345316 | 4.827787 |
| Plod3 | 12.66303 | 12.73327 | 13.49236 | 13.70389 | 13.77471 | 14.52567 | 18.85361 | 17.92444 |
| Nphp1 | 22.08483 | 22.967 | 24.30087 | 18.44294 | 17.5445 | 17.10999 | 18.11512 | 18.06571 |
| Ppid-ps1 | 0 | 0 | 0 | 0 | 0 | 0 | 0 | 0 |

| | | | | | | | | |
|---|---|---|---|---|---|---|---|---|
| AABR07018 | 0 | 0 | 0 | 0 | 0 | 0 | 0 | 0.780076 |
| LOC100911 | 0.651945 | 2.117756 | 2.98024 | 0.910802 | 3.456475 | 0.682263 | 5.670243 | 2.0318 |
| Bgn | 44.24985 | 43.32595 | 42.9631 | 164.6494 | 159.1312 | 162.8393 | 189.7361 | 193.0239 |
| Adamts13 | 2.574098 | 2.424392 | 2.550885 | 3.551379 | 3.456229 | 3.242846 | 3.939928 | 4.268506 |
| AABR07030 | 0.55857 | 0.473997 | 0.29983 | 0.900725 | 0.603973 | 1.714141 | 0.781129 | 0.866404 |
| Krt75 | 0.137012 | 0.062009 | 0.029418 | 0.0505 | 0.088889 | 0.024026 | 0 | 0.12144 |
| Tbp | 4.151128 | 3.61781 | 3.101484 | 3.876792 | 3.214776 | 3.861047 | 4.926509 | 4.375445 |
| Tyw1 | 3.986467 | 3.831735 | 4.549807 | 4.475808 | 4.18496 | 4.394624 | 4.416582 | 5.111429 |
| Daam1 | 5.289672 | 4.866708 | 4.273838 | 5.582589 | 5.689959 | 5.777426 | 5.979281 | 6.424379 |
| Mir25 | 3.117019 | 1.410704 | 2.007791 | 4.595537 | 2.69631 | 1.093202 | 6.276928 | 3.315323 |
| AC110474. | 0 | 0 | 0 | 0 | 0 | 0 | 0.492308 | 0 |
| AABR07047 | 0 | 0 | 0 | 0.043122 | 0 | 0 | 0 | 0 |
| Qser1 | 2.290647 | 2.191513 | 1.986001 | 2.100053 | 2.545818 | 2.166065 | 2.355065 | 2.333576 |
| Eml1 | 25.7822 | 23.40257 | 25.67105 | 20.67992 | 19.21121 | 19.11151 | 23.2894 | 23.18752 |
| Pafah1b3 | 2.443743 | 1.410704 | 1.766857 | 1.792259 | 1.617786 | 2.230132 | 2.008617 | 2.652258 |
| Rundc3a | 0.761434 | 0.469924 | 0.817448 | 1.020555 | 1.272416 | 0.971093 | 1.316509 | 1.165731 |
| Slc4a1ap | 32.58487 | 31.99311 | 30.43508 | 23.10015 | 24.38024 | 23.56901 | 25.52637 | 25.40972 |
| Chchd7 | 44.45014 | 37.20322 | 45.10527 | 40.21095 | 34.89527 | 38.44003 | 33.83378 | 31.48272 |
| Il17b | 0.22834 | 0.086119 | 0.6537 | 0.280541 | 0.411501 | 0.266945 | 0.255456 | 0.202389 |
| Rgs12 | 2.826504 | 3.145413 | 3.696263 | 2.926056 | 2.892754 | 2.998414 | 3.552557 | 3.3816 |
| Zfp518b | 0.879978 | 0.652888 | 0.867277 | 0.717815 | 0.811121 | 0.632431 | 0.968342 | 1.035696 |
| Pcdhb7 | 0.126488 | 0.166967 | 0.158425 | 0.369086 | 0.547077 | 0.351198 | 0.306603 | 0.261596 |
| Rnd2 | 3.265581 | 3.782824 | 3.380602 | 4.907706 | 4.245638 | 4.499415 | 5.00166 | 5.203112 |
| Rps21-ps1 | 18.93112 | 19.00132 | 20.32377 | 18.7104 | 19.97471 | 18.33902 | 17.08008 | 18.13279 |
| AABR07067 | 0 | 0 | 0 | 0 | 0.125828 | 0 | 0 | 0.103143 |
| Edrf1 | 2.34489 | 2.731166 | 2.390462 | 2.578343 | 2.301122 | 2.557044 | 2.557223 | 2.977865 |
| Fnta | 19.02234 | 18.2469 | 18.08206 | 20.25369 | 20.60225 | 19.51775 | 20.02746 | 18.98525 |
| 5S_rRNA | 0 | 0 | 0 | 0 | 0 | 0 | 0.99296 | 0 |
| AABR07058 | 0 | 0 | 0.864895 | 0 | 0 | 0 | 0 | 0 |
| Nek10 | 0.281874 | 0.212618 | 0.386668 | 0.216446 | 0.101595 | 0.192226 | 0.210232 | 0.180439 |
| #N/A | 0.57545 | 0.325547 | 0.617782 | 0.795381 | 0.622225 | 0.252277 | 0.321894 | 0.765074 |
| Nucb1 | 51.84979 | 53.84777 | 55.31775 | 58.22083 | 55.59935 | 52.57067 | 71.02338 | 71.82337 |
| Tbc1d12 | 1.157839 | 1.518456 | 1.553768 | 1.357877 | 1.337316 | 0.922904 | 2.266844 | 2.215768 |
| AABR07065 | 0.298381 | 0.337604 | 0.160166 | 0.274947 | 0.806588 | 0.654052 | 0 | 0.26447 |
| Mtg2 | 12.2168 | 12.75196 | 11.02196 | 13.3185 | 12.46564 | 11.79293 | 11.32551 | 10.98087 |
| Wwp1 | 9.680229 | 9.010752 | 7.941668 | 7.844495 | 8.054464 | 9.044446 | 8.660008 | 9.751461 |
| Ift122 | 2.899588 | 2.710182 | 2.622274 | 3.238173 | 2.862649 | 3.095048 | 3.24393 | 3.39414 |
| Plekhg4 | 0.02691 | 0 | 0 | 0.12398 | 0.029097 | 0.070783 | 0.030105 | 0.059628 |
| 43351 | 26.49076 | 26.19833 | 26.8406 | 29.47888 | 27.4631 | 29.26896 | 38.23444 | 35.20252 |
| LOC684841 | 0 | 0.576638 | 0.136784 | 0 | 0.964374 | 0.223428 | 0.570167 | 0.112931 |
| AABR07065 | 0 | 0.335692 | 0 | 0.956861 | 1.443633 | 0.260139 | 0.165962 | 0.262972 |
| AABR07056 | 0.239114 | 0.135273 | 0.513408 | 0.110167 | 0.387825 | 0.209655 | 0 | 0.105969 |
| Wdr70 | 3.53194 | 3.74646 | 3.870759 | 4.090781 | 3.898599 | 3.655954 | 3.045854 | 4.652322 |
| LOC691015 | 0.174553 | 0 | 0 | 0 | 0 | 0.153048 | 0 | 0.154715 |
| Gtf2i | 29.63429 | 29.66693 | 29.97502 | 25.13327 | 23.73367 | 25.2214 | 29.13955 | 29.45748 |
| AABR07004 | 0 | 0 | 0 | 0 | 0 | 0 | 0 | 0 |
| Pphln1 | 5.830776 | 5.796154 | 5.872205 | 6.191548 | 6.37979 | 6.013191 | 5.731109 | 6.484743 |
| RGD130954 | 5.570133 | 6.830684 | 6.087317 | 7.314806 | 6.527818 | 7.398958 | 7.761535 | 7.982115 |
| AABR07067 | 0 | 0 | 0 | 0.150321 | 0 | 0.286072 | 0 | 0 |
| Klhl11 | 3.526254 | 3.511971 | 3.694984 | 3.735727 | 3.85855 | 4.036031 | 4.133999 | 4.435581 |
| AC131806. | 0 | 0 | 0.086092 | 0 | 0 | 0.070313 | 0 | 0 |
| Acot4 | 0.141968 | 0.137683 | 0.152412 | 0.280324 | 0.131578 | 0.177825 | 0.181517 | 0.089881 |
| Olr1738 | 0 | 0 | 0 | 0 | 0.163491 | 0.044191 | 0 | 0 |
| AABR07068 | 0 | 0 | 0 | 0 | 0 | 0 | 0 | 0 |

| | | | | | | | | |
|---|---|---|---|---|---|---|---|---|
| Zmpste24 | 8.823038 | 8.229108 | 7.219272 | 8.415734 | 8.17365 | 7.81968 | 9.604044 | 9.172997 |
| Arhgef1 | 24.0866 | 24.24645 | 27.65526 | 27.02812 | 25.14898 | 26.02073 | 31.71486 | 31.65669 |
| Prpf38a | 9.866043 | 9.660257 | 9.607331 | 10.43154 | 9.539662 | 9.870505 | 10.08251 | 11.71574 |
| Siglec5 | 0.793716 | 0.94186 | 0.976804 | 1.409241 | 1.549008 | 1.391862 | 0.996264 | 0.943733 |
| Hsd17b7 | 0.544925 | 0.571443 | 0.570743 | 0.587855 | 0.574848 | 0.640938 | 0.639376 | 0.553676 |
| Hoxb6 | 0.232737 | 0.263331 | 0.416431 | 1.787153 | 0.587196 | 0.9523 | 1.562258 | 1.375245 |
| RGD15597( | 0 | 0 | 0 | 0 | 0 | 0.093322 | 0 | 0 |
| AABR07018 | 0 | 0 | 0 | 0.049797 | 0 | 0 | 0 | 0 |
| AABR07014 | 0 | 0 | 0.108739 | 0.093333 | 0 | 0 | 0 | 0 |
| Mblac2 | 1.834475 | 1.611147 | 1.597576 | 1.158454 | 1.262285 | 1.439749 | 1.291676 | 1.569134 |
| Emilin1 | 8.886917 | 9.576313 | 10.66914 | 10.81796 | 10.99681 | 11.61028 | 11.66806 | 11.72463 |
| Fam78a | 7.80006 | 8.591306 | 7.396542 | 4.670888 | 5.123096 | 4.71662 | 5.786711 | 4.566264 |
| Mrrf | 13.4065 | 13.61049 | 13.40176 | 11.77301 | 12.29409 | 12.06315 | 11.62186 | 12.23349 |
| Jup | 70.23128 | 78.11897 | 85.6479 | 57.66932 | 52.63203 | 54.1634 | 67.98386 | 69.47309 |
| Pik3r2 | 14.67794 | 14.51243 | 15.37822 | 13.64923 | 13.70516 | 14.81779 | 17.68274 | 17.11265 |
| Stac | 0.047433 | 0.214672 | 0.101844 | 0.262245 | 0.35902 | 0.374303 | 0.318395 | 0.378379 |
| Cmpk2 | 2.659207 | 2.777324 | 3.381874 | 3.995963 | 3.774834 | 3.838164 | 6.316159 | 6.079818 |
| AABR0707( | 0 | 0 | 0 | 0 | 0 | 0 | 0 | 0 |
| Cbr3 | 2.452524 | 2.466591 | 3.169281 | 2.887655 | 3.8796 | 2.628236 | 3.96323 | 3.180179 |
| Atxn7l2 | 1.806472 | 2.043938 | 1.705705 | 1.80498 | 1.906244 | 1.870166 | 2.167097 | 2.276356 |
| Naxe | 36.56488 | 40.14514 | 39.49969 | 33.1149 | 34.29425 | 36.31146 | 38.67354 | 34.43168 |
| LOC10091( | 0.183258 | 0 | 0.049185 | 0 | 0.148616 | 0.04017 | 0.10251 | 0 |
| AY172581. | 3.080348 | 5.227904 | 8.267377 | 4.25763 | 17.48636 | 2.025639 | 8.615392 | 4.777965 |
| AABR07035 | 0.379463 | 0.57246 | 0.543171 | 0.116553 | 0.820616 | 0.443618 | 0.283018 | 0.560562 |
| Ccng1 | 354.7901 | 345.1163 | 328.144 | 299.2303 | 304.7623 | 300.6048 | 298.708 | 297.4625 |
| Pld6 | 1.017395 | 0.947993 | 1.349236 | 1.047782 | 0.841249 | 0.997 | 1.406032 | 1.326129 |
| LOC685513 | 0 | 0.541092 | 0.513408 | 0.220334 | 0.25855 | 0.41931 | 0.53502 | 0 |
| Cacna1f | 0.025967 | 0 | 0.046461 | 0.031903 | 0.056155 | 0.007589 | 0.009683 | 0.038359 |
| Mphosph8 | 3.494945 | 3.7896 | 3.81458 | 3.435151 | 4.408872 | 3.728318 | 3.877406 | 3.691477 |
| Pabpc6 | 0.085342 | 0.115873 | 0.054972 | 0.031456 | 0.018456 | 0.089794 | 0 | 0.075643 |
| Olr1087 | 0 | 0 | 0 | 0 | 0.060109 | 0 | 0 | 0 |
| Pear1 | 8.54595 | 9.589757 | 9.28789 | 9.592296 | 8.581473 | 9.507701 | 10.76741 | 10.91006 |
| AABR07034 | 0 | 0 | 0.010959 | 0.009406 | 0 | 0 | 0 | 0.009048 |
| Lrig1 | 12.6356 | 12.98364 | 12.75438 | 11.67716 | 11.47188 | 12.25786 | 13.41492 | 13.37091 |
| Avil | 1.077202 | 0.904274 | 1.249707 | 0.848512 | 1.145977 | 0.944492 | 1.399501 | 1.262771 |
| Zfp787 | 8.550197 | 10.03921 | 10.94574 | 8.97871 | 8.512565 | 8.119197 | 9.529484 | 10.75284 |
| Cidec | 0.263278 | 0.417041 | 0.31091 | 0.4852 | 0.284678 | 0.253926 | 0.294543 | 0.326698 |
| Grin3b | 3.857496 | 3.577828 | 4.745574 | 3.401976 | 2.953751 | 3.36774 | 5.926998 | 5.253366 |
| Btf3 | 180.7642 | 178.3546 | 167.5048 | 188.5721 | 193.9524 | 173.6112 | 187.4949 | 183.0004 |
| Mospd2 | 5.222665 | 6.093041 | 4.734686 | 6.138586 | 6.073869 | 5.841072 | 5.427391 | 6.172143 |
| Ptgs2 | 0.227522 | 0.284528 | 0.347105 | 1.32412 | 1.15239 | 1.333441 | 1.607629 | 1.740677 |
| Eya1 | 1.545522 | 1.234366 | 1.171212 | 1.200744 | 1.146868 | 1.062835 | 1.322223 | 1.235572 |
| AC095825. | 0.11118 | 0 | 0.238718 | 0 | 0 | 0.097483 | 0 | 0.295634 |
| Taf4 | 6.888808 | 7.111191 | 6.658965 | 6.196026 | 4.65919 | 5.919792 | 6.755045 | 7.054618 |
| Nr4a3 | 1.763804 | 1.928241 | 1.752819 | 0.560061 | 0.644316 | 0.553816 | 2.893235 | 2.545721 |
| AC128059. | 0 | 0.100765 | 0 | 0 | 0.096297 | 0 | 0 | 0 |
| Kdelr1 | 39.50157 | 38.60885 | 37.2577 | 38.57572 | 36.97556 | 36.45119 | 39.63917 | 42.64596 |
| Acadl | 687.7367 | 699.4123 | 651.9847 | 622.2445 | 630.4721 | 600.2778 | 591.8132 | 594.3787 |
| Ipo5 | 40.64003 | 41.09976 | 38.44963 | 43.79022 | 44.45537 | 43.31224 | 41.53482 | 43.65482 |
| 3082ex4-5 | 6.524827 | 4.353803 | 4.669879 | 6.012371 | 3.618052 | 4.547441 | 3.369086 | 5.338411 |
| Aldh1l1 | 0.308375 | 0.261684 | 0.351751 | 0.497272 | 0.208401 | 0.405575 | 0.431245 | 0.478324 |
| Sort1 | 12.44571 | 14.66626 | 14.34445 | 10.97056 | 11.24833 | 11.1938 | 13.11806 | 13.84804 |
| AABR07063 | 5.512202 | 139.2885 | 94.68322 | 118.5165 | 115.2318 | 82.16276 | 0 | 1.62858 |
| Paics | 9.891933 | 9.110678 | 9.503274 | 9.483559 | 9.398645 | 8.930414 | 10.11212 | 9.99661 |

| | | | | | | | | |
|---|---|---|---|---|---|---|---|---|
| Rn60_X_12 | 0 | 0 | 0 | 0 | 0 | 0.046099 | 0 | 0 |
| Serpini1 | 0.084108 | 0.19033 | 0.288947 | 0.248008 | 0.218269 | 0.235988 | 0.207013 | 0.298198 |
| Rplp0 | 656.6581 | 672.7239 | 695.3461 | 688.7881 | 664.7058 | 670.99 | 744.6095 | 754.3102 |
| Ndnf | 0.055502 | 0.094196 | 0.059585 | 0.102285 | 0.120027 | 0.024332 | 0.124186 | 0.122985 |
| Polr2i | 0.176713 | 9.930469 | 0.442663 | 0.379946 | 14.64924 | 9.089932 | 0.395397 | 2.6627 |
| Il6r | 2.437744 | 2.307086 | 2.49478 | 3.148997 | 2.906877 | 3.116232 | 4.141835 | 4.361773 |
| AABR07051 | 0.126692 | 0.334473 | 0.317361 | 0.155655 | 0.13699 | 0.703528 | 0.66144 | 0.33688 |
| Kctd21 | 0.865851 | 0.755162 | 0.852084 | 1.080418 | 1.189794 | 1.091319 | 1.210844 | 1.055239 |
| Ddah1 | 5.842599 | 6.085712 | 5.369675 | 8.536477 | 7.822435 | 7.932071 | 11.4185 | 11.61648 |
| AC095563. | 0.764466 | 0 | 0 | 0.352213 | 0 | 0 | 0 | 0 |
| LOC100359 | 0.329345 | 0.558958 | 0.707147 | 0.151739 | 0.890291 | 1.010696 | 0.368457 | 0.58383 |
| Ceacam15 | 0 | 0.084401 | 0 | 0 | 0.080659 | 0.261621 | 0 | 0 |
| Ccdc174 | 4.511253 | 4.503768 | 3.988451 | 4.754673 | 4.782307 | 4.912023 | 4.618161 | 4.756443 |
| Pak4 | 7.497156 | 8.676446 | 9.090509 | 7.522019 | 6.995514 | 7.307611 | 9.707343 | 8.43287 |
| Snrnp25 | 17.06842 | 19.62111 | 18.54393 | 19.43966 | 19.12025 | 18.43763 | 17.79691 | 18.63842 |
| Mir499 | 0.80563 | 0.911532 | 0 | 0 | 0 | 0.706377 | 0 | 0 |
| Nrl | 0 | 0.082635 | 0.078407 | 0 | 0 | 0 | 0 | 0 |
| Rab20 | 35.00509 | 35.79829 | 32.33115 | 37.47943 | 37.23754 | 36.78129 | 26.51769 | 31.08956 |
| AY172581. | 43.36553 | 44.43718 | 42.16362 | 45.99127 | 46.89052 | 34.43586 | 36.61542 | 28.28385 |
| LOC363337 | 0.096616 | 0 | 0.069149 | 0.089028 | 0.069646 | 0.254139 | 0.21618 | 0.199816 |
| AABR07007 | 0 | 0 | 0 | 0 | 0 | 0 | 0 | 0 |
| Cabyr | 0.378366 | 0.342483 | 0.32496 | 0.34865 | 0.354573 | 0.176934 | 0.31042 | 0.29065 |
| #N/A | 0.436383 | 0.493746 | 0.312323 | 0.268073 | 0.157285 | 0 | 0.32547 | 0.128929 |
| Slc14a1 | 0.282589 | 0.270546 | 0.280041 | 0.300455 | 0.188037 | 0.324013 | 0.243191 | 0.231206 |
| #N/A | 0 | 0 | 0.430791 | 0 | 0 | 0.175918 | 0 | 0 |
| AABR07020 | 0.076224 | 0 | 0.163663 | 0 | 0 | 0.066833 | 0 | 0 |
| Hspa1l | 2.417894 | 2.858965 | 2.619149 | 4.536276 | 3.603679 | 4.7939 | 4.849563 | 5.077795 |
| Cep55 | 0.21022 | 0.237855 | 0.338528 | 0.116226 | 0.090923 | 0.350211 | 0.16463 | 0.204962 |
| Cryba4 | 1.701892 | 1.703425 | 2.248726 | 2.412657 | 2.123344 | 1.6644 | 1.244924 | 2.320726 |
| Apold1 | 34.37929 | 36.52118 | 35.70271 | 25.11967 | 25.55945 | 25.3157 | 43.40656 | 43.6852 |
| Dap | 25.70571 | 24.09458 | 26.28932 | 35.57569 | 32.77771 | 33.33201 | 34.50272 | 37.57505 |
| Irx3 | 12.16413 | 11.38106 | 16.03671 | 11.36274 | 11.45459 | 10.40181 | 11.17857 | 11.69989 |
| Colgalt2 | 4.691987 | 4.929565 | 5.277011 | 4.606566 | 3.925828 | 3.722134 | 5.155451 | 5.346952 |
| RGD156638 | 1.246313 | 1.551159 | 1.382597 | 1.473817 | 1.909129 | 1.49345 | 1.580229 | 1.933171 |
| AC128792. | 3.458127 | 39.12708 | 2.12144 | 30.0444 | 16.02524 | 42.8824 | 22.66011 | 32.84046 |
| Snurf | 9.959163 | 9.612406 | 9.273889 | 9.045407 | 8.954617 | 7.761958 | 7.907133 | 8.131241 |
| Phf11b | 6.941052 | 7.3423 | 6.305253 | 8.628796 | 7.816127 | 9.074863 | 10.88986 | 9.901764 |
| Thap7 | 3.573389 | 4.637699 | 5.303068 | 4.963318 | 4.744586 | 4.377196 | 5.291139 | 4.681043 |
| AC121615. | 0.654574 | 2.962479 | 0.702727 | 1.809493 | 2.831126 | 0.573931 | 1.464617 | 2.320726 |
| Cplx4 | 0 | 0.052065 | 0 | 0 | 0.149268 | 0.040347 | 0.05148 | 0.040786 |
| Anapc10 | 3.732344 | 3.583128 | 2.974827 | 3.751864 | 3.240813 | 2.975021 | 3.068419 | 3.583842 |
| Borcs8 | 14.85865 | 12.73131 | 11.77019 | 15.41973 | 16.69038 | 17.96104 | 16.30042 | 16.11082 |
| AABR07012 | 0 | 0.361278 | 0 | 0 | 0 | 0 | 0 | 0.283015 |
| Pomgnt1 | 13.38111 | 12.93354 | 13.22536 | 12.29459 | 12.10953 | 12.6468 | 15.61826 | 15.83091 |
| Smc4 | 4.679718 | 4.121345 | 3.592342 | 4.858309 | 4.592819 | 4.687803 | 5.083508 | 5.47212 |
| Alpk1 | 0.840395 | 0.887476 | 1.01583 | 1.015312 | 1.002943 | 0.971562 | 1.030734 | 1.346308 |
| Stoml1 | 3.421979 | 3.490081 | 4.44985 | 4.707623 | 5.21146 | 4.923182 | 5.176372 | 5.703026 |
| Plekha7 | 1.544895 | 1.407461 | 1.443148 | 2.126096 | 1.800639 | 2.040644 | 3.030241 | 2.365184 |
| Stra13 | 71.12386 | 80.4733 | 86.14524 | 85.94339 | 74.36987 | 76.52414 | 81.61048 | 75.7411 |
| Zfp185 | 0.433672 | 0.20445 | 0.232787 | 0.233107 | 0.351693 | 0.411931 | 0.606467 | 0.576578 |
| Iqcb1 | 5.523831 | 4.979131 | 4.823218 | 5.276626 | 4.618995 | 4.439692 | 4.861456 | 4.373801 |
| AABR07045 | 0 | 0 | 0 | 0.041032 | 0 | 0 | 0 | 0 |
| AABR07033 | 0 | 0 | 0.101021 | 0 | 0 | 0 | 0 | 0 |
| Got2 | 446.5316 | 455.8549 | 465.2899 | 385.8614 | 382.0219 | 403.9618 | 396.8254 | 397.0626 |

| | | | | | | | | |
|---|---|---|---|---|---|---|---|---|
| AC135285. | 0.935106 | 0 | 0 | 0.430832 | 0.505558 | 0.819902 | 0.523077 | 0.828831 |
| Rtkn2 | 0.191466 | 0.151644 | 0.205551 | 0.229357 | 0.124218 | 0.235028 | 0.278465 | 0.458205 |
| Scrt2 | 0.114087 | 0.064542 | 0.12248 | 0.105127 | 0 | 0.050016 | 0 | 0 |
| Otud6b | 9.664964 | 9.982887 | 8.785218 | 9.712689 | 10.45059 | 11.10215 | 8.589906 | 10.14851 |
| AABR07072 | 0 | 0 | 0 | 0 | 0 | 0.429107 | 0 | 0.433781 |
| AC123346. | 0 | 0 | 0 | 0 | 0 | 0 | 0 | 0.283015 |
| Stkld1 | 0.020963 | 0.023719 | 0.022505 | 0 | 0.113336 | 0.01838 | 0.117263 | 0.018581 |
| Lpcat3 | 31.1774 | 31.81979 | 30.22132 | 25.50849 | 24.19028 | 26.03349 | 24.78227 | 26.39018 |
| AABR0707( | 0.197981 | 0.448012 | 0.106273 | 0.547295 | 0.535184 | 1.301923 | 0.55373 | 0.701921 |
| Ankrd45 | 0.171411 | 0.096971 | 0.18402 | 0.157948 | 0 | 0.075146 | 0.095883 | 0.075965 |
| Smarcc2 | 27.09291 | 28.5122 | 29.07021 | 27.49401 | 25.88322 | 26.66334 | 30.2775 | 31.16849 |
| Mcee | 52.2371 | 49.77547 | 55.11178 | 47.42221 | 49.79717 | 45.18031 | 45.46977 | 44.58762 |
| Serinc1 | 110.3437 | 108.4589 | 100.8764 | 107.5919 | 111.6522 | 118.3382 | 118.582 | 114.0622 |
| RGD131157 | 0.100481 | 0.096199 | 0.107872 | 0.092589 | 0.100291 | 0.094879 | 0.025942 | 0.08221 |
| Zfp3 | 1.516924 | 2.12728 | 1.995504 | 2.441203 | 2.425689 | 2.098092 | 2.342431 | 1.950508 |
| Cdk11b | 26.22218 | 27.89433 | 26.41103 | 27.51127 | 26.12042 | 26.75098 | 31.67608 | 32.78909 |
| AABR07049 | 0.201407 | 0.151922 | 0 | 0.247452 | 0.072593 | 0.117729 | 0.225326 | 0.119012 |
| Sec61g-ps | 0 | 0.14522 | 0 | 0 | 0 | 0.337607 | 0 | 0 |
| Asb13 | 5.052611 | 4.442941 | 5.74858 | 4.048506 | 5.166396 | 4.430134 | 5.744799 | 5.670992 |
| Zc3hc1 | 7.895081 | 6.561631 | 7.736161 | 8.095098 | 7.978062 | 7.048276 | 6.35952 | 7.379501 |
| Fam177a1 | 9.019967 | 11.81212 | 10.58013 | 8.850256 | 9.211318 | 10.03235 | 12.24018 | 12.14032 |
| Enthd2 | 4.063036 | 4.511606 | 4.848842 | 5.154431 | 4.168529 | 5.219438 | 5.074096 | 5.728533 |
| Pkd2l1 | 0.084325 | 0.076328 | 0.090528 | 0.139864 | 0.072944 | 0.044362 | 0.018868 | 0.074742 |
| Atp9a | 28.17045 | 28.47597 | 31.24004 | 30.55634 | 28.98867 | 28.31772 | 32.89345 | 32.78121 |
| Kpna5 | 3.63614 | 3.61544 | 3.548753 | 3.528241 | 3.633849 | 3.550463 | 2.927692 | 3.515881 |
| Fads3 | 24.58511 | 26.09118 | 28.73286 | 28.20901 | 25.44479 | 29.10252 | 31.12056 | 32.28416 |
| St3gal4 | 13.93712 | 14.99224 | 14.14328 | 16.00626 | 13.64 | 13.82563 | 15.93366 | 15.59925 |
| AABR07044 | 0 | 0 | 0.749575 | 0 | 0 | 1.224386 | 0.781129 | 0.61886 |
| Rn60_X_07 | 0 | 0 | 0 | 0 | 0 | 0.111714 | 0 | 0 |
| Trmt12 | 3.222518 | 2.552289 | 3.182813 | 3.028812 | 3.623841 | 3.305843 | 2.848116 | 2.856278 |
| Tex37 | 0 | 0 | 0 | 0.117404 | 0 | 0 | 0 | 0 |
| AABR0707( | 0.387896 | 0 | 0.416431 | 0 | 1.258278 | 0.340107 | 0 | 0 |
| Exd1 | 0.091469 | 0.051746 | 0.049099 | 0.098332 | 0.148356 | 0.173767 | 0.153497 | 0.108098 |
| AABR07058 | 0 | 0.442161 | 0 | 0 | 0 | 0 | 0 | 0 |
| Prr3 | 8.375474 | 7.042136 | 9.362819 | 8.461115 | 8.350055 | 8.084722 | 9.026251 | 10.21596 |
| Cux2 | 3.774213 | 3.804912 | 4.504519 | 4.103222 | 3.736285 | 3.687947 | 4.464032 | 4.357058 |
| Trim28 | 24.93951 | 28.33762 | 27.41795 | 23.51711 | 24.65911 | 25.20735 | 28.35506 | 27.77993 |
| RGD156488 | 3.500786 | 4.703657 | 3.914914 | 2.688197 | 2.917873 | 2.430015 | 0.979131 | 0.840374 |
| Nxf1 | 21.00685 | 21.13612 | 20.85392 | 28.01726 | 27.34281 | 27.21009 | 28.0561 | 28.08377 |
| Paxbp1 | 9.306965 | 9.154994 | 9.135196 | 9.67863 | 9.734882 | 9.742464 | 10.90096 | 11.75094 |
| Slc24a5 | 2.594275 | 2.71787 | 2.647583 | 3.630052 | 3.636308 | 3.173295 | 3.224844 | 3.804003 |
| Ccdc88c | 4.084876 | 4.354573 | 4.533822 | 3.084506 | 3.121122 | 2.722842 | 2.649169 | 2.711641 |
| Atp5i | 769.3623 | 887.7186 | 920.4994 | 806.7791 | 785.0741 | 816.134 | 682.9472 | 655.9062 |
| Snip1 | 0.169881 | 0.14416 | 0.319162 | 0.215241 | 0.091845 | 0.167571 | 0.166299 | 0.809337 |
| Bahd1 | 4.3014 | 5.099237 | 5.149656 | 4.69839 | 4.703301 | 4.597805 | 5.663815 | 5.911586 |
| AABR07015 | 0 | 0 | 0 | 0 | 0 | 0 | 0 | 0.073674 |
| AC106663. | 0 | 0 | 0 | 0 | 0 | 0.327961 | 0 | 0 |
| Dimt1 | 3.968294 | 3.741615 | 3.424881 | 4.122668 | 3.323312 | 3.274733 | 4.570126 | 4.517312 |
| LOC100361 | 0.401169 | 0.635465 | 0.574241 | 0.369661 | 0.289185 | 0.468994 | 0.418889 | 0.592627 |
| AABR07054 | 0 | 0 | 0 | 0.365554 | 0.428958 | 0 | 0 | 0 |
| AABR07018 | 0.727304 | 0 | 0 | 0 | 0 | 1.275402 | 0 | 0 |
| Chit1 | 0.833272 | 0.471404 | 0.41933 | 0.671849 | 0.39419 | 0.479465 | 0.786567 | 0.553928 |
| Cxcl11 | 16.62517 | 15.76435 | 13.62098 | 9.520381 | 10.04358 | 11.24256 | 10.50458 | 12.91612 |
| Lnx1 | 14.32477 | 13.77897 | 14.86889 | 14.03658 | 13.63672 | 14.04401 | 16.14138 | 15.02023 |

| | | | | | | | | |
|---|---|---|---|---|---|---|---|---|
| Reps1 | 2.821397 | 2.784753 | 2.92154 | 2.433861 | 2.747825 | 2.789608 | 2.663957 | 2.766781 |
| Nudt21 | 18.19953 | 14.54447 | 16.16091 | 17.5806 | 16.89897 | 17.3514 | 18.69562 | 17.54037 |
| Rslcan18 | 2.214807 | 2.754063 | 2.189401 | 2.54602 | 2.79793 | 2.999439 | 2.207965 | 2.021403 |
| Sdf4 | 58.46265 | 55.14797 | 53.95387 | 65.68378 | 65.74483 | 65.36096 | 63.26701 | 62.77206 |
| Gas2 | 0.274311 | 0.31037 | 0.530082 | 0.37915 | 0.444912 | 0.312671 | 0.398953 | 0.364703 |
| AABR07071 | 0 | 0 | 0 | 0.056835 | 0 | 0 | 0 | 0 |
| Rpl32 | 378.7576 | 395.3622 | 394.2545 | 368.9001 | 380.1349 | 366.4672 | 366.452 | 354.9295 |
| Tgm2 | 74.69537 | 75.20268 | 83.8462 | 102.7134 | 97.69241 | 100.97 | 131.356 | 128.2026 |
| Rn50_5_08 | 0.171692 | 0 | 0.368644 | 0.474621 | 0.185648 | 0.301079 | 0.768323 | 0.304357 |
| Fbxo43 | 0.020739 | 0 | 0.022265 | 0 | 0 | 0.018184 | 0.046404 | 0.018382 |
| Rrp1 | 1.517438 | 10.56061 | 11.55715 | 9.919726 | 9.256496 | 8.936882 | 1.729673 | 1.801766 |
| Carm1 | 13.57818 | 13.7017 | 15.37409 | 13.3023 | 12.52332 | 12.19468 | 15.70748 | 14.5941 |
| Nemf | 5.535698 | 5.152137 | 5.20006 | 5.327179 | 5.961535 | 5.186419 | 6.417843 | 6.113421 |
| Tbccd1 | 3.972426 | 3.35774 | 4.816549 | 3.961882 | 3.789994 | 4.466469 | 4.940875 | 4.100881 |
| Ggact | 4.87366 | 5.514317 | 4.953878 | 3.344277 | 4.260704 | 4.955128 | 4.698374 | 5.330776 |
| AC109931. | 5.39061 | 1.742635 | 0 | 5.676839 | 6.661472 | 6.076917 | 2.584618 | 2.730266 |
| Ube2h | 38.54742 | 38.50406 | 35.20554 | 36.52719 | 36.1501 | 36.07696 | 41.44075 | 42.31966 |
| Noxred1 | 0 | 0 | 0.042947 | 0 | 0 | 0 | 0 | 0 |
| Zfp26 | 2.460503 | 2.077871 | 1.761005 | 2.135821 | 2.3906 | 2.360602 | 2.792596 | 2.402113 |
| Tnfaip8l2 | 3.40284 | 3.679035 | 3.693756 | 5.783408 | 5.478279 | 4.806932 | 5.879616 | 6.367335 |
| AC096430. | 0.054378 | 0.092289 | 0 | 0 | 0.029399 | 0.143036 | 0.060836 | 0.024099 |
| Cysrt1 | 0 | 0 | 0.073874 | 0.126815 | 0 | 0 | 0.076984 | 0 |
| Tcea2 | 2.061305 | 1.389437 | 1.318349 | 2.101477 | 2.039169 | 2.345715 | 2.404228 | 2.021403 |
| Zfp637 | 14.2562 | 13.17829 | 13.20427 | 14.93958 | 16.01954 | 15.80864 | 12.97828 | 13.66833 |
| AABR07067 | 0 | 0.011556 | 0 | 0 | 0.011044 | 0 | 0 | 0 |
| Nfam1 | 1.456739 | 1.578832 | 1.366356 | 1.596663 | 1.724375 | 1.962962 | 2.62473 | 2.065888 |
| AABR07036 | 0 | 0.236762 | 0.056162 | 0.144615 | 0.226264 | 0.137606 | 0.234105 | 0.231841 |
| Ppm1m | 2.717507 | 2.928315 | 3.10265 | 3.815734 | 4.244356 | 3.309322 | 3.981248 | 3.708574 |
| Mllt11 | 7.711026 | 5.903254 | 7.372198 | 8.165915 | 7.881522 | 7.803781 | 10.38644 | 11.05108 |
| AABR07052 | 0.080812 | 0 | 0.173513 | 0 | 0.08738 | 0.070856 | 0 | 0.071627 |
| Slc2a2 | 0.024806 | 0 | 0.053262 | 0 | 0 | 0 | 0 | 0 |
| AC094643. | 0 | 0 | 0.85179 | 0.731108 | 0 | 1.391348 | 0 | 0 |
| Slc16a6 | 5.973964 | 4.553309 | 5.742571 | 6.126651 | 7.648769 | 4.909234 | 7.382507 | 7.665596 |
| St3gal5 | 42.20722 | 43.02676 | 39.20389 | 38.75884 | 39.01607 | 38.44046 | 40.27551 | 42.17835 |
| Ust5r | 0 | 0.032771 | 0.031094 | 0.026689 | 0 | 0.05079 | 0.097209 | 0 |
| Hspa2 | 1.58945 | 2.381648 | 2.352031 | 2.889646 | 2.624423 | 3.295749 | 3.460292 | 3.350679 |
| Rps24 | 496.8271 | 464.0074 | 449.4665 | 475.752 | 468.0482 | 428.181 | 503.4408 | 491.2267 |
| Cenpj | 1.98841 | 1.781715 | 1.447002 | 3.344764 | 2.842669 | 2.644412 | 2.508212 | 2.838808 |
| Jmy | 4.174878 | 4.438628 | 4.095619 | 3.399276 | 3.34676 | 2.855849 | 4.610271 | 4.497902 |
| Nat6 | 21.04941 | 22.35912 | 21.80775 | 24.24525 | 23.55624 | 22.23126 | 30.58918 | 31.21482 |
| LOC100909 | 1.173402 | 1.379713 | 1.062118 | 1.611265 | 1.020001 | 1.270919 | 1.261269 | 1.345939 |
| Enam | 0 | 0 | 0 | 0 | 0 | 0.011082 | 0 | 0 |
| AABR07005 | 0.256948 | 0.232579 | 0.11034 | 0.189414 | 0.055567 | 0.540701 | 0.517431 | 0.227745 |
| AABR07035 | 0.580137 | 0.83015 | 0.769359 | 0.487405 | 0.608844 | 0.583469 | 0.68721 | 0.423463 |
| Cul3 | 17.00574 | 15.35689 | 15.57155 | 14.90187 | 14.66273 | 14.7279 | 15.76066 | 16.36625 |
| AABR07030 | 0 | 0 | 0 | 0 | 0 | 0 | 0 | 0 |
| Adm | 4.455068 | 5.126135 | 4.174817 | 6.714387 | 6.531797 | 6.322759 | 6.251284 | 6.558937 |
| Pspc1 | 13.57127 | 14.42463 | 14.76585 | 14.14752 | 14.18033 | 11.85924 | 12.32016 | 14.37797 |
| Efna4 | 0.329864 | 0.559839 | 0.575461 | 0.49393 | 0.356677 | 0.614603 | 0.507426 | 0.69439 |
| Ube2i | 51.15832 | 49.56097 | 47.96812 | 60.49345 | 55.91028 | 52.74872 | 60.18131 | 56.53425 |
| Srms | 0.757212 | 0.401601 | 0.736704 | 0.479697 | 0.665244 | 0.663924 | 1.111864 | 0.776022 |
| Gng5 | 76.5377 | 78.51738 | 70.54512 | 67.16642 | 72.15001 | 65.03179 | 70.94927 | 70.07159 |
| Pitpna | 58.68227 | 56.40057 | 56.76797 | 53.48351 | 56.8994 | 59.23605 | 70.47349 | 67.63583 |
| Mir425 | 0.630915 | 0 | 0.677327 | 2.325452 | 1.364398 | 0 | 1.411679 | 1.118422 |

| | | | | | | | | |
|---|---|---|---|---|---|---|---|---|
| Raph1 | 8.906894 | 7.6597 | 8.614789 | 9.083989 | 10.61486 | 9.139376 | 10.55079 | 10.90093 |
| Cntln | 1.08617 | 0.70901 | 0.807281 | 0.885379 | 0.767916 | 1.062242 | 0.958106 | 0.925698 |
| AABR07065 | 0 | 0.072788 | 0 | 0 | 0 | 0 | 0 | 0.05702 |
| Snrpd3 | 64.11423 | 65.97696 | 61.21704 | 60.861 | 59.33358 | 58.85345 | 56.50094 | 56.51015 |
| Ptp4a2 | 92.82836 | 92.04179 | 84.09995 | 75.65294 | 74.07222 | 68.80985 | 83.0081 | 84.57201 |
| Pdp2 | 8.834893 | 8.960238 | 9.0863 | 7.707731 | 8.081285 | 8.354006 | 6.893942 | 7.457909 |
| Mkl1 | 4.565741 | 4.41994 | 4.760052 | 4.981753 | 4.633885 | 4.263385 | 5.863999 | 5.507798 |
| AABR07026 | 0 | 0 | 0 | 0 | 0.481894 | 0 | 0 | 0 |
| AABR07071 | 0 | 0 | 0 | 0 | 0.042734 | 0.017326 | 0 | 0 |
| AABR07058 | 0.020416 | 0 | 0 | 0 | 0 | 0 | 0 | 0.009048 |
| LOC685085 | 5.943027 | 7.104874 | 8.908231 | 5.269614 | 7.032346 | 7.472165 | 5.770652 | 4.671268 |
| Mab21l1 | 0.109552 | 0.099162 | 0.188178 | 0.343223 | 0.213223 | 0.1729 | 0.171587 | 0.155362 |
| AABR07025 | 0 | 0 | 0 | 0 | 0 | 0 | 0 | 0 |
| Zfp819 | 0.194359 | 0.157077 | 0.208657 | 0.05117 | 0.210158 | 0.292139 | 0.279566 | 0.36915 |
| Dkk2 | 0.87499 | 0.654413 | 0.748302 | 0.491961 | 0.545218 | 0.650163 | 0.530929 | 0.814982 |
| Fam122a | 9.49558 | 10.51714 | 11.18341 | 9.783535 | 10.31076 | 10.81994 | 11.60936 | 11.82558 |
| AABR07065 | 0 | 0 | 0 | 0 | 0 | 0.131938 | 0 | 0 |
| LOC100909 | 0.044303 | 0.15038 | 0.095124 | 0.326586 | 0 | 0.038845 | 0 | 0 |
| AABR07021 | 0 | 0.09316 | 0.26518 | 0.07587 | 0.178058 | 0.144385 | 0.092114 | 0.145958 |
| Rnf222 | 0.019503 | 0 | 0 | 0 | 0 | 0 | 0 | 0 |
| AABR07015 | 0.220953 | 0 | 0.711622 | 0 | 0 | 0 | 0 | 0 |
| Ccr4 | 0 | 0.040946 | 0 | 0 | 0.078262 | 0 | 0.040487 | 0 |
| AC120688. | 0 | 0 | 0 | 0 | 0 | 0 | 0 | 0 |
| AC120718. | 0 | 0 | 0 | 0.398786 | 0 | 0 | 0 | 0 |
| RGD131180 | 4.821951 | 4.981392 | 4.826559 | 5.516484 | 5.365033 | 6.04568 | 5.57701 | 5.966991 |
| Tardbp | 53.49034 | 52.16144 | 52.20874 | 53.72972 | 55.31985 | 51.30169 | 54.45139 | 54.42316 |
| Begain | 0.746469 | 0.639844 | 0.995656 | 1.271465 | 1.296325 | 0.991674 | 1.442473 | 1.443562 |
| AABR07010 | 0 | 0.420956 | 0.099855 | 0.342829 | 0.301719 | 0.48932 | 0.832464 | 0.494649 |
| Kcnj11 | 39.24855 | 43.00183 | 45.26729 | 35.13223 | 35.16045 | 37.00096 | 40.72349 | 39.74453 |
| Mmp21 | 0 | 0 | 0.030323 | 0 | 0 | 0.024765 | 0 | 0.05007 |
| Zbed4 | 1.744539 | 1.828068 | 1.926081 | 1.625792 | 1.436195 | 1.607833 | 1.674481 | 1.475987 |
| AABR07021 | 0 | 0 | 0.097095 | 0 | 0 | 0.0793 | 0 | 0 |
| Dph2 | 3.086066 | 3.629208 | 3.313089 | 3.470643 | 3.494568 | 3.685942 | 3.071957 | 3.079942 |
| Urb1 | 1.764025 | 1.87519 | 1.840339 | 1.789338 | 1.899723 | 1.702615 | 2.124709 | 2.250745 |
| Zc3h12d | 0.312124 | 0.337101 | 0.380779 | 0.549074 | 0.44488 | 0.485144 | 0.41268 | 0.389827 |
| Slc17a9 | 0.230079 | 0.156194 | 0.321105 | 0.784431 | 0.348293 | 0.403466 | 0.463323 | 0.448646 |
| Sall3 | 0 | 0 | 0.034497 | 0 | 0 | 0 | 0 | 0 |
| Lrch3 | 7.491103 | 8.340434 | 7.605382 | 8.380343 | 7.504812 | 6.694114 | 9.532057 | 9.16411 |
| Gpi | 182.5527 | 185.633 | 185.7097 | 181.081 | 176.0808 | 183.6579 | 189.193 | 189.3915 |
| Pcyox1 | 15.60185 | 14.7593 | 16.27103 | 15.24125 | 14.94205 | 15.86025 | 16.95596 | 15.86661 |
| Gpkow | 5.73198 | 5.634432 | 5.220855 | 5.723936 | 5.889414 | 5.332812 | 5.556693 | 6.206993 |
| LOC100912 | 0.732391 | 0.569707 | 0.687984 | 0.351494 | 0.263974 | 0.454864 | 0.921787 | 0.581534 |
| Tmem169 | 0 | 0 | 0 | 0.016628 | 0 | 0 | 0 | 0 |
| Mrpl45 | 30.49335 | 29.07394 | 27.81588 | 26.08514 | 28.22138 | 24.50855 | 26.38303 | 27.57507 |
| Clcnka | 0 | 0 | 0 | 0 | 0 | 0 | 0 | 0 |
| RGD156393 | 0.055414 | 0.062698 | 0.05949 | 0 | 0.239672 | 0.194347 | 0.247977 | 0.098232 |
| Prol1 | 0 | 0 | 0 | 0 | 0 | 0 | 0 | 0 |
| Fhl5 | 0.234195 | 0.26498 | 0.150854 | 0.129481 | 0.303878 | 0.328547 | 0.681217 | 0.788798 |
| AABR07032 | 0 | 0 | 0 | 0 | 0 | 0 | 0 | 0 |
| Zbtb33 | 0.086914 | 0.04917 | 0.069981 | 0.080088 | 0.070484 | 0.133362 | 0.048618 | 0.019259 |
| Smg5 | 29.65135 | 29.33239 | 27.83164 | 28.40042 | 28.32596 | 28.59817 | 32.92915 | 33.105 |
| Sppl2a | 8.166505 | 7.327171 | 6.690807 | 9.769445 | 9.83729 | 9.823564 | 8.527235 | 7.136788 |
| Chrm1 | 0 | 0 | 0 | 0 | 0 | 0 | 0 | 0 |
| AABR07006 | 0.417065 | 0.340199 | 0.416508 | 0.285997 | 1.206073 | 0.382692 | 0.412339 | 0.421247 |

| | | | | | | | | |
|---|---|---|---|---|---|---|---|---|
| Ywhaz | 75.87906 | 74.1238 | 75.15869 | 82.29217 | 80.39249 | 76.88007 | 97.60618 | 93.9638 |
| AC141334. | 0 | 0 | 0 | 0 | 0.198675 | 0 | 0.41112 | 0 |
| Slc9a1 | 6.569269 | 6.527676 | 7.396063 | 6.131374 | 5.597121 | 5.759941 | 6.549184 | 7.215772 |
| #N/A | 0 | 0 | 0.223088 | 0 | 0 | 0 | 0 | 0 |
| Rab13 | 4.56633 | 4.229954 | 4.09954 | 4.084661 | 3.898031 | 4.050589 | 4.481234 | 4.449735 |
| Ctnna2 | 0.073188 | 0 | 0 | 0 | 0 | 0.096257 | 0 | 0 |
| LOC10091 | 2.897719 | 2.419943 | 3.110886 | 2.161537 | 2.909457 | 2.117269 | 1.85248 | 2.140327 |
| Fbxo46 | 2.168472 | 2.798235 | 2.732026 | 2.113758 | 1.802154 | 2.137019 | 2.586387 | 2.589174 |
| LOC501421 | 0 | 0 | 0 | 0 | 0 | 0.096866 | 0 | 0 |
| Azgp1 | 0.084054 | 0 | 0 | 0.038726 | 0.090887 | 0 | 0 | 0 |
| Steap1 | 0 | 0 | 0.04392 | 0.075396 | 0 | 0.143483 | 0 | 0.072523 |
| Scpep1 | 27.53367 | 26.42131 | 27.04941 | 45.0219 | 40.41657 | 45.11736 | 47.9197 | 47.17428 |
| Chek2 | 0.73397 | 0.287464 | 0.575819 | 0.494237 | 0.579961 | 0.618794 | 0.947461 | 0.925788 |
| RGD156414 | 0.244701 | 0.395525 | 0.225173 | 0.096635 | 0.075597 | 0.122602 | 0.078217 | 0.185906 |
| Sptlc3 | 0 | 0 | 0 | 0.007793 | 0 | 0 | 0.009461 | 0 |
| LOC100909 | 0.269927 | 0.101803 | 0 | 0 | 0.09729 | 0.078891 | 0.100661 | 0.07975 |
| AABR07057 | 0 | 0 | 0 | 0 | 0 | 0 | 0 | 0 |
| AABR07030 | 1.707584 | 0.966026 | 2.444268 | 2.360208 | 1.230924 | 1.746747 | 1.91037 | 1.513517 |
| AABR07051 | 0.147926 | 0 | 0.158808 | 0.136308 | 0.479852 | 0.129702 | 0 | 0.262229 |
| Mtmr1 | 7.91926 | 7.757663 | 8.083972 | 6.566178 | 6.750025 | 6.142933 | 7.251904 | 7.948055 |
| Hspa8 | 232.8365 | 227.4902 | 219.8252 | 230.6838 | 230.1714 | 226.682 | 296.1277 | 291.5876 |
| Use1 | 61.95619 | 59.59405 | 62.31936 | 63.86994 | 66.82773 | 60.24052 | 59.20911 | 58.6478 |
| AABR07029 | 0.57545 | 0.325547 | 0.720746 | 0.441879 | 0 | 0.252277 | 0.214596 | 0.680066 |
| Adig | 0 | 0 | 0.23135 | 4.170024 | 0.466029 | 0 | 0.241089 | 0 |
| Repin1 | 4.739314 | 6.207602 | 6.767233 | 5.421217 | 5.023576 | 5.731634 | 6.007344 | 6.228609 |
| Sbspon | 1.208776 | 1.172292 | 0.787888 | 0.5967 | 1.026954 | 0.37852 | 0.72446 | 1.071399 |
| AABR07060 | 0 | 0 | 0 | 0 | 0 | 0 | 0.129326 | 0 |
| Pcyt1a | 29.26403 | 28.20555 | 29.74333 | 27.4594 | 27.99522 | 28.78731 | 30.62752 | 31.49711 |
| Fgf6 | 0.083518 | 0.188994 | 0.089662 | 0.307835 | 0 | 0.366144 | 0.280309 | 0.148053 |
| LOC100909 | 11.60662 | 13.95383 | 21.75977 | 11.49415 | 10.09675 | 10.52152 | 13.31213 | 13.99675 |
| Orm1 | 0.256696 | 0.52279 | 0.551158 | 0.614991 | 0.721659 | 0.405128 | 0.28718 | 0.728071 |
| Kif1b | 30.54754 | 28.35552 | 29.73273 | 29.35364 | 28.21795 | 28.36902 | 33.20983 | 33.37176 |
| NEWGENE_1 | 1.819627 | 1.368839 | 1.847892 | 1.468258 | 1.393229 | 1.069383 | 1.33147 | 1.429748 |
| Glod4 | 19.69562 | 18.99077 | 19.56253 | 20.59949 | 20.94683 | 21.80702 | 19.26016 | 20.89768 |
| C2cd4c | 0.03279 | 0.037101 | 0.105607 | 0.030215 | 0.035456 | 0.02875 | 0.073368 | 0.029064 |
| Map2k4 | 13.46686 | 12.21093 | 12.1403 | 13.36041 | 12.43057 | 13.08316 | 12.02141 | 11.22932 |
| AC119011. | 0 | 0 | 0.689793 | 0 | 0.347377 | 0.281684 | 0.359415 | 0 |
| Trim58 | 0 | 0 | 0 | 0 | 0 | 0 | 0.040182 | 0.095503 |
| Asb6 | 4.745132 | 6.218055 | 5.276138 | 5.666341 | 5.444976 | 5.073307 | 6.12119 | 6.222936 |
| Tpcr12 | 0.054891 | 0.124213 | 0.058929 | 0.202319 | 0.178058 | 0.096257 | 0 | 0.145958 |
| Cdk5r2 | 0 | 0 | 0 | 0 | 0 | 0 | 0 | 0 |
| Swt1 | 3.192662 | 3.134242 | 3.326717 | 3.201493 | 3.147575 | 3.224717 | 3.921986 | 3.80083 |
| Sohlh2 | 0 | 0.025342 | 0 | 0.020639 | 0.048437 | 0.019638 | 0 | 0 |
| AABR07015 | 0 | 0.053234 | 0 | 0 | 0 | 0.041253 | 0 | 0.041702 |
| Slc25a40 | 3.130774 | 3.753176 | 3.481125 | 3.400043 | 3.788268 | 3.594728 | 3.21069 | 3.039242 |
| Tekt1 | 0.109705 | 0 | 0.039258 | 0.033696 | 0 | 0.09619 | 0.204555 | 0.064825 |
| Padi3 | 0 | 0 | 0 | 0.015571 | 0.018271 | 0 | 0.018904 | 0 |
| Kcnq3 | 0 | 0.047724 | 0 | 0 | 0.022804 | 0 | 0 | 0.056079 |
| Slc16a13 | 1.23829 | 1.556741 | 1.624802 | 0.887472 | 0.743859 | 0.940969 | 1.292988 | 1.121948 |
| Nploc4 | 26.1416 | 26.24578 | 24.79638 | 22.48764 | 21.98707 | 21.81845 | 27.28398 | 26.60219 |
| Ccr7 | 0.3305 | 0.83099 | 0.354813 | 0.54141 | 0.635316 | 0.740556 | 0.328666 | 0.846268 |
| Mcm10 | 1.318753 | 1.25713 | 1.460357 | 1.406546 | 1.20139 | 1.201807 | 0.97583 | 0.966394 |
| AABR07060 | 0 | 0 | 0 | 0 | 0 | 0 | 0 | 0.450626 |
| Wbscr17 | 1.150297 | 1.053601 | 1.176112 | 1.110428 | 1.184571 | 1.392803 | 2.144836 | 0.873914 |

| | | | | | | | | |
|---|---|---|---|---|---|---|---|---|
| AABR07068 | 0.149191 | 0.675209 | 0.480497 | 0.274947 | 0.322635 | 0.130811 | 0 | 0.132235 |
| Ctnnd1 | 19.78877 | 20.02819 | 19.75049 | 18.42887 | 17.22501 | 17.42476 | 22.30393 | 22.5765 |
| Stxbp4 | 2.595466 | 2.256868 | 2.321998 | 1.705136 | 2.208772 | 1.980707 | 2.688603 | 2.620003 |
| Nipal2 | 9.425866 | 7.406197 | 9.352658 | 8.400432 | 8.725014 | 9.162027 | 8.414888 | 8.228028 |
| Mib2 | 10.14512 | 9.498024 | 10.62507 | 11.07574 | 10.29907 | 11.19158 | 13.33923 | 13.9158 |
| Txlnb | 70.37475 | 67.47868 | 64.44083 | 57.23239 | 58.36239 | 57.50597 | 52.82413 | 54.60666 |
| Mlip | 54.99541 | 53.8057 | 52.76459 | 56.37265 | 53.80954 | 53.73859 | 50.13495 | 48.62119 |
| Trim71 | 0.020392 | 0 | 0 | 0.01879 | 0.022049 | 0 | 0 | 0.018074 |
| Ift27 | 3.179909 | 3.481856 | 4.12964 | 4.820587 | 5.323958 | 4.182221 | 4.360857 | 5.409723 |
| Nat8f4 | 0 | 0 | 0.082552 | 0 | 0 | 0 | 0 | 0 |
| Plekha3 | 12.54311 | 11.78 | 12.20548 | 11.13096 | 11.09066 | 10.23934 | 13.20315 | 12.97964 |
| AABR07070 | 0 | 0 | 0 | 0 | 0 | 0 | 0.121293 | 0 |
| Nefh | 0.027253 | 0.030835 | 0.029257 | 0.050224 | 0.058936 | 0.023895 | 0.015245 | 0.024155 |
| Tubb2b | 1.801285 | 1.382975 | 1.69207 | 1.86729 | 2.295507 | 1.297338 | 2.195126 | 2.166771 |
| Inhbc | 0.013482 | 0.03051 | 0.014474 | 0.062118 | 0.029157 | 0.08275 | 0.045251 | 0.01195 |
| Skiv2l2 | 6.72964 | 6.427631 | 5.660911 | 5.972919 | 6.221389 | 6.168761 | 6.502165 | 7.785245 |
| Prr5 | 1.38632 | 1.390786 | 1.666899 | 1.141179 | 1.23918 | 1.434321 | 1.840464 | 2.080707 |
| LOC361646 | 1.41174 | 1.716373 | 1.628557 | 1.446301 | 1.70664 | 1.860567 | 1.677491 | 2.168394 |
| LOC100125 | 3.326087 | 3.642885 | 3.170841 | 3.67783 | 3.970481 | 3.219613 | 3.810385 | 3.01883 |
| AABR07065 | 0.887558 | 2.00846 | 4.764251 | 0.81785 | 1.919407 | 3.112846 | 4.964802 | 0 |
| Foxl2 | 0.046547 | 0.052666 | 0 | 0.042892 | 0.100662 | 0.163252 | 0.260376 | 0.371316 |
| Il17rb | 0.111559 | 0.025245 | 0.023953 | 0.082238 | 0.048251 | 0.019563 | 0.024962 | 0.019776 |
| Herpud2 | 14.75813 | 15.3738 | 15.92575 | 15.2403 | 14.52706 | 14.65783 | 18.01948 | 18.92215 |
| AABR07065 | 1.214423 | 1.429025 | 1.564513 | 1.790469 | 1.785868 | 1.874061 | 2.010791 | 1.162516 |
| Alox12e | 0 | 0 | 0.091561 | 0 | 0.023055 | 0 | 0.047707 | 0.018898 |
| Myo1d | 5.636506 | 5.355704 | 5.049727 | 6.839747 | 6.706286 | 6.186318 | 8.259805 | 8.373436 |
| AC126292. | 0.150477 | 0.170257 | 0.161546 | 0.138658 | 0.162708 | 0 | 0.168347 | 0.133375 |
| Socs3 | 7.766904 | 8.513265 | 9.054837 | 10.23212 | 9.907299 | 9.683009 | 12.08351 | 11.67086 |
| Chac2 | 3.126324 | 2.702095 | 2.750308 | 3.480948 | 3.004843 | 3.159952 | 2.866082 | 3.425284 |
| Hey1 | 7.082505 | 5.741972 | 6.166635 | 5.755433 | 5.366777 | 4.74303 | 4.866458 | 6.574154 |
| Ankar | 0.266678 | 0.274304 | 0.156162 | 0.134036 | 0.196606 | 0.085027 | 0.21698 | 0.139673 |
| Scn1a | 1.161406 | 1.386677 | 1.040184 | 1.336259 | 1.318255 | 0.877669 | 1.148578 | 1.364966 |
| Mpdz | 7.061043 | 6.886187 | 6.810471 | 6.58989 | 6.919693 | 6.392615 | 8.460498 | 8.708894 |
| AABR07009 | 0.48487 | 1.097214 | 0 | 0 | 0 | 0 | 1.084901 | 0 |
| Gpr150 | 0 | 0.027726 | 0.026307 | 0.02258 | 0.052993 | 0.085942 | 0.027414 | 0.021719 |
| Cdk4 | 39.33186 | 41.96845 | 43.33483 | 45.34313 | 43.58443 | 47.2738 | 41.24052 | 42.80111 |
| Ccnj | 0.275215 | 0.070771 | 0.01343 | 0 | 0.013527 | 0 | 0.013995 | 0.011088 |
| Sfswap | 7.92472 | 7.084381 | 8.558234 | 8.37236 | 7.890161 | 7.512529 | 9.638292 | 8.69316 |
| Armc6 | 4.028148 | 3.771856 | 3.549051 | 3.942166 | 3.604616 | 4.067755 | 4.226798 | 3.98894 |
| AABR07042 | 0 | 0 | 0 | 0.714861 | 0 | 0 | 0 | 0.687622 |
| Lat2 | 2.478387 | 2.713721 | 2.403219 | 2.283736 | 2.506951 | 3.715218 | 3.398805 | 2.834474 |
| AABR07017 | 0 | 0 | 0 | 0.14234 | 0 | 0 | 0 | 0 |
| Atp5d | 221.9623 | 226.2652 | 232.0633 | 207.6302 | 203.9311 | 205.8846 | 205.7634 | 200.1733 |
| Il18rap | 0.384971 | 0.346227 | 0.434485 | 0.227395 | 0.448284 | 0.268303 | 0.342342 | 0.341219 |
| Pigf | 8.251105 | 6.562746 | 6.490084 | 7.90418 | 6.978438 | 6.804799 | 7.311659 | 7.89264 |
| Enkd1 | 0.44349 | 0.733382 | 0.585987 | 0.880187 | 0.959078 | 0.658058 | 0.877816 | 0.846649 |
| Ccdc184 | 0.149617 | 0.084642 | 0 | 0.034467 | 0.040445 | 0.065592 | 0.041846 | 0.066306 |
| AABR07030 | 0.563074 | 0.637092 | 0.75562 | 0.129713 | 0.456633 | 0.493704 | 0.472457 | 0.748621 |
| Acsl6 | 5.673904 | 5.298401 | 4.482025 | 4.109564 | 4.460681 | 4.290566 | 4.14403 | 4.128663 |
| LOC102552 | 0 | 0.07953 | 0 | 0 | 0 | 0 | 0 | 0 |
| C4a | 0.204248 | 0.177767 | 0.404811 | 0.535663 | 0.645561 | 0.57858 | 2.056527 | 0.877322 |
| LOC102556 | 7.079976 | 8.401424 | 7.786192 | 11.17818 | 10.50294 | 9.652262 | 9.949251 | 11.17316 |
| Actb | 344.5297 | 345.8746 | 344.9178 | 418.2685 | 407.3055 | 423.6703 | 403.4775 | 402.1515 |
| Noc4l | 3.846927 | 4.116428 | 4.482086 | 3.572271 | 3.611459 | 3.2684 | 4.003508 | 3.383291 |

| | | | | | | | | |
|---|---|---|---|---|---|---|---|---|
| AABR07044 | 0 | 0 | 0 | 1.956208 | 1.530338 | 1.240932 | 0 | 0.627223 |
| Dnajc19 | 24.43891 | 23.98137 | 23.04062 | 19.75574 | 20.22747 | 17.92165 | 19.53651 | 17.70329 |
| AABR07049 | 0.061607 | 0 | 0.03307 | 0 | 0.033307 | 0 | 0.241231 | 0.081908 |
| Mir6315 | 0 | 0 | 0 | 0 | 0 | 0 | 0 | 0 |
| Vamp1 | 11.85343 | 10.0362 | 11.95677 | 12.88339 | 13.01049 | 13.3227 | 11.23633 | 11.24666 |
| Dbn1 | 7.722954 | 7.573072 | 8.982009 | 10.91181 | 10.87913 | 10.53343 | 14.20816 | 14.79332 |
| Arid3b | 2.052057 | 2.104136 | 1.913868 | 1.441803 | 1.525466 | 1.585585 | 1.865297 | 1.92115 |
| Dock9 | 16.1107 | 15.30388 | 15.52278 | 14.35317 | 14.20708 | 13.78673 | 15.50993 | 15.59667 |
| LOC316124 | 0.187523 | 0.053043 | 0.025165 | 0.086398 | 0.050692 | 0.08221 | 0.104896 | 0.062329 |
| Foxn3 | 15.32614 | 15.66621 | 15.05284 | 13.0637 | 12.38157 | 12.2598 | 17.71266 | 17.22636 |
| AC135771. | 0 | 0 | 0 | 0 | 0 | 0 | 0 | 0.80025 |
| Syap1 | 14.71782 | 13.8075 | 12.90293 | 13.15801 | 13.84383 | 13.73389 | 12.51699 | 13.76078 |
| Pcdhgc3 | 15.53036 | 17.06439 | 16.53239 | 13.2257 | 14.10511 | 14.55106 | 17.14469 | 16.64761 |
| AC136013. | 0.41233 | 0 | 0 | 0.379946 | 0.445847 | 0 | 0 | 0.365469 |
| Kcnj3 | 12.12413 | 11.93423 | 12.8314 | 10.45881 | 9.080677 | 9.801122 | 9.202955 | 9.221932 |
| A930003A1 | 0.405937 | 0 | 0 | 0 | 0 | 0.177963 | 0 | 0 |
| Ghrl | 5.446056 | 5.213963 | 4.160144 | 3.474226 | 4.076821 | 3.58133 | 4.569604 | 4.641452 |
| AC116288. | 2.233617 | 0.842411 | 1.865057 | 0.686064 | 1.610119 | 1.740834 | 1.943567 | 1.09987 |
| Ndufb5 | 312.9135 | 309.4145 | 292.2603 | 287.247 | 277.6209 | 269.8665 | 250.7225 | 247.107 |
| AC110306. | 0 | 0 | 0 | 0.135163 | 0 | 0 | 0 | 0 |
| AABR07008 | 0 | 0 | 0 | 0 | 0 | 0 | 0 | 0.602786 |
| Ripk4 | 0 | 0.03278 | 0 | 0.026696 | 0 | 0.012701 | 0 | 0 |
| AABR07025 | 0 | 0 | 0 | 0 | 0 | 0 | 0 | 0 |
| Scamp1 | 22.71378 | 21.98653 | 21.54107 | 19.91468 | 21.56927 | 21.49801 | 21.73979 | 20.98418 |
| LOC102551 | 1.778982 | 2.064445 | 1.713968 | 1.807391 | 1.627651 | 1.279846 | 1.377862 | 1.253354 |
| Dlgap3 | 0.127791 | 0.224917 | 0.167679 | 0.222425 | 0.199591 | 0.211645 | 0.222393 | 0.176194 |
| Olr786 | 0 | 0.063301 | 0 | 0.051552 | 0 | 0 | 0 | 0 |
| Ccar1 | 4.707977 | 4.993092 | 5.915701 | 5.262397 | 5.817906 | 5.793626 | 5.451885 | 6.682938 |
| Tmem200c | 0.358905 | 0.29006 | 0.220176 | 0.535446 | 0.351119 | 0.404599 | 0.439767 | 0.439302 |
| Ntng2 | 4.071853 | 5.196391 | 4.345979 | 5.911663 | 5.605936 | 5.791203 | 7.654145 | 7.155222 |
| RGD156152 | 0 | 0 | 0.098628 | 0 | 0 | 0 | 0 | 0 |
| AABR07070 | 0 | 0 | 0 | 0.151264 | 0 | 0 | 0.183651 | 0 |
| AABR07056 | 0 | 0 | 0 | 0 | 0 | 0 | 0 | 0.151681 |
| AABR07005 | 0 | 0 | 0 | 0 | 1.048565 | 0 | 0 | 0.859528 |
| Smad5 | 8.735236 | 8.081827 | 8.400976 | 7.588026 | 7.133158 | 7.060698 | 8.958211 | 9.032886 |
| Pdcd5 | 1.87312 | 1.566474 | 1.836052 | 1.80105 | 1.497018 | 41.7729 | 52.29798 | 18.76792 |
| Fosl2 | 19.21491 | 22.93532 | 24.63323 | 14.65754 | 14.00342 | 13.02145 | 30.00102 | 28.75953 |
| Frmd8 | 6.030345 | 6.555884 | 7.331956 | 6.996119 | 6.736567 | 8.010748 | 8.128292 | 8.661467 |
| Ikzf2 | 0.727764 | 0.973145 | 1.207465 | 0.457231 | 0.786921 | 0.55109 | 0.740173 | 0.762336 |
| AABR07036 | 0.489401 | 1.107469 | 0 | 0.901928 | 0 | 0.429107 | 0 | 0.867561 |
| AABR07043 | 0.277068 | 0 | 0 | 0.255308 | 0.59918 | 0.121467 | 0 | 0.12279 |
| AABR07008 | 0 | 0 | 0 | 0 | 0 | 0 | 0.138498 | 0 |
| AABR07062 | 0 | 0 | 0 | 0 | 0.740164 | 0.300095 | 0 | 0.303363 |
| Pcgf3 | 5.774051 | 6.902863 | 6.978537 | 5.855963 | 6.400464 | 5.572146 | 7.191044 | 8.561901 |
| AABR07058 | 0.204023 | 0.589931 | 0.438064 | 0.313332 | 0.318655 | 0.397528 | 0.101445 | 0.14065 |
| Tmem221 | 0 | 0 | 0 | 0.069629 | 0.081706 | 0 | 0.084538 | 0.401857 |
| Rn60_1_10 | 0 | 0.122924 | 0 | 0 | 0 | 0 | 0 | 0.096296 |
| Acmsd | 1.968254 | 2.461407 | 2.724718 | 1.909125 | 1.7362 | 1.362448 | 1.506628 | 1.331376 |
| Psmb5 | 103.9521 | 109.2011 | 107.7354 | 107.5608 | 101.5703 | 107.3968 | 99.85565 | 100.9104 |
| AABR07042 | 0.988036 | 0 | 0 | 0.455218 | 0 | 0.433156 | 0.552686 | 0.437873 |
| RT1-M6-2 | 2.229494 | 2.707146 | 2.627017 | 2.355034 | 2.763508 | 2.622323 | 2.433423 | 2.747277 |
| Mphosph9 | 1.032837 | 1.349319 | 1.108817 | 1.108704 | 1.738511 | 1.110985 | 1.250791 | 1.472278 |
| AABR07044 | 0.65309 | 0.470235 | 0.828612 | 1.094175 | 0.706176 | 0.624687 | 0.53138 | 1.315604 |
| Mill1 | 0.949298 | 0.849277 | 0.687322 | 0.447542 | 0.907106 | 0.580706 | 0.419873 | 0.410921 |

| | | | | | | | | |
|---|---|---|---|---|---|---|---|---|
| LOC102551 | 0 | 0 | 0.170358 | 0 | 0 | 0 | 0 | 0.14065 |
| Polr2f | 61.71996 | 61.612 | 60.88059 | 59.56607 | 58.15773 | 57.19173 | 55.5947 | 55.00157 |
| Sema6d | 6.59484 | 6.57289 | 5.892587 | 5.733989 | 6.113801 | 5.854867 | 7.331756 | 7.751023 |
| Sall4 | 1.495433 | 1.22525 | 1.273282 | 0.902816 | 0.836374 | 0.904273 | 0.951893 | 0.891268 |
| Tspan14 | 14.18466 | 15.60681 | 15.11364 | 13.79129 | 12.45465 | 12.46829 | 15.07372 | 16.13322 |
| Stau2 | 23.73849 | 24.15465 | 22.3648 | 22.9753 | 22.40818 | 20.50434 | 23.33663 | 21.16098 |
| Kat6b | 6.844544 | 7.977775 | 7.53268 | 6.528831 | 5.504192 | 6.634605 | 7.522694 | 7.072688 |
| Slc22a14 | 0.057324 | 0 | 0 | 0.026411 | 0.092976 | 0 | 0.192396 | 0.025405 |
| Syngap1 | 1.130987 | 1.761251 | 1.631972 | 1.736934 | 1.748907 | 1.450156 | 2.231278 | 2.328271 |
| Igflr1 | 0.386623 | 0.19442 | 0.737892 | 0.514595 | 0.60385 | 0.602651 | 0.672835 | 0.64729 |
| RGD156266 | 1.323625 | 1.872025 | 1.687433 | 1.219669 | 1.073413 | 1.450695 | 0.740407 | 1.026545 |
| Ldlrad4 | 4.542379 | 4.053408 | 4.19566 | 5.74931 | 4.985747 | 5.951599 | 6.040645 | 6.320275 |
| AABR07052 | 0 | 0.580878 | 0.551158 | 0.47307 | 0.555123 | 0.450142 | 0 | 0.455044 |
| AABR07065 | 0.315078 | 0.142598 | 0.811815 | 0.696796 | 0.136276 | 0.497269 | 0.634491 | 0.502684 |
| Rn50_X_07 | 0 | 0 | 0 | 0 | 0 | 0 | 0 | 0.114321 |
| Lix1 | 0 | 0.020673 | 0.058847 | 0.016836 | 0 | 0.032041 | 0 | 0.03239 |
| Csell | 13.59907 | 13.23819 | 13.06269 | 13.67509 | 14.73607 | 14.40831 | 13.69427 | 12.54551 |
| Tmem200a | 0 | 0.028966 | 0.013742 | 0.09436 | 0.041522 | 0.022447 | 0.042961 | 0.056728 |
| Ankrd29 | 0.318528 | 0.14416 | 0.205176 | 0.704425 | 0.206652 | 0.223428 | 0.498896 | 0.056465 |
| Snx22 | 6.740786 | 5.932019 | 5.762529 | 4.025887 | 3.509381 | 2.790988 | 4.189607 | 4.204414 |
| Il17d | 8.677781 | 6.884236 | 7.817002 | 10.66165 | 10.78524 | 13.29334 | 14.50668 | 13.26129 |
| Ak2 | 27.44161 | 30.48628 | 29.42695 | 29.75371 | 30.88195 | 29.18363 | 27.6756 | 29.08827 |
| Psen1 | 11.59933 | 11.34024 | 11.60633 | 12.53025 | 13.09015 | 12.83631 | 13.95323 | 14.62307 |
| AC099150. | 0 | 0.066275 | 0 | 0 | 0 | 0 | 0 | 0 |
| Rpl36a-ps | 34.58435 | 37.28478 | 39.05498 | 33.22101 | 31.92734 | 31.89698 | 33.94626 | 41.78753 |
| Il31ra | 0.07273 | 0.054861 | 0.052054 | 0.156376 | 0 | 0.085027 | 0.081368 | 0.23637 |
| Pip4k2a | 2.830077 | 2.492909 | 3.119833 | 3.640425 | 3.696791 | 3.613871 | 5.014864 | 4.562327 |
| Cetn2 | 25.77257 | 26.56015 | 22.86616 | 24.50473 | 25.02751 | 25.00828 | 21.39534 | 25.20788 |
| Pi4kb | 11.58325 | 11.38965 | 11.19552 | 12.51595 | 11.92838 | 12.18139 | 14.09657 | 13.62796 |
| Tmc8 | 3.748879 | 4.107732 | 4.681316 | 3.67262 | 4.032274 | 4.428828 | 4.723858 | 5.491394 |
| Pde1a | 5.023475 | 5.856063 | 5.164226 | 8.668732 | 9.382219 | 8.835869 | 11.1379 | 11.52267 |
| AABR07072 | 0 | 0 | 0 | 0 | 0 | 0 | 0 | 0 |
| Fhl1 | 84.56481 | 80.7269 | 79.45892 | 156.2452 | 154.2951 | 156.405 | 139.7823 | 143.8285 |
| AABR07027 | 0.033698 | 0 | 0 | 0 | 0 | 0 | 0 | 0.014934 |
| Cd68 | 4.936997 | 3.962703 | 4.439468 | 7.309903 | 7.802135 | 7.436593 | 9.819187 | 10.80886 |
| Irs2 | 9.440526 | 9.833917 | 11.94236 | 7.971676 | 7.795285 | 7.761886 | 16.28403 | 16.55672 |
| AC123293. | 0.396712 | 0 | 0 | 0.365554 | 0 | 0 | 0 | 0 |
| Cdh23 | 2.517356 | 2.931837 | 3.151864 | 2.166514 | 1.983252 | 2.487844 | 2.527101 | 2.662234 |
| AABR07001 | 1.999524 | 1.406337 | 1.334384 | 1.444108 | 1.694585 | 1.231968 | 1.08826 | 1.149586 |
| Styk1 | 0.081187 | 0.13779 | 0.17432 | 0.224433 | 0.219467 | 0.071185 | 0.136243 | 0.07196 |
| Gpsm3 | 2.579999 | 2.554254 | 2.596682 | 2.711685 | 3.051252 | 2.438876 | 4.058984 | 4.037599 |
| AC140988. | 16.79034 | 14.67132 | 16.86545 | 15.62482 | 14.38032 | 17.12683 | 14.3207 | 15.10314 |
| Mc5r | 0.032893 | 0 | 0 | 0 | 0 | 0.028841 | 0 | 0 |
| Gdf10 | 0.098321 | 0.111246 | 0.084443 | 0.507356 | 0.467779 | 0.568974 | 0.461989 | 0.697176 |
| AABR07028 | 0 | 0 | 0 | 0.106284 | 0 | 0 | 0 | 0 |
| Setd5 | 7.001264 | 7.405156 | 6.781271 | 7.394337 | 6.511629 | 6.49374 | 7.725631 | 8.045705 |
| Gprasp2 | 1.548648 | 1.324354 | 1.198599 | 1.12834 | 1.285105 | 1.2947 | 1.47066 | 1.372644 |
| C1qtnf5 | 5.138529 | 4.349407 | 4.547986 | 21.32536 | 19.51038 | 19.56954 | 25.01368 | 22.52929 |
| Trak1 | 77.46613 | 80.75663 | 87.10997 | 64.23673 | 61.50662 | 61.71797 | 82.66458 | 80.22693 |
| Fcrl2 | 0.239583 | 0.271076 | 0.220463 | 2.081508 | 2.109466 | 2.010634 | 1.148719 | 1.122443 |
| L1cam | 0.719444 | 0.683774 | 0.875351 | 1.078381 | 1.006115 | 1.051348 | 1.244884 | 0.952267 |
| LOC689130 | 22.82617 | 19.85837 | 17.91568 | 13.52148 | 13.58525 | 12.61387 | 18.13335 | 17.68172 |
| Map7d1 | 67.49846 | 75.48728 | 81.36569 | 67.9423 | 64.36414 | 63.57283 | 82.63221 | 80.66007 |
| Rbpms2 | 18.47303 | 19.74018 | 19.19846 | 17.06946 | 16.22938 | 15.97221 | 16.9066 | 19.35265 |

| | | | | | | | | |
|---|---|---|---|---|---|---|---|---|
| Krt81 | 0.061354 | 0.104129 | 0.098802 | 0.056536 | 0 | 0 | 0.06864 | 0.081572 |
| Cuzd1 | 0.0504 | 0 | 0.027054 | 0.046442 | 0 | 0.022096 | 0.112771 | 0.089345 |
| Rn50_7_14 | 0 | 0 | 0 | 0 | 0.071134 | 0 | 0.036799 | 0.029155 |
| AABR07044 | 0 | 0 | 0 | 0.129887 | 0.076208 | 0 | 0 | 0 |
| Zfp367 | 2.384359 | 2.409347 | 2.205581 | 2.653093 | 3.275415 | 2.235241 | 2.264871 | 2.458959 |
| Bcor | 3.789424 | 3.927179 | 3.945442 | 3.311195 | 2.702197 | 3.186517 | 3.919654 | 4.147773 |
| Elmo1 | 4.721723 | 4.688815 | 5.042108 | 4.096309 | 4.399447 | 4.822673 | 4.973375 | 5.275895 |
| AABR07015 | 0 | 0.569707 | 0.540559 | 0.927945 | 0 | 0 | 0 | 0 |
| Plekhg5 | 11.99334 | 14.09573 | 13.35845 | 12.36374 | 11.52551 | 11.50162 | 17.22486 | 16.34408 |
| Rpl22l2 | 0.141913 | 0 | 0.152353 | 0.130767 | 0 | 0.12443 | 0.158766 | 0 |
| Ndor1 | 3.579496 | 4.097679 | 3.888027 | 3.70581 | 3.733853 | 4.707758 | 4.452152 | 4.441759 |
| Etl4 | 8.900723 | 9.575205 | 8.903294 | 7.980351 | 7.057764 | 7.766999 | 8.985669 | 9.185228 |
| Snupn | 7.621147 | 6.133865 | 7.464827 | 6.91399 | 6.159238 | 6.578895 | 6.900069 | 8.356702 |
| Dppa5 | 0 | 0.170257 | 0 | 0.138658 | 0 | 0 | 0 | 0 |
| LOC103694 | 0.888621 | 0.816175 | 1.032556 | 0.655064 | 0.87042 | 0.861641 | 0.807016 | 0.806162 |
| Gja8 | 0 | 0 | 0 | 0 | 0 | 0 | 0 | 0.028704 |
| Tgfbrap1 | 3.406797 | 2.569753 | 2.745711 | 2.757062 | 2.850879 | 2.727336 | 3.601471 | 3.588526 |
| Lactb2 | 44.60478 | 42.08151 | 39.0859 | 37.16415 | 40.49853 | 36.81569 | 38.92636 | 35.97995 |
| LOC102551 | 0.059574 | 0.202217 | 0.127914 | 0.164686 | 0.064417 | 0.261175 | 0.199948 | 0.158411 |
| Zfp422 | 8.191809 | 9.069321 | 8.723508 | 8.157174 | 9.21485 | 8.379683 | 8.376277 | 8.84179 |
| AABR07029 | 0.08419 | 0 | 0.045191 | 0 | 0.045516 | 0 | 0.141281 | 0 |
| Lilra5 | 1.398816 | 1.258039 | 1.155168 | 1.817755 | 1.202259 | 1.855448 | 1.564933 | 2.034609 |
| Rps29 | 1.224934 | 4.504354 | 3.945134 | 4.797095 | 3.97351 | 2.685058 | 3.083403 | 2.985729 |
| Sp6 | 0.052497 | 0.029699 | 0.02818 | 0.096748 | 0.056764 | 0.023015 | 0.058731 | 0.046531 |
| Patz1 | 8.941027 | 8.691184 | 8.468193 | 7.344523 | 7.814621 | 6.572144 | 6.837958 | 7.375809 |
| Myh7 | 1726.608 | 1664.02 | 1775.926 | 2065.272 | 1973.27 | 1940.711 | 2444.354 | 2582.984 |
| Rsl1 | 0.539061 | 0.697054 | 0.633832 | 0.662298 | 0.527367 | 0.900284 | 0.918975 | 0.978345 |
| Slc12a7 | 70.38384 | 69.18979 | 69.72291 | 66.84987 | 66.48076 | 67.99897 | 77.32208 | 77.71555 |
| Ky | 6.675216 | 6.662864 | 6.795602 | 3.216876 | 3.588167 | 3.582339 | 2.918504 | 3.255817 |
| Susd2 | 9.173226 | 9.849722 | 10.51176 | 9.484343 | 8.310898 | 9.713243 | 11.04771 | 11.69989 |
| Adrb3 | 0.072496 | 0.027342 | 0.025943 | 0 | 0.052259 | 0 | 0.10814 | 0.064256 |
| Gcnt4 | 0.038279 | 0 | 0.041095 | 0.176364 | 0.165563 | 0.302069 | 0.042825 | 0.305359 |
| AI854703 | 0.631866 | 0.089366 | 0.93273 | 0.87336 | 0.597824 | 0.623274 | 1.502171 | 1.190116 |
| AABR07001 | 0 | 0 | 0 | 0 | 0 | 0 | 0 | 0 |
| AABR07003 | 0 | 0.058896 | 0.111766 | 0 | 0 | 0 | 0 | 0 |
| AABR07072 | 0 | 0 | 0 | 0 | 0.45298 | 0 | 0 | 0 |
| AABR07049 | 0 | 0 | 0 | 0 | 0 | 0 | 0 | 0.078536 |
| AC094571. | 0.187691 | 0.212364 | 0.402998 | 0.691801 | 0.202948 | 0.658272 | 1.259885 | 0.499081 |
| Serf1 | 23.5482 | 19.1909 | 20.68404 | 24.73353 | 25.64038 | 23.10163 | 18.79131 | 23.20726 |
| Rhbdd1 | 5.615815 | 5.1817 | 5.139056 | 5.327513 | 5.444903 | 5.378191 | 6.908678 | 6.281664 |
| Zbtb26 | 1.179944 | 0.987938 | 1.39342 | 1.174254 | 1.607579 | 1.117342 | 1.478477 | 1.213178 |
| Htr2a | 0.147382 | 0.166756 | 0.267763 | 0.43876 | 0.294207 | 0.208747 | 0.279035 | 0.231118 |
| AABR07027 | 0.058379 | 0.132106 | 0 | 0.053794 | 0.126249 | 0.051187 | 0.261247 | 0 |
| Bbs4 | 4.694156 | 4.390606 | 4.031581 | 4.748416 | 5.1434 | 5.487787 | 5.905148 | 5.103748 |
| Ccnk | 8.836206 | 8.573011 | 7.878001 | 7.64208 | 7.34778 | 7.861809 | 10.41991 | 9.794772 |
| AABR07065 | 0 | 0 | 0 | 0.077953 | 0.182948 | 0.074175 | 0 | 0 |
| Zfp467 | 2.478654 | 3.475975 | 2.885866 | 3.651154 | 2.868874 | 3.428282 | 5.135922 | 3.960706 |
| Leng9 | 3.447962 | 3.494831 | 3.778724 | 3.408829 | 3.767067 | 3.715987 | 4.098516 | 4.106634 |
| AC098125. | 1.028674 | 0.945665 | 0.759239 | 0.770154 | 0.764699 | 0.761014 | 1.150834 | 1.111213 |
| Setd2 | 7.33464 | 7.63074 | 7.155807 | 7.042709 | 7.313682 | 7.161555 | 8.367141 | 8.449047 |
| Serpine2 | 20.7507 | 21.70154 | 19.20925 | 55.89203 | 56.26062 | 57.87844 | 57.17333 | 58.0752 |
| AABR07043 | 0 | 0 | 0 | 0.164126 | 0 | 0 | 0 | 0 |
| Afap1l2 | 4.386916 | 4.859092 | 4.288246 | 5.638043 | 5.192894 | 4.959898 | 5.062872 | 6.425996 |
| Kdm7a | 3.740423 | 3.7998 | 3.152017 | 3.150166 | 3.609576 | 3.138548 | 3.352195 | 4.188714 |

| | | | | | | | | |
|---|---|---|---|---|---|---|---|---|
| AC134024. | 0.379463 | 0 | 0 | 0 | 0 | 0 | 0 | 0.672674 |
| Hpse2 | 0.45935 | 0 | 0.123285 | 0.105818 | 0.124172 | 0.302069 | 0 | 0 |
| Snx9 | 15.39828 | 15.39425 | 16.31021 | 14.54093 | 13.66312 | 13.47546 | 17.22692 | 18.07614 |
| Ddr2 | 7.416873 | 7.235985 | 7.902393 | 10.00653 | 10.21384 | 10.0737 | 14.0121 | 13.80528 |
| Tesk1 | 8.717935 | 9.61651 | 11.04956 | 9.067613 | 8.575347 | 8.9477 | 10.17729 | 10.53113 |
| Tubb4a | 0.048803 | 0.165656 | 0.026197 | 0.134911 | 0.184696 | 0.213954 | 0.245695 | 0.302797 |
| Uimc1 | 10.47712 | 10.05286 | 10.00278 | 10.74103 | 10.71237 | 9.789575 | 11.74332 | 12.71869 |
| RGD156240 | 12.61972 | 14.14517 | 13.67469 | 12.60667 | 14.15563 | 13.34002 | 10.81969 | 16.93503 |
| Uba7 | 4.723367 | 4.562568 | 4.692415 | 6.924858 | 5.976313 | 6.255447 | 7.918552 | 7.523301 |
| LOC103694 | 0.055768 | 0 | 0.179611 | 0.102776 | 0.241203 | 0.146692 | 0.124781 | 0.148289 |
| Cyp4b1 | 29.85123 | 30.40676 | 29.82131 | 27.35977 | 27.62144 | 29.85607 | 25.06948 | 26.19946 |
| AABR07057 | 0 | 0 | 0 | 0 | 0 | 0 | 0 | 0.079341 |
| Oaf | 28.70382 | 30.90217 | 34.00319 | 37.65056 | 34.21431 | 39.59548 | 42.35536 | 41.15073 |
| Gsap | 4.056624 | 3.787086 | 3.593326 | 4.207048 | 4.419725 | 4.381824 | 4.469346 | 4.374859 |
| Ptprcap | 4.201816 | 5.108945 | 4.376264 | 3.236138 | 4.543363 | 4.45398 | 3.016935 | 3.613106 |
| Acyp2 | 46.75693 | 45.67263 | 45.70502 | 41.94083 | 45.28807 | 45.43075 | 41.71393 | 44.70301 |
| Dhx40 | 6.56815 | 6.730811 | 6.333948 | 8.019662 | 7.648357 | 8.545553 | 6.655276 | 7.165142 |
| Gja1 | 109.672 | 107.2252 | 107.6361 | 97.29326 | 102.1269 | 104.8236 | 101.6379 | 103.3974 |
| Palld | 29.25461 | 32.54887 | 36.78749 | 23.99026 | 21.67801 | 21.78993 | 35.96074 | 36.40354 |
| Smc1a | 14.99003 | 14.20323 | 13.82638 | 15.20966 | 16.40766 | 15.46632 | 15.39116 | 14.57989 |
| Wasl | 9.679579 | 9.890802 | 9.513843 | 9.019071 | 8.802168 | 9.235612 | 11.94562 | 11.56366 |
| Sv2a | 1.324478 | 1.24882 | 1.211258 | 1.231755 | 1.365836 | 1.698943 | 1.564087 | 1.576137 |
| Has1 | 0.299805 | 0.621894 | 0.48279 | 1.220141 | 1.431772 | 1.314346 | 2.236056 | 3.078062 |
| Zfyve21 | 24.89791 | 26.29276 | 27.93663 | 27.53093 | 26.51647 | 26.61913 | 29.05272 | 28.00058 |
| Slc38a1 | 15.80379 | 15.46641 | 15.27519 | 14.35185 | 13.29709 | 14.6365 | 20.72209 | 20.42599 |
| AABR07067 | 0 | 0.74062 | 0 | 0 | 0.707781 | 0 | 0 | 0.580181 |
| Nr2c2 | 5.463006 | 5.875603 | 5.463487 | 5.627299 | 5.031116 | 5.31814 | 6.669496 | 6.848949 |
| Xpnpep3 | 3.194224 | 3.285559 | 2.926947 | 3.783248 | 3.706803 | 3.557453 | 3.519412 | 3.660541 |
| Tmco4 | 1.572706 | 2.053203 | 2.078031 | 2.118042 | 2.709658 | 2.091155 | 3.074245 | 3.216848 |
| Hspb6 | 821.5853 | 861.8778 | 875.5013 | 936.4055 | 905.8305 | 897.3653 | 1012.71 | 997.5578 |
| AABR07006 | 1.032906 | 1.059969 | 1.521501 | 1.084589 | 1.376602 | 0.716097 | 1.128695 | 1.618121 |
| AABR07065 | 0 | 0 | 0 | 0.386025 | 0 | 0.367316 | 0 | 0 |
| Frem2 | 0.67259 | 0.785163 | 0.670492 | 0.403339 | 0.282824 | 0.294864 | 0.531502 | 0.454209 |
| Eapp | 23.62407 | 22.43832 | 21.78086 | 21.60023 | 21.09757 | 19.55172 | 21.11297 | 21.5467 |
| Aqp12a | 0 | 0 | 0 | 0 | 0 | 0.042008 | 0 | 0 |
| Klf11 | 4.956901 | 4.402203 | 4.414805 | 5.58828 | 5.060394 | 4.807545 | 7.156562 | 7.535302 |
| Golga1 | 3.209411 | 2.799401 | 3.370333 | 3.925205 | 3.722675 | 3.550775 | 3.812508 | 3.713575 |
| Utp14a | 3.124218 | 2.622672 | 2.488487 | 3.120295 | 3.835859 | 4.082466 | 3.607986 | 3.269383 |
| Thoc1 | 7.575603 | 8.452939 | 7.158446 | 7.656164 | 8.719867 | 7.927901 | 8.7877 | 10.05648 |
| AABR07070 | 0 | 0.3898 | 0 | 0 | 0 | 0 | 0 | 0 |
| AABR07061 | 9.0509 | 10.24067 | 7.403215 | 9.531483 | 9.320578 | 5.290558 | 8.438121 | 13.17943 |
| Eps8l3 | 0 | 0 | 0 | 0 | 0 | 0 | 0 | 0 |
| Ppil6 | 0.295186 | 0.333989 | 0.25352 | 0.707205 | 0.446852 | 0.41411 | 0.594433 | 0.62793 |
| Abca8a | 21.39985 | 20.00429 | 19.00091 | 19.74502 | 21.62982 | 20.35715 | 16.94962 | 16.82516 |
| AABR07065 | 0 | 0 | 0 | 0 | 0.164123 | 0 | 0 | 0 |
| Mir374b | 0 | 0.871317 | 0 | 0 | 0 | 0.675213 | 0.861539 | 0.682566 |
| AABR07065 | 0 | 0 | 0.499717 | 0.214458 | 0 | 0 | 0.781129 | 0.206287 |
| Bhlhe40 | 24.70631 | 24.76213 | 24.53629 | 27.84303 | 28.02528 | 27.5371 | 29.16905 | 29.32163 |
| Tob1 | 27.96464 | 30.7642 | 28.46945 | 25.93487 | 26.46851 | 26.28536 | 27.64572 | 27.00647 |
| AABR07001 | 0 | 0.133145 | 0 | 0 | 0 | 0.103179 | 0 | 0 |
| Pigy | 3.533038 | 3.997466 | 4.431358 | 5.640814 | 5.219711 | 4.661992 | 4.343953 | 4.743768 |
| Pcdhga9 | 0.562377 | 0.391571 | 0.650189 | 0.259104 | 0.420985 | 0.189651 | 0.435574 | 0.575149 |
| Plpp3 | 54.25375 | 54.82366 | 55.70692 | 44.42924 | 45.01848 | 42.30575 | 51.56364 | 52.35389 |
| AABR07044 | 0 | 0 | 0 | 0 | 0.716741 | 0 | 0 | 0 |

| | | | | | | | | |
|---|---|---|---|---|---|---|---|---|
| Ccdc63 | 0.284083 | 0.107142 | 0.169434 | 0.2036 | 0.136522 | 0.193732 | 0.282506 | 0.307751 |
| Bcar1 | 12.01346 | 11.90307 | 12.44689 | 11.78112 | 9.524774 | 11.34254 | 13.49644 | 13.17631 |
| Atg4b | 11.93239 | 11.83365 | 12.96452 | 14.10833 | 12.04734 | 11.88041 | 13.99277 | 12.96548 |
| Cldn11 | 0.45265 | 0.512152 | 0.546692 | 0.260687 | 0.214132 | 0.545715 | 0.316503 | 0.401206 |
| Banf1 | 170.8241 | 174.4873 | 181.9906 | 165.7555 | 162.8176 | 157.3609 | 164.5438 | 160.5346 |
| AABR0704( | 0.395214 | 0 | 0.424288 | 0.182087 | 0.64101 | 0.173262 | 0.442148 | 0.175149 |
| Fgg | 0.135663 | 0.038374 | 0.145643 | 0 | 0 | 0.059475 | 0.037943 | 0.090184 |
| Edil3 | 0.059237 | 0.093834 | 0.063595 | 0.043668 | 0.076863 | 0.103879 | 0.079527 | 0.073507 |
| Szrd1 | 28.1859 | 25.86594 | 26.23514 | 21.70279 | 22.17933 | 22.07563 | 27.80747 | 27.535 |
| AC110855. | 4.887486 | 3.949972 | 1.499151 | 1.930125 | 2.2649 | 4.285352 | 2.343387 | 3.094301 |
| Olig1 | 0 | 0 | 0 | 0 | 0 | 0.021257 | 0 | 0 |
| Arhgef28 | 0.828387 | 0.803384 | 0.862103 | 0.950264 | 1.096804 | 1.133966 | 1.011874 | 1.153807 |
| Arhgef26 | 0.372214 | 0.579072 | 0.349646 | 0.610935 | 0.616282 | 0.683312 | 0.416417 | 0.680444 |
| AABR07068 | 0.020416 | 0 | 0.010959 | 0.018812 | 0 | 0 | 0 | 0.027143 |
| Gmeb1 | 2.258125 | 2.273916 | 1.975757 | 1.955927 | 2.258552 | 2.14822 | 2.778919 | 2.391779 |
| AC132671. | 0 | 0.352676 | 0.334632 | 0.287221 | 0.674078 | 0.546601 | 0.348718 | 0.828831 |
| Vwa2 | 0.941686 | 0.710315 | 1.201431 | 0.515606 | 0.427952 | 0.430785 | 0.778686 | 0.677408 |
| Rfx3 | 0.661502 | 0.606858 | 0.652584 | 0.329485 | 0.657277 | 0.470275 | 0.480038 | 0.586322 |
| AABR07015 | 0 | 0 | 0 | 0 | 0 | 0 | 0 | 0 |
| AABR07065 | 0 | 0 | 0 | 0 | 0 | 0 | 0 | 0.276277 |
| Ptgdrl | 0.239114 | 0.315637 | 0.085568 | 0.110167 | 0.129275 | 0.209655 | 0.044585 | 0.035323 |
| Rab9a | 8.192467 | 7.219878 | 6.054944 | 7.359362 | 8.368736 | 6.858296 | 8.059997 | 8.319583 |
| Rn60_20_( | 0.022388 | 0.025331 | 0 | 0.082519 | 0.169456 | 0.03926 | 0.200375 | 0.119062 |
| Chd6 | 3.413867 | 3.591568 | 3.450676 | 3.467677 | 3.480892 | 3.400263 | 3.891871 | 4.025659 |
| Mzt1 | 36.01538 | 32.49977 | 27.99048 | 31.55796 | 29.86419 | 31.77205 | 32.38224 | 27.41786 |
| Rab19 | 0.223256 | 0.168402 | 0.133155 | 0.11429 | 0.026823 | 0.0435 | 0.055504 | 0.043974 |
| Wdr77 | 2.270199 | 2.363133 | 2.372211 | 2.036115 | 1.603759 | 2.123213 | 1.625471 | 1.824386 |
| Ppp1r13b | 8.969517 | 8.576516 | 9.297874 | 7.468425 | 7.461752 | 8.216419 | 9.568368 | 8.141698 |
| Pdpr | 17.29374 | 17.35376 | 15.87118 | 14.61153 | 13.49581 | 12.91677 | 15.43536 | 16.60182 |
| Fam234b | 3.605811 | 3.170023 | 3.129226 | 3.403652 | 3.273998 | 3.18361 | 3.682625 | 3.663718 |
| Arfgef1 | 8.211157 | 8.232662 | 8.637117 | 8.802984 | 8.37312 | 8.158168 | 9.7852 | 9.65716 |
| LOC10369( | 11.6568 | 8.589836 | 7.380238 | 7.27102 | 9.113898 | 7.495173 | 12.03795 | 7.205907 |
| AABR07044 | 0.189633 | 0.551729 | 0.26175 | 0.324517 | 0.11717 | 0.118765 | 0.333384 | 0.048023 |
| Chst12 | 3.147146 | 3.450719 | 3.239336 | 3.79687 | 4.525591 | 3.897326 | 4.718715 | 5.205098 |
| LOC102548 | 0.453385 | 0.897721 | 1.338528 | 0.626664 | 0.612798 | 0.99382 | 0.38042 | 0.70325 |
| Adh6a | 0.037113 | 0 | 0 | 0 | 0 | 0.097621 | 0 | 0.032895 |
| Slu7 | 23.60523 | 22.05944 | 19.10462 | 20.85905 | 22.80747 | 21.98205 | 20.14305 | 19.97146 |
| Cul1 | 22.48066 | 20.66187 | 19.78378 | 22.40544 | 22.59491 | 21.64122 | 22.78085 | 22.67512 |
| Gpr173 | 1.061728 | 0.729358 | 0.732749 | 1.222925 | 1.312035 | 1.063913 | 0.848438 | 0.907453 |
| Xpot | 9.995774 | 10.26317 | 8.889196 | 9.63688 | 9.66293 | 8.855871 | 9.53044 | 9.917632 |
| Ispd | 8.370669 | 8.523916 | 8.061372 | 7.055348 | 7.373971 | 6.519123 | 6.390053 | 5.804542 |
| AC105662. | 0 | 1.161756 | 0.551158 | 1.89228 | 1.110245 | 0.450142 | 1.148719 | 0.910089 |
| Car7 | 0 | 0.038624 | 0 | 0 | 0 | 0.059862 | 0 | 0 |
| Rnf128 | 8.885455 | 7.938399 | 8.3402 | 6.46507 | 7.297663 | 6.534885 | 7.930794 | 8.133838 |
| Negr1 | 0.401271 | 0.317814 | 0.172316 | 0.628585 | 0.564056 | 0.668487 | 0.53871 | 0.675767 |
| Ufl1 | 10.9752 | 10.24381 | 10.50025 | 10.86294 | 11.56784 | 10.83734 | 11.31023 | 11.47704 |
| Pappa | 0.228445 | 0.223229 | 0.367876 | 0.191367 | 0.348066 | 0.282243 | 0.371745 | 0.230094 |
| Klf5 | 0.708265 | 1.111576 | 0.98112 | 0.863167 | 0.790541 | 0.600975 | 1.02242 | 0.769525 |
| Bloc1s2 | 15.23261 | 12.38981 | 12.14995 | 14.65633 | 16.6031 | 13.94599 | 17.04157 | 16.86322 |
| AC097845. | 0 | 0 | 0 | 0 | 0 | 0.347837 | 0 | 0 |
| Npc2 | 53.0747 | 54.6266 | 55.9496 | 82.44525 | 85.79032 | 83.01895 | 85.54481 | 83.14703 |
| Irf2bp2 | 31.76744 | 32.83587 | 37.67534 | 32.11252 | 27.42034 | 29.45912 | 44.38232 | 43.24989 |
| Tas2r126 | 0 | 0 | 0 | 0 | 0 | 0.04953 | 0 | 0 |
| AC123144. | 0.689025 | 0.7796 | 2.219138 | 0 | 2.235099 | 0 | 0.770851 | 1.221435 |

| | | | | | | | | |
|---|---|---|---|---|---|---|---|---|
| LOC500845 | 0 | 0 | 0 | 0 | 0 | 0 | 0 | 0.085478 |
| Rem2 | 0 | 0.032735 | 0 | 0.026659 | 0.062566 | 0.025367 | 0.097102 | 0.025643 |
| Mark2 | 13.72419 | 14.35596 | 15.24437 | 13.66364 | 12.19505 | 12.98652 | 17.04523 | 15.86795 |
| Lhfpl1 | 0.073894 | 0.139345 | 0.158659 | 0.022697 | 0.213067 | 0.12958 | 0.192894 | 0.327478 |
| Pcbd1 | 5.354965 | 5.740002 | 5.688382 | 5.038271 | 5.546446 | 6.425063 | 4.540469 | 4.996181 |
| AABR07015 | 0 | 0 | 0.6537 | 0 | 0 | 0 | 0 | 0 |
| Cdh5 | 78.02331 | 76.42258 | 77.0777 | 56.64754 | 54.86597 | 56.91832 | 69.8811 | 70.58169 |
| Thra | 26.68625 | 28.23426 | 31.13737 | 22.90784 | 23.1603 | 22.96751 | 24.12002 | 23.57036 |
| LOC100363 | 0 | 0 | 0 | 0 | 0 | 0.064126 | 0 | 0 |
| Hyou1 | 16.29311 | 15.89345 | 16.14803 | 15.69308 | 15.28542 | 15.94171 | 24.97912 | 23.44498 |
| Tmem98 | 25.5883 | 25.68977 | 24.41408 | 25.3055 | 26.03155 | 26.41742 | 23.86932 | 23.60656 |
| Abo | 2.800822 | 2.06519 | 1.993312 | 2.000881 | 2.177789 | 2.842063 | 2.112428 | 2.426721 |
| AABR07042 | 0 | 0 | 0 | 0 | 0 | 0 | 0.107495 | 0 |
| Tssk6 | 0.241318 | 0.13652 | 0.25907 | 0.296486 | 0.260933 | 0.246852 | 0.449959 | 0.285189 |
| Psmf1 | 14.11307 | 15.36893 | 18.44151 | 15.27086 | 16.61036 | 18.18001 | 17.01664 | 18.27739 |
| LOC685619 | 4.262342 | 4.057141 | 6.028563 | 4.551006 | 5.340366 | 4.982967 | 6.73648 | 5.516971 |
| Polr1a | 5.703305 | 6.226427 | 5.599381 | 5.359676 | 5.281881 | 4.985394 | 6.536467 | 6.699003 |
| Sat1 | 34.85262 | 33.00392 | 32.80949 | 46.65004 | 48.59629 | 44.99925 | 51.05884 | 50.42375 |
| RGD156131 | 0 | 0 | 0 | 0 | 0 | 0 | 0 | 0 |
| LOC689757 | 0.080316 | 0.318058 | 0.086224 | 0.777083 | 0.3908 | 0.70421 | 0.404342 | 0.498315 |
| Dntt | 0.156394 | 0.058984 | 0.195882 | 0.31224 | 0.310029 | 0.182835 | 0.116644 | 0.115516 |
| Rpl18 | 333.5017 | 346.6052 | 347.2136 | 336.6699 | 343.4299 | 336.7562 | 360.1093 | 331.7919 |
| Ccdc137 | 3.238599 | 3.179624 | 2.888171 | 4.200044 | 3.038643 | 3.876288 | 4.006608 | 4.510835 |
| Pcgf1 | 0.626887 | 0.141859 | 0.179467 | 1.039772 | 1.03936 | 1.942113 | 0.140267 | 0.703812 |
| S100a5 | 0 | 0 | 0.215809 | 0.185233 | 0 | 0.088128 | 0.112447 | 0.178175 |
| Srpx2 | 1.000934 | 0.994398 | 0.812477 | 2.407033 | 2.745334 | 2.889723 | 2.130351 | 2.077293 |
| Thap3 | 10.40325 | 10.58381 | 10.23002 | 10.71397 | 9.216519 | 10.00307 | 11.981 | 11.623 |
| Dnajb7 | 0.039822 | 0.090113 | 0 | 0 | 0 | 0.139664 | 0 | 0 |
| AABR07039 | 0.216388 | 0.244833 | 0.232306 | 0 | 0.935909 | 0 | 0 | 0.383591 |
| Tymp | 9.310487 | 10.25716 | 10.04323 | 10.48803 | 10.59715 | 11.6788 | 11.63719 | 9.851486 |
| Pcsk7 | 8.21812 | 7.99698 | 8.189004 | 8.102622 | 9.377081 | 8.293802 | 10.61635 | 9.940219 |
| Hgfac | 0.025657 | 0 | 0 | 0.047284 | 0.055485 | 0.022496 | 0 | 0.022741 |
| Lrp4 | 3.458351 | 3.572704 | 3.568328 | 3.799287 | 3.696679 | 3.823316 | 4.807537 | 4.608484 |
| Dsc1 | 0.014454 | 0 | 0 | 0 | 0 | 0.012673 | 0 | 0 |
| Mrpl35 | 29.15282 | 28.62579 | 26.09267 | 25.62061 | 27.98145 | 28.686 | 26.0057 | 23.76218 |
| Snx4 | 11.97306 | 11.83524 | 11.62414 | 12.96442 | 11.8947 | 13.492 | 11.70243 | 11.55095 |
| Reep5 | 53.46741 | 54.59133 | 52.45736 | 49.85048 | 47.75816 | 49.54015 | 55.35241 | 56.35363 |
| Lta | 0.1337 | 0 | 0.143536 | 0.041066 | 0 | 0.039076 | 0.099719 | 0.118505 |
| Gpr82 | 0.053056 | 0 | 0.113917 | 0 | 0.229473 | 0.093038 | 0.059356 | 0.094052 |
| Slco1a4 | 0 | 0 | 0 | 0.029962 | 0.052738 | 0.085528 | 0.072753 | 0.02882 |
| Pax9 | 0.205962 | 0.291296 | 0.497506 | 0.284679 | 0.389732 | 0.316029 | 0.806475 | 0.547664 |
| Zfp207 | 28.2776 | 28.71065 | 25.24999 | 33.08786 | 34.84711 | 31.77282 | 30.46403 | 33.1267 |
| AC091481. | 0.296691 | 0 | 0 | 0 | 0 | 0 | 0 | 0 |
| Nat8f2 | 0.076896 | 0.087004 | 0 | 0 | 0 | 0 | 0 | 0 |
| Tmed5 | 10.97353 | 8.942118 | 9.400214 | 9.482972 | 10.57445 | 10.86792 | 11.06811 | 10.7847 |
| Rasa3 | 11.88405 | 12.71763 | 13.04111 | 11.67893 | 13.10325 | 13.80771 | 15.62039 | 15.20341 |
| Mycbp | 10.19363 | 9.026303 | 9.625896 | 10.6182 | 11.90695 | 10.13347 | 11.78559 | 10.48557 |
| Ttf1 | 2.405502 | 2.869497 | 2.126732 | 2.768211 | 2.612804 | 2.309563 | 2.800763 | 2.585552 |
| Guca2b | 0 | 0 | 0 | 0.091736 | 0 | 0 | 0.334133 | 0.529443 |
| AABR07065 | 0 | 0 | 0 | 0 | 0 | 0 | 0 | 0 |
| Traf1 | 1.123497 | 0.868126 | 1.000218 | 1.035258 | 1.096302 | 1.129242 | 0.98101 | 1.238692 |
| Apmap | 11.78568 | 11.81961 | 10.78353 | 10.42811 | 10.82489 | 11.06762 | 12.88563 | 11.81137 |
| Gpr176 | 0.369738 | 0.588777 | 0.514549 | 1.110428 | 0.977271 | 0.792457 | 0.995817 | 1.128805 |
| Daw1 | 0 | 0 | 0.033563 | 0.057616 | 0 | 0.027412 | 0 | 0.05542 |

| | | | | | | | | |
|---|---|---|---|---|---|---|---|---|
| Ddx50 | 13.62474 | 11.97662 | 12.88094 | 16.11714 | 15.10702 | 17.20624 | 16.76404 | 16.28289 |
| AABR07029 | 0 | 0 | 0 | 0.229777 | 0 | 0 | 0 | 0 |
| Ptges3l | 10.07037 | 10.04373 | 10.0504 | 13.57549 | 15.00255 | 12.65592 | 13.81163 | 14.21527 |
| AABR07027 | 0 | 1.117917 | 0.53036 | 0.455218 | 0.534175 | 0.433156 | 0 | 0 |
| AABR07028 | 0 | 0 | 0 | 0 | 0 | 0 | 0 | 0.020154 |
| AABR07051 | 0 | 0 | 0 | 0.120033 | 0 | 0.114215 | 0.145733 | 0.692754 |
| RGD156431 | 0 | 0.042412 | 0 | 0.069081 | 0 | 0 | 0 | 0.033224 |
| Aldh7a1 | 30.74493 | 30.28173 | 30.30557 | 29.4767 | 31.56989 | 28.84807 | 29.91097 | 28.94168 |
| Ly49s6 | 0.800035 | 0.658329 | 0.312323 | 0.603164 | 0.707781 | 0.765241 | 0.569573 | 0.547949 |
| Jade2 | 6.970085 | 6.599177 | 7.889928 | 6.023322 | 5.779401 | 6.507191 | 7.999837 | 8.402628 |
| Lrriq4 | 0.029652 | 0.0671 | 0.095501 | 0.027323 | 0.032063 | 0.025999 | 0 | 0.026282 |
| Kif18b | 0.222496 | 0.377615 | 0.255925 | 0.424686 | 0.240581 | 0.33443 | 0.320038 | 0.366245 |
| Ncaph2 | 16.69638 | 16.4813 | 18.7131 | 17.27999 | 15.32699 | 17.77335 | 18.81611 | 19.74624 |
| Tctn3 | 0.029337 | 0 | 0 | 0 | 0 | 0 | 0 | 0 |
| Tbc1d8b | 1.065879 | 1.320354 | 0.956863 | 1.591786 | 1.222069 | 1.490468 | 1.428894 | 1.408969 |
| Bcap29 | 24.99283 | 22.23585 | 21.262 | 23.1705 | 23.62571 | 21.64616 | 26.28799 | 26.50713 |
| Dgkz | 32.24158 | 35.65309 | 38.48656 | 32.15005 | 30.56628 | 32.87424 | 36.68354 | 35.95776 |
| Ppp6r3 | 23.25312 | 23.44483 | 22.14569 | 20.9382 | 21.78892 | 22.16682 | 21.1943 | 22.80774 |
| Prss41 | 0.046178 | 0 | 0.1983 | 0.042551 | 0 | 0.080978 | 0 | 0.04093 |
| Kif1c | 147.2992 | 155.3868 | 157.9423 | 129.3014 | 123.1051 | 128.7472 | 172.7695 | 169.6864 |
| Tmem38b | 6.799754 | 7.384089 | 6.964339 | 7.472034 | 7.267964 | 7.555331 | 7.126344 | 6.858265 |
| AABR07044 | 0 | 0.529014 | 0 | 0 | 0.252779 | 0.204975 | 0 | 0.414415 |
| Adgre1 | 2.643934 | 2.574492 | 2.49438 | 3.809458 | 3.690513 | 3.793424 | 4.033522 | 4.800522 |
| St13 | 49.59324 | 47.59217 | 49.25009 | 51.94035 | 56.69065 | 54.34043 | 59.99465 | 59.90321 |
| Cebpd | 21.28659 | 21.4318 | 26.01139 | 28.34183 | 28.53496 | 30.1528 | 45.31439 | 46.98502 |
| Arvcf | 6.434323 | 7.01225 | 7.580481 | 6.981305 | 7.062755 | 7.04592 | 7.354245 | 8.344738 |
| Krt77 | 0.578757 | 0.576256 | 1.043839 | 0.469305 | 0.225289 | 0.44656 | 0.414392 | 0.410385 |
| Kcnab1 | 0.510155 | 0.475355 | 0.644334 | 0.801915 | 0.892332 | 0.999857 | 0.9904 | 0.718162 |
| AABR07069 | 0.073035 | 0 | 0 | 0 | 0 | 0.064037 | 0 | 0.064734 |
| Upf2 | 14.03873 | 12.77878 | 11.91637 | 14.45804 | 14.99604 | 14.80082 | 13.6136 | 14.42381 |
| Mir3542 | 0 | 0.506407 | 0 | 0 | 0 | 0 | 0 | 0 |
| LOC100913 | 0 | 0 | 0 | 0 | 0 | 0 | 0 | 0.028991 |
| Akap8 | 11.51392 | 11.14948 | 11.6867 | 12.26308 | 12.09413 | 11.7474 | 13.56715 | 13.77061 |
| Xrcc1 | 14.01129 | 14.17086 | 14.6362 | 13.95579 | 14.38724 | 15.04705 | 14.914 | 15.43428 |
| Slc25a10 | 5.794142 | 7.359812 | 7.159307 | 8.109347 | 8.452003 | 8.267483 | 7.858173 | 8.575542 |
| Sptbn2 | 0.044823 | 0.036225 | 0.034372 | 0.059004 | 0.062314 | 0.039301 | 0.093128 | 0.028378 |
| Xrcc5 | 5.120314 | 4.893226 | 5.19038 | 5.188105 | 5.293885 | 4.954543 | 5.157824 | 4.773445 |
| Wfdc1 | 28.91165 | 31.34524 | 30.2782 | 20.53733 | 22.61297 | 22.24497 | 25.91334 | 28.8383 |
| Rfc1 | 7.831112 | 7.380977 | 7.481929 | 7.585765 | 8.740819 | 8.143176 | 7.846754 | 8.060637 |
| Akr1b1 | 152.4404 | 145.157 | 150.2092 | 146.1298 | 149.1737 | 147.9352 | 132.2752 | 129.5305 |
| Prkcd | 6.437889 | 6.057625 | 5.605191 | 9.479387 | 9.783949 | 10.59117 | 12.2268 | 11.9811 |
| Hid1 | 5.389056 | 5.277636 | 5.558613 | 5.605654 | 6.120912 | 6.088401 | 6.60323 | 5.512477 |
| Gorasp2 | 3.193639 | 2.896278 | 2.983645 | 3.14499 | 3.347793 | 4.403345 | 4.03656 | 2.722639 |
| Stk16 | 14.08219 | 14.29196 | 15.64992 | 13.26961 | 13.42872 | 13.02979 | 14.44825 | 12.32494 |
| Sqle | 1.306267 | 0.934604 | 1.05177 | 1.48689 | 1.744788 | 1.802219 | 1.332447 | 1.276995 |
| Ppp1r14c | 31.58969 | 30.71566 | 31.84343 | 24.33561 | 24.01857 | 23.9352 | 24.36445 | 24.21263 |
| AABR07025 | 0.033038 | 0.056072 | 0.248282 | 0.091331 | 0.142896 | 0.014484 | 0.258734 | 0.043925 |
| LOC100360 | 0.760799 | 0.568458 | 0.585606 | 2.037002 | 0.946813 | 0.704828 | 0.722673 | 2.481039 |
| AC109096. | 90.92997 | 85.88892 | 100.016 | 87.34192 | 83.3975 | 93.6086 | 76.75045 | 84.19378 |
| Rcc1 | 2.933361 | 2.827262 | 2.42018 | 3.253583 | 3.142432 | 3.119708 | 3.433645 | 3.948123 |
| Lect1 | 0 | 0 | 0 | 0.034393 | 0.080716 | 0.098178 | 0 | 0 |
| Slc35b3 | 3.262589 | 2.698803 | 3.119961 | 3.966357 | 3.794598 | 2.932758 | 3.159278 | 3.863826 |
| Adpgk | 4.852833 | 5.306961 | 4.948245 | 4.602664 | 4.45691 | 4.806867 | 5.042961 | 5.70508 |
| Olr319 | 0 | 0 | 0.06045 | 0 | 0 | 0 | 0 | 0 |

| | | | | | | | | |
|---|---|---|---|---|---|---|---|---|
| Xpo5 | 3.804952 | 4.07664 | 3.902296 | 3.74116 | 4.010809 | 4.137615 | 5.017806 | 4.399346 |
| Dgat2 | 12.66879 | 14.0647 | 13.16615 | 7.658183 | 7.905007 | 8.017809 | 9.138036 | 8.590591 |
| RGD156159 | 58.23035 | 51.52137 | 51.18149 | 58.55222 | 60.80019 | 58.31563 | 51.79224 | 54.79237 |
| AABR07046 | 0.858458 | 0 | 1.382414 | 0 | 0.464119 | 0 | 0 | 0 |
| Anxa4 | 27.29819 | 26.96929 | 25.58945 | 34.05021 | 40.10011 | 37.33288 | 37.46737 | 40.48361 |
| Nubp2 | 13.18566 | 14.96158 | 15.69255 | 18.67639 | 17.76073 | 20.08346 | 20.27282 | 19.63434 |
| Cblc | 2.523659 | 2.926786 | 3.217304 | 3.226565 | 3.274555 | 2.821252 | 3.352737 | 3.159542 |
| Mgst3 | 264.4479 | 358.8549 | 289.7109 | 295.2288 | 282.1689 | 324.7685 | 225.9415 | 235.7084 |
| AABR07069 | 0.145461 | 0.987493 | 0.312323 | 0.134036 | 0.629139 | 0.12754 | 0.162735 | 0.128929 |
| Tnfrsf11a | 1.317382 | 1.697577 | 1.316079 | 1.416235 | 1.938856 | 1.844922 | 1.903695 | 2.124494 |
| Tsen34l1 | 3.290297 | 3.975211 | 3.532345 | 3.648533 | 4.100459 | 4.400749 | 1.809324 | 8.749062 |
| Pax4 | 0 | 0 | 0 | 0 | 0 | 0 | 0 | 0 |
| Rbm24 | 37.63049 | 35.93083 | 33.25309 | 31.70076 | 32.64682 | 32.85382 | 40.28257 | 39.57317 |
| Mapre2 | 37.87351 | 38.28813 | 38.22213 | 37.3027 | 38.23587 | 39.8575 | 40.93901 | 41.91865 |
| Akap17a | 20.80086 | 20.83339 | 21.54996 | 21.90043 | 19.90966 | 20.69178 | 24.73204 | 24.62103 |
| Arfip1 | 8.420651 | 7.852802 | 7.592277 | 9.638503 | 9.211828 | 7.729323 | 10.15664 | 9.067158 |
| Mpp5 | 5.705519 | 5.995591 | 5.470633 | 6.11358 | 6.703025 | 6.351907 | 5.912065 | 6.356743 |
| Gpr165 | 0.082181 | 0.092984 | 0.158808 | 0.045436 | 0.071089 | 0.086468 | 0.073553 | 0.116546 |
| Klb | 0 | 0 | 0 | 0 | 0 | 0 | 0 | 0.042857 |
| Rpgrip1l | 0.754318 | 0.711229 | 0.492236 | 0.729146 | 0.735669 | 0.648418 | 0.711521 | 0.80624 |
| RT1-DMa | 0.33185 | 0.300378 | 0.427515 | 0.550416 | 0.789414 | 0.232773 | 0.222755 | 2.76487 |
| LOC103694 | 0 | 0 | 0.044724 | 0 | 0 | 0 | 0 | 0.036925 |
| LOC100912 | 0.475621 | 2.260202 | 1.072281 | 1.314799 | 0.925709 | 0.66724 | 2.075206 | 2.149991 |
| Rn50_20_( | 3.466081 | 5.08762 | 4.425043 | 4.661305 | 3.545238 | 4.024704 | 8.069802 | 6.144319 |
| RGD156442 | 5.824319 | 5.646383 | 5.78382 | 6.049938 | 6.154621 | 6.870924 | 5.834772 | 6.617237 |
| LOC103690 | 0 | 0.066275 | 0 | 0 | 0 | 0 | 0 | 0 |
| Cdpf1 | 13.68067 | 13.65797 | 15.79786 | 13.13587 | 15.3521 | 15.82563 | 12.86162 | 11.10688 |
| LOC100363 | 6.982123 | 7.845834 | 7.649777 | 6.874419 | 6.050077 | 5.031724 | 7.169265 | 6.442929 |
| Pms1 | 3.839815 | 3.607189 | 3.725186 | 3.60316 | 3.732934 | 3.104208 | 3.724353 | 3.762495 |
| Gla | 3.399846 | 3.940589 | 3.738975 | 3.973338 | 4.438344 | 4.217007 | 4.499377 | 3.638193 |
| Rn50_2_20 | 0.512763 | 0.725209 | 0.688105 | 0.649675 | 1.108886 | 0.674387 | 0.358535 | 0.568109 |
| Ankrd13c | 6.819116 | 6.02486 | 6.854095 | 6.233479 | 6.154302 | 6.645988 | 6.625918 | 6.634085 |
| AABR07007 | 0 | 0 | 0.525403 | 0 | 0.529182 | 0 | 1.09504 | 0.433781 |
| AABR07027 | 0.831205 | 0 | 0 | 0.765923 | 1.79754 | 0.728801 | 0.929915 | 0.736738 |
| Cdh7 | 0.008189 | 0.009265 | 0.017582 | 0.022636 | 0.017708 | 0.028719 | 0.018322 | 0.014516 |
| N4bp2l1 | 0.910926 | 1.476365 | 1.189383 | 1.111614 | 1.091454 | 0.971392 | 1.184363 | 1.636619 |
| AABR07064 | 2.566957 | 1.161756 | 1.653475 | 2.36535 | 0.555123 | 2.25071 | 4.594876 | 2.730266 |
| AABR07015 | 8.160923 | 8.848963 | 7.30106 | 8.773297 | 7.353573 | 6.45983 | 7.481592 | 8.639932 |
| RGD130718 | 0.352237 | 0.177129 | 0.252099 | 0.3967 | 0.169275 | 0.24021 | 0.437853 | 0.450963 |
| Crot | 8.865361 | 9.070617 | 9.1819 | 8.642353 | 9.320379 | 9.065419 | 9.018791 | 9.045388 |
| Rplp2 | 21.81913 | 23.83604 | 27.94753 | 180.5333 | 14.96917 | 29.55415 | 15.15121 | 43.74702 |
| Dmxl2 | 0.918962 | 0.803708 | 0.762587 | 0.874251 | 0.939949 | 0.888499 | 1.172582 | 0.893772 |
| Stk36 | 1.207403 | 1.189467 | 1.150958 | 1.333171 | 1.463114 | 1.295937 | 1.467237 | 1.494564 |
| AABR07063 | 7.320894 | 7.63358 | 7.181377 | 8.518371 | 7.636589 | 8.634138 | 8.639953 | 9.618798 |
| Selt | 47.92357 | 51.71011 | 50.58189 | 47.75696 | 49.41733 | 48.33414 | 55.27139 | 53.69289 |
| Olr459 | 0 | 0 | 0 | 0 | 0 | 0 | 0 | 0 |
| Rbfox3 | 0.163007 | 0.230543 | 0.349996 | 0.450613 | 0.132193 | 0.357311 | 0.319138 | 0.433443 |
| Asgr1 | 0.043858 | 0 | 0 | 0 | 0.047423 | 0 | 0 | 0 |
| AABR07019 | 0 | 0 | 0 | 0.096122 | 0 | 0 | 0 | 0 |
| AABR07033 | 0.256696 | 0 | 0 | 0 | 0 | 0 | 0.28718 | 0.455044 |
| Kmt2e | 26.65191 | 26.79496 | 26.70363 | 24.21659 | 26.05058 | 24.72187 | 31.60949 | 33.58125 |
| Slc14a2 | 0.071392 | 0.201941 | 0.153287 | 0.328924 | 0.192987 | 0.34428 | 0.31948 | 0.253113 |
| Nfe2 | 0.760764 | 0.717307 | 0.646577 | 0.671805 | 0.616952 | 0.528072 | 0.425554 | 0.477631 |
| Spib | 0.059417 | 0.067227 | 0.212625 | 0.1095 | 0.128493 | 0.225752 | 0.221576 | 0.210656 |

| | | | | | | | | |
|---|---|---|---|---|---|---|---|---|
| Hspb9 | 0.405309 | 0 | 0 | 0.074695 | 0.175302 | 0.2843 | 0.453442 | 0.143698 |
| AABR0703( | 0.279658 | 0.870154 | 0.750576 | 0.966351 | 1.285157 | 0.674312 | 0.156434 | 0.681655 |
| Bend3 | 0.885318 | 0.687732 | 1.120675 | 0.961897 | 0.728677 | 0.648804 | 0.975672 | 0.948669 |
| Dmrt2 | 0 | 0 | 0 | 0 | 0 | 0 | 0 | 0.059736 |
| LOC10091 | 0.163134 | 0.092289 | 0 | 0.150321 | 0.264591 | 0.071518 | 0 | 0 |
| Wbp11 | 0.121442 | 0.247331 | 0.365053 | 0.26857 | 0.210102 | 0.106481 | 0.244556 | 0.215281 |
| AC093937. | 0 | 0.630315 | 0.598066 | 1.026662 | 0.602367 | 0 | 0.623241 | 0 |
| AC132020. | 1.496169 | 1.692845 | 1.070822 | 0.919107 | 0.89877 | 2.186404 | 0.929915 | 1.326129 |
| AABR0707 | 0 | 0.397648 | 0.377303 | 0.323847 | 0 | 0 | 0 | 0 |
| Zzef1 | 3.79798 | 3.863687 | 3.901938 | 3.224491 | 3.466933 | 3.438769 | 4.475962 | 4.575024 |
| LOC10091 | 2.876782 | 2.702444 | 2.689537 | 2.318263 | 2.536707 | 2.615441 | 2.66024 | 6.153476 |
| Mdga2 | 0 | 0.019306 | 0 | 0 | 0 | 0.014961 | 0 | 0 |
| Nav2 | 3.50388 | 3.644175 | 3.664158 | 2.633153 | 2.593177 | 2.646031 | 2.999745 | 3.261893 |
| Fes | 5.144919 | 5.482544 | 5.76442 | 5.982079 | 6.615063 | 6.938845 | 7.283838 | 8.158608 |
| AC109025. | 0 | 0 | 0 | 0 | 0 | 0 | 0 | 0 |
| Kdelr2 | 12.39707 | 13.76205 | 12.17903 | 15.06058 | 15.11198 | 14.61265 | 16.22445 | 16.35263 |
| Trib3 | 0.838937 | 0.459299 | 0.61012 | 1.072292 | 0.64377 | 0.90168 | 0.81746 | 1.43921 |
| Crll | 15.70442 | 16.22238 | 15.88149 | 15.33531 | 18.25598 | 16.54237 | 16.75989 | 18.31402 |
| Efhb | 0 | 0 | 0.018493 | 0 | 0.018626 | 0 | 0.038543 | 0.030536 |
| Fgf1 | 77.41384 | 74.42112 | 73.97996 | 56.51396 | 53.35663 | 55.54607 | 70.74497 | 73.96994 |
| LOC10036( | 0 | 0 | 0.044689 | 0 | 0 | 0 | 0.04657 | 0 |
| Lin52 | 14.60546 | 16.32386 | 18.93061 | 19.3669 | 22.72604 | 17.17889 | 17.53555 | 16.89236 |
| AABR07002 | 1.528809 | 1.558699 | 1.352699 | 1.315854 | 1.39876 | 1.414113 | 1.221689 | 1.697547 |
| Zfp612 | 7.333295 | 6.339821 | 6.348106 | 6.019745 | 7.147615 | 6.429839 | 7.71307 | 7.438224 |
| Rn50_X_11 | 0 | 0.073923 | 0 | 0 | 0 | 0 | 0 | 0 |
| AABR0707( | 1.848209 | 0 | 4.629731 | 2.83842 | 3.330736 | 3.241023 | 6.203082 | 2.184213 |
| AC112891. | 0 | 0.452287 | 2.574878 | 0.736689 | 0.432233 | 1.401969 | 2.236056 | 1.062928 |
| AABR07013 | 0 | 0.553734 | 0 | 1.352892 | 0.529182 | 0 | 0.54752 | 0.867561 |
| AABR07001 | 0 | 0.75961 | 0 | 0 | 0 | 0 | 0 | 0 |
| Thoc1 | 0 | 0 | 0 | 0 | 0 | 0.044491 | 0 | 0 |
| RGD15602( | 0.098618 | 0 | 0.105872 | 0 | 0.106634 | 0.172936 | 0 | 0.08741 |
| Pcdhga1 | 0.627266 | 0.734195 | 0.510863 | 0.637794 | 0.561314 | 0.531023 | 0.919545 | 0.651835 |
| Tp53tg5 | 0.153566 | 0.289587 | 0.494588 | 0.330178 | 0.166048 | 0.089764 | 0.22907 | 0.226855 |
| Alg1 | 2.566392 | 2.903751 | 2.940928 | 3.613671 | 2.868541 | 3.691363 | 3.774453 | 4.523882 |
| Atf1 | 15.0214 | 14.54814 | 13.46069 | 15.01964 | 16.34875 | 14.74437 | 17.08032 | 15.60225 |
| Pdf | 32.42775 | 32.77455 | 33.19779 | 30.08888 | 29.36886 | 32.32485 | 33.08013 | 34.14401 |
| Neil3 | 0.079543 | 0.03 | 0 | 0.024432 | 0.02867 | 0 | 0 | 0.141006 |
| Trappc2l | 30.89149 | 30.17247 | 33.44744 | 32.52018 | 28.73949 | 34.33941 | 26.6831 | 27.92672 |
| AABR07063 | 0 | 0 | 0 | 0 | 0 | 0 | 0 | 0 |
| Gypc | 23.1251 | 23.89445 | 21.67169 | 20.0321 | 21.67258 | 20.71598 | 19.88458 | 20.94159 |
| Nhsl1 | 4.703017 | 4.904519 | 5.383561 | 5.282174 | 5.085304 | 5.382221 | 5.863749 | 5.951438 |
| AABR07027 | 0.393359 | 0.278167 | 0.158361 | 0.135924 | 0.212667 | 0.172449 | 0.165027 | 0.130745 |
| Mbnl1 | 40.95763 | 42.49041 | 39.99521 | 43.22102 | 49.58514 | 41.48702 | 47.99758 | 46.11614 |
| #N/A | 0.298632 | 0.238509 | 0.264024 | 0.437046 | 0.3419 | 0.354255 | 0.412706 | 0.202412 |
| Dmrtc2 | 0 | 0.068735 | 0 | 0.027989 | 0.065687 | 0 | 0.101945 | 0.026923 |
| Papss1 | 6.313441 | 5.386325 | 5.903317 | 7.43619 | 7.377167 | 7.522208 | 7.575849 | 7.559 |
| Hdac2 | 27.98619 | 28.10138 | 28.00583 | 25.56663 | 24.85359 | 24.70697 | 26.38731 | 28.53491 |
| Prdm4 | 9.970752 | 10.59838 | 10.47427 | 9.295323 | 9.349348 | 10.24496 | 11.13305 | 10.52914 |
| Nuggc | 0 | 0 | 0 | 0 | 0 | 0 | 0 | 0 |
| Ttc5 | 10.01852 | 9.301675 | 9.22307 | 10.37649 | 10.43272 | 10.9861 | 9.729609 | 9.442227 |
| Rn60_12_( | 0 | 0 | 0 | 0.159251 | 0 | 0 | 0 | 0 |
| Hba-a3 | 17.45531 | 19.74986 | 18.73939 | 17.77155 | 15.57449 | 15.1978 | 14.20234 | 15.36331 |
| Nalcn | 0.170345 | 0.192737 | 0.161361 | 0.424731 | 0.411721 | 0.465646 | 0.257835 | 0.275325 |
| Fank1 | 0.068722 | 0.155511 | 0 | 0.126649 | 0.111462 | 0.030128 | 0.076883 | 0.030456 |

| | | | | | | | | |
|---|---|---|---|---|---|---|---|---|
| LOC10091( | 0.176912 | 0.200167 | 0.443161 | 1.2498 | 0.892697 | 0.413644 | 0.659737 | 1.829401 |
| Itpr1 | 5.596095 | 5.903549 | 5.656902 | 5.547552 | 5.592194 | 5.821633 | 6.870051 | 7.048827 |
| Stfa2l1 | 0 | 0 | 0 | 0 | 0 | 0 | 0 | 0 |
| Ttc33 | 9.654052 | 8.639184 | 7.726071 | 7.601891 | 8.097968 | 7.643864 | 7.953114 | 8.245708 |
| Klk15 | 0.068452 | 0 | 0.293951 | 0.378456 | 0.222049 | 0.240076 | 0.382906 | 0.424708 |
| Ccdc106 | 3.59374 | 3.949972 | 3.913225 | 3.264183 | 4.107908 | 3.376065 | 4.882055 | 4.959983 |
| Tmem50a | 32.92438 | 33.36717 | 32.25632 | 38.90031 | 39.47741 | 39.00739 | 38.1902 | 37.68662 |
| AABR07019 | 0 | 0 | 0 | 0 | 0 | 0.150047 | 0 | 0.303363 |
| Gtf2ird1 | 1.178758 | 0.62118 | 0.468051 | 0.610046 | 0.646017 | 0.821166 | 0.433559 | 1.001855 |
| Agpat3 | 6.673318 | 6.454976 | 7.164233 | 4.838978 | 4.782211 | 4.374993 | 6.245908 | 5.767988 |
| Ahi1 | 3.501064 | 3.074715 | 2.631031 | 2.626962 | 2.307444 | 2.455789 | 3.002907 | 2.970175 |
| Rn60_1_22 | 0 | 0.125795 | 0 | 0.204897 | 0 | 0.194966 | 0.124384 | 0.098545 |
| LOC10091( | 0.374454 | 0.162953 | 1.515231 | 0.291961 | 0.373746 | 0.353577 | 0.418922 | 0.893569 |
| Rn50_10_( | 0.131904 | 0.149243 | 0.141607 | 0 | 0 | 0 | 0 | 0 |
| RT1-M10-4 | 0 | 0 | 0 | 0 | 0.056229 | 0 | 0 | 0 |
| Klf16 | 4.586059 | 4.937465 | 6.007882 | 3.983862 | 3.888429 | 4.056488 | 4.678637 | 5.192982 |
| Eif2s3y | 30.5846 | 30.15533 | 28.22275 | 28.15463 | 28.94912 | 28.38157 | 30.39228 | 30.48718 |
| Rptor | 6.839854 | 7.59746 | 7.930462 | 5.740819 | 6.127978 | 6.250785 | 7.21486 | 7.295789 |
| Emp3 | 22.26809 | 21.61919 | 20.51307 | 38.52308 | 35.72888 | 35.33337 | 39.05644 | 34.50846 |
| Rps2 | 0.046097 | 0 | 0 | 0.042476 | 0 | 0.040418 | 0 | 0 |
| Gap43 | 0.813245 | 0.360058 | 0.569394 | 1.661654 | 2.141027 | 1.488113 | 1.26584 | 1.096899 |
| AABR0700( | 0.471765 | 0 | 0 | 0.144904 | 0 | 0.137881 | 0 | 0 |
| AABR07031 | 35.71245 | 48.5023 | 81.38059 | 52.34527 | 26.81066 | 27.36482 | 33.26008 | 37.61271 |
| Nyx | 0.159409 | 0.090182 | 0.085568 | 0.244816 | 0.201095 | 0.116475 | 0.208063 | 0.117744 |
| Ryr1 | 0.039155 | 0.079744 | 0.075664 | 0.057728 | 0.063507 | 0.044631 | 0.048185 | 0.062469 |
| Stx4 | 65.91828 | 67.93125 | 67.39761 | 61.5216 | 60.33988 | 68.10721 | 64.60478 | 69.20224 |
| Anapc16 | 16.58852 | 17.0444 | 18.00136 | 19.62349 | 20.2154 | 19.34069 | 17.0036 | 19.43211 |
| Dbil5 | 0.649932 | 0.315157 | 0.697743 | 0.598886 | 0.903551 | 0.569861 | 0.415494 | 0.576067 |
| AABR07071 | 0 | 0.142085 | 0.404447 | 0 | 0 | 0 | 0.140491 | 0 |
| LOC100361 | 0 | 0 | 0 | 0 | 0 | 0 | 0 | 0.217398 |
| LOC102547 | 0 | 0.017457 | 0 | 0.056869 | 0 | 0 | 0 | 0 |
| Rn50_10_( | 1.200365 | 1.188387 | 0.966501 | 0.967828 | 1.135695 | 1.973402 | 1.342915 | 1.196936 |
| Nrde2 | 2.62563 | 2.439688 | 2.692803 | 1.946345 | 2.20463 | 2.507934 | 2.707695 | 2.795272 |
| G6pc3 | 17.79943 | 17.80307 | 19.45278 | 18.76329 | 19.5927 | 19.13364 | 21.42661 | 19.27891 |
| Bhlhe23 | 0 | 0 | 0 | 0 | 0.025563 | 0 | 0 | 0 |
| Actl7a | 0.06616 | 0.112286 | 0 | 0 | 0.214615 | 0.029005 | 0.185043 | 0.058641 |
| Rims2 | 0.010505 | 0.023771 | 0 | 0.087117 | 0.068151 | 0.018421 | 0.058761 | 0.055865 |
| Ttc1 | 35.87157 | 33.32014 | 32.04631 | 34.63465 | 32.15843 | 30.71631 | 33.84438 | 33.80012 |
| LOC10036( | 0.739632 | 1.338973 | 0.794042 | 1.090466 | 0.799753 | 1.167317 | 0.827467 | 1.311145 |
| AABR07017 | 0 | 0 | 0 | 0.141091 | 0.165563 | 0 | 0.3426 | 0 |
| Umodl1 | 0.013164 | 0 | 0 | 0 | 0.014234 | 0 | 0 | 0 |
| Lrrc69 | 0 | 0.052526 | 0 | 0 | 0.050197 | 0.122113 | 0 | 0.041148 |
| Ucp3 | 1.429682 | 1.514366 | 1.534855 | 1.191262 | 1.282764 | 0.973499 | 6.619063 | 6.29555 |
| Gpx1 | 550.532 | 553.4711 | 584.978 | 741.3185 | 748.6106 | 793.9051 | 712.1426 | 700.7369 |
| AC131411. | 0.058184 | 0 | 0 | 0.053615 | 0 | 0 | 0 | 0 |
| Galk1 | 5.062566 | 5.728056 | 5.265145 | 6.742319 | 6.799833 | 5.9994 | 6.150505 | 6.450356 |
| Neurl3 | 2.383764 | 2.392604 | 3.013161 | 3.188533 | 2.785395 | 3.000286 | 3.570137 | 3.03296 |
| Rps18 | 321.6044 | 313.4205 | 313.8333 | 301.317 | 300.7682 | 296.8674 | 328.0098 | 321.5863 |
| LOC680406 | 0 | 0 | 0 | 0 | 0 | 0 | 0 | 0 |
| Tceal5 | 1.12895 | 0.786064 | 0.466154 | 4.561242 | 5.634081 | 5.025466 | 4.469145 | 4.002579 |
| Wwp2 | 4.750983 | 5.232801 | 5.100484 | 6.392427 | 6.296442 | 6.266931 | 6.608708 | 7.285458 |
| Apool | 27.00002 | 26.22109 | 23.92262 | 24.46879 | 22.12695 | 22.59904 | 19.77752 | 23.37185 |
| LOC102555 | 0 | 0.188094 | 0 | 0.306369 | 0 | 0 | 0 | 0 |
| Wdr92 | 4.145529 | 3.194125 | 4.040936 | 4.007424 | 3.025001 | 3.590205 | 3.243639 | 3.606762 |

| | | | | | | | | |
|---|---|---|---|---|---|---|---|---|
| Aspa | 0.489401 | 0.383355 | 0.606235 | 0.693791 | 1.180484 | 1.353339 | 1.389859 | 1.167871 |
| RGD156344 | 0.182778 | 0.310207 | 0.196224 | 0.505268 | 0.098818 | 0.40065 | 0 | 0.648021 |
| AC096352. | 0 | 0 | 0 | 0 | 0 | 0 | 0 | 0 |
| Tspan17 | 1.62315 | 1.399252 | 1.244683 | 3.311839 | 2.674421 | 2.812474 | 3.06975 | 3.836477 |
| Lgr4 | 6.854413 | 7.298247 | 6.73204 | 6.647045 | 6.456811 | 6.0362 | 6.563358 | 7.640687 |
| Ccpg1 | 54.44022 | 55.26892 | 53.16164 | 50.33317 | 50.33357 | 48.01853 | 49.46138 | 52.50699 |
| AABR07026 | 0.32151 | 0.181887 | 0.129436 | 0.222194 | 0.043455 | 0.105713 | 0.134884 | 0.035621 |
| Rai14 | 4.062992 | 3.634181 | 4.89238 | 4.515431 | 4.660411 | 4.28455 | 5.697224 | 5.730338 |
| Naglu | 4.814767 | 4.772749 | 5.557776 | 6.281938 | 5.943291 | 6.407107 | 6.292251 | 6.250287 |
| LOC100911 | 0 | 0 | 0 | 0.049643 | 0 | 0 | 0 | 0 |
| Nt5dc2 | 1.806726 | 1.846397 | 2.31505 | 3.168542 | 2.93038 | 2.81057 | 2.445103 | 2.763691 |
| AABR07057 | 0 | 0 | 0 | 0.049038 | 0 | 0 | 0 | 0 |
| Adgrg1 | 30.91482 | 28.92878 | 32.69797 | 24.08297 | 23.51881 | 21.87853 | 32.85682 | 34.06993 |
| Map4k3 | 7.188625 | 7.14169 | 7.654705 | 6.17974 | 6.840834 | 6.866675 | 7.600968 | 7.252268 |
| Mast3 | 2.670776 | 3.057132 | 3.536646 | 3.543096 | 3.629504 | 3.954532 | 4.255207 | 4.329197 |
| Cdca8 | 1.001062 | 0.703027 | 0.889411 | 0.795207 | 0.671856 | 0.454 | 1.119944 | 0.97908 |
| AABR07024 | 0 | 0 | 0 | 0.051388 | 0 | 0 | 0 | 0 |
| AABR07064 | 0 | 0.455766 | 0 | 0 | 0 | 0 | 0 | 0 |
| AABR07052 | 0 | 0 | 0.011049 | 0.018967 | 0.011129 | 0.009024 | 0 | 0.036489 |
| Nmu | 0.221889 | 0 | 0 | 0.204462 | 0.159951 | 0.324255 | 0 | 0.065557 |
| Adamts7 | 8.817299 | 9.183032 | 9.991819 | 7.045029 | 6.947994 | 6.450935 | 6.823416 | 7.355492 |
| Nagpa | 4.288272 | 4.242015 | 5.129874 | 4.922407 | 5.007794 | 4.318583 | 5.208744 | 6.038014 |
| AABR07045 | 0 | 0 | 0 | 0 | 0.432233 | 0 | 0 | 0 |
| Pcna | 31.82584 | 28.34786 | 26.89748 | 28.03674 | 28.11601 | 27.39037 | 32.12056 | 30.12942 |
| Usp25 | 9.399911 | 9.418553 | 9.237903 | 10.01907 | 9.810018 | 9.98453 | 11.15712 | 10.76684 |
| AABR07058 | 0.209046 | 0.118263 | 0 | 0 | 0.339057 | 0 | 0 | 0 |
| Olr334 | 0 | 0.062898 | 0 | 0 | 0 | 0 | 0 | 0 |
| AABR07052 | 1.07971 | 1.221641 | 0 | 0.497455 | 0 | 0 | 0.603966 | 0.957 |
| Fat1 | 7.992971 | 7.554968 | 8.222166 | 8.386353 | 8.25848 | 8.606074 | 11.12656 | 11.8336 |
| Pogz | 5.141949 | 5.023022 | 4.965047 | 5.052778 | 4.705355 | 4.893439 | 5.174051 | 5.92397 |
| Myo1f | 2.414567 | 2.514653 | 2.842574 | 3.071918 | 3.337717 | 2.911005 | 3.944527 | 3.392625 |
| AABR07021 | 1.08643 | 1.676242 | 1.484448 | 1.146717 | 1.580561 | 1.420214 | 1.480638 | 1.313123 |
| Tmem173 | 2.797983 | 1.829766 | 2.866024 | 2.696499 | 3.525029 | 2.610824 | 3.130259 | 3.321823 |
| Ugp2 | 103.2636 | 98.23899 | 92.22375 | 90.19278 | 91.26839 | 86.45251 | 83.09425 | 83.15828 |
| AABR07005 | 0.063628 | 0 | 0.204926 | 0.175892 | 0.1032 | 0.055789 | 0.035592 | 0.028198 |
| Zfp648 | 0.032627 | 0 | 0.035027 | 0.060129 | 0 | 0.028607 | 0.036501 | 0.028919 |
| Lxn | 2.833231 | 2.874047 | 1.048846 | 4.546238 | 5.176312 | 3.597777 | 2.732494 | 4.06993 |
| AABR07011 | 0 | 0 | 0.011095 | 0 | 0 | 0 | 0.011562 | 0 |
| AABR07070 | 0 | 0 | 0 | 0 | 0.060819 | 0 | 0 | 0.099709 |
| Klhl34 | 0.89168 | 0.611451 | 0.957275 | 1.170226 | 1.051811 | 1.160893 | 1.360325 | 1.365133 |
| Klre1 | 0.772152 | 1.111922 | 1.281111 | 1.099602 | 0.379507 | 0.800118 | 0.549722 | 0.311089 |
| AABR07009 | 0 | 0.397648 | 0 | 0.323847 | 0 | 0 | 0 | 0 |
| Ccdc181 | 2.139624 | 1.520812 | 1.649145 | 2.376006 | 1.898293 | 2.357056 | 1.565122 | 2.018023 |
| Bnc1 | 0.022161 | 0 | 0 | 0 | 0 | 0 | 0 | 0 |
| Mesp2 | 0 | 0 | 0 | 0 | 0 | 0 | 0.031702 | 0 |
| Fdxacb1 | 1.313626 | 0.979611 | 1.217953 | 1.237965 | 1.097586 | 1.177966 | 1.269223 | 1.402491 |
| AC107331. | 0 | 0 | 0.010959 | 0 | 0 | 0 | 0.01142 | 0 |
| Limd1 | 13.87378 | 14.6479 | 14.27008 | 12.68208 | 13.04025 | 11.95816 | 16.71413 | 16.97284 |
| AABR07044 | 0.043876 | 0.024822 | 0.023552 | 0 | 0 | 0 | 0 | 0 |
| AABR07044 | 0.739632 | 0.167372 | 0.476425 | 0.408925 | 0.159951 | 0.259404 | 0.49648 | 0.131114 |
| Psmd14 | 20.16617 | 17.69258 | 18.24251 | 19.9227 | 21.01896 | 19.88468 | 17.53102 | 20.01333 |
| LOC103690 | 0.209799 | 0.142427 | 0.13514 | 0.038664 | 0.226853 | 0.147162 | 0.046943 | 0.260338 |
| Leng1 | 9.105883 | 10.49546 | 9.471207 | 9.305588 | 10.64356 | 10.02358 | 9.489066 | 8.925869 |
| AABR07027 | 0 | 0 | 0 | 0 | 0.524283 | 0 | 0 | 0 |

| | | | | | | | | |
|---|---|---|---|---|---|---|---|---|
| Gsk3b | 22.16317 | 24.4444 | 28.82555 | 19.85043 | 19.43357 | 19.6232 | 26.08007 | 26.66015 |
| Med13 | 3.742553 | 4.394793 | 3.737739 | 3.60662 | 3.635643 | 3.654071 | 4.237046 | 5.1278 |
| Slc16a10 | 3.329775 | 2.97278 | 3.490944 | 1.989573 | 1.406421 | 1.779101 | 2.29915 | 2.536312 |
| AC113741. | 0.366195 | 1.242998 | 0 | 0.337435 | 1.979808 | 0 | 1.229049 | 1.298308 |
| Ccdc163 | 1.911406 | 1.267769 | 1.521322 | 1.761285 | 2.530018 | 2.05156 | 2.32274 | 1.869433 |
| Bag1 | 65.09709 | 62.8734 | 64.51335 | 61.71827 | 55.92988 | 57.18249 | 57.10661 | 56.52777 |
| Gja4 | 8.408885 | 10.07392 | 9.947924 | 9.146218 | 10.26907 | 11.70993 | 9.075458 | 11.22366 |
| Timm13 | 69.40924 | 70.36347 | 72.28049 | 73.66845 | 69.42412 | 65.93434 | 66.51725 | 62.15509 |
| Blm | 0.492139 | 0.490542 | 0.31449 | 0.28073 | 0.443452 | 0.472604 | 0.406383 | 0.311577 |
| Ccdc84 | 8.282098 | 6.998448 | 6.302737 | 10.095 | 8.728596 | 10.70878 | 11.55273 | 10.54664 |
| Ptpn6 | 5.070148 | 4.461826 | 4.508449 | 6.677573 | 6.368302 | 6.511101 | 7.935429 | 7.739536 |
| Phldb2 | 11.31683 | 11.84683 | 10.93294 | 12.0507 | 11.38059 | 11.77786 | 12.96621 | 11.94242 |
| AABR07058 | 0.640562 | 0.362383 | 0.171921 | 0.590252 | 0.519473 | 0.280823 | 0.358316 | 0.425821 |
| Pla2g4e | 0 | 0 | 0 | 0 | 0 | 0 | 0 | 0 |
| AC095852. | 0.467553 | 0 | 0 | 0 | 0 | 0 | 0 | 0 |
| Cops3 | 32.10047 | 30.81712 | 28.71826 | 28.56347 | 28.50409 | 29.25449 | 27.53298 | 28.96956 |
| AABR07026 | 4.091088 | 1.542958 | 5.856058 | 2.764503 | 5.308361 | 4.782759 | 0.610257 | 5.560072 |
| Gramd1c | 0.113346 | 0.179544 | 0.170358 | 0.271554 | 0.171583 | 0.119258 | 0.202891 | 0.060279 |
| AABR07069 | 1.38553 | 1.728448 | 1.678154 | 2.029643 | 1.728638 | 2.149321 | 1.62956 | 1.983796 |
| #N/A | 1.41849 | 1.524707 | 2.030453 | 2.200256 | 2.045057 | 1.388835 | 2.565559 | 2.640284 |
| AABR07012 | 0.147302 | 0 | 0 | 0 | 0.238914 | 0.064577 | 0 | 0.065281 |
| AABR07030 | 0 | 0.251591 | 0.358077 | 0 | 0.120218 | 0.194966 | 0 | 0 |
| Gid8 | 15.99155 | 15.07117 | 14.18222 | 16.1181 | 15.31301 | 15.01606 | 17.52218 | 18.00144 |
| Fam187b | 0 | 0 | 0 | 0 | 0 | 0.03513 | 0 | 0 |
| AABR07071 | 5.004668 | 4.419549 | 4.717608 | 5.286474 | 4.75154 | 3.852964 | 6.145245 | 7.465272 |
| Caln1 | 0.066623 | 0 | 0 | 0 | 0.072039 | 0 | 0 | 0 |
| Timp4 | 33.92548 | 34.8322 | 37.04912 | 26.86403 | 24.89926 | 27.20858 | 28.86182 | 28.67822 |
| Slc10a1 | 0.188819 | 0.10682 | 0.13514 | 0.347979 | 0.204168 | 0.220743 | 0.211243 | 0.223147 |
| Spred2 | 9.534209 | 10.33803 | 9.615244 | 7.354443 | 6.755651 | 6.713014 | 8.929111 | 8.930793 |
| Usp24 | 11.18875 | 10.25753 | 10.26398 | 9.694402 | 9.422817 | 9.832 | 11.20538 | 11.8032 |
| Metrn | 6.800769 | 5.514571 | 7.179376 | 8.459965 | 7.659972 | 8.695925 | 8.369238 | 7.283664 |
| Pabpc1l | 0.057513 | 0 | 0 | 0 | 0 | 0 | 0.032172 | 0.025488 |
| AABR07013 | 8.45911 | 7.748021 | 5.189369 | 7.423559 | 9.582271 | 9.182897 | 9.914328 | 11.78215 |
| LOC100910 | 3.678128 | 4.075822 | 3.58233 | 3.633835 | 3.034081 | 3.191738 | 2.672581 | 3.797857 |
| Rn50_18_( | 1.985933 | 2.010465 | 1.79539 | 1.637332 | 1.243209 | 1.374685 | 2.455645 | 1.4823 |
| Pim2 | 0.940825 | 1.109797 | 1.203447 | 0.977606 | 1.082235 | 0.684505 | 1.074948 | 0.798415 |
| Senp1 | 0 | 0 | 0 | 0 | 0.059167 | 0 | 0 | 0.0485 |
| Asb5 | 3.320766 | 3.793418 | 3.290819 | 3.383604 | 2.62397 | 2.603687 | 2.214786 | 2.632043 |
| AABR07011 | 0.828575 | 0.937493 | 0.62267 | 1.221598 | 1.075111 | 1.162392 | 1.112367 | 1.395373 |
| AABR07040 | 7.176746 | 7.019113 | 6.007051 | 6.669133 | 6.247548 | 6.559212 | 7.348599 | 6.415015 |
| Spsb1 | 6.648455 | 7.043459 | 6.576162 | 5.965675 | 6.32729 | 6.637187 | 8.413014 | 8.386836 |
| LOC100359 | 0.307583 | 0.290013 | 0.330209 | 0.307044 | 0.221723 | 0.247215 | 0.34411 | 0.613408 |
| Selo | 8.791514 | 9.850844 | 9.18687 | 8.12065 | 8.355273 | 8.548307 | 9.811741 | 10.3961 |
| Nsmce2 | 12.01206 | 12.77686 | 12.30144 | 16.2204 | 14.96367 | 14.90037 | 13.68627 | 14.76826 |
| Rapgef4 | 11.44695 | 9.630742 | 9.619923 | 8.67061 | 8.793011 | 8.447175 | 10.9134 | 10.86525 |
| Phgdh | 1.684539 | 2.136008 | 1.527837 | 3.024184 | 2.135514 | 2.240973 | 1.592151 | 1.415862 |
| Slc38a6 | 2.236508 | 1.763685 | 1.77046 | 2.331472 | 2.418304 | 2.020396 | 1.743892 | 2.783269 |
| Vpreb2 | 0 | 0 | 0 | 0.077329 | 0 | 0 | 0.093886 | 0 |
| #N/A | 0 | 0.784763 | 0 | 0 | 0 | 0.304069 | 0 | 0.307381 |
| Lrrc28 | 1.750891 | 1.879976 | 1.630346 | 1.909711 | 1.79662 | 1.83282 | 2.278626 | 1.85278 |
| Pcdha2 | 0.021929 | 0.024811 | 0 | 0.080826 | 0.094845 | 0.076909 | 0.024533 | 0.038873 |
| AABR07051 | 0.09658 | 0.131131 | 0.082948 | 0.373779 | 0.146203 | 0.203236 | 0.08644 | 0.342416 |
| 43163 | 0 | 0.0312 | 0 | 0 | 0.029817 | 0 | 0 | 0 |
| Dnah9 | 0.019629 | 0 | 0.021073 | 0.039792 | 0.012735 | 0.003442 | 0.017568 | 0.020878 |

| | | | | | | | | |
|---|---|---|---|---|---|---|---|---|
| Klra5 | 0.181984 | 0.102953 | 0.048843 | 0.670765 | 0.344359 | 0.39891 | 0.45809 | 0.443579 |
| AABR07015 | 0 | 0 | 0 | 0 | 0 | 0 | 0 | 0 |
| Exosc6 | 5.242986 | 6.004536 | 6.246462 | 6.598719 | 5.185212 | 6.110719 | 9.227621 | 6.74399 |
| Il3ra | 7.20358 | 5.827827 | 7.933853 | 9.182885 | 7.829485 | 8.246935 | 9.6458 | 8.005925 |
| Cndp2 | 19.16071 | 19.01208 | 19.65482 | 22.20846 | 22.23681 | 22.27556 | 25.22395 | 24.09643 |
| Clic2 | 41.65105 | 40.88221 | 36.49856 | 45.32083 | 43.55578 | 45.10215 | 54.39361 | 52.87685 |
| Tagln2 | 121.8911 | 125.0317 | 128.7773 | 130.064 | 132.8031 | 140.6463 | 182.8908 | 183.332 |
| Ulk2 | 7.811234 | 7.45086 | 7.717782 | 6.795493 | 6.377314 | 6.156678 | 8.873945 | 8.166584 |
| Adamts17 | 0.311424 | 0.176181 | 0.284183 | 0.860894 | 0.589292 | 0.587072 | 0.871018 | 0.841893 |
| Syt13 | 0.030287 | 0.051402 | 0.032515 | 0.167449 | 0.065497 | 0.106222 | 0.118592 | 0.147646 |
| RGD156307 | 1.691704 | 1.479064 | 12.1352 | 1.842264 | 0.582023 | 1.483287 | 10.58137 | 8.042457 |
| N4bp2l2 | 11.14393 | 10.45757 | 8.986968 | 10.36603 | 9.822564 | 10.62771 | 10.6061 | 11.65629 |
| Cdk15 | 0 | 0 | 0 | 0.037146 | 0 | 0 | 0 | 0 |
| AC119015. | 21.23429 | 22.80396 | 21.05766 | 16.08438 | 20.23622 | 18.1449 | 32.01018 | 27.91251 |
| AABR0700( | 0.09785 | 0.055356 | 0.105048 | 0.495906 | 0.158705 | 0.071496 | 0.07298 | 0.144548 |
| #N/A | 0 | 0.418725 | 0 | 0 | 0 | 0 | 0.207013 | 0.164009 |
| RGD15648( | 5.582365 | 4.572163 | 4.8302 | 4.99038 | 4.955033 | 4.56588 | 4.614067 | 4.874078 |
| RGD155992 | 0.154472 | 0.174778 | 0.165835 | 0.14234 | 0 | 0.270882 | 0.345632 | 0.410748 |
| AABR07015 | 0 | 0 | 0 | 0 | 0 | 0 | 0 | 0.072807 |
| Barx1 | 0 | 0 | 0 | 0 | 0.04127 | 0 | 0 | 0 |
| Tmem198 | 0.394718 | 0.186085 | 0.211877 | 0.697124 | 0.46237 | 0.432611 | 0.735988 | 0.408168 |
| Kcne5 | 0.08501 | 0.480922 | 0.091263 | 0.391665 | 0.367679 | 0.372683 | 0.57063 | 0.527438 |
| Trmt10b | 2.819703 | 2.643443 | 4.281229 | 3.674662 | 3.179572 | 3.284652 | 3.334819 | 3.034795 |
| AABR07054 | 0 | 0 | 0 | 0 | 0 | 1.076121 | 0 | 0 |
| Mylk | 8.749867 | 8.678577 | 8.062849 | 10.78296 | 11.12562 | 11.28288 | 11.30241 | 11.32306 |
| RGD130792 | 26.16419 | 27.1904 | 27.71 | 25.14727 | 24.4176 | 25.41659 | 27.76743 | 28.4358 |
| AABR07045 | 0.778675 | 1.468391 | 0.835958 | 1.136071 | 0.631478 | 0.739639 | 0.871147 | 0.575149 |
| Naa30 | 9.193928 | 9.653832 | 10.16937 | 8.610901 | 8.485218 | 9.170683 | 9.142896 | 8.817832 |
| Vgll2 | 0 | 0 | 0 | 0.242022 | 0.213 | 0.172719 | 0.146921 | 0.0873 |
| Fam206a | 2.529495 | 2.740217 | 2.253349 | 2.479606 | 3.782593 | 3.020069 | 3.070728 | 2.862149 |
| AC103170. | 0 | 0 | 0 | 0 | 0 | 0 | 0 | 0 |
| Sh3tc2 | 1.313419 | 1.184025 | 1.203692 | 1.190585 | 1.166165 | 1.170333 | 1.242415 | 1.485946 |
| RGD156274 | 3.826849 | 4.011524 | 2.930232 | 4.122648 | 3.529399 | 3.922839 | 3.903546 | 4.090264 |
| Tmem63a | 2.580881 | 2.868308 | 3.033059 | 3.518018 | 3.021849 | 3.521583 | 3.194906 | 3.519328 |
| Prg4 | 1.135171 | 1.06102 | 0.8831 | 1.940434 | 2.472676 | 2.062762 | 3.0185 | 2.478941 |
| Gstm3 | 0 | 0.180364 | 0 | 0.220334 | 0.344734 | 0.27954 | 0.17834 | 0.282585 |
| Sox18 | 21.98471 | 24.40328 | 24.35891 | 23.50642 | 21.13815 | 20.56879 | 25.95795 | 23.97421 |
| Usp18 | 10.50311 | 9.17522 | 8.705784 | 12.79397 | 13.97767 | 12.75111 | 20.48789 | 20.23673 |
| AABR07014 | 2.948532 | 2.802345 | 2.405732 | 2.716956 | 3.060676 | 3.619385 | 1.979212 | 3.763339 |
| Eif2b5 | 15.33645 | 15.37369 | 15.0204 | 14.99969 | 14.9466 | 16.0568 | 13.92185 | 14.84549 |
| Timd4 | 0.514906 | 0.349555 | 0.608063 | 0.616805 | 0.946492 | 0.270882 | 1.209713 | 1.414798 |
| AABR07061 | 10.38591 | 13.38053 | 14.10139 | 12.62624 | 10.38079 | 11.01948 | 14.10914 | 16.63187 |
| Naa16 | 2.626862 | 2.277587 | 1.624625 | 2.44686 | 2.439029 | 2.002813 | 2.715211 | 3.062245 |
| RGD156179 | 2.100056 | 3.449199 | 3.418181 | 3.620546 | 2.856763 | 3.088685 | 2.955759 | 2.942188 |
| Eps8l2 | 0.67262 | 0.631105 | 0.651652 | 0.710494 | 0.815989 | 0.532217 | 0.844265 | 0.756126 |
| Gabra4 | 0.035551 | 0.053632 | 0.114497 | 0.065517 | 0.025627 | 0.093512 | 0.013257 | 0.042014 |
| Zic5 | 0 | 0 | 0.027504 | 0 | 0.027702 | 0 | 0 | 0 |
| AC106663. | 34.58127 | 27.94791 | 18.03224 | 23.67135 | 21.36699 | 27.72195 | 30.95039 | 19.2664 |
| RGD155978 | 24.45496 | 24.26937 | 22.34208 | 24.85348 | 25.26485 | 23.05605 | 23.07244 | 22.50804 |
| Krt80 | 0.410311 | 0.367529 | 0.440495 | 0.456853 | 0.480635 | 0.569621 | 0.860695 | 0.515212 |
| Atraid | 13.893 | 11.91901 | 12.18333 | 16.08438 | 18.43396 | 14.85862 | 13.43633 | 13.3515 |
| AABR07062 | 0 | 0.151533 | 0.07189 | 0.030852 | 0 | 0.058714 | 0.112375 | 0 |
| Enpep | 11.77165 | 12.22734 | 12.50443 | 9.51345 | 8.808609 | 9.342431 | 9.776962 | 10.2994 |
| Pfn1 | 320.998 | 337.6354 | 352.7414 | 384.7833 | 379.9121 | 396.4063 | 381.949 | 382.7491 |

| | | | | | | | | |
|---|---|---|---|---|---|---|---|---|
| AABR0700( | 6.568338 | 5.590869 | 5.822364 | 8.828825 | 8.14478 | 9.193464 | 15.10123 | 12.49827 |
| Ap3s1 | 4.727196 | 4.564753 | 4.506203 | 5.970621 | 6.124925 | 5.181012 | 7.248998 | 6.610005 |
| Ltb4r2 | 0 | 0.055014 | 0.104398 | 0.13441 | 0 | 0.085264 | 0.054396 | 0 |
| Adora1 | 3.827991 | 3.340397 | 3.867852 | 2.305453 | 2.813541 | 2.895706 | 3.330901 | 3.481643 |
| Tmem91 | 1.513466 | 1.484093 | 0.974881 | 1.952439 | 2.291084 | 1.061607 | 2.596238 | 1.34146 |
| AABR0703( | 0.115598 | 0.261588 | 0.124102 | 0.319557 | 0 | 0.304069 | 0 | 0.10246 |
| Zfp874b | 4.84991 | 4.931285 | 4.749344 | 4.318021 | 3.89767 | 4.051278 | 5.499186 | 4.502034 |
| Tslp | 0.387896 | 0.731476 | 0.416431 | 0.595718 | 1.817513 | 0.680215 | 0.289307 | 1.031434 |
| AABR0706( | 0 | 0.789994 | 0 | 0.643375 | 0.503311 | 0.408129 | 1.041505 | 0 |
| AABR0701: | 0 | 0 | 0 | 0 | 0 | 0 | 0.141509 | 0 |
| AABR0700( | 0.018014 | 0.020382 | 0.038678 | 0.033198 | 0.038956 | 0.015794 | 0.020153 | 0 |
| Nectin1 | 0.763293 | 0.909084 | 0.70084 | 0.953217 | 1.042532 | 1.003884 | 1.640461 | 1.361991 |
| Clk2 | 28.15013 | 29.37995 | 28.89048 | 32.53733 | 31.41677 | 31.82277 | 32.08732 | 33.35499 |
| Foxj2 | 12.43131 | 12.34366 | 12.7572 | 11.35186 | 10.51388 | 10.32095 | 14.70876 | 13.87477 |
| #N/A | 25.5178 | 25.08643 | 23.71636 | 19.87331 | 20.3998 | 21.45504 | 22.05381 | 21.86735 |
| Lingo1 | 0.404617 | 0.38552 | 0.434382 | 0.981154 | 0.967119 | 0.821569 | 1.072107 | 0.811641 |
| AABR0706{ | 0 | 0 | 0 | 0.450964 | 0 | 0 | 0 | 0 |
| LOC10369( | 0 | 0 | 0 | 0 | 0 | 0 | 0 | 0.056158 |
| Prr36 | 0.147417 | 0.111197 | 0.175847 | 0.196212 | 0.212534 | 0.157979 | 0.311523 | 0.333917 |
| Grk4 | 1.82873 | 1.901355 | 1.246934 | 1.685102 | 1.630002 | 1.711772 | 2.488259 | 2.190397 |
| Katnbl1 | 2.199847 | 2.70546 | 3.542514 | 2.952475 | 3.361154 | 2.390069 | 2.942609 | 3.730117 |
| Tulp4 | 4.822903 | 5.480409 | 4.976836 | 4.798496 | 5.15874 | 5.285907 | 6.628277 | 7.232115 |
| AABR0706( | 1.689223 | 0.955638 | 1.813489 | 0 | 0 | 0 | 0 | 0 |
| Srp19 | 33.55016 | 33.73391 | 28.07195 | 33.78357 | 35.67891 | 32.20686 | 32.11709 | 33.17075 |
| AC122959. | 0.489401 | 0 | 0.525403 | 0.901928 | 0 | 0.429107 | 0 | 2.168903 |
| AABR0700( | 3.254379 | 3.514805 | 3.334976 | 1.635699 | 1.279605 | 1.945529 | 3.640855 | 2.622289 |
| Acer3 | 2.864054 | 3.113185 | 2.443684 | 2.627589 | 2.758775 | 2.478308 | 2.280702 | 2.361188 |
| Tjp1 | 23.48839 | 23.12384 | 21.76273 | 19.98578 | 21.12138 | 20.12939 | 22.62318 | 22.89861 |
| Mta2 | 13.98528 | 13.70196 | 14.3932 | 12.83843 | 13.73873 | 13.36868 | 16.68526 | 15.65791 |
| Nipal1 | 0.325373 | 0.303178 | 0.123285 | 0.176364 | 0.041391 | 0.134253 | 0.064238 | 0.067857 |
| AABR0707: | 0 | 0 | 0 | 0.1018 | 0 | 0 | 0 | 0 |
| AC113710. | 0 | 0 | 0 | 0 | 0 | 0.253671 | 0 | 0 |
| LOC681458 | 0 | 0 | 0 | 0.013404 | 0 | 0.012754 | 0 | 0.012893 |
| Vom2r16 | 0.020608 | 0 | 0.022124 | 0 | 0 | 0 | 0 | 0 |
| Ccdc112 | 1.509648 | 1.33445 | 1.51941 | 1.934473 | 1.938428 | 1.840716 | 1.688927 | 1.839855 |
| Phyhipl | 0.356231 | 0.268706 | 0.212465 | 1.240065 | 0.984367 | 0.624687 | 0.309972 | 0.841987 |
| AABR0700: | 0 | 0 | 0 | 1.556553 | 0 | 0.740556 | 0 | 0 |
| Bhlhe22 | 0.185098 | 0.076156 | 0.234845 | 0.325616 | 0.309313 | 0.309834 | 0.225905 | 0.238635 |
| Loxl1 | 7.930822 | 8.740002 | 10.82899 | 27.53867 | 25.66563 | 27.6013 | 29.91111 | 28.21763 |
| Mknk1 | 10.70807 | 11.64668 | 11.22385 | 12.37097 | 12.00178 | 11.91273 | 12.44344 | 12.00164 |
| Scd | 0.046808 | 0.079441 | 0.025125 | 0.291136 | 0.316327 | 0.246245 | 0.078549 | 0.155579 |
| AABR0705{ | 0.166148 | 0.066349 | 0.241325 | 0.063041 | 0.073975 | 0.077124 | 0.076538 | 0.060639 |
| Gzf1 | 6.123237 | 5.985925 | 6.192412 | 7.12061 | 7.071193 | 6.754025 | 6.713397 | 6.946981 |
| LOC10255I | 0 | 0 | 0 | 0 | 0 | 0 | 0 | 0 |
| Trib2 | 9.953045 | 10.44279 | 8.543985 | 9.243412 | 7.696264 | 7.95531 | 10.2381 | 9.723131 |
| Rttn | 1.830795 | 1.322207 | 1.338196 | 1.627182 | 1.642657 | 1.582473 | 1.350948 | 1.898933 |
| Tpk1 | 1.257662 | 1.138389 | 0.877618 | 1.216831 | 1.121911 | 0.868392 | 1.07285 | 1.351609 |
| AABR0703: | 0.218738 | 0.29699 | 0.140898 | 0.161247 | 0.094607 | 0.19179 | 0.195772 | 0.077551 |
| Mum1l1 | 0.9002 | 0.756625 | 0.73172 | 0.545099 | 0.41716 | 0.552507 | 0.402842 | 0.421743 |
| Itgal | 3.47094 | 3.309885 | 3.539339 | 3.315999 | 3.338858 | 2.849935 | 3.636379 | 3.560058 |
| Efcab2 | 0.988036 | 1.117917 | 1.446436 | 0.634547 | 1.068349 | 0.577541 | 0.803906 | 0.743057 |
| Neto2 | 0.165925 | 0.300378 | 0.249383 | 0.45868 | 0.358825 | 0.232773 | 0.408385 | 0.294135 |
| Rn50_7_11 | 0 | 0 | 0.142867 | 0.061313 | 0.143895 | 0.175023 | 0.148881 | 0.17693 |
| Mogat1 | 0.115726 | 0.196407 | 0.186359 | 0.426547 | 0.250265 | 0.050734 | 0.194203 | 0.359007 |

| | | | | | | | | |
|---|---|---|---|---|---|---|---|---|
| AC096352. | 2.929562 | 2.071663 | 3.538206 | 4.386649 | 3.563655 | 3.852964 | 4.506513 | 5.193233 |
| Hmox2 | 20.20281 | 21.9781 | 21.10115 | 21.98877 | 22.00082 | 22.59084 | 21.86043 | 21.7129 |
| Slc30a3 | 7.003508 | 6.714347 | 7.748292 | 9.827974 | 9.682768 | 10.57041 | 11.69301 | 9.998431 |
| Prrt4 | 0.449879 | 0.794066 | 0.56025 | 0.348219 | 0.330784 | 0.268229 | 0.301983 | 0.4466 |
| Aspscr1 | 15.12442 | 15.89535 | 17.76562 | 19.09716 | 16.93543 | 15.45279 | 18.01788 | 17.38792 |
| AABR0705 | 22.18513 | 22.42565 | 20.55288 | 20.23519 | 21.79662 | 17.08216 | 20.28415 | 20.56213 |
| LOC68022 | 0.759928 | 0.703491 | 0.815831 | 0.763902 | 0.373499 | 0.605732 | 0.231865 | 0.489863 |
| Mir215 | 0 | 0 | 1.113231 | 0.477754 | 0 | 0 | 1.160092 | 0.45955 |
| Mrps21l | 0.784508 | 0.665726 | 0.631665 | 0.361447 | 0.424139 | 0.171964 | 0 | 0.869186 |
| Tspyl2 | 6.172845 | 5.712054 | 5.050273 | 6.195516 | 7.046798 | 5.955604 | 7.085613 | 7.444222 |
| Fam111a | 11.17555 | 11.25203 | 9.936397 | 29.33597 | 29.54449 | 29.77031 | 7.894518 | 7.229087 |
| Ncor1 | 10.16808 | 10.6916 | 10.74013 | 8.939638 | 9.051968 | 9.020397 | 10.40941 | 10.8619 |
| Mir343 | 0 | 0 | 0 | 0.518851 | 0 | 1.481112 | 0 | 0.998162 |
| Eif4b | 88.91894 | 83.33835 | 84.65938 | 85.18737 | 83.80627 | 85.07743 | 95.45327 | 97.41971 |
| Fam193b | 10.69583 | 10.61997 | 11.48266 | 13.19469 | 13.19382 | 12.07672 | 14.60343 | 15.94229 |
| Tln2 | 10.28089 | 11.51784 | 11.4718 | 9.318008 | 8.481066 | 8.799845 | 11.766 | 12.13251 |
| Acap2 | 6.261323 | 6.727354 | 6.41882 | 7.039785 | 6.806195 | 6.363663 | 8.472759 | 8.111132 |
| Calm2 | 169.096 | 168.9574 | 156.1228 | 173.5981 | 166.8195 | 161.0968 | 185.0127 | 182.3267 |
| Cacna1d | 0.282719 | 0.186599 | 0.370966 | 0.209859 | 0.178325 | 0.151488 | 0.184505 | 0.208824 |
| Sertad2 | 5.1844 | 5.236009 | 5.304305 | 4.745159 | 4.36426 | 4.576195 | 6.072563 | 5.76712 |
| Hist2h4 | 0.671358 | 0.569707 | 1.441491 | 0.927945 | 1.996307 | 1.61878 | 2.065485 | 1.785174 |
| Scyl1 | 18.23066 | 19.54741 | 19.91327 | 21.45194 | 22.09869 | 20.8829 | 23.42052 | 22.0795 |
| L1td1 | 0.021792 | 0 | 0 | 0 | 0 | 0 | 0 | 0.019315 |
| LOC10090 | 0.36475 | 0.178835 | 0.156633 | 0.168051 | 0.092026 | 0.085283 | 0.217635 | 0.183201 |
| Cnpy2 | 32.58441 | 32.83068 | 35.8264 | 37.71287 | 34.2118 | 37.90935 | 35.63216 | 35.2062 |
| Cers3 | 0 | 0 | 0 | 0 | 0 | 0.023271 | 0 | 0 |
| Misp | 0.733245 | 0.599179 | 0.568523 | 0.450438 | 0.396424 | 0.410748 | 1.093763 | 1.227611 |
| Jmjd4 | 1.192117 | 1.118537 | 1.061309 | 0.937734 | 1.320458 | 1.453151 | 1.138514 | 0.979318 |
| Sh3bgr | 238.1141 | 243.3576 | 224.6764 | 240.0446 | 227.083 | 246.6802 | 247.8857 | 247.2201 |
| Tmco5b | 0 | 0 | 0 | 0.106363 | 0.083207 | 0 | 0.086091 | 0.034103 |
| Rnase10 | 0 | 0.092722 | 0 | 0.075514 | 0 | 0.143707 | 0.275045 | 0 |
| Slc38a2 | 32.28949 | 31.84265 | 28.24856 | 34.60484 | 33.19166 | 33.99752 | 38.51294 | 39.00422 |
| Fgf2 | 0.784045 | 0.84042 | 0.841722 | 1.216785 | 1.160115 | 1.013086 | 1.431146 | 1.645905 |
| AABR0706 | 0 | 0 | 0 | 0 | 0 | 0 | 0 | 0 |
| LOC10369 | 0 | 0 | 0 | 0.120033 | 0 | 0 | 0 | 0 |
| Cldnd2 | 0.07029 | 0 | 0.226382 | 0.194308 | 0 | 0.06163 | 0 | 0.124603 |
| Zmym5 | 9.046789 | 7.253128 | 7.448087 | 8.310688 | 8.761936 | 9.270492 | 11.20777 | 10.03557 |
| Prepl | 42.56814 | 42.30676 | 40.9293 | 45.75552 | 46.67694 | 45.07684 | 40.06724 | 42.51546 |
| AABR0700 | 1.39568 | 1.358066 | 1.31855 | 1.723326 | 1.629859 | 1.370582 | 1.498968 | 1.63292 |
| Tubg2 | 0.518796 | 1.063924 | 0.90506 | 0.567684 | 0.806389 | 0.5686 | 0.507855 | 0.546053 |
| AABR0702 | 0.082336 | 0 | 0 | 0.07587 | 0.178058 | 0.072193 | 0.092114 | 0.291915 |
| AC131360. | 0.435294 | 0.17238 | 0.186927 | 0.501383 | 0.30594 | 0.438917 | 0.194795 | 0.405114 |
| #N/A | 0.178723 | 0 | 0 | 0 | 0 | 0 | 0 | 0 |
| Ankrd12 | 2.619099 | 2.811885 | 2.645019 | 2.753938 | 2.93624 | 2.958589 | 3.169816 | 3.175955 |
| Nqo2 | 46.41178 | 44.78266 | 43.31093 | 41.71153 | 42.05418 | 39.25488 | 42.61479 | 38.83664 |
| RGD15600 | 7.5906 | 8.066844 | 8.181986 | 8.410318 | 8.174377 | 8.541603 | 9.420304 | 9.370067 |
| Sema3d | 1.384447 | 1.291414 | 1.293415 | 1.256237 | 1.759812 | 1.538205 | 2.163672 | 2.01395 |
| AABR0706 | 0 | 0.170257 | 0 | 0 | 0 | 0 | 0 | 0 |
| Rac1 | 136.9755 | 136.1168 | 131.21 | 124.8489 | 118.6845 | 125.4772 | 154.314 | 156.4844 |
| Tbc1d8 | 2.397957 | 2.177681 | 2.213047 | 2.413141 | 2.41092 | 2.849483 | 2.776859 | 2.880515 |
| AABR0701 | 4.204563 | 2.594872 | 4.924219 | 1.761063 | 2.066515 | 2.345996 | 2.138126 | 5.420674 |
| Ctgf | 32.0892 | 33.06023 | 32.88695 | 125.0404 | 120.1036 | 122.4629 | 161.4917 | 159.7227 |
| Nmnat3 | 4.877095 | 4.551096 | 4.723081 | 4.772034 | 4.512404 | 4.099901 | 3.712518 | 4.567919 |
| Pwwp2a | 3.656838 | 4.468543 | 3.925849 | 4.195192 | 3.617989 | 3.71933 | 4.807052 | 5.104949 |

| | | | | | | | | |
|---|---|---|---|---|---|---|---|---|
| AABR07012 | 0.148767 | 0.673291 | 0 | 0.137083 | 0 | 0.130439 | 0 | 0.263719 |
| #N/A | 0 | 0 | 0 | 0 | 0 | 0 | 0.129326 | 0 |
| Panx2 | 4.886288 | 4.512318 | 4.590025 | 3.741066 | 3.379869 | 2.929706 | 4.46168 | 5.222628 |
| LOC100911 | 0 | 0 | 0.570743 | 3.83739 | 0 | 0 | 0 | 0 |
| AABR07035 | 0.070243 | 0.079476 | 0.15082 | 0.258903 | 0.341786 | 0.338739 | 0.039292 | 0.155649 |
| Epc2 | 6.790806 | 6.767247 | 5.902449 | 6.034915 | 6.636171 | 6.564551 | 6.723091 | 7.051582 |
| Rfesd | 10.25584 | 12.21878 | 10.28114 | 9.450355 | 11.08949 | 7.503535 | 11.77772 | 11.67888 |
| Zfp382 | 0.643779 | 0.835002 | 0.589996 | 1.099622 | 0.967761 | 0.853583 | 0.966164 | 1.07164 |
| AABR07035 | 0.286153 | 0 | 0 | 0 | 0 | 0.250899 | 0.64027 | 0 |
| AABR07032 | 0 | 0 | 0 | 0 | 0 | 0.156705 | 0 | 0 |
| Dusp5 | 9.784786 | 9.82186 | 11.15532 | 10.9911 | 11.09511 | 10.857 | 8.912425 | 9.305929 |
| AABR07050 | 0.630915 | 2.141551 | 2.031982 | 1.162726 | 1.364398 | 0.553187 | 2.117518 | 2.236844 |
| RGD131055 | 4.910739 | 4.794663 | 5.058485 | 5.413148 | 5.558038 | 5.150792 | 5.442572 | 5.301804 |
| Mtg1 | 20.8685 | 21.36509 | 23.28142 | 19.66001 | 20.29149 | 20.10679 | 18.42477 | 18.94541 |
| P2rx3 | 0.016795 | 0.038005 | 0.072121 | 0.201184 | 0.127119 | 0.103079 | 0.112735 | 0.163746 |
| Atg4c | 2.870939 | 2.988465 | 3.123231 | 2.927639 | 2.897351 | 2.584368 | 3.554479 | 3.121444 |
| Hes5 | 0 | 0.200167 | 0 | 0 | 0.095646 | 0.155117 | 0 | 0 |
| Oaz2 | 155.7124 | 146.9222 | 154.2777 | 133.1855 | 131.4202 | 125.0299 | 135.1477 | 140.6352 |
| Ikbip | 9.144497 | 8.799004 | 6.796524 | 11.7392 | 11.7048 | 11.64994 | 10.92997 | 12.81595 |
| Myzap | 322.2771 | 323.7595 | 308.0019 | 336.2782 | 333.5114 | 326.4687 | 342.1513 | 348.6815 |
| Gopc | 7.713575 | 7.63835 | 7.631784 | 7.721866 | 8.313888 | 7.812929 | 9.112863 | 9.363853 |
| Impg2 | 0.011983 | 0.067791 | 0 | 0.022084 | 0.025914 | 0 | 0.053624 | 0.010621 |
| Tbl1xr1 | 7.432626 | 7.355594 | 7.008246 | 7.128799 | 7.372533 | 7.499504 | 8.149137 | 8.640269 |
| LOC100361 | 0 | 0 | 0.03613 | 0.124044 | 0.03639 | 0 | 0 | 0 |
| Plekho2 | 8.194107 | 7.410351 | 7.346512 | 9.011943 | 9.145981 | 9.141695 | 11.30293 | 12.02664 |
| AABR07071 | 0 | 0 | 0 | 0 | 0 | 0.521756 | 0 | 0 |
| AABR07031 | 0.17113 | 0 | 0 | 0.31538 | 0 | 0 | 0.765813 | 0 |
| Xpa | 4.349329 | 3.149479 | 4.435818 | 4.08789 | 4.185551 | 4.499925 | 4.525228 | 4.818783 |
| Sec31a | 18.985 | 20.28569 | 21.097 | 18.66406 | 18.35304 | 19.17054 | 22.51958 | 23.09025 |
| Mip | 0 | 0.025817 | 0 | 0 | 0.049344 | 0.020006 | 0.051054 | 0 |
| AC119459. | 9.063333 | 10.25473 | 8.108389 | 15.31109 | 13.61118 | 14.12753 | 11.26628 | 8.033282 |
| Srp9 | 43.05993 | 42.42948 | 42.62928 | 40.40473 | 44.85458 | 40.24432 | 39.87992 | 39.00497 |
| Grik4 | 0.952685 | 0.898265 | 1.039814 | 0.775445 | 0.669581 | 0.696096 | 1.030294 | 1.449574 |
| Hspa4 | 30.09882 | 29.31019 | 28.96728 | 32.48546 | 30.46592 | 31.14465 | 35.68221 | 36.08421 |
| Cthrc1 | 0.387577 | 0.048725 | 0.416088 | 1.785683 | 2.235099 | 1.246035 | 1.734414 | 1.870322 |
| AABR07021 | 0.238569 | 0.404895 | 0.12806 | 0.219832 | 0 | 0.313766 | 0 | 0.211456 |
| AABR07049 | 0 | 0 | 0 | 0.319557 | 0 | 0 | 0 | 0 |
| 5S_rRNA | 0 | 0 | 0 | 0 | 0 | 0 | 0 | 0 |
| Rpl27 | 445.3304 | 434.7873 | 433.8011 | 422.8232 | 415.3904 | 367.3159 | 396.4311 | 391.1106 |
| Usp5 | 7.793618 | 7.819835 | 8.191539 | 12.60152 | 7.296442 | 7.291879 | 7.439613 | 11.32485 |
| AABR07016 | 0 | 0 | 0 | 1.250081 | 0.586762 | 0.475798 | 0.303547 | 1.442938 |
| Smim15 | 11.92155 | 12.00965 | 11.59166 | 10.62388 | 10.45948 | 10.24652 | 11.26066 | 13.37053 |
| Ugcg | 10.82748 | 11.16432 | 10.68198 | 10.32797 | 10.00695 | 10.36451 | 12.40965 | 12.73721 |
| Upk1b | 0.028275 | 0.159961 | 0.060711 | 0.234491 | 0.366885 | 0.54542 | 0.379598 | 0.426051 |
| Ddah2 | 23.45767 | 25.3992 | 28.21744 | 33.44608 | 30.77785 | 31.08166 | 39.29735 | 38.43423 |
| Ctss | 22.32164 | 24.47786 | 18.90975 | 35.53185 | 35.34617 | 36.22143 | 50.4775 | 50.58714 |
| Pgbd5 | 0 | 0 | 0 | 0.144326 | 0.141133 | 0.183109 | 0.087614 | 0.069414 |
| Nme7 | 3.117914 | 4.165322 | 3.508594 | 3.738406 | 3.85878 | 3.293722 | 4.118578 | 3.729143 |
| RGD156131 | 1.781154 | 2.41835 | 1.147309 | 1.641263 | 1.155561 | 1.249374 | 1.594141 | 2.052343 |
| Lrtm2 | 0.516402 | 0.53691 | 0.599341 | 0.514426 | 0.573469 | 0.648579 | 0.234214 | 0.309265 |
| LOC100910 | 0 | 0 | 0.23327 | 0 | 0.234948 | 0 | 0 | 0.385183 |
| AABR07050 | 0.118744 | 0.134353 | 0.127479 | 0 | 0 | 0.104114 | 0 | 0 |
| RT1-Ba | 23.67741 | 23.1045 | 23.06559 | 25.97358 | 26.00843 | 23.78107 | 26.27901 | 26.09429 |
| AABR07063 | 0.021418 | 0 | 0 | 0 | 0 | 0.037558 | 0 | 0.018983 |

| | | | | | | | | |
|---|---|---|---|---|---|---|---|---|
| Sugp2 | 11.89303 | 12.69732 | 12.36197 | 10.41944 | 10.28781 | 9.850277 | 11.13559 | 11.24414 |
| AABR07032 | 0 | 0.871317 | 0 | 0 | 0 | 0 | 0 | 0 |
| Ccrl2 | 3.949677 | 4.143327 | 4.268312 | 4.651776 | 5.260633 | 4.953811 | 5.355142 | 5.448258 |
| AABR07001 | 0 | 0 | 0 | 0 | 0 | 0.194966 | 0 | 0 |
| Nxpe1 | 21.42651 | 19.45559 | 19.39446 | 15.26403 | 17.71684 | 16.6292 | 13.19414 | 13.72487 |
| Snd1 | 15.37363 | 15.21323 | 16.43382 | 16.88404 | 15.75025 | 15.80568 | 17.7708 | 17.08779 |
| Wdr83 | 7.817135 | 7.286843 | 8.48756 | 9.98623 | 9.365047 | 10.08639 | 8.993914 | 7.912908 |
| Suv39h11 | 0.972936 | 1.420427 | 1.398294 | 1.272483 | 1.23867 | 1.609828 | 1.492267 | 1.669087 |
| Xrcc3 | 4.039657 | 4.401397 | 3.962042 | 4.871269 | 4.098391 | 3.498247 | 6.472211 | 6.36542 |
| Phb2 | 148.7136 | 152.4696 | 160.9609 | 138.7601 | 132.5919 | 132.9385 | 132.4705 | 139.4096 |
| Snu13 | 7.442185 | 6.583286 | 5.374863 | 7.107051 | 4.974588 | 4.508399 | 5.752499 | 4.797366 |
| AABR07063 | 1.172371 | 1.326483 | 0.419539 | 1.440392 | 1.690224 | 0.342645 | 0.437199 | 1.039131 |
| Spopl | 7.096009 | 5.910089 | 5.925126 | 4.540759 | 5.044164 | 5.011995 | 6.615583 | 6.231309 |
| LOC100910 | 0.114399 | 0.194155 | 0.199573 | 0.224004 | 0.108235 | 0.32599 | 0.223972 | 0.418263 |
| Rn50_9_08 | 0 | 0 | 0 | 0 | 0 | 0 | 0.154577 | 0 |
| Golph3 | 22.54411 | 20.58591 | 20.16131 | 21.83339 | 22.31886 | 20.84855 | 24.07493 | 24.20821 |
| Eif2ak4 | 1.370988 | 1.353182 | 1.371633 | 1.365452 | 1.457197 | 1.631765 | 1.572962 | 1.67539 |
| Llph | 23.64026 | 20.99394 | 18.73939 | 7.598919 | 23.33264 | 17.233 | 25.21755 | 23.38999 |
| Fam167a | 0.585749 | 0.70693 | 0.461148 | 1.247409 | 1.154125 | 1.129887 | 2.344552 | 1.903653 |
| AABR07062 | 0.405937 | 0 | 0 | 0 | 0 | 0 | 0 | 0 |
| Otud1 | 43.9445 | 47.2524 | 54.82093 | 52.09787 | 46.8543 | 45.24322 | 76.57119 | 74.88073 |
| Ccdc141 | 26.86286 | 26.50153 | 24.55439 | 24.65909 | 25.15004 | 23.51165 | 27.62653 | 28.55258 |
| AABR07015 | 1.454609 | 1.737256 | 1.301346 | 2.308406 | 1.398087 | 0.99198 | 2.169802 | 1.432547 |
| Rasgrf1 | 0 | 0 | 0 | 0.012702 | 0.044714 | 0.024172 | 0.015421 | 0.036653 |
| Gk2 | 0 | 0 | 0 | 0 | 0 | 0 | 0 | 0 |
| Vim | 145.4233 | 147.4699 | 154.6681 | 212.8906 | 207.8903 | 210.905 | 244.3078 | 240.5207 |
| Hyi | 4.536028 | 5.645532 | 5.464903 | 4.829958 | 5.231724 | 3.491092 | 5.187478 | 4.333213 |
| Chsy1 | 4.400966 | 4.081287 | 4.171507 | 5.158979 | 4.849056 | 5.16538 | 6.029858 | 5.480863 |
| Lrig3 | 1.185123 | 1.465647 | 1.819693 | 1.930125 | 1.981788 | 1.872828 | 1.927127 | 2.113082 |
| Rn50_X_05 | 0.084598 | 0 | 0 | 0 | 0.091474 | 0 | 0.094644 | 0 |
| Pikfyve | 2.394031 | 2.677015 | 2.431706 | 2.732967 | 2.673504 | 2.703744 | 2.954323 | 3.105897 |
| Pygm | 283.8956 | 289.1939 | 295.16 | 225.0026 | 224.3085 | 227.8672 | 264.4952 | 264.7448 |
| Galnt1 | 19.79268 | 17.93355 | 17.55575 | 19.33539 | 20.68624 | 19.41911 | 21.13666 | 20.85018 |
| Appl1 | 8.089045 | 7.022735 | 6.567205 | 7.887333 | 8.068163 | 8.624929 | 8.097068 | 7.626519 |
| Gpt | 25.03934 | 28.39893 | 30.30611 | 19.93908 | 19.525 | 19.73795 | 21.85141 | 22.43368 |
| Dcun1d2 | 1.033666 | 9.510826 | 0.690949 | 0.359427 | 0.5483 | 0.598513 | 1.985551 | 0.293872 |
| F13a1 | 6.024398 | 6.831744 | 6.174926 | 8.816684 | 9.181631 | 8.473027 | 13.66264 | 13.45804 |
| Tssk5 | 0.069267 | 0.039186 | 0.037181 | 0.031913 | 0.074898 | 0.060733 | 0 | 0.092092 |
| Usp36 | 11.74563 | 12.2473 | 11.40434 | 8.979487 | 8.918279 | 8.506422 | 10.4673 | 10.48728 |
| Rn50_10_0 | 0 | 0 | 0 | 0 | 0 | 0 | 0 | 0 |
| Olr153 | 0 | 0 | 0.05766 | 0 | 0 | 0 | 0 | 0 |
| Cend1 | 0 | 0 | 0 | 0.107229 | 0 | 0 | 0 | 0 |
| AABR07012 | 0.385044 | 1.306976 | 0.826738 | 0 | 0.416342 | 0 | 0 | 0.341283 |
| Polr3c | 6.399049 | 6.313274 | 6.536996 | 6.549368 | 6.703722 | 6.037803 | 6.267197 | 6.535321 |
| Zkscan3 | 17.6605 | 16.93188 | 18.709 | 16.80156 | 16.41066 | 15.76391 | 17.3118 | 18.02396 |
| Trim62 | 0.990112 | 0.622369 | 0.866106 | 0.844768 | 0.99129 | 0.96459 | 0.902565 | 1.007598 |
| Ptpn11 | 41.16086 | 43.40475 | 41.67128 | 39.08696 | 40.18174 | 40.25186 | 47.58285 | 47.28615 |
| Gnpat | 32.09524 | 30.28638 | 29.41107 | 27.02016 | 24.96261 | 24.8561 | 26.96639 | 25.37634 |
| AABR07065 | 16.2085 | 14.10704 | 13.83145 | 12.25476 | 9.437085 | 11.29642 | 8.369238 | 11.78781 |
| Serp2 | 0.107307 | 0.121413 | 0.057601 | 0.148319 | 0.058015 | 0.188174 | 0.300126 | 0.523114 |
| LOC100911 | 0.327902 | 1.075916 | 0.915261 | 0.513653 | 0.283644 | 1.610026 | 2.751315 | 0.40689 |
| Cdh15 | 0.265336 | 0.150108 | 0.122081 | 0.157176 | 0.245918 | 0.116323 | 0.19083 | 0.11759 |
| Ppp3cb | 22.80925 | 23.76068 | 20.68156 | 22.30187 | 21.88769 | 22.95724 | 24.0137 | 23.38061 |
| Ccl19 | 2.955032 | 0.933064 | 3.098639 | 8.485459 | 6.687698 | 7.049862 | 9.687228 | 6.578436 |

| | | | | | | | | |
|---|---|---|---|---|---|---|---|---|
| Cyba | 28.09213 | 30.16482 | 26.86467 | 43.47809 | 35.90516 | 35.4522 | 40.73465 | 40.37096 |
| RGD135912 | 5.252804 | 6.273484 | 7.762632 | 7.828063 | 7.152317 | 7.334946 | 8.198226 | 6.610117 |
| AABR07069 | 0 | 0 | 0.341752 | 0 | 0.172105 | 0 | 0 | 0.141078 |
| Mfsd6 | 6.229666 | 6.82481 | 6.187486 | 4.594917 | 4.383777 | 4.743795 | 5.073018 | 5.4841 |
| Krt13 | 0 | 0 | 0.085373 | 0.036639 | 0.171974 | 0.104589 | 0 | 0.035243 |
| Syt11 | 1.647578 | 2.142389 | 1.821579 | 2.413223 | 2.180346 | 2.123774 | 3.067476 | 2.768088 |
| #N/A | 0.204554 | 0.231444 | 0.439204 | 0.376978 | 0.442363 | 0.896767 | 2.975003 | 2.356987 |
| AC130391. | 0 | 0 | 0 | 0 | 0.2274 | 0 | 0.23528 | 0.559211 |
| #N/A | 0.14751 | 0 | 0.158361 | 0 | 0 | 0 | 0.165027 | 0.130745 |
| Tln1 | 28.36614 | 30.12156 | 31.48009 | 32.33803 | 31.49042 | 30.93533 | 38.42184 | 39.56533 |
| AABR07052 | 0.581844 | 0.263331 | 0.124929 | 0.750604 | 0.251656 | 0.306097 | 0.911317 | 0.30943 |
| Nucb2 | 8.008581 | 8.219144 | 8.109385 | 11.5838 | 11.31258 | 11.55416 | 14.01776 | 13.92802 |
| #N/A | 0 | 0 | 0 | 0 | 0 | 0 | 0.448925 | 0 |
| Cdc16 | 16.62292 | 14.38015 | 14.80478 | 15.45474 | 16.9263 | 15.16322 | 18.18001 | 14.93193 |
| Lcmt2 | 1.849079 | 1.980565 | 1.958637 | 2.09006 | 2.159333 | 2.118465 | 2.289325 | 1.81375 |
| Ppp6r1 | 28.00288 | 30.60176 | 30.74743 | 30.18575 | 27.53561 | 29.31678 | 36.36648 | 35.32307 |
| Tep1 | 8.105244 | 7.868914 | 8.418578 | 7.285057 | 6.783303 | 7.388473 | 9.017451 | 8.944494 |
| AABR07005 | 11.88767 | 10.04519 | 6.461858 | 7.210239 | 7.159168 | 8.971796 | 9.595764 | 8.402628 |
| Cpne3 | 16.44046 | 16.23335 | 14.32011 | 14.67583 | 14.0116 | 13.74765 | 14.79518 | 14.70693 |
| Gfpt1 | 11.58188 | 10.68228 | 10.81323 | 11.24234 | 11.26563 | 12.03194 | 11.75293 | 12.85393 |
| Wwc1 | 1.309504 | 1.256175 | 1.359994 | 0.62956 | 0.507894 | 0.673928 | 0.621039 | 0.517259 |
| Gnas | 0.831713 | 0.470522 | 0.549475 | 0.884297 | 0.44966 | 0.560959 | 0.858908 | 0.793895 |
| LOC103690 | 0.948276 | 0.476858 | 1.35738 | 1.067977 | 1.367143 | 0.877641 | 1.296643 | 1.260757 |
| Iqsec2 | 24.90574 | 24.87891 | 28.38955 | 23.36425 | 21.98674 | 22.37186 | 23.93398 | 23.82475 |
| Mfhas1 | 4.895398 | 4.643734 | 5.326304 | 4.434975 | 3.600172 | 4.133315 | 5.624275 | 5.683112 |
| LOC102552 | 0.073342 | 0 | 0 | 0 | 0 | 0.064306 | 0 | 0 |
| AABR07015 | 0 | 0 | 0 | 0.098879 | 0 | 0 | 0.180076 | 0 |
| Kcnh8 | 0.014005 | 0.015846 | 0.015036 | 0 | 0 | 0.061399 | 0.015669 | 0.012414 |
| Pigb | 0.382757 | 0.595473 | 0.410914 | 0.727434 | 0.284535 | 0.419502 | 0.588791 | 0.36046 |
| Cops2 | 23.04101 | 22.49329 | 21.40379 | 22.23154 | 23.10199 | 22.62332 | 23.51908 | 22.76843 |
| Igf2bp2 | 0.232544 | 0.263112 | 0.452491 | 0.133925 | 0.125723 | 0.114691 | 0.325199 | 0.141704 |
| RGD131151 | 1.732586 | 1.081567 | 1.122439 | 2.036926 | 1.776519 | 1.440557 | 2.038599 | 2.197607 |
| Adcy1 | 3.010584 | 3.33752 | 3.134114 | 1.653272 | 1.381037 | 1.706462 | 1.786118 | 1.5768 |
| Stxbp5l | 0.015999 | 0 | 0.017176 | 0 | 0.0346 | 0.014028 | 0 | 0 |
| AABR07004 | 0.094952 | 0.107434 | 0.101937 | 0 | 0.10267 | 0 | 0.159342 | 0.04208 |
| Gabpb1l | 0.631607 | 0.519733 | 0.801355 | 0.582001 | 1.055463 | 0.654483 | 0.706613 | 0.610717 |
| Hadha | 269.9119 | 274.6624 | 267.78 | 223.67 | 218.5959 | 220.2743 | 218.0033 | 212.8172 |
| Dnai1 | 0 | 0.023757 | 0 | 0.019348 | 0 | 0.01841 | 0 | 0 |
| Ak3 | 75.29773 | 77.33333 | 77.37324 | 85.16335 | 80.55961 | 77.07541 | 81.51876 | 77.56284 |
| Msn | 87.45566 | 90.53116 | 89.21641 | 95.04555 | 95.95903 | 96.70547 | 103.4024 | 107.3321 |
| Polr3k | 26.80701 | 27.93633 | 27.32261 | 29.15189 | 29.22074 | 29.02366 | 26.8336 | 26.02099 |
| Arhgap31 | 12.10179 | 13.34806 | 13.2221 | 10.9019 | 10.12205 | 10.68998 | 13.98058 | 13.2056 |
| AABR07042 | 0.259237 | 0.146657 | 0 | 0.238877 | 0 | 1.136497 | 0 | 0 |
| Msmo1 | 4.389788 | 4.069603 | 4.104625 | 5.871798 | 5.481574 | 6.208016 | 3.675404 | 4.894987 |
| Gdf9 | 0 | 0 | 0 | 0 | 0.040794 | 0.066159 | 0.084416 | 0.03344 |
| Rab3d | 3.848596 | 3.662815 | 3.7439 | 4.263278 | 4.191478 | 4.641395 | 5.067286 | 5.012128 |
| Map3k9 | 0.249152 | 0.182408 | 0.37762 | 0.229584 | 0.285252 | 0.167055 | 0.295137 | 0.272797 |
| Rn50_15_( | 2.032313 | 3.142604 | 2.145454 | 2.372082 | 2.160885 | 2.791696 | 2.046295 | 2.101563 |
| 43350 | 53.87796 | 56.56117 | 50.75205 | 56.47067 | 61.56072 | 56.57465 | 60.75958 | 58.36687 |
| AABR07017 | 0 | 0 | 0 | 0 | 0.167028 | 0 | 0 | 0 |
| Fry | 29.04291 | 29.50998 | 29.31543 | 24.40014 | 24.20293 | 23.86341 | 29.63289 | 30.22573 |
| Dnajc14 | 13.0023 | 10.91758 | 8.616544 | 8.233931 | 13.9242 | 11.61937 | 16.44202 | 13.02642 |
| AC114460. | 0 | 0.404895 | 0.12806 | 0 | 0 | 0.104589 | 0 | 0 |
| Ffar3 | 0 | 0 | 0 | 0 | 0 | 0.053954 | 0 | 0.109082 |

| | | | | | | | | |
|---|---|---|---|---|---|---|---|---|
| Shb | 14.67358 | 16.1697 | 17.02223 | 14.67459 | 13.83605 | 15.42678 | 19.25592 | 20.12529 |
| Cxadr | 10.83657 | 10.86609 | 9.892167 | 6.816428 | 8.805608 | 7.055014 | 8.421092 | 9.173625 |
| Fam83e | 0 | 0 | 0 | 0 | 0 | 0 | 0 | 0 |
| AABR07057 | 0.321263 | 0.282718 | 0.229931 | 0.592063 | 0.733352 | 0.250386 | 0.55909 | 0.537864 |
| Trbc2 | 4.289018 | 6.432811 | 5.032864 | 4.871269 | 3.343425 | 4.11044 | 3.905644 | 5.039291 |
| Kcnt2 | 0.379463 | 0.429345 | 0.381917 | 0.502637 | 0.35902 | 0.436687 | 0.490859 | 0.493995 |
| AABR07044 | 0 | 0 | 0.56786 | 0 | 0 | 0 | 0.591764 | 0 |
| Nxnl1 | 0.320771 | 0.272203 | 0.258276 | 0.221684 | 0.260134 | 0.140626 | 0.179432 | 0.142158 |
| Lamc1 | 140.5529 | 148.4152 | 147.6237 | 134.3413 | 129.4886 | 134.799 | 163.0051 | 167.1439 |
| RT1-A1 | 20.73439 | 18.51315 | 25.95778 | 31.74147 | 28.61568 | 26.74715 | 42.35438 | 36.57969 |
| Hipk1 | 14.76148 | 14.41226 | 14.18813 | 12.83572 | 12.66146 | 12.43757 | 15.49607 | 16.3635 |
| Rragd | 22.60843 | 24.26646 | 23.06386 | 17.62259 | 18.2071 | 18.83671 | 18.75684 | 17.46575 |
| Nckap5 | 0.44523 | 0.484382 | 0.514751 | 0.370814 | 0.379582 | 0.390378 | 0.363999 | 0.538821 |
| Arl5c | 0.499516 | 0.329688 | 0.759705 | 0.498641 | 0.40509 | 0.72996 | 0.884824 | 0.590328 |
| Icoslg | 8.277255 | 8.403134 | 7.181967 | 6.721657 | 6.60017 | 6.097629 | 7.019166 | 7.922796 |
| Ptprj | 3.839978 | 3.368526 | 2.900991 | 3.599546 | 3.342194 | 3.749309 | 4.985478 | 4.807008 |
| Slc4a5 | 0 | 0 | 0 | 0.013806 | 0 | 0.026274 | 0.050287 | 0.026561 |
| Ccdc102a | 5.216092 | 5.747132 | 6.651301 | 5.792895 | 4.925838 | 4.51356 | 6.625495 | 6.177835 |
| Rbm26 | 7.744876 | 8.521127 | 7.801704 | 8.248795 | 7.762654 | 8.444297 | 9.128799 | 10.46416 |
| Man1c1 | 14.59089 | 16.76552 | 15.52898 | 15.89474 | 13.81502 | 14.62723 | 18.18436 | 19.17558 |
| Paqr3 | 2.707124 | 2.552486 | 2.482439 | 3.118135 | 2.683242 | 2.670309 | 2.965514 | 2.324475 |
| Chrnb2 | 0.023824 | 0.080868 | 0.025577 | 0.021953 | 0.025761 | 0.020889 | 0.053307 | 0.042233 |
| AABR07067 | 0 | 0 | 0 | 0 | 0.347377 | 0.281684 | 0 | 0.284752 |
| Ext1 | 11.35867 | 10.38163 | 10.965 | 9.270792 | 10.68069 | 9.771888 | 11.75109 | 11.00146 |
| Rn50_X_06 | 0.08007 | 0 | 0 | 0 | 0 | 0 | 0 | 0.07097 |
| AABR07025 | 5.405514 | 6.689468 | 5.380018 | 5.032854 | 4.201025 | 3.949633 | 6.614398 | 5.889154 |
| Tsc2 | 7.953028 | 8.26519 | 8.130562 | 8.164471 | 8.379251 | 8.5562 | 8.876778 | 9.125344 |
| Gper1 | 1.801486 | 1.621372 | 2.461468 | 1.622271 | 1.062502 | 1.759038 | 1.786397 | 1.596747 |
| Mon1b | 7.944458 | 8.81253 | 9.086327 | 7.440121 | 7.01816 | 7.443747 | 9.672134 | 9.204664 |
| Trnt1 | 21.77989 | 21.36608 | 18.88263 | 21.28432 | 19.88407 | 21.4708 | 19.94091 | 18.61589 |
| Pianp | 0.41233 | 0.408216 | 0.38733 | 0.664905 | 0.473712 | 0.587488 | 0.374803 | 0.708096 |
| Lsm7 | 45.17634 | 46.86544 | 44.00684 | 45.68227 | 45.48366 | 39.2343 | 43.81845 | 40.70781 |
| AABR07061 | 5.236592 | 7.617803 | 6.424933 | 6.893305 | 7.280037 | 4.919409 | 4.184619 | 6.962178 |
| Sphk1 | 1.101103 | 0.886468 | 0.81838 | 2.517046 | 2.060666 | 1.875197 | 2.297902 | 2.646359 |
| Triap1 | 2.493615 | 2.821408 | 35.04509 | 34.8843 | 3.186548 | 20.07516 | 32.96973 | 19.69101 |
| AABR07071 | 0.405937 | 0.459299 | 0 | 0.561083 | 0 | 0.889816 | 0.908289 | 0.359802 |
| Adap2 | 6.724208 | 7.03491 | 5.092762 | 6.450727 | 5.776791 | 5.815027 | 8.29563 | 7.919452 |
| Wdr73 | 2.422746 | 2.584582 | 2.415189 | 2.678958 | 2.844224 | 2.852585 | 2.826623 | 2.945006 |
| Hcn4 | 8.000085 | 8.396732 | 8.395028 | 10.15257 | 9.707303 | 10.38443 | 11.72118 | 11.74242 |
| AABR07053 | 0 | 0 | 0 | 0.297859 | 0 | 0 | 0 | 0.573019 |
| Gchfr | 8.049912 | 8.544709 | 10.78034 | 5.429434 | 5.563543 | 5.384583 | 6.313403 | 5.148996 |
| Gpc2 | 0.722012 | 0.726153 | 0.581344 | 1.03492 | 0.585526 | 0.738571 | 0.718004 | 0.622179 |
| Pcbp3 | 10.79473 | 12.01161 | 11.0135 | 13.1685 | 14.37638 | 15.39433 | 14.30357 | 15.42627 |
| Ffar4 | 0 | 0 | 0.121509 | 0.069529 | 0.081589 | 0.099239 | 0.084416 | 0.167199 |
| Rpl21 | 400.0626 | 403.3387 | 389.6861 | 401.3102 | 390.6133 | 347.3531 | 414.0953 | 421.4784 |
| Gpr27 | 0.785027 | 0.62698 | 1.735128 | 2.638178 | 1.697677 | 2.550805 | 2.996394 | 1.964636 |
| Miox | 0 | 0 | 0 | 0 | 0 | 0 | 0.021811 | 0.03456 |
| Pnpla2 | 0.673442 | 0.420396 | 0.648192 | 0.770338 | 0.853732 | 0.631197 | 0.701457 | 0.658654 |
| Rn50_6_09 | 0.096884 | 0.16443 | 0.156017 | 0.044637 | 0.10476 | 0.084948 | 0.10839 | 0.300557 |
| Manba | 3.358249 | 3.284487 | 3.070612 | 3.933679 | 3.815865 | 3.655691 | 3.995856 | 3.783792 |
| Ncoa2 | 8.037062 | 8.797174 | 8.87117 | 6.385476 | 6.231309 | 6.430951 | 7.632791 | 8.432287 |
| Tsc22d1 | 56.96676 | 55.7476 | 54.38613 | 104.1374 | 104.2361 | 98.97285 | 107.8856 | 108.696 |
| Arrb1 | 5.359535 | 5.238132 | 5.609442 | 4.531476 | 4.465825 | 4.83399 | 6.275393 | 6.350923 |
| Rtn4ip1 | 19.57163 | 19.74986 | 21.29305 | 19.8879 | 19.47935 | 18.92401 | 16.92576 | 17.81239 |

| | | | | | | | | |
|---|---|---|---|---|---|---|---|---|
| Fcar | 0.030009 | 0.101862 | 0.128867 | 0.110609 | 0.064897 | 0.13156 | 0.067146 | 0.053197 |
| Mettl16 | 7.854888 | 8.419677 | 8.087525 | 7.788225 | 7.847682 | 8.900966 | 10.43218 | 8.875759 |
| AC119336. | 0.494018 | 1.117917 | 1.59108 | 1.365655 | 3.205048 | 0.866311 | 2.210742 | 0.875746 |
| LOC680385 | 2.909218 | 3.635546 | 3.169847 | 3.841046 | 4.131659 | 3.502598 | 3.643325 | 3.386797 |
| Gpat4 | 11.89299 | 10.71595 | 10.81325 | 10.37404 | 10.49368 | 10.42779 | 11.474 | 11.05954 |
| AABR07027 | 0 | 0 | 0.390404 | 0 | 0 | 0 | 0 | 0 |
| Aunip | 0.039883 | 0.135376 | 0.085633 | 0.03675 | 0 | 0.209815 | 0.133857 | 0.10605 |
| LOC100911 | 0 | 0.622043 | 0.147554 | 0.759892 | 0.445847 | 1.084594 | 0.615062 | 0.243646 |
| Anxa5 | 89.90105 | 88.35598 | 89.88348 | 133.8141 | 132.2171 | 128.1321 | 131.6731 | 130.2795 |
| AABR07004 | 0 | 0 | 0.484639 | 0 | 0 | 0 | 0 | 0.400125 |
| LOC103689 | 0.825961 | 1.074716 | 3.768568 | 2.321326 | 9.556165 | 4.671111 | 5.7291 | 7.211088 |
| Klk1c8 | 0 | 0 | 0 | 0 | 0 | 0 | 0 | 0 |
| Cldn12 | 4.304873 | 4.359842 | 4.249893 | 4.479955 | 3.580613 | 3.444576 | 4.580348 | 4.122474 |
| Agmat | 0.105719 | 0.159487 | 0.113496 | 0.032472 | 0.076208 | 0.30898 | 0.118273 | 0.156173 |
| Aldoc | 1.532479 | 0.9304 | 1.203815 | 2.824238 | 2.101621 | 2.359631 | 2.550796 | 2.153422 |
| Mfsd1 | 11.80824 | 12.05169 | 9.779321 | 13.01258 | 13.26317 | 12.02271 | 14.80119 | 15.59254 |
| AABR07030 | 0 | 0.12267 | 0 | 0 | 0.351693 | 0.095061 | 0 | 0 |
| Clec2e | 0.053599 | 0.121289 | 0.086312 | 0.049389 | 0 | 0.046995 | 0.089946 | 0.023754 |
| Mpp2 | 0.679164 | 0.821439 | 0.779411 | 1.186906 | 1.164864 | 1.067779 | 1.231431 | 1.203954 |
| Ldlrad1 | 0.112615 | 0.382255 | 0.120899 | 0.337253 | 0.365307 | 0.271537 | 0.88192 | 0.374311 |
| AABR07061 | 0 | 0 | 0 | 0 | 0 | 0 | 0 | 0.151681 |
| Mtus1 | 176.5936 | 175.3486 | 161.3453 | 172.1066 | 167.8578 | 163.2692 | 184.9757 | 183.5809 |
| Slpi | 10.828 | 9.551918 | 9.457261 | 9.582982 | 9.723726 | 9.118531 | 5.475202 | 3.687135 |
| Ets1 | 29.75833 | 30.2313 | 28.78513 | 25.56045 | 24.67035 | 25.43504 | 28.37822 | 28.92258 |
| Pogk | 4.579359 | 4.547915 | 4.471489 | 4.126845 | 4.455224 | 4.398052 | 6.400848 | 6.192572 |
| Dact1 | 2.010783 | 1.718431 | 1.515686 | 3.548025 | 3.030045 | 3.038459 | 3.470088 | 3.033629 |
| Slc5a4b | 0 | 0 | 0 | 0 | 0 | 0 | 0 | 0 |
| C1qtnf2 | 5.684459 | 7.162572 | 6.195093 | 9.483963 | 8.56788 | 9.892759 | 8.575716 | 7.557627 |
| Extl1 | 1.904677 | 1.92518 | 2.249272 | 1.872091 | 1.83982 | 2.115362 | 2.500219 | 2.419768 |
| Gphn | 6.955978 | 6.562527 | 6.448358 | 6.752015 | 5.668947 | 5.791342 | 7.920591 | 7.391196 |
| Srd5a3 | 22.20225 | 21.50493 | 22.1502 | 20.25185 | 20.06644 | 19.86023 | 23.01988 | 25.4932 |
| AC113837. | 8.624975 | 11.15286 | 7.275291 | 4.541472 | 6.661472 | 20.52648 | 6.203082 | 8.73685 |
| Myadml2 | 21.95575 | 23.05969 | 24.26112 | 21.14962 | 21.02923 | 22.77404 | 20.1396 | 22.73713 |
| AABR07032 | 0 | 0 | 0 | 0 | 0 | 0.956552 | 1.830771 | 0 |
| Ptms | 188.8123 | 207.1649 | 229.6235 | 198.3646 | 198.5277 | 217.528 | 261.6197 | 257.4862 |
| Psmb2 | 49.40405 | 48.0565 | 46.4881 | 48.30772 | 52.5872 | 50.74486 | 50.43318 | 46.41452 |
| Ppp1r42 | 0.344189 | 0.055633 | 0.052787 | 0.226541 | 0.106333 | 0.043112 | 0.110018 | 0.087163 |
| Agtr2 | 0.017781 | 0 | 0.019089 | 0 | 0 | 0.015591 | 0 | 0 |
| 43161 | 10.9208 | 12.63096 | 11.46364 | 11.97632 | 11.72104 | 11.3013 | 10.89035 | 10.85077 |
| Copb2 | 20.61819 | 19.99202 | 20.60311 | 23.46917 | 21.75415 | 22.97809 | 23.14653 | 22.40628 |
| AABR07060 | 0 | 0 | 0 | 0 | 0.245119 | 0 | 0 | 0 |
| Tmem150c | 1.229659 | 1.49068 | 1.2824 | 0.712223 | 1.101679 | 0.985752 | 1.041593 | 1.230039 |
| Recql | 8.636134 | 8.64174 | 8.172802 | 10.39581 | 10.79552 | 9.848198 | 11.39301 | 10.42004 |
| Gar1 | 4.263695 | 4.078616 | 3.828328 | 4.964608 | 5.490414 | 3.704426 | 5.680674 | 4.569305 |
| AABR07014 | 0 | 0.598481 | 0 | 0 | 0 | 0 | 0 | 0 |
| Cnst | 6.4834 | 8.035371 | 7.131675 | 5.459498 | 6.557426 | 6.156914 | 7.097114 | 7.143415 |
| LOC24906 | 0.210667 | 0.374566 | 0.193856 | 0.221853 | 0.3905 | 0.395815 | 0.606048 | 0.26675 |
| AABR07052 | 0.480421 | 0.543574 | 1.547289 | 0.442689 | 2.597363 | 0.421234 | 0 | 1.277464 |
| RGD131193 | 0.155851 | 0.058779 | 0.055772 | 0.09574 | 0.056173 | 0.04555 | 0 | 0 |
| A2ml1 | 0 | 0 | 0.012844 | 0.022048 | 0.012936 | 0.02098 | 0 | 0 |
| Vom2r80 | 0 | 0 | 0 | 0.018856 | 0 | 0 | 0.022894 | 0 |
| AABR07001 | 0.818594 | 1.416542 | 0.775423 | 1.020526 | 1.041334 | 1.287717 | 1.158226 | 1.08834 |
| Abracl | 14.32964 | 14.94315 | 11.83913 | 12.53486 | 13.06673 | 13.72224 | 12.04199 | 17.50056 |
| Crybb1 | 10.69543 | 13.83014 | 13.04798 | 4.415738 | 5.707309 | 5.724087 | 5.827387 | 6.771349 |

| | | | | | | | | |
|---|---|---|---|---|---|---|---|---|
| Abhd18 | 10.77981 | 10.44412 | 9.138237 | 10.94856 | 10.05011 | 9.787809 | 10.63064 | 9.398975 |
| Ncf1 | 64.93261 | 63.2295 | 64.71484 | 48.17472 | 54.65944 | 52.72953 | 49.95247 | 53.0272 |
| AABR07042 | 16.06997 | 12.53959 | 12.49292 | 13.78661 | 14.08073 | 12.14669 | 17.04845 | 13.87524 |
| AABR07057 | 0.392254 | 0.591756 | 0.56148 | 0.783135 | 0.353449 | 0.745179 | 0.877673 | 0.40562 |
| AABR07018 | 0 | 0 | 0.559385 | 0 | 0 | 0 | 0 | 0 |
| Nadk | 10.19029 | 11.15124 | 10.13984 | 11.88459 | 11.87379 | 11.36193 | 13.93577 | 13.72349 |
| Cyp2e1 | 12.11322 | 10.54773 | 11.64519 | 3.685267 | 3.92002 | 4.036438 | 3.669589 | 3.18781 |
| Fitm2 | 34.60562 | 36.46026 | 35.77863 | 20.97022 | 20.23139 | 20.57101 | 29.54169 | 28.20846 |
| Ankrd33 | 0 | 0.055014 | 0 | 0.089607 | 0.052574 | 0.085264 | 0 | 0.129288 |
| Glra4 | 0.074542 | 0 | 0.080026 | 0 | 0 | 0.098038 | 0.083395 | 0.033035 |
| AABR07005 | 0 | 0.637092 | 0 | 0 | 0 | 0 | 0 | 0 |
| Exo1 | 0.020855 | 0.047192 | 0.089555 | 0.17295 | 0.0451 | 0.054856 | 0.046662 | 0.073938 |
| Fermt2 | 45.80604 | 43.1255 | 43.83317 | 45.37968 | 43.92132 | 46.24032 | 53.21606 | 53.13109 |
| Glod5 | 0.224746 | 0.423817 | 0.482559 | 0.759348 | 0.405025 | 1.050975 | 0.670497 | 1.128822 |
| Fam71b | 0.048286 | 0.027317 | 0.025919 | 0.022247 | 0.078316 | 0.042337 | 0 | 0.021399 |
| Dyrk2 | 4.99335 | 4.756583 | 3.83328 | 4.32206 | 4.535756 | 4.539136 | 5.062619 | 5.23943 |
| Myo10 | 16.66536 | 17.42024 | 17.42353 | 11.7639 | 11.77087 | 11.75009 | 13.79519 | 13.6601 |
| Arhgap5 | 8.473954 | 8.313113 | 7.244409 | 7.764238 | 8.618441 | 8.039501 | 8.528232 | 8.509505 |
| Nf1 | 2.694841 | 2.484681 | 2.480666 | 2.551874 | 2.510907 | 2.694642 | 3.008454 | 3.04924 |
| 43357 | 0 | 0 | 0.126333 | 0.036145 | 0.042414 | 0 | 0 | 0.034767 |
| AABR07040 | 23.8452 | 17.98648 | 14.05454 | 24.5574 | 26.28902 | 22.95724 | 17.78463 | 17.81986 |
| Ptpdc1 | 1.891442 | 1.81397 | 1.682484 | 1.676494 | 2.084145 | 1.563651 | 2.700497 | 1.85211 |
| Prkab2 | 13.14529 | 11.6029 | 11.76851 | 10.18614 | 10.90557 | 10.86526 | 10.69867 | 10.57477 |
| Tmem55a | 8.906882 | 8.596479 | 8.056263 | 9.542919 | 10.74311 | 10.8432 | 8.866161 | 10.19462 |
| Hmg1l1 | 0.290691 | 0.375889 | 0.356658 | 0.191329 | 0.269417 | 0.61899 | 0.511048 | 0.404885 |
| Il31 | 0 | 0 | 0 | 0.056108 | 0 | 0 | 0 | 0 |
| Ptpro | 0.908862 | 0.853299 | 0.820021 | 0.98003 | 1.087286 | 0.991875 | 1.157415 | 1.139795 |
| Nxpe4 | 0.317947 | 0.215845 | 0.273069 | 0.351571 | 0.378171 | 0.362409 | 0.142282 | 0.22545 |
| LOC100361 | 0 | 0 | 0 | 0 | 0 | 0.090561 | 0 | 0 |
| Fbxo48 | 0.051541 | 0 | 0.165999 | 0.094986 | 0.167192 | 0.225957 | 0.172986 | 0.274101 |
| Ttc9c | 7.417417 | 8.274255 | 7.794837 | 8.952701 | 8.867566 | 8.450097 | 8.385935 | 9.468099 |
| AABR07043 | 0 | 0.098421 | 0 | 0 | 0 | 0 | 0 | 0 |
| Sprr1a | 0 | 0 | 0 | 0 | 0 | 0 | 0 | 0.057161 |
| Lcmt1 | 7.436581 | 8.28267 | 7.235178 | 7.56632 | 8.124828 | 8.490105 | 7.583074 | 7.449667 |
| Aqp2 | 0 | 0 | 0 | 0 | 0 | 0 | 0 | 0 |
| AC127784. | 0.418927 | 0.473997 | 0.449745 | 0 | 0 | 0.367316 | 0 | 0.742632 |
| Dxo | 4.187737 | 2.434319 | 4.372065 | 4.000443 | 3.655745 | 3.537066 | 3.99734 | 4.631236 |
| Gpr45 | 0 | 0.017075 | 0 | 0 | 0.016318 | 0 | 0 | 0 |
| Hint1 | 219.8481 | 202.2519 | 205.3392 | 213.377 | 212.2864 | 199.0665 | 216.0682 | 204.7746 |
| Rn50_X_0( | 0.155388 | 0.175815 | 0.111213 | 0.238641 | 0.560064 | 0.27249 | 0.173842 | 0.275457 |
| Tmem247 | 0 | 0 | 0 | 0 | 0 | 0 | 0 | 0 |
| LOC691141 | 1.086764 | 1.776121 | 1.037076 | 1.223946 | 1.175102 | 1.199917 | 0.765518 | 1.141633 |
| Fkbp11 | 19.731 | 13.70889 | 12.32289 | 16.93699 | 12.20872 | 11.37995 | 18.67491 | 18.25327 |
| Stk3 | 3.828865 | 4.4615 | 3.55837 | 3.808996 | 3.192612 | 3.975136 | 3.558981 | 3.66386 |
| AABR07034 | 0 | 0.034649 | 0 | 0.018812 | 0 | 0 | 0 | 0 |
| Rhog | 18.43627 | 17.08934 | 19.93189 | 21.77373 | 21.80669 | 21.47735 | 22.12661 | 23.09218 |
| LOC498933 | 0.310265 | 0.585085 | 0.40711 | 0.730627 | 0.708247 | 0.695216 | 0.848494 | 1.130571 |
| LOC100360 | 130.9653 | 133.2259 | 123.7016 | 100.6901 | 96.11644 | 100.2334 | 54.74675 | 57.95107 |
| Znf454 | 0.06168 | 0.097702 | 0.211894 | 0.136405 | 0.20008 | 0.162242 | 0.069004 | 0.196811 |
| Il15ra | 2.207753 | 2.441835 | 2.050591 | 1.66863 | 2.06534 | 1.696509 | 2.442184 | 2.264659 |
| Fgl2 | 8.040996 | 6.751172 | 6.802306 | 13.77165 | 14.4859 | 15.65776 | 16.16403 | 15.19868 |
| AABR07072 | 0 | 0 | 0 | 0.103326 | 0 | 0 | 0 | 0 |
| Gpr4 | 7.380432 | 7.833669 | 8.338398 | 6.574085 | 6.30828 | 5.762422 | 12.85717 | 9.812465 |
| Rps15al4 | 1.926885 | 1.667196 | 0.365053 | 3.342208 | 4.902382 | 0.894438 | 2.282519 | 4.42043 |

| | | | | | | | | |
|---|---|---|---|---|---|---|---|---|
| Trim69 | 0.189236 | 0.178427 | 0.237017 | 0.368123 | 0.318296 | 0.202794 | 0.36461 | 0.372733 |
| Gxylt2 | 1.412115 | 2.973574 | 2.231882 | 5.096399 | 4.750353 | 4.780609 | 7.240801 | 7.266393 |
| Zgpat | 7.852235 | 7.863925 | 7.746373 | 8.751075 | 8.404456 | 9.001472 | 10.14993 | 10.41623 |
| Ccpg1os | 1.136845 | 1.688251 | 0.762797 | 1.505864 | 1.382911 | 2.055872 | 1.589815 | 1.826351 |
| Shisa4 | 46.19485 | 55.15996 | 52.1485 | 56.21409 | 53.28617 | 54.10798 | 59.17643 | 55.63491 |
| Spc25 | 0.208962 | 0.331003 | 0.224334 | 0.385101 | 0.406706 | 0.329793 | 0.187022 | 0.555641 |
| LOC684471 | 0.111536 | 0.189296 | 0.11974 | 0.102776 | 0.060301 | 0.048897 | 0.124781 | 0 |
| Slc25a44 | 4.6815 | 5.599577 | 6.246462 | 4.529509 | 4.33889 | 5.101609 | 5.349685 | 5.483197 |
| Bpnt1 | 19.93249 | 19.33945 | 18.236 | 19.27191 | 18.99853 | 20.99081 | 17.54868 | 18.30233 |
| Mpp4 | 0.227348 | 0.171489 | 0.081358 | 0.023277 | 0.136571 | 0.17719 | 0.141304 | 0.17912 |
| Armc9 | 2.431992 | 2.398257 | 2.251601 | 2.199866 | 2.774431 | 2.660575 | 2.870574 | 2.570894 |
| LOC290595 | 0.223786 | 0 | 0.240249 | 0.41242 | 0.241977 | 0 | 0.751085 | 0 |
| Gpatch3 | 1.587737 | 1.297436 | 1.578275 | 1.73397 | 1.716797 | 1.211668 | 1.710501 | 1.615778 |
| LOC100360 | 0 | 0 | 0 | 0.272617 | 0 | 0 | 0 | 0 |
| Elovl7 | 0.642168 | 0.330265 | 0.689409 | 0.753115 | 0.946865 | 0.767801 | 1.567483 | 1.138372 |
| Zranb1 | 7.255769 | 6.966399 | 6.320648 | 6.342059 | 6.523021 | 7.579707 | 7.073762 | 6.853767 |
| AC133613. | 0.035769 | 0 | 0 | 0.03296 | 0 | 0.031362 | 0 | 0.031704 |
| Tmem179 | 39.93011 | 39.49147 | 37.91676 | 43.56074 | 41.54523 | 42.40766 | 46.62811 | 47.96425 |
| G2e3 | 0.972873 | 0.885393 | 0.73792 | 1.013394 | 1.143427 | 1.066269 | 1.017424 | 0.918543 |
| Tgif2-ps1 | 0.513391 | 0.33193 | 0.551158 | 0.337907 | 0.475819 | 0.514448 | 0.328205 | 0.325032 |
| Hmga1 | 107.8027 | 118.682 | 131.0022 | 108.4206 | 94.37085 | 115.2113 | 118.4351 | 114.4605 |
| Ankrd28 | 6.889873 | 8.02389 | 6.684901 | 7.982421 | 7.823975 | 7.835668 | 6.724417 | 7.576055 |
| Ttc36 | 0 | 0 | 0 | 0.061863 | 0 | 0 | 0 | 0.059506 |
| Rgs3 | 12.36836 | 13.27831 | 12.71216 | 12.78514 | 12.0217 | 13.26184 | 16.36528 | 16.3973 |
| Rn60_20_0 | 0.868423 | 0.687806 | 1.118769 | 1.520414 | 1.220718 | 1.066008 | 2.137417 | 2.540098 |
| Uqcr11 | 835.9993 | 891.8927 | 886.6534 | 819.3534 | 817.794 | 818.2359 | 791.8156 | 782.6511 |
| LOC103690 | 0 | 0 | 0 | 0 | 0 | 0 | 0 | 0 |
| LOC361346 | 2.83392 | 2.52682 | 2.033775 | 2.668115 | 2.331515 | 2.498288 | 3.911388 | 3.644905 |
| Srrm4 | 3.247499 | 3.215093 | 4.108969 | 2.965724 | 2.414138 | 2.593177 | 3.503402 | 3.161122 |
| AC127920. | 0.498723 | 0 | 0 | 0 | 0 | 0 | 0 | 0 |
| Rps29 | 1004.739 | 1149.442 | 1146.85 | 1041.477 | 1027.374 | 962.0966 | 948.4953 | 944.1169 |
| Gpsm1 | 76.3759 | 78.69808 | 83.50081 | 71.79607 | 72.08992 | 74.39958 | 88.23675 | 88.06149 |
| Icam2 | 22.20653 | 20.17007 | 18.10695 | 16.28499 | 15.78617 | 16.60737 | 19.04109 | 20.05734 |
| Ttc4 | 4.888855 | 5.284696 | 4.752577 | 5.143375 | 4.925506 | 4.68033 | 5.010058 | 5.049754 |
| Scimp | 0.909131 | 0.411455 | 0.488005 | 0.418864 | 1.032181 | 0.717414 | 0.711966 | 0.604356 |
| AABR07054 | 0 | 0 | 0 | 0.129713 | 0 | 0 | 0 | 0 |
| Abhd3 | 0.410821 | 0.495812 | 0.352834 | 0.479503 | 0.4146 | 0.744429 | 0.520889 | 0.63116 |
| Nifk | 14.33361 | 16.37958 | 13.50771 | 15.5464 | 13.99136 | 12.41101 | 13.63643 | 15.55599 |
| AABR07002 | 0 | 0 | 0 | 0.127654 | 0 | 0 | 0 | 0 |
| Zfp384 | 12.13206 | 13.18992 | 11.36325 | 11.90168 | 12.4043 | 13.5862 | 13.64206 | 14.11821 |
| AC183952. | 0 | 0 | 0.883008 | 0 | 0.296453 | 0.24039 | 0 | 1.701056 |
| Henmt1 | 0.302443 | 0.293315 | 0.185538 | 0.079626 | 0.233591 | 0.189416 | 0.193349 | 0.306366 |
| Cyb561d1 | 1.47552 | 1.362842 | 1.099147 | 2.386297 | 1.921063 | 1.874599 | 2.122389 | 1.948396 |
| Ddx39b | 92.20458 | 91.96452 | 96.2837 | 102.8958 | 105.1814 | 101.9238 | 118.5598 | 115.7609 |
| Kiss1r | 0 | 0.088103 | 0 | 0 | 0 | 0 | 0 | 0 |
| Abcg5 | 0.042402 | 0 | 0.02276 | 0.019536 | 0.045848 | 0 | 0.094874 | 0.037583 |
| Vkorc1l1 | 0.098618 | 0.223162 | 0.211744 | 0.181744 | 0 | 0 | 0 | 0.174819 |
| Gnai1 | 4.102897 | 4.520071 | 4.327446 | 3.515349 | 2.763023 | 3.439642 | 3.22115 | 4.147002 |
| AABR07072 | 0 | 0 | 0.248204 | 0.319557 | 0.249989 | 0 | 0.258652 | 0 |
| Trip10 | 41.82303 | 40.84705 | 43.59012 | 38.40446 | 37.59132 | 38.37667 | 51.27377 | 51.48588 |
| LOC103692 | 0 | 0 | 0 | 0 | 0 | 0 | 0 | 0.103143 |
| Ntrk1 | 0 | 0.022588 | 0 | 0.018396 | 0 | 0.017505 | 0 | 0 |
| Gemin4 | 1.762538 | 1.766816 | 1.709616 | 1.95178 | 1.905806 | 2.033414 | 1.57402 | 1.466299 |
| Surf1 | 1.759751 | 1.568726 | 1.946454 | 2.06378 | 2.133435 | 2.431317 | 1.730097 | 2.079673 |

| | | | | | | | | |
|---|---|---|---|---|---|---|---|---|
| Ltbp4 | 130.4399 | 139.2017 | 153.8184 | 160.9752 | 150.0717 | 164.8602 | 196.738 | 195.1131 |
| Fut1 | 0.442963 | 0.414028 | 0.558253 | 0.301693 | 0.43732 | 0.371504 | 0.409382 | 0.580395 |
| Il13ra2 | 0.105683 | 0.179363 | 0.028364 | 0.219111 | 0.17141 | 0.046331 | 0.118233 | 0.070254 |
| Slc26a1 | 1.058325 | 0.926024 | 0.954391 | 0.57212 | 0.869707 | 0.767097 | 0.741978 | 0.888017 |
| Smco1 | 0.239114 | 0.865747 | 1.180838 | 0.088134 | 0.05171 | 0.125793 | 0.642024 | 0.508652 |
| Brox | 14.48686 | 13.03845 | 13.19612 | 14.62041 | 13.96915 | 13.7606 | 15.04959 | 14.11891 |
| Slc37a3 | 6.552435 | 6.726938 | 6.306094 | 6.992016 | 6.949916 | 6.794029 | 7.610214 | 7.690916 |
| Plaur | 5.84204 | 6.069176 | 5.587606 | 6.313037 | 6.20206 | 6.612431 | 7.724145 | 10.77984 |
| LOC10255[illegible] | 0 | 0 | 0 | 0 | 0 | 0 | 0 | 0 |
| Hook3 | 4.03193 | 4.395039 | 3.853451 | 3.737919 | 3.641917 | 3.341195 | 4.318212 | 3.83519 |
| Anpep | 3.248069 | 3.309196 | 3.376561 | 5.146279 | 4.449706 | 4.188102 | 4.603903 | 5.197697 |
| AABR0707[illegible] | 0 | 0.630315 | 0 | 0 | 0 | 0 | 0.623241 | 0 |
| Pdzrn3 | 11.9668 | 11.83838 | 12.55981 | 11.39098 | 10.94142 | 10.95064 | 12.51821 | 12.88853 |
| Asxl3 | 0.01574 | 0.026713 | 0.025346 | 0.021755 | 0.00851 | 0.0069 | 0.017609 | 0.013951 |
| AABR0701[illegible] | 0 | 0 | 0 | 0 | 0 | 0 | 0.133755 | 0 |
| Kcnh6 | 0 | 0.017519 | 0 | 0 | 0 | 0 | 0 | 0.013724 |
| Heca | 8.946118 | 8.132295 | 7.52859 | 7.730164 | 6.685094 | 7.240582 | 9.812993 | 9.149295 |
| Psmg4 | 31.09227 | 35.48803 | 30.1587 | 26.63975 | 29.98241 | 30.45023 | 29.39404 | 26.10817 |
| Ubap1l | 0.137805 | 0.31184 | 0.443828 | 0.4656 | 0.397351 | 0.322207 | 0.30834 | 0.122143 |
| Gpn2 | 4.149439 | 5.023538 | 4.454688 | 6.194143 | 5.518676 | 4.947995 | 5.895604 | 5.369667 |
| AABR0706[illegible] | 0 | 0 | 0 | 0.371178 | 0 | 0 | 0 | 0 |
| Fgf14 | 0.211153 | 0.079637 | 0.075562 | 0.324282 | 0.152211 | 0.123426 | 0.314971 | 0.187155 |
| Amigo1 | 8.020717 | 7.607227 | 8.344316 | 7.318011 | 6.623443 | 7.616147 | 8.215988 | 8.439003 |
| AABR0701[illegible] | 0.068632 | 0.077653 | 0 | 0 | 0 | 0.120353 | 0.076782 | 0 |
| RGD73506[illegible] | 39.81992 | 39.30981 | 39.37073 | 34.60461 | 32.03165 | 32.81708 | 39.15033 | 36.07539 |
| Hnrnpll | 10.76214 | 10.57552 | 9.116459 | 10.40594 | 9.66026 | 8.841641 | 12.69994 | 12.15245 |
| Fam114a2 | 16.68727 | 17.30088 | 14.27941 | 15.69835 | 14.72185 | 15.36605 | 15.4273 | 15.53339 |
| Stk4 | 3.054428 | 2.923387 | 2.89208 | 2.869903 | 2.295654 | 2.502511 | 3.193083 | 3.355266 |
| AABR0706[illegible] | 0.503518 | 0 | 0 | 0.463972 | 1.633342 | 0 | 0.563314 | 0.446293 |
| Mdp1 | 5.33331 | 6.909845 | 6.526647 | 5.780191 | 5.826591 | 5.693881 | 5.255616 | 5.951835 |
| Ctsw | 2.632722 | 2.407524 | 2.05204 | 2.160092 | 2.027803 | 2.782696 | 1.412165 | 1.534364 |
| AABR0702[illegible] | 0.477138 | 0.944754 | 0.768358 | 0.54958 | 0.902865 | 0.627533 | 0.400351 | 0.105728 |
| Gng4 | 0 | 0.019652 | 0 | 0 | 0.056341 | 0 | 0 | 0.015395 |
| Mgst1 | 13.11904 | 12.16176 | 13.61071 | 12.95216 | 11.02649 | 12.66273 | 13.50531 | 13.72893 |
| AABR0701[illegible] | 0.166241 | 0 | 0 | 0 | 0 | 0 | 0 | 0 |
| Cebpa | 1.195032 | 1.573782 | 2.229377 | 2.671704 | 2.49961 | 3.452604 | 3.375248 | 3.368656 |
| Cd300lf | 0.753466 | 0.937763 | 1.092007 | 1.70101 | 1.018391 | 1.453408 | 1.68589 | 1.502628 |
| LOC10255[illegible] | 1.286848 | 1.323643 | 1.648396 | 1.293577 | 1.201706 | 1.3591 | 1.537827 | 1.710895 |
| Tmem80 | 17.85552 | 18.32174 | 16.59113 | 17.84316 | 16.11129 | 17.27529 | 17.53063 | 15.88081 |
| Olr1425 | 0 | 0 | 0.059302 | 0 | 0 | 0 | 0 | 0 |
| Mab21l3 | 0.024847 | 0.014057 | 0.026675 | 0.022896 | 0.080601 | 0.098038 | 0.041697 | 0.022023 |
| Faim | 0.226692 | 0.410387 | 0.38939 | 0.125333 | 0.196095 | 0.795056 | 0.355059 | 0.482229 |
| Bcl7c | 31.17525 | 33.17015 | 35.23613 | 29.11827 | 27.90927 | 26.72912 | 33.15441 | 31.72757 |
| Bnipl | 0.031718 | 0 | 0.034051 | 0 | 0 | 0 | 0 | 0 |
| AABR0700[illegible] | 0.151639 | 0.114381 | 0.217059 | 0.139729 | 0.32793 | 0.398871 | 0.50894 | 0.403215 |
| Prr12 | 5.47379 | 6.597249 | 6.830324 | 5.943008 | 5.584193 | 5.954067 | 7.171097 | 6.637677 |
| RGD156125[illegible] | 2.349018 | 1.87991 | 4.674595 | 1.953354 | 2.78776 | 1.255867 | 0.961455 | 2.488306 |
| Ttll11 | 0.171772 | 0.194352 | 0.447849 | 0.203504 | 0.185735 | 0.215157 | 0.384342 | 0.15225 |
| LOC10369[illegible] | 0.038001 | 0.085994 | 0 | 0.035017 | 0 | 0.066639 | 0.085029 | 0.202095 |
| AABR0706[illegible] | 0.045509 | 0 | 0 | 0 | 0.016403 | 0 | 0 | 0 |
| AABR0706[illegible] | 0 | 0 | 0.739713 | 0 | 0 | 0 | 0 | 0.610717 |
| Ltbp2 | 0.905347 | 1.133139 | 1.100968 | 20.63456 | 20.73964 | 21.4188 | 27.33832 | 27.29764 |
| Scamp4 | 9.987471 | 11.43774 | 12.12357 | 12.69967 | 11.61992 | 11.49858 | 16.03012 | 15.71367 |
| Flt1 | 25.78465 | 24.08927 | 23.88142 | 18.41347 | 18.34056 | 17.68577 | 26.58506 | 27.14397 |

| | | | | | | | | |
|---|---|---|---|---|---|---|---|---|
| Tmem140 | 14.2087 | 13.56992 | 10.94839 | 18.54807 | 17.92427 | 18.18495 | 24.74145 | 24.88306 |
| Timm8a2 | 0 | 0 | 0 | 0 | 0 | 0 | 0 | 0.054223 |
| AABR07072 | 0.818218 | 0.462887 | 0.439204 | 0 | 0.442363 | 0.358707 | 0 | 1.450454 |
| Tusc2 | 16.80285 | 17.37221 | 18.25726 | 16.02191 | 15.66739 | 15.15624 | 18.8836 | 16.35774 |
| AABR07054 | 0 | 0.278167 | 0 | 0 | 0 | 0 | 0 | 0 |
| Pstpip1 | 2.172385 | 2.684342 | 2.117387 | 3.239703 | 3.338008 | 4.210499 | 4.956672 | 4.231018 |
| Olr827 | 0 | 0 | 0 | 0 | 0 | 0.046661 | 0.059537 | 0 |
| Pank3 | 4.542246 | 4.668301 | 4.565132 | 4.911627 | 5.562575 | 4.992972 | 4.92364 | 5.46247 |
| Nfatc2ip | 2.106258 | 1.397008 | 1.949312 | 2.320077 | 2.172755 | 1.485906 | 2.085538 | 2.231674 |
| AABR07030 | 1.672622 | 1.455764 | 3.315076 | 3.556742 | 3.199798 | 3.045924 | 2.303083 | 3.649299 |
| Lrrn4 | 0.061175 | 0 | 0.065675 | 0.075161 | 0.022049 | 0.089397 | 0.091253 | 0 |
| Nsun7 | 0.080131 | 0.18133 | 0.043013 | 0.01846 | 0.043323 | 0.105389 | 0.022412 | 0.142049 |
| Ltf | 0 | 0 | 0 | 0 | 0 | 0.01663 | 0 | 0 |
| Nat14 | 2.516598 | 2.145311 | 2.701729 | 2.731909 | 2.497504 | 2.811091 | 2.892594 | 2.444478 |
| Lalba | 0 | 0 | 0 | 0.065295 | 0 | 0 | 0 | 0 |
| Papln | 8.681959 | 9.306216 | 10.95175 | 6.852703 | 6.188455 | 6.049814 | 6.799093 | 6.702969 |
| Aqp6 | 0.30864 | 0.194006 | 0.110448 | 0.1264 | 0.074162 | 0.060137 | 0.076732 | 0.030396 |
| Zfp385a | 6.423907 | 6.792147 | 7.681246 | 8.062601 | 8.670622 | 7.749526 | 11.35016 | 9.974017 |
| Trappc11 | 8.387463 | 8.24238 | 7.858453 | 8.398899 | 8.625293 | 8.269544 | 8.149883 | 8.536362 |
| Rbm19 | 2.023922 | 2.027867 | 2.670199 | 1.876199 | 1.92477 | 1.817337 | 2.427956 | 2.085681 |
| Aldh18a1 | 0.137363 | 0.259033 | 0.032771 | 0.070319 | 0.04951 | 0.026764 | 0.03415 | 0.040584 |
| AC141152. | 1.256782 | 0.473997 | 0.449745 | 1.5441 | 0 | 0.367316 | 0.468677 | 0.742632 |
| AC109547. | 0 | 0 | 0.497506 | 0 | 1.503253 | 0 | 0 | 0 |
| AABR07012 | 0 | 0 | 0 | 0 | 0 | 0 | 0.051799 | 0 |
| Ppdpf | 30.70547 | 27.82935 | 36.62698 | 32.24187 | 32.08609 | 28.3835 | 35.86092 | 35.30316 |
| Gpd1 | 41.18504 | 41.93491 | 43.62733 | 39.21092 | 37.45251 | 41.06876 | 42.38257 | 42.97102 |
| Slamf1 | 0.608464 | 0.53785 | 0.632812 | 0.227774 | 0.390642 | 0.366782 | 0.233998 | 0.320216 |
| Mettl21a | 1.999874 | 1.984269 | 2.213053 | 1.502595 | 1.796484 | 2.535818 | 2.134107 | 1.936211 |
| Rbm27 | 3.949098 | 4.202451 | 3.609184 | 3.490134 | 3.492277 | 3.153644 | 4.450924 | 4.007758 |
| Ccdc170 | 0.049379 | 0.083804 | 0.053011 | 0.02275 | 0.106785 | 0.043295 | 0.110485 | 0 |
| AABR07033 | 1.228012 | 0.89321 | 1.130013 | 0.889086 | 1.232986 | 1.845808 | 1.275713 | 1.632671 |
| Olr1611 | 0 | 0 | 0 | 0.052053 | 0 | 0 | 0 | 0 |
| Rbm47 | 0.357447 | 0.35388 | 0.335774 | 0.349959 | 0.555596 | 0.41135 | 0.774797 | 0.772255 |
| Dnah12 | 0.008538 | 0 | 0 | 0.011801 | 0.009232 | 0.014972 | 0.019103 | 0.011351 |
| Nrf1 | 4.385218 | 4.38079 | 4.271478 | 4.218207 | 4.325331 | 4.688979 | 5.001714 | 4.702124 |
| Dmp1 | 0.44215 | 0.630777 | 0.371486 | 1.558838 | 1.704496 | 1.415865 | 1.50548 | 1.635754 |
| AABR07002 | 0.129139 | 0 | 0 | 0.118997 | 0 | 0.056615 | 0 | 0.057231 |
| Mrps2 | 7.803812 | 7.294879 | 7.622802 | 6.650816 | 6.663602 | 6.680873 | 7.587717 | 6.902063 |
| Slc35f3 | 0 | 0 | 0 | 0 | 0 | 0 | 0 | 0 |
| Mdn1 | 4.345114 | 4.065151 | 3.688413 | 4.014197 | 4.309818 | 3.977155 | 5.087219 | 5.214667 |
| Tmem202 | 0.204235 | 0.13865 | 0.306963 | 0.489306 | 0.132502 | 0.214888 | 0.045698 | 0.543072 |
| Vsig10l | 12.53608 | 13.61971 | 14.09105 | 12.24084 | 11.49609 | 13.29731 | 12.70602 | 12.01553 |
| AABR07031 | 0.45935 | 0.7796 | 0.493142 | 0 | 0 | 0.201379 | 0 | 0.203572 |
| Dscr3 | 11.30685 | 11.40936 | 11.30794 | 13.27076 | 12.57678 | 12.23365 | 12.59375 | 14.004 |
| LOC690206 | 0 | 0 | 0 | 0.046087 | 0.108161 | 0.043853 | 0.167864 | 0.044331 |
| Uts2r | 0.045104 | 0.1531 | 0.145267 | 0.457179 | 0.585246 | 0.435021 | 0.302763 | 0.519715 |
| Iah1 | 14.29727 | 14.03279 | 12.76004 | 17.77747 | 16.5149 | 16.26138 | 14.83888 | 15.72597 |
| AC096430. | 0 | 1.742635 | 0 | 0 | 0 | 0.450142 | 0 | 0.455044 |
| Rtn4rl1 | 4.53838 | 4.221532 | 4.333483 | 3.920567 | 3.845612 | 3.520111 | 3.881234 | 4.680131 |
| Trex2 | 0.15949 | 0 | 0.114149 | 0 | 0 | 0.046614 | 0 | 0 |
| Mpp3 | 4.754182 | 4.410099 | 4.709866 | 3.720452 | 3.874371 | 3.693388 | 5.005899 | 5.143398 |
| Gmpr | 118.6644 | 118.9957 | 125.2739 | 117.398 | 116.2897 | 113.151 | 101.2549 | 100.8349 |
| Lck | 3.028391 | 3.255157 | 2.926054 | 2.348707 | 2.155749 | 2.124236 | 2.625722 | 2.169739 |
| Baz2b | 4.652436 | 4.204483 | 4.611708 | 4.163768 | 4.893998 | 4.320367 | 4.905614 | 5.30941 |

| | | | | | | | | |
|---|---|---|---|---|---|---|---|---|
| Cenpv | 6.676133 | 6.37346 | 7.092597 | 5.959551 | 7.594786 | 5.914615 | 7.157755 | 7.520015 |
| Fndc5 | 83.88645 | 88.00385 | 91.45131 | 48.27529 | 46.71747 | 51.22691 | 52.55943 | 54.46993 |
| Figf | 2.279877 | 2.41835 | 2.734421 | 5.317692 | 5.70077 | 5.325456 | 5.479858 | 6.299113 |
| Hist2h2aa | 3.864152 | 13.87013 | 1.001341 | 11.78703 | 7.636115 | 3.0376 | 2.236056 | 6.968083 |
| AC109542. | 0 | 0.442161 | 0 | 0.720196 | 0 | 1.370582 | 0 | 0.346377 |
| Srp54a | 17.99315 | 17.59231 | 17.4796 | 16.46734 | 18.34548 | 17.1697 | 16.32822 | 16.66329 |
| AABR0707( | 0.495186 | 0.70035 | 0.265807 | 0.912589 | 0 | 1.193994 | 0.69249 | 0.329181 |
| Rpl7a | 26.97042 | 27.6201 | 25.64337 | 22.73581 | 23.98297 | 24.05044 | 26.79624 | 21.81133 |
| 5S_rRNA | 0 | 0 | 0.464613 | 0 | 0.467955 | 0 | 0.484171 | 0.383591 |
| Olfml2a | 2.117053 | 1.952509 | 1.995855 | 1.942584 | 1.962119 | 1.887431 | 1.522584 | 1.986828 |
| Gimap7 | 5.694639 | 4.995827 | 4.208609 | 5.969852 | 3.792682 | 5.716697 | 4.893597 | 3.584415 |
| Arih2 | 16.28665 | 15.93053 | 15.94023 | 14.24813 | 15.17888 | 15.30891 | 15.8924 | 15.38897 |
| Pla2g2f | 0 | 0.1243 | 0.078627 | 0.168717 | 0.079192 | 0.192648 | 0.286778 | 0.129831 |
| Tmem74 | 0.057043 | 0.129084 | 0.06124 | 0.15769 | 0.06168 | 0 | 0.063818 | 0.05056 |
| AC136025. | 0 | 0 | 0 | 0.151739 | 0 | 0 | 0 | 0 |
| Erbb3 | 0.63538 | 0.539177 | 0.720017 | 1.114034 | 1.011457 | 0.982666 | 0.918162 | 1.118516 |
| Pde4dip | 216.0966 | 222.6489 | 221.4192 | 187.6219 | 184.6042 | 186.6719 | 197.6655 | 199.6367 |
| Fh | 181.5154 | 186.5223 | 181.9031 | 196.9348 | 191.0608 | 197.603 | 157.6286 | 160.6814 |
| Dennd4a | 1.552912 | 1.610043 | 1.388185 | 1.590575 | 1.625623 | 1.540609 | 2.08341 | 1.606741 |
| Synpo2l | 38.88664 | 41.59776 | 44.77163 | 47.76025 | 46.86732 | 48.05622 | 53.67768 | 54.01594 |
| AABR07017 | 0.82466 | 0 | 0.442663 | 0 | 0 | 0 | 0 | 0 |
| Abcc2 | 0.137299 | 0.103565 | 0.176879 | 0.13495 | 0.158357 | 0.144461 | 0.215046 | 0.16226 |
| Prss56 | 0.258384 | 0.48725 | 0.030821 | 0.317455 | 0.31043 | 0.251724 | 0.192713 | 0.203572 |
| AABR07046 | 0 | 0.524333 | 0 | 0 | 0 | 0 | 0 | 0 |
| AC135310. | 0 | 0 | 0 | 0 | 0 | 0 | 0 | 0 |
| Tmem135 | 4.862118 | 5.039973 | 4.506531 | 4.048922 | 3.624624 | 4.143954 | 4.375268 | 4.403223 |
| Gm5471 | 36.5949 | 35.5151 | 28.7666 | 28.21821 | 22.35099 | 26.85058 | 31.00533 | 23.34297 |
| Pitpnb | 33.38909 | 35.961 | 33.71275 | 41.32088 | 44.23777 | 42.94987 | 44.02125 | 47.80059 |
| Smpd5 | 0.460107 | 0.553126 | 0.648315 | 1.139421 | 0.684072 | 0.857272 | 0.611262 | 0.815631 |
| Mapk12 | 14.68829 | 14.31842 | 15.12677 | 12.69703 | 12.57128 | 13.25626 | 14.80005 | 15.18172 |
| Smarcal | 0.026528 | 0.075037 | 0.113917 | 0.085555 | 0.172105 | 0.127928 | 0.118713 | 0.141078 |
| Tssk2 | 0.028522 | 0 | 0.03062 | 0 | 0.03084 | 0 | 0.159544 | 0 |
| AC114526. | 0.39079 | 0.442161 | 0.419539 | 0.360098 | 0.422556 | 0.342645 | 0 | 0 |
| Bbx | 4.164079 | 2.850631 | 2.329119 | 2.789105 | 3.386378 | 2.22439 | 4.384552 | 3.039507 |
| Cox16 | 19.79024 | 20.69888 | 18.54469 | 14.60127 | 16.32493 | 16.15951 | 17.5754 | 17.78219 |
| RGD130744 | 0 | 0.014458 | 0 | 0.011775 | 0 | 0 | 0 | 0 |
| AABR07014 | 0.256193 | 0.173922 | 0.495072 | 0.944288 | 0.886458 | 0.359409 | 0.343941 | 0.363323 |
| AC136866. | 0 | 0 | 0 | 0 | 0.592906 | 0 | 0 | 0 |
| Bmx | 0.879428 | 1.567928 | 1.401878 | 0.491126 | 0.806835 | 0.584154 | 0.834794 | 0.637757 |
| AABR07071 | 0.638814 | 0.646705 | 0.541427 | 0.851982 | 0.76345 | 0.943347 | 0.865135 | 0.864219 |
| Wfdc6a | 0.557084 | 0.315157 | 0.373791 | 0.384998 | 0.376479 | 0.305282 | 0.545336 | 0.987543 |
| Mirlet7c- | 0 | 0 | 0 | 0.446788 | 0 | 0 | 0 | 0 |
| Matn2 | 5.04587 | 5.33535 | 5.23972 | 8.399671 | 6.657651 | 7.044522 | 8.114825 | 8.705218 |
| Eva1c | 4.256739 | 3.846982 | 3.822605 | 3.749732 | 4.342217 | 4.248733 | 5.031812 | 5.45774 |
| #N/A | 0 | 0 | 0 | 0 | 0 | 0 | 0.209231 | 0 |
| AABR07061 | 0 | 0.452287 | 0 | 0 | 0 | 0 | 0 | 0 |
| AABR07043 | 0.070669 | 0 | 0 | 0 | 0 | 0 | 0 | 0 |
| AABR07056 | 0 | 0 | 0.15718 | 0.04497 | 0 | 0.042791 | 0.054599 | 0.043257 |
| AABR07036 | 0.0164 | 0 | 0 | 0 | 0 | 0 | 0 | 0.014536 |
| Rnasel1 | 0 | 0 | 0.076539 | 0 | 0 | 0 | 0 | 0.063192 |
| Ssr2 | 25.14975 | 25.20531 | 23.91571 | 30.6981 | 28.502 | 29.60799 | 32.70366 | 30.6452 |
| Rpl4 | 239.6675 | 234.4352 | 222.3596 | 242.8312 | 238.533 | 248.0773 | 240.1676 | 244.3539 |
| AC134087. | 0.978802 | 0.553734 | 0.525403 | 1.352892 | 3.175094 | 1.287322 | 1.09504 | 0.867561 |
| Cplx1 | 0.391521 | 0.304554 | 0.420323 | 0.698994 | 0.449805 | 0.55784 | 0.629648 | 0.542226 |

| | | | | | | | | |
|---|---|---|---|---|---|---|---|---|
| LOC691427 | 630.3941 | 551.3772 | 534.2261 | 476.4668 | 489.1814 | 432.8005 | 418.4162 | 433.7094 |
| AC119459. | 3.977159 | 3.749973 | 5.692978 | 5.497193 | 3.583703 | 5.230764 | 7.41578 | 7.637832 |
| AABR07051 | 0 | 0 | 0 | 0 | 0 | 0 | 0 | 0 |
| AABR07032 | 0.293367 | 0 | 0 | 0.675814 | 0 | 0 | 0 | 0.260025 |
| AC103179. | 0.329345 | 0.322954 | 0.330002 | 0.708117 | 0.997126 | 0.84706 | 0.884297 | 1.167661 |
| AABR07012 | 0.274167 | 0 | 0 | 0 | 0.296453 | 0.24039 | 0 | 0.243008 |
| Kcnip4 | 0.038001 | 0 | 0.081594 | 0.175084 | 0.123271 | 0.03332 | 0 | 0.101048 |
| Atp6v1f | 86.24496 | 88.18327 | 95.91186 | 94.02983 | 92.46289 | 89.40114 | 86.92033 | 89.07545 |
| Pdhb | 232.641 | 224.7627 | 221.9453 | 220.8223 | 223.5752 | 217.9715 | 190.9721 | 198.1268 |
| Scrt1 | 0 | 0.034348 | 0.097771 | 0.027973 | 0.196948 | 0.106468 | 0.067924 | 0.053814 |
| LOC100912 | 1.009154 | 0.456724 | 0.354564 | 0.743917 | 0.277756 | 0.772213 | 0.164218 | 0.178893 |
| Gnb1l | 1.7491 | 2.019413 | 1.954415 | 2.33536 | 2.431642 | 2.034384 | 2.196426 | 2.657682 |
| Tmem88 | 11.69628 | 12.78006 | 14.13526 | 10.68528 | 8.419301 | 8.995371 | 10.20652 | 9.448776 |
| Tdp1 | 1.56894 | 1.567693 | 0.96249 | 1.032655 | 1.388023 | 1.000471 | 0.911824 | 1.029427 |
| Pttg1ip | 35.92901 | 37.72523 | 39.5342 | 38.68133 | 38.87154 | 39.32466 | 44.00116 | 41.46797 |
| Elof1 | 13.13857 | 13.26722 | 12.74009 | 12.19346 | 11.91517 | 13.70828 | 14.8569 | 12.68886 |
| Krt5 | 0 | 0 | 0 | 0 | 0 | 0.030569 | 0.039004 | 0 |
| Pcdhga10 | 0.628825 | 0.883223 | 0.721641 | 0.379631 | 0.515816 | 0.741476 | 0.460915 | 0.595797 |
| Mrps21 | 84.98409 | 78.81416 | 85.75115 | 79.93906 | 77.1256 | 72.44668 | 72.76369 | 78.72181 |
| Npas2 | 0.759814 | 0.59624 | 0.6973 | 0.575921 | 0.331281 | 0.494282 | 0.520996 | 0.467078 |
| Kcna6 | 0.00782 | 0 | 0.008396 | 0.100888 | 0.143755 | 0.123426 | 0.122489 | 0.131702 |
| Rnf13 | 9.607123 | 10.28099 | 9.221506 | 12.07964 | 10.18335 | 9.004468 | 12.75997 | 13.1714 |
| AABR07046 | 166.708 | 124.9345 | 174.1289 | 179.2776 | 228.3987 | 229.7444 | 58.36525 | 71.44707 |
| Hmbox1 | 7.105938 | 7.902989 | 6.588404 | 4.873678 | 5.500722 | 4.920673 | 6.36888 | 6.656207 |
| AABR07051 | 2.053566 | 0.580878 | 0 | 0.94614 | 1.665368 | 0.450142 | 0 | 0.910089 |
| Mir331 | 1.090957 | 0 | 0.585606 | 1.005274 | 0 | 0.478276 | 0 | 0 |
| AABR07070 | 0.770087 | 0.871317 | 0 | 0 | 0.832684 | 1.350426 | 2.584618 | 1.365133 |
| Surf6 | 7.610272 | 9.137849 | 8.670324 | 8.777634 | 7.333217 | 8.170645 | 9.093221 | 9.429741 |
| Siah1 | 7.663918 | 8.870378 | 8.119802 | 8.034471 | 7.933673 | 7.336624 | 8.855168 | 9.287358 |
| Atp2c2 | 0.116585 | 0.13191 | 0.166881 | 0.089523 | 0.04202 | 0.034074 | 0.173906 | 0.051667 |
| AABR07009 | 0 | 0 | 0 | 0 | 0 | 0.429107 | 0 | 0 |
| AABR07017 | 2.226703 | 2.345658 | 1.978352 | 2.547086 | 2.075605 | 2.266551 | 1.718025 | 2.041694 |
| Dlx6as | 0 | 0 | 0.017062 | 0.029289 | 0.034369 | 0.041804 | 0.01778 | 0.042259 |
| Hist1h2af | 0.266493 | 0.301525 | 0 | 0.368345 | 0.432233 | 0.350492 | 0.298141 | 0.354309 |
| U2surp | 9.853244 | 10.30598 | 8.365798 | 10.48357 | 10.48565 | 10.35505 | 10.24844 | 11.18921 |
| Magohb | 3.552462 | 2.307458 | 2.189401 | 2.849117 | 3.91236 | 2.768713 | 3.311947 | 2.565627 |
| AABR07065 | 0 | 0.168802 | 0 | 0.137473 | 0.161318 | 0.392432 | 0 | 0.26447 |
| Map3k7 | 0.53058 | 0.652528 | 0.581994 | 0.499537 | 1.534046 | 0.586573 | 0.65811 | 2.177597 |
| Pttg1 | 0.45143 | 0.397267 | 0.700035 | 0.831951 | 0.542361 | 0.791629 | 0.897849 | 0.444584 |
| AABR07043 | 11.77707 | 17.37036 | 10.38568 | 10.85211 | 16.82757 | 20.4679 | 15.76375 | 10.62501 |
| Zdhhc3 | 19.20084 | 22.09941 | 19.64405 | 17.30457 | 17.73521 | 18.89355 | 18.7875 | 20.85986 |
| Ngrn | 25.90776 | 21.66257 | 22.53791 | 29.88651 | 26.78092 | 26.16298 | 24.1024 | 26.5176 |
| AABR07052 | 0.146683 | 0.165965 | 0 | 0.135163 | 0.158606 | 0 | 0 | 0.260025 |
| Lca5 | 0.557467 | 0.427281 | 0.424725 | 0.38112 | 0.233335 | 0.299579 | 0.342012 | 0.350659 |
| RGD156483 | 0 | 0 | 0.149915 | 0 | 0 | 0 | 0 | 0 |
| Zbp1 | 0.440255 | 0.58115 | 0.393868 | 0.563442 | 0.555382 | 0.772032 | 1.149255 | 1.018908 |
| AABR07056 | 0 | 0 | 0 | 0 | 0 | 0 | 0 | 0 |
| Gigyf2 | 14.29235 | 14.45601 | 13.61071 | 13.87731 | 13.85761 | 14.27492 | 15.59321 | 16.03569 |
| Prame | 0 | 0 | 0 | 0 | 0 | 0 | 0 | 0.033272 |
| AC129138. | 0.401271 | 0.68103 | 0.861581 | 1.294145 | 0.650833 | 1.055505 | 2.020161 | 1.067 |
| Stk31 | 0 | 0 | 0 | 0 | 0 | 0 | 0 | 0 |
| RGD130501 | 1.622178 | 1.26185 | 1.523822 | 1.681619 | 1.891071 | 1.55567 | 1.616324 | 1.280555 |
| Sprr2d | 0 | 0 | 0 | 0 | 0 | 0.186644 | 0 | 0 |
| Gstt1 | 2.320009 | 1.749987 | 2.846489 | 4.631894 | 3.225333 | 4.165238 | 2.966312 | 3.672035 |

| | | | | | | | | |
|---|---|---|---|---|---|---|---|---|
| Trmt10c | 7.192572 | 7.166011 | 7.464296 | 8.082078 | 7.669207 | 8.338533 | 6.973833 | 7.632452 |
| Adgrg2 | 0.344211 | 0.428404 | 0.283308 | 0.592063 | 0.583098 | 0.452706 | 0.860029 | 0.864425 |
| Orai3 | 3.740423 | 4.210952 | 3.935271 | 3.825784 | 3.538907 | 3.787945 | 4.331081 | 4.276766 |
| AABR07065 | 0 | 0 | 0 | 0 | 0 | 0 | 0 | 0 |
| LOC499823 | 0 | 0 | 0 | 0.071805 | 0 | 0 | 0 | 0 |
| AABR07072 | 0 | 0 | 0 | 0 | 0 | 0 | 0.036479 | 0 |
| Mt-cox3 | 66289.51 | 64206.97 | 63109.98 | 62171.64 | 66902.6 | 64556.76 | 53341.86 | 53155.87 |
| Ephb2 | 0.100725 | 0.113966 | 0.060075 | 0.195941 | 0.242028 | 0.215883 | 0.475789 | 0.436469 |
| AABR07071 | 0 | 0 | 0 | 0 | 0.043223 | 0 | 0 | 0 |
| Ikbkap | 6.49491 | 6.313479 | 6.778678 | 6.026438 | 6.754152 | 6.724749 | 7.354675 | 7.088326 |
| Ift22 | 1.3274 | 1.351701 | 0.855029 | 2.018192 | 2.33236 | 1.803991 | 1.559288 | 1.294194 |
| Pole | 0.471831 | 0.608928 | 0.419479 | 0.665748 | 0.422497 | 0.575305 | 0.618591 | 0.725329 |
| Phf8 | 3.146371 | 3.185237 | 2.913626 | 3.01794 | 2.94453 | 2.742608 | 3.694992 | 3.596065 |
| Zcchc12 | 0.30081 | 0.283626 | 0.053823 | 0.323381 | 0.406576 | 0.329688 | 0.308488 | 0.422152 |
| AABR07056 | 0 | 0 | 0 | 0 | 0 | 0 | 0 | 0.055854 |
| Cic | 9.799922 | 11.88484 | 12.10518 | 10.97672 | 10.00202 | 10.7069 | 13.07876 | 13.32846 |
| Rasa4 | 4.394776 | 4.902447 | 5.116783 | 8.270336 | 6.916067 | 7.399143 | 11.03368 | 10.46064 |
| Ggt7 | 0.274887 | 0.399885 | 0.379425 | 0.343761 | 0.700616 | 0.361531 | 0.50523 | 0.382872 |
| AABR07002 | 0 | 0 | 0.037404 | 0 | 0 | 0 | 0.038978 | 0.030881 |
| Mir3572 | 0 | 0 | 0 | 0 | 0 | 0 | 0 | 0.429764 |
| Sec24d | 17.9782 | 17.03534 | 19.18517 | 17.26059 | 18.04271 | 17.54324 | 19.32176 | 19.77899 |
| Grm4 | 0.314915 | 0.356312 | 0.241487 | 0.808364 | 0.802639 | 0.670572 | 0.754957 | 0.638 |
| AABR07058 | 0 | 0 | 0 | 0 | 0 | 0 | 0 | 0 |
| AABR07030 | 0.372711 | 0.140568 | 0 | 0.05724 | 0.201504 | 0.163397 | 0.486468 | 0.330352 |
| Slc25a26 | 39.71122 | 40.92441 | 39.59097 | 45.07687 | 44.06242 | 44.42402 | 43.89888 | 44.23818 |
| Yipf2 | 5.340125 | 5.117289 | 5.411217 | 6.502374 | 5.83312 | 5.566116 | 4.663608 | 5.167902 |
| AABR07037 | 0.073963 | 0.066949 | 0.063523 | 0.054523 | 0.09597 | 0.064851 | 0.066197 | 0.078669 |
| Mok | 1.207412 | 1.416728 | 0.528095 | 1.236203 | 1.063788 | 0.862612 | 0.950562 | 1.387283 |
| Slc10a7 | 1.655786 | 1.568819 | 1.315129 | 1.686999 | 1.601151 | 1.404582 | 2.33435 | 2.207374 |
| Slc9a2 | 0.026308 | 0.014883 | 0 | 0.036363 | 0.028446 | 0.046134 | 0.029432 | 0.081613 |
| Hint2 | 71.13187 | 78.86462 | 87.87685 | 75.67343 | 71.59946 | 69.42019 | 77.07982 | 72.79 |
| Cwc22 | 3.909989 | 4.107971 | 4.004875 | 4.613919 | 5.112204 | 4.495247 | 4.39664 | 4.650293 |
| Setx | 3.515553 | 3.696304 | 3.179527 | 3.124866 | 3.330736 | 3.315358 | 3.743348 | 4.037788 |
| Krt7 | 0 | 0.041347 | 0.039231 | 0.101018 | 0.079027 | 0.032041 | 0 | 0 |
| Scyl2 | 4.360973 | 4.068807 | 3.872888 | 4.923145 | 4.332789 | 4.144015 | 4.687284 | 4.593894 |
| AABR07051 | 0 | 0 | 0 | 0.074008 | 0 | 0 | 0 | 0 |
| Tmem261 | 22.39964 | 22.21859 | 18.82535 | 22.07913 | 21.23344 | 19.42941 | 16.66169 | 19.16196 |
| AC128759. | 0 | 0 | 0 | 0 | 0 | 0.043112 | 0 | 0 |
| Gng11 | 0.581198 | 0.526078 | 1.029522 | 0.696216 | 0.722707 | 0.560554 | 0.585196 | 0.540902 |
| Otor | 0 | 0.064472 | 0 | 0.052506 | 0 | 0 | 0 | 0.050505 |
| Scnm1 | 8.879 | 9.703688 | 10.39874 | 9.762195 | 8.78249 | 8.846722 | 7.675833 | 8.004045 |
| AABR07058 | 0.047134 | 0 | 0 | 0 | 0 | 0 | 0.052731 | 0 |
| Csf2 | 0 | 0 | 0.127479 | 0 | 0 | 0 | 0.26569 | 0.105248 |
| AC135696. | 3.772421 | 3.121207 | 3.543693 | 3.302331 | 2.982816 | 3.969195 | 2.505873 | 3.38548 |
| Ict1 | 44.39719 | 48.37284 | 52.14438 | 50.81731 | 47.04866 | 48.46529 | 46.62717 | 45.68579 |
| Rab13 | 7.767504 | 7.397042 | 7.782984 | 12.94305 | 13.08827 | 12.71303 | 13.03491 | 13.88419 |
| Hmg20b | 23.05714 | 22.17105 | 23.56255 | 21.6179 | 20.38847 | 21.4248 | 27.41221 | 24.99472 |
| Per3 | 13.61283 | 13.06645 | 14.24298 | 12.76579 | 12.24914 | 13.11186 | 15.48736 | 14.11848 |
| Gmppa | 11.84833 | 12.19027 | 12.55517 | 13.03909 | 13.44204 | 13.51059 | 13.94219 | 14.06583 |
| Rn50_X_07 | 1.475096 | 1.147439 | 1.286683 | 1.614101 | 0.996875 | 1.374201 | 0.825136 | 1.14402 |
| Ctrl | 0.524972 | 0.519733 | 0.211346 | 0.120935 | 0.283822 | 0.057537 | 0.367072 | 0.581636 |
| AABR07013 | 0.220604 | 0.249603 | 0.207229 | 0.406556 | 0.238536 | 0.265961 | 0.370203 | 0.268857 |
| Cyld | 4.667991 | 4.747832 | 4.238354 | 5.102157 | 5.74548 | 5.682162 | 5.611239 | 5.920107 |
| Mapk3 | 36.04751 | 38.09139 | 37.46825 | 37.57838 | 35.16255 | 36.76252 | 38.05865 | 36.92834 |

| | | | | | | | | |
|---|---|---|---|---|---|---|---|---|
| Spats1 | 0.464786 | 0.350589 | 0.388094 | 0.380695 | 0.614248 | 0.724489 | 0.462206 | 0.274642 |
| AABR07022 | 1.443672 | 2.227428 | 2.958851 | 1.693092 | 0.709555 | 1.265813 | 1.761945 | 1.163271 |
| Peg3 | 3.93598 | 3.294375 | 3.579034 | 3.741263 | 3.329551 | 3.908311 | 3.958893 | 4.187486 |
| Anapc2 | 17.27997 | 18.91225 | 17.65183 | 17.8077 | 16.59916 | 18.53319 | 20.26925 | 20.11641 |
| AABR07041 | 0.115598 | 0.130794 | 0.124102 | 0.213038 | 0.124995 | 0 | 0 | 0.10246 |
| AABR07029 | 0 | 0 | 0 | 0 | 0 | 0.327961 | 0 | 0.663065 |
| Foxn2 | 4.272971 | 4.236563 | 4.102566 | 4.34329 | 4.15589 | 4.576964 | 4.460073 | 4.451108 |
| Dcun1d4 | 3.305624 | 3.375265 | 3.418964 | 3.343173 | 3.574323 | 3.145796 | 3.630535 | 3.680289 |
| Elovl2 | 0.027539 | 0.01558 | 0.014783 | 0 | 0 | 0.03622 | 0.03081 | 0.012205 |
| Arf5 | 109.0915 | 107.064 | 118.34 | 107.7364 | 104.9954 | 104.0786 | 125.594 | 119.4834 |
| Adgrl3 | 0.286766 | 0.268034 | 0.133853 | 0.29871 | 0.52578 | 0.426349 | 0.362667 | 0.276277 |
| AC111369. | 0.14153 | 0 | 0.151941 | 0.130414 | 0.306068 | 0 | 0 | 0 |
| Fut2 | 0.31588 | 0.059567 | 0.320278 | 0.194047 | 0.151803 | 0.169256 | 0.314127 | 0.27998 |
| Caprin2 | 4.406669 | 3.434758 | 3.337871 | 4.218484 | 4.658982 | 4.228683 | 4.300043 | 4.665321 |
| Arv1 | 4.716647 | 4.034014 | 4.385814 | 4.072428 | 4.256728 | 4.330941 | 4.985929 | 4.509779 |
| Foxo4 | 34.45891 | 34.22457 | 35.04543 | 28.27402 | 25.66852 | 28.21916 | 33.92171 | 33.60977 |
| Bach2 | 1.199646 | 1.357342 | 1.287896 | 0.925473 | 0.693829 | 1.05185 | 1.186051 | 1.298222 |
| Txndc12 | 25.59558 | 23.96567 | 24.0389 | 26.80001 | 27.2912 | 24.37744 | 25.76769 | 25.74728 |
| AABR07001 | 0.039671 | 0 | 0 | 0 | 0 | 0 | 0 | 0 |
| Med9 | 18.88085 | 18.32567 | 16.70427 | 18.11065 | 20.51398 | 18.23016 | 20.41047 | 20.12237 |
| AABR07025 | 0.515075 | 0.582783 | 0.552966 | 1.423863 | 0.928238 | 0.301079 | 1.152485 | 1.21743 |
| Rad52 | 6.336276 | 5.865708 | 7.589452 | 7.38273 | 7.757284 | 8.264607 | 5.975636 | 7.101421 |
| AABR07040 | 0.046839 | 0 | 0 | 0.129481 | 0 | 0 | 0.262007 | 0.041516 |
| Rbbp6 | 6.012221 | 5.493601 | 5.588885 | 5.91959 | 6.008146 | 5.486685 | 6.696791 | 6.548525 |
| Defb19 | 0 | 0 | 0 | 0 | 0.496689 | 0 | 0 | 0 |
| Cdh13 | 16.22237 | 17.23444 | 17.74405 | 19.99365 | 20.24932 | 21.56551 | 22.30323 | 22.35538 |
| Cnot11 | 9.110729 | 9.149062 | 11.26742 | 8.905523 | 9.432095 | 9.445127 | 9.542082 | 10.60342 |
| AABR07024 | 0 | 0.28623 | 0 | 0 | 0 | 0 | 0 | 0 |
| Sp100 | 7.1057 | 7.398915 | 7.352031 | 9.252076 | 7.126531 | 9.842043 | 11.7515 | 18.20983 |
| Glycam1 | 0 | 0 | 0 | 0.148243 | 0 | 0 | 0 | 0 |
| Epsti1 | 1.242138 | 1.183512 | 0.912405 | 1.475907 | 2.898281 | 2.321519 | 2.194182 | 2.984204 |
| Pcdhb20 | 0.837607 | 0.842411 | 0.982486 | 1.186318 | 0.956008 | 0.870417 | 0.659425 | 0.838651 |
| Matn1 | 0 | 0 | 0 | 0.025544 | 0 | 0 | 0.062027 | 0.024571 |
| AABR07034 | 0.010208 | 0 | 0 | 0 | 0 | 0 | 0 | 0 |
| Rae1 | 7.948703 | 6.368847 | 7.141721 | 6.192748 | 7.229976 | 8.01634 | 7.862177 | 7.952455 |
| AABR07019 | 6.234038 | 7.053521 | 9.369694 | 12.06328 | 8.08893 | 7.652414 | 8.369238 | 12.70874 |
| Mir327 | 0 | 0 | 0 | 0 | 0 | 0 | 0 | 0 |
| Tmem88b | 0.054237 | 0.09205 | 0 | 0.124943 | 0 | 0.071333 | 0.091017 | 0.096146 |
| AABR07039 | 0 | 0.132549 | 0 | 0 | 0.126672 | 0 | 0 | 0 |
| Fam53a | 6.940955 | 7.993103 | 8.406206 | 7.124166 | 6.543639 | 7.017119 | 9.589094 | 8.647988 |
| AABR07011 | 0 | 0.053378 | 0 | 0 | 0 | 0 | 0 | 0 |
| AC123486. | 0 | 0.361278 | 0.342794 | 0 | 0.345259 | 0 | 0 | 0.566031 |
| AABR07066 | 0.066539 | 0.225856 | 2.000138 | 0.367877 | 4.820468 | 0.233365 | 0.297762 | 0.412836 |
| Cap2 | 27.31059 | 31.36742 | 29.76256 | 39.91528 | 41.30707 | 41.86321 | 37.41542 | 37.95557 |
| Trnp1 | 4.170737 | 4.369438 | 5.472564 | 3.70083 | 3.841645 | 2.84426 | 5.011668 | 3.559816 |
| Cyp26a1 | 0 | 0.131886 | 0.031284 | 0.026852 | 0 | 0 | 0.097804 | 0.051658 |
| A4galt | 0.158685 | 0.179544 | 0.102215 | 0.175466 | 0.51475 | 0.445231 | 0.213035 | 0.16878 |
| AABR07054 | 0.266203 | 0.387252 | 0.612398 | 0.455549 | 0.534563 | 0.366782 | 0.510542 | 0.404484 |
| AABR07037 | 1.792126 | 0.729974 | 1.423732 | 0.627522 | 1.007656 | 1.099936 | 0.681683 | 0.889532 |
| Ccdc67 | 0.059507 | 0.089772 | 0.085179 | 0.036555 | 0.042896 | 0.086959 | 0 | 0.123069 |
| Tmem212 | 0 | 0 | 0 | 0 | 0 | 0 | 0 | 0.04785 |
| Adprm | 9.703213 | 8.978555 | 8.392659 | 10.38908 | 8.920276 | 8.921119 | 7.559312 | 9.923584 |
| B4galt5 | 7.762406 | 8.233873 | 8.919384 | 9.862974 | 11.08188 | 10.2623 | 16.9614 | 17.14676 |
| Dfnb31 | 19.47536 | 19.14427 | 23.94786 | 12.85478 | 11.59333 | 12.06523 | 13.65623 | 14.33907 |

| | | | | | | | | |
|---|---|---|---|---|---|---|---|---|
| Tspan31 | 18.41409 | 17.58021 | 18.15258 | 20.21281 | 20.11436 | 20.17598 | 20.42043 | 22.0874 |
| Tgs1 | 3.345094 | 2.87646 | 2.40282 | 3.01512 | 2.801532 | 2.687677 | 2.953048 | 2.66304 |
| Sorcs2 | 0.641252 | 0.664233 | 0.736906 | 0.549277 | 0.73244 | 0.847335 | 0.676987 | 0.776511 |
| Oxr1 | 44.94662 | 41.10642 | 40.95601 | 35.5503 | 37.24802 | 33.58948 | 39.6098 | 38.07065 |
| Wbp1 | 0 | 0 | 0 | 0 | 0 | 0.081917 | 0 | 0 |
| Tnfaip8l3 | 0.64011 | 0.473551 | 0.555045 | 1.179672 | 1.038212 | 1.187257 | 1.211907 | 1.418403 |
| NEWGENE_1 | 0 | 0 | 0.028897 | 0 | 0.014552 | 0 | 0 | 0.023857 |
| AABR0700 | 0 | 0 | 0 | 0 | 0 | 0 | 0 | 0 |
| AABR0701 | 0.811875 | 0.765498 | 0.581066 | 0 | 0.585246 | 0.949137 | 0.908289 | 0.119934 |
| AABR0703 | 0 | 0 | 0.090528 | 0 | 0 | 0 | 0 | 0 |
| Dusp18 | 2.522245 | 2.439005 | 2.518876 | 2.459275 | 2.743124 | 2.880102 | 4.216259 | 3.444371 |
| Ripply1 | 0 | 0 | 0 | 0.088376 | 0.311113 | 0.168185 | 0.214596 | 0 |
| Golm1 | 22.19192 | 22.62798 | 21.0465 | 17.21594 | 15.9814 | 18.03509 | 21.88338 | 22.2743 |
| St6galnac | 18.4775 | 20.99464 | 20.89697 | 20.95359 | 19.83895 | 18.84431 | 22.12552 | 21.35298 |
| Naal1 | 0.470304 | 0.550476 | 0.557133 | 0.373592 | 0.350712 | 0.369705 | 0.616872 | 0.790585 |
| AC097745. | 0 | 0 | 0 | 0 | 0 | 0 | 0.689231 | 0.546053 |
| Cyp4f39 | 0.018702 | 0.063482 | 0.080312 | 0.017233 | 0.080889 | 0.049194 | 0.125539 | 0.04973 |
| Ropn1l | 0 | 0 | 0.069149 | 0 | 0 | 0.112951 | 0 | 0 |
| Ptpn5 | 0.0827 | 0.112286 | 0.071027 | 0.15241 | 0.107307 | 0.101517 | 0.111026 | 0.102622 |
| Neto1 | 0.02972 | 0.033626 | 0 | 0.054771 | 0.048203 | 0.013029 | 0.049873 | 0.065855 |
| LOC10036 | 16.77874 | 15.99892 | 18.01305 | 16.27141 | 16.31372 | 17.38106 | 17.2575 | 22.06788 |
| Slc22a17 | 11.24848 | 14.17223 | 14.24097 | 13.29697 | 12.76853 | 14.5779 | 14.7151 | 14.85582 |
| LOC69003 | 1.457571 | 1.394423 | 1.234026 | 1.692518 | 1.588864 | 1.579317 | 1.81627 | 1.659537 |
| AABR0701 | 0 | 0 | 0 | 0 | 0 | 0 | 0 | 0 |
| Prmt1 | 44.42721 | 46.13879 | 46.00149 | 47.0718 | 46.17241 | 44.7904 | 43.57993 | 48.33753 |
| Ppfia2 | 0.015165 | 0 | 0 | 0.027949 | 0.016398 | 0.013297 | 0.050899 | 0 |
| Plagl1 | 0.293303 | 0.395678 | 0.375434 | 0.301452 | 0.439123 | 0.26706 | 0.466961 | 0.449947 |
| AABR0703 | 0 | 0 | 0 | 0 | 0 | 0 | 0 | 0 |
| Hltf | 23.25503 | 22.89655 | 20.70976 | 27.08718 | 28.35328 | 26.56952 | 28.4082 | 26.35362 |
| Cpox | 11.80613 | 12.32391 | 11.44806 | 10.56305 | 10.41854 | 10.9193 | 12.27082 | 11.52768 |
| Hormad2 | 0.055297 | 0.031283 | 0.059364 | 0.050954 | 0.089687 | 0 | 0 | 0.024506 |
| Kansl1l | 4.281601 | 4.146673 | 3.612944 | 3.864147 | 3.54367 | 3.676867 | 3.883304 | 4.216662 |
| Ccnd2 | 52.06965 | 50.82725 | 50.41346 | 81.25227 | 80.64904 | 81.04848 | 83.65095 | 85.01669 |
| Dbnl | 23.50192 | 22.30572 | 24.00601 | 26.74422 | 25.23958 | 25.42802 | 27.33951 | 26.71616 |
| Mns1 | 1.192117 | 1.381722 | 1.061309 | 0.321509 | 0.817427 | 0.280433 | 0.292761 | 0.592745 |
| Mad1l1 | 2.886942 | 3.65206 | 3.293024 | 3.085097 | 2.90483 | 2.830104 | 3.611076 | 3.145241 |
| Poln | 0.082956 | 0.093861 | 0.089058 | 0.095551 | 0.067274 | 0.054552 | 0.139211 | 0 |
| Mpdu1 | 15.70568 | 17.58464 | 16.28874 | 17.04163 | 15.65211 | 14.59771 | 17.11204 | 17.55537 |
| AABR0705 | 0 | 0 | 0 | 0 | 0 | 0 | 0 | 0 |
| LOC10091 | 0 | 0 | 0.116394 | 0.099903 | 0.117231 | 0.095061 | 0.242587 | 0.096096 |
| Ogfod1 | 2.350912 | 3.098399 | 2.634793 | 3.142286 | 2.178863 | 2.672871 | 3.063628 | 2.930961 |
| Tiprl | 10.21407 | 8.965845 | 9.121704 | 9.10646 | 8.8941 | 8.929284 | 8.157359 | 9.346997 |
| Pif1 | 0.437522 | 0.247518 | 0.322924 | 0.352764 | 0.443518 | 0.287715 | 0.397703 | 0.266611 |
| NEWGENE_1 | 7.051482 | 5.624132 | 10.05225 | 9.480197 | 8.374632 | 7.905805 | 8.147536 | 9.118967 |
| Ntm | 0.21011 | 0.128008 | 0.086756 | 0.074465 | 0.052428 | 0.12754 | 0.018082 | 0.085953 |
| Zfp639 | 6.105766 | 5.729897 | 5.552411 | 5.361459 | 5.825361 | 5.605472 | 5.303962 | 5.507347 |
| Mynn | 4.84737 | 4.917201 | 4.306726 | 5.632833 | 6.377456 | 5.652946 | 5.95731 | 5.926158 |
| Akap7 | 8.505135 | 8.168497 | 8.122171 | 5.513342 | 5.079451 | 5.419557 | 7.136376 | 7.450867 |
| Snx8 | 4.942447 | 4.165809 | 5.821598 | 5.513064 | 6.079834 | 6.088012 | 6.93972 | 6.455821 |
| Prrt3 | 0.249362 | 0.310355 | 0.214164 | 0.482531 | 0.674078 | 0.349825 | 0.390564 | 0.198919 |
| Dsc2 | 3.085787 | 2.968363 | 3.027418 | 2.715637 | 2.536829 | 2.58402 | 3.465171 | 2.735086 |
| Zfp263 | 3.068986 | 3.618109 | 2.856989 | 3.599209 | 3.921805 | 3.518856 | 4.41786 | 3.994692 |
| Fam131b | 0.195395 | 0.280035 | 0.111877 | 0.240065 | 0.239448 | 0.205587 | 0.189453 | 0.323285 |
| Cuedc1 | 20.54717 | 22.94925 | 24.76876 | 22.07094 | 19.77344 | 21.28435 | 28.89827 | 27.55212 |

| | | | | | | | | |
|---|---|---|---|---|---|---|---|---|
| 43352 | 22.44254 | 24.03422 | 26.2748 | 23.88544 | 23.46788 | 22.88976 | 31.1005 | 29.3331 |
| Racgap1 | 0.32039 | 0.302088 | 0.32485 | 0.344431 | 0.173216 | 0.124852 | 0.358438 | 0.283977 |
| Btbd18 | 0 | 0.027241 | 0 | 0 | 0 | 0 | 0 | 0 |
| Fam46b | 10.16794 | 10.82781 | 12.31087 | 9.483385 | 9.366465 | 10.25345 | 8.4912 | 9.28656 |
| Gjd3 | 0.437947 | 0.35394 | 0.805995 | 0.634151 | 0.405896 | 0.438848 | 0.979911 | 0.720895 |
| Nubpl | 5.596781 | 5.141233 | 3.777622 | 5.157213 | 4.224219 | 4.397102 | 4.587582 | 5.206285 |
| Pnma5 | 0 | 0 | 0 | 0.025067 | 0 | 0 | 0 | 0 |
| Gak | 5.962165 | 6.62616 | 6.564887 | 6.479985 | 6.332363 | 6.702092 | 8.669952 | 7.744439 |
| Fance | 5.984677 | 6.388094 | 6.909833 | 6.555143 | 6.22695 | 6.163184 | 7.232285 | 7.556434 |
| Zfp763 | 1.261829 | 1.155758 | 1.483669 | 1.32883 | 1.396884 | 1.1854 | 1.478901 | 1.198309 |
| Dip2a | 3.930235 | 3.979449 | 4.29128 | 3.210017 | 3.392522 | 3.494983 | 4.396973 | 4.156524 |
| Ip6k2 | 7.729505 | 7.076569 | 6.936213 | 9.351594 | 8.134502 | 8.691418 | 8.746443 | 8.942966 |
| Htr5b | 0 | 0 | 0 | 0 | 0 | 0.020701 | 0 | 0.020926 |
| Kpna3 | 15.29337 | 13.24906 | 13.76329 | 15.90609 | 15.33583 | 14.75902 | 12.67687 | 13.26449 |
| LOC103692 | 0 | 0.212364 | 0 | 0 | 0 | 0 | 0.419962 | 0 |
| Jun | 64.85983 | 68.32211 | 74.56323 | 59.1878 | 56.56752 | 57.03266 | 81.95026 | 79.29084 |
| Zfp498 | 3.290006 | 4.825445 | 3.597439 | 4.1825 | 3.820949 | 3.31204 | 4.532729 | 3.402111 |
| Atrnl1 | 1.544783 | 1.874506 | 1.674447 | 1.485347 | 1.533173 | 1.419901 | 1.870167 | 1.97776 |
| Dppa1 | 0 | 0 | 0 | 0 | 0 | 0 | 0 | 0 |
| AABR07015 | 1.068692 | 29.0202 | 20.65157 | 18.7104 | 27.73348 | 18.74061 | 0 | 0 |
| AABR07057 | 1.870211 | 2.116056 | 0.669264 | 0 | 2.022233 | 1.093202 | 0 | 1.105108 |
| AABR07014 | 0 | 0 | 0 | 0 | 0 | 0 | 0 | 0.032054 |
| Kcnk5 | 0.226758 | 0.290774 | 0.292127 | 0.376107 | 0.228844 | 0.291605 | 0.372074 | 0.495767 |
| Ahnak2 | 0.57122 | 0.687783 | 0.711622 | 2.544527 | 2.66218 | 2.552442 | 1.965011 | 2.095598 |
| Map3k15 | 0.054776 | 0.092965 | 0.088208 | 0.075711 | 0 | 0.120069 | 0.030641 | 0.024275 |
| Grp | 0 | 0 | 0 | 0.037965 | 0 | 0.036125 | 0 | 0 |
| AABR07033 | 0 | 0.121165 | 0 | 0 | 0.115792 | 0.093895 | 0.359415 | 0.284752 |
| AABR07063 | 0 | 0 | 0 | 0 | 0 | 0 | 0 | 0 |
| Psmc2 | 60.4852 | 62.68489 | 60.46657 | 57.58941 | 54.59369 | 55.03756 | 62.55392 | 63.77083 |
| Kcnj1 | 0 | 0.030229 | 0 | 0 | 0 | 0 | 0 | 0.023681 |
| Mettl21c | 0.094866 | 0.161004 | 0 | 0 | 0.153866 | 0.083178 | 0 | 0.084084 |
| AC135741. | 7.258643 | 12.90585 | 9.462463 | 9.077322 | 6.727427 | 4.545989 | 5.220416 | 11.02919 |
| Msantd4 | 11.3528 | 11.65167 | 10.63327 | 11.28487 | 13.14555 | 11.52173 | 12.12097 | 10.7598 |
| Tsg101 | 36.58098 | 39.64821 | 38.31135 | 40.27141 | 40.40595 | 39.70049 | 40.36455 | 38.80038 |
| AABR07031 | 0 | 0.060132 | 0.057055 | 0 | 0.019155 | 0.046598 | 0 | 0 |
| AABR07024 | 1.122127 | 1.692845 | 4.015583 | 2.412657 | 1.617786 | 2.295724 | 1.673848 | 2.98379 |
| Eif4g2 | 76.62768 | 77.15036 | 68.10873 | 76.5975 | 80.60875 | 77.28509 | 82.87358 | 79.36436 |
| Vgf | 0 | 0 | 0 | 0.036583 | 0 | 0 | 0.044416 | 0 |
| Tpst1 | 5.753137 | 5.319513 | 4.051161 | 4.303735 | 4.515085 | 3.661226 | 4.152487 | 5.071876 |
| Lsm4 | 23.51006 | 24.8445 | 25.67152 | 28.28449 | 27.65864 | 24.69605 | 29.71034 | 28.48048 |
| Ciz1 | 12.54648 | 12.53987 | 13.81314 | 13.65416 | 12.41245 | 13.3934 | 14.66749 | 13.55277 |
| Uqcc2 | 135.6453 | 134.3073 | 133.1048 | 117.9972 | 117.4084 | 113.9181 | 117.6616 | 116.9139 |
| Yipf7 | 19.85323 | 18.09772 | 16.91291 | 18.96056 | 19.03351 | 18.7816 | 20.09773 | 17.70377 |
| Aloxe3 | 1.585329 | 1.548268 | 1.504884 | 2.198916 | 2.111164 | 2.297187 | 2.520373 | 2.884267 |
| Ppp3r1 | 36.31525 | 36.05331 | 31.26055 | 35.44817 | 35.83703 | 33.9377 | 36.65514 | 36.44757 |
| Lpar5 | 0.340702 | 0.462586 | 0.365766 | 0.251155 | 0.294717 | 0.448092 | 0.190581 | 0.301981 |
| AABR07025 | 0.538744 | 0.121913 | 0.347026 | 0 | 0.349522 | 0.094474 | 0.241089 | 0.286509 |
| AC142421. | 0 | 0.135273 | 0 | 0.110167 | 0.129275 | 0 | 0.133755 | 0.211938 |
| Hepacam | 0.584628 | 0.519733 | 0.627635 | 0.577191 | 0.632149 | 0.622445 | 0.420464 | 0.444158 |
| AABR07067 | 0 | 0 | 0 | 0 | 0 | 0 | 1.346774 | 0 |
| Cdc25b | 7.943882 | 8.076597 | 7.381776 | 8.632046 | 7.961591 | 8.213683 | 9.075907 | 9.548604 |
| AABR07014 | 0 | 0.445486 | 0.845386 | 1.451222 | 0 | 0.345222 | 0 | 0.348981 |
| Tstd3 | 3.841262 | 4.531942 | 3.277924 | 5.203473 | 4.118001 | 4.174044 | 3.930131 | 3.783001 |
| Ufc1 | 39.03458 | 34.79556 | 34.15375 | 41.97139 | 39.85963 | 39.93719 | 35.42197 | 37.41807 |

| | | | | | | | | |
|---|---|---|---|---|---|---|---|---|
| Elovl3 | 0.027374 | 0 | 0 | 0 | 0 | 0.024001 | 0 | 0 |
| AABR07021 | 0.089362 | 0.303325 | 0.575613 | 0.494059 | 0.386502 | 0.313409 | 0.499869 | 0.396028 |
| AABR07044 | 0 | 0.294286 | 0.186153 | 0.639114 | 0.093746 | 0.152035 | 0 | 0.15369 |
| Psd2 | 0 | 0.013475 | 0 | 0.021948 | 0 | 0.020884 | 0 | 0 |
| AABR07017 | 0 | 0.080942 | 0.153602 | 0.06592 | 0 | 0.062725 | 0.080034 | 0.126816 |
| Tex24 | 0 | 0 | 0 | 0 | 0 | 0.04828 | 0 | 0 |
| C1qtnf3 | 0.245465 | 0.092577 | 0.087841 | 1.030405 | 1.061672 | 1.028293 | 1.220514 | 1.112014 |
| AABR07067 | 1.861899 | 1.316657 | 0.249858 | 1.501209 | 1.761589 | 1.020322 | 0.520753 | 1.23772 |
| AABR07024 | 0.689025 | 0 | 0.739713 | 0 | 0.496689 | 0.201379 | 0.25695 | 0 |
| Dlx1 | 0.018994 | 0.042981 | 0 | 0.017502 | 0.061613 | 0.083269 | 0.084998 | 0.117846 |
| AABR07035 | 0 | 0.731476 | 0 | 0 | 1.398087 | 0 | 2.169802 | 1.146037 |
| Nfatc2 | 3.664624 | 4.450522 | 5.696247 | 3.272504 | 2.478478 | 2.989838 | 3.324177 | 3.14781 |
| Smad3 | 3.607499 | 4.128767 | 4.051458 | 3.99475 | 3.687152 | 3.746447 | 4.047541 | 4.496781 |
| Bicd1 | 0.632591 | 0.572598 | 0.488972 | 0.664515 | 0.58825 | 0.721054 | 0.934184 | 0.85226 |
| Pitx2 | 0.52923 | 0.630315 | 0.388743 | 0 | 0.060237 | 0.122113 | 0.031162 | 0.049377 |
| Ube2e3 | 31.46652 | 30.3953 | 31.462 | 27.95652 | 25.74674 | 25.94271 | 27.42708 | 31.22053 |
| Krt79 | 0.647884 | 0.988024 | 0.725786 | 0.415304 | 0.335044 | 0.568065 | 0.409683 | 0.499349 |
| Atox1 | 39.77159 | 42.21398 | 43.00232 | 46.15897 | 45.56423 | 44.00484 | 38.03236 | 44.14835 |
| AABR07007 | 0.471765 | 0 | 1.51941 | 1.304139 | 0.510113 | 0 | 0 | 0.418149 |
| Peg12 | 0.422306 | 0.364053 | 0.367016 | 0.500321 | 0.347911 | 0.264484 | 0.449959 | 0.392135 |
| Tsc1 | 4.970232 | 5.033262 | 4.628344 | 4.453357 | 3.696645 | 4.30975 | 5.176464 | 5.293738 |
| Mecom | 4.58121 | 4.11015 | 4.108161 | 3.873759 | 4.557308 | 4.111322 | 4.220797 | 4.528712 |
| LOC100912 | 0 | 0.206085 | 0.488854 | 4.363762 | 0 | 0.239554 | 0.203773 | 3.793882 |
| Rasgrp4 | 1.447793 | 1.832461 | 1.50161 | 2.215936 | 2.069614 | 1.463067 | 1.894256 | 2.501251 |
| Il1b | 1.087831 | 1.612811 | 1.127585 | 2.696092 | 2.433632 | 2.598313 | 1.888474 | 2.726354 |
| Dalrd3 | 7.450681 | 7.406197 | 7.934015 | 8.672222 | 9.197897 | 8.913123 | 9.482887 | 10.1866 |
| AABR07036 | 0.327287 | 0.37031 | 0.351364 | 0.301582 | 1.061672 | 0.286966 | 0 | 0 |
| Sptbn1 | 141.6436 | 140.5771 | 140.6443 | 146.5785 | 147.6136 | 143.6079 | 183.3521 | 185.41 |
| Micu1 | 13.27522 | 13.17523 | 13.11757 | 14.7934 | 14.9255 | 13.69379 | 14.80034 | 14.39257 |
| Mir126a | 13.31337 | 18.57826 | 14.76918 | 20.44624 | 17.27466 | 16.73155 | 20.35569 | 16.12708 |
| Cpped1 | 7.184503 | 7.527845 | 8.772206 | 7.599286 | 7.248776 | 6.80954 | 7.18865 | 7.220036 |
| Tmem121 | 0.328313 | 0.804853 | 0.058744 | 1.109267 | 1.065 | 0.719663 | 0.61217 | 0.6305 |
| Mgat2 | 9.626398 | 10.43898 | 12.09844 | 11.316 | 11.54771 | 11.41398 | 13.1026 | 13.2933 |
| Gnat3 | 0.842598 | 0.600264 | 0.268025 | 0.31632 | 0.472417 | 0.273626 | 0.418961 | 0.331927 |
| Ranbp2 | 9.082071 | 9.015048 | 8.305284 | 10.18936 | 10.76334 | 10.39413 | 10.46176 | 10.44529 |
| Cbx2 | 0.516677 | 0.505596 | 0.404771 | 0.540435 | 0.422781 | 0.37956 | 0.499922 | 0.569351 |
| AABR07018 | 0.815034 | 0.576358 | 0.328122 | 1.126532 | 0.771124 | 0.535967 | 0.455912 | 0.812706 |
| AABR07007 | 1.036949 | 0.586629 | 0.556615 | 0.955508 | 0.560619 | 0.454599 | 1.740139 | 1.378649 |
| Ptgr1 | 51.09078 | 50.68917 | 49.23652 | 61.53058 | 60.57506 | 67.97729 | 52.16508 | 50.55867 |
| Pon1 | 0 | 0 | 0 | 0.034442 | 0 | 0 | 0 | 0.03313 |
| Rad51 | 0.193321 | 0.164051 | 0.207543 | 0.24494 | 0.104518 | 0.254256 | 0.243314 | 0.085675 |
| Nkx3-2 | 0 | 0 | 0 | 0 | 0 | 0 | 0 | 0 |
| Snrpd2 | 32.74984 | 35.3329 | 35.82471 | 33.65773 | 31.57208 | 36.67228 | 34.24271 | 32.87487 |
| AABR07059 | 0.585094 | 0.662006 | 1.256272 | 0.808712 | 0.316327 | 1.795539 | 0.327289 | 1.037196 |
| Ccdc130 | 3.947974 | 3.537821 | 4.374031 | 4.336379 | 4.712851 | 4.236982 | 4.346148 | 4.703039 |
| Dido1 | 4.673734 | 4.591558 | 4.491518 | 4.364547 | 4.24533 | 4.08141 | 5.031984 | 5.551257 |
| Mir128-1 | 0 | 0 | 0 | 0 | 0 | 0 | 0 | 0 |
| AABR07058 | 0 | 0.03929 | 0.01864 | 0.047997 | 0 | 0 | 0.019425 | 0 |
| Cidea | 131.0107 | 122.9483 | 122.7327 | 129.4704 | 116.8747 | 127.9887 | 109.3366 | 110.5958 |
| Idh3g | 310.419 | 322.767 | 327.6537 | 319.4393 | 316.7914 | 325.4296 | 295.5803 | 297.1564 |
| Tbpl1 | 13.70792 | 16.45559 | 11.35131 | 16.48232 | 14.14433 | 14.91396 | 16.41119 | 14.743 |
| Bcan | 0.043675 | 0.074124 | 0.210994 | 0.281711 | 0.425023 | 0.287205 | 0.244306 | 0.232266 |
| AABR07028 | 43.53312 | 39.26177 | 39.28498 | 37.7886 | 33.42775 | 34.29757 | 46.5854 | 49.76978 |
| Snrnp48 | 7.156676 | 5.52996 | 5.996604 | 8.203033 | 8.446191 | 7.231531 | 7.664827 | 8.045183 |

| | | | | | | | | |
|---|---|---|---|---|---|---|---|---|
| Coprs | 16.31692 | 16.3151 | 18.94308 | 18.96908 | 15.89944 | 20.37871 | 21.65085 | 19.00305 |
| LOC10036( | 4.469013 | 4.786285 | 4.651275 | 3.929408 | 4.131415 | 3.290289 | 4.465411 | 4.082059 |
| AABR0706 | 0 | 0 | 0.12248 | 0 | 0 | 0 | 0 | 0 |
| Gbp5 | 3.959457 | 3.850841 | 4.033671 | 5.496013 | 5.429057 | 5.096685 | 6.314628 | 5.988488 |
| H1foo | 0 | 0 | 0 | 0 | 0.04928 | 0 | 0.050988 | 0 |
| Nipa2 | 8.660518 | 7.582288 | 7.646456 | 7.912839 | 7.859838 | 7.529333 | 8.685279 | 8.276715 |
| Tmem255b | 8.802067 | 11.54681 | 11.02451 | 12.51878 | 12.13832 | 10.01059 | 7.4212 | 9.497733 |
| Ppp1r9a | 1.44778 | 1.529919 | 1.334338 | 1.019432 | 0.915648 | 0.778415 | 1.268265 | 1.077437 |
| Gdf7 | 0 | 0.035693 | 0.033866 | 0 | 0.13644 | 0.082978 | 0.105876 | 0.083882 |
| Acot7 | 29.39474 | 27.6893 | 30.32033 | 23.12934 | 21.51655 | 20.41664 | 23.55104 | 25.0329 |
| Gpr50 | 0 | 0 | 0 | 0 | 0 | 0 | 0 | 0.023069 |
| AC095278. | 0 | 0.204662 | 0 | 0.166677 | 0 | 0.0793 | 0.202365 | 0.080163 |
| AC091481. | 0 | 0 | 0 | 0 | 0 | 0 | 0 | 0 |
| Angptl6 | 0.100062 | 0.113216 | 0.035808 | 0.030734 | 0 | 0.11698 | 0.14926 | 0.08869 |
| Sfxn2 | 0.278812 | 0.200749 | 0.081633 | 0.233558 | 0.904425 | 0.333358 | 0.08507 | 0.921101 |
| AC134158. | 0 | 0 | 0 | 0 | 0 | 0 | 0 | 0 |
| Cox11 | 2.102087 | 2.167667 | 1.742534 | 1.986028 | 2.416815 | 1.936434 | 2.351722 | 2.311292 |
| Vipr1 | 0.834819 | 0.815755 | 1.059183 | 0.559457 | 0.451339 | 0.532342 | 0.8066 | 0.470872 |
| Rgs22 | 0.051159 | 0.127344 | 0.054922 | 0.131994 | 0.165951 | 0.143539 | 0.022894 | 0.036276 |
| Impa1 | 13.59721 | 13.73315 | 12.12333 | 14.29897 | 15.42237 | 14.12235 | 13.55039 | 14.34424 |
| AABR0707 | 0 | 0 | 0 | 0 | 0 | 0 | 0 | 0 |
| AABR0701 | 0 | 0 | 0 | 0 | 0 | 0.379459 | 0 | 0 |
| Cyp26b1 | 1.127881 | 1.393342 | 1.569166 | 1.537737 | 1.779565 | 1.574211 | 2.446393 | 2.519645 |
| Fam209a | 0 | 0.092577 | 0.087841 | 0 | 0 | 0 | 0 | 0 |
| Ppp1r10 | 11.88643 | 11.90702 | 13.00468 | 11.06915 | 9.973749 | 10.53267 | 12.90274 | 13.47699 |
| Brap | 8.296475 | 9.252489 | 7.949076 | 7.699677 | 6.94518 | 7.352575 | 7.751407 | 9.462131 |
| Faap24 | 0.718917 | 0.520589 | 0.43221 | 0.741948 | 0.901731 | 1.134625 | 1.351211 | 0.637212 |
| Rdx | 29.62332 | 30.52623 | 27.21488 | 30.60036 | 32.03127 | 30.16512 | 31.76375 | 30.87881 |
| Rsbn1 | 2.408879 | 2.40902 | 2.402557 | 2.405854 | 2.268597 | 2.296065 | 3.120922 | 2.99604 |
| Dpep3 | 0.234299 | 0.430786 | 0.220093 | 0.539744 | 0.886706 | 0.462226 | 0.294889 | 0.23363 |
| AABR0704( | 0 | 0 | 0 | 0 | 0 | 0 | 0.132845 | 0 |
| Arfgef2 | 5.523015 | 5.879833 | 6.033105 | 5.479008 | 5.730203 | 5.393601 | 6.232987 | 6.052205 |
| Tmem214 | 8.972616 | 8.219869 | 9.347152 | 8.616171 | 8.703006 | 8.68951 | 8.69139 | 8.54841 |
| Gtf2h5 | 22.40186 | 21.55181 | 18.98849 | 21.37133 | 22.3753 | 21.02911 | 20.88517 | 19.94001 |
| Zfp202 | 0.866788 | 0.913093 | 0.689892 | 0.963961 | 1.082679 | 1.14 | 1.170356 | 1.165661 |
| Ube2d1 | 37.81128 | 35.11409 | 33.52421 | 29.48408 | 31.64199 | 31.39741 | 30.88618 | 32.55842 |
| Cep295 | 1.431463 | 1.579344 | 1.223297 | 1.693092 | 1.994455 | 1.723432 | 1.665197 | 1.862136 |
| AABR0705 | 0.758926 | 0 | 0.814756 | 0 | 0 | 1.996282 | 0 | 0 |
| Olr1085 | 0 | 0 | 0 | 0.258591 | 0.182066 | 0.147635 | 0.188375 | 0.099495 |
| AABR0706 | 0.9187 | 0.445486 | 1.408976 | 0.604676 | 1.419111 | 1.150739 | 0.734144 | 1.163271 |
| Wsb1 | 14.26057 | 12.73001 | 13.37107 | 16.27637 | 16.79651 | 17.17226 | 16.60158 | 15.90241 |
| Cxcl17 | 0.395214 | 0.819805 | 0.495003 | 0.42487 | 0.284893 | 0.808557 | 1.400137 | 0.642213 |
| Cttnbp2 | 1.208784 | 1.178003 | 1.202983 | 0.780506 | 0.849099 | 0.858721 | 0.720586 | 0.641279 |
| Sec14l5 | 0.586273 | 0.769475 | 0.654578 | 0.410573 | 0.431072 | 0.246742 | 0.577189 | 0.478072 |
| Hnrnpa2b1 | 318.2442 | 311.4973 | 294.9881 | 296.931 | 307.5357 | 300.4293 | 301.4458 | 310.5004 |
| Elp6 | 4.572575 | 4.72377 | 4.823578 | 5.642356 | 4.60031 | 6.379917 | 4.270408 | 5.215906 |
| Samd7 | 0.078275 | 0 | 0 | 0.144255 | 0.211594 | 0.068632 | 0.087571 | 0.034689 |
| Fam179a | 1.153203 | 0.988481 | 0.881633 | 1.127034 | 1.568124 | 1.164331 | 1.739752 | 1.378342 |
| Naprt | 12.04024 | 12.50004 | 12.89793 | 12.12947 | 11.23978 | 11.6103 | 12.85649 | 11.69044 |
| AABR0705 | 0.44508 | 0.590412 | 0.461345 | 0.494976 | 0.265522 | 0.269135 | 0.377744 | 0.36729 |
| Phc3 | 6.133828 | 6.478692 | 5.954572 | 5.140988 | 4.639165 | 4.934734 | 6.643246 | 6.188602 |
| Rnls | 2.800629 | 2.64065 | 2.25499 | 2.983899 | 2.6182 | 3.171808 | 2.97003 | 2.870201 |
| Runx1t1 | 1.237599 | 1.062717 | 1.020207 | 0.946939 | 1.06339 | 1.085128 | 0.865357 | 1.087152 |
| Ppm1l | 10.14709 | 9.789911 | 9.116638 | 6.783552 | 7.092005 | 6.823004 | 8.422539 | 8.61069 |

| | | | | | | | | |
|---|---|---|---|---|---|---|---|---|
| Cox6a2 | 2607.602 | 2481.497 | 2423.417 | 2590.635 | 2570.543 | 2420.322 | 2168.249 | 2116.6 |
| Armc5 | 5.247826 | 5.992142 | 5.427129 | 6.122218 | 6.264397 | 5.783283 | 7.846 | 6.856205 |
| Dpy30 | 31.64398 | 31.82549 | 28.9122 | 25.98776 | 26.77436 | 27.35191 | 31.38464 | 26.85411 |
| Slc4a9 | 0.069728 | 0.019724 | 0.074858 | 0.080315 | 0.037698 | 0.045853 | 0 | 0.030902 |
| Epha10 | 0 | 0 | 0 | 0 | 0 | 0.023546 | 0.09013 | 0 |
| Cela1 | 0 | 0 | 0 | 0 | 0 | 0.166206 | 0.106036 | 0.126012 |
| Vamp5 | 45.00731 | 34.27181 | 45.01127 | 54.24535 | 45.52006 | 52.51657 | 45.3744 | 46.71788 |
| Ftl1 | 3.69099 | 3.327895 | 4.333999 | 6.058213 | 6.1736 | 5.208361 | 5.290686 | 5.265083 |
| AABR0707 | 8.727654 | 6.83649 | 6.486711 | 9.588764 | 9.80005 | 9.418356 | 14.64617 | 10.71104 |
| Lyrm2 | 20.60132 | 22.5822 | 20.62785 | 19.98079 | 22.20276 | 21.26659 | 19.60391 | 21.37826 |
| Chka | 4.009518 | 3.836303 | 4.448919 | 4.587271 | 4.18995 | 5.426686 | 6.11135 | 7.799356 |
| Lrr1 | 0 | 0 | 0 | 0 | 0.037849 | 0.030692 | 0 | 0.062051 |
| Pcdhb5 | 0.405466 | 0.52758 | 0.435294 | 0.448345 | 0.504188 | 0.533269 | 0.544341 | 0.718769 |
| RGD15634( | 0 | 0.153762 | 0 | 0.125224 | 0.293888 | 0.158874 | 0.152036 | 0.160604 |
| Focad | 3.583008 | 3.765023 | 3.799583 | 4.498516 | 4.221439 | 4.568415 | 4.17179 | 4.391786 |
| Th | 0.029652 | 0.10065 | 0.031834 | 0 | 0.032063 | 0 | 0 | 0 |
| Herc3 | 11.9218 | 12.25731 | 12.6415 | 10.754 | 11.45352 | 10.4714 | 11.10099 | 10.604 |
| AABR0703 | 0 | 0.203607 | 0 | 0 | 0 | 0.157782 | 0 | 0 |
| Tap1 | 20.06738 | 21.34486 | 21.00948 | 24.41311 | 24.74713 | 25.15663 | 28.99083 | 30.02428 |
| Polg | 9.689248 | 9.630983 | 10.17982 | 8.713696 | 7.987019 | 8.223321 | 11.12935 | 10.73221 |
| Tlk1 | 0.778376 | 0.364426 | 0.605116 | 0.247325 | 0.217667 | 0.482443 | 0.405378 | 0.880234 |
| Dgat1 | 27.30701 | 30.35396 | 30.40994 | 24.0299 | 21.42386 | 24.91524 | 21.1267 | 22.26409 |
| AABR0707 | 0 | 0.75961 | 0 | 0 | 0 | 0 | 1.502171 | 0.595058 |
| Elf3 | 0 | 0 | 0 | 0.023492 | 0 | 0 | 0 | 0.022597 |
| Aatf | 4.302845 | 4.702873 | 3.802346 | 6.068729 | 5.032409 | 4.696675 | 3.79867 | 4.566213 |
| Mipep | 25.53903 | 23.33925 | 22.98875 | 23.87514 | 20.58142 | 22.43092 | 21.6121 | 21.39827 |
| Cd44 | 6.502353 | 6.57165 | 6.160894 | 9.467252 | 9.733167 | 9.069278 | 9.992789 | 10.43968 |
| Dsg3 | 0.033741 | 0.025451 | 0.012074 | 0.082909 | 0.060806 | 0.059168 | 0.150991 | 0.069781 |
| Mkrn3 | 0.296971 | 0.85869 | 0.602211 | 0.851347 | 0.6779 | 0.549701 | 0.738307 | 0.789661 |
| Rn50_1_2 | 5.574184 | 7.106396 | 4.972821 | 5.787482 | 6.027284 | 5.851171 | 5.269985 | 6.054068 |
| Art1 | 9.322211 | 8.605658 | 9.35765 | 8.062863 | 7.496297 | 6.87537 | 7.040702 | 6.532635 |
| S100a4 | 20.52937 | 24.58903 | 21.95349 | 44.6323 | 46.564 | 44.50822 | 47.81872 | 43.64244 |
| AABR0706 | 0 | 0.079851 | 0.151531 | 0.130062 | 0.152621 | 0.371276 | 0 | 0.813192 |
| Tmed3 | 8.044396 | 7.26398 | 7.266011 | 12.40184 | 12.50379 | 11.73295 | 12.72075 | 11.92929 |
| AABR0704 | 0.447572 | 0.506407 | 0.480497 | 0.41242 | 0.483953 | 0 | 0 | 0 |
| LOC68389 | 21.25745 | 18.42994 | 16.281 | 17.43801 | 15.8842 | 17.65359 | 15.51624 | 15.77787 |
| Ethe1 | 7.444425 | 7.326871 | 8.265767 | 8.034358 | 8.435486 | 8.449696 | 9.07007 | 8.044581 |
| Ppp1r3d | 23.13169 | 25.03449 | 26.15096 | 18.92742 | 18.37651 | 18.33889 | 18.55562 | 18.76524 |
| Pebp1 | 338.3718 | 351.8214 | 372.5992 | 398.9689 | 378.0689 | 363.5874 | 358.2431 | 358.1456 |
| Vsir | 8.877004 | 8.554752 | 9.469904 | 13.08253 | 12.41456 | 12.25205 | 14.03526 | 13.40312 |
| Ercc6 | 1.339649 | 1.669129 | 1.32691 | 1.609173 | 1.448543 | 1.580124 | 1.623635 | 1.611468 |
| Dusp11 | 1.436587 | 0.576766 | 1.144263 | 0.469721 | 2.956397 | 0.934543 | 0.985052 | 1.478693 |
| Tpp2 | 9.261734 | 7.443044 | 9.761263 | 10.10675 | 10.47939 | 10.37073 | 10.41991 | 9.49073 |
| AABR0705 | 0.831205 | 0 | 0 | 1.148884 | 0.89877 | 1.822003 | 0.464958 | 0.736738 |
| Msl3l2 | 3.082386 | 3.174084 | 3.048869 | 3.989181 | 3.969568 | 4.372808 | 4.959548 | 3.867876 |
| Plpp7 | 25.53005 | 26.74523 | 27.89451 | 15.98615 | 16.28077 | 17.54798 | 19.37915 | 18.8965 |
| Cpsf1 | 8.01948 | 7.224592 | 7.857509 | 6.593663 | 7.08097 | 6.519734 | 8.880422 | 8.949745 |
| Slc25a42 | 29.62735 | 31.70539 | 33.5906 | 21.76122 | 21.3369 | 22.55621 | 25.91928 | 25.64795 |
| AABR0707 | 0 | 0 | 0 | 0 | 0 | 0 | 0 | 0 |
| Ptpru | 0.643244 | 0.926292 | 1.014919 | 1.014816 | 1.148679 | 0.982722 | 1.711854 | 1.511733 |
| AABR0702 | 0 | 0.197499 | 0.187394 | 0.482531 | 0 | 0.153048 | 0 | 0.30943 |
| Agpat4 | 4.167964 | 3.403925 | 3.364342 | 3.089818 | 4.405103 | 3.846815 | 4.522694 | 5.610851 |
| Kcng1 | 0.03814 | 0.12946 | 0.020473 | 0.087861 | 0.08248 | 0.066882 | 0.149342 | 0.101416 |
| Sox2 | 0.022542 | 0.229551 | 0.145204 | 0.145403 | 0.268122 | 0.197652 | 0.100878 | 0.119883 |

| | | | | | | | | |
|---|---|---|---|---|---|---|---|---|
| Serinc4 | 37.38546 | 38.62002 | 34.24582 | 46.13411 | 45.36195 | 45.82807 | 47.33817 | 47.3463 |
| Gna15 | 0.692569 | 0.377293 | 0.853668 | 1.205442 | 0.984619 | 1.034566 | 1.219617 | 0.954891 |
| AABR0704: | 3.692469 | 2.658635 | 3.483604 | 2.165205 | 2.903719 | 2.550805 | 2.75398 | 3.173642 |
| Rnd1 | 4.230932 | 4.234743 | 3.983139 | 11.81587 | 12.00017 | 11.50002 | 17.5135 | 16.58692 |
| AABR0704: | 0 | 0 | 0 | 0.073631 | 0 | 0 | 0 | 0.070826 |
| AABR0703 | 0 | 0 | 0 | 0.748111 | 0.438934 | 2.135557 | 0.454145 | 0 |
| Foxr1 | 0 | 0.074528 | 0 | 0.060696 | 0 | 0 | 0.147383 | 0 |
| Plpp5 | 4.416829 | 4.637619 | 4.552078 | 7.423559 | 6.189505 | 7.218674 | 6.403992 | 6.013217 |
| Tcaf1 | 2.487725 | 2.270456 | 2.065759 | 2.343 | 2.377848 | 2.277648 | 2.737026 | 2.473004 |
| Tdrd12 | 0.20785 | 0.166004 | 0.183762 | 0.315454 | 0.423049 | 0.310885 | 0.136784 | 0.28176 |
| Prkca | 4.023678 | 4.108847 | 3.867435 | 4.537535 | 4.774825 | 4.788862 | 6.110356 | 6.141394 |
| Il25 | 0.049402 | 0 | 0 | 0 | 0 | 0 | 0 | 0.043787 |
| LOC100362 | 332.4032 | 347.1548 | 326.8085 | 336.8013 | 335.5047 | 337.4979 | 342.9223 | 349.7094 |
| Lipt1 | 1.313431 | 0.883036 | 1.001341 | 1.368137 | 1.132039 | 1.435349 | 0.95831 | 0.894209 |
| Rtn4 | 80.47863 | 83.69358 | 88.23775 | 97.7268 | 94.28031 | 96.19415 | 104.8545 | 109.0427 |
| AABR0705 | 0 | 0 | 0 | 0.160844 | 0.188742 | 0 | 0 | 0 |
| Zfp455 | 0.794455 | 0.950253 | 0.560476 | 0.648395 | 0.859032 | 0.776188 | 1.041167 | 0.824879 |
| Gpr65 | 1.076266 | 0.503894 | 0.557799 | 0.512967 | 0.64207 | 0.520646 | 0.996479 | 1.250001 |
| Mir106b | 0.638609 | 0 | 0 | 0 | 0 | 1.119865 | 0.714447 | 0.566031 |
| Fcmr | 6.308001 | 6.706822 | 5.887253 | 6.192291 | 6.786475 | 6.642592 | 7.41174 | 7.304948 |
| Baiap3 | 0.47327 | 0.545982 | 0.328761 | 0.359141 | 0.290989 | 0.406827 | 0.436037 | 0.386582 |
| Igsf10 | 1.114636 | 1.199032 | 1.131791 | 4.078015 | 4.019444 | 4.16923 | 5.147735 | 5.071189 |
| AABR0702 | 0 | 0 | 0 | 0 | 0 | 0.129337 | 0 | 0 |
| Ptger4 | 6.612397 | 6.073785 | 6.526345 | 6.158581 | 5.189436 | 5.735414 | 7.63629 | 6.711672 |
| Hs2st1 | 2.633563 | 3.253257 | 2.909249 | 3.29425 | 3.246577 | 2.688385 | 3.657983 | 3.326599 |
| Klrb1 | 0.081187 | 0 | 0 | 0 | 0 | 0 | 0 | 0 |
| Ccno | 0 | 0 | 0 | 0 | 0 | 0 | 0 | 0 |
| Zfp672 | 5.718839 | 5.334693 | 5.735369 | 5.087972 | 5.001235 | 6.098877 | 5.756179 | 5.752159 |
| Bri3bp | 4.286179 | 4.638757 | 5.451761 | 3.734896 | 4.080448 | 4.738506 | 4.482457 | 4.666228 |
| Gm1113 | 0 | 0 | 0 | 0 | 0 | 0 | 0 | 0 |
| Slfnl1 | 0.601216 | 0.85031 | 0.516355 | 0.360098 | 0.487565 | 0.39536 | 0.739875 | 0.772687 |
| Inpp5f | 2.446801 | 2.706645 | 2.251541 | 2.949138 | 2.574824 | 2.863669 | 3.165087 | 3.766218 |
| Dusp13 | 0 | 0 | 0.09915 | 0 | 0 | 0 | 0 | 0 |
| AABR0703 | 1.172371 | 1.326483 | 0.839077 | 1.080294 | 1.690224 | 0 | 2.623194 | 0.346377 |
| Cxadrl1 | 0.037165 | 0 | 0 | 0 | 0 | 0 | 0 | 0 |
| Smlr1 | 0 | 0 | 0 | 0 | 0 | 0 | 0 | 0.082441 |
| AC112093. | 0 | 0 | 0.631665 | 0 | 0 | 0 | 0 | 0 |
| Tcf21 | 19.71221 | 18.52155 | 20.15021 | 20.6912 | 18.07101 | 20.89072 | 21.47785 | 20.20665 |
| Reg3b | 1.206897 | 0.379319 | 0.647841 | 1.050324 | 1.16 | 0.999419 | 0.900149 | 1.010303 |
| Dnajb5 | 21.4731 | 22.77324 | 21.60808 | 25.54369 | 27.29272 | 28.48586 | 32.36781 | 32.51061 |
| Slc1a1 | 1.358517 | 1.378634 | 1.548668 | 1.316347 | 1.423513 | 1.326227 | 1.190809 | 1.253775 |
| AABR0707 | 0 | 0 | 0 | 0 | 0 | 0.342645 | 0 | 0 |
| Shpk | 0.700079 | 0.693093 | 0.807948 | 0.999898 | 0.756985 | 1.043511 | 0.979022 | 0.977311 |
| AABR0700 | 1.873557 | 1.907858 | 1.407968 | 1.208486 | 0.709048 | 1.149916 | 0.733618 | 1.162439 |
| Rxrb | 51.57624 | 62.26511 | 66.92134 | 57.53084 | 54.27436 | 59.71912 | 73.93484 | 72.66212 |
| Ctns | 1.870211 | 2.080789 | 2.041255 | 2.786044 | 2.932237 | 2.159074 | 2.336412 | 2.900907 |
| Popdc3 | 1.359173 | 1.324252 | 1.215966 | 1.60032 | 1.265536 | 1.092414 | 1.351629 | 1.305095 |
| RGD156222 | 0.056673 | 0.192369 | 0.121684 | 0 | 0.245119 | 0.248455 | 0.19021 | 0.100464 |
| #N/A | 0 | 0.222743 | 0 | 0 | 0 | 0 | 0 | 0 |
| Papss2 | 7.09984 | 6.971763 | 7.148217 | 7.779483 | 8.233832 | 7.208913 | 9.671511 | 9.145959 |
| Adcyap1r1 | 1.204583 | 1.183099 | 1.041742 | 1.287264 | 1.628123 | 1.576935 | 1.010726 | 1.134412 |
| Pde9a | 2.430779 | 2.881279 | 2.385917 | 3.583787 | 2.828622 | 3.085324 | 2.693548 | 3.837098 |
| Sfr1 | 30.92319 | 31.84314 | 23.81945 | 33.70859 | 34.02718 | 32.20542 | 31.54132 | 34.09597 |
| Tmem259 | 38.60423 | 43.07222 | 47.77584 | 39.28949 | 35.6962 | 38.45982 | 51.52937 | 50.30691 |

| | | | | | | | | |
|---|---|---|---|---|---|---|---|---|
| Htr1f | 0.037378 | 0 | 0 | 0.068884 | 0.121247 | 0.032773 | 0.125449 | 0.099389 |
| Rpl3 | 158.2615 | 152.9517 | 143.738 | 222.2623 | 226.8396 | 224.1874 | 216.1123 | 216.6584 |
| Hemgn | 0.215166 | 0.24345 | 0.173245 | 0.049567 | 0.348983 | 0.282986 | 0.120359 | 0.286068 |
| Ndufs4 | 128.5727 | 129.4156 | 125.6089 | 122.5655 | 120.2378 | 115.4299 | 117.1302 | 116.1602 |
| Pfdn1 | 57.79293 | 55.37142 | 55.40044 | 57.8599 | 62.38771 | 53.2608 | 59.38355 | 61.7735 |
| Tll2 | 0.343947 | 0.48645 | 0.350787 | 0.269394 | 0.241738 | 0.286494 | 0.327074 | 0.274372 |
| LOC10091( | 0 | 0 | 0 | 0 | 0 | 0 | 0 | 0 |
| Senp8 | 6.132465 | 5.792219 | 4.980631 | 4.422385 | 4.26687 | 4.254805 | 3.519853 | 4.301142 |
| Jagn1 | 14.09168 | 13.63042 | 12.83759 | 14.0909 | 14.61226 | 14.14852 | 13.62665 | 11.82034 |
| Rbm14 | 7.176145 | 6.811333 | 7.81105 | 6.502326 | 5.345407 | 5.260853 | 7.693837 | 7.314659 |
| Pou3f2 | 0 | 0 | 0 | 0 | 0 | 0 | 0.064062 | 0 |
| AABR0703( | 0.254822 | 0 | 0.273568 | 0 | 0.275535 | 0.335142 | 0.142542 | 0.112931 |
| Slc35b2 | 9.896367 | 10.65341 | 12.47606 | 12.42805 | 11.09826 | 11.77612 | 10.62875 | 11.67881 |
| Sox5 | 0.445036 | 0.311714 | 0.409521 | 0.663944 | 0.343722 | 0.427371 | 0.545304 | 0.676213 |
| LOC50111( | 5.442306 | 5.745235 | 4.948093 | 6.214601 | 5.603123 | 6.050392 | 4.894194 | 5.539276 |
| Tusc3 | 4.442773 | 4.546287 | 3.998048 | 6.351473 | 5.898914 | 5.556713 | 6.834269 | 6.312143 |
| Il36rn | 0 | 0 | 0 | 0 | 0 | 0 | 0 | 0.020955 |
| Smarcd3 | 34.05506 | 33.50275 | 37.16514 | 31.78419 | 31.17115 | 30.82006 | 38.69459 | 37.03729 |
| Hand2 | 19.56618 | 22.32193 | 23.09738 | 21.39596 | 21.50778 | 20.14543 | 18.71076 | 13.31269 |
| Guca1b | 0.04894 | 0.055373 | 0 | 0.045096 | 0 | 0 | 0.109504 | 0 |
| Osbpl10 | 2.139351 | 1.80998 | 2.048435 | 2.255483 | 1.917288 | 2.061674 | 3.212777 | 2.357454 |
| Hsdl2 | 147.2922 | 151.6352 | 146.3688 | 125.0544 | 123.1145 | 122.6292 | 120.2328 | 126.5535 |
| Mlap | 0 | 0 | 0 | 0 | 0 | 0 | 0 | 0 |
| Gjb4 | 0 | 0 | 0 | 0.036417 | 0.021367 | 0.017326 | 0 | 0 |
| Nol8 | 0.090736 | 0.076997 | 0.060882 | 0.229926 | 0.183959 | 0.546956 | 0.063445 | 0.211112 |
| Entpd2 | 21.88304 | 22.49749 | 23.66926 | 23.21804 | 22.77338 | 24.32603 | 28.80208 | 27.64965 |
| Prickle3 | 11.38705 | 12.30947 | 12.97741 | 11.49521 | 11.55455 | 11.59521 | 12.68523 | 11.93577 |
| Rela | 21.02782 | 20.56637 | 24.76084 | 22.18104 | 21.39279 | 22.32763 | 25.4914 | 23.76773 |
| Kxd1 | 20.79688 | 20.9438 | 20.58028 | 22.12108 | 21.3939 | 21.81998 | 19.47903 | 21.55099 |
| Ahctf1 | 4.272264 | 4.799139 | 4.145027 | 4.558906 | 4.453605 | 4.542472 | 5.528151 | 5.805215 |
| Metap1d | 4.187319 | 4.64558 | 4.320437 | 4.383919 | 4.386747 | 4.685735 | 4.028379 | 4.592351 |
| LOC100912 | 1.636435 | 2.160141 | 2.488825 | 1.884888 | 1.179636 | 1.673966 | 3.203849 | 1.329583 |
| Zfp37 | 0.592375 | 0.636732 | 0.254381 | 0.409387 | 0.512421 | 0.363576 | 0.662722 | 0.525051 |
| Wdr64 | 0.025943 | 0.073383 | 0.09748 | 0.095622 | 0.126233 | 0.113734 | 0.07256 | 0.114973 |
| Sdccag8 | 2.049591 | 1.732891 | 1.910209 | 2.552747 | 2.362316 | 2.685751 | 2.167003 | 2.09614 |
| Cd2ap | 9.768492 | 9.897841 | 8.713163 | 9.628236 | 9.642906 | 9.012449 | 10.30329 | 11.07056 |
| Fbxo40 | 28.90805 | 28.2498 | 27.04165 | 25.41603 | 23.11489 | 25.91166 | 27.33539 | 28.20123 |
| Ndufb7 | 397.2262 | 395.3174 | 402.9475 | 383.6776 | 362.7922 | 357.4712 | 314.1405 | 322.7273 |
| Drg2 | 14.63376 | 12.98621 | 14.42676 | 13.17597 | 10.65227 | 12.74704 | 15.24806 | 15.93777 |
| AABR07007 | 0 | 0.053961 | 0 | 0 | 0 | 0 | 0 | 0 |
| LOC100911 | 3.281415 | 5.375777 | 8.440063 | 20.91389 | 10.53356 | 4.975068 | 13.23126 | 8.573961 |
| Crtam | 0.10579 | 0.059848 | 0.056786 | 0 | 0 | 0.092757 | 0.236706 | 0 |
| Poldip2 | 126.7846 | 126.0876 | 135.0944 | 129.8505 | 128.8961 | 134.2882 | 123.4008 | 127.1529 |
| AABR0701( | 0.010985 | 0 | 0 | 0 | 0 | 0 | 0.024579 | 0.019473 |
| Ankrd13a | 21.26871 | 21.26407 | 22.40075 | 18.35184 | 19.30985 | 18.73674 | 23.5047 | 24.50187 |
| Nat1 | 2.035019 | 1.880939 | 1.261601 | 2.033657 | 2.138453 | 2.538239 | 2.212557 | 2.210215 |
| AABR07025 | 2.909218 | 2.743036 | 2.862962 | 3.350912 | 1.572848 | 2.338238 | 4.339605 | 3.008348 |
| Hivep1 | 3.117314 | 3.1248 | 3.000432 | 3.382977 | 3.09354 | 3.291517 | 4.37558 | 4.170197 |
| AABR07049 | 0.096527 | 0.218432 | 0.103628 | 0 | 0.052187 | 0.042317 | 0.269975 | 0.128335 |
| Rax | 0.05089 | 0.172739 | 0.109268 | 0.046893 | 0.110053 | 0.04462 | 0.056934 | 0.090213 |
| Setbp1 | 1.505144 | 1.628956 | 1.194337 | 1.133665 | 1.202928 | 0.895109 | 1.976738 | 1.577699 |
| Crhr2 | 4.391341 | 4.445586 | 5.848674 | 4.076873 | 4.034265 | 4.052981 | 4.247942 | 3.687408 |
| Cadps | 3.296607 | 3.472125 | 3.800067 | 2.687729 | 2.71039 | 2.664026 | 3.212214 | 3.285507 |
| Tfpi | 41.48765 | 39.55122 | 37.42991 | 37.22265 | 40.94735 | 38.9904 | 38.47085 | 37.03479 |

| | | | | | | | | |
|---|---|---|---|---|---|---|---|---|
| Prss39 | 0.632608 | 0.437413 | 0.226382 | 0.356231 | 0.342015 | 0.246521 | 0.432504 | 0.373808 |
| RGD131144 | 0.111774 | 0.1897 | 0.119996 | 1.02995 | 0.543866 | 0.637021 | 0.312618 | 0.445817 |
| Tnks2 | 0.705236 | 1.278629 | 1.06726 | 0.15659 | 0.707437 | 0.201151 | 0.180611 | 0.858552 |
| AC132752. | 0 | 0 | 0 | 0.175626 | 0 | 0.041778 | 0 | 0 |
| Clptm1 | 36.15336 | 35.89203 | 38.21829 | 34.00099 | 29.83163 | 33.49254 | 36.25194 | 36.95411 |
| Srsf7 | 29.09771 | 31.79521 | 26.95904 | 37.05069 | 36.79655 | 37.08982 | 41.19321 | 39.83434 |
| Daam2 | 1.623749 | 0.989259 | 1.139784 | 1.266033 | 1.367449 | 1.738563 | 1.589506 | 1.840528 |
| Wdr44 | 2.946715 | 2.682156 | 2.986756 | 2.897312 | 2.759035 | 2.857928 | 3.001981 | 3.064146 |
| Aes | 699.8318 | 695.2154 | 700.4087 | 579.2863 | 549.6859 | 525.0976 | 656.41 | 664.9558 |
| Il1rl2 | 0 | 0.041107 | 0.013001 | 0.100434 | 0.07857 | 0.063711 | 0.081292 | 0.12881 |
| AABR0707C | 0 | 0 | 0 | 0 | 0 | 0 | 0 | 0.433781 |
| LOC10369C | 0 | 0 | 0 | 0 | 0.072223 | 0 | 0.074725 | 0.059202 |
| AABR07062 | 0 | 0 | 0 | 0 | 0 | 0 | 0.461297 | 0 |
| Atp1b2 | 7.048255 | 7.128065 | 6.408385 | 7.424793 | 7.244821 | 6.958127 | 7.826865 | 7.666339 |
| Trmt1l | 5.734049 | 6.337427 | 5.44244 | 6.071007 | 6.261735 | 5.976541 | 7.200943 | 7.371124 |
| Mpz | 1.095639 | 1.27145 | 1.507998 | 4.245448 | 2.885804 | 3.547042 | 1.445759 | 2.141442 |
| Cyp2d2 | 0.031282 | 0.035394 | 0.033583 | 0.028825 | 0 | 0.054856 | 0.10499 | 0 |
| Phb | 131.9838 | 134.4444 | 144.053 | 133.7158 | 125.0644 | 129.1361 | 125.2883 | 125.5247 |
| Kremen1 | 5.401235 | 4.690647 | 5.200911 | 5.260803 | 4.943743 | 5.140844 | 6.016159 | 6.141708 |
| Fam69c | 0.085148 | 0.192682 | 0.319941 | 0.156921 | 0.322242 | 0.186644 | 0.04763 | 0.075471 |
| Zfp295 | 2.848398 | 2.67231 | 3.166761 | 2.736773 | 2.586704 | 2.657459 | 3.765023 | 3.674707 |
| Cd207 | 0 | 0 | 0.03814 | 0 | 0.038414 | 0 | 0.119236 | 0.062978 |
| Cyb5r2 | 9.230603 | 9.238917 | 8.909149 | 6.91083 | 6.76591 | 6.303514 | 7.546499 | 6.372163 |
| Ripply3 | 0.661929 | 0.416078 | 0.671144 | 0.372742 | 0.437393 | 0.612623 | 0.45255 | 0.456323 |
| AC127006. | 0 | 0 | 0 | 0 | 0.419426 | 0 | 0 | 0 |
| Fam170b | 0 | 0 | 0 | 0.028251 | 0.099454 | 0 | 0 | 0 |
| Dnaja1 | 59.35889 | 59.10237 | 54.54209 | 49.75168 | 50.01069 | 47.28336 | 71.82989 | 70.40016 |
| AABR07071 | 0.12235 | 0.138434 | 0.131351 | 0 | 0.529182 | 0.107277 | 0.13688 | 0 |
| Iqca1 | 2.905759 | 3.240762 | 2.718437 | 1.893405 | 1.77296 | 1.728845 | 2.298804 | 2.262777 |
| Vapa | 65.2076 | 62.34814 | 63.21067 | 64.9968 | 61.66452 | 61.94399 | 68.06586 | 65.14433 |
| Stpg1 | 0.09393 | 0.079708 | 0.10084 | 0.173105 | 0.126956 | 0.082358 | 0.157627 | 0.187323 |
| 5S_rRNA | 0 | 0 | 0 | 0.352213 | 0 | 0 | 0.427625 | 0 |
| Cyfip2 | 1.151289 | 0.90182 | 0.998293 | 1.003744 | 0.861834 | 0.978391 | 0.911515 | 0.949798 |
| Pddc1 | 0.714081 | 0.336645 | 0.319421 | 1.644993 | 0.643438 | 0.313053 | 2.063778 | 2.426213 |
| AABR07027 | 0.128505 | 0.145398 | 0.310407 | 0.148016 | 0.138951 | 0.084505 | 0.143766 | 0.170851 |
| Slc38a10 | 11.66009 | 13.09827 | 13.66943 | 14.05618 | 14.95774 | 14.15666 | 15.554 | 15.65672 |
| AABR07029 | 483.8128 | 575.1667 | 587.2888 | 679.4088 | 653.9763 | 637.2864 | 672.5861 | 677.4041 |
| Pithd1 | 30.06603 | 32.00983 | 31.16614 | 30.4649 | 29.39092 | 29.73417 | 33.67791 | 33.5653 |
| Wdr93 | 0.075455 | 0.056916 | 0.108008 | 0.023176 | 0.027196 | 0.044106 | 0 | 0.089173 |
| AABR07007 | 0 | 0 | 0 | 0 | 0 | 2.055872 | 0 | 0 |
| #N/A | 0 | 0.379805 | 0 | 0 | 0 | 0 | 0 | 0 |
| Rn50_6_06 | 0 | 0.14522 | 0.13779 | 0 | 0 | 0 | 0 | 0 |
| Arhgef10l | 10.93658 | 10.92302 | 12.86636 | 13.01322 | 12.64574 | 12.06812 | 14.51888 | 13.89868 |
| #N/A | 9.007685 | 10.08616 | 9.369694 | 12.1708 | 9.891277 | 9.61667 | 9.294181 | 10.54875 |
| Tpcn1 | 13.88611 | 14.506 | 13.84008 | 13.1064 | 12.42278 | 13.20294 | 13.25058 | 14.1809 |
| Prss44 | 0 | 0 | 0 | 0.031456 | 0 | 0.059862 | 0 | 0 |
| Myl12b | 52.14627 | 51.94279 | 55.22774 | 48.81987 | 49.40219 | 51.42268 | 65.46542 | 65.49431 |
| Uvssa | 0.955002 | 1.238118 | 0.854379 | 1.484994 | 0.989603 | 1.256019 | 1.090672 | 1.181525 |
| AABR07043 | 0 | 0 | 0 | 0 | 0 | 0 | 0 | 0 |
| RGD155958 | 0.078275 | 0 | 0 | 0.072127 | 0 | 0 | 0.087571 | 0.069379 |
| Igfbp2 | 0 | 0.092577 | 0.131761 | 0.188489 | 0.398127 | 0.251095 | 0.045769 | 0.036261 |
| LOC100911 | 5.527514 | 5.661626 | 7.620684 | 4.932543 | 5.536423 | 4.489416 | 7.876383 | 5.51817 |
| Scara3 | 0.791776 | 0.825594 | 0.933358 | 1.001399 | 0.788988 | 0.884803 | 1.07686 | 1.210933 |
| Strn | 6.257992 | 5.942321 | 6.315619 | 5.625081 | 6.121353 | 6.189709 | 7.211007 | 7.708044 |

| | | | | | | | | |
|---|---|---|---|---|---|---|---|---|
| #N/A | 25.8189 | 26.84103 | 26.14226 | 24.68388 | 24.76838 | 24.87249 | 31.21056 | 30.25948 |
| RGD156309 | 2.640044 | 2.689521 | 2.812537 | 4.119738 | 3.937436 | 4.035366 | 6.167403 | 6.015867 |
| Tyro3 | 1.082173 | 1.351641 | 1.750217 | 1.307989 | 1.413283 | 1.417114 | 1.477981 | 1.457461 |
| Clic3 | 1.553934 | 1.871636 | 1.865568 | 1.939979 | 1.933187 | 1.728752 | 2.000178 | 2.206689 |
| Fus | 63.33563 | 63.72432 | 61.48668 | 56.49926 | 57.71501 | 56.84892 | 61.65277 | 57.97977 |
| Itm2a | 6.915029 | 7.566902 | 6.064451 | 8.735223 | 8.670651 | 7.77102 | 9.22536 | 8.085852 |
| Ctage5 | 15.63658 | 15.30802 | 13.81048 | 13.77493 | 13.21432 | 14.06022 | 12.45638 | 13.83982 |
| LOC100361 | 0.716115 | 0.810251 | 0.672696 | 0.714861 | 0.709798 | 0.758701 | 0.701013 | 0.581834 |
| Pnisr | 17.57014 | 16.71808 | 16.53395 | 24.62039 | 23.46854 | 23.10268 | 23.61088 | 24.60965 |
| Map3k19 | 0 | 0.013481 | 0 | 0.032937 | 0.012883 | 0.041788 | 0 | 0 |
| Grik1 | 0 | 0 | 0 | 0 | 0 | 0 | 0 | 0 |
| Aldh1l2 | 0.302584 | 0.299564 | 0.101513 | 0.575064 | 0.511218 | 0.381377 | 0.571248 | 0.38553 |
| AABR07061 | 0.323246 | 0 | 0 | 0.148929 | 0 | 0 | 0.180817 | 0.143255 |
| ST7 | 3.647781 | 4.616453 | 4.003151 | 4.083341 | 4.411764 | 3.648519 | 5.411071 | 4.76599 |
| Fbxl19 | 2.765057 | 3.321057 | 3.699164 | 4.233419 | 4.346732 | 4.04689 | 6.210641 | 5.410628 |
| Ppa1 | 95.3668 | 89.7091 | 84.47272 | 91.12323 | 93.80647 | 94.9181 | 86.34416 | 94.69736 |
| Rn60_20_( | 0.955583 | 0.864957 | 0.615527 | 1.93717 | 1.239909 | 0.837856 | 0 | 1.016376 |
| Fdft1 | 36.03266 | 33.78989 | 34.26851 | 32.89709 | 33.33162 | 33.89065 | 28.19781 | 29.42428 |
| Mir93 | 0 | 0 | 0.646186 | 0 | 0.650833 | 0 | 0 | 0 |
| Fastkd2 | 4.351261 | 3.923974 | 3.861963 | 3.414043 | 4.099367 | 3.701867 | 3.470256 | 3.780368 |
| AABR0707( | 0 | 0 | 0.014247 | 0 | 0 | 0 | 0 | 0.023525 |
| Nckap1 | 22.09835 | 23.23407 | 19.8014 | 22.61222 | 21.75533 | 21.79609 | 25.93648 | 25.61142 |
| Rn50_X_07 | 0 | 0 | 0 | 0 | 0.100752 | 0.163397 | 0 | 0 |
| AABR07056 | 0 | 0 | 0.294336 | 0 | 0 | 0 | 0 | 0 |
| Ikbkb | 6.890641 | 7.429152 | 7.128254 | 8.579242 | 8.711156 | 9.30192 | 10.84534 | 11.26033 |
| Ube3c | 11.21886 | 10.4633 | 10.6777 | 10.73214 | 12.00899 | 11.16999 | 11.64384 | 11.60113 |
| Jakmip1 | 0.045241 | 0.076781 | 0.121422 | 0.125062 | 0.073377 | 0.099167 | 0.07592 | 0.040099 |
| Cep41 | 0.741027 | 1.117917 | 0.625067 | 0.877921 | 0.724951 | 0.835371 | 0.868506 | 0.813192 |
| Cecr2 | 3.986253 | 4.405368 | 4.35414 | 2.765559 | 2.706453 | 3.098898 | 3.176199 | 3.38942 |
| Uchl1 | 0.196864 | 0.111371 | 0.15851 | 4.036211 | 3.778382 | 3.279606 | 3.083403 | 2.442869 |
| Mir222 | 0 | 0 | 0 | 0.468477 | 0 | 0 | 0 | 0.450626 |
| AABR07003 | 0 | 0 | 0 | 0 | 0 | 0 | 0 | 0.110511 |
| AABR07031 | 0.758926 | 0.85869 | 0 | 0.699321 | 0.410308 | 0.665427 | 1.27358 | 0.336337 |
| Ripk1 | 3.251176 | 3.312007 | 2.832024 | 3.47559 | 3.465408 | 3.185406 | 3.947928 | 3.753361 |
| AABR07058 | 0 | 0 | 0.393134 | 0 | 0 | 0 | 0 | 0 |
| #N/A | 0.528949 | 0.997468 | 0.946434 | 2.274559 | 1.715834 | 1.236754 | 1.972548 | 1.719056 |
| Kin | 4.997429 | 4.40232 | 4.598623 | 5.887738 | 6.44578 | 5.915363 | 5.670772 | 5.44192 |
| Gnai2 | 186.9865 | 200.0849 | 205.6499 | 212.6712 | 211.8476 | 223.3472 | 264.2122 | 256.3543 |
| Btbd10 | 7.270896 | 7.672397 | 7.999532 | 7.864002 | 7.638882 | 8.115854 | 8.65357 | 8.341358 |
| #N/A | 0 | 0 | 0 | 0 | 0.096516 | 0.026088 | 0 | 0.052744 |
| Ucp2 | 119.8836 | 122.06 | 135.4252 | 157.7943 | 152.2212 | 157.287 | 226.462 | 217.3488 |
| Sarm1 | 0.414882 | 0.396073 | 0.584591 | 0.585393 | 0.434587 | 0.647964 | 0.362619 | 0.517121 |
| Nova1 | 0.493088 | 0.334743 | 0.465838 | 0.254442 | 0.277248 | 0.259404 | 0.441316 | 0.262229 |
| AABR07065 | 1.598274 | 1.280083 | 1.291707 | 1.37346 | 1.728191 | 1.338385 | 1.366172 | 1.957814 |
| LOC100302 | 0.167214 | 0.346858 | 0.448788 | 0.179762 | 0.271209 | 0.244356 | 0.155893 | 0.172912 |
| Ccl9 | 0.081125 | 0.045894 | 0 | 0.149506 | 0.131578 | 0.320085 | 0.090759 | 0.359524 |
| Timp2 | 76.23167 | 82.07903 | 83.97186 | 126.3413 | 125.6536 | 130.8328 | 165.3491 | 162.9398 |
| Ptger1 | 7.212562 | 7.637159 | 8.415193 | 6.219739 | 6.827663 | 6.968311 | 10.4746 | 11.02465 |
| Parm1 | 57.34042 | 56.29617 | 57.91791 | 59.74714 | 59.60691 | 61.45694 | 64.06613 | 66.51641 |
| Csf2rb | 1.28833 | 1.653228 | 2.344532 | 1.795196 | 1.817764 | 1.556657 | 2.232299 | 2.687669 |
| Vps26a | 34.15169 | 35.71138 | 31.82341 | 34.34899 | 31.3765 | 30.96194 | 36.70906 | 38.97553 |
| Slitrk2 | 0.106615 | 0.100525 | 0.076306 | 0.016374 | 0.134495 | 0.0779 | 0.198794 | 0.0315 |
| Tigd5 | 0.789645 | 1.034516 | 0.669264 | 0.765923 | 0.674078 | 0.710581 | 0.906667 | 0.828831 |
| Bckdhb | 89.23938 | 87.64495 | 82.48341 | 89.04544 | 89.74706 | 91.9519 | 74.97582 | 73.41199 |

| | | | | | | | | |
|---|---|---|---|---|---|---|---|---|
| AABR07044 | 0 | 0 | 0 | 0 | 0 | 0.143483 | 0.366154 | 0.145045 |
| Rasgrp3 | 28.08383 | 28.06376 | 26.56209 | 24.97411 | 24.43255 | 24.7449 | 23.75286 | 22.98986 |
| LOC289035 | 0.059204 | 0 | 0.063559 | 0 | 0.032008 | 0.025955 | 0.033117 | 0 |
| Enpp6 | 0.031756 | 0 | 0 | 0.029262 | 0.103012 | 0.111375 | 0.106582 | 0.056294 |
| AABR07051 | 0 | 0 | 0 | 0 | 0.186873 | 0 | 0 | 0 |
| AABR07034 | 1.468203 | 0 | 0 | 0 | 0 | 0.429107 | 0 | 0.867561 |
| Relt | 2.252298 | 1.966676 | 2.050031 | 2.346108 | 2.276548 | 2.232401 | 2.464993 | 2.408608 |
| AABR07071 | 0.164846 | 0.248687 | 0.412935 | 0 | 0.297075 | 0.144537 | 0.122948 | 0.438332 |
| Hist1h4a | 3.18895 | 4.367757 | 3.243355 | 3.09315 | 5.444472 | 3.826207 | 3.379885 | 4.909228 |
| Eepd1 | 12.17542 | 10.83706 | 12.67274 | 13.76219 | 12.38774 | 15.08793 | 15.98235 | 16.81447 |
| Ptgis | 13.95077 | 14.83146 | 16.13468 | 32.23086 | 30.86182 | 32.20514 | 33.32615 | 30.97288 |
| Col6a6 | 2.859125 | 2.537399 | 2.639234 | 1.633292 | 1.708258 | 1.831174 | 2.276137 | 1.844285 |
| Mir3582 | 7.988022 | 7.531726 | 9.052077 | 11.4499 | 8.157481 | 15.56423 | 3.475362 | 12.58699 |
| Srpk3 | 19.3862 | 18.00208 | 20.20427 | 30.69791 | 29.42478 | 29.34554 | 29.43635 | 30.82892 |
| Prkra | 13.25001 | 11.40432 | 11.17914 | 13.77782 | 14.68793 | 11.50057 | 11.53771 | 12.03997 |
| Poc1b | 1.465031 | 1.738473 | 1.937234 | 1.531062 | 1.777302 | 1.911145 | 1.958819 | 1.979466 |
| P3h4 | 4.915329 | 5.052571 | 6.001203 | 7.223169 | 5.940153 | 7.351951 | 7.475835 | 8.257798 |
| Snai3 | 0.420399 | 0.564848 | 0.592364 | 0.508437 | 0.142053 | 0.575947 | 0.293952 | 0.18631 |
| Qtrt2 | 0.962104 | 0.777553 | 1.180434 | 1.562 | 0.891693 | 1.285445 | 1.537655 | 1.218229 |
| 5S_rRNA | 2.980174 | 2.890223 | 2.742349 | 2.353811 | 2.301728 | 1.866442 | 4.286683 | 5.282953 |
| #N/A | 0 | 0 | 0 | 0 | 0 | 0 | 0.210736 | 0 |
| Rpn1 | 43.53198 | 45.96719 | 42.75319 | 48.49255 | 47.4536 | 47.34349 | 51.42344 | 51.75313 |
| Nup62cl | 0 | 0.072344 | 0 | 0 | 0 | 0 | 0 | 0 |
| Gcat | 12.99981 | 12.63438 | 13.59828 | 11.21094 | 12.0021 | 12.47962 | 11.63489 | 12.34963 |
| LOC103692 | 1.498208 | 0.699586 | 0.893568 | 0.92036 | 0.74571 | 0.813199 | 0.851367 | 0.611272 |
| Gfi1b | 0.031004 | 0.070159 | 0.06657 | 0.028569 | 0.134097 | 0.027184 | 0 | 0.02748 |
| Acot9 | 19.85514 | 20.74831 | 20.03335 | 23.65058 | 23.24944 | 24.11784 | 23.94675 | 23.95126 |
| Ergic1 | 5.926654 | 7.479468 | 12.43607 | 10.31132 | 7.147836 | 10.46567 | 10.89624 | 7.769427 |
| Gas2l1 | 5.765056 | 5.566199 | 6.333569 | 5.595593 | 5.132389 | 5.543437 | 6.707676 | 7.238962 |
| Sidt1 | 0 | 0.030689 | 0.009706 | 0.008331 | 0.009776 | 0.015854 | 0.02023 | 0.056095 |
| LOC297756 | 2.276779 | 2.4902 | 1.629512 | 2.377691 | 1.969479 | 2.462081 | 1.867917 | 1.681685 |
| Nusap1 | 1.552672 | 1.47939 | 1.631801 | 1.009039 | 1.237071 | 1.132099 | 1.371364 | 1.144428 |
| Homez | 0.565635 | 0.437888 | 0.287643 | 0.466346 | 0.257521 | 0.287129 | 0.432974 | 0.263869 |
| Zfp317 | 4.131153 | 4.029487 | 3.945671 | 3.517911 | 4.004857 | 3.072623 | 3.123666 | 3.989931 |
| Gsg2 | 0.62672 | 0.892946 | 0.872179 | 0.620275 | 0.72786 | 0.854791 | 0.908893 | 0.596641 |
| Ankrd50 | 4.950323 | 4.865566 | 4.864904 | 4.359945 | 4.3457 | 4.614466 | 5.041722 | 4.63148 |
| Spdye4 | 0 | 0 | 0 | 0 | 0 | 0 | 0 | 0 |
| LOC100911 | 0.112133 | 0.063436 | 0.19261 | 0.113658 | 0.193996 | 0.226131 | 0.250898 | 0.298166 |
| AABR07017 | 2.033628 | 1.53397 | 2.729037 | 3.279339 | 2.748666 | 2.228859 | 3.791888 | 5.107099 |
| Arap3 | 18.64159 | 20.6287 | 20.4981 | 17.35968 | 18.66039 | 18.11567 | 21.86655 | 20.2907 |
| AC125873. | 0.698212 | 0 | 0 | 0 | 0.754967 | 0.306097 | 0.781129 | 0.61886 |
| Gbp4 | 0.837536 | 0.834819 | 0.87774 | 1.231135 | 1.42311 | 0.909198 | 2.587898 | 1.908899 |
| Krt17 | 0 | 0 | 0 | 0.031232 | 0.073298 | 0 | 0 | 0 |
| Ap5s1 | 5.872211 | 5.877499 | 6.546662 | 4.578548 | 4.605158 | 4.158613 | 4.656452 | 5.347822 |
| Isg20l2 | 3.116254 | 3.416847 | 3.000601 | 2.516268 | 3.543249 | 3.098524 | 3.845742 | 4.071949 |
| Nsmf | 6.415957 | 6.446065 | 6.001939 | 9.101125 | 9.01009 | 9.080191 | 10.36475 | 10.12294 |
| AABR07024 | 2.361104 | 1.812789 | 1.538983 | 1.243237 | 1.002975 | 1.330855 | 1.132071 | 2.541214 |
| Fam135a | 6.539721 | 6.483998 | 5.769677 | 6.327844 | 6.373547 | 6.426508 | 6.292706 | 6.983093 |
| AC119762. | 0.385044 | 1.742635 | 0.413369 | 0.354802 | 0 | 0.337607 | 0 | 0.341283 |
| Nek9 | 21.26341 | 23.62561 | 22.55377 | 21.89356 | 20.23358 | 21.50344 | 22.44319 | 24.88684 |
| Trim30c | 0.247278 | 0.23674 | 0.081683 | 0.333022 | 0.164541 | 0.183458 | 0.170242 | 0.303473 |
| Aasdhppt | 25.3289 | 25.01403 | 26.72065 | 20.91119 | 21.74136 | 23.49213 | 22.93156 | 23.53168 |
| Aldh1a3 | 1.345889 | 1.52281 | 1.51133 | 3.207372 | 2.927309 | 3.485974 | 3.617192 | 3.619921 |
| Gale | 3.012009 | 3.067151 | 3.741718 | 3.925276 | 4.838736 | 3.885942 | 5.247106 | 5.148693 |

| | | | | | | | | |
|---|---|---|---|---|---|---|---|---|
| Kif5a | 0.545478 | 0.478632 | 0.645362 | 0.718053 | 0.674078 | 0.605165 | 0.759708 | 0.680825 |
| LOC100909 | 1.282907 | 1.539521 | 1.669433 | 1.61202 | 1.723477 | 1.874756 | 3.087982 | 2.446496 |
| Car8 | 6.453547 | 5.789351 | 5.592123 | 3.695443 | 4.186876 | 2.990908 | 3.816255 | 4.044927 |
| Bzw1 | 40.19534 | 37.51028 | 35.11658 | 40.45986 | 38.68302 | 39.53245 | 39.1004 | 39.12715 |
| AABR07051 | 0 | 0 | 0 | 0.742356 | 0 | 0 | 2.703908 | 0 |
| AABR07024 | 0 | 0 | 0 | 0 | 0 | 0 | 0 | 0 |
| Sumf2 | 3.914552 | 4.270001 | 3.925709 | 3.628705 | 4.207402 | 3.514493 | 4.484323 | 3.677426 |
| AC131806. | 2.731102 | 2.821408 | 4.33428 | 2.844856 | 3.466684 | 2.08229 | 2.258366 | 3.894189 |
| Nphs1 | 0 | 0 | 0.019379 | 0.041583 | 0.029277 | 0.039568 | 0.070681 | 0.039999 |
| Pml | 4.04551 | 4.420144 | 4.175354 | 5.023702 | 4.956347 | 4.810669 | 7.109412 | 7.54082 |
| Il27ra | 0.514978 | 0.432843 | 0.379105 | 0.203371 | 0.190916 | 0.206415 | 0.230454 | 0.221705 |
| Abca17 | 0.00984 | 0.033399 | 0 | 0.0272 | 0.031918 | 0.051764 | 0.077056 | 0.043606 |
| Sec14l4 | 0.082104 | 0.018579 | 0.052886 | 0.075656 | 0.035511 | 0.086387 | 0.091854 | 0.058218 |
| Akap2 | 50.14273 | 50.44553 | 48.53908 | 43.84422 | 43.63618 | 44.68558 | 55.93524 | 59.3943 |
| Sag | 0 | 0 | 0.047602 | 0 | 0.095889 | 0 | 0.049606 | 0 |
| Plek2 | 0.522398 | 0.24458 | 0.386778 | 0.248984 | 0.331126 | 0.315889 | 0.302294 | 0.207564 |
| AABR07032 | 0 | 0 | 0.077117 | 0.066191 | 0 | 0 | 0 | 0 |
| Thegl | 0.039373 | 0 | 0 | 0.072561 | 0.085147 | 0 | 0.022024 | 0.069796 |
| AABR07041 | 0 | 0.110643 | 0.052491 | 0 | 0 | 0.042871 | 0 | 0 |
| Zeb2 | 5.500922 | 5.18939 | 4.847187 | 7.438751 | 7.81746 | 6.827666 | 7.081312 | 7.788521 |
| Bnip3l | 17.84272 | 17.5767 | 16.39623 | 19.33746 | 18.39037 | 19.77811 | 19.48549 | 19.84841 |
| Hbq1 | 0.606087 | 0.548607 | 0.650673 | 0.446788 | 0.393212 | 0.637701 | 0.406838 | 0.214882 |
| Slc6a1 | 0.025688 | 0 | 0 | 0 | 0.013888 | 0.011262 | 0 | 0 |
| LOC100910 | 0 | 0 | 0 | 0 | 0 | 0.071629 | 0.091396 | 0 |
| Spon2 | 0.305584 | 0.188593 | 0.566655 | 0.255985 | 0.450577 | 0.438441 | 0.155397 | 0.344723 |
| Farp1 | 2.512738 | 2.453587 | 2.365006 | 2.875735 | 3.101584 | 2.987862 | 3.671169 | 3.111929 |
| Pafah1b1 | 41.55153 | 43.39843 | 39.45515 | 40.89926 | 42.13078 | 38.84097 | 46.42131 | 46.2223 |
| Dym | 7.576866 | 9.425545 | 8.921435 | 8.745998 | 9.382027 | 8.768577 | 8.727315 | 9.73062 |
| AC106606. | 0 | 0 | 0 | 0 | 0 | 0 | 0 | 0 |
| Ganc | 5.023475 | 4.502771 | 4.202354 | 5.079805 | 5.196667 | 4.928943 | 4.61038 | 4.895884 |
| RGD156202 | 0 | 0 | 0 | 0 | 0.053217 | 0 | 0.018354 | 0.014541 |
| Ehd2 | 78.53596 | 81.40605 | 87.39192 | 65.01199 | 65.78491 | 68.31677 | 84.42319 | 80.18927 |
| Tagln3 | 0 | 0 | 0 | 0 | 0 | 0 | 0 | 0 |
| Olr1640 | 0 | 0.062898 | 0.05968 | 0 | 0 | 0 | 0 | 0 |
| Cubn | 0.084818 | 0.063979 | 0.070823 | 0.056447 | 0.096808 | 0.053711 | 0.052717 | 0.133651 |
| AABR07003 | 0.297957 | 0.252843 | 0.239907 | 0.068639 | 0.241632 | 0.261249 | 0.333341 | 0.066023 |
| Ndrg4 | 93.58627 | 94.55951 | 99.47045 | 135.3984 | 130.1172 | 137.0386 | 144.6424 | 142.353 |
| AABR07042 | 0 | 0 | 0 | 0 | 0 | 0 | 0 | 0 |
| AC121639. | 2.374872 | 4.702347 | 3.951844 | 4.595537 | 3.466684 | 2.394633 | 4.782422 | 5.05192 |
| Zfp956 | 0.719582 | 0.629134 | 0.948089 | 1.205575 | 1.131743 | 1.032431 | 0.658666 | 1.014683 |
| AABR07003 | 0 | 0 | 0 | 0.159251 | 0 | 0 | 0 | 0 |
| Cldn7 | 0 | 0 | 0 | 0.041419 | 0 | 0.039412 | 0 | 0 |
| AC141966. | 0 | 1.150477 | 0 | 0.936954 | 0 | 0.891543 | 1.137566 | 0 |
| Sptssb | 0.024043 | 0 | 0 | 0 | 0 | 0 | 0 | 0 |
| AABR07017 | 0.01455 | 0.032926 | 0.031241 | 0.013407 | 0.015733 | 0.05103 | 0 | 0.03869 |
| Vopp1 | 28.04474 | 28.13481 | 26.48084 | 27.633 | 27.43727 | 26.51669 | 29.97308 | 30.17024 |
| Tuba4a | 171.1657 | 169.8106 | 183.8349 | 149.4248 | 149.1424 | 147.2878 | 158.3162 | 160.2711 |
| LOC100910 | 0 | 0 | 0.058017 | 0 | 0.058434 | 0 | 0 | 0.047899 |
| LOC100912 | 0 | 0 | 0.120899 | 0 | 0 | 0 | 0 | 0 |
| Stom | 12.70613 | 12.23171 | 13.12652 | 14.10742 | 13.15336 | 14.09944 | 17.2911 | 16.45001 |
| Kat14 | 3.136542 | 2.70217 | 2.820309 | 2.787502 | 3.150477 | 2.875763 | 2.93903 | 3.146986 |
| AABR07054 | 0.969739 | 0 | 0 | 0 | 0 | 0 | 0 | 0 |
| B3gat2 | 0 | 0.029684 | 0 | 0.024175 | 0.085104 | 0 | 0 | 0.023254 |
| Cdc42ep4 | 12.32357 | 11.36838 | 11.36282 | 9.906386 | 10.44415 | 9.766968 | 15.17405 | 14.44918 |

| | | | | | | | | |
|---|---|---|---|---|---|---|---|---|
| Gpc3 | 1.190135 | 0.897721 | 1.429791 | 2.34999 | 1.807753 | 1.888258 | 1.648486 | 1.431617 |
| Leprotl1 | 17.67744 | 17.42168 | 17.77292 | 19.19783 | 19.53154 | 19.95881 | 21.2286 | 19.46114 |
| Tmed2 | 74.13602 | 72.07992 | 69.4257 | 79.62507 | 79.32357 | 79.49444 | 81.26453 | 82.28028 |
| Tsn | 17.80441 | 18.02434 | 17.27969 | 18.33619 | 17.04636 | 17.93081 | 18.25375 | 19.1521 |
| Ddx49 | 17.01039 | 17.91185 | 18.29506 | 20.13646 | 19.63496 | 17.58195 | 20.03755 | 20.30463 |
| Terf1 | 4.480368 | 4.395369 | 4.670945 | 4.963725 | 5.600644 | 4.700444 | 4.838595 | 4.981182 |
| AABR07066 | 0 | 0 | 0.26518 | 0 | 0 | 0 | 0 | 0 |
| AABR07011 | 0 | 0.575239 | 0 | 0 | 0 | 0.445772 | 0 | 0.450626 |
| Moap1 | 1.969515 | 1.441914 | 2.145494 | 1.094236 | 1.471935 | 1.676082 | 1.360927 | 1.46329 |
| Ccdc42 | 0.01596 | 0 | 0 | 0 | 0 | 0 | 0 | 0 |
| AABR07070 | 0 | 0.072699 | 0 | 0 | 0 | 0 | 0.143766 | 0 |
| Pdc | 0 | 0.13974 | 0 | 0.07587 | 0.044515 | 0.072193 | 0.092114 | 0.036489 |
| AC120448. | 0 | 0.462887 | 0 | 0.376978 | 0.442363 | 0.358707 | 0.915385 | 1.08784 |
| Cadm4 | 0.583464 | 1.402845 | 1.761711 | 2.18416 | 1.537798 | 2.302119 | 1.631885 | 1.713071 |
| Ube2e1 | 28.21291 | 28.65157 | 26.59466 | 28.97724 | 30.13419 | 29.32252 | 30.25463 | 27.2022 |
| AABR07067 | 0 | 0 | 0 | 0 | 0 | 0 | 0 | 0 |
| Ostc | 37.54538 | 37.5061 | 37.49645 | 42.65395 | 40.11652 | 42.49256 | 44.54645 | 42.91154 |
| Dlx6 | 0.297534 | 0 | 0 | 0.456943 | 0.214479 | 0.173919 | 0.554779 | 0.615344 |
| Rn50_10_0 | 1.063267 | 0.541367 | 1.027337 | 1.371663 | 0.689817 | 1.025501 | 1.665351 | 1.74349 |
| Cpsf7 | 11.19016 | 10.65204 | 10.59261 | 12.10187 | 11.10352 | 11.77972 | 14.18701 | 14.20943 |
| Pdlim1 | 45.74453 | 45.99797 | 46.7784 | 48.05632 | 47.30732 | 50.56524 | 56.51369 | 55.18626 |
| Il21r | 0.523441 | 0.542895 | 0.702434 | 0.884272 | 1.320642 | 0.68843 | 1.342006 | 0.927904 |
| Sebox | 0 | 0.104497 | 0 | 0 | 0.099863 | 0 | 0 | 0 |
| AABR07005 | 1.039272 | 0.682774 | 0.575858 | 0.463378 | 0.435 | 0.338039 | 0.393815 | 0.326863 |
| Ifrd2 | 16.46475 | 16.20579 | 17.81966 | 14.3822 | 14.85038 | 13.75557 | 13.41816 | 15.02065 |
| Twf1 | 4.864892 | 5.483781 | 4.772871 | 6.312867 | 6.462138 | 6.709841 | 6.461844 | 7.493506 |
| Prps1 | 11.39309 | 11.81402 | 11.40823 | 11.74054 | 10.08973 | 10.61526 | 11.79974 | 11.76178 |
| Lect2 | 0.063244 | 0 | 0 | 0 | 0.136769 | 0.110905 | 0.070754 | 0 |
| Tubb1 | 0.152492 | 0.207046 | 0.196453 | 0.084309 | 0.197866 | 0.106964 | 0.204722 | 0.162194 |
| Crebrf | 12.54801 | 11.76519 | 11.61704 | 13.19839 | 14.06422 | 12.79167 | 14.15976 | 14.1727 |
| AABR07063 | 0.105576 | 0 | 0 | 0.389138 | 0 | 0 | 0 | 0.093578 |
| Rnf38 | 8.999945 | 9.437125 | 8.246561 | 9.059018 | 7.995954 | 9.549811 | 9.170889 | 9.958671 |
| #N/A | 0.191117 | 0.432479 | 0.205176 | 0 | 0.206652 | 0 | 0 | 0 |
| AABR07070 | 0 | 0 | 0 | 0 | 0 | 0 | 0 | 0.122466 |
| AC125873. | 0.385044 | 3.049611 | 0.826738 | 0.709605 | 1.665368 | 2.363246 | 1.723078 | 0.341283 |
| Tmem40 | 0 | 0 | 0.092312 | 0.079233 | 0 | 0.075393 | 0 | 0.152429 |
| Ccna2 | 0.881488 | 0.529849 | 0.414021 | 0.558427 | 0.565927 | 0.410598 | 0.523903 | 0.952218 |
| Fabp1 | 1.185237 | 1.219127 | 1.966479 | 0.893577 | 0.466029 | 0.755794 | 0.843812 | 1.52805 |
| Uhrf1bp1l | 1.551088 | 1.335131 | 1.720964 | 1.791712 | 1.693219 | 1.73741 | 2.532359 | 2.3944 |
| AC130253. | 0 | 0 | 0.826738 | 0.709605 | 0 | 0.337607 | 2.153848 | 0 |
| Hes6 | 10.85529 | 10.46448 | 11.56062 | 12.56342 | 10.7517 | 10.92658 | 9.326912 | 9.121261 |
| Fam189b | 2.830127 | 3.240969 | 3.97744 | 3.208446 | 3.412559 | 3.534197 | 3.41568 | 3.496672 |
| Defb1 | 0.57545 | 1.953283 | 1.235564 | 2.333119 | 2.862237 | 1.110021 | 1.54509 | 1.428139 |
| Zbtb37 | 2.022612 | 1.841956 | 1.827156 | 1.318267 | 1.573588 | 1.340885 | 1.434952 | 1.902055 |
| Ric8b | 8.694178 | 7.954053 | 7.947554 | 6.054777 | 6.856754 | 5.8368 | 7.896892 | 8.443627 |
| Otol1 | 0.072279 | 0.08178 | 0 | 0 | 0 | 0 | 0 | 0 |
| LOC690126 | 0.127411 | 0 | 0.273568 | 0.234808 | 0 | 0.111714 | 0 | 0.112931 |
| Wipi2 | 8.578143 | 9.356866 | 8.938327 | 9.84178 | 8.093261 | 8.258708 | 9.65957 | 8.969829 |
| Trpc7 | 0.051423 | 0.13576 | 0.055206 | 0.047384 | 0.092672 | 0.090176 | 0.019177 | 0.197509 |
| Nt5c1b | 0 | 0.156331 | 0 | 0.07639 | 0.08964 | 0 | 0.216408 | 0.097973 |
| RGD156485 | 0 | 0 | 0 | 0 | 0 | 0 | 0 | 0 |
| LOC100361 | 13.90429 | 12.67957 | 12.1051 | 13.51343 | 13.68814 | 14.25351 | 10.60251 | 12.75326 |
| Zfp871 | 4.876377 | 4.214289 | 3.709294 | 2.98054 | 4.557357 | 3.136881 | 4.907185 | 5.560186 |
| Psme3 | 27.60579 | 25.79099 | 24.6604 | 22.05858 | 20.9995 | 23.3045 | 25.69848 | 26.6786 |

| | | | | | | | | |
|---|---|---|---|---|---|---|---|---|
| Cox6a1 | 356.6415 | 373.4126 | 388.6119 | 387.2556 | 384.8062 | 351.1747 | 363.6731 | 354.5765 |
| Cda | 0.166417 | 0.188293 | 0.297766 | 0.357809 | 0.299907 | 0.340468 | 0.3103 | 0.344175 |
| B3gnt7 | 1.283276 | 1.615898 | 1.577664 | 1.010836 | 1.52187 | 1.379249 | 1.829324 | 1.687801 |
| Mbd6 | 11.27112 | 13.30397 | 13.04736 | 11.69626 | 11.01982 | 10.5175 | 15.01076 | 15.05525 |
| Zfp260 | 0.529382 | 0.372333 | 0.445444 | 0.342782 | 0.371295 | 0.664882 | 0.288121 | 0.329721 |
| Chgb | 0 | 0 | 0.022194 | 0 | 0 | 0 | 0.023129 | 0.018324 |
| Cars | 4.854775 | 4.750131 | 5.100625 | 5.380917 | 4.838413 | 4.862603 | 5.991833 | 5.987488 |
| AABR07017 | 0 | 0.127418 | 0 | 0 | 0 | 0 | 0 | 0 |
| Arhgap28 | 0.757635 | 0.907653 | 0.813369 | 0.739197 | 0.674651 | 0.625219 | 0.822678 | 0.632027 |
| Cyb561d2 | 1.824744 | 2.208655 | 2.095653 | 3.167345 | 3.533171 | 3.534746 | 2.848525 | 2.745753 |
| Minpp1 | 6.945375 | 6.536164 | 8.687185 | 7.679867 | 8.010595 | 6.863007 | 9.07754 | 8.462099 |
| Ap3s2 | 22.71581 | 22.7935 | 25.79875 | 26.82566 | 28.11734 | 26.62621 | 24.67779 | 26.70424 |
| Hs3st5 | 4.525875 | 3.855214 | 3.510171 | 3.662989 | 3.758708 | 3.953202 | 2.560552 | 3.126841 |
| Fgfrl1 | 1.098669 | 1.040414 | 0.948722 | 1.441542 | 1.342928 | 1.581092 | 1.536428 | 1.301935 |
| Tmed9 | 30.18653 | 30.36913 | 31.15608 | 38.62329 | 38.90003 | 33.55416 | 37.51437 | 40.35031 |
| Slc25a28 | 16.93477 | 18.53267 | 20.19234 | 19.66579 | 18.49894 | 19.62548 | 21.9741 | 21.53092 |
| Ppil4 | 4.67807 | 4.025107 | 3.971935 | 5.162954 | 5.558392 | 4.819149 | 4.457529 | 4.445956 |
| AABR07029 | 0 | 0 | 0 | 0 | 0.07069 | 0 | 0 | 0 |
| AC098284. | 0 | 0 | 0 | 0 | 0.3905 | 0 | 0 | 0 |
| Rps7 | 589.6313 | 590.3688 | 583.1313 | 522.5361 | 513.9582 | 488.6557 | 566.1181 | 572.5251 |
| RragB | 0 | 0.060459 | 0 | 0 | 0.057778 | 0.011713 | 0 | 0.01184 |
| Rn50_7_14 | 0 | 0.116176 | 0 | 0 | 0 | 0 | 0 | 0.091009 |
| LOC102553 | 1.009715 | 0.945472 | 3.065086 | 3.400819 | 3.237723 | 2.533845 | 14.41245 | 4.227918 |
| Manf | 44.79644 | 47.73662 | 47.35914 | 59.80187 | 58.4339 | 58.53553 | 67.2613 | 68.41192 |
| Prss53 | 1.502949 | 1.760184 | 1.585205 | 2.065215 | 1.311498 | 2.450622 | 3.126875 | 2.874615 |
| LOC100125 | 0.009353 | 0.031747 | 0.010041 | 0.034473 | 0.020226 | 0.073804 | 0.03139 | 0.01658 |
| Tomll1 | 3.122212 | 2.987155 | 2.809676 | 2.36929 | 2.507178 | 2.093427 | 2.054701 | 2.563888 |
| AC109891. | 0 | 0 | 0 | 0.020005 | 0.035213 | 0.019036 | 0.048578 | 0 |
| Sfmbt2 | 0.080285 | 0.045419 | 0.064643 | 0.129464 | 0.108514 | 0.017599 | 0.112274 | 0.177902 |
| P2rx2 | 0.032305 | 0.036551 | 0 | 0.208373 | 0.174653 | 0.226598 | 0.180705 | 0.057267 |
| AC094562. | 3.927444 | 5.184338 | 6.324543 | 3.015821 | 12.74007 | 6.313242 | 7.323083 | 8.702722 |
| AABR07040 | 0.202969 | 0.07655 | 0.290533 | 0.062343 | 0.365779 | 0.118642 | 0.075691 | 0.119934 |
| Rfwd2 | 41.5847 | 41.03904 | 43.07303 | 37.32522 | 39.67739 | 36.66407 | 40.36311 | 40.20316 |
| Fscn3 | 0.116563 | 0.131886 | 0.28156 | 0.214816 | 0.157547 | 0.229956 | 0.423818 | 0.180802 |
| Cenpa | 0.332482 | 0.352676 | 0.133853 | 0.459554 | 0.426916 | 0.382621 | 0.488206 | 0.331532 |
| Mir23a | 0 | 5.52996 | 0 | 0.643375 | 0 | 1.836579 | 0.781129 | 0.61886 |
| Clns1a | 24.67903 | 24.6019 | 20.93105 | 23.64237 | 24.88256 | 23.83105 | 22.66355 | 22.06697 |
| AABR07030 | 0 | 0 | 0.497506 | 0 | 0 | 0 | 0 | 0 |
| Bola3 | 75.31494 | 78.49349 | 86.30748 | 74.07938 | 66.67262 | 70.11096 | 76.80862 | 72.78544 |
| Tbc1d19 | 3.403236 | 3.571122 | 2.975909 | 3.641746 | 3.887604 | 3.128345 | 2.978361 | 3.965182 |
| B4galt2 | 4.720455 | 3.81498 | 5.01202 | 4.540909 | 4.403038 | 4.661946 | 4.932835 | 4.942606 |
| Rn60_14_0 | 0.631866 | 0.983025 | 0.593555 | 1.01892 | 1.451859 | 1.177294 | 0.88363 | 1.190116 |
| Cpeb2 | 12.75367 | 14.13514 | 16.61859 | 8.540958 | 8.945793 | 10.45495 | 12.91568 | 12.12367 |
| AABR07036 | 0 | 0 | 0 | 0 | 0 | 0 | 0 | 0.446293 |
| AABR07007 | 0 | 0 | 0 | 0 | 0 | 0 | 0 | 0.08363 |
| Cdk10 | 14.09099 | 14.42488 | 14.25714 | 13.80869 | 14.11784 | 14.97798 | 14.48195 | 16.62795 |
| Mir199a2 | 0.361144 | 0.68103 | 0.387711 | 1.663901 | 1.562 | 1.266606 | 0.673387 | 1.1737 |
| Vom2r6 | 0 | 0 | 0 | 0 | 0.022416 | 0 | 0 | 0.018375 |
| Fzd8 | 0.17543 | 0.158793 | 0.169502 | 0.290974 | 0.265566 | 0.184581 | 0.157011 | 0.264337 |
| Fam24a | 0 | 0 | 0 | 0 | 0.099164 | 0 | 0 | 0 |
| Grm3 | 0.033557 | 0 | 0 | 0 | 0 | 0 | 0 | 0 |
| Sash3 | 1.106464 | 1.251912 | 1.403834 | 1.223476 | 1.47919 | 1.217095 | 1.507942 | 1.551311 |
| Gstt3 | 2.874992 | 2.323513 | 2.582571 | 4.83883 | 4.092047 | 3.292467 | 3.971285 | 3.640354 |
| Sema4d | 23.77996 | 23.54267 | 22.45744 | 19.45484 | 16.04054 | 18.21965 | 20.29485 | 18.91053 |

| | | | | | | | | |
|---|---|---|---|---|---|---|---|---|
| Izumo2 | 0 | 0 | 0 | 0 | 0 | 0 | 0.094036 | 0.074502 |
| Reep6 | 2.365707 | 2.20269 | 2.089993 | 2.679468 | 3.277444 | 2.91692 | 3.142895 | 3.538424 |
| Notch2 | 2.47289 | 2.432359 | 2.520295 | 2.813399 | 2.937725 | 2.619225 | 3.954336 | 4.079755 |
| Rhod | 0.986706 | 1.116412 | 1.361947 | 1.136514 | 1.486055 | 1.174125 | 0.98561 | 0.780863 |
| Oma1 | 4.897737 | 5.026065 | 4.188085 | 5.247758 | 5.419012 | 5.517728 | 5.256373 | 4.820649 |
| Gucy2f | 0 | 0.015859 | 0 | 0 | 0.015156 | 0.01229 | 0 | 0 |
| Rn50_X_06 | 0 | 0 | 0 | 0 | 0 | 0.418355 | 0 | 0 |
| Mcu | 12.27845 | 10.55078 | 11.79228 | 10.24385 | 11.37474 | 8.699893 | 12.51143 | 10.883 |
| AC119015. | 6.661687 | 4.007218 | 6.336991 | 4.895246 | 7.385545 | 5.545228 | 6.415068 | 5.605618 |
| AABR07052 | 0.357854 | 0.944754 | 0.448209 | 0.329748 | 0.225716 | 0.287619 | 0.266901 | 0.290752 |
| Ptk6 | 0 | 0.082983 | 0.052491 | 0.090109 | 0 | 0.021435 | 0.082051 | 0.086675 |
| Ralgapa1 | 13.29632 | 12.21423 | 11.83621 | 13.12623 | 13.22694 | 12.94378 | 14.71451 | 14.14222 |
| AABR07073 | 0 | 0 | 0 | 0 | 0 | 0.515893 | 1.974764 | 0 |
| Ms4a12 | 0 | 0 | 0.28393 | 0.243703 | 0.214479 | 0.40581 | 0.295882 | 0.351625 |
| Ralgapb | 7.852585 | 7.894732 | 7.25183 | 7.59658 | 7.594488 | 7.517807 | 8.32998 | 9.096484 |
| NEWGENE_6 | 0 | 0 | 0 | 0 | 0 | 0 | 0 | 0.110907 |
| Cnot4 | 4.990821 | 4.469735 | 4.192486 | 4.946189 | 4.026997 | 4.362735 | 5.246136 | 5.559585 |
| Nupr1l1 | 0.409109 | 11.01672 | 0.702727 | 2.337261 | 2.211817 | 1.865276 | 1.373078 | 1.08784 |
| Gnas | 197.656 | 206.1152 | 218.5345 | 187.2434 | 181.7443 | 194.011 | 204.3551 | 195.6371 |
| Gpatch1 | 3.515973 | 3.420832 | 3.118166 | 3.56851 | 3.43445 | 3.663627 | 2.98339 | 3.688471 |
| Synpr | 0.187188 | 0.052949 | 0.05024 | 0.215608 | 0.101202 | 0.041032 | 0.052354 | 0.082957 |
| Rn50_13_0 | 0.094438 | 0 | 0 | 0.04351 | 0 | 0.082803 | 0.105653 | 0.041853 |
| Tspan9 | 24.93799 | 25.42794 | 26.89653 | 22.96755 | 22.19103 | 23.81251 | 27.75592 | 25.28909 |
| AABR07012 | 0 | 0 | 0 | 0 | 0 | 0 | 0 | 0.039569 |
| AABR07067 | 0 | 0 | 0 | 0 | 0 | 0 | 0 | 0.455044 |
| AABR07065 | 0.349106 | 0.197499 | 0 | 0.160844 | 0.188742 | 0.153048 | 0.585847 | 0 |
| Atp13a3 | 5.700594 | 6.899951 | 6.262276 | 5.985832 | 5.518903 | 5.347003 | 5.784309 | 7.461574 |
| Cdc34 | 75.52296 | 78.60331 | 84.72995 | 68.85404 | 68.19579 | 69.11292 | 82.70118 | 78.90911 |
| AABR07015 | 0 | 0 | 0 | 0 | 0 | 0 | 0 | 0 |
| Cers2 | 13.57428 | 15.10532 | 13.46111 | 14.2103 | 14.88951 | 14.25779 | 16.90846 | 17.11706 |
| Tmem45a | 0.097154 | 0.036642 | 0 | 0.029841 | 0.035017 | 0 | 0.217383 | 0.028704 |
| Cpne9 | 0.376109 | 0.261877 | 0.124239 | 0.373229 | 0.375398 | 0.481975 | 0.226571 | 0.538511 |
| AABR07048 | 0.092356 | 0 | 0 | 0.042551 | 0 | 0.040489 | 0 | 0.04093 |
| Lcn10 | 0 | 0.052156 | 0 | 0 | 0.099688 | 0 | 0 | 0.040858 |
| AABR07012 | 0 | 0 | 0 | 0 | 0 | 0.170054 | 0 | 0 |
| Hmgcll1 | 0.460062 | 0.489919 | 0.334113 | 0.436398 | 0.351147 | 0.332198 | 0.272487 | 0.323822 |
| AABR07014 | 0.187691 | 0.318546 | 0 | 0 | 0.202948 | 0.082284 | 0 | 0 |
| AABR07058 | 0 | 0.030199 | 0.114614 | 0.147563 | 0.115438 | 0.093608 | 0.02986 | 0.07097 |
| AC097153. | 0.467553 | 0 | 0 | 0 | 1.516674 | 0.409951 | 0 | 0.414415 |
| LOC684828 | 3.317298 | 3.485269 | 4.070093 | 4.65792 | 4.782595 | 5.263199 | 6.097047 | 5.110497 |
| Gzmc | 0.060122 | 0 | 0.064544 | 0 | 0.065009 | 0 | 0.067261 | 0 |
| LOC685157 | 0 | 0.088963 | 0.168823 | 0.579617 | 0.085019 | 0.137881 | 0.087965 | 0.696915 |
| Cyp4a1 | 0.063978 | 0.18097 | 0.068684 | 0.117906 | 0.207535 | 0.084144 | 0.143151 | 0.17012 |
| Olfm4 | 0.019175 | 0.065086 | 0.020585 | 0.053006 | 0.290266 | 0.117686 | 0.021452 | 0.033991 |
| RGD156636 | 0.668146 | 1.700945 | 0.627635 | 1.539175 | 0.722456 | 1.025204 | 0.747492 | 1.480527 |
| Utp20 | 1.323391 | 1.2422 | 1.255097 | 1.350694 | 1.270541 | 1.373688 | 1.799234 | 1.535929 |
| Ddb1 | 36.10974 | 36.18476 | 36.45687 | 33.68379 | 34.20609 | 35.15125 | 39.30539 | 39.35811 |
| Tle3 | 5.138866 | 5.652874 | 6.12989 | 6.192266 | 5.5922 | 5.622575 | 7.149565 | 7.425934 |
| Foxl1 | 0.153117 | 0 | 0.164381 | 0.188121 | 0 | 0.179004 | 0.0571 | 0.180953 |
| Tcerg1 | 8.686349 | 9.982418 | 8.819839 | 9.945216 | 10.38392 | 9.843475 | 11.42304 | 11.25766 |
| AABR07066 | 1.57899 | 1.013269 | 1.189133 | 0.673199 | 0.560619 | 0.537253 | 1.529213 | 1.315983 |
| Slc26a5 | 0.07029 | 0 | 0.075461 | 0.172718 | 0.126672 | 0.164347 | 0.183487 | 0.083068 |
| Mipol1 | 1.876915 | 2.088247 | 1.947822 | 1.729503 | 2.232429 | 1.755393 | 2.169802 | 2.162684 |
| Slc7a4 | 1.758142 | 1.838172 | 1.935262 | 1.168903 | 1.082878 | 0.956145 | 1.244893 | 0.966558 |

| | | | | | | | | |
|---|---|---|---|---|---|---|---|---|
| AC112801. | 0 | 0.665726 | 0 | 0 | 0 | 0 | 0 | 0 |
| Camk2g | 0.942915 | 1.03594 | 1.320364 | 0.742937 | 0.66493 | 1.186204 | 0.550378 | 1.017437 |
| Fam49b | 15.40405 | 13.12715 | 13.38127 | 13.65246 | 18.0124 | 14.98407 | 13.85685 | 12.57639 |
| Sub1 | 83.12051 | 84.48551 | 77.48588 | 71.86909 | 70.62835 | 76.94928 | 74.47072 | 74.47197 |
| AABR07049 | 0 | 0.553734 | 0 | 0 | 0 | 0 | 1.09504 | 0 |
| Stk32a | 0.052282 | 0.011831 | 0.011226 | 0.028906 | 0 | 0.045841 | 0.035095 | 0.055608 |
| LOC100362 | 5.787812 | 6.288771 | 4.882104 | 6.200951 | 6.556291 | 5.35669 | 4.6508 | 5.231812 |
| Kctd19 | 0.056474 | 0.015975 | 0.015157 | 0.130097 | 0.030532 | 0.099034 | 0.031591 | 0.06257 |
| Nab2 | 1.540174 | 1.709122 | 1.632277 | 1.52838 | 1.900228 | 1.783255 | 2.319529 | 2.15271 |
| AABR07071 | 0.124681 | 0 | 0.133853 | 0 | 0.134816 | 0 | 0 | 0.110511 |
| Mtmr11 | 3.184805 | 3.254031 | 2.652387 | 5.495842 | 5.530765 | 5.923358 | 6.370246 | 6.364246 |
| Thumpd3 | 9.578809 | 10.50864 | 8.209726 | 8.875261 | 9.985476 | 9.697956 | 10.83476 | 10.46027 |
| RT1-N2 | 24.50941 | 25.77662 | 24.51575 | 24.97224 | 25.27582 | 23.43059 | 23.49426 | 23.35081 |
| Tceb3 | 11.95521 | 12.18596 | 11.46586 | 11.55532 | 11.07797 | 10.5744 | 13.4253 | 12.41798 |
| Sftpc | 0.057419 | 0 | 0.061643 | 0.105818 | 0 | 0 | 0.064238 | 0.152679 |
| Mir148b | 0 | 0.61082 | 0 | 0 | 0 | 0 | 0 | 0 |
| Zfp512b | 2.759975 | 2.914596 | 2.923502 | 2.89078 | 2.437195 | 2.565145 | 2.42902 | 2.690933 |
| Kmt5a | 54.79778 | 55.09171 | 54.59369 | 45.31512 | 43.82547 | 41.74475 | 58.52421 | 59.63475 |
| Slc25a46 | 13.49264 | 12.90411 | 14.09622 | 12.51243 | 13.07821 | 12.0119 | 12.58557 | 14.03905 |
| LOC688932 | 0.675515 | 0.703168 | 0.754217 | 0.47307 | 0.671991 | 0.497525 | 8.554933 | 0.838239 |
| Akr1c12 | 0 | 0 | 0.045558 | 0 | 0.137656 | 0.074416 | 0 | 0 |
| Rps6ka6 | 0 | 0 | 0.051958 | 0 | 0 | 0 | 0 | 0 |
| Tnfrsf1a | 15.24888 | 16.31028 | 16.21273 | 19.74403 | 18.58258 | 17.88429 | 22.29838 | 21.51235 |
| Mapkbp1 | 2.452618 | 1.964617 | 2.407796 | 2.193324 | 2.30777 | 2.296359 | 2.857196 | 2.783076 |
| AABR07048 | 0.502311 | 0 | 0 | 0 | 0 | 0.110107 | 0 | 0 |
| Hmx1 | 0 | 0 | 0.056106 | 0.096314 | 0 | 0 | 0.175403 | 0.046322 |
| AABR07016 | 0.358262 | 0.202678 | 0.512822 | 0.165062 | 0.322819 | 0.26177 | 0.334006 | 0.476318 |
| Nup160 | 2.349205 | 2.029757 | 1.834198 | 1.784238 | 1.97055 | 1.997368 | 2.182191 | 2.359846 |
| Ankrd13d | 0.38694 | 0.826965 | 0.392327 | 0.59425 | 0.557857 | 0.885871 | 0.841734 | 0.971733 |
| Smim14 | 26.85817 | 25.46543 | 22.01475 | 39.22007 | 37.96466 | 36.09133 | 36.2587 | 38.83392 |
| Tpra1 | 17.98901 | 17.83171 | 19.98643 | 18.83261 | 16.56582 | 18.25018 | 19.10675 | 20.39679 |
| Nlk | 4.126602 | 4.881286 | 4.40648 | 4.168518 | 4.97505 | 4.517923 | 5.159796 | 4.880077 |
| Syt6 | 0.273119 | 0.061804 | 0.117284 | 0.151001 | 0.255943 | 0.143682 | 0.061111 | 0.161386 |
| Mrc1 | 6.024132 | 5.604537 | 5.360761 | 8.861315 | 9.089033 | 8.861768 | 13.53504 | 13.57933 |
| Alpl | 18.4204 | 19.29416 | 20.92578 | 33.27513 | 31.60212 | 35.96002 | 32.03411 | 31.96681 |
| Grm2 | 0 | 0.012937 | 0.012275 | 0 | 0 | 0 | 0 | 0 |
| Slc26a6 | 4.771085 | 4.52863 | 4.608301 | 3.741589 | 3.230196 | 4.501171 | 3.731507 | 3.791826 |
| Pbp2 | 0 | 0 | 0 | 0 | 0 | 0.035455 | 0 | 0.107524 |
| Mpzl1 | 11.21964 | 11.07228 | 10.87227 | 9.877109 | 9.031057 | 9.91721 | 8.452894 | 8.976297 |
| AABR07049 | 0 | 0 | 0 | 0 | 0 | 0 | 0.323672 | 0 |
| Atrn | 11.26543 | 11.69373 | 11.33384 | 12.22228 | 12.68085 | 12.82448 | 14.53034 | 14.39515 |
| AABR07051 | 2.706787 | 4.476111 | 4.358863 | 2.206406 | 2.589101 | 2.555876 | 3.727056 | 3.59874 |
| Fbxl22 | 6.058479 | 5.712403 | 6.403324 | 9.845371 | 9.839114 | 9.110834 | 8.853378 | 9.043546 |
| Scd4 | 0.14751 | 0 | 0 | 0.067962 | 0.23925 | 0.129337 | 0.082514 | 0.130745 |
| Klk1c10 | 0 | 0 | 0 | 0 | 0 | 0 | 0 | 0 |
| NEWGENE_1 | 0 | 0.021831 | 0 | 0.035559 | 0 | 0.033835 | 0 | 0 |
| AABR07037 | 0.513391 | 0.580878 | 1.653475 | 0.47307 | 0.555123 | 1.800568 | 1.723078 | 0 |
| Ccdc83 | 0 | 0 | 0.063739 | 0 | 0.128396 | 0.104114 | 0.199268 | 0 |
| 43160 | 1.024668 | 1.006816 | 0.521075 | 0.969038 | 0.787234 | 0.89843 | 0.96535 | 1.075517 |
| AC130162. | 0 | 0 | 0 | 0 | 0 | 0 | 0 | 0 |
| Dhrs7 | 22.39039 | 21.8457 | 20.77154 | 17.79124 | 20.39463 | 17.64027 | 18.33323 | 18.875 |
| Agbl3 | 0.511429 | 0.415124 | 0.52518 | 0.501997 | 0.685241 | 0.789612 | 0.634356 | 0.55185 |
| Rapgef1 | 17.84143 | 18.73364 | 19.45129 | 14.65345 | 14.7853 | 14.95892 | 21.77732 | 20.64603 |
| Hnrnpa1 | 103.2743 | 97.7437 | 96.25404 | 112.6234 | 111.323 | 112.464 | 100.0672 | 104.5508 |

| | | | | | | | | |
|---|---|---|---|---|---|---|---|---|
| Etnppl | 0.173896 | 0.170521 | 0.21158 | 0.106826 | 0.100283 | 0.040659 | 0.064849 | 0.082204 |
| 5S_rRNA | 0 | 0 | 0 | 0 | 0 | 0 | 0 | 0.539704 |
| Rn60_1_04 | 0 | 0 | 0 | 0 | 0 | 0 | 0 | 0 |
| R3hdm2 | 36.36686 | 41.94897 | 41.4189 | 34.44904 | 33.22663 | 35.39349 | 42.65056 | 43.6804 |
| G6b | 0.667554 | 0.503537 | 0.637033 | 0.751819 | 0.641615 | 0.910486 | 1.161736 | 1.249116 |
| Gal3st1 | 0.030113 | 0 | 0.064656 | 0.055495 | 0.227923 | 0.079208 | 0.168444 | 0.133452 |
| Rab9b | 0.081694 | 0.107839 | 0.029235 | 0.163102 | 0.014722 | 0.071629 | 0 | 0.07241 |
| Emc4 | 22.043 | 22.31748 | 20.58304 | 23.56706 | 21.16878 | 21.65047 | 25.77231 | 25.70249 |
| Adgrl4 | 8.612895 | 8.788345 | 7.78106 | 6.979173 | 7.575792 | 7.276413 | 7.351801 | 7.505554 |
| Rn50_15_( | 0.312819 | 0 | 0.201499 | 0.05765 | 0.270597 | 0.164568 | 0.349968 | 0.055453 |
| Rn60_2_18 | 1.832455 | 1.993593 | 1.81593 | 2.208084 | 1.600367 | 2.039271 | 2.523162 | 2.498763 |
| AABR07042 | 0 | 0.284171 | 0.94371 | 0 | 0.407356 | 0.110107 | 0.280982 | 0.779141 |
| Yars2 | 6.466169 | 7.73315 | 6.078611 | 6.760996 | 6.122332 | 5.992678 | 7.234063 | 6.265811 |
| AABR07045 | 1.574915 | 0 | 0.422693 | 3.628055 | 2.128666 | 2.07133 | 0.440486 | 1.744907 |
| Tppp | 15.11695 | 15.65083 | 13.259 | 12.60955 | 12.49969 | 12.8214 | 16.19369 | 18.34687 |
| Rpl22l1 | 92.856 | 95.01093 | 88.94516 | 72.03504 | 72.1937 | 69.36367 | 79.40315 | 77.41279 |
| Slc25a38 | 11.60592 | 11.24061 | 11.6955 | 11.0081 | 11.24419 | 11.64144 | 12.25708 | 12.6186 |
| Vps4a | 23.08183 | 22.00022 | 22.86936 | 22.54707 | 22.10004 | 21.93616 | 22.5731 | 24.72657 |
| Mlst8 | 4.900671 | 5.989127 | 5.32363 | 5.011572 | 4.088268 | 4.526421 | 5.677881 | 5.748646 |
| LOC100912 | 0.048397 | 0.164278 | 0 | 0.044596 | 0 | 0.08487 | 0.054145 | 0.042897 |
| Cetn4 | 0.623404 | 0.587793 | 0.446176 | 1.244625 | 0.674078 | 0.364401 | 0.348718 | 0.644646 |
| AC106225. | 0.418927 | 2.84398 | 0.449745 | 0.77205 | 0.45298 | 1.836579 | 1.406032 | 2.227897 |
| Dtx4 | 3.397099 | 3.352978 | 3.608205 | 3.729711 | 3.673234 | 2.820144 | 3.396213 | 4.580591 |
| Wdfy3 | 6.396489 | 6.760199 | 7.431663 | 6.099315 | 6.019856 | 6.401938 | 7.611978 | 8.155718 |
| Mir423 | 0 | 0 | 0 | 0 | 0 | 0 | 1.246482 | 0.493771 |
| Apoh | 0 | 0 | 0 | 0 | 0 | 0.037238 | 0 | 0 |
| AABR07044 | 0 | 0 | 0 | 0.049339 | 0 | 0 | 0.119805 | 0.094917 |
| AC128721. | 0 | 0 | 0 | 0 | 0 | 0 | 0 | 0 |
| AABR07068 | 0.177032 | 0.080121 | 0.038011 | 0.097877 | 0.076569 | 0.186266 | 0.039611 | 0.188294 |
| AABR07071 | 0 | 0.074528 | 0.070715 | 0.060696 | 0.142447 | 0.346524 | 0 | 0.058383 |
| AABR07000 | 0 | 0 | 0.520539 | 0 | 0 | 0.425134 | 0 | 0.429764 |
| Tnfsf14 | 0.654574 | 0.493746 | 0.390404 | 0.268073 | 0 | 0.12754 | 0.081368 | 0.257858 |
| Tor3a | 4.226326 | 4.945653 | 5.003385 | 5.521503 | 5.414978 | 5.152372 | 6.865644 | 6.055183 |
| LOC103692 | 0.173685 | 0.196516 | 0.186462 | 0.080022 | 0.093901 | 0.22843 | 0 | 0.076973 |
| Iyd | 0 | 0 | 0.048949 | 0 | 0 | 0 | 0 | 0 |
| AABR07065 | 0.149191 | 0 | 0 | 0 | 0.161318 | 0 | 0 | 0.26447 |
| AC095289. | 0 | 0.336645 | 0 | 1.096662 | 0 | 0 | 0.665735 | 0 |
| Tex29 | 0.042231 | 0 | 0 | 0 | 0 | 0.037028 | 0 | 0 |
| LOC100912 | 0.682045 | 0.342979 | 0 | 2.37425 | 1.802743 | 2.192732 | 3.645645 | 3.895864 |
| Btn2a2 | 0.136148 | 0.173301 | 0.347138 | 0.345001 | 0.404841 | 0.40289 | 0.228474 | 0.331856 |
| Stat6 | 8.940523 | 8.208234 | 8.684106 | 8.489413 | 9.501534 | 9.40687 | 13.16488 | 13.07154 |
| Pros1 | 5.879456 | 5.592341 | 5.722392 | 7.605599 | 7.195705 | 7.831805 | 8.728067 | 8.690195 |
| Rpap1 | 3.66431 | 3.604677 | 3.910524 | 3.246266 | 3.221464 | 3.498882 | 3.795352 | 4.03814 |
| Zscan5b | 0 | 0 | 0 | 0 | 0 | 0 | 0.124384 | 0 |
| Ercc3 | 12.83632 | 13.01359 | 13.69633 | 16.36772 | 16.23547 | 15.5056 | 14.207 | 14.57847 |
| Rn50_13_( | 0.429229 | 0.121413 | 0.460805 | 0.197759 | 0.348089 | 0.564522 | 0.960404 | 0.665782 |
| RGD156167 | 1.897316 | 1.144919 | 1.765305 | 1.864855 | 1.367694 | 1.21995 | 0.990562 | 1.121124 |
| AABR07051 | 0 | 0 | 0 | 0 | 0 | 0 | 0.313287 | 0 |
| LOC100910 | 0.390063 | 0.110334 | 0.209379 | 0.089857 | 0.316327 | 0.085502 | 0.218192 | 0.086433 |
| Ube2k | 8.764524 | 8.402927 | 8.606321 | 8.18294 | 7.745586 | 8.368241 | 8.838966 | 8.235286 |
| Il4 | 0 | 0 | 0 | 0.121239 | 0 | 0 | 0 | 0.116619 |
| AC126530. | 0.010202 | 0.034629 | 0.032857 | 0.028202 | 0.011031 | 0 | 0 | 0.018085 |
| 5S_rRNA | 0 | 0 | 0 | 0 | 0 | 0 | 0 | 0 |
| Slc14a2 | 0 | 0.020277 | 0 | 0 | 0.019378 | 0.015713 | 0 | 0.015885 |

| | | | | | | | | |
|---|---|---|---|---|---|---|---|---|
| Aldh9a1 | 24.99486 | 30.15743 | 29.31238 | 21.6065 | 25.74191 | 27.00621 | 23.49907 | 26.20751 |
| Ptpn14 | 4.279366 | 4.473803 | 4.352374 | 3.251466 | 3.923663 | 3.861864 | 4.71757 | 4.502818 |
| Ago2 | 4.06693 | 4.088145 | 3.842897 | 2.988721 | 3.325391 | 3.07963 | 4.437093 | 4.855948 |
| Lag3 | 0.224506 | 0.349274 | 0.271149 | 0.336169 | 0.121377 | 0.270664 | 0.439542 | 0.273612 |
| Trim67 | 1.079905 | 0.836011 | 0.976292 | 0.680851 | 0.8604 | 0.581406 | 0.614673 | 0.722078 |
| AABR0703 | 0.042914 | 0.084972 | 0.057589 | 0.029658 | 0.023201 | 0.122288 | 0.072016 | 0.085583 |
| Sema4f | 0.289751 | 0.253331 | 0.212091 | 0.291266 | 0.341786 | 0.404177 | 0.500976 | 0.385231 |
| LOC10255 | 0.042367 | 0 | 0.045484 | 0.03904 | 0.183244 | 0.037148 | 0.047399 | 0.037552 |
| Reep4 | 2.032028 | 2.361284 | 2.74163 | 2.758045 | 3.117653 | 2.455835 | 4.300919 | 4.332346 |
| Ambn | 0.074278 | 0 | 0 | 0 | 0 | 0 | 0 | 0 |
| LOC10368 | 0.038057 | 0.043059 | 0.040856 | 0 | 0 | 0 | 0 | 0 |
| Hnrnpk | 95.92005 | 91.49672 | 85.83001 | 94.43405 | 102.6252 | 101.1245 | 96.29758 | 94.25279 |
| AABR0700 | 0 | 0 | 0 | 0 | 0 | 0 | 0 | 0 |
| Dnaaf5 | 4.696405 | 5.290955 | 5.085169 | 4.587577 | 4.642261 | 4.206177 | 5.750227 | 5.21672 |
| Klf1 | 0.103763 | 0.234807 | 0.445586 | 0.509941 | 0.112198 | 0.333593 | 0.503039 | 0.367883 |
| Ndufs1 | 350.2421 | 346.5885 | 341.7628 | 325.5234 | 333.3267 | 332.9635 | 314.2355 | 318.1289 |
| Ndufab1 | 111.2929 | 107.3291 | 103.1105 | 100.8758 | 100.979 | 98.37026 | 91.46708 | 89.07892 |
| Ppp1r35 | 0 | 0 | 0 | 0 | 0 | 0 | 0 | 0.039004 |
| Alox5ap | 6.724441 | 6.110674 | 7.503334 | 7.416053 | 7.958068 | 7.1959 | 10.89947 | 11.07566 |
| Rnf111 | 4.63381 | 4.614953 | 4.669835 | 4.626687 | 4.745293 | 4.470353 | 6.137166 | 6.486821 |
| Ifitm10 | 14.80387 | 13.8749 | 15.29988 | 11.19798 | 13.61807 | 12.68945 | 16.31472 | 18.50705 |
| Nr2c1 | 4.240781 | 4.27374 | 4.423724 | 4.145023 | 3.341656 | 3.658105 | 4.609941 | 4.200133 |
| Nop2 | 0.925831 | 7.102795 | 6.787876 | 7.407553 | 7.618035 | 7.008938 | 1.38946 | 1.781325 |
| AABR0705 | 0 | 0.263331 | 0.249858 | 0 | 0 | 0 | 0 | 0 |
| Hsd17b13 | 0.322997 | 0.45682 | 0.346758 | 0.334833 | 0.611191 | 0.354005 | 0.271016 | 0.501005 |
| AABR0703 | 0 | 0 | 0.598066 | 0 | 0 | 0 | 0 | 0.493771 |
| Dpm1 | 15.93547 | 16.14725 | 15.98192 | 16.4485 | 17.20614 | 17.11049 | 16.93523 | 16.22589 |
| Psme4 | 19.30011 | 18.72025 | 17.03419 | 20.92494 | 21.49509 | 20.84648 | 22.61942 | 23.20726 |
| Fam216a | 6.683211 | 7.51388 | 6.130413 | 7.483523 | 8.00399 | 8.122191 | 6.625083 | 7.048408 |
| AABR0700 | 0.229228 | 0.086454 | 0.164061 | 0.39898 | 0.192781 | 0.424307 | 0.199461 | 0.270902 |
| Fmo1 | 11.11085 | 12.43476 | 11.79855 | 13.06485 | 14.28622 | 11.56333 | 16.48369 | 16.5705 |
| Eif4a3 | 30.82607 | 33.49576 | 32.71897 | 32.07225 | 35.2192 | 33.57879 | 31.28421 | 31.09773 |
| RGD15608 | 0 | 0 | 0 | 0 | 0 | 0 | 0 | 0 |
| Golph3l | 4.199154 | 4.029157 | 3.756717 | 4.172834 | 4.629514 | 4.548133 | 4.951141 | 5.19974 |
| Rn50_20_ | 0.220488 | 3.07682 | 0.355062 | 3.183014 | 0.357616 | 0.225545 | 0.246672 | 0.390859 |
| Slc39a9 | 8.312841 | 8.410586 | 7.962458 | 7.996321 | 7.15855 | 7.914284 | 8.18626 | 9.647631 |
| Sprtn | 5.220675 | 5.186589 | 6.185707 | 5.015979 | 4.715674 | 5.135724 | 5.876273 | 5.953473 |
| Zfp341 | 1.631337 | 1.891926 | 2.05783 | 1.879016 | 1.852138 | 1.215804 | 2.555094 | 2.277348 |
| AABR0706 | 0.048532 | 0.164735 | 0.026051 | 0.06708 | 0.026238 | 0.127658 | 0.081443 | 0.107541 |
| Spock3 | 0 | 0.071428 | 0.067774 | 0.116343 | 0.136522 | 0.096866 | 0.088283 | 0.125898 |
| Krt78 | 0 | 0 | 0 | 0 | 0 | 0 | 0 | 0 |
| Smim12 | 31.49428 | 36.54026 | 41.37565 | 30.98825 | 32.95765 | 33.88592 | 36.30935 | 35.76899 |
| Ces2h | 0 | 0 | 0 | 0.024127 | 0 | 0 | 0 | 0 |
| Tex2 | 6.633139 | 6.922166 | 6.785291 | 5.442465 | 5.550837 | 5.549748 | 6.381323 | 6.360387 |
| Psmd8 | 34.61184 | 36.74332 | 35.71463 | 41.83633 | 40.22099 | 43.43589 | 42.38614 | 42.16724 |
| Nlrp6 | 0 | 0 | 0 | 0.018236 | 0.021399 | 0 | 0.022141 | 0 |
| #N/A | 0.370077 | 0.83745 | 0.198651 | 0 | 0 | 0.162242 | 0.207013 | 0.328018 |
| Mrps27 | 28.51079 | 28.48268 | 30.96201 | 34.96549 | 33.45673 | 33.48759 | 32.6802 | 30.89432 |
| MGC11620 | 0.31769 | 0.299543 | 0.397904 | 0.487898 | 0.400766 | 0.464252 | 0.385036 | 0.563169 |
| Hsp90aa1 | 0.702516 | 2.534971 | 3.200236 | 3.569122 | 4.147117 | 3.229663 | 1.508162 | 1.262179 |
| Toml12 | 40.26674 | 42.12202 | 41.84771 | 35.76735 | 34.95619 | 33.52982 | 43.11929 | 42.43544 |
| Glo1 | 78.57394 | 82.21233 | 74.29405 | 76.69709 | 73.52799 | 75.48429 | 72.71226 | 75.32921 |
| Stat5a | 3.928894 | 3.854831 | 3.64204 | 3.419934 | 3.417416 | 4.207557 | 4.006205 | 3.816476 |
| Raver1 | 10.50359 | 11.47154 | 12.66337 | 9.832714 | 9.730197 | 9.422799 | 12.07405 | 11.86972 |

| | | | | | | | | |
|---|---|---|---|---|---|---|---|---|
| AABR07035 | 6.006127 | 4.884374 | 4.634472 | 5.257689 | 5.763725 | 5.595314 | 5.627488 | 6.787499 |
| Zfp563 | 1.909174 | 1.807465 | 1.213041 | 1.938742 | 1.980103 | 1.400665 | 1.874361 | 1.761265 |
| Kcnc2 | 0.095107 | 0.086087 | 0.142945 | 0.192802 | 0.08227 | 0.100068 | 0.063841 | 0.050579 |
| AC120721. | 0 | 0 | 0 | 0 | 0 | 0 | 0.653361 | 0.172545 |
| Rap1b | 61.14075 | 61.17118 | 57.83887 | 66.33719 | 62.02971 | 64.44574 | 82.75744 | 76.4376 |
| AABR07022 | 0.228672 | 0.172488 | 0 | 0.070237 | 0 | 0.133667 | 0.170552 | 0 |
| Actn4 | 48.57243 | 50.6295 | 51.46685 | 54.61795 | 53.75122 | 57.09861 | 68.64645 | 69.06839 |
| Zfp143 | 2.95951 | 3.289455 | 2.560468 | 3.160195 | 3.708323 | 3.190202 | 2.824061 | 2.823756 |
| Grifin | 0.276581 | 0.625876 | 0.593854 | 0.424764 | 0.697813 | 0.889189 | 1.03142 | 0.735441 |
| Fam69b | 6.477771 | 7.543287 | 7.969527 | 7.733572 | 6.799814 | 6.944177 | 7.220593 | 8.130399 |
| Galc | 1.046891 | 0.821904 | 0.665168 | 1.082792 | 0.993377 | 1.123978 | 1.003899 | 1.609643 |
| AABR07051 | 0.182248 | 0.206205 | 0.326091 | 0.111956 | 0.065687 | 0.159795 | 0.339818 | 0.323071 |
| Slc39a13 | 8.017853 | 8.358861 | 8.117809 | 8.629503 | 8.340661 | 9.259104 | 8.629691 | 7.876987 |
| Olr1877 | 0 | 0 | 0 | 0 | 0 | 0 | 0.060459 | 0 |
| Wnt1 | 0.044378 | 0.150635 | 0 | 0.020446 | 0.071978 | 0.019455 | 0 | 0.059002 |
| LOC103693 | 0.134272 | 0.101281 | 0.04805 | 0 | 0 | 0 | 0 | 0 |
| Snx29 | 4.170174 | 3.619807 | 4.152284 | 3.681318 | 3.459309 | 3.335429 | 4.629791 | 4.429835 |
| RGD156334 | 0.018221 | 0.103079 | 0.195609 | 0.050369 | 0.098508 | 0.111831 | 0.163075 | 0.161498 |
| Bcl2l14 | 0.380717 | 0.352443 | 0.148627 | 0.095677 | 0.112272 | 0.303467 | 0.116163 | 0.337449 |
| Kdm1a | 7.37757 | 6.627563 | 6.82578 | 8.284167 | 7.696653 | 7.655123 | 8.357388 | 8.280679 |
| Magi3 | 6.905298 | 6.711313 | 6.74196 | 6.272414 | 6.780792 | 6.407037 | 7.315588 | 7.165667 |
| Irgq | 6.39052 | 7.196305 | 8.031166 | 6.642132 | 4.846808 | 5.948435 | 7.556033 | 7.489676 |
| Hook2 | 1.35203 | 1.643919 | 1.538146 | 2.212764 | 1.549209 | 1.999359 | 1.96411 | 1.949589 |
| Ube4b | 7.314664 | 7.833958 | 7.86275 | 6.723024 | 6.50047 | 6.984681 | 7.860569 | 8.298028 |
| AABR07036 | 2.545566 | 2.880188 | 1.952019 | 0.335091 | 2.359271 | 2.550805 | 1.627352 | 0.322323 |
| AABR07029 | 0 | 0 | 0 | 0 | 0 | 0 | 0 | 0.164299 |
| Lamp1 | 276.7137 | 265.148 | 264.7619 | 282.3109 | 281.8203 | 281.1548 | 306.4148 | 303.0425 |
| Rab34 | 9.186039 | 10.33133 | 8.976009 | 11.96191 | 13.38242 | 12.44321 | 13.60001 | 13.40756 |
| Pate1 | 0 | 0 | 0.297451 | 0 | 0 | 0 | 0 | 0 |
| Cenpw | 0.032708 | 0.296063 | 0.175572 | 0.271254 | 0.318303 | 0.086036 | 0.109778 | 0.202937 |
| Rerg | 4.662225 | 3.997103 | 4.824597 | 6.244784 | 5.379009 | 4.446056 | 4.731942 | 5.538217 |
| Rn50_18_( | 1.876038 | 1.753492 | 3.502689 | 3.53255 | 2.822306 | 3.075269 | 2.920108 | 2.385793 |
| Nxt2 | 0.769773 | 0.725801 | 0.987089 | 0.827534 | 0.554896 | 0.881168 | 1.028641 | 0.909717 |
| Gulp1 | 1.765143 | 2.364474 | 2.003902 | 2.748241 | 3.356546 | 2.988622 | 3.245876 | 3.254951 |
| AABR07068 | 0.160878 | 0.091013 | 0.25907 | 0.074122 | 0.434889 | 0.352646 | 0.539951 | 0.071297 |
| AABR07021 | 0 | 0 | 0 | 0.074465 | 0 | 0 | 0 | 0 |
| AABR07047 | 0 | 0.979332 | 0.464613 | 0 | 0 | 1.517834 | 0 | 0 |
| Hes2 | 3.804016 | 4.029337 | 3.649402 | 3.206932 | 3.150557 | 3.264399 | 2.082608 | 2.58257 |
| Arl6ip1 | 20.39515 | 21.602 | 18.18348 | 24.01113 | 23.19085 | 21.57322 | 23.77024 | 25.13935 |
| LOC103694 | 0 | 0 | 0.121684 | 0 | 0.612798 | 0 | 0 | 0.602786 |
| Dpysl4 | 0.259237 | 0.383565 | 0.363941 | 0.955508 | 0.776242 | 0.821775 | 0.981617 | 1.360974 |
| AC141969. | 0.503518 | 0 | 0 | 0 | 0 | 0 | 0 | 0 |
| Wdr90 | 1.46691 | 1.628424 | 1.713485 | 1.75976 | 1.646003 | 1.771542 | 2.508117 | 2.624923 |
| Rn50_X_07 | 0 | 0.210354 | 0.332652 | 0.228417 | 0 | 0.108673 | 0.346655 | 0.109857 |
| Sycp2 | 0.45545 | 0.368086 | 0.372537 | 0.429672 | 0.328314 | 0.275734 | 0.412483 | 0.326795 |
| AABR07072 | 0 | 0 | 0.010959 | 0 | 0 | 0 | 0 | 0.009048 |
| Tlr3 | 2.614034 | 1.896758 | 1.982735 | 3.102548 | 3.348808 | 3.51272 | 3.035722 | 3.085067 |
| Trim11 | 4.778982 | 5.279665 | 5.541954 | 4.715222 | 4.972446 | 4.625049 | 6.027437 | 5.434675 |
| Stard7 | 51.45744 | 50.1302 | 51.84175 | 49.62689 | 48.76387 | 46.81317 | 47.30034 | 46.68293 |
| Pdzd11 | 9.400797 | 8.981984 | 9.1205 | 10.26662 | 10.69202 | 11.29545 | 11.14044 | 9.258215 |
| Rpl21 | 94.33428 | 68.44716 | 66.87323 | 72.1184 | 78.59695 | 58.51196 | 77.72514 | 86.0428 |
| AABR07068 | 0.320281 | 0.362383 | 0.171921 | 0 | 0.519473 | 0 | 0 | 0.14194 |
| Sft2d2 | 10.67402 | 11.67738 | 10.6008 | 10.38402 | 9.752102 | 10.33728 | 9.77798 | 10.8784 |
| LOC100912 | 2.229619 | 1.772715 | 2.587717 | 1.721343 | 1.49864 | 1.637916 | 1.348324 | 1.335285 |

| | | | | | | | | |
|---|---|---|---|---|---|---|---|---|
| Sin3a | 6.96656 | 7.298458 | 7.149283 | 6.940209 | 7.014708 | 6.873168 | 7.766386 | 7.884587 |
| Fbxl12 | 4.053602 | 3.151241 | 3.463678 | 3.786054 | 3.190421 | 4.086123 | 4.59669 | 3.128519 |
| Emb | 1.076381 | 0.608937 | 1.040007 | 1.586948 | 1.338456 | 1.462846 | 1.324628 | 1.049455 |
| Zpbp | 0.193113 | 0.145666 | 0.069107 | 0.118631 | 0.139207 | 0.169322 | 0.072016 | 0.199694 |
| Mttp | 0.183418 | 0.088941 | 0.084391 | 0.108651 | 0.028333 | 0.091898 | 0.146572 | 0.127736 |
| Cyp1b1 | 5.018316 | 3.785325 | 4.686672 | 8.684436 | 8.073175 | 8.531831 | 12.00449 | 12.83767 |
| Rmdn3 | 5.908439 | 3.827771 | 4.655007 | 4.544313 | 3.529247 | 3.822726 | 3.97139 | 4.497858 |
| Prss36 | 0.632105 | 0.983395 | 0.933082 | 1.037506 | 0.939793 | 0.865984 | 0.751369 | 0.630299 |
| Thoc7 | 32.11776 | 29.72873 | 25.91953 | 26.37838 | 28.33112 | 27.16204 | 27.05173 | 25.95956 |
| Gata1 | 0 | 0 | 0 | 0 | 0 | 0 | 0.03143 | 0 |
| Mov10 | 3.474183 | 3.071493 | 3.352251 | 3.62903 | 3.209065 | 3.169493 | 4.610612 | 4.887051 |
| RGD156488 | 0.069961 | 0.079158 | 0.037554 | 0.032233 | 0.037824 | 0.153355 | 0.078269 | 0.06201 |
| Themis2 | 0.766077 | 1.145913 | 1.170921 | 0.957166 | 1.095104 | 0.933545 | 1.350948 | 1.231429 |
| Ubxn4 | 15.52866 | 14.89704 | 15.3353 | 15.33942 | 15.80322 | 15.01024 | 17.35516 | 16.47192 |
| Gtf3c1 | 10.9743 | 11.22778 | 12.125 | 9.115885 | 8.671249 | 9.495404 | 10.68429 | 10.89486 |
| Erc2 | 0 | 0 | 0 | 0.023243 | 0 | 0 | 0 | 0 |
| Mt-atp6 | 64229.46 | 63696.25 | 61273.17 | 62940.65 | 63203.69 | 64373.46 | 49219.81 | 51180.97 |
| MGC94199 | 0.755797 | 1.007854 | 0.144892 | 0.149237 | 0.116747 | 0.189338 | 0.120793 | 0.119625 |
| Muc13 | 0.018393 | 0 | 0.078986 | 0.033898 | 0.019888 | 0 | 0.020578 | 0.048909 |
| Cfap52 | 1.193859 | 1.165392 | 1.382208 | 0.582403 | 0.404989 | 0.554176 | 0.628535 | 0.767697 |
| AABR07026 | 1.222551 | 0.922172 | 2.296851 | 1.126532 | 1.321926 | 0.714622 | 1.253757 | 1.264209 |
| Grpel2 | 2.981072 | 2.784016 | 2.997173 | 3.124827 | 2.779961 | 3.070185 | 3.035109 | 2.684213 |
| Lca5l | 0 | 0 | 0 | 0.05326 | 0.031249 | 0 | 0.032331 | 0 |
| Ndufs8 | 259.0293 | 267.0984 | 275.7546 | 253.1999 | 254.6499 | 244.1202 | 218.9876 | 216.8079 |
| Arsa | 6.580163 | 6.854781 | 7.126465 | 8.614246 | 7.867285 | 8.590698 | 8.869585 | 9.352275 |
| Aftph | 6.634897 | 6.756366 | 6.161382 | 6.893305 | 6.062214 | 6.064726 | 6.829001 | 6.748262 |
| AABR07021 | 0.614623 | 0.417251 | 0.263935 | 0.679622 | 0.39875 | 0.538902 | 0.275045 | 0.326863 |
| Pdcd11 | 3.857242 | 4.023874 | 3.983638 | 4.286482 | 4.095706 | 4.795723 | 5.834301 | 5.565913 |
| Slc35f1 | 0.564177 | 0.841448 | 0.674508 | 0.590758 | 0.942784 | 0.809462 | 0.788971 | 0.852372 |
| A4gnt | 0.05089 | 0 | 0 | 0 | 0 | 0 | 0 | 0 |
| Fmnl1 | 7.106199 | 8.359391 | 7.901422 | 8.665816 | 8.247921 | 8.344717 | 10.75787 | 10.89754 |
| Fkbp7 | 5.76171 | 5.817681 | 5.715774 | 7.056517 | 7.807282 | 7.801626 | 8.241018 | 8.20981 |
| Hlf | 3.302355 | 2.335287 | 2.405732 | 2.771295 | 2.167979 | 2.53357 | 2.441028 | 2.822504 |
| Zbtb47 | 19.68212 | 21.75465 | 23.21216 | 23.76252 | 20.03924 | 22.98873 | 25.50188 | 26.84699 |
| Pcdhb22 | 0.767507 | 0.918021 | 0.565007 | 0.969912 | 0.758761 | 1.134403 | 0.883186 | 1.049575 |
| Cd3d | 1.525221 | 0.657416 | 1.013642 | 0.535402 | 0.314133 | 0.700498 | 0.568783 | 0.386251 |
| #N/A | 0 | 0 | 0 | 0 | 0 | 0 | 0 | 0 |
| Chst13 | 0 | 0.057917 | 0 | 0.094337 | 0.166048 | 0.089764 | 0.171803 | 0.226855 |
| B3gnt4 | 0 | 0 | 0.159033 | 0 | 0.120133 | 0.032471 | 0.041432 | 0.032825 |
| AABR07042 | 0.07847 | 0.05919 | 0 | 0.024102 | 0 | 0.045869 | 0 | 0 |
| AABR07045 | 0 | 0 | 0 | 0 | 0.062497 | 0 | 0 | 0.204921 |
| P4ha2 | 10.09736 | 11.06607 | 10.79155 | 10.59775 | 10.23269 | 10.81859 | 14.99443 | 16.97652 |
| Hs3st2 | 0 | 0 | 0.049336 | 0.444632 | 0.273299 | 0.62455 | 0.282769 | 0.468422 |
| Ube2t | 1.021774 | 0.481704 | 0.457058 | 0.784604 | 0.460346 | 0.522604 | 0.666817 | 0.528295 |
| Spag6l | 0.054181 | 0.091955 | 0.145417 | 0.149777 | 0.087878 | 0.213777 | 0.181846 | 0.192093 |
| Stam | 6.765623 | 7.374301 | 6.41594 | 8.208436 | 7.583808 | 7.909472 | 8.376447 | 7.615819 |
| Pkd1l2 | 0.106739 | 0.06441 | 0.084033 | 0.098355 | 0.084638 | 0.149741 | 0.103492 | 0.145065 |
| Rps6ka3 | 6.979993 | 7.255972 | 6.481765 | 6.152146 | 6.450666 | 6.232102 | 7.764233 | 8.742097 |
| Pabpn1 | 29.49446 | 30.10771 | 28.97811 | 32.7926 | 32.5285 | 31.87431 | 35.20349 | 34.56955 |
| Dbi | 101.3263 | 92.6275 | 91.82933 | 73.60233 | 80.82098 | 74.45592 | 76.00173 | 80.24537 |
| Il2rb | 5.202575 | 5.546865 | 5.606779 | 3.761421 | 3.743101 | 3.491396 | 4.477239 | 4.487151 |
| Nif3l1 | 5.164764 | 5.727588 | 4.920466 | 5.925271 | 4.733953 | 5.038298 | 5.625046 | 6.972788 |
| AABR07052 | 3.39467 | 4.445496 | 5.129148 | 4.489337 | 4.622246 | 4.657592 | 4.430773 | 3.649641 |
| Prr13 | 27.73356 | 27.26884 | 31.10548 | 35.10803 | 31.71244 | 31.23117 | 34.79394 | 34.94833 |

| | | | | | | | | |
|---|---|---|---|---|---|---|---|---|
| Zfp142 | 5.37715 | 5.316558 | 5.307738 | 5.572303 | 5.549148 | 6.09757 | 7.588214 | 7.619862 |
| Dscam | 0.028306 | 0.053378 | 0.030388 | 0.034777 | 0.051011 | 0.016546 | 0.021112 | 0.008363 |
| AABR0700: | 0.295853 | 0 | 0.105872 | 0.272617 | 0 | 0.086468 | 0.220658 | 0.349639 |
| Unc93a | 0 | 0 | 0 | 0 | 0 | 0.03349 | 0 | 0 |
| Bles03 | 17.68443 | 19.90305 | 19.22017 | 19.26095 | 19.66247 | 19.99855 | 24.08403 | 21.57334 |
| Kdelr3 | 11.87681 | 11.94493 | 10.04586 | 16.08438 | 15.63119 | 16.61968 | 15.97154 | 17.17284 |
| Large | 4.761786 | 4.036921 | 4.183951 | 4.223414 | 3.813413 | 4.283427 | 4.621065 | 4.549012 |
| AC121209. | 0.325254 | 0.12267 | 0 | 0.399612 | 0.117231 | 0.095061 | 0.242587 | 0.384385 |
| Msrb2 | 5.565019 | 6.141724 | 6.464109 | 5.338108 | 5.425502 | 4.959404 | 6.276928 | 5.619876 |
| Faah | 0.060191 | 0 | 0.032309 | 0 | 0.032542 | 0.052775 | 0.033669 | 0 |
| Cage1 | 0.402112 | 0.43429 | 0.549427 | 0.404215 | 0.454561 | 0.64104 | 0.327174 | 0.502216 |
| AABR0706: | 0 | 0.168802 | 0 | 0.41242 | 0 | 0.130811 | 0 | 0.132235 |
| Mturn | 5.774189 | 5.782278 | 5.486437 | 3.761176 | 3.624128 | 4.539075 | 4.789241 | 4.500267 |
| Cog1 | 9.619658 | 9.617667 | 9.970556 | 9.331518 | 9.872061 | 9.922068 | 11.03599 | 11.1308 |
| Zfp664 | 11.19272 | 15.07623 | 15.66384 | 12.76928 | 6.051261 | 7.82766 | 13.78901 | 9.093939 |
| Eif4ebp3 | 8.130376 | 8.140923 | 8.251295 | 8.234178 | 7.349412 | 7.891933 | 9.033745 | 9.603004 |
| Casq2 | 352.8954 | 350.0856 | 330.7735 | 370.017 | 367.0276 | 366.3537 | 346.597 | 353.4588 |
| AABR0704: | 0.036365 | 0 | 0.03904 | 0.033509 | 0.039321 | 0 | 0.122051 | 0.064465 |
| Rasgef1a | 0 | 0.035952 | 0 | 0.02928 | 0 | 0.083582 | 0.035549 | 0.056328 |
| AABR0701: | 0 | 0 | 0 | 0.360098 | 0 | 0 | 0 | 0 |
| Chd9 | 6.454703 | 6.624578 | 6.484942 | 6.592534 | 6.933054 | 6.811421 | 9.681567 | 8.391826 |
| LOC29087 | 0 | 0.068975 | 0 | 0.112347 | 0.065917 | 0 | 0.204603 | 0.054033 |
| Snx5 | 24.23925 | 22.99017 | 21.35799 | 21.04114 | 20.91112 | 19.76357 | 26.60614 | 25.39335 |
| Lrg1 | 34.41189 | 37.286 | 44.64505 | 37.9662 | 33.7661 | 37.33705 | 45.92351 | 41.96008 |
| AABR0700: | 0.818218 | 0 | 0 | 0.753955 | 0 | 0 | 0 | 0 |
| AC126581. | 1.098586 | 1.242998 | 1.96567 | 1.349738 | 2.507757 | 1.284321 | 2.321537 | 1.190116 |
| Fbxl18 | 1.093051 | 1.108797 | 1.524149 | 0.358888 | 0.665668 | 0.627909 | 0.674679 | 0.712699 |
| Cbr1 | 0 | 0.062831 | 0.119233 | 0.153509 | 0 | 0.438208 | 0 | 0.09844 |
| Radil | 1.136228 | 1.221309 | 1.143575 | 1.138601 | 1.013584 | 1.469463 | 1.398278 | 1.699474 |
| Abhd2 | 3.398121 | 3.910538 | 3.679281 | 5.914555 | 5.966876 | 6.213608 | 8.090728 | 8.701113 |
| Scarb1 | 6.082018 | 7.195611 | 7.613158 | 7.487956 | 6.057927 | 7.567592 | 8.865342 | 9.752641 |
| Sts | 19.40906 | 19.8493 | 22.7529 | 26.89243 | 25.13145 | 25.00223 | 30.58564 | 29.73262 |
| Epb4.1l5 | 1.664649 | 1.053468 | 1.423628 | 1.065937 | 1.586407 | 1.212182 | 1.451991 | 1.525477 |
| Crem | 11.55085 | 10.8268 | 10.87128 | 10.2727 | 9.509635 | 9.964882 | 15.07057 | 14.21805 |
| Rassf1 | 15.80976 | 16.22646 | 15.36272 | 15.17267 | 15.20294 | 15.25917 | 21.00799 | 19.38554 |
| RGD156403 | 1.116809 | 1.029613 | 1.198966 | 1.981961 | 1.162864 | 1.196823 | 1.573364 | 1.429831 |
| Slc29a3 | 2.093577 | 2.488722 | 2.247588 | 2.759415 | 2.550305 | 2.939364 | 3.528125 | 3.124054 |
| Shisa8 | 0.055209 | 0.062467 | 0.059271 | 0.050873 | 0 | 0.048407 | 0 | 0.048935 |
| Ddx47 | 15.12855 | 14.6 | 14.53969 | 15.88791 | 16.71913 | 14.9472 | 15.33842 | 15.82481 |
| Steap4 | 18.71592 | 20.33288 | 19.50003 | 21.06146 | 19.58058 | 19.62923 | 18.83851 | 19.18238 |
| Tlr11 | 0.276552 | 0.110437 | 0.227038 | 0.02998 | 0.175901 | 0.128372 | 0.145597 | 0.173027 |
| Tdgf1 | 0.209046 | 0 | 0.112212 | 0.288941 | 0.226038 | 0.274937 | 0.818548 | 0.092644 |
| Pex13 | 7.129632 | 6.189216 | 5.498646 | 6.437527 | 6.911668 | 7.41889 | 5.959317 | 6.973073 |
| Smad7 | 9.997685 | 10.33246 | 11.15201 | 11.89757 | 11.03447 | 11.2568 | 14.28129 | 14.89155 |
| Slc35g2 | 0.167196 | 0.15134 | 0.143597 | 0.154065 | 0.289259 | 0.175918 | 0.112231 | 0.088917 |
| Nsa2 | 47.68858 | 41.7048 | 40.60807 | 43.98999 | 45.7378 | 46.09279 | 43.96694 | 41.27738 |
| Ceacam18 | 0.230281 | 0.260552 | 0.247221 | 0.127317 | 0.0498 | 0.040382 | 0.154577 | 0.326575 |
| Katnb1 | 5.578162 | 6.033392 | 6.252325 | 6.023151 | 5.898732 | 6.830076 | 7.58769 | 6.708433 |
| AC140765. | 0 | 0 | 0 | 0 | 0 | 0 | 0 | 0 |
| Qrfpr | 0 | 0.091013 | 0 | 0.037061 | 0.043489 | 0 | 0.089992 | 0.071297 |
| Atp6v1c1 | 25.25214 | 25.1403 | 25.33685 | 27.14239 | 26.16843 | 26.93264 | 27.7471 | 26.64044 |
| Abca13 | 0 | 0 | 0 | 0 | 0 | 0 | 0 | 0.003559 |
| Elovl6 | 0 | 0 | 0 | 0 | 0 | 0 | 0 | 0 |
| AABR0703: | 18.81106 | 17.8324 | 15.28261 | 18.73908 | 19.79039 | 13.81892 | 22.75133 | 27.48821 |

| | | | | | | | | |
|---|---|---|---|---|---|---|---|---|
| Taf1a | 1.618373 | 1.787515 | 1.489223 | 2.272407 | 1.833253 | 1.891988 | 2.155433 | 1.912592 |
| C1s | 53.95277 | 54.03083 | 53.01543 | 93.56234 | 92.2661 | 92.94205 | 123.9391 | 117.0771 |
| Asb18 | 21.34574 | 20.52895 | 26.57519 | 18.93959 | 17.94349 | 16.93493 | 25.3058 | 25.5112 |
| Amigo2 | 2.24957 | 2.25822 | 2.360582 | 2.852172 | 3.017673 | 2.446993 | 2.781636 | 2.398683 |
| AABR07002 | 0.867943 | 0 | 0.310598 | 0.266592 | 0 | 0 | 0 | 0.512868 |
| Abcb6 | 20.30147 | 20.02227 | 20.68718 | 23.50855 | 22.56533 | 24.38218 | 27.70506 | 27.32283 |
| LOC100911 | 0 | 0 | 0 | 0 | 0.165563 | 0 | 0 | 0 |
| Ascl2 | 0.133247 | 0.037691 | 0.286098 | 0.184172 | 0.108058 | 0.116831 | 0.223606 | 0.118103 |
| Pip5k1b | 2.016498 | 1.853777 | 2.074637 | 2.283943 | 2.293973 | 2.283753 | 2.279468 | 2.103827 |
| Gnpnat1 | 5.427014 | 5.709505 | 5.2811 | 4.386649 | 5.422035 | 5.871489 | 5.57442 | 6.301122 |
| Laptm4b | 22.87639 | 21.38765 | 21.7255 | 19.03972 | 18.199 | 19.63498 | 19.08368 | 20.10038 |
| Bysl | 10.82904 | 11.41951 | 10.93405 | 11.61807 | 10.51514 | 10.51703 | 11.36 | 12.53491 |
| AABR07024 | 0 | 0 | 0 | 0.362806 | 0 | 0 | 0 | 0 |
| Tpx2 | 0.748831 | 0.827563 | 0.878701 | 0.657924 | 1.054493 | 0.94669 | 0.876724 | 0.694597 |
| Nr2e3 | 0 | 0 | 0 | 0 | 0 | 0 | 0 | 0.044078 |
| Fbxo33 | 2.355123 | 2.396451 | 2.61322 | 2.70905 | 2.649106 | 2.744059 | 3.660436 | 3.586513 |
| LOC100909 | 19.92814 | 42.79136 | 24.51736 | 28.95188 | 32.40066 | 49.74069 | 24.08481 | 21.53118 |
| AC141521. | 0.158685 | 0.538633 | 0.170358 | 0 | 0 | 0 | 0.177529 | 0 |
| Casz1 | 9.309711 | 10.35885 | 11.42385 | 8.765216 | 8.157481 | 8.416528 | 9.810881 | 10.38084 |
| Agap1 | 10.01155 | 10.25412 | 10.93046 | 9.094763 | 8.298919 | 9.088535 | 9.933095 | 12.01155 |
| Pcdhb17 | 0.305468 | 0.394997 | 0.468485 | 0.341793 | 0.401076 | 0.5548 | 0.537026 | 0.32877 |
| Ilk | 47.02369 | 46.34102 | 47.43711 | 58.04282 | 57.60158 | 56.84399 | 62.09637 | 58.58662 |
| Zcchc2 | 5.32239 | 5.248713 | 6.385234 | 5.721769 | 6.100954 | 6.030978 | 8.118231 | 7.591822 |
| AC142180. | 0.389338 | 0.367098 | 0.139326 | 0.418553 | 0.280657 | 0.398267 | 0.508169 | 0.632664 |
| Adgb | 0.029787 | 0.033703 | 0 | 0.041172 | 0.064417 | 0.039176 | 0.033325 | 0.052804 |
| Rcor1 | 4.470842 | 4.520404 | 3.880636 | 3.418478 | 3.445693 | 3.419607 | 5.16141 | 4.72155 |
| Samd15 | 0.056096 | 0.095206 | 0.21078 | 0.103381 | 0.15164 | 0.024593 | 0.188274 | 0.397768 |
| Adam15 | 18.56445 | 20.22303 | 20.86709 | 20.37355 | 20.46668 | 21.34705 | 29.17028 | 29.52479 |
| Ajuba | 3.568929 | 3.542493 | 4.023047 | 3.88656 | 4.051983 | 3.769312 | 4.410184 | 4.184208 |
| AABR07010 | 0 | 0 | 0 | 0 | 0 | 0 | 0 | 0.107441 |
| Ptgdr2 | 0.032729 | 0 | 0 | 0 | 0.212334 | 0.057393 | 0.073231 | 0.058018 |
| Agtpbp1 | 27.37528 | 24.62373 | 23.55784 | 21.87372 | 22.99801 | 22.94668 | 20.10311 | 22.66284 |
| AABR07062 | 0.137987 | 0.234188 | 0.148137 | 0.254298 | 0 | 0.060493 | 0.077187 | 0.244609 |
| Capn10 | 3.529509 | 3.236541 | 3.987279 | 3.656185 | 4.340228 | 3.458756 | 4.464823 | 3.966703 |
| Cenpl | 1.733737 | 1.281072 | 0.911646 | 1.760587 | 1.339046 | 1.613212 | 1.068774 | 1.913031 |
| #N/A | 86.82896 | 90.36545 | 89.74618 | 77.4433 | 79.69094 | 80.62287 | 90.40844 | 90.97774 |
| Commd3 | 51.62516 | 53.06028 | 47.53157 | 51.24598 | 55.94477 | 53.45865 | 54.12229 | 50.15192 |
| Tmem171 | 1.650765 | 2.645997 | 2.510618 | 4.183065 | 4.065715 | 3.256632 | 5.130006 | 3.779816 |
| Dnajc4 | 22.93905 | 23.07063 | 24.8131 | 23.91306 | 20.79499 | 24.12542 | 19.3122 | 20.64008 |
| AABR07063 | 1.301639 | 1.019591 | 0.64495 | 1.476195 | 1.407443 | 1.755812 | 1.568232 | 1.59744 |
| Trat1 | 0.071514 | 0.101143 | 0.038387 | 0.082371 | 0.077327 | 0.094055 | 0.060005 | 0.04754 |
| Bpifb4 | 0.174553 | 0.169285 | 0.107082 | 0.137866 | 0.026963 | 0.087456 | 0.083692 | 0.132613 |
| Usf3 | 1.6753 | 1.912078 | 1.649317 | 1.590911 | 1.732374 | 1.712653 | 1.947911 | 2.04255 |
| Rbck1 | 35.3837 | 34.02698 | 36.09521 | 31.34199 | 31.03655 | 29.5655 | 32.30369 | 33.29266 |
| AABR07045 | 4.12703 | 4.210241 | 2.977965 | 1.745591 | 2.267827 | 2.788089 | 3.557467 | 2.45865 |
| Mzb1 | 0.454368 | 0.514096 | 0.914612 | 1.046706 | 0.552714 | 0.497988 | 1.016654 | 0.956481 |
| Irf6 | 0.751044 | 0.791166 | 0.806294 | 0.548873 | 0.574066 | 0.703931 | 0.869209 | 0.757507 |
| Aqp9 | 0.091602 | 0.034548 | 0.098341 | 0.028136 | 0.198096 | 0.053545 | 0.136641 | 0.081192 |
| AC133055. | 0.707648 | 0.400335 | 0.759705 | 0.326035 | 1.147754 | 2.171631 | 1.979212 | 0.627223 |
| AABR07058 | 0 | 0 | 0 | 0 | 0 | 0 | 0 | 0 |
| Ruvbl1 | 10.04716 | 9.961963 | 12.95876 | 10.9919 | 10.24963 | 12.48251 | 11.20059 | 12.74433 |
| AABR07032 | 0 | 0.29924 | 0 | 0 | 0 | 0 | 0 | 0 |
| Rnf180 | 0.440791 | 0.565232 | 0.694051 | 0.297859 | 0.254198 | 0.438017 | 0.756143 | 0.260463 |
| Tnmd | 0.039255 | 0.044415 | 0 | 0.397889 | 0.29712 | 0.172093 | 0.219583 | 0.278348 |

| | | | | | | | | |
|---|---|---|---|---|---|---|---|---|
| Mcts1 | 18.87784 | 16.64442 | 15.84673 | 17.21013 | 20.68377 | 17.47656 | 17.91803 | 17.88939 |
| AABR0700[illegible] | 0 | 0 | 1.292372 | 0.554634 | 0.650833 | 0 | 1.346774 | 0.5335 |
| Cdk19 | 6.781459 | 7.231813 | 7.182999 | 6.733384 | 6.00928 | 6.319601 | 6.917372 | 7.730396 |
| AABR0705[illegible] | 0 | 0 | 0 | 0.442689 | 0 | 0 | 0 | 0.425821 |
| Rn60_17_[illegible] | 0.302111 | 0.37031 | 0.648671 | 0.603164 | 0.680559 | 0.706377 | 0.760474 | 0.535552 |
| 4933416C[illegible] | 0.045814 | 0 | 0 | 0 | 0 | 0 | 0 | 0 |
| Neu2 | 0.352957 | 0.471964 | 0.310027 | 0.680038 | 0.797989 | 0.590811 | 0.574359 | 0.625686 |
| AC141169. | 0.530258 | 0.399975 | 0.455413 | 0.336599 | 0.394982 | 0.330617 | 0.540498 | 0.45955 |
| Uhrf1bp1 | 2.601067 | 2.942985 | 3.222013 | 2.04853 | 1.790863 | 2.30011 | 3.108929 | 3.527149 |
| AABR0701[illegible] | 0 | 0 | 0 | 0 | 0 | 0 | 0 | 0 |
| Ints5 | 3.406824 | 3.402236 | 3.159481 | 3.212945 | 2.455833 | 2.79077 | 4.079812 | 3.317348 |
| AY172581. | 492.0856 | 605.5655 | 486.9485 | 481.1121 | 488.7855 | 504.3841 | 1156.186 | 1225.889 |
| Rpsa | 450.5426 | 466.5977 | 504.9132 | 506.9189 | 482.5997 | 477.7003 | 485.8437 | 487.3296 |
| RGD15655[illegible] | 0 | 0.085994 | 0.081594 | 0 | 0 | 0.066639 | 0.085029 | 0.067365 |
| Tyw5 | 4.796065 | 5.064753 | 5.301441 | 4.615802 | 5.032259 | 3.551052 | 5.842568 | 5.227144 |
| Gas6 | 339.8969 | 351.2866 | 366.9222 | 356.5326 | 350.1506 | 361.6342 | 417.9791 | 418.7009 |
| Gstm7 | 188.7345 | 185.1759 | 194.9355 | 224.2566 | 217.7378 | 211.9596 | 197.2332 | 189.5077 |
| Trappc6a | 21.38049 | 22.28766 | 25.22535 | 16.35106 | 17.60285 | 17.03356 | 19.48775 | 21.35549 |
| Epn1 | 68.26143 | 72.75881 | 81.85426 | 59.61074 | 56.43994 | 62.34726 | 72.58994 | 67.47315 |
| Pex12 | 0 | 0 | 0 | 0 | 0 | 0 | 0 | 0 |
| AABR0705[illegible] | 0 | 0.731476 | 0 | 0.238287 | 0 | 0 | 0 | 0 |
| #N/A | 129.529 | 144.7244 | 160.3073 | 181.5538 | 162.6273 | 176.8622 | 223.637 | 226.355 |
| Tuba8 | 61.34199 | 64.90845 | 73.1085 | 44.16337 | 40.36279 | 43.6783 | 46.98631 | 44.6237 |
| AABR0700[illegible] | 1.246808 | 0.940469 | 0 | 0.765923 | 3.59508 | 0.364401 | 1.859831 | 1.105108 |
| Ksr2 | 0.054853 | 0.041375 | 0.039258 | 0.016848 | 0.039541 | 0 | 0.040911 | 0.016206 |
| Tmem8c | 0 | 0.041784 | 0.039646 | 0 | 0 | 0.097139 | 0.08263 | 0 |
| Rnf144b | 7.622557 | 7.782783 | 8.687752 | 7.168213 | 7.16955 | 6.614799 | 10.16324 | 10.30791 |
| Phykpl | 0.065952 | 0 | 0 | 0.030386 | 0 | 0.057827 | 0 | 0 |
| Gdap1 | 0.066674 | 0.030175 | 0.143158 | 0.0983 | 0.043256 | 0.05846 | 0.089511 | 0.094555 |
| Clic6 | 0.053526 | 0.020187 | 0.019154 | 0 | 0 | 0.015644 | 0 | 0.063257 |
| Birc3 | 1.986987 | 1.764397 | 1.809134 | 2.804337 | 2.230089 | 2.22736 | 1.885289 | 2.162444 |
| Gse1 | 4.541762 | 4.391329 | 5.851045 | 4.902492 | 4.158355 | 4.064968 | 5.661821 | 5.844946 |
| AC141997. | 0.140985 | 0.205095 | 0.172979 | 0.185589 | 0.30489 | 0.30021 | 0.247858 | 0.124962 |
| Fiz1 | 5.201015 | 4.389567 | 4.637862 | 4.105653 | 4.433079 | 4.426566 | 4.70042 | 5.285639 |
| Akt2 | 49.64733 | 53.36643 | 54.94305 | 49.2503 | 47.83232 | 47.9973 | 49.81468 | 49.10488 |
| Prss38 | 0 | 0.12267 | 0 | 0.049951 | 0 | 0.047531 | 0.121293 | 0.096096 |
| Serpinc1 | 1.918426 | 1.808841 | 2.097693 | 2.029643 | 2.266437 | 2.21162 | 2.027014 | 2.046773 |
| Efcab1 | 0.611956 | 0.445114 | 0.656974 | 0.604171 | 0.945284 | 0.804845 | 0.880237 | 0.69738 |
| Sh3bgrl2 | 3.237166 | 3.447248 | 3.270875 | 6.316774 | 4.118001 | 3.673159 | 3.834633 | 4.725842 |
| Capn9 | 0.193629 | 0.146055 | 0.115485 | 0.138772 | 0.209368 | 0.188638 | 0.096277 | 0.228831 |
| RGD156010[illegible] | 10.05397 | 10.75925 | 11.60723 | 9.58079 | 9.654766 | 9.791352 | 13.97712 | 13.18332 |
| Fbxo45 | 3.269015 | 3.628949 | 2.251375 | 2.443916 | 2.734421 | 2.812195 | 2.691168 | 3.553526 |
| Tmem30b | 0.017024 | 0.019262 | 0.018276 | 0.015687 | 0 | 0 | 0 | 0 |
| Il15 | 12.85053 | 11.99531 | 11.03669 | 12.92671 | 15.97936 | 10.04673 | 14.85583 | 15.85117 |
| RGD130794[illegible] | 2.957454 | 3.622513 | 3.116758 | 3.000195 | 3.37388 | 3.235425 | 2.853346 | 2.861828 |
| Il4r | 14.7386 | 15.83466 | 15.66317 | 15.07482 | 15.1969 | 14.65717 | 17.38738 | 18.04643 |
| Cpb1 | 0 | 0 | 0.045047 | 0 | 0 | 0 | 0.046943 | 0 |
| AABR0705[illegible] | 0 | 0.586629 | 0 | 0.477754 | 0 | 0 | 0.580046 | 0 |
| AABR0702[illegible] | 0.494018 | 0 | 0 | 0 | 0 | 0 | 0 | 0 |
| Rgp1 | 14.08431 | 13.48006 | 17.10341 | 13.95682 | 14.43025 | 13.72576 | 16.27352 | 17.35428 |
| Zfp566 | 0.650701 | 0.350589 | 0.565508 | 0.371178 | 0.569576 | 0.298852 | 0.797306 | 0.604213 |
| Itga1 | 11.66338 | 11.86128 | 11.17354 | 9.266545 | 10.16789 | 10.36956 | 11.91076 | 12.5968 |
| Fam117a | 3.987941 | 4.249323 | 3.865648 | 4.495301 | 4.45863 | 4.956388 | 4.439873 | 4.684349 |
| Hs3st3b1 | 0.138205 | 0.203284 | 0.400604 | 0.140086 | 0.089664 | 0.096943 | 0.324697 | 0.122498 |

| | | | | | | | | |
|---|---|---|---|---|---|---|---|---|
| Abcf2 | 23.8571 | 26.13137 | 24.7652 | 25.86869 | 26.85525 | 28.66972 | 27.5424 | 28.16216 |
| Maf1 | 85.15911 | 87.37853 | 88.58514 | 76.71773 | 73.49769 | 71.95343 | 79.97673 | 79.85355 |
| Prpf19 | 50.23561 | 53.22372 | 52.70785 | 54.75841 | 52.22985 | 56.03565 | 51.87819 | 51.99129 |
| Slc35c2 | 12.59201 | 12.1652 | 12.69989 | 17.70735 | 16.65702 | 18.96969 | 11.4993 | 12.67545 |
| Arhgdia | 90.05267 | 98.99091 | 100.5049 | 99.04726 | 97.46301 | 103.6739 | 117.481 | 117.8879 |
| Hcfc1r1 | 272.8728 | 287.3806 | 304.6107 | 330.3863 | 320.2831 | 299.6935 | 285.3512 | 287.6437 |
| AABR07029 | 0.079705 | 0 | 0 | 0 | 0 | 0 | 0 | 0 |
| Sox10 | 1.486784 | 2.03432 | 1.893117 | 2.803748 | 2.430151 | 2.485961 | 2.320951 | 1.731542 |
| Nfya | 7.222886 | 6.916963 | 7.4506 | 6.996404 | 7.033706 | 6.523786 | 8.810822 | 8.889528 |
| Shisa5 | 30.77966 | 28.59376 | 29.91345 | 33.17636 | 33.54435 | 33.05985 | 42.8171 | 40.67104 |
| AC109660. | 1.525221 | 0 | 0 | 0 | 0 | 0 | 0 | 0 |
| AABR07050 | 20.9207 | 23.52557 | 29.34919 | 17.14879 | 16.5149 | 15.98004 | 19.38463 | 18.65682 |
| Lrrc10 | 35.84706 | 22.50467 | 48.05487 | 25.92139 | 41.9201 | 37.96648 | 30.71714 | 51.62702 |
| Hspa5 | 88.97301 | 85.5673 | 87.64065 | 103.8893 | 104.2721 | 103.2269 | 135.2341 | 140.24 |
| AABR07047 | 1.021063 | 0.990244 | 1.565966 | 1.881738 | 2.050397 | 0.767373 | 2.611016 | 1.680749 |
| Fdx1 | 32.90037 | 34.99576 | 31.79499 | 32.13208 | 31.82999 | 34.71072 | 28.92493 | 30.48434 |
| Fahd2a | 17.45531 | 16.80169 | 17.43578 | 16.76039 | 17.23294 | 16.56914 | 15.65088 | 18.09494 |
| Gtf2f2 | 6.907383 | 5.933141 | 5.396633 | 6.731446 | 6.686775 | 6.119817 | 5.502427 | 6.763441 |
| LOC100365 | 31.83027 | 38.6284 | 30.86487 | 31.45915 | 30.80931 | 30.27205 | 35.17952 | 37.19987 |
| P2rx5 | 5.803038 | 5.80148 | 5.420971 | 5.147746 | 4.916775 | 5.103297 | 6.201486 | 5.880503 |
| Tmed8 | 3.78999 | 3.623827 | 2.693429 | 3.393951 | 2.655082 | 3.089048 | 3.045686 | 3.311943 |
| Hsf2bp | 0.196587 | 0.222429 | 0.211049 | 0.226434 | 0.265709 | 0.21546 | 0.247425 | 0.196026 |
| Fam115c | 0.247593 | 0.46023 | 0.436683 | 0.374813 | 0.248596 | 0.418673 | 0.455065 | 0.517284 |
| LOC100362 | 0 | 0.098258 | 0 | 0 | 0 | 0 | 0 | 0 |
| Hspa1a | 0.390014 | 0.117675 | 0.334964 | 0.095835 | 0.056229 | 0.706727 | 0.756307 | 0.391781 |
| Ggt6 | 0 | 0 | 0 | 0 | 0 | 0 | 0 | 0 |
| Spsb4 | 9.533089 | 10.46586 | 10.19385 | 10.85435 | 10.89994 | 11.50345 | 13.36845 | 12.16415 |
| Pou2f3 | 0 | 0.042422 | 0.013417 | 0.09213 | 0.027027 | 0.010958 | 0.027964 | 0 |
| LOC689713 | 0 | 0 | 0 | 0.044928 | 0 | 0 | 0 | 0 |
| Srek1 | 11.81654 | 11.73733 | 12.12496 | 13.27248 | 14.2296 | 13.19632 | 14.91752 | 15.8978 |
| Azin2 | 1.713545 | 2.093899 | 2.244311 | 2.210549 | 2.334567 | 2.043315 | 1.917037 | 2.460455 |
| Lrit1 | 0 | 0 | 0 | 0 | 0.013953 | 0.011315 | 0 | 0 |
| Fam101b | 7.354332 | 7.624026 | 7.729997 | 5.109155 | 4.538128 | 5.131619 | 7.323083 | 7.201076 |
| Gdpd1 | 9.525563 | 9.214119 | 9.113595 | 7.367585 | 7.204561 | 7.529803 | 8.558552 | 9.142916 |
| Lman1l | 0 | 0.074669 | 0.070848 | 0.121621 | 0 | 0.028932 | 0.036915 | 0.029247 |
| AABR07012 | 0 | 0 | 0 | 0.965063 | 0 | 0 | 0 | 0 |
| AABR07005 | 0 | 0 | 0 | 0 | 0 | 0 | 0 | 0 |
| LOC100909 | 9.448597 | 12.03417 | 8.158645 | 10.83234 | 15.64594 | 13.71336 | 8.97653 | 14.46413 |
| Glb1l | 3.179197 | 3.340176 | 3.46183 | 4.708133 | 5.107321 | 4.460037 | 5.436738 | 4.428098 |
| AABR07035 | 0.170891 | 0.138111 | 0.262089 | 0.247452 | 0.10559 | 0.171243 | 0.218498 | 0.302939 |
| Tpr | 11.51396 | 10.49285 | 10.64314 | 11.23791 | 11.24627 | 10.47365 | 12.39875 | 12.3883 |
| Rab17 | 0.033719 | 0.076303 | 0 | 0.062142 | 0.07292 | 0.088695 | 0 | 0.059774 |
| Zfp629 | 10.0286 | 10.38692 | 11.22177 | 9.006418 | 8.641997 | 9.953594 | 10.49816 | 11.25018 |
| Serpine3 | 0.173254 | 0.147021 | 0.232499 | 0.199558 | 0.327839 | 0.075954 | 0.484571 | 0.230345 |
| Pcolce | 16.61833 | 16.07402 | 15.21615 | 29.53031 | 30.6869 | 28.73638 | 29.49562 | 31.09919 |
| Rbm3 | 47.79542 | 47.18319 | 43.36178 | 58.89625 | 61.14312 | 56.0107 | 45.82129 | 45.4096 |
| Fam122b | 1.188985 | 0.171738 | 0.135793 | 0.186486 | 0.136769 | 1.197769 | 0.056604 | 0.134535 |
| Mospd3 | 12.11256 | 13.52757 | 14.18065 | 13.56668 | 12.70196 | 11.99361 | 12.49962 | 15.45608 |
| #N/A | 0 | 0 | 0 | 0 | 0 | 0.168803 | 0.43077 | 0 |
| Jmjd8 | 37.15081 | 36.01293 | 36.99045 | 38.9483 | 38.7689 | 40.67994 | 42.74581 | 40.27631 |
| Vip | 0.034957 | 0 | 0.112586 | 0.064423 | 0.113396 | 0.091952 | 0.039109 | 0.030984 |
| Mrgprx3 | 0.122255 | 0.092217 | 0 | 0.375511 | 0.220321 | 0.393042 | 0.182365 | 0.288962 |
| Pdha1 | 326.735 | 322.9875 | 351.6175 | 338.5955 | 338.7118 | 329.5839 | 325.1567 | 318.8901 |
| AABR07051 | 0 | 0 | 0 | 0 | 0 | 0 | 0 | 0 |

| | | | | | | | | |
|---|---|---|---|---|---|---|---|---|
| Ccdc15 | 0.50071 | 0.542924 | 0.38076 | 0.557506 | 0.383499 | 0.493901 | 0.536832 | 0.554755 |
| Fam134b | 1.053337 | 0.510772 | 0.969279 | 1.432804 | 1.301667 | 1.099485 | 1.178427 | 7.913586 |
| AABR07048 | 0 | 0 | 0 | 0 | 0.445847 | 0 | 0 | 0 |
| Tmem87b | 5.001813 | 5.094668 | 4.907065 | 5.528678 | 5.72725 | 5.370115 | 5.836895 | 6.031776 |
| Cpne8 | 2.866182 | 2.436595 | 2.378461 | 2.526865 | 3.367197 | 2.988517 | 3.570545 | 3.748865 |
| Trem3 | 0 | 0.199047 | 0 | 0.054035 | 0.253628 | 0.25708 | 0.065604 | 0.155928 |
| Igtp | 11.43777 | 10.48245 | 9.721014 | 13.12162 | 12.24382 | 12.83667 | 14.45956 | 14.37887 |
| Sema7a | 22.42827 | 22.5745 | 23.22054 | 16.69168 | 17.06018 | 18.42183 | 21.53914 | 20.28644 |
| Cachd1 | 1.469302 | 1.180337 | 1.403877 | 1.516372 | 1.31865 | 1.971076 | 2.104051 | 2.044635 |
| Trim63 | 40.93159 | 40.56221 | 40.23631 | 42.01789 | 42.52661 | 37.72931 | 48.44977 | 47.78541 |
| Rn50_X_07 | 0.074331 | 0.042051 | 0.039899 | 0.136985 | 0.080373 | 0.065173 | 0 | 0.098824 |
| Eif1a | 17.57674 | 16.58988 | 14.81226 | 20.47611 | 19.8425 | 18.6852 | 23.5867 | 21.51212 |
| Apoa5 | 0.053244 | 0.030122 | 0.057161 | 0.049063 | 0 | 0.09337 | 0.059568 | 0.07079 |
| AABR07039 | 0.020463 | 0 | 0.054922 | 0.018856 | 0.022127 | 0.026914 | 0.022894 | 0.009069 |
| Nckap5l | 5.296575 | 5.519033 | 6.050712 | 4.797156 | 4.442181 | 4.634118 | 6.014203 | 6.239427 |
| Tspan10 | 0 | 0 | 0.169843 | 0.048593 | 0.057022 | 0.231191 | 0 | 0 |
| Phospho1 | 77.01641 | 82.81306 | 93.98754 | 55.18092 | 52.23207 | 51.35807 | 62.45319 | 61.36747 |
| Rpl13a | 730.8126 | 701.0232 | 714.0258 | 706.4511 | 692.7006 | 646.0288 | 804.3904 | 778.4291 |
| AABR07049 | 0.286153 | 0.971305 | 0.307203 | 0.527357 | 0.773532 | 0.125449 | 0.160067 | 0.126816 |
| Hhatl | 69.92558 | 75.67201 | 76.78057 | 51.87212 | 48.31377 | 46.46346 | 52.567 | 51.61093 |
| Nap1l4 | 91.84647 | 87.19879 | 86.31045 | 95.54965 | 89.70459 | 89.90137 | 91.75418 | 89.28367 |
| Dynlt1 | 11.27771 | 11.21597 | 12.33869 | 17.20962 | 12.34977 | 14.23412 | 13.42063 | 13.2431 |
| AABR07047 | 0.766331 | 0.481704 | 0.365647 | 0.549223 | 0.828622 | 0.373289 | 1.619414 | 0.452825 |
| Bphl | 16.52495 | 17.34378 | 13.74539 | 13.92074 | 15.6646 | 16.19826 | 13.82836 | 13.54738 |
| Mpv17l2 | 8.531874 | 8.848963 | 10.12192 | 9.314507 | 9.359093 | 9.920131 | 10.82468 | 9.726773 |
| Mdfic | 6.590007 | 5.69674 | 4.61184 | 4.284754 | 4.977986 | 4.401094 | 5.87397 | 6.182234 |
| Atp5a1 | 2028.084 | 2033.607 | 2031.924 | 1932.052 | 1912.729 | 1960.224 | 1722.984 | 1746.844 |
| Tmem260 | 1.708911 | 1.726386 | 1.725421 | 2.212071 | 2.122794 | 2.051346 | 2.173596 | 2.443344 |
| AABR07031 | 0.480421 | 0 | 0 | 0 | 0 | 0.842468 | 0 | 0 |
| AABR07013 | 0.294604 | 0.333331 | 0.158138 | 0.271466 | 0.398189 | 0.322887 | 0.411988 | 0.848648 |
| Wtip | 7.376127 | 7.51635 | 9.345101 | 8.780973 | 7.381237 | 8.877604 | 9.379697 | 10.19251 |
| AABR07061 | 0 | 0 | 0.429146 | 0 | 0 | 0 | 0 | 0 |
| Pate2 | 0 | 0 | 0 | 0 | 0 | 0 | 0 | 0 |
| Gzmk | 2.956141 | 2.442187 | 2.720234 | 2.075404 | 1.674322 | 2.098243 | 1.994818 | 2.287454 |
| LOC498705 | 0 | 0 | 0 | 0.103105 | 0 | 0.196216 | 0 | 0.099176 |
| AABR07030 | 0 | 0 | 0.147554 | 0 | 0.148616 | 0.241021 | 0.153766 | 0 |
| Fastkd1 | 3.491061 | 3.166644 | 3.368346 | 3.406902 | 3.153659 | 3.099712 | 3.559575 | 3.903781 |
| 5S_rRNA | 0 | 0 | 0 | 0 | 0 | 0 | 0 | 0 |
| RGD156480 | 0.145158 | 0.32848 | 0.194796 | 0.100318 | 0.235437 | 0.159094 | 0.324794 | 0.225157 |
| Slc41a2 | 0.222584 | 0.185569 | 0.163498 | 0.690873 | 0.608027 | 0.708747 | 0.786371 | 0.872219 |
| Vwa8 | 3.592672 | 4.06494 | 3.545289 | 1.939488 | 1.765775 | 2.32277 | 3.491532 | 3.152199 |
| Tmem248 | 18.94751 | 18.25782 | 17.58447 | 17.58729 | 18.31132 | 21.99879 | 21.85763 | 21.40789 |
| Inpp4b | 10.27059 | 10.60189 | 10.01414 | 8.763869 | 8.975627 | 9.251979 | 8.326515 | 9.527322 |
| Nars | 18.35099 | 16.42679 | 16.9057 | 21.8809 | 19.50481 | 20.29077 | 21.57892 | 19.82864 |
| RGD156245 | 3.317052 | 2.461042 | 3.298366 | 2.480301 | 2.41072 | 2.121697 | 2.403006 | 2.940068 |
| AABR07061 | 0 | 0 | 0 | 0 | 0 | 0 | 0 | 0 |
| RGD156368 | 0.834903 | 0.856779 | 0.70872 | 0.984028 | 0.86078 | 0.749068 | 0.847164 | 0.894904 |
| Mei1 | 0.013033 | 0 | 0 | 0 | 0 | 0.011427 | 0.014581 | 0.023103 |
| Fam60a | 1.461051 | 1.469431 | 1.817505 | 1.730958 | 0.601834 | 1.545395 | 0.778361 | 2.137781 |
| Pafah2 | 3.06746 | 2.912898 | 2.852072 | 2.776069 | 3.139114 | 2.545468 | 2.910849 | 2.913045 |
| Smarca5 | 6.872399 | 7.150321 | 5.840322 | 7.247232 | 7.268005 | 7.050209 | 7.547976 | 8.229445 |
| Tpbgl | 1.632185 | 1.590248 | 1.460212 | 1.963547 | 1.911929 | 1.550359 | 1.826016 | 2.491515 |
| Them4 | 7.366857 | 6.6766 | 6.653744 | 7.318335 | 6.882183 | 7.110074 | 7.058393 | 6.842112 |
| Edf1 | 121.1679 | 132.079 | 135.4981 | 134.6861 | 132.5876 | 127.0859 | 136.7546 | 136.601 |

| | | | | | | | | |
|---|---|---|---|---|---|---|---|---|
| Tcf15 | 0 | 0 | 0 | 0.13157 | 0 | 0.125193 | 0 | 0.063278 |
| Fam129c | 0.043986 | 0.049769 | 0.047222 | 0.060798 | 0.095124 | 0.077135 | 0.024605 | 0.019494 |
| Adcy10 | 0.201857 | 0.19233 | 0.136867 | 0.107686 | 0.137852 | 0.158358 | 0.106971 | 0.131833 |
| AABR07011 | 0.429229 | 0.485652 | 0.460805 | 0.791035 | 0 | 0 | 0 | 0.380447 |
| AABR07072 | 0 | 0.412889 | 0 | 0.168129 | 0 | 0 | 0 | 0.161723 |
| Il12rb1 | 0.307062 | 0.400877 | 0.329651 | 0.413536 | 0.178781 | 0.248522 | 0.211402 | 0.376843 |
| Dusp19 | 3.938088 | 3.793418 | 2.799482 | 3.726868 | 2.819617 | 4.152835 | 3.929459 | 4.433907 |
| Ubxn8 | 2.502838 | 2.966693 | 2.575 | 2.443544 | 2.674065 | 3.278673 | 3.050069 | 3.420017 |
| Fis1 | 143.0528 | 147.4732 | 151.9515 | 154.2985 | 144.7311 | 141.8837 | 145.0055 | 142.0874 |
| LOC100294 | 21.22114 | 20.73527 | 22.28183 | 16.57041 | 15.51847 | 16.95039 | 21.38086 | 19.52693 |
| AABR07060 | 0.086128 | 0 | 0 | 0.158727 | 0.186258 | 0.075517 | 0.192713 | 0.07634 |
| Ccdc24 | 0 | 0 | 0.081772 | 0.035093 | 0.12354 | 0.100177 | 0.085214 | 0.101268 |
| Polr2h | 0.115598 | 0 | 0 | 0.106519 | 0 | 0 | 0 | 0 |
| AABR07017 | 0 | 0.173244 | 0 | 0 | 0.165563 | 0 | 0 | 0 |
| Nlrc3 | 1.090957 | 1.017811 | 1.253402 | 0.811273 | 1.055463 | 0.906207 | 0.963564 | 1.136613 |
| AABR07070 | 2.651439 | 2.74998 | 3.320904 | 2.239597 | 2.866963 | 3.099712 | 1.977541 | 2.741786 |
| Sfrp1 | 2.48913 | 2.298737 | 2.325571 | 6.236209 | 5.48476 | 6.559212 | 6.367244 | 6.845307 |
| AABR07012 | 1.415295 | 0.80067 | 0 | 0.652069 | 0 | 0 | 0.791685 | 2.508893 |
| Rn60_12_0 | 0.509231 | 0.768228 | 0.546692 | 0.469236 | 0.642395 | 0.818573 | 0.284853 | 0.526583 |
| Mccc2 | 104.7318 | 100.4293 | 103.9608 | 99.20925 | 96.64202 | 98.84633 | 85.81073 | 85.70316 |
| Smox | 3.650509 | 4.443718 | 4.405558 | 4.523731 | 3.212239 | 3.92922 | 4.281187 | 4.1059 |
| A930009A1 | 0 | 0 | 0 | 0 | 0 | 0 | 0 | 0.134535 |
| Taok3 | 1.594148 | 1.740784 | 1.532318 | 1.998448 | 1.463166 | 1.852832 | 2.965525 | 2.940955 |
| Ift74 | 2.28542 | 2.782594 | 2.160186 | 2.929982 | 3.062128 | 2.831538 | 2.44566 | 2.620174 |
| Mink1 | 20.34757 | 22.35808 | 22.04609 | 20.16678 | 20.27492 | 19.7144 | 25.31225 | 23.76923 |
| Ptpn13 | 3.137129 | 3.352319 | 3.16641 | 3.187227 | 3.254417 | 3.356013 | 3.592173 | 4.063944 |
| Hdac11 | 0.071981 | 0.28505 | 0.579569 | 0.397964 | 0.505906 | 0.410233 | 0.603966 | 0.5104 |
| Tex21 | 0 | 0.090781 | 0.028712 | 0.024644 | 0.028919 | 0.02345 | 0.029921 | 0 |
| Cds2 | 27.02216 | 25.21905 | 26.45137 | 20.87876 | 18.94676 | 17.51226 | 24.18495 | 25.11144 |
| Gnpda1 | 6.130393 | 6.55738 | 6.221882 | 7.096747 | 7.297146 | 7.520671 | 6.541426 | 7.83088 |
| RGD156395 | 0.557084 | 0.210105 | 0.39871 | 0.17111 | 0.602367 | 0.162817 | 0.207747 | 0.082295 |
| Zcchc8 | 4.306456 | 3.861581 | 3.852713 | 4.008722 | 4.593155 | 4.289633 | 4.5229 | 4.440214 |
| AABR07065 | 0 | 0 | 0.160166 | 1.374733 | 0.967906 | 1.308105 | 1.669079 | 1.454586 |
| Stat1 | 33.8545 | 33.31879 | 31.64861 | 44.44446 | 41.3713 | 38.90604 | 43.77656 | 44.66114 |
| AABR07026 | 2.344743 | 0 | 2.517231 | 0 | 0.845112 | 2.055872 | 0 | 0 |
| Rn50_1_18 | 0 | 0 | 0 | 0 | 0 | 0 | 0 | 0 |
| Kif19 | 0.090494 | 0.03413 | 0.080959 | 0.055591 | 0.114158 | 0.026448 | 0.05062 | 0.106946 |
| #N/A | 0 | 0 | 0 | 0 | 0 | 0 | 0 | 0.081572 |
| AABR07058 | 0.831205 | 0 | 0 | 0.765923 | 0 | 0 | 0 | 1.473477 |
| Rilpl1 | 88.17913 | 89.51375 | 96.10677 | 79.21412 | 74.74986 | 76.86134 | 87.58724 | 89.86552 |
| Ormdl1 | 4.115479 | 5.941014 | 3.656466 | 3.530717 | 4.296559 | 4.355032 | 4.286683 | 5.534522 |
| Rbm11 | 0 | 0 | 0 | 0.026041 | 0.061114 | 0.024778 | 0 | 0.025048 |
| Nprl2 | 10.60274 | 10.45354 | 9.516095 | 8.513411 | 9.252767 | 10.0139 | 9.230136 | 8.974682 |
| Ddo | 3.923314 | 3.691417 | 2.349813 | 2.816035 | 1.875509 | 2.788182 | 2.125311 | 1.46418 |
| Mroh6 | 0.04842 | 0 | 0 | 0.111542 | 0.078533 | 0.063682 | 0.05417 | 0.107292 |
| Edar | 0.014355 | 0 | 0.046232 | 0.119046 | 0.124172 | 0.050345 | 0.465722 | 0.139956 |
| Etfbkmt | 15.62821 | 15.91063 | 14.15086 | 14.972 | 15.20517 | 15.9914 | 15.69558 | 17.3223 |
| Tmem8b | 2.546334 | 2.380004 | 2.812889 | 2.516372 | 1.955252 | 1.779631 | 2.559725 | 2.780292 |
| Ptges3l1 | 0.717341 | 0.590282 | 0.770112 | 0.751138 | 0.282055 | 0.829091 | 0.583658 | 0.751418 |
| LOC654482 | 1.034585 | 0.900449 | 0.683503 | 1.283328 | 1.333813 | 0.732678 | 0.667758 | 0.846465 |
| Pigx | 19.77908 | 20.72818 | 20.07377 | 20.01833 | 19.34164 | 18.5269 | 23.15576 | 22.17742 |
| Nedd4l | 6.563928 | 7.335313 | 7.098866 | 5.63127 | 6.03111 | 5.514274 | 5.69749 | 7.236595 |
| AABR07029 | 0 | 0.481704 | 0 | 0 | 0.920691 | 0.746577 | 0.476298 | 0.377354 |
| Rbm42 | 23.39139 | 21.5288 | 28.41911 | 24.2744 | 24.04375 | 24.92661 | 29.11285 | 28.32651 |

| | | | | | | | | |
|---|---|---|---|---|---|---|---|---|
| Kat6a | 8.40609 | 8.155845 | 7.992666 | 6.972614 | 7.13501 | 6.515191 | 9.452505 | 9.446921 |
| Dctn2 | 50.19212 | 49.85187 | 46.7788 | 51.60156 | 48.90445 | 51.23419 | 54.59191 | 56.33584 |
| Bid | 1.445846 | 1.754882 | 1.58043 | 2.034771 | 1.847622 | 1.774807 | 2.058698 | 2.376647 |
| Arl1 | 50.69999 | 50.07626 | 41.37371 | 53.21661 | 54.40084 | 54.4352 | 53.05547 | 53.64987 |
| Fchsd1 | 0.920551 | 0.607577 | 0.916209 | 1.281213 | 0.91243 | 1.185487 | 1.062055 | 1.028412 |
| Slc25a13 | 39.34657 | 36.31689 | 38.21441 | 31.67027 | 32.60084 | 33.4556 | 34.05335 | 36.15346 |
| Apoc4 | 0.098804 | 0.111792 | 0.106072 | 0 | 0 | 0 | 0 | 0 |
| Dctpp1 | 7.631623 | 6.062747 | 8.803154 | 9.276572 | 6.93516 | 8.043933 | 8.810407 | 9.570746 |
| Zfp347 | 3.154574 | 3.297309 | 2.773816 | 3.405127 | 4.060708 | 3.398146 | 3.764477 | 4.100881 |
| AABR07055 | 1.274514 | 1.316657 | 0.951842 | 1.174415 | 1.108483 | 1.141789 | 0.929915 | 1.154223 |
| Dolpp1 | 2.093368 | 2.117338 | 2.417619 | 2.542715 | 2.194937 | 2.64196 | 2.412936 | 2.83941 |
| Zfp446 | 1.522757 | 1.531491 | 1.543956 | 1.675997 | 1.326375 | 1.353698 | 1.561627 | 1.855831 |
| AABR07009 | 0 | 0 | 0 | 0 | 0 | 0 | 0 | 0 |
| Adamts14 | 0.61979 | 0.815755 | 0.801177 | 0.641044 | 0.697524 | 0.621066 | 0.778299 | 0.605407 |
| RGD130564 | 3.155982 | 3.636972 | 2.823457 | 10.07069 | 8.21532 | 9.428868 | 6.342313 | 5.335597 |
| Abi2 | 7.928298 | 7.272378 | 7.390674 | 8.417992 | 7.232159 | 7.437861 | 9.344345 | 8.964798 |
| P4hb | 73.23655 | 72.58014 | 70.98983 | 86.50281 | 85.10439 | 84.91136 | 98.0334 | 95.55162 |
| Dfna5 | 0.611573 | 0.994701 | 0.861738 | 0.986195 | 0.991927 | 0.770827 | 0.898013 | 1.253531 |
| Gsta2 | 0.657736 | 0.515214 | 0.543171 | 0.932428 | 1.039447 | 1.597026 | 0.396225 | 0.448449 |
| Zbtb12 | 1.413532 | 1.550134 | 1.587556 | 1.583056 | 1.222745 | 1.353791 | 1.435422 | 1.060132 |
| Acad10 | 19.26705 | 20.26744 | 21.9057 | 17.17146 | 16.77198 | 18.47662 | 15.63605 | 15.12473 |
| Kif4a | 0.26326 | 0.116557 | 0.233474 | 0.179301 | 0.160895 | 0.150539 | 0.179275 | 0.182614 |
| Ccdc91 | 11.14625 | 11.03502 | 11.02272 | 10.78437 | 11.05564 | 12.06594 | 12.61381 | 11.07641 |
| Tcaim | 17.44555 | 17.98352 | 15.24081 | 17.69372 | 15.98344 | 16.32287 | 14.89996 | 15.80013 |
| Cux1 | 31.83559 | 31.72257 | 35.63396 | 33.83553 | 28.36015 | 28.15137 | 38.65171 | 36.39411 |
| Ciart | 11.36732 | 11.64601 | 11.9431 | 9.548436 | 9.255963 | 11.57738 | 4.730198 | 3.532541 |
| Rabep1 | 7.536947 | 7.433968 | 6.599594 | 8.837825 | 8.868193 | 9.13787 | 9.107913 | 10.13435 |
| Tpgs2 | 9.081477 | 10.40871 | 10.76249 | 10.43311 | 10.01096 | 10.65133 | 10.62177 | 9.931034 |
| Grin2b | 0.009954 | 0 | 0.010686 | 0.009172 | 0 | 0.008727 | 0 | 0.017645 |
| Cenpi | 0.154986 | 0.175359 | 0.166387 | 0.10711 | 0.125688 | 0.169865 | 0.130044 | 0.1202 |
| Higd2a | 85.54829 | 81.44372 | 83.12124 | 87.2697 | 86.52219 | 86.60108 | 80.04638 | 82.87213 |
| Med21 | 21.89608 | 18.95389 | 20.67469 | 20.66255 | 23.17672 | 20.0659 | 24.71771 | 22.68115 |
| LOC103689 | 0.046486 | 0.026298 | 0.024953 | 0.021417 | 0.829358 | 0 | 0.026003 | 0 |
| AABR07062 | 5.278823 | 5.614375 | 4.911533 | 5.091225 | 5.631809 | 4.875328 | 6.260055 | 6.644014 |
| Rn50_5_10 | 0 | 0.052387 | 0 | 0 | 0.050064 | 0 | 0.051799 | 0.041038 |
| Chkb | 23.51632 | 24.10212 | 26.15032 | 24.1122 | 23.53932 | 25.37739 | 26.76262 | 28.19703 |
| Lax1 | 0.473685 | 0.401965 | 0.203413 | 0.327362 | 0.307314 | 0.353029 | 0.34446 | 0.377866 |
| Slc9a9 | 1.657149 | 1.857941 | 1.64967 | 2.290497 | 2.101353 | 1.968141 | 2.140464 | 1.922811 |
| Ring1 | 23.7767 | 24.36834 | 26.65119 | 25.64134 | 23.39092 | 21.47411 | 30.01198 | 28.33862 |
| AABR07058 | 0 | 0 | 0 | 0 | 0 | 0 | 0 | 0 |
| Hist1h2ah | 0.133247 | 0.150762 | 0.286098 | 0 | 0.432233 | 0.467323 | 0 | 0 |
| Nfkbid | 0.20174 | 0.195651 | 0.09282 | 0.212452 | 0.249301 | 0.227425 | 0.12897 | 0.28099 |
| Klhl38 | 10.17042 | 10.80893 | 9.924565 | 7.367304 | 7.485042 | 8.685479 | 8.171928 | 7.67279 |
| Traf7 | 13.07646 | 13.97973 | 14.89835 | 14.33755 | 15.00551 | 15.52138 | 16.80249 | 15.9744 |
| Adam11 | 5.033591 | 5.203088 | 5.537311 | 4.199244 | 3.919676 | 3.559826 | 4.982478 | 4.883806 |
| Ppard | 16.46092 | 16.89003 | 19.50921 | 16.74513 | 15.24156 | 14.90189 | 21.03517 | 20.9845 |
| AABR07058 | 0.308035 | 0.290439 | 0.330695 | 0.141921 | 0.388586 | 0.090028 | 0.746667 | 0.273027 |
| Pmm1 | 19.22127 | 18.82512 | 18.47302 | 23.8845 | 22.10929 | 27.14176 | 22.04273 | 24.60435 |
| Cpt1c | 1.745531 | 1.627736 | 1.750382 | 2.350794 | 2.219271 | 2.724596 | 3.176019 | 2.703263 |
| Cisd3 | 48.30003 | 49.65225 | 47.48817 | 50.4494 | 51.01332 | 47.38965 | 46.42854 | 46.16598 |
| Zfp1 | 3.29182 | 2.597376 | 1.487991 | 2.753901 | 2.201206 | 2.240657 | 1.453714 | 2.265059 |
| Scn2b | 0.353483 | 0 | 0.108425 | 0.511846 | 0.273011 | 0.398486 | 0.847416 | 0.447585 |
| Tacc3 | 1.621582 | 1.265341 | 1.530767 | 1.726087 | 1.541777 | 1.127638 | 1.814154 | 1.362946 |
| Rp2 | 0.799861 | 0.650473 | 1.127047 | 1.243756 | 0.648659 | 0.964314 | 1.342274 | 1.440069 |

| | | | | | | | | |
|---|---|---|---|---|---|---|---|---|
| Rfx4 | 0.185977 | 0.180364 | 0.057045 | 0.367223 | 0.114911 | 0.209655 | 0.416126 | 0.565169 |
| Maml1 | 2.974576 | 2.99765 | 3.131788 | 2.776208 | 2.626871 | 2.36491 | 3.79864 | 3.908145 |
| Trpm8 | 0.050388 | 0.028506 | 0 | 0.034823 | 0.013621 | 0.055226 | 0.042279 | 0.022331 |
| Pou5f1 | 0.287725 | 0.195328 | 0 | 0.106051 | 0.062223 | 0.050455 | 0.386273 | 0.051005 |
| LOC100362 | 1.47807 | 0.557456 | 1.360117 | 2.334829 | 1.750427 | 2.777086 | 2.047314 | 3.368796 |
| Lcn2 | 7.893176 | 7.864398 | 5.564902 | 5.156409 | 5.286466 | 5.009792 | 7.776142 | 7.9881 |
| Zbtb42 | 3.692074 | 4.629961 | 5.417026 | 4.309328 | 4.457942 | 4.61303 | 5.197582 | 5.69955 |
| Ankdd1b | 0.191817 | 0.162774 | 0.205927 | 0.132564 | 0.103704 | 0 | 0.268245 | 0.127512 |
| Far2 | 0.095996 | 0.108615 | 0.103058 | 0.088457 | 0.038925 | 0.063127 | 0.067123 | 0.085086 |
| LOC367746 | 0.073963 | 0.083686 | 0.158808 | 0.136308 | 0 | 0 | 0.082747 | 0 |
| AABR07026 | 0.078236 | 0.059014 | 0.09799 | 0.204259 | 0.098695 | 0.011433 | 0.145878 | 0.288935 |
| R3hdm4 | 39.40721 | 39.05905 | 41.30838 | 39.93363 | 34.54203 | 35.34391 | 41.32417 | 39.65946 |
| Dnmbp | 3.482442 | 3.608616 | 3.553543 | 2.962703 | 2.824135 | 3.121429 | 3.172733 | 3.774283 |
| AC111678. | 0 | 0 | 0 | 0 | 0 | 0 | 0 | 0 |
| Map4k1 | 2.618296 | 2.602749 | 2.770752 | 2.981354 | 3.073794 | 2.984442 | 3.410464 | 3.016944 |
| Echdc3 | 24.19525 | 29.08193 | 26.93174 | 19.38968 | 16.37903 | 21.81539 | 18.02015 | 20.71643 |
| Lactb | 8.807339 | 7.630411 | 7.672253 | 7.906929 | 8.108366 | 7.788474 | 8.670868 | 9.189227 |
| Ppp1r1c | 0 | 0 | 0 | 0.028185 | 0.066148 | 0 | 0.03422 | 0 |
| Nudt2 | 27.31129 | 28.74095 | 23.79177 | 25.91273 | 26.27785 | 27.19355 | 27.31793 | 27.02812 |
| Opcml | 1.746796 | 1.632693 | 1.793763 | 1.434647 | 2.258331 | 1.398411 | 1.699338 | 2.490699 |
| Dhx37 | 4.280323 | 4.507055 | 4.781134 | 4.194934 | 4.066441 | 3.763838 | 4.899355 | 4.660092 |
| Panx3 | 0 | 0 | 0 | 0.079691 | 0.093514 | 0.189573 | 0.145131 | 0.15331 |
| RGD156155 | 0 | 0 | 0 | 0 | 0 | 0 | 0 | 0 |
| AC128440. | 0 | 0 | 0 | 0 | 0 | 0 | 0 | 0 |
| Cox6b1 | 221.3012 | 241.0845 | 226.8112 | 229.9881 | 220.6325 | 222.8876 | 178.672 | 194.3719 |
| LOC257642 | 2.945583 | 43.78914 | 29.60238 | 22.24168 | 26.71875 | 20.87674 | 2.013848 | 2.175681 |
| LOC100911 | 1.016109 | 0.43113 | 3.50633 | 1.23726 | 0.490493 | 0.190913 | 0.263895 | 1.608265 |
| AABR07066 | 2.310261 | 0 | 0.826738 | 1.064407 | 0 | 1.688033 | 1.723078 | 2.388983 |
| Arpc1b | 52.05504 | 53.2699 | 55.9215 | 74.41677 | 69.13474 | 76.11023 | 78.67967 | 79.63335 |
| Pggt1b | 7.605287 | 7.126628 | 6.222484 | 7.903265 | 6.919322 | 6.962081 | 8.283564 | 7.423947 |
| Ezh2 | 1.799259 | 2.396278 | 1.971861 | 2.072432 | 2.107638 | 2.218488 | 2.474227 | 2.574893 |
| Vsx2 | 0 | 0.076773 | 0.018211 | 0.062524 | 0.036684 | 0.014873 | 0 | 0.045106 |
| Wdfy2 | 6.237605 | 6.132986 | 5.950791 | 6.412835 | 7.363136 | 6.830451 | 7.999207 | 7.568765 |
| Vom2r44 | 0.045208 | 0.01705 | 0.016178 | 0.027772 | 0.05703 | 0.039638 | 0.025288 | 0.066783 |
| Mettl25 | 2.580283 | 2.111402 | 2.522768 | 2.589918 | 1.768675 | 2.545194 | 2.268126 | 2.593332 |
| Osr1 | 1.172729 | 0.920697 | 0.924979 | 3.61678 | 3.726527 | 3.840197 | 3.721786 | 3.733526 |
| AABR07053 | 0 | 0.186613 | 0.177065 | 0 | 0.089169 | 0.289225 | 0.184519 | 0.073094 |
| AABR07034 | 0 | 0 | 0 | 0.455218 | 0 | 0 | 0 | 0.875746 |
| AABR07000 | 0 | 0 | 0 | 0 | 0 | 0.038714 | 0 | 0 |
| Lancl3 | 0.165846 | 0.140735 | 0.178046 | 0.07641 | 0.224159 | 0 | 0.046385 | 0.073499 |
| Dennd5b | 5.104517 | 5.250474 | 5.039324 | 5.279228 | 4.998374 | 4.538242 | 6.449505 | 6.311995 |
| Tas2r103 | 0 | 0 | 0 | 0 | 0.060301 | 0 | 0 | 0 |
| LOC687679 | 1.782086 | 2.17145 | 1.177344 | 2.021074 | 1.778718 | 1.92312 | 1.380267 | 1.701056 |
| Mcm3 | 1.28593 | 1.475177 | 1.227136 | 2.172379 | 1.911879 | 2.082751 | 2.137981 | 2.184585 |
| #N/A | 0 | 0 | 0 | 0.14447 | 0 | 0 | 0 | 0 |
| Sesn2 | 3.599369 | 3.676358 | 3.277764 | 3.471541 | 3.483064 | 3.757644 | 3.337399 | 4.344766 |
| Twf2 | 237.1362 | 245.5374 | 269.7548 | 231.0782 | 227.0985 | 228.5566 | 217.6743 | 211.6819 |
| Ncln | 19.45219 | 19.8024 | 24.2858 | 20.97334 | 18.92438 | 22.1098 | 29.71121 | 27.75301 |
| Ap3b2 | 0.014454 | 0 | 0.031034 | 0.026637 | 0.031257 | 0.063365 | 0.064681 | 0.051244 |
| Trpc3 | 0.853519 | 1.136138 | 0.898341 | 0.956118 | 0.850514 | 1.012496 | 0.917433 | 0.756517 |
| Phkb | 12.9515 | 12.82018 | 12.4648 | 13.19697 | 14.08393 | 13.29376 | 11.48945 | 12.35457 |
| Gde1 | 29.15099 | 28.57669 | 30.72718 | 29.30028 | 26.57374 | 29.60407 | 30.96316 | 28.41842 |
| Usp45 | 5.651513 | 4.9577 | 4.742447 | 5.158207 | 5.143985 | 5.676586 | 5.042123 | 6.372485 |
| Cdk9 | 15.82865 | 15.50258 | 15.48182 | 15.3061 | 14.67991 | 14.7837 | 16.69348 | 16.52512 |

| | | | | | | | | |
|---|---|---|---|---|---|---|---|---|
| Dgcr6 | 30.57281 | 30.27265 | 33.79051 | 32.5205 | 32.57014 | 30.85307 | 37.46468 | 35.74133 |
| NEWGENE_1 | 3.665944 | 3.725607 | 3.146143 | 3.549958 | 3.785911 | 3.589625 | 3.683798 | 3.609261 |
| AABR07051 | 0.111893 | 0 | 0.120124 | 0.309315 | 0.241977 | 0.098108 | 0.500724 | 0 |
| Cd163l1 | 0.079559 | 0.054011 | 0.051247 | 0.219932 | 0.464542 | 0.446449 | 0.320427 | 0.423104 |
| Pbrm1 | 5.552578 | 5.807161 | 5.274742 | 5.326863 | 5.243557 | 5.581164 | 6.559357 | 6.50807 |
| Bpifc | 0 | 0 | 0 | 0 | 0 | 0 | 0 | 0 |
| Cdyl2 | 1.61008 | 1.763897 | 1.509028 | 1.412976 | 1.215906 | 1.501352 | 1.887061 | 1.676269 |
| Ranbp17 | 0.26326 | 0.479177 | 0.344067 | 0.527357 | 0.457931 | 0.622229 | 0.320135 | 0.4261 |
| Sod3 | 33.63783 | 36.68811 | 40.86227 | 56.01608 | 55.47564 | 54.65631 | 55.90632 | 54.12341 |
| Ssc4d | 2.854701 | 2.657988 | 2.426224 | 2.740099 | 2.411521 | 2.894099 | 2.594892 | 2.345765 |
| Acat2 | 1.070679 | 1.284842 | 1.079779 | 1.793797 | 1.192791 | 1.166353 | 0.83485 | 1.23657 |
| Pla2g1b | 0 | 0.208625 | 0 | 0 | 0 | 0.080835 | 0 | 0 |
| Gltscr2 | 60.19777 | 60.36159 | 62.08727 | 60.16784 | 55.1945 | 56.60542 | 69.54567 | 69.19983 |
| AC108572. | 0.39079 | 0.442161 | 0 | 0.360098 | 0 | 0 | 0 | 0.346377 |
| AABR07013 | 0 | 0 | 0 | 0 | 0 | 0 | 0 | 0.142376 |
| Bcl10 | 8.190398 | 7.592282 | 7.521651 | 10.63873 | 10.06543 | 11.19021 | 11.37102 | 10.23335 |
| Urad | 0 | 0 | 0 | 0 | 0.111681 | 0 | 0 | 0 |
| Fbxo38 | 6.457547 | 5.939841 | 6.277844 | 6.953124 | 7.060035 | 6.595161 | 6.876574 | 7.006165 |
| Fam168a | 5.676652 | 5.464832 | 5.198035 | 5.022018 | 4.913044 | 5.270075 | 5.857132 | 6.976448 |
| Pocla | 1.090957 | 1.12703 | 1.57859 | 1.617179 | 1.948963 | 1.247676 | 1.804238 | 1.681685 |
| Layn | 1.726806 | 1.459465 | 1.362458 | 2.051285 | 1.934659 | 1.568791 | 1.629291 | 2.194409 |
| Fer1l5 | 0.235919 | 0.257727 | 0.244541 | 0.202398 | 0.255096 | 0.27105 | 0.309442 | 0.25237 |
| Shroom4 | 4.913994 | 4.406935 | 4.275426 | 3.784898 | 3.663969 | 3.661757 | 4.420428 | 5.208888 |
| Zfp335 | 6.258336 | 6.858427 | 8.091508 | 6.87712 | 6.421384 | 6.737862 | 8.500898 | 9.056226 |
| Cnksr2 | 0.106462 | 0.195742 | 0.08572 | 0.171676 | 0.143895 | 0.151687 | 0.089329 | 0.141544 |
| Tlk1 | 0.832452 | 0.906337 | 2.040311 | 1.534143 | 1.019001 | 1.101727 | 1.054313 | 1.42 |
| Kcnc4 | 0.073522 | 0.12478 | 0.138128 | 0.050811 | 0.099373 | 0.08058 | 0.205632 | 0.016292 |
| Aox1 | 2.465279 | 2.473313 | 2.086017 | 7.139494 | 6.683873 | 6.271714 | 6.127478 | 5.844871 |
| Rn50_20_( | 0 | 0 | 0 | 0 | 0.415324 | 0.11226 | 0 | 0 |
| Alkbh5 | 12.34011 | 11.43043 | 12.54134 | 9.875013 | 9.939184 | 9.809965 | 13.56013 | 13.1349 |
| Ptger3 | 0.962104 | 0.622043 | 0.811548 | 0.633243 | 0.705924 | 0.813446 | 0.884152 | 0.883216 |
| Myo1e | 5.958218 | 5.476187 | 5.702476 | 5.635168 | 5.800171 | 5.270131 | 6.274834 | 7.387296 |
| LOC100912 | 0.117347 | 0.199158 | 0.157474 | 0.621749 | 0.444098 | 0.154334 | 0.131282 | 0.130013 |
| AC123316. | 0 | 0 | 0 | 0 | 0 | 0 | 0.929915 | 0 |
| AABR07035 | 4.37033 | 5.121424 | 3.435089 | 6.472105 | 5.316272 | 4.653033 | 6.635521 | 6.08715 |
| Nfyb | 7.050532 | 7.827536 | 7.889021 | 7.991354 | 7.802597 | 8.329619 | 10.18381 | 9.681916 |
| Lexm | 0 | 0 | 0 | 0 | 0 | 0.023546 | 0 | 0 |
| Ccdc191 | 0.675183 | 0.746172 | 0.758566 | 0.824716 | 0.797978 | 0.743443 | 0.825631 | 0.946383 |
| Ccdc160 | 0 | 0.183435 | 0.17405 | 0.099594 | 0.175302 | 0.14215 | 0.060459 | 0.095799 |
| Cd52 | 0.395962 | 0.560015 | 0.637635 | 0.456079 | 1.39148 | 1.649103 | 0.775222 | 0.965141 |
| LOC100911 | 0.051643 | 0.409021 | 0.27721 | 0.142761 | 0.335044 | 0.316964 | 0.231103 | 0.228868 |
| Cd3eap | 1.848209 | 1.423152 | 1.98417 | 1.726705 | 1.443319 | 1.935611 | 1.751796 | 1.797425 |
| Wee2 | 0.124904 | 0.247315 | 0 | 0.028773 | 0.101293 | 0.191653 | 0.034934 | 0.166063 |
| Klrk1 | 3.080348 | 3.274041 | 3.20674 | 2.709401 | 2.92701 | 2.86454 | 2.715154 | 3.474884 |
| Olr1658 | 0 | 0 | 0 | 0 | 0 | 0 | 0 | 0 |
| Pcnx1 | 15.54747 | 15.47225 | 15.86581 | 13.1638 | 13.99753 | 14.56542 | 17.66582 | 17.43821 |
| Pgm3 | 2.248685 | 2.281079 | 1.831391 | 2.429329 | 2.962481 | 3.512707 | 2.891642 | 2.886589 |
| Col4a5 | 6.183042 | 6.163639 | 6.221396 | 7.10503 | 7.201258 | 7.171702 | 7.369425 | 7.672472 |
| Fam117b | 11.2267 | 11.70741 | 10.36242 | 8.323991 | 9.075099 | 9.015347 | 12.43846 | 12.44329 |
| Grap | 1.842189 | 2.161548 | 2.08758 | 1.414587 | 2.102595 | 1.974173 | 2.175457 | 1.784011 |
| AABR07058 | 0.015888 | 0.02996 | 0.022742 | 0.00976 | 0.011453 | 0.004643 | 0.005925 | 0 |
| Cnr1 | 0.076656 | 0.086733 | 0.041148 | 0.088295 | 0.082887 | 0.126023 | 0.07504 | 0.076437 |
| Mppe1 | 7.097379 | 7.361155 | 7.912547 | 6.686686 | 6.124677 | 7.080644 | 7.787536 | 7.823125 |
| Rpl21 | 0.433672 | 0.981359 | 0.581969 | 0.399612 | 0.937847 | 0.380244 | 0.36388 | 0.672674 |

| | | | | | | | | |
|---|---|---|---|---|---|---|---|---|
| AABR0706[illegible] | 0 | 0 | 0 | 0 | 0 | 0 | 0 | 0 |
| Hsd17b3 | 0.282804 | 0.42664 | 0.202406 | 0.173729 | 0.254827 | 0.247963 | 0.105463 | 0.417772 |
| Tcf25 | 33.08805 | 35.75809 | 35.86505 | 37.13777 | 35.98079 | 33.70687 | 41.32668 | 41.45165 |
| Heatr5b | 3.150356 | 3.062812 | 3.357056 | 2.752407 | 2.65786 | 2.837259 | 3.09806 | 3.426621 |
| Eif2ak2 | 10.00511 | 9.444625 | 11.09707 | 14.05361 | 14.27689 | 13.06468 | 16.73536 | 17.0273 |
| Ppp2r3a | 42.62706 | 39.50954 | 38.21493 | 45.94965 | 46.69738 | 45.44644 | 39.18268 | 39.09806 |
| LOC36110[illegible] | 0.052576 | 0.118975 | 0.056444 | 0 | 0.17055 | 0 | 0.05882 | 0.046601 |
| AABR0702[illegible] | 0 | 0 | 0 | 0 | 0.238914 | 0 | 0 | 0 |
| AABR0707[illegible] | 0 | 0 | 0 | 0 | 0 | 0.107027 | 0 | 0 |
| Pnol | 9.725594 | 10.88657 | 10.29242 | 10.4288 | 11.86341 | 10.49994 | 9.099403 | 10.24617 |
| Eif3l | 41.21018 | 46.21218 | 38.37175 | 41.92984 | 44.95677 | 42.88999 | 42.53239 | 41.24289 |
| Xpnpep1 | 18.88572 | 17.37827 | 19.02067 | 23.45122 | 20.99512 | 21.53231 | 21.03344 | 22.2752 |
| Magoh | 22.82216 | 21.93119 | 20.55739 | 16.63653 | 18.0854 | 14.18552 | 18.887 | 18.49653 |
| AC115420. | 0 | 0.154296 | 0 | 0.251318 | 0 | 0.119569 | 0 | 0 |
| AABR0705[illegible] | 0 | 0 | 0 | 0 | 0 | 0 | 0.082747 | 0 |
| Efcc1 | 4.247561 | 3.578127 | 4.193895 | 3.856823 | 3.821768 | 3.697081 | 5.792563 | 4.259473 |
| Pms2 | 3.021111 | 2.921046 | 3.636489 | 4.285418 | 3.722039 | 4.623557 | 3.318432 | 4.52785 |
| Agrn | 18.09689 | 18.79625 | 19.90091 | 19.44393 | 18.86137 | 19.81553 | 25.23598 | 25.7096 |
| Sbf1 | 9.979728 | 10.16532 | 11.72772 | 9.81526 | 8.647467 | 9.078461 | 11.51705 | 12.29174 |
| Cstf1 | 5.293186 | 5.386325 | 6.450971 | 5.644359 | 6.389381 | 6.815659 | 5.754184 | 6.10795 |
| Apobec3b | 0.453963 | 0.664707 | 0.774039 | 0.688979 | 0.808481 | 0.772656 | 0.716997 | 0.923083 |
| Tmem165 | 10.9154 | 10.83023 | 10.84701 | 12.9208 | 11.95398 | 12.3682 | 13.65201 | 13.14789 |
| RGD1559[illegible] | 3.185586 | 3.139598 | 3.057359 | 3.524153 | 3.424789 | 3.521418 | 3.584315 | 4.02092 |
| Mir361 | 0 | 0 | 0 | 0 | 0 | 0 | 0 | 0 |
| Iqsec1 | 44.5861 | 44.49295 | 47.19851 | 34.04131 | 32.25499 | 31.39418 | 40.90577 | 41.88463 |
| Kif26b | 0.035282 | 0.015968 | 0.037878 | 0.065022 | 0.06867 | 0.068058 | 0.039472 | 0.075054 |
| Spef1 | 7.042554 | 6.942693 | 7.860063 | 8.224236 | 7.313427 | 7.795069 | 11.85735 | 11.12465 |
| LOC10255[illegible] | 0 | 0 | 0 | 0 | 0 | 0.170054 | 0 | 0.171906 |
| Lamtor5 | 46.17018 | 43.54406 | 43.29886 | 48.08845 | 44.25446 | 48.21282 | 42.85545 | 45.30564 |
| Shcbp1l | 0 | 0.114492 | 0 | 0 | 0.082062 | 0.044362 | 0.056604 | 0.08969 |
| Myocd | 9.032522 | 9.377733 | 8.247858 | 7.800013 | 7.829757 | 7.809113 | 7.945836 | 7.961247 |
| Rn50_5_0[illegible] | 0.544388 | 0.165832 | 0.13487 | 0.192935 | 0.271679 | 0.257018 | 0.187396 | 0.352609 |
| Smim7 | 49.01646 | 51.16169 | 48.28096 | 55.81856 | 57.19669 | 52.7551 | 52.14127 | 52.71415 |
| Lrba | 10.34537 | 10.24778 | 9.187446 | 8.962348 | 8.692052 | 8.475443 | 10.57965 | 10.71853 |
| AABR0706[illegible] | 0 | 0 | 0 | 0 | 0.016146 | 0 | 0 | 0 |
| Xab2 | 8.252509 | 7.915506 | 8.497158 | 7.98458 | 7.098094 | 8.354068 | 10.11383 | 9.093389 |
| Mir490 | 3.117019 | 2.116056 | 2.677055 | 1.148884 | 0 | 0.546601 | 0 | 1.657661 |
| AC098265. | 0 | 0 | 0 | 0 | 0 | 0 | 0.320135 | 0.253631 |
| Tom1 | 35.55054 | 36.08885 | 38.31476 | 35.12419 | 31.53754 | 35.04427 | 33.58785 | 32.65827 |
| Hilpda | 5.749983 | 8.364646 | 9.259462 | 7.379891 | 10.65836 | 7.742443 | 11.71693 | 11.83115 |
| AABR0703[illegible] | 0 | 0 | 0 | 0.017547 | 0 | 0 | 0 | 0 |
| Gemin5 | 2.333187 | 2.237397 | 1.999367 | 2.043889 | 2.172132 | 1.944829 | 2.118646 | 2.457512 |
| AC122630. | 0 | 0 | 0 | 0 | 0 | 0.413644 | 0 | 0 |
| Mir365b | 0 | 0 | 0 | 0 | 0 | 0 | 0 | 0.539704 |
| RT1-CE1 | 19.29698 | 10.29628 | 14.21809 | 14.59947 | 10.63045 | 15.63728 | 15.2711 | 24.2695 |
| Slc39a14 | 9.377978 | 10.34735 | 9.508533 | 8.866156 | 9.409118 | 8.834942 | 11.5954 | 12.16367 |
| Nanp | 1.817987 | 1.833383 | 2.673014 | 2.367135 | 2.393102 | 2.287061 | 2.652891 | 2.802386 |
| Ube2l6 | 5.091647 | 4.078927 | 4.588423 | 6.369825 | 6.269065 | 6.256623 | 7.733675 | 8.070658 |
| Gpr151 | 0 | 0 | 0.0806 | 0.06918 | 0 | 0 | 0.125989 | 0.099816 |
| AABR0705[illegible] | 0.179952 | 0.203607 | 0.386379 | 0.165818 | 0.194579 | 0.315563 | 0 | 0 |
| Crnn | 0 | 0 | 0 | 0 | 0 | 0 | 0 | 0 |
| Rn50_X_0[illegible] | 1.324602 | 0.699405 | 1.516848 | 1.627424 | 1.623242 | 1.393694 | 1.580699 | 2.348121 |
| Stambp | 3.869999 | 4.002425 | 3.310769 | 4.290406 | 4.838413 | 3.499256 | 4.058984 | 4.07333 |
| MGC10934[illegible] | 0.482191 | 0.327346 | 0.103533 | 0 | 0.104277 | 0.169114 | 0.107891 | 0.341912 |

| | | | | | | | | |
|---|---|---|---|---|---|---|---|---|
| Gcgr | 6.692956 | 7.117749 | 7.092802 | 6.273172 | 5.249152 | 7.20326 | 5.238234 | 5.397629 |
| Spred3 | 1.397589 | 0.988317 | 1.172189 | 1.650024 | 1.369519 | 2.144463 | 1.465838 | 1.238753 |
| LOC685135 | 0 | 0 | 0 | 0.17483 | 0 | 0 | 0 | 0 |
| Lysmd3 | 4.122388 | 4.008666 | 3.554737 | 3.676575 | 3.830926 | 4.717739 | 4.093333 | 3.97671 |
| Wipf3 | 28.73904 | 30.70932 | 31.48506 | 24.77738 | 27.02025 | 26.8498 | 33.52533 | 34.78853 |
| Gulo | 0 | 0.025772 | 0 | 0 | 0 | 0 | 0 | 0 |
| AABR07063 | 0.978802 | 0 | 0.525403 | 0 | 0 | 0 | 1.642561 | 0.867561 |
| LOC100361 | 0.54125 | 0.306199 | 0.290533 | 0 | 0.731557 | 0.59321 | 0.454145 | 0.839539 |
| Optc | 0.085079 | 0.192525 | 0.36535 | 0.431182 | 0.32198 | 0.298388 | 0.333138 | 0.565571 |
| Btc | 0.148526 | 0.240071 | 0.20501 | 0.234618 | 0.275312 | 0.390682 | 0.261115 | 0.282098 |
| AABR07043 | 2.23786 | 2.430752 | 4.036176 | 2.392036 | 2.903719 | 2.982479 | 2.804052 | 3.173642 |
| Opalin | 0 | 0 | 0 | 0 | 0 | 0 | 0 | 0 |
| Sec22a | 5.02841 | 5.798124 | 5.948466 | 6.522289 | 6.025882 | 6.823988 | 5.804719 | 6.046662 |
| Tnfaip6 | 1.844141 | 1.651859 | 1.81482 | 1.911716 | 2.243298 | 1.953808 | 3.223661 | 2.588044 |
| AABR07054 | 0 | 0 | 0.135793 | 0.466214 | 0 | 0 | 0 | 0.112112 |
| Aatk | 1.432775 | 1.328415 | 1.367267 | 2.282924 | 1.839721 | 1.893111 | 1.458216 | 1.790262 |
| Scarna3 | 0 | 0.426256 | 0 | 0 | 0 | 0 | 0 | 0 |
| Cfap69 | 0.38284 | 0.2825 | 0.393134 | 0.690207 | 0.737928 | 0.934052 | 0.726256 | 0.826196 |
| Npy | 0.08627 | 0.097611 | 0 | 0 | 0 | 0.151283 | 0 | 0 |
| RGD156578 | 14.65759 | 14.41671 | 12.6344 | 13.28222 | 13.87613 | 12.02522 | 13.88069 | 12.10228 |
| AABR07021 | 0.20174 | 0.163042 | 0.03094 | 0.079669 | 0.436277 | 0.252694 | 0.12897 | 0.204357 |
| Taf15 | 5.275464 | 4.115427 | 4.17314 | 4.886717 | 4.653494 | 4.820291 | 4.845815 | 5.389597 |
| AABR07062 | 0 | 0 | 0 | 0 | 0 | 0 | 0 | 0 |
| Gja5 | 4.181115 | 4.230739 | 4.360967 | 3.179288 | 2.958852 | 3.546786 | 4.29735 | 4.127744 |
| LOC103690 | 2.087104 | 2.077375 | 1.684692 | 2.241305 | 2.205852 | 2.394103 | 2.721194 | 3.115628 |
| Slc7a6os | 6.830338 | 7.133728 | 5.941451 | 8.069086 | 6.93105 | 7.831568 | 7.014485 | 7.016509 |
| Med12l | 0.385641 | 0.826738 | 0.37043 | 0.504975 | 0.482828 | 0.498297 | 0.703924 | 0.701615 |
| Hrh1 | 0.120174 | 0.169964 | 0.032254 | 0.166104 | 0.194914 | 0.052684 | 0.100834 | 0.213033 |
| Fam57a | 3.121148 | 2.690615 | 3.031632 | 2.64776 | 2.839161 | 2.519433 | 3.768928 | 3.38119 |
| Zkscan2 | 0.008002 | 0 | 0 | 0 | 0 | 0 | 0.008952 | 0 |
| Ttll4 | 2.032067 | 2.282647 | 2.040302 | 2.00718 | 2.070784 | 1.691991 | 2.698624 | 2.138022 |
| Snx16 | 3.183996 | 2.62321 | 3.285477 | 4.130286 | 4.345293 | 3.279606 | 3.942534 | 3.123527 |
| Prelid3a | 1.163687 | 0.817236 | 0.732344 | 1.58995 | 1.301667 | 0.844404 | 1.750806 | 1.422667 |
| AABR07021 | 0 | 0 | 0 | 0.344665 | 0 | 0 | 0 | 0 |
| AABR07049 | 0.323246 | 1.828691 | 0.520539 | 1.340365 | 1.572848 | 0.566846 | 0.361634 | 1.719056 |
| AABR07043 | 0 | 0 | 0 | 0.376978 | 0 | 0 | 0 | 0 |
| Slc25a12 | 71.67984 | 72.55128 | 74.032 | 79.63779 | 77.86957 | 78.36975 | 71.0231 | 71.92911 |
| Slc27a4 | 4.131905 | 4.597783 | 4.234234 | 4.263645 | 4.57854 | 5.164818 | 5.978807 | 5.750739 |
| Rn50_X_06 | 13.72699 | 9.635247 | 1.091615 | 5.036128 | 1.786633 | 1.002986 | 1.279762 | 4.055637 |
| Hcfc1 | 2.864883 | 3.040105 | 3.340669 | 2.417683 | 2.45834 | 2.300507 | 2.871105 | 2.946388 |
| Frmpd1 | 0.213981 | 0.215208 | 0.344584 | 0.219083 | 0.269937 | 0.281428 | 0.186194 | 0.094831 |
| Ddx59 | 2.166351 | 1.803125 | 1.710871 | 1.789703 | 1.803951 | 2.008622 | 2.562905 | 2.074638 |
| C1ql1 | 0.042367 | 0 | 0 | 0.07808 | 0.045811 | 0.037148 | 0.047399 | 0.150209 |
| AABR07004 | 0 | 0.115722 | 0 | 0 | 0 | 0 | 0 | 0 |
| Maff | 12.8689 | 13.30902 | 13.43907 | 15.81092 | 14.06082 | 14.54787 | 25.14221 | 24.60615 |
| Mapkapk3 | 8.486412 | 8.390512 | 8.961699 | 8.514184 | 8.833198 | 8.362313 | 10.69209 | 9.349687 |
| Ccdc43 | 8.976359 | 10.26023 | 7.837517 | 9.350235 | 8.514477 | 7.789963 | 8.73248 | 8.973609 |
| #N/A | 0 | 0 | 0.730106 | 0.626664 | 0 | 0 | 0 | 0.602786 |
| Pdrg1 | 33.65125 | 34.04862 | 33.39132 | 41.08803 | 38.04919 | 40.17517 | 39.95749 | 39.52243 |
| AABR07008 | 0 | 0 | 0 | 0.27892 | 0 | 0.088467 | 0.22576 | 0.447153 |
| Rpl39 | 7.384938 | 3.79805 | 5.765965 | 2.47452 | 4.355578 | 4.414854 | 6.008684 | 3.272819 |
| AABR07058 | 0 | 0 | 0 | 0 | 0 | 0 | 0 | 0.081286 |
| Rpl12 | 51.82975 | 224.9664 | 229.6694 | 282.1903 | 283.2092 | 271.4909 | 71.48129 | 65.7407 |
| Brca1 | 0.198127 | 0.165692 | 0.212702 | 0.174629 | 0.288748 | 0.143506 | 0.269842 | 0.297774 |

| | | | | | | | | |
|---|---|---|---|---|---|---|---|---|
| Gpalpp1 | 6.326084 | 6.203311 | 5.433165 | 6.995085 | 8.018356 | 6.84095 | 7.155979 | 6.759698 |
| AC132752. | 4.322267 | 3.949972 | 4.99717 | 4.135983 | 3.055818 | 5.684651 | 3.719661 | 4.715126 |
| LOC500948 | 0.077925 | 0.176338 | 0.250974 | 0.071805 | 0.08426 | 0.273301 | 0.08718 | 0.069069 |
| AABR07064 | 5.735315 | 5.736864 | 5.889522 | 4.672129 | 6.201513 | 6.486332 | 6.04445 | 4.715126 |
| Pcdhga5 | 0.540078 | 0.366643 | 0.324692 | 0.358315 | 0.210232 | 0.303066 | 0.33836 | 0.287219 |
| Lrrc38 | 0 | 0 | 0 | 0 | 0 | 0 | 0 | 0 |
| Tmem45a1 | 0.232148 | 0.262664 | 0.854488 | 0.488949 | 0.753054 | 0.581564 | 0.259717 | 0.558503 |
| LOC365791 | 0 | 0 | 0 | 0.02019 | 0 | 0 | 0 | 0 |
| Hrnr | 0.019926 | 0 | 0 | 0 | 0 | 0 | 0 | 0 |
| AY172581. | 3.173692 | 1.795442 | 2.555371 | 3.655541 | 8.579169 | 6.261066 | 1.775293 | 0 |
| Chrnb3 | 0.047049 | 0.017745 | 0 | 0 | 0.016958 | 0 | 0 | 0 |
| Nsg1 | 2.646063 | 2.476433 | 2.069165 | 0.782646 | 1.624851 | 1.460785 | 1.388782 | 1.592513 |
| Dpys15 | 0 | 0 | 0.023207 | 0 | 0 | 0.009477 | 0.024184 | 0.00958 |
| Traf3 | 4.324555 | 4.438229 | 4.865892 | 5.274893 | 4.78099 | 4.533114 | 5.520418 | 6.081309 |
| Rab22a | 9.213198 | 10.43803 | 9.226346 | 8.523155 | 8.098306 | 8.535794 | 10.00855 | 10.37172 |
| AC108595. | 0.831205 | 0 | 0.892352 | 1.531846 | 1.79754 | 0 | 0.929915 | 0 |
| AABR07062 | 0.39079 | 0 | 1.258616 | 0 | 0.422556 | 0 | 0 | 0 |
| Tmem219 | 3.438048 | 2.831489 | 2.837272 | 3.59905 | 3.287596 | 3.506644 | 3.218363 | 3.482644 |
| Rnf169 | 1.481817 | 2.13128 | 2.237941 | 1.735724 | 1.412168 | 1.585536 | 1.966871 | 1.758632 |
| RGD130299 | 51.17729 | 49.76068 | 53.38244 | 44.54136 | 41.19948 | 42.0931 | 45.06513 | 43.72212 |
| Cdk2ap2 | 14.327 | 12.25659 | 13.13008 | 14.53574 | 11.92905 | 12.78077 | 16.19595 | 17.12337 |
| AC119116. | 0 | 0 | 0 | 0.223394 | 0 | 0 | 0 | 0 |
| LOC103694 | 1.387739 | 0 | 0.015202 | 0.809003 | 0.015312 | 0.893954 | 0 | 0.012551 |
| Mob3c | 1.057897 | 1.145663 | 0.924801 | 1.155847 | 1.111207 | 0.954067 | 1.386419 | 1.513662 |
| Myoz2 | 611.6261 | 599.6927 | 582.6679 | 591.3054 | 608.0255 | 599.3959 | 493.4462 | 494.2141 |
| Cfap74 | 0 | 0 | 0 | 0 | 0 | 0 | 0 | 0 |
| AABR07062 | 0 | 0 | 0 | 0 | 0 | 0 | 0 | 0.311507 |
| AC115255. | 0 | 0 | 0 | 0.031766 | 0 | 0 | 0 | 0 |
| Cd46 | 1.317934 | 0.646178 | 1.084746 | 0.931059 | 0.950042 | 0.731858 | 1.425298 | 1.246027 |
| Psmb9 | 28.78224 | 24.46018 | 27.02011 | 31.48722 | 32.69847 | 34.01897 | 33.0514 | 33.37799 |
| RGD130870 | 20.795 | 20.95959 | 19.73246 | 20.05843 | 17.88695 | 19.81306 | 17.09559 | 18.33581 |
| Cnp | 9.034885 | 8.008091 | 8.184739 | 13.33724 | 13.73114 | 14.20735 | 14.41066 | 14.24105 |
| Myo5a | 1.982632 | 2.168834 | 2.108307 | 2.363737 | 2.255553 | 2.405711 | 3.059059 | 3.014903 |
| Med23 | 4.209657 | 4.061109 | 4.305262 | 4.665048 | 5.102854 | 4.341818 | 5.477982 | 4.948787 |
| Tyk2 | 4.307736 | 4.405347 | 4.735799 | 4.503753 | 4.84824 | 5.002745 | 5.329038 | 5.516141 |
| RGD130662 | 0 | 0.046912 | 0.133535 | 0.038205 | 0 | 0 | 0.046385 | 0 |
| Fdx1l | 27.30363 | 26.97354 | 29.96844 | 24.8463 | 25.55724 | 26.08372 | 25.68303 | 29.8594 |
| Kif18a | 0.115489 | 0.224006 | 0.123985 | 0.197634 | 0.267592 | 0.188056 | 0.147661 | 0.17548 |
| RGD156189 | 9.349327 | 8.495545 | 8.494264 | 7.365187 | 7.018887 | 7.659493 | 10.27896 | 9.832493 |
| Mettl8 | 3.360104 | 3.275396 | 2.830332 | 3.048569 | 3.52144 | 2.810166 | 3.990466 | 4.536068 |
| AABR07061 | 3.103166 | 1.170362 | 1.665723 | 2.144584 | 1.537895 | 1.700536 | 2.459109 | 1.375245 |
| Iglon5 | 0.155388 | 0.11721 | 0 | 0.190913 | 0.112013 | 0 | 0.173842 | 0.229548 |
| Rnf7 | 35.10518 | 36.88947 | 34.15164 | 34.11527 | 32.90958 | 31.43826 | 31.53124 | 30.5242 |
| Grsf1 | 71.08075 | 72.03065 | 69.15237 | 67.86793 | 64.23778 | 62.1849 | 70.22635 | 69.0519 |
| Pgf | 1.67732 | 1.48029 | 1.836724 | 1.792878 | 1.088197 | 1.411848 | 1.200967 | 1.665095 |
| Sugp1 | 10.92218 | 11.10216 | 11.6715 | 10.97085 | 11.89182 | 11.10264 | 11.57019 | 11.44713 |
| Hnrnpd | 35.39192 | 37.50295 | 34.10293 | 31.19305 | 31.74608 | 30.55339 | 31.66157 | 32.90539 |
| Actr10 | 43.20096 | 42.94235 | 38.24581 | 36.57295 | 36.56288 | 34.67078 | 39.48367 | 36.88267 |
| Tm2d3 | 7.103904 | 6.812936 | 7.335962 | 7.917504 | 7.535037 | 7.296488 | 7.644769 | 9.474798 |
| AABR07012 | 0 | 0 | 0.187394 | 0.321688 | 0.377483 | 0 | 0 | 0.154715 |
| Ttc39b | 5.322695 | 4.561066 | 5.98736 | 5.337417 | 5.226368 | 5.6621 | 5.298015 | 5.290145 |
| LOC690468 | 7.604292 | 7.626183 | 9.276924 | 5.255292 | 7.288046 | 6.970516 | 4.64037 | 14.85877 |
| Nod1 | 12.223 | 11.99549 | 12.79067 | 11.78467 | 11.82534 | 12.17242 | 13.51622 | 14.15244 |
| AABR07034 | 0.01022 | 0 | 0.010972 | 0 | 0.01105 | 0 | 0.011433 | 0 |

| | | | | | | | | |
|---|---|---|---|---|---|---|---|---|
| Tmem208 | 15.35542 | 12.39935 | 12.53989 | 17.83794 | 15.46831 | 15.99527 | 16.59165 | 15.70416 |
| Naga | 8.28653 | 9.067956 | 8.763341 | 8.524645 | 9.120584 | 9.889941 | 10.12848 | 9.383757 |
| Tinag | 0 | 0.043185 | 0.040975 | 0.03517 | 0 | 0 | 0.0427 | 0.03383 |
| Gpr141 | 0.351922 | 0 | 0.151124 | 0.129713 | 0.304422 | 0.185139 | 0.472457 | 0.436696 |
| AABR07058 | 0 | 0 | 0 | 0.502637 | 0 | 0 | 0 | 0 |
| Ppfia3 | 0.36034 | 0.443682 | 0.43236 | 0.263678 | 0.355252 | 0.353117 | 0.355705 | 0.385144 |
| Mamstr | 4.796878 | 4.458256 | 6.069354 | 4.577996 | 4.013591 | 3.905485 | 4.088788 | 4.555405 |
| Pot1 | 5.946991 | 5.31492 | 4.918779 | 5.543886 | 5.604703 | 4.605651 | 5.151723 | 4.532748 |
| Tectb | 0.038098 | 0.086213 | 0.02045 | 0 | 0.041195 | 0.016702 | 0.063934 | 0.033768 |
| Il1rap | 3.278227 | 2.812173 | 2.507275 | 2.843065 | 2.826492 | 2.310754 | 3.307972 | 3.703286 |
| AC104314. | 0.732391 | 0.414333 | 0.393134 | 1.349738 | 0.791923 | 1.605402 | 0 | 0.324577 |
| Clic4 | 106.2747 | 102.4802 | 105.3238 | 105.4126 | 98.2798 | 104.0834 | 136.5622 | 135.0606 |
| Zfp251 | 3.540194 | 3.40994 | 3.235476 | 3.165134 | 3.016832 | 2.977114 | 3.518908 | 3.896067 |
| Otog | 0 | 0 | 0 | 0 | 0 | 0 | 0.011674 | 0 |
| Wdr5 | 6.072147 | 5.205389 | 5.756188 | 6.062813 | 4.828276 | 5.11644 | 6.017417 | 5.412029 |
| Mfsd2b | 0.035263 | 0 | 0.037857 | 0 | 0.03813 | 0.061838 | 0.078902 | 0 |
| AABR07028 | 0.092164 | 0.01896 | 0.089949 | 0.023162 | 0.045298 | 0.022039 | 0.093735 | 0.066837 |
| Acbd7 | 0 | 0.241342 | 0.114497 | 0.049138 | 0 | 0 | 0.059659 | 0.047265 |
| Cbs | 0.81789 | 0.97286 | 0.810514 | 0.8696 | 1.065782 | 0.717127 | 1.337335 | 1.319756 |
| 5S_rRNA | 0 | 0 | 0.472422 | 0 | 0.475819 | 0 | 0 | 0 |
| Htr4 | 0 | 0 | 0 | 0.041137 | 0 | 0.039143 | 0.149833 | 0.158276 |
| Map1a | 12.3687 | 12.85583 | 13.13028 | 16.85723 | 16.45755 | 16.73236 | 20.42791 | 20.39108 |
| Rnft2 | 0.237232 | 0.339053 | 0.227875 | 0.437201 | 0.459028 | 0.284639 | 0.32128 | 0.387341 |
| #N/A | 0.18246 | 0 | 0 | 0 | 0 | 0.159981 | 0 | 0 |
| Cdk5rap3 | 18.08709 | 18.9329 | 18.95255 | 19.95993 | 20.66985 | 22.31624 | 20.81058 | 20.11136 |
| Plpp4 | 0.042163 | 0.09541 | 0.135793 | 0.077702 | 0.04559 | 0.036968 | 0.04717 | 0 |
| Plxna3 | 1.030126 | 1.173577 | 1.113533 | 1.217621 | 1.351996 | 1.252043 | 1.367055 | 1.353835 |
| Strip2 | 2.653538 | 2.462868 | 2.448138 | 2.846285 | 2.801985 | 2.908281 | 2.365652 | 2.443836 |
| Cdc42bpb | 13.93344 | 14.33733 | 14.91074 | 12.55253 | 11.58777 | 12.67654 | 16.48284 | 15.9257 |
| AABR07043 | 12.32139 | 11.61756 | 6.062743 | 8.042189 | 3.885859 | 4.051278 | 13.78463 | 13.65133 |
| Galm | 5.13702 | 5.94032 | 5.029023 | 4.504182 | 4.991786 | 4.56367 | 4.000163 | 5.536044 |
| Prr14l | 2.025694 | 1.804442 | 1.89822 | 1.633843 | 1.735146 | 1.750074 | 1.784192 | 2.247634 |
| Fancl | 5.037012 | 4.265396 | 5.169486 | 4.90413 | 4.898378 | 3.972034 | 4.820033 | 4.885739 |
| AABR07052 | 0.498723 | 0.564282 | 2.141644 | 1.148884 | 1.617786 | 0.655921 | 0.557949 | 0.221022 |
| Slain1 | 0.116758 | 0.198159 | 0.156684 | 0.242073 | 0.157811 | 0.15356 | 0.032656 | 0.077616 |
| AABR07030 | 23.73922 | 19.2232 | 20.98811 | 22.94705 | 17.86755 | 24.89585 | 22.39236 | 20.2161 |
| Msl3 | 20.70824 | 21.29075 | 17.82038 | 18.60854 | 19.18551 | 18.85298 | 16.74221 | 18.64444 |
| Gpr21 | 0.089706 | 0.025375 | 0.024076 | 0.020665 | 0.121247 | 0.019664 | 0.05018 | 0.059633 |
| Mtpap | 5.529685 | 5.792309 | 4.761762 | 5.257431 | 5.19744 | 5.328136 | 4.45943 | 5.386162 |
| AABR07073 | 126.6981 | 133.6444 | 141.9666 | 128.1329 | 122.6821 | 131.9827 | 148.1073 | 154.3239 |
| LOC100361 | 1.956198 | 2.213346 | 1.777011 | 1.525243 | 1.301667 | 2.242949 | 1.01008 | 1.333751 |
| Mmrn2 | 22.58793 | 22.37187 | 23.9516 | 19.68124 | 18.6074 | 20.08324 | 23.2446 | 23.78129 |
| AABR07005 | 0 | 0.023456 | 0 | 0.057308 | 0.044832 | 0.018177 | 0 | 0.055124 |
| AABR07008 | 0 | 0 | 0 | 0 | 0 | 0 | 0 | 0 |
| Cebpb | 58.44277 | 78.17888 | 106.8789 | 61.69903 | 53.70215 | 54.53167 | 114.7353 | 116.5018 |
| Mcpt1l3 | 0 | 0 | 0 | 0.064856 | 0 | 0.185139 | 0 | 0 |
| AC132740. | 0 | 0 | 0 | 0 | 0.038207 | 0 | 0 | 0 |
| Polr2l | 7.457685 | 1.528627 | 3.190917 | 3.286591 | 3.213868 | 4.454037 | 1.813767 | 2.059674 |
| Tomm40l | 18.89835 | 20.72322 | 20.60141 | 15.87981 | 17.05877 | 18.46799 | 17.27735 | 16.89813 |
| Taf12 | 7.369019 | 7.971206 | 8.345789 | 8.307499 | 8.785245 | 8.567548 | 7.54957 | 8.038803 |
| Chic2 | 11.30682 | 11.32952 | 13.01299 | 11.52248 | 12.58854 | 11.72017 | 13.39997 | 14.18339 |
| Mir92b | 0 | 0 | 0 | 0.581363 | 0 | 0 | 0 | 0 |
| Znhit6 | 27.77641 | 25.43063 | 24.70574 | 26.61496 | 24.5207 | 24.26614 | 24.0944 | 25.24403 |
| AABR07007 | 1.163687 | 0 | 0.832862 | 1.787153 | 0.838852 | 0.340107 | 1.301881 | 0.343811 |

| | | | | | | | | |
|---|---|---|---|---|---|---|---|---|
| Gpr149 | 0 | 0.031029 | 0.029441 | 0.012635 | 0.029653 | 0 | 0.030681 | 0 |
| Amotl2 | 19.67624 | 20.91801 | 22.28363 | 17.69945 | 16.94002 | 17.68371 | 21.71742 | 21.21746 |
| #N/A | 0 | 0.398987 | 0.757147 | 0.162468 | 0.190648 | 0.618377 | 0 | 0.468834 |
| AABR07002 | 0 | 0 | 0 | 0 | 0 | 0 | 0 | 0 |
| PVR | 3.46041 | 4.517643 | 3.429204 | 3.812979 | 4.47433 | 3.988874 | 4.791814 | 4.783012 |
| Zfp866 | 2.791504 | 3.044294 | 1.408162 | 3.161092 | 3.018413 | 2.771973 | 2.709118 | 2.891592 |
| Il21 | 0.237487 | 0 | 0 | 0.109418 | 0 | 0.104114 | 0 | 0.210497 |
| Kat8 | 12.54419 | 14.31111 | 12.01211 | 13.92841 | 14.35289 | 12.85727 | 14.03388 | 13.70568 |
| Fbxl16 | 1.054591 | 0.617183 | 0.663687 | 0.335091 | 0.589818 | 1.084092 | 0.895044 | 0.709111 |
| Zfp507 | 3.504634 | 2.895842 | 3.075528 | 2.679985 | 1.981238 | 2.142081 | 3.026034 | 3.222335 |
| P2ry1 | 6.502527 | 6.668138 | 6.963205 | 3.898477 | 4.681459 | 4.214722 | 5.285696 | 5.530054 |
| Ppp1r12a | 17.7429 | 17.41431 | 17.68863 | 21.04262 | 21.95383 | 19.97396 | 23.57087 | 22.83106 |
| Fam115e | 0.016795 | 0.019002 | 0.01803 | 0.015476 | 0 | 0.014726 | 0.075157 | 0.07443 |
| Nhlrc2 | 6.182964 | 6.619339 | 5.664919 | 5.972184 | 5.904122 | 5.421228 | 6.942893 | 6.486848 |
| Traf3ip1 | 2.043212 | 2.103996 | 1.774532 | 2.051975 | 2.035531 | 1.79149 | 2.234487 | 2.462147 |
| Ctrb1 | 0.118878 | 0.134505 | 0.127624 | 0.273854 | 0.192812 | 0.104233 | 0.132996 | 0.210736 |
| Etaa1 | 1.285053 | 1.254055 | 0.827752 | 1.40615 | 0.990026 | 1.281662 | 1.222011 | 1.324095 |
| Tlr4 | 2.053258 | 1.879813 | 1.413447 | 1.949768 | 1.762568 | 2.00644 | 2.191884 | 2.111645 |
| Gpr55 | 0.054548 | 0.123437 | 0.117121 | 0.100527 | 0 | 0 | 0 | 0.096697 |
| Ehmt2 | 15.70574 | 17.02416 | 18.66715 | 16.79125 | 16.56038 | 17.33446 | 21.30488 | 21.55514 |
| AC141152. | 0 | 0 | 0 | 0 | 1.155561 | 0.624687 | 0 | 0 |
| Ecscr | 7.656532 | 6.46359 | 8.645672 | 6.835861 | 5.87673 | 6.887173 | 7.278701 | 8.755466 |
| Clasp2 | 13.00492 | 12.96301 | 11.30486 | 11.57062 | 12.6305 | 12.72426 | 12.5327 | 11.76159 |
| AABR07017 | 0 | 0 | 0 | 0.105471 | 0 | 0 | 0 | 0 |
| Rab25 | 0.048397 | 0.164278 | 0.103915 | 0 | 0.156994 | 0 | 0.054145 | 0.042897 |
| Mx2 | 9.410502 | 9.705921 | 9.43842 | 15.27819 | 15.22855 | 15.37967 | 26.95277 | 29.0138 |
| Sgtb | 1.806943 | 1.443156 | 1.407356 | 1.142666 | 1.76227 | 1.739656 | 1.466599 | 1.852812 |
| Rims4 | 0.16014 | 0.060397 | 0.229228 | 0.049188 | 0.115438 | 0.093608 | 0.119439 | 0.094627 |
| AC136815. | 0 | 0 | 0 | 0 | 0 | 0 | 0 | 0 |
| Larp1 | 16.14808 | 16.68608 | 17.74286 | 12.89078 | 13.55876 | 13.36403 | 18.2501 | 18.11013 |
| LOC498339 | 0 | 0 | 0 | 0 | 0 | 0 | 0 | 0 |
| Zfhx2 | 0.324944 | 0.32995 | 0.447241 | 0.322455 | 0.31532 | 0.394492 | 0.503353 | 0.398788 |
| AC096239. | 0 | 0 | 0 | 0 | 0 | 1.133691 | 0 | 0 |
| Itgad | 2.215261 | 2.183049 | 2.178761 | 2.673413 | 2.178978 | 2.506249 | 3.085928 | 3.420284 |
| Tdrd1 | 1.346194 | 1.475926 | 1.12033 | 1.028913 | 1.049401 | 1.180344 | 0.957342 | 1.230197 |
| AABR07010 | 0.24413 | 0.414333 | 0.655223 | 0.224956 | 0.263974 | 0.428107 | 0.136561 | 0.216385 |
| Cyp26c1 | 0.033633 | 0.038054 | 0.036107 | 0 | 0.109099 | 0 | 0.11288 | 0 |
| AABR07062 | 0.048894 | 0 | 0 | 0 | 0.052869 | 0.085741 | 0.054701 | 0.043338 |
| Fgd5 | 10.35668 | 10.52593 | 11.22892 | 9.006515 | 9.503468 | 8.600047 | 12.93789 | 12.43693 |
| Tmem176a | 12.46115 | 14.64781 | 13.22168 | 19.25658 | 20.91887 | 21.25671 | 16.9787 | 18.60878 |
| Bnc2 | 0.104107 | 0.100965 | 0.111766 | 0.602993 | 0.450278 | 0.495527 | 0.598991 | 0.883207 |
| Pax8 | 0.078588 | 0.148198 | 0.028123 | 0.16897 | 0.283254 | 0.160781 | 0.117228 | 0.116094 |
| Tra2a | 13.84581 | 13.84724 | 13.71396 | 15.22689 | 14.5139 | 14.1922 | 18.23475 | 19.24565 |
| Tdrd9 | 0.063016 | 0.071299 | 0.02706 | 0.023227 | 0.05451 | 0.022101 | 0.070499 | 0 |
| Serinc5 | 10.76737 | 10.62942 | 10.51722 | 8.295202 | 8.493377 | 8.237093 | 8.42566 | 8.535404 |
| LOC100364 | 3.491061 | 5.266629 | 7.079324 | 3.216876 | 5.871964 | 9.182897 | 4.773565 | 5.844791 |
| Mcemp1 | 0.279471 | 0.237156 | 0.112511 | 0.321902 | 0.151094 | 0.398191 | 0.31266 | 0.216746 |
| Cep76 | 1.607981 | 1.468635 | 1.247906 | 2.249314 | 1.654894 | 2.649893 | 2.232416 | 2.541379 |
| Hs6st1 | 9.178854 | 9.569831 | 11.31586 | 12.05959 | 11.25868 | 11.53129 | 14.26534 | 13.87182 |
| Ube3b | 16.05034 | 15.2416 | 15.33058 | 12.78568 | 12.68811 | 12.55035 | 13.82568 | 15.46652 |
| LOC100912 | 0.33143 | 0.224998 | 0.569298 | 0.24432 | 0.143348 | 0.290598 | 0.222473 | 0.352515 |
| Cdk2ap1 | 59.75524 | 61.71428 | 68.96584 | 59.78508 | 55.60645 | 55.83891 | 62.74198 | 60.08144 |
| Plcxd2 | 0.066356 | 0.075079 | 0.047492 | 0.010191 | 0.023917 | 0.048484 | 0.061863 | 0.068617 |
| Ankrd40 | 23.96879 | 24.35154 | 23.1555 | 22.58566 | 21.83198 | 21.31453 | 26.53806 | 25.3482 |

| | | | | | | | | |
|---|---|---|---|---|---|---|---|---|
| Hpgds | 1.249996 | 1.010223 | 1.150244 | 0.452502 | 0.482715 | 0.782856 | 0.599332 | 1.345348 |
| Acsl3 | 3.041455 | 2.671788 | 2.778459 | 2.924432 | 3.268255 | 3.080842 | 3.318107 | 3.884621 |
| Khdrbs2 | 0 | 0 | 0 | 0 | 0 | 0.02351 | 0 | 0 |
| Kremen2 | 0.027049 | 0 | 0 | 0 | 0 | 0 | 0 | 0 |
| Sec61g | 123.9997 | 122.6453 | 112.3094 | 98.57582 | 103.1476 | 85.09208 | 102.7546 | 110.9548 |
| Otogl | 0 | 0.009264 | 0.00879 | 0.007544 | 0 | 0 | 0 | 0 |
| Ush2a | 0.006692 | 0.01893 | 0.028738 | 0.021583 | 0.021708 | 0.017603 | 0.007487 | 0.026692 |
| Trim33 | 4.008066 | 4.117932 | 4.566695 | 4.160241 | 3.902138 | 4.066327 | 5.480501 | 4.736731 |
| Klhl31 | 47.69494 | 47.79555 | 47.511 | 40.02052 | 39.90023 | 39.59249 | 43.24966 | 45.11226 |
| LOC685406 | 0.31451 | 0 | 0.168823 | 0.144904 | 0.510113 | 0.137881 | 0.87965 | 0 |
| #N/A | 0 | 0 | 1.59108 | 0 | 0 | 0 | 0 | 0 |
| Dok3 | 0.72016 | 1.358045 | 1.69354 | 2.369997 | 2.150691 | 2.615953 | 2.800708 | 3.525923 |
| AABR07016 | 0 | 0 | 0.453372 | 0 | 0.913266 | 0 | 0.944914 | 0.374311 |
| Etv6 | 3.09 | 3.279611 | 3.346669 | 3.124485 | 3.93253 | 3.740292 | 4.007619 | 3.92645 |
| AABR07021 | 0 | 0 | 0 | 0 | 0 | 0.051706 | 0.131947 | 0 |
| Slc23a3 | 0.148275 | 0.167767 | 0.265305 | 0.045543 | 0.026721 | 0.043336 | 0.165884 | 0.043808 |
| Fam60a | 0.075893 | 0 | 0 | 0.069932 | 0.082062 | 0 | 0 | 0.067267 |
| Grap2 | 0.585391 | 1.023621 | 0.74272 | 0.735566 | 1.323494 | 0.839899 | 1.131208 | 0.6132 |
| LOC100909 | 0 | 0 | 0 | 0 | 0 | 0 | 0 | 0 |
| Klk1c3 | 0 | 0 | 0 | 0 | 0 | 0 | 0.083099 | 0.065836 |
| Prss30 | 0 | 0.029684 | 0 | 0 | 0 | 0.023003 | 0.029351 | 0 |
| Tbkbp1 | 8.534996 | 8.779042 | 9.196863 | 9.294601 | 7.72203 | 8.455374 | 12.1173 | 11.46679 |
| Cnn3 | 24.34094 | 24.11666 | 23.39128 | 30.91595 | 29.25022 | 30.20204 | 31.38254 | 32.1403 |
| Tubgcp4 | 3.632571 | 3.912007 | 3.218507 | 4.113514 | 3.099686 | 3.492034 | 4.431185 | 4.36409 |
| Dlx4 | 0 | 0 | 0 | 0.029621 | 0 | 0 | 0 | 0 |
| Pdss1 | 7.030442 | 5.617434 | 6.108132 | 6.277917 | 5.642659 | 5.719451 | 5.716566 | 5.107203 |
| Tmpo | 10.78599 | 10.80149 | 11.61113 | 9.544576 | 10.75333 | 10.14284 | 14.79061 | 13.26129 |
| Sstr4 | 0.449493 | 0.445008 | 0.723839 | 0.284756 | 0.48603 | 0.44338 | 0.628591 | 0.597612 |
| Rn50_5_08 | 0.104419 | 0.177217 | 0.1121 | 0.096218 | 0.169359 | 0.045777 | 0.058409 | 0.092551 |
| Xdh | 31.56709 | 32.73925 | 32.57722 | 37.99579 | 36.99036 | 40.51789 | 56.10874 | 57.73513 |
| LOC100174 | 13.16629 | 12.30134 | 12.90341 | 13.92448 | 13.26585 | 12.68114 | 13.1676 | 13.70333 |
| AABR07053 | 0.872765 | 2.962479 | 4.684847 | 0.804219 | 0 | 4.591449 | 2.929233 | 3.094301 |
| Dpep2 | 0.834261 | 1.63372 | 1.309001 | 1.626178 | 2.116405 | 1.828702 | 1.794873 | 1.877058 |
| Pzp | 0.022246 | 0.012585 | 0.011941 | 0.061495 | 0 | 0.029257 | 0 | 0.039435 |
| Dennd2d | 0.435023 | 0.246104 | 0.467025 | 0.200428 | 0.676177 | 0.429107 | 0.638774 | 0.506077 |
| AABR07055 | 0 | 0.156745 | 0 | 0.127654 | 0 | 0.242934 | 0 | 0 |
| Rn50_2_22 | 0 | 0 | 0 | 0 | 0 | 0 | 1.38389 | 0 |
| Rn50_X_06 | 0 | 0 | 0 | 0 | 0 | 0 | 0 | 0.076465 |
| Rcn2 | 10.56163 | 11.53792 | 9.970136 | 13.35171 | 14.45801 | 12.72741 | 13.99862 | 14.11112 |
| Itfg2 | 4.084162 | 4.764325 | 4.588544 | 5.017859 | 4.587314 | 3.775314 | 5.312999 | 5.275653 |
| Huwe1 | 16.99473 | 17.63361 | 17.8609 | 15.99605 | 15.63831 | 15.31918 | 19.48376 | 20.05535 |
| Pak1 | 0.546067 | 0.298272 | 0.505377 | 1.318676 | 0.956943 | 1.271275 | 1.242897 | 1.134911 |
| AABR07053 | 5.838093 | 7.206029 | 6.267565 | 7.39012 | 7.396635 | 6.721715 | 5.607766 | 6.429038 |
| LOC100909 | 1.09698 | 1.250803 | 1.049868 | 0.987317 | 0.94708 | 0.827624 | 0.989413 | 1.055218 |
| AABR07044 | 0 | 0 | 0 | 0 | 0 | 0 | 0 | 0 |
| RGD130854 | 0 | 0 | 0 | 0 | 0 | 0 | 0 | 0 |
| Fam20a | 1.315036 | 1.154789 | 1.854272 | 1.790502 | 1.697826 | 1.669305 | 2.173869 | 2.418148 |
| Ibtk | 7.248281 | 7.51401 | 6.574737 | 6.202784 | 7.041141 | 6.581869 | 6.750318 | 7.077269 |
| LOC100158 | 0.478446 | 0.541339 | 0.744781 | 0.529043 | 0.801872 | 0.545353 | 0.535264 | 0.636106 |
| Phtf1 | 2.932191 | 3.0837 | 2.502172 | 3.637171 | 3.902198 | 3.757539 | 3.995365 | 4.598136 |
| Amhr2 | 3.091515 | 3.252437 | 3.027804 | 2.423902 | 3.166873 | 2.496645 | 2.336105 | 2.956492 |
| Lurap1l | 2.147642 | 1.654438 | 2.011295 | 2.400025 | 2.371624 | 1.52247 | 1.891477 | 2.227573 |
| Pkn2 | 10.29961 | 9.660728 | 10.00226 | 9.702373 | 10.1984 | 9.970706 | 10.12345 | 11.39152 |
| AC105648. | 2.909218 | 2.468732 | 3.123231 | 4.021095 | 2.359271 | 5.101609 | 1.627352 | 9.025045 |

| | | | | | | | | |
|---|---|---|---|---|---|---|---|---|
| Angptl4 | 1.580963 | 1.349434 | 2.054583 | 1.380121 | 1.889416 | 1.678019 | 7.757503 | 8.62897 |
| Trim68 | 4.659898 | 5.155937 | 4.532831 | 5.812202 | 5.45625 | 6.004549 | 4.838851 | 5.45382 |
| Tmem167b | 16.34402 | 15.73242 | 14.66561 | 13.78661 | 18.90348 | 17.53876 | 20.10437 | 19.53158 |
| Fnbp4 | 9.960009 | 9.50553 | 9.952214 | 10.56332 | 11.05302 | 10.13602 | 10.88045 | 10.90668 |
| Stub1 | 59.01748 | 57.19705 | 59.55057 | 53.7508 | 55.66355 | 51.88796 | 59.03566 | 57.49112 |
| Bin3 | 9.323453 | 8.526692 | 9.023948 | 12.90905 | 10.8126 | 10.63149 | 11.99797 | 11.51799 |
| AC141169. | 0 | 0 | 0 | 0 | 0 | 0.371777 | 0 | 0 |
| Nsun4 | 0.272208 | 0.461985 | 0.694051 | 0.43895 | 0.367918 | 0.119336 | 0.190334 | 0.120636 |
| Ctdsp2 | 32.11776 | 30.01979 | 26.53497 | 25.34898 | 25.74437 | 27.30381 | 34.29156 | 31.25244 |
| Robo1 | 1.067821 | 0.918223 | 0.940026 | 1.042992 | 1.016065 | 1.151607 | 1.64859 | 1.457552 |
| Efna3 | 0 | 0 | 0 | 0.279728 | 0.164123 | 0.199628 | 0.339621 | 0.067267 |
| Scnn1g | 0 | 0 | 0 | 0.015826 | 0.018571 | 0.015059 | 0.038429 | 0 |
| RGD156453 | 0.021184 | 0 | 0 | 0 | 0 | 0.018574 | 0 | 0 |
| LOC103694 | 3.692469 | 14.43259 | 15.13566 | 8.660819 | 9.074121 | 17.95374 | 7.886397 | 2.082703 |
| Carnmt1 | 11.85934 | 12.45984 | 10.62358 | 12.0278 | 11.86575 | 9.655546 | 11.71693 | 11.02345 |
| AABR07027 | 0 | 0 | 0 | 0 | 0 | 0 | 0 | 0 |
| Phf20 | 4.90337 | 4.727464 | 4.8563 | 4.390988 | 4.648535 | 4.480939 | 6.412829 | 5.662173 |
| Tmem41b | 11.70846 | 10.98518 | 9.8507 | 12.44714 | 13.35686 | 13.51916 | 9.942243 | 10.6141 |
| Cyfip1 | 12.7929 | 12.87247 | 12.75583 | 13.86441 | 13.83277 | 13.34359 | 15.75849 | 16.35913 |
| RGD156233 | 8.62165 | 9.595073 | 9.483496 | 9.116652 | 8.099847 | 7.745359 | 11.46394 | 11.33742 |
| Fbxo6 | 63.03118 | 65.48336 | 70.38123 | 58.63967 | 55.25614 | 56.86128 | 58.24537 | 58.60057 |
| Pip5kl1 | 0.057799 | 0.392381 | 0.124102 | 0.266298 | 0 | 0.202713 | 0.129326 | 0.307381 |
| Nfs1 | 0.96491 | 6.668528 | 1.06389 | 1.513918 | 3.947785 | 0.617376 | 0.758566 | 4.299353 |
| Cbwd1 | 2.028762 | 2.592507 | 2.126758 | 2.353275 | 2.580789 | 1.925311 | 2.483306 | 2.496314 |
| AABR07027 | 0.523659 | 0 | 1.124363 | 0 | 1.13245 | 0 | 0 | 0.464145 |
| AABR07063 | 0 | 1.107469 | 0 | 0.901928 | 0.529182 | 0.429107 | 2.190081 | 0.867561 |
| Cd163 | 6.348189 | 6.476431 | 6.088043 | 7.746445 | 7.768902 | 7.173047 | 12.92637 | 13.78428 |
| Klhl24 | 40.78723 | 41.4747 | 38.35328 | 40.26456 | 41.4288 | 38.3641 | 42.63662 | 46.13087 |
| AABR07026 | 0 | 0 | 0 | 0 | 0 | 0 | 0 | 0.195567 |
| Tulp2 | 0.566799 | 0.391909 | 0.473274 | 0.638346 | 0.578823 | 0.717845 | 0.281826 | 0.390742 |
| Rasa2 | 1.41262 | 1.639035 | 1.333008 | 1.757674 | 1.556633 | 1.759266 | 1.489782 | 1.818301 |
| Mir3074 | 20.58831 | 16.20501 | 17.77839 | 32.58118 | 28.55323 | 28.6475 | 20.52967 | 26.18255 |
| RGD135915 | 7.342106 | 5.450258 | 6.42255 | 6.407501 | 6.25872 | 3.882976 | 4.824108 | 5.027092 |
| Tmem19 | 3.500705 | 2.782439 | 2.57796 | 3.598991 | 3.441147 | 3.551397 | 4.401942 | 3.102849 |
| Tap2 | 0.712315 | 0.561723 | 1.946548 | 1.84977 | 0.653516 | 0.700262 | 0.72446 | 1.128795 |
| Jak2 | 4.987231 | 4.553852 | 4.285637 | 5.200213 | 4.884106 | 5.255041 | 5.824207 | 6.940851 |
| RGD130902 | 0 | 0 | 0 | 0 | 0 | 0.156705 | 0 | 0 |
| LOC102546 | 28.49322 | 27.59171 | 21.49518 | 35.95332 | 29.97662 | 34.43586 | 30.15387 | 35.49345 |
| AABR07042 | 6.037174 | 6.608035 | 7.115331 | 6.590967 | 5.179754 | 4.890641 | 6.38705 | 6.04901 |
| Slc29a2 | 0.343759 | 1.080408 | 0.615078 | 0.211173 | 0.536902 | 0.401877 | 0.299119 | 0.101563 |
| Tas2r135 | 0 | 0 | 0.058197 | 0 | 0 | 0 | 0 | 0 |
| Rn50_19_0 | 1.864883 | 2.025627 | 1.521574 | 2.955676 | 2.581083 | 3.401073 | 2.253256 | 2.380232 |
| Grid1 | 0.969739 | 0.688448 | 0.796118 | 0.210253 | 0.185041 | 0.35011 | 0.212726 | 0.185388 |
| Oas1i | 2.006723 | 0.792878 | 0.444548 | 2.436138 | 0.929932 | 2.345996 | 0.534532 | 1.609262 |
| Olr1075 | 0 | 0 | 0 | 0 | 0 | 0.047828 | 0 | 0 |
| Txnrd3 | 6.125328 | 5.952595 | 5.163922 | 6.180972 | 6.826395 | 5.057677 | 5.675587 | 6.378457 |
| Mir27b | 7.557968 | 4.886563 | 5.216118 | 6.96437 | 8.756059 | 9.940249 | 7.851553 | 4.785002 |
| AC128915. | 0.271326 | 0.460489 | 1.0195 | 1.000065 | 0.440071 | 0.713697 | 0.455321 | 0.360735 |
| Zcchc7 | 5.617197 | 4.60335 | 4.677802 | 6.216068 | 5.449385 | 5.431488 | 7.165242 | 6.235133 |
| Ifna4 | 0 | 0 | 0 | 0 | 0 | 0 | 0.20556 | 0 |
| Mutyh | 2.699922 | 3.125877 | 3.336689 | 4.397168 | 4.141455 | 4.156527 | 3.231289 | 3.895703 |
| Hdx | 0.523659 | 0.325873 | 0.252982 | 0.241266 | 0.198179 | 0.344359 | 0.234339 | 0.603389 |
| AABR07061 | 0 | 0 | 0 | 0 | 0 | 0 | 0 | 0 |
| Nrxn2 | 2.338236 | 2.824967 | 3.162626 | 2.909309 | 3.24251 | 2.860968 | 3.310536 | 3.817137 |

| | | | | | | | | |
|---|---|---|---|---|---|---|---|---|
| Mcrs1 | 18.77164 | 19.63448 | 21.13779 | 17.91234 | 16.68906 | 17.14173 | 18.41855 | 19.59615 |
| Atpaf2 | 18.98354 | 16.57529 | 17.41917 | 19.6379 | 18.55143 | 19.53407 | 16.34921 | 16.41334 |
| Selplg | 10.38218 | 8.611313 | 12.61134 | 16.27495 | 18.87417 | 18.09742 | 22.44357 | 21.4108 |
| AABR07032 | 0 | 0 | 0 | 0 | 0 | 0 | 0 | 0.2813 |
| Atp6v0a1 | 16.78075 | 18.58519 | 17.29573 | 16.80686 | 16.28341 | 16.70665 | 18.47953 | 20.21972 |
| Praf2 | 7.158637 | 7.212027 | 4.842763 | 9.036168 | 9.225016 | 11.17768 | 12.39713 | 10.86482 |
| Apln | 5.245107 | 5.95386 | 6.526791 | 2.840266 | 2.835729 | 2.881787 | 2.514854 | 2.988642 |
| Magea11 | 0.03147 | 0.071213 | 0.033785 | 0 | 0.068056 | 0.055186 | 0 | 0.055787 |
| Mapre3 | 21.56073 | 22.52599 | 23.95572 | 19.73385 | 19.14574 | 20.83557 | 23.99151 | 23.19442 |
| Nrgn | 2.364667 | 3.409116 | 2.989021 | 2.53039 | 2.96928 | 2.842485 | 1.792102 | 1.994506 |
| Pan3 | 6.814743 | 6.946667 | 6.840405 | 6.707225 | 6.593032 | 7.251603 | 7.836466 | 9.088419 |
| Vps45 | 11.80594 | 11.39872 | 12.22379 | 10.70949 | 12.45355 | 9.799384 | 11.50561 | 10.71999 |
| AC141169. | 0.396712 | 2.244302 | 0.85179 | 1.82777 | 2.573751 | 1.739185 | 0.887646 | 1.758126 |
| Syna | 0 | 0 | 0 | 0 | 0.018254 | 0.059206 | 0.018886 | 0.014963 |
| Paip2l1 | 0.281537 | 0 | 0.453372 | 0.259425 | 0.152211 | 0.246852 | 0 | 0.374311 |
| Mief2 | 33.11129 | 33.87489 | 33.90415 | 32.81829 | 31.13937 | 32.64271 | 31.72039 | 32.01171 |
| AABR07029 | 0 | 0 | 0 | 0.778276 | 0 | 1.110834 | 0.472457 | 0 |
| AABR07026 | 0 | 0.028692 | 0 | 0.093469 | 0.02742 | 0 | 0 | 0.044954 |
| Mob1b | 6.332713 | 6.573548 | 5.6135 | 5.496289 | 5.758577 | 5.348766 | 6.781435 | 5.750319 |
| AABR07017 | 0 | 0 | 0 | 0 | 0.050153 | 0.122005 | 0 | 0 |
| Tex26 | 0.060399 | 0 | 0 | 0 | 0 | 0 | 0 | 0 |
| AABR07012 | 0.038963 | 0 | 0 | 0.035903 | 0.04213 | 0.068325 | 0.04359 | 0.034535 |
| Slc4a1 | 2.533647 | 2.945966 | 2.58215 | 1.441677 | 1.186737 | 1.300143 | 3.644397 | 3.632441 |
| Slc1a4 | 1.19075 | 1.393207 | 1.757725 | 2.057304 | 1.872786 | 1.72031 | 2.119342 | 2.122835 |
| Zfp93 | 0.02496 | 0.028241 | 0.080388 | 0.114998 | 0.053978 | 0.021885 | 0.055848 | 0.022123 |
| AABR07021 | 0 | 0.512983 | 0 | 0 | 0 | 0 | 0.253613 | 0 |
| Loxl3 | 1.801204 | 1.815653 | 1.810654 | 3.304389 | 3.293242 | 3.115523 | 3.444002 | 3.512293 |
| Fundc1 | 23.56857 | 20.45626 | 19.43758 | 18.98484 | 19.0992 | 17.92786 | 17.40369 | 20.08298 |
| Dzip1l | 5.625273 | 5.948945 | 5.82666 | 5.248587 | 5.272542 | 5.192488 | 5.344566 | 5.424423 |
| Plxnb1 | 19.80361 | 21.86958 | 22.61429 | 17.28706 | 16.76706 | 17.63684 | 19.86497 | 19.76774 |
| Tor1aip1 | 11.4965 | 11.00656 | 11.57366 | 10.43835 | 10.42747 | 9.600134 | 12.31998 | 12.7094 |
| Cct8 | 48.46081 | 48.90852 | 46.80758 | 51.94725 | 54.62354 | 51.66973 | 50.35818 | 50.94366 |
| Blmh | 24.14567 | 20.88818 | 22.41166 | 25.63303 | 23.76949 | 21.47969 | 24.64946 | 26.17226 |
| #N/A | 0 | 0 | 0.38114 | 0 | 0 | 0 | 0 | 0 |
| Fam120a | 27.54372 | 29.78406 | 30.99723 | 23.76625 | 22.49341 | 23.64294 | 30.88469 | 30.01417 |
| Aasdh | 2.586559 | 2.352029 | 2.453156 | 2.880566 | 2.693859 | 3.019225 | 3.035751 | 3.263081 |
| AC110837. | 0 | 0.42021 | 0 | 0.342221 | 0 | 0 | 0.415494 | 0 |
| Asb3 | 5.808557 | 5.029675 | 5.376836 | 5.133893 | 5.030976 | 5.092948 | 5.37109 | 4.990808 |
| Unc80 | 0.03796 | 0.021475 | 0.061129 | 0.017489 | 0 | 0 | 0.042468 | 0.016823 |
| Grin1 | 0 | 0 | 0 | 0.033255 | 0 | 0 | 0 | 0.010663 |
| Cdhr4 | 0 | 0.1531 | 0 | 0.24937 | 0 | 0 | 0 | 0 |
| Hvcn1 | 1.236295 | 1.448767 | 1.327242 | 1.383311 | 1.909697 | 1.432408 | 2.222858 | 2.191579 |
| Rn60_X_06 | 0 | 0.05262 | 0.049927 | 0.042854 | 0 | 0.12233 | 0.052029 | 0.082441 |
| Hand1 | 0.049542 | 0.280272 | 0.053187 | 0.045651 | 0.053569 | 0.217192 | 0.055425 | 0.131735 |
| Arap1 | 10.59295 | 11.91533 | 11.54955 | 13.262 | 13.65326 | 14.09481 | 18.146 | 17.78979 |
| Lrrc8d | 3.792703 | 3.36902 | 3.446666 | 3.356873 | 3.183667 | 3.150422 | 3.666194 | 4.025147 |
| Il17re | 0.050956 | 0.134527 | 0.07294 | 0.719962 | 0.642812 | 0.565926 | 0.475062 | 0.662419 |
| Fau | 390.9134 | 410.8109 | 407.5817 | 414.2852 | 424.9658 | 416.8425 | 397.5973 | 403.3681 |
| Gpbar1 | 0 | 0 | 0 | 0 | 0.057194 | 0.046378 | 0.059176 | 0.046883 |
| Fmn2 | 0.029068 | 0.049334 | 0.031206 | 0.013392 | 0.047146 | 0.012743 | 0.01626 | 0.064411 |
| AABR07058 | 0 | 0.012221 | 0 | 0 | 0 | 0.009471 | 0 | 0 |
| Pla2g10 | 0 | 0 | 0 | 0.0544 | 0 | 0 | 0.066048 | 0.052328 |
| Zdhhc8 | 7.027731 | 8.004322 | 8.64638 | 8.109048 | 6.539833 | 8.084112 | 9.445212 | 9.949906 |
| Unc93b1 | 8.491771 | 9.557203 | 10.49116 | 10.39171 | 10.46946 | 11.30627 | 12.18943 | 12.06658 |

| | | | | | | | | |
|---|---|---|---|---|---|---|---|---|
| Lamc3 | 0.419016 | 0.336858 | 0.414326 | 0.314982 | 0.226538 | 0.415734 | 0.555129 | 0.488677 |
| Dcaf10 | 1.922634 | 1.871285 | 1.875418 | 2.124052 | 2.61541 | 2.220499 | 2.34755 | 2.418759 |
| Sap25 | 3.753829 | 2.973097 | 3.291147 | 4.150807 | 2.570676 | 3.126793 | 4.689573 | 4.21446 |
| Mir151b | 0.872765 | 0 | 0.936969 | 0.402109 | 0 | 0 | 0 | 1.160363 |
| Suco | 4.302434 | 4.267837 | 4.397481 | 4.534763 | 4.42911 | 3.979083 | 5.423285 | 5.210859 |
| AABR07057 | 0 | 0 | 0 | 0 | 0 | 0 | 0 | 0.087082 |
| Tuba1a | 34.943 | 35.21264 | 39.80816 | 41.47919 | 43.4211 | 42.90463 | 48.07783 | 49.22752 |
| Rock2 | 32.78715 | 32.42504 | 29.03263 | 30.23646 | 31.27356 | 31.04546 | 38.32928 | 37.44443 |
| Chst10 | 0.301069 | 0.408774 | 0.430956 | 0.906249 | 0.737894 | 0.510356 | 0.516461 | 0.960668 |
| AABR07049 | 0.952108 | 0.359088 | 0.340716 | 0.292443 | 0.343167 | 0.27827 | 1.065176 | 0.8439 |
| Pex5 | 0.023514 | 0.026605 | 0 | 1.040032 | 0 | 0 | 0 | 0.020842 |
| AC112624. | 0.095908 | 0 | 0 | 0 | 0 | 0 | 0 | 0 |
| Coa5 | 86.04036 | 82.83302 | 75.95046 | 74.58096 | 75.06251 | 74.8825 | 68.30587 | 71.09135 |
| AABR07027 | 2.266923 | 0.897721 | 0.730106 | 0.731108 | 1.103036 | 0.695674 | 1.394873 | 1.306036 |
| Srpra | 31.65115 | 32.99731 | 32.29897 | 31.25981 | 30.05028 | 31.07971 | 36.29754 | 37.84247 |
| Alg9 | 2.197877 | 2.281462 | 2.576034 | 2.508345 | 2.441941 | 2.91717 | 2.549121 | 2.734471 |
| LOC102546 | 1.165126 | 1.245047 | 0.486437 | 0.596454 | 0.90988 | 0.964828 | 1.158658 | 1.434318 |
| Gprc5b | 18.38537 | 19.16732 | 19.65763 | 15.63291 | 15.75841 | 15.44317 | 15.30113 | 16.09714 |
| Pfdn4 | 13.03918 | 15.14769 | 11.07895 | 13.17163 | 11.08323 | 9.782048 | 12.16938 | 12.05171 |
| AC103129. | 0 | 0 | 0 | 0 | 0 | 0.337607 | 0 | 0 |
| Mir874 | 0 | 0.7796 | 0 | 0.63491 | 0 | 0 | 0 | 0 |
| Senp3 | 10.83195 | 10.64255 | 10.46838 | 11.57058 | 10.76748 | 10.52585 | 11.80956 | 12.08775 |
| Ccdc57 | 1.630535 | 1.767195 | 1.787336 | 1.23361 | 1.280548 | 1.444704 | 1.766565 | 1.430011 |
| Sun2 | 29.70007 | 30.50911 | 32.74609 | 30.36195 | 29.44549 | 30.42331 | 39.71608 | 40.05212 |
| Mir29b2 | 0 | 1.410704 | 2.007791 | 0 | 4.718543 | 3.279606 | 3.487182 | 1.657661 |
| Cep170b | 5.353607 | 5.86698 | 6.305761 | 5.764724 | 5.069315 | 5.02263 | 6.639653 | 6.819484 |
| AABR07068 | 0.029821 | 0.033741 | 0 | 0.01374 | 0.016123 | 0 | 0.050044 | 0.039648 |
| Psmc1 | 66.47941 | 68.86754 | 69.73281 | 71.30342 | 73.67241 | 68.87173 | 70.12737 | 72.00864 |
| AABR07072 | 0 | 0 | 0 | 0 | 0 | 0 | 0 | 0 |
| Pank4 | 16.40053 | 15.41275 | 15.99131 | 13.35229 | 13.5193 | 15.00595 | 16.66447 | 15.51141 |
| Klk13 | 0 | 0.114161 | 0 | 0 | 0 | 0 | 0 | 0.044715 |
| Rn60_20_( | 0 | 0.433745 | 0.493864 | 0.211946 | 0 | 0.134449 | 0.428877 | 0.135914 |
| Ssbp2 | 11.72215 | 11.80255 | 13.48337 | 13.8555 | 12.75044 | 14.59108 | 14.40223 | 13.35848 |
| Smim8 | 27.91464 | 27.82222 | 25.13457 | 20.04165 | 22.46925 | 20.71011 | 21.38805 | 23.63702 |
| Kdm5c | 14.09852 | 13.31899 | 14.33794 | 12.81105 | 12.00955 | 14.28184 | 15.90405 | 16.93315 |
| Fam214b | 11.23111 | 11.66801 | 10.70118 | 12.65587 | 12.6904 | 12.04248 | 14.08088 | 13.98543 |
| AABR07002 | 0.095733 | 0.216635 | 0.102775 | 0.176428 | 0.931632 | 0.587571 | 0.428407 | 0.509117 |
| Nedd8 | 123.2867 | 123.3977 | 131.6922 | 141.1499 | 140.1073 | 139.8223 | 126.6259 | 132.9392 |
| AABR07065 | 0.131353 | 0 | 0.235026 | 0.282418 | 0.284059 | 0.3839 | 0.293903 | 0.155233 |
| Hsdl1 | 7.363069 | 7.140828 | 7.416402 | 6.44423 | 6.117199 | 6.730136 | 6.26557 | 6.274275 |
| Cpa4 | 0 | 0 | 0 | 0 | 0 | 0.03302 | 0 | 0 |
| AABR07044 | 0.711977 | 0.281949 | 0.611482 | 0.229621 | 0.384925 | 0.343344 | 0.916008 | 0.915038 |
| Tnfrsf10b | 1.479728 | 1.302188 | 1.235564 | 1.21201 | 0.977783 | 1.153268 | 1.747423 | 2.695977 |
| AC096430. | 0 | 0 | 0 | 0 | 0.456633 | 0.370278 | 0 | 0 |
| Bud13 | 3.003411 | 3.535243 | 3.016331 | 3.615637 | 3.378494 | 3.355453 | 4.769149 | 3.520805 |
| Cdc40 | 6.190221 | 5.631549 | 6.174119 | 6.571214 | 7.575296 | 6.289404 | 5.357783 | 5.746206 |
| LOC257650 | 1.723643 | 1.547795 | 1.204255 | 1.487427 | 1.715834 | 1.823146 | 1.805901 | 1.988501 |
| Chst8 | 0.025457 | 0 | 0 | 0.023458 | 0.055053 | 0 | 0.142403 | 0.090257 |
| AABR07030 | 0 | 0 | 0 | 0.450964 | 0 | 0 | 0 | 0 |
| Efr3a | 16.93582 | 17.38761 | 15.68263 | 18.85044 | 18.65375 | 18.29142 | 16.57453 | 17.0918 |
| AABR07065 | 0 | 0 | 0 | 0 | 0 | 0 | 0 | 0 |
| Ccnd3 | 50.9958 | 53.38973 | 54.83551 | 60.37323 | 55.22991 | 58.60043 | 67.22981 | 66.47647 |
| AABR07067 | 0 | 0 | 0 | 0 | 0.442363 | 0 | 0 | 0 |
| Ly6i | 0 | 0 | 0 | 0 | 0.059291 | 0.096156 | 0.061345 | 0.048602 |

| | | | | | | | | |
|---|---|---|---|---|---|---|---|---|
| C8g | 3.054148 | 2.519727 | 2.527426 | 2.169339 | 3.302407 | 4.072609 | 4.199873 | 3.609391 |
| Egr2 | 0.777471 | 0.73306 | 1.095498 | 2.611908 | 2.381894 | 2.840364 | 3.044301 | 2.641593 |
| Pm20d2 | 6.101276 | 6.080396 | 5.769302 | 3.872165 | 3.844738 | 3.755351 | 4.339605 | 3.90369 |
| Apobec2 | 117.8015 | 126.6596 | 134.5857 | 117.6925 | 114.7102 | 117.469 | 125.5316 | 124.7148 |
| Tmem215 | 0.034864 | 0.078894 | 0.018714 | 0.064252 | 0.037698 | 0.015284 | 0 | 0 |
| Ppcs | 10.26068 | 11.26601 | 10.59183 | 10.23806 | 9.453498 | 9.023195 | 7.50563 | 8.125904 |
| AC141526. | 4.080461 | 5.386325 | 4.380636 | 3.13332 | 1.470715 | 7.155504 | 3.043359 | 4.822288 |
| AABR07021 | 0.099935 | 0 | 0 | 0 | 0 | 0 | 0 | 0 |
| Gins1 | 1.458661 | 1.430352 | 0.835182 | 1.568115 | 1.10406 | 0.9379 | 1.087923 | 1.163595 |
| Prpf6 | 11.22491 | 11.85376 | 11.52116 | 13.02317 | 11.97183 | 12.01919 | 14.04205 | 14.78813 |
| Chrna1 | 3.868422 | 3.990737 | 3.939241 | 3.826702 | 3.721523 | 4.439315 | 3.691375 | 4.437238 |
| Khdc3 | 0 | 0 | 0.129237 | 0.073951 | 0.043389 | 0 | 0.134677 | 0.035567 |
| Gemin8 | 7.093572 | 6.696889 | 6.014713 | 5.703779 | 6.399956 | 6.893115 | 6.31845 | 6.247338 |
| AABR07065 | 0 | 0 | 0 | 0.399888 | 0 | 0 | 0 | 0.512868 |
| Cfp | 5.49169 | 5.411835 | 5.515314 | 6.823346 | 5.784844 | 7.486733 | 8.878867 | 9.264061 |
| AABR07060 | 3.530024 | 3.967606 | 4.24146 | 5.450019 | 4.448912 | 4.427468 | 3.975388 | 5.200913 |
| Ceacam9 | 0 | 0 | 0.057365 | 0 | 0 | 0 | 0 | 0 |
| Fam131a | 0.71039 | 1.935617 | 0.856035 | 0.841624 | 0.595696 | 2.326232 | 0.87585 | 0.616804 |
| Alpk3 | 27.17725 | 28.19042 | 29.30994 | 30.8759 | 30.4346 | 30.33117 | 41.06488 | 40.76693 |
| Ftl1l1 | 0.474329 | 0.966026 | 0.712911 | 0.34966 | 0.512885 | 0.914963 | 0.530658 | 0.924927 |
| AABR07068 | 0.254204 | 0.479365 | 0.272904 | 0.390398 | 0.458111 | 0.148591 | 0.379189 | 0.67594 |
| Tarbp1 | 1.783188 | 1.654176 | 1.605214 | 1.592109 | 1.580832 | 1.524656 | 1.84626 | 1.963389 |
| AABR07071 | 0.052418 | 0 | 0 | 0 | 0 | 0 | 0 | 0 |
| AC129234. | 2.380269 | 0.897721 | 2.555371 | 1.096662 | 0.428958 | 0.695674 | 1.33147 | 0.70325 |
| Tk2 | 3.792091 | 3.372935 | 3.247428 | 4.302184 | 3.910722 | 4.016797 | 3.702924 | 4.021685 |
| AABR07065 | 0.019168 | 0 | 0 | 0 | 0 | 0 | 0 | 0 |
| Ep300 | 6.039104 | 7.429126 | 6.265801 | 5.153973 | 5.478184 | 5.081866 | 6.756281 | 6.125136 |
| Ubtf | 17.82075 | 18.73304 | 19.27583 | 18.10137 | 17.51536 | 19.19561 | 23.42886 | 23.91622 |
| Adamts3 | 0.262142 | 0.310725 | 0.348432 | 0.195543 | 0.148474 | 0.175121 | 0.125688 | 0.188092 |
| Tma7 | 7.04163 | 4.039744 | 4.897794 | 4.93498 | 5.790939 | 5.391474 | 4.993011 | 24.26213 |
| Ube2q2 | 17.1775 | 15.08743 | 18.39144 | 16.42569 | 14.61872 | 16.64451 | 18.28505 | 18.09797 |
| Cnih3 | 0 | 0.24534 | 0.349181 | 0 | 0.117231 | 0.095061 | 0 | 0.192193 |
| Akap5 | 0.174553 | 0.241387 | 0.187394 | 0.285945 | 0.293598 | 0.102032 | 0.238678 | 0.275049 |
| AABR07020 | 0.216836 | 0 | 0.116394 | 0 | 0.351693 | 0 | 0.121293 | 0.288289 |
| Naf1 | 1.072903 | 1.120559 | 1.269966 | 1.22946 | 1.056002 | 0.988964 | 1.138762 | 1.377683 |
| Rpp21 | 15.1852 | 12.70378 | 15.80827 | 17.3847 | 18.70832 | 13.63717 | 17.19445 | 15.90656 |
| C1ql4 | 0 | 0.082635 | 0 | 0 | 0.078971 | 0 | 0 | 0.129469 |
| Prkd3 | 9.251169 | 9.079742 | 7.876086 | 8.048797 | 8.397998 | 7.602111 | 11.12001 | 10.56436 |
| Nudt6 | 11.19628 | 11.41445 | 9.140147 | 10.4244 | 9.899482 | 9.04996 | 10.56873 | 8.52828 |
| Eme1 | 0.075023 | 0.063664 | 0.080542 | 0.103696 | 0.040561 | 0.016445 | 0.083932 | 0.083121 |
| Cxcr4 | 8.048524 | 8.00155 | 8.35138 | 11.48145 | 12.59896 | 11.89938 | 8.928981 | 8.656084 |
| Chrna7 | 0.046902 | 0.079601 | 0.025176 | 0.064827 | 0.076071 | 0.123371 | 0.104943 | 0.083143 |
| Panx1 | 0.834111 | 0.506407 | 1.048357 | 0.61863 | 0.769925 | 1.016754 | 0.773846 | 0.991763 |
| AABR07042 | 0 | 0 | 0.087567 | 0.375803 | 0.088197 | 0.071518 | 0.091253 | 0 |
| Mir362 | 0 | 0.911532 | 0 | 0 | 0 | 0 | 0 | 0 |
| AABR07048 | 12.77526 | 10.30427 | 11.94975 | 10.14015 | 8.069392 | 7.430605 | 10.47166 | 12.55658 |
| Hmgn5b | 1.596208 | 2.155589 | 1.879467 | 1.565736 | 1.698119 | 1.399557 | 2.304215 | 2.350391 |
| AC111422. | 0 | 0 | 0 | 0 | 0.529182 | 0.429107 | 0 | 0 |
| Nt5dc3 | 6.104835 | 5.341672 | 6.146131 | 4.375284 | 5.22218 | 5.067246 | 6.07095 | 6.541321 |
| Slc6a5 | 0.014951 | 0 | 0 | 0 | 0 | 0 | 0 | 0 |
| AABR07047 | 0.076783 | 0.347505 | 0.164863 | 0 | 0.083024 | 0.134647 | 0.343605 | 0.068056 |
| AABR07044 | 0 | 0 | 0.892352 | 0.765923 | 0 | 0 | 0 | 0 |
| Rab11fip4 | 0.803826 | 0.625275 | 0.755089 | 0.478365 | 0.597551 | 0.440497 | 0.562053 | 0.474981 |
| Ubfd1 | 11.54931 | 11.40716 | 12.04883 | 10.91768 | 10.37247 | 12.34235 | 10.82311 | 11.41696 |

| | | | | | | | | |
|---|---|---|---|---|---|---|---|---|
| Tmem258b | 1.090957 | 0.493746 | 1.873939 | 2.010547 | 1.415563 | 0.956552 | 0.976411 | 1.353757 |
| Ccna1 | 0 | 0 | 0.09171 | 0.026239 | 0.184739 | 0.049934 | 0.031857 | 0.075717 |
| Impdh2 | 36.74241 | 34.70384 | 37.49021 | 43.33656 | 39.34902 | 41.40428 | 40.74833 | 41.65696 |
| Yap1 | 44.33033 | 48.46111 | 48.25186 | 43.18691 | 43.527 | 42.84678 | 46.88494 | 43.55195 |
| Rab3gap1 | 12.75667 | 14.8445 | 13.71947 | 14.30652 | 12.76277 | 14.2102 | 14.06845 | 13.76139 |
| Hamp | 19.20084 | 17.44571 | 16.08464 | 43.56186 | 53.31953 | 43.7463 | 37.75456 | 35.06875 |
| Dusp16 | 5.036454 | 5.474551 | 5.052787 | 4.093714 | 4.779977 | 5.26445 | 7.13547 | 6.666848 |
| Hmgcs2 | 7.443665 | 7.892091 | 7.432421 | 7.530559 | 6.050616 | 7.713268 | 7.250289 | 7.58965 |
| Fermt3 | 2.325004 | 2.630633 | 2.793733 | 3.970319 | 3.574945 | 3.478653 | 4.844272 | 4.14044 |
| Pgam5 | 9.33228 | 9.874929 | 9.426138 | 10.36764 | 9.493935 | 9.404172 | 9.322961 | 10.3454 |
| Amot | 1.119182 | 1.01082 | 0.938023 | 0.741799 | 0.828001 | 0.766102 | 0.955543 | 0.965881 |
| Mast2 | 17.09694 | 16.69624 | 17.45582 | 17.84068 | 17.57108 | 18.31074 | 20.20234 | 20.85449 |
| AABR07051 | 0 | 0 | 0 | 0 | 0 | 0.318851 | 0 | 0 |
| LOC10255( | 7.803357 | 7.60749 | 8.482778 | 7.95928 | 6.792579 | 8.348088 | 6.918152 | 7.134003 |
| Ttc39c | 13.40286 | 12.80575 | 12.96448 | 11.17756 | 11.7988 | 10.46964 | 10.81423 | 10.99165 |
| AABR0701( | 1.246808 | 1.410704 | 2.40935 | 0.459554 | 2.426679 | 1.311842 | 1.673848 | 1.547151 |
| Poc5 | 2.977327 | 3.765023 | 2.527833 | 4.375238 | 4.229329 | 4.555618 | 4.506513 | 4.622479 |
| AABR0703 | 0.149617 | 0.266019 | 0.206516 | 0.256037 | 0.36978 | 0.468515 | 0.717363 | 0.492562 |
| Ctxn3 | 0 | 0 | 0.052688 | 0.045223 | 0.159201 | 0 | 0 | 0.1305 |
| Nop14 | 4.557059 | 3.82608 | 3.699473 | 3.754011 | 3.743493 | 4.080346 | 4.449696 | 4.096238 |
| Pemt | 2.709598 | 2.465955 | 3.667777 | 3.093846 | 2.547695 | 2.375778 | 3.953971 | 2.76712 |
| Fjx1 | 1.444921 | 0.986553 | 1.123293 | 1.767598 | 1.885621 | 2.184324 | 2.229673 | 2.141869 |
| Slc12a2 | 5.849341 | 6.081389 | 5.735113 | 5.676396 | 6.094815 | 5.580608 | 5.830094 | 5.87342 |
| AABR07007 | 0.108418 | 0.36801 | 0 | 0 | 0.234462 | 0.190122 | 0 | 0.096096 |
| Abcb10 | 7.975357 | 7.850483 | 8.889476 | 6.688553 | 6.760405 | 7.113135 | 8.683633 | 8.1097 |
| AC112739. | 0 | 0 | 0 | 0.27892 | 0 | 0 | 0 | 0 |
| AABR0700( | 0.168972 | 0.156423 | 0.230875 | 0.127392 | 0.01661 | 0.026937 | 0.154668 | 0.068076 |
| Hpcal1 | 14.07922 | 12.8788 | 14.64365 | 13.11792 | 12.85026 | 13.49941 | 16.4879 | 17.75981 |
| Mfn1 | 55.43093 | 55.15449 | 50.72236 | 50.08591 | 50.20576 | 49.61681 | 51.43486 | 51.67444 |
| Galnt16 | 1.799656 | 1.745336 | 1.728672 | 2.880226 | 2.999384 | 2.906731 | 4.43548 | 3.574038 |
| #N/A | 0 | 0 | 0 | 0 | 0.345259 | 0 | 0 | 0 |
| Mcoln3 | 0.094694 | 0.035714 | 0.10166 | 0.232685 | 0.238914 | 0.193732 | 0.141253 | 0.167864 |
| Zfp638 | 6.190353 | 5.461135 | 5.039759 | 6.374349 | 6.112658 | 5.855256 | 6.786797 | 7.442732 |
| Spry4 | 6.87055 | 7.337679 | 6.690387 | 5.14389 | 5.155077 | 5.319361 | 7.082881 | 7.212012 |
| Lrrc56 | 0.552883 | 0.893659 | 0.593555 | 0.6065 | 0.512421 | 0.807947 | 0.530178 | 0.770075 |
| Ube2j1 | 23.2116 | 27.84493 | 28.59696 | 28.3463 | 32.50692 | 24.27522 | 34.72843 | 34.88835 |
| Nhej1 | 2.152533 | 1.889604 | 1.792925 | 2.222859 | 2.126856 | 1.561939 | 1.287119 | 1.578949 |
| Plch1 | 0.008376 | 0 | 0 | 0 | 0 | 0.014688 | 0 | 0.007424 |
| Irx5 | 10.99539 | 12.93315 | 15.04342 | 11.70907 | 10.75985 | 11.87926 | 11.39236 | 10.36291 |
| AC128488. | 0 | 0 | 0 | 0 | 0 | 0 | 0.944914 | 0 |
| LOC50095( | 3.665614 | 2.99951 | 3.197408 | 4.433257 | 3.857409 | 4.017517 | 5.27262 | 4.728479 |
| Trappc1 | 18.10447 | 20.0763 | 19.74605 | 24.32596 | 20.04406 | 24.79129 | 18.3178 | 21.35324 |
| Ogdh | 129.5368 | 131.1699 | 132.4001 | 97.22146 | 98.43 | 96.0371 | 119.349 | 118.1282 |
| Jakmip3 | 0 | 0 | 0 | 0.044504 | 0.026111 | 0.010587 | 0 | 0.032106 |
| Rmnd1 | 10.42591 | 11.17244 | 9.275715 | 9.873259 | 11.10301 | 10.47698 | 11.31148 | 10.28847 |
| AABR0702( | 0 | 0.722556 | 0 | 0 | 0 | 0 | 0 | 0 |
| LOC102548 | 0 | 0 | 0 | 0 | 0 | 0.023201 | 0 | 0 |
| LOC10036( | 0 | 0 | 0 | 0 | 0 | 0 | 0.083692 | 0 |
| Pias3 | 6.962595 | 6.62861 | 6.628131 | 7.76535 | 7.065632 | 7.487775 | 7.965901 | 7.988729 |
| Itgb8 | 1.640011 | 2.438165 | 2.108693 | 3.373854 | 3.56726 | 3.193615 | 2.901498 | 3.397421 |
| Ren | 0 | 0 | 0.060645 | 0 | 0.061081 | 0.04953 | 0.063198 | 0.150209 |
| Ctps2 | 3.744996 | 2.933506 | 3.419136 | 3.922779 | 3.322592 | 4.167666 | 3.921162 | 4.298166 |
| Arl14 | 0.090442 | 0 | 0 | 0 | 0.097794 | 0 | 0 | 0 |
| AABR07015 | 0 | 0 | 0 | 0 | 0 | 0.355926 | 0 | 0 |

| | | | | | | | | |
|---|---|---|---|---|---|---|---|---|
| Wnt9b | 0.182852 | 0.397862 | 0.302005 | 0.168491 | 0.106462 | 0.234321 | 0.236038 | 0.28674 |
| AABR07053 | 0 | 0.322009 | 0 | 0 | 0.615462 | 0 | 0.318395 | 0.252253 |
| Sf3b4 | 9.496234 | 9.894254 | 11.62502 | 11.42589 | 10.30508 | 8.805518 | 11.15898 | 13.50351 |
| Tgm3 | 0.333291 | 0.266191 | 0.378857 | 0.126459 | 0.169592 | 0.18909 | 0.175469 | 0.243281 |
| Utp18 | 4.020784 | 3.50226 | 3.220297 | 4.998801 | 4.485635 | 4.196937 | 4.569675 | 4.242643 |
| Glg1 | 17.54184 | 17.2851 | 17.50852 | 19.63853 | 19.51508 | 20.27999 | 23.22622 | 22.89952 |
| Tubgcp2 | 9.005604 | 9.623339 | 8.554073 | 9.442316 | 9.136541 | 9.309625 | 10.19945 | 8.639079 |
| Caap1 | 3.32482 | 3.644319 | 4.573303 | 3.70196 | 4.119363 | 4.312075 | 3.603422 | 3.959969 |
| AABR07071 | 16.12397 | 16.40242 | 18.58058 | 18.40162 | 18.23437 | 13.2296 | 17.5423 | 19.0116 |
| Cyp4f40 | 0 | 0 | 0 | 0 | 0 | 0 | 0 | 0.029414 |
| Rn50_20_( | 0 | 0 | 0 | 0 | 0.059855 | 0 | 0.061929 | 0 |
| LOC102556 | 0.961725 | 1.251368 | 1.26478 | 1.262823 | 1.065897 | 1.559996 | 1.936683 | 1.172084 |
| Thnsl1 | 4.394892 | 4.173443 | 4.065232 | 3.416951 | 4.327835 | 3.664213 | 2.809606 | 3.564997 |
| Rbbp7 | 57.24287 | 54.65635 | 53.87818 | 60.20344 | 59.68637 | 58.55172 | 54.86597 | 56.0983 |
| Eif3j | 82.06211 | 84.37585 | 76.8615 | 71.27396 | 75.13191 | 74.10715 | 76.21506 | 82.55172 |
| Rbm12 | 0.21967 | 0.248546 | 0.74377 | 0.217988 | 0.127899 | 0.57782 | 0.170139 | 0.269591 |
| Ocln | 0.280782 | 0.174731 | 0.195935 | 0.413968 | 0.227704 | 0.393904 | 0.392659 | 0.236428 |
| Il2ra | 0.616814 | 0.483159 | 0.713127 | 0.422634 | 0.444635 | 0.429885 | 0.867004 | 0.574749 |
| Ing3 | 3.424673 | 3.572563 | 3.754831 | 3.580938 | 3.414159 | 3.833306 | 4.374829 | 3.57366 |
| Ttc39a | 0.383958 | 0 | 0 | 0 | 0.063872 | 0 | 0 | 0 |
| AABR07043 | 2.276779 | 4.12171 | 2.444268 | 1.258777 | 2.461848 | 0.798513 | 1.018864 | 1.210814 |
| Ppox | 5.79269 | 6.029824 | 5.068341 | 5.684689 | 5.981692 | 5.815496 | 5.605723 | 5.339723 |
| Pex7 | 5.061139 | 4.466624 | 4.201873 | 3.668731 | 3.429456 | 4.053018 | 3.472802 | 3.469126 |
| Eef2 | 405.2659 | 415.1533 | 444.0984 | 387.944 | 374.2288 | 398.7965 | 478.9603 | 481.5208 |
| Prss46 | 0.082531 | 0 | 0 | 0.190123 | 0.178479 | 0.072363 | 0.138498 | 0.329181 |
| Pih1d2 | 1.204737 | 1.27223 | 1.164026 | 1.184126 | 1.650043 | 1.267578 | 0.628976 | 1.245788 |
| AY172581. | 8.59739 | 3.537288 | 9.229847 | 10.08274 | 9.296233 | 11.64994 | 8.74398 | 10.39131 |
| Cltb | 243.324 | 248.7124 | 259.5056 | 220.0656 | 214.0515 | 207.0756 | 270.8771 | 269.2663 |
| AABR0700( | 0.288783 | 0.326744 | 0.620053 | 0.443503 | 1.040855 | 0.253205 | 0.43077 | 0.255962 |
| Pspn | 1.445344 | 1.006362 | 1.193592 | 0.922034 | 1.803265 | 1.364762 | 0.746301 | 1.872348 |
| AABR07061 | 0 | 0 | 0 | 0 | 0 | 0 | 0 | 0 |
| AC127920. | 0.818218 | 0 | 0 | 0 | 0 | 0 | 0 | 0.362613 |
| Rad17 | 2.463833 | 2.594715 | 2.665429 | 2.497357 | 2.582134 | 3.124113 | 3.562151 | 3.359719 |
| AABR07049 | 0 | 0.452287 | 0 | 0.368345 | 0 | 0 | 0 | 0 |
| Stat3 | 30.81879 | 32.75586 | 30.97839 | 32.32132 | 33.20882 | 32.70422 | 42.31115 | 43.52147 |
| Igip | 4.848696 | 8.411977 | 9.716719 | 10.1272 | 8.38852 | 9.069528 | 9.402477 | 11.46037 |
| Nol11 | 8.703343 | 8.33455 | 7.882026 | 10.08074 | 9.279363 | 8.547683 | 7.860245 | 8.317551 |
| AABR07006 | 0.184387 | 0.173854 | 0.362911 | 0.198223 | 0.299063 | 0.269451 | 0.343807 | 0.394959 |
| Snapin | 52.84666 | 54.97584 | 51.79444 | 53.18919 | 52.38975 | 54.94684 | 49.94103 | 50.4016 |
| Csrnp1 | 6.151091 | 7.596099 | 7.402253 | 5.55095 | 6.023254 | 5.918291 | 7.998045 | 8.250398 |
| AC128960. | 0.165453 | 0.093601 | 0 | 0.076229 | 0.089451 | 0 | 0.092551 | 0.219974 |
| AABR07043 | 0 | 0 | 0 | 0 | 0 | 0 | 0 | 0.132613 |
| Mocos | 0.862132 | 1.190064 | 1.20322 | 1.429958 | 1.32374 | 1.829323 | 2.48845 | 2.521698 |
| #N/A | 0 | 0 | 0 | 0 | 0 | 0 | 0 | 0 |
| Ryk | 30.47345 | 30.62058 | 29.49682 | 30.08218 | 30.21453 | 32.24142 | 34.30772 | 32.71443 |
| Mpc2 | 626.0728 | 605.4566 | 587.2163 | 506.5323 | 536.5868 | 485.0913 | 492.4773 | 478.8915 |
| Tmprss2 | 0.552918 | 0.5474 | 0.445194 | 0.785467 | 0.448396 | 0.585799 | 2.448545 | 2.348293 |
| Ift172 | 11.21669 | 12.90872 | 13.03959 | 12.63913 | 12.02449 | 12.75715 | 12.728 | 12.83927 |
| Stap2 | 2.622042 | 2.881954 | 3.257275 | 2.278045 | 2.430151 | 3.08724 | 2.598175 | 3.320066 |
| Cdc42ep5 | 6.283911 | 8.0128 | 10.60114 | 7.720502 | 7.010406 | 9.882546 | 9.485136 | 10.34381 |
| AABR07067 | 0 | 0.588572 | 0 | 0 | 0 | 0 | 0.581967 | 0.307381 |
| AABR07036 | 1.21311 | 2.287628 | 1.374704 | 3.291398 | 0.801606 | 0.88638 | 1.206377 | 0.358413 |
| AABR07007 | 6.818479 | 4.320282 | 3.220832 | 4.021095 | 3.833816 | 3.826207 | 4.271799 | 4.593103 |
| AABR07043 | 0 | 0.45402 | 0.646186 | 0 | 0 | 0.351835 | 0.224462 | 0.177833 |

| | | | | | | | | |
|---|---|---|---|---|---|---|---|---|
| AC110709. | 0.943978 | 0.730782 | 0.746731 | 0.9614 | 0.966988 | 0.653432 | 0.778164 | 0.924767 |
| Scamp5 | 1.248705 | 1.35273 | 1.026816 | 1.958524 | 1.608757 | 1.910192 | 2.110356 | 1.907446 |
| Cables2 | 3.527427 | 3.332789 | 3.552675 | 3.551967 | 3.97144 | 4.081288 | 4.963423 | 4.996007 |
| Col23a1 | 0 | 0.148495 | 0.093932 | 0.040312 | 0.047304 | 0.038358 | 0.097886 | 0.038776 |
| Dennd2c | 4.089803 | 4.243739 | 4.114869 | 3.920094 | 3.90002 | 3.360695 | 3.885718 | 3.952885 |
| Rasa1 | 12.14968 | 11.69861 | 12.36064 | 10.59435 | 11.39151 | 11.01033 | 13.79321 | 13.54419 |
| Ccdc82 | 1.493446 | 1.384168 | 1.705648 | 1.991027 | 1.580483 | 1.365176 | 1.759673 | 1.506782 |
| AABR07035 | 0 | 0 | 0 | 0 | 0 | 0 | 0 | 0.110248 |
| Ice1 | 5.012221 | 4.758898 | 5.029831 | 4.583522 | 4.366965 | 4.421395 | 5.60745 | 5.770636 |
| Adgra2 | 7.650568 | 7.902898 | 8.213374 | 7.831661 | 7.42544 | 8.402202 | 10.07814 | 10.70392 |
| BC030336 | 13.64926 | 11.73112 | 10.56732 | 11.12604 | 8.940397 | 10.70187 | 10.7185 | 13.7266 |
| AABR07042 | 0.214614 | 0.485652 | 0.460805 | 0 | 0 | 0.062725 | 0.160067 | 0 |
| Chpf | 10.53781 | 11.47618 | 12.00683 | 17.40223 | 17.17892 | 18.58931 | 22.7952 | 22.88107 |
| Cln6 | 5.5785 | 5.061083 | 5.436906 | 4.856108 | 4.642101 | 5.184257 | 5.291889 | 6.357217 |
| Tm2d2 | 34.04192 | 30.13159 | 31.51889 | 35.30809 | 36.76501 | 32.31152 | 32.84568 | 33.09651 |
| Klhl28 | 2.173289 | 1.678348 | 2.129476 | 1.65294 | 2.01424 | 1.890419 | 2.122633 | 2.186191 |
| AABR07017 | 0.153117 | 0.173244 | 0.164381 | 0.282182 | 0.165563 | 0.134253 | 0 | 0.81429 |
| Kcnt1 | 0.156483 | 0.126467 | 0.119996 | 0.288386 | 0.290062 | 0.254808 | 0.225085 | 0.356654 |
| Col22a1 | 0.075705 | 0.097893 | 0.174158 | 0.298966 | 0.187105 | 0.256029 | 0.278283 | 0.268403 |
| Lce1l | 0 | 0 | 0 | 0 | 0.16484 | 0 | 0 | 0.067561 |
| Phc2 | 12.74103 | 13.28522 | 12.4714 | 12.20077 | 11.51059 | 12.46129 | 15.12363 | 15.50021 |
| Ap1m2 | 0 | 0 | 0 | 0.037935 | 0 | 0 | 0 | 0.036489 |
| LOC367195 | 0 | 0 | 0.132903 | 0.114074 | 0.133859 | 0.21709 | 0.138498 | 0.329181 |
| Cd274 | 4.937127 | 6.044006 | 5.242394 | 5.369746 | 5.60099 | 5.13315 | 5.855448 | 5.404266 |
| Pop7 | 10.68439 | 10.82377 | 9.603102 | 11.27624 | 11.0827 | 12.03689 | 10.07684 | 11.50728 |
| Csad | 5.40468 | 6.085882 | 6.968276 | 6.481409 | 6.431199 | 6.734125 | 7.03016 | 6.71578 |
| Supt20 | 16.16828 | 18.34826 | 14.68926 | 20.19071 | 19.30906 | 19.99502 | 21.00409 | 19.20748 |
| RGD130472 | 1.654868 | 1.66848 | 1.495164 | 1.826855 | 1.718518 | 1.479722 | 2.089691 | 2.221972 |
| Vps50 | 3.155287 | 3.871754 | 2.465011 | 3.030324 | 3.155484 | 2.714605 | 3.712296 | 2.993638 |
| Unc5b | 7.691274 | 8.280384 | 8.907631 | 10.15403 | 9.566453 | 10.22593 | 12.62016 | 12.88236 |
| Tlcd1 | 1.050319 | 0.962027 | 0.698029 | 1.198263 | 0.932892 | 1.151151 | 1.161062 | 1.451839 |
| Cast | 25.74041 | 27.08026 | 23.5422 | 27.01443 | 26.23113 | 26.24364 | 30.11078 | 29.97171 |
| Prmt5 | 10.43628 | 10.83381 | 10.89585 | 10.69353 | 11.06289 | 10.5348 | 11.05631 | 10.92213 |
| Ifnb1 | 0 | 0 | 0 | 0 | 0 | 0 | 0.105558 | 0 |
| Pcid2 | 5.069373 | 5.695926 | 4.51635 | 4.622569 | 4.66303 | 5.108469 | 5.33662 | 4.72726 |
| AABR07045 | 0 | 0 | 0 | 0 | 0 | 0.203612 | 0 | 0 |
| LOC100365 | 0.665387 | 0.489355 | 0.250017 | 0.429189 | 0.503631 | 0.612582 | 0.409423 | 0.324371 |
| Hddc2 | 6.422607 | 7.266878 | 6.810477 | 7.043723 | 7.541147 | 7.980622 | 8.24329 | 6.880105 |
| Zfp24 | 12.06522 | 11.22794 | 10.42354 | 11.77548 | 10.7687 | 11.45515 | 12.7792 | 11.29515 |
| Krtcap2 | 14.74287 | 16.30176 | 16.03297 | 19.14247 | 19.409 | 17.50123 | 16.97563 | 16.46146 |
| Dtwd2 | 2.351241 | 2.528945 | 1.93211 | 2.99576 | 2.573751 | 2.977824 | 1.688693 | 1.749549 |
| LOC100911 | 0 | 0.087777 | 0.166572 | 0 | 0 | 0 | 0 | 0 |
| Slc7a15 | 2.41855 | 1.856894 | 2.271909 | 1.054605 | 1.190824 | 1.154962 | 1.038821 | 1.22496 |
| Sharpin | 19.5095 | 22.07408 | 20.32766 | 19.47779 | 19.40357 | 20.46554 | 21.39769 | 18.59411 |
| Lrrc40 | 4.8868 | 4.336045 | 4.638827 | 4.739994 | 4.394085 | 4.44261 | 4.575129 | 5.53965 |
| Aldh8a1 | 0.038833 | 0.109843 | 0.083379 | 0.017891 | 0.020995 | 0.034049 | 0.086889 | 0.068839 |
| RGD156374 | 0 | 0 | 0 | 0 | 0 | 0 | 0 | 0 |
| Gpr155 | 3.764238 | 3.66279 | 3.752496 | 4.013662 | 4.116732 | 4.677261 | 5.1979 | 5.433616 |
| AABR07043 | 0 | 0 | 0.540559 | 0 | 0.544447 | 0 | 1.689942 | 0 |
| AABR07055 | 0 | 0 | 0 | 0 | 0 | 0 | 0.221912 | 0 |
| Plekha4 | 3.491061 | 4.307256 | 4.614221 | 9.343488 | 9.162035 | 8.136939 | 9.028123 | 9.267354 |
| Prorsd1 | 3.66053 | 4.77448 | 3.875232 | 5.059552 | 4.397865 | 4.1011 | 3.355821 | 3.064259 |
| Angel2 | 7.79792 | 7.316209 | 6.703607 | 6.61282 | 6.719823 | 7.369152 | 8.624643 | 8.406812 |
| AABR07026 | 2.143634 | 2.252177 | 1.643806 | 1.693092 | 2.317881 | 2.148046 | 2.055602 | 2.442869 |

| | | | | | | | | |
|---|---|---|---|---|---|---|---|---|
| AABR07006 | 0.029189 | 0.02477 | 0.015668 | 0.006724 | 0.023672 | 0.019195 | 0.016328 | 0.03234 |
| LOC102550 | 0 | 0 | 0 | 0 | 0 | 0 | 0 | 0 |
| Coa6 | 40.3654 | 39.00597 | 33.02817 | 27.74555 | 24.30049 | 26.59214 | 29.29233 | 32.29677 |
| Pm20d1 | 2.018479 | 1.700712 | 2.22844 | 1.767611 | 2.074198 | 1.88277 | 2.098029 | 1.586062 |
| LOC100910 | 1.456211 | 1.761827 | 0.882278 | 0.890132 | 1.060113 | 0.859632 | 1.096849 | 1.022346 |
| LOC100360 | 0.076558 | 0 | 0.08219 | 0 | 0 | 0.067126 | 0 | 0.135715 |
| Adam19 | 13.67878 | 14.23272 | 14.46146 | 13.5947 | 13.05218 | 13.86346 | 18.1551 | 18.62192 |
| Adam10 | 16.29777 | 16.85913 | 15.9817 | 13.02898 | 13.05983 | 12.45817 | 15.46264 | 15.50012 |
| Tlr5 | 0.701494 | 0.640536 | 0.488854 | 0.544336 | 0.731901 | 0.690606 | 0.771032 | 0.829025 |
| Thy1 | 1.419558 | 1.472316 | 1.185323 | 5.232268 | 5.031217 | 6.223349 | 5.734945 | 6.08142 |
| AABR07041 | 0 | 0 | 0 | 0 | 0 | 0 | 0.427625 | 0 |
| 5S_rRNA | 0 | 0 | 0.480497 | 0 | 0 | 0 | 0 | 0 |
| Igdcc4 | 13.12125 | 15.1694 | 17.02651 | 10.04999 | 10.09369 | 9.792585 | 12.17517 | 14.01512 |
| Mpst | 33.04604 | 32.37549 | 37.09382 | 28.50781 | 28.33452 | 26.33386 | 28.25735 | 29.13779 |
| Ddx41 | 8.714875 | 7.34475 | 8.450558 | 10.03213 | 8.953487 | 10.98004 | 11.86561 | 10.17087 |
| Mat2a | 67.14554 | 66.71269 | 60.82699 | 68.58868 | 70.45175 | 66.34271 | 76.77043 | 76.73656 |
| Creb1 | 7.25816 | 6.55879 | 7.582915 | 6.867656 | 7.637455 | 7.858851 | 9.046562 | 10.10325 |
| Bdp1 | 7.496496 | 7.260109 | 7.359102 | 7.181728 | 6.261306 | 6.120102 | 8.80696 | 8.295243 |
| #N/A | 2.464279 | 0 | 2.645561 | 2.270736 | 0.666147 | 2.700852 | 2.756925 | 3.822372 |
| Pfkfb2 | 8.674529 | 8.088855 | 7.894985 | 9.797484 | 8.296429 | 9.582153 | 16.58201 | 14.22706 |
| AABR07039 | 0 | 0.564282 | 0 | 0.459554 | 0 | 0 | 0 | 0 |
| Phyh | 812.3579 | 800.9705 | 775.0981 | 704.3605 | 715.1482 | 705.3372 | 697.3396 | 694.4641 |
| Snrpd1 | 12.3362 | 10.75323 | 12.70314 | 11.94729 | 12.79451 | 11.69936 | 14.43492 | 12.44043 |
| AC113756. | 0 | 0 | 0.432447 | 0 | 0 | 0 | 0 | 0 |
| AABR07035 | 0.025937 | 0.029346 | 0 | 0 | 0 | 0.022741 | 0 | 0 |
| Natd1 | 0.471765 | 0.53378 | 0.844117 | 0.434713 | 0.850188 | 0.551525 | 0.52779 | 2.508893 |
| Grik3 | 0.10087 | 0.065217 | 0.06188 | 0.066391 | 0.015581 | 0.037904 | 0.064485 | 0.025545 |
| Slc15a4 | 4.450144 | 4.666175 | 4.942256 | 4.189008 | 3.878538 | 4.372808 | 5.407815 | 5.763561 |
| Pate4 | 0 | 0 | 0 | 0 | 0 | 0 | 0.117287 | 0 |
| Wscd1 | 4.09531 | 3.245908 | 3.459236 | 3.084063 | 3.034553 | 3.554317 | 4.18628 | 3.998392 |
| Zfp775 | 5.738731 | 6.140884 | 6.320729 | 5.986432 | 6.249112 | 5.874301 | 6.980494 | 6.778031 |
| Krt73 | 0 | 0 | 0 | 0 | 0 | 0 | 0 | 0 |
| Dyrk4 | 0 | 0 | 0 | 0 | 0 | 0.048128 | 0 | 0 |
| Irs3 | 0.66488 | 1.083283 | 0.999307 | 0.931244 | 1.092766 | 1.539033 | 1.636443 | 1.34364 |
| Calcoco1 | 2.990374 | 2.897337 | 7.103378 | 6.001949 | 3.54936 | 5.394613 | 8.017659 | 25.11898 |
| BC028528 | 29.2481 | 30.50225 | 29.57599 | 24.637 | 23.6393 | 20.91732 | 26.85475 | 23.17453 |
| Trem2 | 4.480749 | 4.352821 | 4.324474 | 6.464335 | 5.579055 | 6.428822 | 7.747151 | 8.304067 |
| Pcdhga11 | 0.53941 | 0.878857 | 0.741237 | 0.596454 | 0.97987 | 0.567546 | 0.675884 | 0.611976 |
| Ccdc22 | 3.38239 | 3.388984 | 3.456214 | 4.055516 | 3.701394 | 3.555246 | 4.422344 | 3.900987 |
| AC130053. | 1.707584 | 3.220086 | 1.833201 | 2.622453 | 4.923697 | 1.497211 | 3.820739 | 5.045056 |
| Rn50_13_0 | 0.050742 | 0 | 0 | 0 | 0 | 0 | 0.056768 | 0 |
| Olr1308 | 0 | 0 | 0 | 0 | 0 | 0 | 0 | 0 |
| Rhob | 98.15902 | 102.5903 | 109.0762 | 93.8884 | 88.72162 | 87.24808 | 132.7111 | 130.7289 |
| Ddit4l | 22.84419 | 23.19614 | 21.19186 | 14.62707 | 12.7939 | 12.32604 | 10.41942 | 8.358767 |
| Arhgap10 | 7.540853 | 8.079488 | 7.773481 | 8.072908 | 7.115663 | 8.102556 | 8.593014 | 9.502743 |
| Otud7b | 10.46404 | 10.55856 | 11.40569 | 9.484128 | 9.36083 | 10.24778 | 11.91141 | 12.26503 |
| Itga11 | 0.47626 | 0.320753 | 0.438253 | 1.201626 | 1.471352 | 1.282583 | 1.839493 | 1.336754 |
| AABR07042 | 0.342261 | 0 | 0 | 0 | 0 | 0.300095 | 0.382906 | 0.910089 |
| Yars | 8.286428 | 8.206785 | 8.664945 | 8.428928 | 8.983267 | 9.039473 | 9.655755 | 10.68316 |
| Crb1 | 0.045506 | 0.025744 | 0.085494 | 0.020966 | 0.024602 | 0.01995 | 0.012727 | 0.040334 |
| Opa3 | 21.62776 | 23.75694 | 22.70401 | 18.2548 | 20.13851 | 18.96324 | 22.10689 | 22.88292 |
| Pcdhga4 | 0.389821 | 0.563582 | 0.534747 | 0.439028 | 0.234171 | 0.493704 | 0.557257 | 0.691035 |
| Actn2 | 425.1698 | 432.6333 | 422.599 | 349.6975 | 342.454 | 347.5193 | 404.3876 | 403.984 |
| Lrp12 | 1.32211 | 1.833217 | 1.391539 | 1.791577 | 1.541702 | 1.557001 | 1.740139 | 1.711823 |

| | | | | | | | | |
|---|---|---|---|---|---|---|---|---|
| AC110439. | 0 | 0.553734 | 0 | 0 | 0.529182 | 0 | 0.54752 | 0 |
| Slc15a3 | 4.257203 | 3.606038 | 3.571389 | 3.858536 | 3.144298 | 3.141195 | 4.918926 | 4.020805 |
| RGD131085 | 2.483608 | 2.031908 | 2.645802 | 2.640631 | 2.561544 | 2.596405 | 2.415201 | 3.01415 |
| Tdrd5 | 0.094637 | 0.232001 | 0.203198 | 0.145341 | 0.051165 | 0.110637 | 0.123522 | 0.069901 |
| Klf14 | 0 | 0 | 0 | 0 | 0 | 0 | 0 | 0 |
| Zfp496 | 6.056506 | 6.41628 | 6.900756 | 6.804929 | 5.575757 | 5.748704 | 7.718602 | 6.988765 |
| Clec4f | 0 | 0.092193 | 0.058318 | 0.050055 | 0 | 0 | 0 | 0.024074 |
| Prr7 | 2.807245 | 3.29843 | 4.694506 | 5.123787 | 3.969413 | 4.212772 | 5.496087 | 4.785002 |
| Nilr1 | 0.39822 | 0.067585 | 0.085503 | 0.256861 | 0.387531 | 0.384076 | 0.534613 | 0.494147 |
| Rpl6 | 259.7863 | 261.7682 | 268.3681 | 271.3903 | 275.1589 | 266.8364 | 293.4458 | 273.2092 |
| Rps14 | 789.1636 | 783.3677 | 767.3286 | 682.1046 | 705.5463 | 658.9131 | 772.3926 | 770.2164 |
| Rab37 | 0.463788 | 0.28623 | 0.362114 | 0.233107 | 0.455898 | 0.295745 | 0.188678 | 0.224225 |
| #N/A | 6.287994 | 6.490889 | 5.610857 | 5.530769 | 6.136865 | 5.781824 | 5.778527 | 5.736219 |
| Serpina16 | 0 | 0.037739 | 0 | 0 | 0 | 0 | 0 | 0 |
| Pkd1 | 8.977232 | 9.295144 | 9.75566 | 13.54358 | 13.53562 | 13.81457 | 19.98911 | 19.58664 |
| Tsen15 | 20.81405 | 20.55598 | 19.06719 | 20.44545 | 18.37893 | 16.15262 | 19.69902 | 16.1481 |
| Itih2 | 0 | 0 | 0.020064 | 0.017221 | 0 | 0 | 0 | 0 |
| AABR07009 | 0.093846 | 0 | 0 | 0.086475 | 0 | 0 | 0 | 0 |
| AABR07017 | 0.154472 | 0.349555 | 0 | 0 | 0 | 0 | 0 | 0 |
| Numbl | 3.270534 | 3.742746 | 3.631509 | 6.819501 | 5.233844 | 5.62051 | 7.673295 | 6.758431 |
| Atp6v0c | 161.4199 | 167.6078 | 180.914 | 176.7046 | 173.5079 | 171.212 | 186.2901 | 179.9319 |
| AABR07005 | 20.91732 | 19.47594 | 15.67247 | 7.897184 | 5.732931 | 6.495531 | 11.86319 | 10.87941 |
| AABR07058 | 12.39759 | 9.560886 | 10.66092 | 9.94617 | 9.803898 | 12.05999 | 13.17982 | 13.55807 |
| AABR07034 | 0 | 0 | 0 | 0 | 0 | 0 | 0.011427 | 0 |
| Arl2bp | 65.40959 | 65.78477 | 61.10491 | 59.21495 | 63.07475 | 64.19308 | 62.65007 | 63.73944 |
| Smtnl1 | 0.028568 | 0.096971 | 0.06134 | 0.184273 | 0.061781 | 0.075146 | 0.159805 | 0.075965 |
| Knop1 | 0.137443 | 0.466532 | 0.147554 | 0.33773 | 0.198154 | 0.04017 | 0.153766 | 0 |
| Tarsl2 | 13.06409 | 13.63484 | 12.08455 | 10.42288 | 11.28304 | 10.61412 | 12.07236 | 10.89964 |
| Cct6b | 0.177111 | 0.133595 | 0.15845 | 0.163201 | 0.063836 | 0.051764 | 0.198144 | 0.183146 |
| Gnao1 | 8.529394 | 8.991961 | 9.781797 | 14.15643 | 12.86253 | 15.46757 | 18.03694 | 15.72575 |
| Nfyc | 33.40872 | 32.58915 | 30.85012 | 30.13899 | 31.14419 | 33.21411 | 31.9994 | 31.71215 |
| Taf7l | 0 | 0 | 0 | 0 | 0 | 0 | 0 | 0 |
| Mir3561 | 0.480421 | 0 | 0.515763 | 0.442689 | 0.519473 | 0.842468 | 0 | 1.703285 |
| Ryr2 | 86.99003 | 87.27156 | 86.23156 | 64.75796 | 63.89681 | 63.03046 | 81.54764 | 82.83134 |
| Brd1 | 6.935575 | 7.162615 | 7.308361 | 6.02628 | 6.29139 | 6.662702 | 8.904869 | 8.612472 |
| Atp8b1 | 3.049177 | 2.791453 | 2.564697 | 3.386044 | 3.560042 | 3.435208 | 4.402597 | 4.265701 |
| AABR07026 | 0.33541 | 0.237188 | 0.360084 | 0.888569 | 0.498677 | 0.441132 | 0.609768 | 0.445936 |
| Trove2 | 4.240555 | 4.861749 | 3.796276 | 3.712778 | 4.432917 | 3.829295 | 4.271306 | 5.319501 |
| Cryga | 0.170019 | 0.096184 | 0.182526 | 0.078333 | 0.183839 | 0.149073 | 0 | 0 |
| Rbfox1 | 29.24711 | 30.3844 | 31.12143 | 21.66791 | 23.025 | 23.19874 | 22.45553 | 22.01714 |
| Jak1 | 29.72812 | 29.36934 | 26.84479 | 27.29209 | 26.83994 | 26.36525 | 29.83162 | 29.65943 |
| Suz12 | 8.109661 | 6.847941 | 6.651669 | 7.340472 | 6.699512 | 6.271554 | 7.306356 | 7.845305 |
| Sirt7 | 4.935639 | 5.312031 | 5.9126 | 6.516946 | 6.248001 | 6.095544 | 7.609273 | 7.015528 |
| AABR07073 | 1.589643 | 1.090768 | 1.442338 | 1.086782 | 1.253103 | 1.276901 | 1.112948 | 1.136274 |
| Slc45a3 | 1.094309 | 1.165327 | 1.105704 | 1.038021 | 1.078855 | 1.199364 | 1.29628 | 1.126843 |
| Bicd2 | 22.15407 | 23.29294 | 23.12315 | 21.41532 | 20.96052 | 21.86966 | 26.86117 | 26.88906 |
| Smim22 | 0.243941 | 0.276007 | 0.436476 | 0.374636 | 0.439616 | 0.142592 | 0.27291 | 0 |
| Sema6c | 1.566084 | 2.011901 | 2.224208 | 1.818888 | 2.028532 | 1.616304 | 2.226582 | 2.082147 |
| Sbk3 | 0.865132 | 0.633377 | 0.546338 | 0.468932 | 0.440214 | 0.356964 | 0.341602 | 0.315745 |
| Rad23a | 46.50347 | 46.11786 | 48.77627 | 35.2591 | 36.92028 | 31.77894 | 44.35949 | 42.02585 |
| AABR07066 | 0.094353 | 0.106756 | 0.101294 | 0.260828 | 0 | 0.330915 | 0.422232 | 0.585408 |
| AABR07043 | 0.056612 | 0 | 0.060776 | 0 | 0.244854 | 0 | 0 | 0 |
| Ddx23 | 16.96455 | 20.04642 | 19.02078 | 19.27042 | 18.97668 | 19.96473 | 20.83218 | 21.04224 |
| Ttc22 | 0 | 0.029654 | 0 | 0.072452 | 0.01417 | 0.03447 | 0 | 0.02323 |

| | | | | | | | | |
|---|---|---|---|---|---|---|---|---|
| Htatsf1 | 9.913776 | 8.969831 | 9.043946 | 11.72777 | 11.50704 | 11.27546 | 12.33166 | 12.9679 |
| Aco2 | 889.8247 | 906.3884 | 926.6381 | 900.007 | 883.5926 | 911.6431 | 824.4468 | 821.8896 |
| Akr1c19 | 5.874258 | 5.334648 | 5.476603 | 7.0154 | 5.641357 | 7.895258 | 4.842423 | 5.001121 |
| Dr1 | 0.435367 | 0.236447 | 0.803918 | 0.513502 | 0.376605 | 0.38173 | 0.526035 | 0.555678 |
| Mrps7 | 49.79227 | 50.75139 | 48.26755 | 52.99617 | 52.41984 | 53.74343 | 52.70857 | 51.48892 |
| Otoa | 0.015339 | 0.03471 | 0.032934 | 0.098937 | 0.016585 | 0.134489 | 0.188761 | 0.054381 |
| Atp5g3 | 1271.569 | 1284.72 | 1250.97 | 1215.368 | 1206.933 | 1174.614 | 1025.958 | 1011.402 |
| AABR07044 | 0.489401 | 0.553734 | 0 | 0.901928 | 0 | 0.429107 | 0 | 0.433781 |
| AABR07058 | 0.643316 | 1.164611 | 1.381282 | 0.355674 | 0.417365 | 0.902496 | 1.151541 | 0.570203 |
| Tmem64 | 11.71343 | 13.14925 | 17.45722 | 13.3331 | 11.52318 | 10.14952 | 11.97388 | 12.90649 |
| Nelfcd | 17.39543 | 15.78634 | 16.61795 | 14.37386 | 15.24826 | 14.9373 | 15.87715 | 16.34725 |
| AC139605. | 0 | 0 | 0 | 0.135163 | 0.158606 | 0 | 0 | 0.130013 |
| Ppm1b | 43.14671 | 43.31123 | 42.82234 | 43.93905 | 44.46841 | 45.35389 | 45.89401 | 48.57163 |
| Sltm | 10.73417 | 10.19245 | 10.37897 | 11.35216 | 11.5005 | 11.3679 | 11.77345 | 12.07623 |
| Btnl10 | 0.077065 | 0 | 0.041367 | 0.106519 | 0.208324 | 0.033785 | 0.301761 | 0.068307 |
| Mir98 | 0 | 0 | 0.520539 | 0 | 0 | 0 | 0 | 0 |
| Bcl9l | 7.95896 | 9.036312 | 9.627274 | 7.283173 | 6.950163 | 7.726111 | 9.632464 | 9.435695 |
| Spata2 | 4.583115 | 4.689351 | 4.755473 | 4.142334 | 3.936071 | 4.287659 | 5.078319 | 5.442238 |
| Nt5c | 3.482133 | 2.424535 | 3.450731 | 2.653305 | 2.823885 | 2.642138 | 3.071575 | 2.374144 |
| Col1a1 | 45.75838 | 49.56621 | 53.52548 | 132.5491 | 120.7493 | 127.8354 | 147.9854 | 152.6141 |
| AABR07053 | 0.463415 | 1.572998 | 0 | 0.427019 | 0.334056 | 0.541764 | 0.345632 | 0.410748 |
| AABR07072 | 0 | 0.553734 | 0 | 0 | 0 | 0 | 1.09504 | 0 |
| Ifi44l | 6.054318 | 5.515726 | 5.36014 | 7.679928 | 8.544388 | 8.720995 | 11.61138 | 14.53067 |
| Ccdc189 | 0.597865 | 0.835622 | 0.641846 | 0.518502 | 0.722517 | 0.740059 | 0.826244 | 0.436402 |
| Ypel2 | 4.690207 | 4.3767 | 4.334457 | 7.708026 | 8.313 | 7.313249 | 10.49439 | 12.17149 |
| AABR07037 | 0 | 0 | 0 | 0.043354 | 0 | 0 | 0 | 0 |
| Spic | 0.185695 | 0.105052 | 0.099678 | 0.256666 | 0.200789 | 0.366339 | 0.207747 | 0.16459 |
| Usp28 | 15.64209 | 15.85472 | 16.65823 | 15.24513 | 15.49055 | 14.50625 | 14.96167 | 15.18637 |
| Prkdc | 2.213684 | 2.031573 | 2.094868 | 2.62155 | 2.553199 | 2.505276 | 2.128017 | 2.339984 |
| Spock2 | 4.26139 | 3.56981 | 3.387166 | 3.775676 | 3.854584 | 3.700463 | 6.005157 | 5.88353 |
| E2f7 | 0.497391 | 0.510014 | 0.367112 | 0.286454 | 0.252104 | 0.245313 | 0.278229 | 0.468416 |
| Tst | 29.27317 | 28.83877 | 32.19814 | 28.56338 | 25.33249 | 28.10319 | 26.21034 | 22.9737 |
| Mre11a | 5.112419 | 4.818556 | 4.582436 | 4.737711 | 4.625885 | 4.958901 | 5.100925 | 5.399836 |
| Bloc1s1 | 83.90014 | 92.65855 | 82.89101 | 82.59387 | 81.93732 | 75.23943 | 73.97918 | 77.49869 |
| Fgf7 | 2.191359 | 1.624447 | 2.352564 | 1.114069 | 0.980476 | 1.192584 | 2.197982 | 1.741382 |
| Mapk4 | 0 | 0.038759 | 0 | 0.010522 | 0 | 0.020024 | 0 | 0 |
| AABR07073 | 0 | 0.044382 | 0 | 0.036145 | 0 | 0 | 0 | 0.13907 |
| Gnmt | 0 | 0 | 0 | 0 | 0 | 0.044234 | 0.05644 | 0 |
| Pacs1 | 8.455481 | 8.848429 | 10.59691 | 7.925151 | 7.828268 | 7.50923 | 9.459617 | 10.5663 |
| Mrps24 | 53.92134 | 56.31643 | 60.3232 | 54.10562 | 52.97849 | 55.68836 | 50.60903 | 51.81423 |
| Hfe2 | 72.29365 | 75.27885 | 79.15135 | 50.91868 | 48.70926 | 53.2634 | 58.30806 | 57.67848 |
| Als2cr12 | 0.141759 | 0.096236 | 0.152188 | 0.078375 | 0.183939 | 0.24859 | 0.190313 | 0.100519 |
| Hyal3 | 3.251845 | 3.298692 | 2.40763 | 3.030889 | 2.869521 | 2.818448 | 3.052591 | 4.008677 |
| Trpv6 | 0.089883 | 0.02034 | 0 | 0.099389 | 0.058314 | 0.047286 | 0.060334 | 0.047801 |
| Rn50_20_( | 0.491909 | 0.050597 | 0.240043 | 0.659308 | 0.580248 | 0.352887 | 0.700414 | 0.277457 |
| Cd8b | 0.606005 | 0.685666 | 0.229618 | 0.229933 | 0.308359 | 0.593857 | 0.518448 | 0.47394 |
| Bloc1s5 | 12.60661 | 12.86733 | 12.61912 | 14.73047 | 14.42571 | 14.14537 | 13.34757 | 12.68455 |
| Rpl7 | 95.13498 | 94.49335 | 92.97661 | 84.27203 | 85.35698 | 84.00274 | 91.04451 | 90.73854 |
| Ces5a | 0 | 0 | 0.137034 | 0.058809 | 0 | 0 | 0 | 0.028284 |
| Art2b | 0.146137 | 0.110232 | 0.209184 | 0.044887 | 0 | 0.042711 | 0.272487 | 0.086353 |
| Fbxo5 | 0.397381 | 0.524554 | 0.474015 | 0.874741 | 0.78775 | 0.716204 | 0.716254 | 0.743572 |
| Rtn3 | 20.79013 | 18.88667 | 18.72958 | 20.63481 | 21.85768 | 19.82698 | 21.18922 | 21.26978 |
| Ecm1 | 39.12089 | 38.3113 | 40.82339 | 61.93429 | 57.86411 | 59.63498 | 74.86648 | 74.29637 |
| Usp33 | 9.946214 | 8.631458 | 8.604518 | 9.902316 | 10.06854 | 10.4626 | 11.55951 | 12.83858 |

| | | | | | | | | |
|---|---|---|---|---|---|---|---|---|
| Acox2 | 0.041977 | 0.071242 | 0.135194 | 0.193399 | 0.20425 | 0.055208 | 0.046962 | 0.111618 |
| Lmtk2 | 5.696322 | 5.893433 | 6.131984 | 5.049249 | 4.820362 | 5.001327 | 6.329499 | 6.729625 |
| Afm | 0 | 0 | 0 | 0 | 0 | 0.022764 | 0.029045 | 0 |
| AABR07019 | 0 | 0 | 0 | 0 | 0.069646 | 0 | 0 | 0 |
| Cask | 0.048894 | 0 | 0.262456 | 0 | 0.052869 | 0.085741 | 0 | 0 |
| Il9r | 0.095752 | 0.048151 | 0.114218 | 0.058821 | 0.069024 | 0.055971 | 0.023805 | 0.07544 |
| Pofut2 | 22.76376 | 21.97926 | 24.56278 | 24.05181 | 22.78436 | 23.59748 | 26.78971 | 28.0665 |
| AABR07013 | 3.687741 | 4.172505 | 3.959025 | 5.436973 | 2.392501 | 3.880097 | 5.775953 | 0.653726 |
| Chm | 4.260278 | 4.039237 | 3.980796 | 3.871155 | 3.881468 | 3.579773 | 3.707052 | 3.863506 |
| AABR07033 | 0.168109 | 0.047552 | 0.045119 | 0.038726 | 0.13633 | 0.073699 | 0.188073 | 0.111752 |
| Abhd15 | 1.064898 | 1.587384 | 1.742073 | 1.059139 | 0.877302 | 0.815138 | 1.304823 | 1.258496 |
| Serpina10 | 0 | 0.078114 | 0 | 0 | 0 | 0 | 0 | 0 |
| Rtf1 | 6.93164 | 6.116085 | 6.028288 | 6.451643 | 6.44955 | 6.588397 | 7.298646 | 7.434584 |
| RGD130650 | 8.335399 | 7.719246 | 7.068629 | 7.874375 | 7.846565 | 7.24707 | 7.413201 | 7.90959 |
| Adora3 | 0 | 0 | 0.031888 | 0 | 0 | 0 | 0.03323 | 0 |
| LOC102548 | 0 | 0 | 0 | 0 | 0.009171 | 0 | 0.028467 | 0.007518 |
| AABR07051 | 2.181913 | 0.617183 | 1.756818 | 0.502637 | 0.589818 | 1.913104 | 3.661542 | 1.450454 |
| Rdh14 | 13.70218 | 14.64561 | 12.41924 | 12.47078 | 12.3264 | 13.78666 | 13.31898 | 13.1653 |
| Mkln1 | 14.29242 | 14.2608 | 13.68435 | 15.88716 | 13.85992 | 14.47078 | 15.13836 | 15.04994 |
| AABR07062 | 1.07971 | 0.61082 | 0.579569 | 0.99491 | 0 | 0.94669 | 0 | 2.871001 |
| AABR07017 | 0.283059 | 0 | 0 | 0 | 0 | 0 | 0.633348 | 0 |
| AABR07028 | 0.030913 | 0 | 0 | 0.009495 | 0.011142 | 0.018069 | 0.011528 | 0.009133 |
| Cfap161 | 0.037646 | 0 | 0 | 0.03469 | 0.162825 | 0.033008 | 0.168468 | 0 |
| Plscr3 | 31.5437 | 34.54097 | 33.86417 | 34.16197 | 29.71462 | 32.15993 | 33.26952 | 32.66022 |
| Slc12a5 | 0.030931 | 0.157486 | 0.199237 | 0.128257 | 0.20067 | 0.216962 | 0.121114 | 0.150785 |
| Chd5 | 0 | 0 | 0.008514 | 0 | 0 | 0.006954 | 0 | 0.007029 |
| Sdhaf2 | 54.3713 | 57.52386 | 56.70332 | 56.42546 | 50.60599 | 50.89227 | 49.51573 | 48.74275 |
| Slc6a9 | 0.73032 | 1.161318 | 0.741664 | 1.455051 | 1.259227 | 0.899945 | 0.772885 | 1.137182 |
| Atf2 | 7.87715 | 7.401353 | 6.471155 | 8.804856 | 8.998869 | 8.678408 | 8.582711 | 9.228263 |
| Zmat4 | 0.051114 | 0.043375 | 0.013718 | 0 | 0 | 0 | 0.014296 | 0.022652 |
| C1qc | 40.93189 | 43.2685 | 41.51892 | 62.81761 | 60.46661 | 65.33336 | 89.22068 | 88.05984 |
| Apopt1 | 1.274914 | 1.015096 | 1.115239 | 12.66155 | 1.021145 | 3.726153 | 0.581092 | 6.821972 |
| Csf3r | 0.345101 | 0.361542 | 0.576315 | 0.694883 | 0.58046 | 0.96379 | 0.686371 | 0.736379 |
| Bach1 | 1.520101 | 1.895425 | 3.780072 | 2.029605 | 2.146825 | 2.393646 | 2.550932 | 4.179506 |
| Zfp444 | 7.925931 | 8.251167 | 8.527371 | 7.540045 | 7.348525 | 7.159598 | 7.967055 | 7.981051 |
| Dnmt3b | 0.627387 | 0.591549 | 1.055213 | 0.770817 | 0.814062 | 0.916823 | 1.123029 | 1.186314 |
| Cops9 | 184.897 | 189.6962 | 188.782 | 192.2183 | 193.5185 | 190.838 | 177.2005 | 181.2649 |
| Pgpep1 | 10.51701 | 9.632926 | 10.58501 | 10.0948 | 10.52576 | 10.29165 | 10.43528 | 11.95736 |
| Cnn1 | 2.967402 | 2.606981 | 2.660993 | 6.916283 | 7.323178 | 7.621805 | 5.389789 | 5.662571 |
| AABR07004 | 4.038503 | 1.980063 | 3.323953 | 2.356837 | 3.202301 | 2.360642 | 4.367494 | 4.892019 |
| Tmco6 | 4.378041 | 5.822027 | 5.371551 | 5.370191 | 5.717583 | 4.935433 | 5.375032 | 6.223879 |
| Ccdc183 | 0.059271 | 0.100594 | 0.031816 | 0.245771 | 0.032044 | 0.207875 | 0.198929 | 0.183872 |
| AABR07014 | 0.337845 | 0 | 0 | 0 | 0 | 0 | 0 | 0 |
| Atp13a1 | 6.724693 | 6.761614 | 7.868005 | 8.302395 | 7.313919 | 7.658172 | 9.271865 | 10.22121 |
| Slc25a3 | 1176.565 | 1194.691 | 1160.968 | 1110.68 | 1088.662 | 1086.779 | 1013.471 | 1021.979 |
| Nudt4 | 89.59118 | 85.55347 | 84.42331 | 53.18399 | 52.81027 | 54.0272 | 55.14086 | 54.71076 |
| Zfp800 | 2.321422 | 2.458209 | 2.332439 | 2.125387 | 2.107857 | 2.100663 | 2.780233 | 2.901732 |
| AC141344. | 0.232737 | 0 | 0 | 0 | 0 | 0 | 0 | 0 |
| Fam84a | 0.060208 | 0.017031 | 0.032319 | 0.041609 | 0.016276 | 0 | 0.01684 | 0.013341 |
| Hoxc11 | 0 | 0 | 0 | 0 | 0.061883 | 0 | 0 | 0 |
| LOC103694 | 26.93557 | 27.96652 | 29.08716 | 26.06107 | 22.35775 | 17.15715 | 15.95346 | 26.89374 |
| Tex35 | 0.119968 | 0 | 0 | 0.055273 | 0.129719 | 0.105188 | 0.134215 | 0 |
| Sars2 | 7.204346 | 7.762059 | 7.826676 | 7.054668 | 6.441247 | 6.071649 | 6.784754 | 6.766798 |
| AABR07030 | 0 | 0.094047 | 0 | 0 | 0 | 0.21864 | 0 | 0 |

| | | | | | | | | |
|---|---|---|---|---|---|---|---|---|
| AABR07026 | 0.087862 | 0.07953 | 0.094326 | 0.080962 | 0.076003 | 0 | 0.019659 | 0.062301 |
| Mog | 0.199236 | 0.150284 | 0.178244 | 0.214186 | 0.143621 | 0.291151 | 0.371494 | 0.206025 |
| Tbc1d14 | 8.941816 | 9.532692 | 8.333884 | 8.886487 | 9.253262 | 9.210773 | 10.47796 | 10.40305 |
| AABR07002 | 0.18374 | 0.207893 | 0.197257 | 0 | 0.198675 | 0.161103 | 0.20556 | 0.325716 |
| AABR07002 | 0 | 0 | 0.233917 | 0 | 0 | 0.063682 | 0.081255 | 0.12875 |
| Hmmr | 0.917493 | 0.986195 | 1.132736 | 0.824298 | 1.091279 | 1.146354 | 1.180418 | 1.301152 |
| Psmb7 | 66.17561 | 81.43253 | 63.84738 | 57.12354 | 56.8405 | 62.79546 | 65.57648 | 59.63752 |
| Osbpl6 | 11.56406 | 11.309 | 11.34469 | 7.602803 | 8.502358 | 8.553333 | 7.929683 | 8.057508 |
| AABR07049 | 0.698212 | 0 | 0 | 0 | 0 | 0 | 0 | 0 |
| Tex9 | 3.134357 | 2.794115 | 2.787116 | 4.05513 | 3.594537 | 3.136842 | 4.392079 | 4.321545 |
| Nrxn1 | 1.314126 | 1.576985 | 1.303919 | 1.944803 | 1.786946 | 1.64105 | 2.717616 | 1.95894 |
| Smpd4 | 7.270515 | 6.989733 | 7.691296 | 6.937262 | 6.335156 | 7.466095 | 7.369781 | 7.116893 |
| AABR07042 | 0 | 0.548607 | 0 | 0 | 1.572848 | 0.425134 | 1.084901 | 0.859528 |
| Prss33 | 0 | 0 | 0 | 0 | 0 | 0 | 0 | 0.034924 |
| Ppp2r5a | 26.76794 | 26.29257 | 27.82756 | 26.39424 | 26.93714 | 27.16666 | 30.12732 | 29.16105 |
| Galnt18 | 14.55305 | 15.16676 | 14.45802 | 11.1013 | 11.13034 | 11.35047 | 11.91315 | 10.49323 |
| Atp8b3 | 0 | 0 | 0 | 0 | 0.012945 | 0.010497 | 0 | 0 |
| AABR07018 | 0 | 0.400335 | 0 | 0 | 0 | 0 | 0 | 0.313612 |
| Nt5c3a | 17.83761 | 16.86991 | 17.36769 | 15.1017 | 14.75123 | 14.18453 | 13.97928 | 14.41927 |
| Spdya | 0.825066 | 1.283595 | 0.885761 | 0.950333 | 0.780616 | 1.107735 | 1.009583 | 0.914121 |
| Ficd | 3.747467 | 3.905339 | 3.634947 | 3.362271 | 3.074606 | 3.6893 | 3.879901 | 4.079116 |
| Dlgap2 | 0 | 0 | 0 | 0 | 0 | 0 | 0 | 0 |
| Krt26 | 0 | 0 | 0 | 0 | 0 | 0.046614 | 0 | 0 |
| Slitrk5 | 0 | 0 | 0 | 0.010007 | 0 | 0.009522 | 0 | 0 |
| Zbtb49 | 1.740431 | 1.687899 | 1.971126 | 1.374633 | 1.71646 | 1.710474 | 2.482038 | 2.034237 |
| AC119459. | 0 | 0 | 0 | 0 | 0 | 0.395815 | 0 | 0 |
| Furin | 21.56533 | 22.13522 | 24.89209 | 18.03834 | 16.78491 | 17.71326 | 21.41968 | 22.30379 |
| Derl2 | 9.829935 | 8.764427 | 9.87222 | 11.43716 | 12.44128 | 11.12116 | 11.25069 | 14.13314 |
| Il20rb | 0.662261 | 0.374659 | 0.863332 | 1.002549 | 1.534485 | 0.995436 | 1.32305 | 1.17399 |
| AABR07036 | 0.242435 | 1.097214 | 0.390404 | 0.223394 | 0.131071 | 0.637701 | 0.406838 | 1.07441 |
| Tra2b | 22.48778 | 22.93532 | 22.07356 | 26.07226 | 24.88651 | 23.28124 | 27.8159 | 28.40082 |
| AABR07033 | 1.108273 | 0.62698 | 0.892352 | 1.787153 | 0.59918 | 0.728801 | 0.619944 | 0.736738 |
| Arf6 | 34.83452 | 40.18847 | 43.03361 | 39.88141 | 32.4333 | 35.74685 | 44.13004 | 46.13126 |
| Igdcc3 | 0.107351 | 0.072878 | 0.115248 | 0.118704 | 0.046431 | 0.131776 | 0.04804 | 0.266422 |
| Nme6 | 6.650151 | 5.561463 | 5.794265 | 5.565392 | 6.461219 | 5.380164 | 5.499051 | 5.922834 |
| Txndc11 | 4.686179 | 5.032261 | 4.829676 | 4.333832 | 5.159227 | 4.826026 | 6.634385 | 6.947829 |
| AABR07048 | 0 | 1.462952 | 1.388103 | 0 | 0 | 1.133691 | 0 | 0 |
| Rpl6-ps1 | 19.49675 | 17.16458 | 17.79438 | 18.89742 | 19.68422 | 19.85358 | 17.78912 | 19.62159 |
| Tex13b | 0.021022 | 0.023785 | 0 | 0 | 0 | 0 | 0 | 0 |
| AABR07069 | 0 | 0.018954 | 0.035968 | 0.015436 | 0 | 0 | 0 | 0.014848 |
| S100a3 | 0.601139 | 0.453441 | 0.717068 | 0.92321 | 0.650003 | 0.93703 | 0.971429 | 0.473618 |
| Sec11c | 42.30547 | 42.32113 | 41.74822 | 44.27166 | 42.25769 | 39.016 | 45.23717 | 43.27068 |
| Ankrd54 | 4.159799 | 4.867986 | 5.001707 | 5.12539 | 4.934872 | 4.439303 | 5.823896 | 5.646433 |
| AABR07043 | 0.086842 | 0.098258 | 0 | 0.080022 | 0.093901 | 0.22843 | 0.194311 | 0.230918 |
| LOC100910 | 0.22748 | 0.051477 | 0.073264 | 0.230575 | 0.122985 | 0.059836 | 0.254495 | 0.020163 |
| LOC691519 | 0 | 0 | 0 | 0 | 0 | 0 | 0 | 0 |
| Msantd1 | 0.084837 | 0.083991 | 0.068309 | 0.097718 | 0.1032 | 0.037193 | 0.094912 | 0.17859 |
| Socs7 | 4.560123 | 5.808782 | 6.87327 | 5.175942 | 4.343019 | 4.819167 | 7.73696 | 7.120105 |
| Tex19.2 | 0 | 0 | 0 | 0 | 0.026509 | 0 | 0 | 0 |
| Trip11 | 5.37585 | 4.932733 | 4.693826 | 4.606822 | 4.693673 | 4.532048 | 5.22125 | 5.643356 |
| Stk24 | 12.5512 | 14.53025 | 13.20681 | 12.56113 | 12.67266 | 11.1871 | 15.71557 | 13.81384 |
| AABR07058 | 0.997446 | 1.128563 | 0 | 0.459554 | 0.539262 | 0.437281 | 0.557949 | 0 |
| Defb28 | 0.202969 | 0 | 0 | 0.187028 | 0 | 0 | 0 | 0.179901 |
| Pcp4l1 | 17.03251 | 18.17807 | 16.21054 | 20.20287 | 17.89454 | 19.96512 | 19.25794 | 19.05833 |

| | | | | | | | | |
|---|---|---|---|---|---|---|---|---|
| Nmrk1 | 0.837607 | 0.666909 | 1.132356 | 1.457885 | 1.140501 | 0.680013 | 1.318849 | 1.264851 |
| Trmt61a | 0.130751 | 0.07397 | 0.736942 | 0.120482 | 0.07069 | 0.257947 | 0.109709 | 0.144864 |
| Enpp5 | 4.272278 | 4.419549 | 4.691399 | 4.499127 | 4.962719 | 4.409503 | 4.424576 | 4.868656 |
| #N/A | 0 | 0.237315 | 0 | 0.064423 | 0 | 0 | 0 | 0 |
| AABR07016 | 2.317564 | 2.622215 | 2.014139 | 2.898239 | 2.028625 | 2.128806 | 2.531055 | 2.298717 |
| AABR07056 | 0 | 0 | 0 | 0 | 0 | 0 | 0 | 0 |
| Vps28 | 63.94324 | 58.94635 | 63.1807 | 62.37841 | 61.78055 | 64.0547 | 61.343 | 58.29131 |
| Ifit1 | 6.330503 | 5.386325 | 6.00784 | 14.74661 | 14.97704 | 15.36403 | 23.82481 | 22.75838 |
| Btg1 | 31.16166 | 29.3411 | 27.91682 | 35.44724 | 33.15244 | 34.13751 | 31.81684 | 33.01717 |
| Vamp7 | 13.18414 | 14.07851 | 14.60876 | 13.9051 | 13.7978 | 14.80963 | 14.57212 | 12.24893 |
| Obscn | 37.47599 | 40.5397 | 42.04455 | 32.80331 | 31.96303 | 33.5702 | 42.45685 | 42.76381 |
| Camsap2 | 6.676676 | 6.72568 | 5.988436 | 6.66237 | 6.731348 | 6.19013 | 7.898307 | 7.352048 |
| Abcc5 | 13.67788 | 12.17502 | 12.68529 | 14.11661 | 14.09222 | 13.31674 | 16.33548 | 15.68374 |
| Me3 | 55.97171 | 57.82783 | 57.03929 | 55.72897 | 57.18372 | 55.83029 | 49.47761 | 50.62742 |
| Pafah1b2 | 16.73357 | 17.84444 | 16.93145 | 16.6509 | 17.66021 | 17.43766 | 19.28898 | 19.64147 |
| LOC500354 | 0 | 0 | 0 | 0 | 0 | 0 | 0 | 0.033106 |
| Myl4 | 4.413229 | 4.844305 | 4.667168 | 31.68319 | 28.34687 | 27.6642 | 19.45453 | 17.5733 |
| F2rl1 | 2.781114 | 2.230572 | 3.420332 | 2.46537 | 2.645556 | 2.561951 | 3.485541 | 3.931583 |
| Mex3c | 5.688051 | 5.900985 | 5.546579 | 5.406514 | 6.344259 | 6.073345 | 6.345305 | 6.6162 |
| AC098750. | 0.281034 | 0.211984 | 0.201138 | 0.172641 | 0.202585 | 0.328547 | 0.104803 | 0.249094 |
| Grxcr1 | 0 | 0 | 0.052199 | 0 | 0 | 0 | 0 | 0.086192 |
| Rn50_2_22 | 0 | 0.147021 | 0.278998 | 0 | 0 | 0.113932 | 0.436114 | 0.575863 |
| Dkkl1 | 0 | 0 | 0 | 0 | 0 | 0 | 0 | 0.040714 |
| Rpl18a | 392.7444 | 396.1308 | 409.2376 | 412.1926 | 400.8908 | 382.3664 | 408.1457 | 407.354 |
| Znhit1 | 19.93031 | 19.45508 | 20.73719 | 22.63473 | 21.97292 | 19.38394 | 24.67051 | 21.6733 |
| AABR07024 | 0.881157 | 1.139415 | 0.27028 | 0.579966 | 0.680559 | 1.214085 | 0.704143 | 0.781014 |
| rnf141 | 18.58319 | 18.23064 | 17.06726 | 16.03489 | 13.99592 | 14.83391 | 15.56249 | 17.89934 |
| AABR07044 | 2.581225 | 2.60984 | 3.036429 | 2.47971 | 2.048743 | 1.781684 | 2.058297 | 2.579936 |
| Tsga10 | 1.198424 | 1.562302 | 1.258616 | 1.968536 | 1.493031 | 1.324896 | 1.894529 | 1.939711 |
| #N/A | 0.283059 | 0 | 0.151941 | 0.130414 | 0 | 0 | 0.316674 | 0 |
| Cpsf2 | 4.460504 | 4.718692 | 4.241057 | 4.598609 | 4.509471 | 4.016202 | 4.632172 | 4.715914 |
| Bcl2l1-ps | 0.306233 | 0 | 0.328761 | 0.094061 | 0.220751 | 0.537012 | 0.1142 | 0.633337 |
| AABR07022 | 0 | 0 | 0 | 0.595718 | 0 | 0 | 0 | 0 |
| RGD130622 | 0.59222 | 0.41235 | 0.611333 | 0.398786 | 0.837393 | 0.699003 | 0.560619 | 0.888316 |
| AABR07005 | 0 | 1.356861 | 0 | 2.946756 | 3.02563 | 3.15443 | 2.683267 | 2.480165 |
| Gadd45g | 33.2932 | 31.85344 | 36.15498 | 45.55273 | 44.5188 | 42.1655 | 55.03734 | 53.95155 |
| AABR07009 | 0.133928 | 0 | 0.287561 | 0 | 0.434444 | 0 | 0 | 0 |
| RGD156417 | 1.646727 | 2.701632 | 2.6518 | 2.807179 | 3.02699 | 2.887703 | 3.408227 | 3.794898 |
| Dusp22 | 26.31862 | 23.74076 | 23.6427 | 21.95976 | 23.81275 | 22.75899 | 19.98354 | 23.08701 |
| RGD155944 | 0 | 0 | 0 | 0 | 0 | 0 | 0 | 0.009628 |
| Ckap2 | 0.657399 | 0.441641 | 0.639595 | 0.662558 | 0.688622 | 0.594421 | 0.620552 | 0.564476 |
| AABR07044 | 0 | 0 | 0 | 0 | 0 | 0 | 0 | 0 |
| Scgn | 0.56714 | 0.427795 | 0.202954 | 0.348398 | 0.340689 | 0.221008 | 0.634491 | 0.44683 |
| Sec14l1 | 30.96451 | 30.50971 | 32.15465 | 29.71188 | 28.09983 | 29.11346 | 38.43997 | 38.71028 |
| AC103574. | 0 | 0 | 0.1701 | 0 | 0.085662 | 0.069462 | 0 | 0.070219 |
| Tmem11 | 29.40458 | 30.92819 | 31.06462 | 24.32447 | 24.15218 | 26.6037 | 30.6248 | 27.48182 |
| Tmem201 | 7.818283 | 9.424293 | 10.7513 | 7.331524 | 6.601728 | 7.133641 | 8.824016 | 9.304942 |
| Rfng | 14.67709 | 13.66236 | 13.8363 | 13.0748 | 12.22132 | 12.76195 | 12.46289 | 13.83787 |
| Fam20b | 10.14972 | 10.55724 | 10.39552 | 9.877993 | 9.528635 | 9.953744 | 10.9491 | 11.33944 |
| AABR07072 | 0.45763 | 0.295878 | 0.350925 | 0.301206 | 0.282759 | 0.458572 | 0.365697 | 0.289729 |
| AC134651. | 0 | 0.387252 | 0 | 0 | 0.185041 | 0 | 0.191453 | 0 |
| Tecr | 415.2125 | 428.2681 | 455.6074 | 473.9118 | 448.3825 | 460.0082 | 415.4003 | 417.4926 |
| Spag8 | 0.784391 | 0.461501 | 0.53894 | 0.838431 | 0.50889 | 0.385142 | 0.702033 | 0.750864 |
| Spata25 | 1.124726 | 1.12286 | 0.816815 | 0.792534 | 1.144612 | 0.783128 | 1.07325 | 0.645053 |

| | | | | | | | | |
|---|---|---|---|---|---|---|---|---|
| Zfp799 | 2.51734 | 2.165858 | 1.970592 | 1.981351 | 2.296657 | 1.701388 | 1.994871 | 1.882612 |
| Lysmd2 | 11.04766 | 11.33325 | 11.14875 | 12.69105 | 13.77735 | 12.88318 | 14.33718 | 13.77421 |
| AABR07069 | 0 | 0.042934 | 0 | 0 | 0 | 0.033271 | 0 | 0.067267 |
| Sh3glb2 | 24.72746 | 27.32647 | 28.71007 | 20.66307 | 19.5429 | 20.86767 | 24.87254 | 25.8016 |
| LOC685048 | 0.046755 | 0.105803 | 0.351364 | 0.129249 | 0 | 0 | 0 | 0.248649 |
| Plk1 | 0.608634 | 0.34432 | 0.47749 | 0.496121 | 0.329053 | 0.431026 | 0.864235 | 0.58096 |
| Polr3g | 0.691507 | 0.604588 | 0.53991 | 0.477897 | 0.560787 | 0.592534 | 0.808792 | 0.571127 |
| Ubap1 | 8.144927 | 7.192665 | 6.654046 | 6.498431 | 6.809308 | 6.793114 | 7.712018 | 8.751144 |
| AABR07060 | 29.96265 | 27.99193 | 31.13394 | 30.77562 | 26.45356 | 27.71741 | 25.06378 | 28.01926 |
| Slc22a23 | 5.152423 | 5.313504 | 5.700354 | 3.160338 | 3.11309 | 3.276158 | 3.713794 | 3.10964 |
| Actn3 | 0.163814 | 0.14416 | 0.136784 | 0.218036 | 0.255854 | 0.159592 | 0.223994 | 0.145197 |
| Pnp | 43.84552 | 44.60654 | 46.13153 | 61.19703 | 63.9929 | 64.24797 | 74.29999 | 71.52 |
| Lrrc75b | 11.34803 | 12.18808 | 11.4308 | 9.323584 | 8.971403 | 8.066427 | 9.125554 | 8.526805 |
| Dlg5 | 2.601312 | 2.245375 | 2.7159 | 2.042809 | 2.078156 | 2.343536 | 2.920233 | 2.781068 |
| Fezf2 | 0 | 0 | 0.027132 | 0 | 0 | 0 | 0 | 0.022401 |
| Chtf8 | 10.81302 | 10.73911 | 9.434185 | 10.88107 | 10.11448 | 9.781847 | 12.38516 | 11.47052 |
| Akr7a2 | 13.96425 | 14.58451 | 14.60711 | 13.03247 | 13.50229 | 12.40084 | 12.26773 | 12.21852 |
| Frat2 | 6.649641 | 6.839778 | 8.923518 | 5.500718 | 6.046271 | 5.300374 | 8.453776 | 7.97017 |
| Adamts2 | 9.501253 | 9.716319 | 10.02184 | 16.05567 | 14.20335 | 15.89663 | 18.73826 | 19.17141 |
| Rab11fip1 | 0.670938 | 0.759135 | 0.632454 | 0.588085 | 0.760865 | 0.774807 | 0.952001 | 0.957299 |
| AABR07034 | 0 | 0 | 0 | 0 | 0.096791 | 0 | 0 | 0 |
| AABR07049 | 0.306233 | 0 | 0.164381 | 0.141091 | 0 | 0 | 0.1713 | 0.27143 |
| Hic2 | 0.950422 | 0.996673 | 0.933236 | 0.971898 | 0.990079 | 1.128045 | 1.23186 | 1.212243 |
| Wbp2 | 69.53508 | 73.81914 | 85.00073 | 64.74317 | 56.76532 | 60.21572 | 79.41805 | 78.72324 |
| Abca14 | 0 | 0 | 0.01356 | 0.011638 | 0 | 0 | 0 | 0 |
| Sipa1l1 | 5.149154 | 4.800058 | 5.527945 | 6.609814 | 5.847841 | 6.374646 | 7.33667 | 6.888982 |
| Tbrg1 | 12.17569 | 9.86835 | 12.8092 | 16.00937 | 16.1078 | 16.08995 | 14.83156 | 14.19338 |
| AABR07037 | 0 | 0 | 0 | 0.044928 | 0 | 0.042751 | 0 | 0 |
| Ldoc1 | 0.239114 | 0.135273 | 0 | 0.220334 | 0 | 0 | 0.26751 | 0.105969 |
| Sel1l2 | 0.055006 | 0.062237 | 0.029526 | 0 | 0 | 0.024115 | 0 | 0 |
| Tryx5 | 0 | 0.078685 | 0 | 0 | 0 | 0 | 0 | 0 |
| AC109931. | 1.963722 | 2.221859 | 3.513635 | 6.031642 | 7.077814 | 6.313242 | 6.590775 | 3.481089 |
| Yipf5 | 10.14179 | 9.988063 | 9.783739 | 11.84611 | 12.91228 | 11.7479 | 11.44207 | 13.26853 |
| Tph2 | 0 | 0 | 0 | 0 | 0 | 0 | 0 | 0.01799 |
| Rn50_5_00 | 0.069543 | 0.118027 | 0.149318 | 0.128162 | 0.18799 | 0.213414 | 0.116703 | 0.184918 |
| RGD156095 | 0 | 0 | 0 | 0 | 0.135461 | 0 | 0 | 0 |
| Cx3cr1 | 0.315933 | 0.491512 | 0.381571 | 0.83697 | 0.939438 | 0.692526 | 1.104537 | 0.735072 |
| Sema4g | 0.084735 | 0.123265 | 0.116959 | 0.066925 | 0.209422 | 0.053068 | 0.230222 | 0.085834 |
| Rn50_X_06 | 0.065786 | 0 | 0.070626 | 0 | 0 | 0.057682 | 0 | 0 |
| Serpina12 | 0 | 0 | 0 | 0 | 0 | 0.080552 | 0 | 0 |
| Ccdc159 | 2.610618 | 1.911277 | 2.266861 | 3.042353 | 3.071896 | 2.894902 | 2.319334 | 2.824344 |
| LOC688924 | 0.092847 | 0 | 0 | 0 | 0 | 0 | 0.103874 | 0.082295 |
| Ac1576 | 5.680992 | 5.442874 | 5.164398 | 5.699189 | 6.093236 | 6.628075 | 5.074262 | 5.522637 |
| Prdm8 | 0.17734 | 0.171987 | 0.190386 | 0.116723 | 0.246542 | 0.088852 | 0.453486 | 0.471555 |
| Sec16b | 1.212455 | 1.070032 | 1.14545 | 1.675844 | 1.678093 | 1.70093 | 1.722679 | 1.85916 |
| AABR07045 | 21.5767 | 23.59011 | 21.08181 | 14.07383 | 16.77704 | 15.30483 | 29.29233 | 24.71143 |
| Qdpr | 40.1997 | 41.83109 | 39.60633 | 39.25556 | 35.421 | 36.52446 | 37.00084 | 37.82958 |
| Mir146b | 0 | 0 | 0 | 0 | 0 | 0 | 0 | 0.527438 |
| Hps1 | 13.44186 | 13.83932 | 14.4307 | 19.72391 | 17.56538 | 19.27068 | 20.09839 | 18.4077 |
| Mrfap1 | 0.108343 | 0.081724 | 0.142161 | 0.122019 | 0.130167 | 0.06333 | 0.202016 | 0.16005 |
| Pfn4 | 0.084643 | 0 | 0 | 0 | 0 | 0.049477 | 0 | 0.025008 |
| Spp1 | 1.622911 | 1.877053 | 0.929226 | 30.10836 | 27.37535 | 25.96129 | 40.18618 | 36.15346 |
| RGD130488 | 6.803502 | 7.396277 | 7.168456 | 8.078813 | 7.811571 | 7.970154 | 10.68743 | 10.4815 |
| Cmss1 | 1.187681 | 1.465969 | 1.564835 | 2.537021 | 1.867959 | 2.082719 | 2.174276 | 2.392501 |

| | | | | | | | | |
|---|---|---|---|---|---|---|---|---|
| Cdk6 | 3.640412 | 2.732768 | 2.818424 | 2.290089 | 2.649449 | 2.700852 | 3.328674 | 3.443858 |
| Uqcrb | 120.2533 | 129.612 | 119.5387 | 117.4675 | 116.8218 | 115.4778 | 95.30683 | 93.82138 |
| Ap4s1 | 21.21393 | 22.75919 | 21.69734 | 17.03829 | 17.82369 | 16.54765 | 17.96026 | 17.95598 |
| Kcnj8 | 12.22631 | 12.37981 | 12.90326 | 12.60272 | 12.59274 | 13.91789 | 12.19451 | 11.73679 |
| LOC100911 | 0.20278 | 0.174371 | 0.156742 | 0.149483 | 0.070164 | 0.163574 | 0.08167 | 0.179734 |
| Ilf2 | 16.37512 | 17.82558 | 13.61967 | 16.16909 | 17.5198 | 17.28972 | 19.51537 | 18.48636 |
| AC112093. | 0 | 0 | 0 | 0 | 0 | 0 | 0.492308 | 0.390038 |
| Dnase1l2 | 0.938457 | 0.70788 | 1.276159 | 0.922402 | 0.744143 | 1.09712 | 1.399873 | 1.497243 |
| LOC100911 | 0 | 0 | 0 | 0 | 0.027235 | 0 | 0 | 0 |
| Calr4 | 0.485995 | 0.229117 | 0.086958 | 0.223912 | 0.087583 | 0.17755 | 0.271855 | 0.143587 |
| Cmtm8 | 9.496174 | 9.19479 | 10.3908 | 8.161385 | 7.404862 | 8.446344 | 7.86577 | 7.081552 |
| Mir30b | 0 | 0 | 1.18354 | 0 | 0 | 0.48331 | 0 | 0 |
| Eif4ebp1 | 42.9766 | 42.13778 | 47.22055 | 46.85954 | 49.33177 | 50.1689 | 40.67792 | 40.66158 |
| AABR07056 | 0 | 0 | 0 | 0.009406 | 0 | 0 | 0 | 0 |
| Nutm1 | 0 | 0 | 0.015244 | 0 | 0 | 0 | 0 | 0.037756 |
| Mterf4 | 11.48906 | 11.40292 | 9.780835 | 10.47528 | 11.94347 | 9.967579 | 9.696461 | 9.754552 |
| Stam2 | 7.147128 | 6.859484 | 7.105641 | 7.047059 | 6.645552 | 7.827164 | 7.778102 | 7.222227 |
| AABR07068 | 0 | 0.955638 | 0 | 0 | 0 | 0 | 0 | 0 |
| AABR07072 | 0 | 0 | 0 | 0 | 0 | 0 | 0 | 0 |
| Lgi1 | 0.011945 | 0 | 0 | 0.011007 | 0 | 0.03142 | 0 | 0 |
| LOC500876 | 0.030304 | 0 | 0 | 0.027924 | 0 | 0 | 0 | 0.053721 |
| Xbp1 | 34.15985 | 32.38552 | 36.92465 | 38.0923 | 33.74012 | 34.10673 | 39.73888 | 45.83226 |
| Ccdc126 | 3.070981 | 3.243026 | 2.808466 | 2.934596 | 2.189141 | 2.453294 | 2.697643 | 2.661475 |
| AABR07053 | 0.027766 | 0 | 0 | 0 | 0 | 0 | 0 | 0 |
| LOC102550 | 0 | 0 | 0 | 0 | 0 | 0 | 0 | 0 |
| C1qtnf7 | 5.792836 | 6.070195 | 5.794958 | 13.73895 | 13.16803 | 13.85226 | 15.61276 | 15.37426 |
| Zbtb1 | 4.94452 | 4.37151 | 4.468813 | 4.810479 | 4.376619 | 4.496676 | 5.222963 | 4.994096 |
| AABR07010 | 0 | 0 | 0 | 0 | 0 | 0.037223 | 0 | 0 |
| #N/A | 0 | 0 | 3.502689 | 0 | 0.176394 | 0 | 0 | 0 |
| LOC100362 | 8.218394 | 8.821868 | 8.936086 | 9.805969 | 10.02572 | 8.129728 | 8.369238 | 9.992663 |
| Ndrg2 | 532.3782 | 539.6851 | 557.4734 | 542.7155 | 532.6073 | 535.2952 | 534.5048 | 542.4436 |
| Ccdc33 | 0 | 0 | 0 | 0 | 0 | 0.015402 | 0 | 0 |
| Pecr | 29.46568 | 28.10938 | 24.4721 | 26.32869 | 25.95435 | 25.60527 | 23.6219 | 23.69608 |
| Saysd1 | 6.313622 | 8.719352 | 9.070661 | 7.015527 | 7.629984 | 8.385092 | 6.024664 | 6.501324 |
| Nup133 | 2.153608 | 1.995155 | 2.017213 | 2.250836 | 1.812921 | 1.913632 | 2.441702 | 2.229127 |
| AC103270. | 0 | 0.174778 | 0 | 0 | 0.501084 | 0 | 0.172816 | 0 |
| AC096030. | 0.110128 | 0 | 0 | 0 | 0 | 0 | 0 | 0 |
| Gm11744 | 33.45601 | 30.03624 | 33.57474 | 32.83894 | 33.0298 | 34.43586 | 40.27696 | 39.00109 |
| Kmt5c | 7.927649 | 7.691878 | 9.863244 | 9.166294 | 9.300089 | 9.102913 | 11.93076 | 11.87796 |
| Slc6a13 | 0.338156 | 0.273291 | 0.363032 | 0.178056 | 0.156704 | 0.275317 | 0.405337 | 0.256907 |
| Fam129b | 10.67821 | 11.0509 | 12.30441 | 16.37301 | 15.99532 | 16.75292 | 18.6203 | 20.1912 |
| Gadd45b | 6.203348 | 6.517453 | 6.140753 | 12.13752 | 11.01961 | 10.13651 | 17.25994 | 17.38759 |
| Nacc1 | 13.24156 | 13.93423 | 15.02588 | 13.1815 | 11.22064 | 12.28697 | 15.71597 | 15.78063 |
| Prnd | 0.585094 | 0.99301 | 1.57034 | 0.628998 | 0.105442 | 0.769517 | 0.218192 | 0.172866 |
| Dnajc2 | 9.347515 | 9.678553 | 9.183364 | 9.664335 | 11.04568 | 10.34815 | 9.51446 | 9.208183 |
| AABR07022 | 0.793423 | 0.897721 | 0.85179 | 0.365554 | 0.857917 | 0.347837 | 2.219116 | 2.461376 |
| AABR07043 | 0 | 0 | 0 | 0.153185 | 0.179754 | 0.291521 | 0.557949 | 0.147348 |
| LOC494224 | 0 | 0 | 0 | 0.143184 | 0 | 0 | 0 | 0 |
| Rn5s | 0 | 0 | 0 | 0 | 0 | 0 | 0.49648 | 0 |
| LOC100910 | 0.837755 | 1.188387 | 1.342363 | 1.198263 | 0.338004 | 0.29601 | 0.32174 | 7.92416 |
| Tead3 | 2.763608 | 2.935245 | 3.349737 | 4.099134 | 3.518423 | 3.196974 | 4.049264 | 4.227403 |
| Ryr3 | 0.892662 | 0.738237 | 0.862112 | 0.885318 | 1.00011 | 0.886416 | 1.102949 | 1.128031 |
| AABR07054 | 1.266917 | 1.911277 | 0.793401 | 2.14026 | 1.598216 | 2.221669 | 2.480399 | 3.18164 |
| Prss35 | 0.117262 | 0.208492 | 0.08992 | 0.308721 | 0.344155 | 0.293759 | 0.431045 | 0.445437 |

| | | | | | | | | |
|---|---|---|---|---|---|---|---|---|
| Adgrf4 | 0 | 0 | 0 | 0 | 0 | 0.015005 | 0 | 0 |
| Ogn | 3.035706 | 2.708175 | 2.632289 | 1.452436 | 1.641232 | 2.098655 | 2.351224 | 2.48372 |
| Il34 | 2.900911 | 3.282244 | 3.339057 | 4.409196 | 4.365528 | 4.038167 | 5.487085 | 5.725606 |
| #N/A | 0 | 0 | 0 | 0 | 0 | 0 | 0 | 0 |
| Srp72 | 6.720655 | 7.671003 | 6.305236 | 7.518554 | 8.119374 | 8.536604 | 7.188032 | 8.50729 |
| AABR0707( | 0 | 0 | 0 | 0.610799 | 0 | 0 | 0 | 0 |
| AABR07041 | 0.461588 | 0.563496 | 0.586828 | 0.402949 | 0.538511 | 0.415371 | 0.32615 | 0.506027 |
| Nfkb2 | 9.057428 | 9.688367 | 10.2914 | 10.46794 | 9.517009 | 9.766176 | 11.37344 | 10.62847 |
| Cytl1 | 19.37596 | 17.86435 | 18.83036 | 27.82233 | 25.99016 | 25.50805 | 33.33701 | 31.91937 |
| Fancg | 0.587438 | 0.443106 | 0.57059 | 0.515525 | 0.72593 | 0.416958 | 0.6572 | 0.644646 |
| Krt1 | 1.110924 | 1.084435 | 1.216033 | 1.184249 | 1.130566 | 0.706671 | 0.999156 | 1.235661 |
| Tceanc | 0.875196 | 0.770189 | 0.730784 | 0.985672 | 1.051486 | 0.639478 | 0.815942 | 0.861922 |
| Zfp280b | 0 | 0 | 0 | 0 | 0 | 0 | 0.042576 | 0 |
| Azin1 | 29.79885 | 29.29832 | 27.65433 | 42.08714 | 43.16056 | 42.09276 | 34.98597 | 33.8124 |
| Zap70 | 1.297939 | 1.81567 | 1.317415 | 1.087272 | 0.791031 | 1.138034 | 1.399273 | 1.150427 |
| Ranbp10 | 7.570873 | 7.46236 | 7.784909 | 8.034235 | 7.644879 | 8.167117 | 9.117033 | 9.778726 |
| Ppp1r12b | 26.4599 | 26.22079 | 26.27794 | 24.78193 | 23.80596 | 23.28836 | 29.1163 | 30.98392 |
| Srrm3 | 0.147551 | 0.150253 | 0.174246 | 0.21754 | 0.159545 | 0.116436 | 0.214596 | 0.183095 |
| Dnajb1 | 18.75007 | 20.78076 | 20.41255 | 16.52626 | 15.50378 | 14.84232 | 26.01975 | 26.92637 |
| Rgma | 28.57388 | 31.27 | 33.06458 | 21.94142 | 22.15939 | 23.753 | 22.86178 | 22.88722 |
| #N/A | 0 | 0 | 0 | 0 | 0 | 0 | 0 | 0.81429 |
| Vmn2r45 | 0.020536 | 0 | 0 | 0 | 0 | 0 | 0 | 0.018202 |
| Zscan20 | 1.852171 | 1.778122 | 2.003488 | 1.887716 | 1.851004 | 1.968467 | 2.323293 | 2.139147 |
| LOC68070( | 0.94353 | 0.711707 | 0.928528 | 0.507165 | 0.425094 | 1.03411 | 0.263895 | 1.115063 |
| Lamtor3 | 33.12328 | 31.50844 | 35.55996 | 32.91254 | 35.39491 | 34.57804 | 28.83275 | 29.43547 |
| RGD156111 | 0.077694 | 0 | 0 | 0 | 0 | 0 | 0.173842 | 0.068864 |
| Arfip2 | 16.41232 | 16.39884 | 16.79573 | 22.41296 | 23.90657 | 21.81844 | 23.94244 | 24.3323 |
| Ak6 | 11.91156 | 12.3585 | 11.19538 | 10.43759 | 9.234573 | 10.48348 | 11.36492 | 10.87654 |
| Brs3 | 0.019547 | 0 | 0 | 0 | 0 | 0 | 0 | 0 |
| Pds5a | 6.880825 | 6.331696 | 6.140365 | 6.477007 | 6.518468 | 5.913968 | 7.214248 | 7.817874 |
| 5S_rRNA | 0 | 0 | 0 | 1.072292 | 0 | 0 | 0 | 0 |
| AABR07012 | 9.03974 | 7.817145 | 7.486512 | 8.329764 | 9.425449 | 9.624491 | 7.946132 | 8.47022 |
| Fbxl5 | 14.28353 | 13.84559 | 11.86878 | 13.39501 | 14.57767 | 14.07773 | 14.35112 | 13.76353 |
| Hgf | 0.849698 | 0.634321 | 0.554846 | 1.105835 | 0.994541 | 1.144406 | 1.048605 | 1.203454 |
| Ccl3 | 0.553405 | 0.547882 | 0.148529 | 0.701168 | 0.598389 | 0.606532 | 0.386953 | 0.735765 |
| AABR07062 | 3.450468 | 3.674392 | 1.307399 | 3.17947 | 2.414138 | 4.627041 | 2.497796 | 3.418123 |
| Col2a1 | 0.046158 | 0.091394 | 0.049553 | 8.665999 | 8.784101 | 8.306697 | 4.324783 | 4.776461 |
| RT1-Da | 68.85654 | 72.7182 | 71.63566 | 78.89564 | 70.7929 | 79.09357 | 90.96201 | 86.00479 |
| Lrrc39 | 73.09372 | 71.55288 | 69.8474 | 48.95805 | 50.02476 | 49.53973 | 49.74093 | 49.62721 |
| Clhc1 | 0.02531 | 0.057274 | 0.054343 | 0.023322 | 0.027367 | 0.066575 | 0.084946 | 0.112167 |
| Mmel1 | 0 | 0.065301 | 0.020653 | 0.017727 | 0 | 0 | 0.021523 | 0.034103 |
| Trim37 | 2.932227 | 2.605407 | 2.436535 | 2.686666 | 2.597363 | 2.701707 | 2.965374 | 3.054166 |
| Stil | 0.131133 | 0.111278 | 0.082122 | 0.140973 | 0.129977 | 0.057489 | 0.134481 | 0.203403 |
| 43355 | 0 | 0 | 0 | 0.03548 | 0 | 0 | 0 | 0 |
| Gpd2 | 5.303674 | 5.597638 | 5.693833 | 3.844025 | 3.218734 | 3.437153 | 3.963494 | 3.939102 |
| LOC500877 | 1.274826 | 0.77668 | 1.789717 | 1.807234 | 2.014659 | 1.203751 | 0.987382 | 0.956104 |
| AC128721. | 0 | 0 | 0 | 0 | 0.471854 | 0 | 0 | 0 |
| Fam222a | 0.463672 | 0.262311 | 0.217779 | 0.373848 | 0.438691 | 0.279502 | 0.972629 | 0.976066 |
| AABR07065 | 0.130263 | 0.294774 | 0 | 1.440392 | 0.422556 | 1.484797 | 0.145733 | 0 |
| Aldob | 0.978487 | 0.916231 | 1.086691 | 0.404182 | 0.729672 | 0.562097 | 1.207931 | 0.747657 |
| Trim21 | 3.851379 | 4.038093 | 3.321544 | 4.755519 | 4.802988 | 4.660112 | 5.946078 | 5.587038 |
| Ppp1r1b | 0.210789 | 0.30664 | 0.323279 | 0.249729 | 0.195362 | 0.132014 | 0.168444 | 0.106761 |
| Mvb12b | 8.994147 | 9.551234 | 9.794633 | 6.641035 | 6.648759 | 7.92731 | 8.917917 | 9.347513 |
| AABR07065 | 0.889233 | 1.006125 | 1.485008 | 0.819393 | 0.747845 | 0.606418 | 0.442148 | 0.875746 |

| | | | | | | | | |
|---|---|---|---|---|---|---|---|---|
| Zfp133 | 3.261386 | 2.832546 | 3.082136 | 3.70364 | 2.955298 | 2.960276 | 2.954928 | 2.707514 |
| Angptl2 | 23.51173 | 24.45751 | 24.44842 | 36.3203 | 33.46239 | 37.12878 | 34.84231 | 34.41264 |
| Cenpm | 0.564288 | 0.340515 | 0.403866 | 0.519969 | 0.447448 | 0.395815 | 0.336693 | 0.366781 |
| Cdc73 | 5.770628 | 6.672379 | 6.113623 | 5.71388 | 5.418684 | 5.747633 | 7.362017 | 6.550526 |
| Fzr1 | 9.84234 | 12.29272 | 14.36024 | 12.19112 | 12.09505 | 11.34418 | 14.70335 | 15.79077 |
| Rn60_2_11 | 0 | 0 | 0 | 0.048061 | 0 | 0.045732 | 0 | 0 |
| Exoc8 | 7.121105 | 8.080549 | 7.467686 | 6.295541 | 6.584013 | 6.732435 | 8.774999 | 7.811982 |
| Swi5 | 61.53951 | 59.16308 | 56.87472 | 65.37067 | 69.10426 | 59.99046 | 67.30822 | 64.84481 |
| Mt-nd5 | 5265.694 | 4983.084 | 4678.426 | 4596.124 | 4691.902 | 4415.719 | 4324.669 | 4256.161 |
| Fbxo34 | 2.176834 | 2.797951 | 2.523928 | 2.535416 | 2.542082 | 2.626302 | 3.078276 | 2.732082 |
| Ptpmt1 | 19.80197 | 17.56755 | 19.98178 | 18.24673 | 15.88489 | 15.02297 | 19.16859 | 18.71967 |
| Supt3h | 3.619495 | 3.69897 | 3.321697 | 3.873161 | 4.229329 | 4.504431 | 4.637136 | 4.34651 |
| Sema5b | 1.230823 | 1.464962 | 1.492973 | 1.811702 | 1.451859 | 1.625788 | 1.55582 | 1.487645 |
| AABR07052 | 0 | 0 | 0 | 0 | 0 | 0 | 0 | 0.203572 |
| Itga10 | 0.051971 | 0.058803 | 0.100429 | 0.344802 | 0.337173 | 0.355432 | 0.313971 | 0.331664 |
| Zfp354a | 2.251783 | 2.303849 | 2.288846 | 2.207371 | 2.046285 | 2.037377 | 2.036795 | 2.271891 |
| Fbxo24 | 0.224961 | 0.39594 | 0.348848 | 0.161228 | 0.432439 | 0.372576 | 0.391497 | 0.354478 |
| Lox | 3.064407 | 2.287902 | 2.506542 | 3.803392 | 3.764315 | 3.710446 | 3.731507 | 3.991057 |
| RGD130648 | 0 | 0.128246 | 0 | 0 | 0 | 0 | 0 | 0 |
| AABR07015 | 0 | 3.627525 | 1.147309 | 0 | 1.155561 | 0 | 0 | 0 |
| Rassf10 | 1.030825 | 0.971942 | 1.069768 | 1.868068 | 1.634771 | 1.26536 | 1.268566 | 0.944127 |
| Eef2kmt | 1.158815 | 0.815036 | 0.941452 | 1.096662 | 1.422336 | 1.427962 | 1.156276 | 1.471274 |
| Cox14 | 358.7367 | 392.2731 | 369.6183 | 365.2263 | 367.0701 | 333.2758 | 330.633 | 348.3756 |
| Rn60_20_0 | 1.349637 | 1.259817 | 1.231583 | 1.399092 | 1.42286 | 1.390452 | 1.547662 | 1.555126 |
| Tm4sf4 | 0 | 0.035779 | 0.135793 | 0.087415 | 0.170962 | 0.083178 | 0 | 0.056056 |
| #N/A | 0 | 0 | 0.188021 | 0 | 0 | 0.15356 | 0 | 0 |
| Akr1c13 | 1.159869 | 1.360942 | 1.106838 | 1.781289 | 1.625749 | 2.071613 | 1.393729 | 1.827643 |
| Adam17 | 7.241415 | 7.295819 | 7.34833 | 8.205273 | 8.175887 | 8.099257 | 8.902923 | 8.629721 |
| AABR07049 | 0 | 0 | 0 | 0.94614 | 0 | 0.450142 | 0 | 1.365133 |
| Mir186 | 14.00484 | 11.71213 | 11.11289 | 7.294079 | 17.77684 | 14.9489 | 12.94312 | 15.1117 |
| Gpn3 | 6.890253 | 6.830778 | 6.86876 | 7.618916 | 6.598864 | 6.846897 | 7.488266 | 7.735753 |
| Rarg | 7.845082 | 7.563382 | 8.632751 | 8.825324 | 8.235359 | 8.411924 | 10.66007 | 10.41574 |
| Lyz2 | 87.12985 | 88.57563 | 87.25626 | 175.8198 | 164.919 | 170.7324 | 198.2032 | 191.0406 |
| AABR07034 | 0.117412 | 0 | 0.12605 | 0.108191 | 0.380869 | 0.102947 | 0.131356 | 0 |
| Nfe2l2 | 20.42884 | 19.18484 | 17.25291 | 23.63504 | 24.4947 | 23.72348 | 22.82948 | 23.96172 |
| Polr1d | 22.21417 | 21.64169 | 19.11417 | 21.63772 | 22.17218 | 24.11719 | 23.80387 | 23.10954 |
| Ttc3 | 13.52983 | 12.82112 | 12.45546 | 12.10548 | 12.86575 | 12.72725 | 12.22832 | 12.58751 |
| AABR07054 | 0.051139 | 0.057861 | 0.02745 | 0.023561 | 0 | 0.044838 | 0 | 0.045327 |
| Fgd1 | 2.476006 | 2.965208 | 3.037887 | 2.414879 | 1.738487 | 2.114576 | 2.428287 | 2.308613 |
| Mal1 | 1.619868 | 1.999423 | 1.517701 | 1.845452 | 1.433078 | 1.626891 | 2.306483 | 1.696819 |
| Pcdhga8 | 0.28084 | 0.293315 | 0.162346 | 0.099532 | 0.140155 | 0.227299 | 0.265855 | 0.36381 |
| Mir293 | 0 | 0 | 0 | 0 | 0 | 0 | 0 | 0 |
| Polr1b | 2.868221 | 2.457573 | 2.55605 | 2.270887 | 2.438938 | 2.673563 | 2.803839 | 2.33245 |
| Nppb | 247.4913 | 239.7315 | 231.2307 | 593.9013 | 600.1818 | 566.0737 | 556.4671 | 539.6378 |
| Cuta | 56.19908 | 56.66134 | 61.06341 | 57.93794 | 52.34407 | 49.70907 | 54.9188 | 54.79872 |
| Mep1b | 0 | 0.024916 | 0 | 0 | 0 | 0 | 0 | 0 |
| AABR07032 | 0 | 0.553734 | 0 | 0 | 0 | 0 | 0 | 0.433781 |
| C1qbp | 87.56585 | 85.49179 | 78.99532 | 73.22391 | 73.72397 | 71.70439 | 77.57343 | 83.89659 |
| Mir149 | 0 | 0 | 0 | 1.08434 | 0 | 0 | 0 | 0.521511 |
| Slco3a1 | 20.57233 | 20.27234 | 21.46602 | 23.04151 | 22.79381 | 21.94502 | 27.74247 | 26.60444 |
| #N/A | 0 | 0 | 0 | 0 | 0 | 0 | 0.21698 | 0 |
| AABR07057 | 0 | 0 | 0.146401 | 0 | 0.147454 | 0 | 0 | 0 |
| Klri1 | 0.872765 | 0.7796 | 0.936969 | 0.507928 | 0.89404 | 0.322207 | 0.565291 | 0.651432 |
| AC098155. | 0.793423 | 0 | 0.425895 | 0.365554 | 0.857917 | 0 | 0 | 0.351625 |

| | | | | | | | | |
|---|---|---|---|---|---|---|---|---|
| Slc35a2 | 3.759069 | 4.666716 | 3.979551 | 4.522228 | 4.346893 | 4.577715 | 5.315259 | 5.36798 |
| Trim34 | 3.285625 | 3.163364 | 3.176786 | 5.096103 | 4.898752 | 5.368022 | 5.502301 | 5.969131 |
| Lig3 | 6.655691 | 8.177503 | 7.286957 | 6.74085 | 6.673594 | 7.918064 | 8.03653 | 6.964776 |
| AABR07031 | 0 | 0 | 0 | 0 | 0 | 0.379459 | 0 | 0 |
| AABR07035 | 0 | 0 | 0 | 0 | 0 | 0.433156 | 0 | 0 |
| Mtpn | 22.23157 | 22.89599 | 23.48137 | 25.04603 | 23.0029 | 23.69215 | 25.8094 | 26.56807 |
| Mylk4 | 0.269372 | 0.121913 | 0.404863 | 0 | 0.116507 | 0.094474 | 0.241089 | 0 |
| Fancc | 0.511303 | 0.723144 | 0.731888 | 0.726349 | 0.69108 | 0.877942 | 0.66736 | 0.717556 |
| Trpm1 | 0.031232 | 0.011779 | 0.03353 | 0.028779 | 0.022514 | 0.009128 | 0.069882 | 0.03691 |
| Fabp12 | 0.231026 | 0.261395 | 0.248021 | 0.283842 | 0 | 0.202564 | 0.258462 | 0.068257 |
| AC135285. | 0.265817 | 0.601519 | 0.570743 | 0.979759 | 0.862272 | 0.932274 | 0.297384 | 1.41364 |
| Adam30 | 0 | 0 | 0 | 0 | 0 | 0 | 0.017025 | 0 |
| Casq1 | 10.67765 | 10.47471 | 9.237583 | 18.29118 | 18.42381 | 20.542 | 18.93517 | 19.05412 |
| AABR07012 | 0.100898 | 0 | 0.758241 | 0 | 0.109099 | 0.442336 | 0.22576 | 0.089431 |
| Islr2 | 0.099517 | 0.135119 | 0.170941 | 0.880331 | 0.731724 | 0.977275 | 1.002018 | 1.146691 |
| Hey2 | 3.604687 | 3.39139 | 2.902397 | 3.177161 | 3.346935 | 3.005999 | 2.038299 | 2.569902 |
| Disp3 | 0 | 0 | 0 | 0 | 0 | 0 | 0 | 0 |
| Mir3569 | 0 | 0 | 0 | 0 | 0.745033 | 0 | 0 | 0 |
| Echs1 | 8.615836 | 17.53915 | 11.7137 | 7.906616 | 7.025315 | 7.337649 | 6.399674 | 6.509926 |
| Rn50_6_09 | 1.158092 | 1.652152 | 1.027063 | 1.34552 | 1.524452 | 1.236159 | 1.351954 | 1.428139 |
| Amdhd2 | 3.80649 | 3.461592 | 4.239277 | 5.343248 | 5.42376 | 5.770503 | 5.134118 | 4.572082 |
| Aifm2 | 3.525809 | 2.861879 | 2.945858 | 2.669743 | 2.817865 | 2.405941 | 2.949813 | 2.70389 |
| Ints3 | 8.792957 | 9.010004 | 9.278966 | 8.505889 | 8.298986 | 8.861577 | 11.78398 | 12.07349 |
| Obsl1 | 21.76598 | 23.12899 | 25.96121 | 17.732 | 17.52602 | 18.60138 | 22.18821 | 21.92225 |
| RGD156185 | 0.160632 | 0.121165 | 0.057483 | 0.098677 | 0.115792 | 0.140842 | 0.059903 | 0.142376 |
| Xcl1 | 2.816494 | 1.652379 | 1.007895 | 1.537949 | 1.46632 | 1.006094 | 1.983943 | 1.294429 |
| Abce1 | 11.74818 | 12.056 | 11.52065 | 12.81156 | 11.48863 | 12.57658 | 12.27731 | 13.18441 |
| Cmc1 | 20.52806 | 18.58123 | 19.46013 | 16.48887 | 15.41204 | 16.301 | 14.03952 | 14.89933 |
| G6pc | 0.035382 | 0.040033 | 0.037985 | 0 | 0.038258 | 0.124093 | 0 | 0.031361 |
| Sae1 | 15.13935 | 13.70357 | 15.58552 | 17.4628 | 17.86079 | 19.03424 | 17.57238 | 16.9891 |
| Psat1 | 0.489401 | 0.30763 | 0.291891 | 0.375803 | 0.382187 | 0.309911 | 0.304178 | 0.289187 |
| Mrpl4 | 77.74838 | 80.4964 | 85.72374 | 61.37676 | 60.19298 | 63.05199 | 61.98034 | 64.00473 |
| Smyd2 | 72.1486 | 72.1152 | 74.27936 | 81.21845 | 77.54588 | 83.87046 | 76.17866 | 78.41843 |
| Ubl7 | 28.25914 | 27.97172 | 29.8014 | 26.03969 | 23.32249 | 24.17084 | 28.21699 | 27.6715 |
| Fam227a | 0 | 0.038827 | 0.221041 | 0.284586 | 0.148421 | 0.210617 | 0.268737 | 0.304158 |
| AABR07036 | 0 | 0 | 0 | 0 | 0.845112 | 0 | 0.874398 | 1.039131 |
| Tmem176b | 16.41762 | 16.94043 | 17.78451 | 25.44132 | 26.16737 | 25.05313 | 26.61809 | 26.01578 |
| Znrd1 | 13.62682 | 15.08772 | 15.98769 | 16.86169 | 14.20825 | 14.59364 | 13.17611 | 14.58003 |
| Add3 | 21.10514 | 21.04919 | 19.87576 | 22.24944 | 22.59394 | 23.66645 | 24.76777 | 25.78289 |
| AABR07042 | 0 | 0 | 0 | 0.043354 | 0 | 0 | 0 | 0 |
| Gata2 | 8.44364 | 9.258289 | 9.658119 | 7.554141 | 7.005189 | 7.780291 | 10.51123 | 10.11023 |
| Xrcc4 | 5.42973 | 6.102253 | 5.594431 | 5.238336 | 6.304525 | 5.463728 | 6.237616 | 6.169223 |
| Vnn1 | 19.96305 | 21.26846 | 19.52051 | 31.59594 | 31.59953 | 30.65921 | 29.16193 | 26.5038 |
| Galr1 | 0.046527 | 0 | 0.049949 | 0 | 0.050309 | 0.020397 | 0 | 0 |
| Aamdc | 19.73849 | 24.24983 | 18.73939 | 18.32398 | 15.52938 | 15.88602 | 16.9739 | 18.80082 |
| Ythdf2 | 6.395263 | 5.703623 | 6.300311 | 5.33835 | 5.694793 | 5.277527 | 6.060483 | 5.912961 |
| Dchs1 | 5.386854 | 5.717162 | 6.204874 | 5.60178 | 5.230043 | 5.239866 | 6.933032 | 6.963919 |
| Igfbp3 | 22.64766 | 23.90168 | 24.15022 | 22.49274 | 19.97196 | 20.42975 | 30.18072 | 29.94672 |
| Crabp1 | 0 | 0.077349 | 0 | 0.062994 | 0.147839 | 0 | 0 | 0.060593 |
| Ptpn1 | 9.273451 | 8.726413 | 8.345653 | 8.601523 | 8.504959 | 8.613998 | 10.68288 | 11.06787 |
| Mir3578 | 0 | 0 | 0 | 0.41242 | 0 | 0 | 0 | 0 |
| Rsrp1 | 61.1344 | 60.63521 | 63.68587 | 74.77205 | 74.18132 | 72.43665 | 87.9592 | 84.16702 |
| Mrps30 | 41.56082 | 43.14871 | 45.52799 | 44.5764 | 42.57188 | 44.45934 | 37.48945 | 41.1565 |
| RGD130973 | 20.6169 | 20.50295 | 16.29201 | 18.72166 | 17.48875 | 16.76382 | 18.42988 | 16.1057 |

| | | | | | | | | |
|---|---|---|---|---|---|---|---|---|
| AC130232. | 0.498723 | 1.128563 | 0.713881 | 0.612738 | 0.179754 | 0.728801 | 0.371966 | 0.442043 |
| Mier1 | 14.54357 | 14.71424 | 13.65368 | 14.41616 | 14.4044 | 14.06139 | 15.76436 | 17.31685 |
| Ccdc151 | 0.211817 | 0.133145 | 0.227399 | 0.303615 | 0.356276 | 0.43335 | 0.368623 | 0.45893 |
| RT1-CE4 | 30.25587 | 29.82051 | 21.20421 | 33.21207 | 35.84392 | 35.73884 | 42.64541 | 43.96138 |
| AABR07030 | 0 | 0 | 0 | 0 | 0 | 0.041477 | 0 | 0 |
| Rpl38-ps3 | 1.720946 | 23.08786 | 1.319675 | 0.453081 | 1.063334 | 0.862244 | 1.650272 | 1.307451 |
| AC117869. | 5.492929 | 4.971992 | 7.731635 | 5.061518 | 6.071412 | 4.923232 | 5.189318 | 7.032503 |
| Tecpr1 | 44.22192 | 51.0947 | 51.58432 | 40.93637 | 38.83441 | 42.12993 | 55.6691 | 51.5757 |
| Dsg2 | 10.78751 | 10.38525 | 11.10186 | 10.02596 | 10.5985 | 9.8825 | 11.97495 | 11.54799 |
| AABR07049 | 0.807182 | 0.399564 | 0.379121 | 0.511353 | 0.763695 | 0.840439 | 1.241679 | 0.536584 |
| Sar1b | 73.57743 | 72.14011 | 70.53956 | 69.59293 | 73.37633 | 70.36104 | 71.70133 | 70.09914 |
| Rn50_10_0 | 0 | 0 | 0.220897 | 0.379199 | 0 | 0.090205 | 0.46039 | 0.091188 |
| AABR07049 | 0 | 0.62368 | 0 | 0.507928 | 0 | 0 | 0.616681 | 0.977148 |
| Ccdc115 | 15.14717 | 14.88584 | 13.19501 | 15.03424 | 17.12714 | 15.93726 | 18.30166 | 16.26426 |
| Wdr27 | 0.182906 | 0.310424 | 0.157089 | 0.488767 | 0.355992 | 0.384893 | 0.266015 | 0.518779 |
| Tbx10 | 0.809521 | 1.202165 | 0.760439 | 0.979049 | 1.039447 | 0.97596 | 0.905657 | 1.479883 |
| Btk | 0.69054 | 0.61854 | 0.67956 | 1.219585 | 1.057783 | 1.00911 | 1.094439 | 1.147612 |
| Mug1 | 0 | 0 | 0 | 0.010783 | 0 | 0 | 0 | 0 |
| Zw10 | 2.817719 | 2.828892 | 2.726762 | 3.400941 | 2.403077 | 2.940337 | 3.241137 | 3.412056 |
| 5S_rRNA | 0 | 0 | 0 | 0.419592 | 0 | 0 | 0 | 0 |
| AABR07054 | 3.818348 | 4.526009 | 6.05126 | 2.178093 | 4.915149 | 5.261035 | 5.695731 | 3.706715 |
| Tmem65 | 40.41408 | 39.13853 | 38.78107 | 27.1093 | 28.24912 | 24.82136 | 31.49944 | 31.64472 |
| Aspm | 0.22611 | 0.24365 | 0.219625 | 0.297645 | 0.209562 | 0.236016 | 0.277053 | 0.229043 |
| Mgea5 | 16.0444 | 15.17264 | 14.36954 | 17.37297 | 16.98405 | 16.64044 | 18.4247 | 20.0532 |
| Dgkq | 3.613433 | 3.161022 | 3.730526 | 3.925354 | 3.732392 | 3.785718 | 4.442929 | 4.522755 |
| Zfyve26 | 1.972538 | 2.163258 | 2.064409 | 1.868387 | 1.960102 | 2.07736 | 2.588969 | 2.3344 |
| Adarb1 | 5.162489 | 5.142416 | 5.878157 | 6.851035 | 6.935367 | 6.569525 | 8.879814 | 8.74286 |
| Gpr31 | 0.163644 | 0.061718 | 0.117121 | 0.100527 | 0.176945 | 0.334793 | 0.42718 | 0.386788 |
| Lrrc45 | 2.916015 | 3.414695 | 3.261879 | 3.551341 | 3.395587 | 3.271943 | 3.832642 | 3.759431 |
| Pcdhgb8 | 0.928933 | 0.855501 | 0.510231 | 0.557379 | 0.607337 | 0.606132 | 0.555878 | 0.382958 |
| Wfdc6b | 0 | 0 | 0 | 0 | 0 | 0.083633 | 0.213423 | 0.169087 |
| Cntn3 | 0.101534 | 0.05744 | 0.081752 | 0.093559 | 0.068617 | 0.05564 | 0.156188 | 0.168739 |
| Amh | 0.063016 | 0.142598 | 0.101477 | 0.203232 | 0.034069 | 0.221008 | 0.176247 | 0.195488 |
| Cenpo | 0.760872 | 0.202563 | 0.384398 | 0.61863 | 0.338767 | 0.431675 | 0.250362 | 0.555387 |
| 43353 | 4.294178 | 2.836936 | 3.434351 | 3.877247 | 5.391136 | 4.295797 | 4.449468 | 5.083373 |
| AABR07032 | 0 | 0 | 0 | 0 | 0 | 0 | 0.1741 | 0.068967 |
| Aifm3 | 0.436903 | 0.494335 | 0.368534 | 0.488858 | 0.438673 | 0.930329 | 0.663354 | 0.44257 |
| Olr1596 | 0 | 0 | 0 | 0.049951 | 0 | 0.047531 | 0 | 0 |
| Mrgprd | 0 | 0.123437 | 0 | 0 | 0.235927 | 0 | 0 | 0 |
| AC122603. | 0 | 0.256492 | 0.243369 | 0.313332 | 0.245119 | 0.298146 | 0.126807 | 0 |
| 5S_rRNA | 0 | 0 | 0 | 0 | 0 | 0 | 0 | 0 |
| Rpl11 | 118.9328 | 118.6426 | 109.85 | 112.8925 | 107.761 | 108.6717 | 114.5096 | 107.9447 |
| Nat9 | 9.112307 | 8.599293 | 9.270016 | 9.056629 | 8.43314 | 10.25749 | 9.660237 | 10.05178 |
| Pdia6 | 49.37443 | 51.60879 | 50.01763 | 54.23019 | 56.2382 | 56.14656 | 60.87106 | 59.48829 |
| AABR07021 | 0.083121 | 0.219443 | 0.11898 | 0.280838 | 0.149795 | 0.291521 | 0.185983 | 0.147348 |
| Ap1m1 | 15.43046 | 16.63038 | 17.06051 | 16.51752 | 14.3389 | 17.28629 | 20.0541 | 19.70995 |
| Dlgap5 | 1.454077 | 1.342153 | 1.355644 | 1.181206 | 1.013702 | 1.157508 | 1.027426 | 1.322737 |
| St14 | 0.606808 | 0.816468 | 0.528201 | 0.680047 | 0.7448 | 0.747746 | 0.752262 | 0.857644 |
| LOC679811 | 5.64038 | 6.095537 | 5.84026 | 5.381968 | 6.030463 | 5.74971 | 5.460983 | 5.494612 |
| Dnah1 | 0.06739 | 0.035882 | 0.051069 | 0.116889 | 0.102872 | 0.041709 | 0.088697 | 0.126489 |
| Slc15a5 | 0 | 0 | 0 | 0 | 0.032997 | 0 | 0 | 0 |
| Ly6c | 0.059237 | 0 | 0.063595 | 0.054585 | 0.064053 | 0.207758 | 0.198817 | 0.052505 |
| AABR07032 | 5.199453 | 3.781888 | 4.784524 | 7.186637 | 6.023672 | 5.535789 | 3.323953 | 6.58362 |
| Glb1l2 | 4.062873 | 3.794308 | 4.154051 | 2.792977 | 2.882263 | 2.808398 | 3.318836 | 3.410591 |

| | | | | | | | | |
|---|---|---|---|---|---|---|---|---|
| LOC691427 | 14.59378 | 25.90145 | 12.59533 | 12.1292 | 8.354141 | 16.55932 | 6.722831 | 11.41341 |
| Gcc1 | 3.810318 | 3.875043 | 3.692699 | 3.907247 | 3.767353 | 3.457886 | 3.831557 | 3.758367 |
| Nid2 | 6.547236 | 7.489146 | 7.221624 | 6.617573 | 6.302895 | 6.391296 | 7.297976 | 7.489977 |
| Fbl | 16.31724 | 17.60932 | 17.6604 | 16.13886 | 15.91759 | 16.75626 | 18.2054 | 18.23568 |
| Med22 | 0.036903 | 0.041754 | 0.039618 | 0 | 0 | 0.032357 | 0.041286 | 0.032709 |
| Tbcc | 11.25368 | 12.68167 | 11.9354 | 10.66252 | 10.10766 | 10.3049 | 10.30562 | 10.98021 |
| Prss32 | 0 | 0 | 0 | 0 | 0 | 0 | 0 | 0 |
| Armc8 | 8.219342 | 7.221333 | 7.408105 | 7.378487 | 6.035321 | 7.165427 | 6.29716 | 6.919907 |
| Xylb | 1.408185 | 1.768383 | 1.711132 | 1.882214 | 1.639777 | 1.831695 | 1.887036 | 2.084813 |
| Sycp3 | 6.969451 | 5.914204 | 4.727359 | 5.13766 | 5.549212 | 4.277575 | 5.49342 | 5.222686 |
| Sowahc | 0.457191 | 0.663192 | 0.616676 | 0.788556 | 0.887302 | 0.770895 | 1.127889 | 0.997491 |
| Tango6 | 1.497595 | 1.393222 | 1.786405 | 1.701969 | 1.403418 | 1.415222 | 2.103612 | 1.99109 |
| Agbl1 | 0 | 0.019697 | 0.01869 | 0 | 0.018824 | 0.015264 | 0 | 0.01543 |
| Prpf31 | 6.077409 | 5.126419 | 6.477717 | 6.623766 | 6.312327 | 6.054448 | 5.653761 | 6.023848 |
| Tnni1 | 2.868595 | 1.909224 | 3.07962 | 4.04269 | 4.865522 | 5.030373 | 6.355587 | 4.686321 |
| AABR07071 | 0.060609 | 0 | 0 | 0 | 0 | 0 | 0 | 0 |
| AC096239. | 12.51845 | 12.76759 | 9.653624 | 13.15995 | 11.43889 | 11.90376 | 13.61058 | 15.47151 |
| Rcan1 | 86.73409 | 87.66519 | 86.41326 | 159.956 | 155.6594 | 158.9757 | 191.949 | 195.0874 |
| Naip6 | 0.153841 | 0.135383 | 0.146808 | 0.126008 | 0.166347 | 0.202332 | 0.315537 | 0.28029 |
| Marveld2 | 0.199531 | 0.22576 | 0.357016 | 0.163431 | 0.191778 | 0.233266 | 0.297636 | 0.255457 |
| Ccl5 | 13.35571 | 14.35036 | 14.85397 | 10.97869 | 12.46734 | 12.46852 | 13.11437 | 10.21971 |
| Rn50_10_( | 0.719453 | 0.639593 | 0.38619 | 0.331474 | 0.3334 | 0.315409 | 0.229969 | 0.683236 |
| Erh | 68.73027 | 68.72515 | 57.87164 | 64.21925 | 70.67406 | 62.54161 | 71.72314 | 65.6117 |
| Megf8 | 7.20229 | 7.50806 | 8.590195 | 7.567374 | 7.574045 | 7.570268 | 10.08674 | 10.08944 |
| Hspa14 | 10.22865 | 10.79719 | 9.284321 | 9.755047 | 10.31845 | 10.04053 | 10.54257 | 10.3349 |
| AC095278. | 0 | 0 | 0 | 0 | 0 | 0 | 0 | 0.486016 |
| AABR07006 | 0 | 0 | 0.097601 | 0.083773 | 0 | 0.159425 | 0 | 0 |
| Stmn2 | 0 | 0 | 0 | 0 | 0 | 0.022256 | 0 | 0.022499 |
| Dnah6 | 0.050558 | 0.057205 | 0.013569 | 0.015529 | 0.022778 | 0.025859 | 0.004714 | 0.029875 |
| Efcab14 | 10.17226 | 9.681713 | 9.29537 | 9.900696 | 9.651678 | 9.744587 | 10.72968 | 11.34795 |
| Myo7a | 0.284639 | 0.235014 | 0.22299 | 0.368615 | 0.266185 | 0.296788 | 0.542212 | 0.4023 |
| Syne4 | 0 | 0 | 0 | 0 | 0.03219 | 0 | 0 | 0.026387 |
| LOC364556 | 0 | 0 | 0.026431 | 0 | 0 | 0 | 0 | 0 |
| Ferd3l | 0 | 0 | 0 | 0 | 0 | 0 | 0 | 0.052804 |
| Rn50_10_( | 0 | 0 | 0 | 0 | 0 | 0 | 0.217383 | 0 |
| Lsm2 | 9.662246 | 8.687335 | 10.00257 | 7.710962 | 6.809627 | 8.018016 | 9.941055 | 10.85809 |
| Als2cl | 5.183475 | 5.491828 | 4.876567 | 4.759491 | 4.436321 | 5.21938 | 5.327742 | 5.243753 |
| Smco4 | 8.369248 | 9.206371 | 9.172108 | 7.765488 | 5.718811 | 6.471853 | 5.982008 | 6.593849 |
| Luc7l2 | 19.74355 | 21.98076 | 20.7712 | 24.40912 | 24.15067 | 22.32412 | 27.26721 | 24.86429 |
| Rn50_13_( | 1.442282 | 1.631874 | 1.786594 | 1.226775 | 1.19963 | 1.167317 | 2.358281 | 2.163389 |
| Plin1 | 0.733123 | 1.090192 | 0.854516 | 0.386025 | 0.475629 | 0.385682 | 0.328074 | 0.594106 |
| Nmral1 | 2.917459 | 3.412865 | 3.450595 | 1.777028 | 2.726863 | 2.731457 | 2.489433 | 2.848861 |
| Gtpbp4 | 3.584312 | 3.580692 | 2.890683 | 3.447803 | 3.403022 | 3.265371 | 4.088212 | 3.982815 |
| RGD156430 | 0 | 0 | 0.041643 | 0.035743 | 0 | 0.034011 | 0.043396 | 0.171906 |
| RGD131184 | 5.939811 | 5.896633 | 6.230174 | 6.60571 | 7.013219 | 6.664684 | 7.179868 | 6.696923 |
| Npy1r | 1.163687 | 0.708969 | 0.480497 | 1.010429 | 0.750127 | 0.882971 | 1.226773 | 1.170281 |
| Numb | 8.93447 | 8.361709 | 8.673998 | 9.401611 | 8.527666 | 7.495256 | 11.07525 | 9.801065 |
| Obp3 | 0 | 0 | 0 | 0.110672 | 0.584407 | 0.421234 | 1.074948 | 1.064553 |
| AC123253. | 0 | 0 | 0.010952 | 0 | 0 | 0 | 0 | 0.009042 |
| Rgs18 | 2.662674 | 3.263748 | 2.540934 | 3.407707 | 2.239308 | 2.269784 | 5.047549 | 4.523449 |
| Pde4c | 6.663872 | 7.183428 | 7.67439 | 6.899685 | 5.921651 | 6.565283 | 6.641945 | 6.744173 |
| RGD131008 | 0.55129 | 0.533577 | 0.477755 | 0.538594 | 0.423735 | 0.582376 | 0.572174 | 0.677026 |
| Pigo | 2.885437 | 2.834221 | 3.029619 | 2.702643 | 2.86284 | 2.363143 | 3.05073 | 2.669924 |
| Kcng4 | 0.103108 | 0.102079 | 0.12453 | 0.154391 | 0.222978 | 0.16951 | 0.331639 | 0.388406 |

| | | | | | | | | |
|---|---|---|---|---|---|---|---|---|
| Asf1b | 0.429229 | 0.560368 | 0.567144 | 0.456366 | 0.535522 | 0.492148 | 0.701834 | 0.321917 |
| Dnali1 | 0 | 0.02638 | 0 | 0.021484 | 0 | 0.020443 | 0 | 0.020665 |
| Napepld | 10.04881 | 7.292952 | 8.68201 | 9.370257 | 8.484719 | 8.178957 | 12.40669 | 12.27785 |
| AC098622. | 1.726349 | 0.651094 | 1.235564 | 1.060508 | 3.111127 | 6.559212 | 3.862725 | 5.610546 |
| Irf7 | 33.68087 | 31.83201 | 33.90756 | 41.96775 | 42.56016 | 41.9118 | 72.74832 | 70.71568 |
| Dirc2 | 15.16942 | 15.22925 | 15.12511 | 11.73284 | 11.40949 | 12.04286 | 14.57472 | 12.97517 |
| Lrrc18 | 0 | 0.039186 | 0 | 0.063827 | 0.037449 | 0 | 0 | 0.030697 |
| Slc19a1 | 3.947516 | 4.113152 | 4.190025 | 4.500612 | 4.099586 | 4.478031 | 4.615913 | 4.309355 |
| Pnkp | 3.452055 | 3.541734 | 3.894442 | 4.609657 | 4.207148 | 4.463196 | 5.105703 | 4.952611 |
| Slc35d3 | 0 | 0 | 0 | 0 | 0 | 0 | 0 | 0 |
| LOC690020 | 0.172115 | 0.048685 | 0.046194 | 0 | 0 | 0.037728 | 0 | 0 |
| Irf2 | 8.181438 | 6.215737 | 7.365819 | 7.473696 | 6.781445 | 6.553306 | 8.572722 | 8.484599 |
| Cdc23 | 9.07164 | 9.205136 | 9.558633 | 12.03011 | 10.30208 | 11.19455 | 10.1221 | 11.31646 |
| Cbx1 | 27.21526 | 22.61677 | 23.37386 | 27.41262 | 25.87588 | 22.87496 | 30.65721 | 28.61397 |
| Tle2 | 3.697181 | 4.427817 | 5.08331 | 3.825187 | 4.067844 | 4.720358 | 5.152161 | 5.135876 |
| Ucn | 6.247163 | 5.509171 | 7.594383 | 4.909968 | 6.15894 | 6.202483 | 10.58635 | 7.898611 |
| Sgpl1 | 6.632453 | 5.953673 | 6.03051 | 7.265254 | 7.68383 | 7.551041 | 7.533666 | 7.648273 |
| Ghr | 100.5439 | 95.42341 | 97.85177 | 84.67829 | 82.68801 | 82.93267 | 83.53457 | 88.84548 |
| AC136795. | 0.327287 | 0.37031 | 0 | 0 | 1.061672 | 0 | 0.366154 | 0 |
| Spidr | 1.168797 | 1.083966 | 1.234208 | 1.659639 | 1.429548 | 1.444803 | 1.693446 | 1.443554 |
| AC113837. | 31.93044 | 24.5669 | 33.59378 | 36.48408 | 40.74059 | 31.91617 | 45.72462 | 27.7355 |
| Car5a | 0 | 0 | 0 | 0 | 0 | 0.036732 | 0 | 0 |
| Mir208b | 11.22127 | 9.522253 | 9.53701 | 1.723326 | 4.550023 | 3.279606 | 7.323083 | 4.558569 |
| Ppp1r32 | 0.069961 | 0.277052 | 0.225323 | 0.322332 | 0.37824 | 0.337381 | 0.352212 | 0.465075 |
| Cfap36 | 24.79723 | 21.24898 | 22.31501 | 19.18701 | 22.43608 | 24.55594 | 25.57979 | 23.40119 |
| Klk11 | 0 | 0 | 0.044618 | 0 | 0 | 0.03644 | 0 | 0 |
| AABR07065 | 0 | 0 | 0.786268 | 0 | 0 | 0 | 1.229049 | 0.324577 |
| Nrn1 | 0.608906 | 1.033423 | 1.348255 | 0.876692 | 0.617251 | 0.600626 | 0.468337 | 0.640898 |
| AABR07043 | 0 | 0 | 0 | 0 | 0 | 0.133861 | 0 | 0 |
| LOC102551 | 0 | 0 | 0 | 0 | 0 | 0.257224 | 0 | 0 |
| AABR07047 | 0.855907 | 0.792342 | 1.503606 | 0.932081 | 1.093748 | 0.545789 | 0.78345 | 0.896566 |
| Tax1bp3 | 30.89312 | 30.35626 | 32.86829 | 36.76429 | 37.998 | 36.15665 | 36.9383 | 37.73738 |
| Shank3 | 64.016 | 71.2878 | 81.23237 | 57.76501 | 57.26522 | 57.65803 | 82.32442 | 82.34494 |
| LOC103689 | 0 | 0 | 0 | 0.031787 | 0 | 0 | 0 | 0.030576 |
| Lyrm7 | 10.35038 | 8.832851 | 9.636502 | 9.941601 | 10.55942 | 9.998129 | 10.10757 | 10.26251 |
| Grhl3 | 0.021115 | 0.071673 | 0.090674 | 0.369681 | 0.296812 | 0.222167 | 0.094491 | 0.224586 |
| AABR07071 | 0 | 0 | 0 | 0.198573 | 0 | 0.188949 | 0 | 0 |
| Oas1f | 8.428202 | 9.092574 | 8.662437 | 13.18457 | 12.64558 | 11.228 | 16.92121 | 17.60451 |
| AABR07036 | 0 | 0 | 0 | 0 | 0.076106 | 0 | 0 | 0 |
| Kif5c | 0.128491 | 0.118949 | 0.100322 | 0.15069 | 0.214718 | 0.235564 | 0.169886 | 0.217422 |
| Phldb3 | 0.710232 | 1.019946 | 0.762479 | 0.528595 | 0.679352 | 0.718537 | 0.886257 | 0.677938 |
| Nceh1 | 12.11249 | 12.48492 | 11.43766 | 10.06552 | 10.55994 | 10.05031 | 12.16745 | 12.6189 |
| Arhgef17 | 10.166 | 11.1279 | 12.07083 | 9.633424 | 9.487551 | 9.791519 | 10.56632 | 10.96619 |
| Ivd | 170.0792 | 174.6784 | 182.5122 | 164.2904 | 167.3827 | 171.3969 | 170.8309 | 175.9721 |
| Tfap2e | 0.084826 | 0.031992 | 0.030355 | 0.130273 | 0.030574 | 0.024792 | 0.126533 | 0.075186 |
| AABR07050 | 2.816494 | 2.360541 | 3.695616 | 3.940993 | 3.609403 | 3.018283 | 3.501076 | 2.958694 |
| LOC689927 | 0 | 0 | 0 | 0 | 0 | 0 | 0 | 0.053289 |
| Adam9 | 13.76002 | 14.70707 | 15.04481 | 18.80843 | 18.20924 | 19.32148 | 21.54795 | 23.02724 |
| Ddx42 | 9.474267 | 9.860364 | 9.093301 | 9.275799 | 10.1052 | 10.29351 | 10.55618 | 10.67945 |
| AABR07066 | 0 | 0.75961 | 0 | 0 | 0 | 0.588647 | 0 | 0.595058 |
| Xk | 2.748932 | 3.278411 | 2.911274 | 2.441752 | 2.021754 | 2.377695 | 2.050256 | 2.447491 |
| Tmc2 | 0 | 0 | 0 | 0.01552 | 0 | 0 | 0 | 0 |
| Mir3568 | 0.518474 | 1.173259 | 0 | 0.477754 | 3.924333 | 0 | 0 | 0.45955 |
| Ly6g6c | 0.82466 | 0.933064 | 0.295108 | 1.266486 | 1.33754 | 1.446126 | 1.998952 | 1.096406 |

| | | | | | | | | |
|---|---|---|---|---|---|---|---|---|
| Spop | 66.76782 | 65.23787 | 63.75762 | 50.9026 | 51.25741 | 48.72558 | 55.25405 | 57.01453 |
| Hnrnpul1 | 23.30325 | 22.23836 | 24.08707 | 24.40995 | 22.88481 | 22.7979 | 26.35215 | 25.78447 |
| Ccdc149 | 6.349057 | 5.621995 | 5.92706 | 6.384574 | 5.01454 | 5.203803 | 7.195692 | 7.022123 |
| AABR07012 | 0.101484 | 0 | 0 | 0.093514 | 0 | 0 | 0 | 0.089951 |
| Ndufaf6 | 23.68935 | 24.03623 | 23.78461 | 22.35905 | 20.42973 | 19.67764 | 18.83079 | 17.89424 |
| AABR07005 | 0 | 0 | 0 | 0 | 0 | 0 | 0 | 1.079407 |
| AABR07028 | 4.593502 | 3.118399 | 2.958851 | 1.693092 | 2.980132 | 1.611035 | 0 | 3.257159 |
| Samsn1 | 1.310172 | 1.111798 | 1.142824 | 1.659998 | 1.593753 | 1.471848 | 1.740592 | 1.741905 |
| AABR07041 | 0.10579 | 0.478784 | 0 | 0 | 0 | 0 | 0 | 0 |
| Mark4 | 6.15846 | 6.752946 | 7.040024 | 5.955015 | 5.467001 | 6.599686 | 7.038666 | 7.109592 |
| Dgke | 11.5049 | 12.21496 | 12.38931 | 8.820817 | 8.951494 | 8.641995 | 11.48291 | 11.29724 |
| AABR07001 | 0 | 0 | 0 | 0 | 0 | 0 | 0 | 0.730937 |
| AABR07052 | 0.122065 | 0 | 0.262089 | 0.112478 | 0.065994 | 0.053513 | 0.06828 | 0.054096 |
| Gemin7 | 10.46303 | 9.654635 | 9.16067 | 9.313651 | 9.061799 | 9.841999 | 10.85322 | 10.75952 |
| Wdr43 | 5.387692 | 5.267013 | 5.177773 | 5.358334 | 6.056678 | 5.553632 | 6.129961 | 6.561073 |
| Mrps5 | 13.11831 | 15.44981 | 14.77455 | 15.17798 | 14.59074 | 14.55997 | 13.95588 | 16.09766 |
| Tubd1 | 1.803234 | 1.550609 | 1.974605 | 2.060396 | 2.0668 | 1.581077 | 1.977031 | 1.790092 |
| Ccdc77 | 4.580674 | 4.185186 | 4.271195 | 3.131004 | 3.650815 | 3.035824 | 4.739704 | 4.593798 |
| Echdc1 | 8.432515 | 8.396075 | 8.871787 | 9.842293 | 10.94155 | 8.995592 | 9.11946 | 9.716405 |
| Acp6 | 9.674158 | 9.638329 | 9.64145 | 8.883932 | 10.81754 | 10.76935 | 9.714859 | 9.452641 |
| LOC100362 | 1.024105 | 0.849733 | 1.759108 | 1.572788 | 2.067054 | 1.496561 | 1.298487 | 1.815431 |
| Hecw2 | 5.09223 | 4.553357 | 5.222684 | 3.343826 | 3.28231 | 2.930343 | 4.391638 | 4.469676 |
| RGD130462 | 2.121032 | 1.013269 | 1.113231 | 1.563558 | 2.140545 | 1.115834 | 1.529213 | 2.088862 |
| Lrrc8c | 4.970429 | 4.808007 | 4.603186 | 3.979276 | 3.864961 | 3.39634 | 5.680833 | 5.432137 |
| Rbbp8 | 3.017697 | 3.414382 | 2.934779 | 3.271399 | 3.051857 | 3.626466 | 3.75339 | 4.16944 |
| Saxo2 | 0.018642 | 0.021093 | 0 | 0.051534 | 0.040315 | 0.016345 | 0.020856 | 0.016524 |
| Fpgt | 4.853316 | 4.387907 | 4.017322 | 4.513936 | 5.321388 | 4.613322 | 5.023718 | 4.864579 |
| Dennd4c | 6.784854 | 6.391649 | 6.053934 | 6.095906 | 6.29139 | 6.018851 | 6.643162 | 6.826184 |
| AABR07019 | 0 | 0 | 0 | 0 | 0 | 0 | 0 | 0 |
| Robo2 | 0.132596 | 0.085729 | 0.091511 | 0.410182 | 0.501809 | 0.423519 | 0.296685 | 0.470105 |
| Cox8a | 260.2428 | 265.6057 | 245.8835 | 246.6271 | 248.6815 | 271.3129 | 244.9904 | 239.7615 |
| Mir325 | 0 | 0 | 0 | 0 | 0 | 0 | 0 | 0 |
| Wnt3a | 0 | 0 | 0 | 0.01834 | 0 | 0 | 0.044534 | 0 |
| RGD131159 | 2.606711 | 2.359496 | 2.526397 | 2.095061 | 2.192243 | 1.955429 | 2.405924 | 2.502995 |
| Rn50_20_( | 2.524439 | 3.149236 | 2.779637 | 3.280497 | 2.659648 | 2.383694 | 2.172485 | 2.524399 |
| Ube4a | 12.30297 | 11.76925 | 11.49179 | 11.64525 | 12.29858 | 12.39729 | 12.35015 | 12.33125 |
| AABR07063 | 0.17113 | 0.193626 | 0.183719 | 0 | 0 | 0 | 0 | 0 |
| Tspy26 | 0.202087 | 0.342979 | 0.433908 | 0.23277 | 0.245829 | 0.132893 | 0.141304 | 0.33585 |
| AABR07013 | 1.011902 | 1.717379 | 0.543171 | 1.398642 | 0.547077 | 1.330855 | 3.113195 | 1.345348 |
| Ubox5 | 4.852938 | 5.150729 | 5.471204 | 5.052201 | 4.752081 | 5.146238 | 4.564415 | 4.859694 |
| Pde6h | 0 | 0 | 0.121422 | 0.104218 | 0.122295 | 0.396669 | 0.253066 | 0 |
| Kel | 0.254991 | 0.288511 | 0.248863 | 0.234964 | 0.100261 | 0.162601 | 0.311207 | 0.246558 |
| Paqr8 | 1.408214 | 1.51922 | 1.933708 | 2.866822 | 2.797485 | 2.440732 | 3.444002 | 3.251048 |
| Flt3l | 11.58606 | 9.141856 | 9.001452 | 12.15108 | 12.52783 | 10.2255 | 13.64417 | 11.21515 |
| Klf13 | 9.829484 | 11.6137 | 14.56816 | 9.137637 | 10.25225 | 10.52525 | 14.01361 | 14.1865 |
| Tshr | 0.039754 | 0.02249 | 0.01067 | 0 | 0.064478 | 0.034856 | 0.022237 | 0.035236 |
| Clec1b | 0.874587 | 0.680319 | 0.528146 | 0.503686 | 0.768364 | 0.958549 | 0.550378 | 0.387595 |
| Adgrf5 | 62.77795 | 61.6004 | 59.89646 | 48.92502 | 49.92513 | 48.6024 | 60.90911 | 63.09984 |
| Birc7 | 0 | 0 | 0 | 0 | 0 | 0 | 0 | 0.054096 |
| AC098459. | 6.260342 | 6.499953 | 7.748776 | 9.501321 | 7.804509 | 8.97625 | 9.228527 | 6.462781 |
| Rab35 | 17.43023 | 19.63635 | 17.44702 | 16.24615 | 15.83695 | 18.47134 | 20.45413 | 20.73982 |
| Sprydd7 | 45.03775 | 48.28063 | 40.56558 | 47.86627 | 50.22565 | 47.75508 | 25.40662 | 26.18428 |
| LOC498675 | 0.204754 | 0.752927 | 0.439634 | 0.613187 | 0.498145 | 0.628351 | 0.744478 | 1.043533 |
| AABR07015 | 1.535157 | 0.771981 | 1.098726 | 1.100234 | 1.844382 | 2.392937 | 2.099125 | 1.663061 |

| | | | | | | | | |
|---|---|---|---|---|---|---|---|---|
| Gpr152 | 0.026772 | 0 | 0.057483 | 0.049339 | 0 | 0.023474 | 0 | 0.023729 |
| #N/A | 2.514748 | 1.00423 | 1.746892 | 2.726166 | 0.959704 | 3.112846 | 1.820427 | 2.491175 |
| Msantd2 | 5.472475 | 5.3378 | 4.4907 | 6.491713 | 6.121353 | 5.901321 | 6.826081 | 7.861198 |
| Chmp5 | 24.27875 | 24.05892 | 23.08352 | 28.44011 | 28.69732 | 29.77485 | 27.07322 | 30.2046 |
| Bbs2 | 6.14633 | 6.954284 | 7.4506 | 7.951547 | 8.648522 | 8.065817 | 7.695926 | 7.955228 |
| Tns2 | 43.86876 | 46.083 | 49.11565 | 44.52193 | 41.45312 | 45.49937 | 57.06534 | 58.24177 |
| Dopey2 | 3.101685 | 3.233013 | 3.075548 | 2.735299 | 2.873548 | 2.589748 | 3.453464 | 3.457796 |
| Slc25a45 | 3.079099 | 3.531909 | 2.986447 | 2.935106 | 3.099773 | 2.532186 | 3.706086 | 3.425565 |
| Scara5 | 5.983889 | 5.38207 | 5.698787 | 11.30734 | 11.85226 | 11.40004 | 15.81076 | 15.03156 |
| Col4a3bp | 9.156185 | 9.238315 | 8.818329 | 8.165776 | 8.807475 | 8.053965 | 9.343244 | 9.611702 |
| Aktip | 20.06746 | 21.52317 | 19.06931 | 20.61519 | 20.83469 | 20.99025 | 20.80031 | 19.88415 |
| LOC10255( | 0 | 0 | 0 | 0 | 0 | 0.015128 | 0 | 0.015293 |
| Acsm1 | 0 | 0.085333 | 0 | 0 | 0 | 0.022042 | 0.028125 | 0 |
| Vps25 | 3.281598 | 5.292962 | 3.97275 | 5.018326 | 4.454304 | 4.162913 | 5.467902 | 4.393908 |
| Scnn1b | 0 | 0.064146 | 0.060864 | 0.104482 | 0.040868 | 0.099418 | 0.147994 | 0.100501 |
| Pkp4 | 16.5778 | 16.3151 | 16.11689 | 15.39598 | 15.23269 | 15.54397 | 18.46691 | 17.69974 |
| Ptprs | 20.88651 | 20.24572 | 22.70989 | 17.61106 | 15.58015 | 16.10147 | 23.72565 | 22.82355 |
| Slc2a7 | 0.31012 | 0.350886 | 0.332933 | 0.163293 | 0.23952 | 0.038845 | 0.099128 | 0 |
| Kpnb1 | 18.36364 | 18.26923 | 16.25358 | 17.58528 | 18.22827 | 17.59151 | 19.85203 | 20.14926 |
| Slf2 | 3.777902 | 3.626034 | 3.301853 | 2.640645 | 2.924085 | 2.802857 | 3.802088 | 3.928098 |
| Zfp617 | 1.444293 | 1.307319 | 1.594841 | 1.939251 | 1.338594 | 1.374902 | 1.661976 | 1.828783 |
| AC140696. | 0.295853 | 1.673717 | 0.317617 | 0.272617 | 1.919407 | 2.075231 | 3.971842 | 1.311145 |
| Tbc1d13 | 6.893389 | 6.810554 | 6.824663 | 5.638075 | 5.176793 | 5.93962 | 6.245179 | 6.391681 |
| Unc5d | 0.123671 | 0.119938 | 0.170703 | 0.081399 | 0.171931 | 0.077454 | 0.138358 | 0.078297 |
| Klf12 | 3.189098 | 2.405543 | 2.629799 | 2.086852 | 2.148957 | 2.512531 | 3.050746 | 2.744724 |
| Ms4a4a | 0.073086 | 0.082693 | 0.039231 | 0 | 0 | 0.064082 | 0.081765 | 0.03239 |
| Ptges3 | 46.14767 | 46.42824 | 43.91726 | 42.78503 | 46.07619 | 41.23724 | 51.13357 | 54.27617 |
| Fth117e | 0.095384 | 0 | 0 | 0 | 0.103138 | 0 | 0 | 0 |
| Wfdc15b | 0 | 0 | 0.235716 | 0.10116 | 0.356116 | 0 | 0.122819 | 0.097305 |
| Nlrp1a | 2.59092 | 2.920442 | 2.225215 | 2.450495 | 2.230648 | 2.443172 | 3.193936 | 2.894408 |
| Rn60_20_( | 0.082078 | 0 | 0 | 0 | 0 | 0 | 0 | 0 |
| Morn5 | 0.33113 | 0.160568 | 0.304705 | 0.305124 | 0.511495 | 0.290336 | 0.211688 | 0.419282 |
| Lgr6 | 11.05864 | 12.06254 | 13.07489 | 9.141122 | 8.655546 | 8.587181 | 12.47299 | 12.42846 |
| Eif3d | 27.73127 | 27.46628 | 26.17974 | 27.97357 | 27.11543 | 27.00567 | 32.10712 | 30.51007 |
| Bub1b | 0.218138 | 0.174221 | 0.206634 | 0.189182 | 0.152621 | 0.213765 | 0.258398 | 0.193346 |
| Cdc25c | 0.379349 | 0.357679 | 0.254534 | 0.451508 | 0.307638 | 0.401908 | 0.335982 | 0.406285 |
| Cabp5 | 0 | 0 | 0 | 0 | 0 | 0 | 0 | 0 |
| Dhrs7c | 99.11792 | 98.45426 | 99.3945 | 62.62731 | 60.60301 | 62.29522 | 56.93729 | 55.02037 |
| Actr2 | 33.73996 | 30.05053 | 29.99424 | 32.50585 | 34.00741 | 32.41508 | 38.90443 | 37.5763 |
| RGD156275 | 0.522614 | 0.650444 | 1.178225 | 1.203921 | 1.017171 | 1.28304 | 0.76008 | 1.158047 |
| AY172581. | 5.312485 | 5.152137 | 7.332804 | 4.195925 | 4.923697 | 4.657991 | 4.245266 | 4.708719 |
| Skap1 | 0.722526 | 0.739646 | 0.886489 | 0.538964 | 0.372027 | 0.663679 | 0.692854 | 0.853881 |
| Nlrp6 | 0.018001 | 0 | 0 | 0 | 0 | 0 | 0 | 0 |
| Lrp1 | 16.1041 | 16.79959 | 17.8084 | 28.78332 | 27.5801 | 28.43053 | 40.31329 | 40.46208 |
| LOC300308 | 0.066052 | 0.044841 | 0.042547 | 0.08521 | 0.042853 | 0.08108 | 0.059117 | 0.11709 |
| Oscp1 | 1.902395 | 1.888132 | 1.89902 | 2.521834 | 1.768326 | 2.165502 | 1.829604 | 1.952427 |
| Acot11 | 12.81822 | 11.72062 | 13.12113 | 8.332135 | 9.266967 | 8.625302 | 11.75584 | 11.91189 |
| LOC103692 | 0 | 0 | 0 | 0.078588 | 0.092219 | 0 | 0 | 0 |
| Eif4ebp2 | 24.16198 | 27.47365 | 26.13228 | 17.49142 | 18.09719 | 18.63881 | 22.14092 | 23.64513 |
| Spg20 | 8.248977 | 7.938523 | 7.264856 | 7.202321 | 7.785064 | 7.278705 | 7.48264 | 8.13678 |
| RGD130953 | 2.861296 | 2.927962 | 2.507118 | 2.617185 | 2.638896 | 3.191324 | 2.447893 | 2.237743 |
| Rims1 | 1.374515 | 1.403187 | 1.287015 | 1.018963 | 0.949855 | 0.933332 | 0.98277 | 0.787774 |
| Sdhc | 365.4667 | 365.8197 | 362.137 | 340.5881 | 338.7979 | 336.7596 | 302.007 | 302.9073 |
| Slamf9 | 1.051072 | 1.35913 | 1.168693 | 2.870975 | 2.475966 | 2.36978 | 3.527679 | 3.793014 |

| | | | | | | | | |
|---|---|---|---|---|---|---|---|---|
| Rn50_X_06 | 0 | 0 | 0 | 0 | 0.056623 | 0.091829 | 0 | 0 |
| Mad212 | 11.58198 | 10.86863 | 10.96077 | 11.73452 | 11.45508 | 13.33156 | 11.23794 | 11.67661 |
| AABR07049 | 1.309148 | 0 | 0 | 0 | 0 | 0 | 0 | 0 |
| Zfp560 | 1.174929 | 1.03396 | 0.910983 | 1.03754 | 1.605687 | 1.001562 | 1.095382 | 1.345139 |
| Coro1c | 10.774 | 10.85316 | 10.85808 | 12.61483 | 12.08124 | 12.19183 | 14.26842 | 14.81401 |
| AABR07051 | 5.950673 | 7.780247 | 4.684847 | 6.823676 | 7.578266 | 8.695925 | 8.728523 | 6.212044 |
| Leo1 | 4.132061 | 4.405507 | 4.00949 | 4.191945 | 4.403496 | 4.110705 | 4.622766 | 4.842182 |
| Btg2 | 65.83553 | 64.91552 | 69.47178 | 71.19315 | 69.70625 | 69.55274 | 87.03093 | 87.5209 |
| Rasl10a | 15.11493 | 15.00548 | 16.75029 | 17.10276 | 13.81294 | 15.30483 | 17.78268 | 17.2866 |
| Mbtd1 | 2.793945 | 2.277936 | 2.205499 | 1.968743 | 2.199148 | 2.053453 | 2.206406 | 1.911936 |
| Macf1 | 15.50025 | 15.74481 | 15.53568 | 14.82649 | 15.38711 | 14.59039 | 17.36467 | 18.27598 |
| Vnn3 | 0 | 0.066312 | 0 | 0 | 0.063372 | 0.025694 | 0 | 0.051947 |
| Rida | 2.473981 | 2.266013 | 1.960363 | 2.388232 | 2.484002 | 2.943898 | 3.229076 | 2.871539 |
| Rai2 | 4.452329 | 5.060709 | 5.503416 | 4.29084 | 4.328398 | 5.551283 | 4.432693 | 4.308368 |
| Zc3h13 | 5.870998 | 5.562712 | 5.631792 | 5.511086 | 6.083335 | 5.806897 | 7.220307 | 7.180436 |
| Pkib | 0.596338 | 0.210853 | 0.200065 | 0.274751 | 0.322406 | 0.326793 | 0.625459 | 0.495529 |
| Mrpl47 | 11.50156 | 12.12549 | 10.40109 | 11.57078 | 11.58786 | 10.74897 | 9.415933 | 10.89002 |
| Pomp | 28.00554 | 24.44422 | 57.17263 | 32.11414 | 43.64452 | 26.89388 | 24.26937 | 45.54769 |
| Rabgef1 | 4.317502 | 3.417438 | 3.720027 | 3.483241 | 3.92711 | 3.899319 | 4.996074 | 4.385236 |
| Hus1 | 3.404881 | 3.369273 | 3.432319 | 3.679873 | 3.806451 | 3.835484 | 4.041658 | 4.327384 |
| Cd8a | 2.452295 | 2.134351 | 2.349174 | 2.190164 | 2.162099 | 1.753219 | 1.561695 | 1.571673 |
| Pnpla7 | 4.80146 | 5.275915 | 4.931635 | 4.573253 | 4.842304 | 4.624845 | 6.998653 | 5.718694 |
| Kifc2 | 1.116094 | 0.887378 | 1.165814 | 1.389779 | 1.190508 | 1.335646 | 1.839208 | 1.951763 |
| Ubiad1 | 3.741501 | 3.516397 | 3.611827 | 3.072297 | 2.724275 | 2.923394 | 3.240638 | 3.530231 |
| Erp27 | 0.052105 | 0.176864 | 0.055938 | 0.192052 | 0.056341 | 0.274116 | 0.291466 | 0.461836 |
| Coro1a | 12.90317 | 14.04352 | 12.83279 | 12.55366 | 14.30613 | 14.18496 | 12.78677 | 13.35252 |
| Mrpl53 | 4.94567 | 5.431211 | 70.2727 | 4.691277 | 4.718543 | 4.974069 | 4.231115 | 5.930744 |
| AABR07055 | 1.479263 | 3.682177 | 0.95285 | 2.726166 | 2.879111 | 0.778212 | 0.661974 | 2.097831 |
| AABR07049 | 0.143468 | 0.162328 | 0 | 0.1322 | 0 | 0.377379 | 0.321012 | 0 |
| Poll | 9.218228 | 9.345685 | 9.33295 | 10.23934 | 10.31295 | 10.72338 | 9.72582 | 10.41546 |
| Dpf1 | 0.044586 | 0.252233 | 0.143597 | 0.30813 | 0.337469 | 0.469114 | 0.673387 | 0.316148 |
| Fbxo8 | 15.30299 | 14.53843 | 13.69061 | 15.02333 | 14.52217 | 14.40843 | 14.98929 | 14.1075 |
| Trpm2 | 0.436862 | 0.559326 | 0.506025 | 0.720354 | 0.73342 | 0.675361 | 0.836005 | 1.090308 |
| Baz1a | 1.640019 | 1.609055 | 1.699103 | 1.764843 | 1.748527 | 1.548583 | 1.988748 | 2.277015 |
| Zic2 | 0 | 0.037334 | 0.035424 | 0.030405 | 0 | 0.028932 | 0.110746 | 0 |
| LOC100362 | 0 | 0.033474 | 0 | 0 | 0.03199 | 0.02594 | 0.165493 | 0.026223 |
| LOC685431 | 4.745913 | 4.414139 | 5.008684 | 6.522697 | 5.870998 | 5.0781 | 3.959639 | 5.489889 |
| AABR07057 | 0 | 0 | 0 | 0.040244 | 0 | 0 | 0 | 0 |
| Plxdc2 | 4.958967 | 5.264222 | 4.994886 | 10.3387 | 10.06162 | 10.0055 | 12.72369 | 11.84546 |
| Uap1 | 16.69469 | 17.46664 | 16.87295 | 29.09349 | 29.48198 | 30.94992 | 44.15341 | 44.43328 |
| Abcf3 | 28.43671 | 30.38862 | 30.43943 | 31.69663 | 33.10654 | 32.83769 | 31.04685 | 30.41523 |
| Ccl7 | 1.909816 | 1.951751 | 1.388919 | 3.462872 | 3.463965 | 4.051278 | 2.756925 | 2.402634 |
| AABR07013 | 0 | 0 | 0 | 0 | 0.442363 | 0 | 0 | 0 |
| Fnbp1l | 6.055071 | 6.284046 | 5.828039 | 5.868105 | 4.786273 | 5.345706 | 7.568354 | 7.032503 |
| AC135306. | 0 | 0 | 0 | 0 | 0 | 0 | 0 | 0 |
| Ybx3 | 64.89982 | 70.44551 | 74.73272 | 52.71083 | 52.92014 | 55.22408 | 66.3163 | 65.45923 |
| AABR07013 | 0.440791 | 0 | 0.094643 | 0.487405 | 0.285972 | 0.463783 | 0.197255 | 0.390695 |
| Sfpq | 29.58792 | 29.20209 | 26.60794 | 25.57239 | 24.53719 | 24.83837 | 31.95527 | 31.95847 |
| Derl1 | 16.50768 | 17.45536 | 16.37916 | 18.26738 | 17.21423 | 18.21291 | 19.3798 | 19.23439 |
| Dcaf17 | 1.695112 | 1.770406 | 1.73582 | 1.61004 | 2.086686 | 1.669202 | 2.042294 | 2.080332 |
| Hcrtr1 | 0 | 0.024193 | 0.022956 | 0.05911 | 0 | 0.074993 | 0.047844 | 0.037905 |
| AABR07053 | 1.456739 | 1.821729 | 1.31697 | 1.554274 | 2.072566 | 1.411719 | 2.573265 | 1.630964 |
| RGD130458 | 11.54948 | 11.14735 | 12.13246 | 10.9094 | 11.63783 | 11.65103 | 10.55913 | 11.37431 |
| Vwc2l | 0 | 0 | 0 | 0 | 0 | 0.033539 | 0 | 0 |

| | | | | | | | | |
|---|---|---|---|---|---|---|---|---|
| AABR07044 | 101.0529 | 94.67533 | 99.24312 | 79.33998 | 70.33379 | 81.60287 | 81.353 | 92.06587 |
| Mettl17 | 4.727895 | 4.604538 | 5.589691 | 5.624937 | 6.27701 | 5.614686 | 5.724559 | 5.065813 |
| Fbxl8 | 1.255713 | 1.783533 | 2.09384 | 2.240324 | 2.484457 | 2.202021 | 2.570552 | 2.083917 |
| Skint10 | 0.138994 | 0.039316 | 0 | 0 | 0.037573 | 0.091402 | 0.116625 | 0 |
| Tlk2 | 6.506672 | 6.735098 | 6.695543 | 6.846552 | 6.759063 | 7.274569 | 6.67541 | 6.636042 |
| Lpin1 | 24.33254 | 23.34381 | 24.4335 | 15.94709 | 14.32478 | 15.04879 | 17.28138 | 18.30275 |
| AABR07049 | 0.234825 | 0 | 0 | 0 | 0 | 0 | 0.525423 | 0 |
| Ucp1 | 0.043711 | 0 | 0 | 0 | 0 | 0.038326 | 0 | 0 |
| F8a1 | 18.04291 | 18.69393 | 20.85453 | 20.06566 | 20.07016 | 18.48702 | 18.98685 | 18.25944 |
| Dll4 | 10.50533 | 10.58724 | 10.83441 | 10.49487 | 9.782622 | 10.37883 | 11.02075 | 11.92673 |
| LOC682419 | 0 | 0 | 0 | 0 | 0 | 0 | 0 | 0.018053 |
| Ppp3cc | 24.48228 | 24.61331 | 23.91579 | 20.52739 | 23.27949 | 21.13836 | 24.79708 | 22.76001 |
| Cog7 | 5.125092 | 4.603628 | 4.36809 | 4.326019 | 5.457082 | 4.613745 | 3.982969 | 5.114786 |
| Lrrc42 | 4.752122 | 5.132403 | 4.836683 | 5.630006 | 5.338599 | 5.817097 | 5.834301 | 5.825747 |
| Ak9 | 0.007947 | 0 | 0 | 0 | 0 | 0.006968 | 0 | 0 |
| Lasl1 | 13.43457 | 13.44352 | 13.14342 | 15.20806 | 13.9018 | 12.15942 | 13.65629 | 14.30848 |
| AABR07021 | 0.050709 | 0.114751 | 0.127026 | 0.077878 | 0.12794 | 0.059283 | 0.094552 | 0.089893 |
| Bpgm | 16.59644 | 15.17681 | 12.6918 | 16.48009 | 13.93029 | 14.15309 | 11.78476 | 14.87817 |
| Dlg3 | 1.537377 | 1.631874 | 1.497336 | 1.927789 | 2.313571 | 2.181772 | 2.446938 | 2.093149 |
| Fam180a | 0.210546 | 0.340319 | 0.290617 | 3.46447 | 2.309132 | 2.689993 | 2.321851 | 2.212754 |
| Klrg2 | 0.760872 | 1.114095 | 1.201243 | 1.360986 | 1.54865 | 1.020322 | 1.752533 | 2.578584 |
| AC099294. | 3.491061 | 0 | 0 | 4.503626 | 2.2649 | 3.673159 | 3.905644 | 2.475441 |
| Myoz1 | 1.959994 | 1.880174 | 1.738234 | 3.298017 | 3.363257 | 4.33367 | 3.956491 | 3.172351 |
| Mcur1 | 10.8254 | 9.787022 | 10.62084 | 9.784266 | 9.969147 | 8.49259 | 10.08282 | 9.824636 |
| Ift43 | 39.36862 | 40.84953 | 40.3099 | 53.69174 | 54.17838 | 47.67619 | 52.40307 | 52.31373 |
| Comtd1 | 2.836109 | 2.541468 | 3.434472 | 4.160474 | 5.151962 | 4.615321 | 4.162862 | 4.444371 |
| Klhl25 | 1.007037 | 1.175587 | 1.149761 | 1.44347 | 1.434575 | 1.303433 | 1.931363 | 1.586821 |
| RGD156407 | 0.03117 | 0.035268 | 0.033463 | 0.057444 | 0 | 0.02733 | 0 | 0 |
| Nlgn2 | 3.592827 | 4.465308 | 5.595835 | 5.712156 | 5.716599 | 5.810721 | 8.809568 | 7.953003 |
| Lrp10 | 23.79942 | 25.54387 | 28.58223 | 25.80905 | 23.7694 | 24.35313 | 26.84711 | 28.17398 |
| Csrp1 | 57.76756 | 56.80489 | 58.44111 | 76.29249 | 76.64319 | 76.09438 | 73.96104 | 76.09853 |
| Nr1h3 | 5.02841 | 6.015553 | 6.257924 | 7.289617 | 6.233671 | 6.964399 | 8.599584 | 7.295738 |
| Kat2a | 6.094236 | 7.573254 | 8.325213 | 8.029044 | 8.533174 | 8.120215 | 10.20779 | 10.606 |
| Armcx3 | 17.33063 | 18.39558 | 17.24024 | 19.27828 | 18.90113 | 19.43713 | 20.44884 | 20.84233 |
| Cyb561a3 | 3.45077 | 3.286741 | 2.909279 | 3.323466 | 3.773429 | 3.829056 | 3.162612 | 3.006748 |
| AABR07032 | 0 | 0 | 0.453372 | 0 | 0 | 0 | 0 | 0.374311 |
| Srsf2 | 65.23014 | 67.9765 | 74.90826 | 78.25587 | 76.96611 | 72.19274 | 88.13895 | 86.603 |
| Alx4 | 0.089924 | 0 | 0.096539 | 0.49717 | 0.097234 | 0.210255 | 0.368879 | 0.345386 |
| RGD156366 | 1.152709 | 0.745278 | 1.06072 | 1.213915 | 1.157378 | 1.010696 | 0.921143 | 0.729788 |
| Ppme1 | 9.0716 | 8.80474 | 8.169634 | 8.63644 | 8.437591 | 8.670223 | 9.475517 | 9.58395 |
| Zfp110 | 8.971561 | 9.143009 | 8.561373 | 10.24084 | 9.654953 | 10.39538 | 9.301413 | 9.493451 |
| Slc23a1 | 0.046965 | 0.053139 | 0 | 0.021638 | 0.050783 | 0.061768 | 0.105085 | 0.124882 |
| Dph3 | 9.578344 | 8.443339 | 8.531566 | 7.843739 | 8.785047 | 7.987591 | 8.962984 | 7.888464 |
| Tsen2 | 1.725807 | 1.311078 | 1.005786 | 1.385801 | 1.439555 | 1.318636 | 1.048125 | 1.245587 |
| Ccdc51 | 8.363341 | 8.960916 | 8.604474 | 6.976709 | 8.871888 | 7.388538 | 8.257851 | 7.721714 |
| Ubxn7 | 5.598483 | 6.33442 | 4.781698 | 5.728813 | 4.013409 | 3.362904 | 5.052192 | 4.359072 |
| Msh6 | 3.888677 | 4.26489 | 4.123519 | 3.462355 | 3.430886 | 3.73382 | 3.923438 | 3.816775 |
| Nptx1 | 0.110898 | 0 | 0.10715 | 0.051094 | 0.059956 | 0.077788 | 0.099254 | 0.068806 |
| #N/A | 3.437145 | 3.067502 | 3.275611 | 3.446653 | 3.974904 | 3.30378 | 3.907015 | 4.480166 |
| Nol8 | 0.256948 | 0.232579 | 0.137925 | 0.14206 | 0.013892 | 0.090117 | 0.043119 | 0.011387 |
| Zglp1 | 0.849884 | 0.295878 | 0 | 0.120482 | 0.212069 | 0.229286 | 0.219418 | 0.115891 |
| Dnaja3 | 60.50828 | 63.82076 | 63.65831 | 66.66665 | 63.4777 | 64.17128 | 57.43731 | 59.69155 |
| AABR07010 | 0 | 0 | 0 | 0 | 0.460346 | 0.373289 | 0 | 0 |
| Slc7a9 | 0.061211 | 0 | 0.032857 | 0.028202 | 0.066186 | 0.026835 | 0.06848 | 0.054254 |

| | | | | | | | | |
|---|---|---|---|---|---|---|---|---|
| AABR07057 | 0.284597 | 0.644017 | 0.68745 | 0.524491 | 0.538529 | 0.249535 | 0.557191 | 0.252253 |
| LOC691110 | 0 | 0 | 0.026431 | 0 | 0 | 0 | 0 | 0 |
| AC120096. | 1.309148 | 0.493746 | 1.405454 | 0.804219 | 2.831126 | 0.765241 | 0.488206 | 2.707514 |
| AC139445. | 0.251155 | 0.248649 | 0.067408 | 0.202501 | 0.169732 | 0.110107 | 0.351227 | 0.222612 |
| AABR07017 | 0.047049 | 0 | 0 | 0 | 0.050874 | 0 | 0.052637 | 0.125107 |
| Traf6 | 2.456768 | 1.969879 | 2.118305 | 2.067737 | 2.196293 | 1.696139 | 2.618672 | 2.486186 |
| Stoml3 | 0 | 0 | 0 | 0 | 0 | 0 | 0 | 0 |
| Ephx4 | 0.044378 | 0.150635 | 0.142928 | 0.32714 | 0.383881 | 0.622569 | 0.297888 | 0.511346 |
| Gpatch11 | 2.931026 | 3.079438 | 3.118549 | 3.291631 | 3.735218 | 3.636905 | 3.454768 | 3.409762 |
| Szt2 | 2.68378 | 2.735343 | 2.890429 | 2.603572 | 2.405122 | 2.345611 | 3.026514 | 3.151392 |
| AABR07021 | 0 | 0 | 0 | 0 | 0 | 0 | 0.443823 | 0 |
| AABR07030 | 0.053626 | 0.030338 | 0 | 0 | 0 | 0 | 0 | 0 |
| Actr5 | 3.40526 | 2.578707 | 3.022482 | 2.890741 | 2.783288 | 2.962225 | 3.089719 | 3.279674 |
| Eif2b1 | 12.27261 | 12.85383 | 13.66506 | 15.66412 | 13.35986 | 13.41445 | 14.65776 | 14.62627 |
| Fbxo39 | 0.425207 | 0.444094 | 0.280915 | 0.512369 | 0.495138 | 0.487537 | 0.695258 | 0.782756 |
| Kif21a | 3.011498 | 3.034903 | 3.128321 | 2.460404 | 2.399371 | 2.779457 | 2.932643 | 2.463914 |
| AABR07057 | 0.594424 | 0.512429 | 0.820481 | 0.365159 | 0.306068 | 0.54601 | 0.12667 | 0.150534 |
| AABR07062 | 0.065132 | 0 | 0.069923 | 0.060016 | 0 | 0.057108 | 0.072867 | 0 |
| Creb312 | 11.59937 | 11.30134 | 12.55149 | 13.51597 | 12.95698 | 13.30484 | 17.86892 | 18.2095 |
| AABR07065 | 0 | 0 | 0 | 0.011397 | 0 | 0.010844 | 0 | 0.010962 |
| LOC100911 | 0.149191 | 0 | 0 | 0 | 0 | 0 | 0 | 0 |
| Endog | 40.50107 | 40.42497 | 42.81008 | 43.15655 | 41.3817 | 41.88621 | 38.42436 | 41.67027 |
| AABR07049 | 0 | 0.569707 | 0 | 0 | 0 | 0 | 0 | 0 |
| AABR07045 | 0 | 0.348527 | 0 | 0 | 0 | 0.270085 | 0.344616 | 0 |
| AABR07062 | 0 | 0 | 0.525403 | 0 | 0 | 0 | 0 | 0 |
| Oca2 | 2.431487 | 1.610407 | 1.84635 | 1.293308 | 1.090128 | 1.0053 | 1.172136 | 1.051291 |
| C6 | 1.256363 | 0.987164 | 1.180188 | 1.96164 | 1.566034 | 1.468774 | 2.498779 | 3.278866 |
| Prrc2a | 58.10666 | 65.87256 | 69.65821 | 55.49682 | 53.96395 | 54.21133 | 69.48649 | 69.17693 |
| AABR07005 | 0.727304 | 1.645822 | 0.780808 | 0 | 0.786424 | 1.275402 | 0 | 1.289292 |
| Bri3 | 100.9855 | 111.4344 | 113.9557 | 99.9584 | 90.83007 | 90.95302 | 109.625 | 104.5618 |
| AABR07014 | 0 | 0 | 0 | 0 | 0 | 0 | 0 | 0 |
| Pcdhb3 | 0.327927 | 0.301466 | 0.220032 | 0.566573 | 0.576198 | 0.610995 | 0.458588 | 0.544985 |
| Reep3 | 8.160643 | 7.418378 | 7.420243 | 8.829213 | 9.359478 | 8.977113 | 9.659725 | 9.294355 |
| Fadd | 4.455835 | 2.944633 | 3.979298 | 4.578234 | 5.15913 | 3.837732 | 4.102691 | 4.753294 |
| Abcb9 | 0.684207 | 0.68692 | 0.889724 | 0.63935 | 0.489742 | 0.642161 | 0.689992 | 0.802901 |
| Cnga2 | 0 | 0.022701 | 0 | 0 | 0 | 0 | 0 | 0 |
| AABR07051 | 0 | 0 | 0 | 0.216868 | 0.508966 | 0.619072 | 0 | 0 |
| Il1r2 | 0.109171 | 0.123522 | 0 | 0.033532 | 0.354137 | 0.191443 | 0.20356 | 0.387056 |
| AC121165. | 1.126149 | 0 | 0.604496 | 0 | 0 | 0 | 0 | 0 |
| Tmem81 | 0.545813 | 0.363271 | 0.413622 | 0.41419 | 0.659612 | 0.422267 | 0.466953 | 0.426866 |
| LOC360919 | 0.111559 | 0.151469 | 0.047906 | 0.123357 | 0.168878 | 0.156504 | 0.074885 | 0.138433 |
| Chrna5 | 0.053253 | 0 | 0.019057 | 0 | 0.019194 | 0.031128 | 0 | 0 |
| AABR07065 | 0 | 0 | 0 | 0.160844 | 0 | 0 | 0.195282 | 0 |
| Fam155b | 0.013417 | 0.045542 | 0.072019 | 0.074179 | 0.043522 | 0.070583 | 0.075051 | 0.083244 |
| AABR07044 | 0 | 0 | 0 | 0 | 0 | 0 | 0 | 0 |
| Pcdhb12 | 0.262815 | 0.223022 | 0.329174 | 0.221993 | 0.568356 | 0.460873 | 0.56355 | 0.465892 |
| Zfp609 | 3.56294 | 3.982222 | 3.499083 | 3.363045 | 2.887741 | 3.064226 | 4.013788 | 4.025777 |
| Tnfrsf11b | 0.830632 | 0.755943 | 0.620338 | 1.530789 | 1.386275 | 1.773249 | 1.313105 | 1.21638 |
| Tarm1 | 0 | 0.068339 | 0.129684 | 0.111311 | 0 | 0 | 0.067572 | 0 |
| AABR07004 | 0.745422 | 0.773126 | 0.822488 | 0.629638 | 0.6269 | 0.544656 | 0.694955 | 0.44047 |
| Thsd7b | 0.050693 | 0.057357 | 0.021769 | 0.018685 | 0.010963 | 0.00889 | 0.011343 | 0.053918 |
| Nox4 | 1.256447 | 1.311041 | 1.213989 | 1.80097 | 1.690675 | 1.321985 | 1.28071 | 1.52199 |
| Cdc14b | 2.897553 | 2.969606 | 2.885295 | 3.056935 | 2.769826 | 2.540577 | 3.053732 | 2.959065 |
| Sntb1 | 10.91843 | 11.8351 | 10.8781 | 10.87543 | 11.22184 | 11.52527 | 9.083462 | 9.590496 |

| | | | | | | | | |
|---|---|---|---|---|---|---|---|---|
| Akt3 | 6.74182 | 7.099376 | 5.947874 | 6.027798 | 7.109391 | 5.969761 | 9.110681 | 8.312606 |
| Wnt10a | 0 | 0 | 0 | 0.019583 | 0 | 0.037268 | 0.095105 | 0 |
| Ripk3 | 1.96176 | 2.219639 | 2.330723 | 2.603066 | 2.658599 | 2.591577 | 3.072622 | 2.944378 |
| Mcpt1 | 0 | 0 | 0.057016 | 0.29363 | 0.172279 | 0.046566 | 0.059416 | 0.235368 |
| Akirin2 | 24.9729 | 23.05693 | 24.17419 | 24.11041 | 23.39993 | 21.12743 | 26.83986 | 28.60104 |
| AABR07057 | 0 | 0.481704 | 0 | 0 | 0 | 0 | 0 | 0 |
| Nudcd1 | 2.268863 | 1.980796 | 1.518597 | 1.922898 | 2.104982 | 1.633225 | 1.801882 | 2.048247 |
| Ocrl | 1.867086 | 1.930975 | 1.628604 | 1.935502 | 1.766496 | 1.637062 | 2.447827 | 1.88761 |
| AABR07027 | 0 | 0 | 0 | 0 | 0 | 0 | 0 | 0 |
| Brix1 | 5.49862 | 5.031048 | 4.837575 | 6.017923 | 5.602152 | 5.378156 | 5.50758 | 5.647862 |
| Syt2 | 0.033883 | 0 | 0 | 0 | 0.036637 | 0 | 0 | 0 |
| Odf2 | 8.967921 | 8.282654 | 8.723161 | 8.280838 | 8.381023 | 8.585369 | 9.299818 | 9.873664 |
| Dhcr7 | 0.780355 | 0.90617 | 0.66139 | 0.983986 | 0.821582 | 1.080341 | 0.73518 | 1.055703 |
| Sgo2 | 0.066947 | 0.212093 | 0.114995 | 0.098702 | 0.086867 | 0.211317 | 0.134815 | 0.094941 |
| Vsig1 | 0 | 0 | 0.035491 | 0 | 0 | 0 | 0 | 0 |
| Rad21 | 22.45726 | 22.22692 | 19.76173 | 19.94716 | 18.59817 | 20.24576 | 21.89511 | 22.65906 |
| Dclre1c | 1.501848 | 1.274453 | 1.422644 | 1.872327 | 2.698585 | 2.439994 | 2.223796 | 2.603598 |
| Tmem178b | 0.010931 | 0.006184 | 0 | 0 | 0.00591 | 0.009584 | 0.006115 | 0.024222 |
| Gpc4 | 14.92734 | 13.18518 | 14.49638 | 12.34026 | 12.20054 | 11.66108 | 11.85765 | 11.96842 |
| Scn4a | 0.391296 | 0.459761 | 0.508944 | 0.312026 | 0.252234 | 0.250719 | 0.294649 | 0.266788 |
| B230307C2 | 2.181913 | 4.320282 | 2.342423 | 1.759229 | 0.884727 | 1.434828 | 2.135899 | 1.933938 |
| RT1-DMb | 0.568422 | 1.045108 | 1.296755 | 0.589251 | 0.46097 | 0.934487 | 0.794907 | 0.944665 |
| LOC100909 | 0.290922 | 0 | 0 | 0.134036 | 0 | 0.25508 | 0.32547 | 0 |
| Chrd | 0.961725 | 1.28764 | 1.307799 | 2.584726 | 2.131793 | 2.234589 | 2.54638 | 2.372582 |
| Msra | 11.13839 | 11.81491 | 10.9613 | 10.33487 | 12.62925 | 10.71564 | 10.47082 | 10.96946 |
| Rn60_10_( | 24.05741 | 20.11425 | 21.98939 | 17.80558 | 15.77491 | 19.90757 | 23.99593 | 21.66582 |
| Lum | 52.88568 | 49.48736 | 49.1316 | 104.2172 | 104 | 103.2221 | 102.3996 | 99.69332 |
| AABR07051 | 0 | 0 | 0 | 0.115715 | 0 | 0.220213 | 0 | 0 |
| LOC103690 | 0.49483 | 0.486849 | 0.369552 | 0.51544 | 0.255895 | 0.320685 | 0.409178 | 0.438592 |
| Proser1 | 3.462698 | 3.222556 | 3.235304 | 2.853153 | 3.002999 | 2.9532 | 3.728476 | 3.519584 |
| Meioc | 0 | 0 | 0.024881 | 0 | 0 | 0.01016 | 0 | 0 |
| Gfra1 | 3.200335 | 3.919465 | 4.3419 | 4.698929 | 4.087925 | 4.378682 | 5.685349 | 5.470616 |
| AABR07063 | 0 | 0 | 0 | 0 | 0 | 0.12443 | 0 | 0 |
| AC093995. | 0 | 0 | 0 | 0.096894 | 0.1137 | 0.092198 | 0.11764 | 0 |
| AABR07057 | 0 | 0 | 0 | 0 | 0 | 0.027477 | 0 | 0 |
| Plppr2 | 1.021774 | 1.218023 | 1.116528 | 1.529977 | 1.440224 | 1.215854 | 1.735086 | 2.005366 |
| RGD156024 | 0 | 0 | 0.064917 | 0.05572 | 0.32692 | 0 | 0.338249 | 0.053596 |
| Gp1ba | 0.293421 | 0.376258 | 0.378008 | 0.378527 | 0.253818 | 0.154363 | 0.503342 | 0.433456 |
| Tmem97 | 2.616186 | 2.052967 | 2.400937 | 3.227244 | 3.467616 | 3.514808 | 2.738042 | 2.954671 |
| AABR07002 | 0 | 0 | 0 | 0 | 0 | 0.040668 | 0 | 0 |
| RGD156034 | 41.25992 | 40.9198 | 36.07474 | 39.62297 | 42.73204 | 39.72782 | 45.2394 | 45.29272 |
| Pgr | 0 | 0 | 0 | 0 | 0 | 0.015015 | 0.038316 | 0.07589 |
| AC135400. | 63.8151 | 64.55868 | 72.74106 | 67.62357 | 62.9139 | 50.35782 | 60.26451 | 60.38878 |
| AABR07043 | 0.508407 | 0 | 0 | 0.312318 | 0.366489 | 0.148591 | 0.568783 | 0.150209 |
| Ssbp1 | 33.90472 | 33.74213 | 35.73188 | 29.44259 | 30.61454 | 30.97282 | 29.98741 | 28.71407 |
| AC099089. | 47.81236 | 52.8094 | 49.70011 | 56.99465 | 48.41635 | 38.9275 | 54.3394 | 47.75987 |
| LOC100363 | 0 | 0 | 0 | 0 | 0 | 0 | 0 | 0 |
| Zkscan4 | 0.881581 | 1.147088 | 0.662504 | 1.340365 | 1.096227 | 1.159457 | 1.578038 | 1.44557 |
| Psmd12 | 45.42105 | 42.96196 | 41.62086 | 44.06041 | 44.73984 | 43.79141 | 42.33099 | 43.8438 |
| AABR07010 | 3.337505 | 3.244366 | 2.523257 | 1.992499 | 3.100515 | 2.843895 | 3.102779 | 2.916532 |
| Grm7 | 0 | 0 | 0 | 0 | 0 | 0 | 0 | 0 |
| Rnpepl1 | 33.46894 | 36.871 | 39.87775 | 30.44178 | 29.67825 | 31.54798 | 39.06265 | 39.6912 |
| Ppp5c | 35.38651 | 34.58629 | 34.76001 | 30.84536 | 31.28849 | 31.38459 | 35.5387 | 35.45491 |
| Cxxc4 | 2.241191 | 2.260172 | 2.437445 | 1.984359 | 1.896549 | 1.87964 | 1.787846 | 1.563273 |

| | | | | | | | | |
|---|---|---|---|---|---|---|---|---|
| Impad1 | 0 | 0 | 0 | 0.011897 | 0 | 0.01132 | 0 | 0 |
| Tmem67 | 1.305271 | 0.965072 | 0.998941 | 1.107488 | 1.075995 | 1.257776 | 1.113282 | 1.111108 |
| Foxred1 | 17.06493 | 16.37408 | 15.50639 | 16.36701 | 16.61289 | 16.47836 | 15.28567 | 16.18824 |
| Sgms2 | 0.940043 | 1.032332 | 1.365383 | 1.93624 | 1.704057 | 1.939366 | 1.360995 | 1.519377 |
| LOC100911 | 666.4451 | 705.0951 | 705.2283 | 660.5159 | 643.1214 | 595.3319 | 590.6873 | 573.003 |
| Lrpap1 | 9.869369 | 9.009292 | 9.988864 | 12.31545 | 11.05342 | 10.88263 | 11.77224 | 12.3313 |
| LOC102548 | 0.087277 | 0.296248 | 0.187394 | 0.643375 | 0.377483 | 0.535669 | 0.585847 | 0.154715 |
| Cytip | 3.2414 | 2.955357 | 2.500087 | 3.102816 | 2.586124 | 2.648913 | 2.851778 | 2.064107 |
| Rasip1 | 33.8534 | 37.92176 | 40.95619 | 34.51094 | 29.11389 | 32.26637 | 35.98629 | 37.80151 |
| AC119459. | 0.748085 | 0.846423 | 1.606233 | 0.689331 | 2.426679 | 0.655921 | 1.673848 | 1.326129 |
| Rnf123 | 17.70339 | 17.94605 | 19.33318 | 16.85262 | 15.60681 | 16.6777 | 18.68649 | 18.92484 |
| AABR07013 | 0 | 0 | 0 | 0.099903 | 0 | 0 | 0 | 0 |
| 43345 | 48.08278 | 48.10895 | 44.25573 | 54.3774 | 56.62251 | 55.45422 | 54.21514 | 57.04724 |
| Etv5 | 2.848749 | 2.691089 | 2.668812 | 3.20697 | 3.719621 | 3.746679 | 4.449849 | 3.954226 |
| Ranbp3 | 29.47564 | 27.6923 | 34.74411 | 34.98109 | 32.06197 | 32.42112 | 35.93088 | 36.55104 |
| Slc9a8 | 8.508243 | 7.861326 | 8.898592 | 7.907404 | 7.275518 | 6.861522 | 7.991298 | 8.51365 |
| LOC100362 | 0 | 0 | 0.096595 | 0.041455 | 0 | 0 | 0 | 0 |
| RGD156078 | 0.471765 | 0.711707 | 1.181763 | 0.724522 | 0.566792 | 0.735367 | 0.52779 | 0.557532 |
| Cd9912 | 0.021639 | 0.048967 | 0.046461 | 0.119636 | 0.046795 | 0.056919 | 0.072626 | 0.095898 |
| AABR07060 | 0 | 0.011556 | 0.02193 | 0.009412 | 0.022088 | 0.008955 | 0 | 0 |
| Tbk1 | 11.16742 | 10.87003 | 10.50209 | 11.46961 | 10.82406 | 11.94361 | 11.68947 | 11.21904 |
| LOC103693 | 0.071053 | 0.160786 | 0.07628 | 0.130945 | 0.076828 | 0.140173 | 0.158981 | 0.157444 |
| 5S_rRNA | 0 | 0.524333 | 0.497506 | 1.281057 | 0 | 0 | 0 | 0 |
| Syt8 | 0.980329 | 1.06298 | 0.92089 | 0.677501 | 0.530008 | 0.573036 | 0.868259 | 0.868915 |
| Glce | 3.826783 | 3.702314 | 3.655788 | 3.863522 | 3.694076 | 3.754076 | 4.244007 | 4.040747 |
| Ap3b1 | 9.73437 | 9.022993 | 9.780567 | 9.993321 | 9.918386 | 10.18741 | 11.51867 | 11.37134 |
| LOC103689 | 0 | 0 | 0 | 0 | 0 | 0.012406 | 0 | 0 |
| Elfn2 | 0.585912 | 0.580066 | 0.805925 | 0.523023 | 0.554346 | 0.529783 | 0.573556 | 0.503094 |
| AABR07020 | 0.480421 | 0 | 0 | 0 | 0 | 0 | 0 | 0 |
| Zdbf2 | 0.028982 | 0.043723 | 0.046671 | 0.017804 | 0.010446 | 0 | 0.005404 | 0.025688 |
| Fut4 | 1.638913 | 1.749672 | 1.532449 | 1.461478 | 1.300517 | 1.541299 | 2.188423 | 2.178975 |
| Lef1 | 0.243751 | 0.25281 | 0.196262 | 0.243325 | 0.109819 | 0.195911 | 0.227249 | 0.162037 |
| Mob3b | 0.276702 | 0.313076 | 0.346567 | 0.276218 | 0.398926 | 0.121306 | 0.361156 | 0.163503 |
| AABR07006 | 0.124681 | 0 | 0 | 0 | 0 | 0 | 0 | 0.110511 |
| Hectd2 | 3.328531 | 2.796217 | 3.023638 | 3.3133 | 3.575018 | 3.875011 | 4.944327 | 4.22309 |
| LOC100363 | 0 | 0 | 0 | 0 | 0 | 0.097483 | 0 | 0 |
| Copb1 | 14.36448 | 14.75905 | 13.36278 | 15.65475 | 16.97656 | 16.64607 | 16.47474 | 16.81342 |
| Atp2b1 | 9.653631 | 8.490638 | 8.488052 | 8.605876 | 8.752976 | 8.155718 | 9.393797 | 9.158121 |
| Pef1 | 11.8135 | 13.19008 | 11.57826 | 13.72917 | 12.67266 | 13.6377 | 15.20412 | 12.98501 |
| Pop1 | 0.781814 | 0.530752 | 0.503597 | 0.547512 | 0.676292 | 0.562106 | 0.629755 | 0.568228 |
| Mamdc2 | 4.4792 | 4.054403 | 3.08923 | 6.0035 | 7.661211 | 6.878842 | 6.317061 | 7.194371 |
| 5S_rRNA | 2.200249 | 0 | 0 | 0.405489 | 0 | 0.385836 | 1.969232 | 0.390038 |
| AABR07027 | 0.524533 | 0.692399 | 0.84468 | 0.644449 | 0.56717 | 0.229956 | 1.173649 | 1.472247 |
| Ate1 | 4.189274 | 8.414358 | 4.462589 | 9.336421 | 8.304635 | 4.712463 | 3.342505 | 5.641703 |
| Cyp11b2 | 0 | 0.032862 | 0 | 0 | 0 | 0 | 0 | 0 |
| AABR07050 | 0 | 0 | 0 | 0 | 0 | 0 | 0 | 0.021448 |
| Suclg1 | 185.6418 | 179.7689 | 180.1806 | 170.2606 | 170.4264 | 174.2031 | 146.1068 | 147.0938 |
| AABR07042 | 0.846048 | 0.604588 | 0.52585 | 0.492379 | 0.433336 | 0.624687 | 0.348718 | 0.789363 |
| Mtx3 | 2.458922 | 2.679111 | 2.150956 | 2.601473 | 3.643536 | 2.754869 | 2.801875 | 3.107755 |
| AABR07005 | 1.139516 | 1.582333 | 1.556982 | 1.384116 | 1.120129 | 1.498692 | 2.549679 | 2.662752 |
| AABR07033 | 0 | 0 | 0.040127 | 0.103326 | 0 | 0 | 0.041816 | 0 |
| Ash2l | 16.81197 | 16.75932 | 14.55381 | 16.04867 | 13.97963 | 15.51551 | 16.80537 | 16.40584 |
| AABR07019 | 38.00309 | 38.95543 | 50.31599 | 36.60146 | 36.93096 | 31.39297 | 51.35769 | 47.87639 |
| Alg3 | 8.836509 | 9.954624 | 11.09515 | 7.859278 | 7.851544 | 9.02794 | 10.18677 | 9.943536 |

| | | | | | | | | |
|---|---|---|---|---|---|---|---|---|
| Ift88 | 1.408304 | 1.333278 | 1.326773 | 1.112312 | 1.802473 | 2.016004 | 1.83278 | 1.502995 |
| Mxd1 | 3.050442 | 3.020003 | 3.634581 | 3.8117 | 3.535784 | 3.920765 | 3.774652 | 4.75206 |
| Dmkn | 0.033698 | 0.076254 | 0.036176 | 0.031051 | 0 | 0.059092 | 0 | 0.059736 |
| Acadsb | 36.91284 | 24.91967 | 25.90445 | 16.60476 | 29.97662 | 27.5712 | 25.12823 | 24.93643 |
| Gcsh | 39.26394 | 35.35019 | 38.6359 | 42.91317 | 44.22641 | 40.50195 | 35.61121 | 35.546 |
| Aida | 10.5202 | 10.17417 | 11.25205 | 13.15995 | 12.94289 | 12.83132 | 12.25171 | 15.19368 |
| Prkcg | 1.097641 | 1.06997 | 1.087743 | 0.793586 | 0.566043 | 0.755124 | 1.001286 | 0.942959 |
| Fmnl3 | 13.98029 | 14.07911 | 14.15394 | 13.76955 | 14.19567 | 13.76785 | 14.95691 | 16.10065 |
| AABR07044 | 0 | 0 | 0 | 0 | 0 | 0 | 0 | 0.02921 |
| AC116283. | 0 | 0 | 0 | 0 | 0 | 0 | 0.286478 | 0 |
| Gkap1 | 12.9117 | 12.09401 | 12.66839 | 12.40967 | 11.69916 | 12.46742 | 13.93306 | 14.10978 |
| Sh2b3 | 14.45789 | 15.33002 | 16.38016 | 13.93657 | 14.17856 | 14.2387 | 17.86961 | 17.61625 |
| Nvl | 2.003882 | 2.023705 | 2.115737 | 2.304308 | 2.54282 | 2.65729 | 2.130688 | 2.583477 |
| Adamtsl2 | 2.435956 | 2.59499 | 2.538687 | 7.692148 | 7.116322 | 7.206925 | 13.03652 | 12.81576 |
| E2f6 | 55.01469 | 54.95305 | 50.35476 | 50.84507 | 48.33444 | 50.49771 | 55.41155 | 54.6212 |
| Hps6 | 3.141955 | 2.435816 | 3.789521 | 2.966674 | 3.145695 | 2.822891 | 3.167912 | 3.249016 |
| AABR07025 | 7.480846 | 9.332351 | 8.443021 | 9.544576 | 10.99265 | 9.754726 | 9.013026 | 9.35091 |
| Aar2 | 5.588661 | 5.955897 | 5.596129 | 5.496196 | 4.490624 | 5.61943 | 6.653872 | 5.650331 |
| Nhlrc1 | 17.41134 | 19.60062 | 20.53308 | 14.30173 | 13.31176 | 15.57469 | 11.90385 | 12.54868 |
| Cul7 | 2.437874 | 2.484695 | 2.918401 | 2.772349 | 2.709261 | 2.697359 | 3.539107 | 4.064379 |
| Cnga4 | 0.133416 | 0.075477 | 0.071615 | 0.061469 | 0.096174 | 0 | 0.07463 | 0.078836 |
| AC113635. | 0 | 0 | 0 | 0 | 0 | 0 | 0 | 0 |
| Med17 | 4.823574 | 4.67311 | 3.819081 | 4.528072 | 4.368116 | 4.044281 | 5.160307 | 5.584706 |
| Gadd45a | 34.39019 | 37.38309 | 38.18849 | 60.19005 | 54.38681 | 57.53185 | 51.73956 | 47.91055 |
| Tmem126a | 33.8491 | 33.66941 | 32.28639 | 23.28121 | 23.85306 | 24.039 | 21.87192 | 23.77739 |
| AC113837. | 17.00192 | 23.08425 | 16.79244 | 20.67992 | 16.17786 | 25.64056 | 25.86855 | 25.31701 |
| P2rx6 | 0.251979 | 0.440614 | 0.221331 | 0.422162 | 0.173385 | 0.220936 | 0.256276 | 0.203038 |
| AABR07025 | 0 | 0 | 0 | 0 | 0 | 0 | 0.406838 | 0.322323 |
| Adamts9 | 4.687621 | 4.939324 | 4.601914 | 3.768167 | 3.469151 | 3.331899 | 4.494065 | 4.393791 |
| AC112801. | 0.793423 | 0 | 0 | 0 | 0.428958 | 0 | 0.443823 | 0 |
| Igfals | 0.02314 | 0 | 0.049685 | 0.085291 | 0 | 0.020289 | 0 | 0 |
| Coro7 | 0.853792 | 1.502707 | 1.222134 | 1.573472 | 1.538655 | 1.330855 | 1.804238 | 1.429433 |
| Plcg1 | 25.8655 | 27.7817 | 27.28777 | 24.89866 | 25.24434 | 26.17427 | 29.33309 | 30.80463 |
| Zfp40 | 1.011991 | 0.832742 | 0.750629 | 0.983374 | 1.05446 | 1.177708 | 0.699887 | 1.092682 |
| C1qtnf9 | 14.90163 | 13.60996 | 13.18945 | 11.14858 | 11.06185 | 11.79605 | 13.48336 | 12.6905 |
| Pdia4 | 11.98413 | 11.70236 | 12.69676 | 16.49184 | 16.19175 | 15.83054 | 21.07941 | 19.27129 |
| Pias2 | 15.33553 | 14.80443 | 15.91992 | 11.79752 | 11.83566 | 12.23728 | 14.66978 | 14.51545 |
| Chaf1b | 0.308943 | 0.233037 | 0.138196 | 0.332126 | 0.306218 | 0.45147 | 0.31683 | 0.433567 |
| Vps16 | 7.5266 | 7.564739 | 7.628994 | 9.241139 | 9.220638 | 8.933667 | 8.733952 | 9.261613 |
| Slc50a1 | 5.99212 | 6.720847 | 6.376985 | 9.602614 | 9.408915 | 8.954467 | 8.452514 | 8.59015 |
| Prss57 | 0 | 0.212364 | 0.067166 | 0.288251 | 0.202948 | 0 | 0.069994 | 0.16636 |
| Rab1b | 45.33466 | 46.6426 | 44.31708 | 48.59276 | 44.63583 | 46.85913 | 46.75354 | 50.33475 |
| Meltf | 0 | 0 | 0.02679 | 0.011497 | 0.040474 | 0.076579 | 0 | 0.033177 |
| Mir23b | 2.699274 | 11.60559 | 5.795687 | 7.461825 | 9.339796 | 11.36029 | 9.059484 | 7.656003 |
| Caskin1 | 0.356231 | 0.413135 | 0.535411 | 0.558029 | 0.433336 | 0.437281 | 0.7074 | 0.434149 |
| Gprin2 | 0.038363 | 0.217031 | 0.041185 | 0.03535 | 0 | 0 | 0 | 0 |
| Plekhg2 | 4.83844 | 5.557234 | 5.99092 | 6.615427 | 6.214804 | 6.670474 | 7.669435 | 8.234386 |
| Alb | 0 | 0 | 0 | 0 | 0 | 0 | 0 | 0.022433 |
| Atxn2l | 19.54378 | 20.9288 | 21.47758 | 20.3382 | 20.54458 | 22.22437 | 26.01567 | 26.94765 |
| Slc31a1 | 5.325348 | 5.983538 | 5.399484 | 6.645029 | 6.597962 | 6.290544 | 6.330123 | 7.637417 |
| Snx27 | 11.34442 | 11.08206 | 11.01718 | 9.371756 | 9.281729 | 9.930716 | 11.36809 | 11.39635 |
| Pde7b | 5.363138 | 5.1136 | 5.046049 | 4.386649 | 4.886868 | 4.49106 | 4.280928 | 4.433147 |
| LOC100909 | 1.825392 | 2.194429 | 18.37195 | 17.55615 | 0.863524 | 7.902493 | 13.01881 | 18.40401 |
| RGD156358 | 0.167839 | 0 | 0 | 0.309315 | 0 | 0.147162 | 0.187771 | 0 |

| | | | | | | | | |
|---|---|---|---|---|---|---|---|---|
| Slc37a4 | 13.16745 | 11.34566 | 11.85257 | 13.43994 | 13.41636 | 12.72195 | 11.75659 | 12.25451 |
| Clmp | 2.295456 | 2.627577 | 2.824599 | 4.267452 | 4.223828 | 3.954695 | 4.06984 | 4.140541 |
| Rn50_X_0( | 3.978783 | 2.831781 | 2.686897 | 3.429757 | 3.191955 | 2.982191 | 3.876926 | 3.981638 |
| Nampt | 61.07735 | 56.60647 | 55.4867 | 58.88138 | 62.41106 | 66.03408 | 58.44855 | 59.54948 |
| AABR07051 | 0.402815 | 0.911532 | 1.297342 | 0.371178 | 1.306673 | 0.353188 | 0.901303 | 1.071104 |
| Rcor2 | 2.072033 | 6.512244 | 2.20199 | 1.658581 | 1.674687 | 1.284578 | 1.381493 | 1.409873 |
| Adamts1 | 22.58875 | 22.20513 | 21.96341 | 21.87841 | 23.48547 | 22.40418 | 38.91886 | 36.57253 |
| Srgn | 32.94187 | 32.94258 | 31.61434 | 28.01671 | 28.73851 | 28.77405 | 29.176 | 32.44224 |
| Lhx2 | 0.230252 | 0.260519 | 0.388442 | 0.272788 | 0.213401 | 0.490293 | 0.183997 | 0.349858 |
| Sim2 | 0.106273 | 0.150303 | 0.057045 | 0.171371 | 0.172367 | 0.09318 | 0.237787 | 0.117744 |
| AABR07019 | 0 | 0 | 0.117121 | 0 | 0 | 0 | 0.122051 | 0 |
| Spns2 | 9.041208 | 8.652368 | 9.299463 | 8.755157 | 9.146826 | 8.817385 | 11.91681 | 12.94424 |
| RGD131050 | 24.62215 | 22.1045 | 23.8353 | 27.31005 | 27.11661 | 27.6471 | 26.64366 | 24.83938 |
| Cyp46a1 | 0.773432 | 0.30886 | 0.488429 | 0.670765 | 0.737913 | 0.737983 | 1.145226 | 0.907321 |
| Skor1 | 0 | 0 | 0 | 0.016668 | 0 | 0 | 0 | 0 |
| Rnf10 | 52.91512 | 54.81308 | 57.63047 | 50.74818 | 48.88871 | 49.40847 | 63.81442 | 62.2747 |
| Ccp110 | 1.072633 | 0.922361 | 0.921232 | 1.176183 | 1.136626 | 1.222631 | 1.464017 | 1.340526 |
| Asb9 | 31.27296 | 28.79548 | 28.4588 | 29.00441 | 28.26723 | 32.56144 | 33.11901 | 32.37467 |
| LOC690142 | 0.090442 | 0.102331 | 0.097095 | 0.200013 | 0.176029 | 0.158599 | 0.242838 | 0.673371 |
| Tmem30a | 31.65351 | 33.63445 | 34.05594 | 31.83058 | 33.036 | 34.17944 | 39.83911 | 37.84521 |
| Rdh5 | 0.090677 | 0.025649 | 0.121684 | 0.313332 | 0.171583 | 0.159011 | 0.202891 | 0.100464 |
| Mfng | 6.360832 | 7.196982 | 8.003942 | 6.433751 | 7.037826 | 6.926083 | 9.002841 | 7.499747 |
| Pck1 | 0.439546 | 0.678171 | 0.428983 | 0.368204 | 0.345654 | 0.227733 | 0.692913 | 0.531261 |
| Mir26a | 0.581844 | 1.316657 | 0 | 1.608438 | 1.887417 | 2.040644 | 0 | 0.515717 |
| AABR07024 | 1.217812 | 0.918598 | 1.743199 | 1.496221 | 1.316803 | 1.067779 | 3.179013 | 2.158815 |
| AABR07051 | 0.480421 | 0.362383 | 0.343842 | 0.295126 | 0.69263 | 0.140411 | 0.358316 | 0.283881 |
| Spo11 | 0.215055 | 0.283879 | 0.307834 | 0.330275 | 0.232536 | 0.188561 | 0.080198 | 0.063538 |
| AABR07037 | 0.503518 | 0 | 0 | 0.927945 | 0.544447 | 0 | 0 | 0 |
| Ptx3 | 0.326607 | 0.36954 | 0.321414 | 0.62699 | 0.559162 | 0.620466 | 0.334944 | 0.458355 |
| Tsnaxip1 | 0 | 0.045753 | 0.043412 | 0.018631 | 0.021862 | 0 | 0 | 0.017921 |
| Mapre2 | 6.438971 | 9.069571 | 10.08682 | 7.507389 | 18.75576 | 14.34468 | 18.08259 | 10.30787 |
| Itpr3 | 3.265806 | 3.587416 | 3.93393 | 3.475234 | 3.254685 | 3.567592 | 4.006358 | 4.038796 |
| Nat3 | 0 | 0 | 0 | 0 | 0 | 0 | 0 | 0 |
| Icam5 | 0.184801 | 0.095043 | 0.144288 | 0.077403 | 0.108994 | 0.220955 | 0.357109 | 0.178689 |
| Erap1 | 10.6156 | 8.967261 | 8.480151 | 9.307958 | 9.75316 | 9.342459 | 11.00583 | 12.21435 |
| LOC100360 | 3.572792 | 2.774228 | 3.158746 | 3.873161 | 4.317703 | 3.77758 | 4.036268 | 3.911859 |
| Lpin3 | 2.878536 | 2.935022 | 2.87469 | 3.05341 | 3.022039 | 3.257595 | 3.519948 | 3.560078 |
| Dhrs3 | 18.05982 | 18.4959 | 18.49602 | 19.89078 | 20.07253 | 20.20767 | 23.19152 | 25.65189 |
| AABR07000 | 0.119557 | 0 | 0 | 0.110167 | 0 | 0 | 0 | 0 |
| AABR07051 | 0 | 0.207893 | 0 | 0 | 0 | 0.48331 | 0 | 0 |
| Ccdc87 | 0 | 0 | 0 | 0 | 0 | 0 | 0.018365 | 0 |
| Klf15 | 23.98307 | 24.89068 | 26.76614 | 25.93507 | 25.39618 | 24.82937 | 24.56309 | 23.32196 |
| AABR07053 | 0.748085 | 1.692845 | 2.40935 | 1.378661 | 0 | 1.311842 | 1.673848 | 1.326129 |
| Msr1 | 0.597093 | 0.312583 | 0.287023 | 0.599469 | 0.674536 | 0.64074 | 0.727822 | 0.892587 |
| F7 | 0 | 0 | 0 | 0 | 0 | 0 | 0 | 0 |
| Lrrc17 | 1.64992 | 1.780646 | 1.825796 | 3.578638 | 3.211262 | 3.761293 | 3.862101 | 3.442279 |
| LOC100912 | 1.160005 | 0.562496 | 0.889528 | 0.712599 | 0.776469 | 1.065526 | 0.741578 | 1.224012 |
| Kcnk16 | 0 | 0.025605 | 0 | 0 | 0 | 0 | 0 | 0.020058 |
| Fam71f2 | 0 | 0 | 0.132382 | 0 | 0 | 0 | 0 | 0 |
| Styxl1 | 0.959083 | 0.651094 | 0.720746 | 0.942674 | 0.760498 | 1.177294 | 0.894149 | 1.16178 |
| Znfx1 | 5.164442 | 5.164466 | 5.275294 | 6.445415 | 5.953781 | 5.653575 | 8.411721 | 8.858812 |
| Eif1ad | 3.675882 | 3.485269 | 3.527414 | 3.557486 | 3.330736 | 3.889227 | 3.836721 | 4.222811 |
| Syce3 | 0 | 0.084161 | 0 | 0 | 0 | 0 | 0 | 0 |
| AABR07031 | 0 | 0 | 0 | 0 | 0 | 0 | 0 | 0 |

| | | | | | | | | |
|---|---|---|---|---|---|---|---|---|
| Cabp1 | 5.009831 | 4.594378 | 4.415928 | 4.859329 | 3.706408 | 4.485101 | 5.427784 | 6.82429 |
| LOC10369C | 0 | 0 | 0 | 0 | 0 | 0 | 0.107102 | 0 |
| LOC102549 | 2.897146 | 3.23701 | 2.410461 | 3.170158 | 1.99706 | 2.984759 | 3.970468 | 2.920969 |
| Jam2 | 35.81947 | 36.35426 | 34.16423 | 30.86329 | 30.36342 | 30.25809 | 34.70521 | 34.93528 |
| Sp1 | 5.425359 | 5.692938 | 5.000734 | 5.380925 | 5.71952 | 5.542841 | 6.707762 | 6.933289 |
| Tes | 4.478373 | 3.73759 | 3.546362 | 4.453507 | 4.171176 | 3.868325 | 5.183825 | 4.873328 |
| Arl9 | 0 | 0 | 0.17432 | 0 | 0 | 0 | 0 | 0 |
| Map3k3 | 12.29797 | 11.48178 | 13.16602 | 11.29016 | 10.99729 | 11.80031 | 15.22209 | 14.64129 |
| AABR07073 | 0 | 0 | 0.711622 | 0 | 0 | 0 | 0 | 0.587526 |
| AABR07039 | 0 | 0.493746 | 0 | 0 | 0 | 0 | 0.976411 | 0 |
| AC114866. | 0 | 0 | 0 | 0 | 2.022233 | 0 | 0 | 1.657661 |
| Eif5a2 | 1.505077 | 1.192046 | 1.431134 | 1.674149 | 2.138892 | 2.299966 | 2.309229 | 2.248784 |
| Hsh2d | 0.683801 | 0.848562 | 0.663062 | 1.36182 | 1.049448 | 0.947687 | 1.209203 | 1.114418 |
| Pdha1l1 | 23.49918 | 23.8858 | 25.00729 | 24.35139 | 21.27163 | 26.70651 | 20.42849 | 22.92272 |
| Mov10l1 | 0 | 0.033286 | 0 | 0.040663 | 0.015905 | 0 | 0 | 0.026076 |
| AABR07062 | 0 | 0 | 0 | 0 | 0.370082 | 0.600189 | 0 | 1.213451 |
| Slc25a21 | 1.309766 | 0.699028 | 1.061221 | 1.184126 | 0.855083 | 1.30008 | 1.96296 | 1.314238 |
| Cks1b | 13.80111 | 14.54476 | 15.06156 | 17.10656 | 14.57015 | 16.16304 | 16.0971 | 16.36799 |
| Luc7l | 9.226205 | 7.992372 | 8.202512 | 10.36131 | 10.44379 | 10.11193 | 10.84603 | 11.02065 |
| Smim5 | 5.752724 | 7.725561 | 4.848384 | 7.976134 | 8.720099 | 8.060962 | 6.075001 | 7.958136 |
| Hist2h2aa | 6.928824 | 5.879729 | 5.72195 | 5.525168 | 9.076891 | 9.930614 | 7.45352 | 8.38532 |
| AC109877. | 0 | 0 | 0 | 1.304139 | 0 | 0 | 0 | 0 |
| AC120811. | 0 | 0 | 0 | 0 | 0 | 0 | 1.642561 | 0 |
| LOC498836 | 0 | 0 | 0 | 0 | 0 | 0.123426 | 0 | 0 |
| Crbn | 18.63085 | 17.50973 | 17.38603 | 17.7876 | 18.36406 | 17.0679 | 16.84601 | 18.59048 |
| Samd4a | 6.847031 | 8.133241 | 6.846937 | 7.252712 | 7.472788 | 6.938605 | 11.26353 | 10.24712 |
| Sertad3 | 2.630252 | 2.660369 | 2.738175 | 3.745677 | 2.542411 | 2.725517 | 3.343874 | 2.896492 |
| Ntsr2 | 0.669748 | 0.520979 | 0.898772 | 0.308573 | 0.49788 | 0.183511 | 0.515133 | 0.222612 |
| Dnm1 | 5.639685 | 6.237874 | 6.539703 | 10.69331 | 9.772612 | 9.44599 | 10.81905 | 11.83995 |
| Pdzk1ip1 | 0.342261 | 0.07745 | 0 | 0.31538 | 0.444098 | 0.180057 | 0.306325 | 0.121345 |
| Srd5a1 | 0.481481 | 0.588355 | 0.537577 | 0.514653 | 0.374846 | 0.489709 | 0.517114 | 0.563324 |
| Fuk | 1.810252 | 2.180358 | 2.601677 | 1.937121 | 2.273109 | 1.996839 | 2.596867 | 2.756145 |
| Cpa2 | 0 | 0 | 0 | 0.073895 | 0.043356 | 0.10547 | 0 | 0.035539 |
| AABR07014 | 0.010208 | 0.01155 | 0 | 0.018812 | 0 | 0 | 0 | 0 |
| #N/A | 2.025201 | 2.29142 | 1.863585 | 2.132735 | 5.005305 | 2.283041 | 2.91305 | 5.641544 |
| AABR07052 | 0.278542 | 0 | 0.299033 | 0 | 0.301184 | 0.244226 | 0 | 0.246886 |
| Rmnd5a | 15.66949 | 15.5872 | 14.74646 | 12.02617 | 13.76363 | 12.25564 | 13.02382 | 15.53101 |
| 5S_rRNA | 0 | 0 | 0 | 0 | 0.475819 | 0 | 0 | 0 |
| AABR07072 | 0.095489 | 0.043216 | 0.020503 | 0.035196 | 0.144551 | 0.06698 | 0.042731 | 0.016927 |
| Rpl27a | 212.2876 | 201.4037 | 193.0139 | 206.3883 | 212.9906 | 193.0407 | 211.6142 | 203.8814 |
| Zfp536 | 0.352428 | 0.371255 | 0.574054 | 0.31355 | 0.446778 | 0.394253 | 0.421472 | 0.452404 |
| Kdsr | 4.175678 | 4.175213 | 4.508921 | 5.234693 | 4.567602 | 5.172564 | 5.269089 | 5.271932 |
| Iqcj | 0 | 0 | 0 | 0 | 0 | 0 | 0 | 0 |
| LOC10369C | 0.153296 | 0.312205 | 0.098744 | 0.282513 | 0.198908 | 0.188174 | 0.377302 | 0.380447 |
| RGD156609 | 5.217957 | 4.709041 | 4.134669 | 3.949545 | 3.694233 | 3.540263 | 4.308718 | 4.459758 |
| Fam84b | 1.515413 | 2.730688 | 2.651232 | 1.810139 | 1.395839 | 1.525562 | 1.82096 | 1.0447 |
| Slc5a5 | 0 | 0 | 0 | 0 | 0 | 0.016139 | 0 | 0.032629 |
| Tmem252 | 9.757155 | 8.957326 | 8.796775 | 7.457514 | 6.215663 | 7.118182 | 11.45179 | 12.64835 |
| AABR07068 | 0 | 0 | 0 | 0.099903 | 0 | 0 | 0 | 0 |
| Lancl1 | 19.4255 | 19.98276 | 18.03224 | 14.84337 | 16.3114 | 15.70189 | 17.74515 | 17.65566 |
| Crhbp | 0 | 0.036216 | 0 | 0 | 0 | 0 | 0 | 0 |
| Tbca | 79.46758 | 94.27959 | 90.24494 | 71.36385 | 75.19867 | 78.61849 | 75.13226 | 79.14897 |
| AABR07029 | 0.167303 | 0.063099 | 0.05987 | 0.051388 | 0 | 0.048897 | 0 | 0.098859 |
| Cnot8 | 25.23396 | 23.34074 | 22.68101 | 22.13394 | 21.59932 | 24.03486 | 26.59458 | 25.23427 |

| | | | | | | | | |
|---|---|---|---|---|---|---|---|---|
| Bpifa1 | 0 | 0 | 0 | 0 | 0 | 0 | 0 | 0.038296 |
| Cacng5 | 0.020905 | 0 | 0 | 0 | 0 | 0 | 0 | 0 |
| Stard5 | 4.574394 | 3.795522 | 4.443199 | 4.897573 | 5.747044 | 5.500571 | 5.653761 | 6.564449 |
| Crygc | 0.084735 | 0 | 0 | 0 | 0 | 0 | 0 | 0.075104 |
| Ppig | 9.65569 | 9.842934 | 8.395466 | 9.721692 | 9.4899 | 8.952987 | 10.87138 | 10.32193 |
| Fstl3 | 2.015136 | 1.749065 | 3.082071 | 5.341675 | 4.835449 | 4.985969 | 6.083911 | 5.260475 |
| Dnah11 | 1.026783 | 0.910923 | 1.08144 | 0.799202 | 0.647643 | 0.743416 | 0.874593 | 0.785986 |
| Susd5 | 1.543476 | 1.174831 | 1.566637 | 2.249744 | 2.063414 | 2.435978 | 2.511668 | 2.885361 |
| AABR0701( | 0.196127 | 0.221909 | 0 | 0.060241 | 0.07069 | 0.286607 | 0.146279 | 0.231783 |
| Acsl5 | 10.93003 | 11.13496 | 10.38191 | 11.70429 | 11.98006 | 10.83754 | 12.27579 | 11.93949 |
| Bmper | 2.033762 | 2.410683 | 1.85661 | 2.281984 | 2.154199 | 2.438259 | 2.553889 | 2.906272 |
| Taar4 | 0 | 0.113505 | 0.161546 | 0.046219 | 0 | 0.131938 | 0.168347 | 0.133375 |
| AABR07065 | 0 | 0.169285 | 0 | 0 | 0 | 0 | 0 | 0 |
| Ung | 3.940019 | 3.035197 | 2.519918 | 4.068301 | 3.686204 | 4.165135 | 4.470441 | 4.210495 |
| Tkt | 20.62365 | 19.22687 | 19.08224 | 20.97861 | 22.27279 | 24.18413 | 27.69867 | 25.87771 |
| Havcr2 | 0.07581 | 0.021444 | 0.122081 | 0.157176 | 0.184438 | 0.06647 | 0.254439 | 0.151187 |
| Naaa | 3.914708 | 3.549514 | 3.396694 | 5.262631 | 4.26191 | 4.748964 | 5.129533 | 5.2047 |
| Rgl3 | 2.40323 | 2.043686 | 2.054157 | 2.891521 | 2.896504 | 2.805065 | 3.681878 | 4.029556 |
| LOC102553 | 0 | 0 | 0 | 0 | 0 | 0 | 0 | 0 |
| Acvr2a | 4.138411 | 4.499306 | 3.822339 | 3.280787 | 3.699837 | 3.506934 | 3.879779 | 4.364809 |
| Cln5 | 3.014614 | 3.058043 | 2.120387 | 2.873636 | 3.147256 | 2.529284 | 3.285393 | 3.47821 |
| Fancf | 3.046932 | 3.79805 | 4.76801 | 4.092475 | 5.639915 | 3.848847 | 3.062118 | 3.570348 |
| Helz | 3.796593 | 4.094755 | 4.09404 | 2.742629 | 2.953184 | 3.26213 | 4.115022 | 3.957188 |
| Trmt44 | 0.575774 | 0.793447 | 0.602281 | 0.639385 | 0.766248 | 0.640758 | 0.858867 | 0.811305 |
| Ciapin1 | 22.63249 | 23.62301 | 22.68772 | 23.3054 | 21.38257 | 20.56354 | 23.80101 | 24.17266 |
| LOC10091( | 0.03706 | 0 | 0.039786 | 0.102448 | 0.040073 | 0 | 0 | 0 |
| AABR07067 | 0 | 0.3338 | 0 | 0 | 0 | 0 | 0 | 0 |
| LOC49977( | 3.489299 | 3.61898 | 3.263548 | 4.408792 | 4.687578 | 4.125582 | 5.145751 | 4.45167 |
| Lsm11 | 2.334495 | 2.693163 | 1.719961 | 2.657297 | 2.47476 | 1.565267 | 2.867781 | 2.393756 |
| AC117101. | 0 | 0.553734 | 0 | 0.450964 | 0 | 0 | 0 | 0.433781 |
| Zfp628 | 2.209749 | 2.713425 | 3.015956 | 2.762283 | 2.204148 | 2.388094 | 2.625482 | 3.203619 |
| Rsad2 | 4.042432 | 3.620269 | 3.680403 | 4.988527 | 4.849828 | 5.147532 | 7.926349 | 8.47008 |
| AABR07061 | 0.096795 | 0 | 0 | 0 | 0 | 0 | 0.10829 | 0.343176 |
| Dnajc28 | 12.0616 | 12.17745 | 9.363053 | 10.49873 | 10.91518 | 10.77087 | 10.96127 | 10.62501 |
| AABR07027 | 0.017757 | 0 | 0.019063 | 0 | 0 | 0 | 0 | 0 |
| AABR07036 | 0.600182 | 0.509309 | 0.402709 | 0.414783 | 0.486726 | 0.526241 | 0.75539 | 0.332482 |
| Esrp2 | 0.052695 | 0.014906 | 0.042429 | 0.024278 | 0.014245 | 0.023102 | 0.014738 | 0.046706 |
| Tor1b | 4.426551 | 4.217629 | 4.290435 | 5.366964 | 4.515074 | 4.996854 | 5.774259 | 5.988458 |
| Hpd | 12.6224 | 12.3903 | 13.03822 | 9.399145 | 10.51297 | 10.22981 | 12.40392 | 12.88116 |
| AABR07012 | 0.035818 | 0.010132 | 0 | 0.016502 | 0.019365 | 0.015703 | 0.020036 | 0.02381 |
| Trim29 | 0 | 0 | 0 | 0 | 0 | 0.016048 | 0.040954 | 0.016223 |
| Angpt4 | 0.022494 | 0 | 0.048297 | 0.082909 | 0.048645 | 0.059168 | 0.025165 | 0.07975 |
| hist1h2ai | 0 | 0.301525 | 0.286098 | 0 | 0.432233 | 0 | 0 | 0.118103 |
| 5S_rRNA | 1.309148 | 0.493746 | 1.405454 | 0.804219 | 0.943709 | 0.765241 | 0 | 0.386788 |
| LOC100361 | 0.698212 | 0.394997 | 1.124363 | 0 | 0.377483 | 0.612193 | 0.390564 | 0.30943 |
| AABR07003 | 0 | 0 | 0 | 0.148929 | 0 | 0.141711 | 0 | 0 |
| Bptf | 10.87054 | 9.900165 | 9.813589 | 8.631876 | 8.537344 | 8.051056 | 10.84859 | 11.29569 |
| AC229945. | 0.021724 | 0.061449 | 0.011661 | 0.040036 | 0.035235 | 0.009524 | 0.012152 | 0 |
| Mpp7 | 5.757634 | 4.576296 | 6.666488 | 5.678129 | 5.814034 | 6.195639 | 5.483163 | 4.808046 |
| Phf5a | 38.45484 | 39.70022 | 36.33729 | 40.49671 | 42.15551 | 38.84474 | 37.86691 | 41.38824 |
| AABR07009 | 0 | 0 | 0 | 0 | 0 | 0 | 0 | 0 |
| LOC103693 | 0 | 0 | 0.038218 | 0.098409 | 0.038493 | 0.093639 | 0 | 0.094659 |
| Them5 | 0 | 0 | 0.046732 | 0 | 0.094136 | 0.190833 | 0.194795 | 0.038582 |
| Mt-nd3 | 9408.41 | 9612.392 | 8823.02 | 10621.93 | 11342.07 | 9753.002 | 10280.94 | 10172.78 |

| | | | | | | | | |
|---|---|---|---|---|---|---|---|---|
| AABR07051 | 0 | 0 | 0 | 0 | 0.428958 | 0 | 0 | 0 |
| Pus7 | 2.301799 | 2.532033 | 1.899108 | 2.572715 | 2.373675 | 2.167717 | 3.218938 | 2.758046 |
| Apobec1 | 2.099577 | 0.931597 | 1.149113 | 2.389896 | 2.670873 | 2.454548 | 2.394971 | 2.773194 |
| Slc16a2 | 1.852378 | 2.387479 | 1.936768 | 2.434178 | 2.560292 | 2.189094 | 2.883281 | 3.226601 |
| Gpr3 | 0.238958 | 0.038624 | 0 | 0.125823 | 0.073823 | 0.059862 | 0.190954 | 0.121029 |
| Ubtd2 | 2.153365 | 1.971294 | 2.290759 | 2.705783 | 2.794083 | 2.883601 | 2.16818 | 2.776195 |
| Ppef1 | 0 | 0.063099 | 0.089805 | 0.154163 | 0.060301 | 0.268935 | 0.06239 | 0.197719 |
| AABR07057 | 0 | 0 | 0 | 0.043354 | 0 | 0 | 0 | 0.125107 |
| Gpr19 | 0.310265 | 0.546079 | 0.33309 | 0.69886 | 0.596419 | 0.785896 | 0.539951 | 0.733343 |
| AC134009. | 0.326267 | 0.553734 | 0.350269 | 0.300643 | 0.529182 | 1.144286 | 0.365013 | 1.156748 |
| AABR07072 | 1.415295 | 1.201005 | 0.759705 | 0.652069 | 0 | 2.481864 | 1.187527 | 0.313612 |
| Mpp6 | 8.085423 | 6.980916 | 7.030259 | 6.526796 | 5.900687 | 6.210466 | 6.877655 | 7.679816 |
| Leprot | 0.913185 | 0.72834 | 0.546432 | 0.372451 | 0.712233 | 0.485659 | 0.485693 | 0.716519 |
| Cmtm2a | 0.054949 | 0 | 0.058991 | 0 | 0 | 0.096358 | 0 | 0.048704 |
| Zfp821 | 3.193374 | 2.663934 | 3.283024 | 3.117128 | 3.423687 | 3.227065 | 4.571723 | 4.101748 |
| Lgi3 | 0.047333 | 0.089258 | 0.050815 | 0.116308 | 0.03412 | 0.055335 | 0 | 0.055938 |
| Cep78 | 1.053337 | 1.313414 | 1.453918 | 1.505434 | 1.255179 | 1.300533 | 1.082229 | 1.200375 |
| AABR07067 | 0 | 0 | 0 | 0 | 0 | 0.166357 | 0 | 0.084084 |
| Ap1g2 | 2.743172 | 3.173257 | 3.054857 | 2.980452 | 3.076829 | 3.159089 | 3.641502 | 3.647114 |
| Scml2 | 0.132359 | 0.149758 | 0.219602 | 0.155226 | 0.18215 | 0.158253 | 0.107692 | 0.149311 |
| Acadm | 539.8543 | 534.5204 | 522.7454 | 470.3754 | 477.0149 | 479.951 | 414.7722 | 425.5258 |
| Mptx1 | 0 | 0.089772 | 0.085179 | 0 | 0.171583 | 0 | 0 | 0.070325 |
| Prpf38b | 13.57635 | 13.41903 | 13.91174 | 13.31666 | 14.79126 | 14.37179 | 16.37913 | 16.62769 |
| AC109685. | 0.534346 | 1.209175 | 2.294619 | 0.492379 | 0 | 0.468515 | 0 | 0 |
| AABR07018 | 0 | 0 | 0 | 0 | 0 | 0 | 0 | 0 |
| Rab44 | 0.024143 | 0.054633 | 0.025919 | 0.06674 | 0.026105 | 0.084674 | 0.16206 | 0.064197 |
| Rest | 4.741466 | 4.510184 | 4.804971 | 4.085535 | 4.189219 | 3.544147 | 5.241951 | 4.710875 |
| AC120291. | 0 | 0.604588 | 0 | 0 | 0 | 0 | 0 | 0 |
| AC117091. | 0 | 0 | 0 | 0.051062 | 0 | 0 | 0 | 0 |
| Anxa13 | 0.065132 | 0 | 0.104885 | 0.030008 | 0 | 0 | 0.1093 | 0.05773 |
| AABR07070 | 0.748085 | 0 | 2.40935 | 0.689331 | 1.617786 | 0 | 0 | 0 |
| Cib1 | 14.35744 | 13.54854 | 14.45427 | 18.33509 | 20.16251 | 20.16268 | 20.12806 | 20.22385 |
| Nphp3 | 0.984902 | 0.993535 | 0.662439 | 1.060629 | 0.987975 | 0.936393 | 1.4072 | 1.293675 |
| Atmin | 1.781906 | 1.672304 | 1.601573 | 1.629227 | 1.538412 | 1.622934 | 1.514455 | 1.665095 |
| Cdhr5 | 0.966581 | 0.60043 | 0.773178 | 0.943058 | 1.04515 | 0.814263 | 0.975351 | 0.806333 |
| AABR07007 | 0.211954 | 0.119908 | 0.028443 | 0.024413 | 0.014324 | 0.058075 | 0.029641 | 0.07045 |
| AABR07049 | 0 | 0 | 0 | 0 | 0 | 0 | 0 | 0 |
| Dhx30 | 13.26528 | 13.88537 | 15.12453 | 13.11241 | 12.19585 | 12.83765 | 13.07878 | 14.42358 |
| RGD156203 | 0 | 0 | 0 | 0.038205 | 0 | 0 | 0 | 0 |
| Sys1 | 50.67329 | 57.69353 | 63.08927 | 40.60087 | 37.40517 | 42.2506 | 53.67302 | 46.55517 |
| Zc3h3 | 3.090448 | 3.090514 | 3.854002 | 2.977168 | 3.071624 | 2.956044 | 3.457456 | 3.859806 |
| AABR07065 | 0 | 0 | 0 | 0 | 0 | 0.131938 | 0 | 0 |
| AABR07058 | 3.436514 | 3.826535 | 5.211892 | 2.613711 | 2.595198 | 2.869655 | 3.661542 | 2.224029 |
| Tmem106a | 1.741123 | 1.770505 | 1.892867 | 2.315176 | 2.144792 | 2.318913 | 2.835537 | 2.266029 |
| RGD156227 | 2.045544 | 1.388662 | 3.074431 | 2.18647 | 2.034872 | 1.147862 | 2.471541 | 1.813067 |
| Ublcp1 | 9.601259 | 9.380708 | 7.629221 | 10.00823 | 10.51795 | 10.27442 | 11.3899 | 11.23508 |
| Ep400 | 4.854811 | 5.119397 | 5.239643 | 4.725479 | 4.474015 | 4.804057 | 6.031623 | 6.264817 |
| Asmt | 0.473594 | 0.306199 | 0.363166 | 0.436398 | 0.219467 | 0.474568 | 0.302763 | 0.749588 |
| AC106932. | 0 | 0.148495 | 0 | 0 | 0 | 0 | 0 | 0 |
| Ccdc152 | 30.9756 | 26.48536 | 22.69307 | 34.40012 | 38.13019 | 35.07725 | 37.41969 | 37.56088 |
| AABR07070 | 0 | 0 | 0 | 0 | 0 | 0.304069 | 0 | 0 |
| LOC100912 | 0 | 0.09541 | 0.271585 | 0 | 0.09118 | 0.147873 | 0 | 0.149483 |
| AABR07065 | 0 | 0 | 0.429146 | 0 | 0 | 0 | 0.447211 | 0 |
| Oard1 | 18.75885 | 18.11364 | 19.74524 | 18.24273 | 19.68904 | 17.78718 | 18.11545 | 17.11434 |

| | | | | | | | | |
|---|---|---|---|---|---|---|---|---|
| Tecpr2 | 2.515141 | 2.216286 | 2.102893 | 1.580669 | 2.030289 | 1.697149 | 2.178447 | 2.260114 |
| Apoe | 208.146 | 215.7416 | 234.3184 | 297.8221 | 272.4039 | 283.4623 | 335.0073 | 333.0392 |
| Toe1 | 5.198088 | 5.736172 | 4.650399 | 5.913374 | 5.585922 | 5.87998 | 4.271799 | 4.635764 |
| Tmed7 | 13.90327 | 12.38463 | 11.4073 | 13.96321 | 14.70372 | 13.9548 | 14.42871 | 14.95804 |
| Zfp663 | 0 | 0.029699 | 0 | 0 | 0.028382 | 0 | 0 | 0 |
| Trpc4 | 0.139596 | 0.05923 | 0.149865 | 0.080395 | 0.113207 | 0.030599 | 0.058565 | 0.046399 |
| Gnai3 | 22.03678 | 23.14206 | 22.22089 | 19.50883 | 20.10382 | 18.44881 | 25.56687 | 24.24898 |
| Fam83b | 0.676417 | 0.501111 | 0.691597 | 0.415528 | 0.539842 | 0.501296 | 0.504497 | 0.578129 |
| Asic5 | 0 | 0.062898 | 0.02984 | 0.153672 | 0 | 0.073112 | 0 | 0 |
| LOC303566 | 0.216307 | 0.088997 | 0.189998 | 0.090599 | 0.127576 | 0.137933 | 0.263994 | 0.122006 |
| Peak1 | 4.765291 | 4.639569 | 4.538508 | 3.792252 | 3.602003 | 3.680494 | 5.038733 | 4.971802 |
| Art5 | 2.87117 | 3.21879 | 3.336893 | 2.742759 | 2.705804 | 2.97936 | 2.151248 | 2.521513 |
| LOC103690 | 0.208008 | 0.529539 | 0.762974 | 0.511122 | 0.262402 | 0.273572 | 0.252102 | 0.414827 |
| AABR07044 | 0 | 0 | 0 | 0 | 0.45298 | 0.367316 | 0 | 0 |
| Zrsr2 | 2.364797 | 2.229714 | 2.461828 | 2.789867 | 2.634506 | 2.528988 | 2.585502 | 2.127795 |
| LOC100912 | 3.221507 | 31.46588 | 3.176167 | 16.26612 | 2.48812 | 9.050313 | 2.537566 | 3.088474 |
| Nfkb1 | 13.07116 | 13.3028 | 13.49469 | 13.093 | 13.04983 | 12.8266 | 15.77513 | 15.77565 |
| Rn50_7_14 | 0 | 0 | 0 | 0 | 0 | 0 | 0.116703 | 0 |
| Pi4ka | 5.262028 | 4.613696 | 4.889649 | 4.936644 | 4.658379 | 4.927374 | 6.135917 | 6.397144 |
| Usp16 | 8.676225 | 7.701418 | 7.326873 | 10.01859 | 10.14691 | 9.310216 | 9.341056 | 9.829903 |
| Dyrk1a | 13.96794 | 14.36353 | 14.02483 | 13.10894 | 13.34759 | 11.51907 | 15.54413 | 16.19111 |
| Cep120 | 5.595138 | 4.896552 | 5.589944 | 5.071524 | 5.296785 | 5.015952 | 6.004098 | 5.961219 |
| Sla2 | 0.260527 | 0.421106 | 0.159824 | 0.240065 | 0.201217 | 0.358962 | 0.458018 | 0.494824 |
| Casp6 | 0.379753 | 0 | 0.021457 | 0.092086 | 0.064835 | 0.175246 | 0.111803 | 0.248017 |
| LOC361635 | 8.238279 | 8.466937 | 7.691495 | 9.415623 | 8.853504 | 8.517939 | 8.127895 | 9.205571 |
| RGD156312 | 0 | 0 | 0.157474 | 0 | 0 | 0 | 0 | 0.130013 |
| Hs1bp3 | 3.293346 | 3.472646 | 2.924751 | 3.066465 | 2.945788 | 3.401633 | 3.877352 | 3.484528 |
| Il2 | 0.14153 | 0.080067 | 0 | 0 | 0 | 0 | 0 | 0 |
| AC118165. | 1.353394 | 1.374245 | 1.341189 | 1.630822 | 1.651021 | 2.038615 | 2.290587 | 1.568682 |
| AC117889. | 0.209845 | 0.410106 | 0.327683 | 0.580092 | 0.24753 | 0.451618 | 0.192081 | 0.490354 |
| Prss50 | 0 | 0 | 0 | 0 | 0 | 0 | 0.055848 | 0 |
| Wnk1 | 14.45857 | 14.09874 | 14.26596 | 14.54853 | 14.35005 | 15.10463 | 19.16422 | 20.09576 |
| AABR07037 | 0 | 0.731476 | 0 | 0 | 0 | 0 | 0.723267 | 0.573019 |
| Dcaf12l1 | 0 | 0 | 0 | 0.013669 | 0 | 0.026014 | 0 | 0 |
| Pla2g4a | 2.088722 | 2.049579 | 1.86534 | 3.219147 | 3.537658 | 3.387267 | 4.30131 | 3.981196 |
| Tceal1 | 14.87921 | 13.4681 | 12.51677 | 14.48822 | 13.68738 | 12.87085 | 17.49093 | 14.27079 |
| Cbx6 | 10.98574 | 11.52129 | 14.41679 | 11.90192 | 11.3245 | 11.96922 | 12.53884 | 13.37162 |
| Suv39h2 | 0.343798 | 0.245679 | 0.388515 | 0.200082 | 0.469572 | 0.317308 | 0.323896 | 0.320764 |
| Spcs2 | 17.39626 | 18.1998 | 18.14348 | 16.99307 | 18.08243 | 17.24123 | 20.65129 | 20.66357 |
| AABR07013 | 2.181913 | 0.75961 | 1.441491 | 0.927945 | 0.907412 | 0.588647 | 1.689942 | 1.933938 |
| AABR07034 | 0.429934 | 0.38916 | 0.369249 | 0.316934 | 0.092976 | 0.527753 | 0.288594 | 0.228643 |
| #N/A | 0 | 0 | 1.40986 | 0 | 0 | 0 | 0 | 0 |
| Mrpl38 | 35.79341 | 38.68248 | 39.0727 | 29.69139 | 28.07262 | 28.25236 | 32.36747 | 34.42855 |
| AABR07012 | 0.656215 | 0.742476 | 0.140898 | 0.120935 | 0.993377 | 0.345222 | 0.440486 | 0.348981 |
| AABR07029 | 0 | 0.553734 | 0 | 0 | 0 | 0.858215 | 0 | 0 |
| Msmp | 16.20609 | 15.58596 | 20.33423 | 15.21327 | 16.75676 | 16.60737 | 19.26382 | 20.55885 |
| Rgs14 | 0.690699 | 0.678666 | 0.780537 | 1.055171 | 1.04165 | 0.876535 | 1.362434 | 1.240513 |
| AC105648. | 0.858458 | 2.428261 | 0 | 1.186553 | 1.856476 | 0.752696 | 2.401011 | 3.424022 |
| Zfp167 | 1.353264 | 1.041185 | 1.069272 | 1.087366 | 0.854547 | 0.977712 | 0.968942 | 0.997955 |
| Ssh3 | 3.570404 | 3.443716 | 4.356698 | 5.986697 | 5.44285 | 6.089999 | 5.915218 | 6.726992 |
| Fam228a | 0.032445 | 0.07342 | 0 | 0 | 0.070164 | 0 | 0.108893 | 0 |
| Rbak | 0.82683 | 0.752084 | 1.00949 | 1.210063 | 1.192053 | 0.938191 | 0.979434 | 0.962778 |
| LOC100910 | 0.334251 | 0.378189 | 0.538259 | 0.564664 | 0.421657 | 0.439607 | 0.249296 | 1.185052 |
| Dhfr | 0.760872 | 0.708969 | 0.672696 | 0.752666 | 0.895313 | 0.775052 | 1.189219 | 1.140528 |

| | | | | | | | | |
|---|---|---|---|---|---|---|---|---|
| Pald1 | 10.96973 | 11.25622 | 11.85232 | 8.534347 | 8.358199 | 9.093353 | 10.42074 | 10.08197 |
| Gpr183 | 0.920495 | 0.771479 | 0.95161 | 1.664984 | 2.027499 | 1.942996 | 1.373078 | 1.238929 |
| Grb2 | 15.30977 | 15.04788 | 16.95918 | 14.64994 | 14.05134 | 13.22779 | 17.30962 | 16.28737 |
| Crh | 0.043784 | 0 | 0 | 0.080691 | 0 | 0.03839 | 0 | 0 |
| Ltbp1 | 8.796133 | 9.060584 | 9.632255 | 7.82713 | 7.752161 | 8.315338 | 8.217801 | 8.071444 |
| Hdac8 | 1.412414 | 1.507623 | 1.230219 | 1.154146 | 1.671301 | 1.799194 | 1.103121 | 1.653443 |
| Cracr2a | 0.056126 | 0.063504 | 0 | 0.103437 | 0 | 0.049212 | 0 | 0.19899 |
| Lin37 | 9.444755 | 9.202088 | 10.02689 | 8.89637 | 10.95004 | 11.3176 | 10.97729 | 10.88276 |
| Cacnb4 | 0.110798 | 0.078352 | 0.044606 | 0.178668 | 0.104829 | 0.085004 | 0.185934 | 0.196412 |
| Grb7 | 0.054303 | 0.02048 | 0.116595 | 0.033359 | 0.039144 | 0.047613 | 0.040501 | 0.016044 |
| Zfp30 | 0 | 0.066873 | 0 | 0.027231 | 0 | 0.025911 | 0 | 0 |
| Zfp236 | 2.137202 | 2.397908 | 2.169622 | 2.059987 | 2.059528 | 2.218887 | 2.551074 | 2.821648 |
| Slc38a8 | 0 | 0 | 0.043278 | 0.037146 | 0.087179 | 0.212076 | 0.225499 | 0.178655 |
| Zfp366 | 6.623183 | 7.360299 | 7.458804 | 5.124347 | 4.593601 | 5.121729 | 7.063165 | 7.09946 |
| AABR07001 | 0.327799 | 0.092722 | 0.439892 | 0.377568 | 0.177222 | 0.215561 | 0.183364 | 0.290545 |
| #N/A | 6.416378 | 8.196582 | 8.221628 | 7.628954 | 6.937936 | 9.79993 | 11.11488 | 9.906656 |
| Slc39a1 | 22.58837 | 24.0832 | 21.84837 | 26.07791 | 24.51842 | 25.65193 | 26.53441 | 27.54839 |
| Med27 | 8.99255 | 10.36394 | 8.890732 | 8.980016 | 7.959714 | 9.021536 | 10.85594 | 10.23195 |
| Pknox2 | 4.863023 | 4.65193 | 4.793613 | 4.623665 | 4.756387 | 4.515861 | 4.847022 | 4.467079 |
| AABR07049 | 0 | 0.056054 | 0 | 0 | 0.053569 | 0 | 0 | 0 |
| Parp2 | 4.359423 | 3.826411 | 3.942646 | 5.18563 | 4.770918 | 4.656313 | 4.345079 | 5.807669 |
| Lhb | 0.185169 | 0.79614 | 0.556615 | 0.307127 | 0.120133 | 0.227299 | 0.207159 | 0.3939 |
| AABR07014 | 0 | 0 | 0.535411 | 0 | 0 | 0 | 0 | 0.442043 |
| LOC102552 | 0 | 0 | 0 | 0 | 0.18969 | 0 | 0 | 0 |
| RGD131020 | 0 | 0.019554 | 0.018554 | 0.015925 | 0.018687 | 0.030307 | 0.058005 | 0.015318 |
| Dbndd2 | 17.74048 | 19.41022 | 18.56214 | 20.16425 | 20.25362 | 20.92406 | 21.71108 | 22.22948 |
| RGD156114 | 0.043171 | 0.097691 | 0 | 0 | 0.093359 | 0.151408 | 0.096595 | 0.038264 |
| Map3k13 | 1.028244 | 1.095965 | 0.799917 | 0.837633 | 0.918464 | 0.836234 | 1.016979 | 1.056677 |
| AABR07048 | 0 | 0 | 0 | 0.114888 | 0 | 0.10932 | 0 | 0 |
| AABR07034 | 0 | 0 | 0 | 0 | 0 | 0.33032 | 0 | 0 |
| Oas1a | 4.066226 | 5.720925 | 5.200465 | 8.177945 | 8.831736 | 8.928678 | 10.83876 | 10.37354 |
| LOC100910 | 0.795399 | 0.752422 | 1.175878 | 1.321675 | 0.592168 | 0.800302 | 3.107205 | 1.68738 |
| Ccdc79 | 0.060938 | 0.091931 | 0.065421 | 0.018717 | 0.021964 | 0.142481 | 0 | 0.108024 |
| Zcchc6 | 8.724322 | 9.882467 | 9.044258 | 8.628471 | 8.558212 | 8.604585 | 10.88959 | 11.70106 |
| Fndc8 | 0.022494 | 0.050902 | 0.120743 | 0.145091 | 0.024322 | 0.059168 | 0.075496 | 0.07975 |
| AABR07064 | 0 | 0 | 2.162237 | 1.113534 | 0.435558 | 1.059565 | 0 | 0.357035 |
| Gatad1 | 31.61716 | 36.96577 | 33.16518 | 35.26424 | 33.11883 | 33.95939 | 35.22449 | 35.43851 |
| Sfrp4 | 0.22072 | 0.020811 | 0.078986 | 0.677951 | 0.755762 | 0.935385 | 1.131773 | 1.271631 |
| AABR07002 | 0.851478 | 1.926815 | 1.142646 | 0.980755 | 1.381037 | 0.746577 | 2.38149 | 2.075446 |
| Os9 | 21.96871 | 22.18548 | 22.97739 | 25.99629 | 25.99066 | 25.18112 | 31.62786 | 32.73572 |
| Napb | 1.694979 | 1.463576 | 1.532352 | 1.931769 | 1.929217 | 1.603487 | 2.095874 | 1.976768 |
| Sirt6 | 11.59845 | 11.98506 | 11.84428 | 12.16466 | 11.18176 | 11.60264 | 12.48353 | 14.40354 |
| Csnk2b | 81.74034 | 80.84177 | 86.6347 | 70.1591 | 69.22877 | 70.29942 | 82.84921 | 78.33893 |
| Prrg4 | 0.952617 | 1.04614 | 1.143012 | 1.342516 | 1.575372 | 1.031786 | 1.128437 | 1.490033 |
| Foxd1 | 0 | 0 | 0 | 0 | 0 | 0.018965 | 0.024199 | 0 |
| Hmgxb4 | 3.761514 | 3.602328 | 3.252632 | 3.70268 | 3.650826 | 3.827145 | 4.323115 | 3.572973 |
| Slc30a1 | 9.650578 | 8.308561 | 8.809666 | 8.208579 | 8.742863 | 7.70519 | 9.741665 | 8.99837 |
| Slc25a43 | 0.264474 | 0.217629 | 0.103247 | 0.420941 | 0.10399 | 0.358378 | 0.322781 | 0.319659 |
| L3hypdh | 2.451535 | 3.193091 | 2.999118 | 1.864982 | 1.756932 | 1.974548 | 2.902126 | 2.65298 |
| Zcchc5 | 0.313381 | 0.177288 | 0.168217 | 0.490906 | 0.406625 | 0.549545 | 0.280477 | 0.583306 |
| AC107267. | 0 | 0 | 0 | 0 | 0 | 0 | 0 | 0 |
| AABR07031 | 0.08392 | 0.379805 | 0.090093 | 0.077329 | 0.090741 | 0.220743 | 0.187771 | 0.148764 |
| AABR07001 | 0.924558 | 1.075152 | 0.909857 | 0.709953 | 0.361007 | 1.080871 | 0.373517 | 0.705665 |
| Slc25a19 | 9.000393 | 10.38478 | 10.54091 | 8.91634 | 8.654936 | 8.484198 | 10.10904 | 9.270291 |

| | | | | | | | | |
|---|---|---|---|---|---|---|---|---|
| Unc45a | 9.531169 | 9.904835 | 9.806681 | 10.4339 | 9.568553 | 10.31755 | 11.1066 | 11.93396 |
| Rn50_2_27 | 0.245274 | 0.555031 | 1.316585 | 1.13005 | 1.060843 | 1.075281 | 0.823204 | 0.978292 |
| Cbx4 | 5.070006 | 4.220405 | 5.909516 | 5.339213 | 5.756231 | 4.38188 | 4.173043 | 5.585149 |
| Ndufaf1 | 14.12176 | 13.30473 | 12.44704 | 13.77214 | 13.24955 | 13.17693 | 12.69437 | 13.00386 |
| Uxs1 | 12.1905 | 12.64356 | 11.29557 | 12.6372 | 12.89372 | 12.49128 | 12.31225 | 12.34862 |
| LOC100911 | 0 | 0.041843 | 0 | 0 | 0 | 0 | 0 | 0 |
| Vgll4 | 9.46913 | 9.605543 | 8.413006 | 8.76304 | 9.223776 | 8.910917 | 12.00917 | 11.43179 |
| RGD156003 | 0.033828 | 0.038275 | 0.036317 | 0 | 0.036578 | 0.029661 | 0.113536 | 0.029984 |
| Rnase12 | 0 | 0 | 0 | 0 | 0 | 0 | 0 | 0 |
| Phf3 | 3.709502 | 3.549097 | 3.539106 | 3.270879 | 3.514153 | 3.421835 | 4.045717 | 4.114323 |
| AABR07058 | 0 | 0 | 0.343842 | 0 | 0 | 0 | 0 | 0 |
| Ip6k1 | 13.67644 | 12.60312 | 13.93956 | 14.51542 | 14.46743 | 13.60964 | 17.73394 | 17.26305 |
| Atxn10 | 26.55334 | 26.78803 | 26.2433 | 28.16954 | 30.49852 | 30.27658 | 27.31614 | 28.56084 |
| Dcaf8 | 40.60224 | 39.95497 | 39.51293 | 34.58209 | 33.41345 | 36.21587 | 39.94855 | 41.12334 |
| Dpm3 | 26.71459 | 27.97061 | 18.9772 | 9.307711 | 27.30527 | 16.89746 | 23.64204 | 22.61824 |
| Ddx27 | 5.460878 | 5.715322 | 6.07198 | 5.714896 | 5.757149 | 5.506318 | 7.484 | 6.793633 |
| LOC679794 | 0.329345 | 0.186319 | 1.237507 | 0.303479 | 0.178058 | 0.28877 | 0.736914 | 0.437873 |
| AABR07059 | 0 | 0 | 0 | 0 | 0 | 0 | 0 | 0 |
| #N/A | 0 | 0 | 0 | 0 | 0 | 0 | 0.189594 | 0 |
| AABR07004 | 1.090957 | 1.697253 | 0.878409 | 0.628296 | 0.294909 | 0.717414 | 0.762821 | 0.725227 |
| AC123213. | 0.528949 | 1.196961 | 0.56786 | 0.974811 | 1.715834 | 0.463783 | 4.14235 | 0.937667 |
| Kif11 | 0.33206 | 0.212903 | 0.297073 | 0.31618 | 0.311178 | 0.339676 | 0.421027 | 0.382618 |
| Fam160a2 | 4.350474 | 4.206212 | 3.827583 | 3.366498 | 3.309333 | 4.00417 | 4.660959 | 4.47386 |
| Morn3 | 1.465569 | 1.466889 | 1.089265 | 0.934937 | 1.218999 | 1.087318 | 1.198179 | 1.64874 |
| Unc79 | 0.055282 | 0.211103 | 0.066767 | 0.031838 | 0.014944 | 0.030295 | 0.038654 | 0.030625 |
| Arl5a | 22.06311 | 19.08963 | 18.66378 | 19.63499 | 20.78878 | 19.92715 | 25.02089 | 25.44104 |
| Wdr61 | 20.21668 | 19.66892 | 17.9253 | 19.77588 | 20.88535 | 21.18841 | 20.55265 | 19.25061 |
| AABR07003 | 1.726349 | 1.953283 | 0 | 3.181525 | 3.111127 | 2.018219 | 0 | 3.570348 |
| Reg3g | 0.880669 | 0.919786 | 1.30909 | 0.18727 | 0.219751 | 0 | 0.303155 | 0.120089 |
| AABR07062 | 0 | 0 | 0.360373 | 0.309315 | 0 | 0 | 0 | 0 |
| Siae | 1.483197 | 1.52405 | 1.624802 | 1.980331 | 1.571029 | 2.109943 | 1.608539 | 1.717069 |
| Exoc2 | 8.161837 | 7.284294 | 6.854952 | 9.028215 | 6.295617 | 6.215498 | 6.120199 | 5.924594 |
| Aoah | 0.364285 | 0.515214 | 0.293312 | 0.559457 | 0.459545 | 0.63881 | 0.849053 | 0.726488 |
| Tgfa | 0.151212 | 0.092125 | 0.174823 | 0.803862 | 0.930712 | 0.764901 | 0.793795 | 0.927878 |
| AABR07035 | 1.475096 | 1.669002 | 1.58361 | 1.359243 | 2.392501 | 4.52678 | 1.650272 | 5.88353 |
| Tac3 | 0 | 0.245849 | 0 | 0.333701 | 0.234948 | 0.254022 | 0.48618 | 0.641971 |
| Prrx1 | 5.315338 | 4.083617 | 4.226929 | 8.949203 | 9.082308 | 11.10463 | 14.68287 | 12.33067 |
| Hps4 | 5.286464 | 6.477954 | 6.874678 | 5.863905 | 5.629896 | 6.051966 | 7.878243 | 7.267187 |
| AABR07014 | 0 | 0 | 0 | 0.365554 | 0 | 0 | 0 | 0 |
| AABR07003 | 2.909218 | 2.517139 | 2.755792 | 4.57301 | 4.25594 | 4.126302 | 3.63761 | 3.18531 |
| AABR07064 | 1.378051 | 0.7796 | 1.725996 | 1.481456 | 1.986755 | 2.013793 | 1.798652 | 3.257159 |
| Rn50_X_06 | 0 | 0.470235 | 0 | 0 | 0 | 0 | 0 | 0 |
| Clip4 | 13.61774 | 13.96702 | 11.94113 | 11.13534 | 10.56579 | 10.57286 | 11.77508 | 10.6189 |
| Trpv2 | 0.917059 | 1.355244 | 0.944337 | 1.276173 | 1.659416 | 1.230731 | 1.360973 | 1.492962 |
| Abi1 | 12.80153 | 12.25597 | 11.55844 | 14.53845 | 13.27423 | 14.42862 | 16.94132 | 16.60294 |
| Mark3 | 12.09628 | 13.07712 | 11.9332 | 14.99161 | 11.7695 | 12.34278 | 13.79096 | 14.86355 |
| AABR07005 | 0 | 0 | 0 | 0 | 0 | 0 | 0 | 0 |
| Slc4a8 | 0.497052 | 0.446605 | 0.486534 | 0.40413 | 0.25292 | 0.281999 | 0.408882 | 0.323943 |
| AABR07048 | 4.760538 | 2.308425 | 5.110742 | 5.013313 | 2.941429 | 4.174044 | 3.804199 | 7.233431 |
| Esam | 34.88205 | 35.8148 | 36.37465 | 30.2997 | 27.30731 | 29.6578 | 41.16588 | 41.29955 |
| AABR07033 | 0 | 0 | 0 | 0 | 0 | 0 | 0 | 0 |
| Parp8 | 3.317298 | 4.021464 | 3.36348 | 4.87626 | 4.554853 | 4.755346 | 5.095599 | 5.320518 |
| Rexo1 | 8.148419 | 7.864518 | 9.252047 | 8.590356 | 7.833204 | 7.597509 | 10.41651 | 10.37562 |
| Olr1387 | 0 | 0.126602 | 0 | 0 | 0 | 0 | 0.125181 | 0.049588 |

| | | | | | | | | |
|---|---|---|---|---|---|---|---|---|
| 5S_rRNA | 0 | 0 | 0 | 0 | 0.483953 | 0 | 0 | 0 |
| Gal3st2 | 0 | 0.050254 | 0 | 0.081854 | 0 | 0.077887 | 0 | 0.039368 |
| Cep126 | 0.34127 | 0.314625 | 0.379944 | 0.361054 | 0.36901 | 0.387885 | 0.424219 | 0.448125 |
| Gc | 0.029173 | 0 | 0 | 0 | 0.031545 | 0 | 0 | 0 |
| Scgb1d2 | 0.127102 | 0 | 0.272904 | 0.117119 | 0 | 0 | 0.142196 | 0 |
| Angptl8 | 0.417496 | 0.472377 | 0.384179 | 0.329748 | 0.386942 | 0.366061 | 0.533801 | 0.370047 |
| Nr2c2ap | 8.807934 | 8.836829 | 8.347769 | 9.479427 | 9.747108 | 8.145145 | 9.892417 | 9.66715 |
| Ntmt1 | 21.59937 | 22.07059 | 20.98632 | 22.91156 | 22.35933 | 21.5075 | 24.35174 | 22.81769 |
| AABR07034 | 2.15925 | 2.592285 | 1.840318 | 2.323805 | 2.210007 | 2.355701 | 1.844025 | 2.103774 |
| Eppin | 0.056673 | 0.128246 | 0.162246 | 0.104444 | 0.142986 | 0.049691 | 0.232479 | 0.100464 |
| Slc35f6 | 4.971337 | 4.452165 | 4.695848 | 6.070086 | 5.90728 | 5.390832 | 5.895807 | 5.542962 |
| Pgk1 | 357.0614 | 340.1869 | 320.6425 | 357.5429 | 354.3166 | 374.3859 | 331.8588 | 328.5162 |
| Hsd17b10 | 93.87618 | 90.94269 | 91.78475 | 72.92391 | 74.19921 | 71.55953 | 73.12071 | 66.90471 |
| Rarres2 | 28.96345 | 28.66351 | 28.77242 | 41.492 | 41.33944 | 41.85126 | 41.04383 | 45.11382 |
| Rsl1d1 | 0.06453 | 0 | 0.069277 | 0 | 0 | 0.05658 | 0 | 0 |
| Masp2 | 1.565758 | 1.771582 | 1.344753 | 2.597013 | 2.106884 | 2.410129 | 1.284581 | 2.189655 |
| Sctr | 7.857853 | 7.011983 | 7.799235 | 7.45929 | 7.598831 | 6.109798 | 8.260239 | 7.306476 |
| Aip | 21.0616 | 22.38156 | 24.50095 | 24.00859 | 24.53873 | 22.05916 | 25.56812 | 27.17916 |
| Synpo2 | 61.17986 | 64.85021 | 67.2937 | 55.36253 | 54.53347 | 57.16337 | 67.14563 | 63.70664 |
| Tinagl1 | 28.80126 | 32.21022 | 33.21982 | 25.94703 | 26.27799 | 27.6113 | 45.50963 | 46.96305 |
| Pten | 16.40456 | 15.977 | 16.23006 | 16.18318 | 14.53821 | 15.65267 | 21.3395 | 19.04478 |
| Upb1 | 0.088381 | 0.05 | 0.094883 | 0.16288 | 0.047783 | 0.271225 | 0 | 0.274179 |
| Pik3c2b | 10.60962 | 11.70463 | 12.38092 | 10.17568 | 10.39598 | 10.57616 | 12.18381 | 13.38019 |
| Farsa | 14.57367 | 15.43199 | 13.85858 | 17.38512 | 15.94778 | 17.02908 | 14.99741 | 15.09184 |
| LOC500567 | 0.066539 | 0.150571 | 0.10715 | 0.061313 | 0.071947 | 0.029171 | 0.03722 | 0.029488 |
| Mcpt1l1 | 5.530897 | 5.683826 | 5.610921 | 6.405697 | 5.870745 | 6.139728 | 7.152779 | 7.825704 |
| AABR07063 | 0 | 0.379805 | 0.180186 | 0 | 0.362965 | 0 | 0.751085 | 0.148764 |
| Nxpe3 | 2.07759 | 2.631975 | 2.192299 | 3.043433 | 2.419273 | 2.973776 | 2.582573 | 2.864511 |
| Strbp | 4.000452 | 3.485269 | 3.800846 | 2.98587 | 2.400725 | 2.350092 | 3.311891 | 3.155762 |
| AABR07042 | 0.048758 | 0 | 0 | 0 | 0 | 0 | 0 | 0 |
| Pik3r3 | 4.760538 | 5.183614 | 4.616154 | 5.542271 | 5.092152 | 5.744921 | 5.784019 | 5.852857 |
| AABR07047 | 0.048986 | 0 | 0 | 0.045139 | 0.052968 | 0 | 0 | 0.086837 |
| LOC684762 | 0 | 0 | 0.273568 | 0.117404 | 0.137768 | 0.335142 | 0.142542 | 0.338792 |
| Rpe65 | 0.245619 | 0.305697 | 0.184581 | 0.090531 | 0.132792 | 0.236895 | 0.054957 | 0.195934 |
| AABR0700( | 0.081503 | 0.046109 | 0.087499 | 0.037551 | 0 | 0.035731 | 0 | 0.108361 |
| Rnf146 | 17.01683 | 17.86037 | 17.06676 | 19.60042 | 18.94479 | 18.65046 | 19.60128 | 20.04432 |
| #N/A | 0 | 0 | 0 | 0 | 0 | 0 | 0.340609 | 0 |
| Exo5 | 1.922162 | 1.469484 | 1.477958 | 1.914807 | 1.71328 | 1.617028 | 1.714531 | 1.542546 |
| Asgr2 | 2.918899 | 3.302597 | 2.759461 | 4.054548 | 3.768553 | 4.049031 | 4.678975 | 4.595115 |
| Lonp2 | 28.84067 | 30.75673 | 30.984 | 30.89616 | 30.85719 | 33.03149 | 28.71576 | 28.39329 |
| Krt4 | 0.064017 | 0.072432 | 0.274905 | 0.029495 | 0.03461 | 0.028065 | 0.03581 | 0 |
| Dnajc5g | 0 | 0 | 0 | 0 | 0 | 0 | 0 | 0.059468 |
| Mocs3 | 4.137687 | 4.468798 | 5.192031 | 4.184084 | 4.270656 | 4.711594 | 4.057942 | 3.834139 |
| Man1a1 | 13.85058 | 13.04409 | 12.70792 | 17.40105 | 17.08332 | 17.6397 | 18.45644 | 17.97573 |
| Snrpf | 18.19546 | 17.72602 | 15.95828 | 20.68803 | 17.07345 | 19.03639 | 18.90718 | 16.61957 |
| AABR07049 | 0 | 0.259487 | 0.164141 | 0.140885 | 0 | 0.134057 | 0.17105 | 0.271034 |
| RGD130907 | 7.940086 | 8.48694 | 7.750978 | 8.206756 | 8.604495 | 8.794757 | 9.551207 | 9.684613 |
| Evl | 11.60046 | 11.90182 | 12.94636 | 10.05525 | 10.38198 | 10.20003 | 9.385278 | 10.60156 |
| RGD156068 | 1.043524 | 0.536681 | 1.323978 | 1.048981 | 0.718039 | 0.665427 | 0.63679 | 1.429433 |
| Ccdc177 | 0.124149 | 0.084281 | 0.159938 | 0.297435 | 0.107392 | 0.152395 | 0.083335 | 0.19807 |
| AC115415. | 2.964109 | 3.35375 | 0.53036 | 1.820873 | 0.534175 | 0.866311 | 0.552686 | 1.751491 |
| Mir3547 | 0 | 0 | 0 | 0.87733 | 0 | 0 | 0 | 0 |
| Entpd7 | 0.065348 | 0.172524 | 0.257238 | 0.321152 | 0.211981 | 0.343786 | 0.170588 | 0.308915 |
| Abcg2 | 0.431173 | 0.487852 | 0.439747 | 0.337712 | 0.466221 | 0.207929 | 0.506496 | 0.496821 |

| | | | | | | | | |
|---|---|---|---|---|---|---|---|---|
| Ckmt1b | 0 | 0.076107 | 0 | 0.092973 | 0 | 0.058978 | 0.150507 | 0.02981 |
| AABR07003 | 0.581844 | 0 | 0 | 0 | 1.887417 | 0.510161 | 1.952822 | 1.031434 |
| Mmp10 | 0 | 0 | 0 | 0 | 0.033249 | 0 | 0.034401 | 0 |
| Ccdc32 | 7.316153 | 7.826362 | 7.187928 | 8.294146 | 8.294407 | 8.553079 | 7.341685 | 8.096012 |
| Myl6 | 166.5321 | 170.6124 | 159.7779 | 219.7764 | 223.7392 | 212.0524 | 221.7086 | 214.4557 |
| Lhfp | 19.17236 | 18.76426 | 19.98928 | 25.65474 | 24.37159 | 25.99765 | 26.11572 | 26.57031 |
| Zfp35 | 1.872217 | 1.927677 | 1.82905 | 2.363489 | 2.024402 | 2.150446 | 2.094554 | 2.306621 |
| Zdhhc1 | 21.642 | 19.82274 | 20.15694 | 16.32179 | 16.9589 | 16.26491 | 18.91571 | 19.41075 |
| LOC691422 | 1.426204 | 2.070386 | 1.242231 | 1.264599 | 1.745812 | 1.462846 | 1.68589 | 1.884248 |
| Rab39a | 0.078881 | 0.06375 | 0.060489 | 0.08307 | 0.134032 | 0.088924 | 0.050428 | 0.139833 |
| Calcr | 0 | 0 | 0 | 0 | 0.016304 | 0 | 0.016869 | 0.013364 |
| 1700092M0 | 0.410713 | 0.464703 | 0.220463 | 0.567684 | 0 | 0.720227 | 0.918975 | 0.182018 |
| Aldh1a7 | 0.314196 | 0.315998 | 0.224873 | 0.386025 | 0.603973 | 0.306097 | 0.312452 | 0.216601 |
| Rn60_1_26 | 0.222518 | 0.167846 | 0.159258 | 0.615125 | 0.320807 | 0.130069 | 0.082981 | 0.328715 |
| Tmem82 | 5.090611 | 5.632733 | 5.625835 | 6.415356 | 5.747246 | 6.62954 | 11.93469 | 10.08579 |
| Trem1 | 0.356924 | 0.115384 | 0.273701 | 0.563815 | 0.330803 | 0.491781 | 0.5134 | 0.497137 |
| Ubxn2a | 26.08187 | 26.76525 | 21.77821 | 23.22609 | 28.56631 | 23.16406 | 27.67126 | 25.44734 |
| Spata17 | 0.03057 | 0.034588 | 0.065637 | 0.028169 | 0.099164 | 0.026804 | 0.1368 | 0.081286 |
| Ranbp3l | 0.353142 | 0.399564 | 0.433281 | 0.27892 | 0.354573 | 0.375986 | 0.39508 | 0.313007 |
| Nmt2 | 4.403819 | 4.343904 | 3.091241 | 3.927883 | 4.273397 | 4.67808 | 5.653183 | 6.080177 |
| Csk | 8.364353 | 9.664621 | 9.306201 | 10.36999 | 9.94868 | 9.156228 | 12.05154 | 12.10911 |
| Arpc5 | 56.65614 | 53.96708 | 55.33764 | 58.48513 | 56.82717 | 60.68457 | 69.59576 | 74.48691 |
| Pde2a | 11.59748 | 11.77542 | 11.85257 | 11.78328 | 11.48605 | 11.55633 | 10.72258 | 11.36797 |
| Shbg | 0.940705 | 0.709576 | 0.897695 | 1.05945 | 1.243209 | 0.794263 | 0.77957 | 0.802912 |
| Fxyd2 | 27.75153 | 27.03091 | 31.34745 | 36.02308 | 35.74785 | 34.27717 | 48.86555 | 50.90625 |
| Lyplal1 | 5.299874 | 5.1463 | 4.246085 | 6.778764 | 6.628768 | 6.484901 | 6.946973 | 6.835975 |
| AABR07006 | 0.287725 | 0.325547 | 0.308891 | 2.651271 | 1.555564 | 1.261387 | 0.643788 | 1.020099 |
| AABR07026 | 1.026783 | 0 | 0 | 0.47307 | 0 | 0 | 0.574359 | 0.455044 |
| RGD156547 | 0.433672 | 0.24534 | 0.349181 | 0.499515 | 0.351693 | 0.570366 | 0.121293 | 0.288289 |
| LOC100911 | 8.568969 | 12.3287 | 11.13006 | 15.79194 | 5.26189 | 3.803018 | 3.668939 | 12.75227 |
| Mir30c1 | 0.588381 | 1.331451 | 0.631665 | 0 | 0.636208 | 0 | 0 | 0 |
| Syce2 | 4.520299 | 4.406343 | 3.882263 | 3.716709 | 4.135774 | 4.146328 | 5.057109 | 4.314763 |
| Reps2 | 0.33611 | 0.228176 | 0.324752 | 0.278741 | 0.218058 | 0.294701 | 0.488832 | 0.238329 |
| Mnat1 | 8.492859 | 6.831235 | 6.740994 | 8.790156 | 8.486849 | 7.128921 | 7.970397 | 8.669276 |
| Il12a | 0.03604 | 0.081555 | 0.038691 | 0.033209 | 0.038969 | 0.0632 | 0.08064 | 0.15972 |
| Fam214a | 4.073848 | 4.197817 | 3.930976 | 3.954992 | 3.98547 | 3.954663 | 4.828928 | 4.696259 |
| Vmac | 10.04695 | 11.25283 | 12.42029 | 10.00598 | 10.42469 | 9.076119 | 12.60252 | 11.33378 |
| Tsks | 0.081104 | 0.091765 | 0.08707 | 0.224201 | 0.087696 | 0.071112 | 0.12098 | 0.143772 |
| AABR07015 | 0.107088 | 0.040388 | 0 | 0.164462 | 0.038597 | 0.125193 | 0.199675 | 0.126556 |
| Gramd3 | 31.23545 | 32.0318 | 32.08388 | 31.30799 | 32.51371 | 31.56621 | 33.04541 | 33.41483 |
| Rnf138 | 4.156025 | 3.855925 | 3.480172 | 3.216876 | 3.505203 | 3.571127 | 4.928551 | 5.083495 |
| RGD156516 | 0.171242 | 0.232503 | 0.147072 | 0.157793 | 0.185162 | 0.090087 | 0.153263 | 0.273205 |
| AABR07072 | 0.053163 | 0 | 0.057074 | 0.048988 | 0.11497 | 0 | 0.118954 | 0.047121 |
| AC096981. | 0 | 1.356861 | 0 | 0 | 0 | 0 | 0.894422 | 0 |
| Fam159a | 0.09187 | 0.207893 | 0.098628 | 0.084655 | 0.198675 | 0 | 0.20556 | 0.162858 |
| AABR07042 | 0.111893 | 0.126602 | 0.120124 | 0.103105 | 0 | 0.098108 | 0.250362 | 0.099176 |
| Rmdn2 | 1.15698 | 1.195236 | 0.972072 | 1.228346 | 1.414203 | 1.212919 | 1.322516 | 1.917218 |
| Rnaseh2c | 4.381785 | 4.145032 | 3.932958 | 4.831933 | 5.281661 | 3.77897 | 3.053796 | 5.029831 |
| Heatr4 | 1.93899 | 1.746146 | 1.416075 | 1.543614 | 1.226583 | 1.052448 | 1.313359 | 1.33281 |
| Arhgap40 | 0 | 0 | 0 | 0 | 0.028087 | 0 | 0.02906 | 0 |
| Tp53i3 | 62.9128 | 66.64926 | 64.5248 | 67.56288 | 64.2419 | 67.51893 | 59.30603 | 62.29424 |
| RGD130674 | 3.395454 | 3.627038 | 3.169771 | 4.314215 | 3.648652 | 3.883223 | 3.633523 | 4.018978 |
| Cdyl | 4.077969 | 5.265423 | 3.99167 | 5.106035 | 5.161649 | 5.615743 | 5.769905 | 5.357975 |
| Cln3 | 5.505617 | 6.901832 | 5.843465 | 6.168561 | 5.175176 | 6.74729 | 6.719388 | 7.624846 |

| | | | | | | | | |
|---|---|---|---|---|---|---|---|---|
| Gipr | 0.019388 | 0.043872 | 0.020814 | 0.07146 | 0.083854 | 0.067996 | 0 | 0.085921 |
| Ppp1r8 | 6.350539 | 5.842503 | 6.75065 | 6.293053 | 5.470883 | 6.09759 | 7.221172 | 8.267874 |
| Selenbp1 | 0 | 0.033876 | 0 | 0.027589 | 0 | 0.026252 | 0 | 0.079613 |
| AABR07017 | 0 | 0.135273 | 0 | 0.110167 | 0 | 0 | 0 | 0 |
| Rn50_X_07 | 0 | 0 | 0 | 0 | 0.076106 | 0 | 0 | 0 |
| Ace2 | 0.961708 | 0.874769 | 0.789524 | 1.337952 | 1.10105 | 1.223504 | 1.160301 | 1.337113 |
| Zfp697 | 0 | 0 | 0 | 0.427019 | 0.367462 | 0.081265 | 0.034563 | 0.027383 |
| Ahcy | 13.15522 | 13.35567 | 13.00079 | 12.85028 | 13.31484 | 14.79814 | 15.51609 | 17.01564 |
| Wbscr16 | 10.68481 | 11.1269 | 12.18357 | 11.16475 | 11.19439 | 10.96927 | 11.39662 | 11.23584 |
| Slc39a10 | 3.861856 | 3.763951 | 3.605173 | 3.722937 | 4.00556 | 3.836942 | 3.803894 | 4.492628 |
| Hipk3 | 29.85129 | 28.75369 | 28.85227 | 23.26214 | 23.1415 | 22.44103 | 30.6748 | 31.32148 |
| Aldh5a1 | 8.411306 | 8.644878 | 8.554263 | 7.306776 | 6.959308 | 6.952644 | 7.469949 | 7.480978 |
| Scn2a | 0.187124 | 0.057002 | 0.115898 | 0.225482 | 0.163424 | 0.183002 | 0.185191 | 0.14672 |
| Cd101 | 0.432089 | 0.244444 | 0.267621 | 0.275645 | 0.377364 | 0.393428 | 0.334663 | 0.309332 |
| Adcy2 | 0.423225 | 0.294682 | 0.401933 | 0.584977 | 0.633637 | 0.442446 | 0.619173 | 0.577116 |
| Iqce | 1.76571 | 1.855117 | 2.518444 | 1.580546 | 2.045611 | 1.282775 | 1.975398 | 2.280482 |
| Prox1 | 5.594866 | 5.503209 | 5.742256 | 4.075006 | 3.881792 | 3.896544 | 3.441394 | 3.849165 |
| Fxyd5 | 11.56512 | 11.35838 | 13.92849 | 24.28885 | 21.32866 | 21.61893 | 22.65887 | 19.4608 |
| AABR07030 | 26.7648 | 29.34265 | 29.09067 | 17.61622 | 24.26679 | 21.86404 | 34.0349 | 28.58545 |
| AABR07016 | 0 | 0 | 0 | 0 | 0 | 0.596292 | 0 | 0 |
| Ret | 0.73121 | 0.912185 | 1.19763 | 0.656505 | 0.81092 | 0.690443 | 0.828534 | 0.897381 |
| Gmps | 33.45498 | 31.61373 | 30.27437 | 28.67907 | 26.29045 | 27.74338 | 29.39584 | 29.39039 |
| AABR07049 | 3.288681 | 4.150333 | 4.888536 | 2.797283 | 5.607544 | 2.218091 | 3.820739 | 4.036045 |
| Smc2 | 1.968717 | 2.337783 | 2.092617 | 2.182303 | 2.402742 | 2.067989 | 2.682268 | 2.341026 |
| Chmp2b | 12.95379 | 13.48507 | 13.10259 | 13.37771 | 13.36358 | 13.67583 | 13.58021 | 13.19992 |
| Gda | 24.57674 | 26.71348 | 26.35122 | 36.87241 | 35.2078 | 36.04246 | 62.73688 | 64.49379 |
| Rn60_20_0 | 0 | 0 | 0 | 0 | 0 | 0.066736 | 0 | 0.067463 |
| LOC691722 | 1.499229 | 1.332813 | 0.574828 | 0.69074 | 0.694755 | 1.40842 | 0.119805 | 0.284752 |
| Nans | 9.358363 | 8.303041 | 9.333093 | 11.68631 | 11.25262 | 10.85088 | 12.07165 | 10.28916 |
| AC097778. | 0.045378 | 0 | 0.024358 | 0.125441 | 0.049066 | 0.079574 | 0.050767 | 0.040221 |
| Gle1 | 6.440353 | 5.008126 | 5.078743 | 6.430874 | 6.077551 | 6.755754 | 7.964999 | 7.555852 |
| Smoc2 | 3.540019 | 3.15316 | 3.537641 | 4.771525 | 3.868492 | 4.919999 | 4.276407 | 4.539644 |
| AABR07024 | 0.319304 | 0.361278 | 0 | 0 | 0.690518 | 0.279966 | 0 | 0.283015 |
| Kif22 | 0.977971 | 0.7796 | 1.026053 | 1.085491 | 0.889233 | 0.993904 | 1.193576 | 0.847124 |
| Parg | 3.729128 | 3.846601 | 3.409305 | 3.873364 | 4.146238 | 3.928265 | 4.171983 | 4.519985 |
| Tpbg | 3.392215 | 3.456861 | 4.389406 | 4.409231 | 3.910864 | 3.092481 | 4.523912 | 4.918226 |
| AABR07044 | 0 | 0 | 0 | 0.072235 | 0 | 0.068734 | 0 | 0 |
| 5S_rRNA | 0.443779 | 0 | 0 | 0 | 0 | 0 | 0 | 0 |
| Ctsh | 43.75176 | 48.23177 | 47.88413 | 73.60329 | 75.6194 | 70.45396 | 72.03095 | 70.52713 |
| Coq9 | 243.9546 | 251.9063 | 258.3727 | 211.8893 | 207.8594 | 202.67 | 237.4655 | 236.3193 |
| 43354 | 14.19141 | 13.7903 | 12.92395 | 13.23209 | 12.5108 | 12.95192 | 16.71454 | 17.60665 |
| AABR07070 | 1.057897 | 1.795442 | 1.13572 | 0.974811 | 2.859723 | 0.463783 | 1.183529 | 0.937667 |
| Nom1 | 8.296658 | 7.314762 | 6.963649 | 6.592609 | 5.941869 | 7.482361 | 8.630992 | 6.876225 |
| Cbr4 | 21.14347 | 20.5777 | 18.17833 | 20.3497 | 20.87622 | 19.28487 | 21.4493 | 19.33607 |
| LOC100359 | 0 | 0 | 0 | 0 | 0 | 0.215561 | 0 | 0 |
| Ero1a | 14.61218 | 14.06955 | 12.69909 | 14.41596 | 14.41653 | 14.9965 | 18.70792 | 17.46762 |
| Gcc2 | 2.972807 | 2.572158 | 2.747761 | 2.988354 | 3.334781 | 2.899275 | 3.121314 | 3.184481 |
| Zfyve28 | 0.118415 | 0.193529 | 0.183627 | 0.181859 | 0.241855 | 0.253799 | 0.279676 | 0.233239 |
| Ern2 | 0 | 0 | 0 | 0 | 0.019674 | 0 | 0.020356 | 0 |
| Cox6c | 1569.575 | 1535.992 | 1408.215 | 1154.629 | 1216.752 | 1154.011 | 1143.186 | 1186.886 |
| Tmem56 | 2.784892 | 2.081894 | 2.135543 | 1.832978 | 2.258448 | 2.136571 | 1.446535 | 1.89537 |
| Onecut1 | 0 | 0 | 0 | 0 | 0 | 0.039294 | 0 | 0 |
| Nubp1 | 13.97426 | 13.09101 | 14.23602 | 11.59598 | 12.91676 | 12.31852 | 14.66718 | 14.81669 |
| AABR07001 | 0.405937 | 1.377897 | 0.871599 | 1.496221 | 0 | 1.423705 | 0.908289 | 1.43921 |

| | | | | | | | | |
|---|---|---|---|---|---|---|---|---|
| Ddx10 | 3.183149 | 2.877736 | 2.160948 | 2.659961 | 3.036964 | 2.640493 | 2.827984 | 3.208632 |
| AC103056. | 0 | 0.598481 | 0 | 0 | 0 | 0 | 0 | 0 |
| Klhl35 | 0.151522 | 0.342879 | 0.260269 | 0.279243 | 0.393212 | 0.239138 | 0.305128 | 0.402904 |
| Endov | 2.418847 | 3.024897 | 2.642345 | 3.010933 | 3.303745 | 3.385914 | 2.943476 | 3.234723 |
| Chd8 | 5.582562 | 5.840225 | 6.199213 | 5.309499 | 5.922577 | 5.410323 | 6.273219 | 6.670613 |
| Plk4 | 0.389859 | 0.335242 | 0.435281 | 0.301762 | 0.236068 | 0.437541 | 0.279141 | 0.33173 |
| AABR07065 | 0.342821 | 0.517181 | 0.42938 | 0.579143 | 0.710484 | 0.526025 | 0.639222 | 0.430467 |
| Stard9 | 4.817043 | 5.102916 | 4.398338 | 4.97653 | 5.02009 | 4.788505 | 5.550216 | 5.95256 |
| Accsl | 0.096661 | 0 | 0.025943 | 0.066802 | 0.026129 | 0 | 0 | 0.064256 |
| AABR07026 | 6.193819 | 5.73383 | 6.64946 | 6.226211 | 5.78402 | 8.39297 | 2.519771 | 2.744945 |
| Tipin | 7.056401 | 8.6143 | 5.714849 | 7.928116 | 6.759898 | 6.404148 | 5.955415 | 6.857937 |
| Npnt | 0.788116 | 0.627504 | 0.752082 | 0.887599 | 0.72593 | 1.177294 | 0.751085 | 0.724418 |
| Irgc | 0 | 0.10419 | 0.032953 | 0 | 0.099571 | 0.080741 | 0.068681 | 0.054413 |
| Lsr | 1.552903 | 1.584213 | 1.229858 | 1.125985 | 1.761712 | 1.249981 | 1.395551 | 1.669749 |
| LOC498759 | 0.060191 | 0 | 0 | 0 | 0 | 0 | 0 | 0.1067 |
| Nyap2 | 0 | 0 | 0 | 0 | 0 | 0 | 0 | 0 |
| Suclg2 | 67.5164 | 69.92999 | 65.16138 | 65.71472 | 62.38938 | 64.88861 | 60.93439 | 62.16492 |
| Htra3 | 49.3184 | 51.13918 | 55.94168 | 61.02835 | 57.08663 | 60.084 | 72.51053 | 70.97236 |
| Pcyox1l | 3.864844 | 4.12653 | 4.49979 | 3.210188 | 3.207824 | 3.460291 | 3.532132 | 3.184364 |
| Inppl1 | 32.40112 | 34.32414 | 34.90403 | 28.74406 | 27.47832 | 28.91067 | 32.90834 | 31.87215 |
| AABR07042 | 0 | 0 | 0 | 0.15769 | 0.185041 | 0.075024 | 0 | 0 |
| Npsr1 | 0 | 0 | 0 | 0 | 0.030874 | 0.037553 | 0 | 0 |
| Ywhag | 72.64805 | 72.27172 | 70.23346 | 66.34565 | 68.00574 | 69.44864 | 85.85563 | 90.51479 |
| Sell | 0.772709 | 0.907911 | 1.435765 | 1.040647 | 0.996196 | 0.859919 | 0.764726 | 0.869284 |
| Pgap2 | 8.568969 | 7.592153 | 10.85424 | 9.94307 | 8.579169 | 10.17672 | 10.09381 | 9.644575 |
| AC131475. | 1.671253 | 2.521259 | 3.588393 | 1.539994 | 1.204734 | 1.953808 | 5.60917 | 4.443943 |
| Cpsf3l | 10.11915 | 8.818681 | 11.51593 | 10.76079 | 8.9419 | 10.6794 | 10.02028 | 10.98305 |
| AC097183. | 0.062119 | 0.070284 | 0.400129 | 0.11448 | 0.268671 | 0.49019 | 0.486468 | 0.055059 |
| Ogg1 | 3.283672 | 2.58117 | 2.708862 | 3.822031 | 2.803095 | 2.879126 | 2.590873 | 3.492578 |
| Gpm6a | 1.792992 | 1.670683 | 1.641819 | 3.757881 | 4.048538 | 3.74529 | 4.306828 | 3.131695 |
| Ino80d | 3.275687 | 3.077238 | 2.952058 | 2.533809 | 2.599599 | 2.503229 | 4.034525 | 3.995511 |
| Rn50_3_14 | 0.057043 | 0 | 0.06124 | 0.052563 | 0 | 0 | 0.063818 | 0.05056 |
| Ttr | 16.13558 | 13.86153 | 17.00178 | 21.20109 | 20.4358 | 21.48353 | 20.97682 | 19.73114 |
| Ash1l | 8.058996 | 8.278599 | 7.4674 | 7.241205 | 6.80533 | 6.78472 | 7.585373 | 8.529923 |
| Samd11 | 0.849177 | 0.640536 | 1.181763 | 1.33312 | 1.496331 | 1.516695 | 1.477811 | 1.951361 |
| Brpf3 | 7.22549 | 7.092858 | 8.459752 | 7.493155 | 6.152254 | 6.379465 | 8.477877 | 8.680407 |
| Akap6 | 24.39075 | 24.72985 | 23.28619 | 21.34862 | 21.76604 | 21.59916 | 22.5535 | 22.41299 |
| Uspl1 | 4.164074 | 4.016036 | 3.596115 | 4.07773 | 4.967762 | 4.445945 | 4.641391 | 4.521602 |
| C2cd3 | 1.874395 | 1.863976 | 1.894376 | 1.659714 | 1.812997 | 1.733349 | 2.244435 | 2.206507 |
| Khnyn | 9.812155 | 9.173751 | 9.951834 | 9.612558 | 8.431952 | 8.96555 | 12.07514 | 11.53497 |
| AABR07032 | 0.142106 | 0 | 0 | 0.130945 | 0.230485 | 0.186897 | 0.238472 | 0.188933 |
| RGD156082 | 0 | 0 | 0 | 0 | 0 | 0 | 0 | 0 |
| Mt3 | 0.507176 | 1.578076 | 0.816729 | 1.986207 | 1.233905 | 1.667596 | 1.418515 | 1.348606 |
| Elovl4 | 0.34037 | 0.365857 | 0.310598 | 0.203864 | 0.257626 | 0.268593 | 0.39983 | 0.301687 |
| Srpx | 12.62521 | 13.181 | 12.50662 | 14.03968 | 12.34836 | 11.22075 | 18.8113 | 18.28605 |
| AABR07067 | 0.523659 | 1.184992 | 0.562182 | 0 | 1.698675 | 0 | 0 | 0 |
| Mfap5 | 15.44851 | 21.20646 | 20.54829 | 56.52211 | 62.45672 | 59.65895 | 52.61183 | 58.49639 |
| F3 | 8.334861 | 8.812643 | 8.02235 | 10.93773 | 10.10006 | 10.43282 | 13.27962 | 12.83914 |
| Abca1 | 1.808167 | 1.816628 | 1.767183 | 1.997518 | 1.774417 | 1.945115 | 2.884186 | 3.146975 |
| Ctdspl2 | 4.90218 | 5.245608 | 4.712045 | 6.950833 | 7.642806 | 6.247435 | 5.590627 | 6.298632 |
| Pelo | 15.50005 | 14.94216 | 16.85138 | 21.25795 | 21.72065 | 20.62991 | 21.70346 | 20.38986 |
| AABR07053 | 0 | 0 | 0.183121 | 0 | 0 | 0.299117 | 0 | 0.151187 |
| Bag3 | 104.3216 | 112.904 | 118.7652 | 103.8288 | 93.46801 | 104.1359 | 133.5247 | 133.5398 |
| AABR07052 | 0.119013 | 0.269316 | 0 | 0.109666 | 0.321719 | 0.052176 | 0.066573 | 0.158231 |

| | | | | | | | | |
|---|---|---|---|---|---|---|---|---|
| AABR07034 | 0.106671 | 0.087777 | 0.104108 | 0.06255 | 0.041943 | 0.136043 | 0.097641 | 0.077358 |
| Dcp1b | 2.667502 | 3.193968 | 2.641308 | 2.195494 | 2.240258 | 2.406991 | 2.404811 | 2.685707 |
| AABR07052 | 0.089058 | 0.151147 | 0 | 0 | 0 | 0 | 0 | 0 |
| Zfp853 | 0.338664 | 0.359233 | 0.56809 | 0.487602 | 0.411967 | 0.593882 | 0.520963 | 0.881763 |
| Zfp575 | 0.184063 | 0.312388 | 0.247004 | 0.296812 | 0.348293 | 0.403466 | 0.463323 | 0.448646 |
| Ost4 | 157.9323 | 143.7569 | 155.2159 | 145.4323 | 151.3693 | 146.1581 | 145.4113 | 143.1268 |
| Lrch2 | 0.474206 | 0.740939 | 0.509091 | 0.561809 | 0.488336 | 0.395985 | 0.505258 | 0.460342 |
| Pkia | 118.8396 | 118.1084 | 109.6981 | 112.1745 | 110.5446 | 109.7195 | 103.0805 | 105.6395 |
| Ssfa2 | 5.125837 | 5.074688 | 5.355514 | 5.330533 | 5.37424 | 5.658051 | 6.215853 | 6.571536 |
| AABR07034 | 0.366195 | 0.414333 | 0 | 0 | 0 | 0.32108 | 2.048415 | 0.649154 |
| Tubb3 | 0.474168 | 0.57003 | 0.699943 | 0.955778 | 1.185644 | 1.507097 | 1.757208 | 2.022591 |
| AABR07024 | 1.036949 | 2.933147 | 1.113231 | 0.477754 | 0 | 0.454599 | 1.160092 | 2.297748 |
| Gsr | 16.39057 | 15.95321 | 15.93367 | 16.11411 | 16.67625 | 16.80419 | 17.86778 | 18.4171 |
| Elfn1 | 0.063183 | 0.200167 | 0.189926 | 0.046576 | 0.10931 | 0.055399 | 0.127235 | 0.134405 |
| Aff2 | 0.03213 | 0.021812 | 0.006899 | 0.08882 | 0.083381 | 0.157762 | 0.222865 | 0.15948 |
| Itgb3 | 2.054573 | 1.714431 | 2.012715 | 2.153524 | 1.832803 | 2.229296 | 3.505311 | 3.209636 |
| AABR07010 | 0.749369 | 0.576556 | 0.643597 | 0.718135 | 0.713048 | 0.55192 | 0.368879 | 0.690771 |
| Slc35e2b | 1.744804 | 1.653362 | 2.224375 | 1.788642 | 1.768717 | 1.950553 | 2.415611 | 2.339091 |
| Psd4 | 0.797395 | 0.889856 | 0.76224 | 0.976336 | 0.850401 | 0.670424 | 1.148719 | 0.958498 |
| Osbpl7 | 1.772135 | 1.897946 | 2.294619 | 1.994446 | 2.223359 | 2.336645 | 2.194466 | 1.990393 |
| Pygb | 84.26279 | 85.76687 | 89.39054 | 65.48557 | 67.06771 | 67.43794 | 69.98515 | 70.0303 |
| Mafk | 22.29264 | 23.85663 | 24.86777 | 24.79244 | 23.37686 | 23.9901 | 31.76197 | 31.35832 |
| Sil1 | 5.886341 | 6.878483 | 7.217196 | 6.639252 | 6.851741 | 6.261066 | 9.212331 | 8.381983 |
| Pcdhb15 | 0.399232 | 0.401522 | 0.80958 | 0.756191 | 1.103192 | 0.914011 | 0.669964 | 0.688059 |
| LOC100912 | 0.821426 | 1.85881 | 0.440927 | 0.756912 | 9.326061 | 0.360114 | 0.229744 | 8.372815 |
| Dcdc5 | 0.655319 | 0.606651 | 0.596932 | 0.347671 | 0.665642 | 0.435291 | 0.688709 | 0.404829 |
| Tacc2 | 26.93476 | 29.08682 | 28.2705 | 21.62142 | 21.74206 | 21.74934 | 30.79719 | 31.59616 |
| AC128353. | 0 | 0 | 0 | 0 | 0 | 0 | 0.02284 | 0 |
| Col24a1 | 1.380427 | 0.992602 | 0.789464 | 1.21257 | 1.534485 | 1.459219 | 1.212395 | 1.303586 |
| Trpc6 | 0.3634 | 0.370053 | 0.23408 | 0.586003 | 0.510821 | 0.557601 | 0.833439 | 0.547569 |
| AABR07006 | 0 | 0 | 0 | 0 | 0 | 0 | 0 | 0.390038 |
| #N/A | 0 | 0 | 0 | 0.194569 | 0 | 0 | 0 | 0 |
| Gjd2 | 0.027678 | 0 | 0 | 0 | 0 | 0.024268 | 0.061929 | 0 |
| Doc2a | 0.072379 | 0.040946 | 0.038852 | 0.133388 | 0.234786 | 0.126923 | 0.202435 | 0.064153 |
| Kcnq1 | 37.29494 | 39.16791 | 41.27045 | 36.83311 | 33.41928 | 34.77964 | 35.0053 | 38.5827 |
| Cyp4b1 | 0 | 0 | 0.036961 | 0 | 0 | 0.030187 | 0 | 0.030516 |
| AABR07015 | 0 | 0 | 0.124929 | 0.107229 | 0.125828 | 0 | 0.130188 | 0.103143 |
| Rasd1 | 2.009244 | 1.989194 | 2.190756 | 4.050023 | 3.326742 | 3.63352 | 5.127914 | 4.730496 |
| Carmil3 | 3.799235 | 4.511592 | 4.520688 | 3.60923 | 3.217766 | 3.248666 | 4.658349 | 4.378728 |
| Ldb3 | 306.3537 | 325.7107 | 340.6764 | 314.9931 | 309.1021 | 326.2144 | 318.783 | 318.8226 |
| AABR07062 | 2.869366 | 1.082184 | 1.540224 | 0.881336 | 2.326953 | 1.257931 | 4.280158 | 1.483569 |
| Dnal4 | 8.41532 | 8.961448 | 9.37602 | 8.9599 | 9.214062 | 9.052687 | 9.612474 | 9.903435 |
| Smap2 | 40.22687 | 40.53496 | 42.65264 | 32.83743 | 32.57138 | 31.90984 | 39.28588 | 40.73068 |
| Myo1g | 2.024941 | 2.379929 | 2.561499 | 2.618051 | 2.613869 | 2.904064 | 2.511273 | 2.671339 |
| AABR07029 | 6.9465 | 9.068813 | 9.560912 | 6.893305 | 6.548182 | 7.652414 | 11.75679 | 6.472773 |
| S1pr2 | 5.196472 | 5.728252 | 5.455684 | 6.337515 | 5.680843 | 5.980106 | 5.728088 | 5.571097 |
| AABR07065 | 0 | 0 | 0 | 0 | 0 | 0 | 0 | 0 |
| Mmp23 | 3.232464 | 3.975414 | 3.228848 | 6.682399 | 5.805099 | 7.196473 | 7.4528 | 6.627086 |
| Rdm1 | 7.614362 | 6.749164 | 8.085972 | 8.485459 | 7.609115 | 7.158321 | 7.811289 | 7.747935 |
| AABR07002 | 3.117019 | 2.351174 | 4.907935 | 3.255172 | 3.819773 | 4.555008 | 7.439323 | 4.972984 |
| Gif | 0 | 0 | 0 | 0.03305 | 0 | 0 | 0 | 0.031791 |
| LOC103690 | 0 | 0 | 0 | 0 | 0.117231 | 0.047531 | 0 | 0.096096 |
| Rpl12-ps1 | 22.39747 | 74.00248 | 83.19836 | 41.37367 | 32.97295 | 41.028 | 18.94002 | 14.07348 |
| Fam172a | 4.481768 | 4.430374 | 3.899818 | 6.824993 | 5.305172 | 4.053711 | 4.64455 | 5.352305 |

| | | | | | | | | |
|---|---|---|---|---|---|---|---|---|
| Sap30bp | 6.119833 | 5.6143 | 6.019569 | 6.203104 | 6.349018 | 5.699429 | 6.272963 | 6.78772 |
| Fam196a | 0.035526 | 0.120589 | 0.07628 | 0.032736 | 0.089633 | 0.155748 | 0.079491 | 0.062978 |
| Stac3 | 0.033872 | 0 | 0.036364 | 0.031212 | 0.07325 | 0.029699 | 0.037894 | 0 |
| AABR07026 | 0 | 0.18009 | 0 | 0 | 0 | 0 | 0.178069 | 0 |
| Pdha2 | 0 | 0 | 0.036529 | 0 | 0 | 0 | 0 | 0 |
| Slc4a2 | 7.312882 | 7.17778 | 8.017295 | 7.690976 | 7.935255 | 7.431508 | 10.91326 | 10.13492 |
| Znrd1-as1 | 0 | 0.050771 | 0 | 0.041348 | 0 | 0.039344 | 0 | 0 |
| LOC100911 | 0.933396 | 0.920697 | 1.079142 | 0.650579 | 0.983389 | 0.650525 | 1.05763 | 1.240973 |
| Rn50_20_0 | 8.860393 | 8.560775 | 7.481504 | 8.898392 | 10.76474 | 10.30021 | 9.132971 | 9.97118 |
| Pcmtd2 | 40.48135 | 40.14004 | 40.62226 | 37.16593 | 38.58376 | 37.35811 | 34.27211 | 35.23944 |
| Olr395 | 0 | 0 | 0 | 0 | 0.060301 | 0 | 0 | 0 |
| LOC100912 | 0.101879 | 0.115272 | 0.145832 | 0.12517 | 0.03672 | 0.14888 | 0.227956 | 0.210701 |
| Armcx6 | 1.275893 | 1.689335 | 2.127489 | 1.675977 | 1.497018 | 2.380222 | 2.095563 | 1.876792 |
| AABR07048 | 0 | 0 | 0 | 0 | 0 | 0 | 0 | 0 |
| Siah2 | 3.482463 | 3.706747 | 3.877115 | 3.589273 | 3.124001 | 2.962954 | 4.530932 | 4.20703 |
| #N/A | 0.417258 | 0 | 0 | 0.192244 | 0.451175 | 0 | 0.233405 | 0.369837 |
| AC111632. | 0 | 0 | 0 | 0 | 0 | 0.009203 | 0 | 0.009303 |
| Vps26b | 17.016 | 16.17103 | 17.35594 | 15.18031 | 15.20067 | 15.7157 | 17.05443 | 17.02904 |
| Pabpn1l | 0.092765 | 0.157439 | 0.099589 | 0 | 0.200611 | 0.162673 | 0.570798 | 0.246667 |
| Bex2 | 0.202707 | 0 | 0.07254 | 0.435835 | 0.073061 | 0.059244 | 0 | 0.05989 |
| AABR07052 | 0 | 0 | 0 | 0 | 0 | 0 | 0 | 0.473618 |
| Ankrd34a | 1.188887 | 1.014051 | 1.119258 | 0.758432 | 0.771316 | 0.673562 | 1.002672 | 1.102406 |
| Man1a2 | 6.291738 | 6.556794 | 5.364883 | 6.074985 | 5.956838 | 5.754158 | 6.112743 | 6.964179 |
| Mdfi | 0.550879 | 0.403308 | 0.591404 | 0.686771 | 0.840928 | 0.82396 | 0.833816 | 0.689325 |
| AC095267. | 0.176912 | 1.201005 | 0.189926 | 0.489052 | 0 | 0 | 0.791685 | 0.156806 |
| Sppl3 | 8.196069 | 8.85791 | 8.840508 | 8.229217 | 7.942619 | 8.457486 | 10.22907 | 9.389131 |
| AABR07041 | 0 | 0 | 0 | 0.081785 | 0 | 0 | 0 | 0.039334 |
| Il33 | 8.499293 | 9.350097 | 7.952365 | 8.00931 | 8.634586 | 8.84126 | 12.38278 | 12.03058 |
| Ly9 | 0.345913 | 0.270958 | 0.37136 | 0.392302 | 0.258944 | 0.209975 | 0.357224 | 0.400938 |
| Fhl3 | 8.388564 | 7.968413 | 8.80404 | 7.066359 | 8.15662 | 6.120102 | 7.283688 | 6.963565 |
| Igsf11 | 0.173685 | 0.294774 | 0.202 | 0.413446 | 0.594709 | 0.406098 | 0.259081 | 0.282233 |
| Rimklb | 0.222078 | 0.201016 | 0.238415 | 0.409272 | 0.432233 | 0.584154 | 0.397521 | 0.551148 |
| Eif2b4 | 20.86611 | 22.73882 | 25.38074 | 21.41502 | 19.38037 | 21.52058 | 22.03472 | 24.12606 |
| Ccdc122 | 0.241715 | 0.19535 | 0.278032 | 0.302278 | 0.448052 | 0.363319 | 0.289736 | 0.321367 |
| Zfp513 | 12.11414 | 11.768 | 13.21256 | 13.31964 | 12.86401 | 14.66301 | 14.92964 | 14.84409 |
| Rn50_X_07 | 0.085565 | 0.096813 | 0 | 0 | 0.185041 | 0.075024 | 0 | 0 |
| AABR07028 | 7.023194 | 6.041133 | 8.465794 | 7.114972 | 7.727308 | 7.41834 | 7.122057 | 9.2465 |
| AABR07072 | 0.200764 | 0 | 0.035922 | 0.092498 | 0 | 0 | 0 | 0.326236 |
| Glyctk | 3.604132 | 4.317783 | 4.409825 | 2.954772 | 3.667855 | 3.043926 | 3.439181 | 3.311967 |
| AABR07067 | 0 | 0 | 0 | 0.092086 | 0.108058 | 0 | 0 | 0 |
| Epm2aip1 | 13.89489 | 12.77869 | 12.20138 | 12.73793 | 12.23521 | 12.61414 | 11.99754 | 12.03784 |
| AABR07029 | 0 | 0 | 0 | 0 | 0 | 0 | 0 | 0 |
| AABR07054 | 0.234614 | 0.371637 | 0.100749 | 0.129713 | 0.659581 | 0.20571 | 0.314971 | 0.16636 |
| Atp2a2 | 1645.135 | 1662.638 | 1656.293 | 1288.473 | 1266.196 | 1317.659 | 1375.447 | 1387.784 |
| Olr1432 | 0.161623 | 0 | 0 | 0.049643 | 0 | 0.047237 | 0 | 0.047752 |
| Bik | 0.492985 | 0.433836 | 0.352834 | 0.454266 | 0.177686 | 0.480277 | 0.61281 | 0.291305 |
| AABR07051 | 2.48991 | 1.76076 | 1.837741 | 2.00756 | 1.346152 | 1.70559 | 2.002151 | 1.103466 |
| Necab3 | 0.831205 | 0.758443 | 0.949923 | 1.655381 | 1.739555 | 1.71621 | 1.439869 | 1.996323 |
| Adrbk2 | 0.493806 | 0.406341 | 0.337357 | 0.537755 | 0.461135 | 0.531372 | 0.477115 | 0.616738 |
| Wdr18 | 22.45874 | 22.54478 | 27.52052 | 23.79559 | 22.8943 | 22.41025 | 23.34927 | 24.06182 |
| Ap5z1 | 3.598479 | 2.916902 | 3.055962 | 2.573501 | 3.890983 | 3.626067 | 3.965731 | 4.474836 |
| AC098622. | 8.470958 | 11.47234 | 11.71212 | 8.988329 | 10.82489 | 8.890305 | 6.030774 | 8.873364 |
| Wdsub1 | 7.321915 | 6.088889 | 6.527306 | 7.652795 | 8.140885 | 7.372632 | 6.541568 | 7.063079 |
| Med20 | 11.38265 | 11.97288 | 11.2989 | 13.62472 | 12.92638 | 12.71362 | 13.15035 | 13.05488 |

| | | | | | | | | |
|---|---|---|---|---|---|---|---|---|
| Fyb | 1.474667 | 1.46139 | 1.310192 | 1.368219 | 1.341609 | 1.426746 | 2.048017 | 1.838913 |
| AABR07053 | 1.190135 | 0.44886 | 0 | 0.731108 | 0 | 0 | 0.443823 | 0.351625 |
| Ammecr1 | 2.657375 | 3.06565 | 4.307262 | 3.552967 | 3.098744 | 2.375675 | 3.147833 | 3.694688 |
| Aldoa | 993.3476 | 1007.201 | 1014.972 | 934.1779 | 904.023 | 918.8788 | 954.5167 | 955.9803 |
| Cd79b | 0.433493 | 0.196191 | 0.279229 | 0.359502 | 0.562475 | 0.608139 | 0.387978 | 0.15369 |
| Nudt13 | 7.950416 | 8.228462 | 7.91774 | 8.55646 | 7.508203 | 8.249837 | 6.389383 | 6.755507 |
| Ddx6 | 19.52562 | 18.44687 | 18.06566 | 15.75649 | 15.29941 | 16.33725 | 18.91299 | 19.52575 |
| Slc24a2 | 0.018599 | 0.021044 | 0.049918 | 0.042846 | 0.020111 | 0.008154 | 0.020808 | 0.041213 |
| AABR07025 | 12.25199 | 12.1169 | 10.96108 | 9.491735 | 9.81326 | 10.14575 | 9.848722 | 10.49756 |
| C1qtnf6 | 1.606642 | 1.538172 | 1.516336 | 2.684345 | 2.15723 | 2.29108 | 2.46901 | 2.503818 |
| Dis3l2 | 5.333383 | 5.386325 | 5.789347 | 4.441254 | 4.506733 | 5.040028 | 4.839699 | 5.234983 |
| Rbm41 | 1.9266 | 1.88212 | 1.705109 | 1.749306 | 1.900289 | 1.812848 | 1.797914 | 2.015849 |
| Bmpr2 | 18.16363 | 19.29555 | 17.93053 | 15.61461 | 16.17159 | 15.21385 | 21.30489 | 21.98961 |
| AABR07052 | 0 | 0 | 0.114265 | 0 | 0 | 0 | 0 | 0.094338 |
| Siah3 | 0.06489 | 0.146839 | 0 | 0.059793 | 0 | 0.056895 | 0 | 0 |
| Oas1e | 0.306906 | 0.477469 | 0.041185 | 0.176751 | 0.207408 | 0.134548 | 0.51503 | 0.374036 |
| RGD156253 | 0 | 0 | 0 | 0 | 0 | 0 | 0.023434 | 0 |
| Slc25a14 | 1.570105 | 1.447519 | 2.278693 | 2.786411 | 2.357961 | 2.29445 | 2.569788 | 2.139037 |
| St3gal3 | 12.32139 | 14.03925 | 13.46069 | 10.47417 | 9.945296 | 11.98265 | 11.84313 | 11.92088 |
| AABR07021 | 0.046881 | 0.053043 | 0 | 0 | 0 | 0.041105 | 0 | 0.041553 |
| RGD156226 | 0 | 0 | 0 | 0 | 0.141911 | 0 | 0 | 0 |
| Cdk7 | 8.14751 | 9.478201 | 7.679179 | 9.340453 | 9.719715 | 7.51267 | 9.243454 | 9.120165 |
| AABR07067 | 0 | 0.110747 | 0 | 0 | 0.211673 | 0 | 0 | 0.173512 |
| Col4a6 | 0.20235 | 0.397648 | 0.320136 | 0.461236 | 0.506689 | 0.438882 | 0.559992 | 0.623014 |
| LOC685680 | 0 | 0 | 0.047362 | 0.040651 | 0.047702 | 0 | 0.049355 | 0.078205 |
| Ankrd24 | 0.211518 | 1.042765 | 1.654429 | 0.083531 | 0.555443 | 0.079483 | 0.050708 | 0.026783 |
| Fuca2 | 12.49677 | 11.55564 | 12.2924 | 14.058 | 14.40427 | 13.26542 | 14.30019 | 14.87176 |
| AC141169. | 0 | 0.75961 | 0 | 0 | 2.177789 | 0.588647 | 0.751085 | 0 |
| Lpar6 | 8.4816 | 7.893393 | 8.639386 | 7.33533 | 6.98 | 8.096584 | 7.319035 | 6.388731 |
| Myef2 | 9.859351 | 10.29492 | 8.572686 | 11.48765 | 10.30835 | 11.83584 | 12.51913 | 11.33882 |
| Zfp259 | 5.713961 | 5.135236 | 4.756025 | 6.148276 | 6.276183 | 5.850292 | 5.421509 | 5.545381 |
| Mapk10 | 0 | 0.169285 | 0 | 0 | 0.161779 | 0.104947 | 0.066954 | 0.053045 |
| Rn50_5_04 | 0 | 0.2759 | 0 | 0.056174 | 0 | 0.053451 | 0 | 0.054033 |
| Foxm1 | 0.507388 | 0.336533 | 0.544713 | 0.435294 | 0.624304 | 0.674987 | 0.685086 | 0.635815 |
| AABR07026 | 34.37353 | 37.9805 | 39.78516 | 41.07703 | 31.06979 | 28.96144 | 37.25384 | 34.27534 |
| Pcdhb11 | 1.038738 | 0.562092 | 0.945454 | 0.790694 | 0.75692 | 1.148357 | 1.086305 | 1.120834 |
| Mospd4 | 0.128034 | 0 | 0.137453 | 0 | 0.069221 | 0 | 0 | 0 |
| AC126134. | 0 | 0 | 0 | 0 | 0 | 0 | 0.476298 | 0 |
| LOC686087 | 51.87652 | 48.0826 | 41.682 | 47.05056 | 42.95197 | 41.76645 | 42.43282 | 37.44972 |
| Kap | 0 | 0 | 0 | 0 | 0 | 0 | 0 | 0.065836 |
| Map2k6 | 5.969172 | 6.670112 | 6.408288 | 3.545685 | 4.240687 | 4.498452 | 3.449403 | 3.782248 |
| Chfr | 4.834544 | 4.154237 | 5.047506 | 4.531386 | 5.407163 | 4.486568 | 5.631711 | 5.860716 |
| Slc17a5 | 1.265049 | 1.149107 | 1.281598 | 1.691076 | 1.79173 | 2.030923 | 2.551493 | 2.400479 |
| Tnfaip8l1 | 5.507289 | 6.548081 | 4.409268 | 5.160763 | 5.652158 | 5.401704 | 3.968302 | 4.96412 |
| Sdcbp | 97.33147 | 96.04516 | 88.54704 | 108.3513 | 111.1684 | 108.6007 | 112.0414 | 110.5551 |
| Fbf1 | 3.443271 | 4.015568 | 3.948897 | 3.909197 | 3.913764 | 3.750645 | 4.706757 | 4.489376 |
| Bco1 | 0 | 0.026871 | 0.025496 | 0.043767 | 0 | 0.020823 | 0 | 0.02105 |
| Dnase1 | 0.162375 | 0.13779 | 0.08716 | 0.224433 | 0.13168 | 0.035593 | 0 | 0.143921 |
| Ddx17 | 118.5891 | 117.4264 | 113.0236 | 130.6128 | 132.2506 | 130.8691 | 146.8288 | 147.8089 |
| RGD130511 | 7.219694 | 6.833469 | 5.415625 | 6.503405 | 6.930816 | 6.309945 | 5.98014 | 6.993968 |
| AABR07035 | 0 | 0 | 0 | 0 | 0 | 0.011229 | 0 | 0 |
| AABR07028 | 6.439822 | 7.286356 | 7.459368 | 6.40252 | 6.047064 | 6.389394 | 5.498237 | 8.561901 |
| Tpmt | 13.44094 | 11.74042 | 13.33305 | 12.9798 | 15.46364 | 16.35762 | 12.87178 | 12.62818 |
| LOC687508 | 0.49104 | 0.595274 | 0.677781 | 0.517113 | 0.682656 | 0.738076 | 0.510114 | 0.590674 |

| | | | | | | | | |
|---|---|---|---|---|---|---|---|---|
| Klk5 | 0 | 0 | 0 | 0.056635 | 0 | 0.05389 | 0.068761 | 0 |
| Cdkn2aip | 4.785883 | 4.54755 | 3.990641 | 4.645488 | 5.099543 | 4.603671 | 6.367898 | 5.353937 |
| Gpr18 | 0.479322 | 0.451942 | 0.343055 | 0.257645 | 0.259142 | 0.28018 | 0.223435 | 0.318635 |
| AABR07044 | 0 | 0 | 0 | 0 | 0 | 0.108802 | 0 | 0 |
| Mk1 | 8.980019 | 8.066508 | 7.428022 | 8.410386 | 8.345567 | 8.850985 | 9.717055 | 9.282904 |
| Gnl2 | 5.189644 | 5.182723 | 5.489682 | 5.226351 | 4.980366 | 5.073178 | 5.181343 | 5.274633 |
| Rffl | 4.27694 | 4.863232 | 4.226071 | 4.333175 | 2.922007 | 3.768682 | 4.784851 | 4.243505 |
| Map10 | 0.974947 | 0.912185 | 1.066796 | 0.846546 | 1.074469 | 0.887713 | 0.713168 | 0.797672 |
| Pcf11 | 5.305466 | 4.882672 | 5.695757 | 6.336571 | 5.922906 | 6.793759 | 8.235083 | 7.878831 |
| Zmiz2 | 0.611415 | 1.193673 | 1.621678 | 0.905851 | 1.179636 | 1.08269 | 0.603551 | 1.508897 |
| AABR07068 | 0.689025 | 0 | 0 | 0 | 0 | 0 | 1.541702 | 0 |
| Rn50_X_07 | 73.99098 | 73.62908 | 71.57636 | 56.77561 | 57.12614 | 59.21627 | 61.48785 | 62.63306 |
| Pomt1 | 2.843013 | 2.504046 | 3.216644 | 3.011899 | 3.202964 | 3.298798 | 3.237774 | 3.229098 |
| AABR07055 | 0 | 0 | 0 | 0.313332 | 0 | 0.298146 | 0 | 0 |
| Slc2a1 | 7.104405 | 6.284489 | 7.211012 | 9.593495 | 9.106531 | 9.196488 | 8.208212 | 8.449412 |
| Xrra1 | 0.026704 | 0.090642 | 0.028668 | 0.024606 | 0.086623 | 0.163897 | 0 | 0.071006 |
| Ntrk3 | 0.434572 | 0.368773 | 0.39656 | 1.081191 | 0.892803 | 1.124048 | 0.583416 | 0.924439 |
| Morn1 | 0.80874 | 0.714884 | 0.922499 | 0.838375 | 1.393701 | 0.864221 | 0.989606 | 0.739227 |
| Pcnp | 61.03578 | 59.05339 | 56.68354 | 56.80129 | 59.43489 | 58.00125 | 64.40433 | 63.70469 |
| Milr1 | 0.211793 | 0.658994 | 0.397904 | 0.487898 | 0.74428 | 0.603527 | 0.47389 | 0.750892 |
| Elk1 | 20.21141 | 21.39945 | 22.68452 | 16.15799 | 16.19637 | 15.77763 | 17.47262 | 17.27711 |
| RGD156504 | 0 | 0 | 0 | 0 | 0 | 0 | 0 | 0.077746 |
| Nkapl | 0.134444 | 0.038029 | 0.036084 | 0.061942 | 0.036343 | 0.147351 | 0.112807 | 0.178747 |
| LOC100362 | 8.000349 | 6.85759 | 7.547809 | 8.265583 | 5.767108 | 7.864981 | 6.509407 | 4.942287 |
| AABR07069 | 0 | 0.12267 | 0.116394 | 0.099903 | 0 | 0 | 0.121293 | 0.096096 |
| Sumo4 | 11.69541 | 11.08694 | 12.72544 | 11.01958 | 11.10804 | 10.16216 | 13.0843 | 10.41291 |
| LOC100911 | 0.073465 | 0.132996 | 0.094643 | 0.067695 | 0.095324 | 0.025766 | 0.131503 | 0.104185 |
| RGD156632 | 17.8336 | 15.59199 | 16.88285 | 11.57776 | 13.23529 | 14.35716 | 13.69394 | 12.93284 |
| AABR07034 | 0 | 0 | 0.449745 | 0 | 0.45298 | 0 | 0 | 0 |
| AC131411. | 0 | 0 | 0.573655 | 0 | 0 | 0 | 0 | 0 |
| Pcmt1 | 113.6739 | 123.7658 | 111.7788 | 118.6293 | 113.6424 | 120.0221 | 117.2379 | 120.0263 |
| Scyl3 | 2.807209 | 2.61689 | 2.61568 | 2.635539 | 2.978123 | 2.538765 | 2.903556 | 3.051528 |
| AABR07001 | 0 | 0 | 0 | 0 | 0 | 0 | 0 | 0 |
| AC119536. | 1.731105 | 3.917327 | 1.393839 | 1.595145 | 1.871819 | 1.517834 | 3.873366 | 1.534364 |
| AABR07004 | 0 | 0.356925 | 0 | 0 | 0 | 0.069148 | 0 | 0 |
| Ebp | 3.229397 | 3.710125 | 3.306951 | 3.525134 | 2.900964 | 4.051278 | 3.279407 | 4.535764 |
| Icam1 | 4.061678 | 4.304169 | 4.381741 | 5.550115 | 4.348986 | 6.34082 | 7.425601 | 6.392313 |
| Bend6 | 0.510531 | 0.783943 | 1.096176 | 2.755402 | 1.892675 | 1.886459 | 3.018987 | 2.553445 |
| AC110474. | 0 | 0 | 0 | 0 | 0 | 0 | 0 | 0 |
| AABR07021 | 0 | 0 | 0 | 0 | 0 | 0.259404 | 0 | 0 |
| Mtr | 4.621646 | 3.500522 | 3.485444 | 3.120674 | 3.827148 | 3.237345 | 4.614985 | 4.209243 |
| Pglyrp4 | 0 | 0.035714 | 0.033887 | 0 | 0 | 0 | 0 | 0 |
| Zmynd10 | 1.665775 | 2.021819 | 1.495683 | 1.814028 | 1.408194 | 1.407442 | 2.371849 | 2.228112 |
| Gpr146 | 41.34455 | 42.07862 | 42.55854 | 40.14448 | 36.81243 | 40.82974 | 38.60455 | 37.8988 |
| Sgk1 | 16.61496 | 13.90276 | 13.70291 | 14.304 | 14.35954 | 15.22941 | 18.85458 | 18.8438 |
| AABR07036 | 0 | 0 | 0 | 0 | 0 | 0 | 0.523077 | 0 |
| Rpl8 | 8.659997 | 7.731534 | 8.207561 | 7.917504 | 8.412906 | 8.126983 | 7.947532 | 7.615819 |
| RGD156091 | 1.034389 | 0.731476 | 1.388103 | 0.953148 | 0.838852 | 0.793584 | 0.289307 | 0.458415 |
| Grin3a | 0 | 0 | 0 | 0.074997 | 0.140808 | 0.042817 | 0.018211 | 0.057712 |
| Rn50_10_( | 0.654063 | 0.497215 | 0.581492 | 0.602693 | 0.607775 | 0.645168 | 0.937538 | 1.014525 |
| Zfp780b | 1.021391 | 1.022311 | 0.674787 | 0.796376 | 0.976983 | 0.809445 | 0.922939 | 1.166456 |
| Heatr3 | 5.148465 | 4.6532 | 4.74709 | 4.701368 | 4.864822 | 5.178427 | 4.704762 | 4.261859 |
| Fbll1 | 0.031853 | 0 | 0 | 0 | 0 | 0 | 0 | 0.028233 |
| AABR07039 | 0.957913 | 0.361278 | 0.342794 | 1.176906 | 0.345259 | 1.959765 | 0.714447 | 1.415077 |

| | | | | | | | | |
|---|---|---|---|---|---|---|---|---|
| Unc5a | 0.058249 | 0.219687 | 0.083379 | 0.107348 | 0.146962 | 0.102146 | 0.173777 | 0.154887 |
| LOC498265 | 0.479281 | 0.381607 | 0.552653 | 0.637923 | 0.575822 | 1.042804 | 0.99296 | 0.660817 |
| Pip4k2c | 10.28043 | 10.19804 | 9.92536 | 11.72614 | 9.842359 | 11.09522 | 11.24171 | 11.78555 |
| Rhox8 | 0 | 0 | 0 | 0 | 0 | 0 | 0 | 0 |
| Stk11 | 30.72447 | 31.34396 | 32.10149 | 28.91475 | 27.88464 | 28.61743 | 33.80968 | 32.97678 |
| Tbx22 | 0 | 0 | 0 | 0.013966 | 0 | 0 | 0 | 0 |
| AABR07034 | 0 | 0 | 0 | 0 | 0 | 0 | 0 | 0 |
| AABR07002 | 1.69469 | 1.725716 | 2.365165 | 1.093113 | 0.916222 | 1.337315 | 0.568783 | 1.051462 |
| Degs1 | 24.56133 | 27.62506 | 28.36337 | 27.43411 | 26.479 | 27.31864 | 30.65809 | 28.61744 |
| Pla1a | 13.33392 | 12.89227 | 12.16759 | 13.4595 | 13.53304 | 13.52458 | 14.64617 | 14.8 |
| 5S_rRNA | 0 | 0 | 0 | 0.869426 | 0 | 0 | 0 | 0 |
| Ptbp3 | 10.38699 | 10.04561 | 8.814452 | 8.161333 | 8.178807 | 8.729421 | 10.31862 | 11.05926 |
| Sybu | 1.219782 | 1.437631 | 1.127637 | 1.404976 | 1.72194 | 1.737947 | 1.307778 | 1.156234 |
| Fxn | 50.25925 | 47.07617 | 47.17156 | 41.66686 | 37.09751 | 42.35215 | 39.39314 | 38.4787 |
| Ckmt2 | 1237.239 | 1140.2 | 1117.594 | 1083.695 | 1104.591 | 1080.272 | 1030.184 | 1039.552 |
| AABR07030 | 0 | 0.274304 | 0.130135 | 0.111697 | 0.131071 | 0.106284 | 0.135613 | 0.322323 |
| Stxbp1 | 9.424289 | 10.37314 | 10.03291 | 12.3358 | 12.89782 | 12.34298 | 14.69028 | 12.94923 |
| RGD130903 | 9.271772 | 9.899555 | 10.0473 | 8.343019 | 8.519264 | 6.411998 | 8.717087 | 8.565273 |
| LOC100362 | 30.71162 | 1.413509 | 1.900017 | 1.247099 | 1.801114 | 1.643063 | 20.49881 | 1.107305 |
| Ssbp3 | 11.45245 | 11.17385 | 10.63186 | 11.0373 | 11.39629 | 11.47256 | 15.04075 | 12.65182 |
| AABR07003 | 0.727304 | 5.760375 | 2.342423 | 4.691277 | 10.22351 | 2.550805 | 4.882055 | 1.289292 |
| AABR07001 | 0 | 0 | 0.05766 | 0 | 0 | 0 | 0 | 0 |
| Cyp4f37 | 1.3274 | 2.478119 | 0.926281 | 0.795045 | 1.937653 | 0.581933 | 0.965273 | 2.647216 |
| AC094067. | 0 | 0 | 0.432447 | 0 | 0 | 0.353188 | 0 | 0.357035 |
| AABR07055 | 0.055946 | 0.063301 | 0.180186 | 0.051552 | 0 | 0.098108 | 0 | 0.099176 |
| Lmbrd2 | 1.470953 | 1.564455 | 1.500204 | 1.62651 | 1.542804 | 1.83142 | 2.17224 | 1.642761 |
| LOC100911 | 0.047049 | 0 | 0 | 0.043354 | 0 | 0 | 0 | 0 |
| 5S_rRNA | 0 | 0.462887 | 0 | 0.376978 | 0.442363 | 0.358707 | 0 | 0 |
| RGD156326 | 0 | 0 | 0.13074 | 0.112217 | 0 | 0 | 0 | 0 |
| AABR07013 | 0 | 0.365738 | 0 | 0 | 0 | 0 | 0 | 0.286509 |
| Rnf152 | 0.992538 | 1.090615 | 0.963096 | 0.81785 | 0.577886 | 0.937201 | 0.843483 | 1.031998 |
| Helt | 0 | 0 | 0 | 0 | 0 | 0 | 0.08103 | 0.064197 |
| Gsta5 | 0 | 0 | 0 | 0.060091 | 0.070514 | 0 | 0 | 1.213829 |
| Cnr2 | 0.711856 | 0.780262 | 0.811987 | 0.696944 | 0.625397 | 0.721681 | 0.895942 | 1.143603 |
| Rn60_X_07 | 1.217812 | 0.688949 | 0.32685 | 1.402707 | 1.207069 | 0.444908 | 0.681217 | 0.809556 |
| Ildr1 | 0.021149 | 0.02393 | 0 | 0 | 0 | 0 | 0 | 0.018746 |
| Fam212b | 13.60275 | 13.93592 | 13.00669 | 11.5779 | 11.60929 | 9.997947 | 12.77423 | 12.72966 |
| Ddx46 | 17.09789 | 16.75514 | 16.97123 | 19.49352 | 19.22408 | 19.22209 | 16.34016 | 17.65909 |
| Ippk | 2.198032 | 1.890097 | 1.90666 | 2.47909 | 2.148537 | 2.790639 | 3.265633 | 3.11716 |
| Tmc6 | 7.426844 | 8.428284 | 7.758345 | 7.601662 | 8.030539 | 8.227564 | 9.677042 | 10.74136 |
| Rufy4 | 0.041429 | 0.046875 | 0.022238 | 0.057262 | 0.111991 | 0.145299 | 0.139046 | 0.091801 |
| Eftud2 | 13.725 | 12.91131 | 13.21389 | 12.89361 | 12.04952 | 12.33771 | 14.79144 | 15.3599 |
| LOC257642 | 3.538238 | 36.91978 | 32.49849 | 23.18469 | 24.48541 | 28.67932 | 1.231509 | 1.463521 |
| LOC102553 | 5.680188 | 6.059616 | 6.272274 | 5.134373 | 5.556961 | 5.739311 | 6.475784 | 5.801815 |
| Cd81 | 191.9005 | 182.7396 | 180.515 | 236.679 | 224.871 | 217.8987 | 223.156 | 217.0435 |
| Lamb2 | 128.1455 | 130.2892 | 139.1201 | 115.8613 | 113.1858 | 117.1823 | 146.8751 | 147.3716 |
| Herc6 | 6.463668 | 6.058678 | 5.404086 | 9.46509 | 10.01819 | 8.763283 | 15.85002 | 16.48886 |
| Creb5 | 1.361804 | 1.89007 | 1.676408 | 2.844325 | 2.434532 | 2.977118 | 4.164305 | 3.846418 |
| Ttc19 | 8.329857 | 9.82992 | 8.378915 | 8.555364 | 8.92953 | 8.559264 | 8.678221 | 9.731394 |
| Oasl2 | 4.642636 | 4.912653 | 4.277907 | 6.529588 | 5.670381 | 6.312006 | 9.357565 | 10.52907 |
| Slc16a7 | 2.006357 | 1.787703 | 1.642389 | 0.87817 | 1.301667 | 1.187444 | 1.234543 | 0.978084 |
| Il27 | 0.074278 | 0.084042 | 0.079742 | 0.068444 | 0 | 0.065127 | 0.166198 | 0.065836 |
| AABR07046 | 0.588381 | 0 | 0 | 0 | 0 | 0.515893 | 0 | 0 |
| Nod2 | 0.083121 | 0.120918 | 0.089235 | 0.339194 | 0.320989 | 0.124937 | 0.26569 | 0.263121 |

| | | | | | | | | |
|---|---|---|---|---|---|---|---|---|
| AABR07071 | 0.074436 | 0.084221 | 0 | 0.06859 | 0.12073 | 0.065266 | 0.083276 | 0.098965 |
| LOC100911 | 6.160697 | 22.77503 | 7.006806 | 10.31386 | 9.398612 | 10.91037 | 9.280739 | 10.46151 |
| AABR07053 | 0 | 0 | 0.183719 | 0.47307 | 0 | 0.300095 | 0 | 0 |
| Fam174a | 39.63549 | 37.64375 | 39.22064 | 35.73394 | 37.82546 | 36.53461 | 36.86226 | 37.64364 |
| Kyat3 | 19.41184 | 19.96689 | 17.79212 | 15.20062 | 15.80453 | 15.94393 | 14.16333 | 15.53951 |
| Mir770 | 0 | 0 | 0 | 0 | 0 | 0 | 0 | 0 |
| Tex38 | 0 | 0.177041 | 0.055994 | 0.048061 | 0.056397 | 0.091463 | 0 | 0 |
| AC094126. | 3.378447 | 0.955638 | 3.626978 | 1.556553 | 1.826533 | 0 | 4.72457 | 0.748621 |
| AABR07059 | 0.267173 | 0.151147 | 0.071707 | 0.123095 | 0.144445 | 0.351386 | 0.074725 | 0.236809 |
| AC120712. | 0.829794 | 1.207122 | 0.318156 | 0.983086 | 0.737022 | 0.545673 | 0.232084 | 1.602312 |
| Pmepa1 | 10.68176 | 11.40653 | 13.53714 | 22.10267 | 21.35731 | 21.83501 | 27.86042 | 29.29969 |
| Fos | 33.87802 | 34.63993 | 34.17668 | 38.59643 | 38.37788 | 41.32015 | 43.65277 | 43.28906 |
| Usp12 | 5.998725 | 5.730241 | 7.179033 | 5.255741 | 5.104001 | 5.690809 | 6.491071 | 6.319348 |
| Mvp | 19.21319 | 18.86826 | 19.63198 | 26.28049 | 26.07656 | 25.73161 | 23.75916 | 22.60086 |
| MGC108823 | 1.971114 | 1.932859 | 1.199133 | 1.967662 | 2.02477 | 3.71579 | 2.572727 | 1.630623 |
| Tox2 | 0.233083 | 0.23442 | 0.444852 | 0.190913 | 0.196023 | 0.09083 | 0.115894 | 0.229548 |
| Fam19a2 | 0.202341 | 0.343409 | 0.260672 | 0.410189 | 0.437577 | 0.479015 | 0.407467 | 0.161411 |
| Uqcc1 | 66.73198 | 67.82903 | 66.30682 | 66.149 | 65.35882 | 65.48118 | 61.36405 | 60.58397 |
| AABR07054 | 0.657313 | 0 | 0.470445 | 0.807584 | 0.710743 | 0.960554 | 0.980497 | 1.553624 |
| Sox11 | 0.052105 | 0.058955 | 0 | 0.024007 | 0.056341 | 0.022843 | 0 | 0.046184 |
| Klhl8 | 4.449021 | 4.005665 | 4.207216 | 4.326384 | 3.950884 | 3.867706 | 4.172516 | 4.581042 |
| Ctla4 | 0.083853 | 0 | 0.045011 | 0.2318 | 0.181337 | 0.147044 | 0.093811 | 0.297291 |
| Rn60_19_( | 0.054833 | 0 | 0 | 0 | 0 | 0 | 0.12269 | 0.145805 |
| Churc1 | 130.3033 | 125.5437 | 130.3402 | 108.8001 | 115.5292 | 102.2597 | 104.731 | 115.1987 |
| Best1 | 39.19591 | 40.62302 | 36.5212 | 28.88113 | 29.11009 | 24.22726 | 32.77409 | 31.83329 |
| Rn60_10_( | 0 | 0 | 0.077489 | 0.033255 | 0 | 0.031643 | 0 | 0.031988 |
| AABR07008 | 0.172825 | 0 | 0.185538 | 0.159251 | 0.560619 | 0 | 0.386697 | 0.45955 |
| AABR07061 | 0 | 0 | 0 | 0 | 0 | 0 | 0 | 0.100464 |
| Brwd3 | 1.230885 | 0.921148 | 1.123739 | 0.785911 | 0.848866 | 0.994261 | 0.889125 | 1.056633 |
| Stmn4 | 1.148375 | 1.559199 | 1.35614 | 1.481456 | 1.531457 | 1.476782 | 1.199101 | 1.832152 |
| Klhl5 | 3.178762 | 2.340384 | 2.808459 | 3.78401 | 3.93052 | 3.773976 | 3.913585 | 3.087111 |
| AABR07001 | 0.167036 | 0 | 0 | 0.115438 | 0.135461 | 0.146458 | 0 | 0 |
| Chrna4 | 0.04663 | 0.05276 | 0.033374 | 0.042968 | 0.050421 | 0.054514 | 0.052168 | 0.123992 |
| Ccl11 | 0.258981 | 0.410234 | 0.222426 | 0.190913 | 0.168019 | 0.09083 | 0.057947 | 0.321367 |
| Duxbl1 | 0.635034 | 0.525066 | 0.393317 | 0.585159 | 0.792293 | 0.64246 | 0.901723 | 0.432971 |
| Gsta3 | 0.057863 | 0 | 0.06212 | 0.053318 | 0 | 0.101468 | 0 | 0 |
| Olr201 | 0 | 0 | 0 | 0 | 0 | 0 | 0 | 0.049116 |
| Fam184a | 0.195059 | 0.305585 | 0.257734 | 0.207392 | 0.324484 | 0.289432 | 0.23501 | 0.332482 |
| AABR07057 | 0.494018 | 0 | 0 | 0 | 0 | 0 | 0 | 0 |
| Zar1 | 0 | 0 | 0 | 0 | 0 | 0.071741 | 0 | 0 |
| Mapk1 | 39.47913 | 38.94925 | 40.01388 | 38.41225 | 33.60398 | 39.07881 | 41.4593 | 40.79808 |
| AABR07018 | 0 | 0 | 0 | 0 | 0 | 0 | 0 | 0 |
| Dcst1 | 0.073367 | 0.14527 | 0.118147 | 0.067605 | 0.198328 | 0.273396 | 0.14364 | 0.113801 |
| Utp6 | 8.054043 | 7.290216 | 7.011549 | 8.689882 | 6.998644 | 8.165999 | 8.584554 | 9.345205 |
| Slc35e1 | 7.918749 | 9.874929 | 9.415399 | 8.081419 | 6.951219 | 7.46577 | 11.05012 | 10.07535 |
| Fgd6 | 3.044089 | 3.165271 | 2.982963 | 2.446736 | 2.348163 | 2.960079 | 4.073979 | 3.084775 |
| Ccdc39 | 1.410429 | 1.510949 | 1.643052 | 1.880351 | 2.044251 | 2.368082 | 2.232596 | 2.034791 |
| AABR07062 | 2.523659 | 0 | 0 | 0.290682 | 0.682199 | 0 | 1.058759 | 0.279606 |
| Fam205a | 0.224974 | 0.059893 | 0.15628 | 0.256082 | 0.357736 | 0.290084 | 0.207275 | 0.42227 |
| AABR07058 | 0 | 0 | 0 | 0.419592 | 0 | 0 | 0 | 0 |
| E4f1 | 4.448515 | 4.794638 | 5.166883 | 5.070908 | 5.058914 | 4.959639 | 6.800197 | 6.084364 |
| Mapkapk2 | 141.1765 | 140.7119 | 140.7012 | 109.4005 | 103.9873 | 108.9333 | 130.5515 | 129.4866 |
| Zfp385d | 1.420195 | 1.333372 | 1.55711 | 2.450246 | 2.352464 | 2.225515 | 2.231153 | 2.678276 |
| AABR07027 | 0 | 0 | 0 | 0 | 0.577781 | 0 | 0 | 0 |

| | | | | | | | | |
|---|---|---|---|---|---|---|---|---|
| Tmem160 | 24.48858 | 26.40549 | 26.49598 | 28.98723 | 26.75569 | 25.50805 | 27.5398 | 27.03263 |
| Tmem206 | 4.733218 | 4.080315 | 4.758783 | 5.192231 | 5.036723 | 5.237018 | 5.925709 | 6.12645 |
| Hacl1 | 2.075944 | 2.236984 | 1.804152 | 2.42632 | 2.516772 | 2.300846 | 1.91026 | 1.951529 |
| AABR07017 | 0 | 0 | 0.167316 | 0 | 0 | 0 | 0 | 0 |
| Btnl3 | 0 | 0 | 0 | 0.030852 | 0 | 0.029357 | 0 | 0 |
| AABR07015 | 0 | 0.569707 | 0 | 0 | 0 | 0 | 0 | 0 |
| Olr648 | 0.167839 | 0 | 0.060062 | 0 | 0.060494 | 0.049054 | 0 | 0.198353 |
| Mgat4e | 0 | 0 | 0.023811 | 0 | 0 | 0.038894 | 0 | 0.039318 |
| Olr1572 | 0 | 0 | 0.179039 | 0 | 0 | 0 | 0 | 0.049272 |
| AC114389. | 0 | 0 | 0 | 0 | 0 | 0 | 0.061668 | 0.048857 |
| Sult4a1 | 0 | 0 | 0 | 0.021736 | 0 | 0.020682 | 0 | 0.062722 |
| Mpg | 2.555611 | 3.055227 | 2.536547 | 3.021375 | 2.919761 | 3.128611 | 3.074886 | 2.607077 |
| Mirlet7i | 2.909218 | 1.974986 | 1.249292 | 3.753022 | 2.516556 | 4.081288 | 2.603763 | 1.547151 |
| AC131360. | 1.303481 | 1.025967 | 0.821369 | 1.122773 | 0.949837 | 1.416194 | 1.363171 | 1.15534 |
| Ncbp3 | 5.200227 | 5.060901 | 4.762928 | 5.344705 | 4.993791 | 5.787138 | 5.410945 | 5.431143 |
| Gcsam | 0.55664 | 0.787265 | 0.672287 | 0.423161 | 0.647028 | 0.610078 | 0.700587 | 0.77707 |
| LOC102546 | 64.37742 | 78.4431 | 82.9133 | 47.47642 | 51.49573 | 57.27763 | 40.7853 | 38.6632 |
| AABR07031 | 4.259136 | 4.992357 | 5.296152 | 3.162289 | 3.445723 | 3.116489 | 3.153768 | 3.666448 |
| Kdm3a | 6.4521 | 6.001514 | 5.863085 | 7.593133 | 7.642863 | 7.723041 | 7.150736 | 7.914243 |
| Inafm1 | 21.48979 | 26.55052 | 28.37426 | 26.46769 | 26.02194 | 27.22692 | 31.70046 | 30.6511 |
| AABR07049 | 0.122065 | 0 | 0.087363 | 0.037493 | 0 | 0 | 0 | 0 |
| Akap14 | 0.084826 | 0.063984 | 0.060711 | 0.078164 | 0.061147 | 0.049584 | 0 | 0.300742 |
| Nat8f3 | 2.896514 | 1.724878 | 2.291279 | 4.42496 | 3.791318 | 3.675832 | 6.566258 | 8.174901 |
| Slc25a1 | 2.656464 | 2.556541 | 2.655202 | 3.404449 | 4.160021 | 3.801666 | 3.518496 | 3.301791 |
| Herc4 | 6.351109 | 6.521471 | 5.953201 | 6.695532 | 7.251344 | 6.802146 | 6.738612 | 7.384678 |
| Tbc1d7 | 6.862006 | 3.339837 | 5.020949 | 2.96725 | 2.19692 | 2.554539 | 2.744816 | 3.024079 |
| Nfe2l3 | 0.026937 | 0.060956 | 0.028919 | 0.049643 | 0.029127 | 0.047237 | 0.030136 | 0.095503 |
| Fam217b | 4.622622 | 4.685038 | 4.771071 | 3.815517 | 3.956242 | 4.006172 | 3.753891 | 3.859967 |
| AC126537. | 0 | 0.429345 | 0 | 0 | 0 | 0 | 0 | 0.336337 |
| AABR07027 | 0 | 0 | 0 | 0 | 1.038945 | 0 | 0 | 0 |
| Ston1 | 4.381945 | 3.444083 | 4.417013 | 4.500133 | 4.249853 | 4.340686 | 6.193396 | 5.944498 |
| Wdr62 | 0.195868 | 0.10429 | 0.037108 | 0.148635 | 0.13704 | 0.161635 | 0.206239 | 0.306366 |
| AABR07010 | 0 | 0.108815 | 0 | 0 | 0 | 0 | 0 | 0 |
| Olr1737 | 0 | 0.063915 | 0 | 0 | 0 | 0 | 0 | 0 |
| LOC102551 | 9.90253 | 9.495124 | 7.748016 | 10.36205 | 8.529673 | 8.0939 | 11.45405 | 12.94251 |
| Ccdc148 | 0.090286 | 0 | 0.032309 | 0.138658 | 0.065083 | 0 | 0.033669 | 0 |
| AABR07028 | 0 | 0 | 0 | 0.132929 | 0 | 0 | 0 | 0 |
| Evc2 | 0.864167 | 1.006951 | 1.0939 | 1.556936 | 1.185447 | 1.300533 | 1.471832 | 1.577636 |
| Fam71f1 | 0.053299 | 0.060305 | 0 | 0.122782 | 0.115262 | 0.070098 | 0 | 0.070862 |
| Gpr88 | 0.352887 | 0.285197 | 0.446498 | 0.661956 | 0.422454 | 0.563571 | 0.549892 | 0.860149 |
| AABR07036 | 0.39974 | 0 | 0.858293 | 0 | 0 | 0 | 0 | 0 |
| Kdm3b | 8.26958 | 7.938968 | 7.949342 | 7.925492 | 7.482073 | 7.754005 | 8.772326 | 9.565202 |
| 5S_rRNA | 0 | 0 | 0 | 0.408925 | 0 | 0 | 0.49648 | 0 |
| Tlr13 | 2.519323 | 2.51115 | 2.39877 | 3.344003 | 2.740322 | 3.103041 | 2.415863 | 3.17671 |
| Tmem198b | 1.668084 | 1.415519 | 1.748157 | 1.518775 | 1.76073 | 1.828222 | 1.599581 | 2.041746 |
| Rn60_14_0 | 0 | 0 | 0 | 0 | 0 | 0 | 0.215385 | 0 |
| Mir150 | 0 | 0 | 0 | 0 | 0.666147 | 0 | 0 | 0 |
| Flad1 | 15.66032 | 16.7449 | 18.14948 | 14.27847 | 15.01219 | 14.5982 | 15.28652 | 16.85144 |
| Prmt7 | 7.67258 | 8.579926 | 7.612626 | 7.544061 | 7.449694 | 7.198042 | 6.43155 | 7.375566 |
| Cox7a1 | 2.154976 | 4.876508 | 5.783761 | 5.560032 | 3.728231 | 5.668455 | 4.580694 | 5.348175 |
| Rpl35a | 99.1642 | 109.816 | 112.7594 | 27.17705 | 131.4684 | 105.1547 | 89.22377 | 41.61302 |
| Rn50_X_07 | 0 | 0 | 0 | 0 | 0 | 0 | 0.074725 | 0 |
| AABR07039 | 1.497188 | 1.451998 | 0.995012 | 1.346752 | 0.809444 | 1.594036 | 1.316061 | 1.327032 |
| Ank2 | 13.65883 | 13.51853 | 13.96536 | 9.165946 | 9.387396 | 9.353984 | 11.36576 | 10.48353 |

| | | | | | | | | |
|---|---|---|---|---|---|---|---|---|
| Edaradd | 0.459987 | 0.383492 | 0.545807 | 0.446169 | 0.183244 | 0.127363 | 0.270849 | 0.429168 |
| Fam118b | 3.928345 | 5.301063 | 4.062565 | 4.184374 | 4.169724 | 4.803167 | 4.03198 | 4.599925 |
| Sppl2b | 7.876251 | 7.839692 | 9.39296 | 9.397294 | 9.520777 | 8.160042 | 11.5964 | 10.55399 |
| RT1-M2 | 0.092847 | 0 | 0.099678 | 0 | 0.301184 | 0.203522 | 0.467431 | 0.205738 |
| Eef1d | 27.8056 | 28.11197 | 28.95918 | 31.36334 | 30.68339 | 28.09047 | 27.97077 | 28.83361 |
| Ccl20 | 0.066539 | 0.451712 | 0.2143 | 1.164942 | 1.223104 | 0.583412 | 0.893286 | 0.943624 |
| AABR07071 | 2.327374 | 0.658329 | 0.624646 | 2.144584 | 0.629139 | 3.060966 | 0.650941 | 1.547151 |
| Zfp418 | 0.439668 | 0.377386 | 0.504564 | 0.335285 | 0.508193 | 0.46526 | 0.339228 | 0.443451 |
| Gck | 0.698503 | 0.963207 | 0.726454 | 0.603416 | 0.731679 | 0.842116 | 0.56167 | 0.638466 |
| Ccser1 | 0.317754 | 0.413453 | 0.187621 | 0.263518 | 0.292046 | 0.306468 | 0.444362 | 0.098575 |
| Kmt5b | 7.019353 | 6.435311 | 6.032269 | 6.26222 | 6.698086 | 6.320304 | 7.497299 | 7.935017 |
| Ccr1 | 0.936119 | 0.641924 | 1.004983 | 1.385383 | 1.595 | 1.218749 | 3.554433 | 2.916622 |
| Map3k10 | 7.466032 | 8.464225 | 10.33714 | 7.070756 | 6.791498 | 7.195651 | 9.545903 | 9.401074 |
| AABR07004 | 0 | 0.068497 | 0 | 0 | 0 | 0 | 0 | 0 |
| Tfap2a | 0.031689 | 0.053782 | 0.05103 | 0.0584 | 0.06853 | 0.069462 | 0.106356 | 0 |
| RGD156532 | 0.588381 | 0.748941 | 0.39479 | 0.54217 | 0.477156 | 0.38692 | 0.658255 | 0.195567 |
| AC133405. | 0.978802 | 0 | 0.525403 | 0 | 1.058365 | 0 | 3.285121 | 1.735122 |
| Uba1 | 42.69814 | 44.577 | 44.74666 | 44.24767 | 44.01528 | 44.11717 | 43.65387 | 47.29075 |
| Cntnap3b | 0 | 0 | 0 | 0 | 0.019847 | 0 | 0 | 0 |
| Dcm5 | 0 | 0 | 0 | 0 | 0 | 0 | 0 | 0.061072 |
| Cxcl12 | 23.67716 | 23.04211 | 21.4589 | 20.90109 | 20.32898 | 19.97276 | 25.2034 | 25.2143 |
| Gm8225 | 0.90175 | 1.020288 | 1.382981 | 0.949631 | 0.76611 | 1.185983 | 0.936778 | 0.685085 |
| Defb36 | 0 | 0.244833 | 0 | 0 | 0 | 0 | 0 | 0.383591 |
| Limk1 | 2.958978 | 3.058393 | 3.554416 | 2.711844 | 2.386654 | 3.029178 | 4.34822 | 4.451484 |
| Hes1 | 17.80586 | 19.81824 | 18.06456 | 21.5201 | 19.37086 | 18.69648 | 19.35241 | 18.45009 |
| Gpbp1 | 15.97837 | 14.50738 | 13.89142 | 16.15062 | 15.75085 | 15.14439 | 14.71745 | 16.73425 |
| Kif23 | 0.523659 | 0.533246 | 0.468485 | 0.562953 | 0.415232 | 0.352011 | 0.66396 | 0.587917 |
| Tmf1 | 16.73003 | 15.28821 | 15.05563 | 16.9998 | 16.66647 | 17.45841 | 19.16684 | 17.56751 |
| Opa1 | 35.06724 | 36.1129 | 34.78837 | 33.82049 | 33.87189 | 33.17318 | 32.00837 | 34.81474 |
| Pltp | 33.35903 | 33.17976 | 33.84195 | 35.64775 | 33.80574 | 34.37351 | 47.41742 | 47.88145 |
| Xpnpep2 | 1.549911 | 1.923909 | 1.857784 | 1.705499 | 1.480646 | 1.583258 | 1.868649 | 2.294051 |
| AABR07064 | 0 | 0.558958 | 0 | 0 | 0 | 0 | 0.552686 | 0 |
| AABR07050 | 0.113592 | 0.128524 | 0.365845 | 0.104671 | 0 | 0 | 0.127082 | 0.100682 |
| Mettl22 | 10.27384 | 11.66774 | 13.00508 | 12.43419 | 11.60637 | 11.73071 | 10.63616 | 11.04299 |
| AABR07060 | 0.285719 | 0.233478 | 0.255615 | 0.336412 | 0.257453 | 0.375778 | 0.337408 | 0.295455 |
| AABR07058 | 0.040831 | 0.034649 | 0.021917 | 0.028218 | 0.022075 | 0.026851 | 0.01142 | 0.009048 |
| Dennd1b | 9.284738 | 8.060387 | 7.937961 | 7.839968 | 7.666491 | 7.075153 | 8.045476 | 8.738259 |
| Trappc6b | 25.96182 | 26.72236 | 26.11486 | 23.80053 | 23.38548 | 23.50015 | 25.53183 | 27.40181 |
| Rn60_4_16 | 0 | 0 | 0 | 0 | 0 | 0 | 0.066123 | 0 |
| AABR07064 | 0 | 0 | 0.084348 | 0 | 0 | 0 | 0 | 0 |
| Arhgap19 | 0.58255 | 0.623175 | 0.57992 | 0.595356 | 0.595544 | 0.928691 | 0.67543 | 0.572671 |
| RT1-Db1 | 33.62685 | 32.25882 | 31.59572 | 42.10894 | 40.58672 | 41.47928 | 47.33075 | 45.44649 |
| Actrt3 | 0.042539 | 0 | 0 | 0 | 0 | 0.037299 | 0 | 0 |
| Src | 4.699244 | 4.137992 | 5.176546 | 6.589386 | 5.589347 | 7.004512 | 7.885958 | 6.881591 |
| AABR07012 | 0 | 0 | 0 | 0.160844 | 0.377483 | 0 | 0 | 0 |
| Tenm4 | 0.036782 | 0.020809 | 0.052651 | 0.062138 | 0.059658 | 0.064502 | 0.109735 | 0.070638 |
| Oip5 | 1.768599 | 1.044045 | 1.155733 | 1.204557 | 1.579776 | 1.146177 | 0.774247 | 1.158659 |
| Mir29b1 | 0 | 0 | 0 | 0 | 0 | 0 | 0.723267 | 0 |
| Mpl | 0.24818 | 0.436806 | 0.266437 | 0.254098 | 0.477072 | 0.217604 | 0.246802 | 0.219974 |
| AABR07059 | 0 | 0 | 0 | 0 | 0 | 0 | 0 | 0 |
| AABR07047 | 0 | 0 | 0 | 0 | 0 | 0 | 0.509432 | 0 |
| Mir125a | 0 | 0 | 0 | 0 | 0 | 0.54017 | 0.689231 | 0 |
| RGD130675 | 5.747479 | 4.696613 | 5.827492 | 2.844189 | 2.87716 | 3.07963 | 2.38149 | 1.792431 |
| Krtcap3 | 0.054378 | 0.61526 | 0.233513 | 0.300643 | 0.58798 | 0.381429 | 0.365013 | 0.192791 |

| | | | | | | | | |
|---|---|---|---|---|---|---|---|---|
| Cntf | 1.100929 | 1.353967 | 1.644407 | 1.764283 | 1.293933 | 1.762713 | 1.927832 | 2.078895 |
| Purg | 1.513674 | 2.161203 | 1.77979 | 1.92614 | 1.28599 | 1.358791 | 1.854711 | 1.916635 |
| Spink11 | 0.138169 | 0.312663 | 0.296666 | 0.254634 | 0 | 0.363439 | 0 | 0.122466 |
| Mt1m | 0.280032 | 0.158421 | 0.450948 | 1.290191 | 0.151397 | 0.61383 | 0.313287 | 0.372309 |
| AABR07052 | 0 | 0 | 0.05968 | 0 | 0 | 0 | 0 | 0 |
| Sos1 | 5.213649 | 4.977 | 4.832774 | 4.148064 | 3.712671 | 4.134315 | 4.655618 | 4.859535 |
| Spem1 | 0.041039 | 0 | 0 | 0 | 0.133125 | 0.071966 | 0.045913 | 0.109125 |
| Cited4 | 17.25962 | 21.18903 | 21.61752 | 26.39858 | 21.96344 | 23.57493 | 20.55718 | 17.69163 |
| AABR07049 | 5.072482 | 6.920891 | 5.445634 | 7.286086 | 7.581932 | 4.970799 | 7.510855 | 6.082814 |
| AABR07058 | 0.219564 | 0.248426 | 0.235716 | 0 | 0 | 0.481284 | 0 | 0.38922 |
| Col3a1 | 150.2841 | 155.0312 | 157.0451 | 313.8125 | 300.8539 | 289.9406 | 345.0286 | 349.117 |
| Col8a2 | 0.773758 | 0.833109 | 0.763688 | 5.243906 | 4.884974 | 5.821379 | 6.841393 | 6.493165 |
| Igf2bp3 | 1.471642 | 1.341325 | 1.579901 | 0.301347 | 0.48622 | 0.609326 | 0.137201 | 0.36233 |
| Gtf3c4 | 3.172079 | 3.201749 | 3.05427 | 2.747709 | 2.952859 | 2.774612 | 3.54027 | 3.674595 |
| AABR07009 | 0 | 0.074341 | 0 | 0.060543 | 0 | 0.057609 | 0.220519 | 0 |
| LOC681006 | 0 | 0 | 0 | 0 | 0 | 0 | 0 | 0 |
| Rtkn | 0 | 0 | 0 | 0.027448 | 0.064417 | 0 | 0.099974 | 0.052804 |
| Mfsd12 | 3.002961 | 2.815245 | 4.083569 | 4.717264 | 4.39126 | 4.965084 | 6.783151 | 5.602189 |
| Mxi1 | 18.01609 | 17.99896 | 18.42694 | 18.30833 | 18.81277 | 18.93342 | 19.86971 | 19.55488 |
| C2cd2l | 6.783579 | 7.357056 | 8.383877 | 7.683912 | 7.495998 | 7.978821 | 10.18059 | 10.69074 |
| AABR07014 | 0.104315 | 0.059014 | 0.223977 | 0.048061 | 0.394778 | 0.091463 | 0.058351 | 0.138689 |
| AY172581. | 41.74095 | 37.78234 | 38.29353 | 32.86808 | 40.21019 | 29.2788 | 28.86781 | 24.21627 |
| Slc24a3 | 4.537717 | 3.485269 | 4.515943 | 2.594255 | 2.292119 | 2.526604 | 4.594876 | 4.433012 |
| Aldh3a2 | 13.77328 | 13.45269 | 12.91185 | 14.91526 | 16.05966 | 16.06749 | 16.17718 | 16.06857 |
| Trhr | 0 | 0 | 0 | 0 | 0 | 0 | 0 | 0 |
| AABR07071 | 0 | 0 | 1.499151 | 0 | 0.754967 | 1.836579 | 0.781129 | 0.61886 |
| Lrriq1 | 0 | 0.007444 | 0 | 0 | 0 | 0 | 0 | 0 |
| Trbv19 | 0 | 0 | 0.764873 | 0.164126 | 0.192594 | 0.312343 | 0.398535 | 0 |
| Ank3 | 11.38531 | 11.41287 | 11.04674 | 9.152617 | 9.476571 | 9.007863 | 9.932066 | 10.14891 |
| Sgpp1 | 6.688599 | 6.995478 | 5.430735 | 6.922901 | 7.333578 | 6.768074 | 7.21042 | 7.589064 |
| LOC691485 | 35.26072 | 33.84073 | 38.12311 | 31.15484 | 30.60969 | 34.07407 | 29.54413 | 29.25844 |
| AABR07026 | 0 | 0.034238 | 0.016243 | 0.013942 | 0.01636 | 0 | 0.033854 | 0 |
| Tgds | 5.582912 | 6.114124 | 5.352584 | 7.345261 | 7.037462 | 7.434272 | 8.082947 | 8.335561 |
| Dyrk1b | 13.7848 | 13.84336 | 17.25074 | 13.47255 | 12.52398 | 12.17592 | 13.23174 | 13.46527 |
| AABR07063 | 0 | 0 | 0 | 0 | 0 | 0 | 0 | 0 |
| Orc4 | 5.188842 | 6.267128 | 4.921225 | 5.602644 | 5.61062 | 5.721866 | 6.196797 | 5.840611 |
| Parpbp | 0.061333 | 0.083274 | 0.079014 | 0.022606 | 0.092846 | 0.032266 | 0.054893 | 0.065235 |
| AABR07064 | 0.430995 | 0 | 0 | 0 | 0 | 0 | 0 | 0.095503 |
| Zfp668 | 0.496654 | 0.737547 | 1.016392 | 0.629264 | 0.50346 | 0.530725 | 0.642452 | 0.715339 |
| AABR07049 | 0 | 0 | 0 | 0 | 0 | 0 | 0.54752 | 0 |
| Ttc16 | 0.467379 | 0.572885 | 0.522668 | 0.951066 | 0.673827 | 0.717147 | 0.784324 | 0.811262 |
| LOC100910 | 0.242935 | 0.183246 | 0.260806 | 0.248728 | 0.291869 | 0.142004 | 0.241586 | 0.2871 |
| Fam229b | 1.013031 | 0.705352 | 1.338528 | 1.148884 | 1.348155 | 0.683251 | 0.784616 | 0.621623 |
| Rab18 | 42.7956 | 46.27596 | 40.87125 | 45.61862 | 47.54339 | 49.76708 | 45.68931 | 41.02617 |
| AABR07047 | 0 | 0 | 0 | 0 | 0 | 0 | 0 | 0 |
| Usmg5 | 108.3022 | 145.046 | 132.6968 | 92.90295 | 97.80252 | 106.5872 | 103.6644 | 93.73321 |
| LOC100912 | 1.362854 | 1.568141 | 2.207065 | 3.405603 | 1.373727 | 2.855731 | 1.292119 | 2.763988 |
| Gcnt7 | 0.162375 | 0.321509 | 0.26148 | 0.037406 | 0.175574 | 0.071185 | 0.045414 | 0.143921 |
| AC096024. | 0 | 0 | 0.146401 | 0 | 0.294909 | 0 | 0 | 0 |
| Maoa | 88.74302 | 85.3046 | 82.18161 | 330.8919 | 342.9971 | 330.8797 | 316.8578 | 320.5788 |
| Rufy1 | 12.76739 | 13.46783 | 13.60117 | 12.07233 | 11.85122 | 10.28168 | 15.38232 | 14.43272 |
| R3hcc1 | 14.04653 | 13.7983 | 14.9852 | 15.73236 | 14.61636 | 15.48519 | 14.62973 | 15.15895 |
| Tprg1l | 41.00038 | 38.57874 | 41.45968 | 37.44688 | 37.84614 | 38.35459 | 45.66703 | 42.86061 |
| Npy4r | 0 | 0 | 0 | 0 | 0 | 0.073054 | 0 | 0 |

| | | | | | | | | |
|---|---|---|---|---|---|---|---|---|
| Traf4 | 2.24724 | 3.13396 | 3.141933 | 2.311527 | 2.26038 | 3.047219 | 3.215725 | 3.381497 |
| Cfap53 | 0.966442 | 0.920829 | 1.392485 | 0.796798 | 0.6325 | 0.936575 | 0.995854 | 1.014402 |
| Rn60_20_( | 0.13479 | 0 | 0 | 0 | 0 | 0 | 0.075399 | 0 |
| Slc3a2 | 26.56538 | 27.3748 | 29.86077 | 33.1476 | 32.03279 | 33.32936 | 38.24831 | 36.58476 |
| Tsc22d4 | 1.54632 | 1.276727 | 1.166538 | 0.558396 | 0.655249 | 0.513011 | 0.794844 | 0.759376 |
| Tmem86b | 0.700287 | 0.44019 | 0.668269 | 0.358493 | 0.252403 | 0.341118 | 0.8705 | 0.655183 |
| Gpr37l1 | 0.751151 | 0.849891 | 0.806408 | 1.24588 | 0.951444 | 0.978505 | 0.792334 | 0.627737 |
| Bag4 | 2.138944 | 2.765845 | 2.952376 | 2.815646 | 2.354109 | 2.478244 | 3.247582 | 2.70836 |
| Tcirg1 | 6.646135 | 7.110858 | 7.630839 | 8.751431 | 8.380479 | 9.59486 | 9.928843 | 10.21547 |
| Tacr2 | 0 | 0 | 0.018871 | 0 | 0 | 0 | 0 | 0 |
| Odf4 | 0 | 0.041491 | 0.039368 | 0.033791 | 0 | 0.096459 | 0 | 0.065006 |
| Oxsr1 | 13.13488 | 13.44736 | 12.46117 | 13.67083 | 13.73956 | 14.36794 | 15.85994 | 15.77581 |
| AABR0700( | 1.146699 | 1.729915 | 1.641406 | 2.113276 | 0.826606 | 2.010853 | 0.855251 | 1.016376 |
| AC133316. | 0.436383 | 0 | 0 | 0 | 0 | 0 | 0 | 0 |
| Zfp78 | 1.194126 | 0.755025 | 0.90492 | 1.294517 | 1.177262 | 1.016216 | 1.139474 | 1.151802 |
| LOC10035( | 0 | 0 | 0 | 0.076592 | 0 | 0 | 0 | 0 |
| Spata21 | 0.020528 | 0.139356 | 0.088151 | 0.037831 | 0 | 0.053996 | 0.045931 | 0.072779 |
| Rn50_X_07 | 0.929401 | 1.413051 | 1.309574 | 2.248066 | 1.790063 | 1.986317 | 2.014559 | 2.703009 |
| AABR0702( | 0 | 0 | 0 | 0 | 0 | 0 | 0.723267 | 0 |
| Jph4 | 0 | 0 | 0 | 0 | 0 | 0.024293 | 0 | 0 |
| Faim | 0 | 0.197938 | 0.125207 | 0.107468 | 0 | 0.153389 | 0.065239 | 0.15506 |
| Taok2 | 29.05329 | 30.92384 | 30.45401 | 27.39075 | 26.93354 | 27.94154 | 32.9108 | 34.12832 |
| Rn50_10_( | 0.097985 | 0.095027 | 0.075138 | 0.064492 | 0.10595 | 0.085913 | 0.01566 | 0.12407 |
| Dram1 | 1.489621 | 1.728653 | 1.722219 | 2.270115 | 2.64321 | 2.361029 | 2.585246 | 2.454451 |
| Slc46a3 | 5.25614 | 5.357282 | 4.267077 | 5.043463 | 4.790955 | 5.141813 | 4.4467 | 4.293006 |
| Cby1 | 30.06817 | 28.28689 | 23.21266 | 27.29156 | 27.51976 | 31.30084 | 24.56776 | 25.55294 |
| Gspt1 | 2.130287 | 1.721656 | 3.897232 | 4.386649 | 1.621815 | 1.829718 | 7.538915 | 2.331323 |
| Triqk | 0.957705 | 1.431897 | 0.917997 | 0.945522 | 1.072536 | 1.259574 | 1.377562 | 1.212659 |
| Pcbp4 | 22.45364 | 21.4494 | 25.55118 | 24.22202 | 20.77839 | 23.25244 | 27.3511 | 28.14253 |
| LOC10036( | 3.42553 | 2.932146 | 2.910041 | 3.074147 | 3.897226 | 3.343034 | 2.732618 | 3.77547 |
| Rad54b | 0.031853 | 0.03604 | 0.08549 | 0.044027 | 0.051663 | 0.083786 | 0.017818 | 0.098814 |
| AABR0702( | 0 | 0 | 0.043079 | 0.036976 | 0 | 0.035184 | 0 | 0 |
| Vars | 30.74852 | 30.9943 | 36.74926 | 28.52923 | 27.53057 | 29.82766 | 34.23378 | 33.93568 |
| Txndc5 | 28.72131 | 28.94636 | 30.94145 | 32.32223 | 31.59623 | 32.84112 | 35.70981 | 35.43093 |
| Mrpl15 | 68.86732 | 66.48231 | 67.27028 | 63.04757 | 64.69996 | 61.45828 | 58.58467 | 62.55583 |
| Pfkm | 219.4391 | 230.3553 | 233.95 | 162.4954 | 154.171 | 163.5662 | 177.327 | 180.0162 |
| Olr1 | 0.194153 | 0.28244 | 0.119106 | 0.421704 | 0.524838 | 0.316148 | 0.667145 | 0.651475 |
| Acpt | 0.645994 | 0.45682 | 0.346758 | 0.483647 | 0.261939 | 0.708011 | 0.542032 | 0.465219 |
| Zcchc9 | 9.547738 | 8.959332 | 7.661342 | 9.638616 | 11.099 | 10.85719 | 11.84817 | 12.24627 |
| Kat7 | 15.71223 | 15.361 | 15.73476 | 16.15224 | 15.98066 | 14.09938 | 15.16115 | 16.21135 |
| Rab21 | 32.87436 | 33.08068 | 30.17509 | 29.73929 | 35.16476 | 29.75308 | 35.43523 | 37.87024 |
| Rgs13 | 0.199717 | 0.203374 | 0.064323 | 0.110419 | 0.021595 | 0.035022 | 0 | 0 |
| Mgat3 | 0.585967 | 0.596695 | 0.733919 | 0.773922 | 0.844797 | 0.890546 | 0.786665 | 0.692496 |
| Rnaseh2b | 7.26485 | 7.176709 | 7.245017 | 6.320481 | 7.735752 | 8.148204 | 7.673766 | 6.210393 |
| Ppp1r27 | 0.19911 | 0 | 0 | 0 | 0.358825 | 0.058193 | 0 | 0 |
| Fam114a1 | 7.467675 | 5.806737 | 5.806571 | 9.344797 | 10.99886 | 9.188286 | 12.34266 | 10.29618 |
| AABR0705( | 0 | 0 | 0 | 0.236535 | 0 | 0 | 0 | 0 |
| AC111292. | 0 | 0 | 0 | 0 | 0 | 0.020388 | 0 | 0.02061 |
| Dmtn | 1.618098 | 1.529021 | 2.118919 | 1.605707 | 1.422773 | 1.574656 | 2.168329 | 2.174942 |
| Rn50_19_( | 0 | 0 | 0.154445 | 0 | 0 | 0 | 0 | 0 |
| RGD13110( | 0.277435 | 0.196191 | 0.409536 | 0.67107 | 0.637472 | 0.729767 | 0.853551 | 1.198786 |
| Adprhl1 | 84.88862 | 88.70312 | 89.68909 | 90.46301 | 84.84285 | 89.97116 | 86.87989 | 91.12985 |
| Adck2 | 4.899735 | 4.504354 | 4.79052 | 3.890081 | 3.500473 | 3.452217 | 5.21242 | 4.110225 |
| Btbd11 | 2.074688 | 1.873415 | 1.86323 | 1.378661 | 2.006055 | 1.924036 | 2.343387 | 1.838899 |

| | | | | | | | | |
|---|---|---|---|---|---|---|---|---|
| Armc10 | 6.84096 | 7.463788 | 5.945311 | 7.004084 | 7.367678 | 7.878535 | 7.683733 | 8.253074 |
| Dact3 | 1.86821 | 2.578826 | 2.888125 | 5.388238 | 4.363347 | 4.848622 | 6.019402 | 5.199486 |
| Cnnm3 | 7.548241 | 7.530625 | 8.719496 | 7.942323 | 8.299672 | 8.485292 | 10.59859 | 9.413999 |
| RGD130715 | 3.847058 | 5.327264 | 5.732773 | 5.343823 | 4.718543 | 4.58138 | 5.460194 | 4.987525 |
| Klrg1 | 0.075292 | 0.383355 | 0.28291 | 0.208137 | 0.122119 | 0.264066 | 0.084234 | 0.166839 |
| S100z | 0 | 0 | 0 | 0 | 0 | 0 | 0 | 0 |
| AABR0706( | 0 | 0 | 0 | 0 | 0 | 0 | 0 | 0 |
| Mbd3 | 39.95694 | 41.15334 | 44.64296 | 36.51383 | 37.42035 | 36.36408 | 45.99776 | 46.26787 |
| AC128836. | 1.66708 | 1.55336 | 1.158052 | 3.614467 | 2.862936 | 2.923394 | 2.084473 | 1.912209 |
| Trmu | 5.177235 | 5.447379 | 4.885457 | 5.591043 | 5.134535 | 6.216382 | 5.681384 | 5.407233 |
| Tmem72 | 0.026956 | 0.020333 | 0.009646 | 0.016559 | 0.009716 | 0.023635 | 0.020105 | 0.015928 |
| Cnot9 | 4.034162 | 4.652923 | 3.84412 | 4.29365 | 4.378988 | 4.373461 | 4.653186 | 4.559682 |
| AABR07072 | 3.173692 | 3.590883 | 1.703581 | 2.193324 | 3.431667 | 3.826207 | 1.775293 | 3.867876 |
| Ccdc120 | 1.012503 | 0.924518 | 1.296755 | 1.45676 | 0.979562 | 1.168109 | 1.470578 | 1.259553 |
| Sft2d1 | 22.64193 | 20.93914 | 20.81126 | 23.72168 | 23.08499 | 21.7108 | 24.23196 | 23.41344 |
| Zfp169 | 0.877968 | 0.573953 | 0.754044 | 0.719123 | 0.421926 | 0.78691 | 1.375124 | 0.985703 |
| Cox4i1 | 1616.825 | 1631.511 | 1597.719 | 1418.484 | 1416.88 | 1363.365 | 1391.606 | 1356.688 |
| Zhx2 | 5.392498 | 5.271241 | 5.454442 | 4.411232 | 4.997854 | 5.564418 | 6.545887 | 6.828055 |
| LOC103693 | 0 | 0.137152 | 0 | 0.223394 | 0.393212 | 0.106284 | 0 | 0.107441 |
| Mcfd2 | 26.51439 | 25.12482 | 27.09248 | 26.39525 | 28.00408 | 27.81438 | 26.37899 | 27.82353 |
| RGD156618 | 0.474329 | 0.536681 | 0.509222 | 0.699321 | 0.718039 | 0.332714 | 0.63679 | 0.924927 |
| Nit2 | 66.06868 | 72.85162 | 67.7571 | 66.46541 | 66.4268 | 70.43496 | 55.10834 | 58.65025 |
| Foxr2 | 0 | 0 | 0 | 0 | 0 | 0 | 0 | 0.051061 |
| AABR07065 | 0.010208 | 0.01155 | 0.032876 | 0.009406 | 0 | 0.00895 | 0 | 0.036191 |
| Cdca3 | 0.737054 | 0.476538 | 0.565196 | 0.582142 | 0.645163 | 0.615476 | 0.903115 | 0.435525 |
| Acvrl1 | 15.73499 | 17.28322 | 17.83187 | 17.04097 | 15.47458 | 18.3996 | 23.90838 | 23.88422 |
| AABR07021 | 0 | 0 | 0 | 0 | 0 | 0 | 0.028453 | 0 |
| Mir2964 | 0 | 0 | 0 | 0.482531 | 0 | 0.459145 | 2.929233 | 2.784871 |
| Fam78b | 9.331876 | 9.647046 | 10.52289 | 8.846408 | 7.114111 | 7.946738 | 10.59031 | 11.72264 |
| Slf1 | 1.94103 | 2.354183 | 1.768998 | 2.123138 | 1.751523 | 1.922286 | 1.749729 | 1.943221 |
| AABR07013 | 0.258871 | 0.251058 | 0.238213 | 0.749696 | 0.439864 | 0.616084 | 0.37236 | 0.622794 |
| Hsd17b11 | 10.57831 | 10.59356 | 10.08682 | 13.53146 | 15.09697 | 14.28707 | 14.55428 | 13.36528 |
| Plcd1 | 14.65101 | 13.64143 | 14.15182 | 15.04724 | 13.67604 | 15.37173 | 15.2811 | 15.96186 |
| AABR07053 | 0.052922 | 0 | 0.028407 | 0 | 0 | 0 | 0 | 0 |
| Zfp280c | 1.331993 | 1.769952 | 1.529746 | 1.584176 | 1.842199 | 1.751839 | 1.750089 | 1.96311 |
| Rps29 | 9.851015 | 9.777158 | 12.43108 | 9.236574 | 9.530522 | 7.728181 | 7.153903 | 7.965528 |
| NMS | 0 | 0 | 0 | 0 | 0 | 0 | 0 | 0.093578 |
| LOC499235 | 5.531063 | 5.235681 | 5.101618 | 5.541717 | 6.283903 | 6.598244 | 5.804431 | 5.71723 |
| Hip1r | 15.69771 | 15.76803 | 17.0727 | 15.63224 | 14.75577 | 15.46705 | 19.53272 | 19.44276 |
| Rab27a | 8.531966 | 8.590746 | 7.689031 | 6.870121 | 5.226368 | 6.296942 | 7.202674 | 7.28479 |
| Pigw | 4.372502 | 4.633168 | 4.060821 | 3.997112 | 4.127552 | 4.594491 | 3.571762 | 3.752532 |
| Hsp90b1 | 47.03106 | 44.63574 | 44.91424 | 57.76146 | 57.4005 | 53.50022 | 70.40369 | 70.52394 |
| Dclre1a | 3.444955 | 3.395814 | 3.488244 | 3.703455 | 2.963052 | 2.906125 | 3.430699 | 3.909321 |
| AC123471. | 0.793423 | 0.528071 | 0.501053 | 0.47307 | 0.35326 | 0.531986 | 0.522145 | 0.70325 |
| LOC102550 | 7.389731 | 6.818305 | 9.822241 | 5.714987 | 4.85132 | 5.457287 | 6.594158 | 9.142549 |
| Npm1 | 112.531 | 111.6627 | 107.1917 | 112.8539 | 113.4304 | 105.1675 | 122.9223 | 117.1758 |
| Cog3 | 5.847038 | 5.721079 | 5.230974 | 6.048585 | 6.779592 | 6.206839 | 6.829392 | 7.121891 |
| Thoc6 | 5.300194 | 6.356736 | 7.852334 | 4.786242 | 4.642129 | 5.39077 | 4.802994 | 4.791782 |
| Rsrc1 | 0.260527 | 0.196516 | 0 | 0 | 0.187803 | 0.609147 | 0.097155 | 0.307891 |
| Kcnd3 | 4.138712 | 4.198335 | 4.268071 | 3.0622 | 3.130386 | 2.922725 | 3.352909 | 3.117191 |
| Cdc14a | 2.936516 | 2.885703 | 2.750621 | 2.209985 | 2.150542 | 2.051586 | 2.787876 | 2.9035 |
| Olr462 | 0 | 0 | 0 | 0 | 0 | 0.049212 | 0 | 0 |
| Agap3 | 17.18603 | 19.83947 | 21.23911 | 17.61692 | 17.14432 | 18.47134 | 21.40662 | 21.00306 |
| Trim15 | 0.543967 | 0.328252 | 0.545051 | 0.63491 | 0.627396 | 0.572341 | 0.73028 | 0.739289 |

| | | | | | | | | |
|---|---|---|---|---|---|---|---|---|
| RGD130935 | 0 | 0.549243 | 0.325714 | 0.279566 | 0.131223 | 0.585237 | 0.610964 | 0.484045 |
| Tmem9 | 5.032478 | 4.698555 | 5.55381 | 4.345376 | 4.832701 | 4.906185 | 6.220684 | 5.583467 |
| AABR07028 | 0 | 0 | 0 | 0.042892 | 0 | 0 | 0 | 0 |
| LOC679881 | 1.519054 | 1.468739 | 1.60115 | 1.501548 | 1.403617 | 1.380341 | 1.421358 | 1.224012 |
| Bpifb1 | 0 | 0 | 0 | 0 | 0.033386 | 0 | 0 | 0 |
| Tfpi2 | 1.359778 | 1.440321 | 1.428749 | 1.386278 | 1.470308 | 1.49666 | 1.683095 | 1.641176 |
| Rars2 | 3.950131 | 3.412437 | 3.266499 | 3.418545 | 3.838325 | 3.36987 | 4.120634 | 3.595824 |
| Apof | 0.087568 | 0 | 0 | 0.040345 | 0 | 0.03839 | 0 | 0.038808 |
| Cnep1r1 | 15.97369 | 15.61129 | 14.76286 | 16.767 | 13.46725 | 15.02065 | 16.93827 | 16.25124 |
| Proz | 0.20287 | 0.114769 | 0.245019 | 0.093469 | 0.10968 | 0.266815 | 0.368814 | 0.157337 |
| Taf9b | 5.049219 | 4.797988 | 3.917273 | 3.743934 | 3.945448 | 4.063994 | 4.324894 | 4.353 |
| AABR07015 | 0 | 5.197331 | 3.945134 | 3.386185 | 4.966887 | 1.611035 | 2.055602 | 0 |
| Mrps35 | 16.91714 | 17.49954 | 16.8554 | 16.2353 | 17.22964 | 16.22804 | 16.02047 | 15.6789 |
| Rn50_X_07 | 1.991731 | 1.690162 | 3.385563 | 2.752952 | 1.525488 | 2.255704 | 2.321104 | 1.838927 |
| Ccng2 | 10.65968 | 11.36556 | 10.12427 | 10.68169 | 11.09077 | 11.37178 | 11.28543 | 12.43481 |
| Ngf | 2.22217 | 2.22277 | 1.590428 | 2.374078 | 2.263508 | 2.343728 | 2.702245 | 2.62616 |
| Rpl7l1 | 32.86121 | 33.58276 | 31.71281 | 35.62006 | 30.64187 | 30.23788 | 39.92612 | 37.0815 |
| Wbscr22 | 6.057661 | 6.77138 | 6.855873 | 7.397693 | 6.826268 | 6.207254 | 7.307774 | 8.021464 |
| Slc51a | 0.032667 | 0.073923 | 0.105212 | 0.090305 | 0.105969 | 0.085929 | 0.073094 | 0.144774 |
| Clcnkb | 2.882249 | 1.785039 | 2.182281 | 2.124704 | 2.197977 | 2.39415 | 2.002604 | 1.989962 |
| Gjc1 | 8.015862 | 7.619927 | 7.794362 | 6.871682 | 6.891322 | 6.164178 | 8.526752 | 8.153115 |
| Bmyc | 16.81222 | 19.95775 | 18.86267 | 16.25369 | 16.61424 | 12.14317 | 18.34625 | 16.48937 |
| Vtcn1 | 0.081368 | 0.013152 | 0.024958 | 0.117821 | 0.138257 | 0.142687 | 0.130044 | 0.07212 |
| Abca6 | 0.009678 | 0.01095 | 0 | 0.008918 | 0.020929 | 0.008485 | 0.021654 | 0.034311 |
| Mansc4 | 0.102678 | 0.174263 | 0.496043 | 0.520377 | 0.222049 | 0 | 0.229744 | 0.136513 |
| Rnase13 | 0.453385 | 0.512983 | 0.365053 | 0.313332 | 0.367679 | 0 | 0 | 0.602786 |
| Csf3 | 0 | 0.045859 | 0 | 0 | 0.043825 | 0 | 0 | 0 |
| Tshz3 | 0.8576 | 0.698168 | 0.887005 | 0.828794 | 0.893385 | 1.063727 | 1.298761 | 1.446108 |
| Myadm | 56.55519 | 56.23512 | 59.01921 | 48.76106 | 49.45033 | 50.32872 | 62.71643 | 61.05545 |
| RGD130806 | 0.033122 | 0.074952 | 0.035559 | 0 | 0 | 0 | 0.074111 | 0.029358 |
| Mcm4 | 2.587714 | 2.643961 | 2.727566 | 2.904726 | 2.798058 | 2.708941 | 2.947656 | 2.668939 |
| Txndc2 | 0 | 0 | 0 | 0 | 0 | 0.026804 | 0 | 0 |
| AABR07033 | 0 | 0.459299 | 0 | 0 | 0 | 0.355926 | 0 | 0 |
| Lrrc52 | 0.25796 | 0.250174 | 0.276937 | 0.101872 | 0.358623 | 0.064623 | 0.206139 | 0.065327 |
| Btbd2 | 19.89766 | 22.98475 | 21.92061 | 19.55084 | 18.43611 | 22.59188 | 22.92106 | 23.66894 |
| Rn50_7_14 | 0.224746 | 0.25429 | 0 | 0.207095 | 0 | 0.197058 | 0 | 0.398408 |
| AABR07052 | 0.407517 | 0 | 0 | 0.187755 | 0 | 0.178656 | 0 | 0.180601 |
| Kank3 | 27.39648 | 29.5479 | 32.18422 | 27.03392 | 25.23554 | 25.12784 | 34.19068 | 34.24727 |
| Defa5 | 0 | 0 | 0 | 0.098677 | 0 | 0 | 0.119805 | 0 |
| Tmem68 | 2.77162 | 2.13512 | 1.772645 | 2.898086 | 3.273223 | 2.774862 | 3.848467 | 3.136116 |
| Idh3a | 84.63695 | 83.55154 | 79.73381 | 85.30605 | 85.69334 | 81.95549 | 73.06413 | 70.52743 |
| Nop58 | 10.4902 | 9.367015 | 9.861767 | 11.52119 | 11.03633 | 10.56505 | 11.60909 | 12.21302 |
| AABR07012 | 0 | 0 | 0 | 0.309315 | 0 | 0 | 0 | 0 |
| Sigmar1 | 5.825396 | 5.846998 | 5.615091 | 8.542421 | 8.364689 | 8.897305 | 9.180133 | 9.438359 |
| Mgp | 318.4561 | 318.5979 | 317.0172 | 1037.942 | 1028.503 | 1052.637 | 1100.369 | 1105.915 |
| Vpreb3 | 0 | 0 | 0 | 0.150321 | 0 | 0 | 0 | 0 |
| Uqcr10 | 708.8558 | 743.5099 | 644.0407 | 624.1131 | 614.8376 | 544.7026 | 581.2742 | 605.3132 |
| Snrpa1 | 7.92539 | 7.976962 | 6.681453 | 7.347738 | 7.938718 | 8.611631 | 8.431405 | 9.869011 |
| Myrf1 | 0.11715 | 0.088366 | 0.230574 | 0.12594 | 0.063336 | 0.102717 | 0.087375 | 0.121142 |
| LOC102553 | 0 | 0 | 0 | 0.080826 | 0.023711 | 0.038454 | 0.024533 | 0.019437 |
| Art4 | 2.902052 | 2.480894 | 2.930914 | 2.713744 | 3.254167 | 3.713117 | 2.284706 | 2.476965 |
| Tsen54 | 3.439904 | 4.311237 | 3.749771 | 4.608309 | 4.94982 | 5.080987 | 4.884522 | 4.948693 |
| Tdg | 4.864129 | 5.080184 | 4.528127 | 4.560462 | 5.057223 | 4.697325 | 6.39313 | 6.135339 |
| Pls3 | 17.46099 | 17.32534 | 15.92634 | 25.4857 | 26.49513 | 25.76055 | 27.24158 | 28.15703 |

| | | | | | | | | |
|---|---|---|---|---|---|---|---|---|
| Cd24 | 0.397141 | 0.224673 | 0.243632 | 0.496647 | 0.245385 | 0.472576 | 0.15868 | 0.402293 |
| Klk10 | 1.496169 | 1.76338 | 1.606233 | 2.642434 | 1.21334 | 2.295724 | 1.464617 | 1.326129 |
| Usp46 | 5.936413 | 5.788438 | 5.181397 | 6.285508 | 5.966673 | 5.92445 | 5.921461 | 6.316937 |
| Mki67 | 0.593622 | 0.617683 | 0.438137 | 0.61049 | 0.521523 | 0.645963 | 0.741203 | 0.742256 |
| Serp1 | 17.24665 | 16.04574 | 15.52604 | 20.12868 | 17.17814 | 18.16896 | 22.84464 | 24.10647 |
| Oprk1 | 0 | 0 | 0 | 0 | 0 | 0.017783 | 0 | 0 |
| Socs5 | 8.078182 | 8.090304 | 8.406826 | 7.683112 | 8.052623 | 8.189331 | 8.276312 | 8.750008 |
| Cdan1 | 4.825962 | 5.444383 | 4.938594 | 4.706989 | 4.409568 | 4.825289 | 5.446432 | 5.340609 |
| Pigh | 2.209533 | 2.199984 | 1.826497 | 2.667156 | 2.771397 | 2.227918 | 2.150576 | 3.192222 |
| Slc6a2 | 0.016524 | 0 | 0.03548 | 0.060906 | 0.017868 | 0 | 0.05546 | 0.043939 |
| Msc | 1.79687 | 2.420326 | 1.837195 | 4.021095 | 3.515777 | 3.07597 | 4.977782 | 4.095399 |
| Parp3 | 27.8851 | 28.01044 | 28.27032 | 28.9242 | 28.74432 | 27.0834 | 29.01229 | 29.37515 |
| LOC501038 | 1.053337 | 1.021544 | 0.161546 | 0.693292 | 0.162708 | 0.791629 | 0.336693 | 0.133375 |
| Eif2s2 | 57.77049 | 56.52261 | 52.18018 | 51.38252 | 51.09451 | 53.0546 | 50.94496 | 54.70051 |
| Snx6 | 7.906333 | 7.230041 | 8.604227 | 9.480968 | 9.134552 | 8.261759 | 10.08723 | 9.359701 |
| AC119007. | 0 | 0.219443 | 0 | 0 | 0 | 0 | 0 | 0 |
| Rab33b | 3.466081 | 2.27883 | 2.698606 | 2.891735 | 2.616723 | 2.751584 | 3.423552 | 2.864581 |
| Zcrb1 | 18.33406 | 16.76704 | 17.2814 | 14.53851 | 15.03023 | 16.07532 | 17.83012 | 13.87833 |
| #N/A | 0 | 0.181747 | 0 | 0.148016 | 0 | 0 | 0 | 0.142376 |
| Mfsd6l | 0.10003 | 0.16977 | 0.134236 | 0.207392 | 0.081121 | 0.043853 | 0.055955 | 0.088662 |
| Adam32 | 0.121358 | 0.04577 | 0.021714 | 0.055913 | 0.109352 | 0.088672 | 0.067885 | 0.107566 |
| AABR07051 | 0 | 0.168802 | 0.640663 | 0 | 0 | 0 | 0 | 0 |
| AABR07026 | 0 | 0 | 0 | 0 | 0 | 0 | 0.104243 | 0.123882 |
| Rfc5 | 5.224004 | 5.056154 | 4.865033 | 5.277687 | 6.193087 | 7.560438 | 6.266869 | 6.415468 |
| Slc39a6 | 1.657461 | 1.429714 | 1.620831 | 2.721894 | 2.7149 | 2.215867 | 2.919136 | 3.039998 |
| AABR07004 | 0 | 0.121165 | 0 | 0.098677 | 0 | 0 | 0 | 0 |
| Mau2 | 11.82072 | 12.29708 | 11.39955 | 11.68211 | 12.08651 | 12.20142 | 13.66398 | 13.51603 |
| AABR07051 | 0 | 0 | 0 | 0.31538 | 0 | 0 | 0 | 0 |
| Nek1 | 1.211773 | 1.119236 | 1.101796 | 1.184965 | 1.256792 | 1.582884 | 1.231177 | 1.578203 |
| Set | 3.452698 | 3.320581 | 3.274245 | 3.075475 | 2.800014 | 2.522774 | 3.283316 | 2.805273 |
| AABR07028 | 2.473007 | 2.868043 | 3.65053 | 3.019382 | 2.740877 | 2.818835 | 2.974192 | 2.904332 |
| Gnaz | 0.085079 | 0.096263 | 0.068503 | 0.176392 | 0.091994 | 0.018649 | 0.166569 | 0.169671 |
| Plekhh1 | 0.829488 | 0.734499 | 0.735637 | 0.742186 | 0.62394 | 0.801079 | 1.089384 | 0.671284 |
| Fmc1 | 50.68049 | 47.85202 | 48.85426 | 41.75196 | 41.40733 | 39.59093 | 38.76411 | 39.98736 |
| Card14 | 0.734592 | 0.732209 | 0.67597 | 1.192629 | 1.304928 | 1.410866 | 1.291446 | 1.488241 |
| Ppial4d | 14.52336 | 10.87785 | 29.7927 | 10.93235 | 18.06317 | 51.47444 | 51.64299 | 27.31688 |
| Lypla2 | 17.23953 | 18.27215 | 19.45873 | 15.85415 | 15.32529 | 18.07304 | 19.09624 | 18.11887 |
| Dgkb | 1.586846 | 1.98491 | 1.540927 | 1.205043 | 1.457165 | 1.475248 | 1.712845 | 1.774028 |
| AABR07042 | 0 | 0 | 0 | 0 | 0 | 0.143036 | 0 | 0 |
| Ankub1 | 0 | 0 | 0 | 0 | 0 | 0 | 0 | 0.080163 |
| AABR07039 | 0.189047 | 0.320846 | 0.202954 | 0.261299 | 0.204413 | 0.165756 | 0 | 0.418904 |
| Dera | 8.697349 | 9.53205 | 8.523818 | 7.986341 | 7.569329 | 7.918123 | 9.32337 | 8.998185 |
| Wdr59 | 4.240295 | 4.241864 | 4.246895 | 4.52671 | 4.491779 | 4.216636 | 5.168101 | 4.797287 |
| Nsmaf | 10.2 | 9.774574 | 9.426826 | 10.33062 | 9.724801 | 9.394427 | 10.16103 | 10.73886 |
| Itga8 | 1.001363 | 0.78905 | 0.835066 | 2.051397 | 2.020506 | 2.069562 | 1.940486 | 2.21097 |
| Cmtm7 | 2.154798 | 2.372601 | 2.623825 | 2.505272 | 3.049265 | 2.434571 | 3.349082 | 3.678809 |
| Gtf2h1 | 5.625087 | 5.631915 | 5.322044 | 6.674893 | 6.58554 | 6.652988 | 7.085394 | 6.99446 |
| Nicn1 | 39.53081 | 34.4718 | 35.76228 | 46.36325 | 42.10025 | 44.55126 | 48.03721 | 46.76637 |
| Gpank1 | 8.134879 | 11.08789 | 11.5361 | 11.67977 | 10.92356 | 11.27957 | 11.97938 | 11.60363 |
| Rpe | 9.621335 | 11.01517 | 11.34978 | 12.54511 | 11.63702 | 10.16988 | 12.55082 | 13.34797 |
| Mslnl | 0.279488 | 0.603707 | 0.218217 | 0.398012 | 0.384626 | 0.623778 | 0.454806 | 0.765693 |
| Akr1a1 | 185.642 | 185.1254 | 186.6012 | 225.9387 | 219.1693 | 230.7817 | 221.8082 | 214.7366 |
| Rac2 | 7.997145 | 8.308861 | 9.122036 | 10.55759 | 9.977535 | 11.76517 | 10.96849 | 10.3257 |
| Kifc3 | 18.01161 | 19.29263 | 20.87475 | 17.69959 | 17.75625 | 17.95994 | 19.39318 | 19.9976 |

| | | | | | | | | |
|---|---|---|---|---|---|---|---|---|
| AABR07045 | 0 | 0 | 0 | 0 | 0 | 0 | 0 | 0 |
| Clec4a2 | 0.244701 | 0.50759 | 0.218918 | 0.488544 | 0.352788 | 0.500625 | 0.319387 | 0.795264 |
| Tmem185a | 5.804761 | 5.732333 | 6.077639 | 5.878075 | 5.078948 | 5.773032 | 5.25495 | 5.999526 |
| Ccdc28a | 15.95079 | 13.83316 | 17.31237 | 11.79072 | 15.30466 | 15.13833 | 14.26622 | 14.83711 |
| AC103221. | 9.716501 | 8.366935 | 9.41585 | 9.191073 | 10.22738 | 8.14247 | 10.77419 | 13.03265 |
| Olr1589 | 0 | 0 | 0 | 0 | 0 | 0.048897 | 0 | 0.04943 |
| Nenf | 60.37294 | 66.85961 | 65.75978 | 57.9923 | 56.96883 | 58.90253 | 59.3013 | 53.79542 |
| Zwint | 22.47931 | 23.65453 | 23.39609 | 30.30645 | 29.25445 | 29.74035 | 27.10061 | 26.0844 |
| Tmem266 | 0.501422 | 0.518002 | 0.187238 | 0.32142 | 0.377169 | 0.229381 | 0.12195 | 0.17391 |
| Alkbh6 | 0.193948 | 0.438886 | 0.312323 | 0.312752 | 2.831126 | 0.382621 | 0.10849 | 2.105844 |
| LOC100911 | 0 | 0 | 0.08716 | 0.224433 | 0.087787 | 0.14237 | 0 | 0.359802 |
| Itch | 10.4405 | 9.861233 | 9.337205 | 10.22284 | 10.09153 | 9.647773 | 10.56312 | 10.81503 |
| Entpd1 | 0.343856 | 0.363956 | 0.202438 | 0.367954 | 0.443769 | 0.816949 | 0.310234 | 0.422755 |
| Ccer2 | 0.317369 | 0.287271 | 0.20443 | 0.584886 | 0.274533 | 0.166962 | 0.568094 | 0.61886 |
| Eif3k | 127.3484 | 130.9986 | 128.6854 | 137.7058 | 146.5413 | 139.9553 | 132.4481 | 124.5174 |
| Ifi30 | 20.44054 | 19.56221 | 21.81069 | 24.03105 | 23.80566 | 21.55763 | 25.14085 | 24.84261 |
| Elk3 | 14.57962 | 14.30804 | 15.10734 | 12.18335 | 11.34527 | 12.55493 | 14.96073 | 15.39046 |
| Prkar2a | 25.58973 | 24.71275 | 28.08013 | 19.57954 | 19.09771 | 19.90731 | 26.72812 | 26.1231 |
| H3f3a | 482.5055 | 454.8234 | 428.5438 | 429.6995 | 432.866 | 417.6989 | 444.8729 | 444.7211 |
| Cryba1 | 0.135663 | 0.460489 | 0.072821 | 0 | 0.14669 | 0 | 0.303547 | 0 |
| Arl16 | 11.63687 | 11.42394 | 11.81929 | 11.66906 | 12.8295 | 12.90407 | 11.99773 | 11.83115 |
| Cyp2j4 | 3.223769 | 2.881252 | 2.559337 | 1.822286 | 1.318165 | 1.876484 | 2.606457 | 3.001456 |
| Ascc2 | 7.913073 | 8.242274 | 8.645104 | 7.098572 | 7.247682 | 7.16266 | 8.306004 | 8.437128 |
| AABR07068 | 0.274167 | 0 | 0.294336 | 0 | 0 | 0 | 0 | 0 |
| Tbc1d15 | 10.46344 | 11.03002 | 9.349344 | 10.82439 | 11.6829 | 11.02631 | 12.30587 | 12.29848 |
| Ppp3ca | 16.79492 | 17.57619 | 17.47452 | 14.81201 | 13.63225 | 15.59434 | 15.06175 | 16.5025 |
| Gpc6 | 5.236592 | 5.023334 | 5.377389 | 7.007194 | 6.868557 | 7.106764 | 10.29052 | 9.484706 |
| Asl | 5.385875 | 5.000942 | 6.881933 | 6.71606 | 6.614927 | 6.672871 | 7.761076 | 6.019099 |
| Ccdc74a | 0.080563 | 0.182306 | 0.129734 | 0.222707 | 0.174223 | 0.105957 | 0.315456 | 0.178517 |
| AABR07021 | 0.213303 | 0.362014 | 0.228995 | 0.393101 | 0.461283 | 0.187024 | 0.357951 | 0.189061 |
| LOC100911 | 0 | 0 | 0.440927 | 0.084101 | 0.666147 | 0.060019 | 0.17869 | 0.444932 |
| Kcnn4 | 2.377425 | 2.159035 | 2.283653 | 1.844803 | 2.029481 | 1.947389 | 3.2897 | 2.634038 |
| LOC102553 | 0.054181 | 0.030652 | 0.029083 | 0 | 0 | 0 | 0 | 0.312151 |
| Hoxd4 | 0.038862 | 0.04397 | 0.02086 | 0.053714 | 0.02101 | 0.034074 | 0.065215 | 0.086112 |
| Dnai2 | 0 | 0 | 0.018948 | 0 | 0 | 0 | 0.019745 | 0 |
| Cisd1 | 264.9269 | 267.3158 | 256.1909 | 268.4122 | 266.9124 | 254.4559 | 241.5107 | 248.7997 |
| AABR07045 | 0.116888 | 0.264507 | 0.125487 | 0 | 0.12639 | 0 | 0 | 0 |
| Hist1h2aa | 3.331165 | 3.31677 | 4.291463 | 3.437882 | 3.601941 | 2.336615 | 2.385126 | 3.188784 |
| Nfil3 | 5.129723 | 5.048306 | 6.052057 | 5.883928 | 5.286694 | 5.130241 | 7.502424 | 6.606965 |
| Spata33 | 2.341946 | 3.497739 | 3.218213 | 3.280177 | 4.0517 | 2.546242 | 3.039276 | 3.238222 |
| AABR07009 | 2.244254 | 0 | 4.015583 | 4.135983 | 6.471144 | 4.591449 | 1.673848 | 1.989194 |
| Slc35e3 | 3.647378 | 4.147891 | 4.395165 | 5.161405 | 5.050551 | 5.172314 | 5.600311 | 5.360596 |
| AABR07058 | 0.050841 | 0.115048 | 0 | 0.046848 | 0 | 0 | 0.113757 | 0 |
| Sh3glb1 | 120.7108 | 113.357 | 112.7782 | 86.53022 | 91.71032 | 86.6766 | 93.36628 | 93.54745 |
| AC123495. | 0 | 0 | 0 | 0 | 0 | 0 | 0 | 0 |
| Dpy19l1 | 8.299038 | 8.335347 | 7.824488 | 7.936984 | 7.662193 | 7.896937 | 8.731791 | 9.147532 |
| Engase | 2.04074 | 2.012976 | 2.148733 | 2.398794 | 2.277348 | 2.282534 | 2.722145 | 3.049467 |
| Apopt1 | 1.349637 | 1.730658 | 1.352327 | 1.160728 | 1.653922 | 1.262254 | 1.711236 | 1.03675 |
| Ube2e2 | 14.62212 | 16.38124 | 17.01237 | 14.66842 | 14.09721 | 15.72587 | 16.7615 | 16.98248 |
| Sema4b | 2.936124 | 3.304962 | 3.412085 | 2.914713 | 2.471098 | 2.773447 | 3.183213 | 3.581698 |
| LOC100911 | 0.102678 | 1.487048 | 0.132278 | 0.13246 | 0.199844 | 1.5845 | 0.206769 | 0.200219 |
| Suox | 9.579715 | 11.08227 | 10.69479 | 10.7645 | 10.90087 | 10.66171 | 12.2675 | 11.32836 |
| Tbxas1 | 0.854518 | 0.936633 | 1.146724 | 2.140756 | 1.501464 | 1.358001 | 1.852243 | 2.201198 |
| Oxa1l | 44.46416 | 43.59112 | 46.24938 | 45.43528 | 41.65834 | 41.06078 | 40.60472 | 41.50795 |

| | | | | | | | | |
|---|---|---|---|---|---|---|---|---|
| RGD156088 | 0 | 0 | 0 | 0 | 0 | 0.054856 | 0 | 0 |
| Rhag | 0 | 0.027713 | 0 | 0 | 0.026484 | 0 | 0 | 0 |
| Pdgfd | 1.640916 | 1.910695 | 1.539286 | 2.231359 | 2.704513 | 2.458457 | 2.334832 | 2.499352 |
| Synpo | 36.37351 | 40.14194 | 43.87391 | 29.036 | 26.99203 | 29.67587 | 43.46224 | 42.94471 |
| Plpp1 | 20.1927 | 22.68619 | 21.44916 | 26.14121 | 26.21626 | 25.84054 | 27.68155 | 27.35085 |
| Commd9 | 21.87191 | 23.64471 | 24.5791 | 24.05924 | 21.06884 | 22.89318 | 21.96244 | 24.56731 |
| Ccnb1ip1 | 0.092031 | 0.03471 | 0 | 0.056536 | 0.099512 | 0.080693 | 0.03432 | 0 |
| Uso1 | 11.24948 | 10.90993 | 10.33712 | 12.63743 | 13.87215 | 13.18325 | 13.83482 | 13.70103 |
| Adgrf2 | 0.008676 | 0 | 0 | 0 | 0 | 0 | 0.019412 | 0 |
| Lrrcc1 | 1.11196 | 0.98973 | 0.986842 | 1.379832 | 1.298534 | 1.299957 | 1.642096 | 1.576937 |
| Pdia5 | 1.747539 | 1.568171 | 1.55263 | 2.720833 | 2.410855 | 1.532244 | 3.741598 | 2.991039 |
| Faf1 | 20.18448 | 18.78488 | 17.5491 | 19.04453 | 18.22864 | 19.01887 | 18.98754 | 17.73932 |
| Ercc6l2 | 4.635733 | 4.245032 | 4.350876 | 4.098354 | 4.351667 | 4.402646 | 4.029077 | 4.417255 |
| Pcdhgb7 | 1.62224 | 1.370498 | 1.137831 | 0.936761 | 1.356508 | 1.08101 | 1.306721 | 1.476216 |
| Rn50_13_( | 0 | 0 | 0.099855 | 0.171414 | 0.100573 | 0.163107 | 0.156087 | 0.123662 |
| Arhgap27 | 7.283076 | 7.877242 | 8.723508 | 7.043848 | 7.311029 | 6.50895 | 8.641799 | 10.26099 |
| Zfp523 | 8.111254 | 8.060035 | 9.181698 | 6.815852 | 6.339358 | 6.725035 | 8.674856 | 8.064802 |
| Hist1h1t | 0.210869 | 0 | 0.150921 | 0.064769 | 0.076003 | 0 | 0 | 0.062301 |
| Rnf39 | 1.245518 | 1.654331 | 1.337143 | 2.095793 | 1.522425 | 1.37696 | 1.575182 | 2.447922 |
| Mdh1 | 1111.49 | 1104.804 | 1090.881 | 1031.434 | 1009.502 | 997.25 | 974.2991 | 983.8252 |
| Taf1d | 6.65156 | 7.389093 | 6.058924 | 7.912177 | 7.715308 | 7.846817 | 7.666969 | 7.789349 |
| Rab2b | 5.828555 | 8.397983 | 6.012899 | 9.021238 | 10.29053 | 9.781782 | 9.220717 | 9.081101 |
| Rn50_13_( | 0 | 0.119938 | 0 | 0 | 0 | 0 | 0 | 0 |
| Igfbpl1 | 0.157729 | 0 | 0.253998 | 0.07267 | 0.085275 | 0 | 0.044115 | 0.244655 |
| Fbrs | 11.28801 | 12.81415 | 13.32222 | 12.60574 | 10.02311 | 10.15952 | 14.0921 | 15.53777 |
| AABR07069 | 0.113839 | 0.257607 | 0.122213 | 0.052449 | 0 | 0.199628 | 0.127358 | 0.050451 |
| Asap1 | 5.97637 | 5.574066 | 6.606761 | 5.655827 | 5.239591 | 5.466685 | 7.390848 | 7.258532 |
| Prosc | 32.64236 | 29.95765 | 31.80295 | 34.72246 | 34.1616 | 34.78914 | 34.20062 | 34.60115 |
| AABR07017 | 8.092915 | 8.564257 | 7.793881 | 6.119375 | 6.279951 | 6.699341 | 6.976901 | 7.046568 |
| Col6a2 | 51.18964 | 54.11835 | 55.77694 | 68.59911 | 65.74035 | 65.94538 | 72.44204 | 73.87382 |
| Ptp4a3 | 159.2287 | 165.2469 | 180.0506 | 134.5052 | 131.9343 | 135.4598 | 145.8657 | 145.2946 |
| Timm8a1 | 0.959083 | 0.868126 | 1.441491 | 0.883757 | 0.622225 | 0.840925 | 0.643788 | 0.51005 |
| Lhx3 | 0 | 0 | 0 | 0 | 0 | 0.038072 | 0 | 0 |
| Rn50_6_08 | 0 | 0 | 0.018597 | 0.015962 | 0.037461 | 0.030377 | 0.01938 | 0.030708 |
| Rnf2 | 9.144128 | 8.823744 | 9.374607 | 10.09596 | 9.174807 | 10.2808 | 10.35293 | 10.02768 |
| AABR07008 | 0.573028 | 0.44886 | 0.899112 | 0.406171 | 0.428958 | 0.734323 | 0.197255 | 0.859528 |
| RGD130695 | 25.33695 | 26.11497 | 27.38714 | 32.34199 | 30.6803 | 32.06786 | 29.07391 | 30.34056 |
| Mir34b | 0 | 0 | 0 | 0 | 0 | 0 | 0 | 0 |
| Fbxo7 | 12.1291 | 10.54324 | 11.84058 | 13.51313 | 13.57751 | 13.44753 | 11.45033 | 11.59009 |
| Orc5 | 6.381424 | 6.030422 | 5.080418 | 5.549881 | 6.331591 | 6.349653 | 7.379903 | 6.24933 |
| Ndufa6 | 397.4543 | 394.4624 | 379.1736 | 357.8385 | 348.9349 | 350.8861 | 323.1789 | 328.1336 |
| Lhfpl4 | 0.267719 | 0.060582 | 0.344897 | 0.049339 | 0 | 0.328631 | 0.179708 | 0.047459 |
| Cyp2ab1 | 0.158342 | 0.358313 | 0.461402 | 0.500248 | 0.24459 | 0.257835 | 0.430211 | 0.340841 |
| Tut1 | 9.509461 | 10.30517 | 9.248858 | 7.467547 | 6.710232 | 6.721535 | 9.495249 | 9.658232 |
| Cntnap5c | 0 | 0 | 0.01464 | 0 | 0 | 0.011957 | 0 | 0.012087 |
| Wdr17 | 0.023212 | 0 | 0.049839 | 0.021389 | 0 | 0 | 0.051937 | 0.020574 |
| Rpl30 | 247.2898 | 228.2587 | 210.6047 | 156.2732 | 148.8644 | 133.8592 | 172.4499 | 182.1342 |
| Txnl1 | 77.97489 | 75.48576 | 71.33924 | 74.12918 | 73.04591 | 76.38219 | 80.17109 | 77.13576 |
| Napg | 12.17459 | 13.6522 | 11.9053 | 11.71833 | 13.46926 | 13.41472 | 11.99371 | 13.04146 |
| Ric1 | 5.81584 | 5.338276 | 5.453814 | 5.434396 | 5.189976 | 5.549878 | 6.728614 | 6.614379 |
| AABR07073 | 0.37139 | 0 | 0 | 0 | 0.401578 | 0 | 0 | 0 |
| LOC685025 | 1.296297 | 1.112668 | 1.967516 | 1.79173 | 1.45 | 1.763681 | 1.675276 | 1.921557 |
| Clec12a | 1.223503 | 1.292047 | 1.926479 | 3.382229 | 2.734109 | 2.503126 | 2.828855 | 3.831728 |
| Leprot | 16.18926 | 17.25065 | 16.22345 | 19.98137 | 20.27226 | 20.76072 | 19.82958 | 18.59923 |

| | | | | | | | | |
|---|---|---|---|---|---|---|---|---|
| LOC103689 | 2.152024 | 2.25455 | 3.422719 | 2.20334 | 1.723669 | 28.58299 | 1.961739 | 2.331323 |
| RGD130887 | 0 | 0 | 0 | 0 | 0 | 0 | 0 | 0.032895 |
| Wwtr1 | 17.01018 | 20.18155 | 19.8658 | 16.02578 | 17.8428 | 18.17623 | 23.19199 | 23.36226 |
| Smarcb1 | 0.014103 | 0 | 0.015141 | 0 | 0 | 0 | 0 | 0.012501 |
| Arpc3 | 29.72434 | 28.37674 | 30.22817 | 32.89986 | 34.52592 | 30.54179 | 36.8265 | 33.08707 |
| AABR07004 | 0.110167 | 0.041549 | 0.039424 | 0.033838 | 0 | 0 | 0 | 0 |
| AABR07011 | 0.82683 | 0.207893 | 0.394513 | 1.269819 | 0.993377 | 0.563862 | 0.719461 | 1.791438 |
| Dbf4 | 0.194359 | 0 | 0 | 0.025585 | 0.180135 | 0.04869 | 0.062126 | 0.39376 |
| Mirlet7c- | 0 | 0 | 2.36708 | 2.031711 | 1.788079 | 0.966621 | 2.466723 | 0.488574 |
| Mtss1 | 14.76493 | 14.38384 | 15.91024 | 13.47839 | 13.96947 | 13.91202 | 16.30262 | 15.84588 |
| Hectd1 | 18.73458 | 18.87616 | 18.24922 | 18.57769 | 18.3327 | 18.23629 | 18.62902 | 19.9287 |
| Lfng | 6.861234 | 8.095871 | 7.497509 | 7.38361 | 6.173629 | 7.971116 | 8.964523 | 9.230777 |
| Slc10a5 | 0.216516 | 0.419962 | 0.298856 | 0.456025 | 0.267561 | 0.298322 | 0.276832 | 0.137078 |
| Hdac3 | 19.45757 | 21.18141 | 20.57244 | 18.91414 | 20.00317 | 20.64698 | 21.60335 | 22.66831 |
| Hapln4 | 0 | 0 | 0.015248 | 0 | 0 | 0 | 0.031779 | 0 |
| Pram1 | 0.287438 | 0.502616 | 0.533007 | 0.385255 | 0.565095 | 0.64152 | 0.76008 | 0.463219 |
| LOC100359 | 5.88791 | 7.36935 | 5.761662 | 9.938705 | 6.817238 | 7.081339 | 8.5691 | 11.22261 |
| Tnnc1 | 2795.222 | 2774.304 | 2808.52 | 2978.284 | 2958.801 | 2851.156 | 2456.148 | 2498.273 |
| Scx | 19.9951 | 20.74258 | 22.55667 | 15.87162 | 14.77977 | 15.99314 | 16.11853 | 13.71152 |
| Ermard | 7.069528 | 7.068064 | 7.092817 | 7.367072 | 8.3669 | 8.925938 | 8.455519 | 10.43586 |
| Tspan33 | 0.448926 | 0.388424 | 0.340201 | 0.340668 | 0.228432 | 0.532543 | 0.265891 | 0.608562 |
| Stxbp5 | 6.937894 | 7.340993 | 7.906999 | 6.538056 | 6.748013 | 6.507099 | 8.114045 | 8.381922 |
| Duoxa2 | 0.054378 | 0.061526 | 0.291891 | 0.150321 | 0.235192 | 0.238393 | 0.243342 | 0.192791 |
| Rpl36a | 179.7192 | 175.8889 | 169.8309 | 155.6506 | 168.598 | 154.6954 | 167.7851 | 168.5215 |
| AABR07024 | 0 | 0.188094 | 0 | 0.153185 | 0 | 0.14576 | 0 | 0 |
| Mrgprg | 0.060191 | 0.068103 | 0.193856 | 0.110927 | 0 | 0.052775 | 0.269355 | 0.16005 |
| Msx1 | 2.969656 | 3.060618 | 3.124985 | 3.522127 | 3.020291 | 2.861599 | 4.17757 | 4.221871 |
| Asxl1 | 6.4143 | 6.094167 | 6.03323 | 5.081544 | 5.482858 | 5.839192 | 6.143417 | 5.674957 |
| Ddi1 | 0 | 0 | 0 | 0 | 0.030656 | 0.024859 | 0 | 0.02513 |
| AC123159. | 0 | 0 | 0 | 0 | 0.589818 | 0.478276 | 0 | 0.966969 |
| Ephb4 | 22.31863 | 23.08463 | 25.23911 | 19.4444 | 19.17746 | 19.80736 | 27.61952 | 27.36678 |
| Gsta4 | 28.0676 | 26.42622 | 28.39812 | 25.24296 | 22.48889 | 23.78347 | 19.20191 | 21.35784 |
| Fam228b | 0.149831 | 0 | 0 | 0.069032 | 0.16201 | 0.065686 | 0.251436 | 0.199204 |
| Vwa3a | 0.29376 | 0.361278 | 0.287947 | 0.270688 | 0.400501 | 0.391953 | 0.285779 | 0.283015 |
| LOC102554 | 0.105576 | 0.047782 | 0.090674 | 0.019457 | 0.091327 | 0.203653 | 0.377966 | 0.224586 |
| Ska1 | 0.087277 | 0.230415 | 0.218626 | 0.053615 | 0.157285 | 0.102032 | 0.32547 | 0.103143 |
| Lipa | 26.80326 | 26.22643 | 25.07737 | 24.29202 | 25.08123 | 23.48681 | 23.50326 | 25.0158 |
| Clta | 57.22948 | 55.18919 | 53.93699 | 69.09466 | 70.05057 | 61.06751 | 74.37981 | 73.11647 |
| LOC100362 | 0 | 0 | 0 | 0.010561 | 0 | 0 | 0 | 0 |
| Fam163b | 0.267563 | 0.518974 | 0.430869 | 0.422655 | 0.330642 | 0.485956 | 0.555913 | 0.575947 |
| Ddhd1 | 3.797529 | 3.653086 | 4.043878 | 3.853451 | 3.79035 | 4.273309 | 4.850521 | 4.633275 |
| Fam3d | 0.158685 | 0.179544 | 0.170358 | 0.219332 | 0 | 0.069567 | 0.310676 | 0.035163 |
| LOC100911 | 74.75624 | 67.52177 | 61.02565 | 65.6849 | 67.78891 | 67.62971 | 74.43218 | 74.60344 |
| Zfp324 | 1.649557 | 2.137871 | 1.770904 | 1.658183 | 2.14037 | 2.07746 | 2.382309 | 2.073501 |
| Ctla2a | 14.51481 | 12.45869 | 16.45573 | 11.53002 | 15.55935 | 13.60429 | 14.2787 | 14.91697 |
| Paqr4 | 2.184042 | 2.312179 | 2.934314 | 2.436195 | 2.665401 | 2.530896 | 3.372191 | 3.362222 |
| Hist1h1c | 122.7608 | 119.3337 | 117.1872 | 143.9288 | 140.8031 | 138.7494 | 150.1748 | 142.0764 |
| AC114452. | 0 | 0 | 0 | 0 | 0 | 0 | 0.081481 | 0 |
| LOC102546 | 0 | 0.068286 | 0.08639 | 0.092688 | 0.15227 | 0.176391 | 0.022507 | 0.053493 |
| Ddx11 | 3.080348 | 3.513605 | 3.44138 | 2.63073 | 2.57252 | 2.72281 | 3.86642 | 4.284076 |
| Glis2 | 9.460153 | 9.680957 | 10.67476 | 15.38069 | 15.40267 | 14.53959 | 18.27284 | 19.6571 |
| Ctbp2 | 12.43518 | 14.22615 | 11.88782 | 10.96745 | 12.08004 | 12.02811 | 12.14533 | 12.29906 |
| Tgm1 | 0.031013 | 0.07018 | 0.049942 | 0.514395 | 0.385643 | 0.407887 | 0.173481 | 0.219909 |
| Hdac9 | 2.539273 | 2.491582 | 2.465907 | 2.631107 | 2.358322 | 2.226437 | 2.463621 | 2.502835 |

| | | | | | | | | |
|---|---|---|---|---|---|---|---|---|
| AABR07029 | 10.90957 | 4.114554 | 6.246462 | 12.06328 | 10.22351 | 10.84092 | 8.136759 | 5.801815 |
| Fem1a | 102.0122 | 102.4113 | 110.7715 | 95.93471 | 88.45494 | 97.26759 | 102.2496 | 99.36302 |
| Higd1a | 51.6787 | 52.99027 | 50.09466 | 45.81515 | 48.56012 | 46.63755 | 44.70733 | 42.75983 |
| Shisa7 | 0.0344 | 0.058384 | 0.036931 | 0.087171 | 0.083692 | 0.030162 | 0.06735 | 0.068604 |
| Cd82 | 10.15494 | 10.70421 | 9.566405 | 13.7828 | 13.35791 | 13.72361 | 15.14786 | 13.92436 |
| AABR07056 | 0 | 0 | 0 | 0 | 0 | 0.153048 | 0.195282 | 0.154715 |
| Mos | 0 | 0 | 0.109587 | 0 | 0 | 0 | 0.1142 | 0.135715 |
| Zmym1 | 2.082658 | 2.495043 | 2.177413 | 3.073049 | 2.325541 | 2.768953 | 2.665016 | 2.437154 |
| Ap3m2 | 2.930147 | 2.889068 | 2.741253 | 3.008589 | 4.752489 | 3.523414 | 3.371779 | 3.302072 |
| AABR07002 | 0.231708 | 0 | 0.124376 | 0.106755 | 0 | 0.304742 | 0 | 0.102687 |
| #N/A | 0 | 0 | 0 | 0.786736 | 0.307731 | 0 | 0.318395 | 0.504506 |
| AABR07001 | 0 | 0 | 0 | 0.408925 | 0.239926 | 0 | 0.49648 | 0.590015 |
| Xylt1 | 1.646329 | 1.62393 | 2.016693 | 2.333888 | 2.213778 | 2.109735 | 3.258639 | 2.60041 |
| AABR07065 | 0.150477 | 0 | 0 | 0 | 0 | 0 | 0 | 0 |
| Tlr6 | 0.32002 | 0.422435 | 0.263397 | 0.471817 | 0.426774 | 0.346066 | 0.417695 | 0.416019 |
| AABR07060 | 0 | 0 | 0 | 0 | 0 | 0 | 0 | 0 |
| AABR07001 | 24.62239 | 25.61689 | 29.30578 | 27.80148 | 28.71302 | 27.80063 | 23.5558 | 23.9318 |
| Arhgef5 | 1.215333 | 1.387154 | 1.339073 | 1.178823 | 1.406341 | 1.355375 | 1.478929 | 1.247296 |
| #N/A | 22.04379 | 23.72682 | 22.69216 | 29.23766 | 27.00855 | 27.75787 | 37.36607 | 35.75715 |
| Mblac1 | 2.572652 | 3.333375 | 3.875578 | 4.244135 | 4.082923 | 2.98335 | 3.528078 | 3.089397 |
| AABR07044 | 0 | 0 | 0 | 0 | 0 | 0.134253 | 0 | 0.27143 |
| Ube2a | 14.29519 | 13.47157 | 12.78232 | 12.89731 | 13.19712 | 12.89402 | 11.31607 | 13.2991 |
| LOC102554 | 2.880126 | 2.666231 | 3.091999 | 4.825314 | 4.624172 | 3.673159 | 3.905644 | 4.409379 |
| Jarid2 | 4.980151 | 4.919943 | 5.133714 | 4.326458 | 3.911468 | 4.670743 | 4.601398 | 4.359253 |
| Igsf5 | 0 | 0 | 0 | 0 | 0 | 0 | 0 | 0.054002 |
| #N/A | 0.392254 | 0.443817 | 1.263329 | 0.903617 | 1.272416 | 1.719644 | 1.755346 | 1.043023 |
| Pde3b | 1.515259 | 1.376955 | 1.075946 | 1.506193 | 1.690032 | 1.318119 | 1.975514 | 2.17849 |
| Dnaja4 | 84.7256 | 85.84216 | 84.71188 | 93.86516 | 97.12915 | 104.9474 | 104.1185 | 101.6953 |
| Pwp2 | 4.265462 | 4.795816 | 4.420844 | 4.054054 | 4.423634 | 4.045744 | 4.486889 | 5.361923 |
| LOC306766 | 56.83727 | 59.75297 | 58.678 | 43.39502 | 44.40321 | 44.02251 | 52.18863 | 52.94094 |
| Hmg20a | 8.766038 | 8.648028 | 7.92741 | 10.29259 | 10.14785 | 10.23547 | 9.082395 | 9.224216 |
| Kcng2 | 76.44057 | 80.9121 | 97.72417 | 79.41552 | 75.84582 | 73.86786 | 82.58589 | 83.86432 |
| Inpp5k | 15.04944 | 15.07302 | 13.93089 | 14.75187 | 15.19343 | 13.8686 | 14.51731 | 14.66617 |
| AABR07057 | 0 | 0 | 0 | 0 | 0 | 0 | 0 | 0 |
| AABR07005 | 0.953842 | 1.079227 | 1.331213 | 1.142606 | 0.8251 | 1.087228 | 0.746981 | 1.268156 |
| Ifitm3 | 110.3425 | 109.3578 | 104.4052 | 163.9917 | 157.815 | 150.9275 | 186.7177 | 181.6134 |
| Syf2 | 45.00373 | 41.82072 | 42.84254 | 45.85265 | 43.68139 | 44.49104 | 44.9839 | 47.11726 |
| AABR07013 | 0 | 0.163222 | 0.154871 | 0.265857 | 0 | 0 | 0 | 0.127864 |
| Mgst2 | 13.9097 | 12.65225 | 11.56572 | 15.0791 | 10.46927 | 11.35905 | 13.12052 | 12.44973 |
| Pnn | 18.43295 | 18.08362 | 15.62391 | 21.12464 | 19.75064 | 20.2473 | 20.87118 | 21.91568 |
| AABR07072 | 0.872765 | 2.962479 | 1.873939 | 0.804219 | 0 | 0 | 0 | 0.773575 |
| AABR07071 | 0 | 0 | 0.038427 | 0 | 0.038703 | 0 | 0 | 0 |
| AABR07015 | 11.0244 | 30.14452 | 25.64337 | 50.79277 | 51.65563 | 37.85931 | 3.083403 | 2.442869 |
| Lats2 | 11.50191 | 11.40058 | 11.89448 | 9.946491 | 9.638846 | 10.40901 | 13.05689 | 12.69425 |
| LOC102549 | 5.361936 | 5.9092 | 5.233074 | 7.764135 | 8.508436 | 9.463757 | 8.257945 | 9.505101 |
| Upp1 | 12.15362 | 10.20442 | 11.17191 | 10.58349 | 11.50232 | 11.26459 | 14.60305 | 15.41837 |
| LOC100364 | 2.378107 | 2.152573 | 2.501989 | 4.645624 | 2.982839 | 2.79407 | 3.139414 | 3.625476 |
| Emc7 | 32.81475 | 35.10317 | 35.42582 | 34.63569 | 33.99336 | 36.45795 | 34.40116 | 38.27876 |
| AABR07018 | 0 | 0 | 0.339687 | 0 | 0 | 0 | 0.176993 | 0.28045 |
| AABR07064 | 0 | 0.01155 | 0.010959 | 0 | 0 | 0 | 0 | 0 |
| Nrd1 | 25.00957 | 24.05181 | 23.48038 | 21.32291 | 22.24772 | 22.25142 | 25.23201 | 26.29108 |
| Madcam1 | 0.532257 | 0.138975 | 0.08791 | 0.226363 | 0.309896 | 0.251291 | 0.27483 | 0.399187 |
| Rn60_10_0 | 0 | 0 | 0 | 0 | 0 | 0 | 0 | 0 |
| Rabggtb | 59.0795 | 59.42831 | 53.54716 | 54.98528 | 58.07437 | 59.69576 | 57.61267 | 57.72062 |

| | | | | | | | | |
|---|---|---|---|---|---|---|---|---|
| AC099453. | 0 | 0 | 0 | 0 | 0.31457 | 0 | 0 | 0 |
| AABR07012 | 1.175819 | 0.90276 | 0.721324 | 0.967384 | 0.998954 | 1.104599 | 1.174512 | 0.856082 |
| Psd | 0.052274 | 0.103506 | 0.11224 | 0.144507 | 0.141309 | 0.103127 | 0.131585 | 0.081084 |
| AABR07017 | 0 | 0.093013 | 0 | 0 | 0.177779 | 0.072079 | 0 | 0.072864 |
| Pnma2 | 0.125917 | 0.14247 | 0.050693 | 0.188545 | 0.340382 | 0.303612 | 0.123262 | 0.16741 |
| Pbxip1 | 44.90519 | 45.49237 | 45.09165 | 50.53932 | 48.51727 | 50.40411 | 56.18301 | 53.26128 |
| LOC100910 | 0.087862 | 0.198824 | 0.565955 | 0.242885 | 0.190008 | 0.539264 | 0.196593 | 0.155753 |
| Zbtb7b | 10.53387 | 11.5262 | 12.07667 | 7.948985 | 8.179328 | 8.551953 | 9.84494 | 10.68149 |
| AABR07030 | 0 | 0 | 0 | 0 | 0 | 0 | 0 | 0 |
| Fbxo9 | 11.26893 | 9.814347 | 10.32037 | 13.50359 | 13.2805 | 16.07765 | 15.20602 | 16.99748 |
| Ercc6l | 0.119044 | 0.044897 | 0.0284 | 0.109694 | 0.185929 | 0.057987 | 0.088787 | 0.105514 |
| Ss1812 | 77.37261 | 72.26279 | 78.38739 | 67.76314 | 67.64553 | 68.90993 | 62.48381 | 68.81087 |
| Spty2d1 | 2.575755 | 2.795662 | 3.002973 | 2.427155 | 2.457465 | 2.646751 | 3.338009 | 3.192096 |
| Lonrf2 | 1.394101 | 1.550473 | 1.590198 | 1.14593 | 1.164826 | 1.139007 | 1.506488 | 1.326931 |
| AABR07073 | 0 | 0 | 0 | 0 | 0 | 0 | 0 | 1.141341 |
| Mmp20 | 0 | 0 | 0 | 0 | 0 | 0 | 0 | 0 |
| AABR07058 | 0.172825 | 0 | 0 | 0 | 0.373746 | 0.151533 | 0 | 0 |
| Ccdc88a | 2.567605 | 2.356517 | 2.224119 | 2.53857 | 2.395018 | 2.550805 | 3.254704 | 3.291602 |
| AABR07013 | 0.137084 | 0 | 0 | 0.126317 | 0 | 0 | 0 | 0 |
| Rpusd4 | 5.992989 | 6.714952 | 5.715513 | 6.219293 | 5.253311 | 6.045407 | 5.923561 | 5.492385 |
| LOC100912 | 0.049433 | 0.018644 | 0.318416 | 0.075917 | 0 | 0.028895 | 0.073738 | 0.02921 |
| Nme5 | 0 | 0.03898 | 0 | 0 | 0.037252 | 0.090621 | 0.115628 | 0 |
| Hexa | 17.92391 | 20.11437 | 19.49399 | 23.66778 | 24.03607 | 23.7533 | 27.88342 | 26.37425 |
| Zbtb8b | 0 | 0 | 0 | 0 | 0 | 0 | 0.04346 | 0 |
| AABR07057 | 0.240211 | 0 | 0 | 0 | 0.259736 | 0 | 0 | 0.212911 |
| LOC688390 | 0.295246 | 0.394795 | 0.23052 | 0.346255 | 0.261201 | 0.258872 | 0.390364 | 0.452012 |
| AABR07068 | 0.036517 | 0 | 0 | 0 | 0 | 0 | 0 | 0 |
| AABR07013 | 0 | 0 | 0 | 0 | 0 | 0 | 0 | 0 |
| AABR07072 | 0.010256 | 0.011604 | 0.01101 | 0 | 0 | 0 | 0 | 0 |
| Bnip2 | 28.60147 | 28.19709 | 26.22762 | 30.61846 | 32.85925 | 28.51984 | 33.09602 | 33.49053 |
| Satl1 | 0.05651 | 0.085251 | 0.080889 | 0.104143 | 0.101839 | 0.033032 | 0.147515 | 0.050088 |
| AABR07071 | 34.22029 | 41.06185 | 47.43077 | 35.80364 | 29.64418 | 29.39911 | 32.54704 | 36.10018 |
| Smarcal1 | 2.374794 | 2.589613 | 2.845086 | 2.315136 | 2.605045 | 3.168597 | 2.772327 | 2.821783 |
| Add1 | 31.122 | 33.27528 | 32.81714 | 31.89776 | 31.53801 | 32.32368 | 38.20487 | 38.60976 |
| Fam65a | 14.88427 | 14.86988 | 15.4909 | 13.40315 | 13.95942 | 13.41541 | 19.42356 | 18.91407 |
| Dlat | 117.2392 | 113.5085 | 110.4903 | 109.4047 | 109.0201 | 107.6518 | 108.607 | 105.6287 |
| Oxld1 | 15.46041 | 14.85942 | 16.50851 | 16.62052 | 14.38032 | 14.72179 | 14.78565 | 13.55599 |
| AC123188. | 0.39974 | 0 | 0.429146 | 0 | 0 | 0.350492 | 1.341634 | 0 |
| AABR07027 | 0 | 0 | 0 | 0.156159 | 0 | 0 | 0 | 0 |
| Afg3l1 | 15.93132 | 16.06575 | 16.93032 | 16.14624 | 16.5512 | 17.04134 | 18.02605 | 17.11982 |
| LOC100360 | 4.199154 | 2.794791 | 4.950027 | 3.793485 | 4.273397 | 4.47594 | 6.079541 | 3.430004 |
| Rps13 | 70.08823 | 69.67206 | 64.8175 | 66.79784 | 69.07297 | 60.22435 | 58.47265 | 63.54263 |
| RGD156531 | 0 | 0.150762 | 0.143049 | 0 | 0 | 0 | 0 | 0 |
| Fam8a1 | 28.29564 | 30.99456 | 35.8308 | 33.07287 | 34.39445 | 33.01014 | 37.17975 | 36.06278 |
| AC099450. | 1.563162 | 0.884322 | 1.678154 | 3.60098 | 0.845112 | 2.055872 | 0 | 1.385508 |
| Apba3 | 15.73436 | 14.93757 | 15.28184 | 14.27205 | 11.803 | 14.09769 | 16.3377 | 15.86374 |
| AABR07035 | 0.037205 | 0 | 0 | 0 | 0.020115 | 0 | 0 | 0 |
| Cdk5rap1 | 9.400046 | 8.490309 | 8.83552 | 8.773297 | 10.31681 | 8.542641 | 8.146789 | 8.51052 |
| Ngb | 0.116111 | 0.328434 | 0.124652 | 0.267479 | 0.188323 | 0.203612 | 0.584548 | 0.411659 |
| RGD156221 | 7.88879 | 6.535748 | 7.02727 | 6.944799 | 7.077814 | 7.820097 | 7.293908 | 8.390672 |
| Zfp52 | 1.143624 | 0.976142 | 0.883121 | 0.998341 | 0.954556 | 1.231423 | 1.72836 | 1.600501 |
| Fxyd4 | 0.076896 | 0.043502 | 0.041276 | 0.035428 | 0.041573 | 0 | 0.043014 | 0 |
| AABR07030 | 0 | 0 | 0 | 0 | 0 | 0.433156 | 1.105371 | 0 |
| LOC102552 | 0.986815 | 1.098231 | 1.406757 | 0.655897 | 1.644274 | 0.836872 | 1.683155 | 0.960696 |

| | | | | | | | | |
|---|---|---|---|---|---|---|---|---|
| Mybpc3 | 760.2425 | 784.8968 | 800.9705 | 850.9466 | 828.1297 | 867.9106 | 824.7708 | 817.8316 |
| Sgsm1 | 0.391347 | 0.432248 | 0.480155 | 0.540916 | 0.513834 | 0.449341 | 0.635883 | 0.677223 |
| Abcg4 | 0.563074 | 0.515741 | 0.460569 | 0.592972 | 0.550859 | 0.658272 | 0.749932 | 0.712973 |
| LOC100362 | 0.060609 | 0 | 0 | 0 | 0 | 0 | 0 | 0 |
| Dusp1 | 141.626 | 148.43 | 147.3694 | 139.1515 | 134.303 | 133.0714 | 177.8117 | 176.38 |
| Tmem200b | 2.266128 | 2.356123 | 3.550621 | 4.458477 | 3.576159 | 4.081288 | 5.550126 | 4.994311 |
| Appl2 | 8.939661 | 9.85494 | 9.312793 | 9.24689 | 9.265155 | 8.550876 | 10.71285 | 10.24126 |
| AABR07032 | 0.156316 | 0.088432 | 0 | 0 | 0 | 0 | 0.08744 | 0 |
| AABR07027 | 0.02586 | 0.029259 | 0 | 0 | 0 | 0 | 0.028931 | 0 |
| Tmod4 | 16.49136 | 16.89055 | 16.44591 | 14.58397 | 12.38089 | 13.67155 | 12.41645 | 12.53885 |
| Gsdma | 0.017995 | 0 | 0 | 0 | 0.058374 | 0.015778 | 0 | 0 |
| Med14 | 3.31227 | 2.804714 | 3.062691 | 3.475486 | 3.535297 | 3.297674 | 4.088136 | 3.674522 |
| Nynrin | 2.634763 | 2.161305 | 1.909296 | 2.384477 | 2.28932 | 2.26891 | 3.242422 | 3.286131 |
| Rfx5 | 4.941838 | 4.527093 | 4.228144 | 5.6748 | 5.940278 | 5.190811 | 5.865483 | 6.170074 |
| AABR07035 | 0 | 0 | 0 | 0 | 0 | 0.038103 | 0 | 0 |
| LOC100911 | 0.028367 | 0 | 0 | 0.026139 | 0 | 0 | 0 | 0 |
| Ftsj1 | 3.73816 | 3.476835 | 4.353251 | 4.728982 | 4.590089 | 5.749727 | 4.607384 | 4.464554 |
| Abt1 | 4.745788 | 4.176384 | 5.661008 | 5.905496 | 5.701725 | 4.872412 | 5.581653 | 5.356904 |
| RGD130545 | 2.322828 | 2.532848 | 2.493704 | 2.306746 | 2.836982 | 2.553736 | 2.679464 | 2.506875 |
| Chmp2a | 64.92917 | 62.15713 | 60.63224 | 75.87898 | 73.97358 | 71.50042 | 68.9983 | 64.93983 |
| Kctd4 | 0.27776 | 0.351246 | 0.298193 | 0.331223 | 0.353339 | 0.272192 | 0.365583 | 0.202747 |
| Dusp9 | 0 | 0 | 0 | 0 | 0.021879 | 0 | 0.022637 | 0 |
| Adgrd1 | 1.100785 | 0.956356 | 1.076249 | 1.811304 | 1.806649 | 1.378813 | 2.13315 | 2.421779 |
| Slc8a3 | 0.047627 | 0.053888 | 0.025565 | 0.087773 | 0.115871 | 0.041759 | 0.133208 | 0.084428 |
| Wwc2 | 3.867937 | 3.590883 | 3.865818 | 3.557122 | 3.522409 | 3.665667 | 4.745495 | 4.645509 |
| Pts | 4.47238 | 3.979449 | 4.102154 | 3.60098 | 3.568251 | 4.035602 | 4.614878 | 4.156524 |
| Rn60_3_17 | 0 | 0 | 0 | 0 | 0 | 0 | 0.151774 | 0 |
| Cyp4f18 | 0.146171 | 0.103366 | 0.117693 | 0.101018 | 0.276593 | 0.112143 | 0.204413 | 0.113364 |
| Alad | 38.96206 | 42.7985 | 40.7917 | 36.16368 | 30.89059 | 32.26962 | 37.4891 | 31.56389 |
| AABR07061 | 0 | 0 | 0 | 0 | 0 | 0 | 0 | 0 |
| Tmem253 | 1.197913 | 1.064943 | 1.102317 | 1.41921 | 1.387807 | 0.900284 | 0.765813 | 1.137611 |
| Id4 | 5.34609 | 5.028562 | 5.013305 | 8.457653 | 8.322743 | 7.624177 | 9.583961 | 9.448466 |
| LOC103690 | 0.766501 | 0.551892 | 0.598463 | 0.995241 | 0.414403 | 9.317311 | 0.935484 | 0.772031 |
| Trub2 | 23.12997 | 24.75214 | 24.31056 | 26.12004 | 25.49106 | 24.81864 | 25.65058 | 25.01725 |
| Cep95 | 2.963439 | 3.393148 | 2.914734 | 3.858943 | 3.856701 | 4.107565 | 3.811676 | 4.262397 |
| Tapbpl | 9.634663 | 11.46665 | 10.07515 | 10.82242 | 9.4571 | 10.71176 | 12.95324 | 12.05892 |
| Angptl3 | 0.064371 | 0.036416 | 0 | 0 | 0 | 0 | 0 | 0 |
| AABR07025 | 0 | 0.01155 | 0 | 0 | 0 | 0 | 0 | 0.009048 |
| Prss16 | 0.050767 | 0 | 0.054501 | 0 | 0.109787 | 0.111281 | 0.141989 | 0.15749 |
| Pomgnt2 | 6.28983 | 6.949197 | 8.261925 | 7.159556 | 5.254185 | 6.163744 | 7.533497 | 7.455182 |
| Rps3 | 444.3012 | 458.2504 | 444.8809 | 449.3685 | 440.2418 | 438.2592 | 470.9222 | 486.75 |
| AABR07015 | 4.841618 | 63.57436 | 59.50097 | 44.02658 | 47.11654 | 46.91991 | 1.995585 | 1.355168 |
| Ginm1 | 51.02321 | 51.4161 | 47.20884 | 48.91043 | 48.18358 | 44.59003 | 44.22016 | 48.42284 |
| RGD130459 | 3.34706 | 3.258617 | 3.71863 | 3.514535 | 4.292454 | 3.770755 | 3.592148 | 4.053293 |
| Gramd1a | 9.801293 | 9.267543 | 10.7454 | 11.90372 | 11.9087 | 12.23779 | 13.09631 | 14.50454 |
| Morc3 | 13.58326 | 12.97044 | 12.05195 | 13.73527 | 13.60554 | 14.40775 | 15.38606 | 15.91741 |
| Sdc4 | 7.008783 | 7.88248 | 7.095057 | 5.93467 | 6.645407 | 5.868491 | 9.065553 | 9.290366 |
| Sdc1 | 1.400067 | 1.680702 | 1.814672 | 1.950893 | 2.067728 | 1.751547 | 2.655125 | 2.648367 |
| Pdcl3 | 11.22712 | 13.01204 | 12.05303 | 11.67942 | 12.40556 | 11.76005 | 13.44666 | 14.23669 |
| Bcl7a | 11.33354 | 8.704914 | 9.325287 | 6.555718 | 7.513888 | 7.253473 | 7.126413 | 7.039169 |
| Dcaf13 | 14.37702 | 12.66524 | 12.35523 | 10.9593 | 11.46067 | 12.63645 | 12.13176 | 12.83608 |
| Fancd2os | 0.040752 | 0 | 0 | 0 | 0.088128 | 0.035731 | 0 | 0 |
| Hdac6 | 0.392593 | 0.170847 | 0.308 | 0.306104 | 0.375524 | 0.887045 | 0.219608 | 0.361359 |
| LOC100910 | 0 | 0.043375 | 0 | 0 | 0 | 0 | 0 | 0 |

| | | | | | | | | |
|---|---|---|---|---|---|---|---|---|
| AABR07006 | 0.19491 | 0 | 0.06975 | 0.059867 | 0.070251 | 0.056966 | 0 | 0 |
| Atp8b2 | 5.168961 | 4.000986 | 4.44974 | 5.688884 | 5.954766 | 6.277239 | 7.750038 | 7.869915 |
| Spns1 | 13.70861 | 14.48022 | 15.5404 | 16.43036 | 15.49669 | 16.41154 | 15.89964 | 16.56903 |
| Dusp28 | 7.227182 | 8.282957 | 8.695254 | 7.176254 | 6.029405 | 6.937703 | 7.562684 | 7.48265 |
| AC120076. | 0 | 0 | 0.047163 | 0.02024 | 0.023751 | 0.077038 | 0.024574 | 0.019469 |
| LOC103693 | 0 | 0 | 0.165835 | 0 | 0.167028 | 0 | 0 | 0 |
| LOC103690 | 0.396712 | 0.44886 | 0.212948 | 1.279439 | 0.428958 | 3.565329 | 0.443823 | 0.879063 |
| Vamp3 | 28.67586 | 27.59054 | 28.64434 | 30.87867 | 31.75827 | 32.37594 | 31.06711 | 30.18417 |
| AABR07021 | 0.907126 | 0.653145 | 0.70826 | 0.683903 | 0.713354 | 0.795369 | 0.922593 | 0.58475 |
| Celf4 | 0 | 0.059848 | 0.056786 | 0 | 0.057194 | 0 | 0.059176 | 0.093767 |
| Tescl | 0 | 0 | 0 | 0 | 0 | 0.053327 | 0 | 0 |
| Acsl4 | 1.649987 | 1.519834 | 1.464783 | 2.046689 | 2.070021 | 1.752744 | 1.810433 | 1.996827 |
| Knstrn | 0.304256 | 0.267751 | 0.290346 | 0.311512 | 0.657976 | 0.237131 | 0.340389 | 0.419499 |
| AABR07068 | 1.745531 | 2.413872 | 2.29037 | 1.429723 | 1.258278 | 0.850268 | 3.471684 | 2.406679 |
| RGD156626 | 5.462786 | 3.814765 | 4.077764 | 4.207894 | 4.337829 | 4.191053 | 5.013358 | 4.047558 |
| Ptpn23 | 4.86025 | 5.430832 | 5.682312 | 5.526301 | 5.124751 | 5.770191 | 6.923438 | 6.716013 |
| LOC502618 | 0 | 0 | 0.026745 | 0.045912 | 0.026937 | 0 | 0 | 0.022081 |
| Clec2d | 0.892601 | 0.897721 | 1.277685 | 2.46749 | 0.965156 | 1.739185 | 2.10816 | 2.109751 |
| Fam178b | 0.061318 | 0.173447 | 0.032915 | 0.084754 | 0 | 0.026882 | 0.0686 | 0 |
| Dmrta2 | 0.524972 | 0.705352 | 0.457917 | 0.63491 | 0.496689 | 0.891823 | 1.504995 | 1.134189 |
| Nnt | 378.4397 | 373.005 | 369.7006 | 318.8547 | 309.9952 | 320.9447 | 328.4266 | 332.0631 |
| RGD156001 | 0.887558 | 0.892649 | 0.95285 | 0.363489 | 1.279605 | 0.43234 | 0.99296 | 0.699277 |
| AABR07005 | 0.215498 | 0 | 0 | 0 | 0 | 0 | 0 | 0 |
| Gem | 0.998366 | 1.566871 | 1.625002 | 3.175329 | 2.298331 | 2.259015 | 3.242694 | 2.940157 |
| Fstl1 | 32.82482 | 33.86976 | 34.84103 | 71.75669 | 74.9424 | 71.92882 | 96.37548 | 94.45061 |
| Snrnp35 | 13.67096 | 14.45112 | 14.37194 | 12.16136 | 11.71324 | 12.23557 | 10.90193 | 11.23676 |
| Cpm | 0.336295 | 0.326144 | 0.330088 | 0.363005 | 0.675314 | 0.362261 | 0.333234 | 0.451371 |
| Akr7a3 | 0.12478 | 0.235304 | 0.223265 | 0.076653 | 0.224871 | 0.145876 | 0.139598 | 0.147465 |
| Lrrc55 | 0.087666 | 0.09919 | 0.094115 | 0.053854 | 0.031597 | 0.102488 | 0 | 0 |
| Upk2 | 0 | 0 | 0 | 0.117404 | 0.137768 | 0 | 0 | 0 |
| Trim47 | 19.86951 | 23.20923 | 27.54639 | 25.24618 | 22.48929 | 23.66748 | 29.87871 | 30.21801 |
| Cfb | 0 | 0 | 0 | 0 | 0 | 0 | 0 | 0.014486 |
| Tigit | 0 | 0.163899 | 0.544298 | 0.06674 | 0.156632 | 0.063506 | 0.24309 | 0.128394 |
| LOC100361 | 0.164414 | 0.062009 | 0.235346 | 0.02525 | 0 | 0.072079 | 0.061313 | 0.097152 |
| Eif3m | 71.9981 | 67.32284 | 64.00821 | 56.98253 | 56.31739 | 56.51829 | 64.71824 | 62.45772 |
| Agbl5 | 1.994436 | 1.462284 | 1.884216 | 1.455533 | 1.690739 | 1.482918 | 1.731479 | 1.371788 |
| Hexb | 22.02154 | 21.47202 | 21.93795 | 24.58911 | 23.84659 | 23.68131 | 28.36846 | 27.12537 |
| Fam217a | 0.102477 | 0.193247 | 0.036672 | 0.031476 | 0.221615 | 0.059901 | 0.076431 | 0.121108 |
| 5S_rRNA | 0 | 0 | 0 | 0 | 0 | 0 | 0 | 0 |
| RGD156113 | 0.319304 | 0.361278 | 0.114265 | 0.098075 | 0.345259 | 0.093322 | 0.119075 | 0.377354 |
| Fsd1 | 1.09003 | 0.830602 | 1.134394 | 1.393888 | 1.38309 | 1.267817 | 1.455906 | 1.449221 |
| AABR07040 | 0 | 0 | 0 | 0 | 0 | 0.150047 | 0 | 0 |
| Rinl | 0.216388 | 0.263666 | 0.339525 | 0.276083 | 0.323969 | 0.248108 | 0.223463 | 0.29507 |
| AABR07035 | 0 | 0 | 0 | 0 | 0 | 0.135441 | 0 | 0 |
| Rn50_10_0 | 0 | 0.055555 | 0.131782 | 0.022622 | 0.026546 | 0.15068 | 0 | 0.087041 |
| Atp23 | 3.6816 | 4.329987 | 5.356587 | 4.731575 | 3.666583 | 4.459779 | 4.498175 | 5.753511 |
| Dmwd | 20.41942 | 21.92359 | 25.30996 | 20.66185 | 19.79414 | 21.29681 | 26.68243 | 24.03435 |
| RGD131134 | 9.93859 | 9.842853 | 10.17405 | 10.03128 | 11.06175 | 9.182897 | 10.49359 | 12.10439 |
| Zbtb7c | 0.181601 | 0.293533 | 0.306366 | 0.513967 | 0.378699 | 0.46631 | 0.478894 | 0.402405 |
| Mup4 | 0 | 0 | 0 | 0 | 0.210493 | 0 | 0 | 0 |
| Slc22a5 | 5.735569 | 5.203676 | 4.803993 | 5.203288 | 5.376163 | 4.515158 | 5.204877 | 5.193893 |
| AABR07045 | 0.044643 | 0 | 0 | 0 | 0.048272 | 0.039143 | 0.049944 | 0.079138 |
| Abhd13 | 2.614816 | 2.722308 | 2.401573 | 2.565194 | 2.139335 | 2.283956 | 3.270152 | 3.137188 |
| Csrnp3 | 0 | 0 | 0 | 0 | 0 | 0 | 0 | 0 |

| | | | | | | | | |
|---|---|---|---|---|---|---|---|---|
| LOC10036( | 0 | 0 | 0 | 0 | 0 | 0.055452 | 0 | 0 |
| Tm4sf20 | 0.953346 | 1.155713 | 0.767608 | 0.815723 | 0.809945 | 0.477654 | 0.457098 | 0.543213 |
| AABR0706 | 0 | 0 | 1.405454 | 0 | 0 | 2.295724 | 0 | 1.160363 |
| Grpel1 | 119.9715 | 116.1756 | 110.0576 | 114.5825 | 113.0113 | 113.1515 | 105.5008 | 104.2291 |
| #N/A | 0 | 0 | 0.940103 | 0 | 0 | 0 | 0 | 0 |
| AABR07044 | 0 | 0 | 0 | 0 | 0 | 0 | 0 | 0 |
| Rn60_12_( | 0 | 0 | 0 | 0.063241 | 0 | 0.120353 | 0 | 0 |
| Scn9a | 0.006865 | 0 | 0 | 0.018977 | 0.007423 | 0.036115 | 0.00768 | 0 |
| Zfp275 | 1.966359 | 2.072064 | 1.621765 | 2.231853 | 2.144446 | 2.034889 | 2.35094 | 2.438539 |
| Rn60_16_( | 0.130588 | 0.147755 | 0 | 0.120332 | 0.282407 | 0.229 | 0.292193 | 0 |
| Bscl2 | 8.681475 | 8.829963 | 9.518419 | 8.212394 | 9.536949 | 8.826594 | 9.247491 | 9.08644 |
| Sh2b1 | 14.44825 | 18.62478 | 19.85192 | 17.38703 | 16.12789 | 17.00128 | 19.21995 | 18.54032 |
| Tnrc6c | 7.302669 | 7.260476 | 7.917504 | 6.612528 | 6.117655 | 6.696193 | 8.099123 | 8.499448 |
| AABR07058 | 0.128917 | 0.350071 | 0.16608 | 0.261341 | 0.195154 | 0.180855 | 0.144226 | 0.137118 |
| AY172581. | 300.7291 | 1044.844 | 935.1682 | 865.0242 | 877.3426 | 828.6819 | 193.6538 | 203.5621 |
| 5S_rRNA | 3.27287 | 6.665577 | 2.108181 | 1.809493 | 0.707781 | 4.591449 | 8.055392 | 2.320726 |
| Cfap54 | 0.042678 | 0.048288 | 0.045818 | 0.039326 | 0.046147 | 0.13097 | 0.047746 | 0.113483 |
| Rabgap1 | 10.76556 | 9.652423 | 10.89612 | 11.50763 | 10.77126 | 10.84919 | 11.10948 | 11.30052 |
| AABR07063 | 0.581844 | 0 | 0 | 0 | 0 | 0 | 0 | 0 |
| Mtmr2 | 32.07961 | 32.17077 | 30.97078 | 29.19857 | 28.49842 | 31.8103 | 30.02819 | 30.23387 |
| Lipt2 | 2.929176 | 2.751431 | 3.737989 | 3.004681 | 2.181237 | 2.422928 | 2.256824 | 2.449315 |
| Dnttip1 | 15.14897 | 15.03901 | 14.15228 | 16.30808 | 15.59285 | 16.06049 | 14.13691 | 15.04114 |
| Ldlrad3 | 0.27189 | 0.415301 | 0.729727 | 0.964562 | 0.558581 | 0.548304 | 0.638774 | 0.67477 |
| Rsph10b | 0.01762 | 0.019936 | 0.075664 | 0.032472 | 0.019052 | 0.092694 | 0 | 0.031235 |
| Itga5 | 17.75318 | 18.7927 | 17.65212 | 18.51076 | 16.78706 | 17.93747 | 24.18034 | 23.23366 |
| Exosc10 | 15.32978 | 16.06555 | 15.06448 | 15.74846 | 16.15495 | 15.76533 | 17.35765 | 16.79138 |
| Pcdhga2 | 1.099038 | 0.877771 | 0.994807 | 0.734719 | 1.118469 | 1.133691 | 1.277773 | 0.955031 |
| AABR07058 | 3.871137 | 4.618919 | 4.08035 | 4.021095 | 4.185804 | 4.690189 | 4.803313 | 3.618336 |
| Sytl3 | 0.711493 | 0.536681 | 0.432839 | 0.546344 | 0.435952 | 0.457481 | 0.318395 | 0.315316 |
| Rgs1 | 1.341733 | 1.214486 | 0.905417 | 1.554274 | 2.238372 | 1.781455 | 3.559683 | 2.752252 |
| Rufy3 | 10.97956 | 9.750976 | 10.29752 | 10.00507 | 10.21364 | 9.41343 | 10.56757 | 10.46538 |
| AABR07003 | 1.150899 | 2.604377 | 3.706692 | 0.530254 | 3.733352 | 3.027329 | 7.72545 | 4.080397 |
| AC099294. | 8.34819 | 7.728206 | 9.777072 | 13.98642 | 18.05355 | 21.9591 | 15.28296 | 14.12616 |
| Ipp | 4.776621 | 5.576094 | 4.286906 | 4.145298 | 4.836962 | 4.298943 | 4.156345 | 4.32336 |
| Syt12 | 0.147676 | 0.200506 | 0.063416 | 0.12247 | 0.15968 | 0.116534 | 0.214777 | 0.17016 |
| Dpp3 | 5.863056 | 6.452024 | 6.488369 | 6.716215 | 5.861993 | 6.3731 | 6.604253 | 6.940821 |
| Mccc1 | 37.51652 | 35.79011 | 34.39229 | 38.01525 | 40.91063 | 38.10995 | 35.91133 | 37.22576 |
| Impact | 7.167011 | 7.174822 | 6.657194 | 8.355646 | 8.187605 | 8.387829 | 7.530073 | 7.609164 |
| Bivm | 3.643752 | 3.41598 | 2.682377 | 3.901181 | 3.677274 | 3.377407 | 4.387056 | 4.090875 |
| Wdr36 | 4.571472 | 4.506431 | 4.402246 | 6.761586 | 6.640257 | 6.33066 | 6.233812 | 5.008386 |
| AABR0705( | 0.476054 | 0.538633 | 0 | 0 | 0 | 0 | 0 | 0.42195 |
| Btn1a1 | 0.052401 | 0 | 0.056256 | 0.06438 | 0.05666 | 0.12252 | 0.214954 | 0.123855 |
| Aldh16a1 | 9.850625 | 9.429109 | 11.16749 | 9.80312 | 9.905744 | 10.51991 | 10.31513 | 10.68686 |
| LOC100911 | 0 | 0 | 0 | 0 | 0 | 0.027363 | 0 | 0 |
| AABR07041 | 0 | 0.897721 | 0 | 1.462216 | 0.857917 | 0 | 0 | 1.406501 |
| Rrn3 | 5.67589 | 6.707914 | 4.695281 | 5.785361 | 4.876178 | 6.033307 | 9.046481 | 5.892266 |
| Slc29a4 | 0.02181 | 0.074031 | 0.046829 | 0.040194 | 0 | 0.019123 | 0.0488 | 0 |
| Amer2 | 0 | 0 | 0 | 0 | 0 | 0 | 0 | 0 |
| Fyttd1 | 57.36108 | 48.22859 | 49.60426 | 54.31354 | 57.43269 | 53.57907 | 58.11897 | 58.14728 |
| Crtc3 | 2.336112 | 2.56469 | 2.507966 | 2.706779 | 2.851134 | 2.251108 | 3.389837 | 2.76765 |
| AABR07071 | 0.117325 | 0 | 0 | 0 | 0.169149 | 0.137161 | 0.131258 | 0.173318 |
| Man2b1 | 13.35439 | 14.4677 | 14.53393 | 17.1201 | 16.87665 | 17.02217 | 18.86213 | 18.86468 |
| Pkdcc | 22.80952 | 25.77596 | 27.36658 | 22.70889 | 21.42803 | 22.69735 | 31.8031 | 29.70029 |
| Slbp | 7.589264 | 7.370418 | 6.993322 | 9.149447 | 8.753239 | 9.454613 | 8.773549 | 8.576596 |

| | | | | | | | | |
|---|---|---|---|---|---|---|---|---|
| Klhdc8a | 0.376498 | 0.314862 | 0.3339 | 0.286593 | 0.460202 | 0.315761 | 0.384582 | 0.246654 |
| AABR07039 | 0 | 0.466532 | 0.442663 | 0 | 0 | 0.361531 | 0 | 0.730937 |
| Uts2b | 0 | 0 | 0 | 0.064596 | 0 | 0 | 0 | 0 |
| AABR07030 | 0 | 0 | 0.219602 | 0 | 0 | 0 | 0 | 0.181307 |
| Rhoq | 14.3433 | 14.06391 | 13.08212 | 17.70574 | 17.75414 | 18.22778 | 19.3258 | 20.01393 |
| Adnp | 7.499571 | 7.256563 | 7.99096 | 6.979579 | 6.610853 | 7.371931 | 8.138796 | 8.066317 |
| Zdhhc15 | 0.871857 | 0.554887 | 0.818995 | 0.820119 | 0.785605 | 1.051112 | 1.137961 | 0.869367 |
| Grina | 43.59396 | 45.91591 | 50.54244 | 52.19939 | 48.54271 | 54.27905 | 60.36897 | 57.4095 |
| Eif2s3 | 51.60973 | 44.13416 | 45.8337 | 53.18877 | 55.4301 | 50.52803 | 55.41221 | 53.81515 |
| Tnni3k | 67.40683 | 71.9151 | 69.50068 | 86.22334 | 82.96641 | 84.36901 | 67.77366 | 72.75499 |
| Pa2g4 | 35.714 | 35.54975 | 33.44863 | 34.96838 | 32.26772 | 31.81355 | 37.16082 | 35.84987 |
| AABR07064 | 0 | 0 | 0 | 0 | 0 | 0.24039 | 0 | 0 |
| Lmo7 | 47.82062 | 47.58667 | 46.38688 | 44.58964 | 43.60501 | 43.47629 | 45.2535 | 44.20713 |
| Lman1 | 30.90448 | 31.72928 | 30.48137 | 31.14334 | 31.52571 | 31.16721 | 31.90677 | 33.84568 |
| LOC100364 | 0.11983 | 0.180777 | 0.042882 | 0 | 0 | 0.175112 | 0.134061 | 0.17702 |
| AABR07049 | 0.050303 | 0 | 0 | 0 | 0.054392 | 0 | 0 | 0.044586 |
| AABR07051 | 0 | 0.143461 | 0 | 0.116836 | 0.137101 | 0.333519 | 0 | 0 |
| Myod1 | 0.026882 | 0 | 0 | 0 | 0 | 0.02357 | 0 | 0 |
| LOC301124 | 140.0156 | 141.1774 | 147.3691 | 141.5647 | 133.3948 | 131.9487 | 120.6036 | 124.775 |
| Rdh11 | 4.636758 | 4.496805 | 4.938349 | 5.323782 | 5.451359 | 6.227334 | 5.352078 | 4.859988 |
| Pcdh18 | 1.334937 | 1.064158 | 0.977141 | 1.472382 | 2.044884 | 1.649304 | 1.719751 | 2.187165 |
| Dnaaf1 | 0.069821 | 0.105333 | 0.149915 | 0.150121 | 0.050331 | 0.183658 | 0.104151 | 0.226915 |
| #N/A | 3.156811 | 2.521259 | 3.987104 | 4.448871 | 4.015781 | 3.907616 | 4.570435 | 3.785581 |
| #N/A | 0 | 0 | 0 | 0 | 0 | 0 | 0 | 0.149243 |
| Lap3 | 25.87154 | 27.26673 | 25.64021 | 33.75071 | 31.96349 | 29.44744 | 30.44473 | 31.8914 |
| AC142360. | 0.122925 | 0.278167 | 0 | 0.11327 | 0.265833 | 0.10778 | 0 | 0 |
| Stoml2 | 39.41608 | 41.57544 | 40.36064 | 46.05303 | 45.28926 | 48.46923 | 39.6601 | 40.532 |
| Guf1 | 6.62603 | 5.95747 | 5.961158 | 6.175716 | 6.068003 | 6.157995 | 5.767696 | 6.037783 |
| Rad51ap1 | 0.873206 | 0.778418 | 0.994258 | 0.975303 | 0.915574 | 0.858432 | 1.21373 | 1.102315 |
| Kir3dl1 | 0.284819 | 0.368296 | 0.305771 | 0.187464 | 0.087991 | 0.178378 | 0.182081 | 0.180321 |
| Omd | 0 | 0 | 0 | 0 | 0.029708 | 0 | 0 | 0 |
| Wrap53 | 2.975008 | 3.071958 | 3.503945 | 2.980889 | 2.873288 | 3.317594 | 3.586816 | 4.300959 |
| Etv2 | 0 | 0 | 0 | 0.025694 | 0 | 0 | 0 | 0 |
| AABR07049 | 0 | 0 | 0.351364 | 0.301582 | 0.353891 | 0 | 0.732308 | 0.290091 |
| Pidd1 | 0.019014 | 0.064542 | 0.081653 | 0.052563 | 0.08224 | 0.033344 | 0.127635 | 0.202242 |
| Ccnjl | 0.525359 | 0.734282 | 1.028482 | 0.427145 | 0.601478 | 0.447087 | 0.691468 | 0.479347 |
| AABR07058 | 30.91157 | 29.96109 | 28.89231 | 42.87508 | 40.90378 | 42.26597 | 41.11205 | 45.98342 |
| Atp6v1g3 | 0 | 0 | 0.032704 | 0 | 0 | 0.02671 | 0 | 0 |
| Pdk1 | 136.8346 | 138.7243 | 135.5075 | 114.8419 | 116.329 | 115.1788 | 112.0863 | 111.5242 |
| Tchp | 3.25406 | 3.575405 | 3.574215 | 3.449129 | 3.152475 | 3.364424 | 4.987272 | 4.318017 |
| Cobl | 2.89506 | 2.756599 | 2.604618 | 2.066515 | 1.884845 | 2.279189 | 2.543193 | 2.340152 |
| Ccdc169 | 0 | 0 | 0 | 0 | 0 | 0 | 0 | 0 |
| Aff1 | 7.171282 | 7.328308 | 8.212941 | 4.688519 | 5.657073 | 5.385032 | 9.081025 | 8.839345 |
| Mt-nd4 | 3943.481 | 3633.659 | 3175.918 | 3202.846 | 3418.964 | 3700.814 | 2520.756 | 2389.809 |
| LOC499306 | 0 | 0.076156 | 0.03613 | 0.031011 | 0.07278 | 0 | 0.075302 | 0.059659 |
| Palm2 | 2.878755 | 2.791865 | 2.825625 | 2.88003 | 3.083111 | 2.235627 | 3.312641 | 3.013299 |
| Gprasp1 | 2.384006 | 2.130008 | 2.40935 | 2.636121 | 3.00446 | 2.465111 | 3.099377 | 2.674117 |
| Irf1 | 9.465262 | 10.62266 | 10.82069 | 12.70564 | 13.11142 | 13.12163 | 13.96645 | 12.60697 |
| AABR07001 | 0.297051 | 0.384114 | 0.136673 | 0.430133 | 0.183541 | 0.297663 | 0.094951 | 0.188065 |
| AY172581. | 24.64279 | 19.16898 | 42.99036 | 21.28815 | 35.80541 | 60.76917 | 18.95386 | 19.11186 |
| Dgcr8 | 3.404131 | 3.86467 | 3.790824 | 4.221352 | 3.743225 | 4.117936 | 4.46679 | 4.756005 |
| AABR07017 | 0 | 0.524333 | 0 | 0 | 0 | 0 | 0 | 0 |
| Polr1c | 16.92586 | 16.79206 | 17.63812 | 20.17027 | 20.23383 | 20.84648 | 19.99098 | 18.87377 |
| AC125873. | 0.844612 | 0.318546 | 0.906745 | 0.778276 | 0 | 0.246852 | 0 | 0.748621 |

| | | | | | | | | |
|---|---|---|---|---|---|---|---|---|
| Ccnt1 | 0.604627 | 0.803082 | 0.395108 | 0.436022 | 0.397949 | 0.299643 | 0.47056 | 0.233005 |
| Pnmal1 | 0 | 0 | 0 | 0 | 0 | 0 | 0 | 0 |
| Ano8 | 8.488332 | 9.727542 | 11.14216 | 9.429544 | 9.178311 | 9.849064 | 11.57052 | 11.34183 |
| Ccl28 | 0 | 0 | 0 | 0 | 0 | 0 | 0.038017 | 0 |
| Kif13a | 18.41712 | 19.35973 | 19.08729 | 15.22199 | 15.99525 | 14.70246 | 18.30716 | 19.10213 |
| Kmt2a | 4.963229 | 5.317665 | 5.138219 | 4.100596 | 4.257 | 4.116856 | 5.821889 | 5.823951 |
| Rasgrf2 | 2.282143 | 2.372775 | 2.229303 | 1.799783 | 1.778485 | 1.496232 | 2.438153 | 1.767651 |
| Slc35d1 | 4.904239 | 4.478877 | 3.829101 | 4.021095 | 3.68134 | 3.897282 | 4.322811 | 4.310946 |
| Ubald1 | 1.775116 | 2.454785 | 2.964423 | 2.09006 | 2.026041 | 24.03809 | 2.647895 | 1.923012 |
| Map3k14 | 1.257479 | 1.408692 | 1.577209 | 1.457001 | 1.655865 | 1.386386 | 1.685389 | 1.732544 |
| AABR07032 | 0.409109 | 0 | 0 | 0 | 0 | 0 | 0 | 0 |
| Tsfm | 16.58098 | 16.69375 | 14.83395 | 15.58084 | 13.62384 | 14.20967 | 17.76404 | 14.90413 |
| Stx1b | 0.104977 | 0.059388 | 0 | 0.257952 | 0.208101 | 0.122725 | 0.078296 | 0.062031 |
| LOC687707 | 0.38112 | 0.43122 | 0.409157 | 0.702375 | 0.57694 | 0.334167 | 1.023313 | 0.472928 |
| Lmbr1 | 1.840336 | 1.490704 | 1.706302 | 1.637986 | 2.057767 | 1.631945 | 1.333597 | 1.334603 |
| G4 | 12.24728 | 11.06296 | 11.79553 | 11.42471 | 12.58884 | 12.06415 | 10.71327 | 13.44636 |
| AABR07027 | 0 | 0 | 0.401558 | 0 | 0 | 0 | 0 | 0 |
| Cd4 | 10.77858 | 11.04366 | 10.06077 | 13.65655 | 12.65832 | 13.65096 | 19.39424 | 19.61139 |
| Pcdhb10 | 0.542473 | 0.417894 | 0.532815 | 0.56368 | 0.76129 | 0.678041 | 0.555244 | 0.501281 |
| Parvg | 0.64012 | 0.841082 | 0.886722 | 0.799145 | 0.669825 | 0.742308 | 1.201263 | 1.500785 |
| Sdhaf3 | 37.55091 | 37.1762 | 32.28214 | 27.70838 | 33.94179 | 27.13713 | 31.17951 | 32.37315 |
| AABR07052 | 0.066454 | 0.15038 | 0.142686 | 0.061235 | 0.143712 | 0.058267 | 0 | 0.058902 |
| AABR07028 | 0.273309 | 0.092771 | 0.176049 | 0.075553 | 0.177315 | 0.047927 | 0.030577 | 0.048449 |
| Bank1 | 0.224961 | 0.141407 | 0.107338 | 0.161228 | 0.10811 | 0.219162 | 0.055928 | 0.132929 |
| RGD156623 | 26.62524 | 26.42211 | 28.20404 | 24.61562 | 25.82446 | 25.36155 | 24.04742 | 24.14809 |
| Srsf5 | 106.6845 | 99.66984 | 100.7639 | 158.4993 | 164.2053 | 157.3155 | 111.3109 | 114.3056 |
| Gprin3 | 7.195619 | 5.725893 | 5.602716 | 3.005575 | 2.778757 | 3.674545 | 3.339481 | 3.644477 |
| Lilrb3l | 1.246808 | 1.612233 | 1.266821 | 2.051579 | 1.685194 | 2.12784 | 2.640295 | 2.032609 |
| Rn50_X_06 | 0 | 0 | 0 | 0 | 0 | 0.049799 | 0 | 0 |
| Rab27b | 0.825589 | 0.978597 | 0.928528 | 0.760748 | 0.72266 | 0.206822 | 0.483807 | 0.522686 |
| Ndufb4 | 630.1212 | 638.3032 | 642.9178 | 580.6319 | 593.1645 | 573.6277 | 557.3286 | 539.3899 |
| Cdc37 | 81.07453 | 84.72431 | 85.56715 | 75.38529 | 72.4318 | 76.82831 | 83.57473 | 80.02736 |
| Vps36 | 16.12705 | 14.84429 | 15.53768 | 15.1722 | 14.59259 | 15.45865 | 15.05622 | 16.49331 |
| Decr1 | 142.2653 | 135.9466 | 135.5935 | 107.5487 | 105.1282 | 104.7945 | 102.4979 | 103.0098 |
| Ly49s5 | 0.042923 | 0 | 0.04608 | 0.118655 | 0.092824 | 0.112904 | 0.04802 | 0.114134 |
| Dnase2 | 8.62368 | 7.847351 | 6.973101 | 10.61772 | 10.396 | 10.23182 | 10.6331 | 9.237367 |
| Fut8 | 5.631143 | 5.784536 | 4.55393 | 4.608541 | 5.608172 | 5.018598 | 6.465658 | 6.419553 |
| Tmtc1 | 11.14459 | 10.52726 | 11.90954 | 9.248648 | 9.120776 | 9.38311 | 15.65181 | 16.3425 |
| Ttc23 | 8.519401 | 9.382254 | 7.438846 | 8.617877 | 10.23545 | 9.295774 | 9.785291 | 9.128524 |
| Ptdss1 | 6.916705 | 7.446816 | 7.065811 | 8.402397 | 7.246025 | 7.638424 | 7.764878 | 8.103449 |
| Gnat2 | 0.61809 | 0.660487 | 0.516101 | 0.885959 | 0.594072 | 0.84302 | 0.80674 | 0.821765 |
| Gba2 | 19.15294 | 18.70714 | 19.2536 | 20.68231 | 18.25137 | 19.95103 | 22.18037 | 22.24801 |
| Fn3k | 17.58384 | 15.61955 | 17.05588 | 14.78152 | 15.76091 | 14.80894 | 17.42872 | 15.44872 |
| Gdap1l1 | 0 | 0 | 0.09915 | 0 | 0.049932 | 0 | 0.051662 | 0.04093 |
| Uggt1 | 4.941581 | 4.635632 | 4.361701 | 4.090764 | 4.269672 | 4.087625 | 5.67525 | 5.735432 |
| Pkd1l3 | 0.072163 | 0.081648 | 0.060255 | 0.081272 | 0.043349 | 0.098423 | 0.053821 | 0.106602 |
| Sart1 | 14.15295 | 14.96046 | 15.31898 | 13.80958 | 12.4733 | 12.44332 | 15.70355 | 16.27344 |
| Mb21d1 | 0.522053 | 0.454368 | 0.732905 | 0.666071 | 0.60791 | 0.950683 | 0.988392 | 1.032225 |
| Sp3 | 8.178361 | 11.57369 | 7.836471 | 8.165915 | 12.53878 | 8.904627 | 9.204211 | 8.417365 |
| Myo3a | 0 | 0 | 0 | 0 | 0 | 0 | 0.01204 | 0 |
| Dmc1 | 0 | 0 | 0 | 0.018638 | 0.02187 | 0 | 0 | 0 |
| Taf6 | 14.16107 | 14.80532 | 13.67178 | 14.06326 | 13.6078 | 15.13664 | 15.143 | 15.30149 |
| Cyp1a2 | 0 | 0 | 0 | 0 | 0.029082 | 0 | 0 | 0 |
| Hsd3b1 | 0 | 0 | 0 | 0 | 0 | 0 | 0.048861 | 0 |

| | | | | | | | | |
|---|---|---|---|---|---|---|---|---|
| AC107527. | 0 | 0 | 0 | 0 | 0 | 0 | 0 | 0 |
| AABR07052 | 15.02674 | 16.63404 | 12.29118 | 14.2062 | 14.63041 | 13.11842 | 12.51747 | 13.26129 |
| Actl11 | 0 | 0.016057 | 0 | 0 | 0 | 0.012443 | 0.031753 | 0.012578 |
| LOC102555 | 2.104496 | 2.445493 | 2.625685 | 2.742847 | 2.193559 | 2.244191 | 2.121096 | 2.016561 |
| Cops7a | 50.20291 | 53.01278 | 53.97093 | 49.57118 | 49.90778 | 55.12185 | 54.64259 | 53.58851 |
| Ccr2 | 2.929562 | 2.27883 | 1.96567 | 2.868193 | 0.989904 | 1.926482 | 2.867781 | 1.460597 |
| Mrpl46 | 16.11005 | 15.60775 | 15.3064 | 17.3443 | 17.59841 | 16.35867 | 16.06175 | 14.63098 |
| Cyp2c11 | 0 | 0 | 0 | 0.028368 | 0 | 0 | 0 | 0 |
| Ccdc186 | 9.258902 | 9.123576 | 8.391986 | 9.231035 | 9.047293 | 9.648696 | 8.999963 | 8.324343 |
| Rbl1 | 0.488749 | 0.572746 | 0.524703 | 0.562953 | 0.585099 | 0.44384 | 0.624903 | 0.696218 |
| Scn3a | 0.644351 | 0.486035 | 0.381351 | 0.593744 | 0.607404 | 0.514265 | 0.693146 | 0.78346 |
| AABR07030 | 1.051525 | 0.4759 | 1.354654 | 0.968938 | 0.682199 | 1.106373 | 1.411679 | 1.118422 |
| LOC100910 | 7.44597 | 8.286654 | 7.86268 | 4.94904 | 6.863335 | 5.244312 | 6.145245 | 7.140695 |
| AC119699. | 0 | 0 | 0 | 0 | 0.129275 | 0.104828 | 0.26751 | 0.211938 |
| Rfxapl1 | 0.348473 | 0.430124 | 0.510147 | 0.613016 | 3.185659 | 0.277765 | 0.318973 | 0.140395 |
| Ebf4 | 0.715636 | 0.598481 | 1.03551 | 0.94614 | 0.874739 | 0.736596 | 0.835432 | 0.52399 |
| AABR07000 | 1.163687 | 1.316657 | 0.832862 | 0.714861 | 0.419426 | 0.680215 | 0.867921 | 1.375245 |
| Sytl1 | 0.395513 | 0.447504 | 0.396301 | 0.267263 | 0.741282 | 0.786049 | 0.50148 | 0.514159 |
| Trabd2b | 12.35714 | 13.29391 | 14.02736 | 8.55552 | 8.104576 | 7.104756 | 9.858541 | 9.965206 |
| Bcl2l1 | 31.88224 | 35.5552 | 35.39183 | 27.69059 | 27.93343 | 27.91212 | 40.95265 | 43.83 |
| Abca4 | 0.029242 | 0.057901 | 0.102029 | 0.222303 | 0.181812 | 0.192299 | 0.237185 | 0.252711 |
| Nae1 | 8.276054 | 8.197635 | 8.853254 | 8.847313 | 9.649393 | 9.115756 | 8.995283 | 8.536303 |
| Amd1 | 39.2001 | 37.17584 | 36.85216 | 32.72689 | 33.06255 | 33.84926 | 36.25966 | 35.56494 |
| AABR07017 | 0 | 0 | 0 | 0 | 0 | 0 | 0 | 0 |
| Doc2b | 1.911165 | 1.874074 | 2.325325 | 1.761063 | 1.148064 | 2.159805 | 2.328182 | 2.446832 |
| RGD130762 | 1.201433 | 1.453115 | 2.045914 | 1.717873 | 1.612666 | 1.598289 | 2.271083 | 2.09306 |
| H2afz | 113.3231 | 106.3098 | 107.1659 | 91.98254 | 94.81322 | 87.28535 | 90.16546 | 88.84029 |
| AABR07052 | 0 | 0 | 0 | 0.217356 | 0 | 0 | 0.263895 | 0.418149 |
| AABR07012 | 0 | 0 | 0 | 0 | 0 | 0 | 0 | 0 |
| LOC103690 | 3.173692 | 3.270269 | 3.224635 | 3.707763 | 3.983185 | 3.776516 | 5.579492 | 6.479949 |
| Adra1a | 16.4109 | 16.21445 | 16.37743 | 11.85621 | 11.96876 | 11.03389 | 12.41225 | 11.81419 |
| AABR07003 | 0 | 0 | 0 | 0 | 0 | 0 | 0 | 0 |
| Mettl5 | 17.4876 | 15.07274 | 13.52148 | 14.55182 | 13.67112 | 13.50676 | 15.44554 | 13.99735 |
| Wdr76 | 1.484962 | 1.902073 | 1.534043 | 1.52324 | 1.514781 | 1.375715 | 1.535928 | 1.5397 |
| Vps29 | 44.77162 | 43.63689 | 40.76483 | 48.61904 | 48.84027 | 51.61572 | 45.03523 | 46.98645 |
| Pdcd6 | 25.00293 | 26.89644 | 24.19841 | 33.56603 | 30.5118 | 30.10983 | 26.32675 | 27.76989 |
| Aars2 | 6.778052 | 7.251579 | 7.716794 | 5.754615 | 5.511534 | 5.1522 | 7.170201 | 6.819252 |
| Elp2 | 12.53379 | 12.513 | 12.05545 | 11.16616 | 11.95818 | 12.28254 | 9.580814 | 10.47259 |
| Junb | 65.75844 | 67.05869 | 72.58228 | 94.61187 | 90.38007 | 88.99633 | 103.3364 | 103.476 |
| Dcaf7 | 8.48134 | 7.828229 | 8.094688 | 6.410043 | 6.677039 | 6.759656 | 8.087906 | 8.65214 |
| AC115415. | 0.385044 | 0.871317 | 0 | 0 | 0 | 0 | 0 | 0 |
| Bub1 | 0.990675 | 0.830786 | 0.675669 | 0.730295 | 0.781348 | 0.837967 | 0.730189 | 0.981389 |
| Lmo1 | 1.32572 | 1.730757 | 1.477985 | 2.866058 | 2.370758 | 2.324784 | 3.080401 | 2.350102 |
| Lrrc8e | 0 | 0.01977 | 0.037516 | 0.0161 | 0.037786 | 0.03064 | 0 | 0.123896 |
| Zfp451 | 4.168498 | 3.902757 | 4.046221 | 3.890194 | 4.680142 | 4.168736 | 4.797625 | 4.934198 |
| AC107096. | 0 | 0 | 0 | 0 | 0 | 0 | 0 | 0 |
| Snx24 | 5.430363 | 5.656995 | 5.752795 | 5.2243 | 5.225102 | 5.705226 | 5.111773 | 5.555324 |
| Trim16 | 5.583611 | 5.25482 | 5.308092 | 5.024866 | 5.007721 | 5.524837 | 6.903475 | 6.926332 |
| Gabrp | 0.64462 | 0.729358 | 0.732749 | 0.663874 | 0.492013 | 0.598451 | 0.593907 | 0.436922 |
| Vsig4 | 0.148345 | 0.125884 | 0.159258 | 0.341736 | 0.44111 | 0.227621 | 0.124472 | 0.131486 |
| Zfp879 | 0.444721 | 0.478022 | 0.429693 | 0.717138 | 0.625132 | 0.506912 | 0.621918 | 0.55185 |
| Sytl2 | 0.435742 | 0.145006 | 0.137587 | 1.110082 | 0.637454 | 0.247215 | 0.114703 | 0.204469 |
| Qtrt1 | 0.902389 | 1.825444 | 1.232983 | 1.360663 | 1.419259 | 1.870146 | 1.743773 | 1.405766 |
| Mak | 0 | 0 | 0 | 0 | 0.052477 | 0 | 0.036197 | 0.014339 |

| | | | | | | | | |
|---|---|---|---|---|---|---|---|---|
| Trub2-ps2 | 0 | 0.126873 | 0.120382 | 0 | 0.121247 | 0 | 0.062724 | 0.149083 |
| Atrip | 1.760698 | 1.76831 | 1.210591 | 1.786478 | 1.796861 | 1.821315 | 1.859128 | 2.034033 |
| LOC688321 | 0 | 0 | 0 | 0.065119 | 0 | 0.061963 | 0 | 0 |
| Lrrc4b | 42.11788 | 47.45676 | 50.52967 | 36.93207 | 35.27889 | 39.63485 | 40.0191 | 39.91167 |
| AABR0707( | 0 | 0 | 0 | 0.043354 | 0 | 0 | 0 | 0 |
| Nrk | 0.083977 | 0.135738 | 0.051517 | 0.066327 | 0.103776 | 0.021038 | 0.080529 | 0.106333 |
| Kcne2 | 0.39648 | 0.310569 | 0.360163 | 0.309135 | 0.263821 | 0.374376 | 0.477685 | 0.919099 |
| Prkag3 | 2.138944 | 2.009559 | 1.824732 | 2.111735 | 2.003058 | 1.908917 | 2.07247 | 2.302106 |
| AC112030. | 0 | 0 | 0.525403 | 0 | 0 | 0 | 0 | 0 |
| RGD15660( | 0.078983 | 0.089366 | 0.084794 | 0.14556 | 0.25621 | 0 | 0.088363 | 0.140014 |
| Smarcd2 | 16.49272 | 16.24474 | 15.98124 | 16.92139 | 15.19462 | 17.3851 | 22.88783 | 23.43257 |
| LOC68847: | 0 | 0 | 0 | 0 | 0 | 0 | 0.072327 | 0 |
| Hic1 | 2.776492 | 3.482404 | 1.0629 | 2.141939 | 2.187635 | 1.58521 | 2.07081 | 2.518174 |
| Kbtbd8 | 0.484382 | 0.488484 | 0.406968 | 0.630697 | 0.785633 | 0.47087 | 0.43588 | 0.643998 |
| Fam46a | 1.894764 | 2.067949 | 2.502185 | 2.858415 | 2.828406 | 3.499087 | 4.933643 | 5.276066 |
| Usp49 | 0.634679 | 0.519249 | 0.670886 | 0.593829 | 0.739059 | 0.590733 | 0.830213 | 0.744294 |
| Fzd6 | 3.987885 | 4.357001 | 4.308008 | 3.766546 | 3.880839 | 4.294239 | 4.489353 | 4.32996 |
| Xrn1 | 3.705225 | 3.280921 | 3.011324 | 3.370559 | 3.688763 | 3.165657 | 3.699973 | 3.695691 |
| Mir128-2 | 0.623404 | 0 | 0 | 0 | 0.674078 | 0 | 0 | 0 |
| Kidins22( | 15.37614 | 14.97411 | 14.30875 | 14.00339 | 14.23636 | 14.20753 | 17.22124 | 16.44966 |
| Igsf21 | 0.142299 | 0.161004 | 0.152767 | 0.218538 | 0.051289 | 0.207946 | 0.053066 | 0.126126 |
| Lemd3 | 5.068753 | 5.450201 | 6.108319 | 5.490341 | 4.210392 | 5.106515 | 5.595587 | 6.233232 |
| Nr1h4 | 0.075966 | 0.114603 | 0.108739 | 0.07 | 0.082141 | 0.111012 | 0.254962 | 0.314218 |
| Cox7b | 1373.893 | 1352.328 | 1330.889 | 976.2679 | 988.6433 | 958.0566 | 990.6692 | 968.0049 |
| Trappc13 | 25.59778 | 23.90355 | 21.84293 | 24.36161 | 25.30978 | 24.57543 | 23.23243 | 23.99192 |
| Evi2b | 1.811298 | 0.731928 | 0.972272 | 1.25178 | 1.468898 | 1.361269 | 1.085571 | 1.003402 |
| Col16a1 | 2.218133 | 2.181868 | 2.338401 | 4.428498 | 4.645617 | 4.231386 | 5.108413 | 5.295913 |
| Impa2 | 26.09205 | 26.23028 | 28.10908 | 17.38286 | 18.43181 | 20.20716 | 21.35899 | 21.31361 |
| Gfm1 | 52.75801 | 52.02823 | 51.42418 | 56.08122 | 56.175 | 54.10804 | 48.34941 | 48.55762 |
| Mavs | 30.01701 | 30.20567 | 32.31899 | 31.78627 | 31.63018 | 31.72065 | 42.86921 | 43.10334 |
| Egr1 | 38.7809 | 37.65093 | 39.69397 | 50.11851 | 47.83065 | 52.17822 | 60.15691 | 57.96625 |
| AABR0705 | 0.060539 | 0.068497 | 0 | 0.111568 | 0 | 0 | 0.067728 | 0.053658 |
| Etfdh | 163.9404 | 158.8857 | 155.351 | 137.6742 | 138.3145 | 136.3003 | 123.8249 | 125.6339 |
| Atmin | 0.064411 | 0.170048 | 0.092199 | 0.059352 | 0.232155 | 0.150601 | 0.288239 | 0.152241 |
| Scn8a | 0.04102 | 0.055694 | 0.03523 | 0.022679 | 0.017742 | 0.043159 | 0.018356 | 0.072716 |
| AABR07008 | 3.293454 | 2.608472 | 2.12144 | 1.820873 | 2.492815 | 5.486637 | 4.421484 | 2.335322 |
| Unk1 | 2.827628 | 3.00092 | 3.294493 | 2.969113 | 3.152279 | 3.786167 | 3.678401 | 3.982828 |
| Pygo1 | 1.015196 | 1.354374 | 1.464015 | 1.298478 | 1.19602 | 0.996408 | 1.915529 | 1.181851 |
| Rn50_1_13 | 3.83572 | 0.094346 | 0.268558 | 0.153672 | 0.360653 | 0.073112 | 0.186575 | 0.295634 |
| Gimap5 | 12.51999 | 11.10183 | 11.28894 | 12.28203 | 12.39687 | 11.68676 | 12.47236 | 10.84772 |
| AABR0703( | 0 | 0 | 0 | 0 | 0.017148 | 0 | 0 | 0 |
| Capza1 | 10.52817 | 10.35678 | 10.9207 | 10.49111 | 9.565323 | 11.42899 | 14.14861 | 13.6606 |
| LOC10035( | 0 | 0 | 0 | 0.059134 | 0 | 0 | 0 | 0.056881 |
| Kctd2 | 6.625761 | 7.822682 | 7.147541 | 7.668591 | 6.229861 | 6.314645 | 6.839652 | 6.950829 |
| #N/A | 0.185039 | 0.209362 | 0.198651 | 0 | 0 | 0 | 0 | 0 |
| AABR0703( | 0 | 0 | 0 | 0 | 0 | 0 | 0 | 0 |
| AC097575. | 0.37139 | 0 | 0.39871 | 0.342221 | 0 | 0 | 0 | 0.658362 |
| Pgrmc2 | 42.47745 | 42.87244 | 45.57148 | 44.133 | 43.48187 | 43.67782 | 46.5599 | 47.35449 |
| AC127784. | 0 | 1.260629 | 1.794197 | 1.539994 | 1.204734 | 0.488452 | 0.623241 | 0.987543 |
| AABR07012 | 0 | 1.247359 | 0.59177 | 0.507928 | 0.596026 | 0.48331 | 1.850042 | 2.931443 |
| Snrpb | 48.38197 | 49.71416 | 53.1749 | 51.21821 | 47.26137 | 47.66105 | 52.32764 | 51.54151 |
| Schip1 | 63.27674 | 62.70891 | 63.00811 | 59.0804 | 56.65492 | 56.29584 | 67.97431 | 68.25352 |
| Tbc1d10a | 9.79705 | 10.85071 | 11.851 | 10.96662 | 11.11562 | 10.97955 | 14.62687 | 13.1783 |
| Nsun2 | 9.532841 | 8.248088 | 8.258297 | 8.360167 | 8.87739 | 9.316531 | 9.281535 | 8.487663 |

| | | | | | | | | |
|---|---|---|---|---|---|---|---|---|
| AABR0700( | 0.978802 | 0 | 0 | 0 | 0 | 0 | 0 | 0 |
| LOC10369( | 0.247132 | 0.235468 | 0.195493 | 0.227722 | 0.070321 | 0.55882 | 0.203722 | 0.09223 |
| Ppp1r36 | 3.134208 | 3.187603 | 2.911095 | 4.835049 | 4.493246 | 4.106676 | 4.924737 | 5.181446 |
| #N/A | 0 | 0 | 0 | 0 | 0 | 0 | 0.202715 | 0 |
| Sf3b6 | 31.3914 | 33.92516 | 30.37594 | 34.37387 | 34.70412 | 31.72049 | 34.17439 | 35.80905 |
| Hmgn5 | 0.320444 | 0.392781 | 0.229345 | 0.172245 | 0.375366 | 0.351207 | 0.448123 | 0.213019 |
| St6galnac | 10.00724 | 9.884914 | 10.36445 | 12.75041 | 11.35504 | 11.12656 | 11.92623 | 13.07804 |
| LOC100363 | 25.28397 | 18.81746 | 25.455 | 22.884 | 18.71216 | 19.50873 | 18.35485 | 27.98815 |
| Zfp764 | 1.943979 | 2.520284 | 2.739168 | 2.276443 | 1.66408 | 1.811012 | 2.265455 | 1.866632 |
| Fem1b | 5.040811 | 5.754364 | 5.290837 | 4.852271 | 5.280226 | 5.386616 | 6.570949 | 6.163337 |
| AABR07008 | 0 | 0.204309 | 0 | 0.16639 | 0.3905 | 0.316652 | 0.202016 | 0.16005 |
| Rpl5l1 | 5.230715 | 5.253329 | 7.760756 | 6.931988 | 5.910094 | 7.31746 | 6.772414 | 6.355299 |
| AABR07051 | 0.329345 | 0 | 0 | 0.606958 | 1.424466 | 1.299467 | 0 | 0 |
| AABR07017 | 0 | 0 | 0 | 0.105818 | 0.248344 | 0 | 0 | 0.101786 |
| Tmem132a | 15.82191 | 18.11031 | 19.4595 | 15.01806 | 14.61655 | 14.99056 | 18.07893 | 19.08863 |
| Rasl11b | 11.27223 | 11.3333 | 10.99854 | 16.80314 | 16.97133 | 17.38995 | 13.47288 | 14.31641 |
| AABR07072 | 0.088531 | 0.116863 | 0 | 0.21754 | 0.14359 | 0.025875 | 0.066029 | 0.039235 |
| Chtop | 32.7287 | 31.46339 | 28.40393 | 31.82429 | 33.0133 | 34.29539 | 33.72203 | 34.62831 |
| Ccdc175 | 1.414771 | 1.469179 | 1.539654 | 1.464381 | 1.760285 | 1.818227 | 1.799603 | 1.546005 |
| Acss2 | 21.68062 | 24.19995 | 24.66775 | 20.81937 | 21.72472 | 21.8282 | 22.96797 | 21.96878 |
| Nrbp2 | 20.01307 | 21.50076 | 21.09795 | 26.68662 | 25.6713 | 25.4354 | 27.31439 | 26.69314 |
| Barx2 | 0 | 0 | 0 | 0 | 0.037799 | 0 | 0 | 0.030984 |
| Rnpc3 | 4.563866 | 4.526708 | 3.754241 | 5.188509 | 4.582354 | 5.170901 | 4.973231 | 5.358552 |
| AC096600. | 0 | 0 | 0 | 2.453549 | 0.959704 | 0 | 0 | 0 |
| Rpl34 | 430.2016 | 390.0737 | 406.9048 | 378.917 | 401.8113 | 403.4752 | 414.7396 | 405.108 |
| Man2a1 | 4.496797 | 5.060559 | 4.373389 | 5.502551 | 5.01917 | 4.949692 | 8.087172 | 7.146464 |
| AABR07012 | 0.121217 | 0.137152 | 0 | 0 | 0 | 0.106284 | 0 | 0 |
| Slc35c1 | 3.470361 | 3.71989 | 3.647219 | 2.894852 | 3.120459 | 2.978756 | 3.821183 | 3.674955 |
| Gpatch2 | 3.898284 | 4.202344 | 4.020291 | 4.044665 | 3.451783 | 3.364191 | 4.395567 | 4.298648 |
| LOC102548 | 0.167839 | 0.189902 | 0 | 0 | 0.362965 | 0.147162 | 0.563314 | 0.446293 |
| Nsun3 | 0.625434 | 0.321659 | 0.549363 | 0.104784 | 0.491835 | 0.34897 | 0.763318 | 0.554354 |
| AABR07051 | 0.161623 | 0.182869 | 0 | 0.148929 | 0.349522 | 0.283423 | 0 | 0 |
| Tmem256 | 62.54818 | 64.76792 | 70.45642 | 61.65678 | 57.4552 | 57.24306 | 52.74534 | 58.09399 |
| Akap1 | 51.11092 | 51.37101 | 52.02407 | 54.37104 | 51.21634 | 50.91947 | 52.37972 | 55.60054 |
| Ap2b1 | 5.277357 | 4.8367 | 5.559128 | 5.015159 | 5.539547 | 4.916994 | 6.02733 | 7.284921 |
| Nes | 0.899792 | 0.850258 | 0.827987 | 0.974903 | 0.972932 | 0.814947 | 1.039834 | 0.972812 |
| LOC100362 | 15.58232 | 15.35574 | 16.05407 | 15.41718 | 15.39309 | 14.3709 | 15.9466 | 16.07085 |
| Tas2r120 | 1.179413 | 1.267727 | 1.076249 | 1.086782 | 0.510113 | 0.775583 | 0.52779 | 0.36588 |
| Ankrd42 | 0.79457 | 0.834803 | 0.893641 | 1.115679 | 1.350103 | 1.045019 | 1.439219 | 1.374997 |
| AC115273. | 0 | 0 | 0 | 0 | 1.35894 | 0 | 0 | 0.371316 |
| AABR07073 | 0 | 0.538633 | 1.022148 | 0 | 0 | 0 | 0 | 0 |
| LOC100911 | 0.529283 | 0.711145 | 0.852328 | 0.274339 | 0.608075 | 0.49308 | 0.703164 | 0.381168 |
| Crispld1 | 0.023626 | 0.160387 | 0.101454 | 0.468054 | 0.293778 | 0.466084 | 0.211449 | 0.366458 |
| Zswim8 | 0.3476 | 0.373628 | 0.261219 | 0.408382 | 0.272495 | 0.304776 | 0.418045 | 0.415928 |
| Drc3 | 8.371619 | 7.969623 | 9.080442 | 9.84215 | 10.45675 | 11.39003 | 10.27008 | 11.94905 |
| AY172581. | 83.1694 | 77.54724 | 92.59462 | 78.76615 | 71.61082 | 54.01704 | 67.20006 | 78.49514 |
| Snx12 | 22.49458 | 22.36027 | 21.50956 | 27.14763 | 27.62194 | 23.87553 | 26.79612 | 26.39573 |
| Cxcl14 | 2.046559 | 2.242075 | 2.441235 | 0.927945 | 0.948392 | 0.655107 | 0.872228 | 0.806208 |
| Murc | 45.81215 | 43.21221 | 42.89135 | 41.1247 | 40.87937 | 40.93431 | 39.92383 | 40.18415 |
| Aldh3a1 | 1.405596 | 1.996416 | 1.059508 | 3.747816 | 3.686446 | 3.723505 | 5.420169 | 5.115935 |
| Scrib | 7.977141 | 8.822814 | 10.18555 | 8.264013 | 7.298557 | 7.937983 | 10.48757 | 9.856914 |
| Zfp383 | 0 | 0.068181 | 0.043129 | 0.2036 | 0.043439 | 0.299404 | 0.134832 | 0.017804 |
| Tstd2 | 8.765111 | 7.781275 | 7.962229 | 7.289744 | 6.92593 | 9.242014 | 9.806017 | 10.55781 |
| C3ar1 | 0.569476 | 0.644335 | 0.833685 | 1.192613 | 1.287511 | 1.92918 | 1.332128 | 1.353661 |

| | | | | | | | | |
|---|---|---|---|---|---|---|---|---|
| Mst1 | 0.596035 | 1.132004 | 0.868411 | 0.725759 | 0.598449 | 0.839899 | 0.857337 | 0.849046 |
| #N/A | 0 | 0 | 0 | 0 | 0 | 0 | 0 | 0 |
| Klhl7 | 19.36656 | 16.71845 | 15.86308 | 18.13284 | 17.11974 | 18.4387 | 19.24399 | 21.14217 |
| LOC500007 | 0.556689 | 0.125974 | 0.318742 | 0.410374 | 0.160517 | 0.292863 | 0.788879 | 0.328948 |
| Ppp2r1b | 1.267847 | 1.608389 | 1.319869 | 2.017923 | 1.869415 | 1.987494 | 2.106125 | 2.054543 |
| T2 | 0.070957 | 0.040142 | 0.076176 | 0.098075 | 0 | 0.155537 | 0 | 0.094338 |
| Lama2 | 24.73721 | 25.12238 | 24.76169 | 21.23282 | 21.6681 | 21.47384 | 26.39219 | 27.98355 |
| B4galt7 | 9.145498 | 8.789553 | 9.894093 | 10.08663 | 8.934368 | 10.24794 | 9.40199 | 9.264125 |
| Cgm4 | 0 | 0 | 0 | 0 | 0.063019 | 0 | 0.032601 | 0 |
| Cpt1a | 17.88695 | 16.92454 | 18.41025 | 13.03626 | 12.78488 | 13.05173 | 17.3168 | 17.64545 |
| Zfp62 | 5.005444 | 4.797917 | 3.999005 | 4.091326 | 4.243542 | 3.893035 | 4.241761 | 4.731362 |
| Snx31 | 0 | 0.044988 | 0 | 0.036639 | 0 | 0 | 0.088967 | 0.105728 |
| Csdc2 | 11.33498 | 10.20716 | 11.00664 | 5.711356 | 6.357696 | 6.420956 | 13.27476 | 12.79362 |
| Ftcd | 0.08419 | 0 | 0.060255 | 0.28445 | 0.242755 | 0.049212 | 0.125583 | 0.174117 |
| Mylk2 | 0.047714 | 0.040489 | 0.038418 | 0.06595 | 0.025796 | 0.031377 | 0.05338 | 0.063437 |
| Rwdd4 | 20.38303 | 20.4238 | 18.85861 | 19.81476 | 18.60126 | 19.81041 | 18.90702 | 20.42883 |
| AABR07071 | 0.382233 | 0 | 0 | 0 | 0.413303 | 1.005427 | 0 | 0.677584 |
| AABR07026 | 0 | 0 | 0 | 0 | 0 | 0.189729 | 0 | 0.095898 |
| LOC691995 | 1.955279 | 1.850292 | 1.641128 | 2.06378 | 1.768252 | 2.026097 | 1.988617 | 1.63853 |
| Irak1 | 17.79034 | 17.85448 | 16.90501 | 17.27296 | 16.2115 | 16.74425 | 21.51478 | 21.60369 |
| Ndufa4 | 1125.701 | 1121.364 | 1069.219 | 966.1691 | 963.1598 | 914.1369 | 939.3697 | 947.2701 |
| AABR07009 | 0.988036 | 0.372639 | 0 | 0 | 0 | 0 | 0.736914 | 0 |
| Tesc | 491.3466 | 502.3813 | 531.4142 | 312.4858 | 302.5135 | 312.2185 | 332.7972 | 314.2515 |
| Tomm70 | 22.15362 | 23.90402 | 23.08185 | 24.02622 | 22.27219 | 22.53438 | 22.38261 | 22.83495 |
| Col11a2 | 0.861441 | 0.809946 | 0.794557 | 1.341607 | 1.705497 | 1.468072 | 1.479548 | 1.48406 |
| Tp63 | 0.012591 | 0 | 0.027034 | 0 | 0 | 0.02208 | 0.028172 | 0 |
| Ccdc25 | 7.62397 | 7.050229 | 5.928747 | 6.034456 | 5.97139 | 6.170495 | 8.146631 | 7.753802 |
| Sncg | 8.384232 | 9.245185 | 9.382407 | 16.8264 | 16.05713 | 18.50285 | 14.30833 | 10.95811 |
| Atxn7 | 4.845578 | 4.762828 | 4.920847 | 3.413405 | 3.196269 | 3.904126 | 5.023337 | 5.638062 |
| Atxn7l3b | 9.430335 | 8.798941 | 8.860558 | 7.701283 | 8.795417 | 8.216276 | 10.0669 | 9.982753 |
| Tubb4b | 54.17822 | 56.37164 | 55.77442 | 51.85674 | 51.56877 | 51.01609 | 56.02359 | 60.75877 |
| Eri3 | 21.48345 | 21.44437 | 22.67577 | 23.12724 | 21.66621 | 22.14219 | 22.76367 | 24.5347 |
| AABR07073 | 0 | 0 | 0 | 0 | 0 | 0 | 0.118713 | 0.094052 |
| AC112062. | 14.33829 | 9.874929 | 4.015583 | 12.06328 | 10.78524 | 8.745616 | 5.579492 | 13.81384 |
| Zfp37 | 3.740423 | 6.072161 | 3.666402 | 3.296798 | 3.34108 | 4.848113 | 4.548499 | 4.612623 |
| Cd200r1 | 0.663385 | 0.422206 | 0.42286 | 0.936027 | 0.941467 | 1.036075 | 0.974092 | 1.028984 |
| Strn4 | 14.80089 | 13.97127 | 15.92577 | 14.98525 | 14.18742 | 15.89404 | 18.09979 | 16.88613 |
| Mir99b | 0.748085 | 0 | 0.803117 | 1.378661 | 0.808893 | 0 | 1.673848 | 0 |
| Glrx3 | 47.60155 | 47.27563 | 44.4042 | 49.53522 | 47.64132 | 47.91077 | 49.15074 | 45.70447 |
| Susd4 | 0.354422 | 0.32081 | 0.475619 | 0.293928 | 0.402393 | 0.29522 | 0.297384 | 0.361264 |
| Dhx29 | 3.637324 | 3.932552 | 3.334661 | 3.521894 | 3.870558 | 3.432196 | 4.482664 | 3.878964 |
| AABR07017 | 0 | 0 | 0 | 0.129713 | 0 | 0.123426 | 0 | 0 |
| RGD130770 | 3.03076 | 3.270195 | 3.641575 | 3.144129 | 2.90817 | 2.833358 | 3.413135 | 3.220018 |
| Ythdf3 | 14.05678 | 13.49177 | 12.08352 | 12.37141 | 13.31652 | 13.26541 | 13.80622 | 13.93554 |
| Ankrd23 | 15.52618 | 15.26399 | 14.74013 | 73.37272 | 72.50444 | 70.62152 | 83.81358 | 83.24189 |
| Fam159b | 0.109096 | 0 | 0 | 0 | 0 | 0 | 0 | 0.193394 |
| Eya4 | 3.431891 | 3.883023 | 3.334976 | 3.189614 | 3.358963 | 2.6978 | 3.409164 | 3.199193 |
| Brcc3 | 10.29842 | 9.156967 | 9.403197 | 9.202028 | 9.380845 | 9.832303 | 9.892127 | 9.754978 |
| Adcy7 | 1.836077 | 2.262412 | 2.214164 | 5.075618 | 5.072093 | 5.325816 | 6.415612 | 5.896561 |
| AABR07012 | 0 | 0 | 0 | 0.268073 | 0 | 0.25508 | 0 | 0.515717 |
| AABR07065 | 0 | 0 | 0 | 0 | 0 | 0.468515 | 0 | 0 |
| AC098115. | 0.80563 | 0 | 0 | 0 | 0 | 0 | 0 | 0 |
| Glrx2 | 49.05162 | 46.37467 | 48.39032 | 49.27113 | 48.49945 | 46.68941 | 41.15758 | 48.37294 |
| Treml4 | 0.070669 | 0.479754 | 0.075868 | 0.520952 | 0.076414 | 0.123926 | 0.158123 | 0.438464 |

| | | | | | | | | |
|---|---|---|---|---|---|---|---|---|
| A930018M2 | 10.04278 | 10.37093 | 11.63725 | 5.141126 | 4.395355 | 4.472644 | 6.331067 | 5.298461 |
| AABR0705( | 0 | 0.148495 | 0.070449 | 0.060468 | 0.141911 | 0.057537 | 0.293657 | 0 |
| Golga7 | 24.33834 | 24.71485 | 24.19119 | 27.14697 | 27.89513 | 27.181 | 28.89147 | 27.64169 |
| Atp2a3 | 7.247652 | 6.722832 | 7.963071 | 6.257264 | 5.831695 | 5.392948 | 7.830768 | 7.766619 |
| Ctdnep1 | 34.52736 | 32.08859 | 30.33462 | 31.55877 | 29.79934 | 30.24307 | 37.80913 | 33.69144 |
| LOC103691 | 0 | 0 | 0 | 0.173885 | 0.102023 | 0 | 0 | 0 |
| Myh6 | 1982.868 | 2093.079 | 2135.106 | 992.7754 | 974.6436 | 929.6465 | 1055.282 | 1077.574 |
| Zfp397 | 3.330066 | 3.163812 | 2.565295 | 3.607273 | 3.820645 | 3.410154 | 3.469577 | 2.636164 |
| #N/A | 0 | 0 | 0 | 0 | 0 | 0 | 0 | 0 |
| Vom2r38 | 0 | 0 | 0 | 0 | 0 | 0.018989 | 0 | 0.057586 |
| AABR07061 | 0 | 0 | 0 | 0.114074 | 0 | 0 | 0 | 0 |
| Medag | 3.08867 | 3.921516 | 2.961515 | 5.431465 | 5.532231 | 5.023512 | 7.596751 | 8.798069 |
| Pla2g2a | 41.94208 | 41.45377 | 40.7234 | 91.84578 | 98.90599 | 104.7002 | 97.57211 | 99.11604 |
| Fmo5 | 2.618296 | 1.454789 | 2.183473 | 1.723326 | 1.643064 | 1.57831 | 2.040002 | 1.823427 |
| Zfp70911 | 0.105152 | 0.23795 | 0.150517 | 0.290682 | 0.1895 | 0.061465 | 0.23528 | 0.217471 |
| AABR07013 | 0 | 0 | 0 | 0 | 0 | 0.204975 | 0 | 0 |
| Cct8l1 | 0.031059 | 0 | 0.033344 | 0 | 0 | 0.027233 | 0.034748 | 0 |
| Rpp25 | 2.768075 | 3.518078 | 3.338081 | 3.563954 | 3.772101 | 3.723695 | 3.605863 | 3.12567 |
| Fcgbpl1 | 0.013755 | 0.007782 | 0.007384 | 0.02535 | 0.014873 | 0.018091 | 0.05386 | 0.012192 |
| Mylip | 7.439407 | 7.201073 | 7.015821 | 7.5312 | 7.140082 | 6.433115 | 8.551949 | 8.121406 |
| Slfn13 | 4.89255 | 4.575159 | 5.012626 | 7.349134 | 8.189007 | 7.169241 | 9.922403 | 9.861105 |
| 1700066B1 | 0 | 0.019318 | 0.03666 | 0.015733 | 0 | 0.044911 | 0 | 0 |
| Myl3 | 4851.279 | 5054.015 | 5206.6 | 4321.865 | 4199.796 | 4229.63 | 4826.09 | 4812.393 |
| Rtcb | 25.87591 | 26.34016 | 25.59185 | 25.84989 | 29.60911 | 27.11794 | 24.92034 | 25.50819 |
| Gbas | 211.7571 | 213.212 | 202.0688 | 183.4022 | 179.1373 | 184.041 | 188.033 | 195.0475 |
| Kif2c | 0.642908 | 0.234652 | 0.333969 | 0.343983 | 0.224248 | 0.581887 | 0.464037 | 0.349258 |
| RGD155964 | 0 | 0 | 0.043212 | 0 | 0.043522 | 0 | 0 | 0.035676 |
| Brwd1 | 2.864988 | 2.784857 | 2.588202 | 2.789796 | 2.734128 | 2.865968 | 2.99823 | 3.329521 |
| Eif3a | 30.32625 | 29.38779 | 29.07987 | 34.43155 | 35.14463 | 33.27344 | 36.47681 | 37.43954 |
| Pdcd7 | 7.451002 | 7.957722 | 7.4509 | 8.191911 | 7.830773 | 7.652414 | 8.907154 | 8.949608 |
| Ackr4 | 4.363827 | 4.526009 | 4.712733 | 3.135496 | 3.089522 | 2.960756 | 3.487182 | 3.039046 |
| Plcxd3 | 0.166948 | 0.094447 | 0.089614 | 0.307671 | 0.300864 | 0.317156 | 0.529192 | 0.567234 |
| Lrrc15 | 0 | 0 | 0 | 0.166677 | 0 | 0.158599 | 0.26982 | 0.133605 |
| Tmx3 | 12.78791 | 11.50644 | 10.99921 | 12.72764 | 13.37604 | 13.64126 | 16.00465 | 14.46249 |
| Ppidl1 | 0.985062 | 0.624149 | 1.226732 | 0.907696 | 1.022528 | 1.347378 | 1.190208 | 1.257278 |
| Pygo2 | 6.167974 | 7.15874 | 6.963235 | 7.409779 | 7.682163 | 7.624005 | 8.956751 | 8.317958 |
| Ehbp1 | 8.508196 | 8.262852 | 7.329021 | 11.00395 | 10.97403 | 10.74594 | 10.1305 | 10.86297 |
| Ndc80 | 0.287409 | 0.411907 | 0.534823 | 0.441393 | 0.352207 | 0.436801 | 0.257232 | 0.33966 |
| Timm17b | 4.641296 | 0.913288 | 10.7237 | 1.022706 | 1.745588 | 11.58921 | 0.677279 | 1.430891 |
| AC136563. | 2.197172 | 1.657331 | 0 | 3.374345 | 3.563655 | 5.779446 | 3.277464 | 6.816118 |
| Ccdc65 | 1.912784 | 2.540612 | 2.291582 | 3.039769 | 2.757687 | 3.159811 | 2.543114 | 3.022226 |
| Ccsap | 1.187051 | 1.343093 | 1.420018 | 1.562602 | 1.356887 | 0.892121 | 1.783342 | 1.623306 |
| AABR07066 | 0.052576 | 0.059488 | 0.056444 | 0.532916 | 0.05685 | 4.148899 | 0.41174 | 4.706693 |
| Mmp15 | 24.37205 | 26.86573 | 29.26713 | 19.60246 | 18.40762 | 19.09951 | 20.62531 | 19.19162 |
| Ptp4a1 | 1.906039 | 11.5775 | 1.615464 | 0.739512 | 1.627084 | 1.671217 | 10.66196 | 1.511584 |
| Mastl | 0.173493 | 0.196299 | 0.093128 | 0.186511 | 0.114642 | 0.059157 | 0.118614 | 0.162318 |
| Kctd20 | 12.87687 | 16.34077 | 16.15527 | 11.91206 | 10.42903 | 11.46755 | 18.41717 | 17.05297 |
| Ncor2 | 13.88398 | 16.8032 | 18.79668 | 15.44313 | 14.62477 | 15.66045 | 20.84173 | 21.10422 |
| Spats2 | 3.876725 | 3.159068 | 3.428726 | 4.275594 | 4.474199 | 3.9627 | 4.47194 | 3.916837 |
| Pdcl | 4.278501 | 3.909975 | 4.318434 | 4.39737 | 4.784444 | 4.649162 | 4.847963 | 4.716 |
| Slc25a5 | 144.0063 | 148.1239 | 144.3944 | 169.9371 | 160.9982 | 160.9388 | 164.9973 | 161.1508 |
| Eps8l1 | 0.082078 | 0.061912 | 0.029372 | 0.025211 | 0.029583 | 0.083961 | 0.076521 | 0.12125 |
| Sptan1 | 37.37191 | 38.15772 | 37.83207 | 43.515 | 42.94504 | 42.32573 | 53.67576 | 55.90039 |
| Npepl1 | 51.19729 | 54.46428 | 63.38732 | 48.79156 | 47.05505 | 47.50027 | 55.25387 | 52.65409 |

| | | | | | | | | |
|---|---|---|---|---|---|---|---|---|
| Mmp12 | 0.058509 | 0 | 0 | 0.350442 | 0.284694 | 0.282156 | 0.621848 | 0.726037 |
| Atp5b | 2860.78 | 2867.172 | 2861.514 | 2692.024 | 2686.615 | 2817.124 | 2895.232 | 2896.653 |
| Erf | 8.508933 | 9.212343 | 10.07412 | 9.244942 | 9.24636 | 9.551599 | 12.53469 | 12.032 |
| Pycr1 | 1.756684 | 2.366197 | 2.51455 | 2.119746 | 2.035154 | 2.603777 | 3.603051 | 3.002856 |
| Slc12a6 | 4.594439 | 4.110356 | 3.95741 | 4.026838 | 3.824128 | 3.766093 | 5.163962 | 5.028792 |
| F11r | 11.88634 | 11.75528 | 11.53193 | 10.3443 | 10.49623 | 10.28685 | 12.11587 | 11.82312 |
| RGD135910 | 6.241815 | 5.78946 | 5.999721 | 7.456999 | 7.298536 | 8.050149 | 9.094224 | 8.234315 |
| Plbd1 | 50.21826 | 50.21964 | 50.49672 | 37.20989 | 38.18794 | 38.49842 | 35.53642 | 38.84719 |
| AABR07032 | 0.147095 | 0.166431 | 0 | 0 | 0 | 0 | 0 | 0 |
| RGD156240 | 0 | 0.128246 | 0 | 0 | 0 | 0 | 0 | 0 |
| Tgfb2 | 4.360694 | 4.295913 | 3.712901 | 11.20595 | 12.29599 | 9.706975 | 12.55386 | 12.66153 |
| Idua | 14.01427 | 14.20429 | 16.06855 | 17.41748 | 17.76307 | 17.88922 | 19.85344 | 20.22321 |
| AABR07029 | 0 | 0 | 0 | 0 | 0.566225 | 0 | 0 | 0.92829 |
| Fsd2 | 34.8602 | 35.61391 | 35.09069 | 30.76355 | 31.07084 | 30.26709 | 31.3227 | 31.03238 |
| Rn60_4_14 | 0.930359 | 0.902278 | 0.570743 | 0.36741 | 0.71856 | 0.582671 | 0.594768 | 0.235607 |
| Tmem45b | 0 | 0.017149 | 0 | 0.083797 | 0.098331 | 0.146182 | 0.084782 | 0.040302 |
| Rn60_12_( | 0.316889 | 0.448181 | 0.255151 | 0.365001 | 0.085662 | 0.347311 | 1.240825 | 0.49153 |
| Slc25a30 | 10.25874 | 8.623486 | 8.711171 | 14.58008 | 14.35147 | 13.69558 | 13.4547 | 12.45769 |
| RGD130558 | 2.036924 | 2.064612 | 2.14121 | 2.385285 | 2.06484 | 2.046432 | 2.27882 | 1.767814 |
| Acaca | 2.687923 | 2.583238 | 2.99865 | 2.626021 | 2.381157 | 2.548439 | 2.989014 | 3.150274 |
| AABR07043 | 0 | 0.884322 | 0 | 0.360098 | 0 | 1.027936 | 0 | 0.346377 |
| Rn60_1_26 | 0.782879 | 0.590527 | 0.560314 | 0.801547 | 0.564344 | 0.686429 | 0.778534 | 0.925206 |
| Setmar | 1.003262 | 0.937728 | 0.936579 | 0.924467 | 1.155561 | 1.319492 | 1.122405 | 1.256536 |
| RGD130518 | 1.084313 | 1.3351 | 0.85594 | 1.704435 | 1.51729 | 1.565902 | 2.212088 | 2.148297 |
| LOC501406 | 1.323456 | 0.768949 | 0.99841 | 1.219512 | 1.315004 | 1.003595 | 1.280539 | 0.72919 |
| Celf2 | 10.03573 | 10.80904 | 10.46321 | 9.128972 | 10.64281 | 10.91456 | 12.91885 | 12.71553 |
| AABR07004 | 0.335679 | 0.189902 | 0.360373 | 0 | 0.362965 | 0.735809 | 0.375543 | 0.148764 |
| Fam221a | 0.074862 | 0.127054 | 0.120554 | 0.241438 | 0.242841 | 0.229737 | 0.335009 | 0.132708 |
| Cryz | 3.305582 | 2.867418 | 2.809429 | 3.934369 | 3.157278 | 3.502157 | 3.143417 | 2.929901 |
| Lgals8 | 7.558616 | 7.699365 | 7.193047 | 8.739972 | 8.059822 | 8.40817 | 8.432819 | 8.054339 |
| Xpr1 | 4.794845 | 5.374909 | 4.432632 | 5.338732 | 5.664652 | 5.11891 | 6.531482 | 7.122533 |
| Heatr1 | 0.192676 | 0.104263 | 0.242824 | 0.216139 | 0.235512 | 0.448054 | 0.196813 | 0.452933 |
| Tsr2 | 3.921777 | 1.590248 | 2.920424 | 1.545771 | 2.843382 | 1.729247 | 1.597764 | 1.707894 |
| Efhc1 | 0.969301 | 0.641982 | 0.456852 | 1.023881 | 1.099218 | 0.994987 | 1.216657 | 0.901049 |
| Tmem183a | 11.48739 | 12.451 | 11.55472 | 12.2699 | 12.16004 | 11.31226 | 14.62687 | 12.84196 |
| Usf2 | 68.88403 | 71.39592 | 77.72444 | 58.61622 | 56.7607 | 62.19046 | 71.98872 | 69.04124 |
| AY172581. | 2248.825 | 2072.089 | 1871.596 | 1420.117 | 1581.498 | 1357.028 | 2314.908 | 2314.279 |
| Nedd4 | 121.8864 | 121.4283 | 115.8473 | 136.157 | 138.043 | 141.1484 | 138.4675 | 141.2369 |
| Defb25 | 0 | 0.548607 | 0.520539 | 0 | 0 | 0 | 0 | 0 |
| Fbxo31 | 42.30502 | 46.09074 | 55.82179 | 33.5674 | 31.17232 | 32.23726 | 43.1296 | 38.53332 |
| Cyp4f37 | 1.3274 | 2.703403 | 2.565087 | 0.550416 | 1.076474 | 5.121007 | 0.891022 | 0.411789 |
| Polk | 0.990216 | 1.120383 | 0.992189 | 1.581571 | 1.391918 | 1.201041 | 1.366299 | 1.667587 |
| AABR07043 | 0 | 0 | 0.739713 | 0 | 0 | 0.604138 | 0 | 0.610717 |
| Tex28 | 0 | 0 | 0 | 0 | 0 | 0 | 0 | 0 |
| Skap2 | 5.339523 | 4.98599 | 4.696357 | 4.949799 | 5.251842 | 5.894427 | 5.865664 | 7.099026 |
| Espl1 | 0.153229 | 0.199377 | 0.213851 | 0.141194 | 0.082842 | 0.228397 | 0.179997 | 0.19014 |
| Pcyt1b | 0.265219 | 0.033342 | 0.09491 | 0.217234 | 0.382369 | 0.103353 | 0.131873 | 0.182837 |
| Ctsf | 20.82831 | 22.96199 | 22.63211 | 25.95257 | 24.80084 | 24.96585 | 28.5219 | 30.12085 |
| Agpat1 | 45.28714 | 50.22704 | 52.46418 | 43.02702 | 40.76083 | 41.95118 | 50.90378 | 51.59268 |
| Cplx2 | 3.39653 | 3.65646 | 3.433981 | 3.220927 | 2.424642 | 2.573293 | 3.689211 | 3.127427 |
| Esyt2 | 9.759291 | 9.011165 | 8.749477 | 10.49477 | 11.93579 | 10.56504 | 11.5415 | 11.92367 |
| AABR07031 | 0.781581 | 0 | 0 | 0.360098 | 0 | 0 | 0 | 0 |
| Gata6 | 21.91384 | 19.1234 | 24.58958 | 18.52792 | 17.6453 | 18.34535 | 21.64731 | 20.01739 |
| Cdca5 | 0.106219 | 0.120182 | 0.152044 | 0.097877 | 0.229706 | 0.186266 | 0.277277 | 0.188294 |

| | | | | | | | | |
|---|---|---|---|---|---|---|---|---|
| Cdca7 | 0.696714 | 1.195163 | 0.772095 | 0.84909 | 0.437427 | 0.453233 | 0.502873 | 0.896418 |
| Tctex1d2 | 2.905923 | 2.21431 | 2.928692 | 2.896281 | 2.821507 | 2.807907 | 2.123114 | 3.259004 |
| AABR0704 | 0.55122 | 1.559199 | 0.887655 | 1.269819 | 0.596026 | 0.48331 | 0 | 0 |
| Creld2 | 4.955642 | 5.209724 | 5.739112 | 6.867622 | 6.624243 | 6.705841 | 10.56445 | 10.34124 |
| Snai1 | 3.302933 | 3.809679 | 4.716404 | 6.293887 | 5.859893 | 6.045079 | 6.708716 | 7.190982 |
| Slc27a1 | 54.10816 | 57.29691 | 61.42355 | 39.48397 | 39.57889 | 40.92817 | 51.26618 | 51.93589 |
| Imp3 | 17.23757 | 16.71142 | 17.37581 | 17.52228 | 16.9122 | 15.78211 | 18.26965 | 16.79028 |
| Nelfa | 6.470846 | 5.097904 | 5.583222 | 5.741792 | 5.182839 | 5.631617 | 7.024797 | 8.157059 |
| Pwwp2b | 5.196797 | 6.515599 | 5.604224 | 5.673033 | 5.973586 | 6.116458 | 7.961439 | 8.071188 |
| AC094643. | 0 | 0.665726 | 0 | 0 | 0 | 0 | 0 | 0 |
| AABR0700 | 0 | 0 | 0 | 0 | 0.567644 | 0 | 0 | 0 |
| LOC68756 | 0 | 0 | 0 | 0 | 0 | 0.071185 | 0 | 0.07196 |
| Pim1 | 4.273124 | 4.698003 | 5.366476 | 6.6492 | 5.710199 | 5.33725 | 7.57677 | 6.717421 |
| Mrpl34 | 68.04818 | 71.90885 | 71.87261 | 66.25299 | 73.97617 | 81.85805 | 61.97047 | 62.75308 |
| LOC67982 | 0.379463 | 0.644017 | 0.814756 | 0.524491 | 0.410308 | 0.665427 | 0.63679 | 0.672674 |
| Stk35 | 3.580576 | 3.370223 | 3.446324 | 3.043375 | 2.853654 | 3.423627 | 4.126653 | 4.56895 |
| Mycn | 0.983115 | 0.817236 | 1.055437 | 0.887414 | 0.911167 | 0.70367 | 0.404032 | 0.444584 |
| Zfp709 | 2.52786 | 2.588933 | 2.620239 | 1.967877 | 2.332763 | 2.503037 | 2.584259 | 2.781394 |
| AC120310. | 0.612467 | 0.692977 | 0.986284 | 0.564364 | 0.264901 | 0.429609 | 0.548161 | 0.597146 |
| Dhcr24 | 3.197505 | 3.142604 | 3.301817 | 3.483215 | 3.120464 | 3.670783 | 3.531753 | 3.566652 |
| Syngr1 | 32.46542 | 31.88465 | 38.28449 | 25.43161 | 23.87412 | 25.85476 | 29.82049 | 30.32072 |
| Hspa8 | 23.87086 | 8.088486 | 4.991207 | 3.754301 | 3.189239 | 14.13596 | 9.955199 | 16.72695 |
| Syt5 | 0.57545 | 0.878977 | 0.648671 | 0.636305 | 0.715559 | 0.706377 | 1.03006 | 0.790577 |
| Mir3064 | 144.5925 | 129.111 | 110.7582 | 171.4067 | 152.9653 | 148.0228 | 206.3579 | 174.574 |
| LOC10255 | 0.055064 | 0 | 0 | 0 | 0.05954 | 0 | 0.246413 | 0.170821 |
| Hnrnpul2 | 26.85958 | 27.86168 | 27.66884 | 25.4302 | 24.80838 | 25.40218 | 32.13669 | 31.33071 |
| AABR0702 | 0.100704 | 0 | 0.108112 | 0 | 0 | 0 | 0 | 0 |
| LOC257642 | 0 | 0 | 0 | 0.211637 | 0.248344 | 0.402759 | 0 | 0.101786 |
| Tmsb10 | 201.5107 | 196.6805 | 191.2748 | 210.5245 | 216.2154 | 203.2191 | 215.5038 | 216.8757 |
| AC094348. | 0.991779 | 0.673291 | 1.064738 | 0.913885 | 0.857917 | 1.043511 | 1.997205 | 0.879063 |
| Ly6g6f | 0.041462 | 0 | 0 | 0.114616 | 0 | 0.036354 | 0.046385 | 0.073499 |
| Polr3b | 3.57386 | 3.685174 | 4.421806 | 4.682843 | 3.672515 | 4.422547 | 4.62212 | 4.796466 |
| AC117905. | 0.067395 | 0 | 0.072353 | 0 | 0 | 0.059092 | 0.150797 | 0 |
| Nfx1 | 7.668711 | 8.091646 | 8.613561 | 7.488494 | 8.59563 | 7.773333 | 8.066963 | 8.263984 |
| AABR0702 | 0.37139 | 0 | 0 | 0 | 0 | 0 | 0 | 0 |
| Pde4b | 9.706027 | 9.135608 | 7.667147 | 8.592222 | 8.547227 | 8.343021 | 9.934025 | 8.621717 |
| Cwf19l1 | 5.939338 | 6.46359 | 5.451458 | 6.726195 | 6.520168 | 6.897111 | 7.608398 | 6.972224 |
| LOC10036 | 0.200088 | 0.161707 | 0.122747 | 0.158034 | 0.24726 | 0.150375 | 0.191871 | 0.253354 |
| Anxa8 | 0.217173 | 0.184291 | 0.087431 | 0.300175 | 0.469653 | 0.452242 | 0.091111 | 0.33686 |
| LOC10369 | 1.678395 | 0.379805 | 1.081118 | 0.309315 | 0 | 0.882971 | 0.751085 | 1.190116 |
| Defa11 | 0 | 0 | 0 | 0 | 0 | 0 | 0.288594 | 0.114321 |
| AABR0701 | 1.864159 | 0.766985 | 1.455486 | 1.405431 | 1.099466 | 1.337315 | 1.137566 | 1.20167 |
| Pirt | 1.275274 | 1.442912 | 1.026816 | 0.881336 | 0.775651 | 1.188046 | 1.07004 | 1.554215 |
| Gusb | 2.413275 | 3.044636 | 2.590804 | 3.837423 | 3.833335 | 3.408008 | 4.348454 | 4.27801 |
| Arl13b | 2.492904 | 2.280129 | 1.93911 | 2.585972 | 2.356581 | 2.565348 | 2.989354 | 3.188683 |
| Rbm34 | 2.630168 | 2.727919 | 2.392262 | 2.777038 | 3.574703 | 2.834623 | 2.840345 | 3.302603 |
| Clec12b | 0.193411 | 0.300898 | 0.181684 | 0.334163 | 0.261415 | 0.254374 | 0.297521 | 0.192858 |
| Xpc | 4.165635 | 3.963019 | 4.038794 | 4.515845 | 4.566583 | 4.587657 | 5.353732 | 5.468047 |
| Vps37b | 6.358719 | 5.666328 | 6.001066 | 5.974198 | 5.504966 | 6.686752 | 7.695049 | 7.735753 |
| Pnck | 0.032345 | 0.036596 | 0.034724 | 0.029804 | 0.034974 | 0.141799 | 0.072371 | 0.086006 |
| AC134024. | 1.475096 | 4.172505 | 1.58361 | 2.718486 | 0.7975 | 2.586732 | 0 | 1.307451 |
| Rn60_17_ | 0 | 0 | 0.075461 | 0.129539 | 0.152007 | 0.06163 | 0 | 0.124603 |
| #N/A | 0.225715 | 0.255386 | 0 | 0 | 0 | 0 | 0 | 0 |
| Sln | 4.041665 | 3.380009 | 3.395728 | 23.47887 | 26.9812 | 24.498 | 8.846678 | 9.345205 |

| | | | | | | | | |
|---|---|---|---|---|---|---|---|---|
| AABR07017 | 0 | 0 | 0 | 0 | 0 | 0 | 0 | 0 |
| AABR07052 | 0 | 0 | 0 | 0 | 0 | 0 | 0 | 0 |
| AC098008. | 0 | 0 | 0 | 0 | 0 | 0.041477 | 0 | 0 |
| AC111867. | 0 | 0 | 0 | 0 | 0 | 0 | 0 | 0 |
| Alpk2 | 19.56021 | 17.71678 | 18.71183 | 15.83207 | 16.37612 | 16.62525 | 17.07762 | 18.04251 |
| Iqgap3 | 0.424711 | 0.500989 | 0.388046 | 0.324741 | 0.341982 | 0.372387 | 0.374052 | 0.456536 |
| Spint2 | 15.47175 | 14.63285 | 16.78027 | 16.48649 | 14.79907 | 16.2092 | 18.50743 | 17.79223 |
| Ralb | 14.92899 | 15.94185 | 14.52553 | 15.09229 | 14.465 | 15.96638 | 17.27095 | 16.88415 |
| Baat | 0 | 0 | 0.066023 | 0.085003 | 0 | 0 | 0 | 0.027255 |
| Exoc5 | 6.578998 | 6.061793 | 5.764634 | 6.474612 | 7.205313 | 6.553153 | 7.50912 | 7.653572 |
| Ubr7 | 7.090123 | 7.379417 | 6.414607 | 7.192412 | 7.529952 | 7.858405 | 7.155379 | 7.384552 |
| RGD156079 | 0.495889 | 0.785506 | 1.064738 | 1.005274 | 0.857917 | 1.043511 | 0.554779 | 1.230688 |
| C2cd2 | 6.582944 | 7.943524 | 6.127422 | 7.614671 | 6.228287 | 6.554826 | 9.754317 | 8.829776 |
| Phkg2 | 11.06825 | 11.37861 | 11.33946 | 10.63762 | 10.64889 | 11.89603 | 13.34798 | 11.76186 |
| AC127106. | 0 | 0 | 0 | 0.297859 | 0 | 0.850268 | 0 | 0 |
| Hspe1 | 373.2298 | 372.3625 | 348.8895 | 311.3863 | 297.9574 | 300.0778 | 294.4593 | 297.2615 |
| AABR07043 | 0 | 0.099496 | 0.047202 | 0 | 0.047542 | 0 | 0 | 0.038971 |
| AABR07039 | 0 | 0 | 0 | 0 | 0 | 0 | 0 | 0.250889 |
| Rps27a-ps | 0.210305 | 0.356925 | 0 | 0 | 0 | 0.368791 | 0.23528 | 0 |
| Syngr2 | 87.35034 | 93.07069 | 93.47904 | 71.92063 | 71.40661 | 74.64744 | 86.51456 | 83.94846 |
| Mprip | 28.3005 | 28.37339 | 28.54465 | 30.16461 | 28.55765 | 29.69676 | 35.62833 | 35.96115 |
| Ubash3b | 0.743688 | 0.618997 | 0.715804 | 0.653772 | 0.71171 | 0.637077 | 0.678993 | 0.99254 |
| Sec62 | 27.8824 | 30.99359 | 28.85123 | 31.53175 | 32.07986 | 30.83696 | 31.38694 | 31.98976 |
| Mir290 | 0 | 0 | 0 | 0 | 0 | 0 | 0 | 0 |
| AABR07024 | 0 | 0 | 0 | 0 | 0 | 0 | 0 | 0 |
| AABR07016 | 0.519761 | 0.588085 | 0.697496 | 0.419072 | 0.210754 | 0.569659 | 0.290743 | 0.345518 |
| #N/A | 0 | 0 | 0 | 0 | 0 | 0 | 0 | 0 |
| LOC288913 | 96.00419 | 91.38798 | 99.57429 | 99.57692 | 95.40035 | 97.3248 | 102.3456 | 95.57171 |
| RGD155996 | 0.083121 | 0.094047 | 0.267706 | 0.076592 | 0.269631 | 0.510161 | 0 | 0 |
| Lrp11 | 7.224543 | 7.974859 | 7.584035 | 7.099957 | 6.703246 | 7.191256 | 9.874626 | 9.725893 |
| Dpy19l3 | 0.798219 | 0.778575 | 0.760903 | 0.767231 | 0.528278 | 0.585244 | 0.531188 | 0.616014 |
| Xkr8 | 2.686445 | 2.972536 | 2.714419 | 2.293435 | 2.58443 | 2.372797 | 2.983376 | 2.923887 |
| Obfc1 | 7.783243 | 7.664124 | 7.132155 | 9.002451 | 9.542724 | 8.309151 | 7.942448 | 8.226454 |
| AABR07026 | 0 | 0 | 0 | 0 | 0 | 0 | 0 | 0 |
| AABR07066 | 0 | 0.548607 | 0 | 0 | 0 | 0 | 0.542451 | 0.429764 |
| LOC100912 | 0.149739 | 0.096813 | 0.987492 | 0.15769 | 0.161911 | 0.150047 | 0.14359 | 0.075841 |
| AABR07034 | 19.07541 | 21.16901 | 19.58098 | 21.76688 | 19.55228 | 30.74713 | 16.78024 | 14.45242 |
| Celf6 | 0.030623 | 0 | 0 | 0 | 0 | 0 | 0 | 0 |
| Lao1 | 0 | 0 | 0 | 0 | 0 | 0 | 0 | 0 |
| Mmp24 | 8.549538 | 9.004035 | 8.277018 | 8.352857 | 8.584171 | 9.97268 | 9.692944 | 9.387776 |
| AABR07034 | 1.120983 | 1.811914 | 1.203447 | 0.885379 | 0.865788 | 1.965758 | 1.074948 | 1.277464 |
| Foxn1 | 0 | 0 | 0 | 0 | 0 | 0 | 0 | 0 |
| Nxf7 | 0 | 0 | 0 | 0 | 0 | 0.020397 | 0 | 0 |
| AABR07053 | 0.207801 | 0 | 0 | 0.191481 | 0 | 0 | 0 | 0 |
| AC135265. | 5.553961 | 3.590883 | 1.703581 | 5.848865 | 8.579169 | 11.13078 | 7.988818 | 10.54875 |
| LOC103690 | 0.428727 | 0.069298 | 0.065752 | 0.112873 | 0.198675 | 0.375908 | 0.06852 | 0.162858 |
| Sdr42e1 | 0.661591 | 0.680508 | 0.408938 | 0.554209 | 0.650335 | 0.50977 | 0.560726 | 0.693019 |
| AABR07059 | 4.659479 | 4.897323 | 4.215093 | 3.094826 | 2.736499 | 3.069261 | 5.00113 | 5.282953 |
| Crebbp | 3.280969 | 3.530461 | 3.733781 | 2.961194 | 2.735008 | 2.854033 | 3.293684 | 3.725841 |
| LOC103694 | 0.066349 | 0.075071 | 0.089037 | 0 | 0.107613 | 0.145437 | 0.148457 | 0.132319 |
| AABR07065 | 0 | 0 | 0.694051 | 0 | 0 | 0 | 0 | 0 |
| Mgmt | 10.40971 | 11.34719 | 7.836471 | 11.3468 | 9.745935 | 11.57602 | 8.947476 | 8.664043 |
| Hsd11b1 | 20.86421 | 21.11164 | 20.40288 | 22.39328 | 22.67706 | 23.84823 | 23.22101 | 23.59053 |
| AABR07034 | 0 | 0 | 0 | 0 | 0 | 0.028203 | 0 | 0.02851 |

| | | | | | | | | |
|---|---|---|---|---|---|---|---|---|
| Slc52a3 | 1.874798 | 1.975954 | 1.930001 | 2.555831 | 2.554817 | 2.026633 | 2.729545 | 3.027529 |
| Zmym2 | 6.588337 | 5.44085 | 5.948367 | 7.513021 | 6.837281 | 6.263581 | 7.271898 | 6.432477 |
| Ushbp1 | 19.75392 | 21.5584 | 23.7811 | 20.37268 | 19.0883 | 19.86899 | 22.47961 | 21.92841 |
| Coch | 0.498104 | 0.392057 | 0.441747 | 0.019956 | 0.023417 | 0.037977 | 0.072686 | 0.019195 |
| AABR07057 | 0 | 0 | 0 | 0.661002 | 0.775651 | 0 | 0 | 0 |
| AABR07063 | 0.177753 | 0.241342 | 0.038166 | 0.262067 | 0.153761 | 0.124683 | 0.079545 | 0.06302 |
| AC094950. | 0 | 1.851549 | 1.756818 | 0 | 0 | 0 | 0 | 0 |
| Srebf2 | 8.725667 | 9.076932 | 8.95805 | 8.117247 | 7.501548 | 7.985128 | 8.054891 | 8.875073 |
| B3galnt1 | 7.35009 | 6.8727 | 5.955314 | 7.539552 | 7.347731 | 6.687756 | 8.564284 | 7.645612 |
| Uba2 | 11.77355 | 13.65259 | 10.25007 | 14.00636 | 13.14225 | 13.99519 | 12.58194 | 15.83493 |
| Tango2 | 101.9718 | 102.8849 | 104.8685 | 109.3944 | 103.7676 | 110.96 | 96.22239 | 99.77138 |
| AABR07000 | 1.484159 | 1.223042 | 1.344668 | 1.217396 | 1.539865 | 1.353966 | 1.679606 | 1.916196 |
| Tgm7l1 | 0 | 0 | 0 | 0 | 0 | 0 | 0 | 0 |
| Hspb11 | 5.092796 | 4.067477 | 5.467441 | 5.024068 | 4.794126 | 4.675503 | 4.088861 | 4.726421 |
| LOC103692 | 0 | 0 | 0 | 0 | 0 | 0 | 0 | 0 |
| Fchsd2 | 3.533897 | 3.963819 | 3.908829 | 4.482763 | 4.234696 | 4.091124 | 5.767757 | 5.735712 |
| AABR07024 | 0 | 0 | 0.677327 | 0 | 0.682199 | 0 | 0 | 0 |
| Cog8 | 7.581844 | 8.440582 | 7.904043 | 7.660298 | 6.616504 | 8.14405 | 9.273178 | 8.600083 |
| Krba1 | 3.240398 | 3.913875 | 3.434739 | 3.439453 | 3.503795 | 3.404625 | 3.732287 | 3.10238 |
| Pstpip2 | 4.911318 | 3.133741 | 4.511974 | 3.338547 | 4.074314 | 3.840326 | 4.485726 | 4.781323 |
| AABR07018 | 8.591991 | 8.902786 | 7.864717 | 6.417084 | 5.085269 | 5.313075 | 4.957942 | 4.008162 |
| RGD155997 | 6.54574 | 6.583286 | 6.246462 | 6.590127 | 5.898178 | 3.719924 | 7.729921 | 6.983666 |
| Rn60_20_0 | 1.030348 | 0.068576 | 0.455471 | 0 | 0.458747 | 1.753678 | 0.881482 | 0 |
| LOC102557 | 0 | 0 | 0 | 0 | 0.083514 | 0.203161 | 0 | 0 |
| Ap3d1 | 17.8185 | 18.86572 | 18.36129 | 18.16338 | 18.38625 | 19.33851 | 22.61875 | 22.09518 |
| Alg2 | 2.459795 | 2.979138 | 3.161458 | 3.495679 | 3.221658 | 3.083242 | 3.953448 | 3.930571 |
| Hist1h4b | 0 | 0.29924 | 0.709825 | 0.487405 | 0.142986 | 0.231891 | 0.739705 | 0.351625 |
| Galt | 15.14485 | 13.82641 | 16.34499 | 18.90253 | 19.14855 | 18.44308 | 17.92951 | 17.32998 |
| AC105648. | 3.597659 | 3.618295 | 3.004024 | 3.683445 | 6.051261 | 4.556399 | 5.366534 | 9.212042 |
| AABR07014 | 0 | 0.156745 | 0 | 0 | 0 | 0 | 0 | 0 |
| AABR07050 | 0.489401 | 0.553734 | 0 | 0.450964 | 1.058365 | 0.858215 | 0 | 0.433781 |
| Adck3 | 219.3624 | 225.5734 | 227.9326 | 188.1261 | 182.4344 | 190.0511 | 187.0754 | 183.308 |
| Cbx3 | 39.03641 | 33.96976 | 32.09546 | 34.42057 | 31.60566 | 33.80976 | 35.89642 | 34.51552 |
| AABR07066 | 0.16677 | 0.094346 | 0.223798 | 0.153672 | 0.27049 | 0.255893 | 0.233219 | 0.221725 |
| Dnajc9 | 10.15388 | 8.309392 | 9.872458 | 10.41562 | 9.943466 | 12.03855 | 10.60954 | 11.57533 |
| LOC100909 | 0.130263 | 0.147387 | 0.111877 | 0.168046 | 0.056341 | 0.068529 | 0.058293 | 0.25401 |
| RGD156391 | 0.242716 | 0.205966 | 0.586284 | 1.286005 | 0.262445 | 0.851253 | 0.610964 | 0.96809 |
| Dixdc1 | 0.791562 | 0.84929 | 0.73258 | 1.219847 | 1.224829 | 1.328253 | 1.740592 | 1.874967 |
| Fam13a | 4.688213 | 4.098926 | 4.545039 | 3.704731 | 3.287362 | 3.537633 | 3.94167 | 4.042067 |
| Tmem44 | 4.318302 | 4.444454 | 3.407161 | 3.355906 | 3.009741 | 3.102022 | 5.005744 | 5.003453 |
| Rps6 | 6.827277 | 5.49492 | 5.89384 | 7.717907 | 5.251281 | 6.911858 | 7.086855 | 6.675206 |
| Cd74 | 403.0278 | 404.2434 | 412.9089 | 372.1777 | 365.8557 | 362.594 | 357.3607 | 345.995 |
| Mir378b | 0.623404 | 0 | 0 | 0 | 0 | 0 | 0 | 0 |
| LOC100909 | 0 | 0 | 0 | 0.169756 | 0.1992 | 0.040382 | 0.154577 | 0.163288 |
| #N/A | 0 | 0.558958 | 0 | 0 | 0 | 0 | 0 | 0.437873 |
| AABR07065 | 0 | 0 | 0 | 0 | 0 | 0.133085 | 0 | 0 |
| Apcdd1 | 1.468515 | 1.416099 | 1.271985 | 2.122031 | 2.435959 | 2.267924 | 2.165654 | 2.336996 |
| Sox17 | 16.16454 | 18.34037 | 22.57427 | 16.26417 | 15.38496 | 16.02862 | 22.39113 | 22.16962 |
| Hyls1 | 1.445344 | 1.32085 | 2.387183 | 2.151414 | 1.683047 | 1.462245 | 1.679178 | 1.478169 |
| Sec61-ps | 0 | 0 | 0 | 0 | 0.302794 | 0.245532 | 0 | 0 |
| RGD156008 | 0.270625 | 0 | 0 | 0.24937 | 0.292623 | 0 | 0 | 0.119934 |
| Srgap2 | 6.959639 | 7.85716 | 7.339959 | 7.543084 | 7.343025 | 7.957077 | 9.861295 | 9.171487 |
| Ahsp | 1.410213 | 0.744609 | 0.706512 | 0.519782 | 0.609937 | 0.577022 | 0.631074 | 0.833295 |
| Stk32b | 0.572097 | 0.345227 | 0.327564 | 0.843463 | 0.6186 | 0.836025 | 0.810713 | 1.149376 |

| | | | | | | | | |
|---|---|---|---|---|---|---|---|---|
| AABR07037 | 0 | 0.811638 | 0.385056 | 0 | 0 | 0 | 0 | 0 |
| Nars2 | 10.50244 | 9.168789 | 9.453445 | 8.221903 | 8.793887 | 9.259849 | 9.687744 | 9.308833 |
| Rft1 | 4.211671 | 3.746138 | 3.162435 | 4.105218 | 4.448724 | 3.351266 | 4.221582 | 4.380357 |
| AABR07055 | 0.684522 | 0.774504 | 0 | 0.31538 | 0.370082 | 0.300095 | 0 | 0 |
| Capn7 | 5.951682 | 5.980237 | 5.16565 | 5.416029 | 6.259373 | 5.633838 | 6.592224 | 5.616458 |
| Tcte2 | 0 | 0 | 0 | 0 | 0.334056 | 0 | 0.172816 | 0 |
| Tbca | 0.855652 | 0.580878 | 0.918597 | 0.47307 | 0.740164 | 0.450142 | 0.765813 | 1.213451 |
| Mmp13 | 0.020125 | 0 | 0 | 0.778875 | 0.674596 | 0.882292 | 0.405274 | 0.481626 |
| Exog | 0.731403 | 0.638809 | 0.551023 | 0.673959 | 0.665984 | 0.652546 | 0.961816 | 0.818879 |
| Anxa10 | 1.716915 | 1.368656 | 0.795935 | 0.970816 | 1.012623 | 0.992191 | 0.654821 | 0.933824 |
| Tigar | 8.077105 | 7.805202 | 6.990967 | 7.692013 | 7.960582 | 8.454365 | 6.550241 | 7.347538 |
| AC097038. | 0.340038 | 0.384738 | 0 | 0.626664 | 0 | 0.298146 | 0.38042 | 0 |
| Nxn | 11.04077 | 10.73266 | 12.39554 | 9.600475 | 9.794392 | 10.39638 | 12.65638 | 12.81826 |
| Phactr1 | 13.42716 | 15.47705 | 15.31585 | 8.970134 | 8.438932 | 8.535385 | 11.45405 | 10.71104 |
| Rbmx2 | 3.018209 | 3.55156 | 3.175435 | 3.81019 | 4.014161 | 3.387351 | 3.207806 | 3.049715 |
| Rpl37-ps1 | 1.246808 | 1.007646 | 2.677055 | 1.31301 | 2.118529 | 2.967263 | 1.394873 | 1.420853 |
| Cck | 0.37139 | 0.36018 | 0.284793 | 0 | 0.172105 | 0.465192 | 0.059356 | 0.235129 |
| Kynu | 0.145138 | 0.032843 | 0.031163 | 0.080244 | 0.031387 | 0.025451 | 0.06495 | 0.051457 |
| Sik3 | 9.870474 | 9.922698 | 10.59658 | 9.207938 | 8.528795 | 8.899522 | 11.09151 | 11.19523 |
| Gcnt2 | 1.792782 | 1.493819 | 1.939586 | 1.626366 | 2.0437 | 1.389133 | 2.005685 | 2.143346 |
| Npw | 0.306233 | 0.259867 | 0.246571 | 0.564364 | 0.910596 | 0.604138 | 0.599551 | 1.221435 |
| RGD130819 | 0 | 0 | 0 | 0.085103 | 0.099863 | 0 | 0.103324 | 0 |
| Fam76b | 4.311166 | 3.813327 | 3.844363 | 4.179609 | 3.872014 | 4.075542 | 4.681746 | 4.443546 |
| Mir3075 | 0.463415 | 0 | 0.497506 | 0 | 0 | 0 | 0 | 0 |
| Arnt2 | 0.101028 | 0.057154 | 0.081345 | 0.124124 | 0.136549 | 0.236216 | 0.188375 | 0.10447 |
| Fam136a | 9.058946 | 9.25429 | 7.521409 | 10.20913 | 10.35907 | 10.71433 | 8.421318 | 10.22448 |
| Mlxip | 5.665251 | 6.252665 | 6.803393 | 4.750585 | 5.136113 | 5.495517 | 6.389876 | 6.602773 |
| Ms4a7 | 0.818827 | 0.617643 | 1.255804 | 1.616821 | 1.475643 | 1.53846 | 1.614023 | 2.07362 |
| Rn50_X_06 | 0.098064 | 0 | 0 | 0 | 0 | 0 | 0 | 0 |
| Slamf8 | 0.621923 | 0.750588 | 0.890232 | 1.050642 | 1.658775 | 1.145136 | 1.461138 | 1.267855 |
| Aldh2 | 259.0941 | 277.4876 | 287.264 | 289.133 | 277.3175 | 295.2624 | 337.9548 | 335.8372 |
| AABR07059 | 0 | 0 | 0 | 0 | 0 | 0 | 0 | 0 |
| Zdhhc19 | 0.026528 | 0 | 0.028479 | 0.024444 | 0 | 0.02326 | 0 | 0.023513 |
| AABR07063 | 0 | 0.107923 | 0 | 0 | 0 | 0.083633 | 0.106712 | 0 |
| LOC690871 | 113.6329 | 121.5074 | 116.1594 | 108.969 | 103.8704 | 88.99099 | 94.40796 | 92.11181 |
| Tmem52b | 0.020657 | 0 | 0 | 0 | 0.022336 | 0.036224 | 0 | 0.018309 |
| AABR07012 | 0.038818 | 0 | 0.041674 | 0 | 0 | 0 | 0 | 0 |
| Svip | 8.298667 | 9.33934 | 9.290289 | 6.951723 | 7.533673 | 7.198457 | 6.603187 | 6.686837 |
| AABR07071 | 0 | 0 | 0 | 0 | 0 | 0 | 0.043268 | 0.068559 |
| Cracr2b | 0.03277 | 0.037077 | 0.03518 | 0.090588 | 0 | 0 | 0.073322 | 0 |
| Dtx2-ps1 | 0.080812 | 0.091435 | 0.086756 | 0 | 0 | 0.141711 | 0 | 0.071627 |
| AC115420. | 1.800944 | 0.940469 | 0.892352 | 1.914807 | 1.49795 | 1.822003 | 1.239887 | 1.350687 |
| AABR07059 | 0 | 0 | 0 | 0 | 0 | 0 | 0 | 0.062301 |
| Nectin4 | 0.027496 | 0.062221 | 0.073796 | 0.063341 | 0.014865 | 0.048217 | 0.046142 | 0.048742 |
| AC133270. | 1.090957 | 1.697253 | 1.171212 | 0.879614 | 0.884727 | 0.478276 | 1.373078 | 1.933938 |
| Nebl | 152.2684 | 151.692 | 135.0706 | 140.9403 | 143.0176 | 133.6355 | 139.8694 | 144.3865 |
| Fa2h | 1.066133 | 1.277236 | 1.256773 | 0.809035 | 0.47468 | 0.788153 | 0.818548 | 0.463219 |
| LOC687780 | 8.2066 | 7.811511 | 12.86585 | 11.88323 | 9.437085 | 8.337705 | 9.618378 | 9.467638 |
| AC127605. | 0.094353 | 0 | 0.202588 | 0.086943 | 0 | 0 | 0 | 0.08363 |
| Ttc26 | 0.959083 | 0.89224 | 0.892352 | 0.648089 | 0.967906 | 0.784863 | 0.882227 | 0.812301 |
| Meox1 | 0.85194 | 0.771144 | 1.128022 | 0.837365 | 0.921191 | 1.319668 | 1.080194 | 0.931311 |
| AABR07058 | 0 | 0.115496 | 0.328761 | 0.094061 | 0.110375 | 0 | 0 | 0.27143 |
| Rn60_16_0 | 0 | 0 | 0.116153 | 0 | 0 | 0 | 0 | 0 |
| AABR07065 | 0 | 0 | 0.551158 | 0 | 0 | 0 | 1.148719 | 0 |

| | | | | | | | | |
|---|---|---|---|---|---|---|---|---|
| C4bpa | 0.021418 | 0.024233 | 0 | 0 | 0 | 0 | 0 | 0 |
| AABR0707 | 0 | 0 | 0 | 0 | 0.341099 | 0 | 0 | 0 |
| Nr1d1 | 41.83864 | 40.3499 | 45.15845 | 48.33622 | 46.38512 | 44.80764 | 29.91021 | 28.83175 |
| Vsig10 | 12.13807 | 11.78496 | 11.64425 | 13.01739 | 13.47944 | 13.08761 | 15.73574 | 16.15603 |
| Rpl21 | 87.27654 | 118.1311 | 101.8445 | 99.50336 | 105.5078 | 104.7573 | 110.7408 | 96.86508 |
| AABR0701 | 0 | 0 | 0 | 0 | 0 | 0.121467 | 0 | 0 |
| Vmp1 | 18.25235 | 16.50331 | 17.85518 | 22.37614 | 20.94257 | 20.33655 | 20.24158 | 19.96931 |
| Cacna1e | 0.016043 | 0.018152 | 0 | 0 | 0.026021 | 0.007033 | 0.026923 | 0.01422 |
| Sap30l | 30.52294 | 33.88774 | 35.43075 | 32.78401 | 29.80675 | 27.43161 | 41.61752 | 38.72104 |
| Tmem131 | 7.876527 | 7.595262 | 6.876271 | 6.805952 | 5.511466 | 6.678471 | 8.316978 | 8.379334 |
| Anks4b | 0.747263 | 0.487785 | 0.586249 | 1.059344 | 0.714774 | 1.033202 | 1.286162 | 1.120877 |
| AC135822. | 0 | 0 | 0 | 0 | 0 | 0 | 0.309972 | 0 |
| AABR0702 | 0 | 0.095104 | 0 | 0.077453 | 0 | 0 | 0 | 0 |
| Prrg2 | 4.355386 | 4.182997 | 3.642763 | 3.453319 | 3.449921 | 3.952021 | 3.852763 | 4.084837 |
| AABR0705 | 1.701059 | 0.905726 | 0.984713 | 1.260114 | 1.298351 | 1.564602 | 2.015014 | 1.83293 |
| AABR0700 | 4.656206 | 3.858457 | 4.013069 | 2.870412 | 3.190999 | 2.300037 | 3.631736 | 3.458565 |
| Dazap2 | 114.9243 | 122.09 | 117.416 | 135.5924 | 136.6068 | 125.917 | 130.3475 | 129.2358 |
| Zdhhc13 | 3.106664 | 3.346322 | 2.534753 | 4.25965 | 3.762293 | 3.966035 | 3.670231 | 3.96517 |
| #N/A | 1.505729 | 1.703662 | 0.898054 | 0.616653 | 0.904513 | 0.586767 | 0.748686 | 1.186314 |
| Hnrnpr | 9.987725 | 8.621804 | 8.970983 | 7.406636 | 9.40123 | 8.739801 | 8.458272 | 8.499923 |
| AABR0703 | 0 | 0 | 0 | 0 | 0 | 0 | 0 | 0.019227 |
| Snrnp200 | 14.90231 | 15.1194 | 15.00694 | 14.41991 | 14.23262 | 14.53767 | 16.19841 | 16.72915 |
| Rmrp | 3.514491 | 0.596472 | 0.754606 | 0.809616 | 0.190008 | 0.462226 | 0 | 0.778767 |
| Sypl2 | 0.215434 | 0.156699 | 0.280843 | 0.978392 | 0.965062 | 1.119865 | 1.101798 | 1.172979 |
| AABR0706 | 0 | 0 | 0 | 0 | 0 | 0 | 0.168347 | 0 |
| Pomt2 | 2.752246 | 2.652259 | 2.83113 | 2.854302 | 2.534661 | 2.789369 | 3.231289 | 3.190766 |
| Il11ra1 | 46.80223 | 51.15594 | 52.01739 | 52.78831 | 52.45763 | 53.44628 | 56.08806 | 53.24257 |
| Apoa1 | 0.235617 | 0.066647 | 0 | 0.162834 | 0.382154 | 0.154942 | 0.263598 | 0.261049 |
| Adipor1 | 70.85562 | 70.39727 | 68.12808 | 67.00633 | 63.08167 | 64.80178 | 68.56576 | 66.28681 |
| Serpini2 | 0 | 0 | 0 | 0 | 0 | 0 | 0 | 0 |
| AC142126. | 2.554435 | 10.11578 | 4.113524 | 10.59215 | 6.905184 | 5.599327 | 10.00226 | 9.056491 |
| AABR0706 | 0 | 0 | 0 | 0.223394 | 0 | 0.106284 | 0 | 0.107441 |
| Bmp1 | 9.907454 | 9.845145 | 10.69794 | 16.07556 | 15.40157 | 17.32337 | 20.20826 | 19.71376 |
| Trappc10 | 7.155592 | 7.593568 | 7.639402 | 7.470793 | 6.776516 | 7.011332 | 8.378113 | 9.063523 |
| Vav3 | 1.015622 | 0.896573 | 0.91061 | 1.038697 | 0.844752 | 1.105784 | 1.061316 | 1.167288 |
| Sssca1 | 19.37638 | 18.60684 | 22.18857 | 19.50269 | 18.85626 | 17.51198 | 17.11962 | 16.24948 |
| Cuedc2 | 4.814063 | 4.859092 | 5.61438 | 5.55294 | 4.980684 | 5.982244 | 5.114534 | 4.850194 |
| RGD15644 | 0 | 0 | 0 | 0 | 0 | 0.05466 | 0 | 0.055255 |
| Bcl3 | 2.389108 | 3.017485 | 3.042044 | 3.737378 | 4.175347 | 4.067755 | 4.382195 | 3.98894 |
| Htra4 | 0.464237 | 0.630315 | 0.647904 | 0.556109 | 0.702762 | 0.976904 | 1.402293 | 0.966969 |
| Cdh8 | 0.00928 | 0 | 0 | 0 | 0 | 0 | 0 | 0.024675 |
| Adcyap1 | 0 | 0.030924 | 0.029341 | 0.075553 | 0.029552 | 0.023964 | 0.09173 | 0.096899 |
| Rn50_X_07 | 1.221363 | 0.863696 | 1.475112 | 1.125438 | 1.650802 | 1.472476 | 0.683203 | 1.353193 |
| AABR0700 | 0.171692 | 0 | 0.092161 | 0.158207 | 0.092824 | 0.225809 | 0 | 0.076089 |
| Olfr225 | 0 | 0 | 0 | 0 | 0.045046 | 0 | 0 | 0 |
| Adprh | 11.08601 | 9.426954 | 10.94055 | 10.78637 | 9.865198 | 10.11266 | 11.90182 | 11.29084 |
| Tmub1 | 8.951102 | 9.32467 | 10.28691 | 8.611441 | 8.3143 | 9.231301 | 10.41113 | 10.79976 |
| Gpx4 | 76.41379 | 68.65831 | 68.04087 | 70.15343 | 67.13291 | 64.73351 | 71.5965 | 76.59391 |
| Rn60_7_12 | 0 | 0.138758 | 0.131658 | 0.113005 | 0 | 0 | 0.137201 | 0.108699 |
| Arrdc3 | 18.97406 | 19.80871 | 18.82873 | 18.89291 | 20.24643 | 19.04439 | 23.60145 | 24.25836 |
| AABR0704 | 12.5804 | 11.92109 | 10.63587 | 16.15683 | 17.51387 | 17.37305 | 15.83369 | 14.07768 |
| Gli3 | 0.553505 | 0.41751 | 0.535966 | 0.730047 | 0.692379 | 0.589989 | 0.898501 | 0.875383 |
| RGD15594 | 6.014632 | 4.807394 | 6.338613 | 6.355787 | 6.742196 | 6.241274 | 11.91448 | 10.41759 |
| Eif2b2 | 7.477749 | 8.534293 | 9.423988 | 9.506826 | 9.515207 | 8.894031 | 9.044735 | 8.914046 |

| | | | | | | | | |
|---|---|---|---|---|---|---|---|---|
| Zdhhc7 | 18.57129 | 18.85112 | 20.6986 | 16.8768 | 16.54595 | 17.42298 | 18.70567 | 19.04409 |
| Riiad1 | 0.468599 | 0.132549 | 0.125768 | 0.323847 | 0.253345 | 0.410868 | 0.262124 | 0.623014 |
| Arl8b | 39.69675 | 38.57304 | 35.7958 | 35.60614 | 35.90797 | 34.43586 | 39.96557 | 41.3273 |
| Efcab6 | 4.108423 | 4.250756 | 4.198377 | 5.395201 | 5.535156 | 5.567143 | 6.98051 | 7.808778 |
| Rn50_5_08 | 0 | 0 | 0.052589 | 0.045139 | 0 | 0 | 0 | 0 |
| Plpp6 | 3.876916 | 4.344969 | 3.945134 | 3.911044 | 3.97351 | 3.753711 | 3.638416 | 4.038877 |
| Ccdc59 | 4.457875 | 3.607329 | 3.907405 | 4.107756 | 3.78297 | 4.180791 | 4.608492 | 4.351361 |
| Mrgbp | 0 | 2.119497 | 0.091412 | 2.353811 | 0 | 0.223973 | 0.09526 | 0 |
| AC094348. | 0 | 0 | 0.839077 | 0 | 0 | 0 | 0.437199 | 0.692754 |
| AABR07044 | 0 | 0 | 0 | 0 | 0 | 0 | 0 | 0.053473 |
| Anks6 | 0.717913 | 0.623381 | 0.734878 | 0.884602 | 0.722111 | 0.775855 | 0.849865 | 1.14686 |
| Pax1 | 0 | 0 | 0 | 0 | 0 | 0 | 0 | 0.032871 |
| AABR0705C | 1.223503 | 0.553734 | 0 | 0.225482 | 0.529182 | 1.930983 | 1.916321 | 1.084451 |
| Trafd1 | 33.44077 | 31.79711 | 29.62665 | 36.6856 | 34.49365 | 35.72154 | 43.12417 | 42.91753 |
| AABR07025 | 0.902042 | 0.644601 | 0.662589 | 0.656208 | 0.61602 | 0.749284 | 0.743595 | 1.178247 |
| Sema4c | 8.013894 | 8.803479 | 8.922073 | 9.474806 | 8.940397 | 9.480319 | 11.69321 | 11.21841 |
| Lrrc24 | 0.78971 | 0.92433 | 0.789335 | 0.727686 | 0.647787 | 0.835677 | 0.974888 | 1.351645 |
| Ldlrap1 | 18.42189 | 15.82562 | 15.87049 | 12.25975 | 15.43133 | 16.20218 | 17.30188 | 16.17705 |
| Atp5l | 1477.114 | 1400.004 | 1408.518 | 1124.389 | 1124.616 | 1102.621 | 1128.338 | 1112.1 |
| Trap1 | 38.61254 | 42.86647 | 39.96897 | 42.22959 | 38.45771 | 40.18288 | 36.72354 | 35.03414 |
| Gemin6 | 2.406522 | 2.613952 | 2.376871 | 2.306216 | 1.821496 | 2.236643 | 3.069233 | 2.303662 |
| Chac1 | 0.100126 | 0.264338 | 0.143322 | 0.246033 | 0.469148 | 0.409689 | 0.373389 | 0.26624 |
| Kalrn | 6.880561 | 6.383346 | 6.856531 | 6.216756 | 5.84689 | 5.930132 | 8.428101 | 8.940137 |
| AC099384. | 0.079103 | 0.04475 | 0 | 0.036445 | 0 | 0 | 0.044248 | 0.035056 |
| Ndufa1 | 16.71776 | 13.07385 | 17.68365 | 9.967784 | 14.62084 | 10.34693 | 8.801452 | 11.11334 |
| Ak5 | 0 | 0.019639 | 0.037268 | 0.047981 | 0.037536 | 0.121749 | 0.058255 | 0.076922 |
| Kcnj14 | 0.614038 | 0.716466 | 0.968213 | 1.30844 | 1.161913 | 1.379622 | 1.631526 | 1.666773 |
| AABR07056 | 0.387179 | 0 | 0 | 0 | 0 | 0 | 0 | 0 |
| Tmem107 | 0.605386 | 0.742047 | 0.487441 | 0.976219 | 1.200092 | 0.973139 | 1.015919 | 1.162599 |
| Unc13d | 2.744276 | 2.814491 | 3.583628 | 2.558318 | 2.307936 | 2.47654 | 2.92654 | 2.916021 |
| AABR07026 | 1.342716 | 1.899025 | 1.081118 | 2.010547 | 1.996307 | 1.324456 | 1.502171 | 1.33888 |
| Pabpc4 | 200.8196 | 202.1202 | 202.3509 | 142.708 | 139.3768 | 134.4902 | 172.9717 | 176.0054 |
| Phf21a | 5.2577 | 4.906707 | 5.747478 | 4.8094 | 4.502413 | 5.013747 | 6.05382 | 5.782681 |
| Sgpp2 | 0.039661 | 0.014958 | 0.014193 | 0 | 0.05718 | 0.011592 | 0 | 0.058589 |
| AABR07012 | 0 | 0.828665 | 0 | 0 | 0 | 0 | 0 | 0 |
| Dcun1d1 | 8.714912 | 9.471283 | 7.96082 | 9.509742 | 9.010006 | 8.881269 | 9.664332 | 11.38341 |
| Asb4 | 0.900274 | 1.086525 | 1.17591 | 0.940176 | 0.827435 | 0.947233 | 0.94004 | 0.824556 |
| Actr8 | 11.85499 | 11.07684 | 11.57755 | 11.15883 | 11.55055 | 10.97566 | 11.26628 | 12.42842 |
| Ighv8-4 | 0 | 0.168802 | 0 | 0.962313 | 1.129224 | 0.784863 | 0.166908 | 0 |
| AABR07068 | 0.272739 | 2.468732 | 0.585606 | 0 | 0.884727 | 1.434828 | 1.525642 | 0.241742 |
| RGD155971 | 0 | 0 | 0 | 0 | 0 | 0 | 0 | 0 |
| Mt-co1 | 79174.97 | 76012.03 | 78268.82 | 79759.68 | 81683.49 | 83004.68 | 74376.85 | 72288.97 |
| AABR07071 | 0.351155 | 0.496644 | 0.188493 | 0.121341 | 0.142387 | 0.461839 | 0.392856 | 0.427963 |
| Ptdss2 | 11.89208 | 12.57881 | 12.19787 | 11.83473 | 10.94829 | 11.28498 | 12.3008 | 12.76911 |
| AC142187. | 0 | 0 | 0 | 0 | 0 | 0.866311 | 0 | 0 |
| Psmd13 | 32.92908 | 32.42101 | 30.15273 | 32.31237 | 34.12517 | 34.43586 | 37.17586 | 36.14294 |
| LOC307727 | 0 | 0 | 0 | 0.154657 | 0 | 0 | 0 | 0 |
| Smarca2 | 16.95762 | 16.56029 | 17.0234 | 15.67599 | 14.40015 | 14.91624 | 18.52031 | 19.27096 |
| #N/A | 0 | 0 | 0 | 0 | 0 | 0 | 0 | 0.305359 |
| Kif1bp | 13.8789 | 12.62244 | 14.24542 | 13.3506 | 13.57886 | 13.87947 | 14.34495 | 13.43626 |
| Pibf1 | 2.427014 | 2.303892 | 1.987288 | 2.577544 | 2.223979 | 2.308348 | 2.116964 | 2.260566 |
| Sec14l2 | 0.505658 | 0.595013 | 0.673141 | 0.764148 | 0.677983 | 1.010864 | 1.154043 | 0.860524 |
| Mir301a | 1.047318 | 0.592496 | 0.562182 | 1.930125 | 1.13245 | 0 | 0 | 0.92829 |
| AABR0701C | 0.311085 | 0.469304 | 0 | 0.286652 | 0.89699 | 0.636438 | 0.348028 | 0.45955 |

| | | | | | | | | |
|---|---|---|---|---|---|---|---|---|
| Mfsd10 | 3.807296 | 4.201847 | 4.656928 | 5.26241 | 5.33156 | 6.621755 | 6.249497 | 5.974694 |
| AABR07008 | 0 | 0 | 0.4358 | 0 | 0 | 0 | 0 | 0 |
| Atp8a1 | 2.634763 | 4.099027 | 2.828587 | 1.062176 | 3.739223 | 1.443852 | 4.421484 | 2.335322 |
| Sox15 | 0.958331 | 1.006856 | 0.808366 | 0.63076 | 1.406311 | 1.080341 | 0.689231 | 0.606726 |
| Vamp8 | 56.55519 | 55.2996 | 55.28119 | 65.62426 | 50.96026 | 51.88337 | 58.38938 | 59.25587 |
| Cylc2 | 0 | 0 | 0 | 0 | 0 | 0 | 0.04717 | 0 |
| Clic1 | 30.96381 | 29.97867 | 34.63269 | 43.18903 | 41.54894 | 41.64428 | 50.58676 | 49.38473 |
| Fam83h | 0.228872 | 0.284854 | 0.233423 | 0.263621 | 0.247476 | 0.381283 | 0.217644 | 0.243433 |
| LOC691311 | 0 | 0 | 0 | 0.050954 | 0 | 0 | 0 | 0 |
| AABR07001 | 0.147926 | 0 | 0 | 0 | 0.159951 | 0 | 0 | 0 |
| Veph1 | 6.556876 | 6.713205 | 5.815012 | 5.97623 | 5.394455 | 5.139798 | 5.461789 | 5.337906 |
| Olr63 | 1.097818 | 1.366342 | 1.237507 | 2.124352 | 3.264401 | 2.598933 | 1.535238 | 2.189364 |
| Zfp91 | 26.09722 | 25.5867 | 24.93343 | 24.63023 | 24.53333 | 23.99093 | 26.3667 | 26.07611 |
| Sfrp2 | 1.654602 | 1.922702 | 1.848334 | 16.29728 | 17.09313 | 18.64418 | 17.08511 | 17.99504 |
| Asic2 | 0.061898 | 0.035017 | 0.199355 | 0.199629 | 0.06693 | 0.189954 | 0.103874 | 0.054863 |
| Pragmin | 3.609166 | 3.710102 | 3.839231 | 3.781975 | 3.48611 | 3.926706 | 3.865431 | 3.998729 |
| AC111831. | 1.994892 | 5.078535 | 3.212466 | 3.523245 | 4.134342 | 3.498247 | 3.719661 | 4.42043 |
| Vom2r26 | 0 | 0.023814 | 0 | 0 | 0 | 0 | 0 | 0 |
| Bora | 0.918298 | 0.831208 | 0.86262 | 1.375035 | 0.943295 | 1.187617 | 1.361239 | 1.444731 |
| Senp6 | 11.48477 | 12.18068 | 11.10913 | 11.2669 | 11.0385 | 11.53457 | 12.17377 | 12.68842 |
| AABR07055 | 3.378447 | 2.866915 | 3.626978 | 3.113105 | 0.913266 | 5.183894 | 1.889828 | 0.748621 |
| Ptar1 | 5.512202 | 5.605693 | 4.508725 | 5.200213 | 6.102176 | 5.84 | 5.909857 | 6.950545 |
| Cox6b1 | 775.9915 | 760.9775 | 802.6756 | 770.043 | 756.066 | 791.5474 | 671.4329 | 674.5937 |
| Synm | 20.50732 | 19.50881 | 20.64365 | 25.91254 | 26.09533 | 26.13069 | 28.50737 | 29.05064 |
| AABR07054 | 0 | 0 | 0 | 0 | 0 | 0 | 0 | 0 |
| Acot5 | 0.056217 | 0.031803 | 0.060352 | 0 | 0 | 0 | 0.031446 | 0 |
| Dtymk | 10.1057 | 9.998675 | 10.89608 | 11.48884 | 11.5894 | 11.39232 | 11.0611 | 12.48578 |
| Ccdc190 | 0.195031 | 0.514894 | 0.279172 | 0.329475 | 0.281179 | 0.313507 | 0.363654 | 0.259299 |
| Ttc8 | 1.364671 | 0.968823 | 1.149068 | 1.627341 | 1.562399 | 1.54847 | 1.167502 | 1.328162 |
| Ndufb10 | 302.5416 | 306.3108 | 294.919 | 292.9806 | 285.5996 | 261.3025 | 251.0649 | 233.3638 |
| AABR07063 | 0 | 0.181191 | 0.171921 | 0 | 0 | 0.140411 | 0 | 0 |
| Fam101a | 0.227819 | 0.441886 | 0.244579 | 0.209927 | 0.105573 | 0.171216 | 0.109232 | 0.259621 |
| Fam83d | 0.096483 | 0.19104 | 0.20716 | 0.155584 | 0.052163 | 0.126894 | 0.323821 | 0.192414 |
| Vps54 | 5.688352 | 5.816969 | 4.959221 | 5.124331 | 5.724171 | 5.032183 | 6.506244 | 6.75633 |
| Gjb3 | 0.064411 | 0 | 0 | 0 | 0 | 0 | 0.21618 | 0 |
| Lat | 2.688823 | 1.296708 | 2.082154 | 2.39641 | 1.811158 | 2.048374 | 2.219116 | 1.758126 |
| Cab39l | 19.665 | 18.85595 | 17.93097 | 20.91879 | 23.34577 | 19.80752 | 19.63871 | 19.23544 |
| LOC10091( | 0.293367 | 0.082983 | 0.472422 | 0.135163 | 0.23791 | 0.064306 | 1.80513 | 1.560152 |
| Hdlbp | 58.68682 | 61.60007 | 59.59071 | 64.85109 | 68.15521 | 66.53935 | 72.09003 | 76.50424 |
| AABR07051 | 0.900162 | 0.797079 | 0.798315 | 1.081909 | 0.634782 | 0.720631 | 1.182202 | 0.763168 |
| AABR0703( | 0.489401 | 0.184578 | 0.175134 | 0 | 0 | 0 | 0 | 0.144594 |
| Otc | 0 | 0 | 0 | 0.028185 | 0 | 0 | 0 | 0 |
| Bcas2 | 33.30205 | 33.13868 | 29.95566 | 37.73078 | 33.01258 | 37.24268 | 34.37038 | 35.99666 |
| AABR0702( | 0.021018 | 0.047561 | 0 | 0.019367 | 0.022726 | 0 | 0 | 0.018629 |
| Parl | 22.58922 | 25.21979 | 24.98585 | 26.96499 | 25.21182 | 25.43302 | 23.40515 | 23.16934 |
| Calu | 70.1584 | 67.16703 | 64.08657 | 80.64599 | 82.11878 | 80.43031 | 78.32313 | 78.89586 |
| Stxbp3 | 12.78027 | 12.2302 | 12.13902 | 13.5927 | 12.51797 | 11.78787 | 11.48946 | 12.6886 |
| AABR07035 | 0 | 0 | 0 | 0.033393 | 0 | 0 | 0 | 0 |
| #N/A | 0 | 0 | 0 | 0 | 0 | 0 | 0.203419 | 0 |
| Mt-cyb | 26540.54 | 25248.3 | 24789.5 | 26097.6 | 25822.34 | 25373.56 | 22272.48 | 22539.63 |
| Ccdc187 | 0 | 0 | 0 | 0 | 0 | 0 | 0 | 0 |
| Per2 | 13.49217 | 13.99532 | 14.02781 | 12.79129 | 12.61107 | 13.0447 | 20.00773 | 20.98004 |
| Cpeb4 | 17.7066 | 17.01594 | 17.47349 | 14.65941 | 15.17458 | 13.74553 | 20.47976 | 23.26723 |
| LOC103694 | 0 | 0.070243 | 0.033324 | 0 | 0.033564 | 0 | 0.069454 | 0.027513 |

| | | | | | | | | |
|---|---|---|---|---|---|---|---|---|
| Areg | 0.09187 | 0.15592 | 0 | 0.042327 | 0.099338 | 0.362483 | 0.25695 | 0.203572 |
| Ipo4 | 7.621728 | 7.396234 | 8.726898 | 6.789451 | 6.778859 | 6.398629 | 8.353477 | 8.840861 |
| Soga1 | 3.407102 | 4.083984 | 4.514836 | 3.377823 | 3.380086 | 3.401438 | 5.547743 | 5.282305 |
| Pla2g2e | 0.099272 | 0 | 0.106575 | 0.137213 | 0.053671 | 0.087042 | 0.05553 | 0.131984 |
| Anxa2 | 60.16753 | 60.58441 | 59.97693 | 102.9587 | 101.3938 | 98.60269 | 110.57 | 106.8276 |
| Cdk17 | 4.095101 | 4.746424 | 4.825265 | 4.12847 | 4.350831 | 3.865825 | 5.698835 | 5.817624 |
| AABR07028 | 0.187961 | 0.467872 | 0.121073 | 0.069279 | 0.162592 | 0.131843 | 0.084113 | 0.533117 |
| Pced1a | 7.635478 | 7.877877 | 8.259987 | 9.461928 | 9.99593 | 9.208548 | 10.47324 | 11.20172 |
| AABR07021 | 0 | 0 | 0 | 0.297859 | 0 | 0 | 0 | 0 |
| Trub1 | 1.856007 | 1.599989 | 1.960915 | 1.873117 | 1.672395 | 1.51111 | 2.109378 | 1.370893 |
| Ddt | 73.89796 | 64.99912 | 71.5672 | 79.20498 | 83.90935 | 79.29311 | 80.07213 | 74.81287 |
| Atp5g1 | 1258.742 | 1290.395 | 1339.916 | 1085.05 | 1048.677 | 1046.212 | 1005.86 | 1031.461 |
| Cacna1c | 16.72506 | 17.60874 | 18.77311 | 14.13805 | 13.95823 | 14.63012 | 18.10511 | 17.98397 |
| Ceacam19 | 0 | 0 | 0 | 0 | 0 | 0 | 0 | 0.045864 |
| Gstp1 | 74.22478 | 73.65747 | 74.49455 | 69.27368 | 70.02342 | 75.46932 | 64.29838 | 61.56412 |
| Ykt6 | 14.55935 | 15.12295 | 13.63175 | 15.35127 | 14.81373 | 16.11402 | 16.34176 | 18.02423 |
| Dcst2 | 2.900098 | 3.38967 | 3.333731 | 2.445431 | 2.115209 | 2.063033 | 2.540505 | 2.279501 |
| Ighv1-47 | 0 | 0 | 0 | 1.31301 | 0.962968 | 1.093202 | 0.199268 | 0.157873 |
| Ncapd2 | 1.795762 | 1.989194 | 1.523418 | 2.048155 | 2.32193 | 2.003942 | 2.275951 | 2.036896 |
| LOC102553 | 0.145562 | 0.123522 | 0.078135 | 0.201194 | 0.590228 | 0.414794 | 0.122136 | 0.54833 |
| Igkv8-27 | 0.128348 | 0.14522 | 0 | 0.236535 | 0.277561 | 0.112536 | 0 | 0 |
| Mkrn1 | 10.67124 | 9.50466 | 10.72306 | 9.754465 | 9.74786 | 9.83561 | 9.646318 | 10.18486 |
| Pcp2 | 0 | 0 | 0 | 0.104671 | 0 | 0 | 0 | 0 |
| Zfp655 | 7.913189 | 3.839289 | 2.517651 | 3.271604 | 4.490702 | 2.883296 | 2.740889 | 3.019208 |
| Rnf19b | 13.21094 | 14.66133 | 14.51473 | 14.60803 | 12.64357 | 14.54082 | 16.94962 | 16.09435 |
| 5S_rRNA | 0 | 0 | 0 | 0 | 0 | 1.157508 | 0 | 0 |
| Foxa2 | 0 | 0 | 0 | 0 | 0 | 0.04129 | 0.052684 | 0 |
| Gapdh | 1491.802 | 1552.611 | 1598.571 | 1650.912 | 1569.551 | 1647.606 | 1363.117 | 1372.517 |
| Tnfrsf13c | 0.090131 | 0.067986 | 0.032254 | 0.055368 | 0.097457 | 0.079027 | 0.168057 | 0.053258 |
| AABR07007 | 0 | 0.159273 | 0.151124 | 0.129713 | 0 | 0.370278 | 0.472457 | 0 |
| E2f5 | 1.342716 | 0.876473 | 1.995911 | 1.85589 | 1.340178 | 2.173467 | 1.964377 | 1.7394 |
| Olr744 | 0.056673 | 0 | 0 | 0 | 0 | 0 | 0 | 0 |
| Smndc1 | 11.15843 | 10.11067 | 8.251295 | 9.556766 | 9.01154 | 10.35207 | 12.27636 | 11.49077 |
| Dus3l | 7.667774 | 6.721466 | 7.698039 | 8.39185 | 8.828698 | 7.732724 | 8.783308 | 9.069504 |
| LOC100361 | 0 | 0 | 0 | 0.209796 | 0.246185 | 0 | 0.509432 | 0.201802 |
| AC123144. | 0 | 0 | 0 | 0.63491 | 0 | 0 | 0 | 0.610717 |
| RGD156369 | 0 | 0 | 0 | 0 | 0 | 0 | 0 | 0 |
| AABR07027 | 0 | 0 | 0 | 0 | 0 | 0 | 0.610257 | 0 |
| RGD156110 | 0.46518 | 0.676708 | 0.642086 | 0.48988 | 0.215568 | 0.466137 | 0.446076 | 0.117803 |
| AC133294. | 0.39974 | 0 | 0 | 0 | 0 | 0 | 0 | 0.354309 |
| Rn60_20_0 | 0.584731 | 0.588085 | 0.627746 | 0.598674 | 0.632261 | 0.512693 | 0.290743 | 0.633449 |
| AC114452. | 1.716915 | 0.485652 | 1.382414 | 0 | 0.464119 | 0.376348 | 0.480202 | 0.760894 |
| LOC100363 | 0 | 0 | 0 | 0 | 0.05508 | 0 | 0.113978 | 0.04515 |
| Tril | 4.896133 | 4.21901 | 4.954624 | 4.421959 | 4.253993 | 4.520373 | 4.619059 | 4.397165 |
| Gp6 | 0.08195 | 0 | 0.065984 | 0.018878 | 0.044306 | 0.05389 | 0.045841 | 0.036318 |
| Polr2a | 13.20406 | 12.65604 | 12.94952 | 11.52236 | 10.72106 | 11.47157 | 14.86208 | 14.8324 |
| Mapre1 | 30.66713 | 31.73163 | 30.27132 | 28.85383 | 31.26976 | 31.52039 | 32.82102 | 35.16455 |
| Araf | 29.47126 | 30.27239 | 32.67545 | 29.65924 | 31.13874 | 29.16642 | 31.08779 | 32.88607 |
| Mr1 | 0.452631 | 0.334855 | 0.336412 | 0.641664 | 0.508247 | 0.625829 | 0.428478 | 0.401189 |
| Lmf2 | 11.07518 | 11.374 | 12.38705 | 11.20099 | 12.37183 | 10.69195 | 14.52744 | 15.18647 |
| Mir483 | 0 | 0.811638 | 0 | 0 | 0 | 0 | 0 | 0 |
| Emsy | 8.308421 | 9.141856 | 8.701404 | 8.66262 | 9.368402 | 9.000219 | 9.351943 | 9.954011 |
| Lim2 | 2.210865 | 2.215605 | 2.576948 | 1.979019 | 2.049066 | 2.547728 | 3.81613 | 3.135359 |
| AABR07053 | 0 | 0 | 0.449745 | 0 | 0.45298 | 0 | 0 | 1.113948 |

| | | | | | | | | |
|---|---|---|---|---|---|---|---|---|
| RGD156489 | 35.57736 | 37.20263 | 36.26433 | 26.67384 | 27.3878 | 27.29252 | 31.4044 | 30.77701 |
| Clk3 | 23.47953 | 26.16039 | 24.24468 | 28.46192 | 26.00178 | 27.05628 | 30.47873 | 29.28377 |
| Pbld1 | 5.683524 | 6.752172 | 5.879749 | 5.713247 | 5.279553 | 5.32309 | 5.057877 | 6.022209 |
| AC127919. | 0 | 0.423211 | 0 | 0 | 0 | 0.327961 | 0.418462 | 0 |
| Nat2 | 0.680568 | 0.598913 | 0.649452 | 0.731636 | 0.858536 | 1.093991 | 1.099784 | 0.804295 |
| Prodh2 | 0.247203 | 0.13985 | 0.132694 | 0.037965 | 0 | 0.108374 | 0 | 0.109554 |
| Zfp532 | 4.807363 | 4.992505 | 4.64491 | 3.955175 | 3.870752 | 4.380694 | 5.061331 | 4.770804 |
| Bdh1 | 33.23534 | 32.67767 | 34.61109 | 35.23482 | 32.9818 | 31.4964 | 29.04618 | 30.64877 |
| 5S_rRNA | 0.513391 | 0.580878 | 0.551158 | 0 | 1.110245 | 0.450142 | 1.148719 | 0 |
| LOC102553 | 0.073755 | 0 | 0 | 0 | 0 | 0 | 0 | 0 |
| LOC681367 | 16.24102 | 17.00205 | 17.76168 | 15.66479 | 15.26346 | 16.10334 | 16.81125 | 15.20244 |
| Prmt2 | 7.029873 | 6.903446 | 6.891993 | 7.797746 | 6.712091 | 7.163962 | 9.348617 | 8.51168 |
| Hpd1 | 5.364154 | 5.625194 | 5.89922 | 4.641463 | 4.77454 | 4.645938 | 3.220144 | 2.783132 |
| Tc2n | 0.573685 | 0.960664 | 0.689793 | 0.803514 | 0.992507 | 0.663969 | 0.847193 | 0.650861 |
| LOC100363 | 9.006067 | 8.217694 | 9.200753 | 6.424828 | 6.753864 | 5.73135 | 7.800455 | 5.922518 |
| Cplx3 | 0 | 0 | 0 | 0 | 0.016693 | 0.013536 | 0 | 0.013684 |
| Dner | 2.191903 | 2.557536 | 2.316379 | 2.019752 | 2.444137 | 2.342269 | 2.030731 | 2.124929 |
| Isl2 | 0 | 0 | 0 | 0 | 0.087991 | 0.035676 | 0 | 0.108192 |
| AABR07063 | 0.402042 | 0.265353 | 0.179841 | 0.401338 | 0.326041 | 0.205631 | 0.224893 | 0.207871 |
| LOC103690 | 0 | 0 | 0 | 0.098375 | 0 | 0.046804 | 0 | 0.047313 |
| Dcx | 0 | 0.026849 | 0.019107 | 0.038266 | 0.032073 | 0.036411 | 0.046459 | 0.026291 |
| Crk | 41.49989 | 42.84127 | 43.70031 | 40.16602 | 38.9534 | 39.57514 | 47.64387 | 47.37763 |
| AABR07070 | 0 | 0 | 1.01294 | 0 | 0 | 0 | 0.263895 | 0 |
| Plekha1 | 18.09152 | 18.42503 | 15.67307 | 16.72676 | 18.35456 | 16.96646 | 17.62765 | 17.25922 |
| Stfa2l2 | 0.180884 | 0.204662 | 0.194191 | 0.166677 | 0.684555 | 0.237899 | 0.101182 | 0.641306 |
| Rn50_6_11 | 0 | 0 | 0 | 0 | 0 | 0 | 0.05003 | 0 |
| AABR07044 | 3.093535 | 3.703098 | 3.946823 | 3.47026 | 2.423909 | 2.201379 | 3.862174 | 3.139338 |
| Wisp1 | 0.030955 | 0.035024 | 0.04431 | 0.218684 | 0.3124 | 0.280463 | 0.196244 | 0.182914 |
| Samd4b | 5.799483 | 5.197089 | 6.540037 | 5.647121 | 5.085223 | 6.324192 | 7.087954 | 7.28939 |
| AABR07058 | 0.04917 | 0.055633 | 0.052787 | 0 | 0 | 0 | 0 | 0 |
| Hmgb1-ps3 | 0.245849 | 0.185445 | 0.52787 | 0.302054 | 0.265833 | 0.502976 | 0.458409 | 0.217909 |
| Mcf2 | 0.084789 | 0.172683 | 0.145643 | 0.125008 | 0.128354 | 0.089212 | 0.151774 | 0.135275 |
| Rpl30l1 | 0.300954 | 0.340515 | 0.161546 | 0.415975 | 0.162708 | 0.395815 | 0.336693 | 0.5335 |
| Ccdc166 | 0 | 0 | 0 | 0 | 0 | 0 | 0 | 0.081956 |
| Rn50_X_07 | 0 | 0 | 0 | 0 | 0 | 0 | 0 | 0 |
| Olr807 | 0 | 0 | 0 | 0 | 0 | 0 | 0 | 0.048806 |
| Kcnab3 | 0 | 0 | 0 | 0 | 0.026823 | 0 | 0.027752 | 0.021987 |
| AABR07021 | 0.471159 | 0.456938 | 0.553992 | 0.558198 | 0.339638 | 0.550816 | 0.200804 | 1.571014 |
| Rn50_7_11 | 0 | 0 | 0 | 0 | 0 | 0.096866 | 0 | 0 |
| AC120246. | 0.247593 | 0.14007 | 0.132903 | 0.912589 | 0.133859 | 0.325635 | 0.415494 | 0.438908 |
| AABR07037 | 0.745664 | 0.651937 | 0.582195 | 0.312318 | 0.549733 | 0.891543 | 0.758378 | 0.721002 |
| Eif6-ps1 | 0 | 0 | 0 | 0 | 0.076724 | 0 | 0 | 0 |
| Rn50_X_06 | 0.435475 | 0 | 0 | 0.200637 | 0 | 0 | 0 | 0 |
| Sox8 | 0.145282 | 0.127851 | 0.329268 | 0.490861 | 0.401454 | 0.353842 | 0.523722 | 0.686775 |
| Psmb8 | 17.97123 | 18.09593 | 19.10864 | 16.28246 | 15.66649 | 16.81269 | 22.02937 | 19.81572 |
| RGD130814 | 7.84826 | 7.199948 | 6.888504 | 7.109702 | 6.93805 | 7.32307 | 7.831064 | 7.426323 |
| Mgll | 50.55716 | 52.06176 | 52.61868 | 33.00888 | 32.19899 | 33.30444 | 47.57845 | 48.33053 |
| AC096455. | 0 | 0.806117 | 0 | 0.328253 | 0 | 0 | 0.79707 | 0.947235 |
| Rpl31 | 246.7698 | 250.1534 | 242.4654 | 225.575 | 228.9153 | 213.3579 | 246.4755 | 245.1368 |
| RGD156169 | 0 | 0 | 0 | 0 | 0 | 0 | 0 | 0.032099 |
| Fmo6 | 0.196495 | 0.296433 | 0.4219 | 0.573364 | 0.531168 | 0.57429 | 0.769405 | 0.580544 |
| AABR07026 | 0 | 0 | 0 | 0.096797 | 0 | 0.184211 | 0.058761 | 0.093108 |
| #N/A | 0 | 0 | 0 | 0.156159 | 0 | 0 | 0 | 0 |
| Arhgap30 | 1.968 | 2.355784 | 2.128084 | 2.20766 | 2.02003 | 2.513293 | 2.792025 | 2.730266 |

| | | | | | | | | |
|---|---|---|---|---|---|---|---|---|
| AABR0705 | 0.717341 | 0.405819 | 0.705936 | 0.495751 | 0.452463 | 0.157241 | 0.601897 | 0.317908 |
| Gng8 | 1.684498 | 1.325865 | 1.336656 | 5.129005 | 4.75154 | 4.430908 | 4.34264 | 4.479163 |
| Zdhhc9 | 3.403608 | 2.948439 | 3.159179 | 3.67534 | 4.331986 | 4.352084 | 4.323445 | 4.383768 |
| Steap3 | 6.964668 | 7.445697 | 7.308361 | 10.90521 | 10.19205 | 10.1318 | 13.06438 | 12.70211 |
| Stard8 | 13.03841 | 12.75067 | 12.40218 | 10.87326 | 9.239417 | 11.02878 | 11.20234 | 11.3057 |
| Ndufa4l2 | 7.940962 | 7.916698 | 7.690501 | 6.652076 | 6.364779 | 6.962642 | 9.381002 | 7.038469 |
| Cstf2 | 3.046601 | 3.156098 | 3.398151 | 3.664103 | 4.08572 | 3.712014 | 3.010017 | 3.787509 |
| Rps15al2 | 0.266493 | 1.356861 | 0.572195 | 0.982252 | 0.288155 | 0.584154 | 0.596282 | 0.944825 |
| Mvk | 9.212523 | 9.874929 | 10.6947 | 9.707491 | 7.387617 | 8.57998 | 8.975092 | 9.337601 |
| Psma5 | 44.06211 | 47.09431 | 45.40912 | 45.52724 | 48.70995 | 49.55488 | 46.62387 | 47.05853 |
| Frs3 | 3.478946 | 3.906888 | 3.902103 | 3.133942 | 3.705588 | 3.050343 | 3.398317 | 3.681866 |
| Sumf1 | 7.465866 | 8.11508 | 8.217713 | 8.34816 | 9.04781 | 9.120378 | 9.314422 | 10.46511 |
| Rbm46 | 0.146437 | 0.132549 | 0.15721 | 0.296859 | 0.15834 | 0.128396 | 0.196593 | 0.129795 |
| Mrpl22 | 28.69452 | 27.77768 | 25.34536 | 29.97017 | 27.22497 | 27.41656 | 24.86687 | 25.54468 |
| Zfp939 | 0 | 0.07261 | 0 | 0 | 0.034695 | 0.028134 | 0.538462 | 0 |
| Spg21 | 12.12298 | 11.78946 | 9.967251 | 14.40208 | 13.83063 | 13.38196 | 14.94507 | 13.88291 |
| Abcc8 | 5.72543 | 6.313893 | 5.571493 | 5.861954 | 5.201258 | 5.753989 | 5.993316 | 6.489327 |
| Hrh2 | 0.11579 | 0.131011 | 0.155385 | 0.13337 | 0.125202 | 0.101525 | 0.356236 | 0.20526 |
| Trmt13 | 3.544625 | 2.058762 | 2.004167 | 2.591211 | 2.708478 | 2.465624 | 2.960958 | 2.680983 |
| AC117101. | 0 | 0.131666 | 0 | 0 | 0.251656 | 0 | 0.260376 | 0.103143 |
| Naa38 | 66.58058 | 59.58291 | 63.41345 | 58.36525 | 56.54287 | 54.76158 | 56.77192 | 56.53301 |
| Erал1 | 13.03352 | 12.82332 | 12.96917 | 12.03362 | 12.39401 | 12.85064 | 12.39125 | 13.40154 |
| Ralgps1 | 0.920272 | 0.995172 | 1.040429 | 0.893021 | 0.871793 | 0.792614 | 1.084226 | 1.017799 |
| AABR0706 | 0 | 0.604588 | 0.286827 | 0 | 0 | 0 | 0 | 0 |
| Gng12 | 21.47966 | 22.04051 | 21.86704 | 21.63542 | 22.90529 | 23.70408 | 24.4448 | 23.76528 |
| LOC100362 | 0 | 0 | 0.085179 | 0 | 0.085792 | 0 | 0 | 0.070325 |
| Pds5b | 8.060923 | 7.532399 | 7.060906 | 7.18762 | 7.534491 | 7.322758 | 7.851669 | 8.077886 |
| Heatr9 | 0.028184 | 0.063778 | 0.030257 | 0 | 0.091425 | 0.222406 | 0.094593 | 0.074943 |
| Tns3 | 6.024023 | 5.609333 | 6.019854 | 8.902264 | 8.018023 | 8.526534 | 10.33429 | 10.4597 |
| Pccb | 73.0699 | 74.36193 | 79.82762 | 70.72784 | 68.4439 | 70.11146 | 76.09792 | 74.29904 |
| AABR0700 | 1.0349 | 0.85869 | 0.888825 | 1.048981 | 0.708714 | 1.119128 | 0.771866 | 1.070163 |
| AABR0702 | 80.86889 | 71.99948 | 88.95279 | 92.23068 | 89.59258 | 85.43581 | 112.7199 | 116.9176 |
| Kcnk13 | 0.283477 | 0.49569 | 0.248998 | 0.617412 | 0.250789 | 0.587488 | 0.749607 | 0.63957 |
| AABR0707 | 2.856323 | 2.693163 | 1.533223 | 2.193324 | 4.632751 | 2.504426 | 0.532588 | 2.531701 |
| Ntn4 | 23.41466 | 23.23621 | 25.39898 | 25.55371 | 24.63224 | 26.30406 | 27.94164 | 29.509 |
| AABR0704 | 0 | 0 | 0 | 0 | 0 | 0 | 0 | 0 |
| Relb | 9.281372 | 8.413082 | 9.907531 | 11.32224 | 11.46135 | 12.80797 | 9.911605 | 9.395084 |
| LOC100911 | 0.036982 | 0.041843 | 0 | 0.034077 | 0.039988 | 0.064851 | 0 | 0.065557 |
| Tubb5 | 93.10565 | 100.0901 | 104.4592 | 105.0354 | 100.7084 | 105.2242 | 131.6453 | 133.6795 |
| AABR0701 | 0 | 0 | 0 | 0.120482 | 0.14138 | 0 | 0 | 0.057946 |
| Cnfn | 0 | 0.079211 | 0 | 0 | 0 | 0.184149 | 0 | 0 |
| Kat2b | 17.11178 | 15.52295 | 10.60285 | 12.44413 | 16.92407 | 16.73554 | 16.35759 | 8.259082 |
| Usp31 | 2.23384 | 2.593998 | 2.170975 | 2.090897 | 2.059463 | 2.226657 | 2.433355 | 2.980366 |
| LOC688812 | 0.114087 | 0.086056 | 0 | 0.070084 | 0.04112 | 0 | 0 | 0.134828 |
| Gpatch8 | 4.568531 | 4.450428 | 4.707439 | 3.716119 | 3.897297 | 4.200273 | 5.539132 | 5.093863 |
| Ube2d4 | 0 | 0.040251 | 0 | 0.032781 | 0 | 0 | 0 | 0.031532 |
| Stard13 | 7.769646 | 8.411521 | 7.861141 | 6.816045 | 7.474454 | 7.163673 | 7.389526 | 7.745387 |
| AABR0706 | 1.775116 | 0 | 0 | 0 | 0 | 0 | 0 | 0 |
| Sf3b1 | 28.28648 | 28.33311 | 26.42957 | 31.45123 | 31.30583 | 31.65078 | 35.9857 | 36.63318 |
| LOC10255 | 0 | 0 | 0 | 0 | 0 | 0 | 0.053114 | 0 |
| Dhx32 | 6.033394 | 5.43523 | 5.315556 | 6.284691 | 5.991988 | 6.382602 | 6.713208 | 6.248667 |
| Bod1 | 20.92483 | 19.45205 | 20.38342 | 18.55381 | 18.16267 | 17.02518 | 19.71561 | 17.84684 |
| Fam208b | 2.03377 | 1.912078 | 1.578632 | 1.833592 | 1.87476 | 1.994888 | 2.283476 | 2.690979 |
| Clec4d | 0.281201 | 0.38887 | 0.251573 | 0.690976 | 0.304059 | 0.328743 | 0.594236 | 0.360017 |

| | | | | | | | | |
|---|---|---|---|---|---|---|---|---|
| Spata20 | 0.020187 | 0 | 0.021672 | 0.111611 | 0.043657 | 0.070801 | 0.067754 | 0.089465 |
| AABR0703( | 0 | 0 | 0 | 0 | 0 | 0 | 1.627352 | 0 |
| Cwf1912 | 3.04322 | 2.9459 | 2.87988 | 2.835365 | 2.864032 | 2.846184 | 3.001109 | 2.947112 |
| Tsen34 | 3.350796 | 2.821408 | 3.429977 | 4.77505 | 4.802802 | 4.577784 | 2.876926 | 2.382888 |
| Prss54 | 0.144577 | 0.065433 | 0.062085 | 0.026644 | 0.156329 | 0.101412 | 0.064699 | 0.076888 |
| AABR0701( | 0 | 0 | 0 | 0.182087 | 0.106835 | 0 | 0 | 0 |
| AABR07061 | 0.140091 | 0.190207 | 0.090238 | 0.180723 | 0.302956 | 0.122832 | 0.156727 | 0.074502 |
| AABR07069 | 0 | 0.071043 | 0 | 0 | 0 | 0 | 0 | 0 |
| Mroh7 | 0.603932 | 0.469783 | 0.48627 | 0.741999 | 0.857092 | 0.860483 | 0.872717 | 1.01483 |
| Tph1 | 0.026875 | 0.136835 | 0.043278 | 0.099057 | 0.043589 | 0.070692 | 0.075166 | 0.059552 |
| Zdhhc22 | 0.029485 | 0 | 0 | 0 | 0 | 0 | 0 | 0 |
| Rab30 | 6.725075 | 7.000378 | 5.920234 | 7.684147 | 7.368683 | 5.857242 | 16.45186 | 15.85564 |
| AABR07001 | 0 | 0 | 0.294336 | 0 | 0.148226 | 0.120195 | 0 | 0 |
| Erich1 | 3.533212 | 3.15605 | 3.154286 | 4.626544 | 4.423634 | 5.315384 | 4.535319 | 4.351361 |
| Hes3 | 0.042678 | 0 | 0.045818 | 0.157304 | 0.046147 | 0.03742 | 0 | 0 |
| Capn5 | 2.50125 | 3.192874 | 3.081155 | 4.358443 | 3.93549 | 3.795747 | 3.820739 | 3.766028 |
| Hspb3 | 40.7046 | 41.90621 | 42.4392 | 58.59309 | 59.95325 | 55.30316 | 49.72312 | 50.18488 |
| AC113773. | 0 | 0 | 0 | 0 | 0 | 0.155117 | 0 | 0.156806 |
| Smad2 | 7.814469 | 7.749194 | 7.352718 | 7.366259 | 7.211358 | 8.013377 | 8.893213 | 9.056006 |
| AABR07035 | 0 | 0 | 0 | 0 | 0 | 0 | 0.120051 | 0.095112 |
| Rbfa | 28.59138 | 27.75621 | 27.11178 | 26.59946 | 27.30678 | 26.2153 | 26.75187 | 24.54907 |
| L3mbtl4 | 0.029992 | 0 | 0.032198 | 0 | 0.06486 | 0.052594 | 0.067107 | 0.026583 |
| RGD156446 | 0 | 0 | 0 | 0.02794 | 0 | 0.053173 | 0 | 0 |
| AC107280. | 0 | 0 | 0 | 0 | 0.148616 | 0.241021 | 0 | 0.121823 |
| Pard6a | 0.112181 | 0.228469 | 0.313126 | 0.392806 | 0.339638 | 0.236064 | 0.075302 | 0.397725 |
| Tesmin | 0 | 0 | 0.082472 | 0.070787 | 0.083065 | 0 | 0.028648 | 0.045393 |
| Fbxl15 | 1.672908 | 2.465064 | 3.29958 | 2.760395 | 2.271631 | 2.967727 | 2.655026 | 2.862114 |
| Vangl1 | 2.227711 | 2.220749 | 2.454804 | 1.908061 | 2.164729 | 2.030701 | 2.722826 | 2.522528 |
| Tmco3 | 0.418155 | 0.254759 | 0.448917 | 0.62243 | 0.556487 | 0.479451 | 1.115556 | 0.570203 |
| Mtmr14 | 8.221607 | 7.138503 | 8.318425 | 6.994524 | 7.248364 | 7.39887 | 8.448015 | 7.32217 |
| AC133383. | 0 | 0 | 0 | 0 | 0 | 0 | 0 | 0 |
| Nupl2 | 3.35486 | 3.893196 | 4.04032 | 4.002929 | 4.022873 | 4.770581 | 4.49911 | 3.964772 |
| AABR07018 | 0 | 0 | 0.449745 | 0 | 0 | 0 | 0 | 0 |
| Ncbp2 | 0.427128 | 0.515494 | 4.463215 | 0.734686 | 1.077644 | 0.998684 | 8.888049 | 0.605736 |
| Pde4a | 10.67635 | 12.10324 | 12.26295 | 8.099497 | 6.769596 | 8.052303 | 8.650863 | 8.727987 |
| LOC367975 | 0 | 0.042171 | 0 | 0 | 0.120902 | 0.065359 | 0.083395 | 0 |
| Rgs9bp | 0.220025 | 0.746843 | 0.472422 | 0.270326 | 0.317213 | 0.32153 | 0.164103 | 0.260025 |
| Anapc1 | 2.931785 | 2.857568 | 2.578641 | 2.869692 | 3.131903 | 2.968053 | 3.082364 | 2.974286 |
| Arhgap22 | 0.8063 | 0.838321 | 1.07617 | 1.365465 | 1.531612 | 1.968037 | 2.267322 | 1.950839 |
| Rn60_20_( | 0 | 0 | 0 | 0 | 0 | 0 | 0 | 0 |
| Avpr1a | 1.077242 | 1.151135 | 0.899491 | 1.571674 | 1.13245 | 1.154421 | 1.104739 | 1.034381 |
| LOC100365 | 2.174806 | 2.364193 | 2.632365 | 2.043292 | 1.659943 | 2.262074 | 2.862443 | 2.759169 |
| Wdr5b | 1.525221 | 2.300954 | 1.6071 | 2.186226 | 2.534881 | 1.981208 | 2.148736 | 1.752436 |
| Tmed1 | 20.78888 | 22.09608 | 21.59961 | 23.14699 | 23.50047 | 25.16666 | 23.47792 | 20.93888 |
| Smpd3 | 0.801466 | 0.706614 | 0.77103 | 1.304398 | 1.451859 | 1.414579 | 2.398816 | 2.6201 |
| Tbr1 | 0.025632 | 0.116005 | 0.082552 | 0.070856 | 0.027715 | 0.022474 | 0 | 0.022719 |
| LOC499544 | 0 | 0 | 0 | 0.159251 | 0 | 0.075766 | 0 | 0.076592 |
| AABR07058 | 0 | 0 | 0 | 0.386644 | 0 | 0 | 0.093886 | 0.223147 |
| LOC100359 | 0 | 0 | 0 | 0 | 0 | 0 | 0 | 0 |
| Snrpd2l | 0.8801 | 0.497896 | 1.102317 | 0.810977 | 0.158606 | 0.128612 | 0.656411 | 0.520051 |
| Slc15a1 | 0.017305 | 0.01958 | 0 | 0 | 0 | 0.015173 | 0 | 0 |
| Col6a1 | 80.92669 | 83.18787 | 85.54559 | 101.8096 | 95.24736 | 99.07863 | 109.3399 | 111.3661 |
| AABR07051 | 0 | 0 | 0 | 0.5147 | 0.301987 | 1.101948 | 0 | 0 |
| Efnb3 | 36.088 | 38.36236 | 38.63545 | 31.71841 | 28.014 | 30.48792 | 32.86184 | 32.48869 |

| | | | | | | | | |
|---|---|---|---|---|---|---|---|---|
| AC111943. | 0 | 0 | 0 | 0 | 0.449385 | 0 | 0 | 0.368369 |
| AABR07003 | 0.137084 | 0 | 0 | 0 | 0 | 0.060097 | 0 | 0 |
| AC114446. | 0.25796 | 0.38916 | 0.276937 | 0.158467 | 0.185952 | 0.301573 | 0.288594 | 0.152429 |
| Rpgr | 0.961383 | 1.06556 | 0.800408 | 1.2113 | 0.891025 | 1.152593 | 1.251152 | 1.269487 |
| Eif4h | 72.20854 | 78.43816 | 76.88807 | 68.22291 | 63.76318 | 68.26008 | 80.8184 | 80.54526 |
| AABR07072 | 0 | 0 | 0 | 0 | 0 | 0 | 0 | 0.16878 |
| LOC100911 | 12.35887 | 9.009972 | 22.29577 | 14.0298 | 23.4205 | 7.428986 | 23.87575 | 15.47151 |
| Chst5 | 0 | 0.029759 | 0.056472 | 0.024236 | 0 | 0 | 0 | 0.023312 |
| Mfsd8 | 6.87664 | 7.780595 | 6.533197 | 7.093575 | 7.040801 | 6.803134 | 7.897526 | 8.090851 |
| Sh3rf1 | 2.271928 | 2.295161 | 2.155955 | 2.457985 | 2.259198 | 2.685682 | 3.097737 | 2.957656 |
| Fkbp15 | 4.332238 | 4.677061 | 3.972673 | 4.92345 | 4.196429 | 4.384113 | 5.43236 | 5.279831 |
| AC132699. | 0 | 0 | 0.060255 | 0 | 0 | 0 | 0 | 0 |
| Kirrel3 | 0.185959 | 0.126242 | 0.179675 | 0.137083 | 0.140752 | 0.228268 | 0.041608 | 0.148342 |
| Psrc1 | 0.171692 | 0.129507 | 0.184322 | 0.052736 | 0.216589 | 0.225809 | 0.128054 | 0.177542 |
| AABR07037 | 0 | 0 | 0 | 0 | 0 | 0 | 0 | 0 |
| Ubtd1 | 11.80634 | 12.68721 | 15.73745 | 12.5708 | 11.94143 | 11.24335 | 9.79571 | 10.26427 |
| Cdon | 2.14865 | 2.094779 | 2.206422 | 1.682521 | 1.992716 | 1.712671 | 2.109276 | 2.43138 |
| AABR07054 | 1.909174 | 0.617183 | 1.171212 | 2.764503 | 0.884727 | 0.717414 | 3.051285 | 1.450454 |
| Lrrn1 | 0.514737 | 0.310614 | 0.331562 | 0.442689 | 0.667893 | 0.270793 | 0.153564 | 0.228119 |
| Adamtsl4 | 22.15772 | 23.79977 | 27.20146 | 25.74276 | 25.33687 | 26.95125 | 33.08975 | 34.90595 |
| AABR07068 | 1.700192 | 0.769475 | 0.547579 | 1.096662 | 1.286875 | 0.894438 | 0.19021 | 1.054875 |
| Wrap73 | 3.925538 | 3.061659 | 4.74622 | 4.846385 | 4.120998 | 4.979082 | 3.453681 | 4.357695 |
| Slc25a47 | 0.025988 | 0 | 0 | 0.023947 | 0 | 0.045573 | 0 | 0 |
| Phf21b | 0.777712 | 0.439972 | 0.500954 | 0.740518 | 0.756836 | 1.022847 | 0.783062 | 0.758257 |
| Actr1b | 40.83957 | 44.68543 | 42.73418 | 37.4164 | 37.03133 | 38.30482 | 41.89295 | 45.22174 |
| AC118419. | 0.427826 | 0.096813 | 1.010457 | 0.47307 | 0.462602 | 0.375118 | 0.478633 | 1.06177 |
| Sepw1 | 13.92502 | 11.09543 | 12.84385 | 12.65063 | 9.331051 | 10.31786 | 8.337893 | 27.64011 |
| Gramd4 | 14.09212 | 13.75979 | 14.37373 | 15.01209 | 14.726 | 14.60966 | 14.06318 | 13.76001 |
| RT1-T24-1 | 5.971552 | 4.331109 | 5.753321 | 4.467883 | 5.905077 | 6.265134 | 4.510905 | 5.202407 |
| AABR07058 | 0 | 0 | 0.111988 | 0 | 0 | 0 | 0.233405 | 0.092459 |
| Serhl2 | 12.93919 | 13.83203 | 15.60488 | 11.47778 | 10.83391 | 10.27689 | 10.08136 | 9.886125 |
| Herc1 | 5.892585 | 5.581284 | 5.480375 | 5.860872 | 5.839673 | 6.002091 | 6.738603 | 7.165087 |
| AABR07010 | 2.103049 | 2.617451 | 1.655689 | 1.679493 | 1.212798 | 1.290769 | 1.960665 | 1.926171 |
| Retn | 0.260851 | 0.368926 | 0.07001 | 0.480729 | 0.352569 | 0.343072 | 0.145914 | 0.346808 |
| Hprt1 | 0.770717 | 0.823584 | 1.24112 | 0.789095 | 1.018557 | 1.614328 | 1.437073 | 0.56927 |
| Tgfb3 | 11.57689 | 11.15313 | 12.08508 | 25.38863 | 24.68992 | 25.3152 | 28.34165 | 28.00112 |
| Pkm | 422.4245 | 448.4663 | 448.1456 | 454.8741 | 444.2335 | 427.8686 | 421.0815 | 412.2051 |
| Slco4c1 | 0.021466 | 0 | 0 | 0 | 0 | 0 | 0 | 0.009513 |
| Cfap45 | 0.746505 | 0.969766 | 0.771738 | 1.222888 | 0.807185 | 1.333314 | 1.299132 | 1.078268 |
| Chi3l1 | 2.805828 | 3.271846 | 2.33602 | 14.32556 | 13.03339 | 11.77359 | 12.81239 | 12.28246 |
| Shank2 | 0 | 0 | 0.010467 | 0.008984 | 0 | 0 | 0.021815 | 0 |
| Fars2 | 17.75228 | 17.77114 | 18.88108 | 16.7837 | 19.01689 | 17.24325 | 14.54465 | 17.63576 |
| Pabpc4l | 0.107749 | 0.081275 | 0 | 0.198573 | 0.194179 | 0.157457 | 0.120545 | 0.286509 |
| Dync1li1 | 15.02775 | 15.66255 | 14.76813 | 18.56095 | 19.2804 | 19.23239 | 18.26729 | 17.29018 |
| Syne4l1 | 0.051189 | 0 | 0.054954 | 0.047168 | 0 | 0 | 0.114535 | 0.045371 |
| AC120246. | 44.84945 | 48.85067 | 52.81448 | 58.75722 | 54.96621 | 53.72307 | 61.85265 | 61.98075 |
| AC111292. | 0 | 0 | 0 | 0.446788 | 0 | 0 | 0 | 0 |
| Isg15 | 6.137254 | 6.944014 | 6.417598 | 12.88954 | 12.71206 | 12.50943 | 17.5219 | 16.56652 |
| AABR07033 | 44.11544 | 32.7771 | 38.20588 | 33.13325 | 30.52489 | 35.17141 | 44.05043 | 35.99103 |
| LOC100360 | 0.858458 | 0.647536 | 1.382414 | 0.922874 | 0.618825 | 0.501798 | 1.120472 | 0.253631 |
| Smap1 | 7.377851 | 8.130162 | 7.817395 | 7.750618 | 7.665737 | 7.564617 | 9.060593 | 9.564075 |
| AABR07032 | 0.141913 | 0.321136 | 0 | 0 | 0 | 0 | 0 | 0 |
| Rita1 | 16.52582 | 15.58955 | 17.60945 | 15.94583 | 14.05586 | 14.70558 | 15.00171 | 13.19379 |
| Ubap2 | 13.19063 | 14.34288 | 15.04407 | 12.42535 | 11.10214 | 13.72927 | 13.95186 | 14.5558 |

| | | | | | | | | |
|---|---|---|---|---|---|---|---|---|
| Alkbh8 | 1.489909 | 0.917253 | 0.846801 | 1.150807 | 1.066114 | 1.037398 | 1.32367 | 1.340001 |
| Fam110b | 3.685484 | 4.206212 | 4.610302 | 5.463176 | 4.262282 | 5.0017 | 3.083403 | 4.772117 |
| Mir103a2 | 1.217812 | 0 | 0 | 0.561083 | 0 | 0.533889 | 2.043651 | 0.539704 |
| Epha8 | 0 | 0 | 0.019141 | 0 | 0 | 0 | 0.039894 | 0.015803 |
| Ubxn1 | 59.7414 | 62.53195 | 65.24474 | 65.01714 | 63.80001 | 67.59992 | 66.12091 | 65.41614 |
| Micu3 | 3.03245 | 2.636844 | 2.501934 | 2.535553 | 3.03606 | 2.437284 | 2.544428 | 2.563376 |
| Plxnc1 | 0.786874 | 0.865232 | 0.78527 | 0.479978 | 0.419426 | 0.51502 | 0.694337 | 0.589391 |
| Zfp428 | 3.566138 | 4.778192 | 5.03747 | 3.631956 | 3.754539 | 4.443337 | 4.462094 | 4.533318 |
| Was | 2.409579 | 1.843149 | 1.967453 | 2.095243 | 2.421961 | 2.469801 | 3.493059 | 3.248715 |
| Atrx | 0.889442 | 0.125795 | 0 | 0.102448 | 0.120218 | 0.194966 | 0.248767 | 0.098545 |
| Emc6 | 33.4629 | 32.52643 | 34.90322 | 33.00728 | 33.27579 | 30.64592 | 30.12529 | 30.28309 |
| Alk | 0.05481 | 0.099225 | 0.035306 | 0.101011 | 0.248916 | 0.096116 | 0.171695 | 0.204041 |
| AABR07069 | 0 | 0.049027 | 0.023259 | 0.039928 | 0.07028 | 0 | 0 | 0.076813 |
| Nacad | 0.192433 | 0.204121 | 0.193678 | 0.410052 | 0.403146 | 0.379633 | 0.659313 | 0.479709 |
| Iqcal1 | 0.059597 | 0 | 0 | 0.054916 | 0.02148 | 0.017418 | 0 | 0.052824 |
| Pcgf2 | 4.004453 | 4.313021 | 3.84433 | 4.470511 | 4.246688 | 5.334183 | 5.901544 | 5.528788 |
| Zfp692 | 4.857593 | 5.012961 | 5.616085 | 8.042189 | 7.445774 | 8.260863 | 9.674532 | 8.492769 |
| Rrm2b | 9.040655 | 7.857116 | 9.189958 | 7.606207 | 8.547685 | 8.348088 | 8.208694 | 8.516425 |
| RGD156315 | 0.743365 | 1.30835 | 0.975394 | 1.369963 | 0.71448 | 0.362102 | 1.663287 | 1.171344 |
| Flnc | 47.88525 | 50.46215 | 54.43148 | 55.0052 | 53.40577 | 56.54591 | 72.86596 | 73.92282 |
| Rn50_X_07 | 0.288915 | 0.245171 | 0.310169 | 0.399336 | 0.3905 | 0.126661 | 0.323226 | 0.44814 |
| Prpsap1 | 12.66668 | 11.53532 | 11.2768 | 14.43324 | 14.73187 | 14.16712 | 15.89908 | 15.63581 |
| Syce1 | 0.072429 | 0 | 0.038878 | 0 | 0.156632 | 0.095258 | 0 | 0 |
| Zfp597 | 1.288368 | 1.410704 | 1.11544 | 1.302069 | 1.707663 | 1.494043 | 1.859831 | 1.399803 |
| Lpar4 | 0.213431 | 0.170461 | 0.134783 | 0.196668 | 0.122178 | 0.286209 | 0.168549 | 0.26707 |
| LOC292543 | 0 | 0 | 0 | 0.019394 | 0.091033 | 0 | 0 | 0.018655 |
| LOC100912 | 0 | 0 | 0 | 0 | 0 | 0 | 0 | 0.049272 |
| AABR07032 | 1.32572 | 2.249984 | 0 | 0 | 2.866963 | 3.487176 | 1.483156 | 1.175051 |
| Sparcl1 | 130.4172 | 128.8722 | 122.9772 | 156.7169 | 156.6018 | 159.2575 | 179.7796 | 177.3116 |
| Fbxl13 | 0.107915 | 0 | 0.057927 | 0.02486 | 0.058344 | 0.118275 | 0.211279 | 0.119563 |
| Kif5b | 45.05321 | 43.62487 | 40.44945 | 58.5776 | 59.35437 | 58.35281 | 62.81568 | 64.46847 |
| Wwc3 | 10.00585 | 9.616677 | 8.913308 | 9.590685 | 9.523466 | 9.456073 | 9.594939 | 9.930795 |
| Rhox3 | 0 | 0 | 0 | 0 | 0 | 0 | 0 | 0.044458 |
| LOC688286 | 2.312828 | 2.024361 | 2.389272 | 1.930125 | 1.793046 | 2.2192 | 2.294566 | 2.127332 |
| Pskh1 | 15.02301 | 17.0383 | 16.89617 | 13.34872 | 13.88489 | 14.74031 | 15.72662 | 15.50321 |
| Rps18l1 | 24.05741 | 20.00493 | 31.7394 | 23.94851 | 21.52073 | 20.7547 | 21.50987 | 25.26251 |
| Fgd3 | 1.579382 | 1.786996 | 1.40001 | 1.815835 | 1.504084 | 1.842169 | 2.70715 | 2.247521 |
| AABR07070 | 0.465475 | 0 | 0.249858 | 0 | 0 | 0 | 0 | 0 |
| AABR07042 | 0 | 0 | 0 | 0 | 0 | 0.425134 | 0 | 0 |
| Dusp4 | 1.000958 | 1.162341 | 1.201847 | 1.347107 | 1.509554 | 1.697543 | 1.488192 | 1.657661 |
| Zc3h14 | 11.48716 | 11.18843 | 11.25344 | 12.52098 | 12.17396 | 11.85407 | 12.39144 | 13.60251 |
| Slc4a11 | 0.361738 | 0.46776 | 0.388349 | 0.428564 | 0.558775 | 0.634345 | 0.655223 | 0.870272 |
| Gnl3l | 20.98294 | 20.87054 | 20.77201 | 21.83081 | 21.74937 | 20.62244 | 22.08113 | 21.76883 |
| Usp14 | 21.92592 | 21.87627 | 18.43369 | 23.09014 | 23.73897 | 24.41782 | 21.82191 | 21.93269 |
| AABR07061 | 1.217812 | 1.837196 | 0.4358 | 1.870277 | 1.316803 | 3.203336 | 2.270723 | 2.87842 |
| #N/A | 8.142593 | 6.387653 | 6.565908 | 5.002053 | 4.891371 | 4.854814 | 6.032561 | 4.77938 |
| Acat2l1 | 2.147498 | 2.055979 | 1.56063 | 1.887504 | 1.714751 | 1.622215 | 2.402526 | 1.7863 |
| Slc12a4 | 15.59447 | 17.72396 | 17.72291 | 22.20888 | 20.98256 | 21.81061 | 26.47637 | 27.09591 |
| Crls1 | 9.157035 | 10.2657 | 9.560094 | 9.392589 | 8.781031 | 8.765493 | 8.051475 | 8.439003 |
| Gstk1 | 41.52026 | 41.64824 | 40.22304 | 33.21189 | 36.36634 | 33.57137 | 28.98593 | 31.36379 |
| Usp42 | 1.400158 | 1.783707 | 1.692446 | 1.854052 | 1.401825 | 1.782381 | 1.76369 | 2.215468 |
| Sec63 | 13.48366 | 12.11475 | 12.68451 | 13.26216 | 13.96042 | 13.66736 | 14.55563 | 13.94863 |
| Yes1 | 9.760512 | 8.975084 | 8.349561 | 7.680529 | 7.706022 | 7.416955 | 8.562374 | 10.10683 |
| LOC681180 | 0 | 0 | 0 | 0 | 0 | 0 | 0 | 0.076592 |

| | | | | | | | | |
|---|---|---|---|---|---|---|---|---|
| Rpa1 | 23.19808 | 21.95856 | 22.10224 | 24.40893 | 24.32289 | 26.13165 | 25.03879 | 25.99374 |
| Tlr10 | 0.111464 | 0.100893 | 0.287194 | 0.349214 | 0.19284 | 0.27365 | 0.099761 | 0.395185 |
| Zkscan5 | 5.983108 | 7.111191 | 6.482178 | 6.322476 | 6.439772 | 7.748671 | 7.062093 | 7.516817 |
| AABR07018 | 1.818261 | 0.411455 | 0.780808 | 0.670182 | 0.393212 | 0.318851 | 0.813676 | 0.644646 |
| Bcl11a | 1.712177 | 1.434066 | 1.647156 | 1.557214 | 1.202176 | 1.169796 | 1.74137 | 1.419043 |
| Arpc2 | 137.9438 | 137.1886 | 141.1742 | 155.0685 | 149.0299 | 150.429 | 159.1091 | 151.5481 |
| Mir144 | 0.630915 | 0 | 0 | 0 | 0 | 0 | 0 | 0 |
| AABR07054 | 2.990351 | 3.226073 | 3.210333 | 3.78079 | 3.459011 | 3.231697 | 3.112067 | 5.177715 |
| AABR07027 | 0 | 0.327346 | 0 | 0 | 0 | 0.126836 | 0.323672 | 0.128217 |
| Pik3c2a | 6.359978 | 5.885059 | 5.502836 | 5.320842 | 5.51518 | 5.046397 | 7.058903 | 7.110642 |
| Ahnak | 53.71206 | 55.60891 | 51.87597 | 51.60484 | 50.99522 | 51.82689 | 62.83835 | 65.50774 |
| Clec1a | 5.746124 | 5.316118 | 5.248619 | 4.212459 | 3.896123 | 4.648511 | 5.806967 | 5.163422 |
| Ccdc110 | 0 | 0 | 0.019057 | 0 | 0 | 0 | 0 | 0 |
| AABR07012 | 0 | 0 | 0 | 0 | 0 | 0 | 0 | 0 |
| Ogfrl1 | 8.476829 | 8.270615 | 7.484764 | 7.103541 | 7.306131 | 7.028552 | 9.208616 | 8.765674 |
| AABR07072 | 0 | 0 | 0 | 0 | 0.251656 | 0 | 0 | 0 |
| RGD131033 | 0.076727 | 0.303844 | 0.267706 | 0.088376 | 0.186668 | 0.185003 | 0.171677 | 0.170017 |
| AABR07032 | 0.698212 | 1.579989 | 1.499151 | 0.643375 | 2.2649 | 2.448773 | 0 | 0 |
| AABR07066 | 0.043457 | 0 | 0 | 0 | 0 | 0 | 0 | 0 |
| Ece2 | 0.738923 | 0.772958 | 0.538832 | 1.066297 | 1.160792 | 0.806804 | 1.325798 | 1.186314 |
| Rn60_20_( | 0.06332 | 0.071644 | 0 | 0.700166 | 0.273869 | 0.055519 | 0 | 0 |
| Hspb8 | 156.5071 | 153.053 | 157.4299 | 193.5569 | 181.2048 | 190.3133 | 201.6264 | 197.9882 |
| Gabrb2 | 3.16991 | 2.513447 | 2.840384 | 0.827985 | 0.971597 | 1.02859 | 0.726025 | 0.730066 |
| Habp4 | 63.49281 | 65.3083 | 61.5936 | 55.91084 | 53.53948 | 53.74983 | 65.19781 | 66.13915 |
| Pon3 | 0.577433 | 0.408336 | 0.929866 | 2.327856 | 1.677993 | 1.835314 | 2.018769 | 2.49506 |
| AABR07028 | 0.082989 | 0.563387 | 0.71275 | 0.535296 | 1.076815 | 0.654882 | 0 | 2.206712 |
| #N/A | 0 | 0 | 0 | 0 | 0.202223 | 0 | 0 | 0 |
| Fam64a | 0.474065 | 0.453862 | 0.352342 | 0.470434 | 0.55203 | 0.383687 | 0.407971 | 0.452509 |
| Hpcal4 | 0 | 0 | 0 | 0 | 0 | 0.009828 | 0 | 0 |
| Becn2 | 0 | 0 | 0 | 0 | 0 | 0 | 0 | 0.027843 |
| Mir194-1 | 0 | 0 | 0.677327 | 0 | 0 | 0.553187 | 0 | 0 |
| Trim6 | 0.0431 | 0.09753 | 0.09254 | 0.079429 | 0.069904 | 0.03779 | 0.241089 | 0.191006 |
| Mettl2b | 1.928826 | 1.588922 | 1.580284 | 1.605839 | 1.298933 | 1.587351 | 1.949667 | 1.919566 |
| Atg2b | 2.661388 | 2.453145 | 2.589492 | 2.552257 | 2.361958 | 2.623413 | 2.95925 | 3.372631 |
| Acacb | 55.39958 | 58.09274 | 60.70262 | 43.38753 | 42.57053 | 44.00949 | 54.33286 | 55.8107 |
| Tmco5a | 0 | 0.049375 | 0 | 0 | 0 | 0.038262 | 0.048821 | 0.038679 |
| Paqr6 | 26.14935 | 23.95843 | 25.76365 | 34.50192 | 33.72637 | 34.77477 | 35.12072 | 32.47229 |
| Dus4l | 0.740576 | 0.837927 | 0.58988 | 1.364824 | 1.420729 | 1.026373 | 1.042337 | 0.952853 |
| Eif4g3 | 13.32481 | 13.043 | 14.16647 | 12.90044 | 12.60425 | 12.7192 | 14.97386 | 15.83233 |
| Serbp1 | 134.3664 | 135.0275 | 125.3493 | 142.9282 | 135.1476 | 133.0664 | 133.8632 | 138.1929 |
| Esyt1 | 14.92509 | 16.07167 | 16.48722 | 17.57477 | 17.47159 | 18.63258 | 22.48433 | 21.80205 |
| Itgb1bp1 | 19.02387 | 17.01946 | 16.14869 | 18.67863 | 19.09162 | 18.16126 | 17.77338 | 18.67988 |
| Bet1 | 13.60961 | 12.89734 | 12.27455 | 12.28609 | 12.32548 | 12.56894 | 11.82514 | 14.05294 |
| Gba | 5.71531 | 6.107349 | 4.98202 | 7.269479 | 6.813717 | 6.810078 | 8.470731 | 7.360511 |
| Cfap126 | 3.083182 | 2.834386 | 2.379057 | 2.352729 | 2.917075 | 2.787816 | 2.479204 | 3.202474 |
| Sptlc1 | 7.231813 | 6.561588 | 7.022638 | 6.679735 | 7.409076 | 7.279126 | 7.955466 | 7.587871 |
| Serpinb6b | 2.407629 | 2.462184 | 1.491198 | 2.133207 | 1.70218 | 1.623854 | 1.709367 | 1.313231 |
| Rab3a | 11.56922 | 10.80788 | 13.31913 | 11.11646 | 9.95832 | 10.81126 | 9.749919 | 9.276157 |
| Ssh2 | 7.859429 | 8.304095 | 7.879228 | 6.557497 | 6.793273 | 6.565417 | 7.896877 | 7.588185 |
| RGD130842 | 19.07277 | 18.27732 | 18.4537 | 16.04606 | 16.13162 | 17.34644 | 19.34215 | 20.58691 |
| Gtf2h2 | 6.592955 | 6.449462 | 6.008892 | 5.505604 | 7.017479 | 6.47319 | 6.607582 | 6.452379 |
| Tenm2 | 0.011228 | 0 | 0.006027 | 0.010346 | 0.030351 | 0.004922 | 0.025122 | 0.009952 |
| Rrs1 | 13.28591 | 13.27249 | 14.19369 | 11.37651 | 9.705715 | 10.5126 | 10.87587 | 11.51746 |
| AABR07061 | 0 | 0 | 0 | 0 | 0 | 0 | 0 | 0.346377 |

| | | | | | | | | |
|---|---|---|---|---|---|---|---|---|
| AABR07013 | 0 | 0 | 0.010319 | 0.008857 | 0.010393 | 0 | 0.010753 | 0 |
| AC110862. | 0 | 0 | 0 | 0 | 0 | 0 | 0 | 0.760894 |
| AABR07048 | 0 | 0 | 0 | 0 | 0 | 0 | 0 | 0 |
| AABR07058 | 10.96551 | 7.216294 | 6.606835 | 7.320454 | 11.85685 | 6.377012 | 9.263387 | 8.033282 |
| Rictor | 2.823372 | 3.356397 | 3.532835 | 3.155351 | 3.135381 | 2.826793 | 3.77759 | 4.024282 |
| Ccdc94 | 5.776212 | 4.700982 | 5.222006 | 5.882821 | 5.177385 | 5.819833 | 5.66857 | 6.062854 |
| Trim26 | 8.770532 | 7.67413 | 9.666812 | 8.577377 | 7.789073 | 9.022945 | 11.35585 | 11.62954 |
| Lpl | 558.5508 | 545.1574 | 514.8303 | 408.4889 | 414.3269 | 411.3668 | 498.7756 | 505.8436 |
| AABR07059 | 0.168379 | 0 | 0.723063 | 0.620619 | 1.092396 | 0.59054 | 0.37675 | 0.596971 |
| Micall1 | 16.71301 | 17.58654 | 17.84589 | 17.76329 | 17.24457 | 16.74459 | 27.43011 | 29.00907 |
| Zfp646 | 3.159612 | 3.354146 | 3.451905 | 3.159788 | 3.054702 | 3.568863 | 3.711575 | 3.434757 |
| #N/A | 0.157255 | 0.177927 | 0 | 0 | 0 | 0 | 0 | 0 |
| AABR07018 | 0 | 0 | 0 | 0 | 0 | 0.068632 | 0 | 0 |
| AABR07006 | 0 | 0 | 0 | 0 | 0 | 0 | 0 | 0.595058 |
| Rn60_12_( | 0 | 0 | 0 | 0 | 0 | 0 | 0 | 0 |
| Flywch2 | 0.763846 | 0.672199 | 0.728923 | 1.329503 | 0.917707 | 0.669741 | 0.474754 | 0.451357 |
| Tfec | 0.258278 | 0.365287 | 0.346598 | 0.475987 | 0.279273 | 0.367995 | 0.866851 | 0.972931 |
| Fam175a | 1.087182 | 2.050158 | 1.24497 | 1.870018 | 1.959256 | 2.160682 | 1.824436 | 1.509676 |
| Cmpk1 | 18.66202 | 19.24182 | 17.59454 | 17.99795 | 19.05624 | 17.91305 | 19.40264 | 20.894 |
| Fam219a | 8.543144 | 8.697185 | 8.857668 | 8.334108 | 8.514833 | 8.333104 | 10.60927 | 10.29377 |
| Ppp4r1 | 9.230848 | 9.218417 | 9.087957 | 9.730494 | 9.137702 | 9.284226 | 10.69877 | 11.5108 |
| Ctnnbip1 | 6.927145 | 7.184594 | 7.193269 | 6.136127 | 5.573082 | 5.947191 | 5.858467 | 6.688076 |
| AC097153. | 0 | 0.22965 | 0.2179 | 0 | 0 | 0.177963 | 0.113536 | 0 |
| Mtap | 2.757962 | 2.460398 | 2.941869 | 3.225564 | 3.383587 | 3.100235 | 3.481061 | 3.400388 |
| AABR07070 | 0 | 0 | 0 | 0.415975 | 0 | 0 | 0 | 0 |
| Fmnl2 | 1.893314 | 1.999383 | 2.083409 | 2.282538 | 2.456656 | 2.545425 | 3.424352 | 3.02065 |
| Agfg1 | 16.29689 | 16.53269 | 16.98699 | 16.982 | 15.99892 | 16.52829 | 18.76241 | 19.60191 |
| Atp6v1b1 | 0.070463 | 0.079726 | 0.025216 | 0.162323 | 0.126985 | 0.154456 | 0.052554 | 0.176956 |
| Theg | 0 | 0 | 0 | 0.041996 | 0 | 0 | 0 | 0 |
| Ss18l1 | 2.718772 | 3.031581 | 3.426389 | 2.614165 | 3.238007 | 3.040237 | 3.74695 | 3.282893 |
| Gkn3 | 0 | 0 | 0 | 0 | 0.098818 | 0.08013 | 0.204484 | 0.081003 |
| AC115371. | 4.393513 | 5.105406 | 5.354111 | 2.297768 | 1.925936 | 1.978175 | 2.656901 | 1.26298 |
| Becn1 | 48.66003 | 46.48813 | 46.40161 | 56.3077 | 54.61895 | 52.97825 | 53.40218 | 50.23479 |
| RGD130910 | 12.43061 | 12.78281 | 9.865206 | 12.99123 | 12.69243 | 13.35493 | 14.47013 | 13.16566 |
| AABR07050 | 0 | 0 | 0.759705 | 0 | 0 | 0 | 0 | 0 |
| AABR07026 | 0 | 0.264507 | 0 | 0 | 0 | 0 | 0 | 0.103604 |
| Trpc4ap | 35.38238 | 35.75698 | 37.16298 | 33.87692 | 32.28113 | 30.27874 | 35.05766 | 36.12806 |
| Rtn2 | 36.5073 | 37.11136 | 37.7112 | 33.16624 | 32.86154 | 31.74862 | 28.82305 | 26.72133 |
| LOC102549 | 0.053272 | 0.060274 | 0.05719 | 0 | 0.172805 | 0.046709 | 0 | 0.094434 |
| Wdpcp | 0.762416 | 1.112348 | 1.076976 | 1.09078 | 0.650833 | 0.914771 | 1.054973 | 1.049217 |
| Hmx3 | 0 | 0 | 0.041276 | 0 | 0 | 0 | 0 | 0 |
| Tbl3 | 4.482686 | 4.564753 | 4.724951 | 4.656334 | 5.045352 | 4.841566 | 4.809869 | 5.508338 |
| LOC688473 | 0 | 0.073148 | 0 | 0 | 0.139809 | 0 | 0 | 0 |
| Pacrgl | 1.49799 | 1.478535 | 1.300237 | 1.204126 | 1.309589 | 1.285494 | 1.640228 | 1.581992 |
| Alkbh3 | 53.73741 | 53.13652 | 56.08431 | 63.18863 | 59.18401 | 61.32863 | 56.0274 | 53.3767 |
| Mis12 | 7.251963 | 8.396075 | 9.324429 | 8.469552 | 9.573855 | 8.872364 | 7.4528 | 9.49218 |
| AABR07021 | 0.130915 | 0.074062 | 0.070273 | 0 | 0 | 0 | 0.073231 | 0 |
| RGD130614 | 21.82739 | 21.71024 | 20.19328 | 20.72735 | 20.4067 | 20.29937 | 19.78354 | 20.72804 |
| Tfip11 | 7.931963 | 7.196648 | 8.113796 | 7.736499 | 6.667184 | 7.505883 | 9.074847 | 8.317206 |
| Sulf2 | 13.80693 | 14.34374 | 15.40205 | 23.18973 | 22.8553 | 22.97925 | 31.80591 | 32.27433 |
| Hpf1 | 13.56043 | 11.93835 | 12.12466 | 12.74747 | 12.29638 | 12.33523 | 14.64617 | 12.6774 |
| Nmb | 0.257236 | 0.207893 | 0.118354 | 0.033862 | 0.07947 | 0.128883 | 0 | 0.130286 |
| Aacs | 0.754883 | 0.965521 | 0.810415 | 0.952663 | 1.029178 | 1.007212 | 1.174998 | 1.076363 |
| Ppm1e | 2.997247 | 2.872838 | 3.422688 | 3.219221 | 3.32345 | 3.280802 | 2.92603 | 2.97811 |

| | | | | | | | | |
|---|---|---|---|---|---|---|---|---|
| Ctif | 6.017943 | 5.793221 | 5.782956 | 5.428962 | 5.050977 | 5.559429 | 7.172031 | 6.900632 |
| Snx7 | 4.124713 | 5.275647 | 3.850559 | 6.141809 | 3.975211 | 4.219311 | 5.183004 | 4.530184 |
| Ntng1 | 0.518731 | 0.29346 | 0.375902 | 0.346543 | 0.266426 | 0.397971 | 0.290167 | 0.459777 |
| Rit1 | 32.3681 | 29.09923 | 30.23498 | 35.05223 | 33.73031 | 35.45404 | 35.66499 | 34.41002 |
| LOC100912 | 0 | 0 | 0 | 0.14234 | 0 | 0 | 0 | 0 |
| Kcnd1 | 0.268037 | 0.223463 | 0.18174 | 0.376978 | 0.305078 | 0.445291 | 0.347215 | 0.462645 |
| Slc11a2 | 9.342372 | 10.1348 | 9.964365 | 9.673036 | 10.01412 | 10.23481 | 9.114178 | 10.54386 |
| Itm2b | 227.1279 | 235.5466 | 221.381 | 251.6862 | 249.2358 | 251.6009 | 276.8 | 278.9632 |
| AC127764. | 0 | 0 | 0 | 0.054462 | 0 | 0 | 0 | 0 |
| AC114117. | 0 | 0 | 0 | 0 | 0 | 0.553187 | 0 | 0 |
| LOC100912 | 34.04595 | 31.00055 | 34.63596 | 20.96447 | 18.0561 | 19.90102 | 24.60677 | 25.72198 |
| LOC100910 | 0.455873 | 1.794083 | 0.510687 | 0.602708 | 2.893278 | 0.538739 | 2.816144 | 1.001373 |
| Kif17 | 0.212812 | 0.06421 | 0.167543 | 0.261464 | 0.322155 | 0.29855 | 0.269829 | 0.238926 |
| AABR07057 | 0 | 0 | 0.921609 | 0 | 0 | 0 | 0 | 0 |
| Dhx57 | 3.160898 | 3.434384 | 3.160664 | 3.217577 | 3.491866 | 3.461846 | 3.766066 | 4.217663 |
| Hcn1 | 0 | 0 | 0.040708 | 0 | 0 | 0.033247 | 0 | 0.016805 |
| AABR07018 | 0.099745 | 0 | 0 | 0 | 0 | 0 | 0 | 0 |
| LOC102552 | 1.451026 | 1.495833 | 1.730855 | 7.457843 | 8.367859 | 7.350841 | 12.84245 | 14.97611 |
| Ipmk | 2.93459 | 3.452286 | 3.20263 | 2.372811 | 2.721327 | 2.607132 | 3.293961 | 3.419292 |
| AC134056. | 0.775791 | 0 | 0 | 0 | 0 | 0 | 0 | 0 |
| Naa10 | 9.99307 | 9.15905 | 10.42853 | 11.1116 | 10.86573 | 10.62201 | 10.36786 | 9.550109 |
| Sulf1 | 6.127683 | 5.831651 | 5.902169 | 9.699176 | 9.833393 | 9.992326 | 13.48011 | 13.53249 |
| Stag2 | 10.3072 | 10.54923 | 9.437524 | 10.62117 | 10.08149 | 10.16928 | 11.80634 | 11.50601 |
| Rbm25l1 | 4.46413 | 4.085583 | 3.712983 | 4.281992 | 4.217446 | 4.221407 | 5.215859 | 5.226191 |
| AABR07029 | 0 | 0.44886 | 0 | 0 | 0 | 0 | 0 | 0 |
| Tusc5 | 3.177738 | 3.263108 | 3.583514 | 2.928161 | 3.638162 | 3.418417 | 4.929357 | 4.426066 |
| Sbno2 | 14.61019 | 16.12756 | 15.603 | 16.67595 | 16.82529 | 15.7487 | 20.0661 | 19.78558 |
| #N/A | 0 | 0 | 0 | 0 | 0 | 0 | 0 | 0 |
| Slc47a2 | 0.24413 | 0.207166 | 0.196567 | 0.196837 | 0.296971 | 0.508377 | 0.443823 | 0.324577 |
| AABR07036 | 0 | 0 | 0 | 0.317455 | 0.372517 | 0 | 0.385425 | 0.305359 |
| AC122999. | 0 | 0 | 0.480497 | 0 | 0 | 0 | 0 | 0 |
| Helb | 2.106083 | 1.903757 | 2.002964 | 2.436388 | 2.636196 | 2.468844 | 2.676327 | 2.576893 |
| Gabrq | 0.082336 | 0.031053 | 0.029464 | 0.07587 | 0.029676 | 0.024064 | 0 | 0.048653 |
| Inpp1 | 7.555159 | 6.614428 | 6.914249 | 6.094412 | 6.160444 | 6.081389 | 8.202962 | 7.662567 |
| AC142181. | 2.104541 | 2.241119 | 2.32581 | 2.110362 | 1.874031 | 2.116625 | 1.454229 | 1.316724 |
| Aif1 | 11.41091 | 11.44086 | 10.73421 | 16.29259 | 17.28605 | 14.56188 | 17.44268 | 16.02227 |
| Cldn22 | 1.140644 | 1.290585 | 0.946246 | 1.481037 | 1.233362 | 1.136497 | 0.870069 | 0.64337 |
| LOC100361 | 0.07513 | 0 | 0 | 0.138459 | 0.32495 | 0.131749 | 0.420263 | 0 |
| Ostn | 0 | 0 | 0 | 0.055115 | 0 | 0 | 0.033458 | 0 |
| Alox15 | 0.876318 | 0.589548 | 0.88993 | 0.589251 | 0.512189 | 0.436094 | 0.185478 | 0.566799 |
| Mrps14 | 80.38877 | 73.42143 | 71.26032 | 67.42398 | 65.72802 | 64.28028 | 66.89736 | 65.4821 |
| Kcnk1 | 0.128285 | 0.319326 | 0.165267 | 0.023642 | 0.11097 | 0.11248 | 0.172223 | 0.136446 |
| AC113621. | 0 | 0 | 0 | 0 | 0.803156 | 0.651269 | 0.415494 | 0 |
| Bak1 | 12.14348 | 13.53546 | 13.90915 | 14.76712 | 14.88782 | 13.29937 | 12.62601 | 15.52486 |
| Rn50_9_09 | 0.319694 | 0.795782 | 0.205927 | 0.353503 | 0.691362 | 0.392432 | 0.143064 | 0.453377 |
| Kcp | 0.40463 | 0.406951 | 0.651595 | 0.331423 | 0.303834 | 0.3942 | 0.326937 | 0.268983 |
| Olr299 | 0.056307 | 0 | 0 | 0 | 0 | 0 | 0 | 0 |
| #N/A | 0 | 0 | 0 | 0 | 0 | 0 | 0.186575 | 0 |
| Mtm1 | 28.32666 | 27.44064 | 27.91444 | 21.56158 | 21.05758 | 20.12382 | 23.98272 | 23.57593 |
| RGD156430 | 0.147926 | 0 | 0 | 0 | 0 | 0 | 0 | 0 |
| Tp53rk | 2.293806 | 1.360968 | 2.612703 | 2.835387 | 1.905565 | 2.011211 | 2.409733 | 2.405026 |
| Snph | 0.296767 | 0.442617 | 0.376526 | 0.497199 | 0.437577 | 0.603204 | 0.739477 | 0.633686 |
| Fcnb | 0.734674 | 0.738888 | 0.876355 | 1.579604 | 1.941848 | 1.789341 | 0.547947 | 0.94059 |
| AABR07040 | 0.362812 | 0.592952 | 0.779005 | 0.557196 | 0.523072 | 0.70692 | 0.450998 | 0.75035 |

| | | | | | | | | |
|---|---|---|---|---|---|---|---|---|
| Ptk2b | 2.065545 | 1.859778 | 2.186262 | 2.908592 | 2.658112 | 3.226768 | 3.319798 | 3.300588 |
| Dppa4 | 1.10244 | 1.316657 | 1.052036 | 1.015855 | 1.192053 | 1.87954 | 1.096321 | 1.574294 |
| Plekha5 | 7.985154 | 8.243641 | 8.475708 | 6.069315 | 6.146402 | 6.101494 | 7.141702 | 6.777739 |
| AABR07068 | 0 | 0 | 0 | 0 | 0 | 0 | 0.563314 | 0 |
| AABR07036 | 0 | 0 | 0.520539 | 0 | 0 | 0 | 0 | 0.429764 |
| Phf23 | 9.354387 | 8.93139 | 9.041615 | 9.650627 | 9.644309 | 10.00037 | 10.77819 | 11.2111 |
| Rad51b | 1.64932 | 1.477352 | 0.590217 | 0.633243 | 1.188924 | 1.325615 | 1.230124 | 1.400963 |
| Trim44 | 8.514171 | 7.459479 | 8.574281 | 8.192619 | 6.925055 | 8.489225 | 9.188099 | 9.516646 |
| Ppif | 44.12739 | 43.00695 | 39.81962 | 37.27318 | 36.39746 | 36.70679 | 40.22998 | 41.08672 |
| Emilin2 | 2.984922 | 3.542493 | 3.413494 | 3.662335 | 3.525751 | 3.484835 | 5.825799 | 6.053442 |
| Pcgf5 | 9.558268 | 9.299827 | 7.426547 | 8.019342 | 8.766838 | 8.250259 | 8.238469 | 9.16423 |
| Ctps1 | 27.16567 | 26.35516 | 26.44823 | 31.93565 | 30.76258 | 31.32627 | 29.60801 | 33.15076 |
| AC099450. | 7.824793 | 8.172355 | 9.046601 | 8.319506 | 10.41334 | 8.444043 | 10.1008 | 9.603004 |
| Dpp6 | 0.01171 | 0 | 0.025142 | 0 | 0.012662 | 0.030801 | 0.039301 | 0.031137 |
| Kcnb1 | 6.983169 | 8.362766 | 9.417427 | 5.234532 | 5.616437 | 5.311055 | 8.075801 | 8.317615 |
| Gatm | 7.395029 | 7.0312 | 7.205176 | 6.203429 | 6.809036 | 6.647429 | 6.187542 | 7.050307 |
| Ehhadh | 4.086602 | 3.859534 | 3.752712 | 2.271834 | 2.465024 | 2.546692 | 3.02275 | 2.379848 |
| Psma6 | 116.3256 | 110.1515 | 105.1113 | 112.9144 | 118.5234 | 111.4302 | 114.749 | 114.5121 |
| Zfp354b | 0.539443 | 0.47472 | 0.450432 | 0.607537 | 0.756119 | 0.525538 | 0.402337 | 0.69064 |
| AABR07000 | 0 | 0 | 0 | 0 | 0 | 0.105551 | 0 | 0.1067 |
| AABR07049 | 0.070669 | 0.079959 | 0.151736 | 0.195357 | 0.076414 | 0.123926 | 0.632493 | 0.187913 |
| Akr1c1 | 191.6269 | 186.1874 | 175.3058 | 203.2668 | 205.0273 | 209.7811 | 193.8106 | 201.2578 |
| Nudt15 | 4.966791 | 4.37087 | 3.159803 | 2.03409 | 3.049925 | 3.387134 | 3.292815 | 3.043575 |
| Lrrc3c | 0 | 0 | 0 | 0 | 0.147454 | 0.059784 | 0 | 0 |
| Lonp1 | 47.2268 | 49.22427 | 48.93168 | 43.87688 | 41.73395 | 43.16428 | 49.56402 | 50.02715 |
| AC141521. | 0.38301 | 0.385207 | 0.342654 | 0.549 | 0.322111 | 0.410451 | 0.309468 | 0.622381 |
| AABR07028 | 0.076224 | 0 | 0.081831 | 0.210712 | 0 | 0 | 0.085276 | 0.202683 |
| LOC103690 | 0.015325 | 0 | 0 | 0.028243 | 0.049712 | 0.013437 | 0 | 0.04075 |
| Il19 | 0 | 0.112215 | 0.106474 | 0 | 0.10724 | 0.086959 | 0.332867 | 0 |
| AABR07055 | 0.048986 | 0 | 0 | 0.045139 | 0 | 0 | 0.16441 | 0 |
| Fam76a | 7.996766 | 6.713006 | 7.144968 | 8.034266 | 5.969073 | 6.830628 | 8.657832 | 8.41406 |
| Gal | 0.682494 | 1.474219 | 0.732701 | 0.857579 | 0.737971 | 0.326406 | 2.429459 | 2.144747 |
| AABR07002 | 19.37113 | 19.50901 | 18.51086 | 24.32272 | 24.39832 | 21.27744 | 23.69583 | 23.67895 |
| LOC100911 | 0.177061 | 0.250421 | 0.522739 | 0.203944 | 0.047863 | 0.388119 | 0.792354 | 0.196173 |
| Mir6334 | 8.463179 | 5.386325 | 10.78934 | 6.33627 | 10.86695 | 6.029175 | 5.917643 | 9.845504 |
| AC098981. | 0 | 0 | 0 | 0 | 0 | 0 | 0 | 0.390038 |
| Ccdc40 | 0.014288 | 0 | 0.046018 | 0.013166 | 0.046349 | 0.012528 | 0.015985 | 0.037993 |
| Fam149a | 1.62085 | 1.801361 | 1.822457 | 2.032641 | 2.022233 | 1.740714 | 2.961423 | 2.303724 |
| Rn50_6_09 | 0 | 0.767662 | 0 | 0.397847 | 0 | 0.054081 | 0.069004 | 0 |
| Frmd6 | 12.21079 | 12.20108 | 11.2485 | 10.32281 | 10.72925 | 11.62015 | 10.99967 | 11.5961 |
| Tmem231 | 0.579697 | 0.556521 | 0.528047 | 1.003588 | 0.835757 | 0.785521 | 0.668191 | 0.887497 |
| Msln | 0.263869 | 0.217131 | 0.386291 | 0.884162 | 0.674386 | 0.799244 | 0.295204 | 0.403974 |
| Map9 | 0.429781 | 0.35279 | 0.569962 | 0.543566 | 0.61051 | 0.531999 | 0.603382 | 0.470569 |
| Barhl1 | 0 | 0 | 0 | 0 | 0 | 0.019203 | 0 | 0.019412 |
| Lkaaear1 | 0 | 0 | 0 | 0 | 0 | 0 | 0 | 0 |
| Icmt | 6.586908 | 7.385024 | 7.8429 | 7.504204 | 7.219451 | 6.562962 | 9.479394 | 8.492079 |
| AABR07052 | 0 | 0 | 0 | 0 | 0 | 0.468515 | 0 | 0 |
| Plekhj1 | 33.32377 | 36.33974 | 34.88933 | 37.54971 | 33.97351 | 39.68125 | 44.45333 | 41.68868 |
| Arsi | 0.24328 | 0.275259 | 0.097941 | 0.336259 | 0.295936 | 0.266635 | 0.442277 | 0.215631 |
| AABR07044 | 1.11893 | 1.772423 | 1.441491 | 4.33041 | 3.387672 | 1.962157 | 4.256151 | 3.7687 |
| AABR07037 | 0 | 0 | 0 | 0.027277 | 0 | 0 | 0 | 0.026238 |
| Atp6v1d | 70.29336 | 69.04819 | 68.94762 | 77.82403 | 71.76269 | 70.71682 | 75.4719 | 75.97059 |
| Rac3 | 1.636435 | 1.33723 | 2.04962 | 1.172819 | 1.769454 | 2.072529 | 1.93248 | 1.611615 |
| Myb | 0.051139 | 0.057861 | 0.054901 | 0 | 0.027648 | 0.022419 | 0.028606 | 0.022663 |

| | | | | | | | | |
|---|---|---|---|---|---|---|---|---|
| Syn3 | 0.09256 | 0.340364 | 0.273265 | 0.298517 | 0.25021 | 0.243471 | 0.12944 | 0.123061 |
| LOC690784 | 0.037511 | 0.16977 | 0.080542 | 0.103696 | 0.040561 | 0.03289 | 0 | 0.033248 |
| AABR07037 | 3.665614 | 4.14747 | 2.998302 | 3.538563 | 4.341059 | 3.367062 | 3.319798 | 4.486737 |
| Klhl6 | 1.124599 | 0.978793 | 0.8668 | 1.102701 | 1.387501 | 1.112466 | 1.242021 | 1.367388 |
| RGD130783 | 4.565856 | 4.708878 | 4.901738 | 5.398692 | 5.767108 | 4.676475 | 4.565626 | 6.518088 |
| Slc16a8 | 0.31494 | 0.421129 | 0.307371 | 0.23744 | 0.309582 | 0.251036 | 0.160155 | 0.25377 |
| Armc1 | 14.17455 | 14.18183 | 15.17213 | 16.51071 | 16.73661 | 14.93604 | 17.31659 | 16.03072 |
| AABR07033 | 0 | 0 | 0.685587 | 0 | 0 | 0.559933 | 0 | 0.283015 |
| Maml2 | 3.766888 | 3.400329 | 3.31475 | 3.224463 | 2.871189 | 2.977944 | 3.36217 | 3.320535 |
| Rn60_12_( | 0.104315 | 0.118027 | 0 | 0.096122 | 0 | 0.091463 | 0 | 0 |
| Fzd7 | 1.169877 | 1.449724 | 1.255938 | 2.566656 | 2.20868 | 1.904963 | 2.47219 | 3.077842 |
| AABR07059 | 5.339132 | 6.120989 | 5.390263 | 3.942356 | 4.243821 | 3.410259 | 4.074423 | 2.820599 |
| Rn60_20_( | 0 | 0 | 0 | 0 | 0 | 0.143036 | 0 | 0 |
| Foxc1 | 2.724239 | 2.978114 | 3.277862 | 2.934722 | 2.12032 | 2.642477 | 3.253886 | 3.219504 |
| B9d1 | 0.121781 | 0.206685 | 1.176659 | 0.6733 | 0.06584 | 0.533889 | 0.136243 | 0.377793 |
| AABR07060 | 0 | 0.784763 | 0.372306 | 0 | 0 | 0 | 0 | 0 |
| AABR07064 | 0.149938 | 0.031809 | 0.050303 | 0.025905 | 0.121595 | 0.02465 | 0.083872 | 0.041531 |
| Nkx2-8 | 0 | 0 | 0 | 0 | 0 | 0 | 0 | 0 |
| Coil | 3.436137 | 2.955658 | 3.020162 | 2.832974 | 2.889791 | 2.607576 | 3.147296 | 2.493489 |
| Lgals7 | 0.635288 | 1.951026 | 1.07175 | 0.752649 | 0.785061 | 1.114043 | 1.218399 | 0.72397 |
| Rbm4 | 11.81019 | 14.11905 | 13.25313 | 15.85167 | 14.60138 | 13.75481 | 17.00202 | 15.56368 |
| Cerkl | 0.087131 | 0.246462 | 0.093541 | 0.120432 | 0.094214 | 0.190992 | 0.048739 | 0.193072 |
| AABR07031 | 0 | 0 | 0 | 0 | 0 | 0 | 0 | 0 |
| RGD156545 | 0 | 0 | 0 | 0 | 0 | 0.12443 | 0 | 0 |
| Cldn2 | 0.091806 | 0 | 0 | 0 | 0.019854 | 0 | 0.020542 | 0.016274 |
| Adprhl1 | 19.17577 | 20.72962 | 21.63837 | 20.50443 | 19.74889 | 20.63954 | 33.51155 | 34.39683 |
| Smyd4 | 2.118412 | 2.621052 | 2.143358 | 2.668247 | 2.752025 | 2.672554 | 2.574588 | 2.728677 |
| Slc2a13 | 0.833611 | 0.733593 | 0.813577 | 1.295751 | 1.065257 | 1.107441 | 1.05507 | 1.223988 |
| Dio1 | 0 | 0.036327 | 0.034469 | 0.029585 | 0 | 0 | 0 | 0 |
| Rps6kb2 | 10.36409 | 11.85873 | 11.87943 | 13.35578 | 11.54358 | 13.18675 | 14.73335 | 13.74478 |
| AABR07071 | 0.025249 | 0 | 0 | 0 | 0 | 0 | 0 | 0 |
| AC096809. | 19.76218 | 25.05709 | 30.46182 | 17.85579 | 14.0517 | 15.50709 | 24.25973 | 23.44581 |
| Rn60_7_12 | 4.064221 | 4.42161 | 5.202278 | 3.745019 | 3.887516 | 3.426454 | 3.147833 | 3.186668 |
| Fubp1 | 14.63977 | 15.01298 | 13.36514 | 15.10179 | 15.98312 | 14.66003 | 15.39104 | 15.76948 |
| Nkain2 | 0 | 0 | 0 | 0.074927 | 0.175846 | 0.071296 | 0 | 0 |
| Gba3 | 0.178419 | 0.168227 | 0.15962 | 0.575421 | 0.482304 | 0.495386 | 0.598821 | 0.737993 |
| Rbm38 | 71.8675 | 81.17437 | 88.40464 | 52.70991 | 50.85634 | 55.94554 | 68.43549 | 68.15157 |
| Dusp26 | 1.878308 | 1.795442 | 1.634047 | 3.252685 | 2.836378 | 3.03825 | 3.07959 | 2.669481 |
| Oser1 | 11.98504 | 10.70952 | 10.99393 | 11.57956 | 13.30856 | 10.96169 | 14.89012 | 12.34094 |
| LOC10036( | 0.285567 | 0.24233 | 0.459862 | 0.723633 | 0.424572 | 0.625964 | 0.439285 | 0.569503 |
| AC113621. | 0 | 0 | 0 | 0 | 0 | 0 | 0 | 0 |
| AABR07043 | 0 | 0 | 0 | 0 | 0 | 0 | 0 | 0 |
| Soga3 | 0.201795 | 0.142701 | 0.2708 | 0.337028 | 0.340935 | 0.187993 | 0.18343 | 0.268292 |
| Acy1 | 15.98224 | 17.36885 | 16.01647 | 15.61491 | 14.01192 | 14.04237 | 12.60165 | 14.51926 |
| Arap2 | 2.222033 | 2.178909 | 2.592238 | 1.883715 | 2.066281 | 1.961269 | 1.80643 | 1.562469 |
| Ppp1r1a | 10.88562 | 11.58981 | 11.2146 | 5.918719 | 5.921786 | 6.04684 | 5.484039 | 5.753123 |
| Rpap3 | 12.50599 | 11.87111 | 13.14942 | 14.41552 | 15.24452 | 14.06593 | 13.93875 | 14.63427 |
| AABR07014 | 0 | 0 | 0 | 0.079233 | 0.092976 | 0 | 0 | 0 |
| Anapc7 | 9.949099 | 9.666021 | 9.171474 | 9.344383 | 8.984044 | 8.480544 | 9.605216 | 10.0269 |
| Col5a2 | 13.83339 | 13.56316 | 14.21133 | 23.29759 | 23.48533 | 22.30161 | 27.18789 | 27.01818 |
| AABR07070 | 0 | 0.459299 | 0.4358 | 0 | 0.438934 | 0 | 0 | 0 |
| Spryd3 | 5.813615 | 6.356935 | 6.427594 | 7.775671 | 6.708384 | 7.094492 | 7.207807 | 7.421708 |
| Mir219-1 | 0 | 0 | 0 | 0.438665 | 0.51475 | 0 | 0 | 0.8439 |
| Igfbp5 | 61.08425 | 61.40295 | 62.3027 | 76.7898 | 77.24691 | 79.17856 | 101.8265 | 103.8807 |

| | | | | | | | | |
|---|---|---|---|---|---|---|---|---|
| Wbp11 | 39.69675 | 43.07198 | 43.65327 | 30.63073 | 30.85536 | 32.90185 | 46.1208 | 44.70338 |
| Tmprss11f | 0 | 0.015038 | 0.014269 | 0 | 0 | 0 | 0.044608 | 0 |
| Rpl13l4 | 11.58325 | 12.28678 | 13.98977 | 12.45242 | 11.742 | 11.31993 | 8.504226 | 11.87099 |
| LOC100911 | 0 | 0 | 0 | 0.043708 | 0.034192 | 0.083178 | 0 | 0.014014 |
| Adcy6 | 30.62007 | 31.16759 | 33.45434 | 22.87829 | 23.89768 | 24.22291 | 35.89615 | 35.7065 |
| Arl14epl | 0.024759 | 0 | 0 | 0 | 0 | 0 | 0 | 0 |
| LOC100911 | 0.259958 | 0.211774 | 0.256755 | 0.450337 | 0.393524 | 0.337338 | 0.535126 | 0.414745 |
| Pias1 | 7.171129 | 6.19991 | 6.624433 | 6.849399 | 7.058493 | 7.457448 | 7.276439 | 6.271661 |
| AABR07029 | 0 | 0 | 0 | 0.095551 | 0 | 0 | 0 | 0 |
| AABR07030 | 0.122065 | 0 | 0 | 0.112478 | 0.263974 | 0 | 0 | 0.108192 |
| Tomm40 | 44.02402 | 44.84636 | 45.65784 | 41.45504 | 42.2844 | 38.38891 | 47.90348 | 43.80744 |
| Bmp3 | 0.019998 | 0 | 0.010735 | 0.101353 | 0.097308 | 0.052604 | 0.13424 | 0.088628 |
| Uqcrc2 | 480.0209 | 467.7923 | 452.7966 | 471.976 | 473.0339 | 468.0811 | 419.3108 | 418.8503 |
| Bbip1 | 21.10268 | 18.57076 | 24.33323 | 20.64562 | 18.59247 | 17.13227 | 18.65382 | 22.86088 |
| Tnfrsf12a | 6.253459 | 6.767864 | 7.238891 | 14.63127 | 15.0523 | 13.44536 | 24.39507 | 21.06246 |
| Pglyrp2 | 0.031338 | 0.14183 | 0.033643 | 0 | 0 | 0 | 0.03506 | 0.027776 |
| Fosb | 2.47985 | 2.823048 | 2.417283 | 2.060782 | 2.204363 | 2.214353 | 2.825408 | 3.26331 |
| Tnfsf12 | 24.99128 | 26.65846 | 27.45113 | 27.82869 | 26.61774 | 26.98745 | 33.33003 | 30.90277 |
| AABR07050 | 0 | 0 | 0.413369 | 0 | 0.832684 | 0.337607 | 0 | 0 |
| Kbtbd4 | 8.848361 | 8.479494 | 9.059812 | 9.430108 | 9.261169 | 8.75219 | 8.736619 | 9.377798 |
| Ppp1r14a | 12.37101 | 14.07674 | 14.71482 | 18.13541 | 18.16481 | 20.09144 | 15.64879 | 13.5817 |
| Brd8 | 7.623174 | 7.071007 | 7.227818 | 7.441749 | 7.149225 | 7.58403 | 8.460916 | 8.60321 |
| AABR07021 | 0.498723 | 0.282141 | 0.713881 | 0.382961 | 0.89877 | 0.21864 | 0.32547 | 0.368369 |
| Plcb2 | 0.545478 | 0.700586 | 0.553951 | 0.692824 | 0.765169 | 0.762656 | 1.05558 | 0.770962 |
| Wdr3 | 5.218229 | 4.882636 | 4.238541 | 5.372427 | 5.361103 | 4.682687 | 4.793602 | 5.086337 |
| Dnajc24 | 4.028148 | 4.632375 | 3.190186 | 3.224989 | 4.141369 | 3.705583 | 3.028968 | 3.511817 |
| AABR07065 | 0 | 0 | 0 | 0.135163 | 0 | 0 | 0 | 0 |
| Ttll12 | 4.081589 | 4.814642 | 4.684847 | 4.281165 | 3.591727 | 3.616813 | 4.129102 | 5.214898 |
| Igf1 | 9.781885 | 9.073202 | 8.089478 | 12.00755 | 12.89424 | 14.33502 | 19.79891 | 21.29247 |
| Smurf2 | 7.993119 | 7.617242 | 7.885693 | 8.569808 | 8.692875 | 8.763003 | 9.770582 | 10.98077 |
| Serpinf2 | 0.108463 | 0.147265 | 0.209595 | 0.159911 | 0.070368 | 0.05706 | 0.266956 | 0.211499 |
| Arntl2 | 0 | 0.109519 | 0.069277 | 0.594617 | 0.418651 | 0.396058 | 0.324869 | 0.371774 |
| Fuom | 0.236202 | 0.160351 | 0.202862 | 0.13059 | 5.005869 | 0.082841 | 0.819181 | 0.083743 |
| AABR07008 | 0.40437 | 0.915051 | 1.51941 | 1.67675 | 0.437239 | 1.595484 | 0 | 0.53762 |
| Ddx24 | 14.72559 | 15.67382 | 13.91499 | 17.23082 | 15.44068 | 15.84213 | 15.95497 | 14.43459 |
| Smyd1 | 69.12985 | 69.96199 | 72.17196 | 49.91704 | 52.11065 | 53.50985 | 54.59894 | 58.05059 |
| AC113837. | 27.47065 | 29.13914 | 33.17793 | 44.29796 | 54.76604 | 42.151 | 46.09941 | 43.37094 |
| Mcm8 | 0.535671 | 0.716284 | 0.575077 | 0.867539 | 0.965356 | 1.02475 | 0.799047 | 0.589893 |
| AABR07060 | 0.032811 | 0.111371 | 0 | 0.060468 | 0.035478 | 0.086305 | 0.183536 | 0.058164 |
| Acsl1 | 249.5281 | 246.0989 | 235.8841 | 173.8608 | 169.7214 | 173.3757 | 176.548 | 180.5958 |
| AC132720. | 7.854888 | 7.596099 | 7.135382 | 7.8566 | 7.694854 | 6.887173 | 9.013026 | 10.2945 |
| C2cd4b | 5.989064 | 5.386325 | 5.935055 | 7.263913 | 5.479598 | 5.700039 | 8.475592 | 7.985293 |
| Gsdmc | 0.034248 | 0 | 0 | 0.031559 | 0.037032 | 0 | 0 | 0 |
| Gad1 | 0.352897 | 0.221825 | 0.189428 | 0.216787 | 0.190791 | 0.29223 | 0.131602 | 0.173772 |
| Usp2 | 40.97823 | 42.54238 | 43.52374 | 26.38089 | 25.25654 | 26.20037 | 30.5305 | 31.82931 |
| Med25 | 17.10275 | 19.40188 | 22.34714 | 18.80773 | 18.59029 | 18.1527 | 23.48925 | 21.74122 |
| Mum1 | 5.558877 | 4.772271 | 4.922863 | 5.580701 | 4.817942 | 5.537807 | 6.146429 | 5.770661 |
| Sgta | 27.5021 | 29.6474 | 30.79124 | 30.24109 | 29.37023 | 32.52568 | 33.27251 | 32.96848 |
| Kctd5 | 4.467937 | 4.983385 | 4.569289 | 4.682876 | 4.602153 | 4.158854 | 5.543393 | 4.410599 |
| Gtf3c2 | 11.54378 | 11.48125 | 11.95989 | 12.3671 | 12.66667 | 13.42744 | 14.99767 | 15.40274 |
| AABR07004 | 0 | 0 | 0 | 0.065384 | 0 | 0 | 0.039692 | 0 |
| Gdpd3 | 0 | 0.29924 | 0.227144 | 0.097481 | 0.400361 | 0.046378 | 0.059176 | 0.093767 |
| St3gal2 | 10.74631 | 10.61379 | 11.7812 | 11.84981 | 10.86393 | 11.01891 | 14.16872 | 13.48773 |
| Scfd1 | 13.20129 | 13.08681 | 11.29079 | 12.99642 | 13.3245 | 11.61769 | 14.65982 | 14.08008 |

| | | | | | | | | |
|---|---|---|---|---|---|---|---|---|
| Hccs | 25.44049 | 24.362 | 23.81887 | 25.93757 | 25.50138 | 25.73356 | 23.91759 | 23.92341 |
| AABR0707( | 0 | 0.834501 | 0 | 2.038865 | 1.595 | 0.646683 | 0 | 3.268628 |
| Bcl2l10 | 0 | 0 | 0 | 0.045181 | 0.106035 | 0 | 0 | 0.043459 |
| Ddost | 34.00538 | 37.20633 | 36.85554 | 37.15546 | 35.87582 | 37.58051 | 44.02106 | 45.39413 |
| Erp44 | 18.71405 | 16.97706 | 17.2565 | 16.19729 | 17.8865 | 17.22892 | 18.5811 | 19.28261 |
| B3gntl1 | 2.425953 | 2.705639 | 2.120738 | 3.00185 | 2.698095 | 2.947522 | 2.597732 | 2.979622 |
| AC126960. | 0.108418 | 0 | 0 | 0 | 0.468923 | 0.095061 | 0 | 0.192193 |
| Tyrobp | 23.62111 | 23.18889 | 26.38431 | 30.00817 | 29.57892 | 31.29495 | 32.74135 | 34.6377 |
| AABR07018 | 0 | 0.179544 | 0 | 0 | 0 | 0 | 0 | 0 |
| Slc46a1 | 0.75826 | 0.883934 | 0.740037 | 0.973955 | 0.670824 | 1.148366 | 1.490966 | 1.364534 |
| Chga | 0 | 0 | 0 | 0 | 0.030086 | 0 | 0 | 0 |
| AABR07051 | 0 | 0 | 0 | 0.134036 | 0.157285 | 0.382621 | 0 | 0 |
| Cav3 | 71.87252 | 69.6719 | 75.62236 | 68.15354 | 67.29416 | 69.05837 | 64.21364 | 60.06786 |
| Blvra | 10.95482 | 11.16606 | 9.428835 | 11.74783 | 10.72203 | 8.156135 | 9.350303 | 10.12833 |
| Cchcr1 | 5.560248 | 5.571275 | 5.167438 | 6.01571 | 5.443897 | 5.845426 | 5.972974 | 6.154276 |
| LOC691352 | 0.041893 | 0 | 0.269847 | 0.038603 | 0.045298 | 0.073463 | 0.046868 | 0.074263 |
| Adamts5 | 6.504572 | 7.173298 | 7.42504 | 6.084751 | 5.41297 | 5.8476 | 6.337461 | 6.407539 |
| Tmem42 | 9.482013 | 10.63121 | 10.17955 | 9.027665 | 8.425232 | 9.017123 | 10.7042 | 10.38487 |
| Dnajb13 | 0.185942 | 0.262981 | 0.124763 | 0.278425 | 0.251321 | 0.101896 | 0.468053 | 0.432625 |
| Slc2a10 | 0.305861 | 0.25955 | 0.342043 | 0.634137 | 0.509864 | 0.603403 | 0.48476 | 0.802003 |
| AABR07001 | 0.145866 | 0.055014 | 0 | 0 | 0.210297 | 0 | 0.108792 | 0 |
| Anlnl1 | 0.215276 | 0.142085 | 0.038519 | 0.099184 | 0.09699 | 0.047189 | 0.120421 | 0.095405 |
| AABR07051 | 0.166594 | 0.062831 | 0 | 0 | 0 | 0 | 0.186378 | 0.14766 |
| Olr12 | 0 | 0 | 0 | 0 | 0 | 0.049691 | 0 | 0 |
| Ufsp2 | 11.25049 | 10.49211 | 9.076891 | 10.55537 | 10.91163 | 12.07647 | 12.85354 | 11.96624 |
| Slc25a2 | 0.831205 | 1.187961 | 0.704488 | 0.765923 | 1.182592 | 0.805517 | 0.734144 | 0.697963 |
| Dppa3-ps2 | 0 | 0 | 0 | 0 | 0 | 0 | 0 | 0 |
| AABR07056 | 0.129779 | 0.07342 | 0 | 0.119586 | 0.140328 | 0.227581 | 0.290382 | 0.057515 |
| Pold2 | 14.54069 | 14.03376 | 13.94155 | 15.24883 | 13.97179 | 15.61717 | 13.87627 | 15.58632 |
| AABR07011 | 0.277068 | 0 | 0 | 0.510615 | 0 | 0 | 0 | 0 |
| Cideb | 0.042231 | 0.143346 | 0.136012 | 0.077828 | 0.13699 | 0.037028 | 0.094491 | 0.149724 |
| AABR0706( | 0.068993 | 0.234188 | 0.148137 | 0.254298 | 0.298406 | 0.120987 | 0.46312 | 0.366913 |
| Car12 | 0.060843 | 0.022947 | 0.108866 | 0.056065 | 0 | 0.017783 | 0.045379 | 0.035952 |
| Olr60 | 0 | 0.061526 | 0 | 0.050107 | 0 | 0 | 0 | 0 |
| Pcca | 62.20393 | 61.19484 | 56.70523 | 62.62126 | 61.19259 | 61.97304 | 58.18395 | 56.80646 |
| Mrpl39 | 18.64662 | 18.51916 | 16.79316 | 16.56166 | 20.49836 | 15.214 | 18.77491 | 14.78286 |
| AABR07037 | 0 | 0.116863 | 0 | 0 | 0 | 0 | 0 | 0 |
| Nktr | 6.918624 | 7.153057 | 6.045447 | 7.330079 | 6.767992 | 7.1033 | 9.356216 | 9.008294 |
| Scnn1a | 0.310593 | 0.140568 | 0.200065 | 0.372059 | 0.302255 | 0.313177 | 0.295356 | 0.275294 |
| AC119762. | 0 | 0 | 0 | 0 | 0 | 0.637701 | 0 | 0 |
| AABR07027 | 0.375383 | 0.849456 | 0.604496 | 0.17295 | 0.202948 | 0 | 0.629943 | 0.332721 |
| Sfxn5 | 3.606411 | 4.292764 | 3.491255 | 2.785312 | 3.245877 | 2.741709 | 2.751987 | 3.122633 |
| Gapdhs | 0.769548 | 0.704858 | 0.708136 | 0.540273 | 0.356615 | 0.61048 | 0.778942 | 0.617128 |
| Appbp2 | 3.781436 | 3.888717 | 3.293483 | 3.371068 | 4.106551 | 4.041971 | 4.368155 | 4.340455 |
| Mt-nd6 | 3832.298 | 3830.217 | 3635.766 | 3701.824 | 3759.015 | 3665.551 | 3463.494 | 3371 |
| AABR07052 | 1.309148 | 0 | 2.108181 | 0 | 0 | 0 | 0 | 0 |
| Lnp | 5.387114 | 5.736721 | 5.01796 | 5.146514 | 5.396699 | 5.070737 | 5.672343 | 4.599321 |
| Coq4 | 3.757047 | 4.175684 | 3.640795 | 4.718084 | 4.529801 | 4.576873 | 4.314807 | 4.980351 |
| Mmaa | 3.379606 | 4.047602 | 3.28084 | 3.180433 | 2.954556 | 3.656766 | 3.338501 | 3.521321 |
| Abhd17c | 6.590842 | 6.640743 | 7.617987 | 6.804645 | 6.398318 | 5.799946 | 7.292809 | 7.163656 |
| Pmpca | 59.2156 | 58.44437 | 62.58488 | 58.08956 | 61.27159 | 58.49807 | 66.28095 | 67.46663 |
| Rnaset2 | 30.57356 | 31.26051 | 29.14377 | 36.01717 | 30.04814 | 30.37492 | 32.40726 | 34.88208 |
| AABR07034 | 0.252975 | 0 | 0.090528 | 0 | 0 | 0 | 0 | 0 |
| Akr1b7 | 0.037008 | 0.16749 | 0.07946 | 0.068202 | 0.160064 | 0.032448 | 0.082805 | 0.098405 |

| | | | | | | | | |
|---|---|---|---|---|---|---|---|---|
| AABR07032 | 0 | 0.103583 | 0 | 0 | 0 | 0 | 0 | 0 |
| AABR07056 | 0 | 0 | 0 | 1.391917 | 0 | 0 | 0 | 0 |
| Dkc1 | 34.59644 | 35.91359 | 41.78417 | 41.45836 | 38.06807 | 41.6852 | 45.83808 | 45.13683 |
| LOC361559 | 0.350642 | 0.249376 | 0.172085 | 0.461576 | 0.259985 | 0.298659 | 0.560404 | 0.488387 |
| Lmo4 | 40.21671 | 37.70015 | 39.04278 | 23.83099 | 22.45824 | 23.73069 | 33.59871 | 30.9904 |
| Zfp69 | 0.021576 | 0.341778 | 0.092655 | 0.119291 | 0.326623 | 0.189182 | 0.362081 | 0.13387 |
| Cdkl2 | 0.791578 | 1.102318 | 0.762649 | 1.776763 | 1.975204 | 1.886409 | 1.975529 | 2.032884 |
| Hsd17b4 | 52.24381 | 48.91026 | 51.82646 | 48.3469 | 46.60959 | 48.85887 | 46.76755 | 46.12589 |
| Tiparp | 6.295478 | 6.242096 | 6.042139 | 7.748379 | 7.047747 | 6.924232 | 7.192425 | 6.427839 |
| RGD156111 | 24.72835 | 35.50272 | 32.79393 | 31.21135 | 38.8718 | 38.07987 | 34.87182 | 32.78486 |
| Dhx35 | 4.195024 | 4.066292 | 4.784226 | 3.973929 | 3.914259 | 4.021958 | 3.713627 | 4.529093 |
| Lamp5 | 0 | 0 | 0 | 0 | 0 | 0 | 0 | 0.022619 |
| Cdh24 | 0.446427 | 0.404089 | 0.311524 | 0.452502 | 0.62753 | 0.371856 | 0.424527 | 0.593536 |
| Reck | 4.643795 | 4.637817 | 4.344827 | 5.53411 | 6.031132 | 5.800443 | 7.6623 | 7.576707 |
| Mfsd3 | 0 | 0.027179 | 0.025788 | 0.022134 | 0.025974 | 0 | 0 | 0.021291 |
| Cyp4f37 | 0.340776 | 0.64262 | 0.414624 | 0.628023 | 0.786083 | 0.916298 | 0.432078 | 0.583957 |
| AABR07068 | 0 | 0 | 0.086357 | 0 | 0.086978 | 0 | 0 | 0.499081 |
| Lrrc34 | 0.134478 | 0.060862 | 0.057748 | 0.049567 | 0.174492 | 0.023582 | 0.180538 | 0.023839 |
| AABR07012 | 1.358286 | 1.102906 | 1.080789 | 1.251607 | 1.174956 | 1.134902 | 1.430202 | 1.246408 |
| Agr3 | 0 | 0 | 0 | 0.02964 | 0 | 0.028203 | 0.035986 | 0 |
| RGD131194 | 2.142914 | 2.731166 | 2.538544 | 2.723601 | 2.343735 | 2.980338 | 3.196529 | 2.859973 |
| Smchd1 | 4.435579 | 4.487262 | 3.40801 | 4.295579 | 4.203664 | 4.415294 | 5.370991 | 5.164877 |
| Rad9b | 1.607061 | 1.166465 | 1.269547 | 1.620545 | 1.213105 | 1.701521 | 1.526526 | 0.994405 |
| Mex3b | 0.988619 | 1.258398 | 1.094513 | 0.711698 | 1.25271 | 0.934543 | 0.950489 | 1.02687 |
| AABR07010 | 0 | 0 | 0 | 0 | 0 | 0 | 0 | 0.067365 |
| AABR07063 | 8.015192 | 6.247404 | 8.222384 | 10.17583 | 9.437085 | 7.652414 | 9.365576 | 9.472351 |
| LOC686074 | 0 | 1.123976 | 0.457058 | 0.523069 | 0.153449 | 0.622148 | 0.317532 | 0.880492 |
| Map2k1 | 23.59602 | 22.4699 | 23.84503 | 24.05433 | 22.9146 | 22.59066 | 25.02419 | 25.87077 |
| Mon1a | 8.391651 | 8.268709 | 8.927812 | 8.823961 | 7.684095 | 7.976482 | 8.429651 | 8.64409 |
| Krt31 | 0 | 0.04759 | 0 | 0 | 0 | 0 | 0 | 0 |
| AC098750. | 0.608906 | 0.984212 | 0.840471 | 0.561083 | 0.470287 | 0.762699 | 0.29195 | 0.925206 |
| Loxl2 | 6.220467 | 5.860464 | 6.119346 | 9.636925 | 9.432619 | 9.1264 | 10.00902 | 11.15881 |
| Dock7 | 6.785058 | 6.7724 | 6.888927 | 7.347504 | 7.529952 | 7.443349 | 6.884699 | 6.592018 |
| AABR07053 | 0.01734 | 0 | 0.018615 | 0 | 0.018749 | 0.015203 | 0 | 0.030738 |
| Pum1 | 9.996775 | 10.05166 | 10.04045 | 8.438002 | 8.782612 | 9.090452 | 11.39146 | 11.13637 |
| AABR07003 | 0 | 0.506407 | 0 | 0 | 0 | 0 | 0 | 0 |
| #N/A | 0.164156 | 0 | 0 | 0 | 0 | 0 | 0 | 0 |
| Ldb2 | 1.570396 | 1.338106 | 1.040692 | 1.697167 | 1.341666 | 1.325927 | 1.12788 | 1.082604 |
| Prdm10 | 0.764808 | 0.830024 | 0.720531 | 0.783844 | 0.776344 | 0.807438 | 0.899288 | 0.885404 |
| AABR07017 | 0 | 0 | 0 | 0 | 0 | 0 | 0 | 0.138138 |
| Tctn1 | 2.566072 | 2.85962 | 3.059398 | 3.124988 | 3.318433 | 3.414473 | 3.779656 | 3.77168 |
| Ica1l | 0.031245 | 0.141407 | 0.067086 | 0 | 0 | 0 | 0.034955 | 0.110775 |
| Cnot2 | 18.13297 | 18.29057 | 16.4747 | 17.85698 | 20.38694 | 18.48655 | 18.78231 | 18.68787 |
| Vrk1 | 2.65038 | 2.58842 | 3.055009 | 3.83043 | 2.92615 | 2.935396 | 2.559373 | 3.536109 |
| Eif2b3 | 3.169969 | 3.586671 | 3.878024 | 3.713521 | 4.145055 | 3.835185 | 3.683879 | 4.094758 |
| Fgf9 | 2.330283 | 2.458857 | 2.023854 | 3.16058 | 2.349834 | 2.203895 | 1.640371 | 1.462057 |
| Lrrc29 | 0.499588 | 0.28263 | 0.316928 | 0.146475 | 0.22099 | 0.21902 | 0.177837 | 0.241533 |
| Epha1 | 0.306328 | 0.310112 | 0.190394 | 0.2377 | 0.17433 | 0.212043 | 0.324669 | 0.200063 |
| Tsnax | 35.15244 | 33.132 | 32.45171 | 36.34178 | 36.20323 | 36.85495 | 36.42481 | 34.33349 |
| Rspry1 | 4.059907 | 4.671451 | 4.112323 | 4.290577 | 4.613135 | 3.821179 | 4.644689 | 4.472709 |
| LOC103690 | 0.059271 | 0.033531 | 0.031816 | 0.054616 | 0.064089 | 0 | 0 | 0.052535 |
| AABR07006 | 0 | 0 | 0 | 0 | 0.808893 | 0 | 0 | 0 |
| Ggps1 | 3.718114 | 3.769357 | 3.512637 | 3.522027 | 3.570065 | 3.912055 | 4.991593 | 4.007388 |
| Bckdha | 158.6168 | 160.3426 | 168.1504 | 144.5304 | 140.1527 | 151.9828 | 153.3522 | 145.1923 |

| | | | | | | | | |
|---|---|---|---|---|---|---|---|---|
| Qrich1 | 8.929149 | 9.008593 | 8.918514 | 8.052799 | 8.553137 | 9.343416 | 9.351906 | 9.904417 |
| AABR0701C | 0 | 0 | 0 | 0 | 0 | 0 | 0 | 0 |
| Paox | 5.741877 | 6.45954 | 7.538024 | 7.225877 | 7.060075 | 6.501675 | 9.360332 | 8.637288 |
| Slc25a18 | 0.053326 | 0 | 0.028624 | 0.049138 | 0.05766 | 0.046756 | 0.029829 | 0.047265 |
| Kctd14 | 0.525596 | 0.312993 | 0.71275 | 1.019612 | 0.508496 | 0.921685 | 0.990338 | 0.956242 |
| Ppa2 | 47.12357 | 46.66963 | 45.08585 | 39.38815 | 38.93187 | 35.4082 | 41.89223 | 40.69568 |
| Rps15-ps2 | 0.141913 | 0 | 0.152353 | 0.261535 | 0.153449 | 0.12443 | 0 | 0 |
| Tcam1 | 0.396712 | 0.641229 | 0.760527 | 0.574442 | 0.796637 | 0.670829 | 0.475525 | 0.552554 |
| Pcgf6 | 0.752024 | 0.595616 | 0.645876 | 0.785355 | 0.677627 | 0.725313 | 1.09373 | 1.110927 |
| Ptges | 0 | 0.236242 | 0.089662 | 0.307835 | 0.225768 | 0.256301 | 0.186873 | 0.037013 |
| LOC688754 | 36.45807 | 37.4687 | 33.55812 | 40.15391 | 37.81527 | 36.95953 | 41.9649 | 41.42194 |
| Il1rn | 0.436383 | 0.406615 | 0.496043 | 0.94614 | 1.360051 | 0.967805 | 1.69436 | 1.137611 |
| Gys1 | 32.43254 | 31.91226 | 36.44813 | 27.87204 | 26.24793 | 27.28 | 26.95117 | 27.07409 |
| Map3k5 | 8.137347 | 7.741945 | 8.136301 | 6.620915 | 5.929921 | 6.344547 | 8.762846 | 8.416499 |
| Syngr4 | 0.26265 | 0.371471 | 0.387711 | 0.211769 | 0.142 | 0.230292 | 0.477493 | 0.291 |
| Mir3546 | 8.125746 | 5.107722 | 1.453918 | 0.415975 | 3.416876 | 1.979073 | 0.50504 | 1.600501 |
| Rras | 41.50688 | 45.02968 | 45.62548 | 53.73875 | 47.68212 | 47.31609 | 59.26301 | 58.28686 |
| Fam26e | 3.109941 | 2.76843 | 2.381293 | 2.191409 | 2.324243 | 2.405999 | 1.99546 | 1.945761 |
| Arhgap11a | 0.891602 | 1.023014 | 0.916747 | 1.087721 | 1.004812 | 0.869843 | 1.039632 | 0.934969 |
| Ino80e | 11.99572 | 12.03552 | 12.87817 | 11.00634 | 9.755812 | 11.21455 | 13.64968 | 14.08566 |
| AC080157. | 0.035406 | 0 | 0.019005 | 0.016313 | 0.019142 | 0 | 0 | 0 |
| Ttc7b | 46.57163 | 52.61166 | 53.18565 | 46.85159 | 44.01363 | 45.57579 | 50.31961 | 50.58733 |
| AABR07029 | 2.128696 | 0 | 1.828233 | 2.353811 | 2.762074 | 2.239731 | 4.762981 | 0.754708 |
| #N/A | 0.205357 | 0 | 0 | 0 | 0 | 0 | 0 | 0 |
| Herpud1 | 107.9443 | 112.1625 | 113.535 | 83.64401 | 81.79832 | 83.25428 | 122.0673 | 123.3941 |
| AABR07064 | 0 | 0 | 0 | 0.294226 | 0.345259 | 0 | 0 | 0 |
| Diaph3 | 0.21213 | 0.205728 | 0.422938 | 0.418864 | 0.360444 | 0.318851 | 0.440741 | 0.402904 |
| Rbpjl | 0.242435 | 0.114293 | 0.108446 | 0.279243 | 0.371367 | 0.371992 | 0.519849 | 0.358137 |
| Ogdhl | 1.677097 | 1.783049 | 2.126419 | 1.90508 | 1.422598 | 1.863454 | 1.940961 | 1.935006 |
| Tmem123 | 28.70183 | 30.37478 | 29.10535 | 26.26436 | 27.05696 | 26.56066 | 27.47547 | 25.35173 |
| Nbl1 | 11.75681 | 12.80715 | 13.37335 | 35.02721 | 32.52246 | 34.50621 | 39.58953 | 39.74324 |
| Chrnb1 | 0.184063 | 0.364453 | 0.321105 | 0.212009 | 0.572196 | 0.42364 | 0.308882 | 0.305895 |
| Slc12a8 | 0.256696 | 0.158421 | 0.150316 | 0.365554 | 0.126164 | 0.388759 | 0.261072 | 0.144787 |
| Fam204a | 4.431915 | 4.482237 | 4.359233 | 4.334797 | 4.14964 | 4.580594 | 4.487336 | 4.037953 |
| Ttc21a | 0.088696 | 0.11039 | 0.104743 | 0.098075 | 0.17263 | 0.12443 | 0.109152 | 0.078615 |
| Gucy1a3 | 6.164726 | 5.592995 | 5.809333 | 6.690428 | 6.57942 | 6.416217 | 6.002789 | 5.87897 |
| Testin | 0.038391 | 0.086876 | 0.206078 | 0.495267 | 0.78873 | 0.740556 | 0.472457 | 0.476395 |
| Pstk | 6.04189 | 5.976717 | 5.976712 | 5.782105 | 5.917039 | 5.282398 | 5.697207 | 5.821897 |
| LOC100911 | 6.917657 | 3.534776 | 3.034503 | 3.701235 | 3.860626 | 3.978386 | 2.330072 | 3.823923 |
| Ppp2r3c | 8.309562 | 7.91177 | 6.665387 | 8.321499 | 7.493159 | 7.065882 | 8.454434 | 8.588075 |
| Clec4b2 | 0 | 0 | 0.096017 | 0 | 0 | 0.078419 | 0 | 0 |
| AABR07042 | 0.166241 | 0 | 0 | 0.153185 | 0 | 0.14576 | 0 | 0 |
| Tmod1 | 67.09771 | 67.81719 | 64.81258 | 68.71034 | 64.83255 | 65.65156 | 70.72633 | 71.57857 |
| AABR07049 | 0 | 0.473997 | 0 | 0.386025 | 0 | 0 | 0 | 0 |
| Dusp8 | 9.61823 | 9.945464 | 10.98549 | 8.912059 | 8.320043 | 8.50355 | 15.21408 | 14.58742 |
| Pfkfb4 | 0.577119 | 0.27494 | 0.130437 | 0.447825 | 0.098531 | 0.31959 | 0.849546 | 0.403839 |
| Pomc | 2.765971 | 2.546087 | 1.811866 | 2.159943 | 1.774206 | 1.726418 | 2.517515 | 2.036089 |
| Crb3 | 1.26077 | 1.393327 | 1.290562 | 1.702098 | 1.711991 | 1.233984 | 2.394558 | 2.105026 |
| Wdr33 | 8.910886 | 8.393144 | 9.143993 | 8.243022 | 8.158647 | 8.147919 | 7.975283 | 8.380257 |
| AABR07071 | 0 | 0 | 0 | 0 | 0 | 0.364401 | 0 | 0 |
| LOC683422 | 0 | 0 | 0 | 0 | 0 | 0.227581 | 0.072596 | 0 |
| LOC102555 | 20.29442 | 115.2868 | 124.2895 | 75.96478 | 70.83499 | 65.737 | 15.64611 | 18.54717 |
| Tmem154 | 2.52072 | 2.668071 | 2.444268 | 2.17289 | 2.373925 | 3.992564 | 2.456189 | 2.162167 |
| Gng10 | 22.66585 | 23.04764 | 20.40006 | 29.84312 | 29.10355 | 27.58296 | 25.79474 | 27.79675 |

| | | | | | | | | |
|---|---|---|---|---|---|---|---|---|
| St6galnac | 0.074436 | 0 | 0 | 0.06859 | 0 | 0.032633 | 0.041638 | 0 |
| AC128962. | 0 | 0 | 0 | 0 | 0.583737 | 0 | 0.603966 | 0.4785 |
| AABR07021 | 0 | 0 | 0 | 0.455218 | 0 | 0 | 0 | 0 |
| Mir30a | 0 | 0 | 0 | 0 | 0 | 0 | 0 | 0 |
| Odf3l1 | 0.626271 | 0.381552 | 0.36203 | 0.754649 | 0.781359 | 0.464636 | 1.185706 | 0.640495 |
| Cpsf6 | 5.34033 | 5.695167 | 6.329249 | 5.930666 | 5.940308 | 5.822582 | 7.584622 | 7.187516 |
| Katna1 | 2.969202 | 3.109631 | 2.687091 | 3.617855 | 3.528957 | 3.571605 | 3.486529 | 2.697001 |
| Fam53b | 8.636264 | 8.204981 | 8.712343 | 7.75587 | 6.655369 | 7.019387 | 8.588328 | 8.128616 |
| Nsun6 | 1.98044 | 2.298977 | 2.098517 | 2.298897 | 1.835504 | 2.164927 | 2.3595 | 2.530458 |
| LOC10091( | 3.175508 | 2.304903 | 3.63424 | 2.705267 | 2.753383 | 2.679221 | 3.016373 | 2.788057 |
| Ints8 | 7.797705 | 8.430615 | 7.887657 | 7.536559 | 7.101235 | 7.429578 | 9.925661 | 9.13853 |
| AABR07026 | 0 | 0 | 0.035071 | 0 | 0 | 0.028643 | 0 | 0 |
| Rab5a | 15.37624 | 15.69925 | 14.14406 | 15.52091 | 16.44577 | 15.20735 | 16.90373 | 17.02851 |
| Rnf24 | 1.678395 | 1.709122 | 3.243355 | 1.85589 | 2.540754 | 1.324456 | 2.81657 | 3.272819 |
| 5S_rRNA | 0.45935 | 0 | 0 | 0 | 0 | 0 | 0 | 0 |
| Mrps34 | 49.45993 | 48.85953 | 48.79312 | 52.48399 | 48.32709 | 45.20105 | 45.84039 | 43.94182 |
| Mir292 | 0.638609 | 0 | 0 | 0 | 0 | 0 | 0 | 0.566031 |
| Jund | 80.68531 | 90.30355 | 105.1035 | 97.05232 | 84.74829 | 86.71521 | 122.2288 | 119.2568 |
| Hcar2 | 0.290116 | 0.273544 | 0.259548 | 0.311885 | 0.261415 | 0.084791 | 0.270474 | 0.085715 |
| Atp2c1 | 12.16025 | 11.90789 | 10.96011 | 12.93046 | 13.34049 | 12.30377 | 14.04533 | 14.60389 |
| LOC102549 | 0 | 0 | 0 | 0.260828 | 0 | 0 | 0 | 0 |
| Spag9 | 19.66557 | 20.43407 | 19.20357 | 20.31931 | 21.5093 | 21.06038 | 23.00562 | 24.9479 |
| AC112557. | 0 | 0.457526 | 0 | 0.558917 | 0 | 0 | 0.226196 | 0.358413 |
| Adamts12 | 2.149438 | 2.266641 | 1.797674 | 1.677638 | 1.705259 | 1.44684 | 2.329762 | 2.23977 |
| Mgat4a | 3.699845 | 3.708249 | 3.581073 | 4.147488 | 4.26047 | 4.06142 | 4.440701 | 4.38969 |
| Cdc42 | 162.3344 | 155.6289 | 146.2422 | 142.5719 | 146.9165 | 143.5593 | 154.0386 | 149.4547 |
| #N/A | 0.955002 | 0.36018 | 0.170876 | 0.439998 | 0.34421 | 0.279115 | 0.178069 | 0.705388 |
| Chrna10 | 0 | 0.027304 | 0 | 0.022236 | 0 | 0 | 0.026998 | 0.085557 |
| Fsd1l | 16.38719 | 15.58362 | 14.18279 | 14.40083 | 13.31222 | 12.84028 | 14.18233 | 14.00159 |
| Fam171b | 1.025206 | 0.341168 | 0.517941 | 1.315153 | 0.760763 | 0.8284 | 1.034508 | 1.33631 |
| Enox2 | 0.879213 | 1.03458 | 0.547457 | 0.842566 | 0.665476 | 0.709223 | 0.452467 | 0.810462 |
| AABR07058 | 0.167839 | 0.189902 | 0.180186 | 0.154657 | 0.544447 | 0 | 0 | 0.148764 |
| Arhgap24 | 5.075898 | 6.7821 | 6.380337 | 7.215642 | 6.674451 | 7.179031 | 6.70599 | 6.895484 |
| Pbdc1 | 4.500447 | 5.092045 | 4.727985 | 4.532062 | 4.935787 | 4.819753 | 4.171775 | 4.501838 |
| Ilf3 | 10.84191 | 10.12372 | 11.27632 | 10.19301 | 10.02233 | 10.90024 | 11.86453 | 11.19885 |
| Ing4 | 16.24695 | 18.12164 | 16.41613 | 16.58037 | 16.21349 | 17.10596 | 17.88141 | 16.35754 |
| AABR07027 | 0 | 0 | 0 | 0 | 0 | 0 | 0 | 0 |
| Rph3a | 0.039417 | 0.066898 | 0.021159 | 0.036322 | 0.042621 | 0.051842 | 0 | 0.052406 |
| Gkn1 | 0 | 0 | 0.079629 | 0.068347 | 0 | 0 | 0 | 0 |
| Camta2 | 12.5782 | 12.78979 | 13.25016 | 11.92737 | 11.15099 | 11.61829 | 15.39201 | 15.03922 |
| Lrrc66 | 0.030113 | 0.068142 | 0.113148 | 0.041621 | 0.097681 | 0.118813 | 0.168444 | 0.053381 |
| Gzmm | 2.691182 | 3.425564 | 3.19011 | 3.04811 | 1.394345 | 3.097016 | 2.822602 | 2.236246 |
| Pcdhac1 | 0.387014 | 0.336837 | 0.383524 | 0.914405 | 1.051546 | 0.930993 | 1.065781 | 1.134636 |
| AABR07033 | 0 | 0 | 0.267706 | 0 | 0 | 0 | 0.278975 | 0 |
| Otud5 | 15.70978 | 15.82694 | 15.8258 | 19.28473 | 19.02284 | 18.35007 | 22.49089 | 21.42698 |
| Ebna1bp2 | 2.674004 | 2.672534 | 3.971155 | 0.615997 | 0.626462 | 0.50799 | 0.099719 | 3.871168 |
| Uprt | 2.287435 | 2.726775 | 2.543411 | 2.747643 | 2.826709 | 2.578661 | 2.421987 | 4.199754 |
| AC142458. | 3.117019 | 6.113051 | 5.800287 | 2.87221 | 3.59508 | 4.372808 | 4.417098 | 4.236246 |
| Ano2 | 0.848982 | 0.756204 | 1.260497 | 0.649145 | 0.683611 | 0.475141 | 0.929595 | 0.89659 |
| Gm43337 | 0 | 0 | 0 | 0 | 0 | 0 | 0 | 0 |
| Tcf23 | 0.070039 | 0.066038 | 0.025064 | 0.172102 | 0.176709 | 0.184231 | 0.143654 | 0.23797 |
| Guk1 | 88.54301 | 96.63101 | 92.98756 | 104.929 | 105.3262 | 108.7753 | 101.8917 | 96.6359 |
| Atf6b | 36.6822 | 36.08034 | 42.12167 | 43.40382 | 37.07225 | 39.15295 | 58.64296 | 53.66534 |
| LOC103689 | 1.278223 | 1.09635 | 2.102648 | 1.652765 | 1.047739 | 1.21113 | 1.683733 | 1.425328 |

| | | | | | | | | |
|---|---|---|---|---|---|---|---|---|
| Arhgap9 | 3.577933 | 3.723402 | 3.295793 | 2.991654 | 2.937397 | 3.23396 | 4.076959 | 3.523666 |
| Meis1 | 4.525082 | 4.109408 | 3.755335 | 3.97766 | 4.522719 | 3.850135 | 3.380525 | 3.971225 |
| Clcf1 | 0.324822 | 0.393772 | 0.323809 | 0.534483 | 0.50175 | 0.447549 | 0.752749 | 0.596376 |
| Kcnh1 | 0.025097 | 0.042595 | 0.053888 | 0.046253 | 0 | 0.011003 | 0.028078 | 0.055613 |
| Rnf208 | 0.230281 | 0.208442 | 0.34611 | 0.551707 | 0.597599 | 0.444203 | 0.978987 | 0.571507 |
| Nudt16 | 3.749904 | 3.942462 | 3.38449 | 4.923165 | 4.808249 | 4.830041 | 4.677863 | 4.94147 |
| Tada3 | 16.59065 | 17.73207 | 19.05848 | 17.15501 | 18.78652 | 18.2426 | 17.01918 | 17.48328 |
| Fat3 | 0.275852 | 0.294774 | 0.2509 | 0.331855 | 0.219563 | 0.305693 | 0.257176 | 0.288648 |
| Camk2d | 54.79389 | 56.57229 | 53.47904 | 49.80785 | 51.97257 | 50.80361 | 52.59959 | 54.53022 |
| LOC100909 | 4.850263 | 4.147789 | 4.752963 | 5.222876 | 5.153045 | 4.771942 | 5.363163 | 5.448769 |
| NEWGENE_1 | 0.313854 | 0.679344 | 0.373567 | 0.264056 | 0.265591 | 0.311082 | 0.381659 | 0.393087 |
| Ptges2 | 28.13185 | 29.47413 | 28.43397 | 26.74781 | 25.23537 | 25.60003 | 25.81192 | 25.21362 |
| AABR0703( | 0 | 0 | 0 | 0 | 0 | 0 | 0 | 0 |
| Srsf10 | 15.11231 | 14.56386 | 14.00738 | 15.74704 | 16.57824 | 15.48458 | 19.41354 | 18.65147 |
| Dennd1c | 1.242055 | 1.0289 | 1.762026 | 1.635007 | 1.606823 | 1.769682 | 2.183588 | 1.710319 |
| Adamts4 | 0.130056 | 0.262771 | 0.149596 | 0.171201 | 0.331478 | 0.21992 | 0.322179 | 0.370526 |
| Recql4 | 0.337733 | 0.372331 | 0.269609 | 0.454842 | 0.449459 | 0.463169 | 0.474723 | 0.383781 |
| Fst | 0.668908 | 0.864957 | 0.615527 | 2.377435 | 2.324829 | 2.220317 | 2.512299 | 2.075102 |
| Lgals12 | 0.074996 | 0 | 0 | 0.017276 | 0 | 0.016439 | 0 | 0.016618 |
| Cdhr1 | 0.245101 | 0.305053 | 0.249975 | 0.892113 | 0.96734 | 0.902602 | 0.836336 | 0.738635 |
| Lrrc41 | 7.908974 | 8.005615 | 7.67217 | 8.496996 | 9.012049 | 8.427244 | 9.046599 | 9.634981 |
| Rn50_5_08 | 0 | 0 | 0 | 0 | 0 | 0 | 0 | 0 |
| Myl12a | 472.261 | 455.2606 | 446.9171 | 470.8892 | 477.222 | 518.8243 | 462.6625 | 471.1502 |
| R3hcc1l | 2.827861 | 2.913914 | 3.550906 | 3.675986 | 3.276134 | 3.165753 | 2.090292 | 3.17785 |
| Pip5k1c | 11.73421 | 12.45302 | 13.2465 | 11.87 | 11.87418 | 12.89587 | 15.01888 | 13.18942 |
| Zeb1 | 21.66808 | 19.92547 | 21.04834 | 21.20748 | 20.67828 | 20.64621 | 23.25527 | 24.17269 |
| Snap47 | 6.516121 | 6.870002 | 6.168735 | 6.331859 | 7.173893 | 7.219585 | 5.997638 | 6.379371 |
| Slitrk1 | 0.05186 | 0.073347 | 0.083513 | 0.119468 | 0.140189 | 0.056839 | 0.101533 | 0.057458 |
| Eif5 | 40.53317 | 39.48845 | 35.07821 | 36.01432 | 38.62608 | 35.93251 | 43.20724 | 43.42174 |
| Arhgef6 | 7.55603 | 8.187755 | 7.667947 | 10.0282 | 9.003508 | 9.031277 | 10.64029 | 9.346211 |
| Slc6a18 | 0 | 0 | 0 | 0 | 0 | 0.019885 | 0 | 0 |
| Crlf1 | 5.494026 | 5.434763 | 6.370043 | 11.774 | 12.18674 | 13.10269 | 14.36518 | 12.99495 |
| C9 | 0 | 0 | 0 | 0 | 0.023583 | 0.019123 | 0 | 0 |
| Rab3b | 78.61724 | 81.52618 | 85.72169 | 45.56541 | 41.19793 | 44.39599 | 48.59111 | 50.33289 |
| LOC689065 | 0.172825 | 0.244429 | 0.278308 | 0.119438 | 0.233591 | 0.340949 | 0.241686 | 0.306366 |
| AABR07042 | 0 | 0 | 0 | 0.386025 | 0 | 0 | 0 | 0 |
| AABR07021 | 0.415603 | 0 | 0 | 0 | 0 | 0 | 0 | 0 |
| AC139392. | 0 | 0.955638 | 0.453372 | 0.389138 | 0 | 0.370278 | 0 | 0.748621 |
| Tpm4 | 116.3478 | 116.7231 | 108.7289 | 114.4421 | 115.0781 | 115.3367 | 128.9003 | 132.0087 |
| AC119496. | 0 | 0 | 0 | 0 | 0 | 0 | 1.001447 | 0 |
| RGD156088 | 0.395748 | 0.399795 | 0.348993 | 0.325595 | 0.33622 | 0.247851 | 0.41112 | 0.613849 |
| Nmur1 | 0.084735 | 0.095873 | 0 | 0 | 0 | 0 | 0 | 0.075104 |
| Sapcd2 | 1.925715 | 1.223217 | 2.248726 | 0.87167 | 0.730613 | 1.125645 | 1.058304 | 1.287629 |
| AABR07051 | 0 | 0 | 0 | 0 | 0 | 0 | 0.169811 | 0.134535 |
| Il13 | 0 | 0 | 0 | 0 | 0 | 0 | 0 | 0 |
| Asic4 | 0.019926 | 0.045091 | 0.021392 | 0.146889 | 0.172367 | 0.192184 | 0.133755 | 0.070646 |
| Parn | 6.217071 | 6.116803 | 5.830227 | 6.430732 | 5.819019 | 5.688139 | 6.020667 | 7.514317 |
| Insm2 | 0.070384 | 0 | 0.037781 | 0 | 0.038053 | 0.061713 | 0 | 0 |
| Ereg | 0 | 0 | 0 | 0 | 0 | 0.08754 | 0.027924 | 0 |
| Mt1 | 12.38474 | 14.46969 | 15.31909 | 21.83175 | 23.8717 | 19.82939 | 10.54223 | 11.81244 |
| Mapk9 | 15.33987 | 15.78931 | 15.15086 | 14.18355 | 15.1461 | 13.15795 | 13.59196 | 14.66867 |
| AC105648. | 0.409109 | 0.462887 | 0 | 0.753955 | 0.442363 | 1.793535 | 2.288464 | 0.725227 |
| Alg12 | 2.258922 | 2.962479 | 3.031371 | 3.737253 | 3.719322 | 4.141307 | 4.077952 | 3.549345 |
| AC115418. | 0 | 0 | 0 | 0 | 0 | 0 | 0 | 0 |

| | | | | | | | | |
|---|---|---|---|---|---|---|---|---|
| Tmem263 | 6.646592 | 9.302575 | 7.630491 | 9.948005 | 9.222449 | 8.859508 | 10.1008 | 9.807323 |
| LOC100911 | 0.052959 | 0 | 0.11371 | 0.087839 | 0.057264 | 0.018574 | 0.059248 | 0.037552 |
| Egfr | 0.793423 | 1.011717 | 1.068118 | 0.905181 | 0.939623 | 0.872353 | 1.4935 | 1.395338 |
| AC118434. | 0 | 0 | 0 | 0 | 0 | 0 | 0 | 0.241742 |
| LOC102555 | 1.824461 | 1.471357 | 1.333566 | 1.6454 | 1.972764 | 1.803905 | 2.236553 | 1.668721 |
| Bfsp2 | 2.366439 | 1.950761 | 1.850953 | 2.460941 | 2.083591 | 2.193462 | 2.496183 | 2.067529 |
| Zbtb9 | 3.144576 | 3.533232 | 3.04769 | 2.475036 | 3.305735 | 2.718873 | 4.2265 | 3.367859 |
| Ubxn6 | 25.16139 | 24.44718 | 25.41986 | 24.44894 | 21.21453 | 23.43577 | 26.39598 | 24.60887 |
| Cdkl4 | 0.180572 | 0.204309 | 0.646186 | 0.16639 | 0.58575 | 0.158326 | 0.269355 | 0.05335 |
| Pus7l | 0.678852 | 0.960112 | 0.668059 | 1.094688 | 0.876762 | 0.710955 | 1.244687 | 0.969407 |
| Tmem186 | 13.02099 | 13.42758 | 12.16272 | 13.7225 | 14.10713 | 14.02381 | 12.12986 | 12.4499 |
| Klf7 | 2.873839 | 3.011438 | 2.839832 | 2.572899 | 2.701355 | 2.691588 | 3.89103 | 3.806367 |
| AC114512. | 1.181186 | 0.890971 | 1.268079 | 1.088417 | 0.851466 | 0.690443 | 0 | 1.395925 |
| Cetn1 | 0 | 0.084281 | 0 | 0.205917 | 0 | 0 | 0 | 0 |
| Map2k5 | 5.033179 | 5.10467 | 5.095471 | 4.758028 | 4.568151 | 4.664619 | 4.434696 | 5.431978 |
| Sptlc2 | 10.12651 | 10.32223 | 11.09999 | 11.99323 | 12.95544 | 11.70526 | 15.54773 | 13.20738 |
| Dexi | 17.89294 | 20.96654 | 22.14899 | 16.79873 | 17.72496 | 17.8264 | 20.18569 | 19.45021 |
| A1bg | 0.061898 | 0 | 0.033226 | 0 | 0 | 0 | 0 | 0 |
| Ptgfr | 22.57964 | 20.75018 | 20.64392 | 46.77387 | 46.26405 | 47.90345 | 47.28159 | 48.00755 |
| Fam13c | 2.375574 | 2.41517 | 2.661215 | 1.808303 | 2.084721 | 2.928143 | 2.465101 | 2.563326 |
| Il1r1 | 13.86757 | 13.40871 | 13.72005 | 15.15871 | 16.75889 | 14.84125 | 19.44372 | 18.33685 |
| Timp3 | 78.12947 | 82.70536 | 79.08493 | 107.1065 | 104.4342 | 109.6904 | 135.2005 | 138.0487 |
| Gcn1l1 | 11.21646 | 11.96352 | 12.92893 | 10.62325 | 10.43342 | 10.66131 | 12.78821 | 13.08376 |
| Atp5e | 1096.473 | 1026.677 | 1020.022 | 786.4618 | 795.1311 | 763.4048 | 803.0005 | 801.0527 |
| Tiam2 | 0.556321 | 0.426402 | 0.414219 | 0.661455 | 1.028442 | 0.786746 | 1.074119 | 0.906658 |
| LOC100911 | 0 | 0.044382 | 0.084222 | 0.036145 | 0 | 0.068786 | 0 | 0.034767 |
| LOC100364 | 0 | 0 | 0 | 0 | 0 | 0 | 0 | 0 |
| AABR07001 | 1.926267 | 1.493348 | 1.953083 | 1.150449 | 1.234278 | 0.656815 | 1.356866 | 1.454406 |
| AABR07072 | 0 | 0 | 0.449745 | 0.386025 | 0.45298 | 0.367316 | 0.468677 | 1.113948 |
| Entpd3 | 0 | 0 | 0 | 0 | 0.070756 | 0 | 0.073208 | 0 |
| AABR07035 | 1.745531 | 1.974986 | 1.192506 | 0.584886 | 1.115292 | 1.113078 | 1.420234 | 1.476826 |
| Rn50_13_( | 0 | 0 | 0 | 0.135924 | 0.319 | 0.064668 | 0.082514 | 0.130745 |
| Ube2c | 0.343383 | 0.582783 | 0.399364 | 0.580092 | 0.556943 | 0.526888 | 0.288121 | 0.431173 |
| Olr125 | 0 | 0 | 0 | 0 | 0 | 0 | 0 | 0 |
| Metrnl | 5.746349 | 5.557925 | 6.666578 | 11.05979 | 8.718865 | 7.801399 | 9.694985 | 9.570427 |
| Col19a1 | 0.185366 | 0.209733 | 0.149252 | 0.14234 | 0.100217 | 0.176073 | 0.172816 | 0.177991 |
| Micall1 | 4.028148 | 4.323074 | 4.006498 | 4.69431 | 4.179433 | 4.285006 | 5.053259 | 5.145501 |
| Tm9sf1 | 11.06108 | 11.13547 | 10.68262 | 12.37929 | 13.27863 | 13.30661 | 13.32466 | 13.16204 |
| Dock3 | 0.138019 | 0.165348 | 0.235332 | 0.142141 | 0.105344 | 0.121015 | 0.06358 | 0.079157 |
| Slc7a2 | 1.152511 | 1.114338 | 1.034828 | 2.008134 | 2.265807 | 2.259897 | 3.117951 | 2.823132 |
| Rtn4r | 0.214113 | 0.20765 | 0.197026 | 0.563705 | 0.429961 | 0.241373 | 0.3422 | 0.298224 |
| Rhebl1 | 0.398725 | 0.551392 | 0.475619 | 0.530703 | 0.622752 | 0.660361 | 0.842588 | 0.863891 |
| Tmem114 | 0.036139 | 0 | 0 | 0 | 0 | 0 | 0 | 0 |
| Zp4 | 0 | 0 | 0 | 0 | 0 | 0 | 0 | 0 |
| Stat5b | 17.11689 | 17.89663 | 18.24529 | 14.55639 | 15.39061 | 15.75584 | 17.03703 | 17.20523 |
| LOC10091( | 0 | 0 | 0 | 0.02755 | 0 | 0 | 0.016724 | 0 |
| Gstm6 | 0 | 0.051566 | 0 | 0.041996 | 0 | 0.03996 | 0.101975 | 0.040396 |
| AABR07039 | 2.23786 | 2.025627 | 3.243355 | 3.196255 | 3.145695 | 3.335668 | 2.128075 | 2.97529 |
| AABR07017 | 0 | 0 | 0.281795 | 0.120935 | 0 | 0 | 0 | 0.116327 |
| Rgs19 | 4.66828 | 4.68485 | 5.273176 | 4.787908 | 4.828276 | 5.69482 | 5.858467 | 7.052128 |
| AABR07004 | 0 | 0 | 0 | 0 | 0.658401 | 0 | 0 | 0 |
| Psma2 | 83.52185 | 78.90905 | 75.70798 | 71.16509 | 73.2076 | 70.55281 | 74.87308 | 75.03857 |
| Gpaa1 | 10.78961 | 10.91671 | 10.748 | 10.99378 | 10.51681 | 10.82481 | 12.59324 | 13.03469 |
| Clpp | 64.834 | 64.09661 | 62.87648 | 68.28496 | 65.12626 | 64.35877 | 57.58322 | 61.09261 |

| | | | | | | | | |
|---|---|---|---|---|---|---|---|---|
| Dnajc1 | 3.139713 | 4.345392 | 3.551254 | 4.494671 | 4.728647 | 4.129354 | 4.077091 | 6.06271 |
| Malsu1 | 23.20364 | 22.05004 | 20.24456 | 19.37877 | 18.49517 | 16.22681 | 19.99878 | 19.63452 |
| Rn50_13_( | 0 | 0 | 0.134172 | 0 | 0 | 0 | 0 | 0 |
| Rep15 | 0.146274 | 0.165502 | 0.314068 | 0.134785 | 0.105442 | 0.256506 | 0.109096 | 0.259299 |
| Cd302 | 13.1564 | 13.61271 | 11.56886 | 18.22453 | 18.90537 | 17.34126 | 19.99625 | 20.56048 |
| Nt5c3b | 8.404407 | 8.508224 | 8.913081 | 7.336734 | 8.572479 | 9.248532 | 7.384942 | 7.027019 |
| AABR0707 | 0 | 0.055322 | 0 | 0 | 0.105738 | 0.085741 | 0.054701 | 0 |
| Med15 | 10.01137 | 10.4687 | 11.02521 | 11.38256 | 10.94737 | 11.9069 | 12.69967 | 12.55181 |
| Lingo4 | 0.104251 | 0.090734 | 0.051655 | 0.073895 | 0.043356 | 0.070313 | 0.107659 | 0.042647 |
| AABR0704 | 0 | 0 | 0 | 0 | 0 | 0 | 0 | 0 |
| Ralgds | 4.246271 | 5.11078 | 4.925782 | 4.884399 | 4.606838 | 5.059964 | 5.276605 | 5.380295 |
| Nos3 | 21.46974 | 23.04823 | 25.38851 | 17.6232 | 16.3672 | 17.86085 | 22.11481 | 21.96132 |
| Ift20 | 13.6263 | 12.73392 | 12.88125 | 14.27024 | 13.72819 | 15.49512 | 12.95522 | 15.00434 |
| Rps4x-ps | 82.80496 | 72.4771 | 70.04135 | 72.13149 | 65.65182 | 61.88063 | 68.83096 | 68.76225 |
| AABR0700 | 0.684522 | 0 | 0.367439 | 0 | 0 | 0.300095 | 0.765813 | 0 |
| Emcn | 35.26223 | 36.10388 | 32.80067 | 25.75815 | 29.00376 | 27.51566 | 29.0816 | 27.28105 |
| RGD130974 | 11.6293 | 11.80055 | 10.59671 | 9.974743 | 11.2331 | 10.69864 | 12.46125 | 12.44649 |
| Dctn4 | 19.36161 | 18.71039 | 19.12157 | 19.36475 | 21.12169 | 19.7251 | 21.58382 | 19.51242 |
| Hoxa10 | 0 | 0 | 0 | 0 | 0 | 0 | 0 | 0.018648 |
| RT1-CE6 | 0 | 0.033174 | 0 | 0.10807 | 0.190221 | 0.179956 | 0 | 0.337844 |
| LOC691254 | 0.26096 | 0.164035 | 0.249028 | 0.507646 | 0.219467 | 0.355926 | 0.227072 | 0.334102 |
| Rnf25 | 8.762564 | 9.005936 | 8.657597 | 11.2269 | 11.92848 | 10.40728 | 11.52165 | 12.99607 |
| Tas1r1 | 0.423941 | 0.646511 | 0.593645 | 0.390645 | 0.637776 | 0.387873 | 0.639255 | 0.441109 |
| Vom1r67 | 0.114838 | 0 | 0 | 0 | 0.062086 | 0 | 0 | 0 |
| Wbscr28 | 0.061175 | 0.138434 | 0.197026 | 0.112741 | 0.264591 | 0.321831 | 0.27376 | 0.162668 |
| Pparg | 4.238349 | 5.016338 | 5.268582 | 3.571451 | 3.919556 | 3.056076 | 3.961796 | 4.275672 |
| Cyp2d4 | 3.066339 | 2.997847 | 3.323871 | 2.798078 | 3.058066 | 2.949595 | 4.196514 | 4.063579 |
| Gatad2b | 5.440863 | 4.665661 | 4.590911 | 4.239521 | 4.025297 | 4.368823 | 4.656018 | 5.617798 |
| AABR0701 | 1.354291 | 1.021544 | 2.180877 | 0.415975 | 1.464375 | 1.187444 | 1.01008 | 1.000313 |
| AABR0704 | 0 | 0 | 0.439204 | 0 | 0 | 0 | 0 | 0 |
| Nnat | 6.69597 | 7.479045 | 7.00423 | 5.299935 | 5.847899 | 5.569954 | 6.914911 | 6.619776 |
| LOC100361 | 2.729977 | 1.123215 | 0.532874 | 1.143439 | 1.073413 | 1.37816 | 1.018059 | 1.393169 |
| AABR0705 | 0 | 0 | 0 | 0 | 0.109734 | 0 | 0 | 0 |
| Rgs7 | 0.12052 | 0.090908 | 0.064693 | 0.055527 | 0.021719 | 0.070448 | 0 | 0.035608 |
| Snx30 | 1.655001 | 1.944577 | 1.731191 | 1.349054 | 1.583044 | 1.823185 | 2.183869 | 1.993492 |
| Rasl12 | 3.090448 | 2.865348 | 3.271713 | 4.034279 | 4.455542 | 4.36564 | 3.673547 | 3.728379 |
| AABR0706 | 0 | 0 | 0 | 0 | 0.529182 | 0 | 0 | 0 |
| Srsf4 | 18.83273 | 18.56755 | 20.34562 | 19.30125 | 17.33342 | 16.8249 | 23.59328 | 24.39657 |
| Mir185 | 0.654574 | 2.221859 | 0 | 1.206328 | 0 | 1.147862 | 0 | 1.160363 |
| Nup214 | 4.236029 | 4.665904 | 4.497453 | 4.454798 | 4.226466 | 3.591169 | 5.021543 | 5.171903 |
| Mrpl42 | 100.9858 | 102.6934 | 100.7905 | 74.28538 | 79.65969 | 74.79066 | 70.98261 | 67.5812 |
| Actc1 | 6013.957 | 6279.779 | 6595.308 | 7086.406 | 6884.103 | 7180.915 | 5907.648 | 5903.559 |
| Bclaf1 | 18.77177 | 17.94773 | 16.76774 | 20.93665 | 20.33279 | 19.62267 | 23.81934 | 23.59621 |
| AC093937. | 0.39079 | 0.442161 | 1.258616 | 1.80049 | 1.267668 | 0.685291 | 1.748796 | 1.039131 |
| Hypk | 202.8844 | 206.7658 | 187.6822 | 179.5264 | 177.5624 | 173.0623 | 156.6764 | 168.7584 |
| AABR0704 | 0 | 1.00423 | 0 | 0 | 0 | 0 | 0 | 0 |
| Neurod4 | 0 | 0.017814 | 0.016903 | 0.043524 | 0.034048 | 0.041414 | 0.035228 | 0.069775 |
| AABR0700 | 0 | 0 | 0 | 0 | 0 | 0.095061 | 0 | 0 |
| Cd209e | 0 | 0 | 0 | 0 | 0.078971 | 0.128074 | 0 | 0 |
| AABR07002 | 0.489401 | 0 | 0 | 0 | 0 | 0 | 0 | 0 |
| Elk4 | 9.381849 | 10.58945 | 9.243844 | 9.000422 | 8.598869 | 9.101333 | 10.35639 | 11.08076 |
| Plk5 | 0.175053 | 0.141475 | 0.134236 | 0.207392 | 0.108161 | 0.043853 | 0.11191 | 0.132993 |
| Mettl18 | 2.197903 | 1.263149 | 1.910144 | 1.896692 | 1.961606 | 1.590642 | 2.068612 | 1.54612 |
| Oacyl | 0 | 0 | 0 | 0 | 0.016508 | 0 | 0 | 0.013532 |

| | | | | | | | | |
|---|---|---|---|---|---|---|---|---|
| Letm1 | 24.50469 | 24.05985 | 26.4115 | 21.65044 | 20.51753 | 19.5464 | 20.4037 | 22.37321 |
| RGD156272 | 0.135412 | 0.065663 | 0.083071 | 0.196078 | 0.397424 | 0.322266 | 0.216419 | 0.754429 |
| Krt222 | 0 | 0.018585 | 0.017634 | 0.045408 | 0.017761 | 0.072011 | 0.018377 | 0 |
| Rn50_1_01 | 0.275973 | 0 | 0.148137 | 0 | 0.074601 | 0.18148 | 0.154373 | 0.183457 |
| Sox7 | 6.430063 | 6.210193 | 7.21141 | 5.013504 | 6.124617 | 6.337374 | 6.122651 | 6.816514 |
| Rps27a-ps | 139.3222 | 106.1057 | 97.27289 | 68.61684 | 126.3357 | 98.56871 | 102.8725 | 70.516 |
| Rn50_9_04 | 1.675709 | 1.42199 | 1.124363 | 2.123138 | 2.378146 | 1.561093 | 1.054524 | 2.135068 |
| Fam89a | 3.77377 | 4.005729 | 4.719652 | 3.47738 | 4.290859 | 3.479404 | 4.526601 | 4.620762 |
| LOC103691 | 0 | 0 | 0 | 0 | 0 | 0 | 0 | 0 |
| Zfpm1 | 12.93886 | 15.31126 | 17.9869 | 12.71958 | 11.46019 | 12.56404 | 14.68411 | 15.03061 |
| Dnase1l3 | 0.85907 | 0.422608 | 0.842069 | 0.619512 | 0.888513 | 0.523988 | 0.543224 | 0.728331 |
| Rubcn | 15.87234 | 17.04949 | 17.49097 | 15.45043 | 13.72607 | 13.43643 | 15.85689 | 17.22534 |
| Srsf11 | 17.36239 | 16.73857 | 16.84407 | 18.6958 | 18.45076 | 17.12628 | 20.56032 | 21.68952 |
| Cdca2 | 0.172304 | 0.162461 | 0.077075 | 0.211695 | 0.062103 | 0.113307 | 0.080319 | 0.139994 |
| Bfar | 27.14282 | 27.54321 | 25.63308 | 23.19295 | 23.62635 | 22.61826 | 25.10132 | 26.11407 |
| Mtnr1a | 0 | 0 | 0 | 0.0272 | 0 | 0 | 0 | 0 |
| Kcnn2 | 0.329345 | 0.440391 | 0.353573 | 0.110356 | 0.22662 | 0.131259 | 0.133984 | 0.265377 |
| Ccdc96 | 0.473364 | 0.468641 | 0.285855 | 0.572495 | 0.511842 | 0.70039 | 0.49648 | 0.734241 |
| Gorab | 1.553476 | 1.334951 | 1.625535 | 1.503947 | 1.934904 | 1.637956 | 1.627963 | 2.004382 |
| RT1-DOb | 1.6382 | 1.470054 | 1.394841 | 1.665696 | 1.649199 | 2.228859 | 2.275133 | 2.203062 |
| LOC100361 | 53.66211 | 48.10362 | 51.76524 | 46.8995 | 49.24103 | 41.97463 | 48.72388 | 55.98847 |
| AABR0707( | 0 | 0 | 0 | 0 | 0 | 0 | 0.054701 | 0 |
| AABR07013 | 0.220953 | 0.249998 | 0 | 0 | 0.238914 | 0 | 0 | 0.195842 |
| Slc12a3 | 1.066003 | 0.948644 | 1.144423 | 0.607027 | 0.673461 | 0.672124 | 1.071998 | 0.934235 |
| Wdr37 | 5.205336 | 5.087085 | 4.839718 | 4.80759 | 5.069509 | 4.996204 | 4.787911 | 5.093237 |
| Rad1 | 3.188827 | 3.216778 | 3.010951 | 3.044585 | 3.738831 | 2.728594 | 3.911375 | 3.677746 |
| Zfp346 | 4.211407 | 4.138949 | 4.65322 | 4.19223 | 4.071769 | 4.366391 | 5.089833 | 5.1496 |
| Cd180 | 0.408152 | 0.646527 | 0.832537 | 0.752192 | 1.279854 | 1.002031 | 0.753427 | 0.615001 |
| Ppp4r3a | 7.093943 | 6.093844 | 7.070636 | 6.749483 | 6.605682 | 6.75967 | 7.747017 | 7.788037 |
| Noxo1 | 0 | 0 | 0.05722 | 0 | 0 | 0 | 0 | 0.023621 |
| Plac9 | 3.535252 | 3.999971 | 4.447639 | 7.63499 | 7.704962 | 7.119651 | 6.179817 | 5.875255 |
| Map3k2 | 2.299252 | 1.714164 | 2.047428 | 1.888737 | 1.811612 | 1.750314 | 2.532421 | 2.60667 |
| AABR07019 | 0 | 0.205728 | 0 | 0.837728 | 0.393212 | 0.318851 | 0.203419 | 0.161162 |
| Lzic | 7.735242 | 8.038465 | 8.164876 | 8.3755 | 7.742221 | 7.823191 | 7.574709 | 7.891948 |
| Serf2 | 962.4295 | 1012.938 | 1064.508 | 1068.437 | 1054.686 | 1071.819 | 1056.257 | 1051.49 |
| Olr828 | 0 | 0 | 0 | 0 | 0 | 0 | 0 | 0.047169 |
| Egfl6 | 0.26183 | 0.296248 | 0.324336 | 0.129912 | 0.152445 | 0.282551 | 0.247858 | 0.249924 |
| Grb10 | 11.64116 | 11.03198 | 10.64707 | 12.51666 | 12.89341 | 13.00403 | 18.21867 | 18.1166 |
| AABR07016 | 0.68106 | 0.599345 | 0.731161 | 1.743249 | 1.636489 | 1.061607 | 0.761939 | 1.274387 |
| LOC680273 | 0.482635 | 0.182026 | 0.25907 | 0.296486 | 0.260933 | 0.211587 | 0.179984 | 0.356486 |
| Degs2 | 0 | 0.184578 | 0.043784 | 0.075161 | 0.088197 | 0.071518 | 0.091253 | 0.072297 |
| AABR0705( | 0.494018 | 0.186319 | 0 | 0 | 0 | 0 | 0 | 0 |
| LOC102546 | 1.687522 | 2.685026 | 2.264579 | 2.769817 | 2.39491 | 1.294668 | 4.483821 | 3.505628 |
| LOC100912 | 0 | 0 | 0 | 0 | 0 | 0.03965 | 0 | 0 |
| Ssna1 | 23.77349 | 26.02619 | 30.14397 | 25.81395 | 27.23439 | 28.33741 | 27.0999 | 24.43169 |
| Zfp449 | 1.544907 | 1.256368 | 1.071151 | 1.453229 | 1.444274 | 1.509788 | 1.494323 | 1.668869 |
| AABR07014 | 0 | 0 | 0 | 0 | 0 | 0 | 0 | 0 |
| Susd1 | 0.221889 | 0.401692 | 0.38114 | 0.306694 | 0.239926 | 0.466927 | 0.397184 | 0.354009 |
| Pias4 | 8.78023 | 9.359371 | 9.84006 | 8.591254 | 7.314689 | 8.282432 | 9.960177 | 9.180382 |
| Tnfrsf14 | 1.018792 | 1.412076 | 1.503891 | 1.666329 | 0.881284 | 1.161261 | 1.082791 | 0.970732 |
| LOC103691 | 0.038732 | 0 | 0.083163 | 0.03569 | 0 | 0.03396 | 0 | 0.068661 |
| Cox4i2 | 8.189931 | 8.480271 | 7.833241 | 8.324238 | 7.406547 | 7.616147 | 6.663659 | 6.863189 |
| Ppp1r18 | 15.63518 | 13.77426 | 15.11964 | 15.80943 | 15.38971 | 14.96472 | 20.26262 | 18.93607 |
| Nhlh1 | 0 | 0 | 0 | 0 | 0 | 0 | 0 | 0.018929 |

| | | | | | | | | |
|---|---|---|---|---|---|---|---|---|
| Sec61a2 | 22.01144 | 20.38587 | 22.63019 | 20.20089 | 20.3937 | 21.78993 | 20.10745 | 20.17852 |
| Ero1b | 2.897719 | 2.31586 | 2.147993 | 2.776092 | 2.660786 | 2.823025 | 3.21611 | 3.261451 |
| LOC102547 | 0.341517 | 0.38641 | 0.611067 | 0.524491 | 0.738555 | 1.297583 | 0.63679 | 1.109912 |
| AABR07052 | 0 | 0.022727 | 0.021564 | 0.037018 | 0.065158 | 0.035224 | 0.022472 | 0.017804 |
| Cope | 58.56569 | 60.75274 | 65.54824 | 65.54469 | 65.00217 | 67.65834 | 67.06891 | 66.727 |
| AABR07018 | 0 | 0 | 0 | 0.118267 | 0.138781 | 0.225071 | 0 | 0.227522 |
| Tmem63c | 0.286937 | 0.365237 | 0.34655 | 0.429651 | 0.41368 | 0.534621 | 0.695526 | 0.445071 |
| Xpo4 | 2.500907 | 2.643581 | 2.526591 | 2.544864 | 2.507971 | 2.491137 | 2.639292 | 2.648955 |
| Tmem132e | 1.148751 | 1.396548 | 1.154539 | 1.475183 | 1.691408 | 1.789433 | 2.296902 | 2.123044 |
| AABR07054 | 1.848209 | 1.394108 | 2.204634 | 0.94614 | 0.666147 | 1.620511 | 1.148719 | 1.274124 |
| #N/A | 0 | 0 | 0 | 0.438665 | 0 | 0 | 0 | 0 |
| AABR07060 | 0 | 0.12267 | 0 | 0 | 0 | 0.095061 | 0 | 0 |
| AABR07036 | 0 | 0 | 0 | 0 | 0 | 0 | 0 | 0 |
| Tmem86a | 16.53965 | 17.14344 | 15.05566 | 11.72357 | 10.6304 | 10.14124 | 9.478358 | 9.457182 |
| Rn50_X_07 | 0 | 0 | 0.106407 | 0.091331 | 0.035724 | 0.057936 | 0 | 0.058567 |
| Mylk4 | 0.053874 | 0.060956 | 0.115675 | 0.148929 | 0 | 0.188949 | 0 | 0 |
| Ucn3 | 0 | 0 | 0 | 0 | 0.110807 | 0 | 0 | 0 |
| Ccne1 | 0.348391 | 0.121289 | 0.258937 | 0.246945 | 0.463644 | 0.281972 | 0.359783 | 0.190029 |
| Sf3b5 | 40.89249 | 46.18471 | 48.7961 | 52.99712 | 50.41948 | 49.65471 | 56.19849 | 47.97905 |
| AABR07005 | 7.474375 | 6.791149 | 7.464955 | 6.031642 | 5.951242 | 5.441423 | 6.005435 | 6.524532 |
| Diaph1 | 5.407147 | 5.100044 | 5.120802 | 5.301973 | 5.289361 | 5.291505 | 7.055741 | 6.960516 |
| Lman2l | 18.34344 | 18.82227 | 18.66604 | 14.8568 | 16.2148 | 16.92217 | 20.13967 | 21.58744 |
| Gcdh | 57.83991 | 63.35755 | 59.09653 | 66.03602 | 60.06516 | 59.43171 | 58.61591 | 56.24202 |
| Nfu1 | 35.50872 | 31.8954 | 33.91339 | 33.89467 | 32.6256 | 37.26153 | 32.27703 | 32.47761 |
| AABR07049 | 0.065376 | 0.07397 | 0 | 0 | 0 | 0 | 0 | 0 |
| LOC103695 | 0.238027 | 0.628405 | 0.170358 | 0.146222 | 0.171583 | 0.069567 | 0.443823 | 0.070325 |
| Camp | 0.070384 | 0.079637 | 0.075562 | 0 | 0.076106 | 0 | 0 | 0.062385 |
| Foxj3 | 8.570154 | 8.148199 | 8.232736 | 8.457561 | 8.233226 | 8.03813 | 9.077524 | 9.261723 |
| Arf2 | 8.587338 | 6.912904 | 7.101513 | 9.264957 | 8.661138 | 9.069007 | 10.06461 | 10.02059 |
| Cyb5d1 | 4.725891 | 4.484684 | 5.646366 | 4.42496 | 4.038578 | 4.945665 | 5.798773 | 5.675138 |
| Cldn23 | 0 | 0 | 0 | 0 | 0.032844 | 0 | 0 | 0.026923 |
| Prss12 | 0.922009 | 1.138047 | 1.282287 | 1.158539 | 0.770374 | 0.661433 | 1.078389 | 1.151541 |
| Izumo1 | 0.460905 | 0.441263 | 0.266437 | 0.294027 | 0.575042 | 0.466295 | 0.555305 | 0.722772 |
| Gorasp1 | 13.05445 | 14.12414 | 14.80979 | 13.3744 | 12.03371 | 13.20853 | 14.43905 | 13.82121 |
| Rn50_5_16 | 0 | 0.541587 | 0.102775 | 0.176428 | 0 | 0.083939 | 0 | 0 |
| AABR07065 | 0 | 0 | 0.191218 | 0.492379 | 0.385187 | 0 | 0.199268 | 0 |
| AABR07073 | 10.09135 | 13.73232 | 12.00492 | 12.44026 | 10.17436 | 11.59819 | 10.06924 | 15.35063 |
| Atp6v0e1 | 42.97055 | 39.03171 | 40.65064 | 41.09847 | 41.21137 | 37.86313 | 44.02774 | 43.55486 |
| Ube2j2 | 29.11109 | 29.70184 | 27.48768 | 27.5777 | 26.02868 | 27.31583 | 30.73981 | 29.78617 |
| AABR07073 | 0.038112 | 0.043122 | 0.040916 | 0.140475 | 0.04121 | 0.033417 | 0.042638 | 0.067561 |
| Cep112 | 0.440177 | 0.566735 | 0.602919 | 0.615402 | 0.328246 | 0.359331 | 0.64528 | 0.403605 |
| Kdm5b | 4.47377 | 4.163969 | 4.440798 | 4.113262 | 4.129507 | 4.270508 | 4.250412 | 4.695085 |
| Adrb2 | 2.846531 | 3.162685 | 3.276181 | 2.85927 | 3.632492 | 3.552639 | 4.332167 | 3.29584 |
| Rn50_1_13 | 19.63722 | 14.24269 | 14.5951 | 14.5378 | 18.14824 | 15.89348 | 16.71165 | 15.9178 |
| Med7 | 3.172338 | 2.780484 | 4.125223 | 5.022937 | 3.961643 | 4.505261 | 3.699032 | 3.92068 |
| Myl7 | 9.766502 | 10.7359 | 11.3374 | 25.20577 | 22.45153 | 23.91452 | 52.32201 | 48.66662 |
| AABR07028 | 1.007037 | 0 | 0 | 0.927945 | 0 | 0.441485 | 0 | 0.446293 |
| Zfp791 | 0.109724 | 0.031037 | 0.088347 | 0.101107 | 0.059322 | 0 | 0.030689 | 0 |
| Vcpip1 | 3.5011 | 3.506984 | 3.826014 | 3.307068 | 3.256507 | 3.520881 | 4.253811 | 4.382296 |
| Ets2 | 13.95362 | 13.82053 | 14.01565 | 12.35035 | 12.70638 | 11.09113 | 12.01196 | 13.08699 |
| LOC100911 | 31.303 | 35.22948 | 71.36757 | 44.5325 | 32.31714 | 31.86276 | 31.29483 | 33.13214 |
| Mnda | 5.088851 | 4.791972 | 4.300073 | 8.016979 | 8.555502 | 9.470762 | 11.79039 | 14.20081 |
| Samd5 | 0.946372 | 0.80903 | 0.970836 | 0.678257 | 0.886859 | 0.479428 | 0.752895 | 0.857457 |
| Rn50_5_08 | 0.149191 | 0.03376 | 0.064066 | 0 | 0 | 0.026162 | 0 | 0.026447 |

| | | | | | | | | |
|---|---|---|---|---|---|---|---|---|
| Elp3 | 20.45307 | 22.15419 | 21.69288 | 21.10201 | 22.69004 | 23.02379 | 19.88907 | 19.50755 |
| Cpsf3 | 10.20115 | 8.994898 | 9.466202 | 9.616052 | 9.686431 | 9.993032 | 9.366201 | 9.686147 |
| Fam98c | 13.37002 | 13.63347 | 14.92949 | 13.955 | 12.53816 | 12.55502 | 18.65107 | 17.33686 |
| Rab7b | 1.317764 | 1.101037 | 1.197057 | 1.681294 | 2.455177 | 1.582032 | 2.517576 | 2.605538 |
| AC126134. | 0 | 0.85869 | 0 | 0 | 0 | 0 | 0 | 0 |
| Alkbh2 | 3.291572 | 4.401397 | 3.426631 | 3.860251 | 3.990539 | 3.541975 | 3.62667 | 2.696462 |
| AABR07017 | 2.514748 | 2.175832 | 1.588084 | 2.726166 | 3.039061 | 2.464337 | 4.964802 | 4.064548 |
| LOC100911 | 0 | 0 | 0 | 0.032169 | 0 | 0.03061 | 0.117169 | 0.030943 |
| Casp4 | 6.979268 | 6.8309 | 5.929798 | 8.995679 | 9.352206 | 9.235457 | 8.143412 | 7.704127 |
| Wt1 | 7.27249 | 7.256434 | 8.086321 | 6.093776 | 5.897728 | 5.570701 | 8.359659 | 9.243944 |
| Adat2 | 1.477698 | 1.985436 | 1.983004 | 1.787153 | 0.998633 | 2.672272 | 1.963155 | 1.964636 |
| Cdx1 | 0.128348 | 0.07261 | 0.068895 | 0.118267 | 0.138781 | 0.056268 | 0.215385 | 0.227522 |
| Dmd | 35.94485 | 33.13536 | 31.65069 | 36.55712 | 37.24886 | 35.92513 | 33.98459 | 32.84887 |
| LOC100361 | 7.480846 | 6.066028 | 9.503546 | 6.893305 | 6.471144 | 6.231252 | 9.764111 | 8.840861 |
| Pfkfb3 | 18.59232 | 18.51103 | 20.43475 | 20.39165 | 19.48889 | 20.16028 | 18.04417 | 18.79349 |
| Meis3 | 1.834924 | 1.884488 | 1.757765 | 3.121497 | 3.601863 | 3.291982 | 2.747637 | 2.477109 |
| Galnt14 | 0.09127 | 0.07745 | 0.048992 | 0.147177 | 0.049344 | 0.16005 | 0.127635 | 0.080897 |
| Znrf4 | 0.396378 | 0.34882 | 0.520101 | 0.811659 | 0.571464 | 0.502009 | 0.541994 | 0.936878 |
| AC120450. | 0.489401 | 1.107469 | 2.101614 | 0.901928 | 0.529182 | 0.858215 | 1.09504 | 0.433781 |
| RGD156511 | 0 | 0 | 0 | 0 | 0 | 0.140411 | 0 | 0.14194 |
| Txnrd2 | 27.47545 | 29.4288 | 28.78141 | 25.46489 | 24.69491 | 23.31942 | 24.71587 | 25.81734 |
| Amfr | 67.49385 | 67.7138 | 73.05386 | 61.37595 | 55.9035 | 61.02497 | 72.19243 | 71.29172 |
| Rn50_1_09 | 0.220953 | 0 | 0 | 0.407199 | 0 | 0 | 0 | 0 |
| Olig3 | 0 | 0.028623 | 0 | 0 | 0 | 0 | 0 | 0 |
| Stox1 | 0 | 0.019929 | 0 | 0 | 0 | 0 | 0 | 0.015612 |
| AABR07072 | 0 | 0.987493 | 0 | 0.402109 | 0 | 0.382621 | 0.976411 | 0 |
| Harbi1 | 1.860938 | 1.689347 | 1.672606 | 1.894235 | 2.503557 | 2.20086 | 2.445063 | 2.435803 |
| Pkp1 | 0.125087 | 0.14153 | 0.146497 | 0.19909 | 0.282805 | 0.219353 | 0.318049 | 0.191504 |
| AC119459. | 0 | 0 | 1.58361 | 0.679622 | 1.595 | 1.293366 | 1.650272 | 2.614902 |
| Nop56 | 11.59413 | 12.4965 | 11.41471 | 16.17721 | 12.56628 | 14.79092 | 13.8009 | 13.61265 |
| Fxyd1 | 523.0697 | 550.4764 | 582.8547 | 525.8053 | 523.9441 | 535.6403 | 494.0677 | 484.5223 |
| Scly | 3.01472 | 3.330439 | 3.848115 | 4.33097 | 3.79879 | 4.204318 | 5.019266 | 4.081784 |
| AABR07034 | 0 | 0.306199 | 0 | 2.867757 | 1.02418 | 2.135557 | 0 | 0.359802 |
| #N/A | 0 | 0 | 0 | 0 | 0 | 0 | 0 | 0 |
| Fbxo4 | 3.731039 | 2.735053 | 2.397664 | 1.912693 | 2.642195 | 2.672394 | 3.409845 | 3.283717 |
| Tab3 | 1.196238 | 1.298242 | 1.205611 | 1.192269 | 1.583847 | 1.369943 | 1.037864 | 1.060285 |
| Hsd17b8 | 54.85954 | 55.51312 | 54.6264 | 51.85504 | 55.67516 | 49.75547 | 50.29083 | 52.32834 |
| AABR07005 | 12.03814 | 9.398209 | 11.1144 | 10.87082 | 10.28317 | 10.6606 | 11.44758 | 9.923104 |
| RGD130999 | 7.96006 | 7.248701 | 6.257942 | 7.676923 | 8.488174 | 7.955779 | 9.059167 | 9.967728 |
| Lrp6 | 8.297018 | 8.35108 | 7.824348 | 8.546506 | 8.103241 | 8.132288 | 10.82555 | 10.77561 |
| Creld1 | 22.10267 | 24.57396 | 22.52022 | 26.44847 | 25.00574 | 25.92924 | 28.84873 | 28.63778 |
| Mir1b | 0.61607 | 3.485269 | 1.98417 | 0.567684 | 1.998442 | 1.620511 | 1.378463 | 1.092106 |
| Nrip2 | 2.01943 | 1.418208 | 2.018471 | 2.053325 | 1.807101 | 2.014864 | 1.558103 | 1.543036 |
| Amotl1 | 12.37019 | 12.01722 | 11.94442 | 9.95304 | 10.3145 | 10.83038 | 14.32339 | 14.8162 |
| Rbmxl1 | 8.530353 | 8.469853 | 8.335539 | 7.571636 | 8.960211 | 9.555342 | 10.20557 | 8.702722 |
| Spns3 | 0.31494 | 0.129578 | 0.122948 | 0.263823 | 0.154791 | 0.175725 | 0.160155 | 0.203016 |
| Zscan29 | 2.874871 | 2.343401 | 2.090758 | 2.335748 | 3.041705 | 2.574897 | 3.90795 | 3.068728 |
| Klc2 | 24.15508 | 24.97498 | 26.64487 | 24.6888 | 23.73178 | 24.8769 | 26.74255 | 27.27927 |
| AABR07014 | 2.727392 | 1.542958 | 0.878409 | 1.759229 | 1.179636 | 1.673966 | 3.356413 | 2.175681 |
| Vstm2b | 0.051164 | 0.086834 | 0 | 0.070718 | 0.027661 | 0.06729 | 0 | 0.045349 |
| Twist1 | 3.710202 | 3.645561 | 3.633741 | 3.7187 | 3.906214 | 3.823829 | 4.332862 | 5.134732 |
| Rn50_7_00 | 0.112373 | 0 | 0 | 0 | 0.121508 | 0 | 0.125718 | 0 |
| Sel1l | 15.21724 | 15.66585 | 14.50585 | 14.81331 | 15.0873 | 15.64292 | 18.45197 | 18.59329 |
| Fam169a | 1.419558 | 1.130263 | 0.931325 | 1.308067 | 1.136998 | 1.014175 | 0.794069 | 0.838817 |

| | | | | | | | | |
|---|---|---|---|---|---|---|---|---|
| Myo9a | 3.407017 | 2.93322 | 2.685979 | 2.799873 | 2.726269 | 2.675511 | 3.290867 | 3.747543 |
| Tsx | 0.065869 | 0.223583 | 0.070715 | 0 | 0.21367 | 0.173262 | 0.073691 | 0 |
| Lin54 | 2.040704 | 2.116547 | 1.956095 | 2.137868 | 1.825685 | 1.97035 | 2.228691 | 2.605501 |
| Prr22 | 0.299893 | 0.339315 | 0.52008 | 0.425138 | 0.274382 | 0.404533 | 0.387123 | 0.470279 |
| AABR07062 | 0 | 0 | 0 | 0.207988 | 0 | 0 | 0 | 0 |
| AABR07035 | 1.43687 | 1.264473 | 1.371175 | 1.912472 | 1.208407 | 1.679798 | 1.428894 | 0.849046 |
| Mrpl28 | 69.88888 | 76.32019 | 79.82398 | 79.42441 | 76.88664 | 73.61741 | 71.52779 | 72.6801 |
| AABR07024 | 0 | 0 | 0 | 0 | 0 | 0 | 0 | 0 |
| Zfp113 | 2.107361 | 1.934496 | 1.664775 | 1.722018 | 1.676749 | 1.708284 | 2.090721 | 1.62116 |
| Sh2d5 | 0 | 0.121314 | 0.046043 | 0 | 0.023187 | 0.056406 | 0 | 0.05702 |
| Grid2ip | 0 | 0 | 0.016295 | 0 | 0 | 0.026617 | 0 | 0.026907 |
| C4bpb | 0.329642 | 0.372974 | 0.556115 | 0.867862 | 0.407356 | 0.61935 | 0.526841 | 0.876533 |
| Cav2 | 36.51086 | 33.97262 | 32.25706 | 27.1436 | 28.2088 | 27.56346 | 27.08981 | 27.91963 |
| Fgd4 | 0.762326 | 0.829361 | 0.755451 | 0.83754 | 1.014513 | 0.951196 | 1.033268 | 1.156465 |
| Gpr22 | 9.051436 | 9.001024 | 7.783157 | 8.210533 | 7.97996 | 8.526296 | 7.637255 | 7.974639 |
| AABR07065 | 0.150477 | 0.340515 | 0.484639 | 0 | 0.162708 | 0.131938 | 0.168347 | 0.26675 |
| B4galt4 | 2.497113 | 2.243673 | 2.417985 | 3.428928 | 3.467765 | 3.112483 | 2.98538 | 3.016184 |
| Plaa | 14.92837 | 13.89968 | 12.45814 | 13.64844 | 14.04003 | 14.52084 | 13.87417 | 14.0932 |
| Nfkbie | 3.007281 | 2.773857 | 3.719803 | 4.397602 | 4.064662 | 3.897859 | 5.375747 | 4.722576 |
| AABR07027 | 0 | 0 | 0 | 0 | 0.191941 | 0 | 0 | 0 |
| Ebf3 | 4.022326 | 3.768092 | 3.935155 | 3.736305 | 3.671168 | 3.981847 | 4.887096 | 4.89734 |
| AABR07018 | 0 | 0.069055 | 0.065522 | 0 | 0.065994 | 0 | 0 | 0 |
| AABR07028 | 1.598378 | 1.377897 | 0.817124 | 1.402707 | 0.740701 | 1.267987 | 1.19213 | 1.14687 |
| Col18a1 | 6.31696 | 6.165175 | 7.163409 | 10.74541 | 10.40398 | 10.28881 | 10.6826 | 11.44836 |
| Cul2 | 7.949759 | 7.427744 | 7.672196 | 7.886918 | 7.6525 | 8.160416 | 6.957026 | 8.507078 |
| Cct2 | 51.28811 | 43.24743 | 42.67162 | 51.81398 | 46.4748 | 47.98894 | 52.87913 | 48.67466 |
| Usp21 | 8.125339 | 8.658937 | 8.62164 | 7.944246 | 7.942987 | 7.973422 | 8.984568 | 9.462951 |
| Slc22a20 | 0 | 0 | 0.038192 | 0 | 0.038466 | 0 | 0.039799 | 0 |
| AABR07060 | 15.17853 | 6.869516 | 10.59183 | 10.48981 | 22.15664 | 6.654273 | 10.18864 | 20.18023 |
| Ttbk1 | 0.009783 | 0.011068 | 0.010502 | 0 | 0.031733 | 0.017155 | 0.010944 | 0.008671 |
| Mir100 | 1.963722 | 1.481239 | 0 | 0.603164 | 0.707781 | 1.147862 | 0.732308 | 4.06127 |
| Nkx1-1 | 0 | 0 | 0.042206 | 0 | 0 | 0 | 0 | 0 |
| AABR07002 | 0.142376 | 0.032218 | 0 | 0.026239 | 0.03079 | 0.099868 | 0 | 0.050478 |
| Ltv1 | 7.152148 | 6.869061 | 6.874746 | 8.020902 | 8.602785 | 7.802303 | 8.807859 | 8.280409 |
| Emid1 | 1.275622 | 1.480314 | 1.580148 | 1.175436 | 0.990275 | 1.290538 | 1.975997 | 1.420557 |
| Pde3a | 25.80084 | 27.11714 | 26.61584 | 16.95761 | 18.41141 | 16.79243 | 22.55493 | 20.63319 |
| Per1 | 40.38992 | 40.94352 | 44.06926 | 34.15497 | 32.34573 | 35.75411 | 42.00328 | 41.6229 |
| Cp | 4.727688 | 3.918172 | 3.976328 | 10.32212 | 10.59841 | 10.82515 | 12.0774 | 12.41101 |
| Hpse | 3.256587 | 2.8372 | 2.796923 | 2.79076 | 2.852253 | 3.055255 | 4.554156 | 4.271983 |
| LOC691113 | 5.370864 | 4.227394 | 4.763189 | 5.218009 | 4.797448 | 3.839004 | 5.616813 | 5.847091 |
| AC099294. | 0.781581 | 6.190254 | 7.551693 | 7.20196 | 5.915785 | 10.27936 | 5.246388 | 2.771016 |
| Trex1 | 16.39326 | 17.32716 | 18.04057 | 16.95252 | 16.33662 | 18.11347 | 19.4324 | 19.49501 |
| AABR07010 | 0.096527 | 0.054608 | 0.090674 | 0.055591 | 0.065233 | 0.063476 | 0.134988 | 0.053473 |
| Mia3 | 16.92717 | 16.81086 | 15.60362 | 17.38543 | 17.30666 | 16.07503 | 19.10016 | 18.77216 |
| AABR07058 | 0 | 0 | 0 | 0.368345 | 0 | 0 | 0 | 0 |
| Poglut1 | 2.680082 | 2.977913 | 3.049529 | 3.445596 | 3.696168 | 3.93995 | 3.967886 | 3.584572 |
| Olr1307 | 0 | 0 | 0 | 0 | 0 | 0 | 0 | 0.049588 |
| Polq | 0.098259 | 0.145918 | 0.131859 | 0.19806 | 0.106246 | 0.161538 | 0.123669 | 0.043546 |
| #N/A | 0 | 0.697054 | 0.66139 | 0 | 0 | 0 | 0 | 0 |
| Ecm2 | 0.025821 | 0 | 0 | 0.023793 | 0 | 0.045281 | 0.028888 | 0.068661 |
| Rsg1 | 0.96877 | 0.799869 | 0.983818 | 1.061569 | 1.613742 | 0.757589 | 1.933294 | 1.369228 |
| Oc90 | 0.027388 | 0.030988 | 0.117611 | 0.100948 | 0 | 0.192111 | 0 | 0 |
| Zdhhc17 | 9.326432 | 7.637159 | 7.636008 | 7.574037 | 7.514353 | 8.304699 | 8.972426 | 8.314728 |
| AC127640. | 0.020404 | 0.011543 | 0.010952 | 0.056403 | 0.011031 | 0 | 0 | 0.009042 |

| | | | | | | | | |
|---|---|---|---|---|---|---|---|---|
| Hfm1 | 0 | 0 | 0 | 0.011209 | 0 | 0 | 0 | 0 |
| AC141028. | 0 | 0 | 0 | 0 | 0 | 0 | 0 | 0 |
| Awat2 | 0 | 0 | 0 | 0.051829 | 0 | 0 | 0.062927 | 0.049854 |
| Spag1 | 2.08029 | 1.899233 | 1.771257 | 1.890465 | 2.078744 | 1.710786 | 2.070527 | 2.009176 |
| Hmox1 | 10.43306 | 9.647919 | 10.26717 | 16.91633 | 18.22334 | 16.74149 | 19.49081 | 19.91734 |
| Vdac2 | 203.2073 | 192.4372 | 186.7222 | 188.0546 | 189.3506 | 189.4727 | 178.2388 | 178.3382 |
| 1700025F2 | 0.62902 | 0.355853 | 0.337647 | 1.449043 | 0.340075 | 0.689407 | 0.17593 | 0.278766 |
| Pygl | 0.982543 | 1.070527 | 0.546945 | 1.592789 | 1.377198 | 1.228428 | 1.811687 | 1.612735 |
| AABR07059 | 0 | 0 | 0 | 0 | 0 | 0 | 0 | 0.170017 |
| Gckr | 2.510453 | 2.920472 | 2.125737 | 2.313283 | 2.102794 | 2.201167 | 2.294335 | 2.193799 |
| Rbm15 | 2.964109 | 3.497997 | 3.507219 | 3.024999 | 3.532445 | 2.878388 | 3.440913 | 4.082105 |
| Bccip | 18.66397 | 16.09248 | 16.29512 | 15.72177 | 16.41232 | 15.69916 | 15.69176 | 15.32202 |
| Btbd19 | 3.423396 | 3.445195 | 3.305864 | 3.645933 | 2.846007 | 3.469229 | 4.253355 | 4.086429 |
| Calcb | 0 | 0 | 0 | 0 | 0.05892 | 0 | 0 | 0 |
| Cycs | 613.5977 | 593.7795 | 686.8922 | 608.8339 | 587.6473 | 437.565 | 508.5637 | 404.7346 |
| Brms1 | 0.070957 | 0.40142 | 0 | 0.261535 | 0.076724 | 0.062215 | 0.079383 | 0.125785 |
| AABR07015 | 3.394087 | 2.194429 | 4.164308 | 2.085012 | 2.446652 | 3.401073 | 2.712253 | 2.578584 |
| Zfp319 | 3.562466 | 3.556555 | 3.424583 | 3.046663 | 3.751336 | 3.511468 | 4.506513 | 4.251396 |
| Ier3 | 25.35224 | 25.55181 | 28.47242 | 33.5107 | 34.79856 | 34.69214 | 37.7255 | 39.21509 |
| Dnmt3l | 0.279202 | 0.210603 | 0 | 0.14293 | 0.436074 | 0.408008 | 0.10412 | 0.302464 |
| AABR07070 | 0.246235 | 0 | 0 | 0.075632 | 0 | 0.071966 | 0 | 0.1455 |
| LOC100359 | 1.043524 | 1.610043 | 1.731356 | 2.622453 | 1.230924 | 1.663568 | 2.334896 | 2.522528 |
| LOC498154 | 3.595164 | 3.821228 | 3.898624 | 4.818621 | 4.397865 | 3.852741 | 4.469011 | 4.313138 |
| Ms4a18 | 0 | 0 | 0 | 0 | 0 | 0.045014 | 0 | 0 |
| Rsf1 | 4.112635 | 3.355313 | 3.262252 | 3.609708 | 3.417672 | 3.712964 | 4.300669 | 4.250968 |
| Nlrp10 | 0.07451 | 0.067444 | 0.063993 | 0.24717 | 0.161134 | 0.235191 | 0.100031 | 0.066042 |
| Lysmd4 | 8.82036 | 8.76606 | 9.895761 | 7.065899 | 6.916711 | 7.245989 | 7.912041 | 7.67706 |
| Sage1 | 0 | 0.128246 | 0.243369 | 0 | 0 | 0 | 0 | 0 |
| Asb8 | 32.88012 | 31.52406 | 28.87079 | 31.98803 | 31.24904 | 30.68047 | 28.88576 | 29.72615 |
| Dsg4 | 0.117375 | 0.09486 | 0.108008 | 0.154509 | 0.126916 | 0.088212 | 0.187591 | 0.059449 |
| LOC691807 | 36.51068 | 41.74901 | 37.53083 | 40.65773 | 42.83388 | 42.17331 | 41.93143 | 42.28878 |
| Cel | 0.076187 | 0.086202 | 0.081792 | 0.140407 | 0.08238 | 0.111335 | 0.085235 | 0.135057 |
| Stradb | 14.36666 | 13.63421 | 11.23885 | 13.60775 | 13.0056 | 13.72943 | 12.98282 | 13.54333 |
| Tmem132b | 0 | 0.036755 | 0 | 0.089801 | 0.035126 | 0.056966 | 0.072686 | 0.057586 |
| Fam120b | 5.167339 | 4.987693 | 5.06135 | 5.27692 | 5.457765 | 4.939427 | 6.436566 | 6.515975 |
| Cpa5 | 0 | 0 | 0 | 0 | 0 | 0 | 0 | 0 |
| RGD156371 | 0.14611 | 0.09919 | 0.282346 | 0.188489 | 0.221182 | 0.179353 | 0.098077 | 0.077703 |
| Il1rapl1 | 0 | 0 | 0.025967 | 0.022288 | 0 | 0.063623 | 0.02706 | 0.021439 |
| Ptprn | 0.507141 | 0.524623 | 0.435559 | 0.747697 | 0.70504 | 0.736868 | 0.615998 | 0.667835 |
| Sbk2 | 0.578875 | 0.428249 | 0.52585 | 0.225674 | 0.264816 | 0.253779 | 0.273993 | 0.138138 |
| Def6 | 0.950955 | 1.099871 | 1.111659 | 1.285192 | 1.165354 | 1.074673 | 1.489441 | 1.723219 |
| LOC691170 | 4.652251 | 3.636347 | 3.739835 | 4.473252 | 4.301367 | 3.980569 | 3.520109 | 4.02392 |
| Cybb | 5.068855 | 4.848574 | 4.534783 | 6.972279 | 7.307839 | 7.289206 | 9.944463 | 9.625831 |
| 7420426K0 | 0 | 0 | 0 | 0 | 0 | 0.083633 | 0 | 0 |
| Ism2 | 0.071101 | 0.080447 | 0.076332 | 0.131034 | 0.115321 | 0.062341 | 0.119317 | 0.283592 |
| AABR07032 | 0.131904 | 0.089546 | 0.056643 | 0.121544 | 0.171151 | 0.092523 | 0.118055 | 0.116913 |
| Aplf | 1.889806 | 2.023268 | 2.028828 | 2.265669 | 2.746532 | 2.512201 | 2.387035 | 2.935804 |
| Dcaf5 | 6.592181 | 6.445561 | 6.616909 | 5.854983 | 6.406986 | 6.606942 | 7.076627 | 7.455707 |
| Wdr89 | 1.099285 | 0.904574 | 0.751006 | 0.943883 | 1.053568 | 1.029571 | 0.614915 | 0.797196 |
| Col23a1 | 0.019161 | 0.086717 | 0.143991 | 0.035311 | 0.082872 | 0.0672 | 0.085744 | 0.135864 |
| Efcab3 | 0 | 0 | 0 | 0 | 0 | 0 | 0 | 0 |
| Hist2h2ac | 0.138534 | 1.253959 | 0.446176 | 0.510615 | 0.149795 | 0.242934 | 0.774929 | 0.491159 |
| Prr14 | 10.00603 | 9.678502 | 10.97932 | 9.336502 | 10.23915 | 10.84899 | 12.21838 | 10.99512 |
| Sh2d1b | 0.510531 | 0.247561 | 0.391491 | 0.672049 | 0.630892 | 0.831321 | 0.897537 | 1.745393 |

| | | | | | | | | |
|---|---|---|---|---|---|---|---|---|
| 5S_rRNA | 0 | 0 | 0 | 0 | 0 | 0 | 0 | 0.446293 |
| Tp53bp2 | 2.679416 | 2.891927 | 2.876525 | 2.992354 | 2.603487 | 2.695757 | 3.080495 | 2.823614 |
| Rhou | 1.090957 | 0.771479 | 0.658807 | 1.130933 | 0.811 | 1.853319 | 2.288464 | 2.417423 |
| Bin1 | 5.906633 | 5.388726 | 6.441403 | 7.056187 | 6.083828 | 6.284328 | 7.443883 | 6.828707 |
| #N/A | 0.969211 | 0.645069 | 0.979304 | 1.129496 | 1.140464 | 0.52488 | 0.829179 | 1.035926 |
| AABR07029 | 0 | 0 | 0 | 0.365554 | 0 | 0 | 0 | 0 |
| Grm1 | 0 | 0 | 0 | 0 | 0 | 0.006696 | 0.017088 | 0.006769 |
| Cacng7 | 0.529713 | 0.742047 | 0.595761 | 0.511353 | 0.600046 | 0.862555 | 0.53618 | 1.006095 |
| Angel1 | 2.613812 | 2.450007 | 2.66854 | 2.089749 | 1.662516 | 1.977233 | 1.978146 | 2.248611 |
| Hcfc2 | 5.287292 | 4.42528 | 5.442976 | 4.961019 | 5.50823 | 4.635902 | 6.347347 | 6.398313 |
| Rgs12h | 0.026923 | 0 | 0 | 0 | 0 | 0 | 0 | 0 |
| Ddit4 | 27.58344 | 30.27929 | 28.82815 | 28.61523 | 27.38949 | 25.70677 | 39.71495 | 39.04756 |
| Ankrd39 | 1.112093 | 1.236206 | 0.942555 | 1.096662 | 1.075912 | 1.351432 | 1.004059 | 1.36615 |
| AABR07044 | 0.096367 | 0.174456 | 0.082765 | 0.088799 | 0.02084 | 0.202788 | 0.086249 | 0.068332 |
| #N/A | 0 | 0 | 0 | 0 | 0 | 0 | 0 | 0.156278 |
| AABR07061 | 0 | 0 | 0 | 0.226541 | 0 | 0 | 0 | 0 |
| RGD156568 | 4.70931 | 3.916575 | 4.839688 | 5.118319 | 5.919033 | 5.43492 | 5.133476 | 4.780588 |
| AABR07050 | 0 | 1.346581 | 0 | 0.365554 | 0 | 0 | 0 | 0.351625 |
| Cmtr2 | 1.347953 | 1.409604 | 1.074372 | 1.505558 | 1.347103 | 1.719107 | 1.096749 | 1.59301 |
| Gdi2 | 52.53492 | 52.39312 | 49.86802 | 54.45997 | 53.80313 | 54.5724 | 56.775 | 56.04409 |
| Cdc123 | 15.3687 | 17.11654 | 15.59462 | 18.48779 | 17.26878 | 18.29543 | 16.0715 | 16.00501 |
| Fli1 | 22.24113 | 21.12801 | 23.56838 | 16.43788 | 18.66676 | 18.44148 | 21.85659 | 20.504 |
| #N/A | 0.650508 | 1.104029 | 0.698362 | 0 | 0.527539 | 0.427775 | 0.18194 | 0.864867 |
| Adam22 | 0.184225 | 0.156331 | 0.370832 | 0.063658 | 0.1743 | 0.242293 | 0.334917 | 0.183699 |
| Chordc1 | 11.4158 | 11.51409 | 10.32972 | 10.66949 | 9.874994 | 10.73864 | 11.65855 | 12.05534 |
| Nek7 | 36.6822 | 36.55198 | 35.24124 | 32.26478 | 33.86083 | 34.67572 | 37.7157 | 38.74804 |
| Rn50_X_07 | 0.176316 | 0.099747 | 0.473217 | 0.162468 | 0.381296 | 0.386486 | 0.295882 | 0.70325 |
| AABR07017 | 0 | 0 | 0 | 0 | 0.050874 | 0 | 0 | 0 |
| AC130862. | 0 | 0 | 0 | 0 | 0 | 0.141711 | 0 | 0.286509 |
| Pet100 | 16.58035 | 17.62143 | 18.73939 | 18.58371 | 16.31977 | 16.26378 | 15.31913 | 15.27763 |
| Diaph2 | 0.890072 | 1.202895 | 0.796291 | 0.729037 | 0.802019 | 0.802094 | 1.217056 | 1.292945 |
| AABR07037 | 0 | 0 | 0 | 0 | 0 | 0 | 0 | 0.288289 |
| AABR07070 | 0 | 0.569707 | 0.27028 | 0.695959 | 0 | 0.441485 | 1.689942 | 0.223147 |
| Ppfibp2 | 6.733173 | 5.905382 | 5.83542 | 5.460363 | 5.238206 | 4.475145 | 5.419727 | 6.223533 |
| Ano10 | 57.21988 | 59.27431 | 60.56069 | 46.1981 | 45.48715 | 42.96216 | 47.57711 | 45.81375 |
| Olr1675 | 0 | 0 | 0 | 0 | 0.060494 | 0 | 0 | 0 |
| Fam50a | 0.838813 | 0.316359 | 0.68611 | 0.662514 | 3.023323 | 0.735472 | 0.580931 | 0.601866 |
| AABR07018 | 0 | 0 | 0 | 0 | 0 | 0 | 0 | 0 |
| AABR07037 | 0 | 0 | 0 | 0 | 0 | 0 | 0 | 0 |
| AC098214. | 0 | 0 | 1.756818 | 0 | 0 | 0 | 0 | 0 |
| Pld1 | 2.021264 | 1.744967 | 1.542801 | 1.733323 | 1.831831 | 1.577606 | 2.104447 | 1.998662 |
| Ggcx | 8.93806 | 9.713045 | 9.487154 | 10.37652 | 11.43917 | 10.93624 | 11.75083 | 13.18137 |
| Rpl15 | 105.3776 | 106.2362 | 102.4587 | 107.4247 | 106.2924 | 108.9638 | 109.6931 | 103.3563 |
| Agfg2 | 12.81587 | 12.10978 | 12.59977 | 11.30139 | 12.8394 | 11.47862 | 11.66554 | 11.0947 |
| Tnpo3 | 8.839367 | 7.631345 | 7.690645 | 8.145129 | 8.349933 | 9.09719 | 8.826756 | 9.505693 |
| Lipn | 0 | 0 | 0 | 0 | 0 | 0 | 0 | 0 |
| Acot8 | 2.139064 | 2.210805 | 2.296421 | 2.084778 | 1.979342 | 2.236212 | 2.554163 | 2.388178 |
| Cmc2 | 56.51156 | 53.07774 | 56.21816 | 68.15755 | 72.42963 | 57.2018 | 64.19903 | 59.95209 |
| AABR07053 | 86.40377 | 93.31808 | 77.29997 | 78.41134 | 86.34933 | 76.90676 | 103.9878 | 82.38577 |
| Stard4 | 4.596934 | 4.714292 | 5.481113 | 4.560345 | 4.801386 | 4.270716 | 5.799379 | 5.340184 |
| Nkx6-1 | 0 | 0 | 0 | 0.045054 | 0 | 0 | 0 | 0.014446 |
| Laptm5 | 14.65299 | 15.00498 | 14.77753 | 17.10272 | 15.71603 | 18.94719 | 21.36072 | 20.20304 |
| AABR07015 | 0 | 0 | 0 | 0 | 0 | 0 | 0 | 0 |
| #N/A | 0.57545 | 0.217031 | 0.205927 | 0 | 0 | 1.00911 | 0.214596 | 0.340033 |

| | | | | | | | | |
|---|---|---|---|---|---|---|---|---|
| Thap11 | 8.84287 | 9.516433 | 9.895386 | 10.08591 | 9.12563 | 9.192999 | 9.409638 | 9.701604 |
| AABR07034 | 0.387896 | 0 | 0 | 0.357431 | 0.419426 | 0 | 0 | 0.343811 |
| Muc16 | 0.07263 | 0.152614 | 0.066834 | 0.076486 | 0.123409 | 0.100071 | 0.069647 | 0.018393 |
| Olr1687 | 0 | 0 | 0 | 0 | 0.058615 | 0 | 0 | 0 |
| Ddias | 0.110112 | 0.035596 | 0.016887 | 0.115958 | 0.051027 | 0.096546 | 0.158384 | 0.05577 |
| Olr286 | 0 | 0 | 0 | 0.051388 | 0.060301 | 0 | 0 | 0 |
| AABR07019 | 1.483637 | 2.408519 | 1.066469 | 0.998587 | 0.739343 | 0.904942 | 1.428894 | 1.54372 |
| LOC102553 | 0.10288 | 0.349212 | 0.220897 | 0.0948 | 0.111243 | 0.180411 | 0.115098 | 0.273563 |
| AC105811. | 0 | 0 | 0 | 0 | 0 | 0.48331 | 0 | 0 |
| Srl | 85.62298 | 84.53033 | 85.13163 | 75.9556 | 75.47433 | 78.92543 | 83.98639 | 82.3773 |
| Rtn4rl2 | 2.329159 | 3.044265 | 4.052536 | 3.182339 | 2.301375 | 2.711208 | 4.088347 | 3.950929 |
| Ces1f | 1.437746 | 0.976045 | 0.823207 | 0.912658 | 1.450973 | 1.120549 | 0.643395 | 0.849564 |
| LOC100910 | 1.353874 | 1.477136 | 1.50538 | 2.004978 | 7.005925 | 1.65343 | 2.055602 | 2.185725 |
| Dennd3 | 3.785378 | 3.766956 | 4.044261 | 3.193902 | 3.264597 | 3.303028 | 4.204299 | 4.567881 |
| AABR07034 | 0.872765 | 0 | 0.374788 | 0.321688 | 0.754967 | 0.306097 | 0.585847 | 0 |
| Nr4a2 | 6.169136 | 5.124914 | 5.082738 | 4.457041 | 5.119296 | 4.02538 | 6.32852 | 5.468012 |
| Commd7 | 10.06826 | 10.33927 | 11.16139 | 12.45405 | 11.06417 | 12.57011 | 10.71297 | 12.707 |
| Prune2 | 7.589264 | 9.629589 | 8.089363 | 8.841413 | 9.085393 | 10.124 | 10.85575 | 10.61864 |
| Cct6a-ps2 | 0.164569 | 0.186202 | 0.14134 | 0.272959 | 0.035589 | 0 | 0.073645 | 0.08752 |
| Wdr74 | 7.788133 | 8.64406 | 8.002727 | 8.372534 | 6.817158 | 8.552061 | 7.053395 | 7.396081 |
| Cckar | 0 | 0 | 0 | 0 | 0 | 0 | 0 | 0.030902 |
| LOC690096 | 0 | 0 | 0 | 0.223394 | 0 | 0 | 0.406838 | 0 |
| AABR07020 | 0.746751 | 0.7393 | 0.801685 | 0.516076 | 0 | 0.40922 | 0.626574 | 0.579147 |
| Kbtbd12 | 13.90281 | 13.46375 | 11.61355 | 10.89111 | 11.95701 | 10.29299 | 12.8196 | 13.033 |
| Pctp | 1.174001 | 1.050305 | 0.937946 | 1.182428 | 1.151344 | 1.029365 | 1.527233 | 1.330969 |
| Ttc21b | 2.148754 | 1.875936 | 1.851155 | 2.065547 | 2.337758 | 2.081736 | 2.07747 | 1.845765 |
| Fastk | 90.93249 | 95.48424 | 104.7845 | 81.99134 | 75.90725 | 77.27559 | 95.35938 | 95.16563 |
| Aldh6a1 | 209.8299 | 209.9946 | 205.4723 | 178.8696 | 183.4852 | 177.348 | 175.3087 | 176.6828 |
| Mtmr12 | 5.590668 | 4.896466 | 5.068304 | 4.979861 | 4.477858 | 4.792971 | 7.019041 | 6.184932 |
| Mc4r | 1.16492 | 1.757403 | 1.220839 | 1.27789 | 1.019685 | 1.580742 | 2.606521 | 1.991301 |
| Hspa9 | 122.6368 | 115.8231 | 112.0594 | 120.4966 | 118.22 | 110.8817 | 109.686 | 106.3079 |
| Icos | 0.448594 | 0.338376 | 0.192638 | 0.275575 | 0.161686 | 0.235997 | 0.200747 | 0.185552 |
| Kpna6 | 14.56646 | 15.42526 | 14.54658 | 13.92922 | 13.84026 | 13.88248 | 16.09121 | 15.56999 |
| Npdc1 | 36.83005 | 40.53458 | 44.60112 | 45.9028 | 44.42047 | 45.98226 | 53.13694 | 53.23112 |
| Rassf3 | 73.18496 | 72.66627 | 73.90381 | 63.75574 | 64.36723 | 66.42947 | 71.49466 | 70.3977 |
| AABR07034 | 0 | 0 | 0 | 0 | 0 | 0 | 0 | 0 |
| Mkx | 1.141813 | 0.757325 | 0.887655 | 0.834453 | 0.936613 | 1.035665 | 0.528583 | 0.628166 |
| Fam219b | 14.0869 | 12.7385 | 11.37924 | 13.10704 | 12.5003 | 13.55525 | 14.65385 | 15.43094 |
| Zfp503 | 2.431697 | 3.267229 | 3.480776 | 3.765643 | 3.688406 | 3.583136 | 3.6273 | 3.667061 |
| Neil2 | 1.15587 | 1.876426 | 0.863235 | 1.389246 | 1.412846 | 0.969404 | 1.236912 | 1.603573 |
| Crb2 | 0 | 0 | 0 | 0.012527 | 0.0147 | 0.01192 | 0 | 0.012049 |
| Mybbp1a | 11.10941 | 12.05757 | 12.08428 | 10.00012 | 10.37197 | 8.957616 | 13.79751 | 12.53626 |
| Tbc1d30 | 1.011902 | 0.910731 | 0.715998 | 1.165535 | 0.920085 | 1.068717 | 1.646648 | 1.223044 |
| Pld4 | 6.792563 | 6.702441 | 6.755224 | 9.873819 | 8.910431 | 8.748914 | 13.25445 | 12.76458 |
| Homer3 | 9.80822 | 10.03167 | 11.21389 | 13.09728 | 10.3059 | 12.31674 | 13.11181 | 12.15618 |
| Coasy | 9.110483 | 8.059578 | 8.28449 | 9.417566 | 8.623143 | 8.689582 | 10.56776 | 9.218852 |
| Spry1 | 24.62002 | 26.54273 | 30.01072 | 24.44204 | 23.85292 | 25.84158 | 24.81272 | 23.24684 |
| Mir190 | 0 | 0.697054 | 0 | 0 | 0.666147 | 0 | 0 | 0 |
| Fbxw5 | 56.04667 | 56.31137 | 57.13937 | 50.06341 | 51.34849 | 50.60691 | 52.31947 | 53.5198 |
| AABR07051 | 0.758926 | 0 | 0 | 0 | 0 | 1.996282 | 0 | 1.345348 |
| Fbxo16 | 0.082923 | 0.046912 | 0.044512 | 0.038205 | 0.044832 | 0 | 0 | 0.073499 |
| Drc7 | 0 | 0 | 0 | 0 | 0 | 0.016259 | 0 | 0.016436 |
| Pde7a | 2.747523 | 2.950624 | 2.624681 | 3.475771 | 3.801689 | 3.797285 | 4.037627 | 3.818002 |
| Adra1d | 2.576538 | 2.472921 | 2.975919 | 0.818682 | 0.634049 | 0.763424 | 0.6759 | 0.598491 |

| | | | | | | | | |
|---|---|---|---|---|---|---|---|---|
| Gpr160 | 0.457059 | 0.591018 | 0.420585 | 0.812241 | 0.88252 | 0.5725 | 0.62091 | 1.244279 |
| Prep | 11.07373 | 10.27887 | 10.24575 | 9.957276 | 9.843797 | 10.74915 | 11.38314 | 11.30697 |
| Enho | 0.825093 | 1.120265 | 1.240107 | 1.469896 | 1.249026 | 1.253967 | 1.23077 | 0.92634 |
| AABR07029 | 0 | 0 | 0.114966 | 0 | 0 | 0 | 0 | 0 |
| AABR07034 | 0 | 0 | 0 | 0 | 0 | 0.086468 | 0 | 0.08741 |
| Senp17 | 0 | 0 | 0 | 0 | 0 | 0 | 0 | 0 |
| Pdzd4 | 0.062489 | 0.141407 | 0.167715 | 0.071977 | 0.168922 | 0.150674 | 0.13982 | 0.138468 |
| AABR07001 | 0.37139 | 0.210105 | 0.39871 | 0.085555 | 0.100395 | 0.244226 | 0.103874 | 0.082295 |
| Evx2 | 0 | 0 | 0 | 0 | 0 | 0 | 0.052354 | 0 |
| Dnajb4 | 75.68379 | 70.99678 | 69.73976 | 69.06001 | 68.30896 | 66.83219 | 79.11786 | 80.50882 |
| Olr1058 | 0 | 0 | 0 | 0 | 0 | 0 | 0 | 0 |
| Fyco1 | 34.60985 | 33.98936 | 32.77467 | 29.54227 | 28.20385 | 30.27337 | 30.24621 | 31.6596 |
| Rd3l | 17.49253 | 17.26533 | 16.70163 | 12.62058 | 13.60228 | 14.39111 | 11.36717 | 10.9851 |
| Tlcd2 | 0.024759 | 0.028014 | 0.026581 | 0.159703 | 0.267719 | 0.195381 | 0.249296 | 0.153618 |
| Mln | 0 | 0 | 0 | 0 | 0 | 0 | 0 | 0.225313 |
| Avpi1 | 31.35192 | 31.26454 | 28.57584 | 49.05439 | 44.55628 | 43.83902 | 49.34299 | 48.85513 |
| Plpp2 | 0.227678 | 0.331209 | 0.384099 | 0.809214 | 0.562708 | 0.570366 | 0.764148 | 0.432433 |
| Insig2 | 18.9701 | 19.43099 | 16.84843 | 16.3603 | 17.42671 | 16.26235 | 17.02561 | 18.73442 |
| Ibsp | 0.461254 | 0.184196 | 0.320414 | 3.375219 | 3.579247 | 3.401954 | 3.156894 | 2.477044 |
| Sox13 | 8.592377 | 8.708516 | 8.984089 | 9.078088 | 8.125679 | 8.920318 | 11.69501 | 11.60983 |
| Rprm | 0 | 0 | 0.158584 | 0.578493 | 0.199656 | 0.323798 | 0.537095 | 0.392789 |
| Rogdi | 11.2335 | 11.89642 | 14.00858 | 15.14814 | 14.33153 | 12.22184 | 16.55237 | 15.3602 |
| #N/A | 0.919469 | 1.089875 | 0.658072 | 1.371745 | 2.272475 | 2.303402 | 3.281917 | 2.561336 |
| Rcl1 | 8.61013 | 7.72638 | 8.366984 | 9.541194 | 8.146258 | 8.785904 | 10.37999 | 8.750008 |
| Sike1 | 9.43689 | 9.305302 | 9.444651 | 10.11792 | 10.58543 | 10.34284 | 12.11161 | 10.49457 |
| LOC100910 | 0.575162 | 0.897273 | 0.963633 | 1.043919 | 0.631338 | 0.137537 | 0.857955 | 0.679727 |
| Glp2r | 0.9187 | 0.430124 | 0.986284 | 0.525442 | 0.787851 | 0.666635 | 0.460738 | 0.449263 |
| Pcdh9 | 0.045001 | 0.101833 | 0.048311 | 0.027644 | 0.032439 | 0.013152 | 0.016782 | 0.013295 |
| Ecd | 6.120692 | 5.661138 | 5.840848 | 5.550453 | 5.410129 | 4.578671 | 6.630176 | 5.898691 |
| LOC100911 | 0 | 0 | 0 | 0.01453 | 0 | 0 | 0.017641 | 0 |
| Sfi1 | 2.511978 | 2.729993 | 2.270963 | 2.365449 | 2.847208 | 2.801431 | 2.760986 | 2.97842 |
| Anapc13 | 98.95119 | 101.3783 | 99.20315 | 105.1229 | 105.8915 | 103.084 | 97.00708 | 88.0821 |
| AABR07002 | 0 | 0 | 0 | 0.03639 | 0 | 0 | 0 | 0.035003 |
| LOC100360 | 0 | 0 | 0 | 0.154657 | 0 | 0.294324 | 0 | 0.297529 |
| Msh3 | 1.638996 | 1.761724 | 1.554284 | 1.359243 | 1.270093 | 1.437073 | 1.375227 | 1.440618 |
| Rrp36 | 13.59225 | 23.14942 | 23.27063 | 21.35795 | 20.34388 | 20.32281 | 15.28644 | 15.78855 |
| Igsf7 | 0.135839 | 0 | 0.583327 | 0.938777 | 0.293761 | 0.595519 | 0.911824 | 0.541804 |
| Trim66 | 0.012144 | 0.013741 | 0.026075 | 0.01119 | 0.013131 | 0.031944 | 0.027173 | 0.032292 |
| Pik3c3 | 4.220335 | 4.163897 | 4.385814 | 4.479982 | 4.745923 | 6.053844 | 5.420309 | 4.997566 |
| Dtx1 | 1.028418 | 1.06926 | 0.835514 | 0.896422 | 0.901632 | 0.828605 | 1.585891 | 1.552078 |
| 5S_rRNA | 0.39079 | 0 | 0 | 0 | 0 | 0 | 0 | 0.692754 |
| Scfd2 | 9.755536 | 8.881093 | 9.293452 | 10.0226 | 10.45152 | 10.99195 | 11.13987 | 10.85324 |
| Ptgds | 3.464412 | 2.864483 | 3.004024 | 4.542916 | 3.890096 | 5.899953 | 4.695717 | 4.546969 |
| Stx12 | 42.15088 | 39.38845 | 39.35271 | 44.58318 | 44.10176 | 40.67635 | 44.43358 | 43.73424 |
| AABR07055 | 0 | 0.481704 | 0 | 0 | 0 | 0 | 0 | 0 |
| Rn50_2_20 | 0 | 0 | 0 | 0.050055 | 0 | 0 | 0 | 0 |
| Spag7 | 47.78818 | 46.47026 | 47.62928 | 43.00994 | 43.53087 | 45.35181 | 47.24107 | 43.64633 |
| Il10 | 0.153566 | 0.086876 | 0.082431 | 0.495267 | 0 | 0.20197 | 0 | 0.340282 |
| AABR07008 | 0.534346 | 0.274813 | 0.469354 | 0.26857 | 0.367679 | 0.212961 | 0.271729 | 0.129168 |
| Syncrip | 13.40756 | 13.00763 | 12.46837 | 13.22152 | 12.74881 | 11.67842 | 15.36162 | 13.73411 |
| P4ha1 | 11.85084 | 11.92813 | 12.00699 | 16.08438 | 16.71178 | 14.37398 | 25.10771 | 24.35613 |
| Clca5 | 0.082466 | 0.14929 | 0.05312 | 0.106385 | 0.017834 | 0.159074 | 0.055356 | 0.131569 |
| Fam65b | 3.861204 | 3.977088 | 4.159544 | 3.717442 | 3.599194 | 4.354463 | 5.079423 | 4.590706 |
| Mir101a | 0.630915 | 0.71385 | 0.677327 | 1.162726 | 0 | 2.212746 | 1.411679 | 1.118422 |

| | | | | | | | | |
|---|---|---|---|---|---|---|---|---|
| Dhrs13 | 4.234441 | 5.16371 | 4.646964 | 3.771808 | 3.917281 | 3.052715 | 5.105762 | 5.337878 |
| RGD156405 | 0 | 0 | 0 | 0 | 0.0147 | 0 | 0.030418 | 0 |
| Cldn10 | 0.042713 | 0.048328 | 0 | 0 | 0.09237 | 0 | 0.047785 | 0.037858 |
| LOC291276 | 0 | 0.086622 | 0 | 0.211637 | 0.248344 | 0.134253 | 0.08565 | 0.135715 |
| Haus7 | 2.136064 | 2.416855 | 2.675401 | 3.519079 | 4.549398 | 3.68905 | 4.091513 | 4.905366 |
| RGD156469 | 1.783069 | 2.973097 | 3.425479 | 3.286056 | 2.739799 | 3.373645 | 2.939732 | 2.162684 |
| Clip2 | 1.828078 | 1.912052 | 2.110891 | 2.654069 | 2.562781 | 2.693178 | 2.818057 | 3.137007 |
| AABR07002 | 4.953533 | 1.60134 | 0.759705 | 1.304139 | 4.591015 | 4.963728 | 7.125162 | 5.017786 |
| Med28 | 10.87928 | 10.35237 | 10.57623 | 13.46653 | 11.84492 | 11.56104 | 10.88121 | 12.30907 |
| #N/A | 0.228672 | 0 | 0 | 0.210712 | 0.49452 | 0 | 0.255828 | 0 |
| Hsf5 | 0 | 0 | 0 | 0.031662 | 0.037154 | 0.030128 | 0 | 0.030456 |
| Ankh | 47.69648 | 48.56246 | 50.65586 | 43.24314 | 42.83576 | 44.28804 | 52.95519 | 49.26818 |
| Pxmp4 | 16.50251 | 17.37815 | 16.98001 | 14.57427 | 15.08285 | 14.90383 | 13.60153 | 12.39747 |
| Ugt8 | 0.073444 | 0.049859 | 0.031539 | 0.094747 | 0.047649 | 0.090155 | 0 | 0.052078 |
| Col25a1 | 0.215055 | 0.202771 | 0.038479 | 0.297247 | 0.232536 | 0.157134 | 0.240594 | 0.254152 |
| Mfsd4 | 0.287049 | 0.255186 | 0.24213 | 0.245611 | 0.133021 | 0.21573 | 0.252322 | 0.272599 |
| AABR07070 | 1.475096 | 1.669002 | 3.16722 | 2.038865 | 4.785001 | 1.293366 | 0.825136 | 3.268628 |
| AABR07027 | 0 | 0.188094 | 0 | 0 | 0 | 0 | 0 | 0 |
| Cd28 | 1.23021 | 1.995091 | 1.981063 | 2.456111 | 3.36986 | 1.905613 | 2.43147 | 2.180792 |
| Mfsd4b | 2.496551 | 2.388815 | 1.935705 | 1.87446 | 1.583031 | 1.878197 | 1.724093 | 1.748399 |
| Eef1e1 | 5.227951 | 5.377437 | 4.893578 | 4.896977 | 5.115648 | 4.735405 | 5.099573 | 5.418857 |
| LOC100362 | 13.23694 | 9.381183 | 11.71212 | 8.980445 | 7.235099 | 7.014713 | 9.275905 | 8.638258 |
| Scltl | 2.541007 | 2.443775 | 2.023217 | 3.024357 | 2.449902 | 2.487886 | 2.155764 | 2.627591 |
| AABR07035 | 0 | 0 | 0 | 0.030991 | 0 | 0 | 0 | 0 |
| AABR07063 | 0 | 0.092577 | 0 | 0 | 0 | 0 | 0 | 0 |
| Paf1 | 16.52244 | 16.16728 | 16.81345 | 17.45643 | 19.00026 | 18.00244 | 18.605 | 17.98384 |
| Cdh20 | 2.072216 | 2.171652 | 2.370536 | 3.192877 | 3.030397 | 3.425343 | 3.325435 | 3.793856 |
| Zfp598 | 11.5285 | 10.25787 | 10.47117 | 10.96193 | 10.11425 | 9.295055 | 12.21781 | 12.59924 |
| Slc12a1 | 0 | 0 | 0 | 0 | 0.012135 | 0 | 0 | 0 |
| LOC100360 | 735.1288 | 638.008 | 590.6307 | 612.9899 | 639.0581 | 559.7679 | 665.3375 | 630.6198 |
| Pcdh1 | 3.869291 | 4.069798 | 4.153931 | 3.575855 | 3.631693 | 3.969638 | 4.011431 | 4.374933 |
| Rps27 | 557.9574 | 650.8791 | 653.7416 | 597.9438 | 626.1589 | 560.7743 | 616.6807 | 618.3174 |
| Zfp438 | 0.975125 | 0.600045 | 0.532612 | 0.677845 | 0.776918 | 0.91499 | 0.976092 | 0.742996 |
| Mrpl14 | 42.5556 | 45.82467 | 43.12431 | 45.50454 | 39.56408 | 44.57773 | 43.67895 | 40.48051 |
| Cemip | 0.090105 | 0.144428 | 0.088672 | 0.200651 | 0.202977 | 0.223845 | 0.19321 | 0.18635 |
| Cers6 | 0.992967 | 0.975667 | 1.627073 | 0.577882 | 1.440992 | 0.847723 | 1.081653 | 1.435978 |
| LOC100910 | 0.167036 | 0 | 0 | 0.076959 | 0.225768 | 0.183072 | 0 | 0.074026 |
| Cdh22 | 0.85881 | 1.117458 | 1.037235 | 2.472998 | 2.437624 | 2.051939 | 0.840698 | 1.046658 |
| Il22ra1 | 0.009033 | 0.030662 | 0 | 0.016648 | 0.019535 | 0.00792 | 0 | 0.016013 |
| #N/A | 0 | 0 | 0 | 0 | 0 | 0 | 0.199268 | 0.157873 |
| Myc | 2.328369 | 2.431791 | 3.028426 | 4.208482 | 4.2364 | 4.259702 | 7.589206 | 7.560467 |
| Efr3b | 2.165207 | 2.117136 | 2.051862 | 1.416312 | 1.661968 | 1.687516 | 1.749466 | 1.800665 |
| Troap | 0.208907 | 0.262631 | 0.373791 | 0.513331 | 0.17569 | 0.447748 | 0.363557 | 0.288033 |
| H3f3c | 8.104876 | 8.396171 | 9.718114 | 18.28285 | 16.78758 | 17.62747 | 13.95434 | 15.02058 |
| AC122625. | 0 | 0 | 0 | 0 | 0 | 0.559933 | 0 | 0 |
| Magt1 | 15.14922 | 15.19374 | 13.73079 | 16.83629 | 17.58904 | 15.78699 | 17.38488 | 17.1696 |
| Mpc1l | 0.368342 | 0.277841 | 0.131813 | 0.226275 | 0.06638 | 0.107654 | 0.137361 | 0.32648 |
| Avl9 | 2.484194 | 3.382847 | 2.902953 | 2.167542 | 2.044306 | 2.679309 | 3.098937 | 3.273568 |
| AABR07028 | 0.287725 | 0.081387 | 0 | 0.033141 | 0.038889 | 0.063069 | 0.12071 | 0.031878 |
| Kyat1 | 17.59081 | 17.51981 | 18.01203 | 13.85958 | 12.42738 | 13.64149 | 14.5118 | 15.03477 |
| AABR07051 | 0 | 0 | 0 | 0 | 0 | 0 | 0 | 0.076214 |
| AABR07027 | 0.170019 | 1.731319 | 0.365053 | 0.313332 | 0.551518 | 0 | 0.95105 | 0.602786 |
| Kdm4c | 3.248432 | 3.028841 | 2.906166 | 3.048733 | 3.447436 | 2.742738 | 3.062151 | 2.825927 |
| Thyn1 | 13.09148 | 15.11844 | 13.53185 | 14.95448 | 14.03864 | 16.55388 | 14.76721 | 14.04902 |

| | | | | | | | | |
|---|---|---|---|---|---|---|---|---|
| Gtf2h3 | 4.498449 | 4.921061 | 4.776009 | 5.244408 | 4.675993 | 5.186354 | 4.281936 | 4.626032 |
| AABR07053 | 0.041071 | 0 | 0 | 0 | 0 | 0 | 0.045949 | 0 |
| AABR07057 | 0.067744 | 0 | 0.218182 | 0.124846 | 0.439502 | 0.059398 | 0.227366 | 0.060045 |
| Ccdc18 | 0.171248 | 0.152968 | 0.116113 | 0.066441 | 0.146185 | 0.071124 | 0.121001 | 0.19173 |
| Tnnt1 | 0.948052 | 1.198873 | 1.137535 | 2.312451 | 2.231132 | 2.347066 | 1.809324 | 2.174908 |
| Fitm1 | 42.15503 | 47.59045 | 49.42974 | 27.36358 | 25.0699 | 24.80532 | 28.0347 | 28.43823 |
| Tram2 | 3.279939 | 3.455159 | 2.914116 | 3.33499 | 3.138901 | 2.776686 | 4.470824 | 4.243804 |
| Prpf39 | 9.679427 | 9.654562 | 8.52149 | 10.6868 | 10.87152 | 10.20752 | 12.95029 | 12.58566 |
| Rbsn | 2.959994 | 3.349093 | 3.010493 | 3.282591 | 2.807542 | 3.050645 | 3.334748 | 3.691436 |
| Lamp2 | 46.67072 | 45.98879 | 42.67045 | 49.44082 | 48.94104 | 49.56767 | 50.90528 | 54.57093 |
| Cdca4 | 2.794364 | 2.6733 | 2.414576 | 1.905004 | 2.628464 | 2.171227 | 2.338303 | 2.476783 |
| Mas1 | 0.183648 | 0.326526 | 0.140827 | 0.169224 | 0.170208 | 0.299042 | 0.322861 | 0.18603 |
| AABR07064 | 0 | 0 | 0 | 0 | 0 | 0 | 0 | 0.313612 |
| Exosc2 | 3.622975 | 3.02712 | 3.500545 | 3.107307 | 3.977739 | 3.518726 | 3.772616 | 3.75466 |
| LOC102554 | 0.051925 | 0.05875 | 0.362338 | 0.143539 | 0.028073 | 0.091055 | 0.116182 | 1.127571 |
| Gabarap | 152.1924 | 153.6741 | 143.8966 | 163.1332 | 175.516 | 173.2127 | 177.2507 | 178.7128 |
| Ddx52 | 2.577859 | 2.611709 | 2.315291 | 2.35987 | 3.02424 | 2.496637 | 3.166739 | 2.88224 |
| Gucy1b3 | 4.188622 | 4.14913 | 3.394436 | 5.301387 | 5.974177 | 5.15877 | 5.032483 | 4.564889 |
| Rn60_20_0 | 1.011526 | 0.88038 | 1.085938 | 0.143397 | 0.925479 | 0.682236 | 1.2187 | 0.689666 |
| AABR07016 | 3.452698 | 4.55766 | 9.26673 | 8.484068 | 4.977803 | 7.568322 | 13.51954 | 12.24119 |
| Mrpl17 | 97.61904 | 96.2568 | 95.60094 | 94.33875 | 88.65814 | 90.20846 | 91.22917 | 99.38167 |
| Ulk4 | 0.083763 | 0.047387 | 0.014988 | 0.218689 | 0.045286 | 0.110165 | 0.078092 | 0.247478 |
| Nucks1 | 28.15418 | 30.64299 | 28.20109 | 28.15424 | 28.91362 | 27.95448 | 32.74413 | 28.56278 |
| Zfp296 | 0.310317 | 0.263331 | 0.166572 | 0.46466 | 0.419426 | 0.204064 | 0.347168 | 0.481336 |
| Rn50_9_01 | 0 | 0 | 0 | 0.077953 | 0 | 0.074175 | 0 | 0 |
| Rnf130 | 36.26132 | 37.91267 | 33.51896 | 32.36024 | 30.16497 | 34.39031 | 41.26499 | 36.5146 |
| Grik2 | 0.299234 | 0.284398 | 0.295547 | 0.352937 | 0.517692 | 0.314842 | 0.307988 | 0.307662 |
| Garem2 | 0.472119 | 0.356121 | 0.549088 | 0.344406 | 0.340331 | 0.27597 | 0.902318 | 0.575387 |
| Slc23a2 | 3.25156 | 2.888617 | 2.830103 | 3.019173 | 2.832474 | 2.763473 | 3.423719 | 3.493804 |
| Atpaf1 | 12.86331 | 12.4082 | 12.70726 | 10.72292 | 10.96367 | 11.11601 | 11.21596 | 10.73146 |
| Atp6v1a | 8.120643 | 7.819371 | 7.833791 | 8.692442 | 9.239948 | 9.17387 | 9.761711 | 9.444881 |
| Yipf4 | 20.80251 | 18.49108 | 16.92723 | 20.83899 | 21.03122 | 18.49193 | 19.53895 | 22.72271 |
| Wnk2 | 20.9497 | 23.83571 | 27.87182 | 15.22368 | 14.41038 | 14.8734 | 22.24333 | 22.29434 |
| LOC100361 | 8.623753 | 12.2261 | 10.70822 | 23.2649 | 15.16674 | 8.472316 | 9.066674 | 9.117137 |
| Pard3b | 4.262342 | 4.707815 | 3.610912 | 4.127968 | 3.683912 | 3.660956 | 4.882055 | 5.422737 |
| Pmvk | 18.22257 | 17.30826 | 18.79087 | 22.1823 | 20.53344 | 18.62648 | 23.28365 | 23.37728 |
| RGD130918 | 17.91418 | 17.1334 | 16.82072 | 18.30982 | 19.5662 | 18.61355 | 19.55524 | 19.52268 |
| Mcart1 | 2.193082 | 1.268255 | 2.589855 | 1.796301 | 1.053932 | 1.367393 | 1.635682 | 2.138221 |
| Gstcd | 2.03637 | 1.883608 | 1.994684 | 1.931221 | 1.526863 | 2.020081 | 1.430111 | 1.554616 |
| Hspa12a | 2.125737 | 2.171205 | 1.98908 | 1.859701 | 1.931837 | 1.617269 | 2.054304 | 2.148074 |
| Cd2 | 2.585971 | 1.609248 | 1.665723 | 1.826868 | 1.631101 | 1.549378 | 1.687624 | 1.604452 |
| Rabac1 | 132.9151 | 146.4687 | 157.2442 | 159.8901 | 163.2175 | 174.9672 | 155.1124 | 153.1626 |
| Tmem115 | 9.544999 | 8.715561 | 9.425338 | 10.27225 | 9.105128 | 9.648544 | 10.49118 | 9.541587 |
| AABR07068 | 0.306233 | 0 | 0 | 0 | 0 | 0 | 0.3426 | 0 |
| #N/A | 1.337816 | 1.513675 | 1.43623 | 0.704425 | 1.859864 | 1.005427 | 0.855251 | 1.693961 |
| Pdpn | 2.318578 | 3.199221 | 3.308736 | 4.325065 | 5.625563 | 5.057535 | 4.333747 | 4.285574 |
| Crispld2 | 47.21451 | 48.39557 | 49.57736 | 51.5049 | 49.78044 | 51.67602 | 68.20508 | 66.65571 |
| Isg20 | 3.20993 | 2.27883 | 3.446065 | 3.073818 | 2.858348 | 2.81447 | 4.013613 | 2.510401 |
| Atic | 16.21151 | 14.3592 | 13.96401 | 16.97795 | 16.73145 | 15.98874 | 16.01409 | 15.88258 |
| Fam102b | 16.04762 | 18.84731 | 17.63114 | 11.8471 | 12.73499 | 11.43748 | 13.91123 | 12.85133 |
| AABR07070 | 0.053819 | 0 | 0 | 0.049592 | 0 | 0.047189 | 0.06021 | 0 |
| AC098929. | 0 | 0 | 0 | 0 | 0.31457 | 0 | 0 | 0 |
| AABR07029 | 0 | 0 | 0 | 0.099903 | 0 | 0 | 0 | 0 |
| AC128848. | 61.24405 | 57.71726 | 62.68002 | 112.5906 | 98.11315 | 94.59967 | 66.16027 | 61.88602 |

| | | | | | | | | |
|---|---|---|---|---|---|---|---|---|
| AABR07012 | 0.588381 | 3.328628 | 0.631665 | 2.16868 | 3.817248 | 0 | 0.658255 | 2.607557 |
| Ndufv3 | 294.2003 | 309.9609 | 318.4781 | 268.8322 | 262.7086 | 252.9781 | 244.4651 | 239.4608 |
| LOC103690 | 0 | 0 | 0.286098 | 0.491126 | 0 | 0 | 0.298141 | 0.354309 |
| Txk | 0.355946 | 0.331665 | 0.382131 | 0.385871 | 0.316959 | 0.146868 | 0.281094 | 0.222701 |
| Klf4 | 34.59338 | 36.5364 | 37.39957 | 31.30539 | 27.61724 | 30.38972 | 48.68024 | 49.04324 |
| LOC102553 | 1.148672 | 0.917413 | 0.870475 | 1.203734 | 0.925443 | 1.18489 | 0.705536 | 0.978198 |
| Clmn | 1.035736 | 0.831661 | 0.860848 | 0.697834 | 0.782744 | 0.664012 | 1.121357 | 1.085835 |
| Slamf6 | 0.267971 | 0.126332 | 0.119868 | 0.246924 | 0.241461 | 0.195797 | 0.299794 | 0.296895 |
| RGD156360 | 2.523659 | 1.031117 | 1.806206 | 1.162726 | 2.273996 | 1.536629 | 1.490105 | 1.615499 |
| Abi3bp | 7.173985 | 7.173186 | 6.380795 | 12.91362 | 13.12398 | 13.38487 | 12.87883 | 15.19424 |
| Mt-nd2 | 20671.08 | 19514.55 | 18680.66 | 21452.49 | 21987.25 | 22357.44 | 16840.92 | 16874.38 |
| RGD156092 | 0 | 0 | 0 | 0 | 0.027288 | 0 | 0 | 0 |
| Adamts16 | 0 | 0 | 0 | 0.079039 | 0.015458 | 0.025069 | 0 | 0.050685 |
| Pik3ca | 7.308778 | 7.852353 | 6.746883 | 6.757402 | 6.671374 | 6.939977 | 7.544231 | 7.538456 |
| Rsph1 | 0.053217 | 0.240852 | 0.342794 | 0.392302 | 0.17263 | 0.186644 | 0 | 0.141508 |
| Atp6v0a4 | 0 | 0.019293 | 0.054918 | 0.094275 | 0 | 0.074755 | 0.019077 | 0 |
| Tmem143 | 37.94271 | 37.71389 | 37.25582 | 33.06818 | 29.60808 | 30.53682 | 32.76002 | 34.98346 |
| Fgf23 | 0.069267 | 0.156745 | 0.297451 | 0.574442 | 0.973668 | 0.425134 | 0.542451 | 1.903241 |
| AABR07052 | 0 | 0 | 0 | 0 | 0 | 0.805517 | 0 | 0 |
| Slc20a2 | 43.0392 | 43.83246 | 46.5332 | 36.32515 | 37.84995 | 38.35477 | 39.34556 | 40.60489 |
| Rps3a | 507.997 | 497.7503 | 464.0043 | 419.9777 | 433.0593 | 415.7765 | 485.2408 | 477.3522 |
| Tspan2 | 7.517947 | 6.85136 | 6.911708 | 6.184362 | 6.636259 | 7.023202 | 7.15678 | 7.572194 |
| AABR07029 | 5.818436 | 6.583286 | 9.369694 | 12.73347 | 14.94205 | 10.20322 | 6.509407 | 12.24828 |
| Smarcc1 | 16.28836 | 16.30813 | 17.06202 | 13.94279 | 14.95151 | 13.11292 | 13.4376 | 14.85265 |
| AABR07047 | 0 | 0 | 0 | 0 | 0 | 0 | 0 | 0 |
| AC128750. | 0 | 0 | 1.050807 | 0 | 0 | 0 | 0.54752 | 0.433781 |
| AABR07062 | 0.327287 | 0.205728 | 0.156162 | 0.100527 | 0.039321 | 0.095655 | 0 | 0.064465 |
| #N/A | 8.158459 | 6.225499 | 7.197011 | 4.953522 | 6.633314 | 5.656132 | 8.561286 | 6.838854 |
| Fgf5 | 0 | 0 | 0 | 0.082625 | 0.193913 | 0 | 0 | 0.158954 |
| Osbp | 20.41404 | 21.8203 | 21.74994 | 17.8034 | 17.48655 | 18.3783 | 19.04965 | 19.27356 |
| Atl2 | 19.8779 | 19.45325 | 18.16682 | 18.19176 | 19.00124 | 21.06693 | 22.51156 | 23.98845 |
| Ccdc8 | 2.380991 | 2.137223 | 2.078998 | 2.779053 | 2.52304 | 2.352091 | 2.30858 | 3.024893 |
| Smim18 | 0 | 0.205728 | 0 | 0.167546 | 0.196606 | 0 | 0 | 0.161162 |
| AABR07014 | 0 | 0.132401 | 0 | 0.107828 | 0 | 0.102602 | 0 | 0.10372 |
| As3mt | 16.29307 | 14.93222 | 14.48309 | 14.38286 | 13.4245 | 14.82864 | 16.36871 | 16.08767 |
| AABR07025 | 2.192454 | 2.289839 | 2.89691 | 4.273627 | 4.103081 | 4.805864 | 3.584891 | 3.064405 |
| Rraga | 28.23235 | 28.12222 | 26.22575 | 30.21486 | 31.05906 | 31.1655 | 27.95336 | 27.17444 |
| Rbpj | 0.071246 | 0 | 0.114731 | 0.131301 | 0.038519 | 0.124937 | 0.119561 | 0.063149 |
| Mapk7 | 0 | 0 | 0 | 0 | 0 | 0 | 0 | 0 |
| Clasp1 | 11.67673 | 12.20345 | 11.11939 | 10.69567 | 10.86151 | 11.21227 | 11.6522 | 11.43695 |
| Dlg2 | 0.827593 | 0.725696 | 0.710779 | 1.487058 | 1.297553 | 1.215437 | 1.342517 | 1.888856 |
| AABR07054 | 2.615918 | 2.152573 | 1.480769 | 1.928372 | 1.748561 | 2.835772 | 1.596312 | 1.897051 |
| Ergic3 | 25.54618 | 27.54809 | 31.56742 | 35.44776 | 32.36151 | 37.04688 | 36.87733 | 37.74915 |
| Tspo | 64.5992 | 66.09965 | 65.12998 | 77.64211 | 74.97927 | 80.39139 | 77.85686 | 71.19843 |
| Uba6 | 2.256671 | 2.147312 | 2.18298 | 2.196998 | 2.603929 | 1.950684 | 2.41761 | 2.155692 |
| Tmprss11g | 0 | 0 | 0 | 0 | 0 | 0.034036 | 0 | 0 |
| Slc27a5 | 0.022591 | 0 | 0.048506 | 0.041633 | 0.024427 | 0.059423 | 0.050548 | 0.040047 |
| Zfp667 | 1.96876 | 1.824143 | 1.913881 | 1.628436 | 1.40802 | 1.440774 | 1.838358 | 1.74501 |
| Pcdhb9 | 0.429364 | 0.280272 | 0.354577 | 0.517378 | 0.58926 | 0.695016 | 0.775956 | 0.922143 |
| Pabpc5 | 0 | 0 | 0.048928 | 0.125987 | 0.04928 | 0.03996 | 0.050988 | 0.121187 |
| Fbxl21 | 1.000958 | 0.933846 | 0.867215 | 1.181247 | 1.234226 | 1.21638 | 1.100182 | 1.260757 |
| Lig4 | 5.184095 | 4.881779 | 5.001867 | 7.981722 | 7.521286 | 7.278424 | 7.745216 | 6.659727 |
| Dhtkd1 | 0.578153 | 0.473217 | 0.475418 | 0.657431 | 0.585246 | 0.59321 | 0.688098 | 0.643283 |
| Cyp2s1 | 0.159167 | 0.292646 | 0.149516 | 0.164999 | 0.365723 | 0.29656 | 0.289362 | 0.423233 |

| | | | | | | | | |
|---|---|---|---|---|---|---|---|---|
| Tbc1d24 | 2.737352 | 2.778981 | 2.978979 | 2.384151 | 2.250304 | 2.202843 | 2.705844 | 2.409633 |
| Dtna | 9.60199 | 9.193695 | 9.285383 | 10.03465 | 9.691836 | 10.07162 | 8.118211 | 8.575688 |
| Fam107b | 5.101472 | 5.054466 | 5.535964 | 5.31064 | 6.559743 | 5.36757 | 6.324306 | 5.939299 |
| Olr11 | 0 | 0 | 0 | 0 | 0.058074 | 0 | 0 | 0 |
| AABR07052 | 191.2959 | 180.6597 | 171.6715 | 188.0523 | 189.4265 | 192.3341 | 156.9786 | 165.5206 |
| Lbr | 10.4001 | 10.78132 | 11.16517 | 9.350178 | 9.725821 | 11.1151 | 12.04398 | 12.4071 |
| Lamb1 | 43.51613 | 43.936 | 43.7695 | 38.24349 | 37.81521 | 38.39899 | 43.8892 | 43.38051 |
| Hist1h2bc | 34.98419 | 30.59251 | 32.16698 | 44.74474 | 45.40541 | 43.2051 | 48.95431 | 48.46869 |
| Rnaseh2a | 38.22195 | 40.15433 | 42.73849 | 35.45823 | 34.26101 | 36.55674 | 37.55533 | 35.87469 |
| Tkfc | 1.790477 | 1.650685 | 2.135768 | 1.751697 | 2.175031 | 2.093189 | 2.398781 | 2.370687 |
| AABR07022 | 0.024356 | 0.055116 | 0 | 0.022443 | 0 | 0.064067 | 0.136243 | 0.043176 |
| Lgals3bp | 33.0682 | 33.6143 | 39.15833 | 50.31674 | 47.81228 | 46.62472 | 66.23426 | 65.20839 |
| Igsf6 | 1.079226 | 0.822911 | 0.730433 | 1.361984 | 1.446005 | 1.748536 | 2.047314 | 1.850758 |
| Elmsan1 | 6.343615 | 6.592909 | 7.04653 | 6.03054 | 5.710876 | 6.34229 | 7.985412 | 8.056604 |
| Pak3 | 0.025433 | 0.028776 | 0 | 0.117176 | 0.055 | 0 | 0 | 0 |
| 5S_rRNA | 0 | 0 | 0.419539 | 0 | 0 | 0 | 0 | 0 |
| Gtdc1 | 3.099541 | 3.506984 | 3.327555 | 3.371492 | 3.326289 | 2.840282 | 2.633312 | 2.974495 |
| 6720489N1 | 13.63176 | 19.93795 | 6.246462 | 11.48884 | 19.59319 | 4.664329 | 5.207526 | 14.44007 |
| Fen1 | 1.518901 | 1.718565 | 1.65652 | 1.732847 | 2.58086 | 1.670002 | 1.726252 | 2.094209 |
| Pglyrp1 | 0.061535 | 0.417741 | 0.198184 | 0.283508 | 0.332682 | 0.323721 | 0.550737 | 0.654494 |
| Masp1 | 44.77207 | 44.92944 | 44.18759 | 41.9846 | 40.22878 | 39.16372 | 48.05359 | 47.55969 |
| AABR07022 | 0 | 0 | 0 | 0 | 0 | 0 | 0 | 0 |
| AABR07017 | 0.120381 | 0 | 0 | 0 | 0 | 0 | 0 | 0 |
| Mtcp1 | 7.507659 | 9.981111 | 10.27644 | 9.166366 | 8.523819 | 11.51976 | 12.17889 | 11.47886 |
| Npc1 | 3.348334 | 3.478516 | 3.670901 | 3.459341 | 3.719247 | 3.256094 | 3.700562 | 3.894107 |
| Card6 | 9.450654 | 8.786642 | 9.241484 | 8.654871 | 8.920559 | 8.804238 | 8.612206 | 8.642644 |
| 43164 | 26.41385 | 24.90502 | 24.6884 | 25.81443 | 22.83542 | 24.5633 | 26.03763 | 27.61405 |
| Slit1 | 0.011024 | 0.037421 | 0.059177 | 0.060951 | 0.047682 | 0.067663 | 0.086335 | 0.146572 |
| Tex15 | 0.005558 | 0.006289 | 0 | 0.025609 | 0.024041 | 0.05361 | 0.012437 | 0.004927 |
| AABR07069 | 0.200636 | 0 | 0 | 0 | 0 | 0 | 0 | 0.177833 |
| Cops4 | 32.04577 | 30.90811 | 29.66974 | 35.06375 | 40.1436 | 38.6029 | 40.3194 | 34.54894 |
| B4galnt4 | 0.051579 | 0.02918 | 0.055374 | 0.095057 | 0.041829 | 0.135674 | 0.043279 | 0.091435 |
| Slc4a7 | 2.627729 | 3.110374 | 2.444897 | 1.961913 | 2.214777 | 2.221288 | 2.487512 | 3.069617 |
| RGD130875 | 0.475371 | 0.311393 | 0.564062 | 0.484145 | 0.622225 | 1.93047 | 0.811732 | 0.665282 |
| Rnf19a | 6.240887 | 6.297125 | 6.015222 | 7.006904 | 6.991602 | 6.513782 | 8.045422 | 7.2831 |
| Cd69 | 1.125112 | 1.127524 | 1.104347 | 0.710912 | 0.868977 | 0.563714 | 1.258725 | 0.683823 |
| Proser3 | 3.18361 | 3.41695 | 3.73723 | 3.385944 | 3.136759 | 2.660953 | 4.077626 | 3.850295 |
| Ngly1 | 12.62679 | 12.7395 | 12.04183 | 12.5407 | 12.38256 | 12.49488 | 13.95897 | 13.3695 |
| Chst14 | 3.231522 | 3.483576 | 3.032175 | 3.704565 | 3.411658 | 3.234986 | 3.985351 | 3.360429 |
| AABR07015 | 47.02246 | 831.9124 | 798.5274 | 594.7937 | 575.4696 | 531.2962 | 10.76045 | 20.83917 |
| Sco1 | 11.41564 | 9.386815 | 9.619077 | 10.73311 | 11.0518 | 10.53298 | 10.50663 | 10.2359 |
| Prrg3 | 0.075238 | 0.766158 | 0.080773 | 0.138658 | 0 | 0.065969 | 0.168347 | 0.066688 |
| LOC689899 | 4.43779 | 3.459015 | 3.176167 | 2.998782 | 2.772477 | 3.37225 | 2.427237 | 3.059337 |
| Tex10 | 3.0938 | 2.967806 | 2.454942 | 3.061527 | 2.66168 | 2.936734 | 2.889354 | 2.801801 |
| RGD156261 | 1.388367 | 1.432265 | 1.385288 | 1.693224 | 1.465898 | 1.639803 | 1.827345 | 1.541842 |
| Zbtb20 | 7.073837 | 8.495787 | 7.69903 | 5.561378 | 5.719833 | 5.62647 | 7.943684 | 8.189409 |
| P2rx7 | 2.397876 | 2.212207 | 2.613877 | 1.325729 | 2.173953 | 2.118632 | 1.258776 | 1.046329 |
| AABR07067 | 0 | 0 | 0 | 0 | 0 | 0.098529 | 0 | 0 |
| Trim72 | 154.9295 | 161.2451 | 168.1372 | 178.8857 | 170.1991 | 183.6387 | 175.558 | 174.6902 |
| Mef2c | 23.6097 | 22.36417 | 20.21851 | 20.00667 | 20.85086 | 20.03823 | 16.08559 | 17.4197 |
| Cdk5 | 5.394977 | 5.600149 | 7.067122 | 6.499123 | 6.823601 | 6.683205 | 7.115434 | 6.558573 |
| Zfp386 | 8.257108 | 7.28586 | 7.18297 | 9.265742 | 9.241931 | 8.460609 | 9.15115 | 10.07819 |
| Ston2 | 0.411035 | 0.372054 | 0.38832 | 0.606005 | 0.373335 | 0.461307 | 0.717363 | 0.466331 |
| Tmem147 | 42.08513 | 38.57005 | 40.8566 | 39.38918 | 39.07018 | 36.21498 | 39.61696 | 47.53358 |

| | | | | | | | | |
|---|---|---|---|---|---|---|---|---|
| Slc37a2 | 2.282254 | 1.958958 | 2.433248 | 2.103007 | 1.787004 | 1.987282 | 2.500458 | 2.232138 |
| Kit | 3.474421 | 3.106508 | 3.5478 | 2.686352 | 3.271043 | 2.836281 | 2.94878 | 2.893717 |
| Ascc3 | 2.52476 | 2.33147 | 2.083832 | 2.935626 | 2.600712 | 2.522029 | 3.666459 | 3.222711 |
| AABR0706 | 0 | 0 | 0 | 0 | 0.169022 | 0 | 0 | 0.138551 |
| Gramd1c | 0.166241 | 0.062698 | 0.17847 | 0.102123 | 0.119836 | 0.242934 | 0.061994 | 0.294695 |
| #N/A | 13.43196 | 13.77574 | 13.59803 | 18.31239 | 17.58907 | 18.74197 | 24.02501 | 24.8936 |
| Musk | 0.348495 | 0.332047 | 0.196911 | 0.456334 | 0.297491 | 0.498546 | 0.677161 | 0.666548 |
| AC097153. | 0 | 0 | 0 | 0 | 0 | 1.481112 | 0 | 0 |
| Zfp51 | 1.083577 | 0.990244 | 1.252772 | 1.056077 | 1.284315 | 0.840456 | 0.746005 | 1.348293 |
| Bbs10 | 1.622992 | 1.283225 | 1.511467 | 1.423449 | 1.353189 | 1.234445 | 1.203195 | 1.334547 |
| G6pd | 5.236592 | 5.974747 | 5.361984 | 9.427608 | 8.56475 | 8.989979 | 8.689238 | 10.16049 |
| Zdhhc23 | 0.171504 | 0.258732 | 0.122747 | 0.237051 | 0.339982 | 0.150375 | 0.159893 | 0.202683 |
| Pde1b | 0.600592 | 0.711901 | 0.491256 | 0.632482 | 0.618487 | 0.576752 | 0.543932 | 0.659081 |
| Gfpt2 | 4.860676 | 4.292874 | 4.899145 | 6.315602 | 6.95729 | 6.760697 | 12.9297 | 13.3742 |
| Cerk | 7.153483 | 7.353038 | 6.755551 | 6.859788 | 7.053233 | 7.462831 | 9.847758 | 9.618197 |
| Cped1 | 6.869863 | 6.791084 | 6.543444 | 7.101369 | 6.925618 | 6.847771 | 9.037918 | 9.064978 |
| AABR0705 | 0 | 0 | 0 | 0 | 0 | 0.382621 | 0.488206 | 0 |
| Shc4 | 0.028018 | 0 | 0.060159 | 0.258176 | 0.090887 | 0.221097 | 0.031345 | 0.248339 |
| Meaf6 | 10.42862 | 10.08503 | 10.52595 | 11.04689 | 9.396928 | 9.808116 | 12.76398 | 11.96902 |
| Zc3h10 | 3.987381 | 4.568642 | 5.228966 | 4.209069 | 4.502513 | 4.691022 | 5.025576 | 6.17369 |
| AABR0701 | 0 | 0 | 0 | 0.335091 | 0 | 0 | 0 | 0 |
| Ankrd26 | 0.794358 | 0.759162 | 1.001826 | 0.753289 | 0.950658 | 0.94669 | 1.07851 | 0.963836 |
| Il10ra | 1.182965 | 1.189751 | 1.128879 | 1.695642 | 1.364398 | 1.22162 | 1.676368 | 1.887337 |
| Nup35 | 4.760538 | 5.386325 | 4.738436 | 5.752029 | 5.045233 | 4.892754 | 4.40884 | 4.722489 |
| Ccdc105 | 0 | 0 | 0 | 0 | 0 | 0 | 0 | 0 |
| AABR0702 | 0 | 0 | 0 | 0 | 0 | 0 | 0 | 0 |
| LOC690352 | 0 | 0 | 0.055224 | 0.0474 | 0.055621 | 0 | 0 | 0.091188 |
| AC125248. | 0 | 0 | 0.012659 | 0 | 0.03825 | 0.020678 | 0.013192 | 0.031354 |
| Arhgef33 | 1.289196 | 1.273438 | 1.120409 | 1.5085 | 0.862946 | 1.148311 | 0.96153 | 0.961301 |
| Slc37a1 | 7.611014 | 7.903986 | 8.362715 | 8.017495 | 6.452378 | 7.597586 | 6.496082 | 7.632821 |
| Cyp2d5 | 0 | 0.149056 | 0.212144 | 0.121392 | 0.249282 | 0.259893 | 0.221074 | 0.145958 |
| Pcdh8 | 0 | 0 | 0 | 0.015032 | 0 | 0.028607 | 0 | 0 |
| Rxfp2 | 0.094609 | 0.080284 | 0.025392 | 0.174356 | 0.05115 | 0.12443 | 0.079383 | 0.125785 |
| AABR0706 | 2.58843 | 2.252836 | 1.282544 | 2.018192 | 2.583537 | 1.222059 | 2.673065 | 1.764811 |
| Scgb1c1 | 0.912696 | 0.903588 | 1.224797 | 1.471773 | 1.233606 | 1.000316 | 0.510542 | 0.505605 |
| Tm7sf3 | 16.73049 | 16.26686 | 17.63169 | 19.32954 | 17.67552 | 17.60766 | 21.83688 | 20.1122 |
| Mtbp | 0.524613 | 0.503639 | 0.69974 | 0.556654 | 0.498498 | 0.669064 | 0.480202 | 0.690441 |
| #N/A | 33.22277 | 31.71581 | 31.951 | 31.71595 | 32.64284 | 31.6587 | 42.11595 | 42.32413 |
| Tmem168 | 5.676726 | 6.026819 | 6.148022 | 5.046531 | 6.165202 | 6.468373 | 6.238959 | 5.519205 |
| Tmem63b | 14.97403 | 15.04689 | 17.2437 | 13.08182 | 11.11161 | 12.6598 | 15.88278 | 16.0124 |
| Dcaf6 | 26.12225 | 27.00478 | 26.33073 | 28.19428 | 28.67699 | 28.78971 | 31.78592 | 31.27099 |
| AABR0703 | 15.46149 | 15.32317 | 13.38816 | 15.65107 | 15.49797 | 13.40822 | 12.24723 | 11.40357 |
| Dph1 | 9.258063 | 9.820372 | 10.28854 | 10.76365 | 10.24523 | 11.60546 | 9.872002 | 10.25735 |
| Tdo2 | 0.059642 | 0 | 0.032015 | 0.027479 | 0.06449 | 0 | 0 | 0 |
| Unc13b | 4.418084 | 4.409515 | 4.09404 | 4.036808 | 3.922341 | 3.939022 | 3.537973 | 4.352907 |
| Ncoa1 | 7.432805 | 7.287903 | 6.840972 | 6.602726 | 5.799309 | 5.980299 | 7.418345 | 8.185402 |
| Bcl2a1 | 1.6953 | 1.918152 | 1.752605 | 3.240019 | 2.647815 | 3.358254 | 2.950307 | 2.838298 |
| Dnajc8 | 47.34307 | 48.28639 | 45.35697 | 55.27774 | 52.77064 | 53.22332 | 48.66114 | 51.34014 |
| Btbd1 | 72.95319 | 74.25921 | 72.49972 | 71.41593 | 69.08173 | 68.94815 | 71.2221 | 71.81581 |
| Sh3kbp1 | 140.3423 | 141.2911 | 135.7583 | 163.9342 | 162.7644 | 161.9128 | 150.8689 | 154.9976 |
| Coro6 | 79.80876 | 83.41234 | 86.34938 | 77.49467 | 75.4787 | 79.4699 | 89.42605 | 89.40157 |
| Rnf1111 | 36.67439 | 35.56007 | 37.11966 | 33.20555 | 32.01046 | 31.6362 | 33.32386 | 33.92141 |
| Mxd4 | 77.6482 | 79.20206 | 78.06759 | 75.53931 | 70.05199 | 73.84557 | 90.65243 | 87.19879 |
| Prkar1a | 221.4414 | 232.7426 | 231.1452 | 236.151 | 230.1622 | 230.3729 | 245.9004 | 247.0586 |

| | | | | | | | | |
|---|---|---|---|---|---|---|---|---|
| Nme8 | 0 | 0 | 0.059177 | 0 | 0.029801 | 0.048331 | 0.092502 | 0.024429 |
| Scarb2 | 27.89108 | 26.6982 | 26.87783 | 23.78929 | 25.09227 | 24.0912 | 29.48343 | 28.89552 |
| Wars | 13.97952 | 13.30198 | 13.40871 | 15.14114 | 14.37245 | 15.63303 | 16.94725 | 15.27515 |
| Cxcr5 | 0.210164 | 0.317055 | 0.376041 | 0.225934 | 0.454495 | 0.552817 | 0.470245 | 0.931395 |
| AABR07005 | 0 | 0 | 0 | 0 | 0 | 0 | 0 | 0 |
| Gabbr2 | 0.009565 | 0.010822 | 0.092413 | 0.02644 | 0.124104 | 0.025159 | 0.042802 | 0.03391 |
| LOC102549 | 0.278139 | 0.429137 | 0.35289 | 0.582486 | 0.136703 | 0.399064 | 0.311169 | 0.448233 |
| AABR07017 | 0 | 0 | 0.167316 | 0 | 0 | 0 | 0 | 0 |
| AABR07062 | 0 | 0 | 0.102029 | 0.437869 | 0.205526 | 0.249988 | 0.212649 | 0.084237 |
| Rcan3 | 3.946696 | 4.0843 | 4.547057 | 3.592375 | 4.007292 | 3.671471 | 5.007697 | 3.668795 |
| Mapk8ip3 | 18.51081 | 18.95867 | 19.47593 | 16.91109 | 16.62011 | 17.81514 | 18.80495 | 20.79239 |
| Mrps11 | 21.31035 | 18.73137 | 18.84443 | 19.20388 | 16.63128 | 19.81735 | 17.93006 | 19.35673 |
| AABR07027 | 0.047304 | 0.107045 | 0.050784 | 0 | 0.05115 | 0.082953 | 0.105844 | 0 |
| Cpt1b | 134.6513 | 137.9619 | 139.4014 | 112.9817 | 110.2224 | 115.4172 | 134.0449 | 136.0544 |
| Cgrrf1 | 14.28881 | 14.82359 | 13.00284 | 11.89004 | 12.32599 | 13.25724 | 13.10738 | 14.98035 |
| Robo3 | 0.027648 | 0.031283 | 0.019788 | 0.033969 | 0.039861 | 0.064646 | 0.061863 | 0.040843 |
| AABR07034 | 0 | 0 | 0 | 0 | 0 | 0 | 0 | 0 |
| Tfeb | 14.36843 | 15.75466 | 17.11817 | 13.4855 | 12.89495 | 13.37712 | 16.79526 | 17.14463 |
| Mlycd | 90.48729 | 90.61869 | 97.22596 | 59.75875 | 57.53178 | 62.22474 | 77.91333 | 80.47442 |
| AABR07020 | 0.125064 | 0.101074 | 0.191805 | 0.16463 | 0.038637 | 0.172316 | 0.139916 | 0.11085 |
| Irak2 | 2.47244 | 2.133307 | 2.597035 | 2.147133 | 2.115651 | 2.6981 | 3.422745 | 3.547305 |
| Patj | 3.248729 | 3.426049 | 3.169306 | 3.51475 | 3.080229 | 3.441167 | 3.341301 | 3.289122 |
| Ap1s1 | 19.28656 | 18.33915 | 20.16157 | 21.8647 | 20.01168 | 22.64978 | 19.83335 | 22.13669 |
| AABR07002 | 0.174553 | 0.296248 | 0.655879 | 0.643375 | 0.283113 | 0.841766 | 0.683488 | 0.850933 |
| AABR07065 | 0 | 0.553734 | 0 | 0 | 0 | 0 | 0 | 0 |
| Tspan15 | 6.851059 | 6.201321 | 6.587566 | 6.20319 | 6.184029 | 6.320424 | 6.198378 | 5.544396 |
| Rn60_16_0 | 0 | 0.130794 | 0 | 0 | 0 | 0 | 0 | 0 |
| Creb3l3 | 0 | 0 | 0.029235 | 0 | 0 | 0.023876 | 0.091396 | 0 |
| Prdx6 | 212.445 | 205.0612 | 215.1674 | 255.3561 | 250.8757 | 259.8448 | 217.2851 | 213.9159 |
| Bckdk | 57.73479 | 59.89519 | 58.39004 | 51.24058 | 50.70505 | 54.03309 | 47.79046 | 47.49499 |
| Zfp161 | 2.955907 | 2.951003 | 2.457795 | 3.217766 | 2.945499 | 2.80772 | 3.079991 | 3.454761 |
| AABR07060 | 0.155851 | 0 | 0 | 0 | 0 | 0 | 0 | 0 |
| Hspa12b | 25.77609 | 26.20319 | 28.19172 | 25.49452 | 24.16148 | 24.8374 | 30.43647 | 31.44335 |
| Rnf215 | 5.568607 | 5.825099 | 5.978256 | 5.59597 | 6.293915 | 6.448664 | 6.935183 | 6.910027 |
| Ifne | 0 | 0 | 0 | 0 | 0 | 0 | 0.10278 | 0 |
| Macc1 | 0 | 0 | 0 | 0 | 0.011806 | 0 | 0.024431 | 0 |
| Tnp2 | 0.094694 | 0 | 0 | 0 | 0 | 0 | 0.10594 | 0 |
| Strap | 26.74755 | 26.37751 | 25.0532 | 26.01247 | 24.85184 | 25.08177 | 27.66059 | 27.62769 |
| Slco1a2 | 0.050031 | 0 | 0.035808 | 0.030734 | 0.036065 | 0.043867 | 0.093288 | 0.029563 |
| Gyg1 | 73.30525 | 72.07106 | 70.57495 | 91.80418 | 92.8868 | 95.0689 | 85.08163 | 90.14648 |
| Mlh3 | 3.601285 | 3.569381 | 3.723395 | 3.213373 | 4.068684 | 3.482536 | 3.571847 | 3.739439 |
| LOC103694 | 0 | 0 | 0 | 0 | 0 | 0 | 0 | 0 |
| AABR07053 | 0 | 0 | 0 | 0.009401 | 0 | 0 | 0 | 0.009042 |
| Ptprg | 12.87307 | 12.39938 | 12.1098 | 9.600906 | 9.154621 | 10.00296 | 15.01987 | 13.99496 |
| LOC259244 | 0.379005 | 0 | 0.271258 | 0.640271 | 1.092835 | 0.775395 | 0.706691 | 0.671863 |
| AABR07024 | 0 | 0 | 0.058318 | 0 | 0 | 0 | 0 | 0 |
| Itgax | 1.759352 | 1.445421 | 1.563955 | 1.641825 | 1.563087 | 2.02405 | 1.767695 | 1.658726 |
| Sdhaf4 | 70.81867 | 63.57574 | 66.92638 | 64.18433 | 61.83538 | 59.76171 | 65.65202 | 56.8762 |
| LOC681766 | 0 | 0 | 0.068559 | 0.117691 | 0.138104 | 0.055993 | 0.214334 | 0.169809 |
| Rps6kb1 | 12.67251 | 12.98763 | 11.36193 | 10.87335 | 10.05354 | 11.05088 | 14.0747 | 11.00844 |
| Eci1 | 292.2655 | 309.0798 | 312.7718 | 226.6564 | 215.5409 | 226.782 | 225.4053 | 214.7377 |
| Fam220a | 7.150262 | 8.413793 | 7.229644 | 6.49285 | 6.381981 | 6.497333 | 6.312251 | 6.683322 |
| AABR07054 | 0.070102 | 0 | 0 | 0 | 0 | 0 | 0.156853 | 0 |
| Sec24a | 1.816777 | 1.571928 | 1.480029 | 1.349118 | 1.513786 | 1.471138 | 2.176002 | 1.970249 |

| | | | | | | | | |
|---|---|---|---|---|---|---|---|---|
| Cyp4f5 | 2.171546 | 2.047502 | 2.209872 | 2.54293 | 2.103471 | 2.300683 | 2.480042 | 2.666579 |
| Cenpn | 0.954276 | 0.877272 | 1.312611 | 1.456387 | 1.257561 | 1.725715 | 2.035117 | 2.061693 |
| Podxl2 | 6.51104 | 7.109949 | 6.339783 | 5.162504 | 4.857256 | 4.956552 | 6.211386 | 6.061848 |
| Scn1b | 182.0864 | 183.5282 | 197.6907 | 174.3208 | 165.9735 | 174.9261 | 182.3319 | 185.0392 |
| Osmr | 5.745932 | 5.147679 | 4.650793 | 7.883517 | 7.310556 | 7.644468 | 8.658931 | 9.045128 |
| AABR07031 | 0.033439 | 0 | 0.035899 | 0.030813 | 0 | 0 | 0 | 0.029639 |
| Stk11ip | 4.301259 | 4.261938 | 4.1805 | 5.030521 | 4.293129 | 3.894084 | 5.096795 | 5.053148 |
| Necab1 | 0.166695 | 0.175135 | 0.115044 | 0.241375 | 0.283241 | 0.219237 | 0.213132 | 0.200517 |
| RT1-CE10 | 10.60071 | 10.14548 | 10.90992 | 9.915028 | 14.22027 | 11.9154 | 18.50277 | 15.57748 |
| AABR07006 | 2.394591 | 2.994563 | 3.450212 | 2.496853 | 2.112272 | 1.768067 | 2.749461 | 3.183667 |
| Usp47 | 16.48183 | 15.66421 | 15.25333 | 15.35496 | 14.70743 | 15.69173 | 17.52339 | 17.51069 |
| Spdl1 | 0.708265 | 0.542863 | 0.539616 | 0.673691 | 0.543497 | 0.540877 | 0.894617 | 0.729024 |
| LOC500124 | 0.263808 | 0.328335 | 0.339858 | 0.072927 | 0.199676 | 0.277569 | 0.413192 | 0.350739 |
| Morn2 | 0.397314 | 0.449542 | 0.51185 | 0.512552 | 0.601453 | 0.557384 | 0.800094 | 0.704317 |
| Slc5a2 | 0.024984 | 0.113072 | 0.053643 | 0.092086 | 0.081044 | 0.021906 | 0.055901 | 0.044289 |
| Phf12 | 6.541312 | 7.695097 | 6.997165 | 6.462765 | 7.034725 | 7.112345 | 8.493786 | 8.435198 |
| Pinx1 | 4.055949 | 2.998828 | 2.802286 | 3.441366 | 3.126396 | 3.380208 | 3.099955 | 4.093305 |
| Rpl26 | 353.7018 | 331.8536 | 320.9811 | 294.8739 | 290.5998 | 281.9919 | 318.3769 | 312.6815 |
| AABR07067 | 0.146274 | 0.110334 | 0.052345 | 0.134785 | 0.052721 | 0 | 0.054548 | 0.086433 |
| Fam135b | 0 | 0.013381 | 0 | 0 | 0 | 0 | 0 | 0 |
| AC140696. | 0 | 0.564282 | 0 | 0 | 0 | 0.437281 | 0 | 0.442043 |
| AABR07065 | 0 | 0 | 0 | 0 | 0.72593 | 0 | 0 | 0.595058 |
| Mirlet7d | 5.343461 | 6.045875 | 3.441928 | 5.908547 | 7.51115 | 5.622182 | 2.989014 | 5.68341 |
| Klrb1c | 0.939154 | 1.282458 | 0.973475 | 0.895234 | 0.735357 | 0.653082 | 0.507227 | 0.48797 |
| Ppp1r26 | 2.044412 | 2.058162 | 1.935578 | 1.527843 | 1.322875 | 1.764313 | 1.494782 | 1.555236 |
| AABR07006 | 0.358671 | 0 | 0 | 0 | 0 | 0 | 0 | 0 |
| Lsm14b | 39.89974 | 37.8227 | 42.0251 | 32.0834 | 29.363 | 30.18033 | 36.24343 | 37.12458 |
| Hydin | 0.038307 | 0.007224 | 0.006854 | 0.005883 | 0 | 0.011196 | 0.007143 | 0.016977 |
| Pdcl2 | 0 | 0 | 0 | 0 | 0 | 0 | 0.081368 | 0.128929 |
| AABR07065 | 0 | 0 | 0.362698 | 0 | 0 | 0 | 0 | 0 |
| Zdhhc14 | 1.418568 | 1.565896 | 1.652929 | 2.040437 | 1.851876 | 2.078059 | 2.651503 | 2.284692 |
| AABR07045 | 0 | 0 | 0.14037 | 0.060241 | 0 | 0.114643 | 0 | 0.057946 |
| Rn60_6_01 | 0 | 0 | 0 | 0 | 0 | 0 | 0 | 0 |
| AABR07067 | 0 | 0 | 0 | 0 | 0 | 0 | 0 | 0 |
| AABR07004 | 0.418927 | 0 | 0 | 0 | 0 | 0 | 0.468677 | 0 |
| Dio2 | 0.359903 | 0.30541 | 0.289784 | 0.497455 | 1.264764 | 0.94669 | 0.805288 | 0.7975 |
| Ppp2cb | 26.00125 | 27.77441 | 25.50212 | 27.94724 | 26.04278 | 27.25901 | 31.86119 | 30.30853 |
| Rnf219 | 1.41492 | 1.334096 | 1.5339 | 1.406052 | 1.36494 | 1.53251 | 1.474316 | 1.229524 |
| Usp1 | 5.369518 | 6.411778 | 6.064952 | 6.688394 | 6.16531 | 6.793626 | 7.631269 | 7.301683 |
| AC131854. | 0 | 0 | 0 | 0.392302 | 0 | 0 | 0 | 0 |
| Scrn2 | 6.979785 | 6.468641 | 7.719172 | 7.530462 | 6.599007 | 7.011723 | 7.887152 | 7.554406 |
| Cactin | 5.65591 | 5.038605 | 5.600878 | 5.43634 | 4.973362 | 5.58612 | 6.782148 | 6.381636 |
| AABR07070 | 0 | 0.092941 | 0 | 0 | 0 | 0 | 0 | 0.036404 |
| Prss27 | 0 | 0.111897 | 0.053086 | 0 | 0.053468 | 0 | 0 | 0 |
| Afap1 | 7.405282 | 7.38126 | 7.287539 | 7.04707 | 7.018236 | 7.101672 | 8.67921 | 8.692955 |
| AABR07008 | 0.466417 | 0.047975 | 0.273125 | 0.078143 | 0.596026 | 0.185889 | 0.142311 | 0.375826 |
| RGD156454 | 4.254731 | 4.18735 | 4.189334 | 4.894909 | 4.165021 | 5.055008 | 4.816336 | 3.748865 |
| Ugt1a2 | 1.989165 | 1.805751 | 1.961676 | 2.877285 | 3.676462 | 2.859515 | 2.924058 | 3.854209 |
| Hars | 15.48966 | 14.6356 | 15.40383 | 15.7505 | 16.74875 | 17.32217 | 16.41708 | 15.92112 |
| Lrrc61 | 1.586846 | 1.346581 | 1.521054 | 1.63629 | 1.674981 | 1.540421 | 1.669621 | 1.573941 |
| LOC103689 | 7.747173 | 0.984368 | 2.579631 | 1.068899 | 0.85113 | 3.923073 | 9.269726 | 6.536222 |
| LOC102557 | 0 | 0.148495 | 0 | 0.24187 | 0 | 0 | 0 | 0.116327 |
| Sgms1 | 31.88322 | 32.4507 | 30.53383 | 31.47135 | 31.34784 | 30.8262 | 40.7499 | 41.49979 |
| LOC102555 | 0.042436 | 0.048014 | 0.045558 | 0.039103 | 0.091771 | 0.037208 | 0.047475 | 0.037613 |

| | | | | | | | | |
|---|---|---|---|---|---|---|---|---|
| Ptgr2 | 84.77515 | 85.95188 | 83.01373 | 84.95658 | 90.40199 | 88.63451 | 79.26876 | 75.35732 |
| Copg2 | 14.64963 | 14.62533 | 14.32885 | 17.58017 | 17.59567 | 16.58755 | 15.11139 | 14.72546 |
| Car14 | 74.23393 | 79.48923 | 75.78208 | 68.82827 | 66.54278 | 66.81476 | 67.67701 | 63.90351 |
| Cfap58l1 | 0 | 0 | 0 | 0 | 0 | 0 | 0 | 0 |
| Ttll2 | 0 | 0 | 0 | 0 | 0.034131 | 0 | 0 | 0 |
| Sox30 | 0.045635 | 0.07745 | 0.12248 | 0.105127 | 0 | 0.040013 | 0.051054 | 0.020224 |
| Nrxn3 | 0.017415 | 0 | 0.037392 | 0.016047 | 0 | 0.015269 | 0 | 0 |
| Fam198b | 13.09477 | 13.16492 | 12.60427 | 20.83741 | 22.77411 | 20.84193 | 25.90198 | 24.21526 |
| Tmem159 | 58.23543 | 52.40847 | 52.91568 | 53.98747 | 53.41097 | 53.9441 | 55.49918 | 59.32659 |
| Xrn2 | 7.929565 | 8.27508 | 7.55416 | 8.441816 | 8.857153 | 7.884169 | 9.060728 | 8.461335 |
| Ndufa5 | 823.948 | 831.0133 | 797.7625 | 742.1792 | 758.2854 | 686.615 | 674.0456 | 683.8066 |
| Ednra | 18.17216 | 18.58393 | 18.75863 | 23.6807 | 21.48051 | 21.4959 | 21.95421 | 20.96754 |
| Rhbdd3 | 6.400594 | 7.306059 | 7.661967 | 6.393736 | 6.155287 | 5.636878 | 7.731021 | 7.907287 |
| Hhip | 0 | 0.042872 | 0.122037 | 0 | 0 | 0 | 0 | 0 |
| Txlng | 3.895933 | 2.981926 | 2.890868 | 2.745255 | 3.407263 | 3.240136 | 3.172802 | 3.224641 |
| Atp4a | 1.463831 | 1.758705 | 1.506712 | 1.82166 | 1.501231 | 1.892153 | 4.372746 | 4.427437 |
| Draxin | 0 | 0 | 0.163901 | 0.234466 | 0 | 0.04462 | 0.056934 | 0.180426 |
| Clca4 | 0 | 0.020438 | 0 | 0.03329 | 0 | 0.015838 | 0 | 0 |
| Tgfb1 | 13.67876 | 15.20001 | 15.18416 | 15.73864 | 14.20855 | 16.54209 | 19.60122 | 18.39229 |
| Tbc1d16 | 2.693679 | 2.59288 | 2.719189 | 2.056084 | 1.956248 | 1.974058 | 2.338889 | 2.369724 |
| RGD156564 | 54.14535 | 52.92195 | 54.17138 | 44.27108 | 43.84122 | 34.10154 | 41.80556 | 44.16139 |
| Fcrl5 | 0.030623 | 0.034649 | 0.065752 | 0.169309 | 0.231788 | 0.080552 | 0.03426 | 0.054286 |
| Dgki | 0.092356 | 0.083597 | 0.09915 | 0.255308 | 0.159781 | 0.048587 | 0.289307 | 0.245579 |
| LOC680875 | 0.045338 | 0.051298 | 0 | 0.062666 | 0.024512 | 0.119258 | 0.050723 | 0.100464 |
| Batf3 | 3.14983 | 3.266894 | 4.086032 | 2.297768 | 2.412488 | 1.726108 | 3.670718 | 2.093888 |
| Cd1d1 | 22.14493 | 22.95719 | 21.23116 | 19.09089 | 18.23646 | 19.94215 | 16.63358 | 17.30111 |
| Qprt | 0.304706 | 0.492515 | 0.56078 | 0.240664 | 0.282407 | 0.381667 | 0.243494 | 0.424405 |
| Exd2 | 4.547181 | 4.53085 | 4.488004 | 3.852141 | 4.329957 | 4.668616 | 4.545645 | 4.92748 |
| Zfp516 | 2.779768 | 2.682651 | 3.028145 | 3.176696 | 2.858383 | 2.592621 | 3.399527 | 3.502527 |
| Paip2b | 7.584586 | 8.307722 | 7.362933 | 7.732398 | 9.597036 | 7.852863 | 10.20041 | 10.65603 |
| RGD156362 | 0 | 0 | 0 | 0.075514 | 0.044306 | 0.035927 | 0 | 0 |
| #N/A | 0 | 0 | 0 | 0 | 0.185648 | 0 | 0 | 0.304357 |
| Mlkl | 2.185363 | 2.229118 | 1.759595 | 3.432487 | 2.93585 | 3.193546 | 3.315414 | 3.785946 |
| AC133265. | 6.472192 | 7.54489 | 5.263873 | 5.963871 | 4.453456 | 7.050539 | 5.266037 | 6.258137 |
| LOC680121 | 23.91377 | 50.22107 | 63.15173 | 21.16245 | 58.18637 | 24.96873 | 45.80763 | 43.23181 |
| Eif3h | 0.543356 | 1.152715 | 1.23957 | 1.314288 | 1.175046 | 5.061908 | 4.787074 | 1.083607 |
| Aqp3 | 0 | 0 | 0 | 0 | 0.03158 | 0 | 0.032674 | 0 |
| Tnk2 | 6.17699 | 6.503193 | 6.690868 | 4.721941 | 4.971879 | 4.687382 | 5.6245 | 5.794142 |
| AABR07024 | 0 | 0 | 0 | 0 | 0 | 0 | 0 | 0 |
| RGD156398 | 1.564652 | 1.393665 | 1.32236 | 1.472442 | 1.259878 | 1.050808 | 1.266293 | 1.77042 |
| Eif1ax | 20.17548 | 19.87151 | 18.92407 | 19.82436 | 19.96698 | 20.84323 | 20.69498 | 20.39837 |
| Yjefn3 | 0.208629 | 0 | 0.149318 | 0.51265 | 0.225588 | 0.060975 | 0.155603 | 0.123279 |
| Coro1b | 25.20403 | 27.14436 | 26.46605 | 32.44826 | 30.56244 | 31.6735 | 30.04816 | 31.60293 |
| Hmha1 | 4.575794 | 3.943881 | 4.334477 | 4.68766 | 4.380205 | 5.003275 | 5.495606 | 5.344051 |
| Phex | 0 | 0 | 0.013322 | 0.114344 | 0.093924 | 0.076161 | 0.111061 | 0.142983 |
| Rpf2 | 7.163658 | 8.152742 | 7.15095 | 7.797707 | 8.697218 | 7.052465 | 7.217631 | 8.206087 |
| Fam134a | 8.975833 | 9.547325 | 11.12374 | 10.29095 | 10.35396 | 9.230045 | 11.31434 | 11.67695 |
| Pxdn | 27.07476 | 29.34168 | 29.30067 | 23.87235 | 23.33719 | 23.15413 | 27.40459 | 26.10177 |
| Il4i1 | 1.271937 | 1.745336 | 1.626986 | 1.945088 | 1.638688 | 1.542347 | 1.786302 | 1.751039 |
| Gata3 | 0.570049 | 0.355853 | 0.548676 | 0.163017 | 0.297566 | 0.241292 | 0.483807 | 0.296189 |
| Mthfr | 4.119636 | 4.583163 | 4.848308 | 4.113747 | 3.969913 | 4.065502 | 4.840274 | 5.179237 |
| AABR07001 | 8.142001 | 9.600177 | 8.648948 | 7.028689 | 8.989171 | 8.26611 | 9.588325 | 10.93894 |
| AABR07063 | 0 | 1.021544 | 0.484639 | 0 | 0 | 0 | 0 | 0 |
| Lmo3 | 0.746203 | 0.521476 | 0.589042 | 0.667374 | 0.569547 | 0.384866 | 0.343749 | 0.758661 |

| | | | | | | | | |
|---|---|---|---|---|---|---|---|---|
| Cd99 | 103.9078 | 114.9391 | 122.1679 | 131.4363 | 124.6667 | 127.1023 | 141.9777 | 132.205 |
| Med131 | 6.715402 | 7.003173 | 7.056833 | 6.508837 | 6.392988 | 6.679377 | 9.079073 | 8.849559 |
| Tatdn1 | 11.81499 | 13.16657 | 11.47309 | 11.76239 | 12.0692 | 10.20322 | 14.21442 | 12.94555 |
| #N/A | 0.357447 | 0 | 0 | 0 | 0 | 0 | 0 | 0.316823 |
| Grip2 | 1.957007 | 1.968232 | 2.361877 | 1.744373 | 1.272416 | 1.334593 | 1.931834 | 1.598546 |
| Fam104a | 23.50583 | 27.1616 | 25.10867 | 29.9549 | 28.12039 | 28.63207 | 29.22651 | 30.06513 |
| Cd53 | 5.948571 | 5.056615 | 3.937588 | 5.310969 | 5.698911 | 5.621079 | 6.275697 | 6.092076 |
| Spetex-2E | 0 | 0 | 0 | 0 | 0 | 0 | 0 | 0 |
| Tbx2 | 5.930022 | 5.843794 | 7.649777 | 8.548957 | 6.877438 | 9.015173 | 7.169265 | 7.756947 |
| Sh3gl3 | 0.318872 | 0.240526 | 0.11411 | 0.424419 | 0.383102 | 0.124261 | 0.317102 | 0.219825 |
| Gpr179 | 0.013318 | 0.060274 | 0.085786 | 0.024544 | 0.0144 | 0.023354 | 0.059598 | 0.035413 |
| Plekhs1 | 0.020698 | 0.023419 | 0 | 0 | 0.02238 | 0 | 0.046312 | 0.018346 |
| Cald1 | 18.50568 | 15.54167 | 15.3462 | 22.56916 | 22.48793 | 22.2414 | 25.09398 | 25.42896 |
| Arhgap42 | 1.508483 | 1.267892 | 1.064212 | 2.131346 | 1.475758 | 1.574571 | 1.832278 | 1.986465 |
| Nipsnap1 | 1.389754 | 1.226504 | 1.730708 | 1.895293 | 1.652993 | 1.925289 | 1.088356 | 1.625986 |
| AABR07021 | 0.219564 | 0.621065 | 0.589289 | 0.910437 | 0.712233 | 0.481284 | 0.368457 | 0.38922 |
| AC103129. | 0 | 0.432479 | 0 | 0 | 0 | 0 | 0 | 0.338792 |
| Slc30a5 | 6.37929 | 6.972599 | 6.399761 | 7.219422 | 6.562988 | 7.113894 | 7.136866 | 6.930613 |
| Rbm7 | 14.26782 | 14.08251 | 13.89159 | 14.8256 | 12.67852 | 13.04238 | 16.30182 | 15.10154 |
| Rbl2 | 9.481895 | 9.106879 | 9.589477 | 9.501698 | 8.470075 | 9.211239 | 9.390423 | 9.48346 |
| Ndufa1 | 3.5354 | 2.9704 | 5.110742 | 3.419006 | 2.800846 | 2.731544 | 2.545457 | 7.353102 |
| Capn12 | 0.964447 | 0.808316 | 0.939526 | 1.036817 | 1.081467 | 0.876948 | 0.959094 | 0.902329 |
| Chid1 | 0.032445 | 0 | 0 | 0.08969 | 0.035082 | 0 | 0 | 0 |
| Ywhah | 125.5779 | 120.3634 | 120.3576 | 137.8614 | 140.743 | 135.074 | 140.2105 | 138.3864 |
| Kcnrg | 0.713786 | 0.881035 | 0.766295 | 0.837105 | 0.701642 | 0.853429 | 0.943743 | 0.460119 |
| Oas1h | 0.092356 | 0.069664 | 0.03305 | 0.085103 | 0.033288 | 0.026993 | 0.206648 | 0.08186 |
| Zbtb3 | 0.465016 | 0.876905 | 0.637897 | 0.809377 | 0.698354 | 0.928709 | 0.924869 | 0.938824 |
| Slc7a11 | 0.005888 | 0.03997 | 0.012642 | 0.059679 | 0.089129 | 0.036137 | 0.046109 | 0.031312 |
| Fhdc1 | 0.305935 | 0.461535 | 0.218961 | 0.270161 | 0.427288 | 0.190006 | 0.484878 | 0.305061 |
| AABR07044 | 0 | 0 | 0 | 0 | 0 | 0 | 0 | 0 |
| Ptchd3 | 0 | 0.035942 | 0 | 0 | 0.017174 | 0.013926 | 0.106614 | 0.014078 |
| Brd4 | 8.883639 | 9.060796 | 9.285963 | 7.796449 | 7.712349 | 7.332553 | 10.69014 | 10.71053 |
| Rn50_X_06 | 0 | 0.08195 | 0 | 0 | 0 | 0 | 0 | 0 |
| Golga2 | 20.39083 | 20.107 | 19.55267 | 18.13492 | 18.15743 | 19.11028 | 21.8589 | 22.33996 |
| Kcnu1 | 0 | 0.016794 | 0 | 0 | 0 | 0 | 0 | 0 |
| AC117065. | 1.126862 | 1.574989 | 1.565569 | 1.954557 | 1.9352 | 1.743588 | 1.408998 | 1.880082 |
| Bax | 15.90602 | 19.10711 | 17.51966 | 20.89066 | 20.49355 | 20.91962 | 22.07036 | 23.06994 |
| Mif4gd | 26.28452 | 25.14315 | 25.68852 | 23.24686 | 19.76736 | 21.33652 | 24.49739 | 24.7016 |
| AABR07035 | 0 | 0.459299 | 0 | 0 | 0 | 0 | 0 | 0 |
| Hnrnpl | 84.35313 | 81.88966 | 89.03494 | 81.12804 | 78.136 | 77.99241 | 91.51274 | 93.6089 |
| Psors1c2 | 0 | 0 | 0 | 0 | 0 | 0 | 0 | 0.055124 |
| Msantd3 | 5.596672 | 5.564106 | 5.942116 | 5.024393 | 5.384145 | 5.231906 | 5.869976 | 6.474324 |
| Zfp688 | 1.707584 | 2.576069 | 1.425823 | 2.097962 | 3.21408 | 3.65985 | 2.051878 | 1.849854 |
| AABR07009 | 0 | 0.196516 | 0.186462 | 0 | 0.093901 | 0 | 0 | 0.076973 |
| Ramp2 | 54.78521 | 53.86325 | 56.80464 | 57.67364 | 51.05308 | 48.241 | 67.49023 | 64.53762 |
| Dph6 | 6.229403 | 4.552012 | 4.946084 | 4.663872 | 4.630837 | 3.698193 | 3.992759 | 3.680953 |
| Rn50_X_06 | 0 | 0.096813 | 0 | 0 | 0 | 0 | 0 | 0 |
| Rnf34 | 5.664069 | 5.048306 | 4.847382 | 7.213351 | 6.788924 | 7.402539 | 7.592094 | 7.909413 |
| AABR07052 | 0 | 0 | 0.109801 | 0 | 0 | 0.089677 | 0 | 0.181307 |
| #N/A | 0.160447 | 0.340384 | 0.236844 | 0.498979 | 0.498781 | 0.298945 | 0.38144 | 0.40886 |
| AABR07030 | 0 | 0 | 0.056106 | 0 | 0.226038 | 0.137469 | 0.058468 | 0.185287 |
| Lrrc59 | 15.88545 | 16.66262 | 12.96108 | 17.06597 | 16.61089 | 17.18926 | 20.09274 | 19.62927 |
| Prdx5 | 209.8487 | 211.4397 | 209.7847 | 185.0886 | 187.909 | 191.5917 | 180.7078 | 181.1076 |
| #N/A | 0 | 0 | 6.435749 | 0 | 0 | 0 | 0 | 0 |

| | | | | | | | | |
|---|---|---|---|---|---|---|---|---|
| Hpx | 0.267514 | 0.22701 | 0.143597 | 0.123252 | 0.361574 | 0.381155 | 0.299283 | 0.296389 |
| Ift80 | 1.69474 | 1.37217 | 1.435499 | 1.676252 | 1.798874 | 1.704071 | 2.348257 | 1.819096 |
| Psmd4 | 51.95565 | 51.23147 | 52.53435 | 51.27755 | 50.08915 | 50.55826 | 51.03208 | 53.38992 |
| Gmppb | 3.594541 | 4.190298 | 3.917438 | 4.039914 | 4.828961 | 4.178385 | 6.397701 | 4.899713 |
| AABR07063 | 0 | 1.039466 | 0 | 2.539639 | 0.993377 | 0.805517 | 1.027801 | 0.81429 |
| Thoc5 | 11.17871 | 10.70645 | 11.18222 | 9.048836 | 8.917337 | 9.279031 | 10.18632 | 11.42934 |
| Mcpt8l2 | 1.002501 | 0.53378 | 0.759705 | 0.59773 | 0.637641 | 0.982405 | 0.659737 | 0.73176 |
| AABR07027 | 0.037755 | 0 | 0.040532 | 0 | 0 | 0 | 0 | 0.033464 |
| AABR07021 | 2.770684 | 1.818241 | 2.379605 | 2.450953 | 1.79754 | 2.234991 | 2.541769 | 1.768172 |
| Kcnk3 | 46.17418 | 50.81094 | 52.98897 | 36.55049 | 34.00774 | 35.04122 | 45.08481 | 46.25861 |
| Tmem163 | 0.522498 | 0.689712 | 0.654424 | 0.401218 | 0.470808 | 0.610836 | 0.357224 | 0.437387 |
| AC118412. | 0.748085 | 0 | 0 | 2.067992 | 0 | 0.655921 | 1.673848 | 0.663065 |
| AY172581. | 74.55858 | 1280.174 | 1204.348 | 970.0996 | 977.5363 | 924.2806 | 68.00224 | 67.29621 |
| Adgrb2 | 0.200192 | 0.154979 | 0.271476 | 0.242722 | 0.273429 | 0.434201 | 0.55402 | 0.513642 |
| Sgcb | 61.10833 | 59.17445 | 56.66962 | 62.42724 | 59.03962 | 60.21667 | 53.36008 | 55.37962 |
| Erlin1 | 4.267597 | 4.033288 | 3.629296 | 4.410482 | 3.944937 | 4.079331 | 4.718228 | 4.272094 |
| AABR07062 | 0 | 0 | 0 | 0 | 0 | 0.054856 | 0 | 0.110907 |
| Pelp1 | 5.898557 | 5.972279 | 6.580203 | 5.9137 | 5.69188 | 5.702956 | 6.421563 | 6.659863 |
| Tpsg1 | 0 | 0 | 0 | 0.043354 | 0 | 0 | 0.052637 | 0 |
| Rhot1 | 24.2439 | 23.40217 | 23.37351 | 24.82665 | 25.65056 | 28.67418 | 26.00662 | 27.67986 |
| AABR07071 | 0 | 0 | 0 | 0 | 0 | 0 | 0 | 0 |
| AABR07072 | 0 | 0 | 0 | 0 | 0.90596 | 0 | 0 | 0 |
| LOC691422 | 0.584278 | 1.322166 | 0.548852 | 1.211376 | 0.947657 | 0.640369 | 2.287825 | 1.100484 |
| LOC681292 | 1.918356 | 2.639833 | 2.50477 | 2.388769 | 1.906104 | 3.045812 | 2.088166 | 2.435613 |
| AABR07005 | 0.750467 | 0.943465 | 0.537116 | 1.459888 | 1.081959 | 1.096684 | 1.492603 | 0.591268 |
| Sdr39u1 | 32.45615 | 32.4356 | 33.50065 | 24.83472 | 23.07416 | 24.16387 | 28.35258 | 28.48236 |
| Tfb2m | 14.38986 | 14.84822 | 11.66792 | 13.49314 | 15.61434 | 14.34967 | 14.87999 | 13.06882 |
| A1cf | 0 | 0 | 0 | 0 | 0.013017 | 0 | 0 | 0 |
| Ckap4 | 9.063333 | 10.53377 | 9.75213 | 11.77922 | 11.11117 | 12.25347 | 18.00306 | 17.99747 |
| Nfkbiz | 3.793755 | 3.972804 | 3.032964 | 5.801533 | 5.476783 | 5.361113 | 6.072938 | 6.367464 |
| Tcp11l1 | 1.234318 | 0.942686 | 0.828199 | 1.137375 | 1.000987 | 1.271645 | 2.036827 | 1.832512 |
| Slc9a7 | 0.449318 | 0.415949 | 0.438519 | 0.35757 | 0.265004 | 0.196981 | 0.479828 | 0.307741 |
| NEWGENE_1 | 0.061571 | 0 | 0 | 0.085103 | 0 | 0.134963 | 0.068883 | 0.136433 |
| Amdhd1 | 0 | 0 | 0 | 0 | 0 | 0 | 0.025165 | 0.059813 |
| Dip2c | 15.89429 | 15.85622 | 15.82786 | 13.25342 | 12.66406 | 13.38145 | 13.07342 | 12.93532 |
| Igh-6 | 3.083423 | 2.601778 | 2.63698 | 6.3567 | 8.532934 | 6.827603 | 3.741935 | 3.566784 |
| Mrgprb5 | 0.049309 | 0.055791 | 0 | 0.045436 | 0 | 0 | 0 | 0 |
| AC183952. | 0.274167 | 0 | 0.588672 | 0 | 0 | 0 | 0 | 0 |
| Actr3b | 22.695 | 22.36329 | 20.76976 | 17.14143 | 16.59452 | 17.20774 | 15.08841 | 15.128 |
| Dnajb12 | 8.871403 | 8.504057 | 8.881525 | 8.612576 | 8.031832 | 8.226024 | 8.644931 | 9.360911 |
| Psma4 | 67.32104 | 66.22624 | 67.08635 | 63.98417 | 67.51911 | 60.27789 | 58.68763 | 62.28029 |
| AC112801. | 0 | 0 | 0 | 0.63491 | 0 | 0 | 0.770851 | 0 |
| Notch4 | 13.71103 | 15.44008 | 17.34538 | 9.395406 | 8.397926 | 8.897441 | 19.81514 | 20.3575 |
| Acin1 | 17.74745 | 18.07236 | 18.37087 | 18.58452 | 18.12394 | 19.01897 | 22.79654 | 22.91595 |
| Fabp2 | 0.262486 | 0 | 0.563591 | 0.120935 | 0.141911 | 0.230148 | 0.587315 | 0.116327 |
| Vgll3 | 1.547761 | 2.140379 | 2.169338 | 2.654319 | 2.928751 | 2.67646 | 4.13652 | 3.162894 |
| Cinp | 9.226377 | 10.56013 | 8.260628 | 10.40561 | 9.283011 | 9.2766 | 9.764111 | 9.093457 |
| Stk10 | 7.227025 | 7.932543 | 7.681344 | 6.593046 | 6.49043 | 6.441922 | 10.31477 | 9.087116 |
| Gabrd | 0 | 0 | 0 | 0 | 0 | 0.025608 | 0 | 0 |
| AC120291. | 1.217812 | 0.918598 | 2.178999 | 1.122166 | 0.877868 | 0 | 1.816579 | 3.238222 |
| Nkiras1 | 4.179985 | 5.176127 | 4.138454 | 4.793216 | 5.122391 | 4.499823 | 4.728341 | 4.857573 |
| Memo1 | 19.42415 | 20.31798 | 19.36356 | 21.91664 | 21.60314 | 22.55753 | 21.42043 | 21.78426 |
| Fam83a | 0.015688 | 0 | 0.016842 | 0.014456 | 0.016963 | 0 | 0.052652 | 0.013905 |
| Ctu2 | 3.84685 | 3.463633 | 3.926255 | 3.569684 | 3.837325 | 3.752969 | 4.091531 | 3.745817 |

| | | | | | | | | |
|---|---|---|---|---|---|---|---|---|
| AABR07007 | 0 | 0 | 0 | 0 | 0 | 0.433156 | 0 | 0 |
| AABR07061 | 0 | 0 | 0 | 0 | 0 | 0 | 0 | 0.129649 |
| Tacc1 | 19.91065 | 19.97241 | 21.35351 | 21.31875 | 21.92513 | 21.67713 | 23.93696 | 27.34191 |
| AABR07028 | 0.108418 | 0 | 0 | 0 | 0 | 0 | 0.121293 | 0 |
| Upk1a | 0.086987 | 0.196842 | 0.093386 | 0.240464 | 0.188115 | 0.38135 | 0.389267 | 0.192751 |
| Cpne7 | 2.37819 | 2.27684 | 2.50404 | 2.760332 | 3.189652 | 3.909749 | 3.811841 | 4.438768 |
| Slc25a23 | 24.65451 | 25.22618 | 27.00327 | 20.02383 | 16.20352 | 17.48013 | 20.1106 | 19.26085 |
| 1700007B1 | 0.811055 | 1.157062 | 0.530003 | 0.454912 | 0.381296 | 0.371026 | 0.591764 | 0.156278 |
| LOC100909 | 3.384192 | 3.560349 | 6.50142 | 3.610779 | 5.200027 | 6.663327 | 7.040788 | 7.104263 |
| Depdc5 | 2.796464 | 2.646948 | 2.744378 | 2.662488 | 2.847878 | 2.5742 | 2.729907 | 3.213313 |
| Hba-a1 | 1529.349 | 1744.559 | 1792.106 | 1261.524 | 1253.537 | 1504.195 | 1092.14 | 1290.424 |
| Cox7c | 1675.047 | 1730.988 | 1738.019 | 1287.87 | 1318.564 | 1251.606 | 1299.121 | 1322.618 |
| Chst3 | 0.855069 | 0.988733 | 1.099548 | 1.316073 | 0.924574 | 1.153423 | 1.072248 | 1.51578 |
| #N/A | 6.770543 | 8.61812 | 8.745047 | 6.628714 | 7.664057 | 5.658149 | 9.823287 | 6.188602 |
| Cdkn2b | 0.03417 | 0.096655 | 0.055026 | 0.362095 | 0.277109 | 0.434427 | 0.267597 | 0.181721 |
| Lpar3 | 0.228555 | 0.441139 | 0.375269 | 0.260158 | 0.334356 | 0.42437 | 0.481312 | 0.50049 |
| AABR07021 | 0 | 0 | 0 | 0 | 0 | 0 | 0.329127 | 0 |
| Trbv5 | 0 | 0 | 0.168823 | 0.289809 | 0.510113 | 0 | 0 | 0 |
| Rab6a | 33.28873 | 31.2055 | 31.44493 | 31.82842 | 31.28042 | 29.80006 | 34.95139 | 34.58672 |
| Galntl1 | 14.59705 | 14.28217 | 13.29455 | 13.28523 | 12.9158 | 13.44564 | 15.90665 | 13.53904 |
| LOC100910 | 29.11274 | 16.53963 | 30.1287 | 2.728092 | 15.13935 | 2.325469 | 2.001125 | 2.514803 |
| Inhbe | 0 | 0 | 0 | 0 | 0 | 0 | 0.021523 | 0 |
| AABR07033 | 0.070102 | 0 | 0 | 0 | 0 | 0 | 0 | 0.062135 |
| LOC100360 | 116.9506 | 106.4298 | 104.9406 | 100.6167 | 104.6468 | 82.47602 | 100.8958 | 110.3634 |
| Armc2 | 10.67573 | 10.90141 | 10.61546 | 7.880179 | 8.015059 | 7.929899 | 7.568956 | 8.17833 |
| AABR07044 | 0.338241 | 0.243539 | 0.231079 | 0.226674 | 0.166243 | 0.161766 | 0.206405 | 0.436073 |
| RGD156437 | 21.3904 | 20.98976 | 23.2619 | 17.95969 | 16.91981 | 17.9877 | 20.77809 | 20.66231 |
| Tex30 | 1.880767 | 2.097599 | 2.047968 | 1.658779 | 2.23701 | 1.813964 | 1.893706 | 2.357638 |
| RGD156043 | 1.063758 | 1.477136 | 1.142013 | 1.381207 | 1.359359 | 1.059891 | 1.027801 | 2.057153 |
| Cldn34c4 | 0 | 0 | 0 | 0.073111 | 0 | 0 | 0 | 0 |
| Slc5a8 | 0 | 0 | 0 | 0 | 0 | 0 | 0 | 0 |
| Nup210 | 1.795575 | 1.732843 | 1.457898 | 1.272198 | 1.280757 | 1.402373 | 1.485507 | 1.725248 |
| Alcam | 2.063232 | 2.14852 | 2.313021 | 2.893842 | 3.079886 | 3.089782 | 3.35003 | 3.333818 |
| Mab21l2 | 0 | 0.023437 | 0.044476 | 0.019087 | 0.022398 | 0.07265 | 0 | 0.01836 |
| Zfp286a | 0.441612 | 0.420769 | 0.424194 | 0.556849 | 0.427245 | 0.407585 | 0.286033 | 0.226613 |
| Id3 | 49.29476 | 53.41885 | 56.88876 | 70.84481 | 67.09149 | 68.46096 | 60.85584 | 64.77732 |
| Ptpn21 | 5.309336 | 5.034557 | 4.837825 | 4.718239 | 4.372079 | 4.796047 | 5.538222 | 5.359064 |
| Slc25a34 | 124.9187 | 131.2989 | 140.7171 | 106.1569 | 99.50002 | 102.1685 | 122.2228 | 123.8311 |
| RT1-Bb | 28.161 | 26.50329 | 28.72379 | 31.2734 | 31.1255 | 30.67051 | 37.15409 | 35.94126 |
| AABR07035 | 0.111374 | 0.080191 | 0.032609 | 0.00933 | 0.021896 | 0.044388 | 0.011327 | 0.026923 |
| Tmem204 | 23.11569 | 24.81307 | 26.08879 | 26.92563 | 25.53941 | 24.97104 | 25.42979 | 25.82087 |
| Rn60_10_( | 0.173973 | 0.196842 | 0 | 0 | 0.188115 | 0.457619 | 0 | 0 |
| RGD156021 | 20.92124 | 21.78764 | 20.06587 | 20.60962 | 21.7387 | 20.96036 | 21.64856 | 20.54824 |
| Olfml2b | 2.331474 | 2.808756 | 2.376937 | 5.285898 | 5.187072 | 5.353258 | 5.592002 | 5.783231 |
| Mthfd1 | 8.421282 | 8.799442 | 8.399833 | 8.034951 | 7.644804 | 8.196555 | 8.085492 | 8.244043 |
| Clvs1 | 0.273758 | 0.265495 | 0.293896 | 0.10811 | 0.338297 | 0.137161 | 0.087505 | 0.242645 |
| Eif2d | 13.86539 | 13.86316 | 14.21512 | 11.48199 | 11.72955 | 10.9255 | 14.84261 | 13.69609 |
| Rn50_15_( | 0.114019 | 0.046912 | 0.089023 | 0.324744 | 0.403486 | 0.327182 | 0.56822 | 0.523679 |
| Osr2 | 0.361857 | 0.233957 | 0.221987 | 0.238169 | 0.363323 | 0.566566 | 0.318081 | 0.229094 |
| Cd244 | 1.897079 | 2.07244 | 1.404576 | 1.89878 | 1.732981 | 1.347896 | 1.86622 | 1.971385 |
| Cdadc1 | 56.64485 | 56.07961 | 53.64788 | 49.75518 | 49.57224 | 51.0955 | 47.07289 | 49.03324 |
| AABR07043 | 0 | 0 | 0 | 0.082063 | 0.096297 | 0 | 0 | 0.157873 |
| Ndn | 1.049286 | 1.150117 | 1.33769 | 0.966876 | 0.886389 | 1.437523 | 1.357315 | 1.395051 |
| Ttc37 | 4.41861 | 4.731187 | 4.315574 | 4.727 | 4.777779 | 4.479001 | 5.0398 | 5.03405 |

| | | | | | | | | |
|---|---|---|---|---|---|---|---|---|
| AC094241. | 0 | 0 | 0 | 0 | 0 | 0 | 0 | 0 |
| Colec12 | 6.910778 | 7.326671 | 6.503937 | 7.85555 | 8.296267 | 7.665667 | 8.360541 | 8.601236 |
| Telo2 | 4.784794 | 5.353392 | 5.09859 | 5.326858 | 5.712258 | 5.552159 | 5.691309 | 5.21848 |
| AABR07051 | 0.211579 | 0 | 0 | 0 | 0 | 0 | 0 | 0 |
| Cbln2 | 0.022302 | 0.025234 | 0.023943 | 0.041101 | 0.04823 | 0 | 0 | 0.059303 |
| Trpc1 | 3.124795 | 2.955958 | 2.845967 | 3.197995 | 2.866437 | 2.694907 | 3.180679 | 2.985331 |
| Pex2 | 13.4222 | 12.66765 | 11.67315 | 12.72479 | 10.32672 | 13.12651 | 12.4533 | 13.81282 |
| Mlx | 13.73621 | 13.29967 | 13.46438 | 14.55849 | 17.02491 | 15.49525 | 15.70154 | 13.85939 |
| Uba52 | 724.2913 | 754.3741 | 752.2525 | 735.7646 | 709.1296 | 661.2961 | 685.1151 | 668.4059 |
| Dag1 | 41.88062 | 44.11716 | 43.55172 | 37.39059 | 37.34421 | 36.94238 | 44.41314 | 44.57907 |
| Rab3il1 | 6.528377 | 8.329516 | 7.00863 | 14.42048 | 11.06417 | 11.93533 | 14.91811 | 15.84085 |
| Gpr108 | 8.979135 | 9.039968 | 8.975786 | 9.185646 | 10.53815 | 10.41046 | 10.01778 | 11.00618 |
| RGD156298 | 34.89176 | 35.25542 | 35.88006 | 37.62285 | 36.62731 | 35.5515 | 35.87204 | 33.13175 |
| Zfp322a | 2.254273 | 1.605936 | 1.649257 | 2.323413 | 2.600013 | 1.815496 | 1.849452 | 1.731664 |
| Itga3 | 4.809376 | 5.702084 | 5.533932 | 3.618396 | 3.969385 | 3.353305 | 4.364515 | 4.387498 |
| Cfb | 1.774812 | 1.82556 | 2.07859 | 2.461389 | 2.248617 | 2.310657 | 3.369471 | 3.479897 |
| Ankdd1a | 0.146171 | 0.165386 | 0.274618 | 0.538765 | 0.197566 | 0.320408 | 0.531473 | 0.356287 |
| Gnb4 | 4.676399 | 4.02346 | 5.70026 | 6.329016 | 7.374095 | 5.851427 | 7.520636 | 7.512675 |
| AABR07036 | 0 | 0.497896 | 0 | 0 | 0 | 0 | 0 | 0.390038 |
| Rnf217 | 6.59391 | 7.266914 | 8.136234 | 7.25967 | 5.046487 | 6.382226 | 7.999705 | 6.148121 |
| Pak6 | 5.972086 | 5.192505 | 6.313531 | 2.912551 | 2.908353 | 2.904631 | 3.400155 | 3.528904 |
| Ankrd44 | 1.947069 | 1.913147 | 1.888608 | 1.558076 | 1.514366 | 1.257931 | 1.929893 | 2.058831 |
| AC126148. | 8.830332 | 10.22346 | 9.259462 | 15.13824 | 16.20958 | 12.60398 | 13.55488 | 12.01317 |
| Galnt12 | 0.240763 | 0.170257 | 0.355402 | 0.16639 | 0.162708 | 0.343039 | 0.471371 | 0.560175 |
| Adprhl2 | 14.8674 | 15.25694 | 18.01951 | 15.40856 | 14.7504 | 16.06731 | 17.58243 | 16.79949 |
| AABR07056 | 3.786601 | 3.552885 | 2.379605 | 2.297768 | 1.997267 | 2.996183 | 2.066479 | 3.274393 |
| Rnf113a | 0.04626 | 0 | 0.049663 | 0.042626 | 0.05002 | 0 | 0.051753 | 0.041002 |
| Mrgprb3 | 0.143665 | 0.243825 | 0.123387 | 0.132382 | 0.139809 | 0.151159 | 0.401815 | 0.191006 |
| AY172581. | 4.363827 | 2.962479 | 0.936969 | 1.608438 | 2.831126 | 6.121931 | 1.952822 | 2.320726 |
| Kcnj16 | 0 | 0 | 0 | 0 | 0 | 0 | 0 | 0 |
| AABR07051 | 0 | 0 | 0 | 0 | 0 | 0 | 0 | 0 |
| AABR07048 | 0.793423 | 1.346581 | 0 | 0.365554 | 0.857917 | 0.695674 | 0 | 0.70325 |
| AABR07032 | 0.515075 | 0.777044 | 0.368644 | 0.316414 | 1.299533 | 0.150539 | 0.192081 | 1.521787 |
| AABR07006 | 0 | 0 | 1.124363 | 0.482531 | 1.13245 | 0 | 0 | 0 |
| Rsph3 | 2.728888 | 3.510123 | 3.02215 | 3.335104 | 2.857527 | 2.997161 | 3.117231 | 3.335327 |
| Zswim3 | 1.517853 | 1.761414 | 1.128124 | 1.524161 | 1.704357 | 1.672099 | 2.046436 | 1.759302 |
| Rn50_X_06 | 0 | 0 | 0 | 0.158989 | 0.093283 | 0 | 0.096515 | 0 |
| Lrrc46 | 0.958167 | 1.084121 | 0.805033 | 0.575813 | 0.945961 | 0.730541 | 0.745708 | 0.775422 |
| Bag2 | 12.26589 | 10.10369 | 11.10616 | 13.19664 | 11.98765 | 12.29114 | 11.68677 | 13.5301 |
| Agl | 30.86454 | 31.273 | 28.4174 | 29.12916 | 28.77893 | 28.34235 | 28.92458 | 29.34413 |
| Dcn | 420.9523 | 416.0198 | 384.1373 | 457.8087 | 457.876 | 464.9396 | 527.8283 | 537.5808 |
| AABR07010 | 0.047177 | 0.053378 | 0 | 0 | 0 | 0 | 0 | 0 |
| Esco2 | 0.269927 | 0.229058 | 0.217338 | 0.352364 | 0.194579 | 0.197227 | 0.377479 | 0.338938 |
| RGD156337 | 0.098618 | 0.111581 | 0.105872 | 0.181744 | 0 | 0 | 0 | 0 |
| Armt1 | 12.64524 | 11.53359 | 11.82082 | 12.95998 | 13.20804 | 12.06787 | 11.54852 | 11.68464 |
| Ablim2 | 9.434397 | 9.027966 | 9.778243 | 5.780203 | 5.995963 | 6.072064 | 5.894959 | 6.716428 |
| Tbc1d22b | 6.888355 | 5.875991 | 6.891758 | 5.283918 | 4.016611 | 4.30053 | 5.850397 | 7.160366 |
| Fign | 2.122524 | 1.910711 | 2.511521 | 1.798786 | 1.557957 | 1.168238 | 1.178626 | 1.112301 |
| Gns | 4.01481 | 5.342422 | 4.496307 | 5.997843 | 4.211353 | 0.502884 | 5.670447 | 0.803919 |
| AABR07067 | 0 | 0 | 0 | 0.110546 | 0 | 0.105188 | 0 | 0.106333 |
| Rnf181 | 15.68619 | 17.54802 | 16.96674 | 21.27376 | 21.87108 | 21.89728 | 21.57341 | 20.98584 |
| AC113837. | 11.63687 | 10.24067 | 6.246462 | 13.10579 | 18.17513 | 18.13906 | 18.80495 | 14.32547 |
| Mnd1 | 0.425739 | 1.059749 | 0.365647 | 0.549223 | 1.01276 | 0.821235 | 0.666817 | 0.754708 |
| Ptprz1 | 0.126681 | 0.113158 | 0.114526 | 0.153594 | 0.108141 | 0.14615 | 0.179021 | 0.130013 |

| | | | | | | | | |
|---|---|---|---|---|---|---|---|---|
| C1qb | 21.66199 | 24.23598 | 25.02046 | 35.77772 | 36.85953 | 36.29067 | 52.0392 | 52.88598 |
| Actn1 | 8.623541 | 8.515941 | 8.587296 | 12.22825 | 12.02631 | 12.42376 | 13.24419 | 13.47736 |
| AABR07062 | 1.702957 | 0 | 0.457058 | 0 | 1.841383 | 1.119865 | 0.952596 | 0 |
| Zfand2a | 7.085631 | 6.931082 | 7.303815 | 7.673679 | 6.837435 | 8.613607 | 9.474609 | 9.257882 |
| Nabp2 | 27.68773 | 30.45175 | 32.49391 | 30.54447 | 32.07679 | 32.3437 | 34.19844 | 34.63941 |
| Ube2g2 | 28.53096 | 29.00696 | 28.28495 | 20.17675 | 20.78324 | 22.33484 | 27.00148 | 27.24856 |
| Sdhb | 644.368 | 638.4444 | 648.7461 | 621.8746 | 607.7098 | 581.6147 | 563.8475 | 566.4466 |
| Il17rd | 0.373761 | 0.289348 | 0.3379 | 0.561926 | 0.340331 | 0.55194 | 0.946334 | 0.64513 |
| Dpy19l2 | 0 | 0 | 0.016017 | 0.027495 | 0 | 0.039243 | 0 | 0.026447 |
| Olfm2 | 0.196437 | 0.093583 | 0.099894 | 0.333437 | 0.290659 | 0.253821 | 0.231331 | 0.201603 |
| RGD131042 | 7.728304 | 7.387352 | 7.915365 | 6.957619 | 6.579546 | 6.776184 | 7.50321 | 7.519231 |
| Nts | 0 | 0.043153 | 0 | 0.035144 | 0.08248 | 0 | 0.042669 | 0.033805 |
| LOC100909 | 0.123375 | 0.077552 | 0.029434 | 0.063159 | 0.118581 | 0.060097 | 0.030673 | 0.048602 |
| Kif3a | 2.158702 | 2.639443 | 2.504399 | 3.144154 | 2.955364 | 2.732278 | 3.349921 | 3.070641 |
| Rnf214 | 5.971742 | 7.122633 | 7.105383 | 5.681514 | 5.874382 | 6.597017 | 7.259771 | 7.261226 |
| AABR07026 | 0.137263 | 0.077653 | 0 | 0.189724 | 0.148421 | 0.120353 | 0.076782 | 0.121663 |
| Gosr2 | 18.55784 | 18.82617 | 18.16112 | 19.79914 | 20.96726 | 19.12735 | 22.17404 | 21.3274 |
| Parp14 | 4.219297 | 3.124908 | 3.512657 | 4.364673 | 6.019983 | 4.932644 | 5.127993 | 6.420266 |
| AABR07030 | 0 | 0 | 0 | 0 | 0.262141 | 0 | 0 | 0 |
| Nemp2 | 0.969151 | 0.772977 | 1.091615 | 0.936954 | 1.133825 | 0.933335 | 1.333086 | 1.098402 |
| Bace1 | 10.13913 | 9.422439 | 9.54648 | 10.92524 | 11.82815 | 11.90146 | 12.34331 | 11.86427 |
| Nell1 | 0.09063 | 0.061526 | 0.019459 | 0.066809 | 0.097997 | 0.174822 | 0.202785 | 0.128528 |
| AC135409. | 4.928557 | 4.647026 | 5.291121 | 3.595332 | 3.552785 | 4.50142 | 2.756925 | 3.640354 |
| Eif3g | 59.42064 | 57.85673 | 63.28355 | 58.20044 | 58.05599 | 57.91203 | 63.88165 | 61.56428 |
| Ctbp1 | 113.4001 | 119.003 | 125.0567 | 107.831 | 103.166 | 107.3681 | 112.8851 | 111.2475 |
| AABR07027 | 0 | 0 | 0.021977 | 0.094318 | 0 | 0.017949 | 0.09161 | 0.018145 |
| Gpc1 | 129.8879 | 149.8117 | 151.6426 | 140.6151 | 131.7734 | 139.334 | 159.8877 | 165.6731 |
| Zfp518a | 1.158367 | 1.26695 | 0.969993 | 1.323563 | 1.44458 | 1.388065 | 1.356407 | 1.341579 |
| AC123095. | 14.08159 | 19.91582 | 16.06233 | 11.35368 | 9.992208 | 11.18924 | 15.75386 | 12.48121 |
| Gpr132 | 0.261612 | 0.320668 | 0.280857 | 0.220976 | 0.400742 | 0.439647 | 0.292679 | 0.405789 |
| Pcsk2 | 0 | 0 | 0.012067 | 0 | 0 | 0.01971 | 0 | 0 |
| Tnks1bp1 | 10.16964 | 10.02574 | 10.13722 | 10.37585 | 10.3084 | 10.2953 | 11.52985 | 12.17153 |
| Slc52a2 | 1.572922 | 1.559726 | 1.802472 | 1.416815 | 1.700777 | 1.456619 | 1.641082 | 1.56647 |
| Stab2 | 0.055086 | 0.031163 | 0.029569 | 0.069794 | 0.052118 | 0.036224 | 0.092441 | 0.036619 |
| Rhcg | 0 | 0 | 0 | 0 | 0.029247 | 0 | 0 | 0 |
| LOC102553 | 0 | 0 | 0 | 0.08632 | 0 | 0 | 0 | 0 |
| Yrdc | 7.480846 | 7.335662 | 6.993807 | 8.559187 | 7.313741 | 7.652414 | 8.717956 | 8.536956 |
| AABR07026 | 0 | 0 | 0 | 0 | 0 | 0 | 0 | 0 |
| Notum | 0.025657 | 0.02903 | 0.055089 | 0.047284 | 0.027743 | 0.022496 | 0.229631 | 0.045482 |
| Cd37 | 6.036072 | 5.698814 | 6.008048 | 6.519698 | 6.051261 | 5.783122 | 9.167829 | 6.696446 |
| Rbbp9 | 4.623741 | 4.302869 | 3.906487 | 4.311017 | 3.993751 | 4.24601 | 4.774926 | 4.146752 |
| Abcd1 | 8.633767 | 9.326443 | 10.05879 | 7.616686 | 7.76928 | 7.781774 | 9.234641 | 9.46632 |
| Bcs1l | 10.76534 | 10.99477 | 11.14817 | 10.59287 | 11.33137 | 10.52497 | 9.734505 | 10.35812 |
| Insc | 0.117208 | 0.132615 | 0.111849 | 0.240006 | 0.154899 | 0.216955 | 0.233115 | 0.173145 |
| Pdzd9 | 5.088743 | 5.096319 | 4.835574 | 5.132638 | 5.056245 | 5.2155 | 4.346728 | 4.2666 |
| AC130862. | 1.036949 | 1.759888 | 0 | 0 | 0 | 0.454599 | 0.580046 | 0.919099 |
| AC141136. | 0.4568 | 0.422876 | 0.356658 | 0.612252 | 0.449029 | 0.254878 | 0.371671 | 0.368077 |
| Tmem185b | 15.61529 | 16.65515 | 16.8174 | 14.29723 | 13.01296 | 15.13041 | 17.58096 | 16.74978 |
| App | 120.7115 | 125.0349 | 127.0994 | 133.0858 | 131.6327 | 135.4872 | 166.9361 | 168.4161 |
| AABR07004 | 0 | 0 | 0 | 0.095645 | 0 | 0 | 0 | 0 |
| Klhl29 | 0.259237 | 0.26538 | 0.318066 | 0.944133 | 0.814232 | 1.017436 | 0.842448 | 1.126991 |
| Vat1 | 23.74617 | 24.62114 | 27.77535 | 34.54045 | 32.24729 | 33.83358 | 40.58968 | 41.13489 |
| Hoga1 | 0.502666 | 0.669109 | 0.476156 | 0.953619 | 0.607469 | 0.959252 | 0.52928 | 0.524162 |
| Tbc1d23 | 6.756004 | 6.770489 | 5.639579 | 6.89875 | 7.484071 | 6.927067 | 6.648234 | 7.21026 |

| | | | | | | | | |
|---|---|---|---|---|---|---|---|---|
| Zfp318 | 2.856134 | 2.796787 | 3.289242 | 2.880641 | 2.63909 | 2.9869 | 3.172077 | 3.461297 |
| Cdh11 | 5.486629 | 5.887161 | 4.923029 | 5.736648 | 5.691805 | 5.724888 | 5.639892 | 5.284777 |
| Spast | 4.39609 | 4.507659 | 4.652445 | 4.545669 | 4.402323 | 3.953048 | 4.652682 | 4.527436 |
| Taf5l | 6.84122 | 7.394397 | 7.434053 | 6.739551 | 7.667945 | 7.437025 | 6.813618 | 8.134249 |
| Rn60_7_12 | 0.588381 | 0 | 0.42111 | 0.361447 | 0 | 0.171964 | 0 | 0.173837 |
| Psmg3 | 8.716648 | 8.741741 | 8.365376 | 11.43958 | 12.3527 | 12.68004 | 9.086903 | 10.00868 |
| Dhx8 | 4.347604 | 4.199598 | 4.597768 | 4.67583 | 4.069524 | 4.722308 | 5.255923 | 5.4064 |
| Fam81a | 2.388393 | 2.500137 | 2.354779 | 1.092919 | 1.036528 | 1.353359 | 1.290571 | 1.036874 |
| Aup1 | 21.3806 | 23.89969 | 24.09914 | 27.83965 | 25.54579 | 26.07091 | 26.55454 | 26.90933 |
| Tnn | 0.016364 | 0.037031 | 0 | 0.67856 | 1.061672 | 0.846548 | 1.629386 | 1.958112 |
| Nek4 | 3.92318 | 3.76341 | 4.089703 | 4.060389 | 3.043229 | 4.038082 | 4.102835 | 3.70409 |
| AABR07044 | 1.00456 | 1.020037 | 1.253594 | 0.8149 | 0.919106 | 0.805517 | 1.181491 | 1.377443 |
| AABR07035 | 0 | 0.041579 | 0.039451 | 0.033862 | 0 | 0.161103 | 0 | 0 |
| Dhodh | 2.112746 | 2.143837 | 2.646196 | 1.993165 | 2.121305 | 2.043584 | 2.101019 | 2.184737 |
| RGD135933 | 0 | 0.04917 | 0 | 0 | 0 | 0 | 0.048618 | 0.077037 |
| AABR07051 | 0.110477 | 0 | 0 | 0.3054 | 0.35837 | 0.193732 | 0.247193 | 0.391684 |
| AC113837. | 6.756893 | 14.33457 | 18.13489 | 15.56553 | 20.09186 | 23.6978 | 21.73302 | 15.72105 |
| Jrk | 3.072602 | 2.955029 | 3.157265 | 2.730165 | 2.729082 | 2.886493 | 3.216509 | 3.306986 |
| Ctnna3 | 2.424041 | 2.254264 | 2.923202 | 2.662031 | 2.297933 | 2.416552 | 2.600461 | 2.972648 |
| RGD156260 | 1.801285 | 1.572225 | 1.381282 | 1.304139 | 1.558163 | 1.669618 | 1.95762 | 2.075539 |
| Adh5 | 21.61617 | 20.12714 | 20.1713 | 22.18282 | 23.23216 | 24.55891 | 19.65798 | 20.50874 |
| Rad50 | 3.794632 | 3.766785 | 2.900531 | 4.559572 | 4.507918 | 3.948202 | 4.732056 | 4.34996 |
| LOC685505 | 0 | 0 | 0.133853 | 0 | 0 | 0 | 0 | 0 |
| Ctnnbl1 | 7.032154 | 7.390473 | 6.594593 | 6.966482 | 7.543657 | 8.749098 | 7.400823 | 7.760389 |
| Trappc3 | 21.48742 | 23.21884 | 22.40429 | 22.71993 | 24.028 | 23.75355 | 22.43944 | 24.62881 |
| Slc22a15 | 1.749143 | 2.381892 | 2.276644 | 1.640293 | 1.539838 | 1.384356 | 2.181989 | 1.83847 |
| Bcl6b | 9.890993 | 9.52668 | 10.78663 | 8.681526 | 7.987396 | 8.205876 | 11.87101 | 10.29276 |
| Armc3 | 0.055238 | 0 | 0.019767 | 0.1527 | 0.079638 | 0.048433 | 0.144196 | 0.081601 |
| Allc | 0.075948 | 0.085931 | 0.040767 | 0.034991 | 0 | 0 | 0.042483 | 0 |
| Med12 | 7.810863 | 6.995114 | 6.982802 | 7.336989 | 7.616552 | 8.068863 | 8.717263 | 9.012691 |
| Ift57 | 2.712834 | 2.48811 | 2.824146 | 3.351964 | 3.022238 | 2.793071 | 2.759089 | 2.641329 |
| Pcdhb12 | 0.225491 | 0.274758 | 0.335186 | 0.543427 | 0.318842 | 0.365004 | 0.523944 | 0.338231 |
| Cx3cl1 | 9.076536 | 9.398078 | 9.456589 | 10.24627 | 10.32134 | 9.881819 | 9.910869 | 10.3902 |
| AABR07036 | 0.885977 | 1.213482 | 0.600728 | 1.131492 | 1.058836 | 1.131167 | 0.817299 | 0.978163 |
| Ebpl | 2.810336 | 3.444743 | 3.721059 | 5.438189 | 5.520442 | 5.872783 | 5.082927 | 3.777926 |
| Myoc | 2.737251 | 3.675963 | 3.405497 | 1.838664 | 1.549028 | 1.525249 | 2.947836 | 3.197092 |
| Zfp955a | 8.237134 | 8.446821 | 7.571534 | 6.912204 | 5.976605 | 6.467051 | 5.822328 | 6.903324 |
| Zfp830 | 4.461991 | 5.008144 | 4.675266 | 5.328567 | 4.901881 | 5.226802 | 6.189927 | 6.169619 |
| Mir6320 | 0 | 0 | 0 | 0 | 0 | 0 | 0 | 0 |
| AC105648. | 2.293398 | 0.432479 | 0.820703 | 2.113276 | 0.826606 | 0.670284 | 1.710501 | 1.355168 |
| Gins4 | 2.134346 | 1.965626 | 2.238069 | 2.012453 | 2.52252 | 2.437167 | 2.832055 | 2.683683 |
| AABR07041 | 1.946938 | 0 | 0.39641 | 1.299123 | 0 | 1.324456 | 0.375543 | 0.029753 |
| Zfp394 | 2.532119 | 2.67237 | 2.101614 | 2.411676 | 2.43884 | 2.985095 | 3.475563 | 3.451384 |
| Il5ra | 0 | 0.011855 | 0 | 0 | 0 | 0.009187 | 0 | 0 |
| AABR07053 | 0.295138 | 0.305312 | 0.262532 | 0.497295 | 0.419426 | 0.421437 | 0.443394 | 0.433501 |
| Adamts20 | 0.108819 | 0.095762 | 0.116824 | 0.100272 | 0.078443 | 0.137818 | 0.121741 | 0.139319 |
| St3gal6 | 22.82298 | 20.89505 | 21.08181 | 19.3563 | 20.15697 | 18.19994 | 17.51414 | 17.16278 |
| Slc16a11 | 2.514369 | 2.920754 | 3.131229 | 2.594919 | 2.573751 | 1.499129 | 2.962989 | 1.782888 |
| Hist2h2be | 3.985858 | 3.576746 | 3.688856 | 3.926108 | 4.755697 | 5.422971 | 2.767779 | 2.923749 |
| Rbm5 | 21.57153 | 20.715 | 19.36014 | 24.10283 | 25.55349 | 21.88053 | 24.8442 | 25.11773 |
| AABR07058 | 2.570107 | 0.726989 | 2.414277 | 0.592063 | 1.042132 | 1.126736 | 0.71883 | 0.854255 |
| #N/A | 0 | 0.745278 | 0.707147 | 1.820873 | 1.068349 | 0.866311 | 1.105371 | 0.291915 |
| Orc6 | 0.831205 | 0.901283 | 1.264165 | 1.340365 | 1.086014 | 1.305769 | 1.278634 | 1.289292 |
| Srsf3 | 47.64556 | 45.24968 | 43.17175 | 50.54826 | 54.27682 | 52.81839 | 51.93294 | 54.62207 |

| | | | | | | | | |
|---|---|---|---|---|---|---|---|---|
| Npff | 2.487974 | 3.485269 | 4.833236 | 4.03929 | 3.586946 | 4.466794 | 3.446157 | 3.465337 |
| AABR07017 | 0.189732 | 0 | 0 | 0 | 0 | 0 | 0 | 0 |
| Dnmt3a | 5.876134 | 5.87548 | 6.176369 | 4.117634 | 4.211225 | 4.44527 | 5.702526 | 5.389995 |
| B3galt4 | 1.400872 | 1.05668 | 1.217463 | 1.198645 | 1.298351 | 1.023571 | 0.932877 | 1.093845 |
| Cited1 | 3.214542 | 3.519777 | 3.395354 | 6.449676 | 5.157694 | 5.318807 | 4.930393 | 5.560551 |
| Sdsl | 0 | 0.12899 | 0.040797 | 0.10505 | 0.082181 | 0.099959 | 0.170057 | 0.370508 |
| RGD130469 | 16.24306 | 17.8616 | 19.01368 | 17.53703 | 17.75736 | 18.56026 | 23.46306 | 22.49174 |
| LOC102546 | 0.770087 | 0.647903 | 0.593555 | 1.01892 | 1.067544 | 0.571334 | 0.817358 | 0.787577 |
| Smad9 | 0.05995 | 0.169575 | 0.128719 | 0.193344 | 0.388935 | 0.42051 | 0.368879 | 0.106273 |
| Copz1 | 20.11836 | 17.44571 | 18.33096 | 21.28087 | 23.6895 | 23.21232 | 21.90666 | 21.40225 |
| Doc2g | 54.48208 | 57.38281 | 59.49114 | 54.92925 | 51.69713 | 53.65076 | 62.19605 | 57.57308 |
| AABR07033 | 7.205516 | 6.529596 | 7.053722 | 5.418639 | 5.494112 | 5.712906 | 7.179133 | 7.881344 |
| Fam212a | 1.046409 | 0.617719 | 0.732643 | 0.628842 | 1.033078 | 0.797819 | 0.356292 | 0.645206 |
| Casp3 | 2.788592 | 2.673456 | 2.422409 | 3.256106 | 3.314489 | 3.07963 | 2.738714 | 3.471655 |
| AABR07007 | 0.032667 | 0.295693 | 0.280565 | 0.120407 | 0.141291 | 0.229143 | 0.219281 | 0.086864 |
| Cldn14 | 0 | 0 | 0 | 0.034077 | 0 | 0.032425 | 0 | 0.032779 |
| Lbx1 | 0 | 0.050902 | 0.048297 | 0 | 0 | 0 | 0 | 0 |
| AABR07065 | 12.83836 | 10.35607 | 13.34801 | 9.702873 | 9.064857 | 8.735471 | 12.00691 | 12.169 |
| Dctn6 | 83.32927 | 82.6419 | 78.77837 | 66.63315 | 67.7476 | 63.10582 | 62.65682 | 65.04055 |
| AABR07047 | 0.032265 | 0.073012 | 0.311746 | 0.297308 | 0.069775 | 0.254609 | 0.216579 | 0.257382 |
| Fam83g | 0.166358 | 0.062742 | 0.158752 | 0.119228 | 0.159894 | 0.113449 | 0.165435 | 0.163835 |
| Zfp488 | 0.111338 | 0 | 0 | 0.034198 | 0.120388 | 0.097621 | 0.08304 | 0 |
| Coa3 | 58.16021 | 51.79216 | 59.32843 | 46.18419 | 42.44731 | 47.62913 | 55.26244 | 54.05398 |
| Gtpbp3 | 5.393649 | 4.793277 | 5.435458 | 5.1795 | 4.670128 | 4.221813 | 4.924441 | 4.689075 |
| Ercc1 | 10.09109 | 10.14282 | 10.57049 | 9.16313 | 8.686709 | 8.504273 | 11.23466 | 10.46389 |
| AABR07029 | 0 | 0 | 0 | 0 | 0 | 0 | 0 | 0 |
| Traf3ip2 | 1.989548 | 1.93362 | 1.670389 | 2.749935 | 2.123689 | 2.012812 | 2.739468 | 2.147774 |
| Rpl36al | 167.401 | 167.0526 | 135.699 | 155.8195 | 141.1543 | 132.0624 | 150.3237 | 152.4241 |
| Meox2 | 8.328965 | 7.824487 | 8.614826 | 7.354195 | 7.077814 | 7.722328 | 8.953138 | 8.307582 |
| Snx11 | 6.321695 | 5.727642 | 6.370698 | 7.365187 | 6.233191 | 7.050698 | 8.129232 | 7.986217 |
| Rn50_10_( | 0 | 0 | 0.129834 | 0 | 0 | 0 | 0 | 0.107193 |
| Hbp1 | 21.653 | 19.87641 | 19.09256 | 24.24479 | 23.37039 | 22.13518 | 21.50828 | 21.86888 |
| LOC103690 | 0.325507 | 0.046037 | 1.485173 | 0.299942 | 0.30797 | 0.356756 | 0.136561 | 0.685218 |
| LOC500148 | 0.242435 | 0.091435 | 0.086756 | 0.074465 | 0.08738 | 0.070856 | 0.135613 | 0.214882 |
| Cd160 | 0.107749 | 0.121913 | 0.115675 | 0.397145 | 0 | 0.283423 | 0.120545 | 0.286509 |
| Dll1 | 2.478017 | 3.25494 | 3.027249 | 2.362133 | 2.155875 | 2.110293 | 2.979421 | 3.168356 |
| Tmem156 | 0.131079 | 0.296619 | 0.281443 | 0.120784 | 0.283467 | 0.22986 | 0.109984 | 0.145227 |
| Tshz2 | 8.556187 | 8.69149 | 7.841826 | 8.089589 | 7.749905 | 7.953098 | 10.55061 | 10.12183 |
| Tp53inp1 | 6.63576 | 5.238175 | 6.95824 | 7.915789 | 8.78817 | 9.561759 | 8.287025 | 9.665892 |
| Tex36 | 0.128663 | 0.145576 | 0 | 0 | 0.208682 | 0.225624 | 0.287885 | 0.171061 |
| AABR07011 | 0 | 0 | 0 | 0 | 0 | 0 | 0 | 0 |
| Ankra2 | 0.572618 | 0.680285 | 0.21516 | 0.158294 | 0.185749 | 0.150622 | 0.224217 | 0.101508 |
| Oxct1 | 551.8623 | 543.6518 | 527.1107 | 598.6373 | 603.5202 | 590.0928 | 555.2792 | 560.2069 |
| Ubxn10 | 0.762611 | 1.006668 | 0.92105 | 0.731995 | 0.755883 | 0.724379 | 0.604332 | 0.704104 |
| Fra10ac1 | 4.978542 | 4.954668 | 4.16949 | 4.347346 | 4.960459 | 4.525171 | 4.228361 | 4.574452 |
| Pgap3 | 2.971885 | 3.338004 | 4.145333 | 2.858409 | 2.885074 | 2.662812 | 2.985051 | 3.172492 |
| Cdk14 | 9.314323 | 10.17665 | 10.08541 | 10.64686 | 10.34331 | 10.97258 | 11.64789 | 11.27441 |
| AC097845. | 0 | 0 | 0 | 0.450964 | 0 | 0 | 0 | 0 |
| Rn50_14_( | 0.11715 | 0 | 0 | 0 | 0 | 0 | 0 | 0 |
| Ythdf1 | 10.95279 | 11.31496 | 10.11525 | 9.951523 | 9.4708 | 10.27447 | 11.11187 | 10.718 |
| Dnajb14 | 3.156209 | 2.764724 | 3.643432 | 2.720689 | 3.669638 | 3.511283 | 3.568995 | 3.820249 |
| Cntnap1 | 4.111535 | 4.431011 | 4.476903 | 4.490547 | 4.350704 | 4.615425 | 5.648689 | 6.007399 |
| Itga2b | 1.31384 | 0.873052 | 1.208993 | 1.422036 | 1.262788 | 1.206832 | 1.516529 | 1.146037 |
| Tprkb | 0.095646 | 0.054109 | 0.102682 | 0 | 0 | 0 | 0 | 0 |

| | | | | | | | | |
|---|---|---|---|---|---|---|---|---|
| #N/A | 0 | 0.20291 | 0 | 0.16525 | 0 | 0 | 0.200632 | 0 |
| Camkv | 0 | 0 | 0.015551 | 0.040044 | 0.015663 | 0.012701 | 0 | 0 |
| AABR07021 | 0 | 0 | 0 | 0.092086 | 0 | 0 | 0 | 0 |
| Opn1sw | 0 | 0 | 0 | 0 | 0 | 0.056337 | 0 | 0.028475 |
| Rpl9 | 488.7179 | 475.3527 | 438.9569 | 424.2538 | 422.4757 | 389.2223 | 470.8433 | 460.2228 |
| AABR07067 | 0 | 0 | 0 | 0 | 0 | 0 | 0 | 0 |
| Commd2 | 3.45149 | 4.029568 | 3.374978 | 3.686847 | 4.397634 | 3.797294 | 3.935158 | 3.70225 |
| Micalcl | 0.206535 | 0.191196 | 0.1411 | 0.207615 | 0.203021 | 0.049388 | 0.252067 | 0.266272 |
| Cnbp | 299.1108 | 284.2681 | 268.149 | 287.9613 | 287.561 | 285.9729 | 291.7459 | 286.2888 |
| Abo2 | 0 | 0 | 0.111877 | 0 | 0 | 0 | 0 | 0 |
| AABR07050 | 0.072329 | 0 | 0.077649 | 0 | 0 | 0 | 0.080918 | 0 |
| Rn50_X_02 | 0.200636 | 0.113505 | 0.323093 | 0.323536 | 0 | 0.307856 | 0.056116 | 0.222292 |
| Ido2 | 0.259881 | 0.147021 | 0.0465 | 0.159646 | 0.140503 | 0.379772 | 0.484571 | 0.422299 |
| Kif13b | 3.583228 | 3.447712 | 3.493442 | 3.579121 | 3.437214 | 3.743746 | 5.008316 | 4.82651 |
| #N/A | 0.354508 | 0.343808 | 0.271848 | 0.419998 | 0.273803 | 0.488452 | 0.566583 | 0.493771 |
| AABR07064 | 0 | 0.055949 | 0.053086 | 0 | 0 | 0.043356 | 0 | 0 |
| Mmp19 | 1.619295 | 1.713953 | 1.458027 | 2.430707 | 2.400456 | 2.518999 | 3.27256 | 2.893673 |
| LOC689728 | 6.826846 | 9.087358 | 8.88109 | 8.732929 | 9.9871 | 10.56315 | 7.188303 | 9.467992 |
| Sirt3 | 20.14365 | 21.50635 | 19.43043 | 22.81819 | 21.69916 | 22.87424 | 20.92612 | 20.27069 |
| AABR07038 | 0 | 0 | 0 | 0 | 0 | 0 | 0 | 0.30943 |
| Rpl26-ps1 | 0.358671 | 0.405819 | 1.28352 | 0.110167 | 0.646376 | 0.838621 | 0.53502 | 0.105969 |
| Prr30 | 0.026354 | 0.089456 | 0.028293 | 0 | 0 | 0 | 0 | 0.070077 |
| Tbx21 | 0.77643 | 1.02491 | 1.342938 | 0.775071 | 0.583016 | 0.642954 | 0.844507 | 0.84112 |
| Mms22l | 0.175215 | 0.118949 | 0.188104 | 0.365962 | 0.151566 | 0.194596 | 0.405114 | 0.217422 |
| Csf2ra | 10.13401 | 9.828129 | 12.56694 | 16.04626 | 15.29613 | 15.05096 | 20.73138 | 20.0543 |
| Phf2 | 8.613499 | 9.299577 | 8.924048 | 7.774435 | 7.697789 | 8.562333 | 11.27343 | 10.55962 |
| Cebpg | 7.739184 | 7.40403 | 6.663259 | 7.512633 | 7.838001 | 7.471014 | 9.429782 | 8.924302 |
| Manea | 1.979348 | 2.226443 | 1.963411 | 2.431856 | 2.415593 | 2.415484 | 2.434553 | 2.359712 |
| Slc35a1 | 6.982123 | 6.120677 | 6.111404 | 6.230885 | 6.835511 | 6.094355 | 8.163149 | 8.195717 |
| St8sia4 | 5.072949 | 5.110276 | 5.867771 | 4.37294 | 4.529801 | 4.447966 | 3.551695 | 4.177307 |
| Rbm45 | 5.844281 | 6.217751 | 6.742434 | 7.046405 | 7.545476 | 7.495202 | 6.766025 | 7.551057 |
| Ccr9 | 0.081567 | 0.069217 | 0.043784 | 0 | 0.022049 | 0.017879 | 0.022813 | 0.036148 |
| Lrrc20 | 15.25695 | 16.30586 | 16.15743 | 13.71241 | 13.67311 | 13.80565 | 14.56734 | 14.12254 |
| AABR07026 | 0 | 0.342483 | 0.32496 | 0 | 0 | 0 | 0.11288 | 0 |
| AC115420. | 9.467285 | 12.21813 | 11.4342 | 13.63083 | 12.15625 | 12.32168 | 18.36977 | 17.43822 |
| Tcof1 | 3.588591 | 3.650045 | 4.376103 | 3.07631 | 3.325964 | 3.157443 | 3.958801 | 4.011952 |
| Tmem189 | 15.28909 | 15.87803 | 15.84085 | 19.96083 | 19.82467 | 21.93876 | 22.37316 | 21.73246 |
| Rgs7bp | 1.420781 | 1.148248 | 1.416349 | 1.683249 | 1.316803 | 1.957594 | 1.930115 | 2.428667 |
| Htr6 | 0 | 0 | 0 | 0 | 0 | 0 | 0 | 0.048323 |
| Fam199x | 2.525833 | 2.755794 | 3.050598 | 2.327455 | 2.633605 | 2.491484 | 2.573487 | 2.718508 |
| Rab43 | 5.672975 | 5.739803 | 4.743407 | 6.433751 | 5.603269 | 6.026276 | 7.445135 | 9.379601 |
| Svep1 | 0.40437 | 0.447127 | 0.404518 | 0.956933 | 0.83473 | 0.862206 | 1.233794 | 1.164843 |
| Il7 | 0.096261 | 0.108915 | 0.051671 | 0.221752 | 0.156128 | 0.084402 | 0.269231 | 0.383944 |
| AC130624. | 3.141955 | 0.592496 | 0 | 0.965063 | 0 | 1.377435 | 0 | 2.784871 |
| Fam124b | 0.029958 | 0.033896 | 0.160807 | 0.05521 | 0.032393 | 0 | 0 | 0.849694 |
| AABR07063 | 0 | 0.387252 | 0 | 0 | 0 | 0.300095 | 0 | 0 |
| Ccdc157 | 1.774514 | 1.686535 | 1.847903 | 1.340819 | 1.458255 | 1.524778 | 1.826428 | 1.887408 |
| Rnf166 | 18.58916 | 19.78362 | 18.13076 | 18.47641 | 18.19664 | 17.97336 | 17.79238 | 16.95254 |
| Mtrf1 | 10.60268 | 10.5007 | 9.557956 | 9.496495 | 9.510015 | 10.00609 | 9.628289 | 9.421597 |
| Mir3541 | 0 | 0 | 0 | 0 | 1.088894 | 0 | 0 | 0.446293 |
| Hcar1 | 0.332599 | 0.12544 | 0.277719 | 0.647007 | 0.159838 | 0.356429 | 0.578818 | 0.163777 |
| Mitf | 14.19443 | 13.27231 | 12.51405 | 12.50856 | 13.13055 | 12.35512 | 11.43961 | 11.92731 |
| Cnnm4 | 4.860684 | 4.715809 | 5.096333 | 4.301028 | 4.064639 | 4.142361 | 5.882607 | 5.908766 |
| Fgfr2 | 0.456577 | 0.450366 | 0.339345 | 0.571745 | 0.455714 | 0.708272 | 0.379825 | 0.394311 |

| | | | | | | | | |
|---|---|---|---|---|---|---|---|---|
| Bpifb5 | 0 | 0.040061 | 0 | 0.065251 | 0.038284 | 0.062089 | 0.039611 | 0 |
| Dennd5a | 26.85241 | 26.81139 | 28.40569 | 27.22072 | 26.81756 | 28.33319 | 31.99094 | 30.76092 |
| Baiap2l1 | 6.413424 | 6.869006 | 5.715402 | 4.676136 | 5.04957 | 5.268428 | 5.259384 | 4.746312 |
| Maf | 7.188535 | 7.250077 | 9.307068 | 8.062863 | 8.122201 | 9.324536 | 11.89766 | 12.38516 |
| AABR07005 | 0.011601 | 0 | 0.012454 | 0.01069 | 0 | 0.010172 | 0 | 0.071976 |
| Tpbpa | 0 | 0 | 0.079969 | 0 | 0 | 0 | 0 | 0 |
| Safb | 25.46896 | 25.05376 | 26.58574 | 23.23546 | 21.85156 | 23.58328 | 27.80598 | 28.29411 |
| Mrs2 | 9.645647 | 8.694396 | 10.00908 | 8.4672 | 8.744677 | 8.834239 | 8.957532 | 9.001892 |
| Ubash3a | 0.24666 | 0.139542 | 0.264805 | 0.18183 | 0.186697 | 0.129763 | 0.082786 | 0.109314 |
| LOC502908 | 0 | 0 | 0 | 0.070135 | 0.0823 | 0.066736 | 0.170304 | 0.134926 |
| Rn60_20_( | 0.926085 | 0.698548 | 0.933957 | 1.060224 | 1.062051 | 1.254898 | 1.569793 | 1.343185 |
| Pcsk5 | 0.889251 | 0.734826 | 0.675776 | 1.067995 | 1.080376 | 1.016234 | 1.050746 | 1.31955 |
| Mrm1 | 4.27215 | 3.734217 | 3.936846 | 3.362176 | 3.489343 | 3.311759 | 4.000003 | 3.884128 |
| Ptch1 | 2.828891 | 2.885762 | 2.853812 | 2.457757 | 2.349965 | 2.52762 | 2.67253 | 3.120304 |
| LOC100911 | 10.84671 | 10.40322 | 10.17942 | 10.45816 | 10.56332 | 9.006545 | 13.09918 | 10.31434 |
| AABR07007 | 0 | 0 | 0 | 0 | 0.107037 | 0 | 0 | 0 |
| Srxn1 | 8.155348 | 5.585001 | 6.451264 | 9.55834 | 8.431494 | 10.1614 | 11.44482 | 10.33547 |
| Slc39a4 | 0.062452 | 0 | 0 | 0.057547 | 0.168821 | 0 | 0 | 0.138386 |
| Atp6ap1l | 2.914358 | 3.454481 | 3.128749 | 3.537995 | 2.901153 | 2.839235 | 3.777986 | 2.829153 |
| Magel2 | 0 | 0 | 0 | 0 | 0 | 0 | 0.046533 | 0 |
| Slc43a2 | 2.401465 | 2.664384 | 2.978611 | 3.437445 | 3.176508 | 3.168631 | 4.199524 | 4.308739 |
| AC110474. | 0 | 0 | 0 | 0.81785 | 0 | 0 | 0 | 0 |
| Arhgap29 | 11.41977 | 10.95577 | 10.48846 | 10.2728 | 10.42305 | 9.632143 | 12.12013 | 11.71909 |
| Mfsd2a | 0.024562 | 0.027791 | 0.026369 | 0 | 0 | 0.021536 | 0 | 0.108852 |
| Nudt18 | 21.49799 | 24.23846 | 22.75497 | 20.4014 | 18.75161 | 21.13524 | 21.09217 | 19.7245 |
| 5S_rRNA | 0 | 0.519733 | 0 | 0 | 0 | 0 | 0 | 0 |
| Cog6 | 9.906591 | 10.23329 | 9.369694 | 9.517671 | 9.77956 | 9.226096 | 9.803482 | 9.17061 |
| AABR0700( | 0 | 0.697054 | 0.66139 | 0 | 1.332294 | 0 | 0 | 0 |
| Oat | 54.16996 | 54.6707 | 51.79504 | 57.7555 | 60.91929 | 58.99627 | 55.91181 | 58.21262 |
| Cyb5a | 52.78485 | 58.69658 | 53.06994 | 58.35412 | 57.15099 | 55.21982 | 55.7726 | 50.80843 |
| AABR07022 | 0 | 0 | 0 | 0 | 0 | 0 | 0 | 0 |
| AABR07024 | 0.409109 | 0 | 0 | 0.376978 | 0 | 0 | 0 | 0 |
| Nrp1 | 53.87939 | 53.69374 | 53.02873 | 44.48255 | 45.30043 | 43.34894 | 42.76894 | 43.18508 |
| Chp1 | 28.69251 | 29.90873 | 31.18491 | 25.97653 | 27.40674 | 27.9631 | 30.93008 | 29.08322 |
| Carns1 | 8.020998 | 8.909242 | 8.532234 | 7.120424 | 6.509703 | 7.177659 | 7.084371 | 6.962991 |
| Slc39a8 | 1.663692 | 1.553428 | 1.543312 | 1.473492 | 1.362294 | 1.798624 | 1.915476 | 1.717996 |
| Igkv4-80 | 0 | 0 | 0 | 0 | 0 | 0.166357 | 0 | 0 |
| Vldlr | 20.59696 | 20.30473 | 20.1214 | 21.35169 | 21.17752 | 21.51276 | 22.25429 | 21.99154 |
| Slc35g1 | 0.474329 | 0.751353 | 0.356456 | 1.048981 | 0.871905 | 0.831784 | 0.742921 | 1.135138 |
| Map3k11 | 12.98329 | 13.99769 | 16.77413 | 12.1286 | 11.26318 | 13.00627 | 14.79225 | 16.01823 |
| Ap3m1 | 8.440232 | 8.724209 | 7.458964 | 8.556604 | 9.3056 | 8.796157 | 8.477895 | 8.406936 |
| Lin7b | 1.342716 | 1.139415 | 0.450466 | 0.773287 | 0.453706 | 0.515066 | 1.126628 | 1.041351 |
| AC118496. | 0 | 0 | 0 | 0 | 0 | 0 | 0 | 0 |
| Rnf4 | 28.26097 | 27.43036 | 26.62183 | 32.23258 | 34.37795 | 30.12379 | 27.12253 | 30.63604 |
| AABR07061 | 0.055472 | 0.062764 | 0.178659 | 0.255578 | 0.059981 | 0.048638 | 0.18618 | 0.147504 |
| AABR07021 | 0.121217 | 0 | 0 | 0.055849 | 0 | 0.053142 | 0 | 0.161162 |
| Sepsecs | 2.453284 | 2.941 | 2.915945 | 2.946859 | 3.252715 | 2.765624 | 2.907995 | 2.433332 |
| RGD130477 | 0.04628 | 0 | 0.049685 | 0 | 0.075063 | 0.060868 | 0 | 0.02051 |
| Med8 | 7.03061 | 7.314762 | 8.762399 | 9.382554 | 8.912803 | 8.856961 | 9.22166 | 9.884573 |
| LOC103694 | 0.319548 | 0.090388 | 0.300173 | 0.736127 | 0.04319 | 0.245157 | 0.134061 | 0.283231 |
| AABR0703( | 0 | 0 | 0 | 0.06574 | 0.038571 | 0 | 0 | 0 |
| Enpp7 | 0.039671 | 0 | 0 | 0 | 0 | 0 | 0 | 0 |
| Olr154 | 0 | 0 | 0 | 0 | 0 | 0 | 0 | 0 |
| Tmem129 | 5.075604 | 5.388625 | 5.713033 | 5.727742 | 5.584885 | 5.234487 | 6.35376 | 7.134597 |

| | | | | | | | | |
|---|---|---|---|---|---|---|---|---|
| Lin28b | 0 | 0 | 0 | 0 | 0 | 0 | 0 | 0 |
| Camk4 | 0.198657 | 0.134862 | 0.426541 | 0.366109 | 0.386648 | 0.139346 | 0.355597 | 0.42259 |
| Slc22a12 | 0.071005 | 0.080338 | 0 | 0.130856 | 0 | 0.062257 | 0.119155 | 0.094402 |
| Trappc3l | 0.059984 | 0.271476 | 0.064397 | 0.110546 | 0.259439 | 0.052594 | 0.067107 | 0.106333 |
| Lhx4 | 0.044872 | 0 | 0.096346 | 0.041348 | 0.04852 | 0 | 0 | 0.039773 |
| Klf6 | 15.62948 | 17.2622 | 15.97877 | 15.00974 | 13.92306 | 14.83546 | 19.571 | 19.92825 |
| AC121465. | 0 | 0 | 0 | 0 | 0 | 0 | 0 | 0 |
| Il23a | 0.475745 | 0.422937 | 0.072963 | 0.21919 | 0.36744 | 0.178772 | 0.41819 | 0.421676 |
| Rnf125 | 2.511737 | 2.152964 | 2.369661 | 1.893655 | 1.440253 | 1.835245 | 2.469412 | 1.517917 |
| AABR07056 | 0 | 0 | 0.019978 | 0 | 0 | 0.016316 | 0 | 0 |
| Madd | 1.55822 | 1.300251 | 1.3801 | 1.364046 | 1.569045 | 1.588264 | 1.754162 | 1.579665 |
| Ccdc71 | 6.773383 | 5.820123 | 7.134459 | 6.270847 | 6.183911 | 5.994936 | 7.970946 | 7.10802 |
| RGD131174 | 1.60408 | 1.402454 | 1.3307 | 4.43429 | 4.761904 | 4.705244 | 3.426004 | 3.01158 |
| LOC103691 | 0.03704 | 0.010477 | 0.009941 | 0.042664 | 0.080103 | 0.056835 | 0.041439 | 0 |
| Rps20 | 826.2668 | 814.2253 | 797.7625 | 677.8417 | 682.9595 | 563.5778 | 757.0058 | 737.1718 |
| Hist1h2ai | 0.127411 | 0 | 0 | 0 | 0.137768 | 0.111714 | 0 | 0 |
| Atp2a1 | 0.482191 | 0.21823 | 0.517663 | 0.355456 | 0.521386 | 0.676457 | 0.323672 | 0.512868 |
| Synj2 | 8.608097 | 9.130928 | 9.145078 | 8.479414 | 8.616996 | 8.274828 | 9.329407 | 9.030564 |
| LOC100909 | 0.342261 | 0.857487 | 0.078737 | 0.337907 | 0.343647 | 0.557319 | 0.191453 | 0.606726 |
| LOC690483 | 0 | 0 | 0.027363 | 0.011743 | 0 | 0.011174 | 0 | 0 |
| Tlr2 | 0.888585 | 1.462388 | 1.279164 | 2.437779 | 2.249178 | 1.930073 | 2.146372 | 2.792389 |
| AABR07058 | 64.97253 | 89.97158 | 82.24509 | 76.84759 | 57.67108 | 53.5669 | 96.55621 | 83.37423 |
| Myo19 | 4.069017 | 3.562892 | 3.881432 | 3.235981 | 3.222761 | 3.374561 | 3.232957 | 4.031551 |
| Mir652 | 0 | 0 | 0 | 0 | 0 | 0 | 0 | 0.573019 |
| Tnrc6a | 8.07453 | 7.937381 | 7.467655 | 6.880628 | 7.104848 | 7.339563 | 9.315202 | 9.592815 |
| Doxl1 | 0 | 0 | 0 | 0 | 0 | 0.020191 | 0 | 0 |
| RGD156045 | 0.491955 | 0.268004 | 0.469463 | 0.654792 | 0.295525 | 0.287565 | 0.366919 | 0.290696 |
| AC121413. | 5.471066 | 2.652966 | 0.839077 | 4.321176 | 7.606009 | 3.426454 | 9.618378 | 3.46377 |
| Aqp11 | 0.365061 | 0.32126 | 0.566101 | 0.448519 | 0.526313 | 0.248955 | 0.317655 | 0.251667 |
| AC106663. | 0.787457 | 1.336457 | 1.690772 | 3.990861 | 1.702933 | 1.726108 | 1.321459 | 1.046944 |
| Eif5a | 230.5938 | 239.3742 | 240.7471 | 224.496 | 224.0303 | 227.3093 | 248.0916 | 250.2972 |
| Gm9949 | 0 | 0.749995 | 0 | 0.407199 | 0.238914 | 0.193732 | 0.494385 | 0 |
| C4b | 3.456919 | 3.331883 | 3.341158 | 15.1829 | 14.99426 | 16.01005 | 23.56829 | 24.08457 |
| Txnl4b | 6.914514 | 6.154445 | 7.7201 | 7.362568 | 7.376877 | 7.248237 | 7.632509 | 6.973073 |
| Prokr1 | 0.034679 | 0 | 0 | 0.079889 | 0.018749 | 0.015203 | 0.077596 | 0 |
| Gnrhr | 0 | 0 | 0 | 0.018396 | 0 | 0 | 0 | 0 |
| Aarsd1 | 8.191583 | 8.479591 | 7.705545 | 9.314826 | 8.549057 | 8.529953 | 9.075749 | 8.524542 |
| Krt2 | 3.138651 | 3.551236 | 3.480383 | 2.606735 | 2.210421 | 2.353661 | 3.095562 | 3.257801 |
| Pcbd2 | 59.04803 | 59.71246 | 61.78142 | 56.92362 | 57.21233 | 61.33888 | 54.46543 | 50.88675 |
| Cant1 | 6.491805 | 5.614286 | 6.927257 | 7.283151 | 6.574149 | 7.016146 | 8.820613 | 7.700986 |
| Kctd3 | 5.056216 | 5.303054 | 4.71153 | 5.117162 | 4.79149 | 5.043495 | 5.164095 | 4.796293 |
| Grm8 | 0 | 0 | 0 | 0 | 0 | 0 | 0 | 0 |
| Rn50_5_16 | 0 | 0 | 0 | 0 | 0 | 0 | 0 | 0.073441 |
| Rhno1 | 4.363827 | 5.104836 | 4.764251 | 4.430019 | 5.678246 | 6.03114 | 5.792269 | 4.982349 |
| Spint3 | 0.396712 | 0.897721 | 0 | 0.548331 | 0.214479 | 0.173919 | 1.109558 | 0.527438 |
| Slc25a35 | 3.595547 | 3.372775 | 2.738326 | 2.378676 | 3.156772 | 3.017854 | 2.337886 | 2.969004 |
| St6galnac | 0.932933 | 0.940834 | 0.783832 | 0.579337 | 0.548243 | 1.049169 | 0.680689 | 0.485357 |
| Mak16 | 5.095394 | 5.313708 | 4.975933 | 5.289177 | 5.410006 | 5.194312 | 4.979353 | 5.359707 |
| Calhm2 | 5.491129 | 5.98644 | 6.079299 | 7.563435 | 8.349579 | 8.751587 | 9.246842 | 10.3425 |
| Iars | 9.110751 | 9.031659 | 8.782686 | 8.751417 | 8.427852 | 8.95021 | 8.509505 | 8.890251 |
| Fam171a1 | 7.200567 | 8.53615 | 8.902837 | 6.392748 | 7.676517 | 6.916435 | 9.028691 | 9.017575 |
| Git1 | 49.97348 | 51.73453 | 56.63127 | 45.03112 | 43.54325 | 44.15419 | 55.94555 | 57.28412 |
| RGD130723 | 3.415586 | 3.202386 | 2.739837 | 3.474438 | 3.133016 | 3.120974 | 3.19865 | 3.801262 |
| Ppm1f | 19.52817 | 20.41212 | 19.67996 | 15.43853 | 15.15335 | 16.73005 | 20.77115 | 20.10437 |

| | | | | | | | | |
|---|---|---|---|---|---|---|---|---|
| AABR07014 | 0 | 0 | 0 | 0.236535 | 0 | 0 | 0.28718 | 0.227522 |
| Olr875 | 0.111536 | 0 | 0 | 0 | 0 | 0 | 0 | 0 |
| Ephx1 | 24.6936 | 26.01859 | 23.95188 | 28.95737 | 27.18396 | 30.60966 | 26.85964 | 27.32595 |
| Mcrip1 | 23.92305 | 25.00156 | 25.09477 | 23.13795 | 21.81966 | 25.01879 | 25.1296 | 23.02516 |
| Mef2a | 35.96645 | 33.87342 | 31.67413 | 26.57453 | 28.55984 | 27.39191 | 32.37874 | 31.33546 |
| AABR07043 | 57.88011 | 45.51085 | 48.00743 | 41.41452 | 42.6966 | 39.14239 | 44.83111 | 43.7197 |
| Aldh1a1 | 37.35673 | 35.44226 | 35.31163 | 32.47513 | 32.63819 | 30.96365 | 29.38532 | 28.85909 |
| AC113710. | 1.122127 | 0.846423 | 0.401558 | 0.344665 | 0 | 1.311842 | 0.836924 | 1.326129 |
| Ptprb | 33.47774 | 33.42541 | 32.50315 | 24.70251 | 24.69659 | 24.86298 | 38.35568 | 38.4655 |
| Pnrc1 | 26.50821 | 26.75431 | 28.31859 | 24.75596 | 25.35704 | 23.49909 | 33.29501 | 34.85413 |
| AABR07068 | 2.493615 | 3.761878 | 0.892352 | 0 | 0.89877 | 0.728801 | 2.789746 | 2.946954 |
| AABR07052 | 0 | 0.081219 | 0.038532 | 0.033073 | 0 | 0 | 0 | 0.063625 |
| Sdha | 473.2941 | 476.1303 | 484.8292 | 460.7888 | 448.1807 | 463.0568 | 425.0411 | 430.9599 |
| AABR07014 | 0 | 0 | 0 | 0 | 0 | 0 | 0 | 0 |
| AABR07055 | 0 | 0.120426 | 0.114265 | 0 | 0 | 0.093322 | 0.119075 | 0 |
| Atxn1l | 6.06665 | 5.823359 | 7.712069 | 4.439854 | 4.736304 | 4.72395 | 6.958614 | 6.095424 |
| Rbmx | 13.84599 | 13.98865 | 15.60352 | 15.51516 | 15.85888 | 14.09004 | 14.00938 | 15.1821 |
| Gatad2a | 10.70198 | 10.40315 | 10.89241 | 8.797479 | 8.808974 | 8.586702 | 11.79348 | 11.59889 |
| Ofd1 | 1.960774 | 2.268565 | 2.010053 | 2.47243 | 2.773738 | 1.92603 | 2.358561 | 2.299818 |
| LOC103692 | 0.17954 | 0.970564 | 0.36408 | 0.937489 | 0.301987 | 0.349825 | 0.267816 | 0.300589 |
| Efcab5 | 0.01293 | 0 | 0.013881 | 0 | 0 | 0 | 0 | 0 |
| Osbpl1a | 13.92289 | 13.99842 | 12.89373 | 11.87672 | 12.80015 | 13.4616 | 13.57144 | 14.26494 |
| Faim2 | 0.01533 | 0 | 0.032915 | 0 | 0 | 0 | 0 | 0 |
| Opn3 | 0.598126 | 0.642914 | 0.674233 | 0.799167 | 1.131804 | 0.891543 | 1.003735 | 0.848238 |
| LOC688906 | 6.241878 | 6.491693 | 6.655929 | 6.397171 | 6.393231 | 6.578517 | 7.280957 | 7.972741 |
| AABR07067 | 0.929573 | 0.818041 | 0.332652 | 0.475869 | 1.005133 | 0.81505 | 0.924413 | 0.549284 |
| AC115322. | 0.715381 | 0.323768 | 0.614406 | 0.263678 | 0 | 0.501798 | 0 | 0.507262 |
| Cttn | 1.011526 | 3.22806 | 7.907854 | 9.320814 | 8.918256 | 3.797781 | 8.414836 | 6.758726 |
| Apod | 0.108982 | 0.554887 | 0.116999 | 0.502114 | 0.707045 | 0.095556 | 0.304811 | 0.048298 |
| Zfp870 | 3.170838 | 3.48109 | 3.336689 | 2.835016 | 3.496474 | 3.743627 | 2.774693 | 2.838298 |
| AABR07010 | 1.988579 | 1.499989 | 2.134867 | 1.221598 | 3.583703 | 0 | 1.483156 | 0.587526 |
| Dnm3 | 1.346329 | 1.346179 | 1.747889 | 1.081909 | 1.167998 | 1.276546 | 1.909037 | 1.956486 |
| Commd4 | 29.32255 | 27.41298 | 30.84367 | 35.26191 | 35.93352 | 34.17617 | 34.52784 | 34.44335 |
| Osbpl8 | 15.34022 | 15.97608 | 13.28725 | 15.83808 | 15.85204 | 15.07047 | 15.17273 | 16.56337 |
| Syt3 | 1.712685 | 2.60146 | 2.140925 | 2.486161 | 1.953375 | 2.24224 | 2.283542 | 2.079503 |
| Soat1 | 2.870468 | 2.944267 | 2.448024 | 3.21358 | 2.494639 | 2.822612 | 4.201769 | 5.017143 |
| AABR07050 | 0 | 0 | 0 | 0 | 0 | 0 | 0 | 0 |
| Trpc5 | 0.01542 | 0.052341 | 0.033108 | 0 | 0 | 0 | 0 | 0 |
| Rnf122 | 7.993039 | 8.00167 | 8.616351 | 7.365271 | 7.93142 | 7.700482 | 8.537464 | 6.297447 |
| Lst1 | 1.190135 | 1.481239 | 2.427602 | 2.960988 | 2.316376 | 3.130533 | 2.529792 | 2.320726 |
| Capns1 | 9.33823 | 6.01436 | 5.783761 | 7.347185 | 6.679748 | 53.78734 | 69.27295 | 8.65895 |
| Mctp1 | 0.584278 | 0.661083 | 0.392037 | 0.942181 | 0.789714 | 0.736425 | 1.021351 | 0.873914 |
| AC120727. | 4.219055 | 3.868851 | 3.700511 | 4.929494 | 4.562003 | 4.714758 | 5.244546 | 4.057299 |
| Nepro | 1.075841 | 1.188955 | 1.262424 | 1.106618 | 1.40677 | 1.074921 | 1.511501 | 1.485797 |
| Rn50_11_0 | 0.055887 | 0.126467 | 0.119996 | 0 | 0.06043 | 0.049002 | 0.187571 | 0.049535 |
| Hdac5 | 30.71205 | 32.66712 | 37.0568 | 30.12734 | 28.05046 | 31.11094 | 33.75964 | 31.82981 |
| Ogt | 38.6258 | 39.07884 | 36.10602 | 37.41739 | 38.41703 | 37.13276 | 32.22625 | 35.08908 |
| Tbc1d9 | 1.678213 | 1.330246 | 1.363975 | 1.415355 | 1.291768 | 1.288565 | 1.941154 | 2.1766 |
| Zfp593 | 3.647378 | 3.271991 | 3.719908 | 4.345183 | 3.774834 | 3.7691 | 3.934791 | 3.556137 |
| Bend7 | 2.266222 | 2.518336 | 1.781256 | 2.573003 | 2.669222 | 2.412817 | 2.127882 | 2.188011 |
| Tmem170a | 1.108841 | 0.99154 | 1.152012 | 1.170073 | 1.218312 | 0.909508 | 1.180497 | 1.601047 |
| Asb7 | 1.40502 | 1.945062 | 1.54387 | 0.852959 | 1.358368 | 1.550773 | 2.256101 | 2.080449 |
| #N/A | 0.162124 | 0.36687 | 3.132901 | 0 | 0 | 0 | 0 | 0 |
| Atp10a | 0.667671 | 0.685705 | 0.716787 | 1.504953 | 1.232856 | 1.486051 | 1.528396 | 1.565966 |

| | | | | | | | | |
|---|---|---|---|---|---|---|---|---|
| AABR07013 | 0 | 0 | 0 | 0 | 0 | 0 | 0 | 0 |
| AABR07042 | 0 | 0 | 0 | 0 | 0 | 0 | 0.741578 | 0 |
| LOC100911 | 6.074624 | 2.809463 | 3.522561 | 4.167502 | 3.931453 | 4.237662 | 8.879471 | 8.528324 |
| Dach1 | 6.443014 | 4.872376 | 6.317044 | 4.452738 | 4.691884 | 5.418659 | 4.670592 | 4.603574 |
| Ermap | 0.058971 | 0.033361 | 0.031654 | 0.02717 | 0 | 0 | 0.164934 | 0.104537 |
| Zmynd12 | 0.431587 | 0.434063 | 0.514818 | 0.61863 | 0.259261 | 0.714786 | 0.321894 | 0.467546 |
| Klhdc8b | 14.99384 | 15.92332 | 16.29448 | 28.5749 | 24.70599 | 26.32909 | 26.11137 | 27.34105 |
| AABR07036 | 14.08829 | 14.01538 | 14.64906 | 9.732274 | 10.079 | 8.872139 | 10.58687 | 11.7018 |
| Rpusd3 | 7.034228 | 9.095884 | 9.469584 | 8.779533 | 9.095016 | 8.615085 | 7.70303 | 9.203732 |
| Pitx3 | 0.042061 | 0.09518 | 0.045155 | 0.038758 | 0.04548 | 0.073758 | 0.094112 | 0 |
| Clip1 | 21.97181 | 23.78633 | 22.86649 | 25.10426 | 23.14497 | 23.80665 | 31.92444 | 31.44925 |
| Clic5 | 77.27915 | 77.12774 | 72.84306 | 70.82734 | 73.88744 | 71.99333 | 84.71101 | 87.45937 |
| Map7 | 1.942029 | 1.93239 | 2.040533 | 1.713354 | 1.608425 | 1.811461 | 1.664162 | 1.721317 |
| Rcbtb1 | 7.374571 | 5.928617 | 6.562837 | 4.649468 | 6.470145 | 6.267512 | 6.947656 | 6.70846 |
| AABR07058 | 0 | 0 | 0 | 0 | 0 | 0 | 0 | 0.108699 |
| Fbxo36 | 0.378435 | 0.374659 | 0.050784 | 0.174356 | 0.255748 | 0.041477 | 0.105844 | 0.167713 |
| Prickle1 | 7.472506 | 7.530046 | 7.671241 | 7.50963 | 8.168287 | 7.442549 | 7.863538 | 8.009997 |
| Tulp3 | 10.94299 | 10.82569 | 10.76387 | 13.46231 | 12.5759 | 11.75491 | 16.34474 | 14.42202 |
| Sgk3 | 2.107281 | 2.503503 | 2.11148 | 2.394857 | 1.89881 | 2.432759 | 2.671869 | 2.770551 |
| LOC103691 | 0.824943 | 0.608729 | 0.693101 | 0.694052 | 0.930781 | 0.471724 | 0.521644 | 2.734006 |
| Stx11 | 1.905679 | 1.882867 | 1.844163 | 3.042099 | 2.728096 | 2.941728 | 3.393166 | 3.449567 |
| Sypl1 | 46.92145 | 45.0812 | 42.40273 | 38.85791 | 35.80384 | 38.62372 | 39.48097 | 39.35144 |
| Eif3e11 | 53.69659 | 54.37853 | 49.70559 | 52.11194 | 49.30137 | 48.31659 | 53.37422 | 52.97083 |
| AC120262. | 0 | 0 | 0.390404 | 0.335091 | 0 | 0.637701 | 0.813676 | 0.644646 |
| Alpp | 0 | 0.037264 | 0 | 0 | 0.071223 | 0.086631 | 0.036846 | 0.029192 |
| AABR07005 | 0 | 0 | 0 | 0.427019 | 0 | 0 | 0 | 0 |
| Pde6b | 0.018891 | 0 | 0 | 0 | 0 | 0 | 0.063403 | 0 |
| Plscr1 | 14.71861 | 17.18286 | 14.56831 | 17.75684 | 17.20294 | 15.92647 | 20.51177 | 20.62449 |
| Ntsr1 | 0.378701 | 0.57131 | 0.519493 | 0.484664 | 0.477731 | 0.387386 | 1.082722 | 1.268054 |
| Rn60_12_0 | 0 | 0 | 0 | 0 | 0 | 0 | 0 | 0.043664 |
| Fbln7 | 0.435393 | 0.358274 | 0.297451 | 2.151878 | 2.139929 | 2.498748 | 3.055436 | 2.455795 |
| LOC691670 | 0 | 0 | 0.150316 | 0 | 0 | 0 | 0 | 0 |
| Ffar1 | 0 | 0 | 0 | 0 | 0 | 0 | 0.064734 | 0 |
| Hmgb2 | 11.8187 | 11.16073 | 11.71212 | 13.27799 | 14.59799 | 12.91345 | 12.10343 | 13.33612 |
| Ly6g6e | 0.303864 | 0.114603 | 0.217478 | 0.93333 | 0.87617 | 1.68738 | 0.793216 | 0.448883 |
| AABR07068 | 2.228337 | 2.941468 | 4.385814 | 1.539994 | 2.20868 | 2.279443 | 3.323953 | 3.127219 |
| Mnt | 5.848335 | 6.921585 | 7.318568 | 8.199231 | 7.293616 | 8.902912 | 8.549869 | 8.904464 |
| AABR07056 | 0 | 0 | 0 | 0 | 0 | 0 | 0 | 0 |
| AC110351. | 0.131243 | 0.148495 | 0.140898 | 0.483741 | 0.283822 | 0.115074 | 0.440486 | 0.697963 |
| Ccr3 | 0.032166 | 0.127379 | 0.120862 | 0 | 0.052171 | 0.042305 | 0.053979 | 0.099786 |
| Atp8a2 | 0 | 0 | 0 | 0.02547 | 0.014944 | 0.012118 | 0.030924 | 0 |
| LOC100910 | 13.67096 | 0.749317 | 0.812548 | 0.741015 | 0.818392 | 8.17087 | 0.476298 | 8.092143 |
| LOC102546 | 0.205357 | 0 | 0.220463 | 0 | 0.222049 | 0.360114 | 0 | 0 |
| Crlf2 | 3.764059 | 3.603647 | 5.639579 | 6.288916 | 6.619377 | 6.310522 | 6.75621 | 6.819195 |
| Sigirr | 4.662044 | 4.680531 | 5.145988 | 5.142967 | 5.112001 | 4.490696 | 5.142228 | 5.529002 |
| Rn60_6_01 | 0 | 0 | 0 | 0 | 0 | 0 | 0 | 0 |
| RGD130731 | 0.266765 | 0.332015 | 0.286389 | 0.442464 | 0.317294 | 0.397629 | 0.417822 | 0.780275 |
| Glyatl2 | 0.026731 | 0 | 0 | 0 | 0 | 0.046875 | 0 | 0.047386 |
| LOC100911 | 0.875684 | 3.03844 | 0.752082 | 0.806909 | 3.408713 | 0.972548 | 0.71843 | 1.86279 |
| Adgrg7 | 0 | 0 | 0 | 0 | 0.020695 | 0 | 0 | 0.016964 |
| Hsph1 | 13.23584 | 11.76051 | 12.59443 | 10.89406 | 12.10992 | 11.05892 | 18.56484 | 18.53348 |
| Gch1 | 2.473424 | 2.318809 | 2.048435 | 1.725652 | 1.872134 | 2.106738 | 3.439181 | 2.912652 |
| Hcn2 | 46.4307 | 50.8553 | 61.66077 | 48.19617 | 43.94317 | 47.92019 | 54.2963 | 56.15042 |
| Fam49a | 7.817 | 8.585097 | 7.888954 | 8.544443 | 8.581782 | 8.445048 | 9.726929 | 9.517981 |

| | | | | | | | | |
|---|---|---|---|---|---|---|---|---|
| Rpl101 | 16.96591 | 17.81179 | 15.14913 | 15.8589 | 16.66924 | 16.02001 | 19.61948 | 19.88161 |
| Mydgf | 40.694 | 45.21052 | 50.2351 | 56.00464 | 55.94031 | 55.04206 | 55.29075 | 49.49018 |
| Apc2 | 1.441691 | 1.598318 | 1.990852 | 1.821277 | 1.546308 | 1.636162 | 1.944584 | 1.767338 |
| AABR0704З | 0.489401 | 0 | 0 | 0 | 0.529182 | 0 | 0 | 0 |
| Pcdhb14 | 0.721836 | 0.816723 | 0.962801 | 1.128728 | 1.111637 | 0.901412 | 1.17463 | 1.240823 |
| Rarb | 5.893707 | 6.323532 | 6.909088 | 5.836569 | 6.281217 | 5.746735 | 5.778888 | 6.274666 |
| Plin3 | 18.40958 | 19.29998 | 18.61133 | 30.92304 | 30.6974 | 30.33075 | 26.20074 | 28.82846 |
| Abcf1 | 84.26741 | 82.85174 | 82.20362 | 84.24414 | 86.10044 | 86.87803 | 99.62612 | 95.25177 |
| Acbd6 | 16.22456 | 14.98311 | 17.4181 | 14.7213 | 13.2823 | 15.19069 | 15.05328 | 14.25476 |
| Sema3b | 7.424241 | 7.857471 | 8.283839 | 12.37556 | 10.30525 | 10.78835 | 12.97215 | 12.79126 |
| Itpka | 0.231069 | 0.228763 | 0.310084 | 0.10646 | 0.21862 | 0.278577 | 0.452391 | 0.256009 |
| Pdik1l | 1.469839 | 1.1351 | 1.214783 | 1.117916 | 1.412725 | 1.073963 | 1.383376 | 1.34415 |
| AABR07015 | 0 | 0.096341 | 0 | 0 | 0.092069 | 0 | 0 | 0 |
| Neurl4 | 9.998965 | 10.5599 | 10.962 | 9.260139 | 8.978869 | 9.589846 | 11.58148 | 11.98371 |
| AABR07008 | 2.909218 | 3.291643 | 4.438276 | 2.962912 | 3.807947 | 3.356322 | 3.083403 | 1.62858 |
| AABR07012 | 0 | 0 | 0 | 0 | 0 | 0 | 0.137523 | 0 |
| RGD156434 | 0 | 0 | 0 | 0 | 0 | 0.036484 | 0 | 0 |
| Lasp1 | 15.19046 | 15.66329 | 16.25973 | 17.34851 | 16.73269 | 17.37376 | 21.90018 | 22.25881 |
| AC110102. | 0.038561 | 0 | 0 | 0.071065 | 0 | 0 | 0.04314 | 0 |
| Tnnt3 | 5.643937 | 6.385849 | 5.374182 | 10.58222 | 10.24194 | 11.6615 | 16.30707 | 18.31351 |
| Cpne5 | 0.218647 | 0.274876 | 0.195609 | 0.190281 | 0.288957 | 0.276914 | 0.258202 | 0.204564 |
| Mthfs | 5.327928 | 6.049829 | 5.107028 | 4.92701 | 4.259033 | 4.738268 | 5.322008 | 5.144051 |
| Vav1 | 1.447715 | 1.404018 | 1.176762 | 1.715159 | 1.654844 | 1.287491 | 2.01298 | 1.668136 |
| Rims3 | 0.03572 | 0 | 0 | 0.032915 | 0.038624 | 0 | 0.079925 | 0.094982 |
| Lsm8 | 18.92269 | 17.39573 | 16.08249 | 15.68076 | 15.6298 | 13.76858 | 15.06883 | 13.97677 |
| LOC100911 | 0 | 0 | 0.23135 | 0 | 0.233014 | 0 | 0.241089 | 0.191006 |
| AABR07060 | 8.05091 | 6.970538 | 7.590955 | 37.93161 | 34.97273 | 35.41799 | 19.97205 | 18.86365 |
| Olr1637 | 0.052576 | 0 | 0.056444 | 0.096894 | 0.1137 | 0.138297 | 0.11764 | 0.186404 |
| AABR07044 | 0 | 0 | 0.711622 | 1.832398 | 0 | 0.581196 | 0 | 0 |
| Mical2 | 22.57204 | 22.61604 | 23.38909 | 25.44914 | 25.10929 | 24.90457 | 33.85605 | 36.46472 |
| Rfc3 | 4.558821 | 4.153861 | 3.984646 | 4.275124 | 4.624026 | 4.598523 | 3.430227 | 4.50557 |
| RGD156565 | 0.586733 | 0.829826 | 0.944843 | 1.216466 | 0.634426 | 0.900284 | 0.656411 | 0.780076 |
| Stxbp2 | 0.8159 | 0.783281 | 0.530861 | 1.412509 | 1.096092 | 1.430763 | 1.49366 | 1.490173 |
| Rapgef3 | 15.58065 | 16.18694 | 17.10988 | 15.5625 | 14.86295 | 16.74493 | 22.86741 | 22.43921 |
| Rnft1 | 9.210087 | 8.08851 | 7.674674 | 9.739536 | 10.71749 | 9.536677 | 10.40197 | 10.72898 |
| LOC100360 | 0 | 0 | 0 | 0 | 0 | 0 | 0 | 0 |
| Slc17a2 | 0 | 0 | 0 | 0.017099 | 0.020065 | 0 | 0 | 0 |
| RGD156330 | 0 | 0 | 0 | 0 | 0.080316 | 0 | 0 | 0 |
| AABR07005 | 0 | 0 | 0 | 0 | 0 | 0 | 0 | 0 |
| AABR07064 | 8.533706 | 9.070305 | 10.27196 | 7.267756 | 10.06622 | 9.409635 | 12.00624 | 11.57498 |
| Rhbdl3 | 4.245131 | 4.360769 | 4.737317 | 4.838181 | 4.31841 | 4.726131 | 4.343077 | 5.186049 |
| Gng3 | 2.452524 | 2.569366 | 2.974248 | 2.343604 | 2.946531 | 2.429127 | 2.540532 | 1.85175 |
| Nob1 | 14.35011 | 14.84146 | 13.86151 | 16.34737 | 15.77579 | 15.61513 | 16.24585 | 15.39056 |
| Rps8 | 419.6045 | 417.2158 | 393.6887 | 441.0032 | 445.9023 | 439.4861 | 434.1662 | 429.601 |
| Lynx1 | 31.33162 | 31.39206 | 34.13334 | 21.42925 | 19.76659 | 21.28338 | 22.23784 | 23.46364 |
| Hnrnpa3 | 2.053566 | 2.212869 | 2.04716 | 2.387877 | 3.277867 | 1.800568 | 1.422223 | 2.426903 |
| A2m | 0 | 0 | 0 | 0 | 0 | 0 | 0 | 0 |
| Sez6l | 0.153085 | 0.098976 | 0.129129 | 0.261972 | 0.248292 | 0.297212 | 0.024466 | 0.04846 |
| Sp2 | 6.677534 | 7.48904 | 7.084914 | 6.153084 | 6.016934 | 5.837748 | 7.404997 | 7.735753 |
| AABR07062 | 0.494018 | 0 | 0.53036 | 0 | 0 | 0 | 0 | 0 |
| Zc3h7a | 9.928533 | 9.484426 | 8.511905 | 10.86088 | 9.907406 | 10.3718 | 11.69472 | 11.51563 |
| AABR07013 | 0 | 0 | 0 | 0.106755 | 0 | 0 | 0 | 0 |
| Birc5 | 0.359903 | 0.407214 | 0.965948 | 0.552728 | 0.324298 | 0.999284 | 0.872395 | 0.957 |
| Ppp1r21 | 2.974573 | 2.757121 | 3.193394 | 3.515232 | 3.216362 | 3.403802 | 3.365422 | 3.530244 |

| | | | | | | | | |
|---|---|---|---|---|---|---|---|---|
| AABR0707( | 0 | 0 | 0 | 0.099491 | 0 | 0 | 0 | 0 |
| Gpx3 | 195.7658 | 190.3413 | 189.3342 | 363.3448 | 371.0549 | 359.872 | 376.2109 | 378.0823 |
| Zfp14 | 0 | 0.078685 | 0.261306 | 0.192244 | 0.075196 | 0.182926 | 0 | 0.123279 |
| Prickle2 | 1.384692 | 1.458665 | 1.391357 | 1.709633 | 1.681638 | 1.501177 | 2.396194 | 2.327679 |
| Kirrel2 | 0.0467 | 0.035226 | 0.050135 | 0.057376 | 0 | 0.013649 | 0.017415 | 0.041392 |
| Pick1 | 7.534047 | 7.552052 | 7.657726 | 6.783947 | 5.420645 | 6.555624 | 7.082719 | 7.363354 |
| Alyref | 16.47467 | 18.01897 | 17.81295 | 18.79523 | 17.98348 | 19.84469 | 20.40589 | 20.26941 |
| AABR07005 | 0 | 0 | 0.097941 | 0 | 0 | 0.039995 | 0 | 0 |
| Ubap2l | 20.46804 | 19.87048 | 19.77936 | 18.05328 | 17.90691 | 17.34899 | 20.25418 | 20.76698 |
| Fmo4 | 0.886233 | 0.973238 | 0.615629 | 1.465128 | 1.437406 | 1.211283 | 1.020639 | 0.924132 |
| AABR07047 | 0.010208 | 0.01155 | 0.010959 | 0.009406 | 0 | 0.053701 | 0.01142 | 0.018095 |
| Mbd4 | 2.031835 | 2.403422 | 2.677055 | 2.950221 | 3.095763 | 2.996183 | 2.204244 | 3.028813 |
| Tlr7 | 0.574778 | 0.733355 | 0.958413 | 1.036732 | 1.282668 | 1.254553 | 1.600749 | 1.734312 |
| Grk6 | 7.966806 | 8.33093 | 9.438201 | 8.250255 | 8.248161 | 8.560547 | 10.82399 | 9.697972 |
| Lgals4 | 0 | 0.056214 | 0.053338 | 0.045781 | 0.107443 | 0.043562 | 0 | 0.044037 |
| RGD131081 | 0.025134 | 0.042656 | 0.013491 | 0 | 0.054353 | 0.011019 | 0.056237 | 0.011139 |
| AC120949. | 2.992338 | 5.501746 | 2.007791 | 4.480648 | 4.853358 | 5.57533 | 1.673848 | 4.30992 |
| Ctdspl | 136.4604 | 141.7245 | 145.1957 | 125.8954 | 127.6841 | 129.7121 | 147.6655 | 150.7371 |
| Ankrd16 | 1.387517 | 1.162896 | 1.103399 | 1.444279 | 1.444735 | 1.39681 | 1.264831 | 1.457571 |
| Spc24 | 1.029157 | 1.849409 | 1.624802 | 2.175575 | 1.374651 | 2.441695 | 1.760926 | 1.609752 |
| Syngr3 | 0.171832 | 0.09721 | 0.092237 | 0.237505 | 0.69675 | 0.188328 | 0.288358 | 0.190379 |
| Ppp2r2a | 21.05342 | 21.01997 | 20.11212 | 18.0641 | 19.17159 | 17.67747 | 21.93182 | 22.73283 |
| Pdlim7 | 37.66509 | 38.37916 | 43.95782 | 51.01942 | 46.01416 | 46.64716 | 54.1181 | 55.54929 |
| Ambp | 0 | 0 | 0 | 0 | 0 | 0.037118 | 0 | 0 |
| AABR07029 | 0.563074 | 0.637092 | 0 | 1.556553 | 0.608844 | 0.987408 | 1.259885 | 0 |
| Usp11 | 3.703561 | 3.451932 | 4.04119 | 4.405721 | 4.250792 | 4.045798 | 4.16036 | 4.78944 |
| Socs6 | 12.55786 | 13.88662 | 12.8706 | 13.53841 | 12.30924 | 11.9309 | 11.50202 | 11.68246 |
| AABR07048 | 0 | 0 | 0 | 0 | 0 | 0 | 0 | 0 |
| Mmp28 | 0.033185 | 0 | 0 | 0.030579 | 0 | 0 | 0 | 0 |
| Chodl | 0.04294 | 0.09717 | 0.046099 | 0.811143 | 0.742895 | 0.941256 | 0.936778 | 1.122778 |
| Arhgap26 | 5.066436 | 4.823898 | 4.823391 | 3.153454 | 3.348977 | 3.201777 | 5.411435 | 4.677038 |
| Dot1l | 4.579298 | 4.53114 | 4.925515 | 3.850624 | 3.294743 | 3.702165 | 5.756199 | 5.802779 |
| RGD130843 | 13.79847 | 10.2611 | 11.82989 | 11.14225 | 14.28743 | 11.32899 | 11.89149 | 11.75489 |
| Eef2k | 11.44767 | 12.89447 | 12.52107 | 12.49572 | 12.21183 | 12.32181 | 14.78101 | 14.66535 |
| Myh15 | 0 | 0 | 0 | 0.016798 | 0 | 0 | 0.030593 | 0.016158 |
| Necap1 | 9.336883 | 9.217126 | 8.162509 | 8.96927 | 7.52106 | 7.930184 | 10.00169 | 9.904973 |
| Gatc | 15.41751 | 13.25759 | 14.99727 | 13.11952 | 13.71349 | 13.96477 | 12.89883 | 13.71284 |
| Grxcr2 | 0 | 0.101629 | 0 | 0.082767 | 0 | 0.039378 | 0 | 0 |
| Pcnx4 | 6.165953 | 6.639458 | 5.979975 | 6.244362 | 5.861944 | 5.95478 | 5.615197 | 4.976756 |
| Trmt10a | 2.311779 | 2.242002 | 1.817063 | 3.138261 | 3.146935 | 3.022357 | 2.401579 | 2.561306 |
| Abcc6 | 0.009068 | 0 | 0 | 0 | 0 | 0.015901 | 0.010145 | 0.008037 |
| Pign | 2.333597 | 2.202811 | 2.276213 | 2.03973 | 1.917696 | 2.0461 | 2.043825 | 1.879274 |
| Dedd2 | 5.813206 | 5.778498 | 5.255451 | 4.684349 | 5.038767 | 5.489103 | 6.187594 | 6.466742 |
| Kif16b | 3.60537 | 3.659841 | 3.601942 | 3.723605 | 3.477524 | 3.884447 | 3.846887 | 3.869246 |
| LOC10091( | 0.073137 | 0.358587 | 0.026172 | 0.067393 | 0.105442 | 0.064126 | 0.109096 | 0.108041 |
| Calml4 | 0.116888 | 0.264507 | 0.125487 | 0.323124 | 0.063195 | 0.153732 | 0.196154 | 0.051802 |
| Fam222b | 3.119782 | 3.763267 | 3.833686 | 3.38556 | 3.19216 | 3.368419 | 3.821993 | 4.227812 |
| Zfyve16 | 5.352919 | 4.815299 | 4.396327 | 5.812686 | 5.614188 | 5.108059 | 4.962292 | 5.758854 |
| Nkg7 | 4.220182 | 5.178453 | 3.828706 | 3.505336 | 4.177597 | 3.074863 | 3.52439 | 3.37177 |
| Eefsec | 4.702765 | 3.221336 | 3.65691 | 3.021677 | 3.023532 | 3.343288 | 4.009921 | 4.280951 |
| Cep63 | 8.197244 | 7.474394 | 5.771938 | 6.998038 | 6.67374 | 6.32064 | 6.931979 | 7.586167 |
| LOC100912 | 0 | 0.087389 | 0.082918 | 0 | 0 | 0 | 0 | 0 |
| LOC10036( | 4.324699 | 3.970477 | 3.767333 | 2.846457 | 3.340167 | 2.903512 | 3.953566 | 4.731258 |
| AABR07012 | 0 | 0 | 0 | 0 | 0.566225 | 0 | 0 | 0 |

| | | | | | | | | |
|---|---|---|---|---|---|---|---|---|
| AC121199. | 0 | 0 | 0 | 0 | 0 | 0 | 0 | 0 |
| Fam133b | 9.508175 | 9.393225 | 8.836459 | 9.807548 | 8.362946 | 7.652414 | 9.367196 | 9.55963 |
| Cdr2 | 3.098707 | 2.900682 | 2.632609 | 3.184008 | 2.651544 | 3.322887 | 4.189963 | 4.248242 |
| Ap4e1 | 1.616589 | 1.861756 | 1.890467 | 1.596024 | 2.075743 | 1.860372 | 1.857011 | 2.046947 |
| Dusp12 | 4.21382 | 4.212275 | 4.172442 | 3.882869 | 4.379397 | 4.483836 | 4.531158 | 4.60519 |
| AABR07064 | 0 | 0 | 0 | 0 | 0 | 0 | 0.156087 | 0 |
| AC120262. | 25.76065 | 30.10261 | 33.09618 | 30.35278 | 36.07402 | 34.43586 | 37.3241 | 32.19071 |
| Olr1423 | 0 | 0 | 0 | 0 | 0 | 0 | 0 | 0 |
| Mmrn1 | 2.545566 | 2.983052 | 2.440024 | 2.94252 | 2.850786 | 3.118757 | 3.82682 | 3.928312 |
| Pole2 | 0.339637 | 0.279479 | 0.464065 | 0.170707 | 0.467403 | 0.270722 | 0.449057 | 0.218936 |
| Clgn | 0.046056 | 0.026055 | 0 | 0.127317 | 0.0498 | 0.060573 | 0.025763 | 0.081644 |
| Hrasls5 | 0 | 0.040638 | 0 | 0.033095 | 0 | 0 | 0 | 0 |
| Ly49i3 | 0.198808 | 0.26993 | 0.384179 | 0.329748 | 0.257961 | 0.278903 | 0.578284 | 0.387669 |
| Ccdc13 | 0.025847 | 0.029245 | 0 | 0.023817 | 0.027948 | 0.045325 | 0.028916 | 0.022909 |
| Kazn | 1.518935 | 1.634082 | 1.764336 | 1.697923 | 1.373157 | 1.506467 | 1.950036 | 1.478732 |
| Tmem102 | 0.034361 | 0 | 0 | 0 | 0 | 0 | 0 | 0 |
| Mypop | 1.357893 | 2.048523 | 1.588612 | 1.668326 | 1.505918 | 1.648525 | 1.811295 | 1.74363 |
| Mdh2 | 691.4763 | 687.0076 | 676.1523 | 679.6412 | 671.3567 | 666.1604 | 612.0077 | 632.6309 |
| LOC103689 | 0.034451 | 0.03898 | 0.036986 | 0.063491 | 0.111755 | 0.030207 | 0 | 0.030536 |
| Pfkl | 18.03229 | 19.73551 | 21.05373 | 20.62984 | 18.65478 | 18.14564 | 22.472 | 23.72148 |
| AABR07058 | 0 | 0 | 0 | 0 | 0 | 0 | 0 | 0 |
| Prlr | 0.029238 | 0 | 0 | 0 | 0.031615 | 0 | 0 | 0 |
| AABR07028 | 2.380269 | 2.693163 | 3.407161 | 2.924432 | 0.857917 | 1.391348 | 0.887646 | 0 |
| Wdr46 | 3.978042 | 4.106146 | 4.070884 | 4.287262 | 4.049855 | 4.569011 | 4.502509 | 4.63939 |
| Swsap1 | 5.492204 | 6.448668 | 6.081648 | 7.097922 | 5.154292 | 6.935631 | 7.265117 | 6.031438 |
| Fam175b | 6.234431 | 5.971611 | 6.214984 | 6.611059 | 6.473692 | 6.247262 | 5.4433 | 6.110636 |
| Tp53bp1 | 5.904368 | 5.617278 | 5.329878 | 5.739495 | 6.159508 | 5.847772 | 6.977688 | 6.921269 |
| mrpl24 | 30.48656 | 28.99289 | 29.69617 | 32.17885 | 31.79244 | 30.01436 | 30.24792 | 30.283 |
| AABR07072 | 0 | 0 | 0 | 0.112478 | 0 | 0.107027 | 0.136561 | 0 |
| AABR07003 | 1.232139 | 0.697054 | 0.66139 | 0.189228 | 1.554343 | 1.080341 | 0.459488 | 1.456142 |
| Vps41 | 10.004 | 8.949954 | 8.013326 | 9.593459 | 10.0625 | 9.689469 | 10.71486 | 9.451309 |
| Gbp2 | 10.97553 | 12.06007 | 10.37804 | 17.05683 | 15.83878 | 15.28632 | 19.05596 | 18.2777 |
| AABR07043 | 0 | 0 | 0 | 0 | 0 | 0 | 0 | 0 |
| Gpatch2l | 3.507348 | 3.691993 | 3.652963 | 3.569542 | 3.226408 | 3.059946 | 4.685212 | 4.57804 |
| Tfg | 8.833153 | 8.952546 | 7.753164 | 7.953813 | 9.80005 | 9.208125 | 11.2019 | 8.109789 |
| Cxcl10 | 3.370998 | 3.239395 | 2.875356 | 4.084921 | 4.743509 | 4.210852 | 2.996394 | 2.946954 |
| LOC100910 | 17.99239 | 9.115319 | 11.82023 | 10.14553 | 6.968925 | 7.299226 | 5.70825 | 9.996973 |
| St8sia1 | 0.318774 | 0.426256 | 0.46667 | 0.240331 | 0.282016 | 0.254092 | 0.291789 | 0.12843 |
| Pdcd6ip | 12.73278 | 13.81435 | 13.58226 | 13.81987 | 12.68469 | 12.53292 | 16.34475 | 16.02073 |
| AABR07002 | 0.893382 | 0.816431 | 1.106657 | 1.298149 | 0.594462 | 0.723063 | 0.84571 | 0.883216 |
| Ccbe1 | 3.133004 | 4.268284 | 3.500765 | 3.122608 | 2.488902 | 2.074281 | 2.646682 | 2.60692 |
| Hba-a2 | 504.1043 | 585.5815 | 518.7816 | 386.0251 | 423.2965 | 557.6508 | 320.6906 | 397.5649 |
| Gjb2 | 0.043313 | 0.122518 | 0.0465 | 0 | 0.023417 | 0.018989 | 0.048457 | 0.115173 |
| AC139608. | 0.037809 | 0 | 0 | 0 | 0 | 0 | 0 | 0 |
| Wdr31 | 1.98526 | 1.610503 | 1.729171 | 1.794823 | 1.944121 | 1.674992 | 1.718148 | 1.593632 |
| Itgb4 | 3.144603 | 2.846377 | 3.544729 | 4.720084 | 3.98183 | 4.629179 | 3.860589 | 3.916775 |
| LOC683674 | 8.513555 | 9.2337 | 8.490862 | 7.728857 | 7.598692 | 6.625467 | 8.989182 | 8.952488 |
| Hsbp1l1 | 0.131573 | 0.744341 | 0.282503 | 0.969912 | 0.284535 | 0.692178 | 0.294395 | 0.816336 |
| Tvp23a | 0.358944 | 0.541504 | 0.299716 | 0.808506 | 0.129374 | 0.209815 | 0.446189 | 0.4949 |
| Cd80 | 0.230349 | 0.152033 | 0.082431 | 0.229945 | 0.249073 | 0.20197 | 0.558358 | 0.391325 |
| Tubg1 | 13.01252 | 14.00834 | 15.46169 | 13.30017 | 12.53345 | 13.70788 | 13.99248 | 16.43264 |
| Slc44a3 | 0.17311 | 0.097933 | 0.216819 | 0.558301 | 0.561546 | 0.303567 | 0.258224 | 0.434737 |
| Gng5 | 40.72905 | 44.93808 | 49.15694 | 38.69575 | 35.28649 | 38.37298 | 35.37721 | 34.97906 |
| Hpn | 2.716728 | 1.826491 | 2.113465 | 2.067992 | 1.490066 | 1.104709 | 1.805993 | 2.547564 |

| | | | | | | | | |
|---|---|---|---|---|---|---|---|---|
| AABR07009 | 0.272739 | 0.771479 | 0 | 0 | 0 | 0.358707 | 0 | 0.241742 |
| Cacna2d2 | 8.506206 | 8.356843 | 8.488663 | 9.674634 | 9.845556 | 10.8847 | 10.44906 | 9.640826 |
| AABR07055 | 0 | 0.822911 | 0 | 0.670182 | 0.786424 | 2.550805 | 0 | 1.933938 |
| AABR07062 | 0 | 0 | 0 | 0 | 0 | 0 | 0 | 0 |
| Cyp2c24 | 0 | 0 | 0.028265 | 0 | 0 | 0 | 0 | 0 |
| Enpp3 | 5.822637 | 6.117465 | 6.007428 | 6.567304 | 6.950056 | 6.365041 | 5.752718 | 5.797626 |
| Gli1 | 0.174419 | 0.28843 | 0.043212 | 0.395619 | 0.406208 | 0.329389 | 0.375254 | 0.451896 |
| Rgmb | 10.14565 | 10.98219 | 11.02065 | 9.606459 | 6.996832 | 7.73997 | 9.160836 | 9.311227 |
| Trim23 | 10.81618 | 9.966806 | 9.352258 | 9.85894 | 10.99998 | 9.722866 | 10.94511 | 10.13968 |
| Piezo1 | 4.596302 | 5.00548 | 5.462133 | 4.75296 | 4.417702 | 5.059941 | 6.64907 | 6.586188 |
| Zranb2 | 26.87808 | 25.94306 | 24.68062 | 32.15638 | 32.15653 | 32.18784 | 30.49829 | 30.64537 |
| Dnajc25 | 5.277825 | 6.018264 | 4.957822 | 7.180978 | 5.885175 | 6.326799 | 7.519134 | 8.04031 |
| AC128967. | 0.086842 | 0.098258 | 0 | 0 | 0 | 0 | 0 | 0.076973 |
| Ide | 13.038 | 11.59259 | 11.74888 | 16.16392 | 15.81437 | 16.17027 | 13.28893 | 14.10099 |
| Pigu | 12.69627 | 14.47702 | 12.46346 | 11.8812 | 12.92703 | 11.56525 | 12.6565 | 11.91014 |
| Olr39 | 0.052895 | 0 | 0 | 0 | 0 | 0 | 0 | 0.046883 |
| AABR07069 | 0 | 0 | 0 | 0 | 0 | 0 | 0 | 0 |
| Sorbs1 | 150.7078 | 151.9627 | 152.2159 | 121.2982 | 121.9085 | 123.0515 | 164.1787 | 168.7195 |
| F2rl3 | 0.362468 | 0.277431 | 0.263237 | 0.36347 | 0.288185 | 0.28977 | 0.500928 | 0.274027 |
| LOC100363 | 0 | 0 | 0 | 0 | 0 | 0 | 0.075691 | 0 |
| Prpf8 | 20.65315 | 20.70273 | 19.58238 | 19.01795 | 18.46888 | 18.65783 | 21.70451 | 22.65846 |
| AABR07028 | 0.41233 | 0 | 0 | 0 | 0 | 0.12051 | 0 | 0.243646 |
| Csrnp2 | 3.007814 | 3.012027 | 3.22908 | 2.686629 | 3.190001 | 2.627149 | 3.132939 | 3.309486 |
| Rn50_12_( | 0 | 0 | 0.103724 | 0 | 0 | 0 | 0 | 0 |
| Ascl4 | 0 | 0.270546 | 0 | 0.110167 | 0.129275 | 0 | 0 | 0.105969 |
| Tmbim4 | 23.53457 | 24.44896 | 23.71502 | 26.73335 | 27.20484 | 26.54596 | 31.37983 | 29.23581 |
| Slc6a7 | 0.112979 | 0.234356 | 0.101075 | 0.416424 | 0.183244 | 0.297181 | 0.315991 | 0.267038 |
| AC095947. | 39.09989 | 59.24958 | 57.71731 | 55.33026 | 56.62251 | 64.28028 | 57.02241 | 73.64437 |
| AABR07004 | 1.227326 | 1.190282 | 0.878409 | 0.592393 | 0.568753 | 0.66617 | 1.634617 | 1.036038 |
| Ccny | 40.02972 | 39.71675 | 39.42221 | 38.84112 | 38.30575 | 37.33705 | 40.23672 | 39.05068 |
| Ift52 | 9.455825 | 11.19316 | 10.95551 | 12.19269 | 10.96682 | 10.23362 | 9.985229 | 10.45571 |
| Sertad1 | 16.01939 | 16.1959 | 18.40217 | 19.10278 | 16.7393 | 16.48515 | 18.02219 | 17.46013 |
| Pdgfrb | 12.59957 | 12.62406 | 13.86251 | 21.67476 | 20.22405 | 21.38886 | 22.45078 | 22.01816 |
| Foxg1 | 0 | 0 | 0 | 0 | 0 | 0 | 0 | 0.016512 |
| AABR07014 | 0 | 0 | 0 | 0 | 0.293381 | 0 | 0 | 0 |
| Slc7a1 | 1.297781 | 1.382633 | 1.322062 | 1.239498 | 1.177929 | 1.470127 | 2.670647 | 2.53567 |
| Mrps16 | 63.58103 | 63.93488 | 67.1469 | 57.3155 | 61.00679 | 50.90683 | 54.04846 | 49.24374 |
| RGD131049 | 0.447572 | 0.368296 | 0.82995 | 0.674869 | 0.703932 | 0.249729 | 0.227602 | 0.396705 |
| AABR07035 | 0.039137 | 0.044282 | 0 | 0.252445 | 0.211594 | 0.583368 | 0.087571 | 0.312205 |
| Egfl8 | 3.922057 | 4.096267 | 4.627009 | 6.711753 | 5.499141 | 6.839936 | 5.159308 | 4.851559 |
| Prm3 | 0 | 0 | 0 | 0 | 0 | 0.178309 | 0 | 0 |
| LOC685171 | 0.042958 | 0 | 0 | 0 | 0 | 0 | 0 | 0 |
| Sdpr | 70.905 | 70.06276 | 69.63366 | 68.36239 | 71.82128 | 68.31741 | 67.01689 | 66.83458 |
| Pex1 | 4.665548 | 4.742715 | 4.291363 | 4.791727 | 4.335366 | 3.739208 | 4.811826 | 4.716835 |
| AABR07042 | 0.449493 | 0.50858 | 0.482559 | 0.41419 | 0.48603 | 0.197058 | 0.502873 | 0.199204 |
| Zfp213 | 5.888712 | 5.354918 | 5.291672 | 4.78311 | 4.787326 | 4.417429 | 4.953222 | 5.142133 |
| AC118113. | 0.952108 | 1.077265 | 0.511074 | 0.87733 | 0.51475 | 0 | 0.532588 | 0.8439 |
| Car1 | 0 | 0 | 0 | 0.082555 | 0 | 0.11783 | 0 | 0 |
| RGD156586 | 0.052105 | 0 | 0 | 0 | 0.112682 | 0.182744 | 0 | 0 |
| Acp1 | 14.35242 | 13.63747 | 14.57213 | 11.61903 | 13.39371 | 15.34818 | 15.76641 | 8.119253 |
| Mlf2 | 92.30838 | 91.76028 | 100.0303 | 85.98587 | 79.99947 | 82.48785 | 96.6317 | 96.91991 |
| Nxph1 | 0.161043 | 0.212582 | 0.144075 | 0.123663 | 0.319245 | 0.211804 | 0.180168 | 0.04758 |
| AC135310. | 0 | 0 | 0.669264 | 0 | 0.674078 | 0.546601 | 2.789746 | 0 |
| Ces2g | 0.013777 | 0.015588 | 0.029581 | 0.038085 | 0.014897 | 0.072477 | 0.030826 | 0.012211 |

| | | | | | | | | |
|---|---|---|---|---|---|---|---|---|
| 43165 | 20.3301 | 20.4825 | 19.75816 | 15.97122 | 15.51823 | 16.72421 | 19.4283 | 20.27574 |
| AABR0700( | 0.529305 | 0.399256 | 0.340946 | 0.195094 | 0.152621 | 0.278457 | 0.31582 | 0.37532 |
| Hbe1 | 0 | 0 | 0 | 0 | 0.10004 | 0 | 0 | 0 |
| Ppp1r17 | 0 | 0 | 0.032421 | 0 | 0 | 0.026479 | 0 | 0 |
| Hs6st2 | 0.422519 | 0.448181 | 0.567001 | 0.389334 | 0.856619 | 0.578851 | 0.94539 | 0.397906 |
| AABR07013 | 0.650508 | 0.24534 | 0 | 0.199806 | 0.351693 | 0.190122 | 0.121293 | 0.192193 |
| Psmd7 | 37.9554 | 39.47484 | 37.80919 | 42.11514 | 40.29184 | 40.99921 | 39.2532 | 40.80269 |
| Slc7a3 | 0 | 0 | 0 | 0.038603 | 0.067947 | 0.073463 | 0.070302 | 0 |
| Fstl5 | 0 | 0 | 0 | 0 | 0 | 0.024705 | 0 | 0 |
| Slain2 | 30.12325 | 29.27233 | 29.9572 | 30.30551 | 29.98358 | 30.19757 | 35.85269 | 36.00007 |
| Ftsj3 | 6.130465 | 6.417146 | 6.601151 | 7.391034 | 6.728017 | 7.531714 | 6.961164 | 7.483589 |
| Zfp189 | 2.281371 | 2.00765 | 1.836898 | 2.082729 | 1.918633 | 1.741009 | 2.410503 | 2.1906 |
| Shroom2 | 0.837781 | 1.020826 | 0.94883 | 0.780466 | 0.73665 | 0.774928 | 1.091768 | 1.126091 |
| Rab16 | 19.00932 | 18.83356 | 19.96714 | 17.30457 | 16.6495 | 18.1499 | 21.68918 | 20.10811 |
| Rassf5 | 2.26314 | 2.281755 | 2.23718 | 2.229927 | 2.204813 | 1.984323 | 2.481764 | 2.641477 |
| AABR07003 | 0 | 0 | 0 | 0 | 0 | 0 | 0 | 0.580181 |
| Omg | 0.028491 | 0.128944 | 0 | 0.052506 | 0.061613 | 0 | 0.063748 | 0 |
| Tmem79 | 0.281744 | 0.24633 | 0.357464 | 0.295018 | 0.373883 | 0.325635 | 0.444149 | 0.374585 |
| AABR07065 | 0 | 0 | 0.545807 | 0 | 0 | 0 | 0 | 0.450626 |
| AABR07027 | 0.83354 | 0.332863 | 0.894858 | 1.039159 | 0.265087 | 0.300938 | 0.548546 | 0.434593 |
| AABR07053 | 2.710451 | 2.747806 | 2.537383 | 2.69738 | 2.461848 | 2.433563 | 2.547159 | 3.036643 |
| Efcab12 | 0 | 0 | 0.027721 | 0.023793 | 0.055841 | 0 | 0 | 0.045774 |
| AABR07011 | 0 | 0 | 0 | 0 | 0 | 0 | 0 | 0 |
| LOC171573 | 0 | 0 | 0 | 0 | 0.09597 | 0 | 0 | 0 |
| AABR07037 | 0 | 0.586629 | 0 | 0.477754 | 0 | 0 | 0 | 0 |
| Slc10a6 | 2.637133 | 2.823946 | 3.08391 | 2.907338 | 2.902413 | 3.05546 | 3.582516 | 3.673091 |
| AC118439. | 0 | 0 | 1.338528 | 0.382961 | 0 | 0 | 0 | 0.368369 |
| AABR07002 | 0 | 0.051276 | 0 | 0.02088 | 0.024501 | 0 | 0.050701 | 0 |
| Sgsm2 | 14.41611 | 14.67614 | 14.62706 | 15.74092 | 14.21143 | 13.60541 | 20.34297 | 20.11194 |
| RGD156577 | 12.3981 | 11.74185 | 10.98334 | 13.26919 | 13.26687 | 12.99649 | 14.15813 | 14.55439 |
| Ino80 | 4.310643 | 4.205871 | 3.690181 | 3.714161 | 4.07992 | 3.79921 | 4.997922 | 4.436025 |
| AABR07064 | 0 | 0 | 0 | 0 | 0 | 0 | 0 | 0.433781 |
| Ror2 | 0.385239 | 0.586183 | 0.470624 | 0.612039 | 0.25855 | 0.396015 | 0.743083 | 0.824205 |
| Zfp964 | 0.509396 | 0.461086 | 0.546869 | 0.531974 | 0.624243 | 0.774174 | 0.873831 | 1.083607 |
| Gm1141 | 0.330236 | 0.053378 | 0.050647 | 0 | 0.204045 | 0.082729 | 0.052779 | 0.418149 |
| Cd47 | 23.00185 | 20.78634 | 20.97572 | 19.77303 | 20.80094 | 21.95049 | 29.22916 | 29.56381 |
| Stard3 | 16.94779 | 16.83162 | 16.3724 | 14.60474 | 13.17054 | 13.06528 | 17.00575 | 15.26502 |
| Aaed1 | 9.767737 | 9.056282 | 9.248325 | 10.25067 | 10.12164 | 8.504885 | 9.409972 | 9.318977 |
| AC118802. | 0.588381 | 0.665726 | 0.631665 | 0.722893 | 0.848277 | 0.687857 | 1.097091 | 0.695349 |
| Pigv | 3.89983 | 4.233934 | 3.94471 | 2.534172 | 3.046842 | 3.241488 | 3.35418 | 3.896182 |
| Krt83 | 0 | 0 | 0 | 0 | 0 | 0 | 0 | 0 |
| Coq5 | 25.89745 | 27.47751 | 28.05499 | 30.82753 | 29.74589 | 30.62929 | 28.03346 | 29.117 |
| Lep | 0.254667 | 0.090045 | 0.136701 | 0.044 | 0.103263 | 0.083735 | 0.160262 | 0.112862 |
| Mrps17 | 23.41206 | 25.03567 | 25.00501 | 26.82786 | 25.53224 | 24.43608 | 22.95764 | 24.55983 |
| Lrch1 | 4.094729 | 3.860827 | 3.699034 | 3.911173 | 4.067606 | 3.852964 | 5.195525 | 4.986684 |
| Lars2 | 9.126882 | 9.673804 | 10.64297 | 9.118956 | 9.660803 | 9.382192 | 9.389195 | 9.391385 |
| Clcn3 | 13.74388 | 15.21055 | 13.20982 | 13.94692 | 13.81788 | 14.13073 | 15.72992 | 16.07896 |
| AABR0706( | 0 | 0 | 0 | 0 | 0 | 0 | 0.54752 | 0 |
| Fxr1 | 50.71778 | 45.7722 | 47.2372 | 49.0585 | 46.55089 | 47.51284 | 50.28727 | 46.28172 |
| Haus8 | 5.338984 | 5.999433 | 5.653223 | 6.638176 | 5.417098 | 5.963753 | 5.891195 | 5.380454 |
| Msrb1 | 38.86769 | 41.93152 | 39.33331 | 40.53485 | 34.85966 | 35.18878 | 32.42719 | 37.22775 |
| AABR07006 | 0.239114 | 0 | 0.128352 | 0.110167 | 0 | 0.943448 | 1.471304 | 0 |
| Prelid3b | 42.12429 | 42.82495 | 37.47877 | 41.68808 | 39.28909 | 39.12102 | 42.84253 | 48.23005 |
| Mfn2 | 11.412 | 15.3945 | 18.47768 | 9.763463 | 18.00372 | 14.33057 | 12.53891 | 12.3762 |

| | | | | | | | | |
|---|---|---|---|---|---|---|---|---|
| AABR0705( | 1.146699 | 0.864957 | 0.820703 | 1.408851 | 1.653212 | 0.335142 | 2.565752 | 5.081882 |
| Actg2 | 3.687741 | 3.004204 | 3.563123 | 12.13125 | 11.56375 | 10.76727 | 8.870214 | 8.204256 |
| Spata5 | 2.714883 | 2.75671 | 2.092534 | 3.35158 | 3.21783 | 3.128106 | 2.531076 | 2.452612 |
| AABR07067 | 0.831205 | 0.940469 | 0 | 0 | 0.89877 | 0 | 0 | 0 |
| Ano5 | 10.24992 | 10.2883 | 9.260744 | 3.796661 | 4.301555 | 3.772818 | 2.997355 | 2.806459 |
| Hacd3 | 4.210055 | 4.074295 | 4.327433 | 4.457183 | 4.552272 | 4.194036 | 5.171004 | 4.747842 |
| Cd300le | 13.46757 | 11.42844 | 11.8435 | 16.63446 | 18.20286 | 17.77538 | 22.44009 | 27.23916 |
| Egfl7 | 60.0266 | 60.42968 | 61.58514 | 59.6536 | 55.65029 | 55.5641 | 60.19413 | 59.16576 |
| Pcdhb8 | 0.268543 | 0.075961 | 0.120124 | 0.226831 | 0.41136 | 0.392432 | 0.250362 | 0.257858 |
| AC099294. | 6.71358 | 3.03844 | 5.765965 | 6.186299 | 8.711156 | 14.71618 | 5.257598 | 8.330811 |
| Crygd | 0.429229 | 0.679913 | 0.460805 | 0.316414 | 0.371295 | 0.225809 | 0.09604 | 0.228268 |
| Rhobtb2 | 4.706039 | 4.544615 | 4.762615 | 4.465325 | 4.494365 | 4.678179 | 4.739534 | 5.446466 |
| Glis3 | 0.205137 | 0.168802 | 0.260269 | 0.223394 | 0.201647 | 0.163513 | 0.584178 | 0.380176 |
| AABR07057 | 0.08195 | 0.092722 | 0.175957 | 0.151027 | 0.531667 | 1.724488 | 0.641773 | 0.145272 |
| #N/A | 0 | 0 | 0 | 0 | 0 | 0 | 0 | 0 |
| Afmid | 1.793496 | 1.557337 | 2.350819 | 2.03697 | 2.412827 | 2.907369 | 2.216465 | 2.495404 |
| Ctu1 | 1.936023 | 2.111343 | 2.429025 | 1.740982 | 2.068172 | 1.881574 | 2.374701 | 2.294883 |
| RGD130517 | 26.09314 | 23.93358 | 25.11978 | 25.49222 | 24.81484 | 27.24942 | 26.83037 | 26.27237 |
| Mrap2 | 0 | 0 | 0 | 0 | 0.044726 | 0.036267 | 0.046275 | 0 |
| Tdrd7 | 4.900554 | 5.148693 | 5.080679 | 5.392997 | 6.510075 | 5.254385 | 8.239448 | 6.527817 |
| AABR07054 | 0 | 0 | 0 | 0 | 0 | 0 | 0 | 0 |
| Prdm15 | 1.200433 | 1.358234 | 1.246068 | 1.296615 | 1.186262 | 1.059511 | 1.209582 | 1.261302 |
| LOC499643 | 0.192947 | 0 | 0 | 0.106676 | 0.083452 | 0.067671 | 0.259033 | 0.068408 |
| Nphp4 | 0.363869 | 0.446989 | 0.401797 | 0.498146 | 0.573307 | 0.556042 | 0.744375 | 0.774036 |
| Trim14 | 0.379134 | 0.223065 | 0.260495 | 0.307434 | 0.245971 | 0.305831 | 0.373259 | 0.430137 |
| Rorc | 2.132708 | 2.233575 | 2.308521 | 1.656621 | 1.467497 | 1.792689 | 2.307104 | 2.234021 |
| AABR07006 | 0 | 0 | 0.128352 | 0 | 0 | 0 | 0.066877 | 0.211938 |
| Plekhf1 | 22.95881 | 24.57374 | 26.01707 | 23.60421 | 23.29262 | 21.12439 | 26.12131 | 25.84516 |
| Lclat1 | 3.099208 | 3.361507 | 3.396036 | 4.155678 | 3.166238 | 3.860565 | 3.754201 | 3.902608 |
| Hoxa3 | 0 | 0.044382 | 0.463221 | 0.072289 | 0 | 0.068786 | 0.043884 | 0.13907 |
| AABR07065 | 0 | 0 | 0 | 0 | 0 | 0.095456 | 0 | 0 |
| AABR07061 | 0 | 0 | 0 | 0.062585 | 0 | 0 | 0.075985 | 0 |
| LOC103694 | 0 | 0 | 0 | 0 | 0 | 0 | 0 | 0 |
| Rn50_10_( | 1.665859 | 0.983395 | 1.010838 | 1.935465 | 1.331373 | 2.349704 | 2.511929 | 1.861716 |
| LOC100125 | 5.32325 | 4.248788 | 4.164308 | 5.152325 | 5.131276 | 5.246336 | 5.332174 | 5.888682 |
| Ccm2l | 9.798197 | 10.4703 | 13.2851 | 10.96814 | 10.71237 | 10.56383 | 14.61572 | 13.57375 |
| Xcr1 | 0.11147 | 0.112109 | 0.146263 | 0.068477 | 0.200884 | 0.108596 | 0.180133 | 0.20858 |
| Ttc34 | 0 | 0.035585 | 0 | 0 | 0 | 0 | 0 | 0 |
| Acbd3 | 9.591311 | 10.19118 | 9.851833 | 10.38337 | 9.148438 | 10.87144 | 11.82656 | 13.19448 |
| Pnpla1 | 0 | 0 | 0 | 0.020835 | 0 | 0 | 0.025296 | 0.020041 |
| Ccdc146 | 0.232737 | 0.141794 | 0.192199 | 0.313439 | 0.232297 | 0.204064 | 0.320463 | 0.301496 |
| AABR07057 | 2.473232 | 2.583088 | 3.012599 | 1.402453 | 2.211415 | 2.085126 | 2.766942 | 3.456849 |
| Nradd | 1.395262 | 0.888004 | 1.029808 | 1.084792 | 1.46153 | 1.223367 | 1.560957 | 1.700449 |
| Gcnt1 | 8.521763 | 8.161327 | 7.541461 | 7.447305 | 7.380623 | 7.214871 | 9.709454 | 9.752431 |
| Rarres1 | 3.138033 | 2.440994 | 2.189771 | 7.481947 | 8.22829 | 7.463253 | 7.240801 | 7.753137 |
| Gldc | 0 | 0.01827 | 0 | 0.014879 | 0 | 0 | 0.03613 | 0 |
| Inca1 | 0.279285 | 0.236998 | 0.59966 | 0.321688 | 0.45298 | 0.367316 | 0.468677 | 0.495088 |
| Pars2 | 1.831533 | 1.513675 | 1.86368 | 2.010547 | 1.584328 | 1.550033 | 1.24724 | 0.917562 |
| AABR07057 | 4.225293 | 3.409202 | 4.313034 | 3.893441 | 3.782324 | 3.826207 | 4.572084 | 4.42043 |
| C2cd4d | 0.561428 | 1.559199 | 1.479425 | 0.987637 | 1.15894 | 1.074023 | 1.598802 | 0.497622 |
| AABR07051 | 0 | 0 | 0 | 0 | 0 | 0 | 0 | 0 |
| Thop1 | 5.077511 | 5.769622 | 5.918933 | 5.702826 | 6.479896 | 6.018753 | 6.411888 | 6.48992 |
| Notch3 | 12.58128 | 12.92261 | 15.11348 | 15.88203 | 14.76537 | 15.53922 | 16.7751 | 17.66329 |
| Tesk2 | 1.176762 | 1.25313 | 1.356221 | 1.21191 | 1.459536 | 1.183519 | 1.277788 | 1.089039 |

| VST_3 | YXS_1 | YXS_2 | YXS_3 |
|---|---|---|---|
| 1.231729 | 1.847224 | 1.667593 | 1.625423 |
| 26.84778 | 24.48099 | 24.63989 | 25.08853 |
| 26.60014 | 29.03241 | 28.51333 | 27.26654 |
| 16.42029 | 14.44857 | 14.82056 | 12.44866 |
| 3.484101 | 3.448978 | 3.387111 | 3.433515 |
| 0 | 0 | 0 | 0 |
| 339.2217 | 309.5797 | 302.5577 | 300.1982 |
| 3.655416 | 2.947884 | 2.49913 | 2.296373 |
| 0.328613 | 0.742022 | 0.201263 | 0.235408 |
| 1.550192 | 1.711905 | 1.616604 | 1.400643 |
| 3.031114 | 2.133312 | 1.552661 | 2.039796 |
| 0.051255 | 0.027556 | 0.031392 | 0.091794 |
| 0 | 0 | 0 | 0 |
| 0.133586 | 0.0798 | 0.12727 | 0.124052 |
| 0.18413 | 0.118792 | 0.090218 | 0.219842 |
| 2.244411 | 0.9282 | 1.818724 | 1.649053 |
| 580.6851 | 601.8128 | 590.6552 | 607.9908 |
| 25.53139 | 18.80517 | 18.3344 | 17.20393 |
| 22.45667 | 26.10772 | 24.27785 | 24.13613 |
| 3.408588 | 2.864867 | 3.117773 | 3.03893 |
| 0.031586 | 0.033963 | 0.058036 | 0.037712 |
| 8.380851 | 7.920997 | 8.663905 | 8.31734 |
| 2.594008 | 2.751017 | 2.611615 | 2.927407 |
| 0.066689 | 0.143416 | 0 | 0 |
| 16.90249 | 13.87912 | 13.74548 | 14.1169 |
| 8.06052 | 5.919016 | 6.742897 | 5.774305 |
| 2.844507 | 3.366244 | 3.484307 | 3.556961 |
| 19.10458 | 14.22642 | 13.90921 | 14.26919 |
| 0.879166 | 1.190414 | 1.27634 | 1.205186 |
| 0 | 0 | 0 | 0.081216 |
| 45.47083 | 42.40537 | 41.69164 | 41.64354 |
| 0.12116 | 0.043426 | 0 | 0.04822 |
| 16.9508 | 15.71128 | 15.46465 | 12.66333 |
| 31.38483 | 27.4464 | 24.1719 | 27.05854 |
| 11.02487 | 10.79856 | 11.56432 | 11.63121 |
| 5.17013 | 3.645389 | 3.903631 | 3.460853 |
| 3.991208 | 4.366428 | 3.567213 | 4.377423 |
| 45.45757 | 42.70492 | 41.45751 | 39.12056 |
| 1.133715 | 0.845861 | 0.935258 | 0.690611 |
| 1.229645 | 1.406579 | 1.345986 | 0.99958 |
| 1.440691 | 0.971477 | 0.957146 | 1.224485 |
| 3.151379 | 2.553012 | 2.168058 | 3.144076 |
| 7.827953 | 8.44288 | 8.382717 | 8.056054 |
| 1.984002 | 1.533318 | 1.291072 | 1.110373 |
| 15.15975 | 11.36386 | 12.3797 | 12.48035 |
| 0.078965 | 0.084908 | 0 | 0 |
| 7.268402 | 7.141929 | 6.851394 | 6.852043 |
| 14.35688 | 16.06317 | 16.19414 | 16.19826 |
| 3.63895 | 2.539162 | 2.465817 | 2.865665 |
| 67.071 | 77.36432 | 77.15267 | 77.23792 |
| 42.14103 | 40.31737 | 36.03887 | 39.48542 |

| | | | |
|---|---|---|---|
| 18.89412 | 18.55054 | 17.66396 | 18.32531 |
| 16.3506 | 17.46589 | 16.3271 | 16.14448 |
| 3.865509 | 3.589632 | 2.797929 | 2.377536 |
| 0.956145 | 1.439343 | 1.015045 | 0.608847 |
| 0 | 0 | 0 | 0 |
| 95.85896 | 103.8032 | 100.015 | 105.0014 |
| 2.063492 | 2.532763 | 1.621493 | 1.534003 |
| 65.47906 | 63.28184 | 62.67022 | 63.71512 |
| 2.68414 | 2.231662 | 2.517848 | 2.442262 |
| 5.588272 | 4.408845 | 3.969412 | 4.184872 |
| 10.52149 | 10.15495 | 9.808983 | 9.217937 |
| 12.50994 | 10.40117 | 10.49097 | 10.56306 |
| 3.585673 | 2.474212 | 2.230673 | 2.612487 |
| 0.041952 | 0.360877 | 0.102777 | 0.250445 |
| 2.733669 | 2.129563 | 1.810944 | 2.031585 |
| 0.020577 | 0.066378 | 0.025206 | 0.024568 |
| 3.058389 | 2.627482 | 2.699631 | 2.687348 |
| 63.88148 | 57.52506 | 60.24617 | 57.57769 |
| 10.25591 | 11.34166 | 10.83231 | 10.96317 |
| 0.451337 | 0.647071 | 0.276427 | 0.269436 |
| 0 | 0.044612 | 0 | 0 |
| 0 | 0.03969 | 0.090428 | 0.132212 |
| 5.493554 | 4.366766 | 5.051712 | 4.491706 |
| 0.273655 | 0 | 0 | 0 |
| 0.89323 | 0.664928 | 0.883728 | 0.779344 |
| 2.336158 | 1.32832 | 1.82784 | 1.620979 |
| 9.421305 | 9.272348 | 9.656242 | 8.952927 |
| 13.57606 | 11.59234 | 11.24947 | 13.70624 |
| 0 | 0.249754 | 0 | 0 |
| 4.71446 | 3.168285 | 3.392729 | 3.588373 |
| 30.61163 | 33.21316 | 31.36807 | 31.45657 |
| 0.687809 | 0.866355 | 0.962873 | 0.915061 |
| 0.553675 | 0.396895 | 0.67821 | 2.203531 |
| 43.30848 | 25.28679 | 23.81529 | 27.52204 |
| 0.055475 | 0.039767 | 0.067953 | 0.022078 |
| 0.071603 | 0.230975 | 0 | 0 |
| 26.97714 | 27.89989 | 26.63977 | 26.72285 |
| 22.62111 | 26.40552 | 26.84253 | 25.25233 |
| 49.34553 | 51.75342 | 52.10582 | 46.61438 |
| 0.054418 | 0.073142 | 0.033329 | 0.032486 |
| 29.54138 | 32.39151 | 30.23432 | 29.93384 |
| 0.063658 | 0 | 0 | 0 |
| 6.041734 | 5.98185 | 6.521381 | 4.928047 |
| 97.29759 | 97.75372 | 98.66109 | 97.79433 |
| 67.35928 | 69.23725 | 75.47613 | 72.72009 |
| 2.959533 | 2.310407 | 2.060904 | 2.178203 |
| 6.495112 | 5.50609 | 5.647961 | 5.399265 |
| 13.73142 | 13.3001 | 12.75277 | 13.22895 |
| 10.3216 | 8.020223 | 8.008155 | 8.503418 |
| 7.51512 | 6.984722 | 6.83887 | 6.992865 |
| 4.407831 | 4.410558 | 3.677587 | 3.892815 |
| 0.547311 | 0 | 0.223471 | 0.435641 |
| 11.4697 | 10.84699 | 12.74371 | 11.29008 |
| 5.110379 | 3.730162 | 3.883835 | 3.451594 |

| | | | |
|---|---|---|---|
| 0.390295 | 0 | 0.159361 | 0 |
| 11.06204 | 9.031836 | 9.685681 | 9.440745 |
| 4.412784 | 4.112227 | 4.45296 | 4.064376 |
| 32.74234 | 31.06343 | 29.28669 | 31.25805 |
| 0.049446 | 0.026583 | 0 | 0.059035 |
| 124.9296 | 107.8759 | 108.4074 | 109.0325 |
| 12.89569 | 10.98426 | 12.21302 | 11.2093 |
| 14.94036 | 14.78064 | 16.37114 | 14.68299 |
| 20.05341 | 17.9347 | 17.35794 | 18.7369 |
| 0.190084 | 0.442843 | 0.659709 | 0.264776 |
| 0.048787 | 0 | 0 | 0 |
| 0.167278 | 0.062562 | 0.097997 | 0.078152 |
| 0.398006 | 0.233432 | 0.265924 | 0.388799 |
| 0.107243 | 0.115314 | 0 | 0 |
| 0 | 0.573664 | 0 | 0 |
| 41.58964 | 42.99957 | 44.51919 | 44.14842 |
| 6.371767 | 3.456505 | 3.86731 | 3.289756 |
| 28.0431 | 23.83566 | 22.33963 | 24.37126 |
| 1.948745 | 1.488838 | 1.528559 | 1.382754 |
| 0.380928 | 0 | 0.466608 | 0.151603 |
| 0.37604 | 0.353797 | 0.115155 | 0.224486 |
| 18.07367 | 17.17467 | 18.6268 | 17.82313 |
| 0.112567 | 0 | 0 | 0 |
| 19.13218 | 14.44651 | 15.80706 | 15.13916 |
| 0.576522 | 0.493398 | 0.33148 | 0.238811 |
| 0.220008 | 0.439336 | 0.42349 | 0.375255 |
| 0.572538 | 0.410417 | 1.168859 | 0.68358 |
| 6.233856 | 3.300564 | 3.45825 | 3.054078 |
| 0.173194 | 0.106416 | 0.121228 | 0.103392 |
| 0.012225 | 0.039435 | 0.029949 | 0.014596 |
| 155.4337 | 164.288 | 161.4198 | 159.5159 |
| 2.079781 | 1.569333 | 1.474912 | 1.655434 |
| 6.989218 | 7.926998 | 8.170339 | 7.163542 |
| 0.340636 | 0.340109 | 0.38745 | 0.522902 |
| 48.82426 | 48.96763 | 48.26984 | 53.11526 |
| 13.92974 | 12.30223 | 10.96634 | 12.06591 |
| 11.77509 | 9.796292 | 11.4757 | 12.62217 |
| 84.67802 | 99.54869 | 93.98312 | 100.7495 |
| 0.034182 | 0.07351 | 0.041871 | 0.040812 |
| 0.023227 | 0.099901 | 0.085355 | 0.083197 |
| 0.216191 | 0.217932 | 0.099307 | 0.177458 |
| 0 | 0 | 0 | 0.173857 |
| 0.067668 | 0.097015 | 0 | 0.026931 |
| 0.094289 | 0.06759 | 0.038499 | 0 |
| 69.97413 | 73.71229 | 71.96626 | 69.03841 |
| 7.932501 | 6.855256 | 7.629038 | 5.979036 |
| 0.678964 | 0.158709 | 0.28928 | 0.352456 |
| 0.048538 | 0.052191 | 0 | 0 |
| 6.977814 | 5.708219 | 4.742187 | 4.926725 |
| 0 | 0 | 0 | 0 |
| 9.0649 | 9.202567 | 10.51451 | 8.691671 |
| 39.63324 | 36.27529 | 39.80127 | 36.54371 |
| 0.696347 | 0.540239 | 0.626233 | 0.905071 |
| 55.55205 | 26.61026 | 3.837241 | 5.610305 |

| | | | |
|---|---|---|---|
| 2.06892 | 1.671233 | 1.891247 | 1.904868 |
| 2.416917 | 2.44945 | 2.484132 | 2.089626 |
| 7.398249 | 6.623342 | 6.94643 | 5.72013 |
| 2.901635 | 2.267053 | 1.917779 | 2.018824 |
| 10.23642 | 7.660237 | 7.263944 | 7.678983 |
| 1.631609 | 1.126678 | 0.971795 | 1.28679 |
| 3.799236 | 3.578731 | 3.865327 | 3.41144 |
| 4.529802 | 4.590299 | 4.62586 | 4.335904 |
| 0 | 0 | 0 | 0 |
| 0.925426 | 0.404247 | 0.619925 | 0.483398 |
| 1.024257 | 1.20379 | 1.10875 | 1.393548 |
| 0.180958 | 0.194576 | 0.088664 | 0.172843 |
| 15.38113 | 14.07546 | 14.95232 | 13.1285 |
| 2.569609 | 2.307443 | 2.380425 | 1.902367 |
| 0 | 0 | 0 | 0.166231 |
| 11.91221 | 12.11537 | 11.99453 | 12.4741 |
| 0.172765 | 0.154806 | 0.176354 | 0.160434 |
| 20.97541 | 16.76443 | 17.43473 | 16.37893 |
| 6.64 | 8.613982 | 9.189174 | 8.98018 |
| 0.033842 | 0 | 0 | 0 |
| 14.483 | 9.34377 | 8.465682 | 8.190925 |
| 120.1272 | 146.7017 | 144.084 | 152.9009 |
| 15.40765 | 14.14053 | 14.74363 | 13.57241 |
| 3.44256 | 3.935425 | 2.796455 | 2.942065 |
| 4.081375 | 1.70665 | 4.44389 | 4.87295 |
| 0.149189 | 0.507984 | 0.274117 | 1.009367 |
| 4.332078 | 3.965321 | 4.088837 | 4.146613 |
| 8.128499 | 6.806686 | 6.822913 | 7.54058 |
| 0.122564 | 0 | 0.450394 | 0.146335 |
| 1112.78 | 1276.799 | 1260.075 | 1266.477 |
| 8.979549 | 9.523958 | 9.689833 | 9.700057 |
| 0.012501 | 0 | 0.030625 | 0 |
| 13.07291 | 11.82044 | 11.76099 | 11.65027 |
| 0.118154 | 0 | 0 | 0.282139 |
| 0.2176 | 0.220213 | 0.17247 | 0.183391 |
| 8.398369 | 5.709547 | 6.252065 | 6.042207 |
| 19.89438 | 15.27656 | 16.35427 | 16.52478 |
| 5.356109 | 4.698064 | 4.586731 | 4.684537 |
| 5.028693 | 4.481402 | 3.631148 | 3.854707 |
| 87.97295 | 81.02894 | 79.76275 | 85.49916 |
| 6.713591 | 6.050469 | 7.748287 | 5.791676 |
| 9.101128 | 7.304082 | 8.64389 | 6.771736 |
| 10.73999 | 11.04761 | 10.15193 | 10.72907 |
| 32.60384 | 34.74317 | 35.21891 | 34.34882 |
| 0.114553 | 0.082116 | 0.140319 | 0.136771 |
| 50.26324 | 54.44224 | 52.70279 | 52.49024 |
| 4.549348 | 3.635889 | 3.743628 | 3.494482 |
| 0.537076 | 0.668678 | 0.882941 | 0.70874 |
| 44.3739 | 46.72473 | 48.84215 | 48.47364 |
| 1.25925 | 1.045786 | 0.978162 | 1.124554 |
| 0 | 0.028365 | 0.032314 | 0.031496 |
| 6.680228 | 5.804836 | 5.804061 | 6.445596 |
| 0 | 0 | 0 | 0 |
| 0.093426 | 0.083714 | 0.11444 | 0.018591 |

| | | | |
|---|---|---|---|
| 0.025261 | 0.095065 | 0.061884 | 0.090479 |
| 6.034532 | 5.268154 | 5.575807 | 5.476291 |
| 2.521651 | 2.332801 | 2.560113 | 2.48181 |
| 0.046095 | 0.148692 | 0.028231 | 0.137587 |
| 37.85421 | 34.94452 | 33.34816 | 33.62179 |
| 101.1223 | 124.3212 | 123.6112 | 125.272 |
| 9.168954 | 9.494872 | 9.699739 | 8.770244 |
| 0 | 0.459188 | 0 | 0 |
| 19.25694 | 11.28519 | 10.31514 | 12.0134 |
| 0.059224 | 0 | 0.14509 | 0 |
| 0.038002 | 0 | 0.046549 | 0 |
| 1.030747 | 0.674629 | 0.850874 | 0.722343 |
| 0 | 0.083523 | 0 | 0 |
| 0.125504 | 0.053979 | 0.122986 | 0.119876 |
| 0.201478 | 0.10832 | 0.411326 | 0.441017 |
| 3.072003 | 2.615028 | 2.665438 | 4.584765 |
| 0 | 0 | 0 | 0.280055 |
| 23.20452 | 21.41423 | 21.73366 | 21.86857 |
| 223.3197 | 179.1718 | 181.7358 | 179.3356 |
| 0.687715 | 0.657307 | 0.561599 | 0.612563 |
| 29.38908 | 32.09489 | 30.06595 | 32.8031 |
| 9.523209 | 7.537702 | 8.262858 | 8.053904 |
| 2.214064 | 1.675299 | 1.439736 | 1.207514 |
| 4.165193 | 3.109762 | 3.593468 | 3.684334 |
| 0.494184 | 0 | 0.071216 | 0 |
| 0 | 0 | 0.555486 | 0 |
| 511.9422 | 591.4719 | 575.0608 | 579.9762 |
| 10.19618 | 8.099663 | 7.595836 | 8.301909 |
| 7.836807 | 6.986597 | 6.764203 | 6.652367 |
| 0 | 0 | 0 | 0 |
| 0.146483 | 0.334702 | 0.17943 | 0.098377 |
| 3.698016 | 3.307733 | 2.605632 | 2.852323 |
| 39.64713 | 37.28511 | 36.89074 | 36.77213 |
| 0 | 0 | 0 | 0 |
| 1.992034 | 2.155768 | 2.015043 | 1.749264 |
| 3.158374 | 2.949523 | 3.239593 | 2.792319 |
| 0.632071 | 0.589021 | 0.619392 | 0.301864 |
| 0.380928 | 1.228788 | 0.466608 | 0.454809 |
| 8.823503 | 6.915112 | 7.011101 | 8.59984 |
| 10.65523 | 10.22659 | 9.774682 | 10.5061 |
| 7.308846 | 10.27102 | 9.750557 | 8.639983 |
| 0 | 0.384959 | 0 | 0.427452 |
| 8.165206 | 8.225188 | 7.264437 | 7.080731 |
| 4.191382 | 4.139979 | 4.208789 | 4.567871 |
| 5.839932 | 4.898475 | 4.393006 | 4.339778 |
| 1.541605 | 1.073952 | 1.569192 | 1.192499 |
| 4.056294 | 2.538817 | 3.040519 | 2.710637 |
| 9.539586 | 7.924254 | 8.876795 | 8.065718 |
| 0 | 0 | 0 | 0 |
| 2.441848 | 1.422208 | 2.617195 | 1.943627 |
| 0 | 0 | 0 | 0 |
| 0.139842 | 0.112774 | 0.042824 | 0.083482 |
| 266.0941 | 297.9474 | 293.3554 | 301.5244 |
| 0.174205 | 0.062438 | 0 | 0.069331 |

| | | | |
|---|---|---|---|
| 0.481578 | 0.712003 | 0.479291 | 0.431235 |
| 0.14046 | 0 | 0.172053 | 0 |
| 0.27723 | 0.346432 | 0.266161 | 0.322052 |
| 9.565723 | 8.137061 | 9.06137 | 8.273864 |
| 0 | 0 | 0 | 0 |
| 0 | 0 | 0 | 0 |
| 0 | 0.044024 | 0 | 0 |
| 0.187465 | 0 | 0 | 0 |
| 171.5528 | 165.9444 | 139.3517 | 168.3861 |
| 7.672824 | 5.703344 | 5.753251 | 5.341875 |
| 2.514314 | 2.703534 | 2.387477 | 1.954765 |
| 0 | 0 | 2.24331 | 0.72886 |
| 0.066225 | 0.071209 | 0.324484 | 0.158139 |
| 10.56878 | 9.591674 | 9.51909 | 9.030944 |
| 4.51742 | 4.463545 | 6.580376 | 6.341083 |
| 0.331434 | 0.237585 | 0 | 0.131905 |
| 11.48419 | 8.445877 | 8.858395 | 9.119458 |
| 2.40613 | 2.092049 | 2.087103 | 1.841887 |
| 0 | 0 | 0 | 0.078524 |
| 0 | 0 | 0 | 0 |
| 1.915249 | 0.760388 | 0.902321 | 0.703603 |
| 22.52148 | 21.80664 | 20.67339 | 22.13261 |
| 22.07774 | 20.21271 | 18.69861 | 19.03194 |
| 0 | 0.015449 | 0 | 0 |
| 18.22485 | 16.7347 | 15.83946 | 15.81883 |
| 0 | 0 | 0 | 0 |
| 2.816476 | 3.821034 | 3.045677 | 4.04578 |
| 4.02905 | 6.695317 | 2.24331 | 2.18658 |
| 1.681465 | 0.87661 | 2.995881 | 4.167255 |
| 6.333492 | 6.210275 | 5.989375 | 6.582345 |
| 1.108395 | 0.885804 | 0.935712 | 0.625916 |
| 0 | 0.133942 | 0.11444 | 0.297455 |
| 0.457847 | 0.201397 | 0.229429 | 0.347865 |
| 10.39162 | 10.63159 | 10.81662 | 9.261601 |
| 0 | 0.014516 | 0 | 0 |
| 5.792409 | 5.814453 | 6.0143 | 5.55957 |
| 5.324723 | 5.560684 | 5.583911 | 5.122544 |
| 45.68793 | 42.24559 | 40.69817 | 41.97105 |
| 0.044647 | 0.024004 | 0.027345 | 0 |
| 0.143476 | 0.173558 | 0.087874 | 0.17844 |
| 0.876503 | 0.541918 | 0.778397 | 0.6279 |
| 17.2226 | 16.11857 | 15.74454 | 15.94003 |
| 4.584576 | 4.753889 | 4.537089 | 4.422353 |
| 28.66431 | 24.03399 | 25.42869 | 25.52218 |
| 3.442124 | 3.084306 | 1.405447 | 2.054859 |
| 3.977159 | 4.181961 | 4.118084 | 3.174426 |
| 0.073143 | 0.104863 | 0.104527 | 0 |
| 0 | 0 | 0 | 0 |
| 0 | 0 | 0 | 0 |
| 2.394103 | 1.650177 | 2.356102 | 2.027778 |
| 1.287944 | 1.669991 | 1.252827 | 1.221145 |
| 39.98141 | 28.98424 | 29.55269 | 31.64391 |
| 82.55043 | 38.84986 | 41.6196 | 37.92453 |
| 0.594438 | 0.344171 | 0.504098 | 0.309369 |

| | | | |
|---|---|---|---|
| 0.036812 | 0 | 0 | 0.065927 |
| 0.018279 | 0 | 0 | 0 |
| 5.184796 | 3.977829 | 4.119557 | 5.086148 |
| 5.329018 | 4.966055 | 4.749639 | 4.581051 |
| 0.229199 | 0.123224 | 0.070188 | 0.068413 |
| 1.747378 | 1.40916 | 1.070203 | 1.043139 |
| 5.56705 | 2.03779 | 1.886166 | 3.252674 |
| 0 | 0 | 0 | 0 |
| 0.278456 | 0 | 0 | 0.332463 |
| 76.56412 | 59.91494 | 58.49903 | 56.3453 |
| 0.440889 | 0 | 0 | 0 |
| 30.63898 | 31.85557 | 31.47142 | 31.40593 |
| 0 | 0 | 0 | 0 |
| 0 | 0 | 0 | 0 |
| 0.269368 | 0.15596 | 0.329956 | 0.321612 |
| 0 | 0.019453 | 0 | 0 |
| 0.007086 | 0.007619 | 0.026038 | 0.00846 |
| 1.725219 | 1.855054 | 1.69061 | 2.059822 |
| 0.390295 | 0 | 0 | 0 |
| 0 | 1.528343 | 1.741076 | 0 |
| 0.113102 | 0.040538 | 0 | 0.135038 |
| 7.689651 | 8.214776 | 8.324738 | 8.447354 |
| 56.63504 | 51.92491 | 51.49743 | 51.00064 |
| 2.247968 | 0.489422 | 0.500653 | 0.798533 |
| 1.528455 | 1.442239 | 1.719406 | 1.526954 |
| 13.02398 | 11.91841 | 10.52245 | 10.83534 |
| 0.497795 | 0.42203 | 0.527678 | 0.400038 |
| 0.700236 | 0 | 0 | 0 |
| 5.51853 | 5.054292 | 4.784063 | 5.199543 |
| 7.875937 | 7.980632 | 7.031176 | 7.459016 |
| 12.83915 | 14.97395 | 12.87879 | 13.79031 |
| 7.066308 | 6.078478 | 4.394429 | 4.932286 |
| 4.487895 | 4.041239 | 3.896029 | 3.97867 |
| 34.63443 | 35.28943 | 36.12572 | 35.09178 |
| 1.556762 | 1.580404 | 1.821691 | 1.869077 |
| 5.100149 | 6.098554 | 6.193441 | 4.881949 |
| 2.990008 | 2.906324 | 3.062118 | 3.110089 |
| 4.991048 | 4.905849 | 4.82147 | 4.967189 |
| 5.053877 | 4.321184 | 5.519346 | 5.088972 |
| 0.366277 | 0.328202 | 0.373885 | 0.36443 |
| 0.417826 | 0.328313 | 0.511805 | 0.613984 |
| 0 | 0 | 0 | 0 |
| 11.19574 | 11.45041 | 11.11118 | 11.8687 |
| 2.977873 | 2.687088 | 2.76783 | 2.965833 |
| 18.55687 | 17.74886 | 16.86228 | 18.25929 |
| 4.086806 | 3.449067 | 3.783627 | 3.773051 |
| 61.4343 | 49.51924 | 48.2359 | 47.62911 |
| 0.418909 | 0.275266 | 0.484625 | 1.389323 |
| 0.433859 | 0.46651 | 0.265722 | 0.259003 |
| 4.991552 | 5.035521 | 4.156333 | 4.118188 |
| 34.52277 | 25.77025 | 27.19293 | 25.80206 |
| 14.14898 | 11.52191 | 12.43242 | 12.02792 |
| 0 | 0 | 0 | 0 |
| 0.22814 | 0.070088 | 0.099805 | 0.194562 |

| | | | |
|---|---|---|---|
| 7.810039 | 8.939593 | 9.566708 | 10.22718 |
| 0 | 0 | 0 | 0 |
| 13.09553 | 10.24948 | 12.43998 | 11.5404 |
| 0 | 0 | 0 | 0 |
| 0 | 0 | 0 | 0.118811 |
| 7.552054 | 6.425545 | 6.237854 | 7.093461 |
| 0 | 0 | 0 | 0 |
| 0.924359 | 0.919379 | 0.820896 | 0.924296 |
| 0 | 0 | 0 | 0 |
| 28.64491 | 19.46795 | 19.75709 | 21.36972 |
| 0 | 0 | 0 | 0 |
| 2.78669 | 3.387243 | 2.968247 | 2.459207 |
| 0.191229 | 0.411241 | 0.936965 | 0.456635 |
| 0 | 0 | 0 | 0 |
| 45.35617 | 40.49918 | 39.14305 | 41.04071 |
| 3.773213 | 5.52439 | 5.145135 | 5.525025 |
| 11.44596 | 9.688269 | 10.27109 | 10.4746 |
| 2.939346 | 1.847933 | 1.754293 | 2.125198 |
| 0.050387 | 0 | 0 | 0 |
| 18.86136 | 15.45934 | 21.01133 | 19.6302 |
| 1.142182 | 1.37876 | 1.187903 | 1.170728 |
| 11.18304 | 9.216597 | 7.908951 | 9.37316 |
| 27.5802 | 26.21718 | 24.49911 | 26.03126 |
| 52.65976 | 55.51973 | 57.59306 | 62.97508 |
| 2.896353 | 2.242313 | 2.270601 | 1.79821 |
| 1.304317 | 1.657472 | 1.452445 | 1.395491 |
| 0 | 0 | 0.120758 | 0 |
| 0.816808 | 0.418508 | 0.584201 | 0.405799 |
| 1.543323 | 0.951754 | 1.41784 | 1.463279 |
| 0.073995 | 0 | 0 | 0.044173 |
| 0 | 0 | 0 | 0 |
| 3.034426 | 2.19581 | 1.955359 | 1.905911 |
| 80.75707 | 79.93637 | 80.71663 | 84.87248 |
| 11.90401 | 10.3618 | 12.49844 | 18.27356 |
| 1.230389 | 0.661492 | 1.80856 | 0.73451 |
| 0 | 0 | 0 | 0 |
| 0.081395 | 0.175041 | 0.498513 | 0.291544 |
| 29788.22 | 29643.98 | 29856.8 | 29136.68 |
| 0.158018 | 0.056637 | 0.06452 | 0.094333 |
| 266.6499 | 228.691 | 222.3797 | 217.6584 |
| 0 | 0 | 0 | 0 |
| 0 | 0 | 0 | 0.035007 |
| 11.52191 | 12.46486 | 12.15486 | 12.43705 |
| 12.20399 | 9.440594 | 8.980134 | 9.364531 |
| 0.069989 | 0.075256 | 0.057154 | 0.111418 |
| 0 | 0 | 0 | 0 |
| 0.046774 | 0 | 0 | 0 |
| 487.6754 | 425.4577 | 421.7012 | 413.3408 |
| 0.253277 | 0 | 0 | 0 |
| 246.7168 | 216.7144 | 220.01 | 213.8075 |
| 1.630221 | 0.438227 | 0.499224 | 0.4055 |
| 14.54628 | 13.8591 | 13.75827 | 13.14654 |
| 0 | 0.106003 | 0.120758 | 0.117704 |
| 9.972115 | 7.343765 | 7.364401 | 7.886414 |

| | | | |
|---|---|---|---|
| 0.701247 | 0.931437 | 0.993716 | 0.738752 |
| 171.3701 | 142.6193 | 139.9992 | 154.0953 |
| 1.117832 | 0.814229 | 0.927563 | 0.947159 |
| 15.69019 | 14.95618 | 13.46137 | 12.85279 |
| 6.139443 | 6.297263 | 6.134113 | 5.708754 |
| 0.008121 | 0.017465 | 0.009948 | 0.019393 |
| 1.72827 | 2.072757 | 1.411333 | 1.58728 |
| 3.78243 | 2.605751 | 2.547252 | 2.580586 |
| 18.39746 | 16.55631 | 15.27989 | 15.42344 |
| 9.679648 | 5.887415 | 5.74877 | 4.669494 |
| 0.088919 | 0.430248 | 0.272297 | 0.371576 |
| 4.848246 | 4.003986 | 3.929049 | 4.181845 |
| 35.81761 | 32.35444 | 36.34063 | 34.6653 |
| 18.11578 | 17.58464 | 14.99655 | 15.96577 |
| 8.762479 | 6.149997 | 5.867118 | 6.492461 |
| 40.44076 | 49.14137 | 46.17029 | 46.78407 |
| 0.031224 | 0.033573 | 0.076493 | 0.037279 |
| 35.23824 | 38.84538 | 32.86282 | 38.18357 |
| 0.960664 | 0.937819 | 0.743204 | 0.558399 |
| 1.284877 | 1.584746 | 6.110349 | 4.286392 |
| 0.220445 | 0.592587 | 0.270028 | 0.263199 |
| 0.390295 | 0.23981 | 0 | 0 |
| 15.74504 | 13.15258 | 13.27242 | 11.01648 |
| 20.18674 | 19.91643 | 18.989 | 18.95036 |
| 20.99379 | 18.59792 | 16.75805 | 18.88496 |
| 12.90899 | 14.97631 | 10.6804 | 13.65508 |
| 377.933 | 365.2545 | 354.2897 | 364.3526 |
| 0 | 0 | 0 | 0.022632 |
| 47.02159 | 48.45095 | 50.09779 | 49.89552 |
| 0.695125 | 0 | 0.425738 | 0 |
| 6.16504 | 6.708678 | 6.644052 | 5.485166 |
| 0.400696 | 0 | 0 | 0 |
| 5.268894 | 4.822421 | 5.197092 | 4.363631 |
| 26.14609 | 14.97598 | 13.91942 | 13.98765 |
| 6.724817 | 5.189925 | 6.111614 | 6.669319 |
| 6.847557 | 4.334448 | 4.916208 | 5.023072 |
| 97.30557 | 89.26069 | 84.35962 | 82.84521 |
| 0.504764 | 0.422139 | 0.274799 | 0.28459 |
| 10.45495 | 8.329382 | 8.692507 | 9.960259 |
| 2.102527 | 2.31775 | 2.618721 | 3.079873 |
| 0 | 0 | 0 | 0 |
| 13.9568 | 11.66454 | 12.26397 | 13.56645 |
| 0.259292 | 1.347559 | 0.167629 | 0.16339 |
| 1.75059 | 2.917618 | 2.251557 | 2.090114 |
| 0 | 0.107675 | 0 | 0 |
| 0 | 0 | 0 | 0 |
| 2.762706 | 2.03866 | 2.123361 | 2.457726 |
| 11.38645 | 7.272369 | 7.23594 | 7.582623 |
| 2.048597 | 1.905097 | 1.627704 | 1.983177 |
| 3.087157 | 2.381371 | 2.055182 | 2.323723 |
| 0 | 0 | 0 | 0.181343 |
| 2.930218 | 2.363053 | 1.345986 | 1.311948 |
| 13.75396 | 13.75121 | 13.00513 | 13.9887 |
| 11.95657 | 11.18929 | 11.68453 | 11.82829 |

| | | | |
|---|---|---|---|
| 0.088015 | 0.063092 | 0.107812 | 0 |
| 2.892131 | 2.388314 | 2.097244 | 2.099457 |
| 19.09798 | 24.18168 | 25.60419 | 24.67257 |
| 0 | 0 | 0.047887 | 0 |
| 0.200912 | 0.108016 | 0 | 0 |
| 2.129173 | 1.665024 | 2.845174 | 2.31102 |
| 0 | 0 | 0 | 0 |
| 1.279302 | 0.722761 | 0.956165 | 1.00965 |
| 0.232273 | 0.249754 | 0 | 0 |
| 63.5716 | 56.10985 | 58.42839 | 55.22202 |
| 0 | 0 | 0 | 0 |
| 20.37856 | 19.26908 | 17.87177 | 18.98192 |
| 0 | 0.538942 | 0 | 0 |
| 3.80593 | 4.038269 | 3.285975 | 3.423076 |
| 0 | 0 | 0 | 0 |
| 33.52449 | 31.53069 | 31.51383 | 31.79046 |
| 1.069391 | 0.625368 | 0.781358 | 0.739199 |
| 1.133715 | 0.677242 | 0.771509 | 0.902398 |
| 8.454024 | 6.282966 | 6.125336 | 7.042476 |
| 0.116184 | 0.104106 | 0.071158 | 0.254316 |
| 455.5371 | 455.7034 | 450.5985 | 458.2576 |
| 4.920656 | 4.783251 | 4.840211 | 5.370588 |
| 0.874206 | 0.583707 | 0.647684 | 0.639722 |
| 2.296491 | 2.382978 | 2.813027 | 2.320061 |
| 5.212885 | 4.145989 | 4.234939 | 3.922095 |
| 0 | 0 | 0 | 0 |
| 3.156459 | 2.973043 | 2.937284 | 2.775361 |
| 2.357767 | 2.098101 | 1.825802 | 2.070842 |
| 0 | 0.119346 | 0 | 0 |
| 25.18777 | 22.07491 | 20.83245 | 21.12346 |
| 10.76839 | 8.748734 | 9.819202 | 8.996637 |
| 0.126976 | 0.091021 | 0.051845 | 0.303206 |
| 1.336261 | 1.072558 | 1.429334 | 0.943773 |
| 0.242939 | 0.261222 | 0.099194 | 0.338399 |
| 0.076974 | 0.430387 | 0.396006 | 0.349231 |
| 0.403526 | 0 | 1.482866 | 0 |
| 0 | 0 | 0 | 0 |
| 3.435058 | 2.192119 | 2.360409 | 1.533812 |
| 0 | 0 | 0.138213 | 0 |
| 27.5242 | 28.71738 | 26.46182 | 28.42554 |
| 0.398167 | 0.327395 | 0.358621 | 0.321588 |
| 7.132768 | 9.051461 | 7.79255 | 8.516155 |
| 0.604923 | 0.402658 | 0.652772 | 0.584675 |
| 0.369117 | 0 | 0 | 0 |
| 5.517224 | 5.74615 | 5.501226 | 6.173586 |
| 1.621335 | 0.611702 | 0.871058 | 0.882992 |
| 14.03993 | 11.29314 | 10.9653 | 10.91541 |
| 0 | 0 | 0.027486 | 0.21433 |
| 10.63723 | 9.054056 | 9.606914 | 8.429797 |
| 7.087396 | 6.162646 | 5.766789 | 6.842904 |
| 0.03932 | 0.126836 | 0.096327 | 0 |
| 6.836682 | 5.798122 | 5.956453 | 6.294433 |
| 0.610462 | 0 | 0 | 0 |
| 0 | 0 | 0 | 0 |

| | | | |
|---|---|---|---|
| 0.123039 | 0 | 0.150713 | 0 |
| 14.72464 | 12.74847 | 13.70311 | 13.5849 |
| 11.96755 | 12.50384 | 11.98028 | 11.70491 |
| 37.55006 | 35.67163 | 34.97549 | 34.81761 |
| 0.013984 | 0.015037 | 0 | 0.016696 |
| 397.7848 | 306.9926 | 302.4831 | 302.8759 |
| 5.648942 | 5.213941 | 5.757855 | 5.799323 |
| 1.364389 | 1.014004 | 0.969058 | 1.252558 |
| 0 | 0 | 0 | 0 |
| 10.46035 | 8.621951 | 8.83177 | 9.435783 |
| 0.230666 | 0.425186 | 0.484369 | 0.35409 |
| 0 | 0 | 0 | 0.281441 |
| 0 | 0 | 0 | 0.182215 |
| 13.70727 | 13.43715 | 13.68562 | 12.70718 |
| 4.409718 | 4.502106 | 3.491925 | 3.509983 |
| 55.35983 | 58.88827 | 57.63987 | 57.99204 |
| 13.8231 | 14.10194 | 15.26679 | 14.30578 |
| 28.1028 | 32.52673 | 34.42381 | 30.98942 |
| 2.459475 | 2.513161 | 2.208045 | 2.206924 |
| 0 | 0 | 0 | 0 |
| 0.453486 | 0.162538 | 0 | 0.360959 |
| 0.048538 | 0.052191 | 0 | 0 |
| 11.03683 | 9.437433 | 9.141612 | 8.596688 |
| 10.29736 | 8.28432 | 9.119827 | 8.51329 |
| 78.44515 | 76.47843 | 76.03828 | 78.55614 |
| 0 | 0 | 0 | 0.32673 |
| 0.123946 | 2.532209 | 2.277373 | 0.147985 |
| 5.052506 | 4.47241 | 4.032187 | 4.478623 |
| 2.116981 | 2.121097 | 2.043082 | 1.608451 |
| 7.99767 | 8.38075 | 8.051746 | 7.958563 |
| 5.660919 | 4.108686 | 4.144759 | 4.193555 |
| 1.023621 | 0.967818 | 1.037676 | 0.863934 |
| 5.844072 | 4.346238 | 4.997187 | 4.66164 |
| 2.097039 | 1.978238 | 2.253594 | 1.945298 |
| 0 | 0 | 0.120758 | 0 |
| 0 | 0.09429 | 0.107414 | 0.418793 |
| 0 | 0 | 0 | 0 |
| 8.813297 | 6.441826 | 7.190012 | 6.677419 |
| 0.414708 | 0.364842 | 0.323264 | 0.135038 |
| 42.13299 | 45.21513 | 43.17643 | 44.25033 |
| 21.71715 | 24.43067 | 24.49603 | 23.44386 |
| 6.428873 | 4.937636 | 5.408574 | 6.747904 |
| 0.179008 | 0.384959 | 0 | 0 |
| 0.012323 | 0.066252 | 0.045284 | 0.029426 |
| 3.916471 | 4.580618 | 4.488778 | 4.074465 |
| 2.332394 | 2.247667 | 2.129279 | 2.548316 |
| 0 | 0 | 0 | 0 |
| 0 | 0 | 0 | 0 |
| 1.739817 | 1.312807 | 1.102961 | 1.767486 |
| 27.44438 | 16.68098 | 16.459 | 17.58383 |
| 1.940466 | 1.712 | 1.249409 | 2.079193 |
| 10.22577 | 7.791656 | 7.411848 | 7.444001 |
| 100.3585 | 125.6136 | 113.5124 | 119.5874 |
| 2.164366 | 2.327249 | 2.651185 | 2.15345 |

| | | | |
|---|---|---|---|
| 14.60769 | 11.22411 | 13.09361 | 12.61278 |
| 10.43539 | 9.965264 | 9.981727 | 9.424357 |
| 4.00821 | 3.232392 | 3.727776 | 4.519728 |
| 949.5263 | 885.0692 | 814.9849 | 872.7861 |
| 5.185109 | 4.951996 | 4.457792 | 4.806484 |
| 5.290672 | 3.843805 | 4.553987 | 3.073032 |
| 26.74846 | 25.70173 | 26.19195 | 23.68526 |
| 3.602896 | 2.750089 | 2.833214 | 3.876814 |
| 0 | 0 | 0 | 0.016369 |
| 9.547849 | 8.610522 | 7.796929 | 8.899071 |
| 4.338592 | 3.829271 | 3.626003 | 3.901228 |
| 0 | 0 | 0 | 0 |
| 9.52085 | 9.983646 | 9.870342 | 9.902457 |
| 10.16398 | 8.612443 | 8.931601 | 9.760976 |
| 4.109072 | 3.220387 | 3.845869 | 3.610415 |
| 0 | 0.483014 | 0 | 0 |
| 42.88877 | 43.21998 | 39.82217 | 44.17547 |
| 0.176119 | 0.086078 | 0.117672 | 0.114696 |
| 6.246406 | 6.055853 | 5.76989 | 4.890416 |
| 0.038618 | 0 | 0 | 0 |
| 8.316501 | 8.341208 | 7.904265 | 8.510975 |
| 5.211198 | 3.955325 | 4.631039 | 3.903937 |
| 0.228777 | 0.114797 | 0.186823 | 0.127469 |
| 12.39069 | 10.2399 | 10.08622 | 11.33881 |
| 17.67752 | 15.72518 | 17.914 | 17.43159 |
| 1.112524 | 0.71775 | 0.363402 | 1.41685 |
| 4.115207 | 2.838618 | 3.447729 | 2.503024 |
| 5.96017 | 3.810113 | 3.680382 | 4.230688 |
| 4.266236 | 4.0003 | 3.789336 | 3.765932 |
| 17.29461 | 13.2864 | 15.02628 | 16.00688 |
| 8.772896 | 7.726468 | 7.352618 | 8.131428 |
| 1.186805 | 0.425373 | 0.352423 | 0.38645 |
| 0.529067 | 0.796436 | 0.38884 | 0.379007 |
| 0 | 0 | 0 | 0 |
| 24.01301 | 21.34886 | 22.45517 | 23.42572 |
| 7.797589 | 2.989117 | 2.910651 | 3.043627 |
| 2.50446 | 5.4532 | 4.908439 | 5.457106 |
| 0 | 0 | 0.063123 | 0 |
| 5.95787 | 5.67482 | 5.200501 | 4.312841 |
| 1.654189 | 1.558181 | 1.590863 | 1.46902 |
| 52.43989 | 52.56194 | 51.62755 | 53.66615 |
| 0.485878 | 0 | 0 | 1.160226 |
| 0.313263 | 0.08421 | 0.191862 | 0 |
| 0.226743 | 0 | 0.555486 | 0.270719 |
| 0.080267 | 0.134256 | 0.021849 | 0.042593 |
| 0 | 0 | 0 | 0 |
| 30.37122 | 27.00363 | 24.0646 | 26.0088 |
| 0 | 0.055712 | 0 | 0 |
| 180.039 | 185.5273 | 182.2241 | 193.3496 |
| 0.018564 | 0.039922 | 0.01137 | 0.011082 |
| 34.49183 | 40.21953 | 40.08711 | 39.65075 |
| 13.83694 | 12.36898 | 11.05885 | 12.35526 |
| 10.27649 | 7.338892 | 7.069825 | 7.337409 |
| 0.02471 | 0.079709 | 0.211875 | 0.147512 |

| | | | |
|---|---|---|---|
| 0.642968 | 0.521778 | 0.713287 | 0.695249 |
| 23.4108 | 21.88292 | 22.44425 | 20.94832 |
| 0.438307 | 0.342758 | 0.244042 | 0.333019 |
| 0 | 0 | 0 | 0 |
| 36.26304 | 33.41297 | 32.14401 | 31.74231 |
| 5.539009 | 4.17955 | 4.642278 | 5.14367 |
| 23.30708 | 19.90146 | 20.3463 | 20.14656 |
| 5.698583 | 5.965418 | 5.621182 | 5.806138 |
| 8.101725 | 7.662104 | 7.848982 | 7.430653 |
| 3.065796 | 2.579284 | 2.639757 | 3.078413 |
| 9.371059 | 7.78961 | 7.836108 | 7.059939 |
| 14.88063 | 10.54386 | 10.64081 | 11.12177 |
| 8.277416 | 5.573956 | 6.324204 | 6.463754 |
| 18.52908 | 17.59628 | 17.49135 | 17.03327 |
| 7.092567 | 3.050533 | 1.972379 | 12.95399 |
| 13.62264 | 9.348526 | 10.52593 | 9.957993 |
| 5.647027 | 4.696812 | 5.157759 | 5.191773 |
| 1.920002 | 1.238697 | 1.411114 | 0.458477 |
| 0.377905 | 0.325076 | 0.138872 | 0.18048 |
| 4.499798 | 4.574525 | 3.607798 | 3.41888 |
| 0 | 0 | 0 | 0 |
| 11.98983 | 10.80043 | 10.45041 | 11.78009 |
| 1.024736 | 0.587656 | 0.502089 | 0.570958 |
| 1.489952 | 1.241403 | 1.309088 | 1.024512 |
| 44.3074 | 33.76165 | 33.08794 | 33.1889 |
| 9.296349 | 8.166464 | 8.789686 | 9.303441 |
| 20.65804 | 19.58709 | 17.64737 | 19.00867 |
| 0 | 0.312192 | 0.355647 | 0 |
| 13.54131 | 12.36612 | 11.95587 | 12.48457 |
| 5.22725 | 4.167024 | 4.379054 | 5.021547 |
| 7.761477 | 2.67147 | 19.81929 | 3.481179 |
| 0.22355 | 0.160249 | 0.182554 | 0.266907 |
| 23.80802 | 23.35576 | 22.22442 | 21.40815 |
| 4.68435 | 4.113891 | 3.755219 | 3.821307 |
| 8.494881 | 4.56709 | 1.369156 | 4.537411 |
| 0.752228 | 0.323535 | 0.552854 | 0.449061 |
| 0 | 0 | 0 | 0.437316 |
| 0.065407 | 0 | 0 | 0.117138 |
| 4.745608 | 6.450103 | 6.237557 | 5.825167 |
| 21.16788 | 20.44965 | 19.63281 | 22.81854 |
| 0 | 0 | 0 | 0 |
| 3.033318 | 2.958193 | 1.900997 | 2.947834 |
| 1.374741 | 2.15011 | 1.837041 | 2.089016 |
| 0 | 0 | 0 | 0 |
| 0.520321 | 0.530788 | 0.637354 | 0.446016 |
| 36.3578 | 33.22464 | 27.10341 | 28.51283 |
| 0.069009 | 0 | 0 | 0 |
| 0 | 0 | 0 | 0 |
| 0.393521 | 0.317352 | 0.361525 | 0.117461 |
| 1.778852 | 1.571165 | 1.206209 | 0.94815 |
| 0.420943 | 0.399372 | 0.758269 | 0.679966 |
| 0.105113 | 0 | 0 | 0.125499 |
| 0.021176 | 0.031877 | 0.020751 | 0.010113 |
| 3.72416 | 3.667922 | 3.411777 | 3.54969 |

| | | | |
|---|---|---|---|
| 0.206041 | 0.144005 | 0.214527 | 0.209102 |
| 18.89291 | 17.12469 | 17.60991 | 18.2572 |
| 28.46863 | 30.11335 | 28.53157 | 29.49323 |
| 2.946921 | 2.835151 | 2.707317 | 3.333289 |
| 0.507208 | 0.396639 | 0.470675 | 0.660632 |
| 23.94381 | 17.71541 | 22.06632 | 19.88707 |
| 3.322834 | 3.609174 | 2.809899 | 3.202027 |
| 2.479243 | 1.740626 | 1.589899 | 1.549693 |
| 1.259078 | 0.984606 | 0.140207 | 1.09329 |
| 1.835799 | 2.035642 | 1.756809 | 2.191849 |
| 1.683736 | 0.33795 | 0.247494 | 0.964941 |
| 0.095232 | 0.153598 | 0.116652 | 0.056851 |
| 4.852003 | 4.860083 | 4.542246 | 5.000076 |
| 4.028919 | 4.112776 | 3.706547 | 2.96332 |
| 0.17603 | 0.075711 | 0.043125 | 0.042034 |
| 0.290342 | 0 | 0 | 0 |
| 0.044212 | 0.047539 | 0 | 0 |
| 5.089701 | 5.020444 | 4.84333 | 4.695739 |
| 15.45511 | 14.58177 | 14.88245 | 14.12211 |
| 251.4297 | 285.0714 | 272.0494 | 269.332 |
| 3.197496 | 3.008364 | 3.23127 | 3.69039 |
| 0.051869 | 0 | 0 | 0.123859 |
| 7.790853 | 6.146663 | 6.071186 | 6.636093 |
| 174.8778 | 146.457 | 147.685 | 155.4612 |
| 0.059744 | 0.03212 | 0 | 0 |
| 0 | 0 | 0 | 0 |
| 8.20113 | 7.716032 | 7.620925 | 8.694376 |
| 0.241094 | 0.324047 | 0.590644 | 0.287854 |
| 7.717529 | 6.372639 | 6.807345 | 5.863664 |
| 5.956309 | 4.701616 | 5.356045 | 4.480671 |
| 16.51703 | 16.38532 | 15.2799 | 16.46124 |
| 0.333446 | 0.250978 | 0.122534 | 0.23887 |
| 0 | 0 | 0 | 0 |
| 1.785602 | 2.377119 | 1.041537 | 1.319757 |
| 34.37678 | 26.90496 | 23.11631 | 27.8265 |
| 1.012246 | 0.217685 | 0.49597 | 0.483428 |
| 4.679399 | 2.991736 | 3.872912 | 3.472975 |
| 0.151643 | 0 | 0 | 0 |
| 6.179564 | 5.369571 | 5.441856 | 5.56347 |
| 10.22317 | 13.02269 | 10.20988 | 10.00667 |
| 44.02605 | 37.97458 | 37.18008 | 39.61317 |
| 9.448892 | 6.193026 | 6.53486 | 7.161843 |
| 12.63511 | 11.95375 | 13.23479 | 13.21994 |
| 0.077868 | 0.031398 | 0.035768 | 0.139455 |
| 19.20094 | 18.4152 | 18.94545 | 18.41511 |
| 34.29561 | 32.40474 | 32.89147 | 31.98773 |
| 0 | 0.787684 | 0 | 0 |
| 14.0999 | 14.33254 | 23.59468 | 13.61482 |
| 0 | 0 | 0 | 0 |
| 15.28143 | 15.00264 | 14.61058 | 15.71432 |
| 20.90928 | 22.94122 | 22.63594 | 24.48109 |
| 9.672788 | 6.942758 | 7.451078 | 7.887716 |
| 5.204864 | 4.834433 | 5.105636 | 4.93863 |
| 0 | 0 | 0 | 0 |

| | | | |
|---|---|---|---|
| 17.56617 | 5.607417 | 4.741673 | 5.570977 |
| 0.482921 | 0.519265 | 0.502811 | 0.461266 |
| 9.751626 | 8.596224 | 9.272624 | 8.129076 |
| 10.3065 | 8.921123 | 9.563201 | 8.244638 |
| 3.049645 | 2.012207 | 1.867792 | 2.096401 |
| 9.831982 | 11.12526 | 9.820542 | 10.54235 |
| 0.378573 | 0.271375 | 0.334911 | 0.326442 |
| 0.763427 | 1.00851 | 1.015296 | 0.703151 |
| 0 | 0 | 0 | 0 |
| 0 | 0 | 0.110466 | 0 |
| 0.206003 | 0.537943 | 0.360482 | 0.808143 |
| 0 | 0 | 0 | 0.025256 |
| 14.00383 | 16.46223 | 15.92571 | 15.92193 |
| 10.71614 | 8.5876 | 9.241151 | 8.7057 |
| 12.05097 | 14.53813 | 13.09227 | 12.50596 |
| 54.34045 | 55.01013 | 54.46734 | 48.85866 |
| 36.63372 | 39.33483 | 37.08189 | 36.88811 |
| 0 | 0.136169 | 0.155122 | 0.1512 |
| 6.992756 | 5.646572 | 5.66596 | 5.58765 |
| 0.89588 | 0.272935 | 0.292636 | 0.267409 |
| 1.275986 | 0.635811 | 0.533703 | 0.854624 |
| 0.052268 | 0.056201 | 0.192073 | 0.062405 |
| 6.926464 | 3.826088 | 3.89284 | 4.670027 |
| 0.095458 | 0.261269 | 0.180708 | 0.217582 |
| 0.712951 | 0.701363 | 0.650338 | 0.90556 |
| 0 | 0 | 0 | 0.019706 |
| 3.645187 | 2.785995 | 2.5048 | 2.547608 |
| 1.762638 | 1.147851 | 1.155574 | 0.889225 |
| 36.20565 | 35.2206 | 37.34803 | 36.478 |
| 0 | 0.203173 | 0 | 0 |
| 15.16909 | 18.66884 | 17.73718 | 18.49717 |
| 19.35021 | 13.98932 | 13.50964 | 14.42961 |
| 13.92812 | 7.79255 | 10.12557 | 9.734312 |
| 0.750703 | 0.634228 | 0.394095 | 0.576193 |
| 43.43512 | 46.45417 | 44.66923 | 47.97677 |
| 0.675788 | 0.726646 | 0.678788 | 0.661622 |
| 2.095731 | 1.217702 | 1.179915 | 0.979119 |
| 12.6452 | 10.69876 | 10.04199 | 9.653958 |
| 0 | 0 | 0 | 0 |
| 5.028479 | 3.836186 | 4.679849 | 4.192558 |
| 3.052311 | 3.063217 | 3.240337 | 3.036917 |
| 0 | 0 | 0 | 0.055195 |
| 0.164436 | 0.176811 | 0.230197 | 0.196328 |
| 6.020207 | 5.28004 | 5.312667 | 5.509554 |
| 0.044028 | 0.094682 | 0.080896 | 0.105134 |
| 0 | 0 | 0 | 0 |
| 2.074929 | 1.077074 | 0.744961 | 1.025114 |
| 470.6921 | 458.6635 | 448.7072 | 465.0793 |
| 6.467468 | 5.456365 | 5.7825 | 5.121482 |
| 3.478317 | 3.315075 | 2.743617 | 3.618082 |
| 0.44501 | 0.4785 | 0 | 0.531319 |
| 14.45487 | 8.838007 | 9.54742 | 7.613985 |
| 0 | 0 | 0 | 0 |
| 0.115854 | 0 | 0 | 0 |

| | | | |
|---|---|---|---|
| 0.849714 | 0.413733 | 0.510599 | 0.497686 |
| 59.00023 | 48.46065 | 46.86461 | 50.39638 |
| 39.97396 | 5.688832 | 5.940617 | 7.194119 |
| 29.4026 | 22.05722 | 25.965 | 23.89824 |
| 4.211685 | 3.402349 | 3.234397 | 3.803968 |
| 1.886289 | 0.566447 | 0.791003 | 0.872447 |
| 11.38874 | 11.02408 | 10.90781 | 11.73618 |
| 18.2213 | 16.01909 | 18.81033 | 17.0348 |
| 9.590257 | 8.960232 | 8.60885 | 8.305376 |
| 0.777758 | 0.820802 | 0.511632 | 0.877013 |
| 3.949514 | 2.76754 | 3.261476 | 2.450478 |
| 1.792002 | 0.825798 | 1.567905 | 1.37543 |
| 8.562937 | 12.23263 | 11.61277 | 10.88094 |
| 9.127676 | 8.887664 | 9.296557 | 9.444909 |
| 0 | 0 | 0 | 0 |
| 2.741807 | 3.852245 | 2.597245 | 2.684332 |
| 0 | 0 | 0 | 0 |
| 5.523996 | 4.314563 | 5.078955 | 4.183985 |
| 0.327446 | 0.234725 | 0.334247 | 0.293215 |
| 0.1536 | 0.110106 | 0 | 0.305651 |
| 17.49161 | 15.04638 | 16.78362 | 15.77908 |
| 0 | 0.047429 | 0.054031 | 0.026332 |
| 0 | 0 | 0 | 0.531319 |
| 17.857 | 14.66109 | 14.87821 | 14.68065 |
| 1.20287 | 1.104117 | 1.078116 | 1.120909 |
| 1.737812 | 1.744021 | 1.738429 | 1.763629 |
| 0 | 0 | 0 | 0 |
| 0.072807 | 0.078287 | 0.089184 | 0.173857 |
| 1.891134 | 2.251325 | 1.985568 | 1.774076 |
| 0.233794 | 0.188541 | 0.19092 | 0.116307 |
| 0 | 0.011194 | 0 | 0 |
| 8.382115 | 7.384541 | 7.204476 | 7.842254 |
| 1.381716 | 0.971419 | 1.269373 | 1.173823 |
| 0.351572 | 0.283523 | 0.296071 | 0.288584 |
| 0.624882 | 2.418873 | 1.377781 | 0 |
| 0.402673 | 0.432977 | 0.472692 | 0.260417 |
| 0.081815 | 0.029324 | 0 | 0.065122 |
| 0 | 0 | 0 | 0 |
| 0.009529 | 0.010246 | 0 | 0.022754 |
| 2421.179 | 2649.221 | 2543.796 | 2709.107 |
| 2.024319 | 1.793351 | 2.074162 | 1.793697 |
| 7.257082 | 1.509115 | 1.132138 | 2.002662 |
| 10.39011 | 9.66416 | 9.10938 | 8.777544 |
| 6.035488 | 5.744981 | 5.453865 | 4.252757 |
| 2.441848 | 2.230498 | 2.090852 | 1.896452 |
| 6.182457 | 5.485659 | 5.256354 | 6.186066 |
| 3.852431 | 1.822635 | 1.572979 | 1.809177 |
| 0 | 0 | 0.02053 | 0 |
| 0.425143 | 0.822849 | 0.729076 | 0.20304 |
| 5.934703 | 4.037169 | 4.408365 | 3.573851 |
| 0 | 0.832512 | 3.319369 | 0.924408 |
| 1.735437 | 1.574472 | 1.616478 | 1.251846 |
| 59.60611 | 58.26255 | 57.48279 | 58.35798 |
| 22.42344 | 14.56099 | 12.78209 | 13.99101 |

| | | | |
|---|---|---|---|
| 1.414338 | 1.013851 | 0.577486 | 0.562882 |
| 1.707458 | 1.510222 | 1.248157 | 1.512522 |
| 0 | 0.363117 | 0.20683 | 0 |
| 0.185793 | 0.028539 | 0.032512 | 0.03169 |
| 0.607864 | 0.21787 | 0.744588 | 0.362879 |
| 0 | 0 | 0.595164 | 0 |
| 10.31995 | 8.061052 | 8.875705 | 8.276739 |
| 1.638473 | 1.510097 | 1.269739 | 1.916329 |
| 0 | 0 | 0.251405 | 0 |
| 0 | 0.04256 | 0 | 0 |
| 0 | 0.060591 | 0 | 0 |
| 0 | 0.100391 | 0 | 0 |
| 0.634368 | 0.392729 | 0.847694 | 0.57379 |
| 0.318502 | 0.256854 | 0.097535 | 0.285206 |
| 0 | 0 | 0.144015 | 0 |
| 0 | 0 | 0 | 0.366781 |
| 0 | 0 | 0 | 0.040206 |
| 6.463717 | 5.009904 | 5.014458 | 5.144895 |
| 2.427307 | 1.951386 | 2.000703 | 1.950109 |
| 24.55051 | 26.43282 | 24.25969 | 24.16653 |
| 0.406975 | 0 | 0 | 0 |
| 0.496 | 0 | 0 | 0.592199 |
| 0 | 0.307504 | 0.350307 | 0 |
| 1.827084 | 0.781825 | 0.776463 | 0.868125 |
| 523.5215 | 519.9798 | 508.85 | 536.1278 |
| 27.78319 | 25.28783 | 24.25427 | 25.01345 |
| 4.081375 | 3.318155 | 3.549693 | 3.631603 |
| 0.068512 | 0.036834 | 0.041961 | 0 |
| 6.994659 | 5.121289 | 5.085776 | 5.656825 |
| 8.506943 | 4.174538 | 5.874567 | 5.112508 |
| 686.5107 | 657.3967 | 579.4038 | 875.1242 |
| 0 | 0.396895 | 0 | 0 |
| 0.793601 | 0.284442 | 0.324034 | 0.15792 |
| 13.94268 | 15.08427 | 14.68291 | 15.39503 |
| 13.11134 | 11.2105 | 10.36601 | 10.10387 |
| 15.68529 | 16.93658 | 17.92164 | 16.99631 |
| 0 | 0.031353 | 0 | 0 |
| 0 | 0.080376 | 0 | 0 |
| 2.801714 | 2.674705 | 2.549861 | 2.516641 |
| 6.0159 | 5.242557 | 5.394318 | 5.492632 |
| 187.1411 | 202.7911 | 206.1207 | 201.0713 |
| 0 | 0 | 0.053906 | 0 |
| 0 | 2.226065 | 0 | 0.617947 |
| 1.918681 | 1.83664 | 2.206932 | 1.815882 |
| 2.569211 | 1.589685 | 1.512811 | 1.732871 |
| 66.56154 | 62.49494 | 64.95658 | 62.98531 |
| 14.96336 | 13.51683 | 12.65973 | 12.17569 |
| 0.341334 | 0.152926 | 0.243896 | 0.067922 |
| 0 | 0 | 0 | 0 |
| 0.067397 | 0.108704 | 0.082556 | 0.241406 |
| 0 | 0 | 0 | 0 |
| 12.23792 | 11.07647 | 12.0456 | 11.58152 |
| 0 | 2.730639 | 0 | 0 |
| 12.04866 | 13.54096 | 11.25664 | 12.51618 |

| | | | |
|---|---|---|---|
| 0 | 0 | 0 | 0 |
| 0.165333 | 0.88888 | 0.405042 | 0.1974 |
| 1.709294 | 4.332264 | 2.841526 | 2.623896 |
| 0.046364 | 0.14956 | 0.113585 | 0.055356 |
| 3.216404 | 2.674068 | 2.701033 | 2.850472 |
| 0.129304 | 0.069517 | 0.158387 | 0.038595 |
| 0.020015 | 0 | 0 | 0 |
| 0.018593 | 0 | 0 | 0.044398 |
| 41.79008 | 37.34551 | 37.87763 | 37.05353 |
| 0.5798 | 0.612301 | 0.507293 | 0.469742 |
| 0.51661 | 0.248197 | 0.376992 | 0.472447 |
| 0.328387 | 0.3531 | 0 | 0.392076 |
| 0 | 0.1177 | 0.033521 | 0 |
| 0 | 0 | 0 | 0.045885 |
| 0.75448 | 0.67605 | 0.410747 | 0.900809 |
| 0.248 | 0 | 0 | 0.296099 |
| 0.471446 | 0 | 0 | 0 |
| 0 | 0 | 0 | 0 |
| 2.428313 | 1.759628 | 1.357924 | 2.269001 |
| 0.046455 | 0 | 0.056903 | 0.055464 |
| 2.426828 | 2.72944 | 2.494319 | 2.164804 |
| 0.029034 | 0 | 0 | 0.034665 |
| 1.299904 | 0.587452 | 0.530761 | 0.629804 |
| 218.4158 | 186.8253 | 185.3014 | 206.9447 |
| 0.094872 | 0.081609 | 0.023242 | 0.056636 |
| 0.066923 | 0 | 0 | 0 |
| 8.776686 | 7.224334 | 8.229906 | 7.901338 |
| 16.22596 | 17.99436 | 18.10193 | 17.83276 |
| 2.064652 | 0.971264 | 1.580652 | 2.31102 |
| 16.55103 | 16.40658 | 15.01938 | 15.94917 |
| 14.13979 | 9.275034 | 8.826628 | 8.765745 |
| 0 | 0 | 0 | 0 |
| 0.311216 | 1.17123 | 0.190608 | 1.300515 |
| 16.17393 | 12.939 | 14.81926 | 14.05829 |
| 16.86022 | 17.29207 | 17.64997 | 17.48047 |
| 1.035131 | 0.870189 | 0.899097 | 0.85389 |
| 0 | 0 | 0 | 0 |
| 0.111588 | 0.051422 | 0.039053 | 0.057099 |
| 2280.728 | 2417.946 | 2278.341 | 2381.964 |
| 5.604294 | 4.768062 | 5.221285 | 4.864578 |
| 7.834853 | 6.318361 | 6.995072 | 5.698284 |
| 458.4233 | 410.697 | 404.0715 | 387.0852 |
| 8.720341 | 9.303695 | 9.203257 | 9.342944 |
| 13.54443 | 12.97909 | 11.60504 | 12.73598 |
| 11.69956 | 8.622977 | 8.412609 | 8.747854 |
| 9.591969 | 9.056949 | 8.352348 | 8.0864 |
| 1.614103 | 1.542734 | 0.659052 | 0.642385 |
| 3.182097 | 2.69471 | 2.706265 | 2.421289 |
| 16.79292 | 16.65876 | 15.39436 | 15.23143 |
| 67.02772 | 63.04676 | 63.43718 | 61.55859 |
| 0.072807 | 0.156573 | 0 | 0.130392 |
| 2.674224 | 2.529356 | 2.911753 | 2.719865 |
| 31.97716 | 30.91605 | 30.69793 | 30.0376 |
| 7.898733 | 5.990034 | 6.412878 | 5.850176 |

| | | | |
|---|---|---|---|
| 0.599101 | 0.416038 | 0.336349 | 0.417256 |
| 49.03919 | 65.80059 | 62.97471 | 63.22294 |
| 5.222405 | 5.698007 | 5.832606 | 5.868499 |
| 0 | 0.085761 | 0 | 0 |
| 0 | 0 | 0.045854 | 0 |
| 130.0906 | 118.9825 | 119.44 | 124.9651 |
| 2.380802 | 2.559974 | 3.645379 | 3.553193 |
| 0 | 0 | 0 | 0 |
| 0.975739 | 1.04917 | 2.071691 | 1.00965 |
| 0 | 0 | 0 | 0 |
| 53.56805 | 49.30321 | 49.68516 | 52.3767 |
| 35.75097 | 31.71295 | 31.06686 | 31.34855 |
| 3.981976 | 2.794338 | 2.669855 | 2.402159 |
| 23.44494 | 21.97451 | 22.57646 | 20.97339 |
| 0 | 0.030047 | 0.034229 | 0.066727 |
| 0.076308 | 0 | 0 | 0 |
| 0.039548 | 0 | 0 | 0 |
| 4.824697 | 6.025816 | 5.818968 | 6.247859 |
| 0 | 0.275266 | 0 | 0.152826 |
| 5.076102 | 5.369123 | 5.0351 | 5.121868 |
| 0.184023 | 0.098936 | 0.056354 | 0 |
| 3.011613 | 2.363053 | 3.489593 | 3.498528 |
| 0.577164 | 0.077575 | 0.353491 | 0.344552 |
| 1.143929 | 0.768761 | 1.926687 | 0.341448 |
| 943.5705 | 1040.242 | 985.9749 | 1045.979 |
| 42.84099 | 35.39851 | 33.81758 | 35.57208 |
| 24.25141 | 25.45464 | 24.51111 | 25.70958 |
| 64.43531 | 59.65575 | 62.25224 | 65.37283 |
| 0.342288 | 0.408942 | 0.326104 | 0.272449 |
| 4.249201 | 27.95143 | 5.051863 | 4.327248 |
| 0 | 0 | 0 | 0 |
| 8.335491 | 7.577986 | 8.983353 | 8.670752 |
| 0 | 0.494681 | 0.281768 | 0 |
| 2.912073 | 2.738421 | 2.863884 | 2.442528 |
| 0.824978 | 0.614121 | 0.88098 | 0.959725 |
| 10.76387 | 11.01808 | 8.28807 | 9.628339 |
| 0 | 0 | 0 | 0 |
| 8.84011 | 7.067319 | 7.031472 | 7.042133 |
| 0 | 0 | 0 | 0 |
| 8.8789 | 7.350421 | 6.847331 | 7.47829 |
| 22.57814 | 20.60229 | 22.10618 | 20.74338 |
| 0.289606 | 0.493051 | 0.502556 | 0.576291 |
| 0 | 0 | 0 | 0 |
| 45.35474 | 46.05977 | 46.45817 | 45.98573 |
| 9.313861 | 8.338005 | 8.896752 | 7.090445 |
| 0.241706 | 0.233906 | 0.236857 | 0.447306 |
| 0 | 0.15515 | 0 | 0 |
| 2.025779 | 1.342144 | 1.228181 | 1.490295 |
| 1.028253 | 0.893014 | 0.484436 | 0.991589 |
| 2.731711 | 1.7655 | 1.851056 | 3.400309 |
| 4.071681 | 3.121426 | 3.001737 | 3.240917 |
| 20.93755 | 16.60655 | 16.05001 | 16.18173 |
| 0 | 0 | 0 | 0 |
| 6.6222 | 6.712277 | 7.888437 | 7.562011 |

| | | | |
|---|---|---|---|
| 2.629304 | 2.31001 | 2.670823 | 2.373581 |
| 21.56717 | 21.82394 | 22.04317 | 21.95727 |
| 1.356814 | 0.760635 | 1.008561 | 0.636909 |
| 16.3148 | 17.98208 | 17.0222 | 16.79506 |
| 0.710687 | 0.127362 | 0.290179 | 0.707103 |
| 2.820085 | 2.332551 | 2.524362 | 2.557651 |
| 5.803823 | 4.448088 | 3.963026 | 4.511523 |
| 0.025114 | 0.081012 | 0.092288 | 0 |
| 51.85486 | 47.21562 | 45.57625 | 49.46588 |
| 0 | 0 | 0 | 0 |
| 1.529383 | 1.283497 | 1.325073 | 1.091149 |
| 0.059495 | 0.063973 | 0.121462 | 0.094712 |
| 2.979006 | 3.151532 | 3.138974 | 3.461166 |
| 4.680525 | 3.090991 | 4.108105 | 4.444241 |
| 360.2161 | 301.0664 | 302.9791 | 300.529 |
| 1.764933 | 2.097521 | 1.86607 | 1.486158 |
| 6.268441 | 6.761789 | 6.644741 | 6.956462 |
| 1.133715 | 3.047588 | 1.388716 | 2.030396 |
| 98.95969 | 108.324 | 103.8672 | 105.0333 |
| 0 | 0 | 0 | 0 |
| 0.051869 | 0.055773 | 0.063536 | 0.092894 |
| 15.59745 | 14.05772 | 14.23267 | 13.9146 |
| 2.990459 | 2.647024 | 2.549994 | 2.623593 |
| 1.426181 | 1.582979 | 1.521549 | 2.197143 |
| 35.43752 | 35.52836 | 32.83134 | 39.6289 |
| 18.4575 | 18.73453 | 17.47143 | 17.98998 |
| 6.875044 | 6.799219 | 6.134113 | 6.637694 |
| 3.894716 | 5.133458 | 4.27827 | 3.600069 |
| 0 | 0.286031 | 1.303376 | 0.635208 |
| 0.44418 | 0.382086 | 0.217635 | 0 |
| 28.99627 | 36.66702 | 34.09975 | 37.51262 |
| 0.045917 | 0.032915 | 0.018748 | 0.036548 |
| 3.846711 | 3.465467 | 3.502111 | 3.661806 |
| 0.033548 | 0.048097 | 0.082188 | 0.08011 |
| 32.0421 | 31.88951 | 31.39932 | 32.29569 |
| 5.749274 | 5.287633 | 5.437823 | 5.970605 |
| 2.113743 | 1.7596 | 1.670436 | 1.411101 |
| 5.849581 | 4.331888 | 4.851211 | 4.538304 |
| 1.847629 | 1.731246 | 1.648907 | 1.85932 |
| 0.057094 | 0 | 0 | 0.068167 |
| 3.291213 | 3.029706 | 3.88647 | 2.657385 |
| 11.57877 | 13.28948 | 13.22695 | 13.35845 |
| 0.017153 | 0.018444 | 0 | 0 |
| 0 | 0 | 0 | 0 |
| 5.796506 | 5.151255 | 5.057735 | 5.214247 |
| 0.403526 | 0 | 0 | 0 |
| 0.582564 | 0.479016 | 0.587668 | 0.368233 |
| 0 | 0.147125 | 0.167604 | 0 |
| 96.87076 | 99.95929 | 94.30216 | 95.00264 |
| 7.954369 | 5.923124 | 6.315733 | 5.814017 |
| 5.933782 | 4.828366 | 4.446782 | 5.152456 |
| 1.053876 | 1.339222 | 0.997528 | 0.915108 |
| 0 | 0 | 0 | 0.036373 |
| 0 | 0 | 0 | 0 |

| | | | |
|---|---|---|---|
| 0.710687 | 0 | 0 | 0.424262 |
| 0 | 0 | 0 | 0 |
| 2.430438 | 2.190057 | 1.886896 | 2.063968 |
| 0 | 0 | 0 | 0 |
| 0.092168 | 0.099104 | 0.084674 | 0.096288 |
| 0.101096 | 0.054352 | 0.123835 | 0 |
| 118.6446 | 128.1252 | 130.4283 | 121.0287 |
| 14.37859 | 13.44407 | 14.37944 | 14.63781 |
| 0 | 0.110582 | 0.503897 | 0.613943 |
| 0 | 0 | 0.129902 | 0 |
| 0.011904 | 0 | 0.014582 | 0 |
| 0.130098 | 0.09326 | 0 | 0 |
| 37.02122 | 38.95944 | 41.18736 | 36.86268 |
| 0.107485 | 0.07705 | 0.087774 | 0 |
| 0 | 0 | 0 | 0 |
| 8.763689 | 7.077884 | 7.609821 | 7.510389 |
| 11.43802 | 10.08199 | 9.098312 | 9.104267 |
| 0.174418 | 0 | 0.427297 | 0 |
| 0.273655 | 0.220687 | 0.502811 | 0.571778 |
| 3.077002 | 1.999422 | 2.006568 | 2.484427 |
| 0.283429 | 0 | 0.115726 | 0.1128 |
| 0 | 0.026862 | 0 | 0 |
| 14.61922 | 13.77836 | 12.32496 | 13.24994 |
| 2.45401 | 2.161953 | 2.046085 | 1.908168 |
| 0 | 0 | 0 | 0 |
| 15.29437 | 12.66739 | 13.9632 | 12.62303 |
| 0 | 0 | 0 | 0 |
| 0 | 0 | 0 | 0 |
| 0.556844 | 0.535053 | 0.609528 | 0.565823 |
| 6.487831 | 14.45046 | 11.92065 | 5.947924 |
| 0.187096 | 0 | 0.229179 | 0.111692 |
| 2.183989 | 1.815875 | 1.648683 | 1.773754 |
| 2.887905 | 1.626554 | 1.600282 | 1.990814 |
| 0.785743 | 0.168975 | 0.096248 | 0.281441 |
| 0.953659 | 0.935479 | 0.942726 | 0.838983 |
| 0 | 0 | 0 | 0 |
| 11.85643 | 10.19079 | 10.16393 | 10.27046 |
| 14.85621 | 14.02866 | 14.77594 | 14.09907 |
| 18.09068 | 18.37554 | 17.4351 | 16.98061 |
| 92.61902 | 86.16499 | 83.81407 | 99.60495 |
| 0.018978 | 0.020406 | 0.023247 | 0.045318 |
| 28.9646 | 22.65046 | 24.90729 | 25.58736 |
| 0.44501 | 0.957 | 0.545103 | 1.239743 |
| 2.932644 | 1.898789 | 1.854073 | 1.769537 |
| 19.69707 | 20.24659 | 18.88653 | 19.75075 |
| 5.001685 | 3.728814 | 4.329525 | 3.264558 |
| 0.073995 | 0 | 0 | 0.044173 |
| 0 | 0 | 0 | 0 |
| 22.44641 | 19.92535 | 18.3959 | 19.6622 |
| 5.565512 | 3.398523 | 3.899626 | 3.691629 |
| 0.183775 | 0.039521 | 0.067533 | 0.043884 |
| 0 | 0 | 0 | 0.030418 |
| 0.611846 | 0.425695 | 0.484948 | 0.343771 |
| 15.30724 | 13.60085 | 15.17193 | 15.8346 |

| | | | |
|---|---|---|---|
| 0.48097 | 1.5515 | 1.178304 | 0 |
| 1.95358 | 0.721418 | 0.652633 | 0.68325 |
| 0.063488 | 0.068266 | 0 | 0.075801 |
| 369.8029 | 159.832 | 158.4088 | 151.3341 |
| 0 | 0.009223 | 0 | 0.020483 |
| 1.612964 | 1.589822 | 1.975759 | 1.344045 |
| 0.912768 | 1.177752 | 0.745381 | 0.726531 |
| 0 | 0.024951 | 0 | 0 |
| 2.465859 | 1.527091 | 1.701416 | 1.788825 |
| 5.761504 | 3.757027 | 3.642533 | 4.748686 |
| 0.242489 | 0.61629 | 1.566163 | 1.237038 |
| 0.063914 | 0.137448 | 0.039145 | 0 |
| 0 | 0 | 0 | 0 |
| 11.64056 | 10.82563 | 11.04164 | 10.82048 |
| 2.645336 | 2.844416 | 2.160224 | 2.105596 |
| 6.111772 | 4.172921 | 4.440637 | 4.827764 |
| 44.35013 | 42.03854 | 45.33894 | 45.20961 |
| 0 | 0 | 0 | 0 |
| 11.32162 | 8.466451 | 10.95754 | 8.288466 |
| 494.7366 | 439.1778 | 423.26 | 411.0123 |
| 1.552466 | 1.200524 | 1.380653 | 1.320347 |
| 45.73026 | 57.41599 | 54.52681 | 56.40671 |
| 15.23373 | 14.59574 | 11.88413 | 14.02225 |
| 0.071175 | 0 | 0 | 0 |
| 14.28228 | 12.79987 | 12.96996 | 13.62372 |
| 12.15286 | 12.62015 | 11.88359 | 12.04427 |
| 17.55172 | 17.11885 | 15.44627 | 15.72415 |
| 7.061427 | 5.085119 | 5.681831 | 5.027647 |
| 23.36341 | 22.73786 | 20.74014 | 20.85558 |
| 0.337702 | 0.181558 | 0.41366 | 0.403199 |
| 0.019873 | 0 | 0 | 0.023727 |
| 0 | 0.054526 | 0 | 0 |
| 28.01374 | 23.27964 | 22.66742 | 20.43495 |
| 0.030562 | 0.032862 | 0 | 0 |
| 27.88082 | 27.48181 | 27.35205 | 27.58015 |
| 14.37003 | 13.22329 | 12.65998 | 12.8177 |
| 31.15889 | 29.58717 | 30.3188 | 29.70928 |
| 36.7186 | 33.18245 | 31.18598 | 32.61381 |
| 0 | 0 | 0 | 0 |
| 0 | 0 | 2.120948 | 0 |
| 0 | 0 | 0 | 0 |
| 375.271 | 349.2717 | 341.2941 | 314.6511 |
| 10.24812 | 9.120156 | 7.933065 | 8.764909 |
| 0 | 0 | 0 | 0 |
| 0.043455 | 0.046726 | 0.172995 | 0.025942 |
| 12.45904 | 11.6526 | 10.25704 | 10.68757 |
| 0.623543 | 0.609518 | 0.208307 | 0.20304 |
| 52.07681 | 38.20441 | 37.67686 | 36.22863 |
| 3.457488 | 2.85541 | 3.172345 | 2.935161 |
| 5.218788 | 5.33328 | 4.70201 | 5.278294 |
| 2.50733 | 2.683542 | 2.189716 | 2.037326 |
| 0.004505 | 0 | 0.005518 | 0 |
| 0 | 0 | 0 | 0 |
| 3.675723 | 4.495417 | 3.952561 | 4.522626 |

| | | | |
|---|---|---|---|
| 0 | 0 | 0.01137 | 0.011082 |
| 0.013679 | 0.044125 | 0.067022 | 0 |
| 0.122406 | 0 | 0 | 0 |
| 0.634881 | 0 | 0.38884 | 0 |
| 0 | 0 | 0 | 0 |
| 5.020966 | 4.326214 | 4.134145 | 4.347203 |
| 10.19038 | 7.861725 | 10.01954 | 8.129378 |
| 0 | 0 | 0 | 0 |
| 8.935853 | 8.393801 | 8.854984 | 8.900777 |
| 5.860436 | 6.587906 | 5.139219 | 5.724865 |
| 15.98524 | 15.23308 | 15.15058 | 13.83703 |
| 0.033182 | 0.035679 | 0 | 0 |
| 0.080774 | 0.021713 | 0 | 0.04822 |
| 1.242158 | 0.692553 | 0.591714 | 0.686607 |
| 0.255238 | 0.243952 | 0.208431 | 0.06772 |
| 0.468516 | 0.331431 | 0.241641 | 0.368016 |
| 0.31744 | 0.227553 | 0.129613 | 0.505343 |
| 0.481265 | 0.313626 | 0.214368 | 0.417895 |
| 1.365139 | 1.1743 | 1.103647 | 1.108335 |
| 0 | 0 | 0 | 0 |
| 1.849786 | 0.994498 | 0.898526 | 0.990039 |
| 0.270546 | 0.145453 | 0.331398 | 0.323018 |
| 1.094622 | 0 | 0 | 0 |
| 0.390295 | 0 | 0 | 0.465993 |
| 0.030309 | 0.03259 | 0.012376 | 0 |
| 0 | 0.065181 | 0.074253 | 0.072376 |
| 3.439534 | 2.08034 | 2.150472 | 2.652195 |
| 0 | 0.426662 | 0 | 0 |
| 82.69942 | 78.19479 | 78.94387 | 78.35184 |
| 88.10732 | 89.33362 | 89.90854 | 90.47745 |
| 0.183166 | 0.159075 | 0.129441 | 0.109345 |
| 18.88316 | 13.39964 | 15.06479 | 13.57929 |
| 0.907371 | 0.915617 | 0.735274 | 1.066687 |
| 0.061519 | 0 | 0 | 0 |
| 5.961559 | 5.870154 | 5.095673 | 4.732159 |
| 0 | 0 | 0 | 0 |
| 6.772879 | 6.420327 | 6.902492 | 6.663248 |
| 0 | 0 | 0.67821 | 1.322118 |
| 6.844658 | 7.589361 | 6.887532 | 6.175156 |
| 799.6399 | 784.9755 | 762.222 | 793.2344 |
| 4.226368 | 4.196135 | 4.364535 | 4.300204 |
| 14.69034 | 13.57234 | 12.33631 | 13.08248 |
| 7.711262 | 6.272394 | 6.350163 | 6.296911 |
| 0.030406 | 0 | 0 | 0 |
| 0.104022 | 0.083888 | 0.031855 | 0.077623 |
| 0 | 0.145041 | 0.16523 | 0 |
| 1.322668 | 1.066656 | 0.961975 | 1.102148 |
| 0 | 0 | 0 | 0.02297 |
| 0 | 0.555008 | 0.474196 | 0.616272 |
| 0 | 0 | 0.062716 | 0 |
| 39.35744 | 27.26477 | 25.25092 | 26.7308 |
| 0.059619 | 0 | 0.024343 | 0.142365 |
| 122.4015 | 151.669 | 154.2669 | 150.7882 |
| 0.091805 | 0.131618 | 0.074969 | 0.073073 |

| | | | |
|---|---|---|---|
| 9.567415 | 9.676295 | 10.40432 | 9.801912 |
| 7.089848 | 5.651423 | 5.917534 | 5.367341 |
| 0.065138 | 0.105061 | 0.079789 | 0.155543 |
| 17.4281 | 12.49312 | 11.65886 | 12.16911 |
| 0.156118 | 0.335734 | 0.191233 | 0 |
| 0 | 0 | 0 | 0 |
| 40.19847 | 38.94634 | 43.74454 | 37.03177 |
| 7.230487 | 4.254844 | 4.637743 | 4.614638 |
| 6.489157 | 4.261076 | 3.944025 | 4.391358 |
| 3.357296 | 2.104088 | 2.255964 | 2.015671 |
| 12.79623 | 11.96674 | 12.79837 | 12.02619 |
| 27.38906 | 25.43049 | 26.75286 | 24.80775 |
| 576.8035 | 884.2772 | 852.7977 | 872.4058 |
| 0.024115 | 0.02593 | 0.007385 | 0.014396 |
| 18.79853 | 18.21413 | 19.23115 | 13.56534 |
| 3.882539 | 3.288582 | 2.938736 | 3.192407 |
| 0.266953 | 0.528764 | 0.499102 | 0.805209 |
| 23.12162 | 20.30067 | 19.08303 | 20.03692 |
| 0.202191 | 0 | 0 | 0 |
| 0.472722 | 0.138627 | 0.210563 | 0.205239 |
| 81.61059 | 66.45107 | 66.52363 | 61.23513 |
| 30.43243 | 25.76041 | 28.91899 | 24.67908 |
| 2.205941 | 2.343027 | 2.207823 | 2.473183 |
| 6.658288 | 5.333874 | 4.419133 | 5.320882 |
| 0 | 1.347355 | 0.767448 | 1.496081 |
| 1.882495 | 1.508202 | 1.446848 | 1.41026 |
| 0.043406 | 0.140017 | 0.053169 | 0.12956 |
| 0.881779 | 0.568883 | 0.648067 | 0.421119 |
| 4.493394 | 7.940228 | 6.528081 | 7.735408 |
| 26.49774 | 27.97645 | 28.24965 | 27.37628 |
| 0 | 0.024369 | 0.013881 | 0 |
| 2.056355 | 1.695185 | 1.539315 | 1.636788 |
| 0 | 0 | 0 | 0 |
| 3.874473 | 3.405584 | 3.182791 | 3.579581 |
| 19.64305 | 17.12407 | 16.80915 | 17.28821 |
| 7.202889 | 8.277053 | 6.937164 | 7.680805 |
| 11.50228 | 8.972793 | 9.323882 | 8.448563 |
| 7.236266 | 6.174758 | 5.958703 | 5.795967 |
| 0.085333 | 0 | 0.104527 | 0.203767 |
| 0.352711 | 0.379255 | 0 | 0.421119 |
| 3.419126 | 3.044903 | 3.46873 | 2.905166 |
| 0.112355 | 0.048324 | 0.055051 | 0 |
| 19.11255 | 16.82946 | 15.79664 | 17.21325 |
| 19.69991 | 11.4519 | 10.81638 | 11.46797 |
| 0.525028 | 0.586254 | 0.445237 | 0.602747 |
| 5.151313 | 4.479353 | 3.932299 | 4.849011 |
| 6.062716 | 5.589315 | 5.799026 | 5.765677 |
| 0.243249 | 0.163472 | 0.409698 | 0.290427 |
| 8.017563 | 5.544925 | 6.109251 | 5.662638 |
| 0 | 0 | 0 | 0 |
| 6.802292 | 10.2399 | 1.666459 | 2.436475 |
| 0.666891 | 0.573664 | 0.326757 | 0.636987 |
| 0.225268 | 0.181666 | 0.068984 | 0 |
| 1.418487 | 0.866613 | 0.90826 | 0.885291 |

| | | | |
|---|---|---|---|
| 0.324959 | 0.493291 | 0.515124 | 0.547742 |
| 5.812661 | 6.506958 | 5.559507 | 7.035084 |
| 21.59869 | 19.18208 | 18.05523 | 18.77976 |
| 0 | 0 | 0 | 0 |
| 0.028876 | 0.062098 | 0 | 0 |
| 3.0697 | 2.456347 | 3.20633 | 2.500198 |
| 14.3696 | 10.5566 | 13.55657 | 11.13578 |
| 0.363481 | 0 | 0.445237 | 0 |
| 12.85864 | 10.71351 | 10.57309 | 11.13272 |
| 0.060273 | 0 | 0 | 0.143927 |
| 15.39636 | 14.67073 | 13.09423 | 13.42068 |
| 22.90481 | 22.93134 | 20.96731 | 21.10714 |
| 2.862662 | 2.534904 | 2.12614 | 2.273425 |
| 2.896431 | 1.676989 | 2.027376 | 1.520083 |
| 0 | 0 | 0 | 0 |
| 0.191691 | 0.12367 | 0.046961 | 0.045774 |
| 8.58329 | 7.720279 | 7.435673 | 6.857979 |
| 0 | 0 | 0 | 0 |
| 2.660627 | 3.65006 | 3.596212 | 3.724349 |
| 9.894878 | 7.326732 | 7.268294 | 6.928787 |
| 0.027508 | 0 | 0 | 0 |
| 7.497948 | 6.922994 | 7.387468 | 7.297957 |
| 5.00546 | 3.864112 | 3.170464 | 3.779857 |
| 13.00754 | 11.28509 | 11.38911 | 10.23207 |
| 66.84967 | 72.5299 | 71.89972 | 71.6677 |
| 11.41462 | 11.26389 | 11.98925 | 10.73854 |
| 4.076243 | 3.164681 | 3.317443 | 3.068573 |
| 0 | 0 | 0 | 0 |
| 0 | 0 | 0 | 0 |
| 0.619508 | 0.277554 | 0.484821 | 0.226007 |
| 2.252802 | 8.257982 | 5.76989 | 7.824666 |
| 16.83761 | 12.45919 | 12.60405 | 12.89778 |
| 1.803638 | 0.77575 | 1.325592 | 0.43069 |
| 194.8212 | 226.8003 | 228.1102 | 241.4778 |
| 3.060485 | 2.879457 | 2.40552 | 2.496941 |
| 0.062365 | 0 | 0 | 0 |
| 0.662959 | 0.754784 | 1.05092 | 0.698416 |
| 7.411541 | 5.965853 | 7.049845 | 5.339059 |
| 0.507825 | 0.760176 | 0.597653 | 0.630094 |
| 0 | 0 | 0 | 0 |
| 2.769759 | 2.698996 | 2.902387 | 2.19602 |
| 0 | 0 | 0 | 0 |
| 8.084344 | 6.140748 | 5.996137 | 7.704119 |
| 6.513948 | 5.524415 | 5.956226 | 7.229618 |
| 0 | 0 | 2.953218 | 1.439268 |
| 0 | 0 | 0.094839 | 0 |
| 0 | 0 | 0.057777 | 0 |
| 8.793788 | 7.902876 | 7.256741 | 7.07323 |
| 2.105319 | 1.854356 | 1.728383 | 1.684675 |
| 9.267256 | 7.165081 | 8.027266 | 9.457618 |
| 0.025559 | 0.054965 | 0.062615 | 0.030516 |
| 0.754314 | 0.27036 | 0.38499 | 0.300204 |
| 0 | 0 | 0.234241 | 0 |
| 0 | 0 | 0 | 0 |

| | | | |
|---|---|---|---|
| 1.936611 | 1.685716 | 1.50616 | 1.724985 |
| 12.27029 | 10.16605 | 9.702316 | 10.30426 |
| 22.22708 | 16.3596 | 17.6397 | 18.61397 |
| 0.029594 | 0 | 0 | 0 |
| 0.104022 | 0 | 0 | 0 |
| 0.428973 | 0.384381 | 0.175153 | 0.256086 |
| 0 | 0 | 0.059425 | 0.028961 |
| 0 | 0 | 0 | 0 |
| 0 | 0 | 0 | 0 |
| 0.070542 | 0 | 0 | 0 |
| 0.965451 | 0.872011 | 0.756867 | 0.599403 |
| 0 | 0.019899 | 0.045337 | 0.022095 |
| 3.968004 | 2.844416 | 2.700281 | 4.211192 |
| 515.0494 | 537.9239 | 519.2145 | 534.3271 |
| 37.60782 | 38.6583 | 39.18266 | 37.4105 |
| 0 | 0 | 0 | 0.498694 |
| 2.641269 | 2.236616 | 2.18181 | 2.25773 |
| 0.231896 | 0.083116 | 0.047343 | 0.138436 |
| 0.018279 | 0 | 0 | 0.021824 |
| 5.48247 | 4.65331 | 4.884082 | 5.007308 |
| 3.803075 | 3.254735 | 2.281704 | 2.409337 |
| 0.363481 | 0 | 0 | 0 |
| 0.875295 | 0.376467 | 0.214434 | 0 |
| 7.767156 | 7.873035 | 7.596302 | 7.605803 |
| 0.055691 | 0.059882 | 0 | 0 |
| 0.127146 | 0 | 0 | 0.227708 |
| 3.309057 | 2.680118 | 2.895247 | 2.719411 |
| 126.8609 | 119.2641 | 115.9641 | 111.6386 |
| 30.18706 | 33.07649 | 33.66247 | 33.35264 |
| 7.476196 | 8.203637 | 7.906143 | 7.116552 |
| 0 | 0 | 0 | 0 |
| 5.406189 | 6.285041 | 5.461877 | 6.316788 |
| 266.8527 | 267.8215 | 270.9677 | 263.3506 |
| 0 | 0 | 0 | 0 |
| 9.821019 | 7.609762 | 9.406768 | 9.448547 |
| 2.930491 | 1.871522 | 2.001491 | 2.205339 |
| 0 | 0 | 0.134083 | 0 |
| 11.37451 | 11.9353 | 13.83682 | 11.52007 |
| 0.096781 | 0 | 0 | 0 |
| 3.3013 | 4.253299 | 3.861688 | 4.0126 |
| 0.248 | 0.533328 | 0.303782 | 0 |
| 12.86546 | 14.27993 | 13.38686 | 13.3126 |
| 0.12178 | 0.163681 | 0.149172 | 0.0727 |
| 0.710064 | 0.389236 | 1.074427 | 0.980764 |
| 12.12583 | 12.12675 | 12.41391 | 12.38247 |
| 0.867194 | 0.561377 | 0.606999 | 0.538823 |
| 0.125927 | 0.152329 | 0.038563 | 0.056381 |
| 17.92766 | 14.56856 | 15.1923 | 14.17856 |
| 0 | 0 | 0 | 0.43069 |
| 0.087852 | 0 | 0.107613 | 0.524457 |
| 0 | 0 | 0 | 0 |
| 13.35746 | 10.70012 | 10.00499 | 10.79532 |
| 3.35012 | 3.284395 | 2.474256 | 2.352864 |
| 5.616252 | 3.807141 | 3.589296 | 4.344006 |

| | | | |
|---|---|---|---|
| 0.049165 | 0.052865 | 0.030112 | 0.02935 |
| 26.18025 | 21.72751 | 19.92048 | 20.81582 |
| 0 | 0 | 0 | 0 |
| 0 | 0.787684 | 0 | 0 |
| 8.447019 | 8.48929 | 8.469109 | 8.236635 |
| 9.202725 | 8.335919 | 8.158411 | 8.454448 |
| 7.199055 | 6.480305 | 6.620952 | 6.143525 |
| 0 | 0 | 0 | 0.032339 |
| 5.760091 | 6.95639 | 6.861126 | 7.168016 |
| 0.226743 | 0.406345 | 0.185162 | 0.721919 |
| 0.584246 | 0 | 0 | 0.34878 |
| 0.145251 | 0.056794 | 0.194097 | 0.11036 |
| 11.69517 | 10.77884 | 5.116321 | 12.30111 |
| 1.459975 | 1.412864 | 0.89418 | 1.195292 |
| 3.62296 | 2.226065 | 1.901937 | 1.85384 |
| 8.514869 | 6.645894 | 8.165648 | 7.201138 |
| 4.66824 | 3.883552 | 3.852289 | 3.666866 |
| 12.99734 | 9.536728 | 9.712666 | 9.467048 |
| 0.351967 | 0 | 0.06159 | 0 |
| 14.8729 | 14.19715 | 15.29026 | 14.72239 |
| 0.263655 | 0.113399 | 0.064591 | 0.251832 |
| 7.321864 | 5.608788 | 5.53951 | 5.513698 |
| 6.164481 | 5.124576 | 5.785165 | 5.45904 |
| 61.41388 | 49.09601 | 49.63323 | 47.78175 |
| 0 | 0.624384 | 0 | 0.231102 |
| 0.118074 | 0.092335 | 0.026297 | 0.038448 |
| 0.414053 | 0 | 0.507183 | 0.494357 |
| 9.273537 | 10.1845 | 11.1652 | 10.71725 |
| 4.518024 | 4.013161 | 3.946153 | 4.503056 |
| 1.040047 | 1.069695 | 1.052417 | 0.890829 |
| 9.405153 | 9.266684 | 7.953554 | 8.269249 |
| 0.065407 | 0 | 0 | 0.156184 |
| 0 | 0 | 0 | 0.296099 |
| 0.096781 | 0.52032 | 0.237098 | 0.462204 |
| 1.887504 | 1.705819 | 1.999994 | 2.018545 |
| 2.712758 | 2.264445 | 2.011243 | 2.173467 |
| 0.182262 | 0.195979 | 0.167443 | 0.326418 |
| 38.67195 | 17.29712 | 18.04955 | 17.2858 |
| 3.570233 | 1.377003 | 1.568672 | 1.436336 |
| 1.065692 | 0.755802 | 0.833229 | 1.082878 |
| 0.182437 | 0 | 0.223471 | 0 |
| 2.886332 | 2.795009 | 2.232972 | 2.297421 |
| 29.11773 | 30.57236 | 29.79245 | 30.20762 |
| 0.038462 | 0 | 0.047113 | 0.091843 |
| 2.127049 | 2.233624 | 2.392161 | 1.797017 |
| 11.77941 | 10.69581 | 10.43901 | 10.01578 |
| 0 | 0 | 0 | 0 |
| 0 | 0 | 0 | 0 |
| 7.55382 | 5.61516 | 5.546995 | 5.475742 |
| 0.129041 | 0.11893 | 0.067742 | 0.132058 |
| 7.05698 | 6.070454 | 5.959995 | 4.833673 |
| 6.599584 | 5.703623 | 5.179354 | 5.366743 |
| 0 | 0 | 0 | 0 |
| 13.93368 | 13.34423 | 13.22179 | 11.97799 |

| | | | |
|---|---|---|---|
| 0 | 0 | 0.060379 | 0 |
| 8.565081 | 10.45843 | 8.108745 | 9.255634 |
| 32.89266 | 28.82778 | 29.35489 | 28.39438 |
| 64.38425 | 67.21359 | 69.9826 | 68.38181 |
| 55.70948 | 48.01321 | 50.09099 | 50.41637 |
| 0 | 0 | 0 | 0 |
| 13.06105 | 12.25796 | 12.59036 | 12.15332 |
| 14.45801 | 13.87271 | 13.86666 | 11.74783 |
| 0.258432 | 0.231567 | 0.1319 | 0.257128 |
| 21.88802 | 19.81916 | 18.97165 | 23.53513 |
| 1.304549 | 0.701363 | 0 | 0.778782 |
| 5.076434 | 4.357058 | 4.539137 | 4.56823 |
| 1.919459 | 1.810449 | 1.897453 | 1.869573 |
| 12.01499 | 9.832592 | 10.00497 | 10.21129 |
| 220.1897 | 231.289 | 215.6203 | 223.3082 |
| 1.295675 | 6.733719 | 4.629052 | 6.187873 |
| 9.921776 | 8.434466 | 7.998409 | 7.821454 |
| 3.213996 | 3.455871 | 2.845695 | 2.648602 |
| 4.42149 | 2.646024 | 2.965316 | 2.627571 |
| 0 | 0 | 0 | 0 |
| 52.08005 | 51.29645 | 50.26204 | 51.84432 |
| 5.905578 | 6.150328 | 5.914499 | 5.986659 |
| 0.2976 | 0.159998 | 0.182269 | 0.17766 |
| 0 | 0.033974 | 0 | 0.037725 |
| 0.115479 | 0 | 0 | 0.091918 |
| 0 | 0 | 0 | 0 |
| 1.858885 | 0.845637 | 2.276993 | 1.024344 |
| 0.032502 | 0.052423 | 0.079626 | 0.038806 |
| 35.08108 | 39.80172 | 38.66935 | 36.86281 |
| 0.503873 | 0.451495 | 0 | 0 |
| 0 | 0 | 0 | 0 |
| 0.003647 | 0 | 0 | 0 |
| 0.017989 | 0.096712 | 0.154243 | 0.214776 |
| 0.464905 | 0.434117 | 0.38964 | 0.394393 |
| 2.530164 | 2.316878 | 2.099498 | 2.436197 |
| 2.384258 | 2.229295 | 2.285637 | 2.475374 |
| 0.17429 | 0.187407 | 0.128095 | 0.291331 |
| 0.04068 | 0 | 0.024915 | 0 |
| 4.700876 | 4.087252 | 3.994936 | 3.571655 |
| 0.484836 | 0.43901 | 0.390716 | 0.380835 |
| 11.49784 | 12.63189 | 11.25188 | 12.5341 |
| 0.291807 | 0.295311 | 0.126156 | 0.14346 |
| 0 | 1.003911 | 0 | 0 |
| 13.67002 | 12.43046 | 12.21156 | 12.27696 |
| 1.964614 | 1.982752 | 1.836543 | 2.407375 |
| 0 | 0.076077 | 0 | 0.042237 |
| 4.045808 | 3.194264 | 2.945761 | 2.297014 |
| 0 | 0 | 0 | 0 |
| 24.43377 | 26.80657 | 27.59356 | 27.67844 |
| 0 | 0 | 0 | 0 |
| 0 | 0 | 0 | 0 |
| 4.693473 | 4.232708 | 4.219135 | 3.524948 |
| 5.091889 | 10.06233 | 5.506699 | 3.943428 |
| 0 | 0 | 0 | 0 |

| | | | |
|---|---|---|---|
| 29.00345 | 24.00093 | 23.22757 | 24.60345 |
| 9.158508 | 7.490458 | 6.550388 | 6.531515 |
| 0 | 0.241507 | 0 | 0 |
| 1.989972 | 0.154061 | 1.930552 | 0.095037 |
| 0.023103 | 0 | 0 | 0.082753 |
| 192.2569 | 217.445 | 215.4958 | 220.4872 |
| 0 | 0 | 0.166171 | 0 |
| 0 | 0 | 0.018516 | 0 |
| 6.280799 | 5.937617 | 6.340687 | 5.636793 |
| 16.22654 | 13.57695 | 13.82989 | 14.00113 |
| 3.063714 | 2.231609 | 1.985362 | 2.241948 |
| 3.079951 | 3.311739 | 3.546345 | 2.794749 |
| 6.864819 | 5.672319 | 5.417805 | 5.830881 |
| 0.512 | 0 | 0 | 0 |
| 20.10176 | 18.14722 | 17.15925 | 19.87538 |
| 3.2936 | 5.261607 | 5.071831 | 4.381803 |
| 75.42014 | 66.08191 | 65.88407 | 63.77924 |
| 0 | 0 | 0 | 0 |
| 0.011705 | 0.025172 | 0 | 0.013975 |
| 3.381666 | 2.844416 | 2.104549 | 2.376936 |
| 90.13159 | 51.60437 | 54.5942 | 53.17273 |
| 58.03206 | 51.86615 | 47.8456 | 60.84843 |
| 3.442124 | 3.025558 | 2.492996 | 2.984437 |
| 0 | 0 | 0.140884 | 0 |
| 0 | 0.119346 | 0 | 0 |
| 0 | 0 | 0 | 0 |
| 11.10132 | 9.093606 | 8.482856 | 8.493838 |
| 5.878524 | 6.320924 | 5.040524 | 9.124249 |
| 1.359534 | 0.997769 | 0.88553 | 0.991963 |
| 9.036457 | 8.965362 | 8.888306 | 9.524508 |
| 0 | 0 | 0 | 0 |
| 134.1101 | 124.4583 | 114.3253 | 123.379 |
| 15.27886 | 17.29585 | 17.4699 | 16.12804 |
| 0 | 0 | 0 | 0.015885 |
| 32.39778 | 21.1685 | 19.09738 | 21.34139 |
| 0 | 0 | 0 | 0.083482 |
| 20.27043 | 21.18731 | 18.93647 | 20.61169 |
| 0.009282 | 0 | 0.01137 | 0 |
| 6.403302 | 6.372468 | 5.966113 | 6.994554 |
| 0.694787 | 0.597659 | 0.680849 | 0.774237 |
| 25.02314 | 21.17072 | 21.34249 | 19.74543 |
| 17.39974 | 16.84356 | 16.90233 | 16.31855 |
| 0.817258 | 0.323755 | 0.474196 | 0.410848 |
| 0 | 0.492303 | 0 | 0 |
| 31.20742 | 28.55075 | 29.48376 | 28.8308 |
| 16.33898 | 14.37702 | 12.96323 | 13.77807 |
| 18.32482 | 15.0842 | 14.27622 | 15.92737 |
| 0 | 0 | 0 | 0 |
| 0 | 0 | 0 | 0.085878 |
| 3.70347 | 5.309576 | 5.832606 | 4.000632 |
| 1.820583 | 1.118625 | 1.248843 | 1.242104 |
| 6.680512 | 7.894112 | 7.053681 | 7.913871 |
| 1.785602 | 1.137766 | 1.498656 | 1.579197 |
| 12.82929 | 12.06271 | 11.4632 | 11.60834 |

| | | | |
|---|---|---|---|
| 3.483416 | 3.473711 | 3.68194 | 3.186344 |
| 0 | 0 | 0 | 0 |
| 1.407557 | 1.655692 | 2.036581 | 1.697468 |
| 6.282265 | 3.914141 | 3.200382 | 3.294699 |
| 6.682954 | 6.902239 | 5.304817 | 5.695608 |
| 0.192388 | 0.206867 | 0.235661 | 0.229701 |
| 14.1342 | 12.02129 | 13.48169 | 12.27624 |
| 1.082866 | 1.35842 | 1.473811 | 1.364713 |
| 18.78431 | 17.0665 | 18.37182 | 26.07848 |
| 3.388318 | 3.5017 | 3.065161 | 3.559447 |
| 119.1414 | 122.0509 | 119.9779 | 121.6855 |
| 0 | 0 | 0 | 0.076619 |
| 0 | 0 | 0.577486 | 0 |
| 7.91229 | 7.482531 | 6.2928 | 5.963757 |
| 13.00486 | 11.11326 | 12.79987 | 11.57725 |
| 4.184687 | 3.771521 | 4.1306 | 3.815943 |
| 20.5388 | 19.60783 | 19.39868 | 20.26413 |
| 5.746764 | 5.002249 | 4.748769 | 4.955411 |
| 18.14552 | 13.45188 | 13.09041 | 13.02066 |
| 0.17974 | 0.193266 | 0.128431 | 0.304017 |
| 8.541353 | 8.225679 | 7.626878 | 7.783468 |
| 58.97233 | 59.16996 | 57.52095 | 60.73126 |
| 0 | 0 | 0 | 0 |
| 4.569099 | 4.040096 | 3.636501 | 4.735283 |
| 3.088157 | 3.477616 | 1.942498 | 2.939714 |
| 0 | 0 | 0 | 0 |
| 2.262955 | 1.686955 | 1.842062 | 1.614205 |
| 0.165333 | 0.118517 | 0.337535 | 0.1316 |
| 0 | 0 | 0 | 0 |
| 0 | 0 | 0 | 0 |
| 0.092302 | 0.12406 | 0.113062 | 0.123979 |
| 0.0573 | 0.092418 | 0.035094 | 0.068413 |
| 8.526441 | 7.748536 | 7.600716 | 7.631812 |
| 0 | 0.065389 | 0 | 0 |
| 6.208312 | 4.899472 | 5.581441 | 6.188336 |
| 0.014584 | 0 | 0 | 0 |
| 1.076537 | 0.534256 | 0.811493 | 0.6921 |
| 6.917969 | 6.984329 | 5.864949 | 6.935623 |
| 0 | 0 | 0 | 0 |
| 0.396933 | 0.409732 | 0.194485 | 0.246437 |
| 22.32392 | 19.67251 | 20.92894 | 19.6105 |
| 1.304549 | 1.052044 | 0.399494 | 1.168173 |
| 17.98062 | 17.35506 | 15.4503 | 15.19691 |
| 0.738233 | 1.322984 | 0.45214 | 1.175216 |
| 0 | 0 | 0 | 0 |
| 7.04195 | 5.625455 | 5.53127 | 5.882239 |
| 125.0944 | 116.7209 | 116.1925 | 120.1773 |
| 2.806509 | 2.460675 | 2.334146 | 2.328905 |
| 3.218671 | 2.340321 | 2.417543 | 1.882924 |
| 0 | 0.164629 | 0.125029 | 0.060934 |
| 5.705281 | 4.864338 | 5.364939 | 5.074454 |
| 0 | 0.383996 | 0 | 0.142128 |
| 38.83196 | 48.41387 | 47.91882 | 49.37885 |
| 0.158193 | 0.148836 | 0.193774 | 0.118046 |

| | | | |
|---|---|---|---|
| 0.942059 | 0.795894 | 0.834554 | 0.823492 |
| 15.82483 | 15.76774 | 14.18273 | 14.87187 |
| 11.85728 | 11.4 | 12.65967 | 11.60617 |
| 0 | 0 | 0 | 0 |
| 0 | 0 | 0 | 0 |
| 7.564535 | 7.988572 | 7.114952 | 10.40254 |
| 0.227556 | 0.06117 | 0.139369 | 0.135845 |
| 0.440889 | 0 | 0 | 0 |
| 2.800944 | 1.003911 | 1.143648 | 3.344182 |
| 9.528277 | 8.610451 | 9.622713 | 7.836313 |
| 0 | 0 | 0 | 0 |
| 0 | 0.060519 | 0 | 0 |
| 0.414826 | 0.669066 | 0.65331 | 0.530657 |
| 0 | 0 | 0 | 0.07896 |
| 4.355854 | 3.207988 | 3.471789 | 3.312751 |
| 0.076759 | 0.055024 | 0.07313 | 0.010183 |
| 18.54335 | 17.32857 | 17.12522 | 18.61279 |
| 0 | 0 | 0 | 0 |
| 0.319384 | 0.34342 | 0.153087 | 0.298431 |
| 0.413653 | 0.395363 | 0.450394 | 0.24694 |
| 6.670381 | 5.557108 | 5.705994 | 5.808519 |
| 0.016147 | 0 | 0.039557 | 0 |
| 0 | 0.966028 | 0 | 0 |
| 0.255608 | 0.314107 | 0.492014 | 0.217987 |
| 0.19309 | 0.29067 | 0.047304 | 0.23054 |
| 0.331357 | 0.213777 | 0.081178 | 0.197812 |
| 0.785743 | 0.33795 | 0.769981 | 0.281441 |
| 0.078574 | 0.168975 | 0.288743 | 0 |
| 7.25018 | 7.005691 | 6.090633 | 7.194119 |
| 0 | 0 | 0 | 0.153237 |
| 0.326826 | 0.32439 | 0.246361 | 0.330181 |
| 0.020999 | 0.045159 | 0.064307 | 0.087753 |
| 1.785602 | 2.559974 | 2.916303 | 3.553193 |
| 32.20241 | 40.63787 | 40.87082 | 38.61039 |
| 30.6713 | 26.90621 | 29.04236 | 27.32773 |
| 0.529067 | 0.455107 | 0.518454 | 0.758015 |
| 5.375803 | 3.524945 | 2.907841 | 4.079002 |
| 0 | 0.049612 | 0 | 0 |
| 11.01995 | 12.11456 | 10.98021 | 10.01522 |
| 0.632471 | 0.485764 | 0.479594 | 0.395548 |
| 25.69594 | 27.22101 | 25.85716 | 26.77659 |
| 2.690833 | 2.147539 | 2.825201 | 2.314751 |
| 1.671151 | 0.607409 | 0.576629 | 0.730662 |
| 2.868436 | 1.850584 | 2.108171 | 0 |
| 0.042101 | 0.045269 | 0 | 0 |
| 0 | 0 | 0 | 0 |
| 0.720599 | 0.759636 | 0.778835 | 0.742269 |
| 7.711271 | 7.611959 | 7.401732 | 7.013311 |
| 0 | 0 | 0 | 0 |
| 1.309637 | 1.345142 | 1.460546 | 1.423611 |
| 22.034 | 17.30139 | 15.94486 | 17.91605 |
| 2.68854 | 2.651957 | 2.721701 | 2.918161 |
| 7.78388 | 5.943284 | 5.714587 | 5.963612 |
| 0.235501 | 0.204991 | 0.219787 | 0.174061 |

| | | | |
|---|---|---|---|
| 2.330147 | 0.980416 | 0.992784 | 2.056316 |
| 3.401146 | 3.151147 | 3.165058 | 3.173726 |
| 0 | 0.078647 | 0 | 0 |
| 0.089336 | 0 | 0.10943 | 0 |
| 35.44025 | 29.85468 | 29.74895 | 28.39958 |
| 0 | 0 | 0 | 0 |
| 31.11319 | 30.44294 | 30.44577 | 32.50786 |
| 8.634376 | 5.89363 | 5.365997 | 5.634574 |
| 565.7191 | 619.1144 | 624.2129 | 632.0551 |
| 32.57534 | 34.10074 | 30.89518 | 33.27569 |
| 15.33304 | 16.06729 | 16.86948 | 13.58035 |
| 12.69125 | 11.22233 | 11.22105 | 11.3218 |
| 0 | 0 | 0 | 0 |
| 1.108305 | 0.220687 | 0.45253 | 0.588115 |
| 1.606797 | 0.969208 | 1.584165 | 0.889029 |
| 3.485801 | 3.298356 | 3.714764 | 3.662442 |
| 13.26918 | 10.60865 | 10.40498 | 11.40171 |
| 36.63619 | 41.52269 | 42.53166 | 40.87814 |
| 0.093091 | 0.100097 | 0.114029 | 0.055573 |
| 9.393409 | 9.512674 | 7.322138 | 8.319671 |
| 11.30762 | 8.541798 | 8.576502 | 8.729889 |
| 0.514127 | 0.807965 | 0.726654 | 0.519404 |
| 1.538483 | 1.323412 | 1.287759 | 1.224579 |
| 0.010579 | 0.011375 | 0.038875 | 0.012631 |
| 0.740299 | 0.541288 | 0.398997 | 0.388907 |
| 0 | 0.081012 | 0 | 0 |
| 0.045305 | 0.024358 | 0.110992 | 0.054092 |
| 5.852806 | 6.933264 | 5.832606 | 6.277308 |
| 6.57627 | 6.509036 | 5.864625 | 6.751169 |
| 11.98131 | 9.68302 | 10.81778 | 10.82109 |
| 0 | 0.048738 | 0 | 0 |
| 5.336927 | 5.210369 | 4.752569 | 4.622444 |
| 5.037146 | 4.576891 | 4.543826 | 4.548406 |
| 38.66861 | 35.20477 | 34.38085 | 34.49148 |
| 24.88722 | 14.40932 | 14.36886 | 14.81221 |
| 7.662074 | 6.185522 | 5.832606 | 7.387756 |
| 1.297806 | 0.601498 | 0.71263 | 0.774756 |
| 2.429622 | 2.905455 | 2.405915 | 2.236631 |
| 5.306777 | 5.160645 | 4.862971 | 5.518776 |
| 25.94056 | 24.74414 | 23.82167 | 24.32132 |
| 0.177805 | 0.038237 | 0.043559 | 0.169831 |
| 0 | 0 | 0 | 0 |
| 0.111187 | 0.089666 | 0.136196 | 0 |
| 0.379713 | 0.244974 | 0 | 0.090672 |
| 6.067974 | 4.826071 | 5.282824 | 5.14923 |
| 1.125123 | 0.867401 | 0.780108 | 1.039186 |
| 49.25798 | 47.73639 | 43.66719 | 43.69391 |
| 0.586947 | 0.433894 | 0.584159 | 0.700784 |
| 3.462985 | 3.413299 | 3.181421 | 2.928692 |
| 1.509442 | 2.901796 | 1.344693 | 2.621376 |
| 5.705816 | 4.578056 | 4.860505 | 4.806753 |
| 19.87753 | 15.0061 | 15.03752 | 15.29811 |
| 18.92802 | 18.44291 | 18.51857 | 18.24962 |
| 0 | 0.412899 | 0 | 0 |

| | | | |
|---|---|---|---|
| 0 | 0 | 0 | 0 |
| 1.682816 | 1.028102 | 1.07751 | 1.575392 |
| 188.5211 | 87.16153 | 83.61832 | 83.54251 |
| 4.096073 | 2.613559 | 2.804062 | 2.426056 |
| 0.634881 | 0.273064 | 0.155536 | 0.151603 |
| 0.124584 | 0 | 0.091564 | 0.089248 |
| 3.570566 | 3.126268 | 3.124052 | 3.075501 |
| 5.03259 | 5.335645 | 4.289315 | 3.424511 |
| 6.590686 | 4.634976 | 4.627758 | 5.007506 |
| 0.566858 | 0 | 1.388716 | 0.676799 |
| 0 | 0 | 0 | 0 |
| 0 | 0 | 0 | 0 |
| 2.541632 | 2.336012 | 2.008793 | 2.090206 |
| 24.09145 | 23.9018 | 25.51765 | 25.40412 |
| 2.086036 | 1.511604 | 1.555362 | 1.624317 |
| 1.384736 | 1.15055 | 1.272148 | 1.014527 |
| 25.42194 | 25.5399 | 27.59512 | 24.61155 |
| 33.58961 | 28.17956 | 34.36264 | 39.66355 |
| 0.484466 | 0.446507 | 0.084776 | 0 |
| 3.490292 | 3.35492 | 3.238897 | 2.879175 |
| 1.023155 | 0.6206 | 0.706983 | 0.689104 |
| 0.345044 | 0.309176 | 0.375691 | 0.183095 |
| 6.115498 | 3.420901 | 4.026966 | 3.714102 |
| 18.74101 | 16.12112 | 15.47426 | 19.06086 |
| 0 | 0.113777 | 0 | 0 |
| 3.001199 | 2.600915 | 2.567883 | 2.524338 |
| 21.74145 | 18.6595 | 20.38985 | 17.60964 |
| 0 | 0 | 0 | 0 |
| 0 | 0 | 0 | 0.874632 |
| 0.170871 | 0.244974 | 0.122094 | 0.221012 |
| 0.261627 | 0 | 0.640946 | 0.312369 |
| 70.65165 | 57.67564 | 56.57748 | 57.78499 |
| 1.938141 | 1.74953 | 1.37755 | 1.714103 |
| 0.271316 | 0.145867 | 0 | 0.161969 |
| 10.30029 | 12.11291 | 11.24358 | 11.51966 |
| 8.927033 | 8.70945 | 8.832068 | 8.795055 |
| 3.123773 | 2.650109 | 2.720596 | 3.23348 |
| 0.122343 | 0.07893 | 0.029972 | 0.10225 |
| 36.1138 | 25.1823 | 25.06008 | 26.97031 |
| 0.115854 | 0.249146 | 0.425738 | 0.553295 |
| 0.13489 | 0 | 0.16523 | 0.161051 |
| 0.108712 | 0.350681 | 0.266329 | 0.129797 |
| 4.482822 | 3.693078 | 3.797341 | 3.488287 |
| 0 | 0 | 0.19442 | 0 |
| 28.48157 | 29.71464 | 26.53573 | 26.99685 |
| 0 | 0 | 0 | 0.902398 |
| 6.349648 | 6.067383 | 6.850065 | 6.149322 |
| 8.067432 | 6.163505 | 5.869756 | 6.119639 |
| 0 | 0 | 0 | 0 |
| 4.992005 | 4.108346 | 3.386674 | 3.323955 |
| 0 | 0 | 0.08932 | 0 |
| 0.147532 | 0.237953 | 0.203305 | 0.132109 |
| 0 | 0.049278 | 0 | 0 |
| 0 | 0 | 0.627162 | 0 |

| | | | |
|---|---|---|---|
| 9.085211 | 8.230154 | 8.074281 | 7.766542 |
| 32.75858 | 27.00836 | 26.50136 | 27.2548 |
| 11.50146 | 8.409577 | 9.474463 | 8.170627 |
| 0.897752 | 1.07888 | 0.905617 | 0.945767 |
| 0.471326 | 0.727708 | 0.814196 | 0.721461 |
| 2.116269 | 1.061915 | 1.555362 | 1.684477 |
| 0 | 0 | 0 | 0 |
| 0 | 0 | 0 | 0 |
| 0 | 0 | 0 | 0 |
| 1.283137 | 1.480044 | 1.385994 | 1.32309 |
| 12.09021 | 9.262889 | 9.381433 | 9.381319 |
| 4.74091 | 6.311434 | 5.484631 | 6.221948 |
| 12.01726 | 12.90731 | 12.37022 | 13.31403 |
| 71.81811 | 79.45078 | 79.2213 | 82.59039 |
| 16.72913 | 13.87373 | 13.55504 | 14.23561 |
| 0.474435 | 0.046376 | 0.211326 | 0.257478 |
| 5.369205 | 4.546621 | 3.918782 | 4.82642 |
| 0.744001 | 0 | 0 | 0.444149 |
| 5.409976 | 2.131462 | 2.175213 | 2.514662 |
| 2.671723 | 1.851353 | 1.89087 | 1.677651 |
| 38.53891 | 37.08895 | 35.38956 | 36.60021 |
| 0.041659 | 0.179176 | 0.051029 | 0.149215 |
| 4.901652 | 4.517602 | 12.00831 | 13.37673 |
| 0.460058 | 0.371011 | 0.563537 | 0.274643 |
| 292.3185 | 333.9583 | 335.767 | 320.8026 |
| 1.30604 | 0.994733 | 1.266509 | 1.169508 |
| 0.217425 | 0 | 0.266329 | 0.519188 |
| 0.039352 | 0.016925 | 0 | 0.037587 |
| 3.151451 | 3.901184 | 3.114183 | 3.351621 |
| 0.093121 | 0.033376 | 0.076044 | 0.01853 |
| 0 | 0 | 0 | 0 |
| 11.38436 | 9.593456 | 9.518625 | 10.0288 |
| 0 | 0 | 0 | 0 |
| 13.54955 | 13.81288 | 13.76602 | 15.2776 |
| 1.342855 | 1.562824 | 1.509434 | 1.358088 |
| 10.38575 | 9.30613 | 9.667104 | 9.002298 |
| 0.74213 | 0.154448 | 0.263919 | 0.171496 |
| 5.720549 | 4.791352 | 4.775988 | 5.122529 |
| 190.7563 | 168.7803 | 177.3637 | 160.2061 |
| 5.361026 | 5.083637 | 5.119042 | 4.86359 |
| 1.557076 | 0.924939 | 0.786928 | 0.520019 |
| 1.102223 | 1.629613 | 1.316387 | 1.217298 |
| 0.101096 | 0 | 0.123835 | 0.120703 |
| 7.811228 | 6.520689 | 7.061488 | 6.555157 |
| 2.340707 | 4.707463 | 4.911389 | 5.071831 |
| 0 | 0 | 0 | 0 |
| 43.84906 | 39.72313 | 40.39515 | 37.40099 |
| 615.2983 | 709.7936 | 701.1885 | 693.0159 |
| 41.47364 | 44.55922 | 40.78363 | 42.1046 |
| 5.780862 | 4.252992 | 4.844976 | 2.361227 |
| 0.455656 | 0.263818 | 0.257605 | 0.418484 |
| 13.40936 | 13.23227 | 13.60941 | 13.35873 |
| 108.598 | 89.82366 | 98.23336 | 70.81451 |
| 8.776481 | 10.27118 | 11.0475 | 9.118173 |

| | | | |
|---|---|---|---|
| 0 | 0 | 0 | 0 |
| 0.275326 | 0.296046 | 0.281044 | 0.1461 |
| 765.2548 | 662.408 | 656.1548 | 675.8154 |
| 0.025234 | 0.108531 | 0.092728 | 0.120511 |
| 0.374929 | 1.439806 | 0.524869 | 0.255798 |
| 4.091437 | 4.031806 | 3.60335 | 3.561695 |
| 0.2304 | 0.867088 | 0.705557 | 0.275086 |
| 1.361396 | 1.340393 | 1.064857 | 0.959595 |
| 11.16578 | 9.18529 | 10.31848 | 10.57695 |
| 0 | 0 | 0 | 0.414971 |
| 0.449208 | 0.161005 | 0.550246 | 0.536331 |
| 0 | 0 | 0 | 0 |
| 4.584653 | 4.324281 | 4.302204 | 4.385472 |
| 8.754985 | 8.278987 | 7.778221 | 8.201265 |
| 14.65109 | 18.35705 | 17.56626 | 18.3821 |
| 0.732555 | 0 | 0 | 2.623896 |
| 0 | 0 | 0 | 0 |
| 26.99746 | 27.88605 | 29.39981 | 28.81019 |
| 36.45603 | 43.99956 | 40.09917 | 37.30853 |
| 0.234273 | 0.062976 | 0 | 0.034964 |
| 0 | 0.10281 | 0 | 0 |
| 0.458729 | 0.517914 | 0.393336 | 0.602468 |
| 0.264534 | 0.284442 | 0.810084 | 0.15792 |
| 0.395318 | 0.148774 | 0.217906 | 0.117997 |
| 0 | 0 | 0 | 0 |
| 0.13862 | 0 | 0 | 0 |
| 5.189436 | 4.791965 | 4.973728 | 4.611465 |
| 0.172037 | 0.349414 | 0.257562 | 0.342339 |
| 2.261762 | 1.471985 | 1.895597 | 1.421277 |
| 41.71963 | 31.06326 | 33.08708 | 32.01438 |
| 34.11835 | 26.42035 | 25.73532 | 25.33465 |
| 9.997242 | 14.80113 | 13.47418 | 15.70933 |
| 4.875883 | 3.577137 | 4.230584 | 3.486867 |
| 1.586572 | 1.381025 | 1.226212 | 1.014795 |
| 17.0699 | 11.10932 | 12.10541 | 13.94461 |
| 9.08827 | 8.376194 | 10.57582 | 10.34713 |
| 10.00871 | 7.308476 | 7.822554 | 6.153294 |
| 5.065028 | 4.47 | 4.506881 | 4.478485 |
| 2.380802 | 0.639994 | 0 | 2.131916 |
| 0.083684 | 0 | 0.051253 | 0 |
| 3.599485 | 2.432805 | 2.991888 | 1.933919 |
| 12.06798 | 11.70677 | 10.92609 | 12.21594 |
| 0 | 0 | 0.355647 | 0.346653 |
| 15.97736 | 13.74381 | 12.86098 | 14.27566 |
| 5.580093 | 3.645896 | 3.892071 | 4.209206 |
| 1.086814 | 0.949491 | 1.01925 | 0.898858 |
| 5.719184 | 4.712332 | 3.730935 | 4.029023 |
| 2.590605 | 1.687033 | 1.497259 | 1.72078 |
| 73.91147 | 78.45493 | 83.86185 | 68.16474 |
| 0.460058 | 0.353344 | 0.523284 | 0.470816 |
| 4.587481 | 4.914719 | 4.511861 | 4.457733 |
| 0 | 0 | 0.049597 | 0 |
| 0 | 0 | 0 | 0 |
| 403.3027 | 450.2645 | 448.8485 | 453.9407 |

| | | | |
|---|---|---|---|
| 1.27543 | 0.914277 | 1.041537 | 0.507599 |
| 0.278558 | 0.05616 | 0.191932 | 0.228652 |
| 0.051869 | 0.055773 | 0 | 0 |
| 10.93179 | 9.482606 | 10.84002 | 8.481963 |
| 0 | 0 | 0 | 0 |
| 0 | 0 | 0 | 0 |
| 0 | 0.061489 | 0 | 0 |
| 27.27351 | 28.5727 | 27.12484 | 29.21722 |
| 0.090011 | 0.871069 | 0.220514 | 0.644814 |
| 0 | 0.167592 | 0 | 0 |
| 32.98959 | 30.17728 | 28.29664 | 29.03102 |
| 43.6334 | 52.33305 | 48.7844 | 47.27102 |
| 115.4841 | 107.6115 | 105.2838 | 105.0203 |
| 0.084338 | 0.120914 | 0.103308 | 0.050348 |
| 1.437612 | 1.692027 | 1.832357 | 1.577264 |
| 32.33042 | 29.67679 | 31.27418 | 31.87407 |
| 0.061046 | 0.196921 | 0.149554 | 0.072886 |
| 0 | 0 | 0 | 0 |
| 5.792828 | 5.262244 | 6.30056 | 5.008381 |
| 6.656848 | 6.399936 | 6.61924 | 7.012881 |
| 12.07488 | 9.145682 | 8.186114 | 11.06193 |
| 5.722174 | 4.822471 | 4.799113 | 5.026531 |
| 0.015335 | 0.049468 | 0.037569 | 0.036619 |
| 33.43338 | 33.62607 | 32.31514 | 30.91161 |
| 3.757184 | 3.797542 | 3.252268 | 2.960683 |
| 31.96417 | 29.29236 | 28.63721 | 30.94875 |
| 14.98553 | 13.99967 | 12.46404 | 12.83912 |
| 0 | 0 | 1.555362 | 0 |
| 0 | 0 | 0 | 0 |
| 3.135333 | 3.087723 | 2.727865 | 2.413985 |
| 0 | 0.062286 | 0 | 0 |
| 0 | 0 | 0 | 0 |
| 0.166344 | 0.193768 | 0.152819 | 0.115854 |
| 0 | 0 | 0 | 0 |
| 10.23588 | 9.203136 | 8.258936 | 7.88324 |
| 4.750383 | 4.826346 | 4.650507 | 4.745034 |
| 28.62736 | 25.3497 | 25.08669 | 26.50115 |
| 5.636997 | 8.557017 | 5.117746 | 2.929652 |
| 29.63575 | 23.15463 | 23.60508 | 23.89211 |
| 11.34702 | 9.433928 | 8.546873 | 9.341147 |
| 3.320018 | 3.162782 | 3.067915 | 2.712162 |
| 2.483222 | 2.495475 | 2.528382 | 2.695876 |
| 716.3826 | 768.5238 | 683.9689 | 825.8161 |
| 0.212399 | 0.062286 | 0.236521 | 0.322756 |
| 5.844886 | 5.256985 | 4.817889 | 5.142046 |
| 0 | 0 | 0 | 0 |
| 0.680229 | 0.731421 | 0.416615 | 0.406079 |
| 3.820604 | 3.575596 | 4.029958 | 2.829883 |
| 0.462056 | 0.418382 | 0.327674 | 0.348423 |
| 0 | 0 | 0 | 0.43069 |
| 0 | 0.711104 | 0 | 0 |
| 0.686656 | 0.738332 | 0.638077 | 0.537131 |
| 11.50619 | 11.12174 | 9.67103 | 11.32637 |
| 14.86476 | 14.44968 | 14.34587 | 16.0671 |

| | | | |
|---|---|---|---|
| 3.038548 | 2.621602 | 2.763634 | 2.389614 |
| 18.73267 | 18.29026 | 17.85953 | 17.48134 |
| 2.472525 | 2.122592 | 2.149369 | 2.499734 |
| 62.23948 | 54.48605 | 54.61874 | 54.2393 |
| 0.448973 | 0.268201 | 0.366638 | 0.416928 |
| 0 | 0 | 0 | 0 |
| 361.301 | 332.4654 | 325.4831 | 332.8048 |
| 135.2562 | 94.26488 | 93.56125 | 95.91391 |
| 0.468355 | 0 | 1.147398 | 0.372794 |
| 0.018858 | 0 | 0.046199 | 0 |
| 12.13181 | 8.985804 | 9.407429 | 10.10202 |
| 16.08204 | 14.38877 | 13.80053 | 15.38597 |
| 5.277141 | 5.434217 | 5.668517 | 5.185905 |
| 4.462012 | 4.272336 | 4.060181 | 4.185823 |
| 4.431592 | 3.903327 | 4.793132 | 5.065939 |
| 6.302124 | 3.011734 | 5.146417 | 2.508136 |
| 41.85861 | 40.04812 | 41.32674 | 36.67668 |
| 0 | 0.039113 | 0 | 0 |
| 2.837184 | 1.952449 | 2.124919 | 2.013113 |
| 5.53515 | 4.251221 | 3.874366 | 3.40696 |
| 0.098891 | 0.053167 | 0 | 0.029518 |
| 0.06257 | 0.134558 | 0 | 0.074706 |
| 2.472525 | 2.272674 | 2.10052 | 1.809331 |
| 14.65761 | 12.70877 | 12.35018 | 12.84713 |
| 0.009287 | 0 | 0.011376 | 0 |
| 1.742901 | 1.454275 | 1.36635 | 1.431682 |
| 0.047568 | 0.051148 | 0.116536 | 0 |
| 4.137968 | 3.163065 | 2.570382 | 3.067336 |
| 11.72087 | 9.302178 | 9.144159 | 9.829199 |
| 0 | 0 | 0 | 0 |
| 0 | 0 | 0 | 0 |
| 0 | 0.77575 | 0 | 0 |
| 7.523108 | 6.745135 | 6.180613 | 6.322818 |
| 41.61466 | 46.26362 | 46.08934 | 43.98041 |
| 0 | 0 | 0.03226 | 0 |
| 3.417887 | 2.688078 | 2.775153 | 2.821568 |
| 508.8227 | 402.9103 | 414.9345 | 391.1919 |
| 2.936566 | 2.452983 | 2.839011 | 2.376039 |
| 4.467072 | 4.662496 | 4.048751 | 3.614244 |
| 32.59173 | 28.85593 | 29.67538 | 30.24334 |
| 1.548149 | 1.574677 | 1.025063 | 1.198968 |
| 0 | 0.012358 | 0.014078 | 0 |
| 0.373826 | 0.301469 | 0.40067 | 0.334746 |
| 0.651637 | 0.383704 | 0.532137 | 0.463108 |
| 16.92371 | 15.596 | 15.90959 | 17.57046 |
| 0.107729 | 0 | 0 | 0 |
| 6.076453 | 5.980041 | 5.088286 | 6.435199 |
| 11.96634 | 13.09027 | 12.67294 | 13.34463 |
| 0.674873 | 0.645033 | 0.275556 | 0.58194 |
| 52.70707 | 54.84893 | 51.96766 | 52.44125 |
| 0.731561 | 1.133653 | 0.975176 | 0.719309 |
| 70.51824 | 73.43616 | 69.4637 | 67.25814 |
| 66.86426 | 63.34215 | 64.12539 | 62.64276 |
| 1.721062 | 0 | 0 | 2.054859 |

| | | | |
|---|---|---|---|
| 10.51866 | 9.382528 | 9.674939 | 8.606997 |
| 0.968655 | 0.816904 | 0.79102 | 0.702985 |
| 0 | 0 | 0.214961 | 0.069842 |
| 54.58584 | 58.76384 | 54.49617 | 60.35081 |
| 82.95374 | 83.17041 | 88.51097 | 80.29641 |
| 6.548331 | 9.146222 | 7.993647 | 7.791501 |
| 6.159961 | 4.770239 | 5.434219 | 4.759959 |
| 0 | 0 | 0 | 0.24192 |
| 0 | 0.019321 | 0 | 0 |
| 0.018564 | 0 | 0 | 0 |
| 6.729463 | 5.591379 | 5.870075 | 5.234683 |
| 0.179307 | 0.171379 | 0.146425 | 0.142722 |
| 0 | 0 | 0 | 0.130692 |
| 0.277718 | 0.135736 | 0.092777 | 0.120575 |
| 0.566376 | 0.6235 | 0.396439 | 0.57962 |
| 12.02241 | 10.18505 | 10.53171 | 12.87523 |
| 0 | 0 | 0 | 0 |
| 0.241706 | 0.043316 | 0.049345 | 0 |
| 0.224604 | 0.080502 | 0.183415 | 0.089389 |
| 0 | 0 | 0 | 0.021174 |
| 0 | 0 | 1.230508 | 0 |
| 0 | 0.070766 | 0 | 0 |
| 0 | 0 | 0.474196 | 0 |
| 0 | 0 | 0 | 0 |
| 5.507194 | 5.944424 | 4.332941 | 5.664877 |
| 58.13247 | 52.2596 | 50.54329 | 50.35507 |
| 1.76889 | 0.729841 | 0.907014 | 0.933193 |
| 0.253727 | 0.454702 | 0 | 0.403915 |
| 39.01565 | 41.19247 | 41.31269 | 43.13487 |
| 0 | 0 | 0 | 0 |
| 1.649392 | 1.608767 | 1.633969 | 1.646454 |
| 0 | 0 | 0 | 0 |
| 3.380629 | 3.326185 | 3.27492 | 2.690864 |
| 2.179653 | 1.766459 | 1.853885 | 1.482669 |
| 0.399918 | 0.2913 | 0.252836 | 0.277247 |
| 0.585786 | 0.449908 | 0.538158 | 0.374678 |
| 0 | 0 | 0 | 0 |
| 8.791994 | 8.096445 | 7.917203 | 7.353226 |
| 189.944 | 187.4094 | 188.5606 | 193.4744 |
| 17.15202 | 16.03425 | 17.16856 | 16.12309 |
| 5.742698 | 6.060761 | 5.445499 | 5.476739 |
| 0.596588 | 0.835423 | 0.254922 | 0.72886 |
| 0.032816 | 0 | 0 | 0 |
| 26.53974 | 28.65314 | 27.85367 | 28.92831 |
| 0.019372 | 0 | 0 | 0 |
| 0.100775 | 0.054179 | 0 | 0 |
| 0 | 0 | 0 | 0.06113 |
| 0.553675 | 0.457956 | 0.36519 | 0.508507 |
| 0 | 0.290906 | 0 | 0 |
| 0.059273 | 0.021245 | 0.266219 | 0.047179 |
| 32.94783 | 30.00157 | 30.57195 | 27.05226 |
| 7.933836 | 6.66784 | 7.133173 | 6.906124 |
| 0 | 0 | 0 | 0.032174 |
| 0.511526 | 0.606921 | 0.475337 | 0.515967 |

| | | | |
|---|---|---|---|
| 94.97655 | 78.55473 | 78.18544 | 76.95407 |
| 0 | 0 | 0 | 0.199478 |
| 6.658031 | 6.248104 | 5.964689 | 6.416694 |
| 0 | 0 | 0 | 0 |
| 4.00645 | 4.83014 | 4.253252 | 3.855785 |
| 0 | 0 | 0 | 0 |
| 2.697615 | 2.023695 | 2.228532 | 1.797663 |
| 2.88434 | 2.5407 | 2.395321 | 2.354203 |
| 0.100456 | 0 | 0 | 0 |
| 0.038215 | 0 | 0.046811 | 0.045627 |
| 0.0744 | 0 | 0.045567 | 0.08883 |
| 49.50557 | 28.52035 | 27.57179 | 29.69454 |
| 1.05243 | 0.662419 | 0.282984 | 0.704892 |
| 0.063573 | 0.205071 | 0.584039 | 0.417465 |
| 0 | 0 | 0 | 0 |
| 0 | 0 | 0 | 0 |
| 0 | 0 | 0.545103 | 0 |
| 2.329046 | 1.947807 | 0.950968 | 0.617947 |
| 0.134509 | 0 | 0.164763 | 0 |
| 7.650148 | 6.923201 | 6.903084 | 7.362362 |
| 295.9664 | 282.8917 | 294.0982 | 271.1721 |
| 62.65755 | 58.49244 | 57.64785 | 60.26656 |
| 0.436044 | 0.46886 | 0.534121 | 0.104123 |
| 0.391902 | 2.52837 | 3.120324 | 0 |
| 6.411077 | 6.710945 | 6.812941 | 5.905619 |
| 0.785096 | 0.759762 | 0.769346 | 0.328077 |
| 0 | 0 | 0 | 0 |
| 31.84738 | 32.6367 | 33.47645 | 32.94719 |
| 0.455656 | 0.244974 | 0.186048 | 0.362686 |
| 12.88283 | 11.39738 | 5.11041 | 6.53574 |
| 0.280094 | 0.953716 | 0.914919 | 0.947518 |
| 34.40084 | 32.94885 | 34.41895 | 32.85825 |
| 1.234225 | 1.182858 | 1.205084 | 1.324105 |
| 19.96802 | 20.03 | 19.61549 | 17.55867 |
| 0.059632 | 0 | 0.036522 | 0.071197 |
| 10.36801 | 13.02926 | 13.01361 | 13.7543 |
| 0.292123 | 0 | 0.357829 | 0 |
| 0 | 0 | 0 | 0 |
| 6.05382 | 5.586259 | 5.68969 | 5.466956 |
| 0 | 0 | 0 | 0 |
| 0 | 0.069707 | 0 | 0 |
| 3.842299 | 2.754306 | 3.381726 | 3.092319 |
| 0 | 0 | 0 | 0 |
| 0 | 0 | 0 | 0 |
| 3.592385 | 3.133919 | 2.532306 | 3.43939 |
| 0.099754 | 0 | 0.122191 | 0.0397 |
| 0.015365 | 0.033043 | 0 | 0 |
| 0.038354 | 0.08248 | 0.093961 | 0.091585 |
| 0.975841 | 0.914757 | 1.256631 | 1.135229 |
| 24.92884 | 29.42555 | 27.99282 | 28.75785 |
| 0.634435 | 0.574468 | 0.572626 | 0.598013 |
| 1.029179 | 1.461972 | 1.353192 | 1.465525 |
| 0 | 0 | 0.566272 | 0 |
| 0.796921 | 1.231787 | 0.793137 | 1.248821 |

| | | | |
|---|---|---|---|
| 0.135658 | 0.145867 | 0.332342 | 0.323938 |
| 23.00755 | 20.03862 | 20.73763 | 19.53791 |
| 2.709679 | 2.396672 | 2.542898 | 2.008965 |
| 8.46267 | 8.447915 | 8.934492 | 8.889443 |
| 13.49894 | 11.90028 | 12.71229 | 13.13905 |
| 53.83141 | 64.77968 | 65.49495 | 63.46135 |
| 47.33625 | 53.46955 | 53.99523 | 53.81417 |
| 0.018542 | 0 | 0 | 0 |
| 5.596401 | 4.412884 | 5.255629 | 4.844314 |
| 0 | 0 | 0 | 0 |
| 13.39991 | 12.47516 | 13.21369 | 13.68451 |
| 0 | 0.106003 | 0.241516 | 0 |
| 0.01239 | 0.026646 | 0 | 0.04438 |
| 2.486096 | 1.572466 | 1.540553 | 1.431753 |
| 0.024519 | 0.026364 | 0.060068 | 0.102461 |
| 6.980155 | 6.109758 | 5.792045 | 6.246503 |
| 1.840437 | 1.463875 | 1.369108 | 1.495025 |
| 2.216554 | 1.791811 | 1.679853 | 1.723049 |
| 3.242149 | 3.278016 | 3.289732 | 2.94655 |
| 2.421155 | 1.735576 | 1.977155 | 0 |
| 0.126976 | 0.136532 | 0.103691 | 0.050534 |
| 0.020288 | 0.109074 | 0.024851 | 0 |
| 18.55591 | 17.48943 | 14.82241 | 16.76803 |
| 2.517732 | 1.804806 | 1.514963 | 1.687602 |
| 0 | 0 | 0 | 0 |
| 8.247312 | 6.112503 | 5.603811 | 6.205463 |
| 0.136828 | 0.147125 | 0.167604 | 0 |
| 10.92465 | 10.08565 | 7.907473 | 10.86956 |
| 2.289992 | 2.292514 | 3.482153 | 2.686992 |
| 5.831571 | 6.924744 | 6.211508 | 7.931302 |
| 0.143289 | 0.237035 | 0.135014 | 0.1316 |
| 1.236932 | 0.947325 | 0.986276 | 1.17032 |
| 8.301148 | 7.414394 | 7.639783 | 7.522185 |
| 1.322668 | 1.422208 | 0 | 0.526399 |
| 0.044563 | 0.047917 | 0 | 0 |
| 43.54362 | 38.45575 | 40.29103 | 40.39419 |
| 0.02275 | 0.012231 | 0.222938 | 0.013581 |
| 9.37481 | 8.484662 | 7.56823 | 7.819793 |
| 4.597411 | 3.938422 | 3.279874 | 3.257251 |
| 0 | 0.379255 | 0.432045 | 0 |
| 2.471673 | 2.814018 | 3.11666 | 1.475524 |
| 0 | 0 | 0 | 0 |
| 209.9885 | 226.9823 | 213.886 | 228.4133 |
| 0.305174 | 0.32814 | 0.318842 | 0.364361 |
| 6.982696 | 7.188695 | 7.825337 | 5.232783 |
| 0.620405 | 0.800513 | 0.455969 | 0.814804 |
| 0.102034 | 0.109713 | 0.166646 | 0 |
| 0.256 | 0.412899 | 0.627162 | 0.152826 |
| 3.987717 | 5.983569 | 5.298606 | 5.433603 |
| 0 | 0 | 0.039145 | 0 |
| 1.242903 | 1.059935 | 0.262494 | 0.392312 |
| 9.578166 | 10.38341 | 9.568743 | 9.654843 |
| 407.5536 | 338.0037 | 332.4089 | 354.7807 |
| 4.474173 | 4.402689 | 3.653683 | 3.658413 |

| | | | |
|---|---|---|---|
| 1.93038 | 0.691885 | 0.78819 | 1.536516 |
| 18.78783 | 18.96358 | 19.59874 | 19.51316 |
| 0.028009 | 0.060235 | 0.034309 | 0.100325 |
| 0 | 0 | 0 | 0.562882 |
| 10.41658 | 12.95057 | 13.77851 | 14.03464 |
| 13.73277 | 8.264071 | 8.315227 | 9.385903 |
| 1.795952 | 2.206983 | 1.899918 | 1.824025 |
| 15.20766 | 14.6593 | 14.37465 | 14.62437 |
| 0.063943 | 0.114592 | 0.182758 | 0.076344 |
| 17.03482 | 15.49884 | 15.29188 | 15.85657 |
| 0.820966 | 0 | 0 | 0 |
| 13.46674 | 13.44268 | 13.15548 | 13.67431 |
| 18.26811 | 13.9419 | 13.88418 | 13.65481 |
| 0 | 0 | 0 | 0 |
| 8.756974 | 11.77 | 12.41863 | 11.35777 |
| 0 | 0 | 0 | 0 |
| 5.237765 | 5.734342 | 5.016041 | 4.434385 |
| 1.579679 | 1.213258 | 0.82928 | 2.694364 |
| 248.4822 | 289.9376 | 287.9506 | 281.4182 |
| 0 | 0 | 0 | 0.053082 |
| 0 | 0 | 0 | 0 |
| 0 | 0 | 0.032269 | 0.015726 |
| 0.283429 | 0 | 0 | 0 |
| 21.48477 | 21.2911 | 21.19998 | 20.91835 |
| 2.07387 | 1.427163 | 1.37178 | 1.782787 |
| 0.129111 | 0.180475 | 0.173966 | 0.184982 |
| 0 | 0 | 0 | 0 |
| 5.557709 | 6.310527 | 5.27011 | 4.816586 |
| 0.435134 | 0.41775 | 0.380718 | 0.445309 |
| 7.964709 | 6.758703 | 6.381061 | 6.039786 |
| 0 | 0.089824 | 0 | 0 |
| 0 | 0 | 0 | 0 |
| 0.155608 | 0.167319 | 0 | 0 |
| 0.881779 | 0 | 0 | 0 |
| 9.514935 | 7.31739 | 7.499789 | 7.038471 |
| 1.925377 | 7.245962 | 40.63775 | 1.503061 |
| 29.82403 | 25.18684 | 23.67536 | 21.16633 |
| 7.663491 | 6.748458 | 6.939243 | 6.862358 |
| 0.631672 | 0.614523 | 0.773751 | 1.005578 |
| 0.44501 | 0 | 0 | 0.531319 |
| 0.251937 | 0.135448 | 0 | 0.1504 |
| 0 | 0.121039 | 0 | 0 |
| 0.311216 | 0.334637 | 1.143648 | 0.371576 |
| 7.958021 | 6.142518 | 6.673925 | 6.938829 |
| 0.350421 | 0.421121 | 0.302993 | 0.467606 |
| 0 | 0 | 0 | 0 |
| 0 | 0.531115 | 0 | 0 |
| 1.083253 | 1.46863 | 0.980755 | 1.349581 |
| 101.0136 | 98.64552 | 100.9515 | 96.70546 |
| 0 | 0.483014 | 0.550246 | 0 |
| 2.867841 | 2.232999 | 2.24098 | 2.125274 |
| 1.258659 | 1.259686 | 1.435025 | 1.213779 |
| 0.701824 | 0.406345 | 0.264517 | 0.451199 |
| 0.501855 | 0.518039 | 0.368841 | 0.383481 |

| | | | |
|---|---|---|---|
| 0 | 0 | 0 | 0 |
| 63.10835 | 61.57004 | 56.46558 | 63.01676 |
| 7.350128 | 5.770807 | 5.676026 | 5.835485 |
| 1.101898 | 1.083987 | 1.09128 | 1.147659 |
| 18.61972 | 14.00773 | 14.88839 | 14.89784 |
| 21.4124 | 23.42217 | 25.71429 | 26.4794 |
| 8.068926 | 7.02837 | 7.240476 | 7.094717 |
| 0.026062 | 0.028024 | 0 | 0.031117 |
| 2.620762 | 1.486283 | 1.869251 | 1.782373 |
| 0.781231 | 0.378011 | 0.669865 | 0.792837 |
| 1.429234 | 0.889723 | 1.382134 | 1.167558 |
| 6.561818 | 4.340644 | 4.467528 | 3.963758 |
| 0 | 0.501956 | 0 | 0 |
| 1.489792 | 0.369671 | 1.333568 | 0.410477 |
| 0 | 0.126107 | 0 | 0 |
| 0.016614 | 0.053593 | 0.020351 | 0.039673 |
| 13.16248 | 12.53923 | 12.16957 | 11.70324 |
| 0.046557 | 0.02503 | 0.028514 | 0.027793 |
| 0.115854 | 0.124573 | 0.070956 | 0.069162 |
| 4.199747 | 5.004002 | 4.727261 | 4.675477 |
| 15.05574 | 10.53247 | 10.66534 | 8.554735 |
| 27.55231 | 28.22808 | 31.84568 | 29.1172 |
| 14.13121 | 13.15772 | 12.54324 | 11.67587 |
| 0 | 0.05727 | 0 | 0 |
| 40.9409 | 34.29249 | 27.61857 | 29.22252 |
| 0.154311 | 0.118517 | 0.054006 | 0.05264 |
| 4.369554 | 3.361301 | 3.236811 | 3.691093 |
| 23.99461 | 24.43612 | 24.18063 | 24.23745 |
| 14.23152 | 12.5218 | 11.5001 | 11.75197 |
| 5.256783 | 4.82286 | 4.637076 | 4.626916 |
| 11.84864 | 5.00088 | 5.222217 | 5.387632 |
| 0 | 0.390836 | 0 | 0 |
| 141.3563 | 127.9542 | 125.9163 | 122.4352 |
| 0 | 0 | 0 | 0 |
| 9.784119 | 6.229752 | 6.344898 | 6.917417 |
| 16.91941 | 22.87084 | 22.07975 | 22.31718 |
| 0.360728 | 0 | 0 | 0 |
| 2.742344 | 3.237579 | 3.13979 | 3.267534 |
| 1.425042 | 1.743636 | 1.745568 | 1.408076 |
| 0.406028 | 0.079379 | 0.135642 | 0.132212 |
| 3.115754 | 2.680189 | 3.008349 | 2.49462 |
| 12.54645 | 16.74142 | 16.75717 | 16.15293 |
| 0 | 0 | 0 | 0 |
| 0.393373 | 0.403751 | 0.131414 | 0.256182 |
| 0 | 0 | 0.128471 | 0 |
| 8.160264 | 7.032461 | 7.355332 | 7.45706 |
| 1.719286 | 1.443727 | 0.90257 | 1.192543 |
| 25.00288 | 8.744182 | 10.3982 | 9.794569 |
| 82.16453 | 86.79746 | 84.94311 | 89.76488 |
| 1.089256 | 1.070839 | 1.219891 | 0.817467 |
| 1.151368 | 1.172364 | 0.929542 | 1.239289 |
| 14.91272 | 18.56685 | 18.37378 | 17.80501 |
| 81.28366 | 81.3205 | 82.07208 | 81.1738 |
| 17.68396 | 20.04331 | 20.60416 | 18.10544 |

| | | | |
|---|---|---|---|
| 0 | 0 | 0 | 0.033305 |
| 0.022914 | 0 | 0.056137 | 0.027359 |
| 0 | 0 | 0 | 0 |
| 0 | 0 | 0 | 0.076516 |
| 2.604429 | 1.935376 | 2.505415 | 1.660599 |
| 4.299702 | 3.657106 | 3.893255 | 3.856866 |
| 5.435309 | 4.714773 | 4.252068 | 4.199073 |
| 1.37354 | 0 | 0 | 1.09329 |
| 16.84896 | 15.0535 | 13.77584 | 14.25753 |
| 0.387122 | 0.138752 | 0 | 0.154068 |
| 5.054538 | 3.746806 | 4.291785 | 3.748926 |
| 9.185343 | 8.488354 | 9.008949 | 9.409994 |
| 7.715728 | 6.798431 | 7.219655 | 7.335624 |
| 0 | 0 | 0.167604 | 0 |
| 0.979128 | 1.011928 | 0.85003 | 0.726386 |
| 0 | 0 | 0 | 0 |
| 3.69938 | 3.466079 | 3.244614 | 3.644988 |
| 36.19771 | 38.11186 | 36.52056 | 33.60889 |
| 0.162512 | 0.058247 | 0.199065 | 0.129354 |
| 7.809192 | 7.923217 | 7.43179 | 7.482923 |
| 0.076553 | 0.041157 | 0 | 0.0457 |
| 2.152506 | 0.806567 | 1.078633 | 1.401808 |
| 6.404497 | 2.095885 | 2.387616 | 3.657088 |
| 0.119338 | 0 | 0.116944 | 0.028497 |
| 6.7406 | 7.873738 | 8.095731 | 7.554738 |
| 2514.92 | 2372.102 | 2235.553 | 2321.123 |
| 0.910307 | 0.45176 | 0.314503 | 0.752441 |
| 80.82922 | 78.35073 | 77.81628 | 76.77246 |
| 3.627889 | 3.732124 | 3.963181 | 4.008731 |
| 12.53454 | 10.53723 | 11.52005 | 11.28315 |
| 0.021973 | 0.023627 | 0 | 0 |
| 0.104421 | 0.149706 | 0.170544 | 0.083116 |
| 1.077286 | 1.158359 | 0.439865 | 0.514489 |
| 0.366277 | 0 | 0 | 0 |
| 0 | 0 | 0 | 0 |
| 0 | 0.819192 | 0.466608 | 0 |
| 0 | 0 | 0 | 0 |
| 0.341334 | 0.917554 | 0 | 0.611302 |
| 22.01119 | 19.32056 | 20.90934 | 20.55936 |
| 5.954361 | 5.146288 | 6.162666 | 5.691858 |
| 1.351875 | 0.922929 | 1.340527 | 1.204147 |
| 0.123705 | 0.155184 | 0.227294 | 0.147697 |
| 0.053084 | 0.114157 | 0.130047 | 0.063379 |
| 4.780575 | 4.528401 | 4.089795 | 4.031671 |
| 9.653502 | 9.552143 | 9.309922 | 9.498751 |
| 0.076924 | 0 | 0 | 0 |
| 3.999748 | 4.198358 | 3.907846 | 4.074328 |
| 4.016991 | 2.458137 | 2.800291 | 3.470334 |
| 0.964598 | 0.84861 | 1.074145 | 0.907384 |
| 8.405288 | 7.677999 | 7.745631 | 7.663715 |
| 56.53528 | 32.82406 | 30.91224 | 32.14295 |
| 0 | 0 | 0 | 0.193371 |
| 5.626587 | 5.034165 | 4.371883 | 4.537058 |
| 3.547432 | 3.38184 | 3.314999 | 3.340329 |

| | | | |
|---|---|---|---|
| 0 | 0.742022 | 0 | 0 |
| 0.104421 | 0.336839 | 0.383724 | 0.748041 |
| 16.32626 | 15.14149 | 15.54707 | 15.34533 |
| 14.04813 | 8.756727 | 8.962449 | 8.3256 |
| 11.47981 | 9.507254 | 9.206814 | 10.63584 |
| 0.177506 | 0.167007 | 0.244611 | 0.264917 |
| 11.02819 | 9.955456 | 10.46541 | 10.39282 |
| 14.04888 | 11.64673 | 9.589645 | 14.98103 |
| 8.012932 | 5.996709 | 6.266047 | 5.969823 |
| 0.202837 | 0.109051 | 0.24846 | 0.121089 |
| 23.15542 | 33.05875 | 32.03492 | 32.11942 |
| 0 | 0 | 0 | 0 |
| 43.48163 | 27.30841 | 27.94001 | 26.56185 |
| 4.137477 | 4.177395 | 3.968577 | 3.9352 |
| 3.136386 | 3.188471 | 3.56243 | 4.357449 |
| 42.85026 | 48.2327 | 50.03034 | 48.21611 |
| 7.7804 | 7.234537 | 7.206799 | 6.299093 |
| 97.72938 | 90.36456 | 91.60194 | 92.23075 |
| 38.0422 | 34.06493 | 31.98338 | 34.53765 |
| 15.38246 | 13.94964 | 14.54995 | 13.41292 |
| 11.44753 | 10.12233 | 9.723242 | 9.882038 |
| 1.739603 | 1.618719 | 1.529864 | 1.065126 |
| 2.90785 | 0.928235 | 0.667856 | 0.922203 |
| 25.07389 | 26.12326 | 27.01606 | 25.69344 |
| 19.29131 | 18.75576 | 18.64962 | 17.95733 |
| 0 | 0 | 0 | 1.421277 |
| 5.685216 | 5.483483 | 5.552659 | 5.479895 |
| 3.520595 | 3.572607 | 3.593719 | 3.44154 |
| 3.017254 | 2.230473 | 2.232945 | 2.063901 |
| 1009.678 | 1062.057 | 1037.625 | 1049.749 |
| 0.851847 | 1.05687 | 0.856162 | 1.017061 |
| 0 | 0.409596 | 0 | 0.909617 |
| 0.431991 | 0.46972 | 0.303224 | 0.486798 |
| 22.39533 | 20.41725 | 21.07067 | 21.52999 |
| 0 | 0.046843 | 0 | 0.052014 |
| 7.478565 | 6.552235 | 6.137962 | 5.802358 |
| 3.162376 | 3.354725 | 3.522702 | 3.357597 |
| 3.482313 | 3.231992 | 3.098151 | 2.713447 |
| 9.110537 | 7.270327 | 7.465736 | 7.011634 |
| 0 | 0.336839 | 0 | 0 |
| 11.36515 | 9.059991 | 10.5611 | 9.124249 |
| 0 | 0 | 0 | 0.020924 |
| 0.429457 | 0.577221 | 0.065757 | 0.833218 |
| 16.74953 | 19.21011 | 18.34625 | 18.30953 |
| 0 | 0 | 0 | 0 |
| 0.700236 | 0 | 0 | 0 |
| 0 | 0 | 0.259227 | 0 |
| 29.13878 | 25.87987 | 25.84905 | 23.95115 |
| 26.83685 | 30.14408 | 32.55676 | 30.0795 |
| 0 | 0 | 0 | 0 |
| 4.007763 | 4.035764 | 3.428653 | 3.569808 |
| 0.295019 | 0.465258 | 0.192734 | 0.399202 |
| 53.9566 | 46.74302 | 47.16852 | 47.10202 |
| 0.602735 | 0 | 0.738305 | 0 |

| | | | |
|---|---|---|---|
| 0.200912 | 0.493786 | 0.316416 | 0.445488 |
| 13.22753 | 12.71375 | 11.9418 | 12.25914 |
| 14.05278 | 11.70174 | 12.20964 | 11.58873 |
| 0.411592 | 0.276604 | 0.220574 | 0.644988 |
| 158.5121 | 161.2482 | 150.3635 | 164.0709 |
| 0.718733 | 0.772822 | 0.660295 | 0.643597 |
| 0 | 0.03316 | 0 | 0 |
| 0.032319 | 0.16217 | 0.145155 | 0.11576 |
| 28.3502 | 26.80305 | 26.35872 | 26.37332 |
| 3.174403 | 4.778619 | 1.555362 | 5.306102 |
| 0 | 0.023703 | 0 | 0 |
| 1.076069 | 0.92564 | 0.762617 | 0.92687 |
| 0.645175 | 0.500395 | 0.583001 | 0.74505 |
| 0 | 0.00998 | 0 | 0.022164 |
| 2.884888 | 2.384452 | 2.464836 | 2.010258 |
| 0.283429 | 0 | 0 | 0.676799 |
| 0.694944 | 0.613807 | 0.547234 | 0.740827 |
| 0.585243 | 0.314643 | 0.557572 | 0.562882 |
| 0 | 0 | 0.141913 | 0 |
| 0 | 0 | 0 | 0 |
| 0 | 0 | 0.088776 | 0.173063 |
| 7.149894 | 6.520689 | 6.373681 | 6.927609 |
| 0.061072 | 0.043779 | 0 | 0.583337 |
| 3.812188 | 3.440301 | 3.235395 | 3.43534 |
| 30.33765 | 32.18871 | 29.77828 | 27.82585 |
| 0.090225 | 0.194029 | 0.165778 | 0.107724 |
| 1.899137 | 1.834895 | 2.663444 | 1.90599 |
| 7.945366 | 7.879166 | 8.288078 | 9.184669 |
| 16.764 | 18.87188 | 20.30325 | 18.88772 |
| 3.644318 | 3.857159 | 3.834295 | 3.710052 |
| 10.17454 | 11.16141 | 11.11093 | 10.46977 |
| 8.370857 | 14.37794 | 5.992403 | 11.48703 |
| 0.936063 | 0.158922 | 0.181043 | 0.911735 |
| 4.602294 | 3.90182 | 4.011272 | 3.768938 |
| 0.41226 | 0.332464 | 0.631234 | 1.107489 |
| 0 | 0 | 0 | 0 |
| 19.29866 | 20.54632 | 21.16178 | 19.97317 |
| 22.98915 | 21.05049 | 20.89708 | 20.6221 |
| 1.310217 | 0.630262 | 0.63352 | 1.234998 |
| 9.767394 | 8.372793 | 8.591046 | 9.086456 |
| 6.223442 | 6.234446 | 5.731145 | 6.895901 |
| 2.33412 | 0 | 1.143648 | 0 |
| 0.062081 | 0 | 0 | 0 |
| 7.615918 | 8.141597 | 7.868745 | 7.221696 |
| 0.802723 | 0.745433 | 0.715109 | 0.784153 |
| 11.85285 | 10.59533 | 11.45312 | 10.31777 |
| 0.198282 | 0.192899 | 0.323841 | 0.360745 |
| 0.872545 | 0.893534 | 0.865221 | 0.793732 |
| 14.12906 | 12.50081 | 12.26483 | 10.82562 |
| 1.44291 | 0 | 0 | 0 |
| 84.57914 | 72.02985 | 68.03159 | 76.45023 |
| 44.95221 | 40.72686 | 39.86974 | 43.33405 |
| 0.102731 | 0 | 0.062919 | 0.061328 |
| 2.506108 | 0.673677 | 0 | 0 |

| | | | |
|---|---|---|---|
| 0 | 0 | 0 | 0 |
| 0.078922 | 0 | 0.032224 | 0.062819 |
| 16.9274 | 14.11598 | 14.88894 | 15.61884 |
| 0.201573 | 0.168578 | 0.137173 | 0.213927 |
| 5.330537 | 6.393047 | 5.148264 | 5.630032 |
| 0 | 0.595343 | 0 | 0 |
| 70.22208 | 72.9219 | 74.4154 | 71.44425 |
| 24.26566 | 25.38231 | 24.50894 | 24.44825 |
| 0 | 0 | 0 | 0 |
| 24.22464 | 19.02226 | 18.18489 | 18.19817 |
| 24.11935 | 23.53837 | 23.16183 | 22.34134 |
| 2.43231 | 2.461514 | 2.103103 | 2.220746 |
| 0 | 0 | 0 | 0 |
| 0.329143 | 0.275266 | 0.403176 | 0.261987 |
| 17.61518 | 17.57208 | 17.15224 | 16.55418 |
| 6.828658 | 4.498146 | 4.898183 | 4.994669 |
| 6.587388 | 6.138149 | 6.216662 | 6.305234 |
| 53.78557 | 39.23593 | 38.4319 | 38.71918 |
| 0.125968 | 0 | 0 | 0 |
| 0.620761 | 0.392634 | 0.402557 | 0.305182 |
| 0.213312 | 0.15291 | 0.174194 | 0.198087 |
| 336.8078 | 329.4975 | 319.9846 | 328.4008 |
| 4.471795 | 3.333998 | 3.168235 | 3.441574 |
| 0.836036 | 0.122585 | 0.139647 | 0.181488 |
| 0 | 0 | 0 | 0 |
| 2.41965 | 1.933407 | 1.631498 | 2.014304 |
| 12.12262 | 10.08604 | 10.71096 | 10.77228 |
| 0.07242 | 0 | 0.044354 | 0 |
| 0 | 0.211568 | 0 | 0 |
| 12.01034 | 10.69287 | 10.81674 | 11.26865 |
| 9.949827 | 8.552978 | 8.597194 | 9.299918 |
| 0.116649 | 0.050171 | 0 | 0 |
| 4.706195 | 4.247925 | 4.010633 | 4.347386 |
| 0.013143 | 0.014132 | 0 | 0 |
| 22.91759 | 26.21188 | 25.85514 | 24.36475 |
| 11.75171 | 11.89654 | 11.33783 | 10.34723 |
| 55.74361 | 53.61822 | 54.22312 | 52.45983 |
| 0 | 0 | 0 | 0 |
| 0 | 0.051874 | 0.059094 | 0 |
| 0.088698 | 0 | 0.018108 | 0 |
| 0.749122 | 0.402749 | 0.229404 | 0.391305 |
| 30.36543 | 30.22233 | 28.16315 | 28.88035 |
| 0 | 0.145041 | 0 | 0 |
| 0 | 0 | 0 | 0 |
| 10.44348 | 12.0077 | 10.76594 | 9.814683 |
| 14.53234 | 11.64795 | 10.88927 | 11.85325 |
| 10.81901 | 11.09989 | 9.72101 | 10.03037 |
| 2.870225 | 2.490268 | 2.19435 | 2.375196 |
| 0 | 0 | 0 | 0 |
| 0 | 0 | 0.627162 | 0 |
| 0.996673 | 1.152055 | 1.25137 | 1.070978 |
| 13.03048 | 11.1958 | 10.62847 | 10.86859 |
| 1.095767 | 0.723005 | 0.488084 | 0.698745 |
| 0.056855 | 0 | 0 | 0 |

| | | | |
|---|---|---|---|
| 16.63167 | 13.8687 | 14.04696 | 14.09699 |
| 0 | 0 | 0 | 0 |
| 13.7354 | 16.55596 | 14.70614 | 14.41523 |
| 0 | 0 | 0 | 0.536331 |
| 0 | 0 | 0 | 0 |
| 0.118448 | 0 | 0 | 0.424262 |
| 0 | 0.03665 | 0.041751 | 0.08139 |
| 28.93668 | 32.49302 | 31.47267 | 32.62784 |
| 0.595201 | 0.426662 | 0.526555 | 0.671159 |
| 8.620146 | 7.980058 | 7.622613 | 6.861338 |
| 0.053925 | 0.115967 | 0.033027 | 0.032192 |
| 0.505785 | 0.155385 | 0.336326 | 0.379582 |
| 19.30019 | 17.54522 | 17.53327 | 17.14303 |
| 0 | 0 | 0.032676 | 0 |
| 1.269984 | 1.311656 | 1.586338 | 1.426514 |
| 24.00226 | 24.67481 | 24.43848 | 22.32962 |
| 36.97164 | 33.80059 | 34.21569 | 33.7636 |
| 22.8557 | 22.47593 | 21.27794 | 21.96323 |
| 0.125968 | 0.225747 | 0.051434 | 0.200533 |
| 169.2976 | 162.1004 | 160.7788 | 165.9305 |
| 6.520555 | 6.514561 | 6.659617 | 6.17301 |
| 0 | 0.228569 | 1.041537 | 0 |
| 4.487681 | 2.851379 | 2.33804 | 2.48767 |
| 59.93548 | 55.05023 | 54.29026 | 55.27759 |
| 52.08919 | 29.44308 | 26.52581 | 28.4505 |
| 7.800927 | 8.796507 | 7.787149 | 8.951022 |
| 0.442059 | 0.316885 | 0.309422 | 0.150799 |
| 0.791327 | 0.586814 | 0.584932 | 0.504981 |
| 0.06641 | 0.071408 | 0 | 0 |
| 15.17265 | 13.89225 | 13.63466 | 13.52718 |
| 0 | 0 | 0 | 0 |
| 0 | 0 | 0 | 0.03551 |
| 13.59681 | 11.66682 | 11.1589 | 11.72087 |
| 15.83382 | 14.16733 | 14.25873 | 14.88306 |
| 8.598722 | 7.989899 | 8.88894 | 9.435619 |
| 0.116449 | 0.056346 | 0.03566 | 0.069517 |
| 5.620437 | 4.467739 | 4.544298 | 4.828024 |
| 30.60761 | 30.42642 | 29.23263 | 28.62907 |
| 7.728825 | 7.38063 | 7.216278 | 6.614343 |
| 133.9513 | 134.3126 | 134.8736 | 132.6525 |
| 11.04403 | 7.743733 | 8.230209 | 8.214226 |
| 5.915975 | 5.519922 | 5.245815 | 5.703136 |
| 3.990171 | 3.265517 | 2.986902 | 4.287652 |
| 14.0596 | 12.69609 | 13.51746 | 14.21277 |
| 1.32752 | 1.126915 | 1.027018 | 1.147032 |
| 24.42686 | 25.39642 | 24.34113 | 24.03344 |
| 0.075104 | 0.016151 | 0.073598 | 0.035868 |
| 0.652632 | 0.294734 | 0.511632 | 0.451941 |
| 80.83654 | 72.43338 | 82.74162 | 80.86957 |
| 4.050328 | 2.974244 | 3.236975 | 2.299992 |
| 0 | 0.182464 | 0.041572 | 0.324169 |
| 3.839199 | 2.841437 | 2.992646 | 2.649082 |
| 5.1881 | 4.724885 | 4.206532 | 4.188331 |
| 0 | 0 | 0 | 0 |

| | | | |
|---|---|---|---|
| 4.368267 | 4.354087 | 4.332725 | 3.773149 |
| 8.553132 | 11.89765 | 12.43971 | 13.77973 |
| 53.01128 | 57.5404 | 52.17839 | 51.4581 |
| 0 | 0 | 0 | 0.465993 |
| 40.95646 | 27.7317 | 28.0321 | 26.74495 |
| 20.10836 | 16.98055 | 17.24605 | 16.07372 |
| 2.925805 | 3.145993 | 3.162256 | 3.322021 |
| 191.4959 | 235.4323 | 195.9756 | 254.1244 |
| 0 | 0.568883 | 0.162017 | 0.15792 |
| 1.929931 | 1.377484 | 1.365425 | 1.608993 |
| 3.549652 | 4.52562 | 10.93225 | 2.482316 |
| 0 | 0 | 0.052976 | 0 |
| 39.4309 | 41.16345 | 40.2164 | 38.32878 |
| 43.15753 | 40.7376 | 42.93745 | 39.01957 |
| 25.56373 | 23.74839 | 22.37876 | 23.67614 |
| 9.271969 | 7.171787 | 8.206682 | 7.177807 |
| 6.323284 | 6.174598 | 6.468096 | 5.516463 |
| 0.059782 | 0.096421 | 0.091535 | 0.053532 |
| 0 | 0 | 0.053856 | 0 |
| 0.672448 | 0.621827 | 0.494219 | 0.61018 |
| 0.844898 | 0.38935 | 0.221772 | 0.504382 |
| 0.037881 | 0.040731 | 0.046401 | 0 |
| 2.37864 | 1.488087 | 0.264878 | 0.61963 |
| 6.047834 | 5.31229 | 4.27794 | 4.983369 |
| 6.042279 | 5.940486 | 5.720554 | 5.547149 |
| 0 | 0 | 0 | 0 |
| 13.22378 | 11.57749 | 12.22863 | 13.60432 |
| 9.958059 | 7.387688 | 5.59291 | 7.216713 |
| 3.1712 | 3.478741 | 2.883932 | 3.537656 |
| 4.787995 | 4.661869 | 3.417362 | 4.231197 |
| 0.699379 | 0.376006 | 0.856686 | 0.347926 |
| 6.571138 | 6.614097 | 6.29407 | 6.651061 |
| 0 | 0 | 0 | 0 |
| 1.51162 | 0 | 0 | 0 |
| 0.029783 | 0.016012 | 0.018241 | 0 |
| 1.343189 | 0.962849 | 0.877496 | 0.855306 |
| 2.800944 | 1.505867 | 2.287296 | 0.557364 |
| 10.6157 | 9.087355 | 7.196072 | 7.506312 |
| 0.177937 | 0.229594 | 0.21796 | 0.127469 |
| 8.507942 | 7.336046 | 7.760226 | 6.78819 |
| 30.1021 | 18.39062 | 19.94483 | 20.58401 |
| 0.989178 | 0.888779 | 0.984826 | 0.873637 |
| 1.457537 | 1.333161 | 1.14774 | 1.209117 |
| 8.301482 | 7.354312 | 6.651217 | 7.760922 |
| 51.28823 | 49.09359 | 50.07944 | 50.34756 |
| 0 | 0 | 0.125432 | 0 |
| 0.296442 | 0.39844 | 0.18156 | 0.265453 |
| 0 | 0 | 0 | 0.047614 |
| 0 | 0 | 0 | 0 |
| 0.233412 | 0 | 0 | 0.278682 |
| 32.54948 | 29.59254 | 28.11838 | 26.74971 |
| 0.389497 | 0.209405 | 0.039759 | 0.387533 |
| 0.778228 | 0.433894 | 0.635514 | 0.550616 |
| 0.090045 | 0.096822 | 0.088239 | 0.10751 |

| | | | |
|---|---|---|---|
| 0.294836 | 0.317025 | 0.180576 | 0.088005 |
| 0.572155 | 1.162071 | 0.38936 | 0.834929 |
| 0.865091 | 0.762243 | 0.986083 | 0.903764 |
| 0.020427 | 0 | 0 | 0 |
| 0 | 0.1595 | 0 | 0.088553 |
| 0.066256 | 0.047495 | 0.18937 | 0.263688 |
| 0.506554 | 0 | 0 | 1.209598 |
| 1.813945 | 1.300304 | 1.296135 | 1.624317 |
| 0 | 0 | 0 | 0 |
| 4.601228 | 4.065987 | 3.960373 | 4.007044 |
| 2.702715 | 2.916492 | 2.412024 | 2.339504 |
| 0.015515 | 0.016683 | 0.019005 | 0 |
| 3.399758 | 3.086383 | 3.099539 | 3.05015 |
| 7.298065 | 6.585143 | 6.334806 | 5.829318 |
| 0 | 0.556516 | 0 | 0 |
| 16.00495 | 13.09289 | 12.50542 | 11.64955 |
| 0.959703 | 0.79379 | 0.783709 | 0.73451 |
| 16.49747 | 14.43752 | 14.59644 | 15.47839 |
| 0 | 0 | 0 | 0 |
| 72.43866 | 74.69256 | 74.73964 | 74.22805 |
| 0 | 0 | 0 | 0 |
| 17.32965 | 19.33042 | 20.83074 | 21.85093 |
| 1.527624 | 1.100534 | 1.328569 | 1.404406 |
| 7.84209 | 6.967977 | 7.046294 | 6.671873 |
| 0 | 0 | 0 | 0 |
| 2.240755 | 4.216428 | 6.1757 | 8.694872 |
| 0.363481 | 0.781672 | 1.780948 | 0.867956 |
| 0 | 0.957 | 0.545103 | 0 |
| 0 | 0 | 0 | 0 |
| 0.04614 | 0.049612 | 0 | 0 |
| 0 | 0 | 0 | 0.321193 |
| 0.747381 | 0.65559 | 0.770935 | 0.587062 |
| 0.372364 | 0.100097 | 0.228059 | 0.111146 |
| 3.64462 | 3.298645 | 2.665784 | 2.942733 |
| 17.50393 | 16.08097 | 16.59417 | 15.61403 |
| 32.42817 | 29.42499 | 30.67146 | 31.20275 |
| 0.024109 | 0.025924 | 0.059064 | 0 |
| 30.49027 | 29.3429 | 28.81994 | 29.52435 |
| 1.253054 | 0 | 0 | 0 |
| 19.28971 | 20.18082 | 21.81906 | 19.65927 |
| 6.242897 | 5.770907 | 5.301413 | 6.151606 |
| 0.22355 | 0.480746 | 0.273831 | 0.320288 |
| 45.20123 | 42.89786 | 42.32805 | 42.35406 |
| 0.335437 | 0.377856 | 0.254357 | 0.400494 |
| 0.02762 | 0.089094 | 0 | 0.032976 |
| 7.430603 | 7.043974 | 6.039597 | 6.964742 |
| 26.54081 | 27.48035 | 27.2349 | 26.31125 |
| 11.17357 | 10.33891 | 10.19459 | 10.31734 |
| 0 | 0 | 0.021318 | 0 |
| 9.181893 | 8.632321 | 9.922202 | 9.527795 |
| 0 | 0 | 0 | 0 |
| 11.87626 | 28.28503 | 32.62996 | 26.76904 |
| 0.282453 | 0.264521 | 0.234376 | 0.206692 |
| 0.031244 | 0 | 0.038272 | 0.037304 |

| | | | |
|---|---|---|---|
| 0.375352 | 0.172971 | 0.197047 | 0.192064 |
| 7.179163 | 5.718108 | 5.792372 | 5.87621 |
| 0 | 0 | 0 | 0.182215 |
| 8.033545 | 7.665621 | 7.135982 | 7.273128 |
| 0.622432 | 0.066927 | 0.076243 | 0 |
| 4.66824 | 5.621904 | 5.603876 | 5.963791 |
| 39.85605 | 33.91904 | 32.45336 | 33.00324 |
| 0 | 0 | 0 | 0 |
| 0.631357 | 0.789385 | 1.043143 | 0.701216 |
| 6.210789 | 6.03852 | 5.73253 | 6.563019 |
| 3.228977 | 3.569783 | 3.453883 | 2.515855 |
| 0.101096 | 0.108704 | 0 | 0 |
| 1.440529 | 0.563251 | 0.096248 | 0.469068 |
| 0 | 0 | 0 | 0 |
| 0 | 0 | 0 | 0.056456 |
| 4.647708 | 4.977728 | 3.892904 | 4.101525 |
| 27.94795 | 24.66804 | 25.81038 | 23.31847 |
| 7.690784 | 7.895069 | 7.479039 | 7.349312 |
| 36.83875 | 23.45765 | 24.32253 | 25.11118 |
| 0 | 0 | 0 | 0 |
| 0.964538 | 0.725989 | 1.063338 | 1.26677 |
| 0 | 0 | 0 | 0 |
| 35.72606 | 67.54231 | 32.90716 | 29.79827 |
| 0.169108 | 0.103906 | 0.147961 | 0.057688 |
| 0.060526 | 0.072737 | 0.065417 | 0.059512 |
| 64.28846 | 63.42696 | 62.82122 | 63.28808 |
| 17.44835 | 20.07651 | 18.22689 | 17.71729 |
| 0.590979 | 1.452468 | 0.20683 | 0.302399 |
| 0.114187 | 0.122781 | 0 | 0 |
| 0 | 0 | 0 | 0 |
| 0.014029 | 0.030171 | 0.017185 | 0 |
| 1.227921 | 0.44011 | 1.671234 | 1.14028 |
| 2.694241 | 2.208605 | 2.450675 | 2.420551 |
| 21.0404 | 19.49131 | 20.30112 | 21.25635 |
| 0 | 0 | 0 | 0 |
| 0.03008 | 0 | 0.184226 | 0.071827 |
| 0.028656 | 0.041083 | 0.128703 | 0 |
| 33.81237 | 35.25094 | 37.39371 | 33.91257 |
| 0.807052 | 1.012419 | 1.318103 | 1.124174 |
| 0.139228 | 0 | 0 | 0 |
| 0 | 0.012871 | 0 | 0.014291 |
| 0.126638 | 0.181558 | 0 | 0 |
| 6.831928 | 4.297604 | 4.370643 | 4.243604 |
| 689.0418 | 661.7226 | 647.1988 | 657.9227 |
| 0 | 0 | 0 | 0 |
| 7.264684 | 5.220491 | 4.977979 | 4.551522 |
| 2.831791 | 1.691613 | 2.355311 | 2.212267 |
| 322.5202 | 282.0895 | 275.0458 | 270.8236 |
| 0.045177 | 0 | 0 | 0 |
| 4.81688 | 2.547238 | 1.741076 | 1.791328 |
| 6.728562 | 5.446754 | 5.502458 | 4.587342 |
| 24.01064 | 22.22276 | 23.74408 | 23.95003 |
| 0 | 0 | 0.185162 | 0 |
| 3.42262 | 2.93421 | 3.484267 | 3.120045 |

| | | | |
|---|---|---|---|
| 1.266566 | 0.809769 | 1.509517 | 1.185249 |
| 0.664796 | 0.446767 | 0.203581 | 0.29765 |
| 0 | 0 | 0 | 0.536331 |
| 2.916702 | 2.040422 | 2.625749 | 2.307609 |
| 6.885887 | 7.155344 | 7.801256 | 7.685214 |
| 50.72265 | 52.35272 | 52.9125 | 54.13548 |
| 0.10963 | 0.196468 | 0.447629 | 0.087262 |
| 5.966047 | 3.959069 | 4.494852 | 4.247067 |
| 6.160253 | 4.936647 | 4.959376 | 4.810832 |
| 0 | 0 | 0 | 0 |
| 2.835235 | 2.051398 | 1.78516 | 1.8982 |
| 0 | 0 | 0.059274 | 0 |
| 33.74716 | 36.68782 | 35.18888 | 35.66892 |
| 8.210579 | 5.757085 | 5.956288 | 6.1705 |
| 4.403468 | 3.444458 | 3.530353 | 3.66672 |
| 0.753319 | 0.776261 | 0.653621 | 0.861948 |
| 0 | 0 | 0 | 0 |
| 0 | 0 | 0 | 0 |
| 0 | 0.050314 | 0.011463 | 0 |
| 0.067254 | 0.072316 | 0.082381 | 0.080298 |
| 6.30572 | 7.353118 | 7.146333 | 7.122027 |
| 7.219279 | 5.510473 | 4.476122 | 4.735374 |
| 0.363481 | 0 | 0 | 0 |
| 32.71551 | 24.23148 | 26.39757 | 26.02408 |
| 11.07745 | 9.739219 | 10.50885 | 10.31925 |
| 0 | 0.204389 | 0 | 0.113475 |
| 0 | 0 | 0 | 0 |
| 0.490887 | 0 | 0.6013 | 0.586094 |
| 11.6313 | 9.68611 | 9.85489 | 9.254295 |
| 5.190122 | 5.094191 | 5.151212 | 4.957389 |
| 3.517874 | 2.696122 | 2.429616 | 2.070291 |
| 1.706346 | 1.351929 | 1.254087 | 1.265264 |
| 3.361133 | 2.986637 | 2.859121 | 3.176973 |
| 84.76127 | 91.1683 | 94.17462 | 91.29303 |
| 0.17357 | 0.062211 | 0.07087 | 0.034539 |
| 0 | 0 | 0 | 0.035421 |
| 3.819944 | 2.865643 | 3.427744 | 2.598604 |
| 0 | 0 | 0.011511 | 0.01122 |
| 0 | 0 | 0 | 0 |
| 1.52332 | 2.377685 | 2.166912 | 2.552139 |
| 0.638285 | 0.34316 | 0.46911 | 0.076208 |
| 0.319571 | 0 | 0 | 0 |
| 2.04532 | 1.850584 | 1.52766 | 1.876175 |
| 0 | 0 | 0.024683 | 0 |
| 0 | 0 | 0 | 0 |
| 0.977296 | 1.284366 | 0.665063 | 1.102017 |
| 0 | 0 | 0 | 0 |
| 17.13523 | 14.26871 | 13.81813 | 13.63404 |
| 0 | 0 | 0.024435 | 0 |
| 0.269017 | 0.578525 | 0.164763 | 0.963578 |
| 18.96826 | 18.71496 | 19.47884 | 16.47389 |
| 0.267077 | 0.246151 | 0.046736 | 0.136661 |
| 9.647021 | 9.846055 | 9.194411 | 10.28617 |
| 0 | 0 | 0 | 0 |

| | | | |
|---|---|---|---|
| 26.84224 | 24.27954 | 25.16975 | 26.15083 |
| 4.962264 | 4.635944 | 4.475761 | 3.88504 |
| 1.915996 | 2.009319 | 1.91233 | 1.835728 |
| 1.331917 | 0.358038 | 0 | 1.19268 |
| 1.678099 | 1.578839 | 1.541658 | 1.502672 |
| 61.34024 | 59.26301 | 56.89912 | 59.71972 |
| 11.39427 | 9.833654 | 8.264083 | 9.773518 |
| 68.91018 | 64.93748 | 65.64399 | 68.36255 |
| 0.340952 | 0.50409 | 0.31323 | 0.4198 |
| 11.34396 | 8.302619 | 8.40736 | 7.853303 |
| 6.82225 | 5.508013 | 5.162355 | 5.476608 |
| 11.51921 | 11.70541 | 13.12184 | 11.55992 |
| 0.145615 | 0 | 0 | 0 |
| 0 | 0.011198 | 0 | 0.012435 |
| 0 | 0 | 0 | 0 |
| 27.27235 | 29.29308 | 29.07274 | 27.17588 |
| 3.224003 | 5.33328 | 6.683194 | 4.441491 |
| 0.185517 | 0.199479 | 0.126247 | 0.147665 |
| 1.938238 | 1.702018 | 1.147528 | 1.889894 |
| 2.300188 | 1.594928 | 2.369908 | 1.719649 |
| 0 | 0.072011 | 0 | 0 |
| 0.202191 | 0.217408 | 0.123835 | 0.120703 |
| 17.40265 | 16.06469 | 16.14054 | 14.38162 |
| 0 | 0 | 0 | 0 |
| 5.993628 | 5.609268 | 5.846202 | 6.360961 |
| 0 | 0 | 0 | 0 |
| 29.53259 | 30.82203 | 29.21446 | 28.97701 |
| 0.057231 | 0.092307 | 0.105155 | 0.136661 |
| 9.818757 | 9.569007 | 8.246098 | 9.253005 |
| 11.42029 | 11.68463 | 11.87797 | 11.21764 |
| 7.111487 | 8.644069 | 7.259196 | 8.367693 |
| 0 | 0 | 0.03203 | 0 |
| 9.523209 | 6.695317 | 8.075916 | 7.871689 |
| 3.723775 | 3.299605 | 3.294303 | 3.087496 |
| 1.615714 | 1.12414 | 2.328386 | 1.929079 |
| 1.092111 | 0.841582 | 0.847244 | 0.956211 |
| 0 | 0 | 0 | 0.118811 |
| 2.467905 | 3.059481 | 2.66735 | 2.565232 |
| 1.280811 | 0.729107 | 0.738305 | 0.539726 |
| 8.129569 | 6.24384 | 6.570996 | 6.668943 |
| 8.060538 | 8.618458 | 8.348143 | 8.542531 |
| 0.536059 | 0.27567 | 0.256943 | 0.250445 |
| 9.9684 | 9.157632 | 9.768429 | 9.960495 |
| 14.09354 | 13.90937 | 12.70102 | 13.88224 |
| 11.06802 | 10.07784 | 10.346 | 9.878181 |
| 1.452514 | 1.419842 | 1.326327 | 1.324318 |
| 2.136354 | 1.413618 | 1.68587 | 1.545133 |
| 0 | 0 | 0 | 0 |
| 0.946014 | 0 | 0.772531 | 0 |
| 2.176919 | 1.589612 | 1.711375 | 1.415943 |
| 0.139578 | 0 | 0.024425 | 0.023807 |
| 0.193204 | 0.207744 | 0.107573 | 0.209705 |
| 0 | 0 | 0 | 0 |
| 0.028557 | 0.015353 | 0.021863 | 0.02131 |

| | | | |
|---|---|---|---|
| 0.620539 | 0.266896 | 0.35472 | 0.493928 |
| 0 | 0.029629 | 0.033754 | 0 |
| 7.209267 | 6.939125 | 6.623106 | 6.386203 |
| 9.367091 | 5.802365 | 7.025733 | 5.632431 |
| 24.65179 | 23.29479 | 24.6936 | 22.89835 |
| 54.4288 | 40.99897 | 38.80926 | 35.77245 |
| 183.085 | 136.6635 | 141.5205 | 138.8183 |
| 8.234417 | 7.737134 | 8.069231 | 8.056761 |
| 0.962798 | 0.228365 | 0.208122 | 0.236668 |
| 0.192778 | 0.236898 | 0.286739 | 0.230166 |
| 1.608178 | 1.428473 | 11.64808 | 11.52048 |
| 10.80546 | 9.139999 | 9.706303 | 9.030808 |
| 0 | 0.039415 | 0 | 0 |
| 30.76226 | 25.51177 | 27.86021 | 24.42057 |
| 0.430042 | 0.09567 | 0.090822 | 0.106231 |
| 0.504764 | 0 | 0 | 0.200887 |
| 4.242639 | 4.684122 | 3.480075 | 4.975036 |
| 0 | 0 | 0 | 0 |
| 0 | 0 | 0 | 0 |
| 0 | 0 | 0 | 0 |
| 0.987016 | 0.482407 | 0.549555 | 0.285684 |
| 0.61839 | 0.41558 | 0.18937 | 0.461454 |
| 3.296495 | 3.308274 | 3.454697 | 2.886286 |
| 0.744001 | 0.399996 | 0 | 0 |
| 11.28897 | 8.919839 | 8.825361 | 9.101383 |
| 29.17874 | 29.1058 | 29.56871 | 30.06023 |
| 0.826053 | 0.697886 | 0.722752 | 0.493132 |
| 8.887766 | 9.386383 | 8.908591 | 8.784129 |
| 0.149267 | 0 | 0.146272 | 0 |
| 2.300056 | 2.788872 | 1.798337 | 2.570861 |
| 0.340115 | 0 | 0 | 0 |
| 1.087479 | 1.284163 | 1.070421 | 1.089723 |
| 3.991458 | 3.906678 | 3.196807 | 3.574195 |
| 3.846499 | 3.434203 | 3.163793 | 2.984309 |
| 2.797424 | 1.688956 | 1.612533 | 1.839668 |
| 0.14495 | 0.155858 | 0.088776 | 0.086531 |
| 26.11014 | 25.56841 | 24.16569 | 27.2426 |
| 20.84222 | 11.79051 | 12.43178 | 11.43519 |
| 3.110057 | 2.76754 | 3.94095 | 3.457161 |
| 13.94535 | 15.81693 | 14.75945 | 14.84262 |
| 1.451423 | 0.503436 | 0.458809 | 0.503107 |
| 16.42754 | 15.44518 | 13.17197 | 17.05533 |
| 3.050646 | 2.163555 | 2.512409 | 2.247385 |
| 4.250333 | 2.318217 | 2.339079 | 2.353473 |
| 0.745865 | 0.641598 | 0.63954 | 1.033008 |
| 0.086202 | 0.046345 | 0.039597 | 0.051461 |
| 0.023296 | 0 | 0 | 0 |
| 33.24139 | 22.21864 | 35.21573 | 36.47051 |
| 23.12487 | 22.55128 | 20.29278 | 24.67354 |
| 0.528549 | 0.70205 | 0.285632 | 0.501136 |
| 15.64064 | 13.83232 | 12.52678 | 12.3205 |
| 0.091335 | 0.032736 | 0.037293 | 0 |
| 9.112099 | 9.056608 | 8.643741 | 8.784309 |
| 387.6973 | 350.3024 | 341.522 | 360.5446 |

| | | | |
|---|---|---|---|
| 14.35605 | 9.839257 | 8.658299 | 9.289821 |
| 6.855228 | 5.299246 | 6.21842 | 5.839956 |
| 0.176847 | 0 | 0 | 0.052787 |
| 3.025769 | 4.35428 | 3.511268 | 3.585448 |
| 2.018407 | 1.282453 | 0.786671 | 1.095397 |
| 0.210225 | 0.113023 | 0 | 0.250998 |
| 4.141821 | 4.485562 | 4.087934 | 3.771098 |
| 0.470282 | 0.505674 | 0.57606 | 0.842238 |
| 1.058134 | 0 | 0.259227 | 0 |
| 0.115015 | 0 | 0 | 0 |
| 0.03276 | 0.017612 | 0 | 0 |
| 1.461175 | 1.03106 | 1.09627 | 1.04674 |
| 34.61196 | 31.42748 | 30.85015 | 31.67174 |
| 13.82697 | 12.47167 | 12.72455 | 12.79149 |
| 21.3704 | 25.06765 | 24.3811 | 24.63985 |
| 0.948835 | 0.416425 | 0.355791 | 0.57799 |
| 0 | 0 | 0 | 0 |
| 0.028806 | 0.030974 | 0 | 0 |
| 0.327668 | 0.192178 | 0.164196 | 0.160044 |
| 2.02239 | 1.618861 | 1.734092 | 1.448777 |
| 2.956978 | 2.291119 | 2.343696 | 3.167047 |
| 7.249207 | 6.239874 | 6.274856 | 5.946908 |
| 0 | 0 | 0 | 0 |
| 33.21172 | 26.98625 | 31.74417 | 30.41571 |
| 0 | 0.957 | 0 | 0 |
| 1.614103 | 2.892626 | 2.965732 | 3.372522 |
| 2.103885 | 2.091023 | 2.368147 | 1.914498 |
| 22.44347 | 21.1737 | 21.78454 | 20.21652 |
| 16.63693 | 14.59905 | 14.28871 | 14.63135 |
| 0.174035 | 0.130993 | 0.042636 | 0.124673 |
| 0 | 0 | 0 | 0 |
| 0 | 0 | 0 | 0 |
| 0 | 0 | 0 | 0 |
| 0 | 0 | 0 | 0 |
| 1.823137 | 1.291519 | 1.812837 | 1.434081 |
| 0.719819 | 0.773991 | 0.573121 | 0.472685 |
| 0.768001 | 0 | 0 | 0 |
| 0.290715 | 0.296141 | 0.131196 | 0.182683 |
| 30.70408 | 11.84206 | 12.40447 | 12.82355 |
| 13.06614 | 12.0231 | 12.36287 | 10.95021 |
| 0.202169 | 0.114412 | 0.078203 | 0.190562 |
| 0.23106 | 0.105113 | 0.065315 | 0.074274 |
| 7.182495 | 6.669344 | 6.670122 | 6.847126 |
| 0.038462 | 0 | 0 | 0 |
| 9.512541 | 9.368091 | 8.406968 | 8.682634 |
| 1.806411 | 1.764624 | 1.634229 | 1.48013 |
| 1.15788 | 0.876125 | 0.998074 | 0.955767 |
| 0.318236 | 0.299412 | 0.097454 | 0.189979 |
| 0.292338 | 0.440074 | 0.401063 | 0.446767 |
| 3.346107 | 2.156485 | 2.754035 | 2.507951 |
| 0.789517 | 1.14167 | 0.800358 | 0.747613 |
| 0.271574 | 0.259566 | 0.147848 | 0.144109 |
| 0.242013 | 0.065057 | 0 | 0.144475 |
| 0.052614 | 0.169722 | 0.257795 | 0.314094 |

| | | | |
|---|---|---|---|
| 4.328731 | 4.29646 | 5.710244 | 2.38536 |
| 21.0171 | 22.34518 | 23.59365 | 23.21602 |
| 10.96214 | 8.7554 | 7.234932 | 8.096709 |
| 0.605427 | 0.492641 | 0.400866 | 0.429802 |
| 18.98888 | 16.95306 | 16.74011 | 17.10685 |
| 21.09442 | 21.58086 | 18.43856 | 20.90653 |
| 0.439726 | 0.878091 | 0.384737 | 0.375007 |
| 1.414338 | 0 | 0 | 0 |
| 0.713349 | 0.383517 | 0.436899 | 0.851702 |
| 7.657795 | 7.157159 | 5.801935 | 5.954167 |
| 7.267005 | 25.36709 | 26.54009 | 25.12066 |
| 10.95393 | 10.13882 | 10.02644 | 10.05348 |
| 0 | 0.550532 | 0 | 0.611302 |
| 96.89917 | 84.04782 | 83.95379 | 84.84123 |
| 14.90607 | 12.33568 | 12.95865 | 11.58824 |
| 11.39595 | 13.62241 | 13.311 | 13.27655 |
| 8.2909 | 6.755149 | 6.899975 | 7.068071 |
| 182.446 | 175.6118 | 173.1266 | 173.1428 |
| 0.271357 | 0.291779 | 0.288656 | 0.144941 |
| 6.106244 | 4.864802 | 4.844357 | 5.855095 |
| 2.136617 | 0.820505 | 0.560827 | 0.911075 |
| 22.19913 | 20.83745 | 20.32776 | 21.17347 |
| 0.019815 | 0 | 0 | 0.023658 |
| 0.375887 | 0.23775 | 0.297927 | 0.171596 |
| 36.40385 | 35.29584 | 29.74746 | 31.55862 |
| 0 | 0 | 0 | 0 |
| 1.000104 | 0.537684 | 0.544467 | 0.663373 |
| 1.348372 | 0.938136 | 1.489727 | 1.167957 |
| 245.4761 | 237.9927 | 224.4484 | 230.6289 |
| 0.069972 | 0.075238 | 0.042855 | 0 |
| 0.22355 | 0.080124 | 0 | 0 |
| 38.35994 | 41.31307 | 41.85382 | 39.78963 |
| 1.688512 | 1.573507 | 1.424829 | 0.895998 |
| 0 | 0.396895 | 0 | 0 |
| 0 | 0 | 0 | 0 |
| 0 | 0 | 0 | 0 |
| 9.286012 | 8.356885 | 9.427371 | 8.61654 |
| 40.36354 | 44.03112 | 45.97786 | 43.41619 |
| 1.573665 | 1.228133 | 2.051983 | 1.121263 |
| 0.442254 | 0.25362 | 0.288922 | 0.528029 |
| 0.074868 | 0 | 0.091708 | 0 |
| 0.257277 | 0.148959 | 0.339387 | 0.141773 |
| 0 | 0 | 0 | 0.194031 |
| 3.23869 | 2.681205 | 2.970889 | 2.639909 |
| 38.80083 | 39.03401 | 39.06826 | 39.24051 |
| 9.109643 | 7.571755 | 8.214938 | 7.673562 |
| 1.90272 | 1.126285 | 1.53025 | 1.158821 |
| 0 | 0 | 0 | 0 |
| 153.8264 | 140.014 | 136.8234 | 140.5413 |
| 2.38128 | 2.570772 | 2.670886 | 2.363562 |
| 5.213436 | 1.494876 | 3.405901 | 2.489829 |
| 159.7161 | 68.64554 | 67.86948 | 68.38838 |
| 5.120496 | 5.210893 | 4.760168 | 4.339569 |
| 5.270351 | 5.077044 | 3.604648 | 4.684657 |

| | | | |
|---|---|---|---|
| 0.135273 | 0 | 0 | 0 |
| 0.052556 | 0 | 0 | 0 |
| 4.99846 | 4.759884 | 4.221886 | 5.129275 |
| 0 | 0 | 0 | 0.288584 |
| 0.383228 | 0.309051 | 0.195594 | 0.228777 |
| 7.427896 | 6.917348 | 6.899122 | 6.046019 |
| 10.74603 | 9.96099 | 11.87703 | 9.143366 |
| 1.170769 | 0.813665 | 0.647096 | 0.698919 |
| 0 | 0 | 0 | 0.310662 |
| 0 | 0 | 0 | 0 |
| 9.999566 | 10.68861 | 10.5609 | 11.23391 |
| 0 | 0 | 1.405447 | 2.054859 |
| 5.717264 | 5.698803 | 5.520784 | 4.334833 |
| 18.09056 | 18.4242 | 19.55766 | 20.83835 |
| 0.198527 | 0.049262 | 0.074825 | 0.036466 |
| 3.237056 | 2.994122 | 2.813977 | 3.241509 |
| 0 | 0 | 0.098524 | 0.096032 |
| 140.6647 | 147.5484 | 144.5293 | 145.5869 |
| 11.26439 | 7.374254 | 7.74779 | 7.594287 |
| 349.0989 | 347.0034 | 352.6777 | 343.2745 |
| 8.427917 | 8.736372 | 8.412809 | 7.530123 |
| 0.043584 | 0.035148 | 0 | 0.052038 |
| 8.372861 | 8.012914 | 7.902628 | 8.047247 |
| 0.030602 | 0.098714 | 0.074969 | 0.10961 |
| 11.32317 | 8.7869 | 7.949093 | 9.916258 |
| 0 | 0 | 0 | 0 |
| 0 | 0.167319 | 0.190608 | 0.185788 |
| 3.717532 | 3.529533 | 3.39105 | 4.532977 |
| 23.64225 | 21.38946 | 20.59967 | 21.11612 |
| 0.020748 | 0 | 0.050829 | 0.049543 |
| 7.783392 | 7.384541 | 12.89903 | 5.466451 |
| 39.1183 | 42.56343 | 40.84581 | 41.2256 |
| 1.068401 | 1.257477 | 1.344081 | 1.137711 |
| 36.12288 | 33.88001 | 33.65362 | 31.97245 |
| 1.13558 | 1.473669 | 0.863379 | 1.168813 |
| 0.21693 | 0.233255 | 0.132861 | 0 |
| 0 | 0 | 0 | 0 |
| 0 | 0.390836 | 0 | 0 |
| 405.3366 | 381.8449 | 377.1588 | 393.4191 |
| 8.186097 | 7.012972 | 9.990954 | 7.769353 |
| 0.246715 | 0.795847 | 0 | 0 |
| 12.40917 | 10.42905 | 10.62856 | 10.04757 |
| 12.57015 | 10.69961 | 11.41443 | 10.11924 |
| 0.462791 | 0.387037 | 0.251948 | 0.030697 |
| 39.28232 | 30.31704 | 28.78078 | 27.65847 |
| 48.62608 | 31.59887 | 31.57855 | 31.12453 |
| 0.094947 | 0.051046 | 0.058152 | 0.056681 |
| 4.372205 | 3.525933 | 3.72383 | 4.363749 |
| 3.725065 | 2.960515 | 2.975819 | 1.93371 |
| 0.368034 | 0.395732 | 0.528541 | 0.439414 |
| 0.065859 | 0 | 0 | 0 |
| 0 | 0 | 0 | 0 |
| 21.9854 | 19.96535 | 20.86064 | 20.40111 |
| 0 | 0 | 0 | 0 |

| | | | |
|---|---|---|---|
| 11.49085 | 13.33357 | 12.79831 | 12.73952 |
| 0 | 0 | 0 | 0 |
| 6.445529 | 7.186341 | 6.351189 | 6.786918 |
| 0 | 0 | 0 | 0 |
| 13.94372 | 15.25715 | 16.57864 | 15.21459 |
| 18.41413 | 17.68944 | 16.64624 | 17.35228 |
| 8.60239 | 8.033846 | 8.410034 | 8.149138 |
| 1.598141 | 1.227437 | 1.101151 | 1.056268 |
| 6.122063 | 3.949675 | 4.888279 | 5.306102 |
| 137.8225 | 144.4393 | 140.0243 | 144.6194 |
| 5.044594 | 7.673308 | 5.576393 | 5.288473 |
| 1.776718 | 0.382086 | 0 | 1.272786 |
| 6.661467 | 5.524662 | 6.257062 | 6.419822 |
| 0.117025 | 0.167775 | 0.222983 | 0.20182 |
| 0 | 0 | 0 | 0 |
| 21.62118 | 21.26496 | 20.89396 | 22.34084 |
| 1.522483 | 1.574316 | 1.403568 | 1.317405 |
| 24.74535 | 21.23233 | 21.89141 | 19.99487 |
| 2.414535 | 0.725422 | 0.840893 | 1.003338 |
| 0.369117 | 0 | 0 | 0 |
| 84.30268 | 79.64365 | 76.44636 | 86.23246 |
| 26.95481 | 27.99286 | 27.39098 | 27.10428 |
| 1.543113 | 1.264185 | 1.260131 | 1.754663 |
| 0.16294 | 0 | 0.015353 | 0.014965 |
| 0.024672 | 0 | 0 | 0 |
| 242.4136 | 175.3796 | 178.2761 | 176.7986 |
| 4.032928 | 4.287156 | 3.705024 | 4.322652 |
| 5.888686 | 4.057832 | 4.358942 | 4.596471 |
| 1.904642 | 1.711141 | 1.458151 | 1.780337 |
| 0 | 0 | 0 | 0 |
| 3.359633 | 2.647682 | 2.410477 | 2.653862 |
| 264.4252 | 290.2963 | 282.3783 | 290.0767 |
| 20.71623 | 21.80958 | 20.12971 | 19.39085 |
| 8.842689 | 6.48483 | 7.137892 | 6.22759 |
| 23.39754 | 24.86412 | 23.83323 | 25.68101 |
| 1.152716 | 0.286031 | 0.456181 | 1.926797 |
| 14.25259 | 8.635416 | 9.214971 | 8.92275 |
| 0 | 0.033862 | 0.038575 | 0 |
| 11.70114 | 11.14623 | 10.14854 | 10.51698 |
| 0 | 0.11743 | 0 | 0 |
| 0 | 0 | 0 | 0 |
| 0 | 0 | 0 | 0 |
| 0 | 0.376467 | 0 | 0 |
| 5.798487 | 6.018375 | 5.99291 | 6.250014 |
| 17.27457 | 16.70676 | 15.43856 | 15.39381 |
| 1.033682 | 1.147327 | 0.653513 | 0.995292 |
| 47.21194 | 49.25801 | 47.79409 | 47.09873 |
| 23.53233 | 29.62286 | 28.36702 | 30.40052 |
| 11.33715 | 13.00304 | 16.66459 | 15.79197 |
| 0.341509 | 0.262292 | 0 | 0 |
| 0.506871 | 1.442691 | 0.182611 | 1.317151 |
| 0.155101 | 0.129713 | 0.126658 | 0.267486 |
| 22.20748 | 23.83095 | 22.46452 | 21.84335 |
| 7.061172 | 2.822019 | 4.056799 | 3.879602 |

| | | | |
|---|---|---|---|
| 42.16004 | 31.06635 | 28.17574 | 30.42421 |
| 7.636257 | 5.928362 | 6.464622 | 6.477152 |
| 0 | 0 | 0 | 0 |
| 5.548997 | 5.71799 | 6.199209 | 5.797063 |
| 0.036155 | 0.233255 | 0 | 0.086334 |
| 2.716764 | 2.284081 | 2.656787 | 2.482813 |
| 1.116001 | 1.99998 | 0.911345 | 0.888298 |
| 0 | 0.616861 | 0.234241 | 0.228318 |
| 0.13413 | 0 | 0.328598 | 0 |
| 39.44968 | 34.44878 | 33.53732 | 36.20008 |
| 0.105813 | 0.34133 | 0.259227 | 0.126336 |
| 13.44881 | 11.32076 | 10.76246 | 10.62497 |
| 0 | 0 | 0 | 0 |
| 15.86072 | 14.10263 | 13.74087 | 14.0408 |
| 2.12972 | 2.000733 | 1.455405 | 1.927156 |
| 36.35827 | 31.53535 | 31.39723 | 33.02613 |
| 8.924721 | 8.125805 | 8.476501 | 8.262144 |
| 7.251869 | 6.914873 | 6.368938 | 9.311816 |
| 14.55127 | 14.04637 | 12.54688 | 13.34513 |
| 11.71274 | 11.36019 | 11.5081 | 10.6663 |
| 0.569477 | 1.029835 | 0.729274 | 0.880813 |
| 0.465398 | 0.406593 | 1.068895 | 1.007136 |
| 3.161629 | 1.081679 | 7.452122 | 0.972302 |
| 23.49129 | 26.45118 | 24.37797 | 25.8414 |
| 6.085022 | 5.366518 | 5.360784 | 5.261007 |
| 0 | 0 | 0 | 0 |
| 0 | 0 | 0 | 0 |
| 0 | 0.013693 | 0 | 0 |
| 0.80484 | 0.374231 | 0.532901 | 0.519425 |
| 23.63487 | 19.97711 | 20.44594 | 18.66783 |
| 0.237882 | 0.227364 | 0.226635 | 0.205125 |
| 2.200171 | 1.63558 | 1.231069 | 1.167507 |
| 1.396406 | 3.657106 | 3.895178 | 3.268442 |
| 0 | 0.015643 | 0 | 0 |
| 0.04317 | 0.046418 | 0.052879 | 0 |
| 0.783159 | 0.785957 | 0.895356 | 0.311684 |
| 217.5188 | 240.6793 | 243.2956 | 244.3363 |
| 0.019092 | 0 | 0 | 0.022795 |
| 80.47337 | 89.68403 | 80.21906 | 82.6631 |
| 106.788 | 86.033 | 88.67117 | 84.82451 |
| 27.92817 | 24.08763 | 22.96777 | 24.09544 |
| 13.57821 | 12.74749 | 12.38628 | 13.33423 |
| 0 | 0.253463 | 0 | 0 |
| 4.043115 | 4.264316 | 3.65918 | 3.874114 |
| 0.068611 | 0.036887 | 0.168087 | 0.081918 |
| 4.889204 | 4.075311 | 3.745 | 4.027392 |
| 0.333166 | 0.32958 | 0.293834 | 0.111379 |
| 3.233949 | 1.821456 | 1.735445 | 1.94897 |
| 58.11009 | 52.22529 | 54.82031 | 50.65341 |
| 0 | 0 | 0 | 0 |
| 30.57761 | 34.48819 | 35.6051 | 34.00294 |
| 10.10381 | 10.88163 | 13.44916 | 13.59314 |
| 0.108465 | 0.349883 | 0 | 0.129501 |
| 0.055953 | 0.030082 | 0 | 0 |

| | | | |
|---|---|---|---|
| 19.72936 | 16.45455 | 17.62175 | 17.78012 |
| 8.201525 | 8.660136 | 9.504184 | 9.580852 |
| 0.032929 | 0 | 0 | 0.078632 |
| 0.357039 | 0.139603 | 0.278311 | 0.193766 |
| 3.2651 | 5.266233 | 2.999626 | 3.032058 |
| 0.291131 | 0.394199 | 0.316989 | 0.257478 |
| 0 | 0 | 0 | 0.574253 |
| 0.291676 | 0.392033 | 0.446601 | 0.522368 |
| 167.4603 | 133.7666 | 134.0577 | 135.0426 |
| 42.13463 | 24.28818 | 26.41458 | 29.05483 |
| 15.91767 | 15.61085 | 15.15048 | 14.83134 |
| 18.77584 | 22.63706 | 23.48402 | 21.82641 |
| 0.544984 | 0.652969 | 0.543588 | 0.59491 |
| 1.135518 | 0.651186 | 0.510005 | 0.81345 |
| 7.492095 | 7.44619 | 7.788034 | 7.857801 |
| 4.483133 | 3.374364 | 3.875731 | 3.19099 |
| 0 | 0 | 0.033377 | 0.016266 |
| 6.420606 | 5.523042 | 5.327739 | 6.083239 |
| 9.065912 | 8.727884 | 7.898175 | 6.797561 |
| 17.91903 | 18.5284 | 15.40953 | 18.30288 |
| 3.000042 | 2.619252 | 2.921014 | 2.449158 |
| 0.108317 | 0 | 0.053072 | 0.05173 |
| 0.584246 | 0 | 0.357829 | 0 |
| 10.64767 | 9.314425 | 8.434322 | 8.552525 |
| 0 | 0 | 0 | 0.173857 |
| 6.860807 | 5.395215 | 6.208903 | 4.340244 |
| 9.260546 | 8.726362 | 9.353207 | 8.674415 |
| 1.973143 | 2.001544 | 1.732909 | 1.644637 |
| 9.324907 | 7.361354 | 7.142557 | 8.540347 |
| 19.76152 | 21.11058 | 18.51538 | 19.19747 |
| 0.468662 | 0.377949 | 0.775002 | 0.67147 |
| 38.93197 | 37.24553 | 35.13906 | 43.5703 |
| 8.162751 | 7.679923 | 4.582762 | 3.248634 |
| 2.695611 | 1.552754 | 1.674545 | 1.241391 |
| 48.44057 | 2.438071 | 21.20948 | 2.461086 |
| 0.369117 | 0.79379 | 0.45214 | 0.881412 |
| 7.663796 | 6.619459 | 5.283715 | 7.350141 |
| 2.675059 | 2.199581 | 2.235897 | 2.479955 |
| 12.21898 | 14.12208 | 13.37123 | 13.71308 |
| 0.293926 | 0 | 0 | 0 |
| 7.093357 | 8.276304 | 7.949352 | 7.928519 |
| 0.911835 | 0.843193 | 0.625481 | 0.718532 |
| 15.03665 | 14.94566 | 15.54793 | 15.54262 |
| 0.068611 | 0.110662 | 0.168087 | 0.081918 |
| 411.2922 | 349.0681 | 356.2358 | 337.34 |
| 1.931515 | 1.580231 | 1.491584 | 2.005329 |
| 0 | 0 | 0 | 0.042331 |
| 0.633473 | 1.157948 | 1.603643 | 0.781545 |
| 0.132144 | 0.284178 | 0.107911 | 0.105182 |
| 4.282856 | 3.478226 | 2.948002 | 3.47595 |
| 8.812974 | 7.636849 | 8.103052 | 7.898139 |
| 104.7705 | 75.86684 | 74.09087 | 73.21621 |
| 5.659427 | 5.578227 | 5.242071 | 5.860292 |
| 17.23974 | 20.98255 | 20.76673 | 20.83021 |

| | | | |
|---|---|---|---|
| 0.081861 | 0 | 0 | 0 |
| 9.23083 | 8.847631 | 8.339603 | 7.530276 |
| 1.796832 | 2.41507 | 0 | 1.608993 |
| 3.356023 | 3.396317 | 2.901794 | 3.488375 |
| 11.6496 | 11.18713 | 10.00938 | 10.55417 |
| 0 | 0 | 0 | 0 |
| 0.14506 | 0 | 0.088844 | 0.043299 |
| 0.874835 | 0.268764 | 0.306174 | 0 |
| 133.5903 | 121.213 | 119.6384 | 124.6818 |
| 0 | 0 | 0 | 0 |
| 0.863698 | 5.168402 | 1.885937 | 7.083968 |
| 0.06058 | 0 | 0 | 0.07233 |
| 3.928084 | 3.811632 | 4.258356 | 4.16701 |
| 0.160215 | 0.034455 | 0.235502 | 0.076516 |
| 1.93727 | 2.740872 | 2.16485 | 2.556473 |
| 15.64559 | 13.71289 | 12.91065 | 12.89811 |
| 0 | 0 | 0 | 0 |
| 0 | 0.052405 | 0.089549 | 0.029095 |
| 1.256416 | 1.282277 | 1.330335 | 1.42382 |
| 0.4864 | 0.495479 | 0.533088 | 0.305651 |
| 0 | 0 | 0 | 0 |
| 181.1504 | 195.3956 | 194.6902 | 195.1185 |
| 5.061986 | 6.399936 | 6.404964 | 6.907141 |
| 29.6713 | 24.49556 | 24.53149 | 24.47614 |
| 6.576858 | 5.254598 | 4.838681 | 5.27547 |
| 3.131611 | 2.133312 | 1.953732 | 2.206231 |
| 0 | 0 | 0 | 0.059437 |
| 7.087586 | 6.231516 | 5.468068 | 5.329789 |
| 2.343855 | 2.743716 | 2.192177 | 2.44002 |
| 7.132084 | 6.15524 | 6.391267 | 6.946723 |
| 21.56487 | 19.73428 | 18.98407 | 19.96484 |
| 12.88434 | 6.023469 | 6.861889 | 10.70138 |
| 22.94573 | 22.62961 | 22.48543 | 22.2658 |
| 0 | 0 | 1.215126 | 0 |
| 266.4151 | 219.0401 | 211.7914 | 220.8485 |
| 46.10785 | 43.95974 | 50.60643 | 44.31041 |
| 0.08942 | 0.144224 | 0.164299 | 0.373669 |
| 0 | 0 | 0 | 0 |
| 12.2105 | 11.8903 | 11.35286 | 11.47561 |
| 22.40246 | 20.01942 | 21.85241 | 18.79545 |
| 0 | 0 | 0 | 0 |
| 0.814632 | 1.133568 | 0.860901 | 0.877273 |
| 10.98483 | 9.102673 | 8.729448 | 7.804153 |
| 4.934305 | 3.259183 | 3.108421 | 3.871429 |
| 0 | 0 | 0 | 1.148507 |
| 6.602758 | 6.709571 | 6.08813 | 6.822131 |
| 0.383118 | 0.264825 | 0.301687 | 0.261384 |
| 1.310533 | 2.3486 | 0 | 0 |
| 0.094476 | 0.304759 | 0.289316 | 0.2256 |
| 0 | 0.011697 | 0.013326 | 0.012989 |
| 0 | 0.020008 | 0 | 0 |
| 1.357331 | 0.87098 | 1.09948 | 0.83643 |
| 13.81045 | 12.39634 | 10.88557 | 11.97242 |
| 5.683613 | 9.778151 | 8.122329 | 10.10351 |

| | | | |
|---|---|---|---|
| 10.57166 | 10.68022 | 11.65098 | 11.14829 |
| 45.96522 | 57.88371 | 55.672 | 59.77679 |
| 18.13945 | 12.3258 | 14.19576 | 12.93437 |
| 0.297229 | 0.383517 | 0.21845 | 0.425851 |
| 0 | 0 | 0 | 0 |
| 13.13832 | 10.88528 | 11.99386 | 11.93823 |
| 3.610447 | 6.92037 | 6.890166 | 6.37232 |
| 28.38403 | 36.2663 | 34.29792 | 35.18807 |
| 0.26527 | 0.047539 | 0.054156 | 0.105573 |
| 0.03389 | 0.036441 | 0.041513 | 0 |
| 0.512 | 0 | 1.254324 | 0 |
| 0.151704 | 0.08156 | 0.069685 | 0.113204 |
| 52.03827 | 47.59767 | 46.84441 | 46.23865 |
| 0.885563 | 0.659221 | 0.333769 | 0.40666 |
| 0.021953 | 0 | 0 | 0 |
| 5.191443 | 4.864176 | 4.846021 | 4.165426 |
| 14.40994 | 16.59312 | 16.6804 | 17.19642 |
| 8.26115 | 7.968792 | 7.596138 | 7.521156 |
| 3.065612 | 2.758146 | 2.682245 | 2.433896 |
| 0 | 0 | 0 | 0 |
| 18.7063 | 14.17129 | 18.74766 | 13.70517 |
| 1.818157 | 1.514673 | 1.70544 | 2.073001 |
| 10.07962 | 12.55184 | 12.74063 | 12.13469 |
| 9.331895 | 8.777055 | 8.931178 | 9.314442 |
| 0.490887 | 0.568432 | 0.277523 | 0.045084 |
| 0 | 0.059534 | 0 | 0 |
| 1.292385 | 0.737363 | 0.592307 | 0.682297 |
| 0.202375 | 0.124346 | 0.283308 | 0.414216 |
| 0 | 0 | 0 | 0 |
| 0.140599 | 0.15118 | 0.172223 | 0.167867 |
| 8.81075 | 7.277732 | 7.505298 | 7.797531 |
| 0 | 0.085049 | 0 | 0 |
| 0 | 0 | 0 | 0 |
| 8.593428 | 7.119455 | 7.290757 | 7.947378 |
| 0 | 0 | 0 | 0.015124 |
| 0.380928 | 0 | 0 | 0 |
| 3.283865 | 3.793946 | 3.252223 | 3.503662 |
| 0.027444 | 0 | 0 | 0 |
| 206.121 | 191.0869 | 183.4799 | 205.5311 |
| 0.282588 | 0.050642 | 0.173074 | 0.337395 |
| 0 | 0 | 0 | 0.076826 |
| 1.024788 | 0.944495 | 0.986298 | 0.699168 |
| 14.46218 | 12.06635 | 13.78768 | 11.4028 |
| 4.486197 | 3.59458 | 3.034058 | 3.495029 |
| 0 | 0 | 0.01137 | 0.011082 |
| 23.96543 | 19.37964 | 19.85978 | 20.01534 |
| 0.971756 | 0.707827 | 0.537567 | 0.89824 |
| 57.89157 | 106.4436 | 105.6387 | 106.5821 |
| 0.168255 | 0.168856 | 0.137399 | 0.120532 |
| 2.481177 | 2.279844 | 1.795923 | 2.181401 |
| 16.0055 | 11.13999 | 9.60497 | 9.855628 |
| 0 | 0 | 0.24979 | 0.121737 |
| 11.24889 | 12.02672 | 10.37343 | 10.98867 |
| 0.721455 | 0 | 2.146197 | 5.045226 |

| | | | |
|---|---|---|---|
| 0.277227 | 0.277532 | 0.152226 | 0.239685 |
| 7.953841 | 2.377804 | 2.534016 | 3.662317 |
| 11.12006 | 8.118257 | 8.48003 | 8.380783 |
| 2.132062 | 1.111522 | 1.503657 | 1.619909 |
| 58.11723 | 57.2331 | 58.91521 | 57.04251 |
| 0.342015 | 0.408615 | 0.279295 | 0.22686 |
| 0.101419 | 0.109051 | 0 | 0.181633 |
| 5.655546 | 6.211943 | 5.028109 | 5.336596 |
| 17.6177 | 16.50424 | 16.4661 | 14.63779 |
| 0.114848 | 0.074095 | 0.084408 | 0.054849 |
| 3.347528 | 2.159672 | 2.360876 | 2.131613 |
| 0 | 0.218801 | 0.249257 | 0.485907 |
| 2.058639 | 1.811099 | 1.670202 | 1.276835 |
| 0 | 0 | 0 | 0 |
| 0.796255 | 1.141572 | 0.715258 | 0.950687 |
| 7.087741 | 6.445541 | 6.765453 | 6.301783 |
| 0.032525 | 0.034972 | 0 | 0.232996 |
| 47.37757 | 45.5154 | 49.5382 | 47.54991 |
| 1.1154 | 0.827132 | 0.75381 | 0.849552 |
| 0.533513 | 0.573664 | 0.816892 | 0.47774 |
| 118.1583 | 120.5716 | 105.3109 | 124.4056 |
| 6.39599 | 6.722311 | 7.786441 | 6.947945 |
| 0 | 0.131281 | 0 | 0 |
| 16.99986 | 16.36439 | 14.69897 | 14.93241 |
| 2.447923 | 1.698159 | 0.967265 | 1.037085 |
| 782.5716 | 835.3176 | 764.2092 | 834.3009 |
| 0.045262 | 0.048669 | 0 | 0 |
| 3.417152 | 2.635268 | 2.950612 | 3.076647 |
| 3.163857 | 3.855554 | 4.036964 | 3.80896 |
| 0 | 0 | 0.555486 | 0 |
| 976.6753 | 872.4057 | 805.2821 | 924.1564 |
| 91.7588 | 92.43346 | 89.99622 | 92.84056 |
| 19.1093 | 19.87126 | 20.45477 | 21.27223 |
| 5.402163 | 4.880323 | 4.516304 | 4.861806 |
| 0.950668 | 0.755548 | 0.455672 | 0.493499 |
| 0 | 0 | 0 | 0 |
| 0.622595 | 0.562337 | 0.305053 | 0.713612 |
| 14.69109 | 13.87454 | 13.09848 | 12.34037 |
| 0 | 0 | 0 | 0.300799 |
| 14.12722 | 12.38632 | 10.89305 | 12.36478 |
| 0 | 0 | 0 | 0.595299 |
| 0.196436 | 0.084488 | 0.336867 | 0.234534 |
| 1.91669 | 1.295444 | 1.71054 | 1.340365 |
| 115.8429 | 95.52334 | 93.59128 | 98.1134 |
| 0.035402 | 0 | 0 | 0 |
| 0.019278 | 0.020729 | 0.070841 | 0.023017 |
| 0.35869 | 0.192842 | 0.219684 | 0 |
| 3.698016 | 3.90594 | 3.287104 | 3.477489 |
| 0.105113 | 0.113023 | 0.51502 | 0 |
| 51.4454 | 42.62364 | 45.15983 | 41.77909 |
| 0 | 0 | 0 | 0 |
| 0.090766 | 0.039039 | 0 | 0.021674 |
| 1.926011 | 1.79886 | 1.446528 | 1.628153 |
| 3.34573 | 1.564139 | 1.247299 | 24.48881 |

| | | | |
|---|---|---|---|
| 204.1216 | 157.3132 | 153.9938 | 162.3491 |
| 0.507858 | 0.508417 | 0.343221 | 0.376359 |
| 0.048048 | 0.077497 | 0.058856 | 0.028684 |
| 1.180457 | 0.951971 | 1.02161 | 0.612785 |
| 0.60879 | 0.514333 | 0.798987 | 0.519188 |
| 13.58625 | 13.0758 | 14.37196 | 12.76596 |
| 7.711429 | 6.458851 | 6.86072 | 6.260791 |
| 9.658427 | 8.619792 | 6.270347 | 7.207288 |
| 0.248 | 0 | 0 | 0 |
| 4.493345 | 4.134419 | 4.107469 | 4.564101 |
| 4.9046 | 3.980426 | 4.223442 | 3.797519 |
| 0.506554 | 0 | 0 | 0 |
| 13.15264 | 12.39803 | 11.73902 | 12.68738 |
| 0.014312 | 0.015389 | 0 | 0.008544 |
| 0 | 0 | 0 | 0 |
| 0 | 0 | 0 | 0 |
| 9.694047 | 7.828374 | 8.540862 | 8.016549 |
| 30.50403 | 26.22196 | 26.22647 | 27.33985 |
| 0.501222 | 0.224559 | 0.15349 | 0.448824 |
| 6.112361 | 5.476966 | 5.222539 | 4.009308 |
| 0 | 0 | 0 | 0 |
| 0.384 | 0.20645 | 0.15679 | 0.076413 |
| 9.119057 | 7.753821 | 8.368194 | 8.597472 |
| 0 | 0 | 0.152886 | 0 |
| 35.21603 | 36.34835 | 39.16445 | 36.07857 |
| 10.16131 | 12.45393 | 11.82285 | 10.7236 |
| 15.45934 | 16.28144 | 16.52572 | 15.7288 |
| 3.159833 | 3.045141 | 2.632425 | 2.696322 |
| 0.457847 | 0 | 0 | 0.546645 |
| 6.432563 | 4.809239 | 5.570985 | 5.460104 |
| 1.738051 | 2.221465 | 2.289659 | 1.957682 |
| 0.867718 | 0.349883 | 0.664306 | 0.777008 |
| 0.015793 | 0 | 0 | 0.018856 |
| 9.823942 | 10.186 | 12.58615 | 10.6521 |
| 0 | 0 | 0 | 0 |
| 4.293104 | 2.221901 | 2.029302 | 1.57388 |
| 1.370246 | 0.920854 | 1.04903 | 0.818001 |
| 1.422764 | 1.172398 | 1.10756 | 1.365315 |
| 17.99948 | 17.21699 | 16.66459 | 17.11214 |
| 0 | 0 | 0 | 0 |
| 0.011297 | 0.036441 | 0.041513 | 0.040463 |
| 0.41226 | 0.487614 | 0.40399 | 0.344552 |
| 35.25326 | 30.89624 | 28.0391 | 30.78953 |
| 0 | 0.093034 | 0 | 0.068869 |
| 0.551537 | 0.444783 | 0.112599 | 0.658507 |
| 6.968202 | 6.367941 | 6.238176 | 6.476597 |
| 1.614986 | 2.01661 | 2.233492 | 1.306207 |
| 0.29007 | 0.167946 | 0.382645 | 0.186484 |
| 0 | 0 | 0 | 0.082512 |
| 0.013794 | 0.192814 | 0.016896 | 0.016469 |
| 0 | 0 | 0 | 0.748041 |
| 27.57447 | 8.789141 | 8.763187 | 8.445901 |
| 15.26474 | 11.96139 | 12.34146 | 11.89753 |
| 27.47763 | 27.54691 | 27.8907 | 28.45818 |

| | | | |
|---|---|---|---|
| 24.34635 | 17.10384 | 16.50657 | 16.79408 |
| 0 | 0.059812 | 0.068138 | 0 |
| 0.372 | 0.799992 | 0.455672 | 0 |
| 18.02938 | 17.4973 | 17.58276 | 17.3037 |
| 0 | 0 | 0 | 0 |
| 4.912354 | 3.493415 | 3.311086 | 2.948064 |
| 0 | 0 | 0 | 0.173326 |
| 5.340244 | 4.644587 | 4.752862 | 4.72952 |
| 1.893195 | 1.704282 | 1.483094 | 1.734707 |
| 3.977753 | 2.515945 | 3.15276 | 2.653982 |
| 0.074168 | 0.019937 | 0.045425 | 0.022138 |
| 0.018216 | 0.05876 | 0.044626 | 0.130492 |
| 0.017246 | 0 | 0 | 0 |
| 1.786777 | 2.056069 | 2.534246 | 1.908759 |
| 0 | 0 | 0 | 0 |
| 6.58564 | 6.61215 | 6.374641 | 6.449076 |
| 0.062365 | 0.234706 | 0.11459 | 0.186153 |
| 10.39335 | 8.966407 | 7.574493 | 8.392991 |
| 7.893341 | 8.005654 | 7.6958 | 8.405403 |
| 2.405747 | 2.217254 | 2.023419 | 1.93254 |
| 0.30532 | 0.029845 | 0.050999 | 0.265117 |
| 0.380928 | 0.409596 | 0.933217 | 0.454809 |
| 0.421381 | 0 | 0 | 0 |
| 0 | 0 | 0 | 0 |
| 35.20702 | 33.43482 | 32.07933 | 32.12948 |
| 43.78487 | 50.43265 | 50.74834 | 51.96003 |
| 0.190188 | 0.613501 | 0.423573 | 0.33029 |
| 1.874427 | 1.714671 | 2.227493 | 1.703528 |
| 4.151553 | 3.947255 | 3.679104 | 3.442623 |
| 0.134699 | 0.072418 | 0 | 0.026804 |
| 1.595177 | 0.68609 | 0.976986 | 1.809331 |
| 0 | 0 | 0 | 0 |
| 0.731304 | 0.480541 | 0.547429 | 0.509331 |
| 0.007763 | 0.016695 | 0.009509 | 0.013903 |
| 5.504633 | 4.935898 | 4.574593 | 4.226674 |
| 1.450856 | 2.14271 | 1.905661 | 1.87834 |
| 0.234851 | 0 | 0 | 0 |
| 7.720286 | 7.204277 | 7.293089 | 7.054116 |
| 0.03998 | 0 | 0 | 0 |
| 5.084218 | 4.215395 | 3.551587 | 4.363784 |
| 0.37142 | 0.27956 | 0.227481 | 0.133037 |
| 13.6252 | 13.91917 | 15.27587 | 15.20951 |
| 0.208842 | 0.224559 | 0 | 0 |
| 14.4573 | 12.03115 | 10.89791 | 10.73071 |
| 0.090957 | 0.048901 | 0.055708 | 0 |
| 0.328104 | 0.308696 | 0.251189 | 0.244837 |
| 16.39412 | 13.97881 | 12.85476 | 15.21016 |
| 0 | 0.501956 | 0 | 0.557364 |
| 4.126724 | 3.221301 | 3.718286 | 4.405959 |
| 0 | 0 | 0 | 0 |
| 5.324224 | 4.221052 | 4.594443 | 4.345427 |
| 104.609 | 122.3854 | 120.8881 | 133.5524 |
| 2.409487 | 2.319398 | 1.967626 | 1.808276 |
| 5.663106 | 4.432541 | 4.859125 | 4.5426 |

| | | | |
|---|---|---|---|
| 7.524979 | 6.799401 | 7.435992 | 5.737958 |
| 54.21845 | 69.27681 | 71.09995 | 72.27762 |
| 5.798144 | 2.821196 | 2.400494 | 2.649183 |
| 2.665529 | 3.126686 | 1.780948 | 20.54161 |
| 0.355344 | 0 | 0 | 0 |
| 18.65073 | 15.79965 | 15.24864 | 15.28769 |
| 0.225135 | 0.242078 | 0.137887 | 0.268799 |
| 27.08983 | 22.13512 | 22.51181 | 20.94514 |
| 0 | 0.423136 | 0 | 0 |
| 1.819876 | 1.713317 | 1.733831 | 1.467872 |
| 4.652789 | 4.599481 | 4.917958 | 4.92799 |
| 15.36821 | 17.34418 | 16.44456 | 16.69593 |
| 0.066596 | 0.107412 | 0.203937 | 0.079512 |
| 0.051869 | 0.055773 | 0.127072 | 0 |
| 0 | 0 | 0.183415 | 0 |
| 1.003034 | 1.035379 | 0.835476 | 0.996379 |
| 198.0939 | 231.0309 | 228.7383 | 232.6506 |
| 164.3609 | 184.3762 | 180.491 | 181.9808 |
| 1.73272 | 1.494125 | 1.626294 | 1.350079 |
| 55.46288 | 40.53075 | 39.99615 | 40.68811 |
| 0 | 0.806291 | 0 | 0 |
| 0.099876 | 0.089494 | 0.132536 | 0.119247 |
| 0.234948 | 0.08421 | 0.223839 | 0.218179 |
| 0 | 0.453093 | 0 | 0 |
| 0 | 0.575275 | 0 | 0 |
| 4.201416 | 4.030409 | 3.918677 | 3.786794 |
| 28.95947 | 27.69563 | 30.35684 | 31.25148 |
| 45.80979 | 39.42236 | 41.8027 | 40.60792 |
| 0.470669 | 0.984065 | 0.800742 | 0.718053 |
| 15.50947 | 16.02177 | 15.56072 | 14.75168 |
| 0.108547 | 0.181558 | 0.118189 | 0.1296 |
| 0.025935 | 0.083659 | 0 | 0 |
| 0.355344 | 0 | 0 | 0 |
| 2.76819 | 2.600174 | 2.708761 | 2.469309 |
| 17.80964 | 15.62582 | 19.01278 | 16.98149 |
| 0 | 0.012494 | 0.014233 | 0 |
| 0.046591 | 0.100195 | 0.114141 | 0.166882 |
| 0 | 0.26806 | 0 | 0 |
| 0.678498 | 0.72956 | 0.860792 | 0.520773 |
| 0.672586 | 1.134114 | 1.14218 | 0.693529 |
| 0.82315 | 0.408507 | 0.155122 | 0.377999 |
| 0 | 0.474069 | 0 | 0 |
| 8.411667 | 6.533918 | 6.607053 | 6.211719 |
| 5.647711 | 3.952517 | 3.816838 | 4.592266 |
| 0 | 0 | 0 | 0 |
| 0 | 0.390836 | 0.445237 | 0 |
| 0 | 0 | 0 | 0 |
| 0 | 0 | 0 | 0 |
| 0 | 0 | 0 | 0 |
| 0.064828 | 0.034853 | 0 | 0 |
| 29.92616 | 23.20978 | 24.92471 | 24.6227 |
| 242.8865 | 216.3151 | 216.904 | 215.1488 |
| 0.89002 | 1.4355 | 1.63531 | 1.593956 |
| 0.689765 | 0.693825 | 0.599614 | 0.478187 |

| | | | |
|---|---|---|---|
| 429.325 | 471.1472 | 487.3254 | 455.1194 |
| 5.424613 | 1.944284 | 5.168132 | 5.037438 |
| 0 | 0.073247 | 0 | 0 |
| 0.400135 | 0 | 0 | 0 |
| 0.818557 | 0.407879 | 0.342375 | 0.286043 |
| 0.249299 | 0.26806 | 0 | 0.29765 |
| 0.069109 | 0.07431 | 0.296286 | 0.082512 |
| 89.45596 | 86.71266 | 86.35522 | 90.00685 |
| 196.381 | 219.4004 | 223.1576 | 220.1382 |
| 0 | 0.059362 | 0.067624 | 0 |
| 0.517203 | 0.574066 | 0.592661 | 0.517915 |
| 2.272068 | 1.71014 | 2.385524 | 2.363951 |
| 9.359121 | 11.3704 | 10.5709 | 11.06546 |
| 1.148714 | 1.115631 | 0.907799 | 1.06181 |
| 43.67119 | 37.2197 | 37.81779 | 39.40437 |
| 12.9317 | 10.86608 | 12.11629 | 12.57677 |
| 0.031702 | 0.068175 | 0 | 0 |
| 0.630937 | 0.720821 | 0.410577 | 0.353112 |
| 79.86631 | 74.73396 | 76.60083 | 78.93022 |
| 0.534887 | 0.467302 | 0.559646 | 0.345923 |
| 0.184889 | 0.022939 | 0.052263 | 0.067922 |
| 9.854563 | 8.861005 | 11.17499 | 8.37481 |
| 93.09204 | 88.20885 | 82.13707 | 80.91166 |
| 7.012078 | 6.039724 | 6.295797 | 5.785924 |
| 1.400472 | 1.003911 | 0 | 1.114727 |
| 0 | 0 | 0 | 0 |
| 0.700236 | 0 | 2.573209 | 0.836045 |
| 9.108952 | 8.250634 | 8.505283 | 8.880348 |
| 10.14469 | 7.640614 | 7.724565 | 7.120026 |
| 0.088341 | 0.075992 | 0.043285 | 0.21095 |
| 0 | 0 | 0 | 0 |
| 2.420366 | 2.252177 | 2.537155 | 2.250703 |
| 0.086706 | 0 | 0.017701 | 0.034507 |
| 0.12116 | 0.130279 | 0.296825 | 0.289319 |
| 10.23493 | 9.345938 | 9.528133 | 8.873582 |
| 3.050777 | 3.859258 | 2.564588 | 1.785524 |
| 0.135658 | 0 | 0 | 0 |
| 0.545382 | 0.428542 | 0.35972 | 0.425757 |
| 0.364874 | 0.392333 | 0.446943 | 0.490096 |
| 16.25447 | 15.42153 | 6.090271 | 5.707941 |
| 20.14104 | 19.44992 | 19.17385 | 18.95036 |
| 26.16738 | 25.48443 | 27.80559 | 25.90737 |
| 0.933648 | 0 | 0.326757 | 0.318493 |
| 0.408792 | 0.298898 | 0.160236 | 0.44903 |
| 0 | 0 | 0.155536 | 0 |
| 1.067525 | 0.550018 | 0.599334 | 0.663838 |
| 0.134319 | 0 | 0 | 0 |
| 15.41256 | 14.8209 | 14.80079 | 14.6616 |
| 0 | 0 | 0 | 0 |
| 2.189243 | 1.961666 | 0.670414 | 2.613843 |
| 0.013762 | 0 | 0 | 0 |
| 1.152373 | 1.263879 | 1.524495 | 1.485943 |
| 0 | 0 | 0 | 0 |
| 3.013674 | 2.646387 | 2.522541 | 2.518719 |

| | | | |
|---|---|---|---|
| 7.884534 | 7.520718 | 7.343609 | 6.702398 |
| 0.938967 | 0.504815 | 0.575082 | 0.435975 |
| 4.506519 | 3.96796 | 4.311962 | 4.466871 |
| 0 | 0 | 0.347179 | 0.1692 |
| 0 | 0 | 0 | 0 |
| 0 | 0 | 0 | 0 |
| 52778.5 | 54485.79 | 55334.02 | 54450.51 |
| 0.346002 | 0.153194 | 0.211913 | 0.145803 |
| 0 | 0.039084 | 0 | 0 |
| 7.220466 | 6.692599 | 6.517019 | 7.03894 |
| 1.810496 | 1.589845 | 1.700253 | 1.296983 |
| 0.589921 | 0.612693 | 0.665129 | 0.544259 |
| 4.165986 | 3.229202 | 3.361024 | 2.836562 |
| 0.227937 | 0.196073 | 0.223365 | 0.163287 |
| 0.0573 | 0 | 0.140376 | 0.068413 |
| 13.39284 | 11.82615 | 10.89383 | 12.02156 |
| 11.20046 | 6.556278 | 6.296824 | 6.383987 |
| 0.589175 | 0.422343 | 0.349913 | 0.277114 |
| 0.063361 | 0.170324 | 0.038806 | 0 |
| 0 | 0 | 0.540056 | 0 |
| 20.15117 | 19.92344 | 19.61332 | 19.08393 |
| 0.613609 | 0.857723 | 0.927004 | 0.708197 |
| 0 | 0.071308 | 0 | 0.07918 |
| 0.056484 | 0.060735 | 0.069189 | 0.134878 |
| 48.17421 | 44.50523 | 43.38954 | 44.70167 |
| 5.69807 | 5.647394 | 4.005744 | 4.76224 |
| 0.026902 | 0.014463 | 0 | 0.064239 |
| 1.423195 | 0.568397 | 1.295028 | 0.873885 |
| 1.799372 | 1.474124 | 1.514378 | 1.519926 |
| 0.023922 | 0.051444 | 0.117209 | 0.099964 |
| 73.29296 | 72.9549 | 69.67277 | 75.96314 |
| 4.553001 | 4.681096 | 3.799526 | 3.595154 |
| 3.941879 | 3.398563 | 3.626099 | 3.657123 |
| 0.033228 | 0.035729 | 0.040702 | 0.039673 |
| 4.380635 | 4.230348 | 4.145257 | 3.866915 |
| 0 | 0 | 0 | 0 |
| 20.53169 | 18.02551 | 21.27028 | 17.34219 |
| 0 | 0 | 0 | 0 |
| 0.581328 | 0.539839 | 0.420776 | 0.347038 |
| 0.103626 | 0.055712 | 0.190401 | 0.185586 |
| 7.936008 | 9.075824 | 8.484812 | 7.886856 |
| 0.042859 | 0 | 0 | 0 |
| 0 | 0 | 0 | 0 |
| 2.937145 | 2.374402 | 3.203863 | 3.174036 |
| 46.23587 | 47.73672 | 47.12577 | 46.62064 |
| 12.53673 | 9.429819 | 11.10286 | 9.697713 |
| 25.70288 | 25.53402 | 24.97014 | 24.37518 |
| 14.2648 | 13.45306 | 12.86278 | 12.18481 |
| 14.0392 | 12.78487 | 12.4789 | 12.89647 |
| 0.75448 | 1.442239 | 1.540301 | 0.900809 |
| 0.477354 | 0.577438 | 0.365452 | 0.427452 |
| 0.426263 | 0.21569 | 0.460711 | 0.299374 |
| 5.396034 | 4.44263 | 4.480238 | 4.852155 |
| 38.04471 | 36.88318 | 37.9234 | 36.90054 |

| | | | |
|---|---|---|---|
| 0.32871 | 0.353448 | 0.402645 | 0.448529 |
| 1.909415 | 2.181432 | 2.48507 | 2.137259 |
| 4.222502 | 3.71477 | 3.360577 | 3.403651 |
| 20.42459 | 16.57126 | 17.38064 | 16.81422 |
| 1.051127 | 0.339069 | 0.51502 | 0.376497 |
| 0 | 0 | 0 | 0 |
| 4.436153 | 3.736324 | 4.697977 | 4.352008 |
| 4.252087 | 3.586723 | 3.63696 | 3.544987 |
| 0 | 0 | 0.046011 | 0.029898 |
| 122.5314 | 108.5507 | 110.2512 | 107.5706 |
| 0.294766 | 0.231617 | 0.277743 | 0.20304 |
| 0.128692 | 0 | 0 | 0 |
| 0.3351 | 0.223054 | 0.273648 | 0.247676 |
| 4.318613 | 3.542554 | 3.762972 | 4.332271 |
| 4.829145 | 4.06691 | 3.51611 | 3.749753 |
| 34.10576 | 32.81594 | 30.99583 | 33.23319 |
| 1.344513 | 1.118369 | 1.118667 | 0.893504 |
| 26.89934 | 23.04151 | 24.54373 | 24.00036 |
| 0 | 0 | 0 | 0 |
| 21.34655 | 20.95131 | 20.32037 | 20.00408 |
| 0.780591 | 0.671469 | 0.573699 | 0.931985 |
| 6.761478 | 5.375946 | 6.06591 | 5.798811 |
| 0.085181 | 0.045796 | 0 | 0 |
| 6.845554 | 6.169645 | 6.49152 | 5.73744 |
| 0 | 0 | 0 | 0 |
| 21.64968 | 21.12904 | 20.6639 | 20.69468 |
| 10.07214 | 9.936653 | 9.253209 | 10.6126 |
| 0 | 0 | 0 | 0.274643 |
| 11.75185 | 6.947423 | 9.405579 | 8.944124 |
| 0 | 0 | 0 | 0 |
| 2.080601 | 1.15055 | 1.674781 | 1.525966 |
| 0.832152 | 0.454972 | 0.431917 | 0.707271 |
| 0 | 0 | 0 | 0 |
| 0 | 0 | 0.01137 | 0 |
| 8.003218 | 6.670943 | 7.713479 | 6.666577 |
| 11.33715 | 1.219035 | 7.637936 | 12.85917 |
| 0.506554 | 0 | 0 | 0 |
| 0.19727 | 0.079543 | 0.06041 | 0.147206 |
| 0 | 0 | 0 | 0 |
| 10.30932 | 9.370472 | 8.253688 | 8.500847 |
| 0 | 0 | 0 | 0 |
| 0 | 0.312192 | 0 | 0.346653 |
| 1.936104 | 0 | 0.074112 | 0.216713 |
| 37.21254 | 34.63495 | 35.84112 | 37.11444 |
| 5.197032 | 6.267784 | 5.677758 | 7.630456 |
| 0.026498 | 0.028492 | 0 | 0 |
| 0.432873 | 0.18618 | 0.070698 | 0.206731 |
| 0.79533 | 0.409001 | 0.762432 | 0.825724 |
| 0.749602 | 1.331677 | 1.037972 | 1.050636 |
| 0 | 0.058181 | 0.110466 | 0 |
| 0 | 0 | 0 | 0 |
| 9.430335 | 8.065936 | 9.451184 | 7.463571 |
| 14.7232 | 11.91789 | 10.53719 | 11.29121 |
| 14.86726 | 17.9443 | 18.38434 | 20.01878 |

| | | | |
|---|---|---|---|
| 21.85254 | 18.10906 | 18.29426 | 18.29858 |
| 3.29607 | 2.604573 | 2.669044 | 2.773224 |
| 0.862312 | 0.989021 | 0.915431 | 0.843255 |
| 38.70799 | 39.87382 | 38.93735 | 39.66758 |
| 0 | 0.137019 | 0 | 0 |
| 0.895459 | 0.79435 | 0.712965 | 0.721664 |
| 0.012237 | 0 | 0.01499 | 0 |
| 0 | 0 | 0.738305 | 0 |
| 0.615194 | 0.396895 | 0.602853 | 0.440706 |
| 0 | 0 | 0 | 0 |
| 3.960226 | 3.19728 | 2.531655 | 2.754197 |
| 0.174418 | 0.187544 | 0 | 0 |
| 21.37538 | 19.9395 | 21.44484 | 21.75959 |
| 20.88962 | 22.00434 | 22.86729 | 21.58366 |
| 0.899529 | 0.475684 | 0.541896 | 0.581011 |
| 0 | 0.602347 | 0 | 0 |
| 0.085029 | 0.054857 | 0 | 0.142128 |
| 0.058568 | 0 | 0 | 0 |
| 0.150398 | 0.064687 | 0.110536 | 0.089784 |
| 0.040536 | 0.043586 | 0.099307 | 0.016133 |
| 20.36293 | 16.20656 | 29.08767 | 15.35126 |
| 15.77014 | 14.43751 | 13.27748 | 16.63934 |
| 1.842576 | 1.598898 | 1.610269 | 1.415166 |
| 0.952321 | 0 | 0 | 0 |
| 46.58481 | 42.37698 | 44.87042 | 42.34389 |
| 0 | 0.014828 | 0.067566 | 0.065857 |
| 0.400049 | 0.397066 | 0.364381 | 0.367413 |
| 0 | 0 | 0.07449 | 0 |
| 26.97693 | 23.40837 | 24.60315 | 22.99642 |
| 12.43212 | 11.4873 | 13.4256 | 12.42455 |
| 0 | 0.081097 | 0 | 0 |
| 4.518077 | 3.824457 | 3.14003 | 3.749264 |
| 79.16925 | 66.08137 | 66.12212 | 66.09844 |
| 28.3829 | 24.58468 | 26.30391 | 25.58918 |
| 0.713844 | 0.483282 | 0.582937 | 0.568195 |
| 4.065229 | 3.508694 | 3.170865 | 3.308333 |
| 0.037716 | 0.060831 | 0.184795 | 0.067546 |
| 17.7861 | 18.36285 | 16.67111 | 17.18443 |
| 0 | 0.393842 | 0 | 0 |
| 0.098584 | 0 | 0 | 0 |
| 3.547125 | 3.157344 | 3.452949 | 3.253442 |
| 9.753344 | 10.87032 | 8.893214 | 10.20572 |
| 0.646484 | 0.507984 | 0.182745 | 0.564058 |
| 9.670367 | 9.041852 | 7.08153 | 9.161434 |
| 0.088178 | 0.079012 | 0.126013 | 0.122826 |
| 4.866112 | 4.249066 | 5.00052 | 5.88787 |
| 6.01443 | 4.505928 | 4.228839 | 4.147822 |
| 6.114997 | 7.058664 | 8.096252 | 7.032571 |
| 6.968646 | 6.201848 | 5.77243 | 5.496112 |
| 0.498835 | 0.390091 | 0.499938 | 0.243648 |
| 2.711309 | 3.050952 | 3.128058 | 2.647446 |
| 3.258967 | 3.252426 | 2.940207 | 3.448345 |
| 0.213206 | 0.318405 | 0.188617 | 0.169705 |
| 27.78492 | 26.20243 | 26.93984 | 26.19484 |

| | | | |
|---|---|---|---|
| 29.22466 | 25.79539 | 24.51683 | 25.76848 |
| 0.258959 | 0.278447 | 0.29738 | 0.309183 |
| 0 | 0 | 0 | 0 |
| 9.620729 | 13.28888 | 15.43722 | 14.1512 |
| 0.853334 | 0.428192 | 0.348423 | 1.018837 |
| 5.240284 | 4.523976 | 5.30798 | 3.729913 |
| 0 | 0 | 0 | 0 |
| 7.239179 | 6.680193 | 7.138491 | 6.843068 |
| 8.496448 | 5.796168 | 5.596786 | 6.374677 |
| 1.092738 | 0.939979 | 0.803113 | 0.782803 |
| 4.193055 | 3.80994 | 4.054221 | 3.588043 |
| 10.30116 | 8.336142 | 6.67053 | 8.968059 |
| 0 | 0 | 0 | 0 |
| 11.86385 | 13.12681 | 12.73335 | 13.42508 |
| 0.341334 | 0.183511 | 0.418108 | 0 |
| 83.5015 | 79.82413 | 79.37933 | 81.40251 |
| 4.404276 | 3.574135 | 3.05372 | 3.340291 |
| 2.123959 | 1.619821 | 1.795415 | 1.693299 |
| 0.053262 | 0 | 0 | 0 |
| 16.51985 | 13.58354 | 14.28393 | 13.92272 |
| 1.700573 | 1.828553 | 1.388716 | 1.353597 |
| 0 | 0 | 0 | 0 |
| 0.371141 | 0.280829 | 0.353593 | 0.279004 |
| 2.033188 | 1.335015 | 1.357528 | 1.409421 |
| 0.124519 | 0.080334 | 0.061011 | 0.029734 |
| 0 | 0 | 0 | 0 |
| 0.097374 | 0 | 0 | 0 |
| 0 | 0.096785 | 0 | 0 |
| 63.0331 | 60.37203 | 59.99795 | 61.33251 |
| 0 | 0 | 0.089275 | 0.029006 |
| 0.04313 | 0 | 0 | 0.205982 |
| 10.84326 | 7.096958 | 8.0848 | 7.880349 |
| 10.4531 | 11.25724 | 11.29084 | 11.08295 |
| 42.05053 | 38.21588 | 38.15314 | 37.18831 |
| 0.032217 | 0 | 0.078926 | 0.07693 |
| 3.401146 | 2.925685 | 1.249844 | 0 |
| 79.91057 | 77.44589 | 73.12975 | 75.39494 |
| 0.0361 | 0.038817 | 0 | 0 |
| 5.793801 | 3.598783 | 4.099705 | 3.92887 |
| 28.12012 | 26.80807 | 26.95419 | 28.01991 |
| 14.34719 | 14.26431 | 12.83683 | 13.70384 |
| 114.5386 | 120.0391 | 115.0592 | 113.5827 |
| 19.80652 | 14.85296 | 17.09943 | 16.71064 |
| 2.700974 | 2.643186 | 2.379138 | 2.373325 |
| 35.77661 | 35.71465 | 36.01871 | 33.54009 |
| 0.185879 | 0.433047 | 0.113844 | 0.147953 |
| 0.391902 | 0.210697 | 0.120012 | 0.116978 |
| 0.217425 | 0 | 0.133165 | 0.259594 |
| 0.683484 | 0.489947 | 0.744192 | 0.498694 |
| 1.094622 | 0 | 0 | 0 |
| 10.11947 | 8.866029 | 9.140184 | 9.519252 |
| 0.358015 | 1.924793 | 0.877084 | 1.282355 |
| 3.224159 | 4.590299 | 4.168759 | 3.528688 |
| 36.45819 | 38.41196 | 34.02822 | 35.68954 |

| | | | |
|---|---|---|---|
| 0 | 0 | 0 | 0 |
| 0.568792 | 0.611598 | 0.298598 | 0.291047 |
| 0.157669 | 0.254302 | 0.096566 | 0.188249 |
| 0.010829 | 0.011644 | 0.013265 | 0 |
| 0 | 0.069945 | 0 | 0 |
| 0 | 0 | 0 | 0 |
| 1.016801 | 0.399996 | 0.273403 | 0.562589 |
| 1.693015 | 0.68266 | 1.814589 | 1.010686 |
| 0 | 0.224559 | 0 | 0.249347 |
| 0.034542 | 0.018571 | 0 | 0 |
| 0 | 0 | 0 | 0.701865 |
| 3.267894 | 3.59683 | 3.356782 | 3.440866 |
| 4.603735 | 4.025213 | 3.983356 | 4.153504 |
| 0.782288 | 0.680351 | 0.746867 | 0.892805 |
| 0.025328 | 0.272338 | 0.124098 | 0.18144 |
| 29.29561 | 29.57172 | 29.13687 | 30.6435 |
| 0.358593 | 0.550828 | 0.43925 | 0.336397 |
| 41.33038 | 42.31133 | 42.50525 | 39.37426 |
| 0 | 0.461257 | 3.15276 | 0.512172 |
| 0.457143 | 0.491547 | 0.447973 | 0.305651 |
| 5.593075 | 5.128003 | 4.939517 | 5.127628 |
| 4.930192 | 4.310537 | 4.09411 | 4.107602 |
| 0.414053 | 0.089043 | 0.202873 | 0.296614 |
| 2.008174 | 1.919381 | 1.557913 | 1.545156 |
| 2.489951 | 1.246979 | 1.378768 | 0.895934 |
| 10.97144 | 8.611895 | 8.164304 | 8.842047 |
| 1.190401 | 0 | 0.729076 | 0.355319 |
| 183.1433 | 166.0148 | 168.3489 | 165.9949 |
| 14.13862 | 15.1802 | 15.47687 | 15.16029 |
| 14.93045 | 15.18629 | 20.26583 | 19.75334 |
| 7.544958 | 6.975002 | 8.11493 | 7.415359 |
| 0.945355 | 0.5885 | 0.731361 | 1.128705 |
| 12.71804 | 10.50351 | 11.07397 | 11.36564 |
| 0.51078 | 0.305122 | 0.347593 | 0.338803 |
| 11.23459 | 9.390345 | 9.953866 | 9.105993 |
| 0.698369 | 0.368636 | 0.248858 | 0.439648 |
| 0.370553 | 0.697269 | 1.248223 | 0.442421 |
| 0 | 0.506926 | 0 | 0 |
| 54.03184 | 54.15011 | 58.94151 | 53.80549 |
| 0 | 0 | 0 | 0 |
| 0.175786 | 0.141761 | 0.080747 | 0.05247 |
| 0 | 0.053167 | 0 | 0 |
| 35.67359 | 31.85501 | 35.53558 | 33.53541 |
| 1.064046 | 0.572061 | 0.977532 | 0.317604 |
| 5.025819 | 3.582111 | 3.447499 | 3.737496 |
| 5.169448 | 4.674041 | 4.478008 | 4.787604 |
| 0 | 0 | 0 | 0.693306 |
| 0.015788 | 0 | 0.038678 | 0.01885 |
| 106.8309 | 122.0911 | 118.1477 | 118.2836 |
| 306.4774 | 344.8828 | 342.7144 | 339.8385 |
| 16.1887 | 16.18116 | 15.08192 | 14.33754 |
| 0.158853 | 0.23486 | 0.267551 | 0.118539 |
| 43.02655 | 27.75876 | 29.51439 | 33.56269 |
| 8.451848 | 6.613267 | 7.242152 | 6.916882 |

| | | | |
|---|---|---|---|
| 21.39272 | 20.49835 | 17.01177 | 19.56831 |
| 3.629366 | 3.902502 | 3.533761 | 3.148106 |
| 0 | 0 | 0 | 0 |
| 5.959666 | 3.805367 | 4.616541 | 3.420577 |
| 0 | 0 | 0 | 0 |
| 8.291186 | 7.715727 | 8.116703 | 7.175959 |
| 9.453612 | 6.859858 | 7.601569 | 6.093661 |
| 1.204683 | 1.135096 | 1.521285 | 1.141767 |
| 0.086053 | 0.061686 | 0.105409 | 0 |
| 23.80802 | 27.34053 | 27.14106 | 27.28852 |
| 0 | 0 | 0 | 0 |
| 0.082238 | 0.088427 | 0 | 0.098188 |
| 0 | 0 | 0 | 0.07918 |
| 0.151643 | 0.228278 | 0.074301 | 0.144844 |
| 0.668376 | 0.718676 | 0.169388 | 1.100699 |
| 0 | 0 | 0 | 0.836045 |
| 2.322734 | 2.627616 | 2.608076 | 2.108806 |
| 0.690088 | 0.70492 | 0.295857 | 1.1535 |
| 0.055822 | 0.110042 | 0.056981 | 0.122189 |
| 13.69107 | 12.91879 | 14.00396 | 12.84362 |
| 0 | 0 | 0 | 0.21782 |
| 0 | 0 | 0 | 0 |
| 2.406965 | 2.09299 | 2.038207 | 2.061633 |
| 0 | 0 | 0 | 0 |
| 13.52755 | 11.72036 | 11.42629 | 12.08257 |
| 8.111762 | 8.983896 | 9.306998 | 9.039355 |
| 0.967487 | 0.674787 | 0.832772 | 0.686834 |
| 32.8307 | 29.38836 | 29.2437 | 27.91443 |
| 3.257308 | 2.628732 | 2.657086 | 2.665817 |
| 0.399463 | 0.343621 | 0.163104 | 0.222571 |
| 0 | 0 | 0 | 0 |
| 6.494596 | 5.677672 | 6.218934 | 5.62213 |
| 9.189731 | 8.033707 | 7.483696 | 8.874909 |
| 19.33413 | 19.05673 | 18.74892 | 18.5496 |
| 0.964823 | 0.978986 | 0.865569 | 0.859905 |
| 31.09048 | 35.08671 | 34.69543 | 34.9049 |
| 2.001001 | 1.726842 | 1.63405 | 1.37624 |
| 0 | 0 | 0 | 0 |
| 0.051035 | 0.109752 | 0.062515 | 0.121867 |
| 0.596692 | 1.539834 | 1.169445 | 0.854904 |
| 16.46143 | 11.63419 | 11.83539 | 11.10883 |
| 0.419261 | 0.450813 | 0.660295 | 0.500576 |
| 0.681948 | 0.75915 | 0.737061 | 0.842948 |
| 0.511771 | 0.619071 | 0.757481 | 0.661947 |
| 303.3283 | 323.6037 | 319.8736 | 310.4544 |
| 4.519367 | 4.58735 | 5.004438 | 5.136886 |
| 0 | 0.076531 | 0 | 0.127469 |
| 1.700006 | 0.888346 | 1.109304 | 0.967436 |
| 12.49179 | 11.08258 | 11.19977 | 12.07908 |
| 0.167466 | 0.300114 | 0.341888 | 0.233269 |
| 7.090489 | 5.694148 | 6.43222 | 5.401739 |
| 3.130191 | 2.624152 | 2.372035 | 2.882144 |
| 1.316249 | 0.713055 | 0.812306 | 0.515847 |
| 8.676352 | 9.262886 | 9.424071 | 9.37349 |

| | | | |
|---|---|---|---|
| 2115.556 | 2375.225 | 2377.37 | 2309.83 |
| 7.631993 | 5.068169 | 5.076494 | 6.237416 |
| 29.04579 | 26.84316 | 25.33017 | 25.74542 |
| 0.031702 | 0.068175 | 0.019416 | 0.018925 |
| 0 | 0 | 0 | 0 |
| 0.043091 | 0 | 0 | 0.051449 |
| 50.5726 | 45.51065 | 40.79012 | 41.98806 |
| 8.12829 | 4.510968 | 4.432267 | 5.134129 |
| 10.37786 | 8.533248 | 11.21655 | 8.381891 |
| 20.42445 | 19.11712 | 21.7404 | 20.05213 |
| 8.368288 | 7.287902 | 6.503985 | 7.917084 |
| 0.031829 | 0 | 0.077976 | 0 |
| 0.866417 | 0.594651 | 0.60968 | 0.352155 |
| 1.400472 | 0.265741 | 0.10091 | 0 |
| 3.901649 | 3.553142 | 3.93392 | 4.143411 |
| 0.053925 | 0.028992 | 0 | 0.032192 |
| 10.38359 | 11.23667 | 10.84214 | 11.43158 |
| 0 | 0 | 0 | 0 |
| 28.69027 | 22.09321 | 20.78126 | 24.26187 |
| 10.82183 | 10.13112 | 10.89291 | 11.74099 |
| 0.671164 | 1.574561 | 0.149477 | 0.815905 |
| 22.4316 | 26.37638 | 26.37526 | 26.09878 |
| 0.610462 | 0 | 0 | 0 |
| 0.023182 | 0.024927 | 0 | 0 |
| 4.524722 | 4.23562 | 4.205736 | 3.495595 |
| 22.58735 | 24.45795 | 24.16678 | 23.05799 |
| 10.07869 | 8.099609 | 6.95892 | 6.53172 |
| 0.040907 | 0.010996 | 0.025054 | 0.01221 |
| 0.780099 | 0.645236 | 0.514534 | 0.71646 |
| 5.639682 | 5.910585 | 7.083075 | 6.392551 |
| 6.518135 | 8.489375 | 7.834284 | 8.330365 |
| 48.63691 | 28.93202 | 24.02712 | 25.16074 |
| 0.064173 | 0.552016 | 0.23582 | 0.459712 |
| 12.16776 | 8.243344 | 6.84922 | 9.069302 |
| 0.406975 | 0.875205 | 0 | 0.485907 |
| 13.39692 | 15.96816 | 18.04643 | 15.47926 |
| 8.531015 | 10.76834 | 9.030033 | 8.635608 |
| 20.32052 | 22.99977 | 23.78989 | 23.41036 |
| 366.9824 | 391.3771 | 390.9868 | 398.217 |
| 14.05565 | 9.089962 | 8.951647 | 8.786077 |
| 1.609679 | 1.730819 | 1.545414 | 1.644846 |
| 1.264143 | 0.63433 | 0.671008 | 0.201243 |
| 11.38284 | 10.72387 | 9.99668 | 10.18228 |
| 0.377905 | 0.406345 | 1.388716 | 0.902398 |
| 4.251433 | 3.860279 | 3.97327 | 3.722393 |
| 18.1256 | 26.99372 | 23.80534 | 23.29014 |
| 8.640094 | 7.829435 | 7.476033 | 7.717858 |
| 26.54955 | 25.28032 | 25.78407 | 26.65211 |
| 0 | 0 | 0.123572 | 0 |
| 1.293866 | 0.98149 | 0.814155 | 0.931118 |
| 0.15872 | 0 | 0.19442 | 0.189504 |
| 5.157693 | 3.921924 | 3.734822 | 3.844508 |
| 0.121381 | 0.03729 | 0.063721 | 0.144923 |
| 0.102488 | 0.242443 | 0.150648 | 0.146839 |

| | | | |
|---|---|---|---|
| 46.36142 | 42.62341 | 44.16534 | 41.12252 |
| 1.212851 | 0.940476 | 0.914246 | 0.877203 |
| 2.340105 | 2.188012 | 2.99108 | 2.91544 |
| 16.39483 | 10.65993 | 11.63621 | 10.95329 |
| 0 | 0.052085 | 0 | 0.028917 |
| 0 | 0 | 0 | 0 |
| 0.239578 | 0 | 0 | 0 |
| 5.686937 | 4.733016 | 4.329195 | 3.836106 |
| 2.686995 | 2.351682 | 2.326919 | 2.476977 |
| 0.222349 | 0.179312 | 0.326833 | 0.238926 |
| 5.798459 | 4.843003 | 4.756688 | 5.093731 |
| 0.044921 | 0.096603 | 0 | 0 |
| 354.5206 | 329.8542 | 324.4806 | 334.7353 |
| 0.986592 | 0.632782 | 1.166097 | 0.640634 |
| 110.142 | 88.15332 | 85.16164 | 87.76836 |
| 0 | 0.170665 | 0.38884 | 0.189504 |
| 1.031991 | 0.93211 | 0.758466 | 0.566786 |
| 0.64118 | 0.580575 | 0.413367 | 0.362622 |
| 1.74205 | 0.624384 | 0 | 0 |
| 7.176995 | 5.36169 | 4.625129 | 5.02437 |
| 0.354399 | 0.362924 | 0.434112 | 0.402985 |
| 5.507025 | 2.072245 | 1.957045 | 1.931399 |
| 0 | 0 | 0 | 0 |
| 7.758215 | 7.021247 | 5.89992 | 6.638416 |
| 3.458989 | 3.02271 | 2.819943 | 2.859129 |
| 0 | 0 | 0 | 0.088141 |
| 0.02522 | 0 | 0 | 0 |
| 6.194487 | 5.25842 | 5.45122 | 6.169735 |
| 4.998838 | 4.965075 | 5.448609 | 4.906188 |
| 0 | 0 | 0 | 0.103178 |
| 0.574017 | 0.822954 | 0.803574 | 0.717982 |
| 3.347227 | 2.787456 | 2.737456 | 2.66823 |
| 0 | 0 | 0 | 0 |
| 0.710687 | 1.528343 | 2.176346 | 0 |
| 0 | 0 | 0 | 0 |
| 0 | 0.09094 | 0 | 0.100979 |
| 0.535012 | 0 | 0 | 0.638776 |
| 20.61284 | 13.44929 | 13.17348 | 14.04994 |
| 1.341297 | 1.638908 | 3.061931 | 2.766122 |
| 34.71566 | 33.13682 | 31.56116 | 32.78747 |
| 1.324437 | 1.739057 | 1.637934 | 1.292416 |
| 0 | 0 | 0 | 0 |
| 0.891209 | 0.718709 | 0.662796 | 0.855046 |
| 0.511085 | 0.915912 | 0.62604 | 1.42382 |
| 71.80554 | 67.26869 | 61.29459 | 72.97944 |
| 2.805946 | 2.468547 | 2.048356 | 2.977914 |
| 1.44187 | 0.701363 | 1.303611 | 1.10669 |
| 0 | 0.110821 | 0.252494 | 0.123054 |
| 0 | 0 | 0 | 0 |
| 9.717219 | 7.031612 | 6.658226 | 5.930725 |
| 1.110534 | 0.776989 | 0.810602 | 0.808266 |
| 3.347016 | 3.19148 | 2.398021 | 2.387645 |
| 33.26353 | 28.24465 | 25.87355 | 25.75812 |
| 51.44669 | 45.43299 | 44.41994 | 48.61863 |

| | | | |
|---|---|---|---|
| 0 | 0 | 0 | 0 |
| 212.3911 | 173.0669 | 165.0411 | 166.7632 |
| 0.220105 | 0.15778 | 0.179741 | 0.175196 |
| 112.2696 | 121.3681 | 121.7527 | 113.3606 |
| 59.56334 | 51.38567 | 50.47855 | 52.76815 |
| 0.250199 | 0.168143 | 0.344785 | 0.261384 |
| 0.009054 | 0 | 0 | 0 |
| 4.170041 | 3.806072 | 4.276452 | 4.457774 |
| 12.24759 | 13.77779 | 15.24994 | 11.92039 |
| 8.428421 | 7.445072 | 7.016001 | 7.228117 |
| 0 | 0 | 0 | 0 |
| 0.115854 | 0.373719 | 0.141913 | 0 |
| 10.84988 | 9.205955 | 10.80229 | 9.516111 |
| 0.597368 | 0.476563 | 0.354063 | 0.299095 |
| 6.108871 | 4.073554 | 3.88647 | 4.381859 |
| 6.000275 | 4.407691 | 4.693738 | 4.716902 |
| 0 | 0 | 0 | 0 |
| 36.7153 | 39.47838 | 37.68707 | 39.55449 |
| 21.55641 | 22.86117 | 12.56949 | 22.12345 |
| 0.089002 | 0.04785 | 0.05451 | 0.106264 |
| 2.979289 | 1.997477 | 1.846169 | 1.883179 |
| 120.0598 | 127.6417 | 126.1214 | 127.7168 |
| 0.030523 | 0 | 0 | 0 |
| 0 | 0 | 0 | 0.021453 |
| 0.278456 | 0.088715 | 0.126329 | 0.221642 |
| 30.08378 | 22.86839 | 23.48905 | 22.35984 |
| 12.46459 | 10.32972 | 10.74427 | 11.44371 |
| 25.59143 | 21.64923 | 22.7049 | 23.59681 |
| 22.82852 | 21.88122 | 20.96016 | 22.57814 |
| 5.157335 | 5.005318 | 4.867912 | 4.591537 |
| 4.489005 | 3.902886 | 4.319105 | 3.785356 |
| 2.480002 | 1.466652 | 2.126471 | 1.480497 |
| 0.37705 | 0.666057 | 0.395878 | 0.546645 |
| 0.117949 | 0.05073 | 0.144479 | 0.070413 |
| 2.908163 | 2.047979 | 2.082178 | 2.274044 |
| 11.33505 | 8.81439 | 8.985732 | 9.391641 |
| 26.16873 | 29.79436 | 29.8193 | 29.6849 |
| 320.5289 | 392.2526 | 380.6432 | 374.3977 |
| 15.30671 | 12.57777 | 11.71848 | 11.94132 |
| 0.043366 | 0 | 0 | 0 |
| 4.568903 | 3.709623 | 4.644765 | 9.462812 |
| 0 | 0.103433 | 0 | 0 |
| 124.9318 | 136.853 | 132.141 | 138.3843 |
| 0.009989 | 0 | 0 | 0 |
| 25.03146 | 21.23111 | 20.74029 | 21.63747 |
| 1.798307 | 1.289095 | 1.596225 | 1.618093 |
| 1.322668 | 2.844416 | 5.670589 | 3.158394 |
| 4.22552 | 3.427851 | 3.693155 | 3.36636 |
| 0.219429 | 0 | 0.16127 | 0.052397 |
| 0 | 0.298539 | 0.056682 | 0.110498 |
| 1.594739 | 1.676364 | 1.501521 | 1.506177 |
| 4.713568 | 7.683151 | 5.736989 | 6.559741 |
| 3.135724 | 3.41627 | 2.978064 | 3.44702 |
| 38.6764 | 35.17879 | 35.05647 | 32.24279 |

| | | | |
|---|---|---|---|
| 0.383485 | 0.068724 | 0.31316 | 0.228931 |
| 0.660628 | 0.874271 | 0.373486 | 1.21347 |
| 1.738376 | 0.182766 | 2.242946 | 0.581149 |
| 0 | 0 | 0 | 0 |
| 38.29818 | 35.57719 | 36.02073 | 37.83905 |
| 39.09862 | 34.00308 | 34.29639 | 29.53444 |
| 1.90237 | 1.251747 | 1.51293 | 1.44077 |
| 3.158436 | 2.97765 | 2.530335 | 3.109742 |
| 652.9616 | 704.6527 | 722.1608 | 719.723 |
| 0.05506 | 0.059204 | 0.053956 | 0.039443 |
| 0 | 0 | 0 | 0.531319 |
| 0 | 0 | 0 | 0 |
| 0.374929 | 0 | 0 | 0 |
| 8.007218 | 8.558771 | 7.540325 | 7.330748 |
| 6.767763 | 6.720165 | 5.815688 | 5.977816 |
| 1.40498 | 1.867792 | 1.314214 | 1.86047 |
| 0.056889 | 0 | 0.069685 | 0 |
| 129.4587 | 126.2175 | 125.6073 | 132.1077 |
| 6.106831 | 5.060886 | 5.145253 | 5.73526 |
| 0 | 0.083251 | 0.04742 | 0 |
| 3.659228 | 3.112009 | 3.150014 | 2.872267 |
| 0.032304 | 0.034735 | 0 | 0 |
| 6.940644 | 7.506365 | 6.870612 | 7.756801 |
| 0.300944 | 0.467411 | 0.286715 | 0.279465 |
| 0 | 0 | 0 | 0 |
| 0 | 0 | 0 | 0 |
| 71.92685 | 77.62605 | 75.57029 | 73.72986 |
| 0 | 0 | 0 | 0 |
| 2.09488 | 2.29312 | 2.35801 | 2.163181 |
| 65.72024 | 65.78139 | 64.09684 | 62.55629 |
| 0.17082 | 0.183675 | 0.078466 | 0.178456 |
| 0.347562 | 0 | 0 | 0 |
| 0.95828 | 0.874539 | 0.858171 | 1.076834 |
| 5.465023 | 0.290906 | 1.193033 | 1.033656 |
| 0.321335 | 0.094232 | 0.178914 | 0.453414 |
| 16.31537 | 12.92586 | 13.21995 | 13.36961 |
| 736.8352 | 652.4828 | 644.6304 | 649.4409 |
| 34.53508 | 28.60084 | 30.60471 | 30.87464 |
| 0.045741 | 0.098366 | 0.028014 | 0.13653 |
| 0 | 0 | 0 | 0 |
| 0 | 0 | 0 | 0 |
| 0 | 0 | 0 | 0 |
| 14.47167 | 13.69994 | 13.14909 | 12.48718 |
| 11.24621 | 9.628424 | 9.876962 | 10.08321 |
| 15.21001 | 14.13238 | 14.81391 | 15.9237 |
| 0 | 0 | 0 | 0 |
| 62.95544 | 57.12694 | 55.0966 | 54.32331 |
| 1.519661 | 1.128256 | 0.930735 | 1.036798 |
| 0 | 0.085332 | 0 | 0 |
| 0 | 0.076531 | 0.174368 | 0 |
| 0.0744 | 0 | 0 | 0 |
| 5.026138 | 5.859497 | 6.480673 | 6.569459 |
| 1.228164 | 0.728602 | 0.466885 | 0.522497 |
| 7.070308 | 6.83549 | 7.350109 | 6.460772 |

| | | | |
|---|---|---|---|
| 31.34714 | 27.10392 | 26.99166 | 27.04857 |
| 6.263575 | 3.352642 | 3.199652 | 3.030887 |
| 1.290719 | 1.236703 | 1.534072 | 1.586826 |
| 1.853592 | 1.699026 | 1.730799 | 1.469348 |
| 59.79567 | 50.74789 | 48.61577 | 51.27254 |
| 7.999968 | 8.125895 | 6.834238 | 6.308955 |
| 13.37121 | 14.35581 | 13.78481 | 13.77333 |
| 0.569765 | 0.525123 | 0.299108 | 0.842238 |
| 23.10028 | 20.11052 | 19.32832 | 19.1443 |
| 0 | 0 | 0.039813 | 0.012935 |
| 0 | 0 | 0 | 0.040277 |
| 0.343922 | 0.277354 | 0.231703 | 0.246375 |
| 0 | 0 | 0 | 0 |
| 4.840228 | 4.359089 | 3.912481 | 4.253564 |
| 5.550693 | 3.701669 | 3.672031 | 4.179548 |
| 90.74156 | 86.8242 | 86.33196 | 87.05834 |
| 1.39025 | 1.681735 | 0.638606 | 1.037429 |
| 30.89464 | 35.14898 | 33.70648 | 34.20426 |
| 0 | 1.7655 | 0.670414 | 0 |
| 3.604012 | 3.875239 | 3.5749 | 3.69497 |
| 0 | 0 | 0.014781 | 0.014407 |
| 24.02565 | 22.44853 | 21.36134 | 20.21487 |
| 0 | 0.182205 | 0.103783 | 0.101159 |
| 0 | 0 | 0 | 0 |
| 11.75307 | 8.857843 | 8.381598 | 8.073527 |
| 11.19474 | 10.7487 | 11.14079 | 9.844079 |
| 0 | 0.066349 | 0.05039 | 0.049115 |
| 0.882373 | 0.862525 | 0.884324 | 0.785342 |
| 3.203204 | 3.138362 | 3.446129 | 3.472206 |
| 2.55086 | 1.347355 | 1.260808 | 1.335787 |
| 0 | 0 | 0.566272 | 0 |
| 0 | 0 | 0 | 0 |
| 0.345044 | 0 | 0.422653 | 0.411964 |
| 3.619072 | 3.235319 | 3.31193 | 3.667812 |
| 0 | 0 | 0 | 0 |
| 2.565174 | 0.172389 | 1.571072 | 2.297014 |
| 5.063465 | 6.142542 | 5.208394 | 5.541722 |
| 253.7318 | 217.3269 | 208.1916 | 214.1316 |
| 8.182176 | 7.688746 | 7.437937 | 7.19386 |
| 0 | 0.029091 | 0 | 0.064604 |
| 220.0841 | 207.6847 | 207.6104 | 217.2599 |
| 0.471563 | 0.519727 | 0.346577 | 0.436342 |
| 0.376624 | 0.385684 | 0.395431 | 0.535321 |
| 1.159376 | 1.457324 | 1.040108 | 1.306249 |
| 0.253412 | 0.62671 | 0.527697 | 0.393328 |
| 0.221298 | 0.039659 | 0.180716 | 0.132109 |
| 167.3012 | 99.43982 | 100.6748 | 98.4027 |
| 11.21105 | 9.047726 | 12.3382 | 9.691869 |
| 64.40569 | 73.65614 | 73.43483 | 72.14688 |
| 2.300085 | 1.367163 | 1.452464 | 1.620418 |
| 34.17963 | 32.99813 | 36.14923 | 35.81667 |
| 0.06463 | 0.05212 | 0.059375 | 0.019291 |
| 0.831391 | 0.548566 | 0.509196 | 0.586559 |
| 69.47991 | 90.33591 | 89.55822 | 93.03943 |

| | | | |
|---|---|---|---|
| 0 | 0 | 0 | 0 |
| 23.80802 | 24.50561 | 22.99918 | 22.53407 |
| 0.053834 | 0 | 0 | 0 |
| 0.057751 | 0.031049 | 0.247594 | 0 |
| 0 | 0 | 0 | 0 |
| 0.44501 | 0.957 | 0.545103 | 0 |
| 2.604524 | 2.13032 | 2.126897 | 2.179425 |
| 0.099929 | 0.214898 | 0.306013 | 0.238619 |
| 5.036313 | 3.446119 | 5.23439 | 4.737591 |
| 16.93343 | 14.51181 | 14.28446 | 14.55267 |
| 35.29838 | 18.81268 | 18.24097 | 19.46253 |
| 1.828961 | 1.265859 | 1.150212 | 1.305191 |
| 8.474042 | 7.376197 | 4.448598 | 5.299678 |
| 29.07528 | 22.90636 | 23.5713 | 22.63981 |
| 12.38199 | 10.37695 | 9.665249 | 9.239661 |
| 1.981971 | 1.71189 | 1.930272 | 1.70689 |
| 8.121019 | 5.62252 | 5.868389 | 5.440963 |
| 0.167242 | 0.128448 | 0.175593 | 0.228203 |
| 1.208106 | 1.119849 | 0.96955 | 0.596862 |
| 3.871223 | 1.248768 | 5.216152 | 0.924408 |
| 0 | 0 | 0 | 0 |
| 50.96657 | 43.60155 | 42.40939 | 44.4654 |
| 0 | 0 | 0 | 0 |
| 12.03283 | 14.27459 | 14.62792 | 13.75138 |
| 2.205648 | 1.185819 | 1.244925 | 1.36835 |
| 0.140959 | 0.060627 | 0.069066 | 0.067319 |
| 24.04287 | 19.72853 | 20.74854 | 21.90625 |
| 9.214185 | 5.470464 | 7.524454 | 10.5963 |
| 6.325508 | 5.317231 | 5.029954 | 5.069657 |
| 0.016442 | 0.00884 | 0.01007 | 0.009815 |
| 1.449184 | 1.409841 | 1.69061 | 1.812643 |
| 1.218638 | 1.422208 | 1.092248 | 1.100115 |
| 0.378979 | 0.261964 | 0.663173 | 0.226241 |
| 3.341931 | 3.677003 | 3.585879 | 2.783786 |
| 0.992001 | 0.476591 | 0.672197 | 0.327599 |
| 4.71159 | 4.210605 | 4.177087 | 4.173242 |
| 0.078965 | 0 | 0 | 0 |
| 0.601573 | 0.076744 | 0.77435 | 0.206953 |
| 4.777014 | 1.325553 | 3.208877 | 1.471873 |
| 20.62335 | 20.6013 | 20.96781 | 20.7902 |
| 0.31744 | 0.68266 | 0.38884 | 0.379007 |
| 1.813254 | 1.09184 | 1.57698 | 1.602049 |
| 0 | 0.033139 | 0 | 0 |
| 5.632952 | 5.520585 | 5.318704 | 5.009061 |
| 4.235783 | 2.638501 | 3.292023 | 3.104139 |
| 10.23975 | 8.121118 | 6.760723 | 6.907682 |
| 1.840234 | 2.061171 | 2.723761 | 2.471786 |
| 6.104172 | 6.205713 | 6.104015 | 5.991479 |
| 0.700236 | 0.376467 | 1.715472 | 0.836045 |
| 25.39854 | 25.29689 | 25.75357 | 24.98362 |
| 0.207553 | 0.204575 | 0.190677 | 0.227157 |
| 23.78583 | 21.66316 | 22.12368 | 21.51122 |
| 3.868364 | 1.527666 | 1.912612 | 1.71309 |
| 4.225582 | 4.753938 | 4.073718 | 4.344413 |

| | | | |
|---|---|---|---|
| 0.799674 | 0.805434 | 0.83075 | 0.797656 |
| 2.827976 | 1.33035 | 1.861931 | 3.925131 |
| 4.778371 | 3.199968 | 3.748065 | 3.503148 |
| 37.46046 | 36.24589 | 34.07003 | 36.18378 |
| 0.732555 | 0 | 0 | 0 |
| 0.025477 | 0 | 0 | 0.030418 |
| 4.497308 | 3.666928 | 3.785711 | 3.359151 |
| 2.807295 | 3.134662 | 3.835501 | 2.449367 |
| 0.049241 | 0.008824 | 0.010053 | 0.019597 |
| 7.07294 | 5.602066 | 5.782325 | 4.863256 |
| 0.294339 | 0.143859 | 0.278602 | 0.319478 |
| 0.044735 | 0.038481 | 0.054797 | 0.032047 |
| 0.119451 | 0.048165 | 0.03658 | 0.053482 |
| 59.19595 | 55.73585 | 52.51229 | 54.69982 |
| 0.040318 | 0.130058 | 0 | 0 |
| 0.278456 | 0.228962 | 0.260832 | 0.234679 |
| 0.065317 | 0.070232 | 0 | 0 |
| 0.053702 | 0.019248 | 0.087708 | 0.042745 |
| 0 | 0 | 0 | 0 |
| 7.171639 | 5.643818 | 4.917531 | 5.351603 |
| 20.12407 | 18.98174 | 18.67017 | 18.55347 |
| 0.440889 | 0.474069 | 0.540056 | 0.789598 |
| 0 | 0 | 0 | 0.013944 |
| 0 | 0 | 0 | 0 |
| 0.328387 | 0.380261 | 0.185653 | 0.331757 |
| 3.641104 | 2.927929 | 2.274767 | 2.578478 |
| 46.5249 | 43.4814 | 45.02476 | 44.23146 |
| 8.315677 | 10.45497 | 9.482805 | 9.33145 |
| 0 | 0 | 0.078926 | 0 |
| 4.370077 | 4.720218 | 4.989688 | 4.816288 |
| 0.044752 | 0.01604 | 0 | 0 |
| 80.05697 | 74.08391 | 72.74212 | 73.51095 |
| 0 | 0 | 0.041484 | 0 |
| 0 | 0 | 0 | 0 |
| 0.08141 | 0.092143 | 0.094472 | 0.097199 |
| 0.338663 | 0.29132 | 0.165935 | 0.080869 |
| 145.9716 | 133.4652 | 132.2003 | 133.3204 |
| 0.191229 | 0 | 0.234241 | 0.228318 |
| 2.915268 | 2.554169 | 4.232276 | 2.191539 |
| 0.951726 | 0.767513 | 0.72862 | 0.781214 |
| 0 | 0 | 0 | 0 |
| 0.040872 | 0 | 0.050065 | 0 |
| 0.924583 | 0 | 0 | 0.551952 |
| 0.021862 | 0 | 0 | 0 |
| 0 | 0 | 0.016206 | 0 |
| 30.10893 | 27.50885 | 26.40149 | 24.72815 |
| 164.3872 | 169.3685 | 168.2111 | 166.0382 |
| 0 | 0.052837 | 0 | 0 |
| 0 | 0 | 0 | 0 |
| 16.68645 | 14.7448 | 14.38431 | 15.58091 |
| 3.257407 | 2.84874 | 3.121127 | 2.540927 |
| 0 | 0 | 0.540056 | 0 |
| 0.095423 | 0.025651 | 0 | 0.056965 |
| 14.52037 | 13.15266 | 13.9323 | 14.46388 |

| | | | |
|---|---|---|---|
| 1.752106 | 0.997393 | 1.325592 | 1.415124 |
| 20.5709 | 20.71299 | 18.55652 | 17.74455 |
| 83.04841 | 79.20493 | 76.55668 | 75.3756 |
| 16.33982 | 17.73119 | 16.63828 | 17.89313 |
| 21.45121 | 19.36294 | 18.84274 | 18.13034 |
| 5.957893 | 4.355248 | 4.846082 | 4.21744 |
| 0 | 0 | 0 | 0 |
| 0 | 0 | 0 | 0 |
| 1.606515 | 1.642461 | 1.483959 | 1.383544 |
| 0 | 0 | 0 | 0 |
| 0.058425 | 0 | 0 | 0 |
| 0 | 0.040251 | 0 | 0.044694 |
| 0 | 0.399996 | 0.455672 | 0.444149 |
| 1.95637 | 1.033973 | 1.380979 | 1.979495 |
| 29.22036 | 26.03706 | 21.69024 | 25.25055 |
| 0.256 | 0 | 0 | 0 |
| 42.62984 | 36.17775 | 37.96696 | 37.00684 |
| 0.090182 | 0.193937 | 0.110466 | 0.107673 |
| 2.07867 | 0.883646 | 0.888214 | 0.692602 |
| 13.48055 | 11.54691 | 11.19502 | 10.69368 |
| 55.83799 | 55.37619 | 50.43559 | 57.62405 |
| 0.813102 | 0.426485 | 0.51014 | 0.473562 |
| 0 | 0 | 0.102868 | 0 |
| 0.487744 | 0.409727 | 0.61612 | 0.509549 |
| 15.48252 | 15.1295 | 13.68694 | 15.20098 |
| 7.339564 | 6.359853 | 5.844783 | 6.686036 |
| 11.03269 | 11.83714 | 11.68877 | 10.53223 |
| 0.057507 | 0 | 0 | 0 |
| 0.221857 | 0.238553 | 0.03397 | 0.099332 |
| 14.11131 | 13.284 | 12.81936 | 11.61671 |
| 0.096 | 0.103225 | 0 | 0.114619 |
| 10.13828 | 9.35995 | 9.066558 | 9.677443 |
| 0 | 0 | 0 | 0 |
| 0 | 0 | 0 | 0 |
| 1.75059 | 1.505867 | 2.14434 | 3.344182 |
| 0 | 0 | 0 | 0.093352 |
| 0.526006 | 0.565591 | 0.582954 | 0.717741 |
| 1.469631 | 0.84279 | 1.680175 | 1.637686 |
| 2.044737 | 1.864834 | 1.818556 | 1.450283 |
| 0 | 0.376467 | 0 | 0.418023 |
| 8.409799 | 9.637051 | 8.753745 | 9.805163 |
| 4.008295 | 3.521747 | 3.343289 | 3.854627 |
| 0.523253 | 0.675158 | 1.025513 | 1.374422 |
| 2.018579 | 1.085246 | 1.978086 | 1.312154 |
| 7.357657 | 7.630828 | 7.510479 | 6.790979 |
| 0 | 0 | 0 | 0.039235 |
| 0.231708 | 0.622865 | 0.425738 | 1.106591 |
| 9.100069 | 9.510825 | 9.460812 | 10.89544 |
| 0.12469 | 0.083796 | 0.057276 | 0.130264 |
| 0.025127 | 0.027018 | 0.153895 | 0.060001 |
| 0 | 0 | 0 | 0.049608 |
| 10.63027 | 12.10662 | 10.24751 | 13.89359 |
| 4.545472 | 4.408379 | 4.749057 | 4.1235 |
| 26.35555 | 28.08084 | 27.21883 | 25.67058 |

| | | | |
|---|---|---|---|
| 351.6128 | 328.8331 | 332.6391 | 334.4791 |
| 0.302644 | 0.16271 | 0.494289 | 0.421565 |
| 1.166875 | 0.688056 | 0.876044 | 1.190952 |
| 14.81042 | 13.13137 | 12.59796 | 13.42297 |
| 0.377286 | 0.433657 | 0.350594 | 0.40386 |
| 0.018798 | 0.040426 | 0 | 0 |
| 6.378131 | 5.371639 | 5.522791 | 5.289346 |
| 0 | 0 | 0 | 0 |
| 0.425505 | 0.718972 | 0.397114 | 0.532223 |
| 3.241287 | 2.406459 | 2.316027 | 1.888894 |
| 8.119789 | 7.372726 | 7.072802 | 7.923246 |
| 26.09098 | 24.37236 | 24.96835 | 24.92102 |
| 2.988717 | 3.550145 | 3.430945 | 4.240945 |
| 1.628827 | 1.00414 | 1.024198 | 1.024227 |
| 39.78828 | 34.07173 | 35.29711 | 33.99638 |
| 22.08829 | 19.6112 | 16.77502 | 18.53594 |
| 5.06244 | 4.173871 | 4.378417 | 4.325626 |
| 0.059446 | 0 | 0 | 0 |
| 0 | 0 | 0 | 0 |
| 571.3112 | 482.5005 | 471.6933 | 479.9787 |
| 0.012147 | 0.013061 | 0.014879 | 0 |
| 0.093365 | 0 | 0 | 0 |
| 3.830812 | 1.361688 | 1.085857 | 0.907198 |
| 70.46498 | 55.68854 | 57.7041 | 56.1775 |
| 2.733247 | 1.985076 | 1.90896 | 1.746181 |
| 0.042522 | 0.045722 | 0.052086 | 0.020308 |
| 2.442384 | 2.828205 | 2.838313 | 2.592071 |
| 0 | 0.021227 | 0.012091 | 0.011785 |
| 0.127755 | 0.078497 | 0.178846 | 0.130742 |
| 0.088123 | 0 | 0.035982 | 0.140286 |
| 7.142407 | 8.319917 | 6.561682 | 3.553193 |
| 0.184558 | 0.330746 | 1.281063 | 0.220353 |
| 42.4343 | 40.2443 | 41.68598 | 37.95646 |
| 0.185483 | 0.3419 | 0.292117 | 0.316367 |
| 0.377905 | 0.365711 | 0.162017 | 0.428639 |
| 1.904642 | 0 | 0 | 1.516029 |
| 22.24279 | 23.38523 | 23.57261 | 22.15029 |
| 0.421381 | 0.453093 | 0 | 0 |
| 75.36666 | 81.83486 | 77.53684 | 80.20529 |
| 3.593664 | 2.495572 | 3.607167 | 2.622063 |
| 5.259226 | 5.832532 | 4.766617 | 4.758709 |
| 0.933648 | 1.081135 | 0.703784 | 1.800713 |
| 12.2004 | 13.88343 | 15.12939 | 14.87548 |
| 0 | 0 | 0 | 0 |
| 0 | 0 | 0 | 0 |
| 16.2703 | 14.32383 | 13.67066 | 13.93201 |
| 1.53247 | 0.4708 | 1.072663 | 0.784153 |
| 0 | 0 | 0 | 0.022506 |
| 0.287132 | 0.137218 | 0.293096 | 0.247593 |
| 0 | 0 | 0.102147 | 0 |
| 0 | 0 | 0 | 0 |
| 1.518299 | 1.081818 | 1.075548 | 1.179393 |
| 3.387808 | 4.818775 | 4.280512 | 5.032197 |
| 20.0821 | 22.10949 | 21.00976 | 20.78005 |

| | | | |
|---|---|---|---|
| 0 | 0.082182 | 0 | 0 |
| 3.159465 | 3.445424 | 3.403497 | 3.183661 |
| 4.083431 | 2.682154 | 2.48993 | 2.591625 |
| 0.92925 | 0.758 | 0.863508 | 0.765156 |
| 5.04901 | 5.93012 | 5.677197 | 4.63712 |
| 0 | 0.027409 | 0 | 0 |
| 0 | 0.116628 | 0 | 0.129501 |
| 12.67347 | 11.55071 | 10.42329 | 11.70887 |
| 4.293879 | 6.430853 | 6.480673 | 5.218216 |
| 0.488092 | 0.46651 | 0.365368 | 0.161877 |
| 0.044459 | 0.095611 | 0.163378 | 0.053082 |
| 13.94629 | 12.22716 | 12.47214 | 12.18795 |
| 0 | 0 | 0 | 0.638776 |
| 0.240485 | 0.258583 | 0.294576 | 0.143563 |
| 9.185388 | 8.037914 | 8.30176 | 7.766815 |
| 0 | 0.06117 | 0.069685 | 0.067922 |
| 5.113961 | 4.466871 | 4.971066 | 5.238223 |
| 2.008802 | 1.119989 | 2.187227 | 2.043086 |
| 197.7302 | 205.6302 | 204.7682 | 203.9127 |
| 4.139183 | 3.371228 | 3.329675 | 3.171712 |
| 0 | 0.091509 | 0.104247 | 0 |
| 0 | 0 | 0 | 0 |
| 26.21217 | 23.60447 | 23.13029 | 25.26251 |
| 0 | 0 | 0 | 0 |
| 0 | 0 | 0 | 0 |
| 0.15872 | 0 | 0 | 0 |
| 5.605433 | 6.1569 | 5.463454 | 6.404743 |
| 82.04331 | 80.05115 | 80.21922 | 79.71098 |
| 0.352711 | 0 | 0 | 0 |
| 18.6036 | 14.99589 | 14.33992 | 14.55461 |
| 0.029447 | 0 | 0.180353 | 0.21095 |
| 0.341994 | 0.367731 | 0.354468 | 0.251276 |
| 0 | 0.090299 | 0 | 0 |
| 0 | 0 | 0 | 0 |
| 0 | 0 | 0 | 0 |
| 0.270686 | 0.304286 | 0.437069 | 0.293804 |
| 0 | 0.183511 | 0 | 0.101884 |
| 0.145615 | 0.156573 | 0.118911 | 0.115904 |
| 0 | 0 | 0 | 0 |
| 5.745526 | 5.096781 | 4.222701 | 4.287412 |
| 0 | 0 | 0 | 0 |
| 1.143929 | 0.461257 | 0 | 0 |
| 0.087262 | 0.125106 | 0.10689 | 0.034729 |
| 0.034871 | 0.037495 | 0.106785 | 0.104085 |
| 1.822624 | 1.061552 | 0.93024 | 1.088059 |
| 1.667425 | 1.386749 | 1.308767 | 1.294999 |
| 41.08514 | 39.07647 | 38.14051 | 38.93414 |
| 7.817858 | 6.17314 | 5.466922 | 6.580373 |
| 0.092819 | 0 | 0 | 0 |
| 11.00825 | 9.643822 | 9.592194 | 10.03571 |
| 1.778637 | 1.290356 | 0.944977 | 1.074593 |
| 0.255657 | 0.160356 | 0.07829 | 0.15262 |
| 1.848891 | 1.835107 | 1.881486 | 1.799945 |
| 0.688035 | 1.523147 | 1.313762 | 1.473828 |

| | | | |
|---|---|---|---|
| 0 | 0.575275 | 0 | 0 |
| 0.509462 | 0.988717 | 0.639273 | 1.00884 |
| 14.93348 | 13.6813 | 13.35911 | 13.78724 |
| 77.02966 | 69.07867 | 68.43282 | 67.83027 |
| 0 | 0 | 0 | 0 |
| 0.028524 | 0.010224 | 0.03494 | 0.05676 |
| 5.012215 | 4.850478 | 4.118638 | 4.637852 |
| 0.051352 | 0.096629 | 0.015726 | 0.091967 |
| 2.307188 | 2.210536 | 1.715472 | 1.800713 |
| 0 | 0 | 0 | 0.270719 |
| 6.055118 | 4.793465 | 3.955767 | 4.358653 |
| 10.24984 | 8.304717 | 7.957573 | 8.158519 |
| 24.88797 | 11.34834 | 11.36456 | 10.72551 |
| 13.18955 | 11.73291 | 10.52576 | 12.00208 |
| 0.156632 | 0.05614 | 0.063954 | 0.062337 |
| 0 | 0 | 0 | 0 |
| 2.693669 | 2.392667 | 2.110887 | 2.46701 |
| 55.18351 | 53.52433 | 59.10856 | 53.21356 |
| 13.22581 | 13.56322 | 13.81761 | 13.50565 |
| 12.30941 | 0.713306 | 25.03988 | 1.261402 |
| 0.038587 | 0.041491 | 0 | 0 |
| 0.014669 | 0 | 0 | 0 |
| 21.95777 | 15.93991 | 15.42801 | 16.04925 |
| 3.170894 | 2.751672 | 2.804292 | 2.403486 |
| 0 | 0 | 0 | 0 |
| 0 | 0 | 0 | 0.113475 |
| 0.217177 | 0.467042 | 0.199519 | 0.777894 |
| 2.485666 | 2.143752 | 2.378714 | 1.731191 |
| 0.996888 | 0.5885 | 0.454924 | 0.44342 |
| 39.33895 | 28.02035 | 27.96531 | 28.88709 |
| 21.29591 | 20.53505 | 19.05738 | 19.2912 |
| 4.725486 | 5.340022 | 4.141568 | 4.827431 |
| 0.165563 | 0.124616 | 0.202803 | 0.276744 |
| 13.70338 | 8.913192 | 9.641132 | 9.516826 |
| 30.72137 | 22.22285 | 22.6753 | 23.24039 |
| 0 | 0 | 0 | 0 |
| 4.89215 | 3.53051 | 3.925016 | 3.510881 |
| 0 | 0 | 0 | 0 |
| 9.105198 | 8.210609 | 8.010011 | 8.598078 |
| 0.263072 | 0 | 0 | 0 |
| 14.63136 | 11.90645 | 12.01951 | 11.7742 |
| 3.029251 | 2.544706 | 2.666997 | 2.825601 |
| 8.786548 | 7.060019 | 6.931124 | 6.896062 |
| 0.201195 | 0 | 0 | 0.240216 |
| 0.061046 | 0 | 0 | 0 |
| 0.035089 | 0.018865 | 0 | 0 |
| 1.400472 | 0.501956 | 1.715472 | 2.786818 |
| 0 | 0.058049 | 0 | 0.064457 |
| 1.176916 | 0.790929 | 1.081225 | 0.556216 |
| 0 | 0 | 1.100492 | 0 |
| 19.95374 | 17.68782 | 17.66498 | 16.73386 |
| 0.68745 | 0.641352 | 0.495338 | 0.470741 |
| 20.98586 | 21.03869 | 20.9239 | 20.69549 |
| 101.374 | 90.8591 | 95.23026 | 89.42564 |

| | | | |
|---|---|---|---|
| 0.052708 | 0.056674 | 0.090388 | 0.138446 |
| 0 | 0 | 0 | 0 |
| 0 | 0.037647 | 0 | 0 |
| 44.59643 | 41.8918 | 41.67321 | 42.97481 |
| 0.539559 | 0.507644 | 0.908763 | 0.563679 |
| 0.054763 | 0.088326 | 0.1677 | 0.032692 |
| 0.012381 | 0.106499 | 0.106158 | 0.029564 |
| 22.62013 | 22.37954 | 24.67483 | 23.29177 |
| 7.765754 | 6.779355 | 6.60625 | 6.609677 |
| 0 | 0.305851 | 0.209054 | 0.067922 |
| 2.435276 | 1.722728 | 2.04102 | 2.065921 |
| 0.342561 | 0.122781 | 0.279741 | 0 |
| 6.641265 | 6.551438 | 6.605062 | 5.746943 |
| 1.074046 | 1.539834 | 0.877084 | 2.137259 |
| 16.17149 | 16.56738 | 16.94757 | 18.28889 |
| 86.38911 | 77.89636 | 82.17725 | 68.52586 |
| 13.11411 | 14.46416 | 14.92623 | 13.27197 |
| 23.70094 | 21.21158 | 21.51156 | 20.56981 |
| 5.752008 | 4.990564 | 4.940141 | 5.22569 |
| 0.132022 | 0 | 0.053906 | 0.052543 |
| 0.850287 | 0.609518 | 0.810084 | 0.676799 |
| 1.142785 | 2.047979 | 0.466608 | 2.728852 |
| 4.633445 | 3.498102 | 3.783747 | 3.609593 |
| 8.461628 | 7.106407 | 6.602054 | 6.620281 |
| 1.013107 | 0 | 0.62049 | 0 |
| 0 | 0 | 0 | 0 |
| 0 | 0 | 0 | 0.05813 |
| 0.485878 | 0.522444 | 0 | 0 |
| 0.096584 | 0.034618 | 0 | 0.076878 |
| 0.059894 | 0.064402 | 0.073366 | 0.214532 |
| 0 | 0.474069 | 0 | 0 |
| 0.462934 | 0.142221 | 0.081008 | 0.07896 |
| 6.132968 | 5.3492 | 6.190494 | 6.128227 |
| 0 | 0.254724 | 0.096726 | 0.09428 |
| 0.02073 | 0 | 0 | 0 |
| 0.542633 | 0 | 0 | 0 |
| 0 | 0.290906 | 0 | 0 |
| 0 | 0 | 0.047037 | 0 |
| 4.410169 | 1.852369 | 1.012898 | 0.822736 |
| 0.386871 | 0.232952 | 0.20851 | 0.24019 |
| 12.48082 | 9.840291 | 9.33217 | 9.761748 |
| 8.665474 | 5.811663 | 5.775036 | 5.751745 |
| 4.103127 | 3.338172 | 4.093482 | 3.848309 |
| 0 | 0 | 0.041278 | 0 |
| 14.86663 | 14.79565 | 15.98064 | 15.5339 |
| 0.390295 | 0.734419 | 0.478082 | 0.465993 |
| 1.955248 | 0.865692 | 0.986189 | 1.373215 |
| 0 | 0 | 0 | 0 |
| 0.354682 | 0 | 0.108615 | 0.211736 |
| 8.515523 | 8.414802 | 8.401018 | 8.74896 |
| 0 | 0 | 0 | 0 |
| 0.027829 | 0.029924 | 0.011363 | 0 |
| 0.393521 | 0 | 0 | 0 |
| 0 | 0.017522 | 0.019961 | 0 |

| | | | |
|---|---|---|---|
| 25.49986 | 27.87917 | 29.93705 | 30.29949 |
| 4.642138 | 3.914895 | 4.013836 | 3.953086 |
| 5.012215 | 4.025634 | 4.230324 | 3.740203 |
| 0.459319 | 0.329257 | 0.531374 | 0.457002 |
| 0.637407 | 1.05585 | 1.371633 | 1.028421 |
| 0.068288 | 0.031469 | 0.059748 | 0.104827 |
| 0.359276 | 0.218911 | 0.278721 | 0.285971 |
| 0.077049 | 0.082847 | 0.094379 | 0.045996 |
| 3.720394 | 3.033845 | 2.599746 | 3.338921 |
| 0 | 0.036312 | 0 | 0 |
| 0 | 0 | 0 | 0 |
| 97.38128 | 90.97893 | 90.53063 | 83.63238 |
| 0 | 0.069376 | 0 | 0 |
| 5.535045 | 5.577157 | 5.814646 | 5.251833 |
| 0.408856 | 0.439626 | 0.154098 | 0.337952 |
| 321.9953 | 333.3554 | 348.2118 | 336.5106 |
| 89.3219 | 100.8002 | 107.8645 | 104.3823 |
| 0 | 0 | 0 | 0 |
| 11.69939 | 7.35119 | 7.961596 | 8.162644 |
| 6.114853 | 4.543213 | 5.003073 | 4.988658 |
| 18.58432 | 13.28594 | 14.64304 | 17.15128 |
| 4.230803 | 4.515627 | 4.092386 | 4.175293 |
| 7.309751 | 6.954652 | 6.71542 | 6.74172 |
| 0 | 0.227553 | 0 | 0 |
| 0.256987 | 0.59213 | 0.49467 | 0.613659 |
| 0.506554 | 0.544675 | 1.24098 | 0 |
| 17.10198 | 15.35762 | 16.02253 | 16.3846 |
| 22.40888 | 21.89526 | 22.01279 | 20.36939 |
| 6.692401 | 6.575701 | 5.794915 | 6.796414 |
| 0.463191 | 0.323732 | 0.141844 | 0.331816 |
| 14.69333 | 16.86182 | 15.92667 | 14.65856 |
| 33.17251 | 29.28611 | 28.61865 | 28.38764 |
| 0 | 0 | 0.042667 | 0 |
| 4.192608 | 3.682982 | 3.668306 | 3.664929 |
| 0.434392 | 0.251506 | 0.69582 | 0.239373 |
| 9.11283 | 8.744145 | 7.89139 | 7.944021 |
| 6.339157 | 4.730895 | 6.594922 | 4.87295 |
| 2.262133 | 1.874124 | 2.134988 | 2.125274 |
| 0.154454 | 0.023725 | 0.054056 | 0.105377 |
| 0.057403 | 0.154308 | 0.210944 | 0.154207 |
| 0 | 0 | 0 | 0.025357 |
| 37.13178 | 37.5776 | 38.34894 | 38.82796 |
| 0 | 0 | 0 | 0 |
| 6.776001 | 6.395438 | 6.875753 | 6.791767 |
| 42.914 | 38.08783 | 36.70817 | 39.29799 |
| 0 | 0 | 0 | 0 |
| 0 | 0.361834 | 0 | 0.602662 |
| 29.82091 | 36.09342 | 34.27975 | 33.63652 |
| 0.481456 | 0.647112 | 0.324361 | 0.517351 |
| 9.322939 | 5.049405 | 4.610254 | 4.431829 |
| 42.36409 | 43.73234 | 45.42908 | 44.78546 |
| 71.40129 | 74.71695 | 77.52622 | 77.68742 |
| 3.915273 | 3.798854 | 3.536381 | 3.871918 |
| 12.23228 | 11.26537 | 10.46314 | 11.48573 |

| | | | |
|---|---|---|---|
| 5.427205 | 4.881385 | 4.975485 | 6.11302 |
| 1.169144 | 1.485699 | 1.47551 | 1.353597 |
| 0.069184 | 0.148782 | 0.063559 | 0.061952 |
| 0 | 0.190333 | 0 | 0 |
| 75.92829 | 65.86745 | 67.0487 | 61.97281 |
| 0 | 0 | 0 | 0.165505 |
| 69.17477 | 51.29221 | 49.31219 | 52.49245 |
| 3.086811 | 3.353158 | 3.412695 | 2.967294 |
| 1.425128 | 0.36056 | 0.30806 | 0.40036 |
| 7.566974 | 7.951523 | 7.768031 | 7.520257 |
| 1.126777 | 1.107129 | 0.951874 | 0.835022 |
| 0.331434 | 0.178188 | 0 | 0.065953 |
| 8.476051 | 8.582818 | 9.003027 | 9.176379 |
| 0 | 0 | 0 | 0 |
| 0.020176 | 0.021695 | 0 | 0.096358 |
| 0 | 0.08752 | 0 | 0 |
| 4.428726 | 3.797166 | 3.191091 | 4.337271 |
| 0.082839 | 0.017815 | 0.020294 | 0.039562 |
| 0.220299 | 0.03384 | 0.03855 | 0.112725 |
| 9.270381 | 7.086371 | 7.246884 | 6.882503 |
| 7.058742 | 6.768953 | 7.143997 | 6.565709 |
| 8.206814 | 7.432661 | 6.982935 | 7.595489 |
| 2.275295 | 1.953275 | 1.438485 | 1.774543 |
| 7.818949 | 7.351908 | 8.002082 | 7.415799 |
| 1.322668 | 1.77776 | 0.405042 | 2.368795 |
| 0 | 0 | 0.206464 | 0 |
| 294.4884 | 283.403 | 285.3984 | 275.9991 |
| 12.00405 | 9.94948 | 7.474558 | 8.121584 |
| 0 | 0 | 0 | 0 |
| 0.029741 | 0.095939 | 0.182155 | 0.355097 |
| 4.982312 | 4.558376 | 4.711054 | 5.400723 |
| 3.634246 | 2.312749 | 2.271264 | 2.922252 |
| 1.186026 | 0.648095 | 0.476326 | 0.58035 |
| 3.283865 | 2.122567 | 1.875654 | 2.334837 |
| 0.073143 | 0.62918 | 0.179189 | 0.261987 |
| 0 | 0 | 0 | 0 |
| 0.787042 | 0 | 0 | 0.469844 |
| 3.606161 | 2.294877 | 2.794603 | 2.899669 |
| 24.42316 | 20.47979 | 20.59552 | 20.07469 |
| 0.721455 | 0.110821 | 0 | 0 |
| 1.051687 | 0.370446 | 0.510853 | 0.649479 |
| 0 | 0 | 0 | 0 |
| 2.852265 | 2.191944 | 2.178493 | 2.063306 |
| 0 | 0.121182 | 0 | 0.201838 |
| 0.384557 | 0.265063 | 0.144939 | 0.223684 |
| 0.009282 | 0.00998 | 0 | 0 |
| 2.686961 | 2.222441 | 1.788075 | 2.051328 |
| 6.231286 | 5.201498 | 5.448452 | 4.747788 |
| 47.27714 | 50.83508 | 48.20721 | 46.30531 |
| 10.44767 | 9.667988 | 8.299054 | 7.711185 |
| 85.34789 | 67.74228 | 80.5405 | 55.3118 |
| 0 | 0 | 0.535101 | 0 |
| 10.34837 | 9.472632 | 9.817668 | 9.771281 |
| 2.410939 | 1.649696 | 1.543728 | 1.373847 |

| | | | |
|---|---|---|---|
| 7.820561 | 8.012684 | 7.075217 | 7.349824 |
| 3.836364 | 3.801542 | 3.255694 | 4.011609 |
| 1.468121 | 0.789303 | 1.858281 | 0.87643 |
| 0.087798 | 0.094406 | 0.143395 | 0.034942 |
| 0.023826 | 0.064047 | 0.102147 | 0.085341 |
| 12.87321 | 6.422374 | 5.930308 | 6.178985 |
| 4.570967 | 4.309329 | 3.980395 | 4.73328 |
| 0.736423 | 0.753218 | 0.638044 | 0.879251 |
| 25.96328 | 26.40816 | 26.35929 | 25.37354 |
| 0 | 0 | 0 | 0 |
| 5.0903 | 3.108 | 3.571942 | 3.374722 |
| 0.063615 | 0.102604 | 0 | 0.11393 |
| 1.275114 | 0.875964 | 1.026816 | 0.95856 |
| 17.61436 | 14.93639 | 15.10338 | 15.23438 |
| 11.01069 | 10.74474 | 10.67106 | 10.84768 |
| 0.022936 | 0 | 0 | 0 |
| 48450.55 | 52284.07 | 55951.56 | 53656.66 |
| 0.147266 | 0.158349 | 0.631365 | 0.058609 |
| 0.016725 | 0.161853 | 0.081947 | 0.019969 |
| 0.532142 | 0.686627 | 0.7822 | 0.737006 |
| 1.760126 | 1.693368 | 1.475173 | 0.553026 |
| 2.95449 | 2.698768 | 2.951439 | 2.928173 |
| 0 | 0 | 0.032189 | 0.031375 |
| 214.3995 | 261.0626 | 257.9447 | 262.6703 |
| 9.317615 | 6.617813 | 7.264524 | 7.804633 |
| 7.345269 | 5.676218 | 6.226127 | 5.762543 |
| 0.447099 | 0.240373 | 0.547663 | 0.133453 |
| 6.144911 | 4.518045 | 4.296262 | 4.279745 |
| 0.734528 | 0.752196 | 0.956867 | 1.016192 |
| 0 | 0 | 0 | 0.055249 |
| 11.0899 | 7.981826 | 8.197685 | 8.878199 |
| 8.223384 | 5.954258 | 5.483299 | 5.819714 |
| 2.949192 | 2.133312 | 2.36457 | 2.240752 |
| 24.9293 | 22.40416 | 20.69548 | 23.21354 |
| 0.897287 | 0.64321 | 0.806013 | 0.642789 |
| 0.462292 | 0.639106 | 1.051649 | 0.394252 |
| 0 | 0 | 0 | 0.184581 |
| 0.232727 | 0.100097 | 0 | 0 |
| 0.033675 | 0 | 0.041249 | 0 |
| 0 | 0 | 0.029134 | 0 |
| 0.210225 | 0 | 0.128755 | 0.06275 |
| 16.13964 | 15.47274 | 14.62564 | 14.42784 |
| 0.313401 | 0.247123 | 0.3583 | 0.349239 |
| 0.541971 | 1.165517 | 0.663874 | 0.369763 |
| 0.147799 | 0.026487 | 0.060348 | 0.117643 |
| 8.73576 | 6.438523 | 7.736618 | 7.173716 |
| 0.103527 | 0.02783 | 0.103036 | 0.154508 |
| 7.755671 | 7.71467 | 7.641007 | 7.629855 |
| 35.1165 | 32.86667 | 31.83508 | 30.51871 |
| 75.04353 | 80.08568 | 77.53819 | 76.96984 |
| 4.039267 | 4.304122 | 4.212314 | 4.56199 |
| 6.002545 | 5.58479 | 4.800185 | 5.272929 |
| 5.316077 | 4.79419 | 4.026109 | 4.948024 |
| 38.02838 | 28.58855 | 31.67964 | 30.58993 |

| | | | |
|---|---|---|---|
| 7.616487 | 5.89657 | 6.33504 | 5.926424 |
| 0.025738 | 0 | 0.010509 | 0.010243 |
| 0.269017 | 0.096421 | 0 | 0.107064 |
| 0 | 0 | 0 | 0.124401 |
| 22.75683 | 20.19264 | 19.94083 | 21.40396 |
| 15.16296 | 9.324992 | 9.687604 | 10.54969 |
| 4.841464 | 4.333709 | 4.142128 | 3.843706 |
| 0.197168 | 0 | 0.120758 | 0.353112 |
| 5.309106 | 4.995072 | 5.690347 | 4.704576 |
| 0.164193 | 0 | 0.033521 | 0.032673 |
| 0.365638 | 0.464638 | 0.264656 | 0.23812 |
| 0.135658 | 0.145867 | 0.166171 | 0.161969 |
| 4.435715 | 4.477522 | 5.13772 | 5.187932 |
| 11.45075 | 10.55113 | 10.11063 | 10.1018 |
| 15.50853 | 4.950586 | 13.87656 | 12.4407 |
| 9.615831 | 8.293321 | 8.931984 | 8.404511 |
| 344.0255 | 345.7838 | 350.4176 | 346.9512 |
| 0.132267 | 0 | 0 | 0.07896 |
| 0.057786 | 0.093203 | 0.070784 | 0.103491 |
| 0 | 0 | 0 | 0 |
| 8.596079 | 7.064887 | 7.682434 | 6.635469 |
| 0.055432 | 0 | 0 | 0 |
| 25.99129 | 20.72892 | 22.92291 | 20.07343 |
| 46.11605 | 35.40829 | 31.83364 | 37.06774 |
| 0 | 0 | 0 | 0 |
| 1.44291 | 1.790192 | 1.49554 | 1.06016 |
| 1.405176 | 0.638765 | 0.895602 | 1.227591 |
| 0.151483 | 0.108589 | 0.123703 | 0 |
| 1.627237 | 0.999827 | 1.328828 | 1.295224 |
| 8.266124 | 5.423795 | 6.566938 | 5.297273 |
| 9.201944 | 6.588078 | 6.605602 | 7.424889 |
| 31.22152 | 37.10079 | 34.08754 | 34.5246 |
| 1.949795 | 1.379297 | 1.257027 | 1.776596 |
| 14.16373 | 15.80493 | 15.65939 | 14.52376 |
| 19.46121 | 18.23753 | 18.6532 | 18.89383 |
| 1.316399 | 0.687513 | 1.013565 | 0.943028 |
| 3.51819 | 2.202407 | 2.479448 | 2.129039 |
| 0.075302 | 0 | 0 | 0 |
| 14.71723 | 12.77948 | 15.17779 | 14.40147 |
| 17.6207 | 18.59591 | 19.09356 | 19.00031 |
| 0.192298 | 0.031811 | 0.199312 | 0.123628 |
| 0.190084 | 0 | 0 | 0.340426 |
| 6.799631 | 7.11104 | 6.2753 | 7.028538 |
| 15.2327 | 14.37639 | 13.98739 | 14.67936 |
| 0.243249 | 0.196167 | 0.111736 | 0.145214 |
| 39.89577 | 44.38946 | 37.94025 | 40.78928 |
| 0.293151 | 0.090061 | 0.102596 | 0.100002 |
| 6.502238 | 6.631186 | 5.501426 | 5.84251 |
| 0.462292 | 0.497082 | 0 | 0 |
| 0.146286 | 0 | 0.089595 | 0 |
| 26.78403 | 25.4236 | 24.58122 | 24.64416 |
| 0 | 0 | 0 | 0 |
| 0 | 0 | 0.472063 | 0 |
| 25.42604 | 18.88913 | 22.08462 | 16.00662 |

| | | | |
|---|---|---|---|
| 2.59278 | 2.034417 | 2.188837 | 1.966152 |
| 124.0903 | 60.92222 | 63.88766 | 59.53859 |
| 25.45157 | 29.18472 | 29.10551 | 28.29472 |
| 2.353119 | 2.497133 | 2.449092 | 1.909726 |
| 0 | 0.56574 | 0.322243 | 0 |
| 27.30299 | 23.83424 | 25.83011 | 25.1489 |
| 0 | 0 | 0 | 0 |
| 0.151451 | 0.227988 | 0.111309 | 0.216989 |
| 2.578486 | 3.224356 | 2.76072 | 3.238209 |
| 6.031366 | 4.623469 | 4.948878 | 4.341356 |
| 20.90461 | 18.98127 | 20.73815 | 19.44338 |
| 12.3294 | 11.27768 | 11.89073 | 12.15621 |
| 0 | 0 | 0 | 0 |
| 0.855094 | 0.749178 | 0.814664 | 0.680625 |
| 0 | 0 | 0 | 0 |
| 3.133202 | 2.194484 | 2.13023 | 2.77992 |
| 33.59577 | 16.07095 | 14.25748 | 17.68701 |
| 0.144291 | 0.15515 | 0.176746 | 0.344552 |
| 10.45658 | 11.19635 | 11.36864 | 10.96593 |
| 11.40828 | 10.27313 | 9.195266 | 9.977381 |
| 0.33728 | 0.533328 | 0.48605 | 0.402695 |
| 59.36225 | 48.72066 | 49.14853 | 47.87288 |
| 7.497445 | 6.526122 | 5.863381 | 6.14137 |
| 0.826053 | 0.507554 | 0.722752 | 0.493132 |
| 0.054171 | 0.029124 | 0.033178 | 0.016169 |
| 4.10856 | 4.46426 | 4.555896 | 4.595592 |
| 0.153024 | 0.16454 | 0.249924 | 0.182703 |
| 30.40483 | 23.66198 | 23.47219 | 25.32637 |
| 4.425283 | 3.521154 | 3.559552 | 3.768938 |
| 0 | 0 | 0 | 0 |
| 0.08928 | 0.032 | 0.072908 | 0.035532 |
| 22.41721 | 24.35155 | 24.92406 | 24.89523 |
| 0.250941 | 0.067457 | 0.153692 | 0.149805 |
| 5.160153 | 3.338116 | 3.494425 | 2.855077 |
| 1.544304 | 1.176204 | 2.088703 | 1.613342 |
| 93.06156 | 85.3811 | 88.18147 | 83.68394 |
| 50.72482 | 49.52812 | 50.26577 | 44.66429 |
| 4.794961 | 2.689991 | 3.370858 | 3.833217 |
| 22.54388 | 20.50245 | 19.87215 | 24.21202 |
| 2.367146 | 1.272645 | 1.115221 | 1.304423 |
| 0.065027 | 0.139842 | 0.17922 | 0.135868 |
| 0.204069 | 0.316949 | 0.055549 | 0.162432 |
| 1.988991 | 2.338954 | 1.865978 | 1.906155 |
| 33.90974 | 32.75332 | 31.80678 | 33.26054 |
| 2.952933 | 4.233549 | 2.48677 | 3.378747 |
| 0.774665 | 0.944025 | 0.695864 | 1.04823 |
| 0.847763 | 0.671012 | 0.70672 | 0.590442 |
| 0.027764 | 0.059708 | 0.034009 | 0.033149 |
| 0 | 0 | 0.394095 | 0.384129 |
| 0 | 0 | 0.333292 | 0 |
| 11.16959 | 9.476244 | 10.2812 | 8.710743 |
| 0 | 0 | 0 | 0 |
| 0.320647 | 0.402241 | 0.785536 | 0.223321 |
| 0.178471 | 0.076761 | 0 | 0.127851 |

| | | | |
|---|---|---|---|
| 17.759 | 18.40825 | 16.83235 | 15.97063 |
| 0 | 1.177 | 0 | 0.653461 |
| 6.792871 | 6.72791 | 7.240267 | 7.559147 |
| 0 | 0 | 0 | 1.043139 |
| 0.618093 | 0.590763 | 0.925365 | 0.464648 |
| 0 | 0 | 0 | 0 |
| 0.612706 | 0.345095 | 0.536085 | 0.592199 |
| 0.310726 | 0.380194 | 0.367491 | 0.281441 |
| 3.375878 | 3.021324 | 2.909494 | 2.546292 |
| 0 | 0 | 0 | 0.823929 |
| 3.359593 | 3.22147 | 2.832573 | 3.26451 |
| 1161.691 | 700.9812 | 596.1266 | 702.2781 |
| 496.4804 | 433.5091 | 412.8132 | 442.79 |
| 0 | 0 | 0 | 0 |
| 5.362458 | 5.3492 | 5.381509 | 4.70545 |
| 436.6662 | 386.1794 | 374.1939 | 373.8102 |
| 199.036 | 218.5026 | 217.3072 | 211.3832 |
| 21.36554 | 21.59398 | 20.85232 | 24.03652 |
| 70.18487 | 76.19785 | 72.45757 | 76.3601 |
| 0 | 0.029871 | 0 | 0 |
| 0.11757 | 0 | 0 | 0 |
| 236.2514 | 175.0855 | 174.1859 | 183.0951 |
| 47.04381 | 55.66851 | 56.07566 | 54.87335 |
| 1.133715 | 1.219035 | 0.462905 | 0.451199 |
| 0.099754 | 0.053631 | 0.020365 | 0.01985 |
| 0.100739 | 0.072214 | 0.041133 | 0 |
| 9.945724 | 8.907588 | 8.853582 | 8.317943 |
| 0.029985 | 0 | 0 | 0 |
| 0.109128 | 0.078227 | 0.133673 | 0.086862 |
| 0.016224 | 0 | 0 | 0.01937 |
| 2.172682 | 1.721404 | 1.792924 | 2.020644 |
| 6.189335 | 6.124561 | 5.59741 | 5.942533 |
| 0.256394 | 0.236305 | 0.157032 | 0.065597 |
| 5.499492 | 5.002344 | 4.490975 | 5.46256 |
| 48.45605 | 53.80591 | 54.85707 | 57.16629 |
| 0.049292 | 0.053002 | 0.060379 | 0.058852 |
| 1.71211 | 1.910429 | 2.215915 | 1.812755 |
| 0.874418 | 0.555587 | 0.632921 | 0.37964 |
| 5.367627 | 3.537419 | 4.0298 | 3.3077 |
| 0.13694 | 0.273457 | 0.287557 | 0.420427 |
| 13.53536 | 12.0878 | 11.07958 | 12.80499 |
| 3.309007 | 3.377116 | 3.022788 | 2.611534 |
| 0.04644 | 0 | 0.018962 | 0 |
| 15.96939 | 11.51727 | 10.49631 | 12.55607 |
| 3.429342 | 2.758936 | 3.142959 | 3.269674 |
| 18.47302 | 15.29732 | 14.37859 | 15.62961 |
| 0.038154 | 0 | 0 | 0 |
| 0 | 0 | 0 | 0 |
| 0 | 0 | 0 | 0 |
| 18.43337 | 14.58328 | 14.29863 | 14.4885 |
| 0.704379 | 0.969458 | 0.41415 | 0.437316 |
| 11.67855 | 11.77183 | 10.85136 | 10.38613 |
| 5.175274 | 4.694075 | 4.09684 | 4.518663 |
| 0.125669 | 0.486456 | 0.323264 | 0.345098 |

| | | | |
|---|---|---|---|
| 27.95124 | 26.9429 | 28.3904 | 28.94237 |
| 83.29876 | 78.43862 | 77.82794 | 81.14583 |
| 52.99932 | 53.20968 | 51.15256 | 54.619 |
| 12.69283 | 12.90268 | 13.64455 | 12.89995 |
| 123.7511 | 100.0024 | 97.41693 | 101.5155 |
| 291.3649 | 336.8021 | 316.2304 | 338.5927 |
| 0.072475 | 0 | 0.044388 | 0.129797 |
| 2.059327 | 2.298821 | 2.503264 | 2.289809 |
| 8.902044 | 7.423606 | 7.124163 | 7.510862 |
| 40.95054 | 32.11352 | 29.86222 | 30.95372 |
| 0 | 0.497082 | 0 | 0 |
| 20.07343 | 21.45861 | 25.16026 | 24.66334 |
| 43.05199 | 26.95481 | 38.2165 | 36.11142 |
| 136.6778 | 107.3351 | 106.5896 | 108.6428 |
| 1.856893 | 2.139254 | 1.299745 | 0.15836 |
| 28.66736 | 33.56865 | 34.05125 | 31.76401 |
| 18.24132 | 19.73777 | 19.24478 | 17.54968 |
| 5.82047 | 5.445249 | 5.478138 | 4.711416 |
| 34.77839 | 25.72523 | 29.5919 | 27.45016 |
| 5.906721 | 6.291965 | 6.222347 | 6.535148 |
| 3.834524 | 3.392423 | 2.616052 | 2.781705 |
| 0.2011 | 0.24026 | 0.191592 | 0.106713 |
| 0.675405 | 0.53603 | 0.51215 | 0.383999 |
| 0 | 0 | 0 | 0 |
| 0.520136 | 1.067715 | 1.013611 | 1.10089 |
| 0 | 0.102604 | 0 | 0 |
| 12.5477 | 14.0088 | 14.61305 | 13.40325 |
| 0 | 0 | 0.055681 | 0.027137 |
| 0 | 0.095344 | 0 | 0.052934 |
| 13.73061 | 13.5478 | 13.6048 | 13.88193 |
| 2.80461 | 1.909928 | 2.328462 | 2.232373 |
| 0 | 0 | 0 | 0 |
| 6.722265 | 7.416396 | 7.590965 | 8.10964 |
| 8.616664 | 8.686058 | 8.355854 | 6.483489 |
| 0.030004 | 0 | 0.073505 | 0 |
| 0 | 0 | 0 | 0 |
| 0 | 0 | 0 | 0.494357 |
| 11.47598 | 11.19522 | 11.07194 | 13.51756 |
| 4.976352 | 3.374814 | 4.401012 | 3.476151 |
| 0.133192 | 0.047738 | 0.081575 | 0.053008 |
| 12.47494 | 11.23709 | 11.23592 | 10.84604 |
| 0.091982 | 0.098904 | 0 | 0.036607 |
| 10.26075 | 10.35272 | 10.16831 | 10.53753 |
| 0.157539 | 0.423486 | 0 | 0.047023 |
| 31.90425 | 19.80144 | 19.42975 | 20.44487 |
| 45.3931 | 38.52086 | 41.15973 | 41.73439 |
| 0.184023 | 0.197872 | 0.197238 | 0.137321 |
| 14.5744 | 13.16996 | 12.6189 | 12.92328 |
| 0.350118 | 0.188233 | 0.214434 | 0.627034 |
| 41.31894 | 40.79282 | 38.4914 | 37.85134 |
| 0 | 0.136714 | 0.077872 | 0.037951 |
| 0.333498 | 0.039844 | 0.22695 | 0.132726 |
| 305.106 | 331.0489 | 336.4289 | 321.3856 |
| 0 | 0.08767 | 0 | 0 |

| | | | |
|---|---|---|---|
| 0.512204 | 0.509955 | 0.348562 | 0.498296 |
| 1.961197 | 1.127958 | 1.5643 | 14.26723 |
| 0 | 0 | 0 | 0 |
| 5.806332 | 5.023471 | 4.636271 | 4.161938 |
| 4.409415 | 3.317363 | 2.795509 | 3.044393 |
| 0 | 0.172003 | 0.326574 | 0 |
| 15.08044 | 13.1773 | 12.67589 | 12.35533 |
| 23.3177 | 19.09729 | 18.99712 | 20.31635 |
| 1.616594 | 1.192356 | 1.260131 | 1.339925 |
| 47.81696 | 42.45021 | 39.46853 | 39.16008 |
| 0.067588 | 0 | 0.206977 | 0 |
| 22.069 | 15.8941 | 15.5198 | 16.70928 |
| 0.09683 | 0.026029 | 0.029652 | 0.173415 |
| 0 | 0.020008 | 0.034189 | 0.022216 |
| 6.246583 | 5.76506 | 5.521248 | 5.82395 |
| 0 | 0 | 0 | 0 |
| 65.38572 | 70.02471 | 69.18013 | 73.80119 |
| 770.6486 | 652.9608 | 645.6847 | 648.121 |
| 0.520394 | 0 | 0.478082 | 0.155331 |
| 51.83421 | 60.32635 | 60.41819 | 61.23851 |
| 90.4566 | 90.00959 | 91.04307 | 89.53632 |
| 11.62641 | 11.30743 | 11.68121 | 8.890294 |
| 0.464547 | 0.416256 | 0.663874 | 0.369763 |
| 13.01183 | 18.49592 | 16.48131 | 18.51749 |
| 9.472614 | 8.281381 | 7.987985 | 7.181897 |
| 5.376231 | 5.163606 | 4.767611 | 4.63033 |
| 1736.559 | 1972.714 | 1972.006 | 1944.105 |
| 2.710082 | 1.94932 | 1.733467 | 2.08719 |
| 0 | 0.46972 | 0.535101 | 0 |
| 0.468794 | 0.792116 | 0.246102 | 0.719634 |
| 9.664849 | 7.211826 | 7.501252 | 8.654496 |
| 0 | 0.390836 | 0 | 0 |
| 0.145615 | 0 | 0 | 0 |
| 1.493335 | 1.743352 | 1.358851 | 2.343324 |
| 0.101744 | 0 | 0 | 0.121477 |
| 0.124976 | 0.268764 | 0 | 0 |
| 4.085202 | 3.499712 | 3.560269 | 2.718617 |
| 0 | 0.483014 | 0 | 0 |
| 0.494969 | 0.177406 | 0.44462 | 0.275785 |
| 0.692403 | 0.389437 | 0.339257 | 0.419706 |
| 3.728769 | 2.093395 | 5.2546 | 4.01858 |
| 20.88921 | 15.9129 | 18.66898 | 18.91269 |
| 9.236643 | 9.656647 | 9.747128 | 9.286798 |
| 20.19042 | 18.02369 | 18.4676 | 18.58854 |
| 3.0409 | 2.046916 | 2.392398 | 2.892734 |
| 0 | 0 | 0 | 0.043266 |
| 0.91807 | 0.930209 | 0.886677 | 0.906413 |
| 0.011851 | 0.025485 | 0 | 0 |
| 1.729192 | 0.634892 | 0.59411 | 1.233704 |
| 3.162781 | 3.079467 | 2.867495 | 2.973383 |
| 8.099754 | 7.62833 | 7.514658 | 6.62899 |
| 3.174403 | 1.861799 | 2.82793 | 1.821204 |
| 6.884248 | 6.64033 | 6.551864 | 6.587635 |
| 141.0408 | 137.9023 | 133.2559 | 131.6289 |

| | | | |
|---|---|---|---|
| 0.129832 | 0.069802 | 0 | 0 |
| 0.019998 | 0.021503 | 0 | 0.023877 |
| 0.183547 | 0.083099 | 0.130166 | 0.149942 |
| 0.780591 | 0 | 0.956165 | 0.465993 |
| 0 | 0.535186 | 0.60968 | 0.198087 |
| 0.365121 | 0.23094 | 0.315703 | 0.384649 |
| 4.113175 | 4.318656 | 4.445584 | 4.10206 |
| 2.831224 | 2.636446 | 3.003419 | 2.652512 |
| 146.2611 | 138.8009 | 137.69 | 142.6864 |
| 20.73512 | 20.91635 | 20.83562 | 21.45399 |
| 0.078316 | 0.168419 | 0.095931 | 0.093505 |
| 0.06926 | 0.037236 | 0 | 0.206731 |
| 0 | 0 | 0 | 0.250998 |
| 0 | 0 | 0 | 0 |
| 1.061615 | 1.366068 | 1.151172 | 0.95583 |
| 3.214585 | 3.240474 | 2.214914 | 2.638658 |
| 6.423028 | 3.551866 | 3.701577 | 3.228184 |
| 2.57384 | 0.691885 | 2.36457 | 1.536516 |
| 0.694561 | 0.248944 | 0.283595 | 0.552847 |
| 84.43122 | 108.1704 | 109.1899 | 108.6697 |
| 4.486897 | 3.790731 | 3.729503 | 3.717186 |
| 0 | 0 | 0 | 0 |
| 2.646272 | 2.029856 | 1.713651 | 1.750812 |
| 2.394355 | 2.550259 | 2.102837 | 2.157537 |
| 24.0429 | 21.44409 | 21.15954 | 21.22356 |
| 4.156956 | 3.512919 | 3.755505 | 3.653257 |
| 0.719968 | 0.105566 | 0.36078 | 0.234437 |
| 0.048637 | 0 | 0.029789 | 0 |
| 22.25134 | 24.68078 | 26.20934 | 26.27541 |
| 8.197548 | 6.673814 | 7.258482 | 6.347859 |
| 0.337702 | 0.272338 | 0.310245 | 0.302399 |
| 4.608434 | 4.067316 | 4.12769 | 4.245941 |
| 0.406975 | 0 | 0 | 0 |
| 43.74066 | 41.24578 | 39.39159 | 39.46052 |
| 0 | 0 | 0.870538 | 0 |
| 0.13282 | 0 | 0 | 0 |
| 0.068571 | 0.044239 | 0.050397 | 0.049122 |
| 0 | 0 | 0 | 0 |
| 0.75581 | 0.81269 | 0 | 1.804796 |
| 90.8002 | 99.65943 | 94.7274 | 103.835 |
| 4.645468 | 3.746304 | 3.003239 | 3.08136 |
| 0 | 0 | 0 | 0 |
| 9.486009 | 8.733246 | 9.189392 | 9.882318 |
| 1.840052 | 2.059285 | 2.023933 | 2.510774 |
| 0.110069 | 0.071012 | 0.026965 | 0.052567 |
| 0.208842 | 0.098245 | 0.079943 | 0.046753 |
| 20.47045 | 16.2052 | 15.58996 | 16.61256 |
| 3.523386 | 2.742185 | 3.329395 | 2.323723 |
| 0.681923 | 0.605722 | 0.508446 | 0.247794 |
| 1.157837 | 1.089351 | 0.842094 | 0.993598 |
| 24.4714 | 20.23674 | 18.90029 | 18.18765 |
| 6.380168 | 6.054154 | 7.022897 | 6.073009 |
| 1.161367 | 0 | 0 | 0.924408 |
| 30.13932 | 27.54482 | 26.44687 | 27.5895 |

| | | | |
|---|---|---|---|
| 8.687108 | 8.744797 | 8.244418 | 8.285106 |
| 58.64975 | 51.04088 | 52.29108 | 54.10364 |
| 2.270846 | 1.850584 | 1.815369 | 2.083398 |
| 52.86744 | 43.16394 | 46.88351 | 43.64813 |
| 1.124788 | 0.768789 | 0.929201 | 0.905703 |
| 34.82661 | 36.91864 | 35.99184 | 38.60549 |
| 0 | 0 | 0.110049 | 0 |
| 7.972919 | 6.826598 | 6.7821 | 4.407061 |
| 3.305532 | 3.58367 | 3.078599 | 2.902895 |
| 0.95736 | 0.81269 | 0.740648 | 0.812158 |
| 3.287843 | 2.418873 | 2.47294 | 3.340131 |
| 1.884642 | 1.34409 | 1.342724 | 1.423573 |
| 0 | 0 | 0.45926 | 0 |
| 0.805102 | 0.371011 | 0.479006 | 0.645411 |
| 6.005149 | 4.399956 | 6.119029 | 5.900838 |
| 8.63319 | 7.751698 | 7.231705 | 7.509303 |
| 99.57474 | 78.68141 | 76.39331 | 77.81907 |
| 0.5561 | 0.747438 | 0.894049 | 0.497966 |
| 0.736093 | 0.395745 | 0.338122 | 0.219714 |
| 1.423736 | 1.445833 | 1.138424 | 1.322118 |
| 14.92517 | 20.85055 | 22.02312 | 21.21134 |
| 0.270605 | 0.179058 | 0.178484 | 0.273382 |
| 11.88939 | 11.35903 | 11.03014 | 10.44869 |
| 15.43729 | 17.48624 | 16.59473 | 16.84241 |
| 39.30997 | 38.37751 | 37.97742 | 38.9808 |
| 4.979044 | 6.573594 | 5.442736 | 5.493222 |
| 10.28682 | 7.95977 | 8.327488 | 6.673895 |
| 11.26055 | 10.89719 | 10.18079 | 9.283117 |
| 0.009051 | 0.019464 | 0 | 0.021612 |
| 0.211392 | 0.284126 | 0.107891 | 0.126196 |
| 79.2815 | 82.12195 | 78.92305 | 84.33849 |
| 26.26679 | 20.05421 | 18.29117 | 19.90504 |
| 0.063404 | 0.0909 | 0.051776 | 0.050467 |
| 6.144006 | 5.264463 | 6.075631 | 4.737591 |
| 0 | 0 | 0 | 0 |
| 27.79257 | 25.554 | 23.17063 | 25.90297 |
| 0.32304 | 0.185254 | 0.342939 | 0.411405 |
| 1.862998 | 1.870637 | 1.711524 | 1.733664 |
| 28.20568 | 27.62629 | 22.66172 | 23.2267 |
| 0 | 0 | 0 | 0.245048 |
| 0.363481 | 0.130279 | 0.148412 | 0.144659 |
| 0.262059 | 0.14089 | 0.224701 | 0.219019 |
| 8.258972 | 8.880517 | 9.723738 | 8.552395 |
| 17.22554 | 14.21447 | 14.09639 | 13.82688 |
| 5.292765 | 4.536663 | 3.760739 | 4.340293 |
| 20.43621 | 22.86895 | 22.43819 | 24.16752 |
| 0.18673 | 0.250978 | 0.285912 | 0.724573 |
| 25.36072 | 22.56031 | 21.31144 | 22.76896 |
| 2.720917 | 2.100492 | 1.816013 | 1.915861 |
| 49.20962 | 50.58262 | 46.92632 | 50.22988 |
| 2.28431 | 2.498569 | 1.49554 | 2.304138 |
| 0.780591 | 0.394982 | 0.281225 | 0.219291 |
| 1.881253 | 1.558126 | 1.525882 | 1.396236 |
| 1.090964 | 0.488778 | 0.91874 | 0.678414 |

| | | | |
|---|---|---|---|
| 0.603958 | 0.233788 | 0.207145 | 0.403813 |
| 3.713617 | 3.039239 | 3.057457 | 2.76208 |
| 0.011454 | 0.024633 | 0 | 0.013676 |
| 0.156976 | 0.056263 | 0.064095 | 0 |
| 2.624003 | 1.37633 | 1.019138 | 1.146191 |
| 7.230783 | 7.544581 | 6.232819 | 5.883352 |
| 5.87507 | 5.354588 | 5.51733 | 4.843365 |
| 0.348835 | 0.23443 | 0.106824 | 0 |
| 0.054556 | 0.082126 | 0.066826 | 0.052109 |
| 0 | 0.144631 | 0 | 0.080298 |
| 0.094853 | 0.152987 | 0.058094 | 0.127405 |
| 38.94761 | 41.35942 | 39.60375 | 41.28335 |
| 3.34684 | 3.345876 | 2.899501 | 2.994625 |
| 0 | 0 | 0 | 0.133453 |
| 3.248095 | 2.212549 | 2.416365 | 2.720731 |
| 21.06574 | 22.24899 | 27.10177 | 26.342 |
| 8.877956 | 9.546084 | 9.221179 | 8.605523 |
| 0 | 0 | 0 | 0 |
| 25.57061 | 24.83599 | 25.45723 | 26.69802 |
| 2.037235 | 1.039583 | 1.099694 | 1.154337 |
| 4.746981 | 4.064027 | 4.505698 | 4.4992 |
| 0.157278 | 0.232533 | 0.168572 | 0.187782 |
| 0.033675 | 0 | 0 | 0 |
| 0 | 0 | 0 | 0.888298 |
| 195.7549 | 209.5059 | 208.4989 | 219.5628 |
| 27.97443 | 30.2397 | 19.32051 | 20.69735 |
| 1.237423 | 0.177406 | 0.24252 | 0.256086 |
| 0.700236 | 1.1294 | 1.715472 | 1.254068 |
| 81.19227 | 57.61631 | 55.97917 | 58.27611 |
| 7.61613 | 7.501396 | 6.679696 | 6.729015 |
| 2.402957 | 1.905779 | 1.899668 | 2.217884 |
| 0.030849 | 0.016586 | 0.018894 | 0.018416 |
| 7.938072 | 6.844353 | 6.113238 | 6.150859 |
| 0.095917 | 0.066301 | 0.025177 | 0.0409 |
| 1.990112 | 1.73443 | 2.129812 | 1.850849 |
| 3.50374 | 1.450808 | 0.986318 | 1.507022 |
| 0.149972 | 0.080629 | 0.275556 | 0.179059 |
| 0.449208 | 0 | 0 | 0 |
| 0 | 0 | 0 | 0 |
| 0 | 0.081076 | 0.046181 | 0.135038 |
| 6.653912 | 6.264695 | 6.798743 | 5.483592 |
| 0 | 0 | 0 | 0 |
| 1.994389 | 2.010451 | 0.458058 | 1.041774 |
| 2.387298 | 1.414447 | 1.929614 | 1.37668 |
| 0 | 0 | 0.174629 | 0 |
| 4.393564 | 3.929995 | 3.587856 | 3.527535 |
| 219.6298 | 240.2363 | 236.1062 | 244.9053 |
| 29.43739 | 24.21206 | 22.65542 | 23.82187 |
| 0.078856 | 0.042395 | 0.064395 | 0.078459 |
| 0.867406 | 0.801782 | 0.708338 | 0.744933 |
| 12.87536 | 13.8011 | 14.78666 | 13.86885 |
| 28.46649 | 24.95282 | 28.17338 | 27.09149 |
| 5.512923 | 4.73875 | 5.458108 | 4.951171 |
| 18.37513 | 16.76064 | 14.73831 | 16.43723 |

| | | | |
|---|---|---|---|
| 36.06702 | 31.69007 | 31.07631 | 34.73605 |
| 3.433226 | 3.273076 | 3.423034 | 3.086236 |
| 0.406975 | 0.218801 | 0.249257 | 0.36443 |
| 0.564274 | 0.108902 | 0.212675 | 0.138198 |
| 6.219815 | 6.250373 | 5.726818 | 5.463019 |
| 0 | 0.02298 | 0.026179 | 0 |
| 1.835855 | 1.574216 | 1.280953 | 1.664746 |
| 0.395499 | 0.514793 | 0.433461 | 0.484632 |
| 54.22939 | 42.54772 | 40.20043 | 41.61842 |
| 2.298333 | 2.500372 | 2.715921 | 2.873224 |
| 1.858619 | 1.141996 | 1.120265 | 1.23283 |
| 0.167662 | 0 | 0.102687 | 0 |
| 70.43265 | 64.05191 | 59.04445 | 60.78565 |
| 0.355344 | 0 | 0 | 0 |
| 0 | 0 | 0.178914 | 0 |
| 9.750522 | 9.615984 | 9.122606 | 8.8562 |
| 0 | 0 | 0 | 0 |
| 6.709089 | 6.711237 | 6.553191 | 6.114835 |
| 6.495562 | 6.109891 | 6.402451 | 6.214649 |
| 1.638958 | 1.484043 | 1.584947 | 1.338884 |
| 1.7026 | 1.322196 | 1.274507 | 1.422971 |
| 0.184931 | 0.246572 | 0.217466 | 0.264958 |
| 4.820727 | 4.248286 | 4.581966 | 4.425371 |
| 9.749047 | 7.982263 | 7.888192 | 8.342787 |
| 0.084705 | 0.143124 | 0.192691 | 0.11558 |
| 1.585297 | 1.182472 | 1.591983 | 1.278894 |
| 0.133706 | 0.143768 | 0.102362 | 0.199548 |
| 6.305372 | 4.488267 | 5.058893 | 4.574983 |
| 0.232841 | 0 | 0 | 0.695001 |
| 13.92375 | 12.46205 | 13.03601 | 13.44467 |
| 1.093544 | 0.671909 | 0.497532 | 0.895293 |
| 6.625256 | 5.910918 | 5.215677 | 5.918432 |
| 0.266757 | 0.889179 | 0.98027 | 0.191096 |
| 0 | 0 | 0 | 0 |
| 6.528579 | 5.951654 | 5.476217 | 5.253007 |
| 9.872353 | 7.702578 | 8.406031 | 7.690349 |
| 0.024418 | 0.026256 | 0 | 0.029154 |
| 0.913771 | 0.644791 | 0.769519 | 0.511404 |
| 0.049139 | 0.264187 | 0.180576 | 0.17601 |
| 0.720091 | 0.483927 | 1.212829 | 0.644814 |
| 0.32871 | 0.252463 | 0.345125 | 0.280331 |
| 1.843955 | 1.606258 | 1.65829 | 1.644223 |
| 0.141968 | 0.061061 | 0.20868 | 0.135602 |
| 3.564838 | 2.555411 | 3.223009 | 2.330793 |
| 0.0517 | 0 | 0 | 0 |
| 17.98523 | 17.83533 | 16.69491 | 17.27862 |
| 3.196339 | 2.148053 | 2.677356 | 2.413225 |
| 7.812295 | 6.013707 | 5.320374 | 5.343774 |
| 12.21253 | 13.90656 | 14.12499 | 13.77958 |
| 1.954274 | 1.801155 | 1.937869 | 2.111083 |
| 0.004816 | 0.010356 | 0.011797 | 0 |
| 0.043565 | 0.009369 | 0.032018 | 0.052014 |
| 7.942902 | 6.65015 | 6.587652 | 5.507295 |
| 0.49292 | 0.318009 | 0.241516 | 0.941633 |

| | | | |
|---|---|---|---|
| 1.175705 | 0 | 0 | 2.105596 |
| 0.300011 | 0.230421 | 0.104997 | 0.358198 |
| 39.0906 | 37.62345 | 38.38177 | 38.3087 |
| 3.041429 | 2.601043 | 2.971753 | 3.124614 |
| 18.10651 | 13.27182 | 14.42402 | 14.01692 |
| 38.12125 | 39.44983 | 41.85207 | 40.39791 |
| 0.191229 | 0.051405 | 0.05856 | 0.228318 |
| 0 | 0 | 0 | 0 |
| 0 | 0 | 0 | 0 |
| 10.00787 | 10.01656 | 9.830235 | 9.769519 |
| 42.34391 | 38.49828 | 41.32281 | 41.35349 |
| 21.51898 | 18.29888 | 17.93556 | 18.49678 |
| 17.41184 | 14.97776 | 15.93085 | 15.69769 |
| 0 | 0 | 0.151891 | 0 |
| 0 | 0 | 0 | 0 |
| 4.39793 | 3.880127 | 3.522355 | 3.332302 |
| 3.812613 | 3.490874 | 3.161028 | 3.120846 |
| 26.16848 | 21.22143 | 20.49679 | 21.72879 |
| 12.67956 | 10.81386 | 10.7934 | 11.35174 |
| 6.008764 | 5.53332 | 5.506306 | 5.891117 |
| 0.849853 | 0.678831 | 0.624603 | 0.753763 |
| 14.35353 | 12.77935 | 12.09541 | 12.9138 |
| 3.59382 | 3.248495 | 3.019494 | 3.022413 |
| 0 | 0 | 0 | 0.812158 |
| 40.11442 | 47.03295 | 45.15076 | 46.42921 |
| 0.102662 | 0.09659 | 0.070736 | 0.09193 |
| 11.50774 | 9.033198 | 9.513905 | 9.386864 |
| 0 | 0 | 0 | 0 |
| 45.07002 | 43.21959 | 44.78964 | 46.1147 |
| 0 | 0.074202 | 0.056354 | 0.054929 |
| 8.614707 | 8.017265 | 8.346345 | 8.792676 |
| 0.190388 | 0.184244 | 0.163248 | 0.113657 |
| 50.58741 | 56.23157 | 49.45431 | 50.42097 |
| 9.742948 | 10.69097 | 10.42172 | 10.25575 |
| 0 | 0.014599 | 0 | 0.016211 |
| 9.414061 | 8.343756 | 8.41884 | 7.737612 |
| 2.267431 | 1.828553 | 0 | 0.676799 |
| 0 | 0.279779 | 0 | 0 |
| 34.89179 | 38.76145 | 37.99953 | 38.79635 |
| 11.01705 | 8.232074 | 8.920456 | 8.694872 |
| 0 | 0 | 0.021209 | 0 |
| 2.483075 | 2.352824 | 2.470554 | 2.271772 |
| 0 | 0 | 0 | 0 |
| 0.553675 | 0 | 0 | 0 |
| 10.52799 | 10.24784 | 8.552075 | 15.26026 |
| 11.95441 | 13.73195 | 12.80358 | 12.91304 |
| 2.623375 | 2.473032 | 2.200983 | 1.802072 |
| 8.076817 | 4.941895 | 5.422792 | 5.366355 |
| 0.068267 | 0 | 0.041811 | 0.040753 |
| 0 | 0 | 0 | 0 |
| 0 | 0 | 0 | 0.072055 |
| 1.525641 | 1.295097 | 0.983576 | 1.629795 |
| 4.068808 | 4.138527 | 3.872689 | 3.315219 |
| 0.175381 | 0.09429 | 0.107414 | 0 |

| | | | |
|---|---|---|---|
| 6.033629 | 5.336205 | 4.895166 | 5.551011 |
| 1.985657 | 1.494564 | 1.362076 | 1.564709 |
| 0 | 0 | 0 | 0 |
| 3.567816 | 3.771571 | 4.554707 | 3.918286 |
| 34.17529 | 36.73078 | 36.59895 | 40.27408 |
| 0.020712 | 0 | 0 | 0 |
| 0 | 0 | 0 | 0 |
| 0.615194 | 0.132298 | 0.150713 | 0.293804 |
| 0.425489 | 0.207959 | 0.379048 | 0.554194 |
| 0.212227 | 0.207453 | 0.378127 | 0.322494 |
| 2.930218 | 3.675861 | 4.985133 | 2.721078 |
| 0 | 0.018686 | 0 | 0 |
| 5.766347 | 5.354809 | 4.494852 | 4.972297 |
| 2.794779 | 1.540117 | 1.925659 | 1.710121 |
| 0.345044 | 0 | 0 | 0 |
| 1.764227 | 2.081834 | 1.706672 | 1.587899 |
| 0 | 0 | 0 | 0 |
| 0.741636 | 0.504509 | 0.593272 | 0.487915 |
| 0.078445 | 0.084348 | 0 | 0 |
| 13.16216 | 12.16062 | 13.7178 | 13.89913 |
| 0.078618 | 0.084534 | 0 | 0.093865 |
| 5.352387 | 4.506423 | 4.731647 | 5.064148 |
| 0 | 0.108935 | 0.124098 | 0.24192 |
| 4.481874 | 4.154454 | 3.691523 | 4.2809 |
| 2.611249 | 2.11733 | 2.010038 | 2.14661 |
| 6.688542 | 5.822015 | 6.086198 | 6.312562 |
| 0.572131 | 0.45643 | 0.746031 | 0.528847 |
| 0.081955 | 0.088123 | 0.267704 | 0.130467 |
| 4.504829 | 2.56724 | 2.317592 | 2.312769 |
| 0 | 0 | 0 | 0 |
| 2.459232 | 1.729519 | 2.214501 | 2.095015 |
| 4.047645 | 3.264194 | 3.684114 | 3.523828 |
| 1.495983 | 0.863133 | 1.117357 | 0.784153 |
| 0.340115 | 0 | 0.416615 | 0 |
| 0.881779 | 0.790116 | 1.44015 | 1.052798 |
| 0 | 0 | 0 | 0 |
| 74.27981 | 79.57431 | 81.03544 | 76.98053 |
| 5.604627 | 4.490597 | 5.153688 | 5.394088 |
| 1.386875 | 1.366977 | 0.424704 | 8.003308 |
| 3.33589 | 3.025603 | 2.858431 | 2.917039 |
| 0.151334 | 0.185969 | 0.251577 | 0.193591 |
| 2.784961 | 1.31468 | 1.913694 | 1.567933 |
| 0.038524 | 0 | 0.047189 | 0.091992 |
| 0.093 | 0 | 0.113918 | 0 |
| 25.07141 | 17.96334 | 16.82771 | 19.18468 |
| 11.2865 | 8.975904 | 8.745596 | 10.07433 |
| 8.600531 | 9.292673 | 8.745073 | 7.950678 |
| 0 | 0.664928 | 0 | 0 |
| 43.182 | 40.07477 | 38.45997 | 40.4444 |
| 0.183492 | 0 | 0 | 0.328619 |
| 5.188928 | 5.579431 | 8.22547 | 2.18658 |
| 0 | 0 | 0 | 0 |
| 272.3703 | 196.4977 | 197.8707 | 203.8296 |
| 0.21932 | 0.168447 | 0.153515 | 0.102873 |

| | | | |
|---|---|---|---|
| 6.583158 | 6.838052 | 7.124391 | 6.448211 |
| 4.383699 | 4.876142 | 5.925187 | 5.955828 |
| 0.070857 | 0 | 0.086795 | 0.253799 |
| 4.383699 | 5.201218 | 5.184539 | 5.414389 |
| 0.333941 | 0.232341 | 0.312805 | 0.328348 |
| 0 | 0 | 0 | 0.024192 |
| 1.08561 | 0.324253 | 0.221632 | 0.32404 |
| 0 | 0 | 0 | 0 |
| 0 | 0 | 0 | 0 |
| 1.44291 | 5.430249 | 1.767456 | 0.86138 |
| 0 | 0.015334 | 0.017468 | 0 |
| 1.930782 | 2.012207 | 1.63735 | 1.312221 |
| 0.029484 | 0.063405 | 0 | 0.011734 |
| 5.671578 | 4.363747 | 4.75501 | 4.649811 |
| 10.01107 | 10.32535 | 9.923813 | 9.646499 |
| 2.267431 | 0 | 0 | 0 |
| 0.710687 | 0 | 0 | 0 |
| 4.295865 | 3.155663 | 3.126006 | 3.148519 |
| 2.238068 | 1.571591 | 1.734396 | 1.881403 |
| 42.87245 | 53.78206 | 46.63143 | 50.32719 |
| 15.47862 | 12.29959 | 12.78836 | 13.54888 |
| 0 | 0 | 0 | 0 |
| 0.012876 | 0 | 0 | 1.660335 |
| 1.566589 | 1.226423 | 1.245636 | 0.918805 |
| 507.1423 | 630.3515 | 628.9548 | 619.2058 |
| 0.020219 | 0.021741 | 0 | 0 |
| 0 | 0 | 0 | 0 |
| 0 | 0.067412 | 0 | 0 |
| 1.358176 | 1.116767 | 1.516869 | 0.667714 |
| 32.5703 | 26.83944 | 24.85566 | 28.01258 |
| 19.20372 | 19.90895 | 18.6258 | 19.99374 |
| 14.42346 | 10.16869 | 9.65764 | 9.561656 |
| 2.981877 | 2.195707 | 2.281551 | 2.070836 |
| 5.046676 | 4.863247 | 3.948453 | 4.281571 |
| 5.225807 | 4.606638 | 4.232878 | 4.710422 |
| 0.075401 | 0.040538 | 0 | 0.090025 |
| 31.86755 | 27.29311 | 24.58621 | 29.6422 |
| 0.090011 | 0.145178 | 0.091881 | 0.12538 |
| 10.02829 | 9.314738 | 9.676047 | 9.589129 |
| 3.525374 | 3.285258 | 2.475835 | 4.377478 |
| 3.174403 | 2.654788 | 3.024314 | 2.947834 |
| 0.188392 | 0.506424 | 0.115383 | 0.056233 |
| 34.91591 | 31.26593 | 28.32715 | 27.79185 |
| 69.02258 | 71.50907 | 71.6571 | 73.41028 |
| 1.769206 | 1.082372 | 1.307759 | 1.347527 |
| 11.8352 | 11.29546 | 11.26626 | 10.57058 |
| 31.51062 | 31.7487 | 31.16441 | 30.41115 |
| 36.76214 | 37.36081 | 35.81064 | 37.03144 |
| 6.58258 | 7.276413 | 6.706743 | 6.46369 |
| 0.15872 | 0.511995 | 0.583261 | 0.189504 |
| 5.871934 | 5.127602 | 5.993896 | 5.3112 |
| 24.67234 | 28.89476 | 6.737334 | 4.315429 |
| 14.74109 | 13.00744 | 12.09513 | 11.90101 |
| 0 | 0 | 0 | 0 |

| | | | |
|---|---|---|---|
| 17.96044 | 12.57531 | 13.08316 | 15.53075 |
| 10.39141 | 9.480491 | 9.064371 | 8.700875 |
| 0.034706 | 0.037317 | 0 | 0.082873 |
| 0.128 | 0.275266 | 0.235186 | 0 |
| 0 | 0 | 0 | 0 |
| 0.337292 | 0.341952 | 0.318722 | 0.368192 |
| 5.036812 | 3.405855 | 1.971764 | 3.719809 |
| 5.070909 | 3.755685 | 4.510411 | 3.567324 |
| 0.017321 | 0.03725 | 0.127303 | 0.041361 |
| 3.37053 | 2.513886 | 2.8638 | 2.419195 |
| 0 | 0.358038 | 0.407875 | 0.79512 |
| 135.2972 | 121.6542 | 121.2515 | 125.5256 |
| 3.913156 | 3.306928 | 3.35679 | 3.357629 |
| 0 | 0 | 0 | 0 |
| 5.44447 | 5.258862 | 5.858981 | 5.343562 |
| 0.032065 | 0.034478 | 0.039277 | 0 |
| 0.083804 | 0.065535 | 0.065325 | 0.081866 |
| 0 | 0.104276 | 0.11879 | 0.057893 |
| 1.08695 | 1.107238 | 0.887621 | 0.796871 |
| 0.400135 | 0 | 1.96054 | 0.47774 |
| 0 | 0 | 0 | 0 |
| 19.90058 | 21.7359 | 21.28841 | 20.99644 |
| 0.340601 | 0.366234 | 0.27814 | 0.338883 |
| 0.497729 | 0 | 0 | 0 |
| 21.69066 | 18.95493 | 17.94416 | 16.28516 |
| 0.153353 | 0.041223 | 0 | 0.045774 |
| 1.162785 | 0.93772 | 1.226501 | 1.226336 |
| 2.902958 | 3.405191 | 3.001737 | 2.633245 |
| 16.72581 | 14.69172 | 14.60873 | 15.06896 |
| 10.27013 | 14.05476 | 14.86743 | 10.03254 |
| 4.897415 | 5.177476 | 4.66306 | 4.839959 |
| 2.20674 | 1.91762 | 2.002493 | 1.914214 |
| 4.205652 | 4.305341 | 3.810777 | 4.092728 |
| 1.587202 | 0.731421 | 0.555486 | 0.541439 |
| 0.185793 | 0.171236 | 0.130047 | 0.158448 |
| 26.45336 | 20.70735 | 22.55274 | 20.97173 |
| 19.89132 | 15.95561 | 18.48855 | 16.55094 |
| 0.101962 | 0.065781 | 0.149874 | 0.097389 |
| 5.276853 | 5.234574 | 6.224348 | 4.942651 |
| 149.3574 | 139.8877 | 133.855 | 140.7969 |
| 1.915588 | 1.618375 | 1.676036 | 1.633652 |
| 24.01233 | 22.0331 | 21.7729 | 21.65277 |
| 0 | 0 | 0 | 0.022506 |
| 0.44501 | 0 | 0 | 0 |
| 121.3254 | 108.5605 | 107.4186 | 117.6932 |
| 0.064 | 0 | 0 | 0 |
| 0 | 0 | 0.039356 | 0 |
| 1.670738 | 1.690798 | 1.20384 | 2.053445 |
| 17.75193 | 16.76804 | 17.80382 | 19.56798 |
| 9.032322 | 8.260536 | 8.538454 | 8.234616 |
| 12.8951 | 10.72707 | 11.95337 | 11.02693 |
| 0 | 0 | 0 | 0 |
| 24.8604 | 23.84499 | 23.27811 | 23.12648 |
| 0.352711 | 0.379255 | 0 | 0 |

| | | | |
|---|---|---|---|
| 0 | 0.026813 | 0.015273 | 0 |
| 20.88251 | 21.67938 | 21.63493 | 22.61271 |
| 1.122264 | 0.517167 | 0.196384 | 0.765671 |
| 0 | 0 | 0.341088 | 0 |
| 4.906829 | 4.613632 | 4.09684 | 3.940695 |
| 2.844118 | 2.992391 | 2.247632 | 1.606582 |
| 0.323919 | 0.232197 | 0 | 0 |
| 13.9025 | 13.58978 | 11.99806 | 11.921 |
| 0.958934 | 0.853325 | 1.215126 | 0.868558 |
| 3.398313 | 3.341261 | 3.288028 | 2.194474 |
| 5.179237 | 5.923109 | 5.115678 | 5.84417 |
| 23.90293 | 20.61313 | 20.22735 | 19.51943 |
| 0.045814 | 0.016421 | 0.018706 | 0.018233 |
| 5.966348 | 5.843359 | 6.848361 | 6.974066 |
| 2.045757 | 2.065038 | 2.403617 | 2.018824 |
| 0.162143 | 0 | 0.264818 | 0.193591 |
| 1.168492 | 1.272134 | 1.288183 | 1.168412 |
| 2.080797 | 1.394539 | 1.256952 | 1.42936 |
| 0.0992 | 0 | 0 | 0.11844 |
| 22.11313 | 18.0139 | 17.89805 | 18.32209 |
| 0.323919 | 0 | 0 | 0.386742 |
| 8.477099 | 6.981748 | 7.246571 | 7.235593 |
| 12.42721 | 12.80485 | 11.70605 | 12.38297 |
| 0 | 0.167867 | 0 | 0 |
| 0.088015 | 0 | 0 | 0.105085 |
| 26.03942 | 18.31832 | 17.6119 | 16.10086 |
| 1.643044 | 1.351001 | 0.907645 | 1.192411 |
| 0.097077 | 0 | 0.059456 | 0 |
| 0 | 0.312192 | 0 | 0 |
| 17.51515 | 17.43167 | 17.39871 | 17.51328 |
| 0 | 0.088888 | 0 | 0 |
| 0.43184 | 0.545802 | 0.686735 | 0.434185 |
| 0 | 0 | 0.720075 | 0 |
| 2.911025 | 2.054128 | 2.387803 | 2.249838 |
| 0.967883 | 1.214177 | 1.406428 | 1.325543 |
| 0.110993 | 0.238692 | 0.407875 | 0.39756 |
| 0.091746 | 0.032883 | 0 | 0.10954 |
| 0 | 0.095611 | 0 | 0 |
| 12.83678 | 12.40413 | 11.36371 | 11.03914 |
| 20.76589 | 13.93764 | 14.68953 | 14.47597 |
| 0.554461 | 0.290824 | 0.331304 | 0.274487 |
| 0.16674 | 0.076838 | 0.11671 | 0.113759 |
| 18.53872 | 15.93608 | 14.66789 | 14.72556 |
| 0.04584 | 0.04929 | 0.028075 | 0.05473 |
| 8.158929 | 9.137287 | 8.443079 | 9.177086 |
| 7.759652 | 2.275533 | 3.888404 | 4.211192 |
| 0.222356 | 0.034156 | 0.11673 | 0.151704 |
| 2.431008 | 1.950998 | 2.179405 | 1.745705 |
| 13.70655 | 14.1429 | 14.43432 | 14.33016 |
| 15.06973 | 16.43461 | 15.99929 | 15.06389 |
| 0.301367 | 0.129619 | 0.295322 | 0.503744 |
| 59.03458 | 58.7583 | 55.89977 | 58.14948 |
| 0.020112 | 0.043252 | 0.049272 | 0.060033 |
| 25.84951 | 24.38864 | 24.76529 | 23.48343 |

| | | | |
|---|---|---|---|
| 1.217802 | 0.78567 | 0.895029 | 0.726996 |
| 3.847761 | 2.807475 | 3.156172 | 2.932794 |
| 0 | 0 | 0.05973 | 0 |
| 0 | 0 | 0 | 0.088096 |
| 108.1303 | 92.11285 | 101.511 | 97.40401 |
| 0 | 0.01601 | 0 | 0.026666 |
| 0.027383 | 0.019629 | 0.026088 | 0.007265 |
| 5.403932 | 4.068933 | 4.259 | 4.017921 |
| 44.79739 | 51.0002 | 48.99919 | 47.99058 |
| 0.142991 | 0.153752 | 0 | 0.512172 |
| 0 | 0 | 0 | 0.178777 |
| 3.055909 | 1.274119 | 1.871629 | 1.116917 |
| 1.152001 | 0.825798 | 0.470371 | 0.458477 |
| 3.555663 | 3.074643 | 3.25895 | 2.70154 |
| 0.053622 | 0 | 0 | 0 |
| 0.179768 | 0.072486 | 0.137626 | 0.134146 |
| 0 | 0 | 0 | 0 |
| 0.822635 | 0.884544 | 0.355647 | 0.808857 |
| 0 | 0.328202 | 0 | 0 |
| 0 | 0 | 0 | 0 |
| 0 | 0.025651 | 0 | 0 |
| 12.08039 | 11.90578 | 11.16432 | 12.10256 |
| 31.08203 | 24.1075 | 25.58726 | 23.08326 |
| 3.78063 | 3.530261 | 2.973581 | 3.088442 |
| 0 | 0 | 0 | 0 |
| 4.613319 | 4.499886 | 6.094972 | 5.626094 |
| 13.49725 | 12.47888 | 12.27658 | 12.63891 |
| 0.510902 | 0.631753 | 0.563234 | 0.518492 |
| 0.094947 | 0.051046 | 0 | 0.283405 |
| 56.8369 | 43.70688 | 44.12472 | 42.96106 |
| 13.51389 | 13.99453 | 13.10948 | 13.75255 |
| 0.793601 | 0.853325 | 1.944202 | 1.895036 |
| 2.24659 | 1.694101 | 1.858428 | 1.323739 |
| 0.020228 | 0.032625 | 0.024777 | 0.036226 |
| 0.370841 | 0.212667 | 0.211985 | 0.413248 |
| 0 | 0.406345 | 0 | 0 |
| 0 | 0 | 0 | 0 |
| 0 | 0.084348 | 0 | 0.093659 |
| 14.16891 | 11.21658 | 11.03936 | 11.18382 |
| 5.441011 | 3.931279 | 4.443219 | 3.918394 |
| 19.34362 | 19.16038 | 18.69465 | 17.57465 |
| 1.60946 | 0.625956 | 0.692111 | 1.083462 |
| 7.614278 | 6.918849 | 7.487805 | 7.426493 |
| 1.237182 | 1.089174 | 0.956631 | 0.913975 |
| 0 | 0 | 0 | 0.748041 |
| 0 | 0 | 0 | 0.056794 |
| 2.141651 | 1.362505 | 1.530294 | 1.42767 |
| 7.4602 | 6.025698 | 6.936383 | 6.270031 |
| 0.71783 | 0.467789 | 0.426321 | 0.545396 |
| 5.264085 | 3.050651 | 3.391537 | 3.673078 |
| 2.465875 | 2.863565 | 2.748665 | 3.061892 |
| 2.451437 | 2.010451 | 1.475965 | 1.885115 |
| 10.95529 | 10.41959 | 9.524363 | 9.713042 |
| 3.968004 | 3.55552 | 2.430252 | 3.158394 |

| | | | |
|---|---|---|---|
| 8.34794 | 5.749095 | 5.715212 | 5.570684 |
| 4.845881 | 4.858162 | 5.706434 | 4.947219 |
| 16.34029 | 15.02594 | 18.65709 | 15.53694 |
| 11.15139 | 10.1293 | 10.95534 | 9.839611 |
| 60.9588 | 65.15223 | 62.05282 | 62.7594 |
| 11.59653 | 9.965952 | 10.54604 | 9.912228 |
| 0 | 0.103643 | 0.118069 | 0.115083 |
| 0.525973 | 0.199608 | 0.151595 | 0.258582 |
| 34.79146 | 29.76397 | 31.80714 | 28.95615 |
| 1.504993 | 1.1902 | 1.213144 | 0.741939 |
| 0.069009 | 0.074202 | 0 | 0 |
| 0 | 0.016792 | 0 | 0 |
| 0 | 0 | 0 | 0 |
| 6.715083 | 10.17426 | 7.851585 | 10.9329 |
| 11.41385 | 10.8046 | 11.62233 | 10.07435 |
| 0 | 0.064892 | 0 | 0 |
| 6.059939 | 4.996719 | 5.057617 | 5.098416 |
| 10.04038 | 11.05666 | 11.68006 | 11.21585 |
| 15.95412 | 14.63874 | 15.19522 | 13.67474 |
| 11.63091 | 8.291415 | 9.28809 | 9.513542 |
| 61.77217 | 61.87428 | 54.61031 | 60.03387 |
| 0.315338 | 0.226046 | 0.193133 | 0.188249 |
| 0.806248 | 0.560951 | 0.813312 | 12.4291 |
| 2.257096 | 2.496967 | 2.179917 | 2.643031 |
| 0 | 1.02399 | 0 | 0 |
| 0.89002 | 0.4785 | 0.545103 | 0 |
| 12.99388 | 10.36196 | 10.53212 | 10.87135 |
| 44.30938 | 44.14533 | 42.25399 | 42.79624 |
| 0 | 0 | 0 | 0.079847 |
| 0.429489 | 0.307874 | 0.420874 | 0.444416 |
| 2.053544 | 1.785824 | 1.493227 | 1.445698 |
| 21.56966 | 22.31772 | 14.45689 | 14.0913 |
| 6.040314 | 5.735254 | 4.197053 | 4.048742 |
| 3.498859 | 4.045042 | 3.222434 | 3.455038 |
| 0.706586 | 0.358776 | 0.504883 | 1.968463 |
| 6.61334 | 4.544649 | 4.11742 | 4.547612 |
| 0 | 0 | 0 | 0 |
| 32.91109 | 27.35659 | 24.58844 | 29.81895 |
| 6.265269 | 5.517739 | 5.993405 | 6.696745 |
| 0.277847 | 0.336102 | 0.297799 | 1.078139 |
| 0 | 0 | 0.060379 | 0.058852 |
| 3.12014 | 1.75041 | 2.99108 | 1.376736 |
| 0.380375 | 0.818002 | 0.593003 | 0.825724 |
| 1.303359 | 0.591722 | 0.319303 | 0.829943 |
| 0 | 0 | 0 | 0 |
| 5.894344 | 6.521642 | 6.006308 | 5.772823 |
| 4.417984 | 5.278298 | 7.215595 | 7.033124 |
| 0.616788 | 0.663206 | 0.302208 | 0.58913 |
| 6.396541 | 5.774378 | 5.379446 | 5.756351 |
| 0 | 0 | 0 | 0 |
| 2.654851 | 2.977428 | 2.657542 | 3.067505 |
| 0.47616 | 0.435196 | 0.349956 | 0.113702 |
| 0 | 0 | 0 | 0.586094 |
| 3.555588 | 2.89321 | 2.57494 | 2.86837 |

| | | | |
|---|---|---|---|
| 18.68734 | 17.72813 | 18.24432 | 18.62832 |
| 17.13135 | 21.22222 | 18.94999 | 19.05406 |
| 25.12442 | 18.36064 | 17.32275 | 18.68093 |
| 0 | 0 | 0 | 0 |
| 20.01427 | 17.69177 | 18.52968 | 18.00403 |
| 11.68109 | 9.204402 | 9.830235 | 9.04933 |
| 3.236343 | 4.16256 | 2.958981 | 3.050546 |
| 0.028615 | 0.246151 | 0.385569 | 0.239157 |
| 23.90025 | 24.28221 | 22.69132 | 24.72881 |
| 3.363989 | 3.095089 | 2.421402 | 2.318762 |
| 8.210986 | 6.993 | 6.109901 | 6.871606 |
| 10.42496 | 10.20912 | 10.78272 | 10.13977 |
| 1.082183 | 1.163625 | 1.325592 | 0.43069 |
| 0.01535 | 0.016505 | 0 | 0 |
| 0 | 0.275266 | 0.15679 | 0 |
| 32.63799 | 31.91126 | 32.78904 | 33.65149 |
| 0 | 0.825798 | 0.470371 | 0 |
| 0.023059 | 0 | 0 | 0.055062 |
| 5.916787 | 6.362066 | 7.204476 | 6.181294 |
| 0 | 0 | 0 | 0 |
| 0.02746 | 0.029527 | 0 | 0.032786 |
| 0.070857 | 0.038095 | 0.086795 | 0.0423 |
| 3.471672 | 4.406466 | 4.720704 | 4.233726 |
| 2.091661 | 2.130004 | 1.838703 | 1.953797 |
| 0.022696 | 0.024404 | 0 | 0.108391 |
| 0 | 0.221643 | 0.252494 | 0 |
| 3.216093 | 2.289408 | 2.13388 | 2.382128 |
| 18.0009 | 18.99951 | 18.34098 | 15.64605 |
| 5.294956 | 5.278867 | 5.320974 | 5.58537 |
| 20.15913 | 18.37306 | 17.35822 | 19.52223 |
| 12.88524 | 11.63157 | 11.91381 | 11.70396 |
| 50.14701 | 47.56414 | 47.05879 | 46.45507 |
| 25.06589 | 21.3946 | 23.64419 | 22.99165 |
| 0 | 0 | 0 | 0 |
| 31.60181 | 30.47807 | 27.8216 | 28.79912 |
| 3.21769 | 2.466884 | 2.739557 | 2.704734 |
| 0 | 0 | 0.41366 | 0 |
| 5.254742 | 4.346306 | 4.951278 | 3.635638 |
| 0 | 0.074229 | 0.105701 | 0.082423 |
| 0 | 0.023524 | 0 | 0 |
| 0 | 0 | 0 | 0 |
| 1.886977 | 1.813135 | 0.934397 | 0.862833 |
| 0.084576 | 0 | 0 | 0 |
| 0.045048 | 0.048438 | 0.165542 | 0 |
| 18.56951 | 13.75885 | 12.60358 | 12.77804 |
| 3.509188 | 2.52094 | 2.908892 | 2.85339 |
| 0.617788 | 0.182677 | 0.13243 | 0.202842 |
| 417.1399 | 370.7487 | 369.6363 | 380.6638 |
| 0 | 0 | 0 | 0 |
| 0 | 0.085261 | 0.032376 | 0.047336 |
| 0.009822 | 0.010561 | 0 | 0 |
| 0.053682 | 0.057722 | 0 | 0 |
| 10.37713 | 8.619391 | 8.018101 | 8.574927 |
| 12.39937 | 9.532637 | 9.208061 | 10.73122 |

| | | | |
|---|---|---|---|
| 0.501327 | 0.398901 | 0.196508 | 0.335193 |
| 2.29339 | 2.051618 | 2.38324 | 2.076086 |
| 3.868448 | 4.648938 | 3.414548 | 3.260277 |
| 0 | 0.853325 | 0 | 0.473759 |
| 5.506526 | 4.552346 | 4.349017 | 4.255034 |
| 0 | 0 | 0 | 0 |
| 52.73985 | 39.0408 | 38.66762 | 36.49439 |
| 37.36275 | 35.5679 | 36.78424 | 35.32718 |
| 0.766529 | 0.353235 | 0.491826 | 0.56655 |
| 0.288582 | 0.6206 | 0.353491 | 0.344552 |
| 1.047681 | 0.02299 | 0 | 0 |
| 0 | 0 | 0.035608 | 0 |
| 68.99342 | 83.68714 | 83.35094 | 82.84477 |
| 1.339845 | 0.997393 | 1.641209 | 1.968869 |
| 38.39312 | 34.97164 | 33.72498 | 35.01394 |
| 2.805258 | 1.813767 | 2.088688 | 2.386126 |
| 1.530306 | 0.949311 | 0.720965 | 0.63246 |
| 14.74693 | 16.0516 | 17.42567 | 16.66045 |
| 12.99772 | 10.90801 | 11.80501 | 11.65788 |
| 0.350118 | 0 | 0.428868 | 0.418023 |
| 0.626527 | 0 | 0 | 0 |
| 12.1079 | 11.13032 | 10.93774 | 11.48507 |
| 1.42021 | 1.359278 | 1.204373 | 1.583855 |
| 41.96795 | 34.68936 | 34.06553 | 33.09242 |
| 1.133715 | 5.485659 | 3.471789 | 4.060792 |
| 7.434139 | 6.685021 | 6.167382 | 6.028025 |
| 0.040674 | 0.029157 | 0.016608 | 0.048563 |
| 72.92866 | 69.53487 | 68.51686 | 68.82715 |
| 0 | 0 | 0.161568 | 0 |
| 16.54456 | 14.54488 | 15.23698 | 15.66861 |
| 0.045873 | 0 | 0 | 0 |
| 0 | 0.074963 | 0 | 0.249712 |
| 13.79945 | 11.87037 | 12.1626 | 12.08228 |
| 25.25666 | 23.70347 | 22.52805 | 21.20636 |
| 17.33594 | 15.21837 | 13.80834 | 14.41445 |
| 15.20372 | 13.8553 | 13.12336 | 14.03823 |
| 0.783445 | 0.655204 | 0.213258 | 0.41573 |
| 131.3191 | 127.394 | 123.3114 | 121.6479 |
| 0.238876 | 0.171236 | 0.292606 | 0.237672 |
| 6.178195 | 6.170622 | 6.966196 | 7.530763 |
| 0 | 0 | 0 | 0 |
| 0.873986 | 0.730924 | 0.793012 | 0.772958 |
| 1.495009 | 1.527143 | 1.465019 | 1.249474 |
| 0.384 | 0.412899 | 0 | 0 |
| 3.766116 | 3.765365 | 3.10245 | 3.392132 |
| 6.31331 | 5.745629 | 5.427305 | 5.108424 |
| 1.716566 | 1.47125 | 1.615089 | 1.306922 |
| 0.069445 | 0.049781 | 0.028355 | 0.082914 |
| 0 | 0 | 0 | 0 |
| 17.10375 | 17.36867 | 17.35829 | 15.9984 |
| 0.125305 | 0 | 0 | 0 |
| 70.2156 | 65.31886 | 64.55239 | 66.60889 |
| 0 | 0 | 0.911345 | 0 |
| 0 | 0 | 0 | 0.05953 |

| | | | |
|---|---|---|---|
| 3.702827 | 2.550643 | 2.055232 | 3.177582 |
| 2.592151 | 1.061047 | 1.028328 | 0.738554 |
| 4.482375 | 6.320924 | 4.950514 | 5.176256 |
| 127.9035 | 128.2124 | 123.4195 | 130.1216 |
| 0.031702 | 0 | 0.019416 | 0.018925 |
| 7.452946 | 9.201067 | 7.641559 | 9.228003 |
| 5.565512 | 5.984356 | 7.574813 | 3.691629 |
| 0.09087 | 0 | 0 | 0 |
| 1.149505 | 0.665546 | 1.245589 | 1.319663 |
| 14.66061 | 12.28854 | 12.02892 | 11.37391 |
| 3.879634 | 3.698822 | 3.199854 | 4.261516 |
| 0 | 0.039233 | 0 | 0.087128 |
| 6.49123 | 5.963702 | 6.995101 | 5.788118 |
| 0 | 0.56574 | 0.322243 | 0 |
| 8.795115 | 5.715775 | 5.761573 | 7.000608 |
| 4.27269 | 3.497108 | 4.11407 | 3.933892 |
| 0 | 0.052244 | 0 | 0 |
| 0.949157 | 1.970301 | 2.664396 | 0.692538 |
| 40.65446 | 36.51204 | 36.63836 | 37.82576 |
| 0.94887 | 0.649269 | 0.739642 | 0.823929 |
| 0.077049 | 0.165694 | 0.377515 | 0.45996 |
| 1.87322 | 1.949219 | 1.36933 | 1.599237 |
| 0 | 0 | 0 | 0 |
| 0.721455 | 1.939374 | 1.767456 | 1.72276 |
| 3.408264 | 3.278996 | 3.173875 | 2.593875 |
| 0.017429 | 0 | 0 | 0 |
| 6.947078 | 6.756589 | 6.320161 | 7.480406 |
| 23.14174 | 20.98949 | 20.65896 | 21.19315 |
| 0.227013 | 0.305122 | 0.208556 | 0.338803 |
| 6.493097 | 4.945405 | 4.750039 | 17.33527 |
| 16.67193 | 17.38338 | 16.81193 | 15.83386 |
| 0.098584 | 0 | 0 | 0 |
| 0.229262 | 0.151702 | 0.172818 | 0.21056 |
| 0.49292 | 0.318009 | 0.483032 | 0.235408 |
| 0.925555 | 1.1028 | 1.24098 | 1.000531 |
| 18.49411 | 13.85715 | 12.71078 | 14.08788 |
| 0.13282 | 0 | 0 | 0 |
| 10.46614 | 8.897856 | 8.123473 | 7.894687 |
| 10.28676 | 7.126877 | 8.898296 | 8.483347 |
| 0.068218 | 0.055014 | 0 | 0.040724 |
| 8.359602 | 7.60584 | 9.152128 | 8.884125 |
| 0.021324 | 0.068786 | 0.18284 | 0.076379 |
| 0.869446 | 0.676295 | 0.883728 | 1.148507 |
| 0.296673 | 0.07975 | 0.181701 | 0 |
| 0 | 0 | 0 | 0 |
| 12.42158 | 10.01729 | 9.298357 | 8.101967 |
| 4.56496 | 1.459966 | 1.347751 | 1.145966 |
| 0 | 0 | 0 | 0 |
| 5.896495 | 6.048437 | 5.741944 | 5.596739 |
| 0 | 0.007309 | 0 | 0 |
| 0.209455 | 0.150145 | 0.171044 | 0.083359 |
| 0 | 0 | 0.92581 | 0 |
| 0.548186 | 0.556694 | 0.46631 | 0.581783 |
| 12.77504 | 10.92008 | 10.7148 | 11.44692 |

| | | | |
|---|---|---|---|
| 2.380802 | 1.493318 | 2.430252 | 1.658157 |
| 0.025892 | 0 | 0 | 0 |
| 41.10842 | 36.86113 | 36.90307 | 40.54846 |
| 44.18993 | 46.53815 | 43.08078 | 44.12012 |
| 14.26211 | 14.1592 | 13.72824 | 14.63786 |
| 33.99257 | 42.23958 | 40.99026 | 44.21751 |
| 6.559455 | 5.805906 | 5.903646 | 6.016999 |
| 6.697486 | 6.692577 | 7.827056 | 7.629122 |
| 3.78213 | 3.274264 | 3.421162 | 2.940973 |
| 10.58931 | 10.20391 | 9.223247 | 9.446642 |
| 0.848055 | 0.950272 | 1.104412 | 0.927267 |
| 20.92718 | 19.81375 | 20.28272 | 20.0177 |
| 0 | 0 | 0.202521 | 0.394799 |
| 7.363306 | 7.149694 | 8.855503 | 6.180624 |
| 11.20233 | 12.62779 | 13.23947 | 12.72829 |
| 1.813945 | 0.731421 | 0.277743 | 2.436475 |
| 4.299777 | 3.063217 | 2.969405 | 2.68305 |
| 0.660794 | 0.292568 | 0.523744 | 0.626522 |
| 0.044626 | 0.047985 | 0 | 0 |
| 4.143709 | 4.01472 | 3.945797 | 4.020833 |
| 2.838752 | 1.900543 | 2.689953 | 2.366131 |
| 2.605064 | 1.461451 | 1.109916 | 1.595725 |
| 5.92132 | 5.503085 | 5.476326 | 4.78718 |
| 0 | 0 | 0 | 0.117704 |
| 8.097778 | 8.573007 | 8.271634 | 8.824004 |
| 0 | 0 | 0 | 0 |
| 0.181581 | 0.015019 | 0.136875 | 0.100061 |
| 18.53319 | 14.96129 | 15.05301 | 14.57022 |
| 50.1955 | 53.73396 | 54.56719 | 53.01028 |
| 3.92494 | 2.47398 | 2.456627 | 2.247601 |
| 0 | 0 | 0 | 0 |
| 0.114807 | 0.061723 | 0.210944 | 0.20561 |
| 7.800735 | 5.470652 | 5.468068 | 5.114444 |
| 7.498826 | 5.7917 | 6.009203 | 5.928961 |
| 0.74213 | 0.720757 | 0.762432 | 0.800317 |
| 32.80358 | 26.52256 | 28.50112 | 28.5394 |
| 1.484838 | 2.104586 | 2.190844 | 1.651945 |
| 0 | 0.008189 | 0.009329 | 0 |
| 12.53054 | 15.11874 | 13.70097 | 17.22856 |
| 0 | 0 | 0 | 0 |
| 6.219846 | 4.255957 | 3.536017 | 3.87298 |
| 19.15136 | 20.73368 | 17.19253 | 18.48052 |
| 118.9537 | 140.063 | 133.536 | 138.2929 |
| 0.010979 | 0 | 0 | 0.013108 |
| 10.81749 | 10.45045 | 9.213996 | 8.154168 |
| 0 | 0 | 0 | 0 |
| 0 | 0 | 0.029472 | 0.028727 |
| 0 | 0 | 0 | 0 |
| 8.134066 | 7.688735 | 7.579376 | 6.604903 |
| 3.46803 | 3.412056 | 3.292258 | 3.64377 |
| 0.154097 | 0 | 0.062919 | 0.061328 |
| 4.423985 | 3.56769 | 3.315601 | 3.04063 |
| 0 | 0 | 0 | 0 |
| 0 | 0 | 0 | 0 |

| | | | |
|---|---|---|---|
| 0.191845 | 0.233788 | 0.250663 | 0.152702 |
| 0 | 0 | 0 | 0 |
| 13.5114 | 10.98802 | 9.055187 | 10.71752 |
| 0.267406 | 0.460048 | 0.174694 | 0.170277 |
| 4.20739 | 4.336824 | 4.371789 | 3.672282 |
| 23.37429 | 21.78945 | 21.56235 | 21.18617 |
| 9.95792 | 9.728989 | 9.349506 | 8.952135 |
| 3.590099 | 3.013726 | 4.088996 | 2.970394 |
| 16.27554 | 15.90944 | 16.47629 | 18.95036 |
| 0 | 0 | 0 | 0 |
| 0 | 0 | 0 | 0 |
| 1.72712 | 1.316424 | 0.990847 | 1.148507 |
| 2.854465 | 3.29948 | 3.365385 | 3.68499 |
| 54.32986 | 53.43713 | 53.95616 | 53.68612 |
| 67.96658 | 83.14616 | 81.99787 | 80.89743 |
| 0.138285 | 0.165213 | 0.376419 | 0.18345 |
| 0.344674 | 0.356886 | 0.31274 | 0.335315 |
| 0.949157 | 0.479367 | 0.669402 | 0.635304 |
| 4.019536 | 3.372137 | 3.868565 | 3.348835 |
| 0 | 0.028877 | 0.065794 | 0.032065 |
| 2.898368 | 0.890426 | 2.535916 | 0.988715 |
| 5.899333 | 5.550387 | 5.161598 | 4.81096 |
| 4.479344 | 4.024702 | 4.171516 | 3.333408 |
| 488.2563 | 422.1456 | 421.3193 | 430.973 |
| 0.412747 | 0.040346 | 0.045962 | 0.1792 |
| 1.02243 | 1.256429 | 1.610229 | 1.177131 |
| 17.76718 | 10.6984 | 13.05807 | 8.485237 |
| 279.5112 | 285.387 | 268.1773 | 280.0201 |
| 0.175059 | 0.188233 | 0.321651 | 0.627034 |
| 1.415339 | 1.630557 | 1.981352 | 0.965624 |
| 0.168851 | 0 | 0 | 0 |
| 0 | 0 | 0 | 0 |
| 3.222657 | 2.557195 | 2.723149 | 2.942347 |
| 0.726963 | 0 | 0 | 0 |
| 43.18565 | 37.49773 | 37.60635 | 36.07759 |
| 9.405639 | 10.11348 | 7.560786 | 10.87891 |
| 8.201278 | 8.010319 | 9.58562 | 7.680439 |
| 0.335324 | 0.22535 | 0.30806 | 0.266907 |
| 49.92659 | 49.72226 | 47.27279 | 46.18918 |
| 8.496973 | 8.515507 | 8.16484 | 8.687224 |
| 0.300891 | 0 | 0 | 0 |
| 0 | 0 | 0 | 0 |
| 2.100938 | 1.719574 | 1.670849 | 1.722193 |
| 0 | 0 | 0.206099 | 0 |
| 32.38591 | 30.33247 | 29.5919 | 29.59004 |
| 488.4365 | 576.1276 | 565.4894 | 544.5268 |
| 1.843472 | 0.765852 | 0.795472 | 0.875402 |
| 13.95844 | 13.12888 | 12.74321 | 12.94284 |
| 3.371952 | 2.453767 | 2.670149 | 2.358629 |
| 9.795301 | 7.119167 | 8.443391 | 7.796721 |
| 0 | 0.339069 | 0 | 0.188249 |
| 0.551537 | 0.461257 | 0.600526 | 0.878009 |
| 5.952006 | 3.733296 | 3.037816 | 3.257094 |
| 0 | 0 | 0 | 0 |

| | | | |
|---|---|---|---|
| 1.264942 | 1.262985 | 1.328108 | 1.294522 |
| 1.618607 | 1.558584 | 1.597974 | 1.75947 |
| 5.125338 | 5.439945 | 3.402353 | 3.395273 |
| 0.159118 | 0.12832 | 0.097454 | 0.142484 |
| 3.943087 | 3.968563 | 4.532402 | 3.737267 |
| 13.74652 | 11.92688 | 11.60709 | 11.4552 |
| 1.386875 | 1.025232 | 0.990977 | 1.586863 |
| 0 | 0 | 0 | 0 |
| 5.484317 | 4.974939 | 4.871094 | 4.640568 |
| 10.54177 | 8.322629 | 7.925132 | 8.901143 |
| 13.60458 | 12.06203 | 10.37882 | 10.68629 |
| 0.260197 | 0.419668 | 0.07968 | 0.077665 |
| 23.29383 | 15.21767 | 14.27659 | 15.45523 |
| 5.540011 | 5.152624 | 4.58133 | 5.833028 |
| 31.80574 | 34.87617 | 33.01817 | 32.7579 |
| 2.321204 | 2.023695 | 2.228532 | 2.022371 |
| 0.139228 | 0.149706 | 0 | 0 |
| 0.264251 | 0.174854 | 0.087147 | 0.157751 |
| 0.157343 | 0.211481 | 0.180688 | 0.09393 |
| 0 | 0 | 0 | 0 |
| 15.37141 | 13.40713 | 13.09361 | 14.17887 |
| 0 | 0 | 0 | 0 |
| 0 | 0 | 0 | 0.1344 |
| 6.0348 | 4.589753 | 5.048314 | 4.041963 |
| 9.715255 | 9.717577 | 10.3783 | 11.53207 |
| 6.442862 | 6.523194 | 5.933416 | 6.260638 |
| 21.37236 | 19.96072 | 20.58883 | 19.02023 |
| 2.026215 | 1.585967 | 1.514726 | 1.405268 |
| 2.815206 | 2.62153 | 2.540937 | 3.007398 |
| 13.02087 | 9.652885 | 8.950875 | 9.129372 |
| 1.171073 | 0.929122 | 1.00274 | 1.14028 |
| 27.95533 | 25.94413 | 26.8996 | 24.41518 |
| 12.13706 | 10.22386 | 11.32722 | 10.28395 |
| 0 | 0 | 0 | 0 |
| 5.265774 | 5.593221 | 5.41109 | 4.834731 |
| 0 | 0 | 0 | 0 |
| 0.635284 | 0.487924 | 0.555839 | 0.361189 |
| 7.416495 | 7.358241 | 6.8464 | 6.673265 |
| 11.0033 | 11.90118 | 10.6951 | 11.1222 |
| 16.45234 | 18.4393 | 14.92806 | 18.18819 |
| 2.269945 | 2.440774 | 2.618853 | 1.827807 |
| 0 | 0.075851 | 0 | 0 |
| 1.197764 | 1.45681 | 1.779847 | 1.898944 |
| 19.56903 | 20.60467 | 21.80114 | 18.64993 |
| 4.958056 | 4.350448 | 4.182517 | 3.71375 |
| 0.070621 | 0.056952 | 0 | 0.021079 |
| 0 | 0 | 0.093471 | 0 |
| 5.544937 | 4.490076 | 4.587981 | 4.58872 |
| 0 | 0.984606 | 0 | 0.546645 |
| 0.090182 | 0 | 0 | 0 |
| 9.44345 | 5.797463 | 5.666518 | 5.732723 |
| 3.282271 | 2.485412 | 3.28438 | 3.69808 |
| 8.355345 | 7.928036 | 7.16921 | 7.293132 |
| 3.759161 | 2.245592 | 2.046528 | 2.327238 |

| | | | |
|---|---|---|---|
| 0.019906 | 0.035674 | 0.048768 | 0.047534 |
| 0.090011 | 0 | 0 | 0 |
| 29.16483 | 26.45307 | 30.13513 | 32.4525 |
| 2.147799 | 2.001511 | 1.92932 | 2.206904 |
| 1.861641 | 1.945355 | 1.140185 | 0.876559 |
| 0 | 0 | 0 | 0 |
| 18.39952 | 16.25743 | 15.8759 | 16.31552 |
| 15.07108 | 13.68927 | 14.25406 | 14.55448 |
| 0.805724 | 0.577574 | 0.452353 | 0.427552 |
| 5.772728 | 2.81443 | 3.777139 | 2.91105 |
| 0 | 0 | 0 | 0 |
| 0 | 0 | 0 | 0 |
| 13.90155 | 14.7149 | 14.24334 | 13.31437 |
| 30.67459 | 34.66588 | 35.56116 | 36.99759 |
| 10.5736 | 7.596215 | 7.828046 | 8.98963 |
| 76.42558 | 78.35139 | 79.24574 | 75.25123 |
| 9.700385 | 7.763271 | 7.867236 | 9.043289 |
| 8.438827 | 7.344816 | 7.652463 | 6.592415 |
| 5.601888 | 2.409388 | 3.430945 | 0.668836 |
| 14.59535 | 9.63886 | 9.230733 | 9.368071 |
| 0 | 0 | 0 | 0 |
| 699.7615 | 895.6634 | 897.5673 | 892.5378 |
| 11.67509 | 11.69219 | 10.09489 | 9.361297 |
| 0 | 0 | 0 | 0 |
| 0 | 0.050718 | 0 | 0 |
| 1.143929 | 0.922513 | 14.18742 | 0.512172 |
| 0.013103 | 0.070445 | 0.0321 | 0.046933 |
| 5.581368 | 4.125966 | 4.700269 | 4.456459 |
| 0 | 0 | 0 | 0 |
| 4.196412 | 3.556931 | 3.774175 | 3.926991 |
| 7.285784 | 5.782935 | 5.532609 | 6.156786 |
| 0 | 0.027147 | 0 | 0 |
| 0 | 0 | 0 | 0 |
| 1.6928 | 1.014109 | 1.125643 | 1.212669 |
| 4.266223 | 3.343846 | 12.05954 | 3.321133 |
| 24.64752 | 20.0754 | 22.70521 | 20.76789 |
| 8.683652 | 4.513697 | 4.184151 | 4.569707 |
| 0.470863 | 0.80164 | 0.576772 | 0.702733 |
| 4.187248 | 4.283225 | 3.51772 | 3.561488 |
| 4.658091 | 3.339097 | 3.803873 | 4.325626 |
| 0 | 0.099224 | 0 | 0.165265 |
| 0.051035 | 0 | 0 | 0 |
| 133.7847 | 107.3188 | 103.3511 | 107.5858 |
| 8.628411 | 13.28667 | 10.9606 | 12.40041 |
| 9.03941 | 8.585399 | 9.646747 | 8.85991 |
| 12.18738 | 11.36027 | 11.03087 | 11.57135 |
| 1.855974 | 0.886955 | 0.858851 | 0.615538 |
| 0 | 0 | 0 | 0 |
| 9.863743 | 8.501682 | 8.43846 | 8.879331 |
| 0.020689 | 0.033369 | 0.012671 | 0.012351 |
| 21.5018 | 25.56273 | 24.00504 | 23.53498 |
| 0.413539 | 0.275266 | 0.410067 | 0.282139 |
| 402.2421 | 457.8554 | 458.6175 | 455.515 |
| 1.708992 | 1.457411 | 1.487026 | 1.519782 |

| | | | |
|---|---|---|---|
| 0.44501 | 0 | 0 | 0 |
| 2.728774 | 2.410993 | 3.834854 | 2.954943 |
| 2.779485 | 2.540361 | 3.064193 | 2.986704 |
| 0.100395 | 0.107951 | 0.140545 | 0.102743 |
| 0.01629 | 0 | 0 | 0 |
| 7.390488 | 6.438342 | 5.93443 | 6.745833 |
| 0 | 0 | 0 | 0.029487 |
| 5.645201 | 4.328204 | 4.028707 | 4.747359 |
| 0.506939 | 0.175207 | 0.15524 | 0.172931 |
| 269.2403 | 265.3583 | 261.9146 | 265.2417 |
| 738.0487 | 643.2497 | 643.2495 | 657.9017 |
| 0.230029 | 0.288564 | 0.281768 | 0.045774 |
| 5.699074 | 5.589028 | 6.00315 | 4.853953 |
| 0 | 0 | 0 | 0 |
| 19.6283 | 13.12883 | 12.55364 | 12.77122 |
| 17.35279 | 17.91236 | 17.17473 | 17.2929 |
| 0 | 0 | 0 | 0 |
| 0 | 0 | 0 | 0.101884 |
| 0 | 0 | 0 | 0 |
| 7.375219 | 4.184398 | 4.3297 | 4.97092 |
| 183.3073 | 193.2325 | 183.0861 | 196.3857 |
| 10.63271 | 14.77045 | 13.99502 | 16.08547 |
| 12.00216 | 12.48327 | 11.81635 | 11.91931 |
| 0.027862 | 0 | 0 | 0 |
| 62.57214 | 63.05503 | 64.88721 | 59.42579 |
| 0.129886 | 0.111728 | 0.06364 | 0.062031 |
| 0.499906 | 0.089588 | 0 | 0 |
| 11.8293 | 12.71954 | 13.84939 | 13.55863 |
| 0.187887 | 0.057722 | 0.065757 | 0.192281 |
| 16.77723 | 13.6598 | 12.82666 | 13.98608 |
| 32.74552 | 29.17294 | 32.86185 | 28.73353 |
| 0 | 0 | 0.031241 | 0 |
| 0 | 0.93944 | 0.535101 | 1.043139 |
| 81.54389 | 100.3482 | 99.01051 | 97.95437 |
| 8.105309 | 7.918854 | 7.958267 | 7.567511 |
| 4.352429 | 2.96427 | 3.241412 | 2.782195 |
| 0.953083 | 0.327939 | 0.280189 | 0.682759 |
| 5.162568 | 4.132323 | 4.001384 | 4.496696 |
| 0.077299 | 0.249348 | 0.18937 | 0.184581 |
| 21.53836 | 27.85508 | 26.97724 | 26.76225 |
| 28.70723 | 29.67446 | 28.87079 | 29.12111 |
| 8.352929 | 7.718516 | 6.687913 | 7.479723 |
| 7.197138 | 5.826148 | 6.067251 | 5.979166 |
| 1.277595 | 1.092978 | 1.256535 | 1.180222 |
| 0.936517 | 1.0542 | 0.985843 | 0.838615 |
| 26.3873 | 24.98866 | 25.45741 | 24.12465 |
| 0.36969 | 0.238507 | 0.090568 | 0.088278 |
| 2.121213 | 2.057549 | 1.471779 | 2.072142 |
| 0.416467 | 2.189288 | 2.210609 | 2.375701 |
| 40.41241 | 37.68313 | 39.57525 | 40.68164 |
| 0.428973 | 0.184503 | 0.420368 | 0.409738 |
| 0.102954 | 0.055351 | 0 | 0 |
| 21.63259 | 19.71098 | 18.28047 | 19.65268 |
| 0.035748 | 0.012813 | 0.043788 | 0 |

| | | | |
|---|---|---|---|
| 12.1749 | 9.968042 | 10.28633 | 10.3676 |
| 829.6886 | 911.4504 | 896.8813 | 918.5023 |
| 4.919739 | 4.798771 | 4.476686 | 4.447391 |
| 1.710187 | 7.883393 | 0.640093 | 1.474687 |
| 55.0665 | 55.56454 | 53.87994 | 52.46038 |
| 0.097631 | 0.149969 | 0.102506 | 0.049957 |
| 1022.696 | 1244.162 | 1250.556 | 1248.766 |
| 0.89002 | 0 | 1.63531 | 0 |
| 0.350978 | 0.377392 | 1.003151 | 1.257149 |
| 13.53298 | 12.5304 | 13.60941 | 14.36238 |
| 17.26048 | 14.54897 | 15.0734 | 15.14729 |
| 0 | 0 | 0 | 0 |
| 50.76669 | 47.85206 | 48.60888 | 49.21571 |
| 12.23574 | 10.8792 | 10.95567 | 11.30319 |
| 0.175188 | 0.037674 | 0.042918 | 0.209165 |
| 0.440889 | 0 | 0 | 0 |
| 9.988447 | 9.601585 | 8.578864 | 9.397203 |
| 5.563181 | 4.974154 | 5.007053 | 5.308958 |
| 2.983614 | 3.797404 | 3.132602 | 2.907984 |
| 157.2597 | 101.6694 | 98.00781 | 104.0887 |
| 0.280921 | 0.453093 | 0.172053 | 0.167702 |
| 0 | 0 | 0 | 0 |
| 11.26055 | 7.341668 | 7.400228 | 6.87164 |
| 0.799463 | 0.515777 | 0.665912 | 0.572711 |
| 10.9917 | 7.422271 | 7.997618 | 8.320312 |
| 0 | 0 | 0 | 0 |
| 0.168851 | 0 | 0.20683 | 0.1008 |
| 14.30305 | 15.53876 | 14.9375 | 13.97465 |
| 2.09117 | 2.272593 | 2.273867 | 2.216365 |
| 6.520194 | 6.890698 | 6.937059 | 7.384414 |
| 0.381607 | 0.197564 | 0.398189 | 0.35437 |
| 22.26148 | 29.27692 | 25.66774 | 24.70656 |
| 5.222064 | 4.135416 | 4.538152 | 4.391794 |
| 83.32808 | 75.95836 | 70.35315 | 73.03497 |
| 2.610981 | 1.255976 | 1.767456 | 1.72276 |
| 0.010383 | 0 | 0 | 0.012397 |
| 0.071335 | 0 | 0 | 0 |
| 0 | 0.049325 | 0 | 0 |
| 9.651901 | 8.462285 | 8.46799 | 9.159999 |
| 49.73755 | 54.30441 | 53.92273 | 55.86604 |
| 59.39641 | 70.4416 | 67.77169 | 70.78202 |
| 0.128901 | 0.110881 | 0.157894 | 0.123121 |
| 3.364731 | 3.9834 | 3.413802 | 3.408631 |
| 0.065384 | 0.017576 | 0.020023 | 0.019516 |
| 0.365964 | 0.349783 | 0.298853 | 0.339844 |
| 0.551037 | 0.662213 | 0.397046 | 0.425706 |
| 13.17324 | 11.57878 | 12.86315 | 11.42126 |
| 89.49154 | 78.77226 | 78.51541 | 76.9824 |
| 0 | 0.0624 | 0.035543 | 0 |
| 0.044294 | 0 | 0 | 0.052885 |
| 0.461707 | 0.604378 | 0.368841 | 0.527287 |
| 21.04776 | 19.93845 | 18.74494 | 19.62486 |
| 76.09826 | 53.62761 | 52.94257 | 53.86503 |
| 12.14234 | 9.063766 | 10.31001 | 9.989377 |

| | | | |
|---|---|---|---|
| 0.095423 | 0.184688 | 0.187017 | 0.136716 |
| 6.361943 | 6.598574 | 6.629137 | 6.755583 |
| 0 | 0 | 0 | 0 |
| 0 | 0 | 0 | 0 |
| 0.355675 | 0.047805 | 0.108919 | 0 |
| 0.096741 | 0.041609 | 0.118501 | 0.092403 |
| 26.47444 | 26.13236 | 24.42517 | 26.70164 |
| 4.023891 | 0.72112 | 3.285975 | 2.402159 |
| 3.837979 | 3.413299 | 3.339194 | 3.854311 |
| 0 | 0.123273 | 0.046811 | 0 |
| 1.444773 | 1.32213 | 1.110793 | 1.376317 |
| 0 | 0 | 0.038448 | 0 |
| 7.277469 | 6.959485 | 6.747397 | 6.956195 |
| 7.795885 | 7.684032 | 7.989016 | 7.969494 |
| 0 | 0 | 0 | 0 |
| 0.007712 | 0 | 0 | 0.018416 |
| 2.480002 | 1.599984 | 1.822689 | 0 |
| 14.01673 | 13.31461 | 11.35247 | 12.58954 |
| 15.46116 | 14.5321 | 14.9391 | 15.61985 |
| 0.490887 | 1.05566 | 1.202599 | 0.586094 |
| 0.772152 | 0.553508 | 0.315276 | 0.307303 |
| 0.00937 | 0 | 0.022954 | 0 |
| 0.034232 | 0.110423 | 0.083862 | 0.081741 |
| 35.45548 | 32.82726 | 31.42568 | 32.10126 |
| 0.225002 | 0.181451 | 0.155031 | 0.18469 |
| 0 | 0 | 0.008833 | 0 |
| 47.92424 | 54.81714 | 53.98464 | 55.07257 |
| 1.16662 | 1.293014 | 0.857413 | 0.900017 |
| 9.622863 | 7.534261 | 6.751938 | 7.956921 |
| 0.011619 | 0 | 0 | 0 |
| 92.47997 | 57.58768 | 57.57692 | 61.23227 |
| 1.030464 | 0.923345 | 0.894087 | 0.820214 |
| 0.604353 | 0.212446 | 0.284726 | 0.430164 |
| 2.383623 | 1.88055 | 1.883158 | 1.852375 |
| 8.218788 | 8.26824 | 7.207339 | 8.846393 |
| 1.121944 | 0.83833 | 0.791967 | 0.817348 |
| 178.1193 | 187.0994 | 182.2989 | 177.4549 |
| 10.7869 | 11.62929 | 10.38922 | 9.888623 |
| 5.586949 | 3.174368 | 2.41081 | 2.539349 |
| 4.28422 | 3.816928 | 2.998769 | 2.630642 |
| 5.350989 | 5.142185 | 4.749679 | 4.691295 |
| 0.10779 | 0.231803 | 0.132034 | 0.193043 |
| 0 | 0 | 0 | 0 |
| 10.11557 | 8.924917 | 8.250789 | 7.57159 |
| 1041.945 | 1159.839 | 1163.281 | 1184.861 |
| 52.66081 | 68.13766 | 68.23312 | 68.78088 |
| 2.800944 | 1.978724 | 2.436468 | 1.793257 |
| 0 | 0 | 0 | 0 |
| 0.013687 | 0 | 0.050296 | 0 |
| 0 | 0 | 0 | 0 |
| 21.82702 | 36.79237 | 24.44224 | 27.52246 |
| 0.054543 | 0 | 0 | 0.065122 |
| 7.039744 | 8.074169 | 7.353635 | 7.914792 |
| 0 | 0 | 0 | 0 |

| | | | |
|---|---|---|---|
| 0.143807 | 0.154629 | 0.07829 | 0.057233 |
| 0.241553 | 0.129866 | 0.221913 | 0.0721 |
| 10.9494 | 8.988728 | 11.0219 | 9.031744 |
| 0 | 0.179647 | 0 | 0.598433 |
| 0 | 0.142024 | 0.242688 | 0 |
| 1.001126 | 1.054041 | 0.536508 | 1.020979 |
| 75.84654 | 60.61152 | 56.92264 | 58.65675 |
| 8.2577 | 10.3951 | 10.82435 | 10.09973 |
| 0 | 0 | 0 | 0 |
| 3.82886 | 3.157405 | 3.350046 | 2.818494 |
| 2.208805 | 1.713139 | 1.929417 | 1.750927 |
| 7.314929 | 7.078885 | 6.762442 | 6.558473 |
| 1.763557 | 0.474069 | 0 | 0 |
| 0 | 0 | 0 | 0 |
| 29.4207 | 27.98616 | 27.29821 | 26.91448 |
| 10.40414 | 11.02381 | 10.97683 | 9.973875 |
| 0 | 0 | 0 | 0 |
| 0 | 0 | 0 | 0 |
| 14.9572 | 16.23038 | 15.49759 | 16.3836 |
| 0.820562 | 1.033569 | 1.119998 | 1.035692 |
| 4.64802 | 3.872905 | 3.990923 | 3.961375 |
| 0 | 0.016213 | 0 | 0 |
| 0.024171 | 0.02599 | 0.088821 | 0 |
| 0 | 0 | 0 | 0 |
| 2.382541 | 1.926058 | 1.768102 | 2.097137 |
| 0 | 0 | 0 | 0 |
| 23.11567 | 21.12099 | 20.43601 | 22.65244 |
| 11.82163 | 10.14246 | 11.04966 | 10.32762 |
| 1.376435 | 0.647509 | 0.895703 | 0.77034 |
| 0.661334 | 0.711104 | 0.405042 | 0.394799 |
| 29.80803 | 24.33524 | 23.57737 | 24.24195 |
| 0 | 0.270897 | 0.617207 | 1.203198 |
| 47.1594 | 41.64701 | 40.62992 | 42.67549 |
| 0.078091 | 0.230912 | 0.11957 | 0.233092 |
| 5.258214 | 5.496877 | 5.081166 | 4.81316 |
| 6.724817 | 5.281561 | 5.58017 | 5.032052 |
| 0.587852 | 0 | 0 | 0 |
| 19.66972 | 16.81013 | 19.65063 | 17.93371 |
| 0 | 0.041108 | 0 | 0 |
| 0.015232 | 0.016379 | 0.037317 | 0.018187 |
| 0.364409 | 0.848971 | 0.371977 | 0.145028 |
| 36.8262 | 39.40847 | 37.99813 | 40.95799 |
| 6.051971 | 5.345384 | 4.315546 | 4.387056 |
| 0.078965 | 0.42454 | 0.096726 | 0 |
| 0.351639 | 0.066724 | 0.076011 | 0.049393 |
| 0 | 0 | 0.090851 | 0 |
| 0.086785 | 0.124422 | 0.082682 | 0.149669 |
| 7.139661 | 6.791166 | 6.794616 | 6.590005 |
| 0 | 0 | 0 | 0 |
| 5.91493 | 5.51983 | 5.694267 | 5.243811 |
| 14.70051 | 13.28749 | 14.02603 | 15.25053 |
| 0.453486 | 0 | 1.110973 | 0 |
| 0.184558 | 0 | 0 | 0 |
| 21.03454 | 21.73173 | 21.19113 | 23.60599 |

| | | | |
|---|---|---|---|
| 1.438636 | 0.940275 | 0.656514 | 0.74095 |
| 0.475566 | 0.159799 | 0.400491 | 0.425851 |
| 4.595115 | 3.341691 | 4.133129 | 3.259992 |
| 0 | 0 | 0 | 0 |
| 2.809798 | 2.697549 | 2.950106 | 2.216533 |
| 0 | 0 | 0.940743 | 0 |
| 57.21723 | 55.44427 | 60.71403 | 59.41142 |
| 24.63693 | 1.312173 | 1.579429 | 1.731924 |
| 30.51726 | 28.26128 | 25.6124 | 24.45919 |
| 13.86595 | 13.20108 | 13.85907 | 11.95655 |
| 43.88269 | 41.71652 | 40.13065 | 42.3007 |
| 7.550113 | 7.077503 | 7.227916 | 6.850977 |
| 15.99643 | 13.35888 | 14.30393 | 13.83079 |
| 47.76508 | 58.09018 | 56.28754 | 54.77515 |
| 18.95892 | 19.02666 | 16.554 | 16.83187 |
| 0 | 0 | 0 | 0 |
| 17.72874 | 60.79536 | 55.83161 | 60.06907 |
| 4.353467 | 3.596871 | 3.156469 | 3.000209 |
| 6.698554 | 5.481675 | 5.645629 | 5.290531 |
| 0.340723 | 0.091591 | 0 | 0.305104 |
| 0 | 0 | 0 | 0 |
| 0.118154 | 0 | 0.14473 | 0.14107 |
| 0 | 0 | 0 | 0 |
| 409.9871 | 350.9717 | 340.1766 | 355.7057 |
| 23.75726 | 21.45138 | 20.9551 | 26.0618 |
| 0.915693 | 0.984606 | 0.981448 | 0.136661 |
| 15.96969 | 17.64413 | 15.97237 | 17.02617 |
| 2.272187 | 2.684818 | 2.752672 | 2.861932 |
| 1.65827 | 1.146257 | 1.334825 | 1.527343 |
| 0 | 0.138377 | 0 | 0 |
| 4.401483 | 4.292699 | 4.210705 | 4.581963 |
| 0.371275 | 0.49902 | 0.227392 | 0.443283 |
| 0 | 0 | 0 | 0 |
| 0.621349 | 0.64584 | 0.659625 | 0.667673 |
| 4.725256 | 2.345015 | 1.335711 | 2.169889 |
| 56.52767 | 43.16727 | 42.96865 | 44.12479 |
| 0 | 0.130945 | 0 | 0.290798 |
| 3.443928 | 2.978586 | 1.742445 | 3.396763 |
| 21.99878 | 22.72585 | 22.31299 | 19.73587 |
| 0 | 0 | 0 | 0 |
| 0.560409 | 0.562411 | 0.411875 | 0.579886 |
| 0.086261 | 0 | 0.105663 | 0 |
| 0.572997 | 0.492895 | 0.491315 | 0.342064 |
| 39.58306 | 35.64499 | 35.59751 | 37.2639 |
| 0 | 0 | 0.264717 | 0.344031 |
| 28.69035 | 27.64238 | 26.70559 | 25.77588 |
| 9.596059 | 9.156753 | 9.338939 | 10.15252 |
| 15.23122 | 13.39614 | 12.36259 | 13.59483 |
| 10.69028 | 11.45425 | 10.58899 | 11.30043 |
| 0.237783 | 0.255678 | 0.509716 | 0.567801 |
| 0 | 0.334637 | 0 | 0 |
| 424.5154 | 495.2347 | 496.5193 | 506.8493 |
| 0.741772 | 0.429474 | 0.31452 | 0.545007 |
| 0.962548 | 0.937956 | 0.957977 | 1.041492 |

| | | | |
|---|---|---|---|
| 2.050566 | 1.769036 | 1.75241 | 1.679627 |
| 13.29365 | 12.60184 | 10.70542 | 11.95392 |
| 0 | 0 | 0 | 0 |
| 24.98606 | 24.20794 | 24.05015 | 23.16413 |
| 0 | 0 | 0.052077 | 0 |
| 0.681141 | 0.389089 | 0.46932 | 0.559108 |
| 0.52875 | 0.46098 | 0.665183 | 0.614237 |
| 8.38158 | 8.332542 | 7.766482 | 7.677916 |
| 31.3691 | 27.27952 | 25.56549 | 31.03681 |
| 3.283125 | 3.684025 | 3.925203 | 3.66368 |
| 0.248259 | 0.177961 | 0.081093 | 0.059282 |
| 72.36634 | 48.04736 | 51.81119 | 49.40095 |
| 8.832465 | 11.59751 | 10.87112 | 10.8666 |
| 2.820548 | 2.246205 | 2.150637 | 2.377691 |
| 0 | 0 | 0 | 0 |
| 11.36168 | 10.08551 | 10.18938 | 10.63978 |
| 14.24412 | 14.48464 | 15.10495 | 13.16807 |
| 7.626812 | 5.910475 | 4.460723 | 7.793439 |
| 20.47027 | 10.89372 | 10.9412 | 10.65324 |
| 0.699361 | 0.735993 | 0.747303 | 0.781702 |
| 0 | 0 | 0 | 0 |
| 0.139228 | 0.149706 | 0.170544 | 0.332463 |
| 1.591417 | 1.314551 | 1.252242 | 1.245741 |
| 81.72186 | 79.99516 | 80.2443 | 85.52755 |
| 0.011485 | 0 | 0 | 0 |
| 7.28817 | 6.775937 | 5.735216 | 6.029661 |
| 15.13181 | 13.57588 | 13.13405 | 11.93077 |
| 0 | 0 | 0 | 0 |
| 0 | 0.116894 | 0 | 0.259594 |
| 0.025008 | 0 | 0.030633 | 0.089576 |
| 0 | 0 | 0 | 0 |
| 3.571203 | 3.199968 | 2.187227 | 4.97447 |
| 12.36511 | 10.75385 | 9.10052 | 9.986934 |
| 0 | 0 | 0 | 0 |
| 0.189705 | 0.135988 | 0.116187 | 0.113249 |
| 0 | 0 | 0 | 0 |
| 0.825919 | 0.501956 | 0.527838 | 0.342993 |
| 0.187118 | 0.082847 | 0.080896 | 0.07885 |
| 0 | 0 | 0 | 0 |
| 0 | 0 | 0 | 0 |
| 3.211639 | 2.514931 | 2.180813 | 2.334062 |
| 0.084426 | 0 | 0.103415 | 0.1008 |
| 5.540625 | 6.315947 | 5.000835 | 4.774894 |
| 0.322508 | 0.12385 | 0.084653 | 0.275042 |
| 1.554495 | 1.552936 | 1.43148 | 1.513747 |
| 24.24891 | 29.62933 | 24.57255 | 24.47755 |
| 39.99032 | 38.0339 | 38.59168 | 37.74399 |
| 0 | 0 | 0 | 0 |
| 21.43301 | 13.57845 | 12.91404 | 15.35394 |
| 0.098516 | 0.05885 | 0.134083 | 0.104554 |
| 0.05131 | 0.027586 | 0 | 0 |
| 37.77802 | 24.01298 | 23.21795 | 23.45303 |
| 10.3319 | 9.257877 | 9.749655 | 10.2798 |
| 1.767193 | 1.42514 | 1.443119 | 1.992719 |

| | | | |
|---|---|---|---|
| 3.819469 | 3.285529 | 3.158029 | 3.116169 |
| 97.5458 | 112.8478 | 104.8055 | 112.1552 |
| 20.67996 | 20.50782 | 20.16932 | 19.08869 |
| 12.58707 | 12.3592 | 10.80198 | 11.29373 |
| 0.051628 | 0.055514 | 0.216826 | 0.008806 |
| 18.05585 | 15.33625 | 15.81984 | 16.09346 |
| 0 | 0 | 0 | 0 |
| 1.194668 | 2.079788 | 0.905901 | 1.494294 |
| 0 | 0 | 0 | 0 |
| 0.110478 | 0.356377 | 0.270655 | 0.131905 |
| 8.136656 | 8.615084 | 7.627645 | 7.781709 |
| 0 | 0.538942 | 0.613959 | 0 |
| 44.17806 | 41.46481 | 44.00775 | 45.42614 |
| 0.027846 | 0.00998 | 0 | 0 |
| 0 | 0 | 0 | 0 |
| 9.457228 | 11.1149 | 11.53949 | 10.63496 |
| 7.156315 | 5.791551 | 7.434017 | 6.491229 |
| 0 | 0 | 0 | 0 |
| 0 | 0 | 0.355647 | 0 |
| 0.010861 | 0 | 0 | 0 |
| 0 | 0.029629 | 0.033754 | 0 |
| 41.81337 | 36.61039 | 37.99556 | 36.78 |
| 3.206119 | 2.691198 | 2.280341 | 3.012959 |
| 0.025247 | 0 | 0 | 0 |
| 0 | 0 | 0.086281 | 0 |
| 15.68247 | 8.270439 | 8.90838 | 8.611636 |
| 4.91426 | 4.587042 | 3.560528 | 4.244482 |
| 0 | 0 | 0 | 0 |
| 0.148337 | 0.1595 | 0 | 0 |
| 10.53876 | 9.580588 | 6.924019 | 10.63813 |
| 535.5262 | 590.4276 | 575.2648 | 590.5315 |
| 0 | 0 | 0 | 0 |
| 22.16033 | 24.13617 | 24.86316 | 26.17317 |
| 7.51388 | 6.354546 | 6.2049 | 6.551987 |
| 2.115977 | 1.921924 | 1.980156 | 2.19684 |
| 0 | 0.151031 | 0 | 0.335405 |
| 0 | 0 | 0 | 0 |
| 34.72003 | 42.31069 | 43.74454 | 32.37354 |
| 12.02745 | 9.003974 | 8.829951 | 10.11577 |
| 0.153742 | 0.141696 | 0.107613 | 0.262228 |
| 19.84649 | 12.83467 | 13.4318 | 13.24671 |
| 15.78655 | 10.27928 | 10.22949 | 10.53932 |
| 16.78181 | 14.89196 | 13.52599 | 14.58054 |
| 0.443352 | 0.286031 | 0.108615 | 0.317604 |
| 11.06983 | 8.412037 | 8.920131 | 8.77531 |
| 1.082183 | 0.387875 | 0.441864 | 0.86138 |
| 0 | 0.487614 | 0 | 0 |
| 0 | 0 | 0.173074 | 0 |
| 0 | 0 | 0 | 0.481789 |
| 0.62533 | 1.112501 | 0.348174 | 1.208153 |
| 3.963951 | 3.460367 | 3.316463 | 3.687481 |
| 0.945345 | 0.893809 | 1.042179 | 0.965227 |
| 1.824002 | 1.961271 | 0.705557 | 1.833906 |
| 0.350342 | 0.212922 | 0.130609 | 0.309171 |

| | | | |
|---|---|---|---|
| 0 | 0 | 0.019061 | 0 |
| 14.54492 | 1.655279 | 7.28263 | 11.40825 |
| 5.139596 | 4.005899 | 3.297704 | 3.993537 |
| 0 | 0.180917 | 0 | 0 |
| 9.408515 | 6.590224 | 7.639243 | 6.782761 |
| 0.602735 | 0 | 0 | 0 |
| 0.331357 | 0.23753 | 0.514124 | 0.303311 |
| 11.44646 | 9.65619 | 10.46829 | 10.01838 |
| 33.67025 | 23.52857 | 22.83965 | 24.19391 |
| 1.042873 | 0.574353 | 0.498513 | 0.455538 |
| 1.643982 | 1.34175 | 0.994746 | 0.662159 |
| 0.884235 | 0.522929 | 0.758185 | 0.475079 |
| 0 | 0 | 0 | 0 |
| 35.13428 | 36.56592 | 35.84352 | 33.36419 |
| 1.137292 | 0.899856 | 1.550806 | 1.665309 |
| 10.37725 | 8.229393 | 8.653718 | 8.978461 |
| 30.23504 | 29.66124 | 30.03231 | 30.21581 |
| 0.348835 | 0.317382 | 0.295821 | 0.496586 |
| 25.90104 | 27.45174 | 28.62891 | 29.88326 |
| 24.40256 | 21.7927 | 20.82608 | 22.67263 |
| 0 | 1.796473 | 0 | 0 |
| 0 | 0 | 0 | 0 |
| 2.220041 | 1.824633 | 1.844179 | 1.949877 |
| 0.786451 | 0.768761 | 0.175153 | 0.341448 |
| 32.0848 | 30.7366 | 31.25814 | 31.3127 |
| 0 | 0 | 0 | 0 |
| 23.16384 | 18.49993 | 18.70774 | 16.27979 |
| 11.81205 | 9.932261 | 9.111882 | 9.027855 |
| 0 | 0 | 0 | 0 |
| 7.660803 | 7.331467 | 6.78338 | 6.678896 |
| 0 | 0 | 0 | 0.421119 |
| 6.458403 | 9.406564 | 7.119951 | 8.341898 |
| 14.19656 | 12.72767 | 12.99529 | 12.27727 |
| 1.170886 | 0.616655 | 0.761029 | 0.722764 |
| 1.006416 | 0.338174 | 0.462294 | 0.300402 |
| 2.399258 | 1.786029 | 2.71284 | 2.86459 |
| 4.952572 | 23.16275 | 22.23536 | 23.12629 |
| 90.61448 | 64.92031 | 68.17885 | 69.83377 |
| 49.9513 | 54.12008 | 51.89498 | 50.85948 |
| 0.11507 | 0.12373 | 0 | 0.082433 |
| 0.034986 | 0.01881 | 0.042855 | 0.041772 |
| 3.148293 | 2.121839 | 2.380279 | 2.661804 |
| 0.119239 | 0.042737 | 0.109544 | 0.118637 |
| 0 | 0 | 0 | 0 |
| 4.174505 | 4.529658 | 4.459679 | 4.52897 |
| 1.070023 | 0.958792 | 0.655349 | 0.851702 |
| 0 | 0 | 0 | 0 |
| 1.133088 | 1.64337 | 1.64617 | 1.79331 |
| 0 | 0 | 0 | 0 |
| 0.70565 | 0.758755 | 1.089855 | 0.805879 |
| 5.19469 | 4.33043 | 4.075246 | 4.655127 |
| 0.136906 | 0.117768 | 0.10062 | 0.065384 |
| 8.488357 | 8.540911 | 8.511886 | 8.679555 |
| 0.898416 | 0.869425 | 0.770344 | 0.85813 |

| | | | |
|---|---|---|---|
| 3.132635 | 2.559974 | 2.558161 | 2.792685 |
| 36.10939 | 24.88964 | 24.95379 | 24.80386 |
| 0.171035 | 0.514937 | 0.377108 | 0.490096 |
| 6.282832 | 5.468867 | 6.004568 | 6.567139 |
| 15.66841 | 14.02061 | 14.41073 | 14.23655 |
| 0 | 0 | 0 | 0.056625 |
| 7.432382 | 7.366107 | 7.448815 | 8.156798 |
| 62.9783 | 59.49605 | 56.45281 | 54.36126 |
| 4254.064 | 4438.016 | 4483.68 | 4297.103 |
| 2.755302 | 2.775363 | 2.909514 | 2.930468 |
| 17.50891 | 17.28651 | 16.79246 | 17.62419 |
| 3.875107 | 2.968086 | 2.99108 | 1.837995 |
| 1.584295 | 1.656638 | 1.922837 | 1.527135 |
| 0 | 0 | 0 | 0 |
| 0.453666 | 0.284555 | 0.254699 | 0.225689 |
| 2.200009 | 1.803459 | 2.187894 | 2.054545 |
| 0.318198 | 0.537656 | 0.250565 | 0.325639 |
| 3.468844 | 3.240732 | 3.529284 | 3.440034 |
| 0 | 0 | 0 | 0 |
| 1.943512 | 3.134662 | 3.570983 | 4.640905 |
| 0.812347 | 0.537527 | 0.765434 | 0.708773 |
| 1.310011 | 1.07176 | 0.837216 | 1.054057 |
| 325.1027 | 319.8497 | 333.5312 | 347.3144 |
| 1.135176 | 1.715447 | 1.615993 | 1.172187 |
| 0.057507 | 0.061835 | 0.035221 | 0.068661 |
| 0 | 0 | 0 | 0 |
| 1.484339 | 1.092032 | 1.100492 | 1.492399 |
| 8.981087 | 7.330295 | 7.167849 | 6.569891 |
| 0 | 0.501956 | 0 | 0 |
| 13.84187 | 6.548771 | 4.74747 | 7.93271 |
| 7.160308 | 7.121733 | 6.578127 | 5.80622 |
| 10.77454 | 10.64259 | 10.1215 | 10.2914 |
| 188.1034 | 125.5033 | 123.612 | 119.2917 |
| 0.106762 | 0 | 0 | 0 |
| 22.45612 | 19.19703 | 18.7594 | 19.56643 |
| 27.06596 | 22.63556 | 22.65507 | 20.52623 |
| 12.02498 | 11.77853 | 11.5525 | 11.40242 |
| 0.06975 | 0 | 0 | 0 |
| 2.324517 | 2.310815 | 2.542923 | 2.757897 |
| 1.740744 | 1.123048 | 0.852912 | 1.82256 |
| 0.314297 | 0.316829 | 0.264681 | 0.257988 |
| 0 | 0.711104 | 0 | 0 |
| 2.532095 | 2.464001 | 2.620873 | 2.569711 |
| 532.2794 | 510.5473 | 506.7076 | 513.4364 |
| 54.86808 | 54.22189 | 53.98776 | 55.17307 |
| 0 | 0 | 0 | 0 |
| 0.44501 | 0.957 | 0 | 0 |
| 82.25387 | 85.19104 | 82.11837 | 88.36523 |
| 0 | 0 | 0 | 0 |
| 29.30864 | 26.09639 | 22.39947 | 26.74608 |
| 0 | 0 | 0 | 0 |
| 0 | 0 | 0 | 0 |
| 0.584758 | 0.404206 | 0.972101 | 0.299216 |
| 0.360728 | 0 | 0.441864 | 0.86138 |

| | | | |
|---|---|---|---|
| 5.340783 | 4.798357 | 5.466251 | 4.789548 |
| 5.733967 | 4.130278 | 4.00054 | 3.744284 |
| 7.944894 | 7.28145 | 7.331579 | 7.146175 |
| 0.393521 | 0 | 0 | 0 |
| 0 | 0.966028 | 0 | 0 |
| 26.49368 | 22.2827 | 24.31734 | 22.48911 |
| 0.097975 | 0.052674 | 0 | 0 |
| 0.813618 | 0.604062 | 0.545769 | 0.69387 |
| 0.028399 | 0.020358 | 0.011596 | 0 |
| 0.280094 | 0.45176 | 0.514642 | 0.334418 |
| 1.208529 | 0 | 0.592143 | 0.865753 |
| 0 | 0 | 0 | 0.016522 |
| 18.82435 | 10.96735 | 11.92458 | 10.69812 |
| 0.183492 | 0.09865 | 0 | 0.219079 |
| 1.212571 | 0.330821 | 0.376869 | 0.388947 |
| 2.440478 | 2.279364 | 2.312968 | 2.36082 |
| 0 | 0.012623 | 0 | 0 |
| 0 | 0.673677 | 0 | 0 |
| 7.288498 | 6.525086 | 9.006519 | 6.938669 |
| 1.785602 | 1.033836 | 1.402069 | 1.202619 |
| 5.240353 | 4.104307 | 3.803873 | 4.40287 |
| 2.564799 | 2.473043 | 2.168445 | 2.529674 |
| 12.10311 | 11.50411 | 11.65494 | 11.56039 |
| 22.47217 | 21.45124 | 20.49701 | 21.8884 |
| 0 | 0.157054 | 0 | 0.46504 |
| 1.612496 | 1.427874 | 1.161874 | 1.245741 |
| 12.42158 | 11.14516 | 11.86809 | 10.43094 |
| 14.8624 | 16.05664 | 14.92664 | 16.65165 |
| 0.064346 | 0.034594 | 0 | 0.038413 |
| 16.8635 | 17.07531 | 16.53124 | 16.99369 |
| 0.321396 | 0.292417 | 0.272552 | 0.206624 |
| 65.02471 | 76.24484 | 75.60098 | 77.31024 |
| 77.99962 | 71.22417 | 70.84302 | 75.65707 |
| 31.49931 | 32.21583 | 28.26373 | 27.96639 |
| 0.312032 | 0.201309 | 0.229329 | 0.111765 |
| 0.710687 | 0.382086 | 0 | 0.848524 |
| 27.16103 | 18.08106 | 19.60713 | 18.26638 |
| 16.63906 | 13.51362 | 13.22636 | 15.53366 |
| 24.91125 | 19.37555 | 19.53656 | 19.53037 |
| 0 | 0 | 0 | 0 |
| 10.38591 | 9.651501 | 9.746659 | 9.56685 |
| 7.024775 | 6.057003 | 6.940679 | 5.57829 |
| 27.84508 | 24.84346 | 24.17073 | 22.34011 |
| 0 | 0.022745 | 0.025911 | 0.025256 |
| 20.35904 | 20.23496 | 17.47321 | 19.67 |
| 6.066029 | 5.93404 | 5.810259 | 6.697973 |
| 6.938797 | 5.679763 | 5.305352 | 5.379102 |
| 30.12847 | 18.93126 | 20.03974 | 19.17868 |
| 0.124324 | 0 | 0.076144 | 0.371091 |
| 10.01857 | 9.964599 | 10.12439 | 8.904788 |
| 0 | 0 | 0 | 0 |
| 90.86692 | 76.77528 | 77.25884 | 79.63939 |
| 40.10279 | 42.84461 | 44.6393 | 44.23881 |
| 16.93116 | 17.76608 | 17.5701 | 16.09607 |

| | | | |
|---|---|---|---|
| 1.058134 | 0.325076 | 0.740648 | 1.082878 |
| 16.43446 | 14.26387 | 12.83812 | 13.28327 |
| 0.299607 | 0.46022 | 0.498065 | 0.511021 |
| 37.49227 | 27.06039 | 30.23147 | 27.79387 |
| 0 | 0 | 0 | 0 |
| 1.117748 | 2.644105 | 1.642988 | 2.669065 |
| 7.325545 | 5.370575 | 5.166411 | 5.433321 |
| 55.72484 | 56.33536 | 55.74085 | 56.63005 |
| 11.92304 | 11.86562 | 11.71493 | 12.45858 |
| 0.32111 | 0.542576 | 0.618099 | 0.383389 |
| 68.93779 | 72.4651 | 73.23058 | 68.41021 |
| 0.093548 | 0.100588 | 0.572948 | 0.111692 |
| 0 | 0 | 0.613959 | 0.598433 |
| 17.59039 | 12.48252 | 14.89484 | 15.035 |
| 0.365893 | 0.33978 | 0.142607 | 0.297858 |
| 1.058134 | 1.137766 | 0.958013 | 0.659143 |
| 1.255808 | 0.590763 | 0.737088 | 0.843395 |
| 0 | 0 | 0 | 0 |
| 3.229736 | 2.619148 | 2.961611 | 2.391236 |
| 0 | 0 | 0 | 0 |
| 4.133337 | 4.4444 | 3.442858 | 5.13239 |
| 31.90658 | 37.90335 | 36.56579 | 32.31404 |
| 0.264343 | 0.210546 | 0.305812 | 0.274699 |
| 19.99329 | 17.42052 | 18.31555 | 17.36439 |
| 4.06248 | 3.916716 | 3.831827 | 4.073325 |
| 2.34974 | 2.294926 | 2.141813 | 2.386741 |
| 9.141382 | 6.9071 | 6.896415 | 6.927609 |
| 0.3472 | 0.106666 | 0 | 0.05922 |
| 3.726957 | 3.588749 | 3.588597 | 2.590178 |
| 0.648238 | 0.633657 | 0.697795 | 0.445615 |
| 0 | 0 | 0.106241 | 0 |
| 0.173107 | 0.024818 | 0.098954 | 0.096451 |
| 0.114599 | 0.092418 | 0.105282 | 0.239445 |
| 0.529067 | 0.393842 | 0.299108 | 0.534497 |
| 5.110142 | 4.8748 | 3.049519 | 3.754612 |
| 0.070752 | 0 | 0 | 0 |
| 0.76617 | 0.579732 | 0.695185 | 0.271042 |
| 0 | 0 | 0 | 0.117704 |
| 0.0496 | 0 | 0 | 0 |
| 0 | 0.110821 | 0 | 0 |
| 0 | 0.506926 | 0 | 0 |
| 109.3843 | 97.75011 | 96.38628 | 93.29497 |
| 8.647595 | 8.714806 | 8.199332 | 9.028782 |
| 59.33153 | 52.87911 | 50.90334 | 52.73517 |
| 0.201549 | 0.135448 | 0.246883 | 0.18048 |
| 18.54338 | 17.55259 | 17.48876 | 16.89929 |
| 1.200405 | 0.823083 | 1.513025 | 0.934709 |
| 0.775457 | 0.689501 | 0.730674 | 0.623172 |
| 5.157526 | 5.236428 | 4.967168 | 5.562639 |
| 0.147786 | 0.062013 | 0.105967 | 0.073162 |
| 0 | 0 | 0 | 0 |
| 0.107729 | 0.057918 | 0.06598 | 0.064311 |
| 6.754049 | 5.809871 | 6.2049 | 6.854386 |
| 2.932023 | 2.501124 | 2.897148 | 3.547359 |

| | | | |
|---|---|---|---|
| 10.92827 | 19.02495 | 16.89225 | 14.91176 |
| 4.111805 | 3.276071 | 3.352262 | 3.138721 |
| 8.1301 | 6.155641 | 6.185895 | 7.121005 |
| 18.3852 | 17.25436 | 14.37162 | 16.70392 |
| 0.067112 | 0 | 0.041104 | 0.040064 |
| 12.41978 | 9.672 | 11.47315 | 9.655817 |
| 0 | 0 | 0 | 0.056568 |
| 6.113956 | 7.310923 | 7.541598 | 6.634971 |
| 2.25135 | 2.118182 | 1.65464 | 1.679997 |
| 4.551322 | 5.761104 | 5.433886 | 4.746189 |
| 1.065951 | 0.825244 | 0.770369 | 0.763614 |
| 1.830804 | 1.8547 | 1.797784 | 1.391018 |
| 0 | 0 | 0.01939 | 0.0189 |
| 6.556119 | 5.942037 | 5.944212 | 5.486461 |
| 5.625956 | 2.69471 | 1.315625 | 3.35855 |
| 0 | 0.059259 | 0 | 0 |
| 15.23072 | 11.03289 | 9.033666 | 8.230966 |
| 201.0872 | 177.8446 | 176.6555 | 175.9852 |
| 0.31086 | 0.200553 | 0.171351 | 0.139182 |
| 0.26207 | 0.260116 | 0.172855 | 0.192552 |
| 11.88217 | 11.17934 | 8.454603 | 10.84865 |
| 0.51401 | 0.401959 | 0.686862 | 0.50212 |
| 68.44807 | 59.66999 | 62.93639 | 54.13394 |
| 10.48639 | 8.956904 | 8.584555 | 8.987278 |
| 10.60243 | 9.621771 | 9.200637 | 10.32773 |
| 0 | 0 | 0.305372 | 0.595299 |
| 0.082667 | 0 | 0 | 0 |
| 0.069243 | 0.024818 | 0 | 0.027557 |
| 0.007662 | 0.012358 | 0.018771 | 0.02287 |
| 11.07935 | 10.20741 | 10.39444 | 9.861003 |
| 0.531632 | 0.285821 | 0.317036 | 0.242204 |
| 0.05414 | 0.029107 | 0.066317 | 0.09696 |
| 0.044773 | 0.072214 | 0 | 0.053457 |
| 0 | 0 | 0 | 0 |
| 0 | 0 | 0 | 0 |
| 9.334941 | 8.51919 | 9.320639 | 10.58348 |
| 6.412094 | 5.80225 | 5.569435 | 5.826292 |
| 7.081632 | 7.216798 | 5.631928 | 7.63483 |
| 25.436 | 22.41791 | 26.83836 | 22.37961 |
| 1.916748 | 1.084735 | 1.977155 | 2.16805 |
| 0.571393 | 0.348157 | 0.163313 | 0.272885 |
| 3.192388 | 1.982228 | 2.478444 | 1.932615 |
| 3.513571 | 3.316427 | 3.544355 | 2.999156 |
| 0.035271 | 0.113777 | 0.086409 | 0.042112 |
| 7.801499 | 7.209315 | 6.337034 | 6.893287 |
| 0.793601 | 0.831445 | 0.872398 | 0.898927 |
| 10.70671 | 8.627612 | 9.122085 | 8.981214 |
| 0.764478 | 0.11743 | 0.200663 | 0 |
| 0 | 0.009975 | 0 | 0.011076 |
| 2.824681 | 1.735576 | 1.812392 | 1.445367 |
| 6.764519 | 6.515437 | 6.180781 | 7.366175 |
| 0.815343 | 0.487058 | 0.429086 | 0.526399 |
| 2.652544 | 2.805664 | 3.037264 | 2.650641 |
| 0.246108 | 0.252028 | 0.114843 | 0.167909 |

| | | | |
|---|---|---|---|
| 0.420318 | 0.322821 | 0.294205 | 0.358456 |
| 0 | 0.022796 | 0 | 0.025312 |
| 11.97682 | 12.99559 | 13.42213 | 12.47421 |
| 3.139519 | 2.250527 | 4.48662 | 4.373161 |
| 74.81487 | 34.15029 | 34.91286 | 33.45368 |
| 13.81133 | 13.96699 | 13.65683 | 13.50347 |
| 0.062984 | 0 | 0 | 0.0376 |
| 4.339793 | 4.470143 | 4.992989 | 4.576174 |
| 4.788206 | 4.29046 | 3.714621 | 4.065329 |
| 0 | 0.019632 | 0 | 0 |
| 0.078252 | 0 | 0.047926 | 0.046714 |
| 8.275459 | 6.846577 | 6.985471 | 6.885611 |
| 11.1075 | 8.728785 | 8.901273 | 9.014884 |
| 29.18403 | 25.32448 | 28.11776 | 22.92383 |
| 5.347467 | 3.889639 | 5.249827 | 5.046649 |
| 9.940894 | 9.07219 | 7.367502 | 6.981713 |
| 8.353962 | 6.517802 | 7.048125 | 6.778046 |
| 88.32807 | 86.48652 | 93.97398 | 84.35252 |
| 0 | 0.639994 | 0.364538 | 0 |
| 1.06278 | 0.711885 | 1.088412 | 0.977681 |
| 30.19554 | 28.72166 | 27.02915 | 21.49248 |
| 0 | 0 | 0 | 0 |
| 7.227435 | 6.857074 | 8.853063 | 2.537995 |
| 0.381692 | 0.273611 | 0.311696 | 0.22786 |
| 24.63699 | 22.2839 | 23.35479 | 22.24952 |
| 0 | 0.081269 | 0.046291 | 0 |
| 0 | 0.716077 | 0 | 0.39756 |
| 0.58828 | 0.781388 | 0.974927 | 0.537111 |
| 0 | 0 | 0 | 0.165747 |
| 0 | 0 | 0 | 0 |
| 0.919775 | 1.141148 | 0.693326 | 0.929215 |
| 37.8325 | 33.0494 | 32.86627 | 30.43087 |
| 84.65675 | 75.78221 | 72.27312 | 76.23165 |
| 0 | 0 | 0 | 0 |
| 9.8901 | 10.14842 | 9.541896 | 11.14167 |
| 0.2112 | 0.020645 | 0.094074 | 0.045848 |
| 0.195951 | 0 | 0.240025 | 0 |
| 18.5058 | 14.66036 | 14.1176 | 14.2571 |
| 0 | 0.068817 | 0 | 0 |
| 0.180565 | 0.216995 | 0.117094 | 0.152178 |
| 0.770004 | 0.72112 | 0.699791 | 0.741407 |
| 11.73462 | 12.16821 | 12.36096 | 11.61807 |
| 11.94452 | 11.00984 | 9.943123 | 10.2168 |
| 169.6127 | 211.5385 | 206.9186 | 207.2596 |
| 0.154264 | 0.055291 | 0 | 0 |
| 2.84558 | 2.345793 | 2.091373 | 1.35899 |
| 0 | 0 | 0.066964 | 0 |
| 21.93047 | 16.08465 | 15.96709 | 17.45589 |
| 10.85056 | 8.885653 | 9.319552 | 9.335429 |
| 0 | 0 | 0 | 0.72886 |
| 2.353217 | 2.736128 | 2.661842 | 2.903721 |
| 0 | 0 | 0 | 0 |
| 0.471446 | 0 | 0.577486 | 0 |
| 1.999624 | 0.537527 | 1.224694 | 0.596862 |

| | | | |
|---|---|---|---|
| 54.74226 | 61.30007 | 58.12767 | 59.11629 |
| 26.41381 | 23.44023 | 23.0595 | 23.44766 |
| 6.676789 | 5.748809 | 5.964816 | 6.892857 |
| 0.092279 | 0.099224 | 0 | 0.110177 |
| 17.6598 | 19.22325 | 17.67942 | 17.90913 |
| 0 | 0 | 0 | 0 |
| 3.341477 | 6.287656 | 0 | 2.992163 |
| 2.084831 | 1.000772 | 0.866454 | 1.022342 |
| 0 | 0 | 0.11783 | 0 |
| 7.483756 | 6.838988 | 7.494528 | 7.346275 |
| 11.92819 | 11.05798 | 10.54371 | 11.73973 |
| 0.749859 | 0 | 0 | 0 |
| 0.110993 | 0.059673 | 0 | 0.06626 |
| 10.85332 | 9.485065 | 8.316131 | 10.64235 |
| 5.690055 | 4.954308 | 4.895921 | 4.357865 |
| 14.97759 | 16.41951 | 15.08948 | 14.67876 |
| 1.869225 | 1.904113 | 1.887965 | 1.644453 |
| 3.910969 | 3.574502 | 4.646922 | 3.96907 |
| 8.920021 | 7.640074 | 8.359129 | 9.215796 |
| 10.1777 | 10.07203 | 9.929405 | 8.602939 |
| 1.676184 | 1.1348 | 1.140666 | 1.260063 |
| 4.666301 | 4.969134 | 4.988993 | 4.980911 |
| 1.757208 | 1.88945 | 1.784956 | 1.176935 |
| 4.756722 | 5.114699 | 4.903934 | 5.046398 |
| 3.43804 | 2.98519 | 2.965732 | 3.237621 |
| 0.016951 | 0.018227 | 0.020764 | 0.020239 |
| 5.217349 | 3.924777 | 3.864828 | 3.742471 |
| 6.586162 | 6.195372 | 6.136665 | 6.078826 |
| 0 | 0 | 0.114365 | 0.111473 |
| 0.353094 | 0.231504 | 0.295375 | 0.298188 |
| 237.5031 | 220.3129 | 227.4127 | 223.4994 |
| 0 | 0.522444 | 0 | 0 |
| 0 | 0 | 0 | 0.021608 |
| 2.528286 | 2.428294 | 2.411434 | 2.303287 |
| 2.295458 | 1.835334 | 2.88386 | 1.967652 |
| 11.36381 | 11.24741 | 12.13923 | 11.70326 |
| 0 | 0 | 0 | 0 |
| 0.252666 | 0.246983 | 0.168816 | 0.137123 |
| 0.460058 | 1.484043 | 2.535916 | 0.274643 |
| 5.024547 | 4.716847 | 5.293672 | 5.361844 |
| 0.102842 | 0.221164 | 0.125974 | 0 |
| 0.358332 | 0.113323 | 0.077458 | 0.050333 |
| 3.543658 | 2.241378 | 1.422587 | 2.062141 |
| 11.08962 | 9.390299 | 9.933259 | 8.109762 |
| 16.61025 | 14.37328 | 14.24241 | 13.69337 |
| 0.054222 | 0.038868 | 0.01107 | 0.01079 |
| 0.596443 | 0.90855 | 0.547948 | 1.068183 |
| 62.50185 | 68.30176 | 67.1898 | 67.54205 |
| 0 | 0 | 0 | 0 |
| 8.304345 | 7.417084 | 7.218978 | 9.195324 |
| 21.89243 | 19.59214 | 18.91128 | 18.97759 |
| 24.50213 | 31.38394 | 35.70983 | 31.53329 |
| 0.977455 | 0.750726 | 0.627162 | 0.38901 |
| 2.946921 | 1.167415 | 1.709884 | 1.111096 |

| | | | |
|---|---|---|---|
| 0.048687 | 0 | 0 | 0.11626 |
| 3.228206 | 1.880207 | 1.977155 | 2.569541 |
| 7.778717 | 6.088588 | 5.394723 | 6.385077 |
| 28.49748 | 26.41428 | 25.05369 | 25.1523 |
| 6.754705 | 7.402335 | 8.02465 | 8.153149 |
| 60.1913 | 51.8573 | 50.41603 | 51.76519 |
| 3.85694 | 3.662276 | 3.487691 | 3.303053 |
| 3.823186 | 3.031275 | 3.61877 | 3.158394 |
| 16.79976 | 8.425403 | 7.95495 | 7.903469 |
| 9.717742 | 8.769927 | 9.662713 | 8.704524 |
| 21.06954 | 20.34158 | 22.48839 | 20.9188 |
| 0.015689 | 0 | 0 | 0 |
| 0 | 0 | 0.028001 | 0 |
| 3.93626 | 3.618097 | 3.032955 | 3.790073 |
| 0.034367 | 0.073908 | 0.021049 | 0.082066 |
| 18.88037 | 19.93023 | 19.13824 | 19.6713 |
| 24.49325 | 22.95041 | 22.9423 | 24.13763 |
| 0.080569 | 0.173264 | 0.049345 | 0.336682 |
| 19.47815 | 18.81258 | 18.77669 | 18.87348 |
| 3.846278 | 3.235978 | 3.326752 | 3.260152 |
| 1.088152 | 1.250736 | 1.470791 | 1.747197 |
| 0.807052 | 1.446313 | 0.329526 | 2.248348 |
| 5.906846 | 5.768683 | 5.68657 | 6.189781 |
| 0.048194 | 0.241833 | 0.196782 | 0.095903 |
| 3.299082 | 2.553196 | 2.651185 | 2.057277 |
| 0 | 0.142916 | 0.040702 | 0 |
| 51.37596 | 40.55222 | 44.72016 | 47.01609 |
| 0 | 0.09326 | 0.424962 | 0.103554 |
| 0.099824 | 0 | 0 | 0 |
| 2.933774 | 2.1126 | 2.036403 | 2.154737 |
| 0 | 0 | 0 | 0 |
| 0.172054 | 0.277504 | 0 | 0.410848 |
| 13.32526 | 12.45536 | 13.10223 | 12.82975 |
| 29.21322 | 28.70848 | 28.17758 | 28.39553 |
| 0.245023 | 0.326191 | 0.200089 | 0.264683 |
| 0.474292 | 0.386353 | 0.457735 | 0.27456 |
| 0 | 0.031411 | 0 | 0 |
| 57.03047 | 75.17956 | 73.90701 | 75.18531 |
| 37.86474 | 31.82339 | 33.17949 | 31.79576 |
| 0.665294 | 0.817557 | 0.814935 | 1.021277 |
| 2.76035 | 11.13032 | 3.381221 | 6.59143 |
| 0.625704 | 0.605513 | 0.498186 | 0.859116 |
| 0 | 0 | 0 | 0 |
| 42.57419 | 23.03872 | 22.17849 | 23.26359 |
| 0.072073 | 0.077497 | 0.088284 | 0.057367 |
| 1.972621 | 1.631596 | 1.970224 | 1.884166 |
| 11.45225 | 13.18848 | 12.78304 | 11.56979 |
| 0 | 0 | 0 | 0 |
| 21.99758 | 23.21395 | 25.07787 | 25.19131 |
| 8.91981 | 7.898543 | 6.29565 | 7.004942 |
| 2.066104 | 2.05703 | 2.015284 | 2.649548 |
| 0.996112 | 1.717764 | 1.772689 | 1.7503 |
| 308.7992 | 362.0981 | 355.9007 | 357.4983 |
| 4.130137 | 1.614894 | 1.254324 | 1.589385 |

| | | | |
|---|---|---|---|
| 0 | 0 | 0 | 0 |
| 10.88082 | 11.16299 | 10.94378 | 11.85887 |
| 0.595201 | 0.639994 | 0 | 0 |
| 1.03868 | 1.037073 | 0.781558 | 1.098401 |
| 15.00212 | 10.87914 | 10.73528 | 10.49712 |
| 8.53722 | 7.369623 | 5.891521 | 6.029661 |
| 5.003659 | 4.389637 | 4.270459 | 4.680078 |
| 86.81286 | 86.51154 | 84.64336 | 87.60659 |
| 18.35479 | 15.41387 | 14.844 | 15.98606 |
| 1.96143 | 1.968438 | 2.402603 | 2.252632 |
| 17.70507 | 16.18446 | 15.95908 | 15.51529 |
| 0 | 0 | 0.097917 | 0 |
| 3.374365 | 1.727767 | 2.033867 | 2.43008 |
| 5.032741 | 5.711019 | 4.572363 | 5.543203 |
| 6.759497 | 6.111892 | 6.228723 | 6.548101 |
| 0.338904 | 0.255086 | 0.415132 | 0.323707 |
| 11.02428 | 10.79555 | 8.470089 | 10.98827 |
| 40.42109 | 24.77394 | 42.38294 | 26.06077 |
| 5.004234 | 4.76485 | 4.148456 | 4.868352 |
| 4.250496 | 3.588591 | 3.509591 | 3.621328 |
| 1.612359 | 1.918136 | 1.806931 | 1.474524 |
| 4.985149 | 5.04434 | 6.929192 | 5.563562 |
| 1.522287 | 1.371415 | 1.243125 | 1.473675 |
| 4.019447 | 2.950129 | 3.293549 | 3.226639 |
| 0.331654 | 0.152834 | 0.232144 | 0.169705 |
| 13.07282 | 10.59852 | 9.374482 | 10.84402 |
| 6.481073 | 6.542157 | 52.33144 | 4.737591 |
| 4.304275 | 1.157051 | 1.647629 | 2.569541 |
| 0.130455 | 0 | 0.159797 | 0 |
| 10.68508 | 8.968276 | 8.869627 | 10.00777 |
| 0.364874 | 0.239759 | 0.173811 | 0.169416 |
| 12.94616 | 13.19444 | 13.12516 | 13.5994 |
| 0.940822 | 0.550774 | 0.460974 | 0.499241 |
| 2.033537 | 1.827752 | 1.660619 | 1.718233 |
| 0 | 0 | 0 | 0 |
| 0.107607 | 0.115705 | 0.065905 | 0.128477 |
| 5.522291 | 4.561552 | 5.241282 | 4.846752 |
| 0 | 0 | 0 | 0 |
| 12.65699 | 6.851558 | 6.483043 | 6.83876 |
| 44.8425 | 24.74604 | 23.31486 | 23.68585 |
| 31.86365 | 32.31193 | 31.51134 | 30.96253 |
| 4.201416 | 1.325163 | 1.372378 | 1.872742 |
| 0 | 0 | 0 | 0 |
| 7.214552 | 6.267242 | 6.162841 | 6.25634 |
| 0 | 0 | 0.355647 | 0 |
| 66.69674 | 67.3241 | 63.88925 | 63.29671 |
| 0.320647 | 0.430972 | 0.294576 | 0.191418 |
| 32.12579 | 28.9706 | 28.88959 | 28.69207 |
| 19.24977 | 18.47481 | 19.08316 | 18.08618 |
| 1.754775 | 1.657354 | 1.65567 | 1.330678 |
| 0.019443 | 0.020906 | 0.095265 | 0.069642 |
| 3.416085 | 1.274365 | 2.476509 | 3.995391 |
| 12.08281 | 10.68511 | 11.38857 | 11.28033 |
| 0 | 0 | 0 | 0 |

| | | | |
|---|---|---|---|
| 94.02212 | 105.6898 | 96.86136 | 104.3545 |
| 6.122063 | 5.354003 | 5.432656 | 5.490191 |
| 2.235039 | 1.959164 | 1.428393 | 2.030396 |
| 0.031597 | 0 | 0.038703 | 0 |
| 7.221207 | 6.903458 | 6.867474 | 6.554995 |
| 16.41933 | 25.04912 | 24.09509 | 23.31108 |
| 0 | 0 | 0 | 0 |
| 0 | 0 | 0.048686 | 0 |
| 18.38639 | 16.52577 | 16.2466 | 17.56192 |
| 11.51513 | 12.35927 | 12.4683 | 12.25271 |
| 0 | 0 | 0 | 0 |
| 24.19888 | 23.35223 | 24.2713 | 25.8082 |
| 4.944812 | 3.99727 | 4.44472 | 4.119952 |
| 5.920439 | 4.1032 | 4.468113 | 4.958138 |
| 0 | 0 | 0 | 0 |
| 14.64604 | 12.60567 | 12.75128 | 13.64426 |
| 0.122959 | 0.165266 | 0.094135 | 0.091754 |
| 13.33387 | 15.59695 | 15.53111 | 15.42631 |
| 2.075276 | 1.673591 | 1.641744 | 1.583021 |
| 2.981131 | 0.970467 | 1.340059 | 1.306171 |
| 1.749991 | 1.881691 | 1.844499 | 1.554902 |
| 1.904642 | 2.047979 | 3.110723 | 1.516029 |
| 3.835629 | 2.749525 | 2.420365 | 2.3129 |
| 10.97383 | 11.04005 | 9.865239 | 10.45925 |
| 54.75274 | 49.05083 | 46.78673 | 46.48974 |
| 3.837342 | 3.88211 | 3.942316 | 3.842621 |
| 1.613364 | 1.484723 | 1.40652 | 1.110644 |
| 0.028343 | 0 | 0 | 0.03384 |
| 8.175809 | 5.951736 | 6.054465 | 6.224719 |
| 29.39403 | 26.5583 | 25.4062 | 27.02917 |
| 78.4505 | 54.63235 | 52.37647 | 51.83386 |
| 8.474782 | 7.233689 | 6.171504 | 7.44106 |
| 9.915469 | 7.531798 | 7.77935 | 7.210926 |
| 20.88304 | 18.28553 | 17.99776 | 17.59677 |
| 3.173221 | 3.450152 | 3.539519 | 3.238353 |
| 0 | 0 | 0 | 0 |
| 92.70225 | 77.29749 | 76.18285 | 81.22438 |
| 0.46335 | 0.117228 | 0.033386 | 0.162711 |
| 0.973284 | 1.127033 | 1.100492 | 1.072662 |
| 9.94933 | 9.39498 | 8.787218 | 8.238273 |
| 9.623494 | 8.605827 | 8.079187 | 8.059085 |
| 0.106762 | 0 | 0.104621 | 0.025494 |
| 8.73891 | 8.922798 | 8.635567 | 8.382116 |
| 1.546849 | 1.036524 | 1.070959 | 1.498899 |
| 6.337284 | 8.053156 | 7.233419 | 8.116671 |
| 4.781292 | 4.143136 | 4.065254 | 6.548117 |
| 3.546799 | 3.545477 | 3.720113 | 3.444736 |
| 0.151258 | 0.043371 | 0.098816 | 0.144475 |
| 4.136529 | 3.585222 | 3.480319 | 3.502059 |
| 0.023364 | 0.175857 | 0.143096 | 0.181321 |
| 0.297229 | 0.127839 | 0.21845 | 0.425851 |
| 60.53031 | 64.56908 | 61.02602 | 64.44248 |
| 0 | 0 | 0 | 0 |
| 0 | 0.119695 | 0.068178 | 0.09968 |

| | | | |
|---|---|---|---|
| 0.258783 | 0.278258 | 0.554732 | 0 |
| 0 | 0 | 0 | 0 |
| 3.571203 | 0.853325 | 0.972101 | 1.421277 |
| 0.08564 | 0.122781 | 0.104903 | 0.170417 |
| 0 | 0 | 0 | 0 |
| 2.304286 | 1.702236 | 1.960721 | 1.617116 |
| 0.038062 | 0 | 0 | 0.045445 |
| 0.443878 | 0.607452 | 0.197715 | 0.192716 |
| 3.283865 | 3.633347 | 3.439518 | 3.253098 |
| 3.096312 | 2.775142 | 2.998806 | 2.862367 |
| 0 | 0 | 0 | 0 |
| 0 | 0 | 0 | 0 |
| 3.437718 | 3.38184 | 3.01635 | 2.969181 |
| 13.27066 | 11.18849 | 11.68368 | 13.99896 |
| 0.862502 | 0.351777 | 0.218586 | 0.674685 |
| 2.583129 | 3.170911 | 2.892538 | 3.269434 |
| 0.360338 | 0.35978 | 0.378331 | 0.215112 |
| 0.118448 | 0 | 0.072545 | 0.07071 |
| 18.30421 | 13.03614 | 13.43225 | 12.99263 |
| 0 | 0 | 0 | 0 |
| 0.135658 | 0 | 0 | 0.161969 |
| 40.51825 | 39.5117 | 38.99996 | 43.77631 |
| 0 | 0 | 1.121655 | 0 |
| 0 | 0 | 0 | 0 |
| 0 | 0 | 0 | 0 |
| 1.491933 | 1.5269 | 1.012835 | 1.28768 |
| 3.189212 | 1.535473 | 1.78807 | 2.083845 |
| 71.89069 | 68.06014 | 65.72517 | 69.18127 |
| 0.661334 | 2.133312 | 1.620168 | 1.579197 |
| 105.6032 | 100.0384 | 99.59012 | 101.862 |
| 0 | 0 | 0 | 0.051217 |
| 0.46591 | 0.360701 | 0.182626 | 0.24476 |
| 10.22022 | 7.042035 | 7.302749 | 7.737545 |
| 5.163186 | 4.43369 | 3.601458 | 4.195336 |
| 0.885024 | 0.631278 | 0.751348 | 0.784658 |
| 0 | 0 | 0 | 0 |
| 0 | 0 | 0 | 0 |
| 0.430166 | 0.10674 | 0.040532 | 0.118522 |
| 0 | 0.550532 | 0 | 0.611302 |
| 0.554693 | 0.627829 | 0.500653 | 0.453136 |
| 0.162304 | 0.04363 | 0.024851 | 0.096892 |
| 0 | 0.017356 | 0.019772 | 0 |
| 0.15872 | 0.511995 | 0.583261 | 0 |
| 0.121999 | 0.170534 | 0 | 0.043698 |
| 0.236015 | 0.063444 | 0 | 0.070447 |
| 0.577526 | 0.256961 | 0.268334 | 0.237771 |
| 3.870811 | 3.841022 | 3.368018 | 3.713383 |
| 1.543417 | 1.0593 | 1.146409 | 1.254645 |
| 0 | 0 | 0 | 0.065572 |
| 0.828433 | 0.688328 | 0.645761 | 0.606951 |
| 0.036876 | 0.039651 | 0.011293 | 0.022014 |
| 1.383671 | 0.791674 | 1.275056 | 0.757812 |
| 3.112035 | 2.484017 | 2.666067 | 2.165539 |
| 9.823879 | 9.826972 | 9.243924 | 9.82765 |

| | | | |
|---|---|---|---|
| 8.011878 | 6.62428 | 7.100241 | 7.174325 |
| 0.019325 | 0 | 0 | 0.023073 |
| 3.853047 | 2.608565 | 2.330712 | 2.356963 |
| 0.193169 | 0.259632 | 0 | 0.057658 |
| 28.83115 | 24.75956 | 24.729 | 26.76913 |
| 0 | 0 | 0 | 0 |
| 2.10127 | 1.451497 | 1.653534 | 1.39885 |
| 1.764048 | 1.668618 | 1.88463 | 1.916151 |
| 0.369117 | 0 | 0 | 0 |
| 5.992618 | 4.929746 | 4.421991 | 4.504123 |
| 0.015405 | 0.016564 | 0 | 0.036785 |
| 9.567471 | 9.024436 | 8.799999 | 9.431142 |
| 1.045686 | 0.783051 | 0.777681 | 0.758015 |
| 0.073049 | 0.170185 | 0.208787 | 0.072681 |
| 0 | 0 | 0 | 0 |
| 21.18352 | 20.89158 | 20.63391 | 20.63969 |
| 2.490253 | 1.878682 | 2.459977 | 1.606505 |
| 0.034789 | 0.010688 | 0.006088 | 0.011867 |
| 12.26143 | 13.88951 | 14.1128 | 12.73175 |
| 0.301064 | 0.316364 | 0.226297 | 0.392133 |
| 2.480002 | 1.866648 | 2.430252 | 1.480497 |
| 1.356767 | 0.555761 | 0.553979 | 0.925662 |
| 0.132267 | 0.426662 | 0.324034 | 0 |
| 2.317402 | 1.864934 | 1.553219 | 1.861973 |
| 8.932404 | 13.83974 | 12.57844 | 13.60395 |
| 22.84165 | 20.12083 | 23.24433 | 22.76141 |
| 100.5254 | 57.55178 | 59.54179 | 58.43107 |
| 0.114187 | 0 | 0 | 0 |
| 0.547761 | 0.378632 | 0.623039 | 0.326999 |
| 3.84711 | 3.304684 | 3.251306 | 3.541165 |
| 0.010537 | 0 | 0 | 0.025161 |
| 5.500309 | 3.696403 | 3.584174 | 3.665349 |
| 0 | 0 | 0 | 0 |
| 0.095615 | 0.10281 | 0 | 0 |
| 0 | 0 | 0 | 0 |
| 1.74205 | 1.373645 | 1.158392 | 1.644126 |
| 0.164952 | 0.118244 | 0.067351 | 0 |
| 0.533613 | 0.478142 | 0.326818 | 0.445974 |
| 2.95442 | 2.392885 | 2.725956 | 2.473778 |
| 0 | 0 | 0 | 0 |
| 44.73894 | 42.87764 | 42.53749 | 39.36637 |
| 0 | 0.016743 | 0 | 0.037182 |
| 58.53872 | 61.29257 | 56.23552 | 62.76034 |
| 0.154097 | 0.497082 | 0.188757 | 0.183984 |
| 29.13456 | 27.16176 | 26.69159 | 29.67819 |
| 61.4178 | 41.92422 | 48.1824 | 53.14341 |
| 0.111775 | 0 | 0.136916 | 0 |
| 1.042102 | 1.07743 | 0.343672 | 0.909235 |
| 42.8496 | 44.8776 | 43.92237 | 41.2517 |
| 4.060614 | 3.447003 | 2.513152 | 2.704764 |
| 0.018993 | 0 | 0 | 0 |
| 39.94726 | 36.95211 | 37.18564 | 38.55688 |
| 36.18727 | 37.26545 | 35.21145 | 35.42814 |
| 1.497416 | 1.638688 | 1.6063 | 1.85131 |

| | | | |
|---|---|---|---|
| 0.01174 | 0 | 0 | 0 |
| 1.374896 | 1.010849 | 1.065185 | 0.911975 |
| 14.88318 | 16.68482 | 17.23693 | 17.37621 |
| 1.885535 | 2.054467 | 1.940096 | 1.951067 |
| 591.8715 | 617.648 | 629.1059 | 656.3169 |
| 13.26057 | 10.65074 | 10.6977 | 10.25466 |
| 0.47616 | 0.511995 | 0.29163 | 0.094752 |
| 2.403695 | 1.815366 | 1.647431 | 1.60577 |
| 41.13225 | 36.00239 | 35.12592 | 38.29145 |
| 0 | 0.731421 | 0.833229 | 0 |
| 19.4924 | 18.71668 | 19.96295 | 19.97471 |
| 0 | 0 | 0.120758 | 0 |
| 56.83783 | 48.10281 | 47.85259 | 46.67811 |
| 4.325399 | 3.113749 | 3.44239 | 3.296984 |
| 35.80009 | 36.04967 | 32.47746 | 33.2619 |
| 8.423695 | 8.437904 | 8.200405 | 7.19902 |
| 0 | 0 | 0.100217 | 0.048841 |
| 0.810283 | 0.563758 | 0.467076 | 0.56908 |
| 0.059028 | 0.042314 | 0.120508 | 0.140953 |
| 0 | 0.009986 | 0 | 0 |
| 10.80806 | 10.97009 | 10.05622 | 9.116729 |
| 0.16152 | 0.06947 | 0.098925 | 0.096423 |
| 0 | 0 | 0 | 0 |
| 0.92855 | 1.158179 | 0.59145 | 0.886912 |
| 4.85137 | 4.218725 | 3.644607 | 3.576525 |
| 10.89404 | 10.39608 | 10.4392 | 10.37844 |
| 0 | 0 | 0 | 0 |
| 0.532767 | 0.698175 | 0.754568 | 0.854754 |
| 0 | 0 | 0 | 0 |
| 0.035138 | 0.028337 | 0.037661 | 0.020976 |
| 1.790709 | 1.408567 | 1.251316 | 1.678843 |
| 0.166231 | 0.099301 | 0.113123 | 0.198472 |
| 0.712878 | 0.315629 | 0.590709 | 0.150201 |
| 0 | 0 | 0.138872 | 0.13536 |
| 4.727188 | 3.504727 | 4.178546 | 3.238965 |
| 0.101096 | 0 | 0 | 0 |
| 16.6056 | 14.47476 | 13.0761 | 12.8478 |
| 10.18384 | 8.885557 | 9.576338 | 9.252289 |
| 14.48321 | 11.73322 | 11.14444 | 11.74246 |
| 0.611374 | 0.550368 | 0.557311 | 0.543217 |
| 6.566028 | 4.989893 | 4.98901 | 4.214466 |
| 0.400135 | 0 | 0 | 0 |
| 1.192388 | 0.598325 | 0.778979 | 0.85419 |
| 8.947387 | 7.922029 | 8.518318 | 8.867007 |
| 0 | 0 | 0 | 0 |
| 0 | 0 | 0 | 0 |
| 148.4236 | 168.6206 | 166.5001 | 170.9869 |
| 0.404898 | 0.391833 | 0.099194 | 0.773484 |
| 3.478042 | 2.582235 | 3.347409 | 2.471786 |
| 2.449094 | 3.798181 | 3.346104 | 2.474231 |
| 0 | 0 | 0 | 0.081158 |
| 15.98478 | 14.57321 | 15.09951 | 15.27305 |
| 48.42839 | 43.87568 | 39.8791 | 41.5565 |
| 9.467314 | 8.677242 | 9.842255 | 10.1773 |

| | | | |
|---|---|---|---|
| 1.881644 | 1.433136 | 1.504569 | 1.372913 |
| 4.507344 | 4.044443 | 3.680771 | 4.127098 |
| 0 | 0 | 0.037533 | 0 |
| 28.47626 | 18.99903 | 25.53195 | 19.98149 |
| 35.32064 | 43.89114 | 45.65055 | 43.53872 |
| 14.6785 | 11.83737 | 11.76122 | 11.97425 |
| 1.105564 | 0.792511 | 0.884013 | 1.154988 |
| 16.46694 | 14.44738 | 14.35465 | 14.85607 |
| 0 | 0 | 0 | 0 |
| 0 | 0 | 0 | 0 |
| 12.6025 | 12.01686 | 12.92494 | 11.07212 |
| 17.70863 | 16.82557 | 16.4679 | 17.52503 |
| 2.72565 | 2.072591 | 1.77081 | 1.744009 |
| 12.35737 | 5.418009 | 5.442285 | 5.459313 |
| 54.05295 | 56.42486 | 55.84577 | 58.12349 |
| 3.192039 | 3.527076 | 3.002712 | 3.07417 |
| 9.244141 | 7.6893 | 7.264051 | 7.496847 |
| 5.843222 | 5.330495 | 5.539453 | 5.065387 |
| 15.5122 | 15.60488 | 16.60162 | 15.99086 |
| 3.800135 | 2.714623 | 3.049376 | 2.867236 |
| 0.161753 | 0.130445 | 0.198135 | 0.048281 |
| 0.414053 | 0 | 0 | 0 |
| 5.455149 | 4.834033 | 4.331642 | 4.713044 |
| 51.60643 | 38.69873 | 38.49238 | 37.93127 |
| 22.43688 | 27.11531 | 26.40294 | 27.40667 |
| 31.5379 | 19.94785 | 15.90711 | 11.07489 |
| 0.208294 | 0.470336 | 0.204116 | 0.348169 |
| 0.330667 | 0 | 0.405042 | 0 |
| 4.627096 | 4.00468 | 4.444936 | 4.068439 |
| 0 | 1.5515 | 0.441864 | 0 |
| 0.021041 | 0.022625 | 0.025774 | 0.100488 |
| 2.846611 | 2.597075 | 1.584947 | 1.750849 |
| 30.10667 | 26.63269 | 25.78357 | 27.50458 |
| 1.020583 | 0.737589 | 0.717292 | 1.098668 |
| 13.04026 | 13.93028 | 13.42384 | 14.09866 |
| 18.37992 | 12.42066 | 13.99926 | 12.32709 |
| 12.89548 | 13.23318 | 14.22893 | 14.72446 |
| 0.351151 | 0.302062 | 0.200729 | 0.503107 |
| 9.519569 | 8.357103 | 8.026522 | 7.714884 |
| 9.523209 | 7.081322 | 7.89288 | 6.675053 |
| 0.284445 | 0.12234 | 0.139369 | 0.067922 |
| 51.53464 | 46.15438 | 46.67348 | 46.23191 |
| 0 | 0.024398 | 0.013897 | 0.081274 |
| 9.817741 | 7.917446 | 4.810397 | 6.447031 |
| 0.688327 | 0.505029 | 0.595164 | 0.560776 |
| 0.034884 | 0 | 0 | 0 |
| 8.381032 | 7.019367 | 6.890646 | 7.306346 |
| 0.023014 | 0 | 0 | 0 |
| 26.31055 | 22.6564 | 21.17782 | 23.3867 |
| 7.364346 | 6.906145 | 6.920041 | 6.745044 |
| 10.89915 | 11.27106 | 10.63353 | 11.30052 |
| 4.082158 | 3.623439 | 4.127794 | 3.794434 |
| 35.78984 | 7.02738 | 15.62986 | 1.114727 |
| 0 | 0 | 0.186942 | 0 |

| | | | |
|---|---|---|---|
| 12.15729 | 11.26488 | 11.90213 | 12.12347 |
| 4.11346 | 2.559318 | 2.317493 | 2.288034 |
| 2.976003 | 3.701924 | 2.358774 | 2.299125 |
| 59.16603 | 56.19572 | 59.41019 | 57.35303 |
| 0.732555 | 0 | 0.448662 | 1.749264 |
| 1.370246 | 1.166415 | 5.338396 | 1.817781 |
| 38.36338 | 32.76767 | 30.96584 | 32.65061 |
| 31.05065 | 27.85675 | 28.58394 | 29.57702 |
| 0.239277 | 0.418086 | 0.183185 | 0.107131 |
| 0.096633 | 0.051953 | 0.118368 | 0.086531 |
| 0 | 0 | 0 | 0 |
| 12.63936 | 9.673514 | 9.376792 | 10.22702 |
| 25.45051 | 28.5318 | 31.09681 | 28.90152 |
| 0.703278 | 0.422585 | 0.481405 | 0.543321 |
| 0 | 0 | 0 | 0 |
| 63.50355 | 61.26456 | 59.15116 | 61.96769 |
| 1.23868 | 0.880942 | 1.039403 | 0.873378 |
| 31.06521 | 35.51317 | 30.20619 | 30.85697 |
| 0.296058 | 0.141484 | 0.14103 | 0.137464 |
| 40.23274 | 34.37968 | 35.17958 | 31.37642 |
| 0.144291 | 0.15515 | 0.201995 | 0.123054 |
| 8.043614 | 7.781165 | 7.381377 | 7.837099 |
| 0.454178 | 0.429755 | 0.378307 | 0.412122 |
| 0.529067 | 0 | 0.648067 | 0.631679 |
| 1.476467 | 1.587581 | 1.35642 | 1.762824 |
| 0.145615 | 0 | 0 | 0 |
| 0 | 0.035044 | 0.239532 | 0.233475 |
| 0 | 0 | 0 | 0 |
| 0.32173 | 0.212888 | 0.12126 | 0.265935 |
| 0.018385 | 0 | 0.02252 | 0 |
| 11.88908 | 15.93158 | 15.95368 | 12.98231 |
| 4.36512 | 3.483868 | 3.458617 | 3.965304 |
| 0.109086 | 0 | 0 | 0 |
| 0.24442 | 0.229962 | 0.205835 | 0.218868 |
| 12.12285 | 10.05681 | 10.03191 | 8.790372 |
| 3.599072 | 3.253479 | 2.809014 | 2.966144 |
| 3.393537 | 3.092586 | 2.889274 | 3.070576 |
| 24.96464 | 20.58815 | 20.17145 | 22.20447 |
| 0 | 0 | 0 | 0 |
| 0 | 0.179647 | 0 | 0 |
| 0 | 0 | 0.018284 | 0 |
| 24.08258 | 25.47322 | 25.63176 | 25.26455 |
| 0 | 1.462842 | 0.833229 | 0 |
| 1.037245 | 0.601219 | 0.506234 | 0.561158 |
| 0 | 0.054994 | 0 | 0 |
| 3.300579 | 1.588351 | 1.413622 | 1.460547 |
| 1.20547 | 0.756111 | 0.984406 | 1.139421 |
| 0 | 0 | 0 | 0 |
| 0 | 0 | 0 | 0 |
| 0.930228 | 0.81269 | 0.56973 | 0.624737 |
| 9.122668 | 6.668185 | 6.894233 | 6.950757 |
| 3.771938 | 3.192439 | 2.836248 | 2.809113 |
| 0 | 0 | 0.08285 | 0 |
| 0 | 0 | 1.666459 | 0 |

| | | | |
|---|---|---|---|
| 5.658301 | 6.135689 | 5.168875 | 6.1832 |
| 0 | 0 | 0 | 0 |
| 2.963654 | 2.72639 | 2.904202 | 2.752127 |
| 34.85524 | 34.89092 | 35.77272 | 33.99747 |
| 6.594131 | 5.663014 | 6.103362 | 5.786839 |
| 4.898687 | 3.771681 | 3.457091 | 3.465943 |
| 0.073823 | 0 | 0 | 0 |
| 14.21344 | 13.20308 | 12.82337 | 13.67242 |
| 0 | 1.29619 | 0.738305 | 0 |
| 0 | 0 | 0.972101 | 0 |
| 1.700573 | 0 | 0 | 0 |
| 2.11149 | 2.081194 | 1.999682 | 1.598973 |
| 1.825215 | 0.992071 | 0.663355 | 0.790264 |
| 24.24005 | 24.25635 | 24.20002 | 24.08992 |
| 0.040126 | 0.014382 | 0 | 0.079847 |
| 0 | 0 | 2.287296 | 0 |
| 0.988724 | 0.749025 | 1.018435 | 0.965851 |
| 16.55436 | 13.8765 | 15.40825 | 13.38923 |
| 12.0269 | 9.337828 | 9.152334 | 7.668832 |
| 6.159776 | 4.99379 | 5.629004 | 4.902969 |
| 5.936861 | 6.774487 | 7.717443 | 8.679555 |
| 0.64346 | 1.38377 | 0 | 0.768258 |
| 0 | 0 | 0 | 0 |
| 0.128 | 0 | 0 | 0 |
| 16.94093 | 16.28765 | 16.66828 | 15.08808 |
| 10.8615 | 7.591288 | 7.246211 | 8.290301 |
| 3.406323 | 3.078204 | 3.462278 | 3.89391 |
| 0.380624 | 0.327415 | 0.512859 | 0.272667 |
| 12.11357 | 8.4655 | 7.549973 | 8.418749 |
| 0.062243 | 0.200782 | 0.304973 | 0.297261 |
| 0.52537 | 0.489587 | 0.386123 | 0.564538 |
| 2.323058 | 2.169591 | 2.081331 | 2.092095 |
| 0.109378 | 0.156813 | 0.08932 | 0.130592 |
| 0 | 0 | 0 | 0 |
| 4.472225 | 2.545831 | 3.544678 | 3.140944 |
| 0 | 0 | 0 | 0 |
| 14.1383 | 15.51738 | 13.68419 | 15.43726 |
| 0 | 0 | 0.490135 | 0 |
| 0.173654 | 0 | 0.021271 | 0.041467 |
| 205.2851 | 178.9318 | 173.8746 | 171.36 |
| 0.563569 | 0.570335 | 0.39254 | 0.435388 |
| 5.25388 | 5.150806 | 4.542781 | 4.770536 |
| 0 | 0 | 0 | 0.064239 |
| 2.759946 | 0.299763 | 0.136595 | 3.794511 |
| 3.727947 | 4.190705 | 4.22051 | 4.450975 |
| 2.041417 | 1.042648 | 1.562863 | 1.035872 |
| 0 | 0.017996 | 0 | 0.019983 |
| 12.70067 | 9.170057 | 10.05331 | 10.29177 |
| 0 | 0 | 0 | 0 |
| 17.4389 | 18.50979 | 18.23672 | 18.59919 |
| 0 | 0 | 0 | 0 |
| 76.35275 | 64.31374 | 63.54471 | 65.1294 |
| 0.050709 | 0.054526 | 0 | 0 |
| 25.26508 | 22.17732 | 21.83328 | 19.92997 |

| | | | |
|---|---|---|---|
| 0.039287 | 0 | 0 | 0 |
| 0 | 0 | 0 | 0 |
| 5.942102 | 4.515096 | 4.658321 | 3.783766 |
| 0 | 0.082847 | 0 | 0 |
| 10.05617 | 9.138995 | 9.277194 | 9.611936 |
| 5.973969 | 3.994478 | 3.935549 | 3.296584 |
| 0.824015 | 0.950674 | 0.857736 | 0.975386 |
| 2.526253 | 1.509095 | 2.031722 | 2.163144 |
| 0.118891 | 0.255678 | 0.364083 | 0.354876 |
| 11.278 | 10.39435 | 9.820083 | 10.05845 |
| 3.119889 | 1.920826 | 2.24983 | 2.087794 |
| 0 | 0.098083 | 0.167604 | 0.054455 |
| 0 | 0 | 0 | 0 |
| 3.73509 | 3.824935 | 3.268002 | 3.792095 |
| 25.33256 | 18.66057 | 19.76964 | 19.24233 |
| 0.172335 | 0.074122 | 0.168877 | 0.144031 |
| 5.705148 | 3.408056 | 3.07608 | 4.133566 |
| 3.298744 | 3.502099 | 3.273488 | 3.273798 |
| 0.025355 | 0 | 0 | 0.030272 |
| 4.414733 | 4.430507 | 3.888404 | 3.112377 |
| 3.804557 | 2.642554 | 2.691972 | 3.329245 |
| 4.461069 | 4.039407 | 3.451246 | 3.476102 |
| 4.33642 | 3.827767 | 4.059185 | 3.791296 |
| 0.765186 | 0.69286 | 0.616641 | 0.753313 |
| 24.43827 | 24.23055 | 24.20012 | 26.72093 |
| 0.033699 | 0 | 0 | 0 |
| 0 | 0 | 0 | 0 |
| 5.071674 | 4.006017 | 3.915884 | 3.845556 |
| 2.497345 | 2.372004 | 2.651185 | 2.88231 |
| 1.335029 | 0 | 0 | 0 |
| 2.897149 | 2.445249 | 2.384939 | 2.510598 |
| 8.728309 | 5.125535 | 5.23439 | 5.102021 |
| 0.088015 | 0.094639 | 0 | 0 |
| 10.29263 | 11.3575 | 11.28491 | 11.96653 |
| 0 | 0 | 0 | 0 |
| 0.750396 | 0.44011 | 0.584932 | 0.57014 |
| 0.047916 | 0.103043 | 0.146732 | 0.071511 |
| 5.752383 | 4.240341 | 4.511187 | 4.513844 |
| 12.25299 | 15.97691 | 16.49089 | 14.92573 |
| 0 | 0 | 0 | 0 |
| 0.016643 | 0.035791 | 0.040773 | 0 |
| 0.081815 | 0.043986 | 0.025054 | 0 |
| 0.12116 | 0 | 0.296825 | 0 |
| 0.793601 | 0.426662 | 0 | 0.473759 |
| 0 | 0.68266 | 0 | 1.137022 |
| 0 | 0.316046 | 0 | 0 |
| 11.21369 | 10.36671 | 10.28474 | 9.678208 |
| 0.039507 | 0.03186 | 0 | 0.011792 |
| 5.321921 | 4.745432 | 5.29996 | 5.243422 |
| 38.35066 | 35.69237 | 35.42987 | 35.20727 |
| 0.240485 | 0 | 0 | 0 |
| 0.06474 | 0.034806 | 0 | 0.92755 |
| 0.158324 | 0.08512 | 0.096968 | 0 |
| 9874.035 | 9171.623 | 8806.397 | 8838.057 |

| | | | |
|---|---|---|---|
| 0 | 0 | 0 | 0 |
| 2.926342 | 2.646453 | 2.753693 | 2.267565 |
| 3.294192 | 1.36854 | 1.421468 | 2.055936 |
| 2.841437 | 2.267833 | 2.045269 | 1.993548 |
| 0.186243 | 0.200259 | 0.190111 | 0.148243 |
| 2.759061 | 2.564759 | 2.202218 | 1.678967 |
| 0.101419 | 0.054526 | 0.031058 | 0.151361 |
| 0.042782 | 0.092003 | 0 | 0 |
| 0.783674 | 0.640415 | 0.345579 | 0.44912 |
| 0.89002 | 0.638 | 0.181701 | 0 |
| 3.2173 | 0.345942 | 2.36457 | 1.152387 |
| 7.29127 | 6.446213 | 7.914085 | 7.351225 |
| 0.558107 | 0.512288 | 0.600268 | 0.438816 |
| 0.049964 | 0 | 0 | 0 |
| 3.125187 | 3.413299 | 3.134837 | 3.084943 |
| 0.100426 | 0.215966 | 0.070294 | 0.154161 |
| 1.524652 | 1.07191 | 1.077452 | 1.050205 |
| 0.086261 | 0 | 0 | 0 |
| 3.462308 | 2.882223 | 2.71337 | 2.666978 |
| 0.218824 | 0.15294 | 0.107217 | 0.117569 |
| 422.9368 | 516.268 | 517.7072 | 506.7403 |
| 0 | 0.077575 | 0 | 0.172276 |
| 15.8252 | 13.40819 | 14.73923 | 14.90687 |
| 0 | 0 | 0 | 1.160226 |
| 0.098788 | 0 | 0 | 0 |
| 0.087812 | 0.09442 | 0.080672 | 0.026211 |
| 5.023595 | 4.567476 | 4.221785 | 3.902438 |
| 0 | 0 | 0 | 0 |
| 0 | 0.054179 | 0 | 0 |
| 0.029612 | 0 | 0 | 0.035355 |
| 0 | 0 | 0 | 0.812158 |
| 19.82648 | 17.24124 | 18.51305 | 19.92052 |
| 1.068205 | 1.148595 | 0.779795 | 0.914667 |
| 1.821242 | 1.863764 | 1.953946 | 1.484637 |
| 1.085708 | 0.722685 | 0.781058 | 0.740731 |
| 0.012046 | 0.025904 | 0.05902 | 0.057527 |
| 0 | 0 | 0 | 0.164786 |
| 14.20354 | 13.78821 | 13.03685 | 14.01639 |
| 0 | 0 | 0 | 0 |
| 49.29944 | 53.16472 | 47.95698 | 48.69669 |
| 3.917147 | 3.632035 | 3.633427 | 2.880681 |
| 0 | 0 | 0.167604 | 0.163365 |
| 2.182402 | 3.946627 | 4.009917 | 3.671633 |
| 2.304649 | 1.896277 | 1.546524 | 1.914178 |
| 1.339201 | 1.679983 | 2.187227 | 1.243618 |
| 11.04472 | 8.968534 | 9.571312 | 9.055683 |
| 6.107989 | 5.39234 | 5.60275 | 5.58989 |
| 0.553675 | 0.330746 | 0.150713 | 0.33053 |
| 0 | 0 | 0 | 0 |
| 35.41386 | 33.68906 | 32.25352 | 27.22061 |
| 0 | 0 | 0 | 0 |
| 0.153353 | 0.082447 | 0.093923 | 0.091548 |
| 0 | 0 | 0 | 0 |
| 17.77962 | 17.74358 | 18.57995 | 17.04869 |

| | | | |
|---|---|---|---|
| 2.676953 | 2.051153 | 2.63358 | 2.239817 |
| 342.8587 | 249.0157 | 241.7786 | 261.1366 |
| 4.872475 | 3.858785 | 5.146417 | 4.006051 |
| 14.04223 | 13.37 | 13.533 | 12.66116 |
| 0.023868 | 0 | 0 | 0 |
| 0.063467 | 0.119426 | 0.116613 | 0.151552 |
| 24.47595 | 22.61523 | 22.94498 | 21.12422 |
| 0.410041 | 0.629857 | 0.672683 | 0.61196 |
| 0.050548 | 0 | 0.061917 | 0.030176 |
| 0.178806 | 0.057679 | 0.219024 | 0.106743 |
| 5.616669 | 5.09731 | 4.725318 | 4.938284 |
| 2.275415 | 1.983079 | 2.171091 | 2.344964 |
| 0.330995 | 0.762654 | 0.540592 | 1.129118 |
| 0 | 0 | 0 | 0 |
| 2.753032 | 2.434734 | 2.314685 | 2.275599 |
| 4.12493 | 13.4025 | 4.100765 | 9.885594 |
| 15.06322 | 12.44892 | 12.91441 | 13.24957 |
| 0 | 0 | 0 | 0 |
| 6.309777 | 5.292479 | 4.887065 | 4.797997 |
| 9.704761 | 9.370305 | 9.562643 | 8.434061 |
| 16.10691 | 14.75729 | 14.85896 | 14.44314 |
| 6.240132 | 5.325372 | 5.278089 | 5.367754 |
| 0.643003 | 0.436669 | 0.207271 | 0.404059 |
| 0.054522 | 0.136793 | 0.445237 | 0.065097 |
| 8.620015 | 8.021964 | 8.503158 | 8.197049 |
| 0 | 0 | 0 | 0 |
| 4.264592 | 3.843755 | 3.226466 | 3.519264 |
| 0 | 0 | 0 | 0 |
| 1.767063 | 1.458965 | 1.507433 | 1.582336 |
| 0.433662 | 0.205171 | 0.297474 | 0.393505 |
| 0 | 0 | 0 | 0 |
| 20.1696 | 17.22276 | 16.8862 | 16.27946 |
| 0 | 0 | 0 | 0 |
| 0.026978 | 0 | 0 | 0.016105 |
| 4.437217 | 3.108476 | 2.594099 | 3.23086 |
| 16.09414 | 12.20276 | 12.24402 | 11.98261 |
| 13.9042 | 11.04172 | 10.63761 | 11.85248 |
| 0.394881 | 0.300757 | 0.362774 | 0.392889 |
| 19.34603 | 17.07804 | 16.87036 | 15.7129 |
| 2.594464 | 1.312807 | 1.308597 | 0.911075 |
| 0.469124 | 0.252214 | 0.574641 | 0.560109 |
| 0 | 0 | 0 | 0 |
| 34.80897 | 37.89939 | 38.88404 | 39.03339 |
| 0.477354 | 0.898237 | 0.292361 | 0.427452 |
| 0 | 0 | 0 | 0 |
| 20.44635 | 18.3209 | 13.42515 | 16.8244 |
| 1.156933 | 0.710857 | 0.708578 | 0.710392 |
| 2.732068 | 1.678672 | 0.478082 | 1.397978 |
| 1.102542 | 0.952647 | 0.964665 | 1.057804 |
| 7.220466 | 4.578195 | 4.541783 | 4.87174 |
| 0.029502 | 0 | 0 | 0.035224 |
| 1.120378 | 0.919372 | 0.866765 | 0.633634 |
| 0.455898 | 0.544675 | 0.248196 | 0.423359 |
| 0.976739 | 0.711104 | 0.585753 | 0.947518 |

| | | | |
|---|---|---|---|
| 8.966034 | 7.781496 | 8.629276 | 9.596249 |
| 1.488001 | 0.99999 | 1.253099 | 0.814273 |
| 15.04734 | 16.43783 | 16.89398 | 17.45873 |
| 0 | 0.042809 | 0.097535 | 0.047534 |
| 8.23464 | 6.312827 | 6.397654 | 6.254075 |
| 1.550853 | 0.964062 | 1.869996 | 1.822707 |
| 0.051035 | 0.109752 | 0.062515 | 0.060934 |
| 11.25991 | 9.850001 | 10.63662 | 10.2537 |
| 0.138529 | 0.135413 | 0.293096 | 0.105252 |
| 0.098754 | 0.070791 | 0.080644 | 0.078605 |
| 0 | 0 | 0 | 0.032083 |
| 2.67515 | 2.334403 | 2.161331 | 2.300838 |
| 0.109968 | 0.157658 | 0 | 0.043765 |
| 7.323482 | 6.57661 | 6.391222 | 6.30967 |
| 0.596132 | 0.160249 | 0.456385 | 0.444844 |
| 12.60978 | 9.106628 | 10.60474 | 8.988314 |
| 27.7283 | 22.53117 | 21.57895 | 22.58952 |
| 11.06731 | 9.405656 | 9.829711 | 8.990827 |
| 5.02491 | 4.711477 | 4.727144 | 4.703594 |
| 0 | 0 | 0 | 0 |
| 5.38143 | 4.39148 | 4.061048 | 3.872299 |
| 0.303072 | 0.289672 | 0.288743 | 0.281441 |
| 0 | 0 | 0.555486 | 0 |
| 0 | 0 | 0 | 0 |
| 0.062859 | 0 | 0 | 0 |
| 22.88682 | 19.85466 | 19.96025 | 20.97087 |
| 0.157019 | 0 | 0 | 0.093736 |
| 1.138232 | 0.99077 | 0.846508 | 1.164849 |
| 0 | 0 | 0 | 0 |
| 0.342561 | 0 | 0 | 0 |
| 10.80283 | 6.395614 | 7.049537 | 8.71384 |
| 0.865531 | 2.065318 | 0.885929 | 0.707807 |
| 0.147761 | 0.218462 | 0.113123 | 0 |
| 11.0044 | 9.995625 | 10.46307 | 9.200329 |
| 0.122722 | 0.021993 | 0.050108 | 0.146523 |
| 0.366277 | 0 | 0.897324 | 1.311948 |
| 37.79336 | 33.10256 | 35.9494 | 32.17986 |
| 1.471799 | 0.233788 | 0.266329 | 0.379407 |
| 2.322734 | 2.289408 | 2.13388 | 2.773224 |
| 31.7736 | 24.22628 | 23.70854 | 23.95625 |
| 2.068499 | 1.700835 | 1.987259 | 1.840154 |
| 14.60492 | 12.8767 | 11.83326 | 13.41085 |
| 75.51052 | 81.06585 | 82.33841 | 81.67041 |
| 1.273837 | 1.232732 | 1.123455 | 1.064627 |
| 0 | 0 | 0 | 0.046965 |
| 3.840569 | 3.602415 | 3.446085 | 3.581939 |
| 9.37726 | 9.200214 | 8.603652 | 7.667273 |
| 0.393521 | 0.235076 | 0.294576 | 0.208819 |
| 2.540214 | 2.034601 | 2.22255 | 2.38298 |
| 0.51292 | 0.582161 | 0.523573 | 0.442289 |
| 0.566858 | 0 | 0 | 0 |
| 0.152616 | 0.164101 | 0.186942 | 0 |
| 0.723932 | 0.803523 | 0.858157 | 0.780692 |
| 9.542618 | 9.808597 | 7.924736 | 9.732659 |

| | | | |
|---|---|---|---|
| 12.92065 | 11.72223 | 10.73963 | 11.31168 |
| 1.338156 | 0.719431 | 0.54638 | 0.798844 |
| 4.149116 | 4.461366 | 5.001681 | 4.324772 |
| 11.11707 | 12.49096 | 12.24052 | 11.06598 |
| 11.92051 | 10.92571 | 12.95835 | 10.97633 |
| 0 | 0.036158 | 0 | 0 |
| 11.35659 | 8.420181 | 10.50142 | 9.526878 |
| 0.246078 | 0.132298 | 0.113035 | 0 |
| 0.108712 | 0 | 0 | 0 |
| 3.88171 | 3.770126 | 3.130306 | 3.774166 |
| 0.145615 | 0.313147 | 0 | 0.173857 |
| 19.2382 | 14.95064 | 16.48106 | 14.56161 |
| 28.67843 | 26.21258 | 26.27529 | 29.10602 |
| 40.67121 | 40.19138 | 41.80116 | 39.14767 |
| 27.91702 | 18.19271 | 21.61321 | 21.06665 |
| 6.75669 | 5.873163 | 5.387286 | 5.716869 |
| 0.898416 | 0 | 0.183415 | 0.178777 |
| 0 | 0.051664 | 0 | 0 |
| 0.154097 | 0 | 0 | 0 |
| 1.240001 | 1.066656 | 0.911345 | 1.332447 |
| 0 | 0.517167 | 1.178304 | 0.574253 |
| 0.523364 | 0.378774 | 0.295884 | 0.312435 |
| 4.582389 | 4.621741 | 4.283432 | 4.201205 |
| 1.435145 | 1.763599 | 1.569593 | 1.713488 |
| 0.031417 | 0.047294 | 0.061574 | 0.060017 |
| 24.31574 | 22.84058 | 21.9185 | 21.06931 |
| 18.77321 | 18.17162 | 18.07152 | 17.98731 |
| 2.093013 | 2.813159 | 1.922837 | 1.249474 |
| 0.246396 | 0.397409 | 0.301817 | 0.147092 |
| 0 | 0 | 0 | 0 |
| 1.816566 | 1.213398 | 1.786868 | 1.741681 |
| 9.180924 | 6.105414 | 6.641765 | 6.244644 |
| 0.717691 | 0.415532 | 0.642432 | 0.428443 |
| 0.835555 | 0.420787 | 0.621868 | 0.631398 |
| 2.011946 | 0.72112 | 0.821494 | 2.402159 |
| 0.526872 | 0.212446 | 0.161345 | 0.235897 |
| 11.57583 | 6.736775 | 4.531599 | 6.483019 |
| 8.035775 | 7.782322 | 6.643616 | 7.168651 |
| 0 | 0 | 0 | 0.43069 |
| 4.201416 | 3.346372 | 2.3826 | 2.693924 |
| 2.506108 | 3.368387 | 0.767448 | 2.742816 |
| 0.125968 | 0 | 0 | 0 |
| 10.75201 | 12.93327 | 13.5467 | 11.56772 |
| 1.718807 | 1.665173 | 1.33412 | 1.503567 |
| 16.03783 | 12.83731 | 14.89228 | 13.35157 |
| 14.32154 | 14.478 | 13.51588 | 13.29935 |
| 0 | 0 | 0.627162 | 0 |
| 0.451967 | 0.328752 | 0.521059 | 0.587239 |
| 3.710341 | 4.654499 | 5.302369 | 5.168281 |
| 40.68016 | 37.45691 | 36.49754 | 38.8622 |
| 0.055111 | 0 | 0 | 0 |
| 5.314611 | 3.938422 | 4.07608 | 3.544261 |
| 10.76165 | 9.36744 | 8.552745 | 8.412941 |
| 0 | 0 | 0.062314 | 0 |

| | | | |
|---|---|---|---|
| 0 | 0 | 0 | 0 |
| 0.040387 | 0.043426 | 0.098942 | 0.04822 |
| 0.436739 | 0.210087 | 0.253408 | 0.397944 |
| 0.026527 | 0.028523 | 0 | 0 |
| 0.115573 | 0 | 0 | 0 |
| 0.488092 | 0.524824 | 0.465014 | 0.712257 |
| 11.07495 | 8.073506 | 7.664397 | 9.338221 |
| 25.15924 | 25.57928 | 23.3584 | 22.99492 |
| 2.51794 | 2.04669 | 2.092909 | 1.825247 |
| 0.326373 | 0.203173 | 0.084165 | 0.041018 |
| 6.708735 | 5.238458 | 5.282803 | 5.530632 |
| 337.0205 | 354.6357 | 361.6732 | 365.0041 |
| 70.32445 | 76.82673 | 80.0652 | 74.5598 |
| 41.29533 | 27.48763 | 29.42111 | 29.43176 |
| 0.029338 | 0 | 0.035937 | 0 |
| 2.183061 | 1.905097 | 1.666459 | 1.624317 |
| 7.873095 | 11.19083 | 12.48429 | 10.59117 |
| 29.68727 | 25.69363 | 23.13791 | 27.03554 |
| 67.25034 | 70.52121 | 69.85906 | 68.72305 |
| 50.23493 | 44.98573 | 43.77106 | 44.16296 |
| 22.84283 | 17.89466 | 16.76695 | 18.05406 |
| 0.080365 | 0.086413 | 0 | 0.095951 |
| 13.25952 | 11.9897 | 11.57582 | 11.84942 |
| 15.82775 | 15.76247 | 17.27336 | 15.88533 |
| 0.151258 | 0.032528 | 0 | 0 |
| 6.55182 | 5.110026 | 5.990855 | 6.224974 |
| 0.152616 | 0.164101 | 0 | 0.36443 |
| 2.535001 | 2.187567 | 2.670064 | 2.486874 |
| 3.528462 | 3.265972 | 2.695742 | 3.648198 |
| 0 | 0 | 0 | 0 |
| 6.237097 | 6.431216 | 5.273862 | 6.307525 |
| 0 | 0 | 0.218245 | 0 |
| 0.231708 | 0 | 0.283825 | 0 |
| 0.11167 | 0.192118 | 0.246217 | 0.079997 |
| 0.111166 | 0.079688 | 0.04539 | 0.132726 |
| 19.46888 | 17.04825 | 18.67307 | 16.95505 |
| 0.276837 | 0 | 0 | 0 |
| 1.322668 | 1.346018 | 1.47551 | 1.268997 |
| 4.912138 | 3.450215 | 4.318652 | 3.689172 |
| 27.52946 | 31.83418 | 30.70503 | 31.72778 |
| 0.087315 | 0.156478 | 0.106955 | 0.03475 |
| 0 | 0.032799 | 0 | 0 |
| 5.032789 | 4.675992 | 3.920325 | 3.850356 |
| 18.85448 | 14.68512 | 12.58758 | 14.15548 |
| 18.95668 | 16.76497 | 18.48022 | 18.68251 |
| 0.208537 | 0.149488 | 0.34059 | 0.248983 |
| 9.663773 | 7.041862 | 7.435056 | 7.742885 |
| 11.68053 | 9.579879 | 9.891341 | 9.249864 |
| 1.293914 | 0.556516 | 0.845305 | 1.647858 |
| 0.406396 | 0.169937 | 0.055312 | 0.080869 |
| 2.24604 | 1.932056 | 1.650738 | 2.145324 |
| 0 | 0 | 0 | 0 |
| 0.376333 | 0.255571 | 0.242621 | 0.307431 |
| 0.490079 | 0.337255 | 0.552285 | 0.397887 |

| | | | |
|---|---|---|---|
| 0.030582 | 0.065767 | 0.037461 | 0.036513 |
| 0.529067 | 0.568883 | 1.296135 | 0 |
| 0 | 0 | 0 | 0 |
| 8.305593 | 7.608391 | 7.408052 | 7.341059 |
| 222.0315 | 166.6574 | 165.6507 | 174.1208 |
| 27.07534 | 23.06925 | 22.19413 | 22.96959 |
| 2.23014 | 1.830514 | 1.960189 | 1.422802 |
| 18.15618 | 18.20006 | 18.50938 | 16.15326 |
| 1.712807 | 1.236575 | 1.468642 | 1.139359 |
| 0.184439 | 0.09916 | 0.050205 | 0.134573 |
| 0 | 0 | 0.016794 | 0 |
| 0.18673 | 0 | 0 | 0.891782 |
| 0.126976 | 0.409596 | 0.272188 | 0.189504 |
| 0.539559 | 0.290082 | 0.247844 | 0.322102 |
| 11.6062 | 9.625064 | 9.797444 | 8.289937 |
| 0.556371 | 0.548388 | 0.340756 | 0.276782 |
| 24.63533 | 27.6754 | 26.64833 | 25.31593 |
| 0.083391 | 0.059778 | 0.034049 | 0 |
| 0.252301 | 0.320613 | 0.421431 | 0.246464 |
| 5.929545 | 4.940543 | 5.219475 | 4.597123 |
| 11.82335 | 10.68102 | 9.937451 | 9.163317 |
| 68.95721 | 53.60525 | 51.861 | 53.08385 |
| 10.99452 | 10.06412 | 10.53423 | 11.06507 |
| 1.267227 | 0.987881 | 0.776129 | 0.869977 |
| 51.56576 | 29.72873 | 30.37256 | 28.29455 |
| 6.725227 | 5.452513 | 5.198244 | 6.483772 |
| 1.046506 | 1.406579 | 0.961419 | 0.312369 |
| 1.400472 | 0 | 1.143648 | 1.114727 |
| 0.197168 | 0.424012 | 0.724547 | 0.235408 |
| 15.96821 | 11.7914 | 12.49002 | 14.24148 |
| 0 | 0 | 0 | 0 |
| 4.299988 | 5.711496 | 4.492578 | 3.623974 |
| 0.458434 | 0.328623 | 0.336928 | 0.510857 |
| 6.478125 | 7.319834 | 7.262738 | 8.25892 |
| 0.032771 | 0.035237 | 0.160567 | 0.039127 |
| 4.961153 | 3.947541 | 4.159395 | 3.896254 |
| 10.15071 | 10.0216 | 9.0428 | 8.042886 |
| 0.172077 | 0.026432 | 0.030112 | 0.02935 |
| 0.129832 | 0.069802 | 0 | 0.11626 |
| 33.38703 | 38.63961 | 37.31956 | 37.37515 |
| 3.70347 | 3.738375 | 3.240337 | 3.880312 |
| 0.280278 | 0.066971 | 0.152586 | 0 |
| 0.145023 | 0.051979 | 0 | 0 |
| 0 | 0 | 0 | 0 |
| 0 | 0 | 0.102326 | 0 |
| 0 | 0.328202 | 0 | 0.36443 |
| 1.32648 | 1.204983 | 1.51278 | 1.037628 |
| 4.441552 | 3.37116 | 3.760391 | 4.913057 |
| 1.559216 | 1.560488 | 1.645471 | 1.474978 |
| 0 | 0.025397 | 0 | 0 |
| 62.39922 | 61.15119 | 65.14872 | 63.30121 |
| 3.892895 | 3.113622 | 3.946346 | 3.411523 |
| 6.870845 | 5.793071 | 5.210069 | 4.401207 |
| 6.855118 | 5.658247 | 5.923196 | 5.807369 |

| | | | |
|---|---|---|---|
| 0.052887 | 0.037912 | 0.043188 | 0.084193 |
| 6.986211 | 6.697666 | 7.212487 | 6.193717 |
| 0 | 0.029274 | 0 | 0 |
| 0 | 0 | 0 | 0 |
| 0 | 0 | 0 | 0.076413 |
| 1.217407 | 0.829664 | 1.071166 | 0.900773 |
| 0 | 0 | 0 | 1.643483 |
| 16.89976 | 14.85633 | 13.1759 | 14.80785 |
| 11.52671 | 12.11018 | 11.8547 | 12.14055 |
| 4.03639 | 3.129428 | 3.670217 | 3.349543 |
| 31.17927 | 30.71716 | 31.87972 | 30.73639 |
| 8.585782 | 9.156559 | 8.832079 | 9.361863 |
| 0.255226 | 0.098515 | 0.048097 | 0.093762 |
| 0.498676 | 0.308724 | 0.296165 | 0.270634 |
| 0.562453 | 0.381967 | 0.163175 | 0.265081 |
| 2.500072 | 1.775705 | 1.826201 | 1.889559 |
| 3.725042 | 3.906305 | 3.909902 | 4.219632 |
| 21.40587 | 16.01419 | 14.51613 | 13.95783 |
| 26.15103 | 27.63147 | 27.77431 | 25.62811 |
| 0 | 0 | 0 | 0 |
| 1.031425 | 0.925734 | 0.678696 | 0.977033 |
| 29.2763 | 30.68351 | 30.83243 | 32.14196 |
| 4.830613 | 4.204789 | 4.226526 | 4.806251 |
| 2.499111 | 1.858161 | 2.409899 | 2.105596 |
| 14.18265 | 12.62217 | 13.52026 | 13.34577 |
| 62.93032 | 33.7569 | 33.73115 | 33.04745 |
| 0 | 0.074418 | 0 | 0.165265 |
| 0.389497 | 0.942322 | 0.715657 | 0.697559 |
| 11.97376 | 11.54988 | 10.59438 | 10.24319 |
| 0.020631 | 0 | 0 | 0.049264 |
| 6.750575 | 5.678655 | 7.04295 | 6.152935 |
| 4.451698 | 3.939838 | 3.460554 | 4.211192 |
| 0 | 0 | 0 | 0 |
| 1.131791 | 0.977919 | 1.287332 | 0.965214 |
| 4.541138 | 4.019688 | 3.860029 | 3.361853 |
| 4.69828 | 4.656496 | 4.20368 | 3.829095 |
| 0 | 0 | 0 | 0.085106 |
| 0.403526 | 0 | 0.494289 | 0 |
| 67.39931 | 57.62439 | 53.69942 | 57.10984 |
| 238.5271 | 266.898 | 260.1924 | 270.7846 |
| 15.78122 | 11.36749 | 12.19905 | 12.17512 |
| 1.923881 | 1.034333 | 1.767456 | 1.148507 |
| 7.230585 | 6.910877 | 7.680798 | 6.316788 |
| 20.61054 | 17.86945 | 17.46289 | 18.72341 |
| 0.074517 | 0 | 0 | 0 |
| 15.75636 | 16.19598 | 15.82529 | 17.78881 |
| 3.310284 | 2.704527 | 2.948175 | 2.450775 |
| 0.358915 | 0.231555 | 0.161203 | 0.257115 |
| 0 | 0 | 0 | 0 |
| 1153.733 | 1231.988 | 1199.85 | 1199.33 |
| 2.441848 | 2.188012 | 2.658738 | 2.429534 |
| 0.020375 | 0 | 0 | 0 |
| 15.09777 | 12.59786 | 13.17985 | 13.74377 |
| 2.2147 | 0.79379 | 0.45214 | 2.203531 |

| | | | |
|---|---|---|---|
| 3.022115 | 2.532513 | 2.346251 | 2.100577 |
| 0 | 0 | 0 | 0 |
| 0.248 | 0.44444 | 0.236275 | 0.427699 |
| 2.508139 | 3.609688 | 2.930483 | 3.17887 |
| 6.573164 | 5.337188 | 5.44588 | 5.422388 |
| 0.326137 | 0.381175 | 0.364755 | 0.237021 |
| 0.597474 | 0.307253 | 0.54094 | 0.434215 |
| 5.668987 | 4.8802 | 4.605467 | 4.521918 |
| 0.043947 | 0.023627 | 0.026916 | 0 |
| 3.584003 | 6.881651 | 5.330876 | 5.501718 |
| 6.02236 | 5.507273 | 6.480673 | 6.451187 |
| 0.504295 | 0.513707 | 0.325117 | 0.602102 |
| 0.251198 | 0.150057 | 0.068378 | 0.066648 |
| 1.250008 | 1.045395 | 0.992422 | 1.16079 |
| 0 | 0 | 0 | 0 |
| 0 | 0 | 0 | 0.019536 |
| 62.31501 | 68.22753 | 68.55086 | 67.15038 |
| 74.68304 | 49.85655 | 48.90784 | 51.91157 |
| 2.969816 | 4.204532 | 3.607485 | 3.457161 |
| 31.77291 | 32.84407 | 33.16996 | 32.93466 |
| 0.077804 | 0 | 0 | 0.092894 |
| 0 | 0 | 0 | 0 |
| 86.2293 | 83.71228 | 82.8942 | 83.74594 |
| 1.135002 | 0.552094 | 0.76135 | 0.419446 |
| 10.22405 | 9.796612 | 8.938279 | 9.549014 |
| 1.013107 | 2.178702 | 3.10245 | 2.419195 |
| 11.31542 | 9.764584 | 8.592941 | 10.03929 |
| 0.451872 | 0.364409 | 0.415132 | 0.404634 |
| 3.960146 | 2.264268 | 2.771932 | 3.22719 |
| 3.532442 | 2.835822 | 2.995598 | 2.958012 |
| 4.030627 | 3.334945 | 3.983243 | 3.376808 |
| 0.051869 | 0 | 0 | 0.061929 |
| 19.29095 | 18.98982 | 20.13539 | 20.275 |
| 8.053049 | 8.07071 | 7.658018 | 7.774694 |
| 1.515706 | 1.014765 | 1.57638 | 0.92191 |
| 8.905116 | 8.615298 | 8.075916 | 8.554995 |
| 22.29709 | 26.29022 | 25.84484 | 25.66159 |
| 4.373187 | 4.116391 | 4.415529 | 4.087006 |
| 2.243653 | 2.068883 | 2.104044 | 2.225714 |
| 11.76314 | 9.972287 | 10.00861 | 11.40946 |
| 0.193824 | 0 | 0.07914 | 0.308554 |
| 0.199648 | 0 | 0 | 0 |
| 1.959983 | 2.851303 | 2.824507 | 3.028387 |
| 0.232122 | 0.232952 | 0.20851 | 0.369523 |
| 20.8467 | 11.69873 | 12.1446 | 11.30791 |
| 0 | 0 | 0 | 0 |
| 58.4097 | 29.54244 | 33.78104 | 31.75522 |
| 12.02161 | 8.093003 | 9.443572 | 8.237479 |
| 2.781709 | 2.490893 | 2.121153 | 2.188484 |
| 5.580545 | 4.997337 | 5.015703 | 5.095145 |
| 20.29194 | 16.62862 | 15.95024 | 16.04496 |
| 0.155101 | 0 | 0 | 0 |
| 135.5241 | 130.5851 | 123.6272 | 133.8638 |
| 0.054109 | 0.232725 | 0.198839 | 0.064604 |

| | | | |
|---|---|---|---|
| 0.176356 | 0.180146 | 0.108011 | 0.094752 |
| 2.284251 | 2.202945 | 2.624961 | 2.10872 |
| 0.08098 | 0 | 0.049597 | 0 |
| 0.84685 | 0.33112 | 0.424361 | 0.41363 |
| 0.251051 | 0.224954 | 0.256266 | 0.249785 |
| 149.2033 | 135.3421 | 138.1468 | 130.6896 |
| 0.328528 | 0.485722 | 0.377271 | 0.318699 |
| 104.533 | 109.2308 | 111.799 | 107.1835 |
| 5.942642 | 5.271193 | 5.087346 | 5.445621 |
| 0.998938 | 0.358038 | 0.815749 | 1.59024 |
| 1.994107 | 1.101064 | 1.089281 | 0.997388 |
| 1.414338 | 1.013851 | 0.577486 | 1.125764 |
| 17.95503 | 14.51125 | 15.05768 | 14.60684 |
| 0.183846 | 0.086486 | 0.070374 | 0.137189 |
| 0.093491 | 0.094244 | 0.093047 | 0.125576 |
| 2.82567 | 2.109248 | 2.917733 | 2.676657 |
| 0.46335 | 0.674063 | 0.600955 | 0.553216 |
| 1.824522 | 2.409638 | 1.967685 | 2.249418 |
| 1.301148 | 0.875753 | 0.742155 | 0.628516 |
| 2.484743 | 1.547492 | 2.003969 | 2.08547 |
| 71.75131 | 78.87956 | 81.02639 | 77.93548 |
| 32.2961 | 28.74527 | 29.9238 | 30.62866 |
| 7.780017 | 7.988126 | 8.061034 | 7.123846 |
| 0.524361 | 0.477081 | 0.765823 | 0.457506 |
| 2.427485 | 0.602347 | 0.686189 | 0.445891 |
| 0.397252 | 0.60189 | 0.597195 | 0.409621 |
| 31.92633 | 31.23781 | 30.80608 | 32.01238 |
| 0 | 0.00998 | 0 | 0 |
| 0.8329 | 1.034333 | 1.005869 | 0.854377 |
| 0.726963 | 0.390836 | 0.323809 | 0.216989 |
| 0 | 0 | 0 | 0 |
| 0 | 0 | 0 | 0 |
| 0.050334 | 0 | 0.030828 | 0 |
| 0.164534 | 0.10615 | 0.080617 | 0.314311 |
| 38.15544 | 44.16844 | 43.49957 | 42.85633 |
| 0.031306 | 0.134647 | 0.038347 | 0 |
| 0 | 0 | 0.259227 | 0 |
| 5.195516 | 3.499245 | 3.566701 | 4.02184 |
| 4.566948 | 4.554132 | 4.087541 | 4.839749 |
| 324.0766 | 359.1509 | 355.6268 | 369.0253 |
| 1.956824 | 2.337876 | 2.929619 | 1.038376 |
| 10.51347 | 8.366155 | 10.23955 | 9.251257 |
| 39.50432 | 40.32567 | 42.03799 | 41.7456 |
| 2.612031 | 2.501654 | 2.535155 | 2.488087 |
| 7.774048 | 7.836656 | 7.340354 | 6.574616 |
| 6.062751 | 4.819215 | 4.426056 | 4.998582 |
| 0 | 0.047276 | 0 | 0 |
| 7.309817 | 4.067377 | 3.850835 | 4.638414 |
| 7.773538 | 7.525384 | 7.011374 | 6.595323 |
| 4.156956 | 4.063451 | 2.777431 | 5.414389 |
| 0 | 0 | 0 | 0.038939 |
| 0 | 0 | 0.060379 | 0.058852 |
| 52.77923 | 42.04938 | 57.38909 | 25.91405 |
| 4.847399 | 4.658692 | 3.888404 | 4.55833 |

| | | | |
|---|---|---|---|
| 6.43704 | 5.951172 | 5.950511 | 5.817989 |
| 0.075376 | 0.092627 | 0.09233 | 0.038569 |
| 0.092399 | 0 | 0 | 0.073546 |
| 0 | 0 | 0.354566 | 0 |
| 0 | 0 | 0.037899 | 0 |
| 10.47952 | 8.337444 | 7.857397 | 8.724741 |
| 0 | 0 | 0 | 0 |
| 1.653939 | 1.86031 | 1.319534 | 0.974367 |
| 10.5914 | 7.20297 | 6.764049 | 7.781898 |
| 34.70185 | 36.23082 | 37.21226 | 35.10118 |
| 0.050709 | 0 | 0 | 0 |
| 0.123518 | 0.332033 | 0.189125 | 0.147474 |
| 1.99943 | 1.088221 | 1.148984 | 1.267287 |
| 0 | 0 | 0.45214 | 0 |
| 4.66778 | 4.035427 | 4.137415 | 4.172814 |
| 0 | 0 | 0 | 0 |
| 0.009544 | 0 | 0 | 0 |
| 17.25682 | 16.43652 | 15.59276 | 17.138 |
| 0.421754 | 0.272096 | 0.258309 | 0.201421 |
| 0.06144 | 0 | 0 | 0.073356 |
| 0 | 0 | 0 | 0.580113 |
| 1.130943 | 0.858392 | 0.937128 | 0.933287 |
| 5.89897 | 6.166308 | 5.314562 | 5.65406 |
| 0.677704 | 0.380194 | 0.433114 | 0.703603 |
| 0.160865 | 0 | 0.197047 | 0.192064 |
| 10.63407 | 8.788395 | 8.569129 | 9.044968 |
| 0 | 0 | 0 | 0.048179 |
| 10.80413 | 5.505096 | 5.889835 | 5.461981 |
| 0.387122 | 0.26016 | 0.207461 | 0.519979 |
| 7.115368 | 7.314212 | 6.902905 | 6.354545 |
| 0.368504 | 0.353783 | 0.193453 | 0.282841 |
| 0.161547 | 0.217131 | 0.296825 | 0.144659 |
| 22.74381 | 22.46108 | 23.09205 | 21.74572 |
| 0.392483 | 0.523305 | 0.288457 | 0.243674 |
| 13.88984 | 13.4959 | 12.41781 | 12.70635 |
| 0 | 0 | 0 | 0 |
| 7.842643 | 6.505346 | 6.953381 | 6.019527 |
| 0 | 0 | 0 | 0.036327 |
| 3.590481 | 3.135191 | 3.335448 | 3.510037 |
| 0 | 0 | 0 | 0 |
| 11.87647 | 12.21291 | 11.92077 | 12.3929 |
| 0.369117 | 0 | 0 | 0 |
| 0.213334 | 0.137633 | 0.209054 | 0.356593 |
| 1395.637 | 1732.268 | 1707.736 | 1708.665 |
| 0.146963 | 0 | 0 | 0 |
| 0.249038 | 0.374892 | 0.305053 | 0.059468 |
| 2.19331 | 1.217224 | 2.079978 | 1.605008 |
| 2.145526 | 1.048633 | 1.164729 | 1.077056 |
| 0.489835 | 0.768102 | 0.775014 | 0.633574 |
| 25.06289 | 22.91963 | 24.08845 | 25.65482 |
| 4.199979 | 3.308274 | 2.572329 | 3.032058 |
| 7.819302 | 8.28227 | 9.72101 | 8.360454 |
| 6.563674 | 7.56356 | 6.685596 | 6.263731 |
| 13.67889 | 11.96798 | 13.34714 | 13.7548 |

| | | | |
|---|---|---|---|
| 1.609088 | 1.322496 | 1.280024 | 1.369108 |
| 0.360728 | 0 | 0.441864 | 0.86138 |
| 3.127024 | 3.260465 | 2.785722 | 3.733504 |
| 962.0274 | 1117.192 | 1088.822 | 1137.711 |
| 0.315338 | 0.254302 | 0.193133 | 0.376497 |
| 6.407338 | 7.89384 | 7.985796 | 7.404692 |
| 19.74883 | 18.44548 | 18.02963 | 18.18476 |
| 0.008456 | 0.009092 | 0.041432 | 0.030288 |
| 10.0266 | 10.82554 | 10.66447 | 9.606553 |
| 2.408094 | 1.467281 | 1.514196 | 1.840089 |
| 4.795731 | 4.461366 | 5.478381 | 4.546371 |
| 1.632208 | 1.608024 | 1.685301 | 1.275374 |
| 21.68505 | 20.40569 | 20.44201 | 19.68996 |
| 0 | 0 | 0 | 0 |
| 0 | 0 | 0.072275 | 0.140895 |
| 0.558137 | 0.450105 | 0.341838 | 0.458141 |
| 0 | 0 | 0 | 0 |
| 3.17264 | 2.501696 | 2.526059 | 2.399046 |
| 13.05792 | 13.9982 | 12.70899 | 13.61223 |
| 0 | 0.045837 | 0 | 0.050896 |
| 0 | 0 | 0 | 0 |
| 10.08667 | 8.601813 | 8.094924 | 8.347019 |
| 0.089002 | 0.4785 | 0 | 0 |
| 0.435778 | 0.30197 | 0.510071 | 0.254367 |
| 0.020057 | 0.043134 | 0 | 0 |
| 0.56326 | 0.044316 | 0.740435 | 0.246038 |
| 14.305 | 15.72268 | 16.10944 | 14.56572 |
| 0 | 0.656404 | 0 | 0 |
| 6.290898 | 6.764332 | 5.674619 | 5.813959 |
| 11.06431 | 9.984962 | 9.77143 | 9.465352 |
| 6.757664 | 5.073982 | 4.793371 | 4.986249 |
| 0.163255 | 0.117027 | 0.066658 | 0.129945 |
| 0 | 0 | 0 | 0 |
| 0.859093 | 0.735576 | 0.818475 | 0.531851 |
| 31.49985 | 26.78127 | 32.00456 | 25.65588 |
| 0.985584 | 0.794817 | 0.754542 | 1.005129 |
| 0.116421 | 0.187773 | 0 | 0 |
| 0 | 0 | 0 | 0 |
| 41.83092 | 44.73974 | 42.51806 | 37.1923 |
| 0 | 0 | 0 | 0 |
| 3.18487 | 5.13683 | 5.219949 | 4.79338 |
| 6.042645 | 5.620248 | 5.403302 | 4.978077 |
| 2.300605 | 1.9337 | 1.607489 | 1.508807 |
| 4.66824 | 3.741852 | 4.262689 | 4.458909 |
| 113.3704 | 98.51833 | 97.28616 | 93.01894 |
| 4.797936 | 3.584883 | 3.900621 | 3.789222 |
| 0.021595 | 0.02322 | 0 | 0 |
| 0.147647 | 0.158758 | 0.180856 | 0.088141 |
| 148.1867 | 119.62 | 114.4182 | 114.7315 |
| 6.775239 | 6.267016 | 6.494992 | 6.355866 |
| 0 | 0 | 0 | 0 |
| 7.088473 | 6.793459 | 8.494086 | 6.991396 |
| 11.14626 | 14.7711 | 13.71321 | 12.54926 |
| 0.478393 | 0.754447 | 0.664128 | 0.913882 |

| | | | |
|---|---|---|---|
| 0 | 0 | 0 | 0 |
| 5.175657 | 4.17955 | 4.554297 | 5.34713 |
| 0.399524 | 0.078108 | 0.266938 | 0.303553 |
| 0 | 0 | 0 | 0.134718 |
| 9.886544 | 8.923927 | 8.807469 | 8.310761 |
| 5.572704 | 4.304876 | 5.229135 | 5.262204 |
| 4.392053 | 4.74339 | 4.621528 | 3.372718 |
| 0.733079 | 0.494947 | 0.584722 | 1.078807 |
| 7.77944 | 6.607743 | 6.508642 | 6.215532 |
| 1.744177 | 0.527467 | 0.467356 | 0.702829 |
| 0 | 0 | 0.767448 | 0 |
| 62.01582 | 67.46362 | 66.84893 | 58.72927 |
| 3.591327 | 3.029358 | 2.654632 | 3.141965 |
| 0.309195 | 0.332464 | 0 | 0 |
| 8.597668 | 8.48694 | 8.661146 | 8.80673 |
| 0.048563 | 0.104435 | 0.029743 | 0.057982 |
| 0.928612 | 0.424892 | 0.411429 | 0.424614 |
| 1.700573 | 1.087248 | 1.013387 | 0.823134 |
| 67.00934 | 61.88017 | 63.25094 | 55.81572 |
| 0.577748 | 0.724765 | 0.412823 | 0.459867 |
| 18.75949 | 20.32748 | 19.10835 | 20.07784 |
| 0 | 0 | 0.097699 | 0 |
| 0.091687 | 0.032862 | 0.037436 | 0.07298 |
| 8.597342 | 10.19249 | 5.940617 | 10.26478 |
| 0 | 0 | 0 | 0 |
| 10.63457 | 11.74395 | 10.50338 | 11.9536 |
| 0.080162 | 0.057463 | 0.065461 | 0.271175 |
| 13.93101 | 12.44322 | 12.8209 | 13.46473 |
| 0 | 0 | 0 | 0 |
| 0 | 0 | 0 | 0 |
| 118.344 | 123.4177 | 127.1576 | 120.6839 |
| 3.098414 | 2.761941 | 2.65476 | 2.54929 |
| 0 | 0 | 0 | 0.072055 |
| 4.328731 | 2.961954 | 2.410168 | 3.288906 |
| 0 | 0 | 0 | 0 |
| 3.478592 | 4.27472 | 3.430945 | 4.315073 |
| 7.04422 | 5.017278 | 4.47983 | 4.753723 |
| 2.652704 | 1.033973 | 1.50283 | 1.464826 |
| 0 | 0 | 0.103968 | 0 |
| 0 | 0 | 0 | 0.321193 |
| 3.982475 | 3.759359 | 3.91393 | 3.690552 |
| 0 | 0.030862 | 0 | 0 |
| 2.175632 | 1.74712 | 1.619231 | 1.479641 |
| 36.33511 | 38.22331 | 39.49462 | 40.43788 |
| 18.53741 | 14.16817 | 15.36642 | 14.87007 |
| 0.425143 | 0.457138 | 0 | 0 |
| 9.412474 | 7.077965 | 7.00817 | 5.508826 |
| 1.058134 | 0.126418 | 0.720075 | 0.140373 |
| 0.059206 | 0 | 0 | 0.017672 |
| 0.957153 | 0.599526 | 0.887867 | 0.632418 |
| 1.12521 | 0.672161 | 0.721965 | 1.151522 |
| 4.737705 | 4.081447 | 4.305142 | 4.548758 |
| 0 | 0.031143 | 0 | 0 |
| 1.74205 | 0.312192 | 0.355647 | 0.693306 |

| | | | |
|---|---|---|---|
| 0.105931 | 0.132887 | 0.237889 | 0.126476 |
| 0.694064 | 0.433894 | 0.593146 | 0.828677 |
| 11.55509 | 9.719875 | 11.82822 | 11.23846 |
| 0 | 0.069376 | 0 | 0 |
| 31.87634 | 30.72954 | 29.73339 | 30.40321 |
| 0 | 0 | 0 | 0 |
| 14.99024 | 0 | 0 | 0 |
| 1.69507 | 0.662777 | 1.321302 | 1.655857 |
| 31.67776 | 26.152 | 27.96566 | 30.18607 |
| 15.01779 | 15.0517 | 13.16387 | 14.41017 |
| 0.857947 | 0 | 1.57638 | 0 |
| 11.87462 | 9.228549 | 9.408978 | 9.404995 |
| 1.432641 | 1.457637 | 1.320875 | 1.416219 |
| 43.51121 | 43.62255 | 48.52125 | 43.78187 |
| 1029.631 | 1134.146 | 1142.381 | 1065.119 |
| 0.440889 | 0.355552 | 0.270028 | 0.394799 |
| 14.55732 | 13.22396 | 12.71491 | 12.80006 |
| 8.945325 | 9.107806 | 8.96951 | 9.782357 |
| 1.703954 | 2.34113 | 1.391477 | 1.469312 |
| 13.20572 | 12.30627 | 11.83159 | 11.68255 |
| 3.30667 | 2.844416 | 4.860505 | 10.26478 |
| 0 | 0 | 0 | 0.058309 |
| 1.388047 | 0.973374 | 1.404557 | 0.864655 |
| 0.366277 | 0 | 0 | 0 |
| 0.050872 | 0.0547 | 0.062314 | 0.121477 |
| 1.645161 | 1.524479 | 1.556454 | 1.533063 |
| 0 | 0 | 0 | 0 |
| 0.372 | 0 | 0.911345 | 0.444149 |
| 0 | 0 | 0 | 0 |
| 0 | 0.316046 | 0.360037 | 0.350933 |
| 1.128137 | 0.970429 | 1.201175 | 0.953217 |
| 0.065859 | 0 | 0 | 0 |
| 0 | 0.06376 | 0 | 0 |
| 1.274346 | 0.957 | 0.817655 | 0.627922 |
| 0.369117 | 0.992238 | 1.017315 | 0.881412 |
| 0.019231 | 0 | 0.07067 | 0.022961 |
| 12.31255 | 13.05738 | 13.54614 | 14.44825 |
| 17.90542 | 16.67454 | 17.38162 | 16.28137 |
| 3.021972 | 2.750812 | 2.42862 | 2.65356 |
| 11.66644 | 9.152859 | 8.891319 | 8.521628 |
| 0.113013 | 0.121518 | 0.046144 | 0.044977 |
| 15.08485 | 13.89634 | 13.34119 | 13.90946 |
| 21.52016 | 28.75166 | 25.65996 | 30.13278 |
| 6.050386 | 6.082584 | 6.32669 | 5.168281 |
| 225.0539 | 199.4169 | 197.5153 | 197.8365 |
| 151.1451 | 148.1475 | 145.0478 | 150.7708 |
| 15.57571 | 10.81338 | 10.22209 | 10.02695 |
| 3.252552 | 1.828553 | 1.981953 | 2.188097 |
| 7.704511 | 9.474472 | 8.852588 | 8.525072 |
| 9.878706 | 6.671003 | 6.531849 | 6.509511 |
| 1.140231 | 1.177 | 0.893886 | 0.952964 |
| 0.06754 | 0 | 0 | 0.24192 |
| 0 | 0 | 0 | 0 |
| 0.23754 | 0.127708 | 0.15871 | 0.103131 |

| | | | |
|---|---|---|---|
| 0 | 0 | 0.124363 | 0.121218 |
| 10.95419 | 6.768948 | 8.15274 | 7.516131 |
| 0 | 0 | 0 | 0 |
| 40.51498 | 36.76531 | 37.05701 | 40.41712 |
| 14.1976 | 18.56685 | 17.34826 | 16.49306 |
| 0 | 0 | 0.62049 | 0 |
| 0.142279 | 0.050996 | 0 | 0 |
| 1.536001 | 2.477394 | 0 | 0.916953 |
| 0.364409 | 0.195916 | 0.223186 | 0.217542 |
| 1.616844 | 1.101064 | 0.396102 | 0.450433 |
| 29.31102 | 17.70507 | 17.8463 | 18.56152 |
| 43.87029 | 32.64008 | 31.53058 | 31.62767 |
| 6.661775 | 6.009329 | 6.517184 | 5.017842 |
| 24.5338 | 23.00772 | 22.71764 | 22.87042 |
| 1.971555 | 1.734487 | 1.536822 | 1.640621 |
| 0.23549 | 0.151927 | 0.20192 | 0.168698 |
| 0.386374 | 0.217618 | 0.270445 | 0.197705 |
| 63.62557 | 68.32935 | 66.43492 | 70.31335 |
| 0.398461 | 0.428448 | 0.488084 | 0.475741 |
| 0.02369 | 0.025472 | 0.029018 | 0 |
| 4.286477 | 3.998225 | 3.922143 | 3.905172 |
| 0.22874 | 0.040992 | 0.093396 | 0.091035 |
| 0.04986 | 0 | 0 | 0 |
| 103.4208 | 105.0079 | 103.5257 | 111.4088 |
| 32.22361 | 34.17091 | 36.40033 | 33.05472 |
| 0.131264 | 0.035286 | 0 | 0.078361 |
| 0 | 0.33795 | 0.192495 | 1.125764 |
| 0 | 0 | 0 | 0 |
| 1.119237 | 1.042373 | 1.003947 | 0.841771 |
| 1.253054 | 0.898237 | 1.023264 | 0.914272 |
| 3.734049 | 2.944379 | 2.185321 | 3.236367 |
| 0.037971 | 0 | 0 | 0 |
| 0.070668 | 0.060789 | 0.051938 | 0.03375 |
| 0.423882 | 0.151927 | 0 | 0.112465 |
| 0.64406 | 0.573129 | 0.462473 | 0.318196 |
| 0 | 0.113148 | 0.128897 | 0 |
| 0.050387 | 0 | 0 | 0 |
| 0.272871 | 0.322747 | 0.200548 | 0.293215 |
| 0 | 0 | 0 | 0 |
| 0 | 0 | 0 | 0 |
| 42.30739 | 41.14058 | 40.08678 | 43.59485 |
| 0 | 0 | 0 | 0.14805 |
| 0.166781 | 0.197266 | 0.040859 | 0.079651 |
| 8.921351 | 7.445115 | 8.220451 | 8.076161 |
| 10.49101 | 10.28152 | 10.71682 | 10.63069 |
| 3.509549 | 3.310072 | 3.073684 | 3.191568 |
| 2.032891 | 1.672419 | 1.687946 | 1.531233 |
| 0 | 0 | 3.513618 | 0 |
| 0.445235 | 0.24584 | 0.23584 | 0.229876 |
| 0 | 0 | 0 | 0 |
| 6.363917 | 5.624257 | 5.381973 | 5.849563 |
| 133.3475 | 153.2944 | 150.133 | 153.4934 |
| 2.307991 | 1.772631 | 1.750118 | 1.705861 |
| 0 | 0 | 0 | 0 |

| | | | |
|---|---|---|---|
| 30.46497 | 27.7565 | 27.41823 | 28.80733 |
| 5.191993 | 5.2889 | 3.598308 | 5.301751 |
| 2.230853 | 1.766105 | 1.84177 | 1.609821 |
| 0.283429 | 0.152379 | 0 | 0.338399 |
| 0 | 0 | 0 | 0.0727 |
| 0 | 0 | 0 | 0 |
| 0.050872 | 0.109401 | 0 | 0.060738 |
| 0 | 0 | 0 | 0 |
| 0.101096 | 0.054352 | 0 | 0 |
| 0 | 0.053894 | 0 | 0 |
| 0.085795 | 0.046126 | 0.078819 | 0.025609 |
| 3.332246 | 2.545831 | 2.577947 | 2.041614 |
| 3.174403 | 2.275533 | 3.888404 | 2.526715 |
| 0.850287 | 0.803455 | 0.694358 | 1.015198 |
| 5.968539 | 4.675509 | 5.083278 | 4.579671 |
| 0.620034 | 0.598665 | 0.542496 | 0.558993 |
| 44.64603 | 56.24732 | 42.01354 | 54.79209 |
| 3.649913 | 4.883275 | 4.914542 | 4.091682 |
| 7.481894 | 7.052629 | 7.132628 | 7.1359 |
| 34.23607 | 29.08434 | 26.21528 | 28.84695 |
| 0 | 0.039782 | 0 | 0 |
| 0.128553 | 0.110582 | 0.157468 | 0.21488 |
| 7.693422 | 4.471571 | 3.990284 | 5.709935 |
| 3.303971 | 2.268899 | 2.618721 | 2.486199 |
| 7.737297 | 7.29132 | 7.515147 | 7.147163 |
| 2.788641 | 2.623693 | 2.519208 | 2.538738 |
| 0.097975 | 0.026337 | 0.090009 | 0.087733 |
| 4.495448 | 3.961242 | 3.657803 | 3.914083 |
| 0 | 0.371011 | 0 | 0.823929 |
| 0 | 0 | 0 | 0 |
| 5.550896 | 4.742207 | 5.141486 | 4.394112 |
| 0.041906 | 0.191505 | 0.12833 | 0.137593 |
| 0 | 0 | 0 | 0.052205 |
| 0 | 0 | 0 | 0 |
| 11.59878 | 10.17426 | 11.96432 | 12.20841 |
| 0.054731 | 0.1177 | 0.100562 | 0.098019 |
| 0 | 0 | 0 | 0 |
| 1.489467 | 1.084521 | 1.091818 | 1.260246 |
| 0.072696 | 0 | 0.059365 | 0.086796 |
| 0.733436 | 0.394316 | 0.350939 | 0.451525 |
| 0.363481 | 0 | 0.445237 | 0 |
| 9.283585 | 8.172317 | 8.265409 | 8.073943 |
| 0 | 0 | 0 | 0 |
| 3.054408 | 2.389896 | 2.405198 | 2.458337 |
| 1.715406 | 1.650344 | 1.680994 | 1.681602 |
| 0 | 0 | 0 | 0 |
| 0 | 0 | 0 | 0 |
| 16.22177 | 17.5142 | 17.27953 | 17.75727 |
| 7.363096 | 7.348581 | 6.85163 | 7.771187 |
| 6.662327 | 7.795807 | 6.960723 | 6.082832 |
| 39.13273 | 71.20848 | 31.17427 | 127.5882 |
| 0 | 0 | 0 | 0 |
| 1.264143 | 1.777518 | 1.667593 | 1.354519 |
| 10.60063 | 11.93756 | 11.13447 | 12.5645 |

| | | | |
|---|---|---|---|
| 0.352222 | 0.165694 | 0.323584 | 0.341685 |
| 4.325752 | 4.298925 | 4.054323 | 4.773457 |
| 10.87788 | 9.13563 | 8.772703 | 8.873529 |
| 0.168851 | 0 | 0.155122 | 0.1512 |
| 28.39965 | 27.82084 | 27.32679 | 28.89061 |
| 0.302516 | 0.390339 | 0.741119 | 0 |
| 2.116269 | 1.137766 | 0 | 1.263358 |
| 0.413573 | 0.326111 | 0.43905 | 0.411487 |
| 0.754235 | 0.789654 | 0.899568 | 0.805726 |
| 0.274486 | 0.233007 | 0.283136 | 0.34497 |
| 7.390448 | 6.778997 | 7.138842 | 7.126207 |
| 2.579417 | 1.220356 | 1.074261 | 0.862313 |
| 9.590559 | 8.385998 | 8.035302 | 9.633324 |
| 0 | 0 | 0 | 0 |
| 0.028815 | 0.061966 | 0.017648 | 0.017202 |
| 1.003147 | 0.719094 | 0.57343 | 0.159694 |
| 0.89002 | 0 | 0 | 0 |
| 44.87964 | 46.02552 | 48.09228 | 46.65514 |
| 0 | 0 | 0 | 0 |
| 0 | 0 | 0 | 0 |
| 25.49825 | 18.72185 | 19.8328 | 19.74011 |
| 1.40564 | 1.196544 | 1.004385 | 1.188768 |
| 0.196761 | 0.423136 | 0.241017 | 0 |
| 4.45037 | 3.972104 | 3.616429 | 3.490247 |
| 19.85101 | 17.23196 | 18.66111 | 18.1892 |
| 16.20479 | 15.66079 | 14.10656 | 12.38762 |
| 0.841217 | 0.870391 | 0.797123 | 0.625362 |
| 18.33816 | 16.37525 | 16.82169 | 16.3963 |
| 31.71238 | 41.24545 | 40.78366 | 38.12745 |
| 49.92043 | 40.50448 | 38.19277 | 40.06247 |
| 2.079781 | 1.750787 | 1.893921 | 1.862363 |
| 0.449208 | 0 | 0.550246 | 0.536331 |
| 0 | 0 | 0 | 0.123321 |
| 10.59684 | 9.670181 | 10.9308 | 11.02894 |
| 0.202069 | 0.170717 | 0.176799 | 0.24126 |
| 0 | 0 | 0.034109 | 0 |
| 8.442561 | 8.978891 | 10.26629 | 10.11663 |
| 26.38186 | 26.55108 | 25.51765 | 22.27948 |
| 0 | 0 | 0 | 0 |
| 0 | 0 | 0 | 0 |
| 0.635651 | 0.486727 | 0.412907 | 0.68994 |
| 49.02728 | 29.11233 | 28.74687 | 33.19954 |
| 0 | 0 | 0 | 0.046183 |
| 6.706747 | 5.014074 | 4.55139 | 5.367914 |
| 0.15872 | 0.34133 | 0.19442 | 0.379007 |
| 0.055743 | 0.053945 | 0.027313 | 0.046588 |
| 1.81794 | 2.330667 | 0.856477 | 1.168745 |
| 0.587852 | 0 | 0 | 0 |
| 0.35104 | 0.21569 | 0.245713 | 0.478998 |
| 0.034158 | 0.036728 | 0.041841 | 0 |
| 0 | 0 | 0 | 0 |
| 0.560189 | 0 | 0 | 0 |
| 2.709856 | 3.954432 | 2.489527 | 3.119877 |
| 0.247228 | 0.265833 | 0.181701 | 0.295177 |

| | | | |
|---|---|---|---|
| 1.523365 | 1.544409 | 1.54612 | 1.922752 |
| 2.130105 | 1.761854 | 2.167658 | 1.017294 |
| 0 | 0.135091 | 0 | 0 |
| 0.509263 | 0.136897 | 0.155952 | 0.456025 |
| 0 | 0 | 0 | 0 |
| 4.496152 | 4.672079 | 5.058039 | 4.861416 |
| 0 | 0.200625 | 0 | 0.044554 |
| 19.91119 | 17.39175 | 16.73932 | 19.24779 |
| 5.833305 | 4.959494 | 5.317476 | 4.535129 |
| 0.099824 | 0.107336 | 0 | 0.357554 |
| 355.8063 | 171.5102 | 172.2485 | 176.5665 |
| 6.808777 | 2.818656 | 2.265764 | 2.303309 |
| 0.408881 | 0.679462 | 0.500848 | 0.488183 |
| 3.271701 | 3.168359 | 3.431443 | 3.196016 |
| 0 | 0 | 0 | 0 |
| 0.045134 | 0 | 0 | 0 |
| 0.027085 | 0.524227 | 0 | 0 |
| 6.085284 | 4.809564 | 3.663297 | 4.346888 |
| 21.00993 | 19.55117 | 19.52108 | 19.79037 |
| 11.46396 | 10.14284 | 10.39365 | 10.09489 |
| 0.284558 | 0.101991 | 0.116187 | 0.226498 |
| 26.22333 | 23.74469 | 25.35916 | 25.54179 |
| 5.029652 | 4.339488 | 3.357161 | 4.207196 |
| 16.21693 | 14.92439 | 14.99126 | 15.31507 |
| 0 | 0 | 0.017292 | 0 |
| 0 | 0 | 3.110723 | 0 |
| 0 | 0 | 0 | 0 |
| 0.323919 | 0 | 0 | 0 |
| 11.03884 | 12.5916 | 11.72306 | 12.00806 |
| 7.512943 | 6.66784 | 6.88644 | 6.46821 |
| 28.24218 | 35.55112 | 35.15162 | 33.6111 |
| 0.013758 | 0 | 0.016852 | 0.065705 |
| 6.813927 | 6.77211 | 7.049672 | 5.866617 |
| 14.42573 | 15.07275 | 15.26289 | 15.20899 |
| 0 | 0 | 0 | 0.531319 |
| 6.049698 | 5.602375 | 5.992161 | 5.18399 |
| 0.066923 | 0.083953 | 0.013663 | 0.066586 |
| 0 | 0 | 0.120012 | 0.233955 |
| 0.691519 | 0.455241 | 0.605042 | 0.539192 |
| 0 | 0 | 0 | 0 |
| 0.796847 | 0.761614 | 0.412122 | 0.50741 |
| 0.368165 | 0.607004 | 0.24052 | 0.351656 |
| 1.062858 | 0.685707 | 0.607563 | 0.930598 |
| 45.09842 | 43.10761 | 44.24736 | 42.14822 |
| 0 | 0 | 0 | 0.044942 |
| 102.8074 | 92.75751 | 81.42924 | 109.826 |
| 2.163411 | 2.16813 | 3.241766 | 1.529738 |
| 0.110735 | 0.396895 | 0.135642 | 0.044071 |
| 0.248 | 0 | 0 | 0 |
| 316.945 | 239.1849 | 245.3447 | 236.9842 |
| 13.75257 | 14.01936 | 13.47669 | 12.60277 |
| 14.1619 | 12.98663 | 13.05954 | 13.46305 |
| 41.22886 | 42.26832 | 38.7833 | 40.51908 |
| 0 | 0 | 0 | 0 |

| | | | |
|---|---|---|---|
| 3.30271 | 3.193581 | 3.376159 | 3.829789 |
| 0.99441 | 0.870317 | 0.878149 | 1.049219 |
| 0 | 0.065894 | 0.075066 | 0 |
| 37.62456 | 34.94726 | 36.14853 | 37.75311 |
| 0.741032 | 1.225846 | 0.651688 | 0.862068 |
| 0.742895 | 0.798803 | 0.563327 | 0.675793 |
| 0.565928 | 0.944253 | 0.788836 | 1.397978 |
| 3.368896 | 2.240678 | 2.084593 | 2.778281 |
| 9.201874 | 8.264948 | 8.207693 | 8.981077 |
| 0 | 0 | 0 | 0 |
| 0 | 0 | 0.040845 | 0 |
| 16.08949 | 14.64136 | 15.3259 | 14.88807 |
| 1.39025 | 3.737189 | 2.128688 | 2.489829 |
| 0 | 0 | 0 | 0 |
| 0.894198 | 0.72112 | 1.056206 | 0.686331 |
| 0 | 0 | 0.092581 | 0 |
| 0.037331 | 0.120422 | 0.137184 | 0.089143 |
| 2.350432 | 1.561826 | 1.940967 | 1.450444 |
| 1.175705 | 0 | 0 | 0 |
| 0 | 0 | 0 | 0 |
| 0.053025 | 0.11403 | 0.064951 | 0.126617 |
| 33.81503 | 31.48632 | 28.9447 | 31.83053 |
| 0.076369 | 0.177919 | 0.15591 | 0.121574 |
| 2.3617 | 1.139011 | 1.25501 | 0.974472 |
| 4.107896 | 4.523223 | 4.596413 | 4.433017 |
| 26.93123 | 26.09522 | 26.46623 | 27.14181 |
| 3.893882 | 1.530246 | 2.348538 | 2.784736 |
| 1.088544 | 0.936372 | 0.495257 | 0.668399 |
| 30.96691 | 27.09369 | 27.02845 | 31.15282 |
| 3.331498 | 3.378351 | 2.853266 | 2.619419 |
| 0.043445 | 0.046715 | 0.070956 | 0.051871 |
| 0 | 0.039233 | 0 | 0 |
| 34.93002 | 34.38214 | 32.75815 | 34.69622 |
| 35.23951 | 30.60244 | 30.38743 | 32.50493 |
| 57.2474 | 64.31731 | 62.54839 | 66.69613 |
| 181.9292 | 210.7613 | 206.5962 | 214.3674 |
| 0.69356 | 0.298302 | 0.278037 | 0.165615 |
| 0.770961 | 0.276327 | 0.35976 | 0.657491 |
| 11.73364 | 9.90855 | 9.182634 | 10.18862 |
| 16.4301 | 17.42655 | 19.08654 | 15.4055 |
| 36.73146 | 32.41829 | 34.6102 | 30.01645 |
| 0.054481 | 0.019527 | 0.04449 | 0.043365 |
| 0.834746 | 0.744789 | 0.522128 | 0.508924 |
| 5.633448 | 6.201628 | 5.914755 | 5.164641 |
| 0.1207 | 0.064892 | 0 | 0 |
| 10.31122 | 5.949236 | 6.058517 | 6.205576 |
| 0 | 0 | 0 | 0 |
| 0.042288 | 0 | 0.051799 | 0 |
| 2.27975 | 1.773293 | 1.168502 | 2.104166 |
| 0 | 0 | 0 | 0 |
| 0.946014 | 0.542511 | 0.424892 | 0.338848 |
| 89.91084 | 101.1494 | 99.64503 | 100.1558 |
| 4.61442 | 4.170384 | 4.336691 | 4.369507 |
| 2.28557 | 2.125998 | 2.288603 | 1.754262 |

| | | | |
|---|---|---|---|
| 7.849612 | 6.104656 | 6.561304 | 6.719569 |
| 5.283122 | 3.379203 | 3.162883 | 3.792777 |
| 9.553353 | 8.863607 | 7.825093 | 7.696422 |
| 4.698952 | 5.052581 | 4.220965 | 4.737591 |
| 0.239621 | 0.110423 | 0.125793 | 0.122612 |
| 0 | 0 | 0.19442 | 0 |
| 0 | 0 | 0.08045 | 0.078415 |
| 45.93607 | 43.67729 | 43.61401 | 43.25952 |
| 2.496721 | 2.39698 | 1.638372 | 1.596941 |
| 5.067451 | 5.094155 | 4.003489 | 5.262664 |
| 0.032681 | 0 | 0 | 0.029264 |
| 4.6635 | 4.081894 | 4.144999 | 3.717644 |
| 2.164366 | 2.327249 | 1.767456 | 3.876211 |
| 1.760566 | 1.319932 | 1.147528 | 1.099224 |
| 22.6564 | 19.71155 | 20.20972 | 22.11187 |
| 0.940257 | 0.763033 | 0.608469 | 0.508355 |
| 1377.413 | 1518.194 | 1490.386 | 1497.727 |
| 6.020803 | 5.774513 | 5.88368 | 6.212798 |
| 0.110222 | 0 | 0 | 0.1316 |
| 27.29527 | 31.29372 | 26.0516 | 26.57506 |
| 0.690088 | 0.371011 | 0.105663 | 0.308973 |
| 58.73263 | 63.65072 | 58.95017 | 62.10484 |
| 0 | 0 | 0 | 0 |
| 0 | 0.00998 | 0 | 0.011082 |
| 0.542542 | 0.68632 | 0.781851 | 0.533455 |
| 24.56993 | 21.19899 | 19.35943 | 21.89677 |
| 0 | 0 | 0 | 0 |
| 1.428481 | 1.535985 | 4.082824 | 0 |
| 12.08715 | 16.47573 | 13.08597 | 13.5568 |
| 2.336361 | 1.829528 | 2.146399 | 1.728273 |
| 0.638916 | 0.289263 | 0.329526 | 0.160596 |
| 14.15935 | 10.72812 | 11.48958 | 11.52001 |
| 16.60057 | 17.47684 | 15.89359 | 17.39705 |
| 0 | 0 | 0 | 0.028727 |
| 1.506926 | 1.726336 | 1.69061 | 1.496524 |
| 9.743217 | 5.914132 | 4.812381 | 6.566957 |
| 0 | 0 | 0 | 0 |
| 6.07774 | 5.98673 | 5.900286 | 6.17395 |
| 20.38493 | 17.53052 | 17.92787 | 18.18187 |
| 6.548096 | 6.466901 | 6.255448 | 5.84232 |
| 3.786564 | 3.291153 | 3.09217 | 3.46607 |
| 69.78048 | 52.53781 | 53.1463 | 54.27907 |
| 3.156209 | 4.08269 | 3.575433 | 3.796684 |
| 0.763894 | 0.319426 | 0.51984 | 0.456025 |
| 6.219483 | 5.848912 | 6.320085 | 6.494538 |
| 116.8191 | 97.70638 | 99.08748 | 100.0709 |
| 6.904995 | 5.680842 | 6.205336 | 6.228069 |
| 5.590548 | 5.182134 | 5.313103 | 5.869242 |
| 0.473791 | 0.084908 | 0.386906 | 0.282841 |
| 3.38327 | 3.129577 | 3.485712 | 3.286894 |
| 2.829729 | 2.516507 | 2.593138 | 2.60377 |
| 0 | 0 | 0 | 0 |
| 21.93564 | 19.57536 | 19.10681 | 19.44906 |
| 0.890328 | 0.567307 | 0.686664 | 0.236223 |

| | | | |
|---|---|---|---|
| 0.165525 | 0.237309 | 0.13517 | 0.197628 |
| 5.472005 | 5.436505 | 5.291679 | 5.539925 |
| 0 | 0 | 0 | 0 |
| 1.557065 | 1.566229 | 1.538134 | 1.649161 |
| 0 | 0 | 0 | 0 |
| 1.578432 | 1.357776 | 1.546768 | 1.444834 |
| 3.9316 | 3.340232 | 3.151153 | 3.216346 |
| 0.039813 | 0 | 0 | 0 |
| 15.66151 | 13.30915 | 15.4711 | 11.51096 |
| 0.16141 | 0.074382 | 0.084735 | 0.247777 |
| 4.376013 | 4.030393 | 3.976278 | 3.811485 |
| 3.341477 | 0 | 4.093057 | 0.997388 |
| 15.70181 | 17.93583 | 17.98257 | 18.97577 |
| 1.886531 | 1.785085 | 1.941121 | 2.522711 |
| 11.87029 | 11.08528 | 10.90516 | 9.61706 |
| 2.928416 | 1.826304 | 2.080511 | 1.503442 |
| 36.75625 | 31.71466 | 30.5405 | 32.68362 |
| 6.735928 | 6.993101 | 6.909707 | 7.012294 |
| 0.089113 | 0.06388 | 0.072771 | 0.106396 |
| 2.207001 | 2.076457 | 2.128935 | 2.272726 |
| 7.13942 | 6.970068 | 6.476608 | 7.703786 |
| 17.54275 | 17.11141 | 17.88154 | 16.75611 |
| 0.084557 | 0.102285 | 0.064735 | 0.063098 |
| 0.0264 | 0.047311 | 0.010779 | 0.021013 |
| 0.233412 | 0.150587 | 0.114365 | 0.111473 |
| 0.41226 | 0.110821 | 0.126247 | 0.246109 |
| 0.036855 | 0 | 0.045144 | 0 |
| 1.321875 | 1.022558 | 1.048401 | 1.158141 |
| 62.01781 | 54.61279 | 53.06648 | 53.24054 |
| 0 | 0 | 0.073784 | 0 |
| 3.137326 | 2.269399 | 2.532878 | 2.826379 |
| 0 | 0 | 0 | 0 |
| 0 | 0 | 0.45214 | 0 |
| 0.067018 | 0.144122 | 0.205229 | 0.320062 |
| 24.28166 | 20.98228 | 19.95772 | 22.092 |
| 0.408721 | 0 | 0 | 0 |
| 0 | 0 | 0.22695 | 0.22121 |
| 36.08721 | 31.15252 | 29.13059 | 34.59126 |
| 0 | 0 | 0 | 0 |
| 2.949192 | 2.671445 | 2.430252 | 2.219412 |
| 72.10153 | 85.2076 | 87.48909 | 83.68203 |
| 11.18861 | 8.925953 | 8.242069 | 8.433783 |
| 0 | 0 | 0 | 0 |
| 9.369425 | 7.532939 | 6.348889 | 7.820425 |
| 1118.484 | 525.7036 | 523.9854 | 522.5927 |
| 0 | 0.1595 | 0.181701 | 0.354212 |
| 554.5527 | 621.0123 | 636.3231 | 603.3148 |
| 7.737055 | 7.130848 | 8.664967 | 7.390114 |
| 0.177539 | 0.11454 | 0.065242 | 0.169578 |
| 0 | 0.02144 | 0 | 0.095228 |
| 2.20879 | 1.828553 | 1.77181 | 1.890369 |
| 4.475283 | 4.217037 | 3.890369 | 4.050532 |
| 5.814756 | 4.65601 | 4.849455 | 4.302145 |
| 26.88578 | 16.22178 | 16.0297 | 16.47589 |

| | | | |
|---|---|---|---|
| 0.257942 | 0.499237 | 0.379151 | 0.40036 |
| 1.020344 | 1.584746 | 0.902665 | 1.962716 |
| 5.500348 | 5.977206 | 5.93116 | 5.257197 |
| 0.68436 | 0.471574 | 0.596248 | 0.575416 |
| 23.7884 | 15.87057 | 15.57926 | 14.83378 |
| 0 | 0.039659 | 0 | 0 |
| 8.335339 | 7.402335 | 7.647286 | 7.279303 |
| 5.004111 | 4.456328 | 4.605103 | 4.488647 |
| 2.290392 | 2.743601 | 2.141083 | 2.014975 |
| 0.045077 | 0.048469 | 0.073621 | 0.107639 |
| 4.901652 | 3.764668 | 4.383985 | 5.20206 |
| 28.67888 | 27.7045 | 29.21879 | 28.12661 |
| 0.273655 | 0.29425 | 0.670414 | 0.980191 |
| 56.58141 | 48.80732 | 49.66257 | 48.08952 |
| 9.971302 | 8.021429 | 7.750671 | 7.701647 |
| 0 | 0 | 0 | 0 |
| 2.498636 | 2.305045 | 2.4346 | 2.406933 |
| 16.59842 | 17.76946 | 16.10529 | 16.39185 |
| 0 | 0 | 0.357829 | 0 |
| 0.068218 | 0.244506 | 0.055708 | 0.108598 |
| 0.055175 | 0.039552 | 0.045057 | 0.021959 |
| 0 | 0.145867 | 0 | 0.161969 |
| 0 | 0.045551 | 0.103783 | 0.050579 |
| 6.409852 | 6.030709 | 5.888689 | 5.056467 |
| 2.611347 | 2.005621 | 1.9375 | 1.9954 |
| 0 | 0.104702 | 0 | 0 |
| 13.69273 | 12.4187 | 12.64902 | 12.75563 |
| 0 | 0 | 0 | 0 |
| 1.113102 | 1.414484 | 0.853888 | 1.181321 |
| 3.034869 | 2.194264 | 3.589296 | 2.374001 |
| 2.08004 | 2.236577 | 2.616754 | 3.288906 |
| 0.154723 | 0.103979 | 0.094762 | 0.138549 |
| 0.76518 | 0.599443 | 0.629322 | 0.678663 |
| 38.17125 | 42.84508 | 36.5411 | 38.29789 |
| 1.479056 | 0.97474 | 0.876644 | 0.550662 |
| 23.80802 | 15.73318 | 9.645065 | 10.58555 |
| 19.33143 | 19.45381 | 18.48067 | 20.60251 |
| 1.812702 | 1.23964 | 1.438834 | 1.194678 |
| 0 | 0 | 0 | 0 |
| 0 | 0.088427 | 0 | 0.196377 |
| 0.343798 | 0.369671 | 0.315845 | 0.102619 |
| 9.616898 | 7.940661 | 7.392018 | 8.224984 |
| 4.529637 | 4.828391 | 4.367735 | 4.453772 |
| 10.14583 | 9.62592 | 9.918591 | 8.878168 |
| 2.162518 | 1.022766 | 1.015752 | 1.009478 |
| 3.524192 | 2.417871 | 2.255081 | 2.370758 |
| 6.49477 | 4.708454 | 5.837113 | 6.062944 |
| 47.58597 | 42.46679 | 43.73533 | 42.37794 |
| 12.76413 | 11.54208 | 10.70435 | 12.03567 |
| 12.24116 | 10.85712 | 9.742189 | 13.54187 |
| 0.300344 | 0.397473 | 0.311299 | 0.413763 |
| 216.2673 | 190.8233 | 192.2479 | 191.5151 |
| 9.194581 | 8.646022 | 7.836761 | 8.139473 |
| 19.25544 | 18.05682 | 16.85248 | 18.71676 |

| | | | |
|---|---|---|---|
| 0.358015 | 0 | 0 | 0 |
| 0.815851 | 0.358875 | 0.408828 | 0.265659 |
| 6.210789 | 5.434806 | 5.460215 | 5.299866 |
| 14.46412 | 15.03851 | 15.86274 | 16.5558 |
| 8.913348 | 7.230141 | 8.619615 | 7.374772 |
| 0 | 0 | 0 | 0 |
| 58.68277 | 57.22756 | 49.4077 | 50.50532 |
| 26.49959 | 24.85873 | 25.38824 | 25.49139 |
| 0.297352 | 0.298415 | 0.339952 | 0.544369 |
| 3.218492 | 1.185173 | 1.72818 | 0.10528 |
| 0.073823 | 0.238137 | 0 | 0 |
| 10.84734 | 10.10142 | 9.808647 | 9.028363 |
| 0.484128 | 0.281971 | 0.506539 | 0.325139 |
| 0.173149 | 0.12412 | 0.141397 | 0.137821 |
| 129.8763 | 128.8492 | 127.019 | 126.64 |
| 23.48757 | 20.9176 | 22.12049 | 21.201 |
| 15.48956 | 12.51156 | 11.6866 | 12.50783 |
| 24.9359 | 27.36615 | 29.25313 | 28.80611 |
| 439.203 | 400.8758 | 416.3714 | 396.851 |
| 0 | 0.066321 | 0.075552 | 0 |
| 11.98182 | 12.99508 | 12.26245 | 12.20007 |
| 3.399387 | 2.013017 | 2.89669 | 2.705794 |
| 7.40694 | 9.306929 | 8.424875 | 8.085488 |
| 0 | 0 | 0 | 0 |
| 11.53836 | 10.13505 | 10.15232 | 8.731396 |
| 16.0699 | 16.5749 | 16.02337 | 16.13144 |
| 9.481804 | 7.056625 | 6.745536 | 6.228902 |
| 6.787083 | 6.238954 | 4.955596 | 6.895856 |
| 0.109883 | 0.118153 | 0.134599 | 0.306121 |
| 0.581866 | 0.52138 | 0.237581 | 0.926291 |
| 0.165982 | 0 | 0.40663 | 0 |
| 2.702225 | 1.773937 | 1.567905 | 1.562216 |
| 0 | 0.079461 | 0 | 0.088232 |
| 0.10601 | 0.037996 | 0.021642 | 0 |
| 0.016049 | 0 | 0 | 0.019161 |
| 241.617 | 261.7723 | 254.3241 | 258.1368 |
| 0 | 0 | 0 | 0 |
| 3.634813 | 3.126686 | 3.561897 | 2.748526 |
| 7.458228 | 7.157479 | 6.517044 | 7.396443 |
| 2.385061 | 2.930919 | 2.71284 | 2.440834 |
| 2.720917 | 0.731421 | 0.833229 | 0 |
| 5.279391 | 4.275721 | 4.435599 | 4.828504 |
| 0 | 0.099416 | 0.056627 | 0.11039 |
| 91.65233 | 102.0593 | 104.1099 | 100.7229 |
| 0 | 0 | 0 | 0.414971 |
| 9.251584 | 7.609102 | 7.392387 | 7.863811 |
| 3.140304 | 2.481314 | 2.112735 | 2.314946 |
| 2.127177 | 0.967688 | 1.302816 | 1.367552 |
| 16.11217 | 14.15557 | 14.1202 | 13.68492 |
| 3.660656 | 3.492219 | 3.320877 | 3.581947 |
| 0.336113 | 0.220861 | 0.091492 | 0.222945 |
| 10.51376 | 11.02471 | 11.78761 | 12.03417 |
| 2.039647 | 1.227117 | 1.368179 | 1.391562 |
| 42.64336 | 45.45973 | 44.3288 | 44.85565 |

| | | | |
|---|---|---|---|
| 0 | 0 | 0 | 0 |
| 0 | 0 | 0 | 0 |
| 2.593025 | 1.806857 | 2.005125 | 2.196559 |
| 45.04609 | 41.8624 | 41.68661 | 42.19521 |
| 29.25494 | 21.20277 | 20.70912 | 20.53278 |
| 23.12147 | 19.86064 | 22.46232 | 22.10582 |
| 0.055789 | 0.029994 | 0 | 0 |
| 13.99379 | 13.40907 | 13.80409 | 12.43479 |
| 0.007889 | 0 | 0 | 0 |
| 1.307689 | 1.174167 | 0.957789 | 0.659936 |
| 3.945173 | 1.914825 | 1.375931 | 2.911247 |
| 19.28424 | 16.3994 | 19.37869 | 16.94422 |
| 4.121195 | 4.431344 | 3.822771 | 4.06298 |
| 1.298083 | 1.20544 | 1.204586 | 1.291536 |
| 0.253727 | 0.09094 | 0.051799 | 0.050489 |
| 9.121848 | 7.748581 | 7.128741 | 8.16826 |
| 8.865909 | 7.23203 | 7.419487 | 7.277487 |
| 0.063914 | 0.068724 | 0.23487 | 0 |
| 2.018881 | 1.111881 | 1.749179 | 1.352198 |
| 995.3676 | 1061.151 | 1041.074 | 1076.36 |
| 7.954336 | 5.478621 | 6.645309 | 5.908312 |
| 8.446672 | 7.92479 | 6.948409 | 9.096174 |
| 0 | 0 | 0 | 0 |
| 0.071711 | 0.269877 | 0.08784 | 0.04281 |
| 15.22626 | 13.92363 | 12.44789 | 14.28379 |
| 0.15527 | 0.111303 | 0.253592 | 0.308973 |
| 7.490495 | 5.938003 | 5.505174 | 5.944639 |
| 34.05466 | 34.9167 | 34.64802 | 36.0492 |
| 7.748429 | 8.168645 | 7.794483 | 6.382827 |
| 75.65732 | 50.4651 | 48.80763 | 50.29667 |
| 148.087 | 161.4694 | 157.8364 | 160.7425 |
| 0.697671 | 0.187544 | 0.427297 | 0.416491 |
| 0.078965 | 0.084908 | 0.096726 | 0.04714 |
| 0.031503 | 0.10162 | 0 | 0.018806 |
| 10.18739 | 9.155231 | 9.328499 | 9.301279 |
| 0.280566 | 0.30168 | 0.294576 | 0.526399 |
| 28.95624 | 26.59658 | 25.6596 | 26.42374 |
| 0.566858 | 0 | 0 | 0 |
| 12.02902 | 11.4706 | 10.7192 | 11.67537 |
| 7.150013 | 5.771918 | 6.309117 | 4.359189 |
| 334.0491 | 343.7397 | 335.8543 | 344.1855 |
| 0 | 0.157054 | 0 | 0 |
| 0.164548 | 0.464445 | 0.251948 | 0.392923 |
| 9.294174 | 11.24283 | 11.62862 | 10.66082 |
| 0 | 0.026666 | 0.030378 | 0 |
| 0.084426 | 0.04539 | 0.025854 | 0.0504 |
| 177.863 | 130.4976 | 136.3894 | 125.6643 |
| 80.09613 | 75.02926 | 73.66985 | 74.34742 |
| 12.58974 | 11.83975 | 11.9166 | 10.01314 |
| 6.73251 | 5.629203 | 5.892441 | 5.401107 |
| 0 | 0 | 0 | 0 |
| 1.625815 | 1.332979 | 1.742563 | 1.747024 |
| 3.0409 | 1.595 | 1.726161 | 2.213827 |
| 18.56634 | 15.40725 | 16.05167 | 15.96744 |

| | | | |
|---|---|---|---|
| 2.246724 | 25.95042 | 2.396961 | 2.076752 |
| 0 | 0 | 0 | 0.040291 |
| 23.47555 | 18.65191 | 19.05247 | 20.29656 |
| 0.012824 | 0 | 0 | 0.015311 |
| 36.79422 | 28.15404 | 30.00365 | 32.71144 |
| 0.033391 | 0 | 0.040902 | 0.039868 |
| 0.751832 | 0.359295 | 0.716285 | 0.698171 |
| 0.151483 | 0.19003 | 0.061852 | 0.120575 |
| 3.007329 | 0.538942 | 0.613959 | 0 |
| 16.20599 | 14.77271 | 13.85282 | 14.29501 |
| 19.28519 | 18.9838 | 18.83504 | 18.13944 |
| 8.979535 | 7.978207 | 7.969682 | 8.513031 |
| 0.225002 | 0.423386 | 0.172257 | 0.235061 |
| 20.54091 | 22.62722 | 21.34381 | 20.20394 |
| 0 | 0 | 0 | 0.015419 |
| 0.570252 | 0.408778 | 0.552992 | 0.453901 |
| 11.60789 | 8.55872 | 7.370557 | 8.485237 |
| 2443.385 | 2689.464 | 2724.691 | 2742.306 |
| 15.36814 | 16.8859 | 17.5904 | 19.50183 |
| 9.116474 | 7.490156 | 7.587828 | 7.17267 |
| 0.576291 | 0.671299 | 0.735326 | 0.430038 |
| 8.138796 | 7.014231 | 6.662973 | 6.970576 |
| 0.296673 | 0.212667 | 0.302835 | 0.236142 |
| 165.4106 | 147.629 | 135.7454 | 135.4569 |
| 0 | 0 | 0 | 0 |
| 0.218924 | 0.1177 | 0.201124 | 0.065346 |
| 3.9836 | 5.548288 | 4.093631 | 5.298866 |
| 5.566892 | 5.414671 | 5.647816 | 5.479623 |
| 0.02578 | 0 | 0 | 0 |
| 0 | 0.533328 | 0 | 0 |
| 27.60436 | 26.18832 | 24.41187 | 25.05945 |
| 22.82877 | 22.3751 | 20.54157 | 20.89899 |
| 0.13624 | 0.146494 | 0.083442 | 0.162664 |
| 0.313569 | 0.349655 | 0.298743 | 0.360519 |
| 0.1728 | 0.103225 | 0.094074 | 0.022924 |
| 0.23808 | 0.142221 | 0.162017 | 0.15792 |
| 24.81734 | 24.00375 | 24.52676 | 23.99513 |
| 69.89984 | 63.06447 | 62.37575 | 57.92762 |
| 0 | 0 | 0 | 0 |
| 0.521344 | 0.579264 | 0.468311 | 0.560211 |
| 4.669336 | 4.55474 | 4.48662 | 4.289703 |
| 0.108218 | 0.116362 | 0 | 0.172276 |
| 69.47123 | 107.3134 | 73.1875 | 69.45159 |
| 2.236263 | 2.228615 | 1.937521 | 1.172187 |
| 14.96179 | 13.09043 | 11.98576 | 11.54682 |
| 3.182145 | 2.859679 | 3.143917 | 3.133743 |
| 147.8407 | 126.7568 | 120.4858 | 121.5314 |
| 0.066225 | 0 | 0 | 0 |
| 0.073171 | 0.059008 | 0.134443 | 0.152884 |
| 3.415785 | 3.99116 | 4.016716 | 4.703605 |
| 20.18013 | 12.23607 | 11.57842 | 11.57326 |
| 12.5995 | 12.21609 | 11.58607 | 11.0145 |
| 0.282002 | 0.257741 | 0.22453 | 0.235687 |
| 2.692175 | 2.400298 | 2.288447 | 2.139065 |

| | | | |
|---|---|---|---|
| 5.290672 | 10.66656 | 12.15126 | 9.475181 |
| 102.3691 | 113.6419 | 111.9223 | 112.2 |
| 40.86731 | 46.86567 | 49.06431 | 46.29409 |
| 0.09384 | 0.109311 | 0.038316 | 0.037347 |
| 16.23155 | 10.86221 | 9.925097 | 10.52216 |
| 0 | 0 | 0.19442 | 0 |
| 0.092819 | 0 | 0.170544 | 0 |
| 2.252696 | 2.222593 | 2.21357 | 1.9507 |
| 3.615927 | 3.478782 | 4.056249 | 3.72645 |
| 0.316036 | 0 | 0 | 0.251553 |
| 0 | 0 | 0 | 0 |
| 0.605289 | 0.433894 | 0 | 0.481789 |
| 3.070765 | 1.919207 | 1.833709 | 1.489448 |
| 0 | 0 | 0 | 0 |
| 0.455888 | 0.260743 | 0.285155 | 0.324268 |
| 0 | 0 | 0.22963 | 0 |
| 26.17306 | 31.19439 | 30.34942 | 31.30306 |
| 1.386216 | 1.167415 | 1.258665 | 1.296279 |
| 35.83677 | 26.05459 | 26.46595 | 25.69307 |
| 3.62214 | 2.353067 | 2.172207 | 2.477662 |
| 0 | 0.299412 | 0 | 0 |
| 13.4058 | 11.7507 | 13.30316 | 13.33144 |
| 3.571203 | 2.559974 | 2.819093 | 3.032058 |
| 4.505895 | 4.893445 | 4.415505 | 4.357642 |
| 0.0277 | 0 | 0 | 0 |
| 0.891686 | 0.191758 | 1.092248 | 0.425851 |
| 2.191488 | 1.388184 | 1.475095 | 1.528464 |
| 99.6627 | 98.64378 | 102.2222 | 99.85503 |
| 5.086038 | 4.550774 | 4.302742 | 4.354118 |
| 51.3784 | 57.44173 | 55.82354 | 57.98618 |
| 8.821664 | 8.598924 | 9.074368 | 9.048043 |
| 87.69332 | 92.12008 | 90.3451 | 93.99916 |
| 16.58183 | 14.97698 | 15.30845 | 15.3798 |
| 0 | 0 | 0.071303 | 0 |
| 1.994843 | 1.398893 | 0.956165 | 0.828431 |
| 184.4782 | 123.4639 | 116.2355 | 119.3873 |
| 44.25168 | 45.33019 | 45.1638 | 42.5051 |
| 0 | 0.141045 | 0 | 0 |
| 12.27601 | 12.66654 | 12.75883 | 11.39983 |
| 21.80785 | 18.82094 | 20.7377 | 19.70945 |
| 0 | 1.70665 | 0.972101 | 0 |
| 0.032547 | 0.034996 | 0 | 0 |
| 28.40255 | 35.03123 | 36.83751 | 29.92163 |
| 13.12851 | 12.18544 | 11.99931 | 11.8908 |
| 8.104859 | 6.331851 | 8.376615 | 8.467183 |
| 15.51377 | 12.30998 | 13.27934 | 11.71612 |
| 3.286848 | 2.604152 | 1.907119 | 2.736701 |
| 36.30671 | 32.89152 | 33.32918 | 31.19087 |
| 0 | 0.154681 | 0.176212 | 0 |
| 0 | 0 | 0 | 0 |
| 25.78065 | 26.76034 | 26.36678 | 26.51471 |
| 0.148916 | 0.280216 | 0.273617 | 0.266698 |
| 0 | 0.172971 | 0 | 0 |
| 58.20936 | 54.55873 | 52.03599 | 49.77686 |

| | | | |
|---|---|---|---|
| 0 | 0 | 0 | 0 |
| 1.260088 | 0.574814 | 1.169328 | 1.504479 |
| 0.202015 | 0.127775 | 0.131004 | 0.056752 |
| 0.07475 | 0 | 0.091564 | 0 |
| 0.157432 | 0.215447 | 0.333091 | 0.222141 |
| 55.26405 | 54.27903 | 51.78006 | 53.14507 |
| 0.239678 | 0 | 0.195725 | 0.095388 |
| 10.36674 | 10.80784 | 10.26017 | 11.90673 |
| 0 | 0 | 0 | 0.592199 |
| 16.87572 | 13.24083 | 13.40481 | 12.44875 |
| 0.108246 | 0.05173 | 0.088395 | 0.10052 |
| 71.70137 | 69.51544 | 72.20397 | 72.08075 |
| 3.295925 | 2.552567 | 2.453511 | 2.581265 |
| 1.52694 | 1.355303 | 1.296919 | 1.341517 |
| 0 | 0.419668 | 0.478082 | 0 |
| 0.014499 | 0 | 0.017761 | 0 |
| 0 | 0 | 0 | 0 |
| 3.356771 | 2.898287 | 2.933487 | 2.596104 |
| 0.124649 | 0 | 0 | 0 |
| 6.322353 | 5.831053 | 6.480673 | 6.25362 |
| 0.194779 | 0 | 0.018353 | 0.071556 |
| 0 | 0.033684 | 0 | 0.037402 |
| 29.32062 | 19.81546 | 22.99769 | 23.02024 |
| 0 | 0 | 0 | 0 |
| 0 | 0.23486 | 0 | 0 |
| 0.36609 | 0.39364 | 0.239164 | 0.611929 |
| 0 | 0 | 0 | 0 |
| 0.292123 | 0 | 0 | 0 |
| 0 | 0 | 0 | 0 |
| 32.95515 | 31.69982 | 29.43631 | 29.26271 |
| 0.08564 | 0.036834 | 0.020981 | 0.10225 |
| 35.33093 | 28.82984 | 27.02111 | 30.62036 |
| 2.769648 | 2.372372 | 2.779257 | 2.260592 |
| 39.10588 | 33.11813 | 32.02442 | 32.37901 |
| 18.58531 | 17.21158 | 17.03108 | 18.28144 |
| 104.9018 | 121.4412 | 122.2155 | 123.9572 |
| 15.41853 | 15.52238 | 14.07232 | 14.52861 |
| 0.363481 | 0.390836 | 0 | 0.433978 |
| 0 | 0 | 0 | 0 |
| 17.59962 | 15.81276 | 15.68074 | 16.8804 |
| 7.337064 | 3.381098 | 5.04392 | 6.078418 |
| 61.09057 | 60.30379 | 61.89477 | 60.87306 |
| 0 | 0 | 0 | 0 |
| 38.16191 | 32.91064 | 33.68422 | 30.35838 |
| 2.132062 | 1.528343 | 2.611615 | 3.394095 |
| 17.88396 | 16.8742 | 15.14287 | 16.60159 |
| 0 | 0 | 0 | 0 |
| 8.639121 | 7.612867 | 7.099782 | 7.555326 |
| 0 | 0.227049 | 0.193989 | 0.441195 |
| 8.157331 | 7.458092 | 7.421468 | 7.743413 |
| 1.459496 | 1.196616 | 1.25144 | 1.154447 |
| 0.069921 | 0 | 0.042824 | 0.041741 |
| 0.449208 | 0 | 0 | 0 |
| 0.912016 | 1.043919 | 1.225262 | 0.684953 |

| | | | |
|---|---|---|---|
| 833.4466 | 859.844 | 837.3746 | 852.466 |
| 0.669336 | 0.592165 | 0.508537 | 0.617067 |
| 0.853334 | 0.602964 | 0.507703 | 0.436644 |
| 0 | 0 | 0 | 0 |
| 181.2902 | 163.5951 | 157.4442 | 160.0111 |
| 4.900833 | 3.353417 | 3.206228 | 3.45761 |
| 9.976236 | 8.895997 | 9.051953 | 8.70796 |
| 0 | 0 | 0 | 0 |
| 0 | 0 | 0 | 0 |
| 12.79237 | 12.76166 | 14.45093 | 12.21874 |
| 0 | 0.017594 | 0 | 0 |
| 3.934809 | 3.20715 | 3.165626 | 3.120372 |
| 3.285635 | 2.842883 | 2.840317 | 2.89108 |
| 5.942026 | 5.592088 | 4.568293 | 5.324258 |
| 0 | 0 | 0 | 0 |
| 0 | 0 | 0 | 0 |
| 5.300274 | 4.429236 | 3.669637 | 4.264691 |
| 4.536618 | 5.710864 | 4.066108 | 4.227501 |
| 2.681207 | 2.612339 | 2.359317 | 2.521779 |
| 66.88055 | 62.53372 | 61.95156 | 64.60069 |
| 0.430847 | 0.383397 | 0.345771 | 0.549886 |
| 0.036798 | 0.019783 | 0.022537 | 0 |
| 1.823137 | 0.999389 | 0.744402 | 1.024344 |
| 0.06496 | 0.267755 | 0.159143 | 0.168046 |
| 4.869823 | 4.535153 | 4.29118 | 4.033578 |
| 3.90878 | 3.990673 | 3.869059 | 4.101198 |
| 0 | 0 | 0 | 0 |
| 0.049842 | 0.053593 | 0.122106 | 0.079346 |
| 30.26356 | 36.4838 | 34.35038 | 37.61298 |
| 0 | 0.758511 | 0.432045 | 0.421119 |
| 1.400472 | 0.920252 | 0.95304 | 1.486303 |
| 9.751626 | 9.635328 | 8.788367 | 8.740942 |
| 8.363697 | 3.338356 | 7.838898 | 0.643026 |
| 24.70887 | 25.5009 | 24.9068 | 27.21828 |
| 4.356602 | 3.105628 | 3.478613 | 3.082404 |
| 12.87603 | 9.501495 | 9.927182 | 9.977577 |
| 0 | 0 | 0.071698 | 0.104827 |
| 0 | 0 | 0 | 0 |
| 0.046162 | 0.074454 | 0.056545 | 0.055115 |
| 8.02946 | 6.849107 | 6.978247 | 7.042787 |
| 474.3947 | 391.9387 | 384.9232 | 405.0552 |
| 39.04284 | 37.87018 | 36.32961 | 42.05044 |
| 48.41728 | 46.80979 | 46.36174 | 44.916 |
| 3.698162 | 3.234453 | 3.164476 | 3.042199 |
| 15.3209 | 11.86866 | 10.33822 | 10.97918 |
| 14.52536 | 11.6061 | 12.80605 | 12.26128 |
| 8.994993 | 11.56516 | 12.42476 | 12.31621 |
| 2.313267 | 1.769529 | 1.768609 | 1.631202 |
| 13.95942 | 11.27083 | 11.68347 | 11.29904 |
| 8.048842 | 6.308941 | 8.661374 | 8.532154 |
| 12.27775 | 11.21806 | 11.5717 | 10.7474 |
| 0 | 0 | 0 | 0.044242 |
| 0.398173 | 0.442902 | 0.487732 | 0.475398 |
| 0 | 0 | 0 | 0 |

| | | | |
|---|---|---|---|
| 0 | 0 | 0 | 0.070535 |
| 7.941864 | 5.091609 | 5.208453 | 4.971848 |
| 16.45314 | 15.41843 | 14.60154 | 14.07617 |
| 7.506396 | 7.127115 | 7.876273 | 7.305077 |
| 0.019973 | 0 | 0.024466 | 0 |
| 0 | 0 | 0 | 0 |
| 1.713456 | 0.581812 | 1.988388 | 0.323018 |
| 31.65244 | 26.17585 | 28.33845 | 26.57206 |
| 1.349746 | 0.241887 | 1.194077 | 0.716234 |
| 0 | 0.103433 | 0 | 0 |
| 0.055303 | 0 | 0.067742 | 0 |
| 2.077371 | 1.706305 | 1.896687 | 1.722412 |
| 0.522578 | 0.264426 | 0.677772 | 0.40372 |
| 1.587202 | 1.327394 | 0.648067 | 1.263358 |
| 3.531426 | 4.840375 | 4.75355 | 3.336005 |
| 7.484613 | 5.814546 | 5.559133 | 6.423605 |
| 0.067958 | 0 | 0.055496 | 0.027046 |
| 1.984002 | 1.939374 | 0.994194 | 1.076725 |
| 0.055756 | 0.029976 | 0.102446 | 0 |
| 1.521565 | 0.737837 | 1.20599 | 0.569936 |
| 331.0135 | 354.3471 | 351.5836 | 350.9817 |
| 0.807052 | 0.867788 | 0.54921 | 0.428257 |
| 0 | 0 | 0 | 0 |
| 2.723427 | 1.700352 | 1.937028 | 1.398551 |
| 93.61676 | 45.02962 | 45.12517 | 44.99152 |
| 14.02243 | 11.23891 | 11.53876 | 10.16849 |
| 0.349476 | 0.291226 | 0.214041 | 0.250353 |
| 0.075641 | 0.122 | 0.231636 | 0.225779 |
| 0.026571 | 0.028571 | 0 | 0.031725 |
| 0 | 0 | 0 | 0 |
| 30.89194 | 24.2953 | 22.44943 | 25.50281 |
| 0 | 0 | 0 | 0 |
| 0 | 0.070815 | 0.080672 | 0.078632 |
| 0.274085 | 0.107168 | 0.091564 | 0.148747 |
| 65.2842 | 55.37743 | 55.31771 | 55.3193 |
| 1.682956 | 1.279207 | 1.403948 | 1.507022 |
| 26.82636 | 27.16707 | 24.70832 | 23.59127 |
| 0.031061 | 0.033398 | 0.038047 | 0.07417 |
| 0 | 0 | 0.478082 | 0 |
| 0.096781 | 0 | 0.118549 | 0.115551 |
| 1.345143 | 1.0005 | 0.904376 | 1.159241 |
| 0 | 0.167319 | 0 | 0.185788 |
| 0.272438 | 0.211568 | 0.185398 | 0.234922 |
| 0 | 0 | 0 | 0 |
| 0.044647 | 0.048007 | 0 | 0.026653 |
| 5.153633 | 5.067849 | 5.071831 | 3.891749 |
| 26.05406 | 25.03623 | 22.10154 | 24.25408 |
| 11.82115 | 9.717211 | 10.06835 | 9.681833 |
| 0.483591 | 0.469255 | 0.332301 | 0.295733 |
| 0.059004 | 0 | 0.072275 | 0 |
| 4.601754 | 5.225855 | 5.379684 | 5.821983 |
| 0.040593 | 0.043648 | 0 | 0.048466 |
| 2.929106 | 2.303831 | 2.49162 | 2.514962 |
| 0 | 0 | 0.023008 | 0 |

| | | | |
|---|---|---|---|
| 0 | 0 | 0 | 0 |
| 0.804953 | 0.532634 | 0.68262 | 0.628393 |
| 1.785602 | 0.639994 | 2.187227 | 1.421277 |
| 102.7013 | 112.7551 | 110.2116 | 113.7608 |
| 0 | 0 | 0 | 0 |
| 0 | 0 | 0 | 0.902398 |
| 0 | 0 | 0 | 0 |
| 0.012485 | 0.006712 | 0.045878 | 0 |
| 2.463296 | 1.939062 | 1.992768 | 1.594213 |
| 0.474973 | 0.510718 | 0.145452 | 0 |
| 8.481871 | 7.53996 | 7.200748 | 7.219186 |
| 19.65755 | 17.32419 | 17.01344 | 18.49514 |
| 8.144201 | 7.908792 | 7.459374 | 7.790777 |
| 0.30478 | 0.176463 | 0.14359 | 0.055983 |
| 1029.913 | 964.936 | 960.9877 | 942.0055 |
| 8.332808 | 5.759942 | 2.916303 | 2.842554 |
| 0.077614 | 0.146046 | 0.118839 | 0.115834 |
| 11.08984 | 10.76057 | 10.83946 | 10.65604 |
| 0 | 0 | 0 | 0 |
| 27.02929 | 30.80092 | 30.6154 | 30.84341 |
| 2.537847 | 2.107419 | 2.369977 | 2.880055 |
| 15.29806 | 14.98956 | 14.96684 | 14.15347 |
| 1.07544 | 0.757625 | 0.408828 | 0.531319 |
| 0.016022 | 0.017227 | 0.03925 | 0 |
| 24.30098 | 20.79433 | 20.86202 | 21.29167 |
| 17.68114 | 14.77083 | 15.89772 | 14.89197 |
| 1.097325 | 0.884929 | 1.320137 | 0.795447 |
| 2.752003 | 3.716092 | 3.214205 | 5.27248 |
| 0.690088 | 0.602893 | 0.396237 | 0.46346 |
| 2.684067 | 2.286361 | 1.451747 | 1.914458 |
| 9.740149 | 10.61598 | 10.71074 | 9.752697 |
| 6.802292 | 3.37579 | 1.922837 | 0.624737 |
| 14.49184 | 14.84043 | 15.21549 | 9.063217 |
| 4.15951 | 4.596093 | 4.926187 | 3.923603 |
| 0.28199 | 0.187703 | 0.180933 | 0.176357 |
| 7.10224 | 6.439199 | 6.038357 | 6.626814 |
| 37.12699 | 40.48187 | 39.37305 | 39.00008 |
| 8.457834 | 6.976901 | 7.895971 | 6.732143 |
| 4.22833 | 3.189365 | 3.09217 | 3.616769 |
| 6.083579 | 4.910854 | 4.78584 | 4.579612 |
| 0 | 0.9309 | 0 | 0.516828 |
| 0.111178 | 0.068312 | 0.07782 | 0.018963 |
| 10.6948 | 9.920141 | 9.457687 | 9.560736 |
| 0.028377 | 0.030512 | 0 | 0 |
| 2.885821 | 0.77575 | 0.883728 | 0 |
| 5.090357 | 6.936827 | 4.87133 | 6.478576 |
| 0.059495 | 0.042648 | 0 | 0.071034 |
| 0 | 0.058347 | 0.132937 | 0.097181 |
| 57.07869 | 49.81572 | 53.13328 | 52.24154 |
| 2.797232 | 2.781598 | 2.653527 | 2.51109 |
| 0.106683 | 0 | 0 | 0 |
| 17.9414 | 15.26029 | 15.20903 | 16.02052 |
| 33.21572 | 33.61778 | 31.5055 | 33.74288 |
| 9.891256 | 7.760309 | 8.453052 | 7.44969 |

| | | | |
|---|---|---|---|
| 0.267924 | 0.256077 | 0.437582 | 0.142172 |
| 0.374929 | 0 | 0 | 0 |
| 0 | 0 | 0 | 0 |
| 0 | 0 | 0 | 0 |
| 20.60606 | 16.01394 | 16.89778 | 17.26599 |
| 8.130399 | 7.3966 | 7.696162 | 7.135609 |
| 1.15613 | 0.745882 | 0.687855 | 0.512705 |
| 60.61445 | 55.1268 | 52.24015 | 54.35002 |
| 51.25458 | 45.13071 | 48.00845 | 45.52876 |
| 70.62049 | 76.3079 | 73.39974 | 76.84867 |
| 38.09284 | 35.94675 | 32.65283 | 34.82426 |
| 0 | 0 | 0 | 0 |
| 45.7374 | 46.35142 | 46.6086 | 44.60549 |
| 33.30521 | 38.23641 | 31.58886 | 30.82454 |
| 0.145282 | 0 | 0.088979 | 0 |
| 0 | 0 | 0 | 0.054612 |
| 0 | 0 | 0 | 0 |
| 0 | 0 | 0 | 0 |
| 123.736 | 126.9041 | 124.0133 | 122.9944 |
| 5.822341 | 4.641415 | 5.492405 | 4.914043 |
| 1.738013 | 1.463407 | 1.250327 | 1.174791 |
| 0.190464 | 0.250309 | 0.129613 | 0.126336 |
| 3.714727 | 3.268052 | 3.10245 | 2.419195 |
| 0 | 0 | 0 | 0 |
| 30.8437 | 27.80137 | 27.58881 | 28.32152 |
| 0.335324 | 0.120187 | 0 | 0.40036 |
| 40.36693 | 40.47687 | 39.93577 | 42.87995 |
| 5.594655 | 6.453647 | 6.137603 | 5.954867 |
| 1.058669 | 0.517428 | 0.618922 | 0.890543 |
| 0.295982 | 0.119346 | 0.271916 | 0.220867 |
| 0 | 0 | 0 | 0 |
| 3.57186 | 4.094829 | 3.217102 | 3.198462 |
| 0 | 0 | 0 | 0 |
| 0 | 0.319997 | 0.364538 | 0 |
| 0.190188 | 0.130137 | 0.084715 | 0.26836 |
| 0.505806 | 0.74027 | 0.636785 | 0.704558 |
| 46.191 | 43.06252 | 42.37518 | 42.18362 |
| 0 | 0.029784 | 0 | 0 |
| 109.4958 | 129.1822 | 128.5833 | 128.14 |
| 4.84028 | 4.211452 | 3.938685 | 4.594646 |
| 2.400731 | 2.083063 | 2.463842 | 2.379401 |
| 0.028788 | 0 | 0 | 0 |
| 9.046395 | 8.135264 | 6.907384 | 8.162433 |
| 2174.823 | 2723.642 | 2692.226 | 2319.137 |
| 0 | 0.065809 | 0.074969 | 0 |
| 3.68962 | 2.841437 | 2.870497 | 2.887202 |
| 3.012444 | 2.789046 | 2.54547 | 2.820242 |
| 11.95635 | 11.73061 | 12.70807 | 12.33116 |
| 0.192934 | 0.124472 | 0.283595 | 0.092141 |
| 18.20614 | 23.34094 | 21.4434 | 13.37673 |
| 4.816167 | 3.847295 | 3.560229 | 4.059002 |
| 0 | 0 | 0 | 0 |
| 22.02524 | 20.23714 | 18.74174 | 19.45331 |
| 1.280001 | 0 | 1.567905 | 0.916953 |

| | | | |
|---|---|---|---|
| 0.52588 | 0.462646 | 0.585603 | 0.570794 |
| 0.026077 | 0 | 0 | 0 |
| 11.71808 | 11.10714 | 10.16303 | 9.945487 |
| 0 | 0 | 0 | 0 |
| 20.19775 | 19.04107 | 19.59506 | 18.96444 |
| 6.057296 | 5.301091 | 5.174086 | 4.984083 |
| 1.682546 | 2.231309 | 2.129691 | 2.522251 |
| 4.152276 | 4.213786 | 4.484305 | 4.180227 |
| 3.497224 | 3.374731 | 3.4051 | 2.355412 |
| 1.84532 | 1.570312 | 1.359 | 1.797716 |
| 0 | 0 | 0 | 0 |
| 15.63067 | 18.50142 | 18.36384 | 17.89945 |
| 0 | 0.153958 | 0.136412 | 0.189947 |
| 1.108962 | 0.745262 | 1.103695 | 0.910279 |
| 1.882583 | 1.533531 | 1.746987 | 1.339542 |
| 13.42782 | 12.22086 | 12.6869 | 12.80381 |
| 0 | 0 | 0 | 0 |
| 18.70339 | 11.70943 | 11.80527 | 13.81459 |
| 0.640198 | 0.361116 | 0.29568 | 0.400977 |
| 1.333099 | 1.049832 | 0.850972 | 0.85187 |
| 28.80971 | 31.40809 | 30.22499 | 26.75345 |
| 0.060426 | 0 | 0 | 0 |
| 0.173963 | 0.080166 | 0.213091 | 0.089015 |
| 0.113642 | 0.122194 | 0.083522 | 0.027137 |
| 29.03614 | 22.31329 | 22.95603 | 26.60093 |
| 107.015 | 103.7007 | 97.91858 | 90.57631 |
| 3.091718 | 3.575653 | 3.478867 | 3.605505 |
| 1.963757 | 1.327878 | 1.066335 | 1.764511 |
| 0 | 0 | 0 | 0 |
| 0.714956 | 0.845637 | 0.569248 | 0.512172 |
| 548.2138 | 587.5536 | 597.6494 | 606.7038 |
| 82.90685 | 87.75637 | 86.99432 | 84.60147 |
| 15.17697 | 15.43703 | 16.53898 | 17.58996 |
| 102.6335 | 110.1806 | 112.3205 | 106.4853 |
| 0.03903 | 0.041967 | 0.095616 | 0.186397 |
| 10.44416 | 8.933898 | 7.725035 | 9.083425 |
| 6.939445 | 5.342713 | 5.632478 | 4.86663 |
| 15.51047 | 15.52812 | 15.77252 | 16.92767 |
| 9.330404 | 8.884943 | 7.338203 | 8.838022 |
| 7.442727 | 7.839044 | 7.703945 | 7.145361 |
| 0.68692 | 0.470028 | 0.650192 | 0.782867 |
| 23.58475 | 21.07377 | 21.2721 | 20.7934 |
| 15.70839 | 17.19217 | 15.37609 | 16.3269 |
| 0.125968 | 0 | 0.308603 | 0.1504 |
| 5.406551 | 4.876142 | 4.741336 | 4.850649 |
| 0.072908 | 0.101913 | 0.062515 | 0.052229 |
| 17.41749 | 15.67641 | 14.98641 | 14.69162 |
| 0.730307 | 0.746005 | 0.760386 | 0.741157 |
| 11.89846 | 11.36175 | 9.190773 | 11.79428 |
| 0.009785 | 0 | 0 | 0 |
| 0.018392 | 0.019776 | 0 | 0 |
| 15.53835 | 14.99536 | 15.77284 | 14.80356 |
| 0 | 0 | 0 | 0 |
| 0 | 0 | 0 | 0 |

| | | | |
|---|---|---|---|
| 0 | 0 | 0.036499 | 0 |
| 13.07223 | 14.05601 | 17.6065 | 16.17255 |
| 0 | 0.02775 | 0.015807 | 0.015407 |
| 2.241161 | 1.835173 | 2.13285 | 2.67583 |
| 56.9743 | 53.51898 | 51.64246 | 56.01783 |
| 2.663835 | 1.074115 | 2.651185 | 2.18658 |
| 15.06989 | 16.33338 | 15.43817 | 15.83786 |
| 0 | 0.060199 | 0 | 0 |
| 8.953887 | 8.922809 | 9.974601 | 8.774842 |
| 0.666625 | 0.511995 | 0.466608 | 0.473759 |
| 0.781207 | 0.646152 | 0.708488 | 0.609855 |
| 0.764916 | 0.20562 | 0.468482 | 1.369906 |
| 5.882635 | 6.564037 | 5.166411 | 7.553641 |
| 0.217425 | 0 | 0 | 0.129797 |
| 0.604923 | 0.216816 | 1.834819 | 0.309534 |
| 1.329741 | 0.517167 | 1.005024 | 0.675592 |
| 1.763557 | 1.137766 | 0.86409 | 0 |
| 0.6953 | 0.360923 | 0.646109 | 0.543893 |
| 10.34598 | 12.67608 | 12.33459 | 12.64579 |
| 42.44468 | 44.27236 | 40.50346 | 41.99079 |
| 0.272548 | 0.142955 | 0.170996 | 0.055557 |
| 8.007423 | 7.688562 | 7.151339 | 7.35419 |
| 35.64444 | 41.63794 | 42.30019 | 41.06908 |
| 0 | 0.070008 | 0.026584 | 0 |
| 2.16261 | 2.200789 | 2.317905 | 2.028749 |
| 2.147243 | 1.701249 | 1.568897 | 1.889039 |
| 97.58809 | 91.19908 | 90.98258 | 94.60376 |
| 0 | 0 | 0 | 0.256086 |
| 0.132267 | 0 | 0 | 0 |
| 4.792524 | 2.216428 | 2.903678 | 2.953303 |
| 13.00742 | 12.95681 | 12.87235 | 12.4353 |
| 0 | 0 | 0.045214 | 0 |
| 13.56683 | 16.05608 | 14.51407 | 13.51594 |
| 1.375744 | 1.369703 | 1.810011 | 1.581732 |
| 45.76556 | 41.10518 | 45.38928 | 44.99588 |
| 26.37388 | 26.11809 | 26.5915 | 23.28258 |
| 6.859096 | 6.827489 | 6.75803 | 6.958027 |
| 10.91559 | 9.68539 | 9.812254 | 10.98026 |
| 107.0894 | 75.57883 | 73.92983 | 73.33567 |
| 8.970271 | 7.951889 | 8.136082 | 8.134723 |
| 0 | 0 | 0.857736 | 0 |
| 0.805435 | 0.820468 | 0.649077 | 0.847768 |
| 2.318211 | 3.340181 | 2.385292 | 1.328555 |
| 0.127103 | 0.102501 | 0.019461 | 0 |
| 4.783402 | 4.049603 | 4.301769 | 3.947189 |
| 0 | 0 | 0.22607 | 0 |
| 4.76378 | 5.309403 | 4.742823 | 5.064396 |
| 7.42592 | 5.803629 | 5.986631 | 4.857978 |
| 0.517191 | 0.370742 | 0.802458 | 0.617499 |
| 0.067445 | 0.435123 | 0.413074 | 0.241577 |
| 0.444821 | 0.304371 | 0.421037 | 0.555234 |
| 0.256376 | 1.804387 | 0.028549 | 0.083482 |
| 1.690805 | 1.283329 | 1.005097 | 1.128116 |
| 0 | 0.015817 | 0.018019 | 0 |

| | | | |
|---|---|---|---|
| 0.101962 | 0.054817 | 0.187343 | 0.060868 |
| 1.78088 | 1.740821 | 1.5204 | 1.610817 |
| 0 | 0 | 0 | 0 |
| 43.27448 | 46.22756 | 45.09569 | 48.52782 |
| 0 | 0 | 0 | 0 |
| 0.098177 | 0.117296 | 0.093535 | 0.09117 |
| 0.91516 | 0.298192 | 0.305728 | 0.297996 |
| 2.17068 | 1.736525 | 1.808065 | 1.700142 |
| 0 | 0 | 0 | 0 |
| 0.287276 | 0.154448 | 0.175946 | 0.085748 |
| 22.35643 | 17.47742 | 17.6677 | 17.90533 |
| 0 | 0 | 0 | 0 |
| 2.857354 | 2.840909 | 2.637019 | 3.224599 |
| 0.469171 | 0.504479 | 0.457413 | 0.52587 |
| 5.122956 | 2.787036 | 2.876149 | 3.058272 |
| 0.479446 | 0.544168 | 0.587285 | 0.466427 |
| 4.056769 | 2.9974 | 3.28969 | 3.152381 |
| 3.619026 | 3.576367 | 3.504208 | 3.549335 |
| 0 | 0 | 0 | 0 |
| 17.03843 | 14.63319 | 14.54112 | 13.94181 |
| 0.086261 | 0.139129 | 0.264158 | 0.257478 |
| 6.257237 | 5.661482 | 4.991365 | 5.830881 |
| 0.161176 | 0.222822 | 0.14102 | 0.247418 |
| 942.459 | 1054.088 | 1046.697 | 1041.992 |
| 25.40432 | 22.29892 | 22.02686 | 23.00107 |
| 1.382306 | 0.853852 | 0.756546 | 0.807644 |
| 5.787335 | 3.62431 | 3.883952 | 4.122002 |
| 20.04669 | 23.19977 | 20.55589 | 24.5269 |
| 48.58659 | 53.62742 | 53.1825 | 57.4896 |
| 44.21916 | 41.86525 | 41.12267 | 41.36349 |
| 56.98713 | 34.23659 | 32.05138 | 32.96637 |
| 0 | 0 | 0 | 0 |
| 120.2909 | 144.2414 | 145.8544 | 145.1017 |
| 0.078091 | 0.230912 | 0.167397 | 0.163164 |
| 0.029839 | 0.06417 | 0.063964 | 0.035627 |
| 3.294192 | 6.440187 | 4.035136 | 2.145324 |
| 3.408264 | 2.957526 | 3.125046 | 3.521959 |
| 1.529335 | 1.229617 | 1.232003 | 1.085698 |
| 0.45493 | 0.244584 | 0.185752 | 0.452636 |
| 13.17516 | 11.45025 | 10.34122 | 11.07241 |
| 0 | 0 | 0 | 0 |
| 12.94074 | 10.78383 | 11.34818 | 10.99098 |
| 0.058353 | 0 | 0 | 0 |
| 7.247185 | 5.914855 | 5.70426 | 7.019511 |
| 0 | 0.180917 | 0.206099 | 0.200887 |
| 0.070438 | 0.037869 | 0 | 0 |
| 1.35081 | 0 | 0 | 0 |
| 46.62567 | 46.1552 | 42.77883 | 45.99119 |
| 0.506554 | 1.089351 | 0 | 0.604799 |
| 1.503665 | 0 | 0.613959 | 0.598433 |
| 53.29375 | 46.92974 | 46.46458 | 48.24233 |
| 70.0749 | 68.69582 | 66.90637 | 70.42115 |
| 14.99372 | 11.66998 | 11.45004 | 11.16049 |
| 8.772753 | 8.940932 | 7.959377 | 8.50737 |

| | | | |
|---|---|---|---|
| 0.44501 | 0 | 0 | 0.531319 |
| 0.272024 | 0.114455 | 0.188336 | 0.225936 |
| 5.315577 | 3.753022 | 3.64783 | 4.473152 |
| 0 | 0 | 0 | 0 |
| 34.81603 | 29.5911 | 25.55685 | 27.96707 |
| 0.145689 | 0.391633 | 0.29743 | 0.637799 |
| 12.45367 | 10.61263 | 10.98897 | 11.55416 |
| 29.73448 | 20.32598 | 23.53068 | 21.34966 |
| 1.988605 | 1.583897 | 1.037509 | 1.055241 |
| 6.547974 | 5.346573 | 5.388957 | 5.692438 |
| 0.164193 | 0.17655 | 0 | 0 |
| 6.306053 | 4.48211 | 4.254988 | 6.380593 |
| 0.149736 | 0 | 0 | 0 |
| 0 | 0 | 0 | 0.124673 |
| 19.20565 | 16.70104 | 15.86045 | 17.64402 |
| 14.94651 | 10.90954 | 9.98059 | 8.550899 |
| 0.026834 | 0.043279 | 0.032869 | 0.048057 |
| 32.92333 | 30.18711 | 30.59059 | 28.64917 |
| 1.409801 | 1.553796 | 1.55421 | 1.346584 |
| 22.23854 | 22.3049 | 23.61931 | 24.6282 |
| 28.01844 | 23.63596 | 23.71087 | 25.22653 |
| 0.063573 | 0 | 0 | 0.037951 |
| 5.766742 | 4.317331 | 4.951278 | 4.182593 |
| 0 | 0 | 0 | 0 |
| 396.3058 | 369.5646 | 369.123 | 339.8181 |
| 7.276506 | 5.38942 | 4.227697 | 4.947567 |
| 0 | 0.118517 | 0 | 0.263199 |
| 3.919563 | 3.303769 | 3.377093 | 3.37101 |
| 4.577392 | 4.291633 | 3.6582 | 3.632338 |
| 0.457847 | 0.492303 | 0.373885 | 1.09329 |
| 0.465303 | 0.667094 | 0.379974 | 0.370365 |
| 0 | 0 | 0 | 0 |
| 56.33009 | 61.99153 | 56.70589 | 61.2171 |
| 54.90478 | 54.40564 | 51.96814 | 50.71987 |
| 5.499939 | 5.612231 | 5.816653 | 5.250921 |
| 1.015977 | 0.783074 | 0.825992 | 0.633349 |
| 16.27466 | 15.23523 | 15.19637 | 15.24085 |
| 0.375352 | 1.037827 | 0.459777 | 1.024344 |
| 1.186961 | 1.091319 | 0.779647 | 0.985857 |
| 0.380928 | 0 | 0 | 0 |
| 0.432873 | 0.9309 | 0 | 0 |
| 0.391035 | 0.38812 | 0.331607 | 0.323222 |
| 0.225567 | 0.323389 | 0.171044 | 0.218017 |
| 0.442499 | 0.382339 | 0.367805 | 0.433978 |
| 11.91714 | 10.07619 | 11.41442 | 11.59587 |
| 93.13138 | 105.4107 | 81.48494 | 107.8499 |
| 27.90714 | 23.06203 | 25.46059 | 23.03705 |
| 1.152001 | 1.30222 | 1.302567 | 1.37543 |
| 39.34312 | 46.73916 | 48.66469 | 46.35979 |
| 6.14576 | 3.479577 | 4.063837 | 3.798731 |
| 10.42111 | 9.027059 | 9.442366 | 9.900514 |
| 0.109588 | 0.039278 | 0 | 0.065421 |
| 9.053179 | 10.45964 | 9.6626 | 9.174253 |
| 1.741763 | 1.366669 | 0.922607 | 0.983583 |

| | | | |
|---|---|---|---|
| 0.677464 | 0.728448 | 0.616454 | 0.855077 |
| 0.007559 | 0 | 0 | 0 |
| 19.35829 | 18.98598 | 19.06257 | 18.43006 |
| 0.371209 | 0.290288 | 0.62005 | 0.362622 |
| 2.107729 | 1.590202 | 1.283772 | 1.54328 |
| 0.06452 | 0 | 0.039516 | 0.038517 |
| 27.54336 | 25.1158 | 24.52523 | 24.23291 |
| 9.792916 | 9.701319 | 9.478476 | 10.08216 |
| 0 | 0.028492 | 0 | 0.158183 |
| 18.10224 | 18.48246 | 17.25384 | 16.63357 |
| 4.611907 | 3.950929 | 3.537719 | 3.267719 |
| 0.036155 | 0 | 0 | 0 |
| 14.28288 | 13.46918 | 13.75994 | 13.11239 |
| 0.051035 | 0.13719 | 0.187544 | 0.0914 |
| 0.054232 | 0.011663 | 0.013286 | 0.03885 |
| 20.04886 | 20.66931 | 20.37533 | 19.43262 |
| 0.347562 | 0.373719 | 0 | 0 |
| 0 | 0.211568 | 0 | 0 |
| 1.551643 | 1.390346 | 0.98992 | 1.698199 |
| 21.11191 | 18.6716 | 19.44202 | 20.15067 |
| 951.1078 | 1008.01 | 976.5343 | 979.8665 |
| 0.598944 | 0 | 0.366831 | 0 |
| 316.8497 | 403.0074 | 392.6384 | 406.2429 |
| 23.08657 | 22.42069 | 22.83831 | 23.44305 |
| 1.566608 | 2.645386 | 3.17577 | 3.09546 |
| 0 | 0 | 0 | 0 |
| 7.110189 | 6.020658 | 7.675198 | 6.685242 |
| 11.24178 | 6.460722 | 8.072263 | 6.865328 |
| 5.732979 | 4.829105 | 5.417926 | 5.402783 |
| 8.656231 | 8.084129 | 8.429485 | 7.278233 |
| 60.07659 | 56.29767 | 57.19283 | 60.26837 |
| 23.10364 | 21.00492 | 23.06583 | 21.97794 |
| 0 | 0 | 0 | 0 |
| 11.90401 | 9.752283 | 5.554863 | 14.21277 |
| 2.661767 | 3.498102 | 4.166147 | 3.354567 |
| 1.093322 | 0.648608 | 0.507986 | 0.810229 |
| 17.76627 | 15.78528 | 14.8201 | 15.48494 |
| 1.360458 | 1.462842 | 0 | 0 |
| 45.6608 | 43.77927 | 44.23764 | 43.02739 |
| 0.241706 | 0.121285 | 0.217119 | 0.327062 |
| 4.115874 | 4.007898 | 4.025591 | 3.760822 |
| 0.128 | 0.137633 | 0 | 0.152826 |
| 3.504132 | 2.86513 | 3.062733 | 3.050652 |
| 13.49313 | 13.23784 | 13.42202 | 11.74009 |
| 79.88027 | 35.00453 | 36.23151 | 33.92866 |
| 0.0992 | 0 | 0 | 0.11844 |
| 2.959189 | 3.847193 | 3.130495 | 2.922853 |
| 9.458889 | 9.94698 | 9.083757 | 9.350954 |
| 6.174991 | 3.098528 | 2.843465 | 3.208455 |
| 0.264534 | 0 | 0.324034 | 0.315839 |
| 0 | 0 | 0 | 0 |
| 0 | 0 | 0 | 0 |
| 46.91285 | 41.80211 | 44.29827 | 46.17652 |
| 0.257037 | 0.069095 | 0.078713 | 0.383611 |

| | | | |
|---|---|---|---|
| 6.450271 | 6.312265 | 8.433753 | 7.441695 |
| 0.179008 | 0.12832 | 0.365452 | 0.071242 |
| 27.65737 | 26.46907 | 26.78328 | 26.45175 |
| 8.003295 | 7.316036 | 7.418028 | 6.762586 |
| 33.99336 | 33.17917 | 31.9765 | 31.47046 |
| 0 | 0 | 0 | 0 |
| 1093.708 | 1769.211 | 1793.691 | 1737.127 |
| 3.236042 | 2.659391 | 2.519912 | 2.952945 |
| 0 | 0 | 0.326757 | 0 |
| 0.019692 | 0 | 0 | 0 |
| 0.112567 | 0 | 0 | 0 |
| 7.760922 | 5.071538 | 4.070481 | 4.27471 |
| 106.4491 | 60.60717 | 61.82975 | 62.07416 |
| 2.338288 | 2.034265 | 1.97892 | 1.979636 |
| 0.159358 | 0.13708 | 0.03904 | 0.228318 |
| 0 | 0.228569 | 0 | 0 |
| 0 | 0 | 0.069189 | 0 |
| 2.86179 | 2.595195 | 2.44961 | 3.046329 |
| 0 | 0.033622 | 0.00766 | 0.037333 |
| 8.052371 | 6.205998 | 6.974801 | 6.891041 |
| 10.05544 | 6.814949 | 6.743329 | 6.839593 |
| 0 | 0 | 0 | 0 |
| 4905.72 | 4905.104 | 4860.656 | 5043.234 |
| 24.51871 | 25.13578 | 25.86742 | 24.6981 |
| 190.1663 | 208.4284 | 209.3413 | 207.4264 |
| 0.452588 | 0.304155 | 0.461989 | 0.405275 |
| 0 | 0 | 0 | 0.087396 |
| 2.900806 | 3.404163 | 2.885079 | 3.110375 |
| 37.12658 | 30.7097 | 32.1277 | 31.74787 |
| 9.139073 | 8.941754 | 8.247346 | 8.189984 |
| 2.952384 | 2.615847 | 2.02521 | 2.030396 |
| 0.379512 | 0.136024 | 0.216941 | 0.151039 |
| 0.109651 | 0.088427 | 0.033579 | 0.032729 |
| 14.9404 | 13.13378 | 13.44035 | 12.93568 |
| 1.110683 | 0.963121 | 0.833856 | 1.026656 |
| 8.372582 | 7.55119 | 7.263691 | 7.693988 |
| 10.00213 | 8.685097 | 9.487856 | 8.489176 |
| 0.470411 | 0.355942 | 0.405487 | 0.332827 |
| 1.467932 | 1.183803 | 10.33911 | 1.643095 |
| 2.330855 | 3.938422 | 1.223624 | 1.19268 |
| 2.747564 | 2.62909 | 3.056792 | 2.76882 |
| 1.480292 | 1.326412 | 1.133278 | 1.288723 |
| 0.143422 | 0.359836 | 0 | 0.057079 |
| 21.31725 | 21.20848 | 20.18475 | 22.57009 |
| 2.098025 | 11.37766 | 1.787772 | 3.049484 |
| 0.148992 | 0.084814 | 0.085884 | 0.15696 |
| 16.48424 | 15.05867 | 14.79243 | 14.69247 |
| 22.23286 | 18.0391 | 16.80521 | 18.612 |
| 3.780791 | 3.613619 | 3.579658 | 3.162028 |
| 4.688451 | 3.485999 | 3.910127 | 3.771546 |
| 160.7782 | 137.8775 | 139.4017 | 139.238 |
| 0.149267 | 0.066875 | 0.152367 | 0.044554 |
| 55.71414 | 47.45907 | 45.51998 | 45.51511 |
| 54.06774 | 51.93402 | 54.97897 | 54.70633 |

| | | | |
|---|---|---|---|
| 0.638427 | 0.171618 | 0.130338 | 0.158802 |
| 2891.285 | 2937.776 | 2944.627 | 2958.277 |
| 13.03635 | 11.10633 | 11.30058 | 11.59695 |
| 3.384847 | 1.799343 | 1.677107 | 2.679085 |
| 5.392146 | 4.742821 | 4.915051 | 4.338361 |
| 12.1492 | 10.5455 | 10.37071 | 10.32353 |
| 8.645464 | 6.422112 | 7.63938 | 6.421848 |
| 37.27312 | 41.94469 | 39.04154 | 38.83145 |
| 0.133753 | 0 | 0 | 0 |
| 0 | 0 | 0 | 0 |
| 12.15183 | 6.799645 | 6.489978 | 6.672759 |
| 19.34517 | 14.5746 | 14.27656 | 14.99442 |
| 0 | 0 | 0.583261 | 0 |
| 31.92166 | 36.57898 | 35.50099 | 34.87004 |
| 1.208529 | 0.519792 | 0.148036 | 0.288584 |
| 0.027564 | 0.103733 | 0 | 0.032909 |
| 0.216109 | 0.464746 | 0.97063 | 0.344031 |
| 14.17678 | 12.69362 | 12.84658 | 12.17563 |
| 1.65923 | 1.452173 | 1.465241 | 1.796752 |
| 2.900545 | 2.952599 | 2.579043 | 3.181833 |
| 1.421374 | 1.146257 | 0.435269 | 0.848524 |
| 0.711868 | 0.680392 | 0.871984 | 0.661059 |
| 1.110578 | 0.916942 | 1.020281 | 0.970802 |
| 2.348904 | 1.278428 | 1.420854 | 1.835023 |
| 1.040788 | 0.699447 | 0.517923 | 0.543658 |
| 12.31349 | 12.17089 | 10.03151 | 11.38419 |
| 0.457847 | 0 | 0.186942 | 0.182215 |
| 0.23825 | 0.146389 | 0.041691 | 0.203185 |
| 2.354502 | 2.747158 | 3.467041 | 2.811153 |
| 9.614595 | 7.39025 | 7.859209 | 7.683194 |
| 6.963771 | 5.773236 | 5.167506 | 5.61522 |
| 0.297077 | 0.27029 | 0.261259 | 0.181894 |
| 2.246818 | 1.263364 | 1.237219 | 1.796593 |
| 0.967369 | 0.554757 | 0.737305 | 0.538995 |
| 14.80552 | 11.13032 | 11.25793 | 11.72226 |
| 72.13584 | 67.16223 | 69.60694 | 77.24672 |
| 2298.797 | 1637.672 | 1627.459 | 1543.665 |
| 139.4241 | 125.231 | 124.2687 | 125.9383 |
| 0 | 0.237035 | 0.270028 | 0 |
| 44.25886 | 45.60424 | 44.27572 | 45.96187 |
| 0.301749 | 0.324458 | 0.73924 | 0.93671 |
| 1.51567 | 1.339287 | 1.01101 | 1.415454 |
| 0 | 0 | 0 | 0 |
| 0.038712 | 0 | 0 | 0 |
| 6.580842 | 5.189137 | 4.872447 | 4.784152 |
| 0.167196 | 0.277159 | 0.136535 | 0.158035 |
| 0.160774 | 0.259311 | 0.131291 | 0.223949 |
| 28.85589 | 25.1188 | 25.48439 | 25.84695 |
| 48.1589 | 49.78191 | 48.7792 | 53.61039 |
| 3.208386 | 2.901734 | 3.048528 | 2.899835 |
| 11.7633 | 9.844503 | 10.64025 | 9.542381 |
| 0 | 0 | 0 | 0 |
| 19.63466 | 24.33186 | 24.24572 | 20.8802 |
| 0.096584 | 0.069235 | 0.078872 | 0.422828 |

| | | | |
|---|---|---|---|
| 0.735699 | 0.593299 | 0.625816 | 0.536791 |
| 2.750191 | 2.087408 | 2.311905 | 1.995905 |
| 0.250611 | 0.808413 | 0 | 0 |
| 10.64442 | 6.638383 | 6.258534 | 6.862799 |
| 7.785355 | 5.39272 | 5.821891 | 5.98799 |
| 53.04123 | 54.34923 | 53.10857 | 54.12095 |
| 15.64103 | 15.71821 | 15.9659 | 16.90167 |
| 6.886348 | 6.022091 | 5.659096 | 5.43793 |
| 9.770123 | 7.598672 | 7.769855 | 8.564513 |
| 0 | 0 | 0 | 0 |
| 0 | 0 | 0.146181 | 0 |
| 0 | 0.158758 | 0 | 0 |
| 6.854658 | 5.242134 | 6.1065 | 5.120537 |
| 63.71044 | 64.64944 | 59.24538 | 63.32275 |
| 0.345044 | 0.556516 | 0.422653 | 0.411964 |
| 4.111848 | 3.983691 | 3.541164 | 4.054808 |
| 0.383118 | 0.568883 | 0.379902 | 0.653461 |
| 2.595798 | 2.088036 | 1.723325 | 2.294863 |
| 0.612604 | 0.299412 | 0.204653 | 0.465448 |
| 3.597383 | 3.404468 | 3.606708 | 3.383118 |
| 25.95438 | 36.71309 | 34.70441 | 32.80174 |
| 3.909289 | 10.94863 | 3.22951 | 3.256387 |
| 0.811043 | 0.675158 | 0.640946 | 0.812158 |
| 194.7283 | 167.3535 | 161.9201 | 158.6739 |
| 0.100139 | 0.080756 | 0.030666 | 0.05978 |
| 33.12988 | 28.70699 | 28.4094 | 28.31425 |
| 0 | 0 | 0 | 0 |
| 0 | 0 | 0 | 0 |
| 205.6788 | 173.5935 | 169.9164 | 173.1323 |
| 1.082183 | 1.745437 | 1.10466 | 0.646035 |
| 0.075401 | 0 | 0 | 0.135038 |
| 4.563396 | 4.559877 | 4.488792 | 4.485347 |
| 0.122564 | 0 | 0 | 0 |
| 7.953922 | 7.902404 | 8.788407 | 7.748046 |
| 0 | 0 | 0.41366 | 0 |
| 9.442275 | 8.785343 | 8.261482 | 6.994225 |
| 7.317617 | 6.405477 | 6.110349 | 6.201937 |
| 0.155948 | 0.083842 | 0.063675 | 0.186194 |
| 0.197475 | 0.477756 | 0.453546 | 0.618908 |
| 0.305231 | 0.656404 | 0.373885 | 0 |
| 0 | 0 | 0 | 0 |
| 1.540972 | 0.994165 | 1.321302 | 2.575777 |
| 1.014649 | 0.623434 | 1.242869 | 1.038376 |
| 4.330497 | 3.84205 | 3.163689 | 3.245984 |
| 2.728285 | 2.203855 | 2.311095 | 2.560568 |
| 2.873239 | 2.375142 | 2.705743 | 2.359707 |
| 0.175867 | 0.165465 | 0.080784 | 0.104988 |
| 5.858622 | 4.157404 | 4.109956 | 3.912131 |
| 7.520312 | 7.740875 | 6.874143 | 7.444785 |
| 0.058822 | 0 | 0.036026 | 0 |
| 0.670649 | 1.442239 | 0 | 2.402159 |
| 0 | 0.068724 | 0.15658 | 0.15262 |
| 0 | 0 | 0 | 0 |
| 10.06648 | 121.2981 | 108.0402 | 100.9202 |

| | | | |
|---|---|---|---|
| 0 | 0 | 0 | 0.141421 |
| 0.598944 | 0 | 0 | 0 |
| 0 | 0 | 0 | 0 |
| 0 | 0.616861 | 0 | 0 |
| 18.05831 | 20.52999 | 20.29023 | 19.72138 |
| 0.336887 | 0.441756 | 0.543504 | 0.441467 |
| 19.29893 | 12.79987 | 12.76987 | 13.95436 |
| 16.3962 | 13.9748 | 14.10703 | 14.63384 |
| 0 | 0 | 0.102747 | 0 |
| 6.949963 | 7.13009 | 7.125747 | 6.499143 |
| 7.193103 | 6.273943 | 6.514522 | 6.030007 |
| 0.721455 | 1.066656 | 1.325592 | 0.43069 |
| 8.071326 | 8.644514 | 8.073216 | 8.249205 |
| 11.49819 | 11.37443 | 10.50532 | 10.85339 |
| 0 | 0 | 0 | 0 |
| 294.6131 | 297.9925 | 307.7956 | 297.8827 |
| 0 | 0 | 0 | 0 |
| 0 | 0 | 0 | 0 |
| 0.191229 | 0.10281 | 0 | 0.114159 |
| 88.94033 | 90.38387 | 86.62591 | 87.86077 |
| 34.8807 | 30.68057 | 32.153 | 30.71238 |
| 0.800596 | 0.551611 | 0.685517 | 0.705302 |
| 32.32024 | 26.02218 | 29.19511 | 27.95648 |
| 0 | 0 | 0.720075 | 0 |
| 0 | 0 | 0.20182 | 0 |
| 0.472616 | 0.444661 | 0.14473 | 0.493744 |
| 0.183139 | 0 | 0 | 0 |
| 96.45856 | 85.64278 | 82.8937 | 86.65484 |
| 0.604648 | 0.487614 | 0.462905 | 0.09024 |
| 8.491941 | 8.112981 | 8.046819 | 8.347667 |
| 0.594418 | 0.470955 | 0.597823 | 0.537882 |
| 2.658308 | 2.472099 | 2.332162 | 2.594863 |
| 8.202515 | 7.482512 | 7.399575 | 7.424582 |
| 0 | 0.111546 | 0 | 0 |
| 0 | 0 | 0.540056 | 0 |
| 0.155608 | 0.146404 | 0.095304 | 0.185788 |
| 16.58483 | 18.65051 | 24.79731 | 18.89362 |
| 0 | 0 | 0 | 0 |
| 0 | 0 | 0.030909 | 0 |
| 8.919332 | 9.142765 | 8.183503 | 9.924078 |
| 1.892993 | 1.40916 | 1.070203 | 0.347713 |
| 0 | 0.022949 | 0 | 0 |
| 0 | 0 | 0 | 0 |
| 0.377905 | 0 | 0.462905 | 0 |
| 7.936008 | 8.533248 | 3.534913 | 11.19794 |
| 1.336591 | 0.598824 | 0 | 0.132985 |
| 0.346365 | 0.705659 | 0.424271 | 0.718256 |
| 5.269165 | 4.740693 | 3.898942 | 4.390938 |
| 3.761022 | 3.669052 | 3.440793 | 3.213102 |
| 0.120662 | 0.064871 | 0.129326 | 0.162072 |
| 0 | 0 | 0 | 0 |
| 9.465493 | 8.191918 | 7.776808 | 7.786876 |
| 21.80272 | 18.81401 | 19.89155 | 17.5107 |
| 0.029248 | 0 | 0 | 0 |

| | | | |
|---|---|---|---|
| 3.946597 | 3.942285 | 4.204968 | 3.708286 |
| 7.654833 | 6.651367 | 6.24169 | 6.015336 |
| 21.82081 | 22.98583 | 18.88271 | 22.29816 |
| 0.019692 | 0.211743 | 0 | 0.14107 |
| 0 | 0 | 0 | 0 |
| 0.096978 | 0.208552 | 0.316774 | 0.038595 |
| 0.744001 | 0 | 1.822689 | 0 |
| 8.801327 | 8.706127 | 8.019335 | 8.580075 |
| 7.717431 | 5.396556 | 6.734683 | 5.570684 |
| 14.9133 | 13.45848 | 10.60177 | 10.906 |
| 99.77664 | 115.3812 | 111.9578 | 114.4715 |
| 1.809784 | 1.434324 | 1.2422 | 1.248042 |
| 0 | 0 | 0.027409 | 0 |
| 3.759161 | 5.448 | 3.937343 | 2.992163 |
| 0.118448 | 0 | 0 | 0 |
| 5.494694 | 4.11331 | 4.07243 | 4.085705 |
| 0 | 0 | 0 | 0 |
| 7.958175 | 7.985022 | 8.471942 | 8.019497 |
| 3.530798 | 3.194955 | 3.01529 | 3.117161 |
| 3.997288 | 4.061952 | 5.577923 | 4.685145 |
| 5.838928 | 4.951936 | 5.641208 | 5.105797 |
| 7.274674 | 5.925866 | 6.075631 | 3.947992 |
| 0.110222 | 0.059259 | 0.270028 | 0 |
| 0.07023 | 0 | 0 | 0 |
| 22.7172 | 20.52069 | 20.96208 | 21.42371 |
| 3.937814 | 3.556696 | 3.395761 | 3.573175 |
| 0.601213 | 0.77575 | 0.589152 | 0.143563 |
| 19.12657 | 17.1579 | 18.252 | 18.87787 |
| 3.271331 | 1.954179 | 4.007134 | 4.339778 |
| 0 | 0 | 0 | 0 |
| 0 | 0 | 0 | 0 |
| 183.5126 | 207.2607 | 202.3447 | 204.7599 |
| 31.77289 | 30.81278 | 31.49607 | 29.90712 |
| 0.341198 | 0.285347 | 0.185752 | 0.090527 |
| 10.82975 | 10.0838 | 9.922543 | 9.775614 |
| 0.236896 | 0.076417 | 0.203126 | 0.254557 |
| 0.496575 | 0.355964 | 0.540682 | 0.395257 |
| 1.762178 | 0.974085 | 0.942459 | 1.022342 |
| 4.004605 | 4.028174 | 3.481208 | 3.59368 |
| 3.973917 | 4.196679 | 3.071318 | 3.417278 |
| 8.704008 | 6.537569 | 5.017295 | 5.501718 |
| 349.0755 | 388.735 | 381.5473 | 391.7769 |
| 0 | 0.609518 | 0 | 0 |
| 0.837573 | 0.090061 | 0.102596 | 0.050001 |
| 0 | 0 | 0 | 0.536331 |
| 0 | 0 | 0 | 0 |
| 2.276102 | 1.664228 | 1.394026 | 1.340657 |
| 25.34336 | 20.75714 | 19.75965 | 20.5798 |
| 1.364791 | 1.576205 | 2.04327 | 1.207029 |
| 0 | 0 | 0 | 0 |
| 0.123039 | 0.264597 | 0 | 0.293804 |
| 9.2277 | 7.209295 | 7.546873 | 7.089743 |
| 0.51292 | 0.4596 | 0.733003 | 0.714466 |
| 0.867007 | 0.559353 | 0.807134 | 0.621097 |

| | | | |
|---|---|---|---|
| 0 | 0 | 0 | 0 |
| 9. 203995 | 9. 210187 | 9. 221383 | 9. 305793 |
| 4. 1617 | 3. 879831 | 3. 0912 | 3. 224469 |
| 0. 933648 | 1. 003911 | 0 | 1. 114727 |
| 6. 340729 | 5. 384848 | 5. 936495 | 5. 609564 |
| 0 | 0. 151031 | 0. 172053 | 0. 167702 |
| 1. 400472 | 0. 669274 | 1. 143648 | 1. 114727 |
| 0. 530694 | 1. 121588 | 1. 098377 | 1. 070601 |
| 0. 630058 | 0. 627291 | 0. 971863 | 0. 752256 |
| 1. 383775 | 1. 678672 | 1. 564633 | 1. 1438 |
| 7. 116051 | 8. 426189 | 8. 41531 | 7. 636087 |
| 0. 309195 | 0 | 0 | 0 |
| 12. 54914 | 9. 958623 | 10. 04577 | 9. 496284 |
| 11. 67509 | 12. 47167 | 11. 63717 | 12. 98282 |
| 2. 99144 | 3. 098528 | 2. 991942 | 2. 588609 |
| 2. 267431 | 1. 393183 | 1. 785492 | 1. 353597 |
| 0. 14473 | 0. 103748 | 0. 059094 | 0. 1728 |
| 0. 052789 | 0. 028381 | 0 | 0. 063028 |
| 11. 70743 | 10. 59859 | 10. 9258 | 10. 49777 |
| 2. 438932 | 1. 888728 | 1. 857518 | 1. 478611 |
| 0. 696141 | 0. 374265 | 0. 596904 | 0. 498694 |
| 0 | 0 | 0. 205736 | 0. 200533 |
| 4. 239348 | 3. 816964 | 3. 941584 | 3. 155853 |
| 0. 842762 | 0 | 0 | 0 |
| 0. 260281 | 0. 131703 | 0. 06564 | 0. 109681 |
| 8. 711336 | 8. 156234 | 9. 255224 | 7. 95986 |
| 6. 889578 | 6. 286663 | 5. 677758 | 6. 263682 |
| 1. 382744 | 0. 648095 | 0. 694875 | 0. 465646 |
| 0 | 0. 04794 | 0. 054612 | 0 |
| 1. 50803 | 1. 135064 | 0. 946702 | 1. 012787 |
| 349. 0743 | 328. 7029 | 326. 2964 | 330. 803 |
| 0 | 0. 04966 | 0 | 0 |
| 0 | 0 | 0 | 0 |
| 0 | 0. 09326 | 0 | 0 |
| 96. 91389 | 102. 738 | 99. 01267 | 100. 1483 |
| 0 | 0 | 0 | 0 |
| 0. 035297 | 0 | 0 | 0 |
| 6. 496766 | 8. 63449 | 8. 798338 | 8. 624021 |
| 0 | 0. 037814 | 0. 043077 | 0 |
| 0. 029797 | 0. 064079 | 0 | 0. 106729 |
| 0. 073482 | 0 | 0 | 0 |
| 0. 881779 | 1. 219035 | 1. 697319 | 1. 654397 |
| 0 | 0 | 0 | 0 |
| 0. 050004 | 0. 053767 | 0 | 0 |
| 0. 992001 | 0. 799992 | 1. 063235 | 0. 444149 |
| 143. 3725 | 150. 2157 | 147. 1198 | 144. 221 |
| 0. 532235 | 0. 899312 | 1. 234044 | 1. 043972 |
| 12. 55547 | 9. 297419 | 9. 140651 | 8. 202396 |
| 0 | 0 | 0 | 0 |
| 0. 968987 | 0. 791852 | 0. 712162 | 0. 671014 |
| 1. 265285 | 0. 888494 | 0. 885645 | 0. 924909 |
| 0 | 0 | 0 | 0 |
| 0 | 0 | 0 | 0 |
| 0 | 0 | 1. 143648 | 0 |

| | | | |
|---|---|---|---|
| 0 | 0 | 0 | 0 |
| 0 | 0 | 0 | 0 |
| 30.11105 | 27.96907 | 26.68988 | 28.95172 |
| 16.1641 | 13.93251 | 13.10852 | 13.60063 |
| 102.0344 | 83.31842 | 85.01355 | 89.21972 |
| 0 | 0 | 0.154302 | 0 |
| 21.64585 | 17.24833 | 16.60118 | 18.17158 |
| 0.029176 | 0 | 0.008935 | 0.034835 |
| 40.93766 | 36.37122 | 33.04081 | 33.55146 |
| 8.727422 | 7.212122 | 7.583995 | 6.619576 |
| 0.705616 | 0.81492 | 1.152436 | 0.748862 |
| 0 | 0.270897 | 0 | 0.300799 |
| 0.07643 | 0 | 0.093621 | 0.091254 |
| 3.960329 | 3.862244 | 3.384491 | 3.903701 |
| 1.577092 | 1.255529 | 0.854458 | 0.959588 |
| 3.726992 | 3.07774 | 2.300903 | 2.705499 |
| 126.6986 | 128.4761 | 129.0243 | 128.5444 |
| 3.909623 | 2.381371 | 2.85125 | 2.698201 |
| 0.76064 | 0.49073 | 0.37269 | 1.089797 |
| 8.484774 | 7.683813 | 7.290757 | 8.510383 |
| 0.03945 | 0 | 0 | 0 |
| 16.62327 | 15.41328 | 15.35528 | 15.54359 |
| 0.479356 | 0.515431 | 0 | 0.190775 |
| 1.259314 | 0.827497 | 0.651305 | 0.634834 |
| 0 | 0.147125 | 0 | 0 |
| 3.073538 | 2.639782 | 3.053842 | 2.635782 |
| 58.84598 | 48.5095 | 48.14539 | 49.74885 |
| 0.214245 | 0.115184 | 0.196826 | 0.255798 |
| 68.70819 | 73.37329 | 68.89676 | 72.68517 |
| 0.07049 | 0 | 0 | 0 |
| 9.871619 | 6.24384 | 8.535521 | 8.319671 |
| 0.220445 | 0.118517 | 0.135014 | 0 |
| 20.72023 | 13.67379 | 12.45846 | 13.3593 |
| 8.641724 | 7.9735 | 7.793302 | 8.104359 |
| 0.9235 | 0.774758 | 0.90746 | 0.823929 |
| 18.25131 | 19.47912 | 19.70026 | 18.60875 |
| 5.542608 | 4.808417 | 5.130533 | 5.527189 |
| 0.056686 | 0 | 0 | 0 |
| 4.824756 | 3.856937 | 2.93951 | 3.619167 |
| 0.886469 | 0.476591 | 0.41366 | 0.655199 |
| 0.016876 | 0.009073 | 0 | 0 |
| 0.074555 | 0.080166 | 0 | 0.059344 |
| 2.359979 | 0.895618 | 0.850234 | 0.828733 |
| 0.078059 | 0 | 0.095616 | 0 |
| 0 | 0 | 0 | 0 |
| 11.11354 | 9.35796 | 10.20035 | 11.77389 |
| 9.501696 | 9.291473 | 8.388763 | 10.18867 |
| 71.98606 | 75.8104 | 72.97016 | 68.01391 |
| 0 | 0 | 0.136595 | 0.266281 |
| 25.19848 | 16.84274 | 17.93578 | 16.70297 |
| 13.29817 | 12.30018 | 12.96135 | 11.77996 |
| 0.976992 | 0.477508 | 0.519797 | 0.577348 |
| 9.081669 | 5.934609 | 6.146055 | 9.045853 |
| 9.598102 | 9.239642 | 8.835819 | 8.49472 |

| | | | |
|---|---|---|---|
| 18.94614 | 20.04463 | 19.23729 | 19.73432 |
| 0.426094 | 0.11454 | 0.39145 | 0.127184 |
| 38.57458 | 38.53099 | 40.60161 | 37.47004 |
| 7.281749 | 3.640996 | 3.487083 | 3.661272 |
| 0 | 0 | 0 | 0 |
| 3.842698 | 3.449229 | 4.256779 | 3.969602 |
| 3.668693 | 3.586171 | 4.14819 | 4.012658 |
| 0 | 0.416256 | 1.517426 | 0.554645 |
| 0 | 0.382086 | 0 | 0 |
| 0 | 0 | 0 | 0 |
| 0.933648 | 0.644789 | 0.836816 | 0.951596 |
| 0 | 0 | 0 | 0 |
| 0.89002 | 1.4355 | 0.817655 | 1.062637 |
| 43.7024 | 34.61432 | 34.92044 | 35.5262 |
| 0.647544 | 0.789113 | 0.898951 | 0.979303 |
| 11.87509 | 10.2399 | 11.31101 | 10.63369 |
| 1.361873 | 1.144866 | 1.273892 | 1.093859 |
| 17.06112 | 16.3994 | 15.19897 | 14.25907 |
| 1103.954 | 1199.485 | 1183.64 | 1125.699 |
| 37.64544 | 40.31816 | 45.22553 | 39.35062 |
| 2.494591 | 2.258801 | 2.090732 | 1.933355 |
| 0.485568 | 0.391583 | 0.334566 | 0.36234 |
| 8.882341 | 9.125228 | 8.017969 | 9.060188 |
| 0.035964 | 0 | 0 | 0.042939 |
| 15.87202 | 12.73978 | 8.762601 | 10.14245 |
| 0.015783 | 0.033941 | 0.135327 | 0.056531 |
| 2.023987 | 1.425856 | 1.645697 | 1.916563 |
| 0 | 0 | 0 | 0 |
| 1.330313 | 0.641227 | 0.786671 | 0.547698 |
| 2.830988 | 2.290875 | 2.252247 | 2.909633 |
| 1.984002 | 1.80511 | 1.682482 | 1.275505 |
| 170.8415 | 179.208 | 175.8047 | 179.6111 |
| 6.420925 | 5.328199 | 5.578271 | 5.020561 |
| 0 | 0.012926 | 0.02945 | 0.043058 |
| 0 | 0 | 0 | 0 |
| 10.18358 | 8.558162 | 8.387032 | 7.925955 |
| 0.791327 | 1.202968 | 1.086302 | 0.928513 |
| 12.75016 | 11.84019 | 11.44372 | 11.23736 |
| 0 | 0 | 0 | 0 |
| 0.248 | 0.533328 | 0.607563 | 0.592199 |
| 0.03029 | 0 | 0 | 0 |
| 70562.76 | 77822.46 | 80082.28 | 80370.44 |
| 0.399129 | 0.171666 | 0.04889 | 0.285923 |
| 12.54355 | 11.68034 | 12.14054 | 11.96632 |
| 0.898416 | 0 | 0 | 0.536331 |
| 34.83138 | 34.90577 | 31.61808 | 34.84305 |
| 0 | 0 | 0 | 0 |
| 19.39326 | 18.58312 | 18.69347 | 18.46921 |
| 0 | 0 | 0 | 0 |
| 13.82103 | 14.44394 | 14.91536 | 13.83222 |
| 2.805344 | 1.76966 | 1.786894 | 1.764036 |
| 0.919584 | 0.573498 | 0.585739 | 0.439174 |
| 0.47616 | 1.535985 | 1.749782 | 0.568511 |
| 0.282868 | 0.101385 | 0.461989 | 0.337729 |

| | | | |
|---|---|---|---|
| 6.696893 | 4.42427 | 4.379668 | 4.573836 |
| 0 | 0 | 0 | 0 |
| 1.796832 | 3.542103 | 3.48489 | 3.396763 |
| 0.746918 | 0.26771 | 0.609946 | 0.743151 |
| 61.58342 | 53.41813 | 51.13251 | 52.1135 |
| 0 | 0 | 0 | 0 |
| 45.42213 | 35.429 | 31.45228 | 33.7614 |
| 0.228923 | 0.067132 | 0.165699 | 0.223628 |
| 0 | 0 | 0 | 0 |
| 0 | 0 | 0 | 0 |
| 5.573297 | 6.07983 | 6.19181 | 6.112604 |
| 1.896656 | 1.82472 | 2.32326 | 1.251439 |
| 28.06676 | 24.3842 | 24.79395 | 24.20185 |
| 18.8675 | 9.247388 | 9.887039 | 10.29243 |
| 0.140709 | 0.151299 | 0.137887 | 0.1008 |
| 3.651998 | 3.485739 | 3.529713 | 3.989969 |
| 4.837186 | 4.551065 | 3.888404 | 2.346235 |
| 0 | 0 | 0 | 0 |
| 1.231629 | 1.212088 | 1.355231 | 1.146493 |
| 12.06751 | 11.17224 | 11.74532 | 10.9949 |
| 2.304002 | 2.477394 | 4.703714 | 4.584765 |
| 6.802292 | 5.678139 | 6.614672 | 6.625503 |
| 675.1813 | 755.4693 | 738.3439 | 753.0641 |
| 31.29343 | 24.73714 | 24.68296 | 23.99843 |
| 0 | 0 | 0 | 0.085555 |
| 0 | 0.027482 | 0.031308 | 0 |
| 12.6101 | 9.623964 | 8.770836 | 9.736402 |
| 0.266011 | 0.349593 | 0.253434 | 0.070579 |
| 1.046239 | 1.072651 | 0.864311 | 0.668153 |
| 237.0345 | 287.7816 | 293.082 | 289.3329 |
| 0 | 0 | 0 | 0 |
| 0.41431 | 0.381848 | 0.326249 | 0.317999 |
| 0.109664 | 0.165084 | 0.053732 | 0.183306 |
| 6.908087 | 5.673625 | 5.669605 | 5.167025 |
| 0.058568 | 0 | 0 | 0 |
| 1.963961 | 2.154861 | 1.669264 | 1.770615 |
| 19.26194 | 19.58905 | 16.70584 | 19.49984 |
| 0.066689 | 0.860496 | 0.081689 | 0.079623 |
| 72.21882 | 70.19804 | 69.18989 | 69.66802 |
| 0.676162 | 0.880111 | 1.002615 | 0.934771 |
| 0.296673 | 0 | 0 | 0.708425 |
| 0.111252 | 0 | 0.034069 | 0 |
| 35.06035 | 32.18654 | 32.88823 | 35.84316 |
| 0 | 0.010275 | 0.011705 | 0.022818 |
| 21.78512 | 24.01022 | 25.11261 | 24.10598 |
| 77.75679 | 73.37836 | 73.84027 | 71.34289 |
| 11.82855 | 11.78562 | 11.39253 | 11.73511 |
| 0 | 0.035432 | 0.080728 | 0 |
| 0 | 0 | 0 | 0 |
| 22253.19 | 23519.87 | 22947.6 | 23228.67 |
| 0 | 0.016394 | 0 | 0 |
| 21.02088 | 18.97294 | 19.19324 | 18.40312 |
| 22.28761 | 21.79144 | 20.58314 | 19.22318 |
| 0.028225 | 0 | 0.034574 | 0.0337 |

| | | | |
|---|---|---|---|
| 0.125305 | 0.089824 | 0.204653 | 0.099739 |
| 9.556516 | 7.162694 | 7.987077 | 7.081532 |
| 4.887367 | 4.738454 | 4.320913 | 4.638912 |
| 0.180535 | 0.04853 | 0.110571 | 0.107775 |
| 112.1192 | 73.78531 | 72.73802 | 71.43571 |
| 5.345453 | 5.064145 | 4.974402 | 4.554283 |
| 0.273459 | 0.220529 | 0.125612 | 0.122436 |
| 10.69366 | 8.86695 | 7.983176 | 8.162419 |
| 0 | 0 | 0 | 0 |
| 1.406381 | 1.469015 | 1.230508 | 1.231374 |
| 80.58702 | 87.24123 | 89.02399 | 89.29735 |
| 1017.507 | 1206.063 | 1179.205 | 1206.844 |
| 18.47808 | 18.87832 | 18.76859 | 19.15495 |
| 0.023526 | 0.050592 | 0 | 0 |
| 59.31882 | 65.84693 | 63.40778 | 67.42288 |
| 18.14358 | 15.12182 | 18.13053 | 15.54724 |
| 2.960454 | 2.527874 | 2.529291 | 3.133641 |
| 0.161959 | 0 | 0 | 0 |
| 2.101044 | 1.755762 | 1.846293 | 1.86777 |
| 0.132359 | 0.426959 | 0.162129 | 0.276552 |
| 0 | 0.125489 | 0 | 0.139341 |
| 10.52614 | 9.331762 | 9.774892 | 9.527701 |
| 0 | 0 | 0 | 0 |
| 2.930585 | 2.779657 | 3.137379 | 2.417985 |
| 16.51099 | 14.56561 | 12.89873 | 14.95278 |
| 0 | 0 | 0 | 0.95548 |
| 0 | 0 | 0 | 0 |
| 1369.318 | 1577.373 | 1557.69 | 1628.483 |
| 0.027318 | 0.058749 | 0.033463 | 0 |
| 0.128 | 0.137633 | 0.313581 | 0.764128 |
| 2.441848 | 2.221674 | 2.24331 | 2.018382 |
| 0 | 0 | 0 | 0 |
| 12.33554 | 10.09504 | 12.22217 | 10.05324 |
| 8.947343 | 7.394628 | 7.870083 | 8.381345 |
| 0.103513 | 0 | 0.380387 | 0 |
| 0.626527 | 0 | 0 | 0 |
| 0 | 0 | 0 | 0.02297 |
| 0 | 0 | 0 | 0 |
| 0.181279 | 0.32487 | 0.222054 | 0.144292 |
| 0 | 0 | 0 | 0 |
| 0.708924 | 0.317615 | 0.14473 | 0.493744 |
| 1.170886 | 0.419668 | 1.434247 | 2.329963 |
| 0 | 0 | 0 | 0 |
| 5.277568 | 4.121321 | 3.876366 | 4.259431 |
| 0.018629 | 0.100155 | 0.068458 | 0 |
| 15.31874 | 14.03426 | 13.79332 | 13.90721 |
| 34.00159 | 28.90653 | 29.37466 | 30.40584 |
| 32.75772 | 30.74603 | 30.30055 | 30.28971 |
| 0.474894 | 0.374464 | 0.368416 | 0.321299 |
| 15.70242 | 12.90363 | 13.41027 | 12.81977 |
| 0 | 0 | 0 | 0 |
| 9.726519 | 9.613877 | 9.67856 | 8.35802 |
| 3.3314 | 2.28514 | 3.236428 | 1.64587 |
| 0 | 0 | 0.466608 | 0 |

| | | | |
|---|---|---|---|
| 31.13435 | 29.73086 | 30.92783 | 32.08549 |
| 31.91606 | 24.30004 | 25.919 | 25.45813 |
| 6.436505 | 5.506407 | 4.949227 | 4.5436 |
| 0.340115 | 0 | 0 | 0 |
| 0.928255 | 0.480573 | 0.589577 | 0.656763 |
| 0.037463 | 0.040283 | 0.13767 | 0.134188 |
| 5.464136 | 5.111555 | 4.895564 | 4.604006 |
| 30.6419 | 36.29933 | 37.01809 | 38.54609 |
| 0.466824 | 1.003911 | 1.143648 | 0 |
| 0 | 0 | 0 | 0.040036 |
| 16.42409 | 14.98884 | 14.03207 | 14.17158 |
| 8.153102 | 7.158591 | 5.673051 | 7.142386 |
| 2.46854 | 3.325888 | 2.950912 | 3.906071 |
| 0.897235 | 0.560906 | 0.715657 | 0.772298 |
| 7.726876 | 5.822965 | 5.420036 | 8.594686 |
| 0 | 0 | 0 | 0 |
| 1.494814 | 2.377478 | 2.365085 | 1.896276 |
| 0 | 0 | 0 | 0 |
| 0.487432 | 0.589629 | 0.597068 | 0.363731 |
| 0 | 0.052191 | 0 | 0 |
| 0.053944 | 0.011601 | 0.006608 | 0.019322 |
| 47.73005 | 42.61857 | 41.47527 | 43.78396 |
| 0 | 0.230628 | 0 | 0 |
| 16.94902 | 15.48492 | 14.97164 | 14.21828 |
| 0.328954 | 0.088427 | 0.100736 | 0 |
| 0 | 0 | 0 | 0 |
| 3.139071 | 3.112298 | 3.645379 | 4.283301 |
| 0.234562 | 0.161417 | 0.137914 | 0.123224 |
| 8.043101 | 6.670943 | 6.079589 | 6.79885 |
| 0 | 0 | 0 | 0 |
| 0.149033 | 0.640995 | 0.365108 | 0.177938 |
| 0.154197 | 0.182382 | 0.09444 | 0.110462 |
| 0.684139 | 0.29425 | 0.335207 | 0 |
| 0 | 0 | 0 | 0 |
| 0.075461 | 0 | 0 | 0 |
| 0 | 0 | 0 | 0 |
| 0 | 0.048507 | 0.02763 | 0 |
| 3.651788 | 0.285173 | 0.599754 | 2.484495 |
| 0 | 0 | 0 | 0 |
| 0.562837 | 0.605195 | 0.551547 | 0.537599 |
| 0.708847 | 0.662777 | 0.943787 | 0.80953 |
| 0 | 0 | 0 | 0 |
| 0.098994 | 0 | 0 | 0 |
| 0.924726 | 0.552399 | 0.755146 | 0.543275 |
| 21.97062 | 16.73021 | 23.17719 | 15.77641 |
| 7.11227 | 6.014319 | 6.674273 | 6.735774 |
| 47.57593 | 43.43545 | 46.04172 | 44.63792 |
| 0.647837 | 1.741479 | 0.793552 | 0.386742 |
| 248.361 | 205.0665 | 206.6394 | 199.0782 |
| 0 | 0 | 0 | 0 |
| 0.684909 | 0.448276 | 0.474196 | 0.817745 |
| 0.095519 | 0 | 0 | 0.057022 |
| 0 | 0 | 0 | 0 |
| 2.930617 | 2.077539 | 2.430252 | 1.625644 |

| | | | |
|---|---|---|---|
| 0.706631 | 0.292235 | 0.399494 | 0.389391 |
| 5.594053 | 2.649484 | 2.610397 | 3.259992 |
| 4.207105 | 3.639774 | 3.593549 | 3.34871 |
| 13.03092 | 9.028176 | 8.476721 | 8.774018 |
| 11.79141 | 11.96961 | 12.07901 | 11.9272 |
| 7.170179 | 7.329725 | 6.061457 | 7.897659 |
| 3.813601 | 3.152821 | 3.217078 | 3.264588 |
| 0.969283 | 0.390836 | 0.445237 | 0.289319 |
| 10.90199 | 9.998553 | 9.868298 | 9.475181 |
| 45.25648 | 42.57321 | 42.02482 | 42.99047 |
| 3.895214 | 3.731445 | 2.920641 | 3.607803 |
| 9.038849 | 8.160751 | 8.385685 | 8.21916 |
| 0.159785 | 0.143175 | 0.195725 | 0.15898 |
| 27.97585 | 27.44145 | 24.89698 | 28.35738 |
| 0 | 0 | 0.142956 | 0 |
| 14.36377 | 11.65703 | 10.48976 | 10.65958 |
| 6.312272 | 6.405392 | 6.414375 | 6.748946 |
| 0.131609 | 0.113211 | 0.290179 | 0.09428 |
| 2.664436 | 2.864953 | 2.237236 | 2.026731 |
| 0.105813 | 0 | 0 | 0 |
| 54.04522 | 53.14377 | 52.82979 | 52.21345 |
| 12.85844 | 13.72539 | 13.28331 | 14.01003 |
| 1.058957 | 0.851998 | 0.843596 | 1.007935 |
| 0 | 0 | 0.297582 | 0 |
| 22.76411 | 20.78395 | 21.94446 | 20.98746 |
| 0.144291 | 0.077575 | 0.176746 | 0.086138 |
| 7.76735 | 7.244193 | 7.06881 | 7.107747 |
| 0.10251 | 0.082669 | 0.031392 | 0.061196 |
| 10.45434 | 8.002725 | 7.3704 | 7.743708 |
| 77.16371 | 71.66447 | 73.03413 | 70.2004 |
| 0.878293 | 1.011848 | 0.806882 | 1.048637 |
| 128.3825 | 99.80659 | 84.16672 | 83.47755 |
| 0.538961 | 0.403146 | 0.574075 | 0.587536 |
| 0.865746 | 0.9309 | 1.590711 | 3.100968 |
| 29.85151 | 25.85014 | 24.69417 | 24.91703 |
| 0.220445 | 0 | 0 | 0 |
| 9.926003 | 10.4152 | 8.281948 | 10.07633 |
| 0.100881 | 0.072316 | 0 | 0 |
| 136.8197 | 105.5304 | 101.5979 | 105.0441 |
| 0 | 0 | 0 | 0.070975 |
| 0.063658 | 0 | 0 | 0 |
| 18.19523 | 18.20006 | 17.98327 | 8.577778 |
| 2.469534 | 2.701365 | 2.749994 | 2.067776 |
| 0.103739 | 0.037182 | 0 | 0.041286 |
| 5.17006 | 5.14015 | 5.233406 | 4.627511 |
| 0 | 0.034782 | 0.039624 | 0 |
| 7.564691 | 6.895216 | 7.315402 | 7.150635 |
| 0 | 0.433894 | 0 | 0 |
| 34.41927 | 30.01278 | 31.04679 | 29.41246 |
| 0 | 0 | 0.052879 | 0 |
| 6.037727 | 6.347839 | 5.697474 | 5.784785 |
| 19.74483 | 19.16033 | 18.42928 | 20.8467 |
| 2.394772 | 1.909788 | 2.208209 | 1.866444 |
| 0.454568 | 0.229115 | 0.139203 | 0.288326 |

| | | | |
|---|---|---|---|
| 0.128494 | 0.039475 | 0.067455 | 0.065749 |
| 0 | 0 | 0 | 0 |
| 2.982408 | 2.556668 | 2.586132 | 2.85112 |
| 2.79886 | 3.466632 | 3.384995 | 3.510893 |
| 0.131463 | 0.056543 | 0.128826 | 0 |
| 0 | 0 | 0 | 0 |
| 0.280244 | 0.13697 | 0.124828 | 0.182508 |
| 0 | 0 | 0 | 0 |
| 0.983897 | 0.627384 | 0.742739 | 0.956169 |
| 0.146624 | 0.105105 | 0.119735 | 0.058354 |
| 0 | 0.057657 | 0.032841 | 0 |
| 17.08144 | 10.56428 | 11.03601 | 12.07112 |
| 0 | 0 | 0 | 0.148825 |
| 4.328731 | 3.127241 | 4.018202 | 3.835833 |
| 0.194034 | 0.041727 | 0 | 0.092667 |
| 3.659408 | 3.041243 | 2.714501 | 2.976588 |
| 51.75077 | 38.65059 | 40.19107 | 38.05997 |
| 0 | 0 | 0 | 0 |
| 8.41244 | 8.189283 | 7.003129 | 6.777274 |
| 0.097574 | 0 | 0 | 0 |
| 23.99573 | 30.67932 | 27.66847 | 28.42554 |
| 0 | 0 | 0 | 0.032561 |
| 0 | 0 | 0.033773 | 0 |
| 0 | 0 | 0 | 0 |
| 0.326417 | 0.263236 | 0.499795 | 0.243578 |
| 0.139705 | 0.100146 | 0.057043 | 0 |
| 3.113085 | 2.434448 | 2.036645 | 2.323038 |
| 2.766299 | 2.091733 | 2.054966 | 2.002999 |
| 1.784139 | 1.541016 | 3.797643 | 0.523812 |
| 7.009742 | 8.192689 | 7.510359 | 7.470267 |
| 0 | 0 | 0 | 0.528847 |
| 3.989188 | 4.352482 | 3.688794 | 3.59551 |
| 0 | 0 | 0.466608 | 0.454809 |
| 4.660624 | 0.640342 | 5.89921 | 3.21507 |
| 8.859676 | 9.546698 | 9.628649 | 9.430169 |
| 0.067781 | 0.109323 | 0.083026 | 0.040463 |
| 0.533513 | 0.573664 | 0.245067 | 0.398117 |
| 3.249347 | 2.768629 | 2.747456 | 2.869718 |
| 2.219308 | 1.278389 | 1.189337 | 1.372186 |
| 0.049292 | 0 | 0 | 0 |
| 1.578132 | 0.731421 | 0.599925 | 0.844645 |
| 2.908146 | 2.293137 | 2.921053 | 2.662001 |
| 1.772118 | 1.297937 | 1.226924 | 1.318553 |
| 21.30192 | 24.56036 | 20.56761 | 23.76632 |
| 2.224164 | 1.414575 | 1.53032 | 1.525521 |
| 0.023307 | 0.150366 | 0 | 0.027827 |
| 0 | 0 | 0 | 0.093814 |
| 0 | 0 | 0.186942 | 0 |
| 0 | 0 | 0 | 0.076106 |
| 0.933648 | 0.430248 | 0.816892 | 0.47774 |
| 0.015736 | 0 | 0 | 0 |
| 111.3744 | 78.06733 | 75.02934 | 76.59349 |
| 0.126976 | 0 | 0 | 0 |
| 32.81462 | 29.77813 | 28.63414 | 30.78605 |

| | | | |
|---|---|---|---|
| 0 | 0.406345 | 0 | 0 |
| 0 | 0.067015 | 0 | 0 |
| 0.234562 | 0.504428 | 0.191547 | 0.466758 |
| 0.784978 | 0.844053 | 0.895979 | 0.83072 |
| 82.88406 | 71.87329 | 72.65721 | 71.12276 |
| 0 | 0 | 0 | 0 |
| 34.11904 | 11.70986 | 25.97 | 18.18958 |
| 0.023916 | 0 | 0 | 0.028554 |
| 6.750909 | 7.943209 | 8.337136 | 7.003155 |
| 3.310897 | 2.231239 | 1.909181 | 2.158205 |
| 5.580646 | 4.588716 | 4.302577 | 4.566116 |
| 0 | 0.054876 | 0 | 0 |
| 0.219818 | 0.127271 | 0.269261 | 0.14132 |
| 0.130098 | 0.195845 | 0.223105 | 0.248529 |
| 0.044335 | 0 | 0 | 0 |
| 12.66166 | 13.08984 | 12.42653 | 12.66424 |
| 2.224038 | 1.743739 | 1.749971 | 1.751817 |
| 2.480002 | 0.533328 | 2.430252 | 1.184398 |
| 0.171617 | 0.570374 | 0.458658 | 0.465687 |
| 33.59513 | 27.82044 | 27.60298 | 28.22006 |
| 0.772988 | 0.664928 | 0.946852 | 1.29207 |
| 4.401192 | 4.322518 | 4.542131 | 4.675526 |
| 0.023631 | 0.025409 | 0.057892 | 0.028214 |
| 0.872175 | 0.836427 | 0.721857 | 0.759891 |
| 46.19732 | 46.10814 | 44.44511 | 45.79847 |
| 0.466824 | 0 | 0.190608 | 0.092894 |
| 14.62365 | 11.12199 | 12.01473 | 15.96941 |
| 14.16272 | 14.20333 | 14.22899 | 13.70257 |
| 3.666343 | 4.391379 | 4.093057 | 5.541042 |
| 0 | 0 | 0 | 0 |
| 10.02745 | 12.34292 | 13.19531 | 12.20029 |
| 6.878256 | 6.62906 | 6.448319 | 6.389819 |
| 1.338604 | 0.959562 | 1.483528 | 0.989376 |
| 0.118595 | 0.06376 | 0.290541 | 0.212395 |
| 1.868823 | 1.465235 | 0.906128 | 1.394548 |
| 27.34423 | 16.30407 | 14.63162 | 15.17331 |
| 426.4705 | 474.7994 | 461.6294 | 470.1605 |
| 0 | 0 | 0 | 0 |
| 1.131321 | 0.838006 | 0.985446 | 1.410771 |
| 13.74591 | 5.458666 | 5.166114 | 4.600307 |
| 0.008865 | 0 | 0.010859 | 0 |
| 15.81201 | 18.87316 | 19.25826 | 18.91454 |
| 0.130634 | 0.035116 | 0.040004 | 0 |
| 18.00055 | 17.65987 | 18.44417 | 16.40901 |
| 0.046545 | 0 | 0.057015 | 0 |
| 66.59768 | 51.19949 | 48.28763 | 50.00575 |
| 0 | 0 | 0 | 0 |
| 17.39399 | 11.84524 | 10.91949 | 11.03275 |
| 54.30637 | 58.96519 | 54.58853 | 34.52509 |
| 1.040788 | 0.279779 | 1.434247 | 0.310662 |
| 9.2435 | 8.576325 | 7.628695 | 7.096602 |
| 0 | 0 | 0 | 0 |
| 14.13194 | 14.67425 | 14.2047 | 14.5578 |
| 15.23981 | 15.10852 | 15.73904 | 15.37292 |

| | | | |
|---|---|---|---|
| 1.035998 | 0.964007 | 0.878552 | 0.903909 |
| 4.604244 | 3.728726 | 3.426745 | 3.514051 |
| 0 | 1.786029 | 0 | 0.661059 |
| 0 | 0 | 0 | 0.019357 |
| 66.3942 | 60.95864 | 63.09072 | 68.54159 |
| 2.246762 | 2.580564 | 1.720071 | 1.889956 |
| 0.816275 | 0.444271 | 0.617207 | 0.493311 |
| 4.437338 | 5.046544 | 4.33787 | 4.380998 |
| 2.437892 | 2.488633 | 2.305826 | 2.358049 |
| 0.202191 | 0.326111 | 0.247669 | 0.241406 |
| 31.85686 | 29.68438 | 27.96518 | 31.65878 |
| 0.129581 | 0.117897 | 0.073259 | 0.142812 |
| 0.059101 | 0.063549 | 0 | 0 |
| 0.42651 | 0.29398 | 0.254523 | 0.339487 |
| 0.054191 | 0.05827 | 0 | 0 |
| 4.936664 | 4.216428 | 4.245794 | 4.222029 |
| 8.97958 | 6.863132 | 6.896863 | 7.591735 |
| 10.52398 | 10.16303 | 8.123813 | 8.724437 |
| 0.751042 | 0.726807 | 0.643979 | 0.717364 |
| 74.24555 | 74.07535 | 71.72272 | 73.73434 |
| 0.262709 | 0.42372 | 0.241349 | 0.392076 |
| 15.19781 | 14.49897 | 14.31483 | 15.96526 |
| 0 | 0 | 0.040336 | 0 |
| 1.398249 | 1.178401 | 1.897911 | 1.488957 |
| 0.205488 | 0.110476 | 0.125853 | 0.095411 |
| 0.019138 | 0.041157 | 0 | 0 |
| 0 | 0 | 0 | 0 |
| 1.20547 | 0.648095 | 0 | 0.719634 |
| 182.8414 | 130.3004 | 129.784 | 125.463 |
| 0.073595 | 0.052756 | 0.090148 | 0.087869 |
| 64.35372 | 55.44949 | 55.87906 | 51.92751 |
| 9.868772 | 9.925235 | 9.428649 | 9.013657 |
| 0 | 0 | 0 | 0 |
| 2.499842 | 2.133312 | 1.944202 | 1.989788 |
| 16.26231 | 17.59108 | 16.37432 | 15.92141 |
| 29.43058 | 22.48243 | 21.63014 | 20.24402 |
| 2.674615 | 1.360031 | 1.33953 | 1.604541 |
| 0 | 0 | 0 | 0 |
| 0 | 0.474069 | 0 | 0.526399 |
| 1.508959 | 1.261959 | 0.924181 | 1.415558 |
| 12.48026 | 10.82937 | 11.19209 | 9.966915 |
| 0.657853 | 0.656836 | 0.671517 | 0.841546 |
| 20.91905 | 20.16916 | 21.02895 | 21.24161 |
| 22.47457 | 22.24491 | 21.15469 | 22.56732 |
| 2.583816 | 1.587581 | 1.35642 | 0.881412 |
| 4.113342 | 5.20133 | 5.965623 | 5.657607 |
| 2.012792 | 1.518218 | 1.471951 | 2.116223 |
| 27.47768 | 22.79505 | 21.06567 | 23.46405 |
| 7.969958 | 7.720992 | 7.984744 | 7.934831 |
| 30.18339 | 36.20382 | 35.32509 | 36.63208 |
| 2.301112 | 1.581921 | 2.010048 | 1.655201 |
| 14.0267 | 12.76944 | 12.03686 | 11.6784 |
| 9.748611 | 9.876351 | 9.421902 | 7.972608 |
| 0 | 0.084488 | 0 | 0 |

| | | | |
|---|---|---|---|
| 24.80905 | 25.52207 | 25.63616 | 26.56501 |
| 0.283791 | 0.108981 | 0.148981 | 0.145214 |
| 7.4868 | 7.969731 | 7.581165 | 6.912711 |
| 0.330667 | 0.355552 | 0.405042 | 0 |
| 0.909861 | 1.260964 | 1.263112 | 1.327732 |
| 153.5538 | 142.7744 | 150.1863 | 141.8734 |
| 0 | 0 | 0.702724 | 0 |
| 4.489694 | 4.351616 | 2.865955 | 3.246476 |
| 0.131536 | 0.141435 | 0 | 0 |
| 6.756486 | 6.944808 | 6.55081 | 6.002315 |
| 67.2742 | 62.64672 | 63.0873 | 62.0522 |
| 5.167185 | 4.314476 | 5.233257 | 3.84292 |
| 0 | 0 | 0 | 0 |
| 0.290342 | 0 | 0 | 0 |
| 8.37819 | 8.408127 | 8.073284 | 8.069187 |
| 0 | 0 | 0 | 0 |
| 0.017442 | 0.150035 | 0.149554 | 0.187421 |
| 0.634881 | 2.730639 | 0 | 0.758015 |
| 0.039515 | 0 | 0 | 0 |
| 1.343797 | 0.913303 | 0.714323 | 0.862756 |
| 0 | 0.495279 | 0.141055 | 0.206231 |
| 200.2398 | 193.3914 | 189.9133 | 190.8824 |
| 0.748965 | 1.342217 | 1.612446 | 1.409083 |
| 65.13217 | 68.29998 | 65.37476 | 66.85477 |
| 2.395569 | 1.446712 | 1.286309 | 1.175419 |
| 0.226384 | 0 | 1.386515 | 0.450484 |
| 0.170057 | 0.365711 | 0.208307 | 0 |
| 0.530541 | 0.213925 | 0.487404 | 0.316719 |
| 0 | 0 | 0 | 0 |
| 0 | 0 | 0 | 0 |
| 1.721062 | 1.233723 | 0 | 0 |
| 0.058785 | 0.168558 | 0.09601 | 0.116978 |
| 1.615407 | 1.43921 | 1.922216 | 1.653182 |
| 3.179003 | 2.506713 | 3.00656 | 2.712799 |
| 55.60404 | 59.92675 | 56.75272 | 57.95379 |
| 0 | 0 | 0 | 0 |
| 34.35201 | 32.99376 | 31.14716 | 26.7835 |
| 1.129578 | 1.074442 | 0.931301 | 0.959621 |
| 15.08654 | 13.63961 | 14.29951 | 14.2505 |
| 134.6691 | 127.2147 | 124.8846 | 125.8143 |
| 23.12363 | 17.66147 | 17.587 | 17.94199 |
| 17.8103 | 17.77432 | 17.60533 | 14.49659 |
| 12.40656 | 11.95555 | 10.50331 | 10.2002 |
| 8.328366 | 6.352175 | 5.821724 | 6.523026 |
| 2.759716 | 3.391318 | 3.058496 | 2.510444 |
| 7.988321 | 6.496968 | 6.382417 | 6.502085 |
| 1.473529 | 1.267538 | 2.011243 | 1.005324 |
| 10.62364 | 10.8278 | 11.02091 | 12.43618 |
| 8.032346 | 7.611716 | 8.266613 | 8.138229 |
| 19.271 | 18.97723 | 17.90907 | 17.60515 |
| 6.432069 | 6.378952 | 5.431017 | 5.62919 |
| 0.025523 | 0.016466 | 0.006253 | 0.030473 |
| 10.60753 | 10.36029 | 10.75567 | 10.2726 |
| 0 | 0 | 0 | 0 |

| | | | |
|---|---|---|---|
| 0 | 0 | 0.021412 | 0 |
| 0.780591 | 0 | 0.956165 | 0.931985 |
| 0 | 0 | 0.466608 | 0 |
| 7.73252 | 8.642648 | 7.851585 | 10.32552 |
| 3.833569 | 3.469255 | 3.049104 | 3.282658 |
| 6.496238 | 5.17692 | 4.909886 | 4.400665 |
| 12.20706 | 9.123982 | 9.169617 | 9.343993 |
| 497.2381 | 592.1762 | 587.3916 | 580.0483 |
| 0 | 0.164629 | 0 | 0.182801 |
| 28.31464 | 24.36814 | 24.26077 | 23.78564 |
| 3.785623 | 2.998373 | 3.239762 | 3.127566 |
| 0 | 0 | 0 | 0.341448 |
| 0 | 0 | 0 | 0 |
| 0 | 0 | 0 | 0 |
| 0 | 0 | 0 | 0.064166 |
| 0.771735 | 0.580869 | 1.13438 | 0.737129 |
| 0.792622 | 0.410353 | 0.57535 | 0.52575 |
| 1.581709 | 1.771609 | 2.381479 | 1.495046 |
| 18.37275 | 16.84699 | 17.81664 | 17.57936 |
| 11.09205 | 10.41553 | 9.817949 | 9.909823 |
| 11.25709 | 10.25402 | 10.02404 | 10.35082 |
| 7.161153 | 5.744824 | 6.429644 | 6.401343 |
| 0.092279 | 0.79379 | 0.339105 | 0.110177 |
| 3.793851 | 3.387947 | 2.894642 | 3.224505 |
| 0 | 0 | 0 | 0 |
| 3.256656 | 2.082534 | 2.1791 | 1.918446 |
| 19.31933 | 16.19129 | 17.59459 | 16.32075 |
| 0.07475 | 0.114823 | 0.091564 | 0.101998 |
| 0 | 0 | 0 | 0 |
| 3.260391 | 3.19756 | 3.510974 | 2.694973 |
| 0.249299 | 0.26806 | 0 | 0.198433 |
| 2.483377 | 2.205874 | 2.512914 | 3.738507 |
| 56.62647 | 54.62591 | 54.19837 | 54.9269 |
| 13.39201 | 13.07679 | 11.95264 | 12.33368 |
| 0 | 1.38377 | 0 | 0 |
| 0.212572 | 0.114285 | 0.130192 | 0.1269 |
| 36.33657 | 36.36873 | 35.18109 | 38.90097 |
| 28.98796 | 36.83188 | 34.57364 | 35.43277 |
| 0 | 0 | 0 | 0 |
| 1.240571 | 0.804283 | 0.916233 | 0.740589 |
| 0.069921 | 0 | 0 | 0 |
| 4.965409 | 4.323069 | 4.357433 | 3.937546 |
| 0 | 0 | 0.072007 | 0.070187 |
| 1.391096 | 1.27765 | 1.561988 | 1.003458 |
| 54.68287 | 57.13213 | 54.7154 | 61.04724 |
| 10.58134 | 8.244684 | 8.734821 | 7.415359 |
| 0.05952 | 0 | 0 | 0 |
| 20.6257 | 21.54377 | 21.03069 | 20.85094 |
| 8.273951 | 7.97477 | 8.201321 | 8.448861 |
| 31.8886 | 20.42089 | 20.29948 | 19.92241 |
| 14.32035 | 13.8315 | 13.14513 | 13.74608 |
| 0.06683 | 0.107788 | 0.040931 | 0.159582 |
| 1.208681 | 1.171283 | 1.133255 | 1.122413 |
| 3.159358 | 3.303795 | 3.380913 | 3.295415 |

| | | | |
|---|---|---|---|
| 7.844594 | 6.295356 | 5.718547 | 5.604393 |
| 6.03354 | 3.945166 | 3.728607 | 4.056156 |
| 0.224048 | 0.291627 | 0.288886 | 0.309738 |
| 35.3897 | 32.89005 | 28.75458 | 32.85791 |
| 0 | 0 | 0 | 0 |
| 0.679863 | 0.468961 | 0.282831 | 0.16847 |
| 10.6141 | 9.609811 | 9.073946 | 9.966541 |
| 275.7118 | 235.3426 | 234.7001 | 229.7367 |
| 0 | 0 | 0 | 0 |
| 0 | 0 | 0 | 0 |
| 26.97751 | 24.35806 | 28.6514 | 24.81735 |
| 1.622045 | 0.561993 | 0.596065 | 0.451882 |
| 0.258012 | 0.332915 | 0.28444 | 0.261845 |
| 0 | 0 | 0.478082 | 0 |
| 3.787286 | 3.369415 | 3.024973 | 3.295356 |
| 0 | 0.055611 | 0 | 0.041167 |
| 0 | 0 | 0 | 0 |
| 14.74807 | 8.039328 | 6.931607 | 8.05157 |
| 3.392941 | 3.562777 | 3.322713 | 2.880005 |
| 0 | 0 | 0 | 0 |
| 13.3 | 9.715894 | 10.44646 | 11.6369 |
| 13.72659 | 7.368605 | 7.998784 | 7.100169 |
| 12.12993 | 10.14774 | 10.07668 | 9.510405 |
| 5.555436 | 4.409383 | 4.225542 | 4.350258 |
| 0 | 0 | 0 | 0 |
| 4.370672 | 3.106955 | 3.301475 | 3.131013 |
| 19.83488 | 17.1135 | 16.15023 | 16.79189 |
| 0.163069 | 0 | 0 | 0 |
| 0.721455 | 0.208856 | 0.271916 | 0.39756 |
| 0.313263 | 0.336839 | 0 | 0.37402 |
| 0 | 0 | 0 | 0 |
| 2.768496 | 2.025597 | 2.549773 | 2.187058 |
| 0 | 0.026834 | 0.030569 | 0.029796 |
| 8.018596 | 7.168897 | 7.28386 | 6.884521 |
| 2.026215 | 1.14987 | 1.723583 | 1.276797 |
| 16.69387 | 10.88058 | 10.75918 | 9.751157 |
| 1.22576 | 1.013851 | 0.461989 | 0.675458 |
| 0.136631 | 0 | 0.083682 | 0.081565 |
| 0 | 0 | 0.03331 | 0 |
| 0.409184 | 1.018895 | 1.160718 | 0.797098 |
| 69.62238 | 69.60363 | 64.3163 | 65.37875 |
| 0.02333 | 0.075256 | 0 | 0.222836 |
| 0.337702 | 0.363117 | 0.41366 | 0 |
| 15.92675 | 13.85917 | 13.77702 | 13.57565 |
| 0.523253 | 0.500117 | 0.142432 | 0.208246 |
| 0.45991 | 0.428586 | 0.413127 | 0.305061 |
| 0 | 0 | 0 | 0 |
| 0 | 0 | 0 | 0 |
| 23.25525 | 25.1305 | 25.46865 | 25.82035 |
| 0 | 0 | 0 | 0 |
| 2.416413 | 2.105962 | 2.523724 | 2.034734 |
| 0.772749 | 0.580314 | 0.510841 | 0.615081 |
| 0.630922 | 0.438967 | 0.363685 | 0.265866 |
| 1.063022 | 0.354731 | 0.493908 | 0.481418 |

| | | | |
|---|---|---|---|
| 3.095043 | 2.076424 | 2.381647 | 2.068748 |
| 1.670738 | 1.616826 | 1.159699 | 0.997388 |
| 6.789107 | 7.322089 | 6.482069 | 6.795682 |
| 0 | 0 | 0 | 0 |
| 0 | 0 | 0 | 0 |
| 11.27525 | 10.36144 | 10.28062 | 10.59421 |
| 0.874835 | 0.940673 | 0.995064 | 1.715978 |
| 9.625978 | 9.245375 | 7.427131 | 7.034811 |
| 41.05718 | 46.46327 | 45.72353 | 45.6421 |
| 5.620109 | 3.489432 | 3.74024 | 3.31103 |
| 9.367645 | 8.036283 | 9.279146 | 9.125246 |
| 31.90328 | 29.35742 | 29.49898 | 30.63325 |
| 8.209663 | 7.650498 | 9.385803 | 6.534608 |
| 0.04259 | 0 | 0.026085 | 0.012713 |
| 8.161935 | 8.530691 | 8.791731 | 8.279781 |
| 7.063298 | 5.974624 | 6.713957 | 6.004446 |
| 3.209208 | 3.186553 | 3.291538 | 2.988303 |
| 109.9103 | 109.9921 | 113.1921 | 115.5089 |
| 0.563181 | 0.546961 | 0.556334 | 0.585646 |
| 0 | 0 | 0 | 0.392076 |
| 0.128518 | 0.13819 | 0.078713 | 0 |
| 194.1242 | 180.9151 | 176.4286 | 176.7653 |
| 3.568416 | 2.518008 | 3.892957 | 3.528229 |
| 0 | 0.133332 | 0.075945 | 0 |
| 52.144 | 50.88761 | 50.4703 | 49.92507 |
| 0.619144 | 0.395283 | 0.639904 | 0.438915 |
| 0.13862 | 0 | 0 | 0 |
| 0 | 0.014984 | 0 | 0.016638 |
| 0 | 0 | 0 | 0.107673 |
| 0 | 0 | 0 | 0 |
| 8.725699 | 7.667312 | 6.378515 | 6.161202 |
| 2.087433 | 3.154471 | 2.902482 | 3.974187 |
| 26.4211 | 22.68595 | 20.27186 | 20.56808 |
| 0.442753 | 0.216397 | 0.295821 | 0.480567 |
| 6.733582 | 7.757498 | 6.480673 | 4.594027 |
| 0 | 0 | 0 | 0 |
| 0.012992 | 0.02794 | 0.063657 | 0.015512 |
| 2.293593 | 1.659764 | 1.858743 | 1.520194 |
| 0.056085 | 0 | 0.0687 | 0 |
| 10.65001 | 9.978528 | 9.8661 | 9.911741 |
| 1.086176 | 0.480908 | 0.645676 | 0.66749 |
| 0.458056 | 0.445621 | 0.293901 | 0.156256 |
| 0.674318 | 0.692108 | 0.563174 | 0.466592 |
| 0.019915 | 0 | 0.024394 | 0 |
| 0 | 0 | 0 | 0.243648 |
| 7.350676 | 9.191903 | 8.803934 | 7.638654 |
| 0 | 0.522444 | 0 | 0 |
| 42.59472 | 35.12595 | 35.13703 | 38.03855 |
| 0.497729 | 0.148663 | 0.372582 | 0.165073 |
| 3.662773 | 3.719621 | 3.988107 | 1.943627 |
| 0.026917 | 0 | 0 | 0 |
| 74.73585 | 71.14117 | 70.18058 | 70.42681 |
| 2.314669 | 1.955536 | 2.734034 | 2.566195 |
| 0.0465 | 0.074999 | 0.113918 | 0.027759 |

| | | | |
|---|---|---|---|
| 0.18937 | 0.135748 | 0.180416 | 0.150732 |
| 0.034109 | 0.110028 | 0.167123 | 0.040724 |
| 4.126724 | 2.901304 | 5.249345 | 2.08454 |
| 1.179913 | 0.986774 | 1.05989 | 1.111351 |
| 4.923264 | 4.938222 | 3.960411 | 5.00079 |
| 0.286373 | 0.447891 | 0.159448 | 0.310832 |
| 15.26008 | 14.92804 | 16.49058 | 14.24702 |
| 0 | 0 | 1.06694 | 0 |
| 3.762117 | 3.179842 | 3.026352 | 2.502878 |
| 0.094853 | 0 | 0 | 0.113249 |
| 2.954896 | 1.688494 | 2.151032 | 1.713597 |
| 3.215128 | 4.28679 | 4.174586 | 3.91547 |
| 0 | 0 | 0 | 0 |
| 3.099411 | 2.457678 | 2.330697 | 2.614664 |
| 0.609042 | 1.190686 | 0.542568 | 0.198318 |
| 0 | 0 | 0 | 0 |
| 0.085211 | 0.082462 | 0.073064 | 0.030521 |
| 0.044171 | 0 | 0 | 0 |
| 2.740755 | 2.613021 | 2.864826 | 2.901456 |
| 1.072805 | 1.064807 | 0.60651 | 0.886759 |
| 14.91294 | 15.33806 | 13.70042 | 12.04759 |
| 0.039614 | 0.127786 | 0.048524 | 0.141891 |
| 0 | 0.177161 | 0 | 0 |
| 0 | 0 | 0 | 0 |
| 0 | 0.053856 | 0 | 0.039868 |
| 35.89852 | 37.74235 | 35.93982 | 37.56674 |
| 2.397432 | 2.175531 | 2.529273 | 2.200581 |
| 1.179108 | 1.04556 | 0.937869 | 0.996425 |
| 0 | 0 | 0.071522 | 0.069713 |
| 14.2423 | 13.10463 | 13.88716 | 13.28217 |
| 0.022959 | 0 | 0 | 0 |
| 23.63322 | 25.7877 | 27.06466 | 26.38024 |
| 3.695574 | 3.820857 | 3.482153 | 4.072914 |
| 16.40697 | 14.9762 | 15.42841 | 14.21705 |
| 0 | 0 | 0 | 0 |
| 0.378549 | 0.726852 | 0.264968 | 0.355118 |
| 65.4333 | 75.78373 | 74.97091 | 82.2641 |
| 3.268634 | 2.786367 | 2.452797 | 2.636878 |
| 12.68978 | 13.23417 | 13.31317 | 12.52057 |
| 0.778995 | 0.349008 | 0.437346 | 0.34878 |
| 0 | 0 | 0 | 0.494357 |
| 0 | 0 | 0.066204 | 0 |
| 0.252301 | 0.09865 | 0.224763 | 0.246464 |
| 13.26361 | 14.13184 | 14.13741 | 15.40318 |
| 1.589446 | 1.998735 | 1.880954 | 1.78514 |
| 5.533175 | 7.403928 | 7.342532 | 7.413764 |
| 14.97052 | 12.11294 | 12.54688 | 12.28045 |
| 0.078187 | 0.084071 | 0 | 0.093352 |
| 10.11211 | 8.831807 | 10.25096 | 9.205343 |
| 26.97393 | 17.93467 | 16.30579 | 17.21261 |
| 0.369117 | 0.396895 | 0 | 0 |
| 7.909708 | 6.044678 | 6.016234 | 6.028947 |
| 0.432873 | 0.46545 | 0.530237 | 0 |
| 100.3602 | 65.52494 | 62.03573 | 65.02476 |

| | | | |
|---|---|---|---|
| 47.72576 | 45.87112 | 45.49164 | 47.22308 |
| 0.012085 | 0.02599 | 0 | 0 |
| 15.14059 | 10.26349 | 9.138645 | 9.824497 |
| 0 | 0 | 0 | 0.017165 |
| 35.35461 | 35.34024 | 35.48045 | 35.57653 |
| 0 | 0 | 0 | 0 |
| 0.746955 | 0.203334 | 0.196891 | 0.293512 |
| 7.018926 | 6.522228 | 6.58092 | 5.664417 |
| 0.094289 | 0 | 0 | 0 |
| 0.332979 | 0 | 0 | 0.26504 |
| 42.96844 | 50.72804 | 46.83271 | 49.83631 |
| 0.054553 | 0.087988 | 0.044549 | 0.119412 |
| 429.5886 | 431.8834 | 445.2159 | 431.9623 |
| 13.73995 | 17.32122 | 16.83041 | 12.72786 |
| 21.70659 | 15.312 | 14.29382 | 15.23113 |
| 0 | 0.03064 | 0.034905 | 0.034022 |
| 3.223283 | 1.784991 | 2.270683 | 2.015059 |
| 32.10523 | 26.09909 | 24.08131 | 28.12983 |
| 0 | 0.376467 | 0 | 0.418023 |
| 8.131664 | 8.712841 | 7.751088 | 7.589261 |
| 15.72288 | 11.27076 | 11.27376 | 11.44653 |
| 8.798468 | 7.040114 | 7.902349 | 7.52224 |
| 0.831391 | 0.528249 | 0.509196 | 0.631679 |
| 0.710487 | 0.518914 | 0.673246 | 0.432145 |
| 4.772736 | 4.33894 | 4.482687 | 4.219806 |
| 3.062318 | 2.582572 | 3.23625 | 3.871322 |
| 0 | 0 | 0 | 0 |
| 4.81688 | 4.627483 | 4.812142 | 5.609684 |
| 18.9207 | 10.00333 | 9.432267 | 9.156215 |
| 10.54979 | 8.719859 | 9.21209 | 8.552152 |
| 0.374774 | 0.212094 | 0.120808 | 0.188405 |
| 0.410736 | 0.283916 | 0.107812 | 0.140114 |
| 0.064433 | 0.323317 | 0.052617 | 0.128216 |
| 1.103074 | 1.38377 | 1.125986 | 0.878009 |
| 14.79137 | 16.04977 | 15.5743 | 15.42237 |
| 57.11263 | 62.60366 | 60.11987 | 59.89763 |
| 46.05486 | 24.34074 | 23.90412 | 25.1636 |
| 0.811805 | 0.777674 | 0.65088 | 0.599175 |
| 0.119338 | 0.06416 | 0.036545 | 0.035621 |
| 178.8022 | 224.8878 | 222.1058 | 215.2049 |
| 9.767394 | 7.876844 | 7.627254 | 7.361487 |
| 6.09746 | 7.70745 | 7.811017 | 7.391197 |
| 0.062284 | 0 | 0.038147 | 0.037182 |
| 0.267406 | 0.325867 | 0.240205 | 0.255415 |
| 32.36196 | 37.38872 | 39.08949 | 41.80041 |
| 23.5727 | 22.46441 | 20.62843 | 22.15898 |
| 5.703698 | 4.969797 | 4.264235 | 5.588831 |
| 33.25854 | 30.79786 | 32.63588 | 33.37289 |
| 5.275696 | 3.6645 | 4.292496 | 3.954059 |
| 14.20722 | 12.27271 | 12.99591 | 12.24615 |
| 0 | 0 | 0 | 0 |
| 0.336679 | 0.206867 | 0.235661 | 0 |
| 14.75343 | 10.58642 | 10.71997 | 9.760525 |
| 13.35734 | 10.92701 | 11.85003 | 11.12649 |

| | | | |
|---|---|---|---|
| 23.76831 | 24.31869 | 24.34712 | 22.92536 |
| 1.341297 | 0 | 0.821494 | 1.601439 |
| 0 | 0.04794 | 0.054612 | 0 |
| 45.97874 | 38.64493 | 38.50046 | 36.43725 |
| 19.47791 | 17.57838 | 18.31297 | 15.49165 |
| 2.6786 | 2.507453 | 2.354659 | 1.994115 |
| 0 | 0.106003 | 0.120758 | 0.117704 |
| 37.66642 | 21.22698 | 25.53579 | 23.75866 |
| 0 | 0 | 0.176746 | 0 |
| 1.587898 | 0.921097 | 0.588635 | 0.823206 |
| 0 | 0 | 0 | 0 |
| 0 | 0 | 0 | 0 |
| 61.93957 | 63.72501 | 64.79236 | 64.87663 |
| 9.574732 | 9.187284 | 9.729775 | 9.124882 |
| 6.23813 | 4.922507 | 5.114699 | 4.534873 |
| 0.038093 | 0 | 0 | 0 |
| 5.914572 | 5.377556 | 5.080603 | 5.649353 |
| 9.142906 | 9.438855 | 9.189226 | 8.490343 |
| 0.612901 | 0.340875 | 0.465987 | 0.302802 |
| 0.845941 | 0.336429 | 0.397452 | 0.401237 |
| 0 | 0 | 0 | 0 |
| 0.114187 | 0.0877 | 0.139871 | 0.136334 |
| 0.100988 | 0.162883 | 0.061852 | 0.120575 |
| 0 | 0 | 0 | 0 |
| 13.29901 | 11.39989 | 10.44249 | 11.06672 |
| 0.79559 | 0.983783 | 0.77963 | 0.902398 |
| 0.336272 | 0 | 0.082381 | 0 |
| 0.177011 | 0.317221 | 0.289101 | 0.140895 |
| 13.25122 | 15.32502 | 14.46429 | 15.61032 |
| 0 | 0.270897 | 0 | 0.300799 |
| 0.0384 | 0.289029 | 0.047037 | 0.045848 |
| 0.250941 | 0.134913 | 0.768459 | 0.374513 |
| 0 | 0.059488 | 0.022589 | 0.044036 |
| 0 | 0 | 0 | 0 |
| 56.0623 | 65.88461 | 67.92739 | 65.50967 |
| 15.07135 | 17.31971 | 16.38437 | 17.60078 |
| 0 | 0 | 0.115042 | 0 |
| 8.594301 | 7.225032 | 7.286386 | 6.613498 |
| 0.451872 | 0.197388 | 0.276755 | 0.303476 |
| 0 | 0 | 0 | 0.789598 |
| 0.341334 | 0.183511 | 0 | 0 |
| 3.715265 | 3.872573 | 3.715036 | 3.055293 |
| 0.466497 | 0.394118 | 0.448976 | 0.278487 |
| 4.10226 | 3.849585 | 4.056512 | 3.517572 |
| 3393.125 | 3481.466 | 3465.4 | 3449.842 |
| 0 | 0.639994 | 0.729076 | 1.421277 |
| 6.231147 | 4.957288 | 4.897271 | 4.945443 |
| 5.109277 | 3.998436 | 3.481047 | 5.306102 |
| 3.612477 | 3.128565 | 3.36381 | 3.512941 |
| 8.049015 | 6.749771 | 7.394576 | 7.599296 |
| 66.89434 | 65.49243 | 67.14433 | 69.79228 |
| 31.74403 | 30.57311 | 26.77751 | 28.19302 |
| 0 | 0.082447 | 0 | 0 |
| 0.100953 | 0.072367 | 0.04122 | 0.16071 |

| | | | |
|---|---|---|---|
| 0 | 0 | 0 | 0 |
| 0 | 0 | 0 | 0 |
| 45.27179 | 37.43065 | 36.28011 | 36.62667 |
| 0.300618 | 0.225289 | 0.223172 | 0.261034 |
| 36.71467 | 25.83496 | 23.89314 | 22.67949 |
| 0.392386 | 0.274245 | 0.168225 | 0.140547 |
| 1.919406 | 1.408978 | 1.446848 | 1.189906 |
| 48.3933 | 48.93103 | 53.61101 | 50.50975 |
| 6.695374 | 7.634248 | 6.962457 | 7.003745 |
| 36.46784 | 32.71078 | 36.56951 | 43.31511 |
| 3.73133 | 4.088803 | 4.017468 | 4.157067 |
| 0 | 0 | 0 | 0 |
| 0.507417 | 0.720197 | 0.5221 | 0.484664 |
| 7.06515 | 5.466182 | 6.284824 | 5.238693 |
| 0.043684 | 0 | 0 | 0.026078 |
| 0.371839 | 0.355398 | 0.278346 | 0.320635 |
| 0 | 0.157295 | 0 | 0 |
| 0.097824 | 0.210373 | 0.179741 | 0 |
| 1.670993 | 0.640579 | 1.103514 | 1.179699 |
| 0 | 0.031449 | 0.035827 | 0 |
| 2.889214 | 2.962153 | 1.94786 | 3.262386 |
| 4.839897 | 4.455826 | 3.884529 | 3.701316 |
| 1.461291 | 1.156213 | 1.249603 | 1.28384 |
| 1.236051 | 0.785361 | 0.911882 | 0.905593 |
| 0 | 0 | 0 | 0 |
| 9.555601 | 8.010804 | 5.554863 | 7.3481 |
| 0.516163 | 0.277504 | 0.632261 | 0.924408 |
| 27.44338 | 25.472 | 24.1279 | 25.78724 |
| 8.98243 | 7.982981 | 7.999484 | 8.152851 |
| 0 | 0 | 0 | 0 |
| 0.711868 | 0.425245 | 0.387549 | 0.94437 |
| 10.88431 | 6.639214 | 6.514411 | 5.623226 |
| 7.030493 | 6.10938 | 6.561389 | 6.372621 |
| 0 | 0.016953 | 0 | 0.018825 |
| 10.67899 | 9.965001 | 9.807999 | 9.570569 |
| 0 | 0.437602 | 0 | 0 |
| 0 | 0 | 0 | 0 |
| 1.322178 | 0.985699 | 0.820581 | 0.841927 |
| 1.071538 | 0.961421 | 0.869241 | 0.898095 |
| 0 | 0 | 0 | 0 |
| 3.951393 | 3.555345 | 3.044847 | 3.387826 |
| 0.085232 | 0.061097 | 0.069602 | 0.033921 |
| 19.02257 | 17.60083 | 16.21589 | 17.33656 |
| 3.094916 | 2.564066 | 2.765594 | 2.907674 |
| 3.284636 | 3.387671 | 3.585506 | 3.067985 |
| 2.16653 | 1.843182 | 2.245553 | 2.188767 |
| 0.185839 | 0.133216 | 0.101173 | 0.073961 |
| 0.190581 | 0.252214 | 0.14366 | 0.332565 |
| 34.40269 | 35.29368 | 32.10137 | 32.96027 |
| 5.068199 | 3.677931 | 4.087678 | 4.059013 |
| 0.080842 | 0 | 0.264068 | 0.128695 |
| 0 | 0 | 0 | 0 |
| 4.584447 | 3.199059 | 3.826561 | 3.600623 |
| 153.0444 | 161.7807 | 162.427 | 163.8067 |

| | | | |
|---|---|---|---|
| 9.501223 | 7.902823 | 8.040994 | 8.493912 |
| 0 | 0.595343 | 0 | 0 |
| 8.502865 | 7.410452 | 6.980124 | 7.765374 |
| 0.145467 | 0.052138 | 0 | 0.173679 |
| 0.905535 | 0.540935 | 0.462172 | 0.630678 |
| 38.71095 | 42.0185 | 39.20487 | 41.8409 |
| 0.387122 | 0.138752 | 0 | 0.154068 |
| 0.489559 | 0.470991 | 0.72592 | 0.86138 |
| 0.843367 | 0.857818 | 0.97722 | 0.870864 |
| 0.075943 | 0 | 0.139536 | 0.045336 |
| 40.97456 | 39.7613 | 42.12438 | 40.52152 |
| 1.050354 | 0.45176 | 0.400277 | 0.445891 |
| 28.43288 | 34.35956 | 33.65877 | 35.33362 |
| 8.045669 | 9.557237 | 8.978678 | 8.889443 |
| 0.17912 | 0.2568 | 0.255976 | 0.392076 |
| 2.462899 | 1.7655 | 1.508433 | 3.920765 |
| 59.9461 | 48.45088 | 54.08975 | 51.58488 |
| 1.538684 | 1.967491 | 1.961182 | 1.688155 |
| 0.936335 | 0.785795 | 1.007069 | 0.722568 |
| 13.00526 | 12.32998 | 11.41969 | 12.43878 |
| 0 | 0 | 0 | 0 |
| 53.03844 | 56.36901 | 58.51429 | 58.37131 |
| 2.322734 | 1.248768 | 2.370978 | 1.386612 |
| 0 | 0.200782 | 0 | 0 |
| 121.9095 | 124.4409 | 120.5469 | 122.8687 |
| 0 | 0 | 0 | 0 |
| 0.633778 | 0.355552 | 0.371289 | 0.361899 |
| 0.404149 | 0.375305 | 0.29253 | 0.241266 |
| 1.722171 | 2.572696 | 2.946899 | 2.589848 |
| 27.21347 | 25.34051 | 22.44446 | 22.74044 |
| 41.35566 | 17.94121 | 19.20373 | 16.88113 |
| 0.564865 | 0.494898 | 0.384398 | 0.349699 |
| 0.318289 | 0.182529 | 0.233928 | 0.354686 |
| 4.862915 | 4.381611 | 3.557476 | 3.601913 |
| 0.137106 | 0.078048 | 0.098791 | 0.096292 |
| 5.948113 | 4.866574 | 4.386852 | 4.610553 |
| 0.523637 | 0.412899 | 0.171044 | 0.083359 |
| 5.650825 | 5.746967 | 5.450944 | 5.144428 |
| 3.246549 | 3.927233 | 3.976777 | 6.298842 |
| 8.667831 | 7.879202 | 7.858301 | 6.36596 |
| 0.040663 | 0.087446 | 0 | 0 |
| 0 | 0 | 0 | 0 |
| 72.69983 | 74.58018 | 74.9414 | 72.57586 |
| 0 | 0.409596 | 0.933217 | 0 |
| 15.76674 | 13.4268 | 13.62925 | 15.22797 |
| 0.800966 | 0.386075 | 0.169159 | 0.725478 |
| 2.131425 | 2.200157 | 2.245318 | 2.137642 |
| 2.132858 | 1.548025 | 1.796155 | 1.145935 |
| 9.407457 | 8.418214 | 8.597455 | 8.41773 |
| 0 | 0 | 0 | 0 |
| 0 | 0.063444 | 0.072275 | 0.070447 |
| 111.2953 | 115.5587 | 128.3642 | 133.7941 |
| 2.809642 | 1.987558 | 1.7208 | 1.85384 |
| 27.62797 | 22.16097 | 24.75593 | 22.37981 |

| | | | |
|---|---|---|---|
| 0.033842 | 0 | 0 | 0 |
| 0 | 0 | 0 | 0 |
| 0 | 0 | 0 | 0.536331 |
| 0 | 0.72112 | 0 | 0 |
| 0.61327 | 0.518118 | 0.590236 | 0.523009 |
| 6.908578 | 6.285651 | 6.070207 | 5.932563 |
| 3.525462 | 3.358917 | 3.03383 | 2.770625 |
| 8.201926 | 8.028401 | 8.046553 | 7.828186 |
| 2.595963 | 2.414121 | 2.40638 | 3.183214 |
| 2.996433 | 3.104774 | 2.869589 | 3.024686 |
| 9.343585 | 8.183108 | 8.511513 | 8.108147 |
| 0 | 0 | 0.036386 | 0 |
| 15.95289 | 15.03373 | 14.67439 | 12.52897 |
| 4.273235 | 2.789716 | 3.178022 | 1.45772 |
| 0 | 0 | 0.511632 | 0 |
| 46.1363 | 48.35823 | 44.53755 | 47.32332 |
| 0 | 0 | 0 | 0 |
| 126.1159 | 104.5031 | 101.2282 | 113.3297 |
| 0.175867 | 0.094551 | 0.053856 | 0 |
| 14.76689 | 12.23623 | 11.98571 | 12.21753 |
| 0 | 0 | 0 | 0.153652 |
| 23.44923 | 24.38915 | 24.8742 | 23.82172 |
| 0 | 0.197681 | 0.225197 | 0.219502 |
| 2.474927 | 2.673089 | 2.638237 | 2.452529 |
| 4.086106 | 3.660158 | 3.958709 | 3.850693 |
| 150.9746 | 137.556 | 141.5379 | 137.9586 |
| 0.289459 | 0.155622 | 0.709131 | 0 |
| 0 | 0 | 0 | 0.026199 |
| 13.92955 | 15.39008 | 16.24864 | 15.62413 |
| 1.370903 | 0.412741 | 0.649311 | 0.872953 |
| 0.591603 | 0.808051 | 0.587569 | 0.725434 |
| 0 | 0.328202 | 0 | 0.182215 |
| 6.586925 | 6.908065 | 7.15936 | 7.476763 |
| 3.946081 | 4.211621 | 3.831116 | 3.699334 |
| 11.78097 | 11.06345 | 11.17163 | 10.63203 |
| 16.69109 | 16.43281 | 15.12293 | 15.56339 |
| 0 | 0.787684 | 0 | 0 |
| 0.035842 | 0.077079 | 0.043904 | 0.12838 |
| 0 | 0 | 0 | 0 |
| 14.50583 | 13.77472 | 13.65497 | 13.84768 |
| 0.095834 | 0.103047 | 0.06708 | 0.065384 |
| 1.886289 | 3.453499 | 2.747694 | 3.226025 |
| 1.218148 | 0.504524 | 0.49738 | 0.441708 |
| 0 | 0.243807 | 0 | 0 |
| 22.16103 | 17.77955 | 17.95724 | 17.98986 |
| 1.864118 | 0.392166 | 3.474744 | 1.403133 |
| 3.045644 | 2.356295 | 2.775265 | 2.572046 |
| 7.36915 | 5.688832 | 4.3976 | 4.511991 |
| 0.821249 | 0.688782 | 0.905372 | 0.705981 |
| 0 | 0.027556 | 0 | 0 |
| 0.159215 | 0.03424 | 0.065009 | 0.101384 |
| 103.3934 | 103.5296 | 100.338 | 102.3439 |
| 52.63824 | 43.60872 | 46.4287 | 45.82028 |
| 1.537211 | 1.310223 | 1.125188 | 2.999231 |

| | | | |
|---|---|---|---|
| 3.534553 | 3.109543 | 2.730575 | 3.165054 |
| 3.302534 | 3.216341 | 2.884802 | 2.79569 |
| 0.696203 | 0.544434 | 0.387634 | 0.428209 |
| 0.045642 | 0.110423 | 0.111816 | 0.081741 |
| 0.376908 | 0.495334 | 0.205193 | 0.850016 |
| 6.125512 | 4.866871 | 4.102784 | 4.071085 |
| 17.96044 | 16.06258 | 15.58973 | 16.25155 |
| 0.310055 | 0.273454 | 0.285912 | 0.386827 |
| 53.19152 | 55.02184 | 51.42777 | 52.6068 |
| 4.846221 | 6.14835 | 5.088218 | 4.684015 |
| 0.421875 | 0.320205 | 0.577561 | 0.407402 |
| 25.95438 | 30.81911 | 29.14955 | 31.61898 |
| 0 | 0 | 0 | 0.394799 |
| 19.33403 | 15.16226 | 15.8048 | 15.5482 |
| 1.895768 | 1.69147 | 1.531646 | 1.58923 |
| 0.413906 | 0.236152 | 0.10347 | 0.090768 |
| 0.496082 | 0.355611 | 0.221845 | 0.329054 |
| 2.259155 | 1.214586 | 1.862602 | 0.570586 |
| 0.017048 | 0.018331 | 0.020883 | 0 |
| 0.824617 | 0.57514 | 0.368548 | 0.439056 |
| 9.287789 | 8.565034 | 8.019093 | 7.662287 |
| 0 | 0 | 0 | 0.096195 |
| 477.5782 | 462.4249 | 480.9804 | 455.0422 |
| 3.351949 | 2.937675 | 2.671637 | 3.015246 |
| 15.24746 | 14.0304 | 13.71569 | 13.5028 |
| 24.67748 | 19.99853 | 20.3743 | 20.68954 |
| 6.517581 | 5.907633 | 6.499001 | 5.948785 |
| 0.011789 | 0.152115 | 0.043322 | 0.098529 |
| 40.86421 | 39.06061 | 41.33579 | 37.12757 |
| 10.10089 | 8.141196 | 8.060134 | 8.794982 |
| 0 | 0 | 0 | 0 |
| 14.92997 | 9.822443 | 9.825913 | 9.986432 |
| 0 | 0 | 0 | 0 |
| 53.04485 | 70.85046 | 69.23999 | 71.00201 |
| 0.314297 | 0.211219 | 0.481238 | 0.046907 |
| 0 | 0 | 0 | 0 |
| 0 | 0 | 0 | 0 |
| 0.384 | 0.412899 | 0 | 0 |
| 129.0315 | 102.8901 | 107.4207 | 98.16015 |
| 0 | 0 | 0.498513 | 0 |
| 0.347 | 0.400752 | 0.472276 | 0.537055 |
| 0.038524 | 0 | 0.047189 | 0 |
| 1.351681 | 1.387341 | 1.429929 | 1.540481 |
| 0.138018 | 0.148404 | 0 | 0.164786 |
| 0.107485 | 0 | 0 | 0 |
| 0.054356 | 0.116894 | 0.066582 | 0.086531 |
| 6.301138 | 6.511056 | 6.185683 | 7.363163 |
| 0.032 | 0.068817 | 0.039198 | 0.038206 |
| 0 | 0 | 0 | 0 |
| 12.11822 | 13.95153 | 9.446123 | 13.88394 |
| 13.86875 | 15.35813 | 14.60592 | 14.57915 |
| 1.116001 | 0.399996 | 1.367017 | 0 |
| 3.89798 | 3.137223 | 3.54531 | 3.093368 |
| 0.462292 | 0 | 0 | 0 |

| | | | |
|---|---|---|---|
| 9.537183 | 8.902625 | 9.970632 | 7.88324 |
| 0.048155 | 0.155338 | 0.070784 | 0.022998 |
| 1.454362 | 1.034333 | 0.841646 | 0.847707 |
| 0 | 0 | 0 | 0.14805 |
| 2.047243 | 1.366332 | 1.85917 | 1.580368 |
| 2.797327 | 2.181515 | 1.995662 | 2.86274 |
| 3.9316 | 3.28804 | 2.894412 | 3.366495 |
| 24.81328 | 21.70026 | 21.94631 | 22.78905 |
| 0.164193 | 0.17655 | 0.201124 | 0.130692 |
| 1.337433 | 0.663731 | 0.462072 | 0.573219 |
| 13.42479 | 13.63315 | 13.84637 | 13.5797 |
| 3.548561 | 2.873685 | 3.310054 | 3.066803 |
| 1.074046 | 0 | 0 | 0.854904 |
| 0 | 0 | 0 | 0 |
| 4.884913 | 4.921066 | 5.98365 | 4.728153 |
| 14.46178 | 12.04169 | 10.39843 | 10.76275 |
| 22.5801 | 20.79521 | 20.05481 | 21.66531 |
| 0 | 0.03026 | 0 | 0 |
| 48.80605 | 62.27195 | 60.94986 | 58.00667 |
| 2.441848 | 2.120689 | 2.147442 | 1.75674 |
| 21.181 | 13.82453 | 12.0135 | 13.67772 |
| 131.5979 | 100.3779 | 96.09786 | 98.3836 |
| 12.52614 | 12.43921 | 12.88179 | 12.92826 |
| 776.0781 | 799.9408 | 775.8144 | 796.6732 |
| 0.946446 | 0.666751 | 0.73957 | 0.662418 |
| 0.035667 | 0.038352 | 0 | 0 |
| 0.034845 | 0 | 0.042683 | 0 |
| 0.97308 | 0.627787 | 0.874096 | 0.697084 |
| 1.142785 | 0 | 0 | 0.454809 |
| 0.014875 | 0.015995 | 0.018221 | 0.053281 |
| 1.515056 | 1.939374 | 0.972101 | 1.464346 |
| 0.268259 | 0.072112 | 0 | 0 |
| 0.312236 | 0.335734 | 0.509955 | 0.465993 |
| 0 | 0.053333 | 0 | 0 |
| 9.986728 | 7.158866 | 7.484317 | 5.584487 |
| 0.126414 | 0.045309 | 0.154848 | 0.201243 |
| 4.982448 | 4.112176 | 3.958782 | 4.324927 |
| 12.51282 | 11.96429 | 10.74364 | 10.96836 |
| 0.132882 | 0.119069 | 0.16277 | 0.167468 |
| 3.010538 | 1.413671 | 1.143648 | 1.387722 |
| 0.36157 | 0.209344 | 0.306621 | 0.232452 |
| 0.805686 | 0.606424 | 0.542798 | 0.673363 |
| 0 | 0 | 0 | 0 |
| 0.055756 | 0 | 0.068297 | 0.06657 |
| 16.87517 | 17.11282 | 15.94411 | 17.08398 |
| 0 | 0 | 0 | 0 |
| 0.041441 | 0.08912 | 0.050762 | 0 |
| 2.238361 | 1.75041 | 2.866452 | 2.55101 |
| 0 | 0 | 0 | 0 |
| 5.573661 | 4.643674 | 3.978832 | 4.010426 |
| 0 | 0 | 0 | 0 |
| 74.63842 | 68.53936 | 68.93686 | 66.93337 |
| 13.18344 | 10.7775 | 11.6421 | 11.51664 |
| 59.47645 | 68.07844 | 68.72362 | 69.207 |

| | | | |
|---|---|---|---|
| 4.690231 | 4.303169 | 3.934199 | 4.078183 |
| 18.23051 | 21.04182 | 21.94059 | 22.14681 |
| 0.113642 | 0 | 0.139203 | 0 |
| 0.266011 | 0.143015 | 0.217229 | 0.26467 |
| 20.18763 | 13.24416 | 12.58108 | 13.8134 |
| 6.868591 | 7.984325 | 7.238647 | 9.050368 |
| 0 | 0.047805 | 0 | 0.053082 |
| 12.18665 | 10.81457 | 11.3127 | 11.97326 |
| 0.072919 | 0.086247 | 0.08932 | 0.130592 |
| 0.332979 | 0 | 0 | 0 |
| 6.258109 | 4.778619 | 4.808924 | 3.89836 |
| 23.16671 | 22.03775 | 20.6953 | 22.99179 |
| 13.65895 | 11.86773 | 11.29226 | 10.24935 |
| 68.58278 | 61.83971 | 63.24657 | 60.4189 |
| 0 | 0.334637 | 0 | 0 |
| 27.16513 | 22.50567 | 24.21161 | 22.37233 |
| 12.79348 | 11.86346 | 11.93551 | 11.69288 |
| 22.27303 | 20.00822 | 19.83853 | 19.86048 |
| 0 | 0 | 0 | 0 |
| 0.319929 | 0.057334 | 0.032657 | 0 |
| 0.527309 | 0.198448 | 0.419844 | 0.377748 |
| 11.55483 | 9.59137 | 9.643242 | 10.4227 |
| 0.687173 | 0.468563 | 0.533783 | 0.660361 |
| 0 | 0 | 0 | 0 |
| 0 | 0.358875 | 0.136276 | 0.332074 |
| 4.056745 | 4.552883 | 3.571617 | 4.2381 |
| 2.706995 | 3.696609 | 3.415341 | 3.102731 |
| 5.67643 | 4.61038 | 4.826838 | 5.098566 |
| 1.436691 | 2.206874 | 1.508433 | 1.225239 |
| 0 | 0 | 0 | 0 |
| 6.752111 | 5.749451 | 4.972058 | 4.520127 |
| 0.827451 | 1.294142 | 1.934988 | 1.25737 |
| 0 | 0.099224 | 0 | 0.33053 |
| 0.073059 | 0.058918 | 0.067119 | 0.109035 |
| 1.967924 | 1.680372 | 1.819735 | 1.773717 |
| 3.668777 | 3.441277 | 3.466098 | 3.541543 |
| 0 | 0 | 0 | 0 |
| 23.62447 | 21.26927 | 21.00267 | 21.11612 |
| 2.976003 | 0.639994 | 0.729076 | 0.710639 |
| 5.186748 | 5.065092 | 4.718162 | 5.035382 |
| 66.98889 | 76.37956 | 77.44936 | 78.11729 |
| 5977.109 | 6892.503 | 6738.09 | 6975.579 |
| 22.8622 | 19.03693 | 19.00756 | 18.56218 |
| 0.355344 | 1.146257 | 1.305807 | 2.121309 |
| 168.6096 | 163.9696 | 151.1991 | 159.3288 |
| 0 | 0 | 0 | 0 |
| 0.042949 | 0 | 0.035073 | 0.034186 |
| 0 | 0 | 0 | 0 |
| 0.13282 | 0 | 0.244042 | 0 |
| 0 | 0 | 0 | 0 |
| 10.759 | 9.117413 | 8.655405 | 9.310967 |
| 0.136436 | 0.097802 | 0.083562 | 0.162897 |
| 1.713036 | 1.739623 | 1.126886 | 1.628645 |
| 0.013882 | 0 | 0 | 0 |

| | | | |
|---|---|---|---|
| 23.1288 | 26.36802 | 23.62 | 25.31864 |
| 0.334209 | 0.189137 | 0.086186 | 0.147011 |
| 0.029872 | 0 | 0.073182 | 0 |
| 0.12547 | 0.269826 | 0.076846 | 0.149805 |
| 6.775348 | 7.035639 | 7.339629 | 7.30992 |
| 169.3209 | 87.18004 | 99.74291 | 78.13113 |
| 1.80941 | 1.126389 | 0.933217 | 1.478128 |
| 4.988011 | 4.983011 | 4.983281 | 5.617529 |
| 0 | 0 | 0 | 0.189188 |
| 15.25009 | 14.15944 | 14.31708 | 15.70401 |
| 0.509444 | 0.584302 | 0.374418 | 0.44605 |
| 16.87405 | 14.50087 | 15.35967 | 14.49534 |
| 20.33187 | 17.15085 | 18.98369 | 16.81755 |
| 0.235012 | 0.042116 | 0.191915 | 0.187061 |
| 25.68697 | 26.46002 | 25.30234 | 26.63086 |
| 0 | 0 | 0 | 0 |
| 0.245023 | 0.146368 | 0.233438 | 0.260039 |
| 0.61874 | 0.491746 | 0.428383 | 0.41755 |
| 1.752299 | 1.268932 | 1.839801 | 1.344956 |
| 1.900533 | 1.436016 | 1.321302 | 1.103905 |
| 55.55205 | 44.60945 | 41.86772 | 43.52955 |
| 0 | 0 | 0 | 0 |
| 0.200912 | 0 | 0.246102 | 0 |
| 1.001984 | 0.819754 | 1.040584 | 0.975259 |
| 4.755046 | 4.93659 | 4.673047 | 4.828949 |
| 2.65504 | 3.230488 | 2.695922 | 2.419195 |
| 5.017364 | 4.418153 | 4.759215 | 4.638862 |
| 0.8536 | 0.339202 | 0.545528 | 0.420955 |
| 7.150103 | 6.258885 | 6.753002 | 6.565524 |
| 0.024232 | 0.078167 | 0 | 0 |
| 7.433729 | 5.508805 | 5.59881 | 5.517194 |
| 2.576906 | 2.49201 | 2.580799 | 2.070479 |
| 0.165333 | 1.066656 | 0.202521 | 0.1974 |
| 7.978176 | 7.980083 | 7.375984 | 7.310289 |
| 1080.823 | 1074.432 | 1044.91 | 1101.083 |
| 0 | 0 | 0 | 0 |
| 0.311336 | 0.157537 | 0.246764 | 0.065597 |
| 16.81942 | 11.3567 | 11.20388 | 11.04623 |
| 1.857851 | 0.665889 | 0.505717 | 1.068012 |
| 0.219429 | 0.393237 | 0.358378 | 0.087329 |
| 0 | 0.07975 | 0.136276 | 0 |
| 0 | 0 | 0 | 0 |
| 2.733247 | 3.145185 | 3.17181 | 3.549615 |
| 0.164477 | 0 | 0 | 0 |
| 24.18778 | 23.1183 | 23.33042 | 21.48483 |
| 1.653538 | 1.243011 | 1.398105 | 0.995855 |
| 0 | 0 | 0.128755 | 0 |
| 0.38335 | 0.390505 | 0.469574 | 0.457699 |
| 9.577391 | 9.389999 | 9.369648 | 9.874737 |
| 2.08436 | 1.369636 | 0.936167 | 0.940144 |
| 0.105657 | 0 | 0 | 0 |
| 7.08599 | 7.716321 | 7.850522 | 7.813657 |
| 18.01542 | 17.12484 | 16.58388 | 16.48844 |
| 0 | 0 | 0 | 0 |

| | | | |
|---|---|---|---|
| 23.00097 | 21.65131 | 20.90841 | 20.86146 |
| 3.304056 | 2.788202 | 3.201914 | 3.320683 |
| 0.621079 | 0.779123 | 0.507183 | 0.247179 |
| 0 | 0.039278 | 0.067119 | 0.021807 |
| 69.20937 | 64.13466 | 62.58029 | 65.62515 |
| 0.116706 | 0.125489 | 0.285912 | 0.139341 |
| 0.69576 | 0.537711 | 0.612557 | 0.402371 |
| 2.516421 | 2.073707 | 2.00863 | 2.037872 |
| 2.311351 | 1.374082 | 1.470062 | 1.485956 |
| 2.054025 | 1.606258 | 1.601108 | 1.560618 |
| 0 | 0.46545 | 0 | 0 |
| 0 | 0 | 0 | 0 |
| 0 | 0 | 0 | 0.178777 |
| 7.887804 | 10.54523 | 9.533138 | 10.83027 |
| 0.030042 | 0.12921 | 0 | 0.035868 |
| 0.097975 | 0.158023 | 0 | 0.116978 |
| 0 | 0 | 0 | 0 |
| 0.341159 | 0.445441 | 0.358195 | 0.174568 |
| 51.09362 | 47.24447 | 49.06926 | 42.87786 |
| 6.240338 | 5.979179 | 5.953698 | 5.999855 |
| 6.953238 | 5.46079 | 5.57375 | 6.063573 |
| 20.43794 | 19.60476 | 19.25178 | 18.87619 |
| 59.80575 | 57.56188 | 60.69021 | 62.58168 |
| 33.31835 | 33.51743 | 32.39752 | 30.19413 |
| 0 | 0 | 0 | 0 |
| 0.288582 | 0.077575 | 0.088373 | 0.172276 |
| 0 | 0 | 0 | 0.076413 |
| 9.175546 | 8.400496 | 8.734996 | 7.971652 |
| 10.5862 | 7.92569 | 9.484348 | 9.531763 |
| 5.198258 | 5.291359 | 6.367474 | 3.558365 |
| 0.02762 | 0.059396 | 0.033832 | 0 |
| 0.857432 | 1.434159 | 1.330366 | 1.160226 |
| 0.290145 | 0.450639 | 0.355406 | 0.269436 |
| 14.79464 | 15.8253 | 13.52693 | 16.51553 |
| 0 | 0 | 0.106629 | 0 |
| 0 | 0.174148 | 0 | 0.193371 |
| 13.51601 | 10.26656 | 12.91072 | 12.43618 |
| 44.35997 | 38.75493 | 40.51627 | 39.33772 |
| 32.44578 | 28.66239 | 30.56619 | 31.78929 |
| 0.06931 | 0 | 0.0849 | 0.041376 |
| 0.358846 | 0.415532 | 0.371934 | 0.247179 |
| 4.708387 | 3.966772 | 3.955433 | 4.146177 |
| 3.777571 | 3.485176 | 3.141952 | 3.229543 |
| 12.66709 | 20.18441 | 19.25508 | 14.39499 |
| 3.981546 | 4.2375 | 3.632937 | 3.395543 |
| 52.48956 | 122.5061 | 122.5776 | 130.9428 |
| 0 | 0.492303 | 0 | 0 |
| 0.099772 | 0.02682 | 0 | 0.029781 |
| 4.427277 | 3.729845 | 4.039356 | 3.691983 |
| 12.54302 | 12.09861 | 12.65291 | 11.73517 |
| 31.41599 | 41.4317 | 33.9849 | 34.97833 |
| 14.35945 | 6.965698 | 5.631482 | 6.629657 |
| 0.439827 | 0.493489 | 0.374786 | 0.707785 |
| 0.027132 | 0.029173 | 0.033234 | 0.032394 |

| | | | |
|---|---|---|---|
| 20.84065 | 21.57428 | 21.91454 | 19.85668 |
| 9.019967 | 9.056783 | 9.011415 | 9.190883 |
| 16.09715 | 14.68606 | 15.16753 | 14.42557 |
| 2.525512 | 1.169481 | 1.987105 | 1.694748 |
| 0 | 0 | 0 | 0 |
| 3.219752 | 3.608345 | 2.555237 | 3.411065 |
| 5.78387 | 2.603364 | 2.141917 | 3.854311 |
| 0.031744 | 0.034133 | 0.038884 | 0.037901 |
| 7.241688 | 6.656352 | 5.436771 | 5.252799 |
| 8.756556 | 8.380229 | 7.321356 | 8.459338 |
| 1.595599 | 1.264185 | 1.954489 | 1.303464 |
| 0.058353 | 0.125489 | 0.214434 | 0.278682 |
| 33.42044 | 34.7485 | 33.43845 | 32.51296 |
| 10.31681 | 6.460888 | 3.471789 | 5.00831 |
| 19.12683 | 15.39896 | 16.53393 | 15.63576 |
| 3.413981 | 1.849254 | 1.980885 | 2.696979 |
| 0.145234 | 0.066927 | 0.050829 | 0.024772 |
| 0.921084 | 0.516732 | 0.637711 | 0.382514 |
| 2.225049 | 0.4785 | 0.545103 | 0 |
| 0.29123 | 0 | 0 | 0.173857 |
| 27.47918 | 29.1824 | 28.94041 | 31.01494 |
| 72.86011 | 74.79459 | 73.60193 | 77.96721 |
| 0 | 0 | 0 | 0 |
| 0 | 0 | 0 | 0 |
| 0.016016 | 0 | 0 | 0 |
| 0 | 0 | 0.972101 | 0 |
| 2.282422 | 2.708072 | 2.458371 | 1.973344 |
| 0.196461 | 0.166774 | 0.215319 | 0.123455 |
| 0 | 1.442239 | 0.821494 | 0.80072 |
| 14.06497 | 12.54468 | 11.7815 | 10.40977 |
| 510.1974 | 536.1057 | 521.1045 | 530.859 |
| 5.568876 | 3.527798 | 3.728003 | 4.38109 |
| 0.246078 | 0.132298 | 0.45214 | 0 |
| 0 | 0.207285 | 0 | 0 |
| 3.225372 | 2.851552 | 2.809484 | 3.137792 |
| 0.998938 | 0.978638 | 1.49554 | 0.980648 |
| 54.17321 | 53.24219 | 49.6935 | 52.38787 |
| 10.28944 | 9.180598 | 7.374559 | 8.887067 |
| 9.763227 | 8.965623 | 9.095742 | 7.2687 |
| 11.35409 | 8.816739 | 8.541953 | 8.404171 |
| 30.49567 | 25.36213 | 23.36462 | 27.05079 |
| 1.120378 | 0.602347 | 0.686189 | 0.668836 |
| 2.722726 | 1.429773 | 1.396102 | 0.982798 |
| 15.39651 | 13.17012 | 12.4927 | 10.80641 |
| 8.643072 | 8.033962 | 8.68686 | 8.542783 |
| 0.13017 | 0.279932 | 0.159448 | 0.124333 |
| 3.597907 | 3.445538 | 3.029925 | 3.154665 |
| 27.25113 | 27.38944 | 29.08308 | 28.62037 |
| 2.480002 | 1.599984 | 0.303782 | 1.776596 |
| 0 | 0 | 0 | 0.027773 |
| 5.889119 | 3.532096 | 3.334989 | 4.098649 |
| 0 | 0 | 0 | 0.121998 |
| 17.89262 | 16.03263 | 16.80311 | 15.44599 |
| 0.717109 | 1.20802 | 0.673443 | 0.97035 |

| | | | |
|---|---|---|---|
| 3.574143 | 3.00876 | 3.103522 | 3.1233 |
| 0.059894 | 0.064402 | 0 | 0.143022 |
| 2.330546 | 2.280283 | 2.462385 | 2.030867 |
| 0.50343 | 0.496206 | 0.205554 | 0.55098 |
| 0 | 0 | 0 | 0 |
| 1.451709 | 1.717056 | 0.711293 | 1.906591 |
| 73.94637 | 77.12998 | 82.44019 | 77.41737 |
| 0 | 0 | 0 | 0.10891 |
| 1.843902 | 1.438406 | 1.682908 | 1.554016 |
| 0.058496 | 0.041932 | 0.023885 | 0.046561 |
| 0 | 0 | 0 | 0 |
| 0.942044 | 0.966897 | 0.681869 | 0.664626 |
| 26.26828 | 28.73924 | 28.07249 | 27.88835 |
| 1.066429 | 0.731012 | 0.718462 | 0.668462 |
| 8.131735 | 7.109566 | 7.555189 | 6.869261 |
| 0 | 0.147125 | 0.167604 | 0 |
| 3.094264 | 2.297873 | 2.563184 | 2.25916 |
| 14.21058 | 13.51256 | 14.35419 | 12.47179 |
| 4.042311 | 2.716577 | 3.640828 | 3.513269 |
| 0 | 0 | 0 | 0 |
| 4.88647 | 3.774152 | 3.661186 | 3.639033 |
| 0 | 0 | 0 | 0 |
| 1.176559 | 1.339521 | 1.186867 | 1.570016 |
| 10.71765 | 7.352193 | 7.303862 | 8.004797 |
| 7.568697 | 7.312278 | 7.481655 | 6.886485 |
| 53.63976 | 44.90236 | 49.19065 | 42.95225 |
| 10.69589 | 9.052864 | 8.760748 | 9.282857 |
| 0.064696 | 0 | 0.039624 | 0 |
| 11.73149 | 13.35639 | 23.66855 | 5.767502 |
| 0 | 0 | 0 | 0 |
| 1.785602 | 1.279987 | 1.458151 | 0 |
| 0 | 0 | 0 | 0 |
| 0.077677 | 0 | 0.031716 | 0.030914 |
| 8.242693 | 6.315236 | 6.545857 | 7.13272 |
| 1.873711 | 1.375127 | 1.639396 | 1.455899 |
| 21.29241 | 25.19624 | 23.52791 | 24.92421 |
| 42.03007 | 43.06877 | 42.27883 | 43.2175 |
| 12.5817 | 6.00615 | 5.98689 | 5.426843 |
| 4.293735 | 4.202943 | 3.554698 | 2.934478 |
| 6.104621 | 4.223815 | 4.48662 | 4.373161 |
| 8.528247 | 5.3492 | 5.223229 | 5.939666 |
| 19.15857 | 16.83202 | 16.77089 | 19.13633 |
| 0.109714 | 0.023594 | 0.16127 | 0.039298 |
| 18.87589 | 16.36044 | 17.34115 | 17.1308 |
| 0.363481 | 0.390836 | 0 | 0 |
| 3.896256 | 2.965585 | 2.734872 | 2.543751 |
| 0.050872 | 0 | 0 | 0.060738 |
| 0.044673 | 0.030022 | 0.088922 | 0.066672 |
| 0.280094 | 0 | 0 | 0 |
| 0 | 0.025246 | 0 | 0.056066 |
| 1.428481 | 0.97279 | 0.787402 | 0.909617 |
| 0 | 0.026778 | 0 | 0.029734 |
| 8.084498 | 8.355841 | 7.82127 | 8.509934 |
| 0.027829 | 0.009975 | 0.011363 | 0 |

| | | | |
|---|---|---|---|
| 0 | 0.011893 | 0 | 0 |
| 0 | 0.111062 | 0 | 0 |
| 0 | 0 | 0.062649 | 0.183194 |
| 1.552414 | 2.721288 | 2.205204 | 2.383073 |
| 21.34512 | 20.49941 | 21.49051 | 21.78203 |
| 177.5788 | 186.8751 | 184.2811 | 178.9417 |
| 0.142991 | 0.307504 | 0 | 0.170724 |
| 1.720663 | 0.871708 | 0.648518 | 1.027191 |
| 0 | 0 | 0 | 0 |
| 2.411346 | 2.627388 | 2.362973 | 1.573865 |
| 4.129815 | 3.786207 | 3.780721 | 3.702414 |
| 16.05093 | 13.7961 | 14.62065 | 16.02084 |
| 4.551994 | 3.094844 | 2.950793 | 2.652055 |
| 0 | 0 | 0 | 0.059158 |
| 500.2859 | 470.6086 | 503.1109 | 442.112 |
| 0.06452 | 0.069376 | 0 | 0.077034 |
| 2.939262 | 3.950578 | 2.700281 | 3.684793 |
| 4.721378 | 3.801829 | 3.682659 | 3.109241 |
| 37.43259 | 28.93884 | 30.49953 | 29.72824 |
| 0.225668 | 0.121326 | 0.10366 | 0.168398 |
| 0.074633 | 0.1605 | 0 | 0.089108 |
| 3.450438 | 1.669548 | 1.373621 | 2.059822 |
| 3.995521 | 3.657106 | 4.530179 | 4.494469 |
| 0 | 0.050196 | 0 | 0 |
| 4.272239 | 4.140345 | 4.009832 | 3.895181 |
| 0.162605 | 0.131131 | 0.066393 | 0.113249 |
| 7.189373 | 9.36197 | 8.18689 | 10.52478 |
| 0.103065 | 0 | 0 | 0 |
| 28.89063 | 32.45229 | 30.8785 | 29.19583 |
| 0.137222 | 0.032789 | 0.205439 | 0.109224 |
| 44.75027 | 36.97741 | 38.506 | 39.95368 |
| 0.115461 | 0.2483 | 0.198003 | 0.110283 |
| 13.48885 | 13.37154 | 13.4465 | 12.76792 |
| 0.147692 | 0.031761 | 0.036182 | 0.070535 |
| 6.248698 | 5.625172 | 5.547799 | 5.942471 |
| 0.023832 | 0 | 0 | 0 |
| 0 | 0.057142 | 0.32548 | 0.095175 |
| 0.065981 | 0.047297 | 0.02694 | 0 |
| 0.527018 | 1.03419 | 0.952196 | 0.880924 |
| 0.141714 | 1.110193 | 0.917544 | 0.942684 |
| 1.556457 | 1.549621 | 1.15334 | 1.307713 |
| 4.250703 | 3.537819 | 3.529603 | 3.074351 |
| 9.385159 | 5.542521 | 5.850334 | 5.237159 |
| 0 | 0 | 0 | 0 |
| 0.420237 | 0.069517 | 0.079194 | 0.038595 |
| 0.071964 | 0.128966 | 0.1763 | 0.143202 |
| 3.048757 | 1.708636 | 2.104899 | 2.029608 |
| 7.648712 | 7.320865 | 7.310652 | 7.601519 |
| 0.863268 | 0.806099 | 0.695683 | 0.569596 |
| 0.139381 | 0.056201 | 0 | 0.145612 |
| 0.073312 | 0 | 0 | 0 |
| 0.629842 | 1.896277 | 1.080112 | 0.1504 |
| 12.88704 | 11.38795 | 11.35583 | 11.37707 |
| 1.458987 | 0.92701 | 0.568638 | 0.554259 |

| | | | |
|---|---|---|---|
| 0 | 0.492303 | 0 | 0 |
| 2.851798 | 2.981908 | 3.08065 | 2.949125 |
| 3.162003 | 1.399986 | 1.670799 | 2.146721 |
| 7.578657 | 6.071807 | 4.914679 | 5.702851 |
| 0.93314 | 1.031236 | 0.762017 | 1.052225 |
| 0.721455 | 0 | 0 | 0 |
| 0.006944 | 0.014933 | 0 | 0 |
| 0.573411 | 0.76454 | 0.67429 | 0.492929 |
| 2.633038 | 2.367679 | 2.083583 | 2.211726 |
| 6.322462 | 5.759647 | 5.351261 | 5.242147 |
| 0 | 0 | 0 | 0 |
| 39.83691 | 40.24518 | 39.0649 | 38.40754 |
| 1.277331 | 0.896563 | 0.869241 | 0.931985 |
| 0.070101 | 0.094221 | 0.085868 | 0.104621 |
| 0 | 0 | 0 | 0.191418 |
| 0 | 0 | 0 | 0 |
| 4.831144 | 3.817333 | 4.214181 | 4.544591 |
| 0.360728 | 0.387875 | 0 | 0 |
| 1.281399 | 1.557551 | 1.29664 | 1.330369 |
| 57.34122 | 50.09005 | 51.54959 | 50.48188 |
| 16.34635 | 15.6541 | 14.61504 | 15.24742 |
| 21.61036 | 17.80729 | 16.98506 | 16.46183 |
| 1.330883 | 0.477014 | 1.086821 | 0.882781 |
| 0.251272 | 0.180121 | 0.076947 | 0.050001 |
| 11.75943 | 12.70819 | 13.33271 | 12.25193 |
| 37.9507 | 37.95385 | 39.69654 | 40.616 |
| 0.160323 | 0.258583 | 0.392768 | 0.478545 |
| 0 | 0 | 0 | 0 |
| 0 | 0 | 0 | 0 |
| 14.43995 | 15.82607 | 14.91042 | 15.72073 |
| 0.989191 | 0.701032 | 0.688457 | 1.234726 |
| 0 | 0 | 0 | 0 |
| 0 | 0 | 0 | 0 |
| 5.44071 | 5.413162 | 5.090683 | 5.165435 |
| 48.39142 | 48.48452 | 44.71259 | 49.04148 |
| 0 | 0 | 0 | 0 |
| 0.87169 | 0.507699 | 0.444897 | 0.563741 |
| 0 | 0.124271 | 0 | 0 |
| 0 | 0 | 0 | 0.117704 |
| 0 | 0 | 0 | 0 |
| 1.859172 | 1.53073 | 1.587635 | 1.446012 |
| 12.69608 | 11.18292 | 9.196057 | 9.676198 |
| 108.3297 | 91.41785 | 91.28888 | 87.12086 |
| 12.46789 | 14.50652 | 15.55362 | 15.3099 |
| 8.431214 | 8.492288 | 8.336738 | 8.277518 |
| 0 | 0.011472 | 0 | 0 |
| 1.963741 | 2.915917 | 2.634524 | 2.277616 |
| 52.17925 | 64.21269 | 69.99127 | 70.35322 |
| 98.80329 | 83.19917 | 94.05077 | 96.64685 |
| 5.318341 | 5.221315 | 4.052539 | 4.396031 |
| 0 | 0.015935 | 0 | 0 |
| 19.48786 | 15.05016 | 14.40842 | 15.68306 |
| 0 | 0 | 0 | 0.125223 |
| 0.697671 | 0.562632 | 0.213649 | 0.208246 |

| | | | |
|---|---|---|---|
| 10.03132 | 8.589573 | 9.336019 | 9.318825 |
| 0 | 0.379255 | 0 | 0 |
| 0.028304 | 0.050723 | 0.046226 | 0.033793 |
| 0 | 0 | 0 | 0 |
| 0.057214 | 0.04614 | 0.087603 | 0.017078 |
| 0 | 0.054526 | 0.062115 | 0 |
| 1.137905 | 1.059561 | 1.250152 | 1.554686 |
| 0.187096 | 0.100588 | 0.11459 | 0.223383 |
| 0 | 0.538942 | 0 | 2.39373 |
| 83.68283 | 88.70738 | 86.93081 | 85.1476 |
| 3.578506 | 3.45518 | 2.728443 | 2.703043 |
| 0.987764 | 0.843433 | 1.067591 | 0.763102 |
| 1.714705 | 1.749198 | 1.88496 | 1.627316 |
| 4.246545 | 3.834833 | 3.840315 | 4.020474 |
| 0.634881 | 0.853325 | 0 | 0.568511 |
| 6.280473 | 5.951565 | 6.437553 | 6.56402 |
| 13.38424 | 11.984 | 9.446749 | 11.94051 |
| 8.872555 | 8.215238 | 8.0304 | 8.356992 |
| 0.149642 | 0.225265 | 0.07332 | 0.178665 |
| 6.946817 | 7.252052 | 7.517948 | 7.207043 |
| 0.031702 | 0 | 0 | 0 |
| 0 | 0.118517 | 0 | 0 |
| 0.424385 | 0.273794 | 0.103968 | 0.405355 |
| 13.00607 | 12.73132 | 14.23566 | 12.13577 |
| 0.993035 | 1.308016 | 1.064344 | 1.748808 |
| 1.965911 | 1.958238 | 1.728508 | 1.569597 |
| 93.63404 | 94.31652 | 90.22596 | 98.82691 |
| 173.6994 | 196.5475 | 197.0921 | 194.1303 |
| 6.043792 | 5.952016 | 5.465906 | 5.192804 |
| 1.815866 | 1.979641 | 1.482866 | 1.83682 |
| 108.6399 | 116.1834 | 118.2368 | 113.0602 |
| 0.326324 | 0.175441 | 0.366412 | 0.194807 |
| 16.53377 | 14.47013 | 13.53269 | 13.6971 |
| 58.05292 | 41.48859 | 40.72063 | 42.62783 |
| 71.69905 | 81.20332 | 79.7241 | 80.16176 |
| 0.380928 | 0 | 0 | 0.454809 |
| 0.787634 | 0.769917 | 0.657813 | 0.940394 |
| 15.03139 | 12.24277 | 12.63171 | 13.08738 |
| 4.483678 | 3.599319 | 3.611288 | 3.703296 |
| 1.507994 | 2.407653 | 1.343403 | 2.182383 |
| 0 | 0 | 0.015142 | 0.044277 |
| 12.91641 | 11.04138 | 9.730095 | 11.42335 |
| 0.857382 | 0.719536 | 0.922151 | 1.023669 |
| 12.32894 | 7.567948 | 7.4777 | 6.802694 |
| 13.47862 | 13.13849 | 10.98628 | 12.6035 |
| 10.88903 | 10.09354 | 8.508878 | 9.246357 |
| 25.66231 | 21.85947 | 20.691 | 21.78376 |
| 0 | 0 | 0 | 0 |
| 53.55008 | 59.85416 | 59.38242 | 62.80832 |
| 0.690088 | 0 | 0 | 0 |
| 0.075401 | 0 | 0 | 0.045013 |
| 0 | 0 | 0 | 0 |
| 4.149731 | 2.686323 | 3.50112 | 3.640087 |
| 0.807873 | 0.625443 | 0.791667 | 0.694482 |

| | | | |
|---|---|---|---|
| 1.039003 | 0.478798 | 0.290903 | 0.74431 |
| 10.26058 | 10.84576 | 11.22638 | 10.71408 |
| 1.400472 | 1.183181 | 1.654205 | 0.656893 |
| 0 | 0 | 0 | 0 |
| 0 | 0 | 0 | 0 |
| 0.066689 | 0 | 0 | 0 |
| 0.142053 | 0.168017 | 0.104402 | 0.118722 |
| 0.422128 | 0.363117 | 0.103415 | 0.1008 |
| 0 | 0 | 0 | 0 |
| 80.581 | 83.42798 | 86.54121 | 82.78208 |
| 0 | 0.054526 | 0 | 0 |
| 31.79421 | 34.2517 | 34.38158 | 33.46719 |
| 12.55547 | 12.88175 | 13.80425 | 13.0107 |
| 0.135081 | 0.193662 | 0.055155 | 0.10752 |
| 0 | 0 | 0 | 0 |
| 51.56941 | 37.73837 | 40.7852 | 41.27473 |
| 0.561928 | 0.572417 | 0.760775 | 0.423735 |
| 17.10526 | 17.56592 | 16.3619 | 18.3002 |
| 3.182627 | 5.70357 | 6.37658 | 5.920761 |
| 11.36321 | 11.47952 | 10.02236 | 11.39453 |
| 0.638015 | 0.072214 | 0.205663 | 0.240555 |
| 16.31838 | 15.8052 | 13.31314 | 15.20739 |
| 2.428578 | 0.898988 | 1.511796 | 1.378496 |
| 7.829144 | 7.547479 | 7.771296 | 9.549049 |
| 10.94668 | 11.31778 | 10.41274 | 9.598857 |
| 1.434266 | 1.209906 | 0.504735 | 1.069092 |
| 0.69134 | 0.650447 | 0.49399 | 0.550283 |
| 0.040919 | 0.102663 | 0.100245 | 0.081425 |
| 4.880866 | 6.174335 | 5.004787 | 4.851855 |
| 0 | 0.015417 | 0.035126 | 0.017119 |
| 2.734942 | 2.48834 | 2.343841 | 2.320453 |
| 89.43468 | 100.7367 | 92.5074 | 103.9194 |
| 0 | 0.019306 | 0.021993 | 0 |
| 0.457847 | 0.164101 | 0.186942 | 0.182215 |
| 1.477911 | 1.121741 | 1.049687 | 1.201079 |
| 24.00317 | 21.33312 | 22.07147 | 22.28998 |
| 0.555829 | 0.132813 | 0.3026 | 0.294947 |
| 0.022085 | 0.011874 | 0.054106 | 0 |
| 4.727834 | 4.819552 | 4.663076 | 4.618464 |
| 1.364791 | 1.00551 | 0.804924 | 0.935448 |
| 0 | 0 | 0 | 0 |
| 10.23694 | 9.604015 | 9.317182 | 9.178955 |
| 4.422355 | 4.690029 | 3.636103 | 4.195118 |
| 41.74787 | 41.96916 | 42.79983 | 42.31803 |
| 0 | 0 | 0 | 0.462204 |
| 0 | 0 | 0 | 0 |
| 45.16522 | 44.50674 | 45.55532 | 45.8896 |
| 0.209455 | 0 | 0 | 0 |
| 0.176683 | 0.569939 | 0.703375 | 0.21095 |
| 14.16985 | 13.05141 | 12.83763 | 13.72598 |
| 24.58254 | 18.9735 | 17.62706 | 19.37923 |
| 0.044992 | 0.145132 | 0.055111 | 0.053718 |
| 4.382662 | 3.970466 | 3.648159 | 4.509926 |
| 1.721062 | 0 | 2.108171 | 0.684953 |

| | | | |
|---|---|---|---|
| 5.433277 | 4.14012 | 4.297158 | 4.086332 |
| 0 | 0 | 0.015142 | 0.044277 |
| 0.038839 | 0 | 0 | 0 |
| 0.139228 | 0 | 0 | 0 |
| 3.825764 | 2.847932 | 2.487329 | 3.443391 |
| 3.413337 | 3.211437 | 3.553918 | 3.871579 |
| 3.392172 | 2.223806 | 2.190039 | 1.880805 |
| 3.86076 | 0.691885 | 3.94095 | 4.609548 |
| 10.98656 | 10.41636 | 10.58189 | 9.715576 |
| 0.20793 | 0 | 0 | 0.248258 |
| 0 | 0 | 0 | 0.037304 |
| 51.90071 | 54.05548 | 53.67137 | 54.65204 |
| 13.82737 | 17.99807 | 17.57423 | 20.19165 |
| 0.026713 | 0.071809 | 0.065443 | 0.047841 |
| 0.391097 | 0.315397 | 0.119766 | 0.3113 |
| 0.223725 | 0.120281 | 0.182697 | 0.333894 |
| 2.682594 | 4.326717 | 1.642988 | 2.402159 |
| 0 | 0 | 0 | 0 |
| 1.938946 | 2.325427 | 1.735623 | 2.315001 |
| 1.835698 | 1.808104 | 1.613493 | 1.639613 |
| 5.244837 | 3.928673 | 4.643948 | 3.869814 |
| 14.54935 | 8.817689 | 8.748909 | 8.527663 |
| 3.003681 | 2.50511 | 2.358514 | 2.367837 |
| 0.030582 | 0 | 0 | 0 |
| 0.0744 | 0 | 0 | 0 |
| 17.83767 | 18.44339 | 16.33489 | 17.38253 |
| 3.490505 | 3.803011 | 3.518861 | 3.927759 |
| 12.72184 | 11.4124 | 10.61445 | 12.15138 |
| 0.02041 | 0 | 0 | 0 |
| 617.0886 | 549.9816 | 562.6818 | 513.0352 |
| 4.044527 | 4.138117 | 3.601934 | 4.348523 |
| 609.4018 | 507.6534 | 481.2753 | 550.8904 |
| 0.995566 | 1.137395 | 0.933674 | 0.631472 |
| 42.07089 | 45.88511 | 37.80119 | 41.52288 |
| 0.238968 | 0.220244 | 0.234174 | 0.244556 |
| 1.116744 | 0.664265 | 0.698516 | 0.595745 |
| 0.037971 | 0.122487 | 0 | 0.045336 |
| 0.839479 | 1.511424 | 1.315266 | 1.888044 |
| 0.008214 | 0.008832 | 0.010061 | 0 |
| 0.161959 | 0 | 0 | 0.386742 |
| 7.38975 | 4.027664 | 3.39134 | 3.52433 |
| 1.871585 | 2.116977 | 2.500964 | 2.002412 |
| 0.401022 | 0.340422 | 0.361952 | 0.403199 |
| 17.37148 | 14.51081 | 16.41336 | 15.08411 |
| 0 | 0 | 0 | 0 |
| 18.04958 | 16.56352 | 16.73515 | 15.73419 |
| 0.279109 | 0.480183 | 0.27351 | 0.133297 |
| 3.758109 | 2.450353 | 3.158716 | 2.601498 |
| 0.032703 | 0 | 0.040059 | 0.078092 |
| 14.95352 | 16.4402 | 16.83512 | 17.29204 |
| 0 | 0 | 0 | 0 |
| 0.463793 | 0.498696 | 0.18937 | 1.476652 |
| 3.076855 | 2.47028 | 2.881126 | 2.987866 |
| 15.05218 | 12.53541 | 14.64177 | 12.62705 |

| | | | |
|---|---|---|---|
| 4.000019 | 4.568345 | 3.764758 | 4.047301 |
| 0 | 0 | 0 | 0 |
| 0.184797 | 0.13247 | 0.150908 | 0.36773 |
| 0.155715 | 0.11456 | 0.060233 | 0.048925 |
| 1.571989 | 0.763358 | 1.490762 | 1.997962 |
| 29.38736 | 33.11022 | 31.14549 | 32.1885 |
| 4.319382 | 2.764551 | 2.897407 | 2.987854 |
| 12.87137 | 10.5417 | 11.24772 | 9.598857 |
| 3.324246 | 2.985452 | 3.111803 | 2.863978 |
| 50.72322 | 41.85052 | 41.06529 | 40.90545 |
| 2.892081 | 2.576634 | 2.252069 | 1.948475 |
| 0.238557 | 0.25651 | 0.146107 | 0.056965 |
| 0 | 0 | 0 | 0 |
| 3.674469 | 3.106302 | 3.166183 | 3.20714 |
| 3.234208 | 3.376069 | 0.086752 | 0.225488 |
| 170.9428 | 161.2597 | 160.1483 | 157.8966 |
| 3.048775 | 2.28981 | 2.439636 | 2.816946 |
| 5.186933 | 3.967841 | 3.467251 | 3.432652 |
| 0.778271 | 0.684689 | 0.346663 | 0.422371 |
| 9.941812 | 11.25263 | 14.74175 | 11.24527 |
| 100.0192 | 98.07303 | 98.37454 | 95.46118 |
| 0.114248 | 0.218393 | 0.093297 | 0.045469 |
| 30.19839 | 28.90969 | 31.07223 | 28.65816 |
| 0.282169 | 0.113777 | 0.302431 | 0.21056 |
| 0.076924 | 0 | 0 | 0.091843 |
| 34.29489 | 37.68851 | 37.61105 | 34.23473 |
| 0.195906 | 0.304271 | 0.333292 | 0.376841 |
| 0.697605 | 0.692405 | 0.810693 | 0.55527 |
| 3.122983 | 2.910817 | 3.16778 | 2.753626 |
| 10.60728 | 11.97722 | 12.59601 | 11.48788 |
| 9.2564 | 8.229163 | 8.213525 | 7.572693 |
| 21.34873 | 18.67937 | 18.71561 | 18.24233 |
| 22.78806 | 26.78616 | 25.30342 | 27.21681 |
| 17.57259 | 10.3618 | 9.836736 | 21.65756 |
| 4.587592 | 4.60682 | 4.279182 | 4.407061 |
| 21.5842 | 19.739 | 18.53401 | 20.51221 |
| 19.43512 | 18.89652 | 17.51194 | 19.4682 |
| 2.747506 | 1.596261 | 1.737026 | 2.936468 |
| 1.959786 | 1.525957 | 1.804581 | 1.29097 |
| 2.331539 | 2.215868 | 2.422959 | 2.334747 |
| 1.567606 | 1.264185 | 1.44015 | 1.684477 |
| 153.8156 | 154.9412 | 150.8364 | 167.5713 |
| 11.13724 | 9.823565 | 10.79125 | 10.12879 |
| 0 | 0 | 0 | 0 |
| 1.39025 | 1.681735 | 2.128688 | 1.4524 |
| 4.807884 | 3.898017 | 3.527278 | 3.898536 |
| 67.73576 | 51.39778 | 49.67429 | 50.28965 |
| 5.150774 | 3.261506 | 3.645379 | 2.596564 |
| 16.88798 | 16.18019 | 16.24864 | 20.78132 |
| 13.01335 | 14.81849 | 16.25395 | 15.99574 |
| 0.048937 | 0.05262 | 0 | 0 |
| 0 | 0 | 0 | 0 |
| 0.197168 | 0 | 0 | 0 |
| 60.88833 | 66.64761 | 67.37666 | 64.52925 |

| | | | |
|---|---|---|---|
| 1.605035 | 1.725825 | 3.276745 | 1.916329 |
| 244.5437 | 282.6472 | 269.815 | 275.4835 |
| 0.242321 | 0.521114 | 0.148412 | 0.433978 |
| 0.285582 | 0.307074 | 0.373138 | 0.431896 |
| 51.7417 | 50.18242 | 49.15561 | 50.26732 |
| 0.655361 | 0.990958 | 0.903113 | 0.904727 |
| 1.367113 | 0.881999 | 0.955148 | 1.088175 |
| 0.101527 | 0.174668 | 0.09949 | 0.048487 |
| 1.402347 | 1.507883 | 1.327367 | 1.141588 |
| 12.98964 | 7.8094 | 6.922009 | 8.716714 |
| 16019.52 | 17533.98 | 17997.38 | 17053.19 |
| 0 | 0 | 0 | 0 |
| 0 | 0.013977 | 0.031846 | 0.01552 |
| 7.875162 | 7.426369 | 7.456171 | 7.205348 |
| 0.096781 | 0.052032 | 0.118549 | 0.231102 |
| 0.077525 | 0 | 0 | 0.092561 |
| 33.39638 | 38.71181 | 39.13849 | 41.76746 |
| 0.881779 | 0.270897 | 0.154302 | 0.300799 |
| 0 | 0 | 0 | 0 |
| 41.14334 | 42.10348 | 42.65208 | 42.03257 |
| 466.5507 | 420.3012 | 423.023 | 400.335 |
| 7.171615 | 5.893754 | 6.226927 | 6.113978 |
| 9.258676 | 6.399936 | 6.480673 | 11.05438 |
| 14.67773 | 13.93485 | 14.55296 | 13.69197 |
| 0 | 0.167867 | 0 | 0.186397 |
| 0.44501 | 0 | 0 | 0 |
| 0.1984 | 0.177776 | 0.081008 | 0.07896 |
| 6.843369 | 7.667556 | 6.903326 | 5.904823 |
| 0.081534 | 0 | 0 | 0 |
| 19.15663 | 18.62328 | 18.48911 | 17.57004 |
| 22.85866 | 19.91238 | 21.59665 | 20.57945 |
| 2.756794 | 2.0486 | 1.962471 | 1.861145 |
| 0.330667 | 0 | 0 | 0 |
| 0.053202 | 0.057206 | 0 | 0 |
| 15.97078 | 13.57248 | 14.59059 | 14.18624 |
| 2.606998 | 2.390958 | 3.099452 | 2.197143 |
| 26.86416 | 27.37906 | 29.29086 | 25.23946 |
| 0.194351 | 0.104489 | 0.039678 | 0.154697 |
| 0 | 0.143309 | 0.010884 | 0.010609 |
| 11.84877 | 12.92151 | 12.92841 | 13.11658 |
| 1.523864 | 0.849616 | 0.967876 | 1.078172 |
| 2.508384 | 2.325136 | 2.383899 | 2.271978 |
| 36.41027 | 29.73819 | 29.74712 | 30.58084 |
| 80.0222 | 57.66807 | 63.60947 | 65.52444 |
| 1.993972 | 2.019288 | 1.927327 | 2.017101 |
| 0 | 0 | 0.021618 | 0 |
| 0.061626 | 0.022088 | 0.050324 | 0.073578 |
| 1.621032 | 1.424734 | 1.640313 | 1.800789 |
| 0.765821 | 0.403654 | 0.404659 | 0.322712 |
| 0.082883 | 0.17824 | 0.050762 | 0 |
| 1.005973 | 0.927154 | 1.095325 | 1.029497 |
| 6.772458 | 5.710218 | 6.084767 | 5.485631 |
| 0.727048 | 0.805818 | 0.767268 | 0.721155 |
| 0.180912 | 0.233432 | 0.132962 | 0.259199 |

| | | | |
|---|---|---|---|
| 2.540204 | 2.96968 | 2.777431 | 2.768259 |
| 8.778643 | 8.988832 | 9.167023 | 10.6404 |
| 5.742009 | 4.906954 | 4.668542 | 4.191233 |
| 0 | 0 | 0 | 0 |
| 166.4582 | 197.2515 | 197.7267 | 193.1562 |
| 11.83372 | 11.10284 | 11.17681 | 10.92469 |
| 44.9843 | 37.19559 | 37.60972 | 37.57509 |
| 49.87392 | 41.21855 | 42.16194 | 40.07732 |
| 40.09419 | 41.16179 | 39.48732 | 41.21503 |
| 2.673252 | 2.118003 | 1.772679 | 1.631859 |
| 0.066441 | 0.023814 | 0 | 0 |
| 66.80977 | 47.89179 | 48.10971 | 49.50545 |
| 1.685335 | 1.330453 | 0.914611 | 1.044308 |
| 8.308665 | 6.099409 | 7.300082 | 6.637648 |
| 0.046252 | 0 | 0 | 0 |
| 0 | 0 | 0 | 0 |
| 2.881968 | 3.053285 | 2.881261 | 3.263814 |
| 10.43018 | 14.62842 | 7.406484 | 9.023982 |
| 2.367649 | 1.555785 | 1.288974 | 1.256378 |
| 0.503577 | 0.481311 | 0.137077 | 0.334025 |
| 48.40725 | 52.33382 | 52.59918 | 51.81292 |
| 0 | 0 | 0.167604 | 0 |
| 0 | 0 | 0 | 0 |
| 16.38402 | 11.37766 | 7.944051 | 11.2072 |
| 3.64432 | 4.057468 | 3.842445 | 3.921524 |
| 9.623583 | 8.037789 | 8.97324 | 7.831742 |
| 25.78153 | 26.42748 | 25.95698 | 25.8019 |
| 0.060147 | 0.032337 | 0.073675 | 0.047875 |
| 0.015163 | 0 | 0.006191 | 0.018104 |
| 0 | 0 | 0 | 0 |
| 35.70477 | 35.42417 | 31.52952 | 30.52407 |
| 0.093802 | 0.075646 | 0.043087 | 0.041998 |
| 2.205581 | 2.687775 | 2.326438 | 2.677239 |
| 0.546003 | 1.19865 | 0.445875 | 0.6519 |
| 8.028882 | 6.994532 | 6.240763 | 6.232302 |
| 1.081519 | 0.59717 | 0.716096 | 0.593289 |
| 4.28815 | 3.490874 | 4.109336 | 3.795456 |
| 12.56939 | 11.55174 | 11.96981 | 11.62072 |
| 3.794476 | 2.910758 | 3.627666 | 2.624321 |
| 583.0536 | 653.0547 | 507.0796 | 522.1018 |
| 9.535279 | 9.928417 | 10.38633 | 10.62806 |
| 0.205242 | 0.073562 | 0.083802 | 0.081683 |
| 4.752637 | 2.796205 | 3.514941 | 2.355412 |
| 3.118955 | 2.485667 | 2.292291 | 2.219719 |
| 1.804538 | 1.660869 | 1.528194 | 1.480682 |
| 7.35224 | 8.200596 | 8.274392 | 7.573721 |
| 0.771517 | 1.605056 | 1.7052 | 1.241552 |
| 0 | 0 | 0 | 0 |
| 183.1157 | 178.7164 | 175.4417 | 175.7842 |
| 17.3003 | 18.4293 | 18.80908 | 18.64772 |
| 6.435817 | 6.750783 | 6.009017 | 6.367537 |
| 9.196157 | 7.373634 | 7.689218 | 6.99092 |
| 0.762455 | 0.353654 | 0.366255 | 0.464091 |
| 41.60254 | 39.56057 | 46.67424 | 40.07643 |

| | | | |
|---|---|---|---|
| 2.504601 | 2.477643 | 2.384233 | 2.238501 |
| 2.832451 | 1.942554 | 2.11286 | 2.135303 |
| 3.395665 | 2.551035 | 2.804288 | 2.763913 |
| 0 | 0 | 0 | 0 |
| 0.100775 | 0.108359 | 0.061721 | 0 |
| 24.05516 | 14.12143 | 14.19252 | 14.67125 |
| 0.617091 | 0.286932 | 0.510736 | 0.258867 |
| 0 | 0.550532 | 0 | 0 |
| 1.250561 | 0.550094 | 0.789131 | 0.769175 |
| 1.422436 | 1.070639 | 1.415681 | 1.316194 |
| 9.543216 | 6.733378 | 7.793146 | 7.381087 |
| 0.181939 | 0.223579 | 0.095512 | 0.217226 |
| 0.442093 | 0.531289 | 0.796369 | 0.434689 |
| 13.13217 | 7.02604 | 7.82874 | 6.985376 |
| 9.47029 | 8.440388 | 8.413321 | 8.384844 |
| 8.886325 | 7.060257 | 7.053437 | 6.527389 |
| 0 | 0.853325 | 0.48605 | 0 |
| 0.050954 | 0.191758 | 0.249657 | 0.273761 |
| 10.73894 | 11.19853 | 11.06954 | 10.69284 |
| 5.392661 | 4.49075 | 4.216342 | 5.096049 |
| 0 | 0 | 0 | 0 |
| 1.072939 | 0.912391 | 0.790279 | 0.820531 |
| 1.792773 | 1.053805 | 1.141926 | 1.541144 |
| 4.701404 | 4.839446 | 3.932883 | 4.175697 |
| 0 | 0 | 0 | 0.066923 |
| 0.116706 | 0 | 0 | 0 |
| 0 | 0 | 0 | 0 |
| 0.032166 | 0 | 0 | 0.025603 |
| 1.246688 | 1.100419 | 0.980078 | 1.044158 |
| 7.537989 | 7.6511 | 7.143997 | 7.467645 |
| 0.059894 | 0.032201 | 0.146732 | 0.035755 |
| 0 | 0.01595 | 0 | 0 |
| 0.150548 | 0.046251 | 0.026344 | 0.102712 |
| 1.991546 | 1.362724 | 2.217721 | 1.513147 |
| 1.244864 | 1.115457 | 1.016576 | 1.114727 |
| 19.91174 | 18.38379 | 18.63471 | 18.24678 |
| 0.520394 | 0.450754 | 0.495789 | 0.621323 |
| 43.82774 | 33.71521 | 33.26799 | 32.91379 |
| 5.639341 | 5.18352 | 5.180825 | 4.615418 |
| 15.92436 | 15.85631 | 15.63954 | 16.10656 |
| 32.254 | 31.71349 | 32.95934 | 31.37261 |
| 10.92911 | 14.28951 | 16.21565 | 15.74432 |
| 10.32558 | 9.228637 | 8.861694 | 8.990952 |
| 0 | 0.087471 | 0.099646 | 0.064751 |
| 3.873561 | 4.319672 | 4.434024 | 4.291601 |
| 7.770048 | 6.989029 | 6.97262 | 7.292442 |
| 2.683398 | 2.394221 | 1.608512 | 2.863004 |
| 50.07783 | 46.84579 | 47.0973 | 45.51954 |
| 72.50193 | 72.36785 | 69.04376 | 71.70731 |
| 153.1312 | 167.4807 | 171.6671 | 165.4527 |
| 91.08072 | 78.71403 | 78.94442 | 80.17114 |
| 31.60577 | 33.44914 | 35.71709 | 31.99112 |
| 88.91011 | 81.32592 | 81.41712 | 85.12224 |
| 249.6024 | 239.6807 | 233.1009 | 235.928 |

| | | | |
|---|---|---|---|
| 0.150366 | 0.080841 | 0.122792 | 0 |
| 30.61982 | 25.55203 | 24.21646 | 23.55108 |
| 15.96232 | 15.46068 | 15.08565 | 14.13191 |
| 0.605154 | 0.342471 | 0.156056 | 0.570412 |
| 0.061759 | 0 | 0 | 0 |
| 0.095667 | 0.056109 | 0.021306 | 0.031151 |
| 0.574795 | 0.370832 | 0.309796 | 0.411765 |
| 0 | 0 | 0 | 0 |
| 0.34567 | 0.278763 | 0 | 0.103178 |
| 5.295534 | 3.670551 | 4.66394 | 3.814457 |
| 20.87476 | 18.45968 | 18.26639 | 18.30579 |
| 18.25638 | 18.02314 | 17.9163 | 17.46323 |
| 0 | 0.092501 | 0.105377 | 0 |
| 138.0916 | 137.2401 | 135.1333 | 139.8741 |
| 13.82053 | 12.96434 | 12.34414 | 11.51632 |
| 0 | 0.018022 | 0.04106 | 0.030016 |
| 0 | 0 | 0 | 0.541439 |
| 18.23464 | 16.13283 | 16.12748 | 16.90102 |
| 78.20153 | 84.70961 | 82.30928 | 82.358 |
| 0.11372 | 0.192151 | 0.059699 | 0.058189 |
| 3.639134 | 3.026057 | 2.516104 | 3.051111 |
| 3.424442 | 3.378681 | 3.288139 | 3.294847 |
| 23.45374 | 19.9617 | 20.44016 | 20.60006 |
| 0.47616 | 0.255997 | 1.166521 | 0.189504 |
| 0 | 0 | 0 | 0 |
| 5.525411 | 6.11598 | 5.042981 | 6.661732 |
| 0 | 0 | 0 | 0 |
| 0 | 0 | 0 | 0 |
| 213.201 | 231.5496 | 232.2331 | 233.2513 |
| 48.70091 | 65.6033 | 61.96602 | 66.34035 |
| 3.675948 | 2.81923 | 2.549949 | 2.516927 |
| 0 | 0 | 0 | 0 |
| 32.95726 | 28.2339 | 28.3424 | 27.6973 |
| 7.031583 | 5.773297 | 6.20241 | 6.319322 |
| 0 | 0 | 0 | 0 |
| 0 | 0 | 0 | 0.011854 |
| 0 | 0.092585 | 0 | 0 |
| 27.95785 | 25.41574 | 24.89207 | 25.74389 |
| 0.030329 | 0.065222 | 0.055726 | 0.054316 |
| 85.08808 | 81.30671 | 78.70882 | 80.19518 |
| 3.006783 | 3.251646 | 3.630165 | 3.228886 |
| 0 | 0 | 0.091277 | 0 |
| 0 | 0 | 0 | 0 |
| 14.05351 | 14.10624 | 14.30963 | 13.47354 |
| 0.689255 | 0.247042 | 0.844286 | 0.34289 |
| 0 | 0 | 0 | 0 |
| 1.5692 | 1.632511 | 1.372965 | 1.532899 |
| 56.83693 | 62.41461 | 57.77057 | 65.33363 |
| 0.116137 | 0.124877 | 0 | 0 |
| 12.6503 | 12.54735 | 11.37882 | 11.56462 |
| 229.241 | 246.2101 | 247.7407 | 249.9563 |
| 6.64355 | 8.592565 | 6.805672 | 7.254583 |
| 0 | 0 | 0 | 0 |
| 1.904642 | 1.912144 | 2.594914 | 1.798351 |

| | | | |
|---|---|---|---|
| 2.735609 | 2.012593 | 2.292731 | 2.283867 |
| 1.735437 | 1.370373 | 1.361827 | 1.715893 |
| 5.736873 | 5.995892 | 5.228264 | 6.082381 |
| 183.9149 | 215.8283 | 214.7218 | 221.1807 |
| 9.147423 | 6.574943 | 6.54124 | 6.493894 |
| 0.030406 | 0 | 0.037245 | 0 |
| 4.987246 | 4.964422 | 4.719946 | 4.614402 |
| 0.129921 | 0.209548 | 0.145881 | 0.180972 |
| 17.86508 | 13.209 | 12.20675 | 13.88828 |
| 3.437982 | 2.526088 | 3.158451 | 2.326037 |
| 16.24569 | 17.73506 | 16.90578 | 16.52214 |
| 0.644022 | 0.513782 | 0.661639 | 0.669712 |
| 0.167916 | 0.232139 | 0.293834 | 0.085921 |
| 0.794805 | 0.155385 | 0.265521 | 0.258806 |
| 0.045435 | 0.170991 | 0.027827 | 0.027124 |
| 7.655071 | 7.399971 | 6.707168 | 7.588691 |
| 3.249868 | 3.376653 | 2.236429 | 3.444199 |
| 321.5629 | 287.0073 | 290.7351 | 272.1066 |
| 0 | 0.095344 | 0.054307 | 0.052934 |
| 0 | 0.011563 | 0.013172 | 0.012839 |
| 0 | 0 | 0 | 0 |
| 0.610462 | 0 | 0 | 0 |
| 6.316414 | 6.791769 | 5.951639 | 3.480679 |
| 0.530049 | 0.221643 | 0.68534 | 0.668009 |
| 1.741872 | 1.652612 | 1.739203 | 1.992322 |
| 0 | 0 | 0 | 0 |
| 34.47561 | 38.27346 | 38.47132 | 36.3347 |
| 0.005805 | 0.012485 | 0.014222 | 0.013863 |
| 0.066133 | 0 | 0.081008 | 0 |
| 0 | 0 | 0 | 0 |
| 2.48541 | 1.809827 | 1.483682 | 2.234978 |
| 0.059446 | 0.127839 | 0.21845 | 0.14195 |
| 0 | 0 | 0 | 0.093969 |
| 0.89002 | 0 | 0 | 0 |
| 0 | 0.409596 | 0 | 0 |
| 0.89996 | 0.263915 | 0.200433 | 0.488411 |
| 31.63388 | 25.26057 | 24.32399 | 24.42624 |
| 1.526236 | 1.125711 | 1.00429 | 1.084312 |
| 7.602027 | 5.335417 | 6.896268 | 6.076269 |
| 0 | 0 | 0 | 0 |
| 7.749966 | 8.127447 | 6.367815 | 7.806077 |
| 6.399053 | 5.752394 | 6.227239 | 5.593357 |
| 0 | 0 | 0 | 0 |
| 0 | 0 | 0 | 0 |
| 8.587321 | 7.3373 | 7.671252 | 6.783368 |
| 0.269889 | 0.373114 | 0.519503 | 0.368266 |
| 4.464004 | 4.332264 | 4.262289 | 3.771851 |
| 3.34406 | 2.64591 | 2.730814 | 2.084205 |
| 17.00044 | 15.51661 | 15.58792 | 15.42975 |
| 1.614686 | 1.5515 | 1.09414 | 1.29207 |
| 12.35606 | 7.412584 | 1.292033 | 1.034474 |
| 0 | 0.12832 | 0 | 0.142484 |
| 38.92302 | 40.89416 | 40.17071 | 39.41433 |
| 0 | 0 | 0.047266 | 0.138212 |

| | | | |
|---|---|---|---|
| 77.80254 | 87.99081 | 87.51937 | 85.09953 |
| 15.45289 | 15.0483 | 15.02236 | 14.2726 |
| 66.66246 | 75.77524 | 72.83758 | 73.31516 |
| 0 | 0.015293 | 0 | 0 |
| 0 | 0 | 0 | 0 |
| 0.145234 | 0.044618 | 0.050829 | 0.14863 |
| 0 | 0 | 0 | 0 |
| 24.17264 | 12.49458 | 12.56434 | 12.48925 |
| 56.90777 | 52.99717 | 55.09666 | 52.32143 |
| 7.518323 | 8.054021 | 7.734505 | 7.438616 |
| 671.5083 | 710.8852 | 724.1618 | 711.8881 |
| 21.80365 | 19.31808 | 18.05473 | 18.64879 |
| 8.240743 | 6.977972 | 7.034457 | 6.395363 |
| 0 | 0.037047 | 0 | 0 |
| 2.682961 | 2.800847 | 3.286425 | 3.514318 |
| 4.789049 | 2.744411 | 2.907899 | 3.211185 |
| 0.046274 | 0.099513 | 0.113365 | 0 |
| 0 | 0 | 0 | 0 |
| 21.93898 | 16.22115 | 14.19994 | 16.12552 |
| 2.193445 | 2.181629 | 2.261394 | 2.16056 |
| 41.8374 | 41.50638 | 37.94025 | 36.9808 |
| 0.222765 | 0.059882 | 0.034109 | 0.066493 |
| 0.235141 | 0.216717 | 0.164589 | 0.100266 |
| 0.020613 | 0.044329 | 0.075748 | 0.098443 |
| 2.983462 | 1.539834 | 1.607987 | 2.137259 |
| 18.96857 | 22.40501 | 22.12258 | 22.89534 |
| 0.435392 | 0.510718 | 0.193935 | 0.378062 |
| 4.894983 | 3.915255 | 3.888404 | 4.363361 |
| 3.35778 | 2.944337 | 2.382824 | 3.195377 |
| 8.584094 | 9.703447 | 8.17823 | 9.197788 |
| 0 | 0 | 0 | 0.088969 |
| 0 | 0 | 0 | 0.186397 |
| 3.326951 | 2.719416 | 2.600055 | 2.66012 |
| 5.350117 | 7.09506 | 8.956436 | 8.304091 |
| 61.69679 | 34.6206 | 32.96811 | 50.86819 |
| 6.175881 | 2.523451 | 1.739947 | 1.62221 |
| 0.026557 | 0 | 0.09759 | 0 |
| 5.780419 | 5.38942 | 5.969898 | 6.405333 |
| 0 | 0 | 0 | 0.062542 |
| 1.664897 | 1.432153 | 1.668578 | 1.084255 |
| 19.91456 | 20.3568 | 19.86715 | 20.01256 |
| 0.316176 | 0.067994 | 0.232375 | 0 |
| 31.0921 | 25.85588 | 23.58842 | 25.29709 |
| 5.788329 | 4.171225 | 4.107258 | 4.514774 |
| 0.146685 | 0.072795 | 0.027643 | 0.080831 |
| 7.047175 | 6.676413 | 6.392536 | 6.094437 |
| 12.1861 | 9.867831 | 10.43511 | 10.66519 |
| 26.94081 | 23.52341 | 22.22422 | 23.16671 |
| 1.476467 | 1.69342 | 1.35642 | 1.498401 |
| 0.339604 | 0.4036 | 0.350307 | 0.42681 |
| 5.187923 | 5.089613 | 4.665317 | 5.164878 |
| 10.28694 | 8.63101 | 8.40048 | 8.467183 |
| 0 | 0 | 0 | 0 |
| 0.419085 | 0.536457 | 0.366677 | 0.357404 |

| | | | |
|---|---|---|---|
| 145.1642 | 115.6566 | 111.3953 | 118.9888 |
| 9.434038 | 7.670543 | 7.820077 | 8.139218 |
| 12.14695 | 12.1323 | 11.44037 | 9.668552 |
| 0 | 0 | 0 | 0 |
| 1.988848 | 1.750837 | 1.695359 | 1.694146 |
| 28.32887 | 28.41859 | 27.7868 | 29.03238 |
| 6.389911 | 4.610666 | 4.325535 | 5.119609 |
| 0 | 0 | 0.084408 | 0 |
| 8.088205 | 8.276081 | 7.403947 | 7.216713 |
| 0.161083 | 0.069282 | 0.236777 | 0.115395 |
| 0 | 0.026042 | 0.014834 | 0 |
| 0.018821 | 0 | 0 | 0 |
| 24.48528 | 17.98144 | 18.02747 | 17.54049 |
| 1.61986 | 1.545115 | 1.312136 | 1.82485 |
| 1.1626 | 1.630557 | 1.764642 | 1.146678 |
| 0.798592 | 0.322009 | 0.489107 | 0.119185 |
| 0 | 0 | 0 | 0 |
| 6.406062 | 6.978996 | 6.55349 | 7.413167 |
| 15.31995 | 12.79987 | 13.77847 | 12.81209 |
| 9.415436 | 9.807967 | 10.32107 | 9.592159 |
| 3.533009 | 2.943286 | 2.885113 | 2.850155 |
| 1.36417 | 1.030276 | 0.537109 | 0.833764 |
| 0.059004 | 0 | 0 | 0.035224 |
| 133.1751 | 140.2208 | 143.1023 | 139.5224 |
| 0.236015 | 0.697886 | 0.795027 | 0.634027 |
| 0.111972 | 0.0301 | 0.137157 | 0.300799 |
| 0.587272 | 0.707245 | 0.517942 | 0.701173 |
| 0.04283 | 0.028783 | 0.026232 | 0.025568 |
| 0.417278 | 0.324047 | 0.31236 | 0.207587 |
| 0 | 0.083251 | 0 | 0 |
| 0 | 0.015529 | 0 | 0 |
| 10.45247 | 9.217088 | 8.877532 | 9.390508 |
| 0 | 0 | 0 | 0 |
| 22.37294 | 22.55987 | 21.74483 | 21.45195 |
| 0 | 0.043537 | 0 | 0 |
| 1.687657 | 2.009094 | 0.959796 | 1.799085 |
| 21.69488 | 17.11699 | 16.56598 | 20.74448 |
| 24.75 | 23.03778 | 23.71052 | 22.9786 |
| 1.476467 | 0 | 0 | 0.440706 |
| 93.42552 | 83.88946 | 84.34359 | 84.49088 |
| 0.056551 | 0 | 0 | 0 |
| 5.612893 | 5.311067 | 5.477378 | 5.517571 |
| 2.242785 | 1.484043 | 2.535916 | 1.579197 |
| 0 | 0.084908 | 0 | 0 |
| 66.91793 | 63.71322 | 63.71535 | 63.29026 |
| 5.015321 | 4.885205 | 4.047409 | 3.240582 |
| 0 | 0 | 0 | 0 |
| 8.502865 | 6.896257 | 8.719151 | 6.236216 |
| 0.093 | 0.199998 | 0.227836 | 0.222075 |
| 0.437681 | 0.215701 | 0.223386 | 0.457247 |
| 0.047521 | 0 | 0 | 0 |
| 13.64585 | 17.77912 | 17.36043 | 15.6026 |
| 187.5465 | 205.8019 | 201.1391 | 205.8762 |
| 0 | 0 | 0 | 0 |

| | | | |
|---|---|---|---|
| 0.39528 | 0.196167 | 0.223471 | 0.10891 |
| 1.852049 | 1.672192 | 1.645183 | 1.401021 |
| 56.16252 | 54.51797 | 52.75933 | 53.53072 |
| 4.754176 | 4.339842 | 4.276638 | 3.961542 |
| 1.670738 | 0.898237 | 1.023264 | 0 |
| 11.94194 | 10.74327 | 10.40683 | 11.02347 |
| 0.697082 | 0.288285 | 0.394095 | 0.576193 |
| 0 | 0 | 0 | 0 |
| 2.065881 | 2.275533 | 2.098504 | 1.744637 |
| 47.61605 | 57.80365 | 55.73989 | 57.09935 |
| 0.369526 | 0.596003 | 0.840619 | 0.850875 |
| 0.680229 | 0.365711 | 1.249844 | 1.624317 |
| 1234.339 | 1081.122 | 1107.525 | 1052.932 |
| 0.55568 | 0.278146 | 0.316862 | 0.274532 |
| 57.71886 | 61.47833 | 60.81589 | 58.96558 |
| 4.565297 | 3.747102 | 3.951782 | 4.015369 |
| 0.170667 | 0 | 0.069685 | 0.203767 |
| 7.291248 | 5.597961 | 5.590049 | 6.1376 |
| 0.042782 | 0 | 0 | 0.051079 |
| 28.04583 | 25.3004 | 26.54859 | 25.13857 |
| 0 | 0.089198 | 0 | 0 |
| 0 | 0 | 0 | 0 |
| 1.527432 | 0.785487 | 1.301558 | 1.110063 |
| 2.215796 | 2.078395 | 2.771932 | 1.913799 |
| 1.592256 | 0.244584 | 0.092876 | 0.452636 |
| 25.5469 | 29.25186 | 26.79416 | 29.9972 |
| 13.03855 | 14.06933 | 11.28709 | 12.23935 |
| 0 | 0 | 0 | 0 |
| 16.91229 | 12.88025 | 15.01644 | 13.83347 |
| 6.844233 | 3.84736 | 3.865928 | 4.337771 |
| 2.441129 | 1.418018 | 1.374804 | 1.909553 |
| 0.389991 | 0.319498 | 0.318473 | 0.266074 |
| 0.167958 | 0.090299 | 0.137157 | 0.167111 |
| 0 | 0 | 0 | 0 |
| 12.46326 | 12.11263 | 13.19187 | 13.39244 |
| 4.181848 | 2.503767 | 2.037337 | 2.780143 |
| 0 | 0 | 0 | 0 |
| 0 | 0 | 0.610744 | 0 |
| 13.27836 | 17.18775 | 17.97437 | 16.56053 |
| 9.923344 | 9.138463 | 8.645981 | 9.936997 |
| 60.79623 | 54.75376 | 56.94223 | 57.90019 |
| 0 | 0 | 0.767448 | 0 |
| 20.6783 | 18.63167 | 17.87848 | 19.21999 |
| 23.15056 | 19.39796 | 19.47456 | 19.16048 |
| 0 | 0.12832 | 0.438542 | 0.142484 |
| 2.423805 | 1.807535 | 1.91547 | 2.007058 |
| 10.3654 | 9.403987 | 8.371972 | 9.011091 |
| 9.453346 | 8.122431 | 6.899644 | 6.829429 |
| 0 | 0 | 0.06506 | 0 |
| 4.4294 | 1.587581 | 0.90428 | 1.322118 |
| 4.687699 | 4.972367 | 4.526413 | 4.613636 |
| 20.04127 | 21.51076 | 21.45834 | 20.91569 |
| 0.014265 | 0.015338 | 0.034947 | 0 |
| 3.596452 | 3.33737 | 3.500167 | 2.999902 |

| | | | |
|---|---|---|---|
| 0 | 0 | 0 | 0 |
| 0 | 0.143015 | 0 | 0 |
| 26.62502 | 22.12311 | 22.50545 | 22.76474 |
| 0 | 0.106003 | 0 | 0 |
| 0.395482 | 0.170098 | 0.096887 | 0.094437 |
| 4.241779 | 4.493929 | 4.304412 | 4.369341 |
| 19.1854 | 20.59675 | 18.94857 | 22.68993 |
| 0.641294 | 0.965378 | 0.510599 | 0.650821 |
| 5.884523 | 5.921029 | 3.041949 | 4.125249 |
| 2.95135 | 3.203755 | 2.993956 | 3.052802 |
| 1186.547 | 1957.459 | 1925.914 | 2053.797 |
| 1282.72 | 1324.49 | 1302.861 | 1287.545 |
| 1.631915 | 0.955454 | 1.130309 | 0.958909 |
| 7.310746 | 5.378532 | 6.834165 | 9.647457 |
| 0.341779 | 0.083523 | 0.057089 | 0.222582 |
| 0.660145 | 0.630956 | 0.509136 | 0.613028 |
| 0 | 0 | 0 | 0 |
| 0.142991 | 0 | 0 | 0 |
| 35.28494 | 32.59434 | 33.91175 | 32.34811 |
| 13.90766 | 13.86247 | 13.63755 | 12.18856 |
| 2.355564 | 29.67037 | 31.39577 | 16.40579 |
| 0 | 0 | 0 | 0 |
| 0 | 0 | 0 | 0 |
| 109.6933 | 102.9679 | 106.067 | 93.2779 |
| 7.891244 | 11.30432 | 10.5905 | 10.55173 |
| 0.195721 | 0.21045 | 0.239743 | 0.267063 |
| 20.84779 | 22.06191 | 22.37457 | 23.3152 |
| 2.052205 | 2.259187 | 1.795569 | 1.341791 |
| 1.09917 | 1.370993 | 1.561824 | 0.839905 |
| 0 | 0 | 0 | 0 |
| 0.013882 | 0 | 0 | 0 |
| 1.625847 | 1.349878 | 1.436934 | 1.392405 |
| 2.988455 | 2.213646 | 2.074358 | 2.616577 |
| 0.094177 | 0.141771 | 0 | 0 |
| 0.443825 | 0.3636 | 0.517764 | 0.302802 |
| 65.12891 | 56.74695 | 54.44152 | 56.85109 |
| 5.38612 | 5.255729 | 4.535185 | 5.251264 |
| 126.7096 | 131.399 | 133.7492 | 138.9163 |
| 35.83036 | 26.21047 | 30.72844 | 26.95623 |
| 0.018413 | 0.079195 | 0.067664 | 0.109921 |
| 26.81266 | 22.25309 | 21.2905 | 23.42252 |
| 0.158193 | 0 | 0 | 0.188874 |
| 19.93761 | 19.81024 | 22.04284 | 19.09815 |
| 5.887186 | 3.361914 | 3.381492 | 3.805854 |
| 7.514563 | 7.511716 | 7.542314 | 7.453923 |
| 0.39117 | 0.152948 | 0.392035 | 0.127374 |
| 12.91523 | 12.51373 | 12.45912 | 12.14405 |
| 0.461834 | 0.628339 | 0.588802 | 0.517646 |
| 0.235025 | 0.20217 | 0.201521 | 0.196425 |
| 1.725004 | 1.567005 | 1.42081 | 1.34937 |
| 53.68385 | 46.61743 | 50.32608 | 49.32993 |
| 0 | 0.087074 | 0 | 0 |
| 1.252269 | 0.577076 | 1.022623 | 0.747572 |
| 4.782184 | 4.372872 | 4.741468 | 4.32906 |

| | | | |
|---|---|---|---|
| 0.052556 | 0 | 0 | 0 |
| 7.619887 | 7.093798 | 6.808411 | 6.340856 |
| 6.469571 | 5.269512 | 4.73503 | 5.561519 |
| 0 | 0 | 0 | 0 |
| 0 | 0.043611 | 0.024841 | 0.024213 |
| 2.806424 | 2.792244 | 2.924148 | 2.891912 |
| 12.87951 | 10.88344 | 10.92491 | 10.05315 |
| 13.94664 | 16.16407 | 15.39034 | 14.76537 |
| 685.9923 | 704.9072 | 687.6457 | 723.6106 |
| 43.49189 | 42.60698 | 41.8431 | 42.55058 |
| 15.3247 | 8.736959 | 8.302825 | 8.796587 |
| 10.79627 | 9.843264 | 8.595999 | 9.157402 |
| 36.56048 | 33.78281 | 35.08382 | 32.90733 |
| 2.080165 | 1.714269 | 1.878486 | 1.867239 |
| 4.442924 | 4.564683 | 4.829637 | 4.943573 |
| 3.716692 | 1.595054 | 1.916913 | 1.479179 |
| 0.36551 | 0.285831 | 0.20351 | 0.238037 |
| 7.308509 | 5.858174 | 5.751221 | 5.182704 |
| 0.400135 | 0 | 0 | 0 |
| 8.682239 | 7.326173 | 7.86901 | 8.878624 |
| 3.620257 | 3.491549 | 3.639032 | 3.794472 |
| 2.174249 | 1.736708 | 1.959421 | 1.724446 |
| 14.19145 | 11.64537 | 6.40443 | 10.47844 |
| 0.410483 | 0.264825 | 0.100562 | 0.098019 |
| 17.806 | 16.56454 | 14.63403 | 15.35594 |
| 3.611093 | 2.979864 | 2.571696 | 2.807461 |
| 0 | 0.045229 | 0.051525 | 0 |
| 0.091444 | 0.098325 | 0.128013 | 0.171567 |
| 1.587202 | 6.826598 | 2.916303 | 1.895036 |
| 0 | 0 | 0 | 0.032137 |
| 0 | 0.423136 | 0 | 0 |
| 0 | 1.163625 | 0 | 0.43069 |
| 1.248945 | 0.167867 | 0.764932 | 0.186397 |
| 0 | 0 | 0 | 0.568511 |
| 3.473908 | 3.566832 | 3.103471 | 2.681949 |
| 1.380175 | 1.484043 | 1.625587 | 1.394341 |
| 0 | 0 | 0 | 0 |
| 0.795495 | 0.448046 | 0.881619 | 0.94978 |
| 13.35946 | 12.98108 | 13.54936 | 13.60914 |
| 28.19081 | 34.71714 | 35.01722 | 33.89879 |
| 535.6933 | 382.5388 | 382.4711 | 366.6315 |
| 0 | 0 | 0 | 0 |
| 0.347712 | 0.307901 | 0.250541 | 0.390729 |
| 0 | 0 | 0 | 0 |
| 11.94801 | 11.54354 | 12.3359 | 11.27683 |
| 6.570877 | 7.506968 | 6.875041 | 7.245994 |
| 7.181759 | 5.959169 | 6.668131 | 6.068814 |
| 1.366496 | 1.620812 | 1.622086 | 1.530606 |
| 7.66514 | 5.372947 | 2.614719 | 3.634647 |
| 0.054543 | 0 | 0 | 0 |
| 23.53991 | 19.05566 | 17.57007 | 21.38318 |
| 19.39913 | 9.481386 | 12.24127 | 13.33544 |
| 0.851669 | 0.582758 | 0.569035 | 0.462204 |
| 0.109128 | 0.136897 | 0.193083 | 0.180962 |

| | | | |
|---|---|---|---|
| 54.5628 | 38.52962 | 35.92196 | 34.43612 |
| 14.38041 | 8.997524 | 9.333527 | 9.858379 |
| 0.387122 | 0 | 0.948391 | 0.462204 |
| 10.1136 | 7.92143 | 8.112196 | 7.631224 |
| 35.96614 | 28.03781 | 32.17989 | 29.63911 |
| 26.61335 | 26.7209 | 26.85188 | 26.05428 |
| 544.8636 | 621.4991 | 636.2302 | 615.5 |
| 0.608169 | 0.384669 | 0.503944 | 0.4912 |
| 0.040697 | 0.04376 | 0 | 0.032394 |
| 0.244426 | 0.171844 | 0.184248 | 0.179589 |
| 7.552333 | 6.209946 | 6.629086 | 7.908039 |
| 0.03468 | 0 | 0 | 0 |
| 0.137114 | 0.080418 | 0.122149 | 0.029765 |
| 2.991833 | 2.723377 | 2.869766 | 2.456995 |
| 6.763086 | 7.145585 | 7.203713 | 7.068353 |
| 0.124813 | 0.201309 | 0.229329 | 0 |
| 23.09631 | 19.99594 | 19.71387 | 19.07828 |
| 5.705179 | 6.063285 | 5.917019 | 4.438277 |
| 0 | 0 | 0 | 0 |
| 0.89569 | 0.745624 | 0.920193 | 0.914171 |
| 11.2302 | 10.67231 | 11.63377 | 10.67554 |
| 0.115373 | 0.230389 | 0.121134 | 0.078714 |
| 5.601888 | 2.409388 | 2.744756 | 2.4524 |
| 63.58852 | 58.46741 | 56.69124 | 60.75767 |
| 113.1556 | 118.8269 | 115.6602 | 119.8578 |
| 0.093073 | 0.080062 | 0 | 0.022225 |
| 166.922 | 181.4482 | 175.8465 | 181.9951 |
| 1.144583 | 1.238271 | 1.178391 | 0.989298 |
| 15.20512 | 11.61669 | 15.68432 | 11.94351 |
| 0.475764 | 0.170523 | 0.121411 | 0.236682 |
| 0 | 0.010989 | 0 | 0 |
| 12.56925 | 11.36483 | 10.94246 | 11.09987 |
| 1.719513 | 1.710681 | 1.496045 | 1.304716 |
| 0.043828 | 0.040394 | 0.084364 | 0.044853 |
| 0 | 0.026446 | 0 | 0 |
| 0.085181 | 0 | 0.10434 | 0.101701 |
| 8.928009 | 7.161833 | 7.707372 | 7.343265 |
| 0.149736 | 0 | 0 | 0 |
| 0.069989 | 0.200684 | 0.057154 | 0.167127 |
| 7.124233 | 4.650946 | 4.897608 | 4.730358 |
| 4.776531 | 3.718249 | 3.748226 | 3.44552 |
| 9.272323 | 8.643777 | 8.656074 | 8.512062 |
| 10.25088 | 11.36386 | 10.08061 | 9.653308 |
| 0.118418 | 0.267393 | 0.275602 | 0.155524 |
| 3.939345 | 4.269425 | 4.94029 | 4.031463 |
| 1.414338 | 1.520777 | 0.577486 | 0 |
| 0.490887 | 0.324818 | 0.416284 | 0.315589 |
| 16.82162 | 17.84446 | 16.45094 | 15.76497 |
| 168.6033 | 135.0189 | 132.3861 | 131.4022 |
| 0 | 0 | 0 | 0 |
| 1.18984 | 0.71211 | 0.549986 | 0.603088 |
| 44.05989 | 26.33509 | 27.43179 | 28.78481 |
| 0.69905 | 1.474406 | 1.086821 | 1.091438 |
| 7.798099 | 6.106732 | 6.112104 | 6.541318 |

| | | | |
|---|---|---|---|
| 3.768108 | 3.097153 | 3.354732 | 3.021835 |
| 5.70693 | 3.117695 | 3.382528 | 3.26402 |
| 5.519055 | 4.566804 | 4.395668 | 4.55568 |
| 8.420681 | 8.129924 | 5.885264 | 7.246023 |
| 0.356674 | 0.191758 | 0 | 0 |
| 8.466409 | 7.295765 | 9.120342 | 9.606615 |
| 4.838309 | 4.250763 | 4.3076 | 4.339563 |
| 1.433061 | 1.350281 | 1.429649 | 1.499331 |
| 28.6767 | 24.46637 | 25.41262 | 24.61017 |
| 1.755842 | 1.343987 | 1.330563 | 1.332447 |
| 3.722427 | 3.418858 | 3.958066 | 3.42588 |
| 1.420908 | 1.502657 | 1.415356 | 1.55667 |
| 0 | 0 | 0.040931 | 0 |
| 2.119318 | 2.360783 | 2.01704 | 2.111664 |
| 0.039515 | 0 | 0.048403 | 0 |
| 0.301367 | 0.324047 | 0 | 0.119939 |
| 16.89602 | 10.73538 | 18.81486 | 17.42211 |
| 3.13316 | 2.489162 | 2.713408 | 2.430348 |
| 2.475914 | 3.311571 | 2.884865 | 2.41536 |
| 1.6145 | 1.534727 | 1.633703 | 1.648262 |
| 21.21761 | 19.73136 | 19.74076 | 19.02906 |
| 4.158928 | 3.69032 | 4.215255 | 3.636272 |
| 0.226743 | 0 | 0 | 0 |
| 7.556899 | 7.717969 | 7.24432 | 7.845691 |
| 23.43904 | 20.66872 | 22.34039 | 21.39783 |
| 2.040889 | 1.95233 | 1.724093 | 1.999787 |
| 10.34575 | 8.538348 | 8.50661 | 9.327928 |
| 0.117198 | 0.036005 | 0.061525 | 0.05997 |
| 0.034529 | 0.037128 | 0.042296 | 0.041226 |
| 9.538705 | 8.146004 | 8.077701 | 8.510703 |
| 3.28902 | 2.250527 | 1.99151 | 2.275828 |
| 0.457392 | 0.525732 | 0.231836 | 0.320129 |
| 10.64394 | 9.054466 | 8.337623 | 9.235802 |
| 0.862149 | 0.699073 | 0.692503 | 0.928112 |
| 4.344219 | 3.068306 | 5.11266 | 3.152744 |
| 2.814627 | 2.501196 | 2.621396 | 2.610651 |
| 6.217174 | 5.983216 | 5.916557 | 5.708488 |
| 6.426706 | 5.479427 | 5.049359 | 5.425462 |
| 0 | 0 | 0.964067 | 0 |
| 0.695125 | 1.494876 | 1.277213 | 0.414971 |
| 2.708022 | 2.183864 | 2.598412 | 2.209379 |
| 0 | 1.050246 | 0.037389 | 1.275505 |
| 3.347247 | 3.557543 | 2.559616 | 3.395819 |
| 0 | 0.010244 | 0 | 0 |
| 0.636414 | 0.420479 | 0.450829 | 0.439429 |
| 0.131931 | 0.14186 | 0.134671 | 0.157519 |
| 16.52078 | 19.37015 | 20.37531 | 18.13074 |
| 1.981462 | 2.196137 | 1.979053 | 1.965403 |
| 4.124224 | 3.359546 | 6.123471 | 3.581171 |
| 25.93973 | 23.1061 | 21.50164 | 21.83425 |
| 0.292123 | 0.628215 | 0 | 0.697559 |
| 0.598944 | 0.644019 | 1.100492 | 1.072662 |
| 1.448636 | 1.049725 | 1.118688 | 1.090398 |
| 50.7941 | 47.74298 | 48.92946 | 46.51453 |

| | | | |
|---|---|---|---|
| 4.524602 | 4.749274 | 4.090742 | 4.244538 |
| 0 | 0 | 0 | 0 |
| 5.467396 | 5.331053 | 5.616471 | 5.177722 |
| 1.213148 | 1.108779 | 1.07736 | 1.339803 |
| 5.185906 | 3.801942 | 5.139623 | 4.897074 |
| 0.172772 | 0.185775 | 0.12698 | 0.041256 |
| 22.7774 | 21.97225 | 20.76197 | 21.19301 |
| 0.933648 | 0.714322 | 0.769763 | 0.450178 |
| 0.46335 | 0.351685 | 0.333864 | 0.097626 |
| 21.8342 | 21.77072 | 21.21174 | 22.4003 |
| 61.44427 | 60.10679 | 60.44338 | 58.36971 |
| 7.468012 | 6.991481 | 7.366989 | 6.68137 |
| 0.413693 | 0.889652 | 0.608091 | 0.987856 |
| 2.922774 | 2.705664 | 2.726625 | 2.28791 |
| 0.178226 | 0.159699 | 0.036386 | 0.070931 |
| 0 | 0 | 0 | 0 |
| 0 | 0 | 0 | 0 |
| 11.71067 | 13.14635 | 13.03653 | 13.93797 |
| 64.69428 | 72.83708 | 69.51558 | 72.28887 |
| 0.17603 | 0.283916 | 0.179686 | 0.175142 |
| 0.184884 | 0.018073 | 0.061764 | 0.100337 |
| 0.033746 | 0.036286 | 0 | 0 |
| 56.70459 | 54.81107 | 52.75967 | 54.64939 |
| 4.923206 | 4.687561 | 4.442356 | 4.958205 |
| 11.26928 | 9.914213 | 9.602794 | 9.732225 |
| 0 | 0.522444 | 0 | 0 |
| 2.365727 | 1.74572 | 1.76143 | 1.993803 |
| 164.1933 | 153.3908 | 143.0349 | 137.9187 |
| 8.463317 | 6.63382 | 8.017411 | 7.248041 |
| 8.523316 | 6.228245 | 6.690501 | 6.521309 |
| 0.109968 | 0 | 0 | 0 |
| 21.17864 | 20.9584 | 21.08356 | 19.11465 |
| 0.48097 | 0.079564 | 0.135958 | 1.06016 |
| 0.146963 | 0.039506 | 0 | 0 |
| 0.587852 | 0.632092 | 0.600062 | 0.233955 |
| 3.185626 | 3.46714 | 2.649021 | 3.339634 |
| 0.238378 | 0.224278 | 0.182497 | 0.071153 |
| 10.16557 | 8.902072 | 8.345936 | 9.195952 |
| 8.933855 | 7.041187 | 6.130539 | 7.14827 |
| 0 | 0 | 0.071654 | 0.209525 |
| 0.369117 | 0 | 0 | 0 |
| 0.937221 | 0.111973 | 0.063779 | 0.186499 |
| 558.2699 | 602.2818 | 597.0936 | 590.4765 |
| 0.577865 | 0.869894 | 0.601664 | 0.827928 |
| 4.763975 | 4.02664 | 4.761311 | 4.24473 |
| 3.215168 | 3.414713 | 2.996036 | 2.44926 |
| 11.69098 | 9.710247 | 10.29808 | 10.18654 |
| 0 | 0.4785 | 0 | 0 |
| 0.053262 | 0 | 0 | 0.063592 |
| 10.46965 | 10.06031 | 10.87339 | 9.416711 |
| 3.764846 | 3.649996 | 2.835032 | 3.352851 |
| 6.278356 | 5.519079 | 5.427957 | 5.513347 |
| 1.46015 | 0.999114 | 1.533062 | 1.154682 |
| 0 | 0 | 0 | 0 |

| | | | |
|---|---|---|---|
| 0 | 0 | 0 | 0 |
| 0.013172 | 0 | 0 | 0 |
| 0 | 0 | 0 | 0.054247 |
| 0 | 0 | 0.035783 | 0 |
| 453.9452 | 393.3707 | 390.4149 | 384.7457 |
| 0 | 0 | 0.490135 | 0 |
| 3.69814 | 2.987711 | 3.403578 | 3.365241 |
| 0.290238 | 0.220292 | 0.188216 | 0.326145 |
| 285.1453 | 266.3184 | 267.1206 | 252.6792 |
| 0 | 0.050945 | 0 | 0 |
| 0.065768 | 0 | 0 | 0 |
| 0.091218 | 0.196167 | 0.279339 | 0.10891 |
| 0.275693 | 0.211743 | 0.192973 | 0.188093 |
| 4.908694 | 3.834444 | 3.456825 | 3.308145 |
| 0.506554 | 0.297096 | 0.507674 | 0.054982 |
| 0.089926 | 0 | 0 | 0 |
| 3.253565 | 2.017336 | 2.036321 | 2.240018 |
| 9.274905 | 6.831833 | 7.782772 | 8.893882 |
| 17.21477 | 22.43448 | 20.87592 | 21.00572 |
| 0.31744 | 0 | 0 | 0 |
| 0.43485 | 0.233788 | 0.399494 | 0.259594 |
| 0.023964 | 0.051534 | 0.029354 | 0.028612 |
| 0.921727 | 1.075442 | 0.840779 | 0.889762 |
| 0.308023 | 0.159891 | 0.091073 | 0.240948 |
| 22.30357 | 12.45612 | 12.80778 | 12.70842 |
| 11.18213 | 9.578342 | 9.21242 | 8.878053 |
| 8.723338 | 8.241065 | 7.408109 | 6.488711 |
| 2.547101 | 2.30873 | 1.89521 | 2.161447 |
| 7.864512 | 6.765097 | 7.146256 | 6.111919 |
| 4.077124 | 3.99996 | 3.572471 | 3.695321 |
| 8.275304 | 8.045227 | 6.153221 | 7.639069 |
| 0 | 0 | 0.068138 | 0 |
| 14.47104 | 16.20307 | 15.1518 | 17.19608 |
| 0.183492 | 0.197301 | 0 | 0.328619 |
| 18.56219 | 10.55809 | 9.226721 | 11.72353 |
| 4.252245 | 3.716486 | 3.899546 | 3.760208 |
| 21.46719 | 19.15376 | 20.56099 | 21.06353 |
| 1.384187 | 1.984476 | 2.147665 | 1.542471 |
| 0 | 0 | 0 | 0 |
| 3.281037 | 3.219261 | 3.014267 | 3.672551 |
| 6.696006 | 6.506601 | 6.743951 | 5.921988 |
| 1.278564 | 0.624494 | 0.603938 | 0.673473 |
| 0.262588 | 0.141175 | 0.214434 | 0.052253 |
| 2.380802 | 2.047979 | 0.583261 | 1.137022 |
| 0.190682 | 0.058581 | 0 | 0.943182 |
| 0 | 0 | 0 | 0 |
| 1.597421 | 1.700288 | 1.91719 | 1.387082 |
| 15.92628 | 17.35823 | 17.2818 | 17.65461 |
| 8.806883 | 9.232262 | 9.285292 | 8.552559 |
| 0 | 0 | 0 | 0 |
| 0.134414 | 0.144529 | 0.24697 | 0.120362 |
| 11.64574 | 12.72413 | 12.35875 | 12.46271 |
| 6.495444 | 6.09597 | 5.46451 | 5.511264 |
| 0.361937 | 0.366283 | 0.352069 | 0.381295 |

| | | | |
|---|---|---|---|
| 0.225363 | 0.034618 | 0.039436 | 0.192194 |
| 29.95328 | 26.10883 | 26.92609 | 27.08733 |
| 5.265508 | 5.540016 | 4.577313 | 4.123563 |
| 12.75474 | 8.160329 | 7.316997 | 7.511943 |
| 0.010549 | 0 | 0 | 0.037783 |
| 0 | 0 | 0 | 0 |
| 28.84576 | 26.49224 | 26.37457 | 26.25708 |
| 9.015044 | 7.985964 | 8.558878 | 9.567551 |
| 0.157001 | 0.217049 | 0.109894 | 0.187451 |
| 0 | 0 | 0 | 0 |
| 1.607616 | 1.454219 | 1.594121 | 1.096806 |
| 1.117492 | 0.830368 | 0.689986 | 0.672537 |
| 4.468172 | 3.477836 | 3.451367 | 4.040886 |
| 3.062256 | 3.161004 | 2.5407 | 2.612947 |
| 11.49578 | 11.4479 | 12.40129 | 11.77574 |
| 0 | 0 | 0 | 0 |
| 10.21273 | 10.14197 | 9.80069 | 8.776192 |
| 0.028394 | 0 | 0.03478 | 0.0339 |
| 3.449219 | 2.261461 | 1.391169 | 1.807985 |
| 0 | 0 | 0 | 0 |
| 4.356677 | 3.749918 | 3.791454 | 4.151193 |
| 0 | 0 | 0 | 0 |
| 11.41048 | 9.944842 | 11.13563 | 10.04086 |
| 0.022334 | 0.04803 | 0.027357 | 0 |
| 20.30381 | 21.86876 | 19.39994 | 22.14977 |
| 0.417685 | 0 | 0 | 0 |
| 9.248009 | 10.63215 | 9.740609 | 9.207737 |
| 0 | 0 | 0 | 0 |
| 55.68506 | 56.34804 | 55.93654 | 51.55769 |
| 57.01228 | 50.38029 | 50.31595 | 48.05812 |
| 0 | 0.106003 | 0 | 0 |
| 0 | 0 | 0 | 0 |
| 43.65059 | 41.42215 | 40.27008 | 40.01849 |
| 27.53516 | 31.36582 | 30.60721 | 30.24187 |
| 7.159931 | 6.227207 | 6.930436 | 6.655543 |
| 1.630284 | 1.121272 | 1.547206 | 1.700973 |
| 0 | 0 | 0 | 0 |
| 21.69686 | 20.40787 | 20.36377 | 20.5975 |
| 0.819479 | 0.695645 | 0.898137 | 0.566451 |
| 15.59157 | 15.8749 | 15.55805 | 17.27081 |
| 9.122137 | 8.398142 | 7.886392 | 7.740965 |
| 0.610462 | 0.738454 | 0.654299 | 1.09329 |
| 0.098584 | 0 | 0 | 0 |
| 28.90974 | 30.81451 | 28.70012 | 28.87674 |
| 0.100881 | 0.054237 | 0.061786 | 0 |
| 0.165333 | 0.118517 | 0.405042 | 0.1974 |
| 2.934508 | 2.427193 | 2.586125 | 2.156093 |
| 0.042082 | 0 | 0.077321 | 0.025122 |
| 5.805043 | 8.217201 | 8.28086 | 8.510117 |
| 0.690088 | 0.039054 | 0.04449 | 0.043365 |
| 0 | 0 | 0.039732 | 0 |
| 0 | 0 | 0 | 0 |
| 0 | 0 | 0 | 0.059592 |
| 6.750012 | 5.902588 | 5.927243 | 5.898725 |

| | | | |
|---|---|---|---|
| 0.061046 | 0 | 0 | 0 |
| 0.469658 | 0.466156 | 0.398281 | 0.258806 |
| 0.225974 | 0.104135 | 0.158172 | 0.077086 |
| 0.054543 | 0 | 0.334055 | 0 |
| 0.163208 | 0.131618 | 0.049979 | 0.097431 |
| 18.61876 | 17.35623 | 16.09876 | 16.56341 |
| 0 | 0.406345 | 0 | 0.451199 |
| 0.432592 | 0.531598 | 0.340645 | 0.368923 |
| 1.834048 | 1.934864 | 1.695525 | 1.817913 |
| 0 | 0 | 0 | 0.020203 |
| 1.797667 | 1.352116 | 1.670488 | 1.533087 |
| 7.437284 | 7.809562 | 6.512306 | 7.284154 |
| 3.235398 | 2.309751 | 2.111498 | 2.026438 |
| 0.033681 | 0.027162 | 0.061884 | 0.010053 |
| 729.9793 | 667.1709 | 664.2962 | 663.7404 |
| 0 | 0 | 0.141913 | 0.276648 |
| 0.438453 | 0.37716 | 0.751901 | 0.942283 |
| 9.651626 | 8.131425 | 8.514206 | 8.761298 |
| 0.377905 | 0.310735 | 0.217838 | 0.318493 |
| 0.034765 | 0.01246 | 0 | 0.013836 |
| 1.928147 | 1.026754 | 1.057202 | 0.855068 |
| 85.53253 | 86.28062 | 88.5692 | 77.90705 |
| 3.346438 | 3.750321 | 3.738295 | 4.023611 |
| 0 | 0 | 0 | 0 |
| 8.628682 | 8.589983 | 8.151952 | 8.130775 |
| 0 | 0 | 0 | 0.025 |
| 0.215382 | 0.285035 | 0.162355 | 0.158249 |
| 3.553436 | 4.585029 | 5.223229 | 3.394095 |
| 0.221298 | 0.118976 | 0.271074 | 0.704584 |
| 1.790077 | 1.539834 | 3.069793 | 1.709807 |
| 255.1456 | 243.636 | 238.7616 | 244.1225 |
| 0.401823 | 0 | 0 | 0 |
| 23.99161 | 6.605764 | 7.32778 | 6.372625 |
| 7.125641 | 7.601802 | 6.298119 | 7.139749 |
| 0.063068 | 0 | 0.05794 | 0.03765 |
| 0 | 0 | 0 | 0 |
| 8.730809 | 9.000575 | 9.353347 | 9.065211 |
| 3.135599 | 2.786092 | 3.357888 | 2.914291 |
| 45.26004 | 56.13277 | 51.64287 | 58.62768 |
| 8.114345 | 6.443081 | 7.165149 | 5.536061 |
| 4.894625 | 4.457563 | 3.527722 | 3.762318 |
| 0 | 0 | 0.055051 | 0 |
| 0 | 0 | 0 | 0 |
| 5.851124 | 5.062096 | 4.283835 | 3.372522 |
| 0.180364 | 0 | 0.220932 | 0.646035 |
| 2.682594 | 3.064758 | 3.388662 | 3.236241 |
| 0.756103 | 0.475905 | 0.655095 | 0.37431 |
| 5.079789 | 4.951885 | 4.376164 | 4.298822 |
| 9.049909 | 8.584512 | 8.664494 | 8.662727 |
| 8.189352 | 8.754581 | 8.588314 | 8.734106 |
| 9.710804 | 8.602463 | 9.552047 | 9.33245 |
| 57.64128 | 55.25619 | 53.74197 | 56.12456 |
| 3.56815 | 3.189415 | 3.248648 | 2.979005 |
| 19.45525 | 20.16486 | 18.74855 | 18.06801 |

| | | | |
|---|---|---|---|
| 0 | 0.250978 | 0 | 0.278682 |
| 0 | 0 | 0.062115 | 0.060544 |
| 26.70616 | 23.88144 | 23.92098 | 23.89815 |
| 23.41364 | 23.61336 | 22.32357 | 22.99817 |
| 30.28845 | 34.71575 | 34.19899 | 31.33743 |
| 35.64764 | 53.28587 | 47.47274 | 48.3429 |
| 31.35533 | 26.16863 | 29.01755 | 25.60233 |
| 0.680229 | 1.097132 | 0.833229 | 0.812158 |
| 38.36124 | 38.1256 | 36.94672 | 35.56586 |
| 35.46065 | 37.01627 | 33.94504 | 35.13468 |
| 2.267431 | 0 | 2.777431 | 1.804796 |
| 0.032636 | 0 | 0 | 0 |
| 437.9129 | 476.85 | 470.0922 | 469.6644 |
| 0 | 0 | 0.198388 | 0 |
| 0 | 0.104064 | 0 | 0.115551 |
| 6.95023 | 5.653143 | 5.927743 | 6.110719 |
| 13.553 | 13.12343 | 13.41676 | 12.87628 |
| 11.85054 | 10.819 | 10.88404 | 10.53916 |
| 2.372759 | 2.306283 | 2.380991 | 1.872629 |
| 0.435347 | 0.46811 | 0.133317 | 0.151603 |
| 0 | 0 | 0 | 0 |
| 13.74112 | 14.80893 | 12.51352 | 13.0389 |
| 0 | 0 | 0 | 0.033285 |
| 1.006164 | 0.731004 | 0.466342 | 1.071437 |
| 7.102594 | 7.034364 | 6.616789 | 6.221296 |
| 0.375669 | 0.504926 | 0.805291 | 0.448529 |
| 0 | 0.559557 | 0.318722 | 0.621323 |
| 5.87241 | 5.65502 | 4.679951 | 4.392655 |
| 0.198194 | 0.106555 | 0.060693 | 0.118317 |
| 3.311428 | 3.008123 | 2.797413 | 2.454004 |
| 0 | 1.29619 | 0 | 0.719634 |
| 2.021369 | 1.790834 | 1.639058 | 1.699584 |
| 32.80336 | 30.05943 | 28.70117 | 29.9047 |
| 15.20099 | 15.186 | 15.2805 | 15.0644 |
| 1.749335 | 2.248006 | 2.900624 | 2.394266 |
| 5.000661 | 3.986845 | 3.376457 | 3.174574 |
| 0.807052 | 0 | 0 | 0 |
| 0 | 0 | 0.017175 | 0 |
| 8.045676 | 7.750676 | 7.034299 | 8.1777 |
| 1.947929 | 1.978162 | 2.518625 | 2.842554 |
| 7.838032 | 7.304179 | 7.520781 | 53.10781 |
| 0.830126 | 0.535559 | 0.406737 | 0.317161 |
| 4.438146 | 3.397122 | 3.962118 | 3.472736 |
| 1.54701 | 0.880641 | 1.003219 | 1.005012 |
| 0.152453 | 0.054642 | 0.062248 | 0.060674 |
| 33.14116 | 34.27274 | 35.71999 | 34.7391 |
| 35.08417 | 33.53744 | 34.74083 | 31.92384 |
| 2.060517 | 1.696452 | 1.584086 | 1.502853 |
| 3.695574 | 3.438771 | 3.017866 | 3.478947 |
| 2.207854 | 2.413577 | 2.434009 | 1.845244 |
| 1.577444 | 1.154087 | 1.095606 | 1.22323 |
| 1.743517 | 1.77776 | 1.47288 | 1.399743 |
| 0 | 0 | 0 | 0 |
| 1.522443 | 0.944047 | 0.938159 | 0.81407 |

| | | | |
|---|---|---|---|
| 0 | 0.390836 | 0 | 0 |
| 0 | 0 | 0 | 0 |
| 3.749612 | 3.078039 | 2.716286 | 3.562219 |
| 4.902091 | 4.306799 | 4.430291 | 4.24688 |
| 0 | 0 | 0.032841 | 0.032011 |
| 0.348835 | 0.281316 | 0.373885 | 0.832983 |
| 26.44923 | 24.13976 | 25.28981 | 24.73911 |
| 11.5052 | 12.49233 | 12.61264 | 11.92815 |
| 9.949621 | 8.878944 | 8.581019 | 8.768078 |
| 0.038246 | 0.041124 | 0.046848 | 0.045664 |
| 32.23941 | 25.92836 | 27.027 | 24.85536 |
| 87.91235 | 96.54065 | 94.31999 | 93.41237 |
| 1.402682 | 1.642908 | 1.457384 | 1.465388 |
| 7.117408 | 4.96499 | 6.232494 | 4.986321 |
| 0.111513 | 0 | 0.136595 | 0 |
| 0.215068 | 0.277504 | 0.421507 | 0.102712 |
| 8.631404 | 7.637114 | 7.789813 | 8.860404 |
| 14.43068 | 14.06025 | 13.65621 | 12.19126 |
| 2.107753 | 2.094681 | 2.073294 | 2.173382 |
| 1.630686 | 0.631227 | 1.398227 | 0.545147 |
| 3.319212 | 2.020687 | 2.541115 | 2.564273 |
| 38.97495 | 40.89184 | 43.00031 | 39.27991 |
| 55.80117 | 48.32956 | 47.42807 | 45.08147 |
| 0.330667 | 0.355552 | 0.405042 | 0 |
| 0 | 0.161005 | 0.036683 | 0 |
| 0 | 0 | 0 | 0 |
| 0 | 0 | 0 | 0 |
| 20.9263 | 13.60051 | 14.87765 | 14.5476 |
| 0.97566 | 1.069656 | 1.241977 | 1.46182 |
| 0 | 0 | 0 | 0.053482 |
| 2.159458 | 0.773991 | 1.013983 | 0.773484 |
| 0 | 0.068449 | 0 | 0.076004 |
| 0 | 0 | 0 | 0 |
| 13.64001 | 11.95544 | 12.80946 | 13.17642 |
| 0.184201 | 0.495159 | 0.338449 | 0.32989 |
| 2.870471 | 2.723377 | 2.0683 | 1.411197 |
| 8.955536 | 8.99806 | 6.973551 | 8.180013 |
| 0 | 0.12397 | 0 | 0 |
| 0.238677 | 0.12832 | 0.146181 | 0.427452 |
| 0.043872 | 0.015725 | 0.071654 | 0.122223 |
| 0.012567 | 0 | 0.046181 | 0 |
| 0.774245 | 0.416256 | 0.368819 | 7.292551 |
| 0.18673 | 0 | 0 | 0.222945 |
| 6.845277 | 5.742754 | 5.851034 | 4.80495 |
| 5.075064 | 4.140899 | 4.826984 | 4.063338 |
| 0.060968 | 0 | 0 | 0 |
| 0.291081 | 0.391234 | 0.237702 | 0.376497 |
| 0.145838 | 0 | 0 | 0.02902 |
| 2.070263 | 1.484043 | 4.096479 | 2.028132 |
| 0.017404 | 0 | 0 | 0 |
| 17.78347 | 20.32527 | 19.39688 | 19.28582 |
| 3.598514 | 2.901995 | 3.581425 | 3.375773 |
| 60.35863 | 61.43535 | 61.21826 | 64.97267 |
| 8.567624 | 9.982535 | 10.317 | 9.09271 |

| | | | |
|---|---|---|---|
| 16.16869 | 19.13999 | 20.25967 | 21.3413 |
| 52.30116 | 48.21799 | 45.44279 | 50.22988 |
| 1.908236 | 1.659664 | 1.806502 | 1.887042 |
| 0 | 0 | 0 | 0 |
| 1.53151 | 1.240422 | 0.77963 | 0.71242 |
| 5.805708 | 6.226069 | 5.527017 | 5.883683 |
| 29.06857 | 24.64728 | 27.67941 | 25.4686 |
| 94.3818 | 95.78805 | 92.77689 | 90.54785 |
| 15.17257 | 13.53749 | 13.91917 | 12.8734 |
| 12.76209 | 10.13387 | 9.941678 | 9.509142 |
| 0.2889 | 0.225922 | 0.193026 | 0.12543 |
| 1.569876 | 1.15196 | 1.403256 | 1.165137 |
| 0 | 0.083251 | 0 | 0 |
| 11.76181 | 11.13761 | 11.29435 | 12.04936 |
| 3.06302 | 2.994122 | 2.728705 | 2.327238 |
| 0 | 0 | 0 | 0 |
| 0.018918 | 0.040683 | 0 | 0 |
| 21.78716 | 17.09088 | 16.28596 | 18.08381 |
| 0 | 0 | 0 | 0 |
| 15.75301 | 8.829152 | 8.582185 | 9.270937 |
| 0.220905 | 0.2019 | 0.257062 | 0.382436 |
| 5.657866 | 4.781347 | 4.74747 | 5.267815 |
| 2.350713 | 1.314363 | 1.520348 | 1.145105 |
| 0 | 0.034925 | 0.039786 | 0 |
| 16.19065 | 11.69173 | 11.19685 | 13.26763 |
| 0 | 0 | 0 | 0 |
| 21.83463 | 11.22556 | 10.13688 | 11.55263 |
| 0 | 0.051405 | 0 | 0.057079 |
| 1.20547 | 1.29619 | 1.476609 | 1.439268 |
| 36.17226 | 33.34023 | 31.63675 | 33.18227 |
| 4.438784 | 4.02338 | 4.673274 | 4.029507 |
| 0.533513 | 0.71708 | 0.163378 | 0.796234 |
| 1.348896 | 1.015287 | 1.211684 | 1.073675 |
| 23.49903 | 20.94826 | 20.02238 | 21.12086 |
| 9.411547 | 9.262219 | 8.011285 | 8.665744 |
| 0.134509 | 0 | 0.164763 | 0 |
| 0 | 0.018143 | 0.020668 | 0.020146 |
| 0 | 0 | 0 | 0 |
| 0 | 0.29425 | 0 | 0 |
| 9.405639 | 8.722875 | 11.66521 | 7.860891 |
| 5.206021 | 5.63877 | 5.365997 | 5.048377 |
| 1.734496 | 2.220273 | 2.782249 | 1.972284 |
| 16.91008 | 12.75801 | 14.45754 | 14.64966 |
| 429.639 | 370.8285 | 365.5435 | 377.2102 |
| 22.13525 | 27.19478 | 27.24288 | 26.90566 |
| 2.445268 | 3.250761 | 2.668513 | 2.070208 |
| 0.010412 | 0.011196 | 0.012754 | 0.037296 |
| 0.049714 | 0.267276 | 0.353196 | 0.29678 |
| 7.598683 | 6.127903 | 5.654279 | 5.892842 |
| 0 | 0 | 0 | 0 |
| 11.42682 | 9.776717 | 9.82633 | 9.162079 |
| 0 | 0 | 0 | 0.125777 |
| 0.927231 | 0.762421 | 0.734922 | 0.325608 |
| 3.789724 | 3.2698 | 3.444158 | 2.700244 |

| | | | |
|---|---|---|---|
| 0 | 0 | 0 | 0 |
| 376.0305 | 279.7138 | 280.5849 | 273.3345 |
| 0.031618 | 0.033997 | 0.077458 | 0.113249 |
| 2.046801 | 1.840701 | 1.671451 | 1.76248 |
| 0.028309 | 0.01522 | 0.034677 | 0 |
| 7.65816 | 7.926397 | 7.593874 | 6.810934 |
| 20.11644 | 17.60342 | 16.03421 | 15.33063 |
| 0 | 0 | 0.101613 | 0 |
| 19.53446 | 19.06046 | 19.23552 | 20.12281 |
| 1.374679 | 1.223283 | 0.638712 | 0.877244 |
| 0.009282 | 0.029941 | 0.01137 | 0.011082 |
| 2.54736 | 1.956477 | 2.811721 | 2.406395 |
| 1.812568 | 0.932639 | 0.830894 | 1.022311 |
| 10.05614 | 8.66763 | 8.479914 | 9.400574 |
| 0.045177 | 0 | 0 | 0.107877 |
| 0.022854 | 0.012287 | 0.013997 | 0.027286 |
| 6.122063 | 3.291396 | 2.499688 | 3.248634 |
| 149.0698 | 141.3516 | 133.2465 | 147.3096 |
| 1.144841 | 1.356611 | 1.058913 | 1.338985 |
| 1.486281 | 1.420564 | 1.685724 | 1.774543 |
| 0.273431 | 0.042001 | 0.239237 | 0.139912 |
| 20.52276 | 19.77766 | 18.75476 | 20.07221 |
| 57.24057 | 47.23546 | 48.0535 | 49.2239 |
| 0 | 0 | 0 | 0 |
| 0.512 | 0 | 0 | 0.611302 |
| 5.065243 | 3.754629 | 4.108183 | 4.05373 |
| 12.38923 | 10.76511 | 11.17388 | 11.16166 |
| 0 | 0 | 0 | 0.43069 |
| 0 | 0 | 0 | 0 |
| 1.073753 | 0.377856 | 0.143484 | 0.326329 |
| 4.780727 | 5.27136 | 4.770003 | 4.275766 |
| 5.905498 | 4.690094 | 4.557481 | 4.801389 |
| 11.79317 | 12.39466 | 10.75285 | 11.53961 |
| 15.09163 | 14.70293 | 15.10422 | 14.28794 |
| 0.033153 | 0 | 0.020305 | 0 |
| 10.9401 | 9.373979 | 9.422439 | 8.617237 |
| 13.75087 | 13.10791 | 13.6781 | 13.24488 |
| 0.040837 | 0 | 0 | 0 |
| 4.794107 | 5.737371 | 5.806064 | 6.144315 |
| 2.327312 | 2.300647 | 2.597889 | 2.106426 |
| 0.01649 | 0 | 0.0101 | 0 |
| 1.976424 | 2.073012 | 2.227886 | 2.142593 |
| 6.313139 | 6.903302 | 7.287481 | 7.230947 |
| 3.564883 | 3.80598 | 3.77829 | 3.491562 |
| 0.133006 | 0.071508 | 0.162922 | 0.132335 |
| 0.212572 | 0.114285 | 0.195288 | 0.06345 |
| 3.868364 | 3.781351 | 4.221532 | 3.750884 |
| 5.52955 | 4.558668 | 4.666506 | 4.712778 |
| 3.621198 | 3.080105 | 3.707445 | 2.839328 |
| 3.559646 | 3.703264 | 3.850652 | 2.952945 |
| 8.023698 | 7.23676 | 6.364308 | 7.093299 |
| 0 | 0.075515 | 0 | 0 |
| 3.685244 | 3.068586 | 3.523235 | 2.951213 |
| 0.47616 | 0 | 0 | 0 |

| | | | |
|---|---|---|---|
| 0 | 0.308431 | 0 | 0 |
| 9.613538 | 9.296384 | 8.219391 | 8.935943 |
| 4.317675 | 3.007888 | 3.327242 | 3.218899 |
| 2.257431 | 1.919275 | 2.05781 | 2.193813 |
| 5.170805 | 4.559954 | 3.690946 | 4.219417 |
| 0 | 0 | 0 | 0 |
| 40.70404 | 39.63831 | 32.926 | 33.46879 |
| 0.049446 | 0 | 0 | 0 |
| 4.009337 | 2.722195 | 2.683404 | 2.726582 |
| 0.364981 | 0.241507 | 0.309513 | 0.301686 |
| 0.104697 | 0.04503 | 0.025649 | 0.125002 |
| 0 | 0 | 0 | 0 |
| 0.470014 | 0.505386 | 0.708593 | 0.474838 |
| 0.023502 | 0.075814 | 0.028789 | 0 |
| 1.992493 | 1.533793 | 1.442204 | 1.486833 |
| 0 | 0 | 0 | 0.074608 |
| 2.263662 | 1.838279 | 1.473664 | 1.795496 |
| 620.9219 | 744.1975 | 738.9147 | 738.2883 |
| 0.156632 | 0.033684 | 0.038372 | 0.037402 |
| 23.15942 | 22.91237 | 22.45755 | 22.55045 |
| 0 | 0 | 0.571824 | 0 |
| 0 | 0 | 0 | 0 |
| 2.164366 | 0.77575 | 2.651185 | 3.44552 |
| 4.188351 | 4.344337 | 3.601654 | 3.662109 |
| 6.218982 | 5.910231 | 6.078837 | 5.625108 |
| 6.43379 | 5.885925 | 5.841791 | 5.497097 |
| 6.918958 | 6.415181 | 6.18736 | 6.698985 |
| 29.75256 | 31.06017 | 28.94348 | 30.99347 |
| 0.110993 | 0 | 0 | 0 |
| 0.373459 | 1.003911 | 1.829837 | 1.337673 |
| 9.554941 | 8.605911 | 7.8819 | 9.176999 |
| 19.05793 | 12.91853 | 11.56647 | 13.42795 |
| 0.512 | 0 | 0 | 0 |
| 3.887348 | 3.343918 | 3.265171 | 3.239432 |
| 8.712167 | 10.40869 | 11.50498 | 7.434373 |
| 4.66083 | 3.476508 | 2.520262 | 4.211192 |
| 7.081361 | 10.76502 | 7.4777 | 6.413969 |
| 0.210807 | 0.226672 | 0.193667 | 0.18877 |
| 16.36161 | 14.51373 | 15.46852 | 13.83314 |
| 1.343497 | 0.873482 | 0.880249 | 0.9699 |
| 3.372076 | 3.000702 | 2.207702 | 2.429534 |
| 294.9288 | 661.9165 | 610.277 | 658.5336 |
| 0 | 0.042349 | 0.096486 | 0 |
| 0 | 0.036967 | 0.084225 | 0.041048 |
| 1.566765 | 1.538182 | 1.209911 | 1.463977 |
| 4.394401 | 4.013108 | 3.825113 | 4.276409 |
| 9.18424 | 8.841085 | 8.584788 | 10.09045 |
| 0.598192 | 1.029135 | 0.293096 | 0.71421 |
| 0.616506 | 0.701897 | 0.621908 | 0.432986 |
| 0.331637 | 0.30029 | 0.192425 | 0.125039 |
| 14.18717 | 14.82253 | 15.51375 | 14.67567 |
| 0.209878 | 0.169255 | 0.192813 | 0.375875 |
| 34.73441 | 38.83246 | 41.41996 | 43.11894 |
| 2.219695 | 1.886297 | 2.236563 | 1.966278 |

| | | | |
|---|---|---|---|
| 0.496 | 0.399996 | 0.151891 | 0.14805 |
| 9.381072 | 12.44326 | 11.37503 | 12.2046 |
| 1.984002 | 0.711104 | 0.810084 | 1.579197 |
| 0 | 0.474069 | 0 | 0 |
| 0 | 0 | 0 | 0 |
| 5.724239 | 4.898146 | 4.169155 | 4.67944 |
| 0.365996 | 0.288596 | 0.26899 | 0.305886 |
| 8.607935 | 9.802495 | 9.654275 | 8.976488 |
| 10.43761 | 8.68886 | 9.181959 | 8.843973 |
| 6.623175 | 5.068144 | 4.635937 | 4.809784 |
| 29.49667 | 27.79626 | 27.6931 | 26.94903 |
| 7.686055 | 6.732531 | 7.531869 | 6.714695 |
| 0.078965 | 0 | 0.096726 | 0.09428 |
| 13.49343 | 12.83565 | 13.8573 | 11.91783 |
| 13.79068 | 12.36516 | 12.32551 | 10.99479 |
| 0 | 0 | 0 | 0 |
| 0 | 0.062591 | 0 | 0 |
| 162.4378 | 174.1015 | 176.3223 | 168.0619 |
| 0.378059 | 0.3127 | 0.284981 | 0.231478 |
| 0 | 0.066149 | 0 | 0 |
| 22.74025 | 21.89095 | 19.92028 | 20.9 |
| 0 | 0 | 0 | 0.149215 |
| 3.311327 | 3.05349 | 3.05493 | 3.102788 |
| 0 | 0 | 0 | 0 |
| 0 | 0 | 0 | 0.129797 |
| 25.23103 | 22.65724 | 22.99522 | 24.17805 |
| 0.325316 | 0.294567 | 0.188757 | 0.470182 |
| 86.97864 | 45.05555 | 52.10461 | 52.303 |
| 1.753716 | 1.085703 | 0.65096 | 0.571049 |
| 40.97727 | 43.35781 | 40.37958 | 38.77275 |
| 10.81151 | 10.55722 | 9.871633 | 10.469 |
| 18.23854 | 19.30401 | 17.84268 | 18.46321 |
| 22.35433 | 15.45604 | 14.18304 | 15.15948 |
| 0 | 0 | 0 | 0 |
| 0 | 0 | 0 | 0 |
| 2.69606 | 1.685656 | 1.920286 | 2.32423 |
| 54.04853 | 56.51344 | 52.46463 | 55.25888 |
| 0.295982 | 0.596731 | 0.815749 | 0.706773 |
| 0.14235 | 0.076531 | 0.348736 | 0.084979 |
| 4.7812 | 4.930321 | 5.040524 | 4.866266 |
| 0 | 0 | 0 | 0 |
| 0 | 0 | 0 | 0 |
| 69.75922 | 62.35773 | 60.74031 | 62.89452 |
| 4.960465 | 5.001156 | 4.844717 | 4.616678 |
| 0.613082 | 0.21974 | 0.750979 | 0.487992 |
| 5.473565 | 5.075168 | 4.664141 | 5.422282 |
| 0.865746 | 0.46545 | 0.530237 | 0.516828 |
| 0.040732 | 0 | 0.099788 | 0 |
| 0 | 0 | 0 | 0.169705 |
| 14.26458 | 14.93923 | 12.10307 | 10.0657 |
| 96.93605 | 102.5686 | 97.48066 | 99.42347 |
| 0.219654 | 0.104971 | 0.179373 | 0.058279 |
| 0.566858 | 0 | 0 | 0 |
| 0.025054 | 0.02694 | 0.03069 | 0 |

| | | | |
|---|---|---|---|
| 19.53674 | 20.4667 | 21.18913 | 20.23513 |
| 0.545467 | 0.379511 | 0.23582 | 0.191547 |
| 0 | 0 | 0 | 0 |
| 0.05492 | 0 | 0 | 0 |
| 0.744376 | 0.568023 | 0.38237 | 0.831408 |
| 0.098584 | 0.106003 | 0.241516 | 0.235408 |
| 40.1498 | 41.23687 | 38.85955 | 38.16326 |
| 0 | 0 | 0 | 0 |
| 0.01281 | 0.027549 | 0.015692 | 0 |
| 35.77912 | 34.29329 | 35.99878 | 33.67635 |
| 7.29345 | 6.227207 | 5.887804 | 6.077666 |
| 2.170477 | 1.858795 | 2.282222 | 2.041043 |
| 0.786904 | 1.053154 | 1.13822 | 1.029476 |
| 20.74806 | 20.11064 | 18.81427 | 19.87115 |
| 2.669107 | 1.730749 | 2.271147 | 2.189387 |
| 0.595201 | 0 | 0 | 0.710639 |
| 0.025906 | 0.055712 | 0 | 0.092793 |
| 0.314413 | 0.388159 | 0.299547 | 0.431006 |
| 0 | 0 | 0 | 0.551952 |
| 0.535012 | 0.862913 | 0.21845 | 0.585545 |
| 2.504032 | 2.819682 | 3.06725 | 2.495327 |
| 0.093917 | 0 | 0.057521 | 0.084099 |
| 0.037791 | 0 | 0 | 0 |
| 0 | 0 | 0 | 0 |
| 0 | 0 | 0 | 0 |
| 4.153558 | 3.91363 | 3.356896 | 2.607379 |
| 0 | 0 | 0 | 0 |
| 0.020604 | 0.066464 | 0 | 0 |
| 20.96107 | 17.22348 | 16.22936 | 16.60336 |
| 15.18169 | 13.66637 | 13.33868 | 12.36327 |
| 4.174167 | 4.127049 | 3.76619 | 3.403529 |
| 0 | 0 | 0 | 0 |
| 0.748908 | 0.571481 | 0.606638 | 0.548032 |
| 1.01902 | 0.498049 | 0.491724 | 0.663631 |
| 0.257384 | 0.092251 | 0.157638 | 0.102434 |
| 27.83023 | 21.67972 | 19.49793 | 20.88491 |
| 15.5921 | 14.8376 | 15.01243 | 15.28313 |
| 9.128465 | 8.489034 | 7.630741 | 8.763315 |
| 1.070023 | 0.767034 | 0.436899 | 0.425851 |
| 3.628084 | 4.033365 | 3.339374 | 3.842282 |
| 0 | 0 | 0.041484 | 0 |
| 29.19881 | 28.80176 | 30.04222 | 28.99077 |
| 0.014473 | 0.046686 | 0.088641 | 0.0864 |
| 23.73499 | 26.77765 | 26.65823 | 25.63531 |
| 4.63144 | 4.540577 | 4.171444 | 4.174381 |
| 10.22274 | 11.07755 | 11.02255 | 10.9716 |
| 16.32263 | 15.4016 | 15.30817 | 15.48767 |
| 0 | 0 | 0 | 0 |
| 43.84673 | 47.95221 | 49.39775 | 42.26554 |
| 6.783291 | 4.969783 | 5.702268 | 6.312376 |
| 37.04079 | 36.17547 | 34.6332 | 36.04712 |
| 0.217425 | 0 | 0.133165 | 0.259594 |
| 44.6198 | 42.49209 | 41.95905 | 42.0582 |
| 25.42409 | 16.72838 | 16.70789 | 24.94505 |

| | | | |
|---|---|---|---|
| 2.780499 | 1.868594 | 3.405901 | 3.319772 |
| 8.98669 | 4.146437 | 3.368125 | 3.08277 |
| 3.10161 | 2.552317 | 2.500519 | 2.890733 |
| 0 | 0 | 1.851621 | 0.902398 |
| 2.805286 | 13.41506 | 13.02163 | 12.58216 |
| 5.13139 | 4.851953 | 3.791294 | 3.111931 |
| 23.71032 | 15.19875 | 14.12273 | 15.24325 |
| 61.48226 | 57.00163 | 57.44476 | 59.70145 |
| 0.183139 | 0.240681 | 0.249257 | 0.145772 |
| 8.54647 | 4.594826 | 3.73885 | 6.559741 |
| 0.312236 | 0.335734 | 0 | 0.372794 |
| 5.551117 | 4.718441 | 4.607325 | 4.816234 |
| 0.220445 | 0.218801 | 0.270028 | 0.242953 |
| 0.596132 | 0.160249 | 3.103421 | 0.177938 |
| 0.147418 | 0 | 0 | 0 |
| 2.597928 | 2.467199 | 2.671245 | 2.196159 |
| 2.417919 | 1.870093 | 1.506865 | 1.595376 |
| 27.97341 | 26.82923 | 28.66281 | 27.25537 |
| 0.037611 | 0.040442 | 0.046071 | 0.044906 |
| 6.92597 | 5.024121 | 5.957368 | 5.213883 |
| 0.109968 | 0 | 0 | 0 |
| 1.525275 | 1.321377 | 0.97402 | 0.897603 |
| 0.245624 | 0.03773 | 0.214908 | 0.083789 |
| 0.614462 | 0.437081 | 0.405283 | 0.564335 |
| 0.372324 | 0.400344 | 0.35472 | 0.345749 |
| 2.596365 | 2.671128 | 2.29692 | 2.430188 |
| 0 | 0 | 0 | 0.064899 |
| 26.99746 | 28.23246 | 26.67687 | 27.23313 |
| 2.604306 | 3.615311 | 3.285305 | 3.341452 |
| 0.178337 | 0.115055 | 0 | 0.08517 |
| 0.098994 | 0 | 0 | 0 |
| 0 | 0 | 0 | 0 |
| 0 | 0.09094 | 0 | 0 |
| 2.700218 | 1.274676 | 2.258824 | 1.022219 |
| 5.346955 | 4.196018 | 4.734109 | 4.435191 |
| 14.28811 | 14.26347 | 11.27718 | 13.78925 |
| 0.168931 | 0.036329 | 0.165542 | 0.134463 |
| 0 | 0 | 0 | 0 |
| 12.5594 | 11.29373 | 10.64099 | 9.103539 |
| 0 | 0 | 0 | 0 |
| 0.195348 | 0.262561 | 0.139584 | 0.233235 |
| 2.292144 | 2.139125 | 3.708287 | 2.581793 |
| 1.625527 | 1.108399 | 1.068421 | 1.230748 |
| 9.119586 | 9.017742 | 9.491686 | 8.251477 |
| 8.239181 | 4.103629 | 4.150544 | 4.173338 |
| 0.044048 | 0.015788 | 0.053956 | 0 |
| 0.380928 | 0.136532 | 0 | 0.227404 |
| 1.433695 | 1.261301 | 1.436865 | 1.400529 |
| 5.605593 | 3.657106 | 3.741817 | 4.662391 |
| 0.78896 | 0.698628 | 0.909568 | 0.831156 |
| 0.116706 | 0 | 0.285912 | 0.139341 |
| 6.796631 | 6.690237 | 5.873869 | 6.57703 |
| 17.61266 | 12.93114 | 13.09605 | 14.70059 |
| 1.054288 | 0.981352 | 1.098673 | 1.202402 |

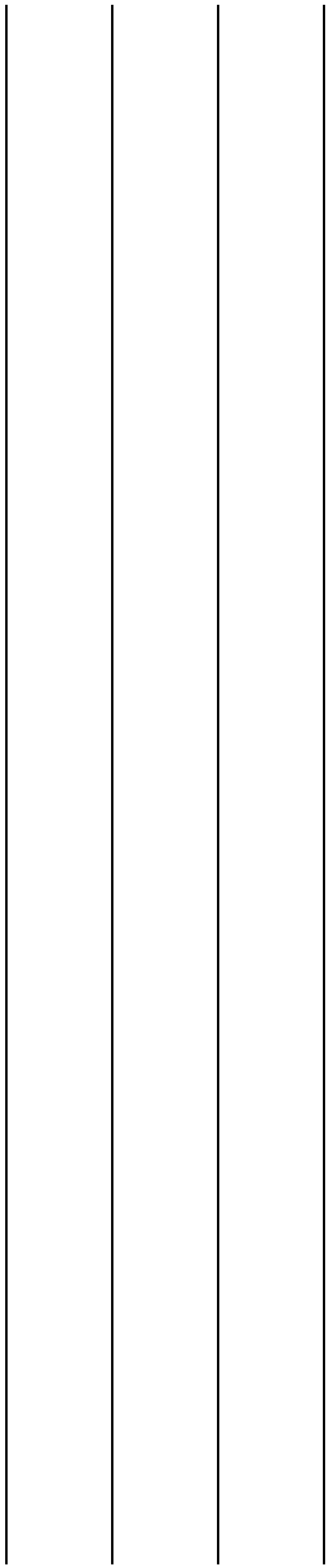

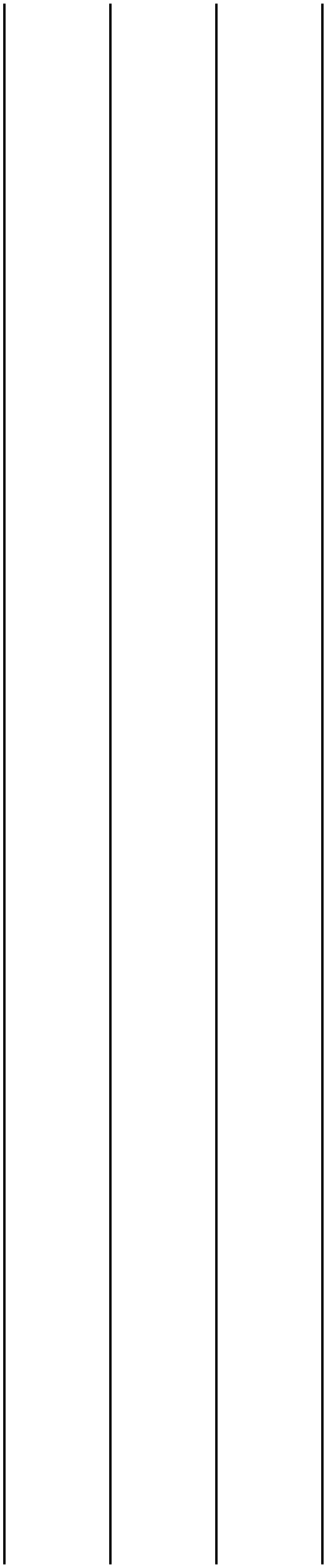

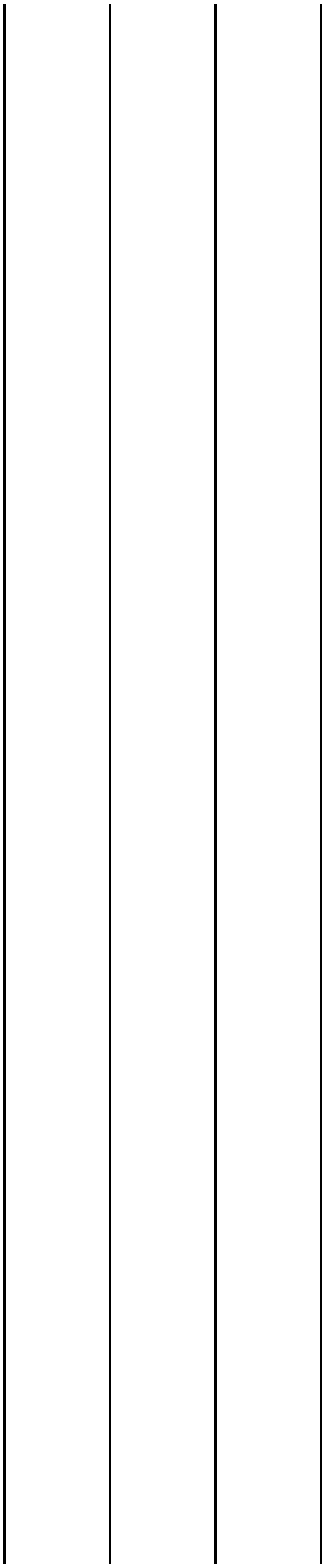

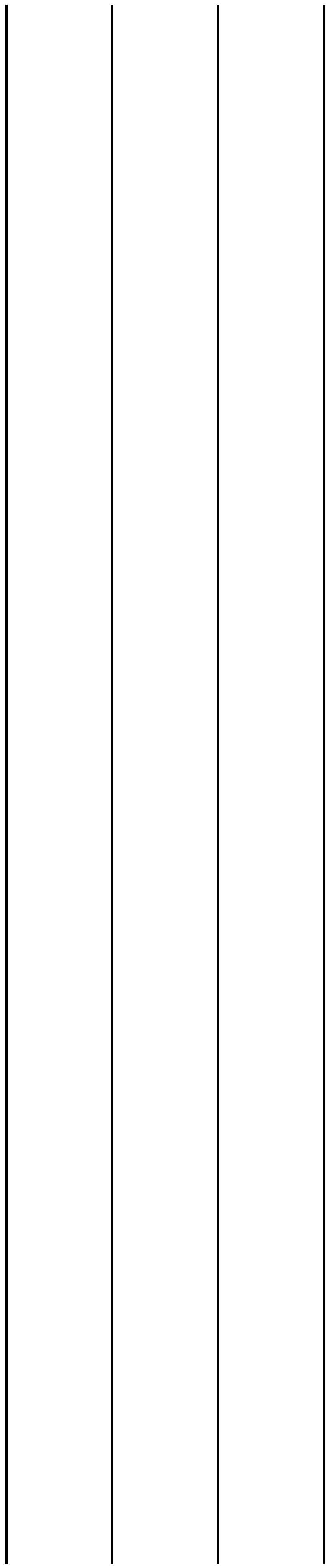

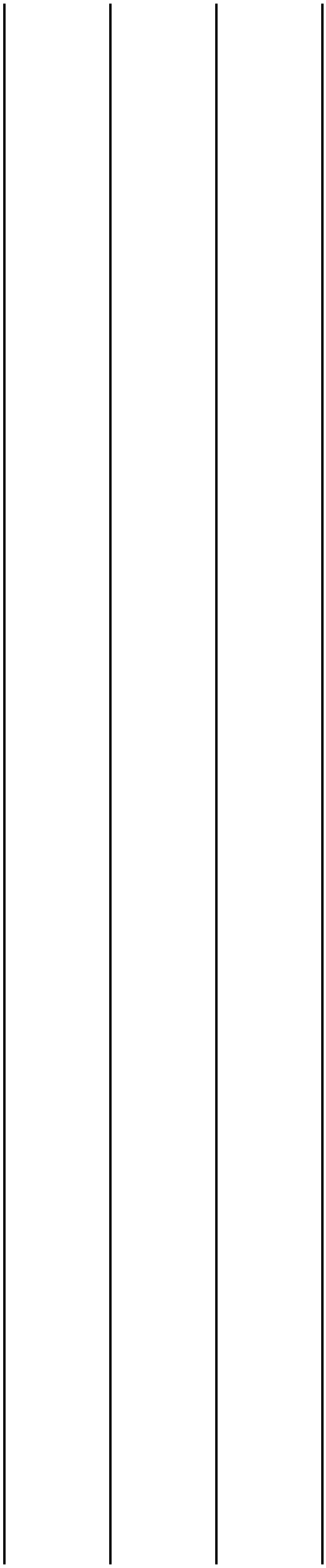

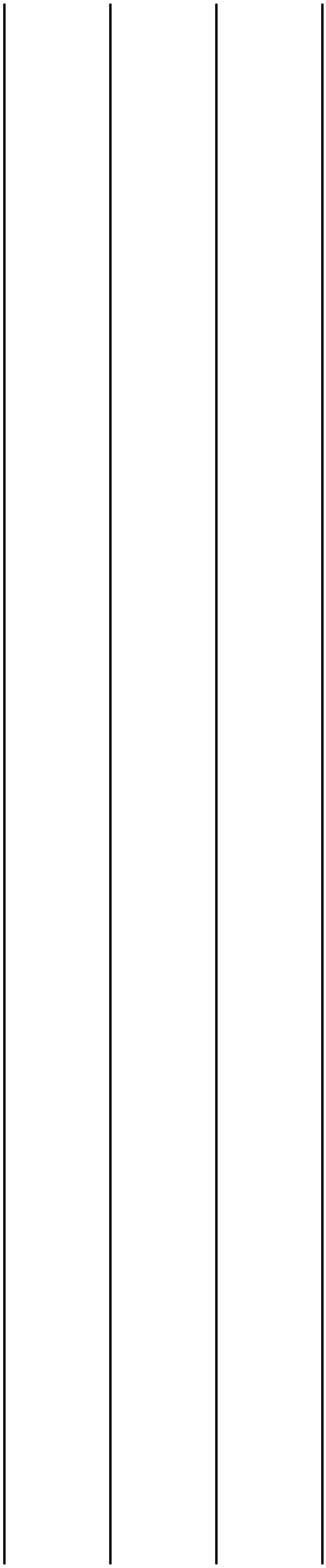

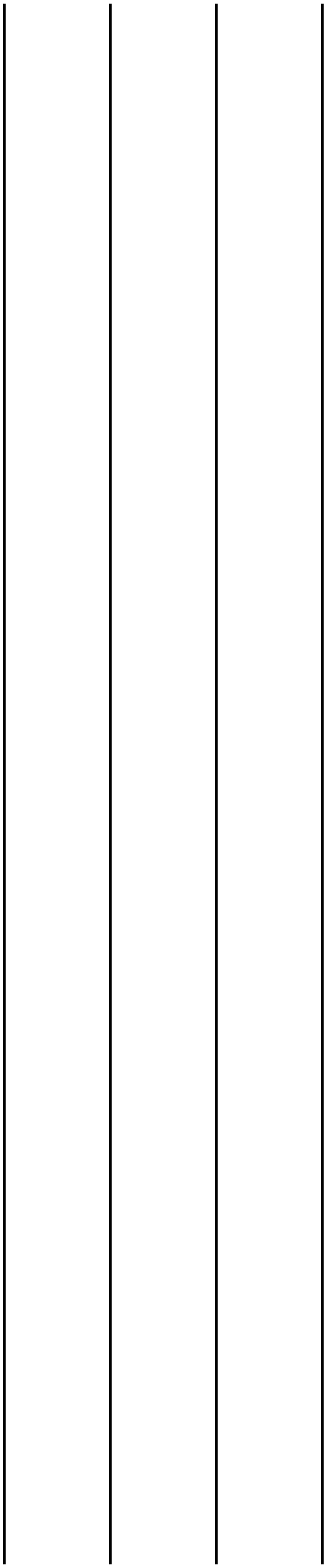

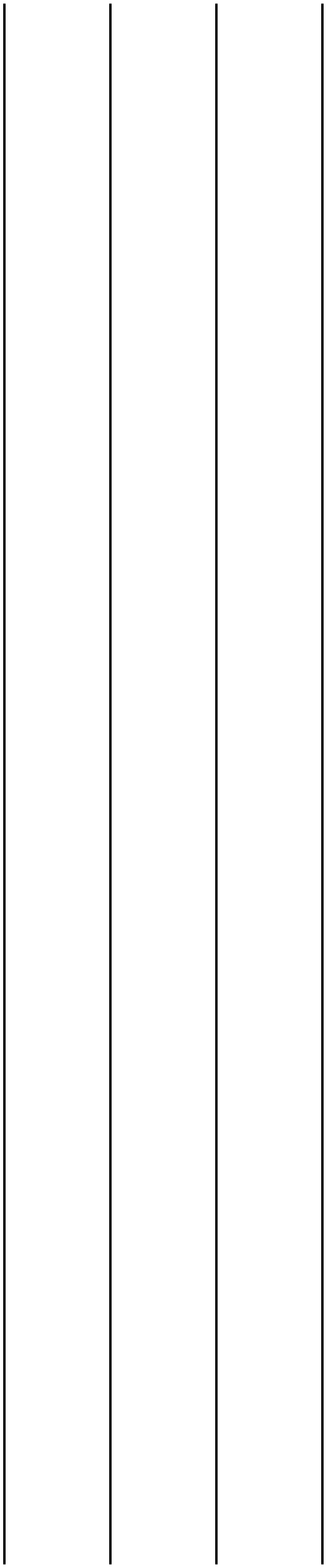

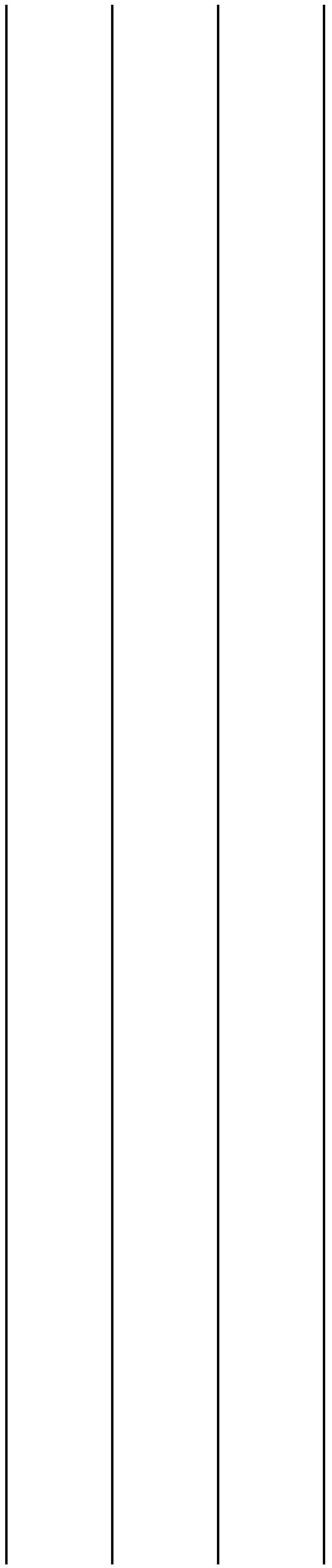

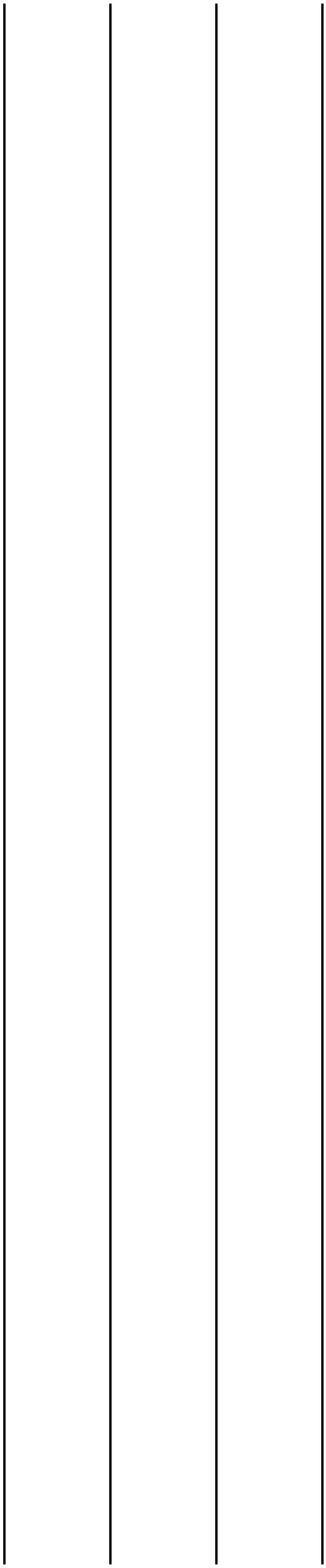

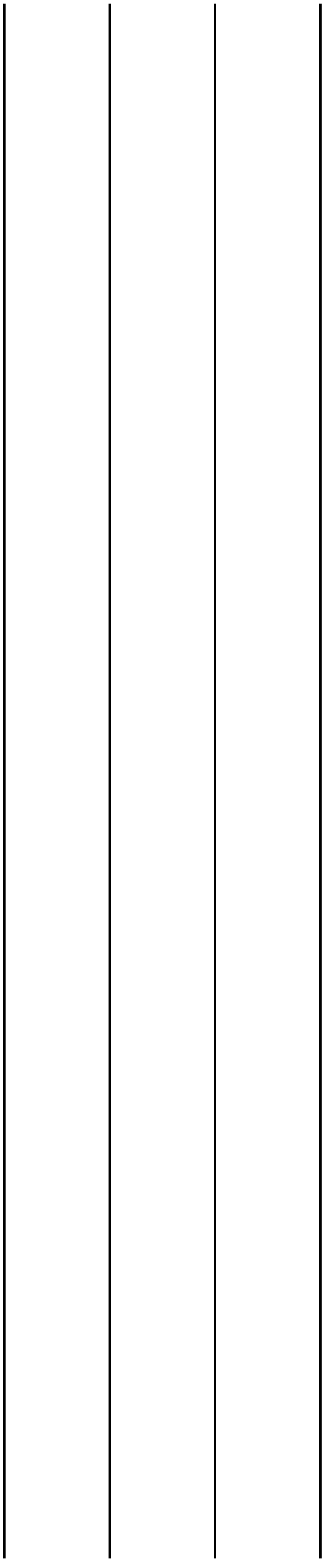

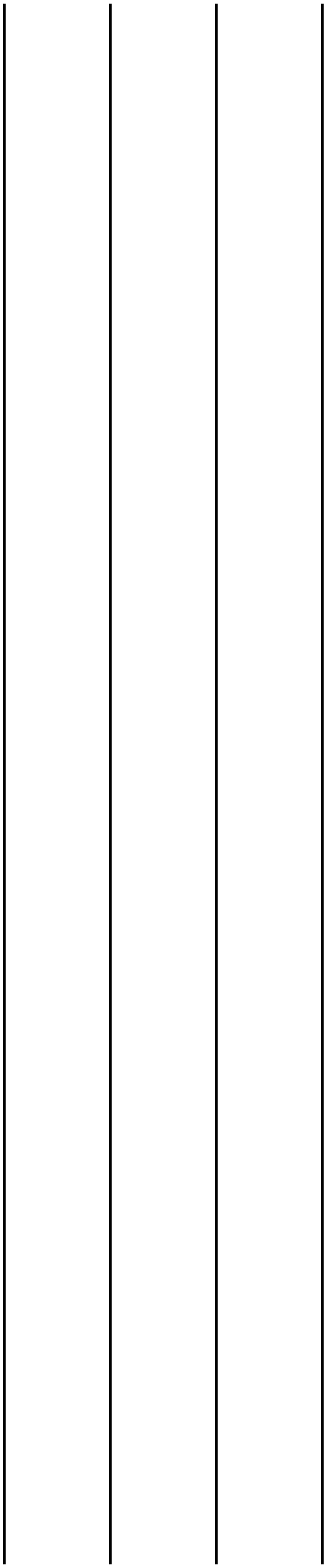

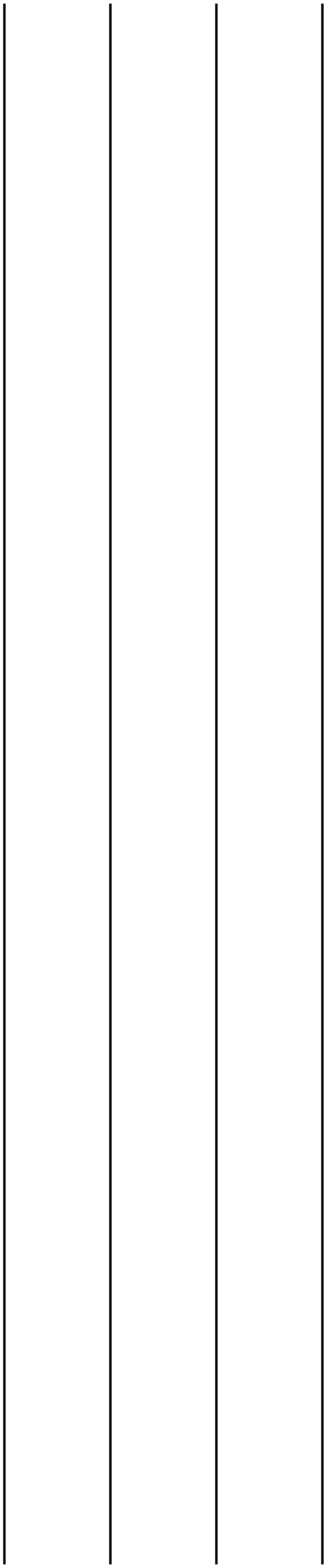

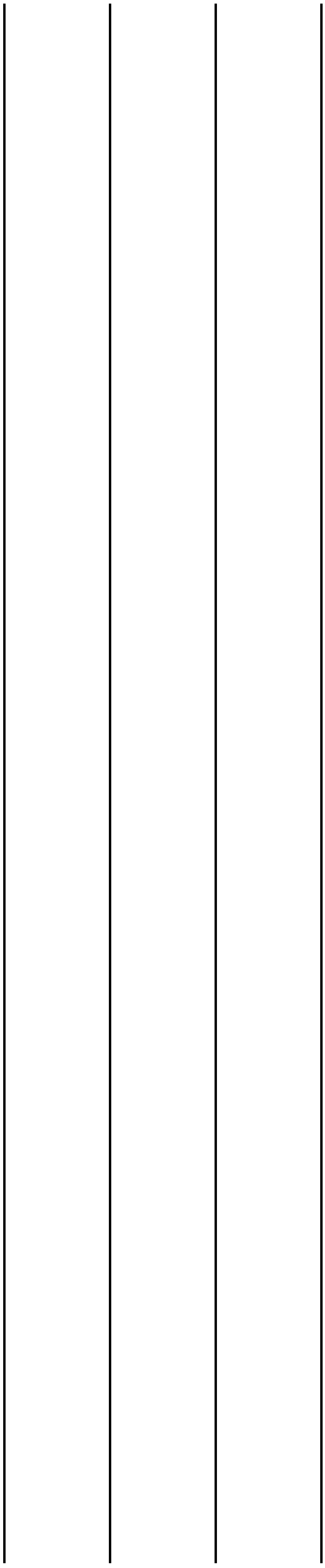

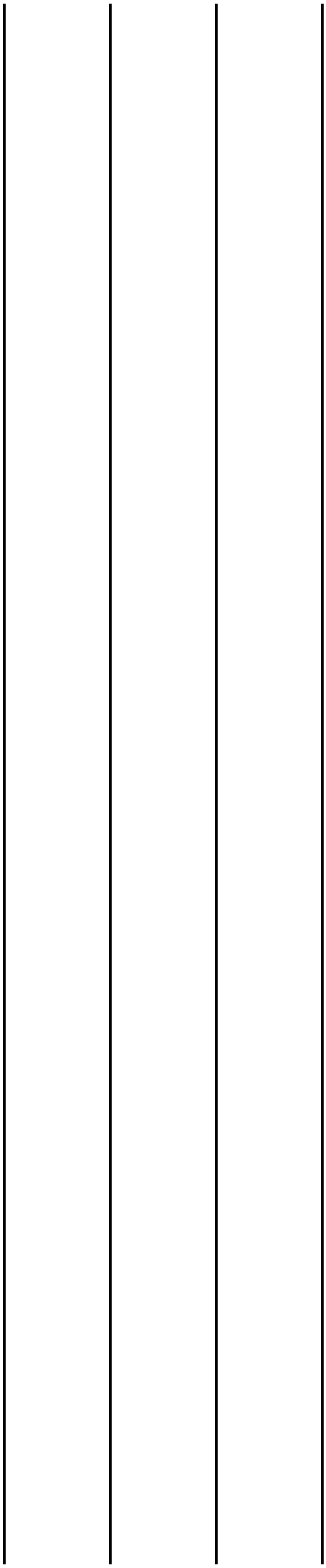

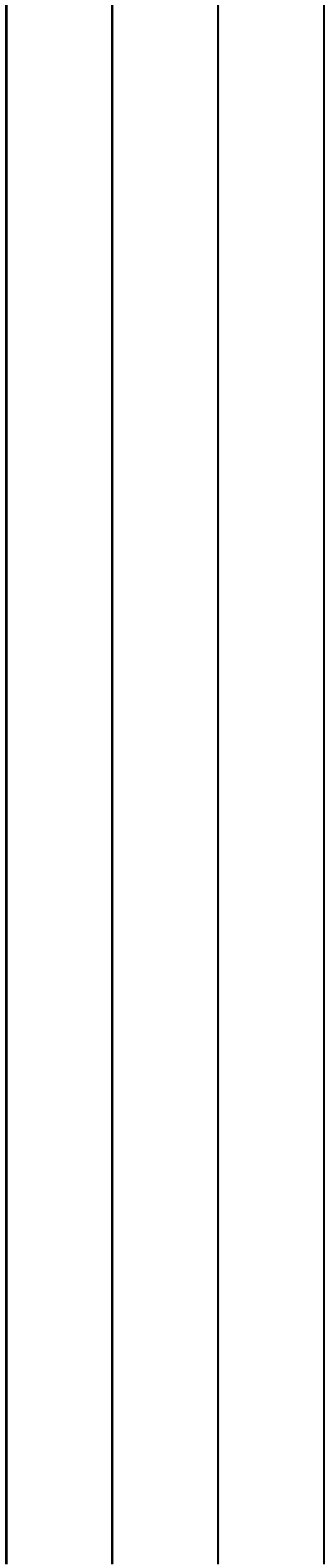

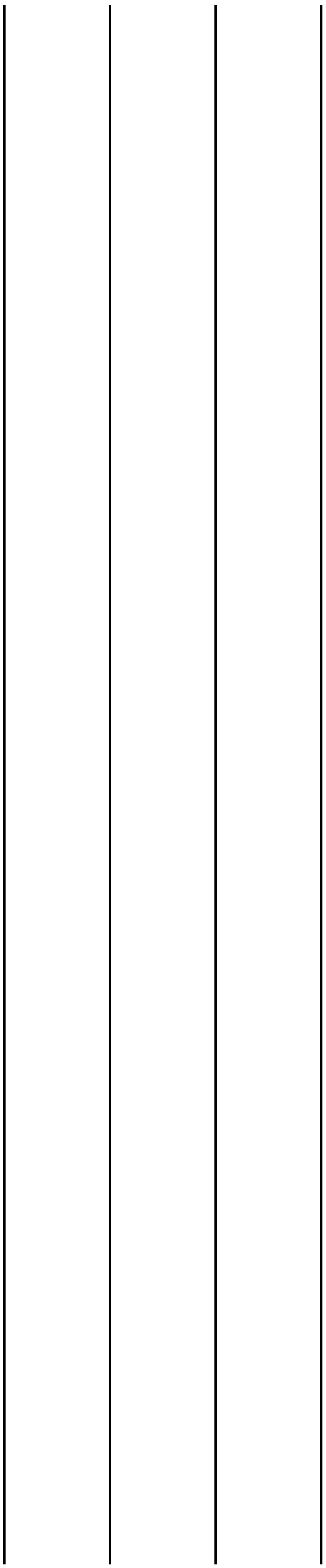

Supplement: Supplementary Materials — Antibodies such as Nrf2 (ab89443), Tlr4 (19811-1-AP), and Myd88 (sc-74532) were used for IF staining. As for F-actin staining, the samples were incubated with 0.1% Triton X-100 for 15 min. Rhodamine phalloidin (PHDR1, cytoskeleton) was used for the staining of F-actin after the treatment of 0.1% Triton X-100 for 15 min and then followed by 4,6-DAPI for 10 min before observation. Table S1: the RNA-seq data of failing heart treated with or without YXS or VST. Figure S1: the enrichment of DEs in YXS-mediated protection against H2O2-induced damage; (A) enriched GO terms of upregulated DEs; (B) enriched GO terms of downregulated DEs. Figure S2: YXS decreased the levels of Tlr4 and Myd88, enhanced Nrf2 expression, and improved cytoskeleton arrangement; (A) the IF staining of Tlr4 (red) and Myd88 (green) in H2O2-induced H9C2 cell and the related quantification, nucleus (blue), scale bar: 100 μm (n = 3–5); (B) the IF staining of Nrf2 (green) and F-actin (red) in heart tissue, nucleus (blue), scale bar: 100 μm; (C) the IF staining of Nrf2 (green) in H2O2-induced H9C2 cell and the related quantification, nucleus (blue), scale bar: 100 μm (n = 3–5). [file Xiang.Table S1 RNA-Seq.pdf › Xiang.Table S1 RNA-Seq.pdf]
